# Small-quantity lipid-based nutrient supplements for children age 6–24 months: a systematic review and individual participant data meta-analysis of effects on developmental outcomes and effect modifiers

**DOI:** 10.1093/ajcn/nqab277

**Published:** 2021-09-29

**Authors:** Elizabeth L Prado, Charles D Arnold, K Ryan Wessells, Christine P Stewart, Souheila Abbeddou, Seth Adu-Afarwuah, Benjamin F Arnold, Ulla Ashorn, Per Ashorn, Elodie Becquey, Kenneth H Brown, Jaya Chandna, Parul Christian, Holly N Dentz, Sherlie J L Dulience, Lia C H Fernald, Emanuela Galasso, Lotta Hallamaa, Sonja Y Hess, Lieven Huybregts, Lora L Iannotti, Elizabeth Y Jimenez, Patricia Kohl, Anna Lartey, Agnes Le Port, Stephen P Luby, Kenneth Maleta, Andrew Matchado, Susana L Matias, Malay K Mridha, Robert Ntozini, Clair Null, Maku E Ocansey, Sarker M Parvez, John Phuka, Amy J Pickering, Andrew J Prendergast, Abu A Shamim, Zakia Siddiqui, Fahmida Tofail, Ann M Weber, Lee S F Wu, Kathryn G Dewey

**Affiliations:** Institute for Global Nutrition & Department of Nutrition, University of California Davis, Davis, CA, USA; Institute for Global Nutrition & Department of Nutrition, University of California Davis, Davis, CA, USA; Institute for Global Nutrition & Department of Nutrition, University of California Davis, Davis, CA, USA; Institute for Global Nutrition & Department of Nutrition, University of California Davis, Davis, CA, USA; Public Health Nutrition, Department of Public Health and Primary Care, University of Ghent, Ghent, Belgium; Department of Nutrition and Food Science, University of Ghana, Legon, Accra, Ghana; Francis I Proctor Foundation, University of California, San Francisco, San Francisco, CA, USA; Center for Child Health Research, Faculty of Medicine and Health Technology, Tampere University, Tampere, Finland; Center for Child Health Research, Faculty of Medicine and Health Technology, Tampere University, Tampere, Finland; Department of Paediatrics, Tampere University Hospital, Tampere, Finland; Poverty, Health, and Nutrition Division, International Food Policy Research Institute, Washington, DC, USA; Institute for Global Nutrition & Department of Nutrition, University of California Davis, Davis, CA, USA; Helen Keller International, New York, NY, USA; Department of Infectious Disease Epidemiology, London School of Hygiene & Tropical Medicine, London, United Kingdom; Program in Human Nutrition, Department of International Health, Johns Hopkins Bloomberg School of Public Health, Baltimore, MD, USA; Institute for Global Nutrition & Department of Nutrition, University of California Davis, Davis, CA, USA; Brown School, Washington University in St. Louis, St Louis, MO, USA; School of Public Health, University of California, Berkeley, Berkeley, CA, USA; Development Research Group, World Bank, Washington, DC, USA; Center for Child Health Research, Faculty of Medicine and Health Technology, Tampere University, Tampere, Finland; Institute for Global Nutrition & Department of Nutrition, University of California Davis, Davis, CA, USA; Poverty, Health, and Nutrition Division, International Food Policy Research Institute, Washington, DC, USA; Brown School, Washington University in St. Louis, St Louis, MO, USA; Departments of Pediatrics and Internal Medicine and College of Population Health, University of New Mexico Health Sciences Center, Albuquerque, NM, USA; Brown School, Washington University in St. Louis, St Louis, MO, USA; Department of Nutrition and Food Science, University of Ghana, Legon, Accra, Ghana; Independent consultant, Dakar, Senegal; Division of Infectious Diseases and Geographic Medicine, Stanford University, Stanford, CA, USA; Department of Public Health, School of Public Health and Family Medicine, College of Medicine, University of Malawi, Blantyre, Malawi; Malawi Epidemiology and Intervention Research Unit, Karonga, Malawi; Department of Nutritional Sciences and Toxicology, University of California, Berkeley, Berkeley, CA, USA; Center for Non-communicable Diseases and Nutrition, BRAC James P Grant School of Public Health, Dhaka, Bangladesh; Zvitambo Institute for Maternal and Child Health Research, Harare, Zimbabwe; Mathematica, Washington, DC, USA; CDC Foundation, Atlanta, GA, USA; Infectious Diseases Division, International Centre for Diarrhoeal Disease Research, Bangladesh (icddr,b), Dhaka, Bangladesh; Department of Public Health, School of Public Health and Family Medicine, College of Medicine, University of Malawi, Blantyre, Malawi; School of Engineering, Tufts University, Medford, MA, USA; Blizard Institute, Queen Mary University of London, London, United Kingdom; Center for Non-communicable Diseases and Nutrition, BRAC James P Grant School of Public Health, Dhaka, Bangladesh; Healthy Systems and Population Studies Division, International Centre for Diarrhoeal Disease Research, Bangladesh (icddr,b), Dhaka, Bangladesh; Nutrition and Clinical Sciences Division, International Centre for Diarrhoeal Disease Research, Bangladesh (icddr,b), Dhaka, Bangladesh; Division of Epidemiology, School of Community Health Sciences, University of Nevada, Reno, Reno, NV, USA; Program in Human Nutrition, Department of International Health, Johns Hopkins Bloomberg School of Public Health, Baltimore, MD, USA; Institute for Global Nutrition & Department of Nutrition, University of California Davis, Davis, CA, USA

**Keywords:** language development, motor development, social-emotional development, executive function, child undernutrition, complementary feeding, nutrient supplements

## Abstract

**Background:**

Small-quantity (SQ) lipid-based nutrient supplements (LNSs) provide many nutrients needed for brain development.

**Objectives:**

We aimed to generate pooled estimates of the effect of SQ-LNSs on developmental outcomes (language, social-emotional, motor, and executive function), and to identify study-level and individual-level modifiers of these effects.

**Methods:**

We conducted a 2-stage meta-analysis of individual participant data from 14 intervention against control group comparisons in 13 randomized trials of SQ-LNSs provided to children age 6–24 mo (total *n *= 30,024).

**Results:**

In 11–13 intervention against control group comparisons (*n *= 23,588–24,561), SQ-LNSs increased mean language (mean difference: 0.07 SD; 95% CI: 0.04, 0.10 SD), social-emotional (0.08; 0.05, 0.11 SD), and motor scores (0.08; 95% CI: 0.05, 0.11 SD) and reduced the prevalence of children in the lowest decile of these scores by 16% (prevalence ratio: 0.84; 95% CI: 0.76, 0.92), 19% (0.81; 95% CI: 0.74, 0.89), and 16% (0.84; 95% CI: 0.76, 0.92), respectively. SQ-LNSs also increased the prevalence of children walking without support at 12 mo by 9% (1.09; 95% CI: 1.05, 1.14). Effects of SQ-LNSs on language, social-emotional, and motor outcomes were larger among study populations with a higher stunting burden (≥35%) (mean difference: 0.11–0.13 SD; 8–9 comparisons). At the individual level, greater effects of SQ-LNSs were found on language among children who were acutely malnourished (mean difference: 0.31) at baseline; on language (0.12), motor (0.11), and executive function (0.06) among children in households with lower socioeconomic status; and on motor development among later-born children (0.11), children of older mothers (0.10), and children of mothers with lower education (0.11).

**Conclusions:**

Child SQ-LNSs can be expected to result in modest developmental gains, which would be analogous to 1–1.5 IQ points on an IQ test, particularly in populations with a high child stunting burden. Certain groups of children who experience higher-risk environments have greater potential to benefit from SQ-LNSs in developmental outcomes.

This trial was registered at www.crd.york.ac.uk/PROSPERO as CRD42020159971.

## Introduction

Brain development occurs rapidly in utero and during the first few years after birth, laying the foundation of the neural structures that underlie children's development of cognitive skills, such as language and executive function, as well as social-emotional and motor skills (1). Adequate availability of nutrients, such as iron, iodine, zinc, B-vitamins, and essential fatty acids, is necessary for the neurodevelopmental processes that occur during this period, such as myelination, synaptogenesis, and axon and dendrite growth (2). Inadequate dietary intake during this foundational period could lead to lasting structural and functional neurodevelopmental deficits (3). At age 6–24 mo, children are at particular risk of inadequate dietary intake of these nutrients as they transition from exclusive breastfeeding to joining family meals, in what is called the complementary feeding period (4). Small-quantity (SQ) lipid-based nutrient supplements (LNSs) were designed to fill this gap between the needs and dietary intakes of key nutrients experienced by many children during this time period, for prevention of undernutrition in low- and middle-income countries (LMICs). SQ-LNSs are typically made from vegetable oil, peanut paste, milk powder, and sugar, with added vitamins and minerals, thus providing many of the micronutrients and fatty acids that are necessary for brain development (5). SQ-LNSs provide ∼120 kcal/d, whereas other LNS products (medium- and large-quantity) provide more energy and are designed for treatment of moderate and severe acute malnutrition.

Two previous systematic reviews and meta-analyses have addressed the effects of LNSs provided during the complementary feeding period on developmental outcomes (6, 7). In a 2019 Cochrane review by Das et al. (6), the authors provided a narrative review of effects on these outcomes, but were not able to generate pooled estimates owing to differences between studies in measurement and reporting of developmental outcomes. The other meta-analysis by Tam et al. (7) generated pooled estimates of the effects of LNSs using published data from studies on various developmental outcomes, including a total of <3600 children. SQ-LNSs had significant positive effects on mean language scores (effect size: 0.13 SD; 5 studies), social-emotional scores (0.12 SD; 5 studies), and motor scores (0.13 SD; 6 studies), and no effect on executive function (3 studies) or on the prevalence of children standing or walking without support at age 12 mo (4 studies), although heterogeneity across trials was moderate to substantial.

Here, we report an individual participant data (IPD) meta-analysis (8) of SQ-LNSs provided during the complementary feeding period, which adds to the current evidence-base in several ways. First, we included a larger number of trials (13 trials) and children (30,024) than previous meta-analyses. Second, we analyzed IPD, rather than aggregate data from published reports, which enabled harmonization of the calculation of developmental outcomes across trials. Third, we examined study-level and individual-level factors that may modify the effect of SQ-LNSs on developmental outcomes. Identifying characteristics of children and populations who experience greater benefits from SQ-LNSs, or are more likely to respond to the intervention, may be useful to inform public health programs and policies. Our first objective was to generate pooled estimates of the effect of randomized controlled trials of SQ-LNSs provided to infants and young children in the age range of 6–24 mo, compared with children who received no intervention or an intervention without any nutritional supplement, on developmental outcomes. The other 2 objectives were to identify study-level modifiers (Objective 2) and individual-level modifiers (Objective 3) of these effects.

## Methods

The protocol for this IPD meta-analysis was registered as PROSPERO CRD42020159971 (9). The detailed protocol was posted to Open Science Framework before analysis (10) and updated after consultations with co-investigators before finalizing the analysis plan. The results are reported according to Preferred Reporting Items for Systematic Reviews and Meta-Analyses (PRISMA)-IPD guidelines (11). The analyses were approved by the institutional review board of the University of California, Davis. All individual trial protocols were approved by appropriate institutional ethics committees. The methods are presented in detail in a companion article published in the same journal issue (12) and summarized here.

### Inclusion and exclusion criteria for this IPD meta-analysis

We included prospective randomized controlled trials of SQ-LNSs provided to children in the age range of 6–24 mo that met the inclusion criteria listed in Dewey et al. (12). In addition to those criteria, for the analyses presented here, we only included trials that measured ≥1 developmental outcome of interest, as described below.

### Search methods and identification of studies

We identified studies cited in a recent systematic review and meta-analysis of child LNSs (6) and through database searches, as described in Dewey et al. (12).

### Data collection

We invited all principal investigators of eligible studies to participate in this IPD meta-analysis. We provided a data dictionary listing definitions of variables requested for pooled analysis. For further details, see Dewey et al. (12). The variables requested for this IPD meta-analysis were *1*) intervention group, as determined by each trial design; *2*) randomization cluster, if cluster-randomized; *3*) child sex; *4*) child age at developmental assessment; *5*) whether each motor milestone had been attained by the child at the time of assessment; *6*) continuous unstandardized developmental outcome scores of interest measured at baseline (before child supplementation) and postsupplementation, as available, calculated according to the established method for the tool used in each study; and *7*) indicator variables for potential effect modifiers, as prespecified in the analysis plan. Study-level effect modifiers included variables reflecting sample characteristics and study design (**Box 1**). Individual-level effect modifiers included maternal, child, and household characteristics (Box 1).

Box 1. Potential effect modifiers^1^

**FIGURE 1:**
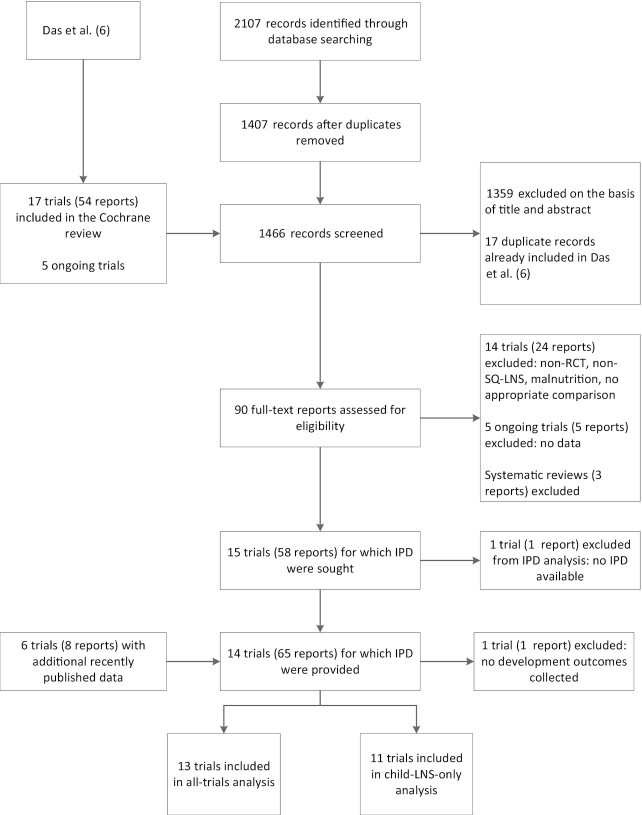
Study inclusion flow diagram. IPD, individual participant data; LNS, lipid-based nutrient supplement; RCT, randomized controlled trial; SQ, small-quantity.

**FIGURE 2:**
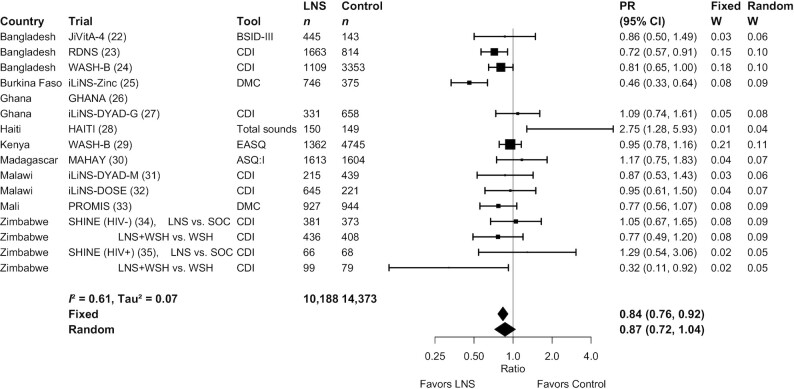
Forest plot of the effect of small-quantity LNSs on the prevalence of children in the lowest decile of language scores. Individual study estimates were generated from log-binomial regression controlling for baseline measure when available and with clustered observations using robust SEs for cluster-randomized trials. Pooled estimates were generated using inverse-variance weighting in both fixed- and random-effects models. Individual trial estimates for the SHINE trial are split by comparison in the figure to reflect the crossover design. For calculating the pooled estimates, the trial was analyzed with LNS intervention arms combined and non-LNS intervention arms combined. ASQ:I, Ages and Stages Questionnaire Inventory; BSID, Bayley Scales of Infant Development; CDI, MacArthur-Bates Communicative Development Inventory; DMC, Developmental Milestones Checklist; EASQ, Extended Ages and Stages Questionnaire; LNS, lipid-based nutrient supplement; PR, prevalence ratio; SOC, standard of care; WSH, water, sanitation, and hygiene intervention.

**FIGURE 3:**
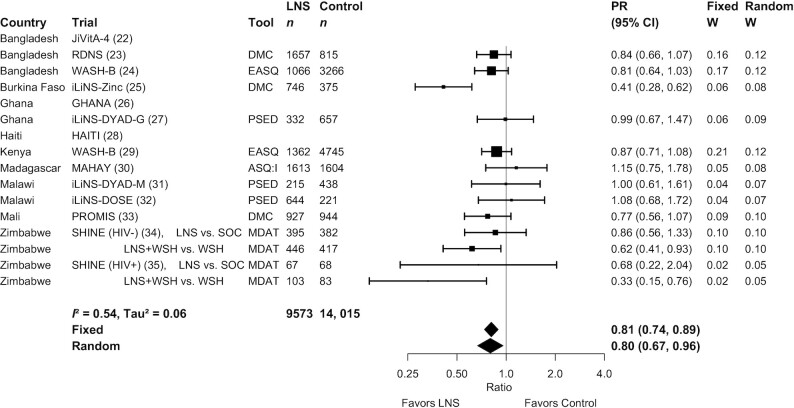
Forest plot of the effect of small-quantity LNSs on the prevalence of children in the lowest decile of social-emotional scores. Individual study estimates were generated from log-binomial regression controlling for baseline measure when available and with clustered observations using robust SEs for cluster-randomized trials. Pooled estimates were generated using inverse-variance weighting in both fixed- and random-effects models. Individual trial estimates for the SHINE trial are split by comparison in the figure to reflect the crossover design. For calculating the pooled estimates, the trial was analyzed with LNS intervention arms combined and non-LNS intervention arms combined. ASQ:I, Ages and Stages Questionnaire Inventory; DMC, Developmental Milestones Checklist; EASQ, Extended Ages and Stages Questionnaire; LNS, lipid-based nutrient supplement; MDAT, Malawi Developmental Assessment Tool; PR, prevalence ratio; PSED, Profile of Social and Emotional Development; SOC, standard of care; WSH, water, sanitation, and hygiene intervention.

**FIGURE 4:**
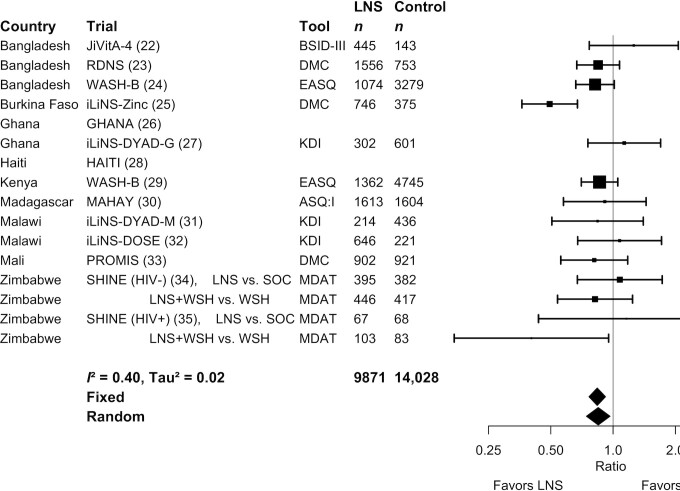
Forest plot of the effect of small-quantity LNSs on the prevalence of children in the lowest decile of motor scores. Individual study estimates were generated from log-binomial regression controlling for baseline measure when available and with clustered observations using robust SEs for cluster-randomized trials. Pooled estimates were generated using inverse-variance weighting in both fixed- and random-effects models. Individual trial estimates for the SHINE trial are split by comparison in the figure to reflect the crossover design. For calculating the pooled estimates, the trial was analyzed with LNS intervention arms combined and non-LNS intervention arms combined. ASQ:I, Ages and Stages Questionnaire Inventory; BSID, Bayley Scales of Infant Development; DMC, Developmental Milestones Checklist; EASQ, Extended Ages and Stages Questionnaire; KDI, Kilifi Developmental Inventory; LNS, lipid-based nutrient supplement; MDAT, Malawi Developmental Assessment Tool; PR, prevalence ratio; SOC, standard of care; WSH, water, sanitation, and hygiene intervention.

**FIGURE 5:**
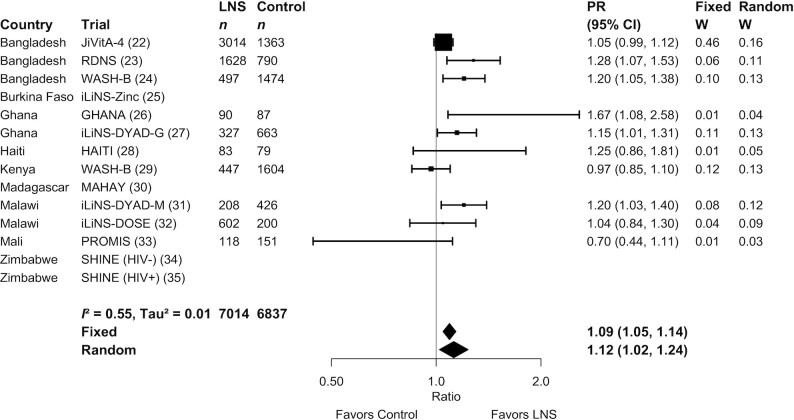
Forest plot of the effect of SQ-LNSs on the prevalence of children walking without support at age 12 mo. Individual study estimates were generated from log-binomial regression controlling for baseline measure when available and with clustered observations using robust SEs for cluster-randomized trials. Pooled estimates were generated using inverse-variance weighting in both fixed- and random-effects models. LNS, lipid-based nutrient supplement; PR, prevalence ratio.

**FIGURE 6:**
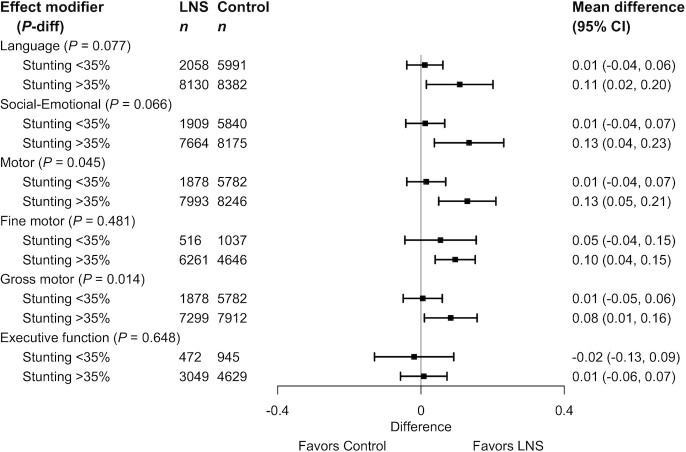
Pooled effects of SQ-LNSs on all continuous developmental outcomes stratified by study-level stunting burden of children at age 18 mo in control groups. Individual study estimates for interaction effect were generated from log-binomial regression controlling for baseline measure when available and with clustered observations using robust SEs for cluster-randomized trials. Pooled subgroup estimates and statistical testing of the pooled interaction term were generated using inverse-variance weighting random effects. LNS, lipid-based nutrient supplement; *P*-diff, *P* value for the difference in effects of small-quantity lipid-based nutrient supplements between the 2 levels of the effect modifier.

**Table:** 

Study-level effect modifiers^2^	Individual-level effect modifiers
• Geographic region (WHO region: Sub-Saharan Africa vs. Southeast Asia Region)• Stunting burden among control group children at 18 mo of age (≥35% vs. <35%)^3^• Malaria prevalence (country-specific, closest in time to the study: ≥10% vs. <10%)^4^• Anemia prevalence (country-specific, closest in time to the study: ≥60% vs. <60%)^5^• Source water quality (study-specific, <75% vs. ≥75% prevalence of improved drinking water)^6^• Sanitation (study-specific, <50% vs. ≥50% prevalence of improved sanitation)^7^• Duration of child supplementation (study target: >12 mo vs. ≤12 mo)• Child age at baseline or endline• Frequency of contact for intervention delivery or outcome assessments during the study (weekly vs. monthly)• Compliance (mean percentage compliance in SQ-LNS group: ≥80% vs. <80%)^8^	• Maternal height (<150.1 cm vs. ≥150.1 cm)^9^• Maternal BMI (<20 kg/m^2^ vs. ≥20 kg/m^2^)• Maternal age (<25 y vs. ≥25 y)• Maternal education (no formal or incomplete primary vs. complete primary or greater)• Maternal depressive symptoms (< study 75th percentile vs. ≥ study 75th percentile)^10^• Child sex (female vs. male)• Child birth order (firstborn vs. later-born)• Child baseline stunted (length-for-age *z* score < −2 SD vs. ≥ −2)• Child baseline acute malnutrition (weight-for-length *z* score < −2 SD or midupper arm circumference < 125 mm)• Child baseline anemia (Hb < 110 g/L vs. ≥110 g/L)• Household socioeconomic status (< study median vs. ≥ study median)^11^• Household food security (moderate to severe food insecurity vs. secure to mild food insecurity)^12^• Household source water quality (unimproved vs. improved)^6^• Household sanitation (unimproved vs. improved)^7^• Home environment (< study median vs. ≥ study median)^13^• Season at the time of developmental outcome assessment (rainy vs. dry)^14^

1 Comparisons follow the format nonreference vs. reference category. Hb, hemoglobin; SQ-LNS, small-quantity lipid-based nutrient supplement.

2 We identified potential study-level effect modifiers before receipt of data, and categorized individual studies based on the distribution of effect modifier values across all studies before conducting hypothesis-testing.

3 Based on 18-mo data because baseline data were not available for all trials; cutoff chosen at approximately the median across trials.

4 World Malaria Report 2018 (74); cutoff chosen based on the median across trials.

5 Country-specific prevalence of anemia among children age 6–59 mo, based on national surveys (see Supplemental Table 3).

6 Improved water source includes piped water, boreholes or tubewells, protected dug wells or springs, rainwater, and packaged or delivered water (see Supplemental Table 3) (75); based on baseline data, excluding arms that received water, sanitation, and hygiene interventions; cutoff chosen at approximately the median across trials.

7 Improved sanitation includes flush/pour flush to piped sewer system, septic tanks, or pit latrines; ventilated improved pit latrines, composting toilets, or pit latrines with slabs (see Supplemental Table 3) (76); cutoff chosen at approximately the median across trials.

8 Study-specific, as reported based on a study-defined indicator (see Supplemental Table 3).

9 Cutoff based on −2 SD for height at 19 y of age at https://www.who.int/growthref/hfa_girls_5_19years_z.pdf?ua=1.

10 Study-specific (see Supplemental Table 3); cutoff chosen to reflect the top quartile for risk of depression.

11 Based on a study-defined, study-specific assets index.

12 Study-specific (see Supplemental Table 3).

13 As measured by the Family Care Indicators, Home Observation for the Measurement of the Environment Inventory, or other similar tools (see Supplemental Table 3).

14 Rainy compared with dry, based on study- and child-specific average rainfall during the month of measurement and 2 mo prior (see Supplemental Table 3).

### IPD integrity

We checked data for completeness by ensuring that the study sample sizes in our pooled data set were the same as in study protocols and publications. We also checked summary statistics, such as means and SDs, in our data set against published values for each trial to ensure consistency. Implausible values were inspected for errors and truncated to 5 or −5 SD from the mean *z* score (≤0.2% of values for each outcome, with the highest percentage for motor, gross, and fine motor scores: 0.18–0.20%).

### Assessment of risk of bias and quality of evidence in each study and across studies

Independent reviewers (KRW, CDA, ELP) assessed risk of bias in each trial against the following criteria: random sequence generation and allocation concealment (selection bias), blinding of participants and personnel (performance bias), blinding of outcome assessment (detection bias), incomplete outcome data (attrition bias),  selective  reporting   (reporting  bias),    and   other sources of bias (13) (**Supplemental Figure 1**). Any discrepancies were resolved by discussion or consultation with the core working group, as needed. To assess risk of bias across studies, for each outcome that was measured in a subset of studies, we compared study-level maternal education (percentage who completed primary) and child 18-mo stunting burden to check for substantial differences between the trials included and trials excluded. The same reviewers also assessed the quality of evidence for each outcome across all studies based on the 5 Grading of Recommendations Assessment, Development and Evaluation (GRADE) criteria: risk of bias, inconsistency of effect, imprecision, indirectness, and publication bias (14).

### Specification of outcomes and effect measures

The following primary and secondary outcomes were prespecified in the analysis plan. Primary outcomes were language, motor, and social-emotional *z* scores reported on a continuous scale; whether the child was in the lowest decile of continuous language *z* scores, motor *z* scores, and social-emotional *z* scores; and walking without support at 12 mo. Secondary outcomes were continuous gross and fine motor *z* scores if these were reported separately, continuous executive function *z* scores and whether the child was in the lowest decile of executive function *z* scores, plus whether the child had achieved 4 motor milestones at 12 mo (crawling, standing with support, standing without support, and walking with support) and 5 motor milestones at 18 mo (the same 4 plus walking without support).

*z* Scores were standardized within each study by regressing the unstandardized developmental score on child age and sex and calculating the standardized residuals. This approach is analogous to calculating length-for-age *z* score (LAZ) in that the score represents deviations from the mean score for a given child's age and sex in units of SD. However, developmental outcome *z* scores were calculated in reference to each within-study distribution, rather than an external standard. For example, a female child with a language *z* score of −1 scored 1 SD below the mean of other female children of the same age in her study sample.

The lowest decile of each *z* score was also defined for each study based on the within-study distribution. Given that most of the developmental assessment tools used in these studies do not have validated cutoffs to identify children at risk of delayed neurodevelopment, we used the lowest decile of scores as a proxy for children who may be at the greatest risk of experiencing developmental delay. We thus considered the lowest decile of scores to be an adverse developmental outcome. We selected the lowest decile as the cutoff because it is sufficiently low to capture poor development in populations that as a whole may lag behind populations with no environmental constraints on achieving developmental potential, and high enough to allow adequate power to detect group differences. In addition, some standard developmental assessments have used the lowest decile to define delay, for example, the MacArthur-Bates Communicative Development Inventory (CDI), which was adapted to assess language in 6 of the studies included in this IPD meta-analysis (15).

If any study used multiple tools or scores to assess the same domain at endline (e.g., language), we selected the tool or score that was used in the greatest number of other studies included in this IPD meta-analysis. For social-emotional scores, if any study reported a social-emotional difficulties score for which a higher score indicated greater problems, we reversed those scores so that for all scores, a higher score represented greater social-emotional competence. For the milestone assessment, we only used reports or observations of the child's ability on the day of assessment, not retrospective reports of age of milestone achievement, because the latter is subject to potential recall inaccuracy. We used milestone data collected within 1 mo of the target age (12 or 18 mo). If both observation and parent-report data existed at the same time point, we used observation data.

The principal measure of effect for continuous outcomes was the mean difference between intervention and comparison groups at endline, defined as the principal postintervention time point as reported for trials with infrequent child assessment or at the age closest to the end of the supplementation period for trials with monthly child assessment. The principal measure of effect for binary outcomes was the prevalence ratio at endline or at the targeted age of milestone assessment (12 or 18 mo). We also estimated prevalence differences as secondary assessments of binary outcomes. Prevalence ratios quantify the relative difference in proportions between groups, whereas prevalence differences are the difference in absolute percentage points. Prevalence differences are less consistent than prevalence ratios (13); however, they are important for estimating the public health impact of an intervention.

The treatment and comparisons of interest were provision of children with SQ-LNSs (< ∼125 kcal/d, with or without co-interventions), compared with children who received no intervention or an intervention without any type of LNS or other child nutritional supplement (herein labeled “control”). Examples of other types of interventions that have been delivered with or without LNSs are water, sanitation, and hygiene (WASH) interventions or child morbidity monitoring and treatment. In several trials, child LNS has been delivered to children whose mothers received maternal LNS during pregnancy and postpartum. Given that maternal supplementation may have an additive effect, we originally planned to include trial arms that provided both maternal and child LNSs in a sensitivity analysis only (i.e., the all-trials analysis). However, to maximize study inclusion and participant sample size, and to allow for sufficient numbers of trials to examine effect modification for certain outcomes, we decided after initial registration of the protocol but before completing statistical analyses that the results of the all-trials analysis would be presented as the principal findings if the following criteria were met, as determined for each outcome: if the main effects did not differ between the child-LNS-only analysis (excluding maternal plus child LNS arms) and the all-trials analysis (including maternal plus child LNS arms) by >20% for continuous outcomes or by >0.05 for prevalence ratios. Two additional sensitivity analyses were also conducted, as described below.

### Synthesis methods and exploration of variation in effects

We conducted 3 types of analyses, corresponding to the 3 objectives, to investigate *1*) full-sample main effects of the intervention, *2*) effect modification by study-level characteristics, and *3*) effect modification by individual-level characteristics. We used a 2-stage approach for all analyses. This approach is preferred when incorporating cluster-randomized trials because it allows intracluster correlations to be study-specific (8). All analyses followed a complete-case intention-to-treat framework (16).

In the first stage, we estimated intervention compared with control group effects (mean differences or prevalence ratios) within each individual study. Given that continuous outcomes represented deviations from the study sample mean score for a given child's age and sex in units of SD, these first-stage individual study estimates represent mean differences between SQ-LNS and control groups in units of SD. For longitudinal study designs that provided baseline developmental assessment data, we adjusted for baseline score when estimating the intervention effect. For cluster-randomized trials, we used robust SEs to account for participant dependence within clusters.

In the second stage, first-stage estimates were pooled using inverse-variance weighted fixed effects. A fixed-effect approach generates estimates viewed as a typical intervention effect from the studies included in the analysis. This was prespecified in our statistical analysis plan because we anticipated similar intervention effects and similar individual-level effect modification patterns across studies. As a robustness check of this assumption, we also conducted a sensitivity analysis calculating pooled estimates using inverse-variance weighted random effects (17, 18). If there were <3 comparisons to include in a pooled estimate then the pooled estimate was not generated (e.g., if <3 comparisons were represented within a study-level effect modification category). This was the case for most of the milestones, therefore we did not examine study-level or individual-level effect modification for any of the 9 individual milestones specified as secondary outcomes.

For Objective 1, we pooled the first-stage estimates to generate a pooled point estimate, 95% CI, and corresponding *P* value. For Objective 2, we used a bivariate random-effects meta-regression to test the association of study-level characteristics with study intervention effect estimates. For Objective 3, we first estimated the parameter corresponding to the interaction term of the effect modifier and the intervention for each study (19). We then generated pooled intervention effect estimates within each category of the effect modifier to determine how the intervention effect in one subgroup differed from the intervention effect in the specified reference subgroup. For further details, see Dewey et al. (12).

Heterogeneity of effect estimates was assessed using *I*^2^ and Tau^2^ statistics, within strata when relevant (20). We used a *P* value of <0.05 for main effects and a *P*-for-interaction < 0.10 for effect modification. Given that developmental outcomes are interrelated and the effect modification analyses are inherently exploratory, we did not adjust for multiple hypothesis testing because doing so may be unnecessary and counterproductive owing to increasing the likelihood of type 2 error (21).

### Sensitivity analyses

Two sensitivity analyses were conducted in addition to those aforementioned (the child-LNS only analysis, all-trials analysis, and fixed- and random-effects models). First, we excluded passive control arms, defined as groups of participants who received no intervention and had no contact with project staff between enrollment and endline. Second, we separated comparisons within trials that included multicomponent interventions to attempt to isolate the effect of SQ-LNSs. For example, if a trial provided a water intervention to one group, a water plus sanitation intervention to a second group, and a water and sanitation plus SQ-LNS intervention to a third group, this sensitivity analysis would only compare the water and sanitation plus SQ-LNS arm with the water and sanitation arm. The all-trials analysis and child-LNS-only analysis would include both groups that did not receive SQ-LNSs in the comparison group, whereas the sensitivity analysis would exclude the group that received the water intervention only. Behavior change communication and other messaging promoting recommended infant and young child feeding (IYCF) practices were not considered additional components. **Supplemental Table 1** lists all trial arms and specifies which comparisons were made in each sensitivity analysis.

In addition, we conducted post hoc analyses to examine effects within subgroups of trials based on 2 aspects of the intervention design: *1*) whether the trial was or was not conducted within an existing program, and *2*) the extent of the social and behavior change communication on IYCF that was provided (minimal compared with expanded).

## Results

### Literature search and trial characteristics

Of the 1466 publications identified through the search strategy and review of other meta-analyses and systematic reviews, 90 titles and abstracts were identified as relevant. Based on review of the full texts, 14 trials met the inclusion criteria and IPD were requested (22–36). Investigators for 1 trial were unable to participate (36). In that trial, only fine and gross motor outcomes were reported, therefore we examined pooled main effects on these 2 outcomes both without and with this trial, by calculating Hedges’ *g* (37) based on endline values extracted from the published report. For all other analyses, 13 trials were included in the IPD meta-analysis for developmental outcomes (22, 26, 30, 33, 38–47) (**Figure 1**). One trial reported child development as a primary outcome (29), and the other 12 reported child development as secondary outcomes. One trial, SHINE in Zimbabwe (35, 46), contributed 2 comparisons because it was designed a priori to report results separately for HIV-exposed and HIV-unexposed children. Thus, 14 SQ-LNS against control group comparisons from 13 trials were analyzed. Of the 14 comparisons, language outcomes were reported for 13 comparisons, motor outcomes for 12, social-emotional outcomes for 11, executive function for 7, and various motor milestones for 7–10 comparisons.

The included trials were conducted in Bangladesh (3 trials), Burkina Faso (1 trial), Ghana (2 trials), Haiti (1 trial), Kenya (1 trial), Madagascar (1 trial), Malawi (2 trials), Mali (1 trial), and Zimbabwe (1 trial). Child SQ-LNS was given starting at age 6 mo in 11 trials, 6–11 mo in 2 trials, and 9 mo in 1 trial (**Table 1**). Duration of supplementation ranged from 6 to 18 mo. Four trials (23, 27, 30, 31) included intervention arms that provided SQ-LNSs to mothers during pregnancy and/or the first 6 mo postpartum. The majority of trials provided a peanut- and milk-based SQ-LNS providing ∼120 kcal/d and 1 RDA of most micronutrients (for further details see **Supplemental Table 2**). Two trials targeted a subsample of children for developmental assessment (methods described in Supplemental Table 1).

**TABLE 1:** Characteristics of trials included in the individual participant data analysis^1^

						Participants, *n*	
			Infant SQ-LNS supplement		Maternal LNS supplement	Any growth, developmental, or	Any developmental
Country	References	Trial name	Age at start, mo	Duration, mo		biomarker outcome	outcome
Bangladesh	Christian et al. (22)	JiVitA-4	6	12	N	4568	4558
Bangladesh	Dewey et al. (23), Matias et al. (38)	RDNS	6	18	Y/N	2567	2565
Bangladesh	Luby et al. (24), Tofail et al. (39)	WASH B-B	6	18	N	4824	4572
Burkina Faso	Hess et al. (25), Prado et al. (40)	iLiNS-Zinc	9	9	N	2647	1176
Ghana	Adu-Afarwuah et al. (26)	GHANA	6	12	Y	194	194
Ghana	Adu-Afarwuah et al. (27, 41)	iLiNS-DYAD-G	6	6	N	1113	1103
Haiti	Iannotti et al. (28, 42)	HAITI	6–11	3–6	N	322	322
Kenya	Null et al. (29), Stewart et al. (43)	WASH B-K	6	18	N	6815	6756
Madagascar	Galasso et al. (30)	MAHAY	6–11	6–12	Y/N	3438	3217
Malawi	Ashorn et al. (31), Prado et al. 44)	iLiNS-DYAD-M	6	12	Y	675	674
Malawi	Maleta et al. (32), Prado et al. (45)	iLiNS-Dose	6	12	N	999	999
Mali	Huybregts et al. (33)	PROMIS	6	18	N	1927^2^	1927
Zimbabwe	Humphrey et al. (34), Gladstone et al. (46)	SHINE	6	12	N	4347	1961
	Prendergast et al. (35), Chandna et al. (47)						

1 LNS, lipid-based nutrient supplement; SQ, small-quantity.

2 Developmental assessments were conducted only in the cross-sectional sample, not the longitudinal sample, therefore the longitudinal sample was not included in the participant count.

The most commonly used developmental assessment tools were the CDI vocabulary checklist to assess language (6 trials) (Supplemental Table 1) and the A not B task to assess executive function (6 trials; all trials that measured executive function used this same task). Other tools used were the Developmental Milestones Checklist (DMC; 3 trials), Extended Ages and Stages Questionnaire (EASQ; 2 trials), Ages and Stages Questionnaire: Inventory (ASQ:I; 1 trial), Kilifi Development Inventory (KDI; 3 trials), Malawi Developmental Assessment Tool (MDAT; 1 trial), and Bayley Scales of Infant Development-III (BSID; 1 trial). All endline assessments were conducted when the children were age 12–24 mo. In this age range, all of these tools assess similar developmental skills and many items overlap between the tools. Parent-report was used to assess social-emotional development in all studies and to assess language in all studies except 1. Direct child assessment was used to assess executive function in all studies. Motor development was assessed by parent-report in 6 studies and direct child assessment in 6 studies.

All potential study-level and individual-level effect modifiers showed substantial variation between trials (**Supplemental Tables 3**, **4**). For example, at the study level, 8 study sites had a high burden of stunting (≥35% at 18 mo) and 5 had lower rates of stunting (<35% at 18 mo). Study-specific prevalence of improved water quality ranged from 27% to 100%, and prevalence of improved sanitation ranged from 0% to 97%. Frequency of contact during the study was weekly in 7 trials and monthly in 6 trials. Mean estimated reported compliance with SQ-LNS consumption was categorized as high (≥80%) in 7 trials and lower than that in the other trials.

### Main effects of SQ-LNSs on developmental outcomes

Results from the child-LNS-only and all-trials analyses were similar: for nearly all outcomes, the mean differences, prevalence ratios, and prevalence differences for intervention against control group comparisons were almost identical or slightly less favorable when the maternal LNS arms were included (**Supplemental Figure 2**A–G). Therefore, **Table 2** presents results from the all-trials analyses, inclusive of maternal plus child LNS trials and arms. SQ-LNSs had a significant positive effect on all primary developmental outcomes, with effect sizes of 0.07–0.08 SD in mean language, social-emotional, and motor scores (Table 2, **Supplemental Figure 3**A, B), and relative reductions in the percentage of children in the lowest decile of these scores ranging from 16% to 19% (Table 2, **Figures 2** –**4**). For the prevalence of children walking without support at 12 mo, there was a relative increase of 9% (4 percentage point difference) (Table 2, **Figure 5**). In the JiVitA-4 trial, milestone data were collected using monthly surveillance rather than data collection at a single time point, the method used in all other trials. This trial also contributed 30% of the total sample size. Therefore, we conducted a sensitivity analysis excluding the data from this trial and found a similar estimate of an increase of 13% (95% CI: 1.07, 1.20) in the prevalence of children walking without support at 12 mo.

**TABLE 2:** Pooled fixed-effects estimates of the effect of randomized controlled trials of small-quantity lipid-based nutrient supplements provided to infants and young children age 6–24 mo, compared with children who received no intervention or an intervention without any nutritional supplement, on developmental outcomes^1^

Outcome	*n* participants (*n* intervention vs. control group comparisons)	Comparison (95% CI)	*P* value	Heterogeneity *I*^2^ (*P*-for-heterogeneity)^2^	Quality of the evidence (GRADE)
Continuous outcomes: MDs					
Language *z* score MD^3^	24,561 (13)	0.07 (0.04, 0.10)	<0.001	0.64 (0.001)	High
Social-emotional *z* score MD^3^	23,588 (11)	0.08 (0.05, 0.11)	<0.001	0.66 (0.001)	High
Motor *z* score MD^3^	23,899 (12)	0.08 (0.05, 0.11)	<0.001	0.60 (<0.001)	High
Gross motor *z* score MD	22,871 (11)	0.06 (0.03, 0.09)	<0.001	0.52 (<0.001)	High
Fine motor *z* score MD	12,460 (9)	0.09 (0.04, 0.13)	0.001	0.00 (0.989)	High
Executive function *z* score MD	9095 (7)	0.00 (−0.04, 0.05)	0.855	0.04 (0.395)	High
Binary outcomes: PRs					
Language lowest decile PR^3^	24,561 (13)	0.84 (0.76, 0.92)	<0.001	0.61 (0.004)	High
Social-emotional lowest decile PR^3^	23,588 (11)	0.81 (0.74, 0.89)	<0.001	0.54 (0.001)	High
Motor lowest decile PR^3^	23,899 (12)	0.84 (0.76, 0.92)	<0.001	0.40 (0.027)	High
Executive function lowest decile PR	9095 (7)	0.93 (0.81, 1.06)	0.273	0.16 (0.272)	High
12-mo milestones					
Walking without support PR^3^	13,851 (10)	1.09 (1.05, 1.14)	<0.001	0.55 (0.022)	High
Walking with support PR	13,729 (9)	1.00 (1.00, 1.01)	0.051	0.41 (0.122)	High
Standing without support PR	13,891 (10)	1.03 (1.00, 1.05)	0.021	0.65 (0.008)	High
Standing with support PR	13,838 (9)	1.00 (1.00, 1.00)	0.229	0.00 (0.869)	High
Crawling PR	13,488 (9)	1.00 (1.00, 1.01)	0.320	0.26 (0.249)	High
18-mo milestones					
Walking without support PR	6437 (7)	1.00 (0.99, 1.01)	0.485	0.28 (0.233)	High
Walking with support PR	—	—	—	—	—
Standing without support PR	6437 (7)	1.00 (1.00, 1.01)	0.207	0.00 (0.527)	High
Standing with support PR	—	—	—	—	—
Crawling PR	—	—	—	—	—
Binary outcomes: PDs					
Language lowest decile PD	24,561 (13)	−0.01 (−0.02, 0.00)	0.005	0.66 (<0.001)	High
Social-emotional lowest decile PD	23,588 (11)	−0.02 (−0.02, −0.01)	<0.001	0.68 (0.001)	High
Motor lowest decile PD	23,899 (12)	−0.02 (−0.02, −0.01)	<0.001	0.25 (0.195)	High
Executive function lowest decile PD	9095 (7)	−0.01 (−0.02, 0.01)	0.293	0.21 (0.293)	High
12-mo milestones					
Walking without support PD	13,851 (10)	0.04 (0.02, 0.06)	<0.001	0.57 (0.012)	High
Walking with support PD	13,729 (9)	0.00 (0.00, 0.01)	0.039	0.42 (0.086)	High
Standing without support PD	13,891 (10)	0.02 (0.01, 0.04)	0.006	0.56 (0.015)	High
Standing with support PD	13,838 (9)	0.00 (0.00, 0.00)	0.211	0.00 (0.801)	High
Crawling PD	13,488 (9)	0.00 (0.00, 0.01)	0.267	0.22 (0.243)	High
18-mo milestones					
Walking without support PD	6437 (7)	0.00 (0.00, 0.01)		0.40 (0.126)	High
Standing without support PD	6437 (7)	0.00 (0.00, 0.01)		0.00 (0.607)	High

1 GRADE, Grading of Recommendations Assessment, Development and Evaluation; MD, mean difference; PD, prevalence difference; PR, prevalence ratio.

2 *I* ^2^ describes the percentage of variability in effect estimates that may be due to heterogeneity rather than chance. Roughly, 0.3–0.6 may be considered moderate heterogeneity. *P* value from chi-squared test for heterogeneity. *P* < 0.05 indicates statistically significant evidence of heterogeneity of intervention effects beyond chance.

3 Primary outcome.

For the secondary outcomes, SQ-LNSs had a significant positive effect on mean gross and fine motor scores of 0.06 and 0.09 SD, respectively, but no significant effect on executive function mean or percentage in the lowest decile. Including the effect estimates from the published report by Smuts et al. (36), for which IPD were not available, results were similar (gross motor: 0.06; 95% CI: 0.03, 0.09; fine motor: 0.09; 95% CI: 0.04, 0.13). For consistency with other analyses, Table 2 reports the estimates excluding this trial by Smuts et al. Including the JiVitA-4 data, SQ-LNSs increased the prevalence of children standing without support at age 12 mo by 3%, and no significant effects were found on any other milestones examined (Table 2). Excluding the JiVitA-4 data, SQ-LNSs increased the prevalence of children standing without support at age 12 mo by 6% (95% CI: 1.02, 1.08). Pooled estimates were not generated for 3 of the 5 milestones at 18 mo owing to lack of variance, because almost all children had attained the milestones by this age (see **Supplemental Table 5** for the percentage of children in the control arms who attained each milestone at 12 and 18 mo in each trial).

Supplemental Figure 2A–G shows the results of all 8 analyses, that is, fixed- and random-effects models for each of the *1*) all-trials analysis, *2*) child-LNS-only analysis, *3*) sensitivity analysis excluding passive arms, and *4*) sensitivity analysis separating multicomponent arms to compare only pairs of arms that included the same nonnutrition components. Results were similar regardless of whether fixed-effects or random-effects models were used, although CIs were wider for the latter, as expected. Results were also similar in the sensitivity analyses. For example, across the 8 analyses, effect sizes on language scores ranged from 0.05 to 0.09 and reductions in the percentage of children in the lowest decile of language ranged from 11% to 20%.

In addition, effects of SQ-LNSs on the prevalence in the lowest decile of motor and social-emotional scores were evident in both the studies implemented through existing programs and those implemented by the research teams, although effects on language were smaller in studies implemented through existing programs (**Supplemental Figure 4**A–C). Effects were also evident when stratified by whether the trial reinforced the normal IYCF messages already promoted in that setting, or the trial provided expanded behavior change communication for IYCF in the SQ-LNS intervention arms. Effects on motor and social-emotional development were slightly smaller in the trials that provided expanded IYCF messages in both the intervention and control arms; however, there were only 2–3 trials in this group (**Supplemental Figure 5**A–C).

### Risk of bias and quality of evidence

In general, we rated individual trials as having low risk of bias, except for the lack of blinding of participants due to the nature of the intervention. Because of the latter, outcome assessment was not blinded when development was assessed by parent-report (language and social-emotional outcomes in most trials and motor outcomes in half of the trials) (**Supplemental Table 6**A–M, Supplemental Figure 1). In analyses that included a subset of studies, there was a mix of high and low child stunting burden and maternal educational levels among both the included studies and the excluded studies, with the following exceptions. For social-emotional outcomes, all of the 4 excluded studies were among the 7 studies in the category for higher maternal education (>50% completed primary). For motor outcomes, all of the 3 excluded studies were in the category for higher maternal education.

For all developmental outcomes, we rated the overall quality of evidence as high. All included studies were randomized controlled trials, therefore GRADE ratings started as high and we did not downgrade the quality of the evidence based on the following 5 criteria. *1*) Heterogeneity across trials was low to moderate (*I*^2^ = 0.00–0.60) for 20 outcomes and substantial (*I*^2^ = 0.61–0.68) for 5 outcomes (Table 2), therefore, inconsistency was not considered high enough to downgrade the quality of the evidence. *2*) Precision was rated as high because all but 2 trials had sample sizes >600. *3*) Directness was high because all trials were directly aimed at evaluating SQ-LNSs. *4*) Funnel plots revealed no indication of publication bias across studies. *5*) We did not consider risk of bias in individual studies high enough to downgrade the quality of the evidence. As aforementioned, the main potential source of bias was the lack of participant blinding and therefore the lack of blinding of outcome assessment when development was assessed by parent-report. Parent-report methods were used for language and social-emotional outcomes in most trials and motor outcomes in half of the trials. To explore this potential bias, we calculated pooled effect sizes for motor outcomes stratified by parent-report compared with directly observed assessments and found that effects of SQ-LNSs were larger among studies that used parent-report (0.13; 95% CI: 0.02, 0.23; 6 comparisons; compared with 0.07; 95% CI: −0.01, 0.15; 6 comparisons for direct child observation). However, 3 studies included in this IPD meta-analysis used direct observation for at least a subgroup of items or children to check the validity of the parent-report assessments and found similar intervention effects on observed motor and language outcomes compared with the corresponding parent-report outcomes (28, 43, 45). Although the parents may have reported more accurately because they knew their children were also being observed, the assessment methods were substantially different (e.g., a parent-report vocabulary checklist compared with the observed MDAT language subscale which assesses many different types of language skills). Thus, this consistency suggests that reporting bias did not account for the effects of SQ-LNSs, at least in those 3 trials. Given this evidence and given that the pooled effect size on observed motor outcomes (0.07) was in the same range as all primary outcome pooled effect sizes (0.06–0.08), we did not consider that this risk of bias was high enough to downgrade our confidence in the accuracy of the pooled estimates.

### Effect modification by study-level characteristics

Study-level effect modification results were consistent across all fixed- and random-effects analyses and across all sensitivity analyses (data not shown, available on request). The results presented below refer to the fixed-effects all-trials analysis. For some outcomes, we were unable to generate pooled estimates for effect modification by certain potential study-level effect modifiers because <3 comparisons were categorized into 1 of the study-level effect modification categories (e.g., social-emotional development by geographic region). We were unable to examine potential effect modification by child age at baseline because there was insufficient heterogeneity in this aspect of study design: most of the trials began supplementation at 6 mo of age.

The study-level stunting burden significantly modified the effect of SQ-LNSs on language, social-emotional, motor, and gross motor development. Among studies with higher 18-mo stunting burden in the control group (≥35%), effects on these developmental scores ranged from 0.08 to 0.13 SD, whereas effect sizes among studies with lower stunting burden (<35%) were 0.01 SD (**Table 3**, **Figure 6**). There was also a greater reduction in the prevalence of children in the lowest decile of language scores among studies with higher stunting burden (**Table 4**).

**TABLE 3:** Study-level effect modifiers of effects of SQ-LNSs provided to infants and young children age 6–24 mo on continuous developmental outcomes^1^

	Language		Social-emotional		Executive function		Motor		Fine motor		Gross motor	
	*n* (comp)	Pooled MD (95% CI)	*n* (comp)	Pooled MD (95% CI)	*n* (comp)	Pooled MD (95% CI)	*n* (comp)	Pooled MD (95% CI)	*n* (comp)	Pooled MD (95% CI)	*n* (comp)	Pooled MD (95% CI)
Geographic region		*P*-diff: 0.978		*P*-diff: —		*P*-diff: —		*P*-diff: 0.591		*P*-diff: —		*P*-diff: 0.929
Africa	16,735 (9)	0.08 (−0.01, 0.17)	16,784 (9)	0.10 (0.00, 0.20)	3872 (5)	−0.02 (−0.09, 0.05)	16,649 (9)	0.11 (0.03, 0.20)	9427 (7)	0.08 (0.02, 0.13)	15,602 (8)	0.06 (−0.01, 0.14)
Southeast Asia	7527 (3)	0.08 (−0.04, 0.21)	6804 (2)	—	5223 (2)	—	7250 (3)	0.09 (0.04, 0.14)	3033 (2)	—	7269 (3)	0.05 (−0.07, 0.16)
18-mo stunting burden, %		*P*-diff: 0.077		*P*-diff: 0.066		*P*-diff: —		*P*-diff: 0.045		*P*-diff: —		*P*-diff: 0.014
<35	8049 (4)	0.01 (−0.04, 0.06)	7749 (3)	0.01 (−0.04, 0.07)	1417 (2)	—	7660 (3)	0.01 (−0.04, 0.07)	1553 (2)	—	7660 **(**3**)**	0.01 (−0.05, 0.06)
≥35	16,512 (9)	0.11 (0.02, 0.20)	15,839 (8)	0.13 (0.04, 0.23)	7678 (5)	0.01 (−0.06, 0.07)	16,239 (9)	0.13 (0.05, 0.21)	10,907 (7)	0.10 (0.04, 0.15)	15,211 (8)	0.08 (0.01, 0.16)
Malaria prevalence, %		*P*-diff: 0.365		*P*-diff: 0.863		*P*-diff: 0.242		*P*-diff: 0.557		*P*-diff: 0.626		*P*-diff: 0.387
<10	19,060 (8)	0.05 (−0.03, 0.13)	18,089 (6)	0.09 (−0.01, 0.20)	7039 (4)	0.02 (−0.04, 0.08)	18,535 (7)	0.08 (0.02, 0.14)	8211 (5)	0.10 (0.03, 0.16)	18,554 (7)	0.08 (−0.01, 0.17)
≥10	5501 (5)	0.11 (−0.02, 0.25)	5499 (5)	0.11 (−0.02, 0.23)	2056 (3)	−0.04 (−0.14, 0.05)	5364 (5)	0.12 (−0.01, 0.26)	4249 (4)	0.07 (−0.01, 0.15)	4317 (4)	0.04 (−0.02, 0.10)
Anemia prevalence, %		*P*-diff: 0.530		*P*-diff: 0.496		*P*-diff: —		*P*-diff: 0.269		*P*-diff: 0.928		*P*-diff: 0.558
<60	19,750 (8)	0.06 (−0.01, 0.13)	19,078 (7)	0.08 (−0.01, 0.17)	7905 (5)	0.02 (−0.03, 0.07)	19,438 (8)	0.07 (0.02, 0.13)	9114 (6)	0.08 (0.03, 0.14)	19,457 (8)	0.07 (0.00, 0.15)
≥60	4811 (5)	0.10 (−0.06, 0.26)	4510 (4)	0.13 (−0.02, 0.29)	1190 (2)	—	4461 (4)	0.15 (−0.02, 0.32)	3346 (3)	0.09 (−0.01, 0.19)	3414 (3)	0.05 (−0.02, 0.12)
Source water quality		*P*-diff: 0.536		*P*-diff: 0.681		*P*-diff: —		*P*-diff: 0.530		*P*-diff: 0.234		*P*-diff: 0.777
<75% improved	9819 (6)	0.09 (−0.04, 0.22)	9843 (6)	0.11 (−0.02, 0.24)	847 (2)	—	9795 (6)	0.12 (−0.01, 0.24)	5958 (4)	0.01 (−0.09, 0.11)	8748 (5)	0.06 (0.01, 0.12)
≥75% improved	7520 (7)	0.05 (−0.03, 0.14)	6606 (5)	0.08 (0.00, 0.16)	4495 (5)	0.02 (−0.06, 0.09)	6944 (6)	0.08 (0.02, 0.14)	5453 (5)	0.09 (0.02, 0.15)	6963 (6)	0.04 (−0.04, 0.12)
Sanitation		*P*-diff: 0.991		*P*-diff: 0.660		*P*-diff: 0.012		*P*-diff: 0.963		*P*-diff: 0.364		*P*-diff: 0.276
<50% improved	9468 (7)	0.07 (−0.04, 0.18)	9490 (7)	0.08 (−0.03, 0.19)	2037 (4)	−0.09 (−0.18, 0.01)	9489 (7)	0.09 (−0.02, 0.19)	5646 (5)	0.04 (−0.04, 0.12)	8368 (6)	0.03 (−0.03, 0.09)
≥50% improved	7871 (6)	0.07 (−0.03, 0.17)	6959 (4)	0.11 (0.02, 0.20)	3305 (3)	0.06 (−0.01, 0.13)	7250 (5)	0.10 (0.03, 0.17)	5765 (4)	0.09 (0.02, 0.16)	7343 (5)	0.07 (−0.02, 0.16)
Supplement duration, mo		*P*-diff: 0.524		*P*-diff: 0.702		*P*-diff: —		*P*-diff: 0.621		*P*-diff: —		*P*-diff: 0.643
≤12	9644 (9)	0.05 (−0.05, 0.16)	8806 (7)	0.11 (−0.01, 0.24)	3872 (5)	−0.02 (−0.09, 0.05)	9307 (8)	0.11 (0.01, 0.22)	8186 (7)	0.08 (0.02, 0.14)	8186 (7)	0.05 (−0.05, 0.15)
>12	14,917 (4)	0.09 (0.02, 0.16)	14,782 (4)	0.07 (0.01, 0.13)	5223 (2)	—	14,592 (4)	0.07 (0.02, 0.13)	4274 (2)	—	14,685 (4)	0.07 (0.03, 0.11)
Frequency of contact		*P*-diff: 0.853		*P*-diff: 0.897		*P*-diff: 0.192		*P*-diff: 0.932		*P*-diff: 0.791		*P*-diff: 0.150
Weekly	8680 (6)	0.08 (−0.04, 0.20)	7960 (5)	0.10 (−0.02, 0.23)	6480 (4)	−0.02 (−0.07, 0.04)	8482 (6)	0.10 (−0.02, 0.21)	3008 (4)	0.08 (0.00, 0.15)	7361 (5)	0.02 (−0.05, 0.09)
Monthly	15,881 (7)	0.07 (−0.03, 0.16)	15,628 (6)	0.09 (−0.01, 0.20)	2615 (3)	0.04 (−0.05, 0.13)	15,417 (6)	0.09 (0.03, 0.16)	9452 (5)	0.09 (0.03, 0.15)	15,510 (6)	0.10 (0.02, 0.17)
Mean SQ-LNS compliance, %		*P*-diff: 0.918		*P*-diff: 0.863		*P*-diff: —		*P*-diff: 0.898		*P*-diff: —		*P*-diff: 0.525
<80	6290 (6)	0.07 (0.01, 0.13)	6339 (6)	0.10 (0.01, 0.20)	3872 (5)	−0.02 (−0.09, 0.05)	6204 (6)	0.09 (0.02, 0.16)	6210 (6)	0.08 (0.02, 0.14)	6278 (6)	0.08 (−0.02, 0.18)
≥80	15,054 (6)	0.07 (0.01, 0.13)	14,032 (4)	0.10 (0.01, 0.20)	5223 (2)	—	14,478 (5)	0.09 (0.02, 0.16)	3033 (2)	—	13,376 (4)	0.08 (−0.02, 0.18)

1 comp, number of intervention against control group comparisons; MD, mean difference in random-effects models; *n*, number of individual participants; *P*-diff, *P* value for the difference in effects of small-quantity lipid-based nutrient supplements between the 2 levels of the effect modifier in random-effects models; SQ-LNS, small-quantity lipid-based nutrient supplement.

**TABLE 4:** Study-level effect modifiers of effects of SQ-LNSs provided to infants and young children age 6–24 mo on binary developmental outcomes^1^

	Language lowest decile		Social-emotional lowest decile		Executive function lowest decile		Motor lowest decile		Walking without support at 12 mo	
	*n* (comp)	Pooled PR (95% CI)	*n* (comp)	Pooled PR (95% CI)	*n* (comp)	Pooled PR (95% CI)	*n* (comp)	Pooled PR (95% CI)	*n* (comp)	Pooled PR (95% CI)
Geographic region		*P*-diff: 0.624		*P*-diff: —		*P*-diff: —		*P*-diff: 0.610		*P*-diff: 0.525
Africa	16,735 (9)	0.84 (0.70, 1.02)	16,784 (9)	0.79 (0.63, 1.00)	3872 (5)	0.97 (0.72, 1.31)	16,649 (9)	0.83 (0.70, 0.98)	4923 (6)	1.09 (0.91, 1.30)
Southeast Asia	7527 (3)	0.77 (0.66, 0.90)	6804 (2)	—	5223 (2)	—	7250 (3)	0.88 (0.72, 1.08)	8766 (3)	1.15 (1.02, 1.29)
18-mo stunting burden, %		*P*-diff: 0.046		*P*-diff: 0.228		*P*-diff: —		*P*-diff: 0.466		*P*-diff: 0.711
<35	8049 (4)	1.16 (0.73, 1.83)	7749 (3)	0.91 (0.77, 1.08)	1417 (2)	—	7660 (3)	0.90 (0.76, 1.07)	4014 (5)	1.15 (1.00, 1.33)
≥35	16,512 (9)	0.78 (0.66, 0.92)	15,839 (8)	0.75 (0.59, 0.95)	7678 (5)	1.00 (0.78, 1.27)	16,239 (9)	0.83 (0.70, 0.98)	9837 (5)	1.09 (0.94, 1.27)
Malaria prevalence, %		*P*-diff: 0.322		*P*-diff: 0.902		*P*-diff: 0.990		*P*-diff: 0.491		*P*-diff: 0.768
<10	19,060 (8)	0.93 (0.73, 1.18)	18,089 (6)	0.81 (0.67, 0.98)	7039 (4)	0.94 (0.75, 1.16)	18,535 (7)	0.87 (0.78, 0.97)	10,979 (5)	1.11 (1.00, 1.23)
≥10	5501 (5)	0.78 (0.58, 1.05)	5499 (5)	0.80 (0.56, 1.12)	2056 (3)	0.95 (0.64, 1.41)	5364 (5)	0.82 (0.60, 1.10)	2872 (5)	1.12 (0.90, 1.41)
Anemia prevalence, %		*P*-diff: 0.644		*P*-diff: 0.534		*P*-diff: —		*P*-diff:0.093		*P*-diff: 0.720
<60	19,750 (8)	0.87 (0.78, 0.97)	19,078 (7)	0.83 (0.71, 0.98)	7905 (5)	0.91 (0.77, 1.07)	19,438 (8)	0.88 (0.79, 0.98)	11,807 (5)	1.11 (1.01, 1.22)
≥60	4811 (5)	0.90 (0.53, 1.55)	4510 (4)	0.76 (0.49, 1.15)	1190 (2)	—	4461 (4)	0.75 (0.54, 1.05)	2044 (5)	1.14 (0.89, 1.45)
Source water quality		*P*-diff: 0.737		*P*-diff: 0.394		*P*-diff: —		*P*-diff: 0.303		*P*-diff: —
<75% improved	9819 (6)	0.85 (0.63, 1.15)	9708 (5)	0.77 (0.55, 1.06)	847 (2)	—	9795 (6)	0.82 (0.64, 1.05)	1177 (2)	—
≥75% improved	7520 (7)	0.93 (0.68, 1.26)	6606 (5)	0.87 (0.74, 1.02)	4495 (5)	0.85 (0.69, 1.03)	6944 (6)	0.93 (0.80, 1.08)	10,284 (8)	1.14 (1.07, 1.23)
Sanitation		*P*-diff: 0.994		*P*-diff: 0.888		*P*-diff: 0.286		*P*-diff: 0.599		*P*-diff: 0.631
<50% improved	9468 (7)	0.88 (0.68, 1.14)	9355 (6)	0.84 (0.62, 1.14)	2037 (4)	0.95 (0.72, 1.27)	9489 (7)	0.85 (0.68, 1.08)	2344 (3)	1.10 (0.98, 1.24)
≥50% improved	7871 (6)	0.91 (0.63, 1.33)	6959 (4)	0.81 (0.70, 0.95)	3305 (3)	0.79 (0.64, 0.97)	7250 (5)	0.90 (0.77, 1.05)	9117 (7)	1.15 (1.00, 1.33)
Supplement duration, mo		*P*-diff: 0.545		*P*-diff: 0.731		*P*-diff: —		*P*-diff: 0.839		*P*-diff: 0.618
≤12	9644 (9)	0.92 (0.69, 1.23)	8806 (7)	0.78 (0.57, 1.06)	3872 (5)	0.97 (0.72, 1.31)	9307 (8)	0.87 (0.69, 1.08)	7142 (6)	1.13 (1.03, 1.23)
>12	14,917 (4)	0.82 (0.73, 0.93)	14,782 (4)	0.83 (0.74, 0.94)	5223 (2)	—	14,592 (4)	0.84 (0.74, 0.94)	6709 (4)	1.06 (0.85, 1.34)
Frequency of contact		*P*-diff: 0.370		*P*-diff: 0.991		*P*-diff: 0.724		*P*-diff: 0.977		*P*-diff: 0.433
Weekly	8680 (6)	0.79 (0.62, 1.02)	7960 (5)	0.81 (0.57, 1.13)	6480 (4)	0.94 (0.77, 1.16)	8482 (6)	0.87 (0.66, 1.15)	8951 (6)	1.14 (1.05, 1.23)
Monthly	15,881 (7)	0.95 (0.71, 1.26)	15,628 (6)	0.81 (0.66, 0.98)	2615 (3)	0.96 (0.66, 1.41)	15,417 (6)	0.86 (0.76, 0.97)	4900 (4)	1.05 (0.83, 1.34)
Mean SQ-LNS compliance, %		*P*-diff: 0.616		*P*-diff: 0.575		*P*-diff: —		*P*-diff: 0.330		*P*-diff: 0.700
<80	6290 (6)	0.88 (0.75, 1.04)	6339 (6)	0.82 (0.65, 1.02)	3872 (5)	0.97 (0.72, 1.31)	6204 (6)	0.92 (0.78, 1.09)	2695 (4)	1.08 (0.90, 1.29)
≥80	15,054 (6)	0.88 (0.75, 1.04)	14,032 (4)	0.82 (0.65, 1.02)	5223 (2)	—	14,478 (5)	0.92 (0.78, 1.09)	11,156 (6)	1.08 (0.90, 1.29)

1 comp, number of intervention against control group comparisons; *n*, number of individual participants; *P*-diff, *P* value for the difference in effects of small-quantity lipid-based nutrient supplements between the 2 levels of the effect modifier in random-effects models; PR, prevalence ratio in random-effects models; SQ-LNS, small-quantity lipid-based nutrient supplement.

Anemia prevalence among children age 6–59 mo modified the effect of SQ-LNSs on the prevalence of children in the lowest decile of motor scores. In countries with ≥60% child anemia prevalence, SQ-LNSs reduced this adverse motor outcome by 25%, compared with a 12% reduction in countries with <60% anemia prevalence (Table 4).

Study-level sanitation (<50% compared with ≥50% prevalence of improved sanitation in the study sample) modified the effect of SQ-LNSs on executive function (Table 3). Among the 3 comparisons with higher prevalence of improved sanitation, the pooled effect of SQ-LNSs on executive function was 0.06 SD (95% CI: −0.01, 0.13 SD), whereas among the 4 comparisons with a lower prevalence of improved sanitation, the pooled effect size was −0.09 SD (95% CI: −0.18, 0.01 SD). No other study-level characteristics significantly modified effects of SQ-LNSs on any other developmental outcome (**Supplemental Figure 6**A–P).

### Effect modification by individual-level characteristics

Individual-level effect modification results were consistent across fixed- and random-effects models and across all sensitivity analyses (data not shown; available upon request). The results presented below refer to the fixed-effects all-trials analysis. The following individual-level characteristics did not significantly modify the effect of SQ-LNSs on any developmental outcome: indicators of household food insecurity, water quality, sanitation, and home environment; maternal BMI and depressive symptoms; child sex; and season at outcome assessment (**Tables 5**, **6**, **Supplemental Figures 7**, **8**).

**TABLE 5:** Individual-level effect modifiers of effects of SQ-LNSs provided to infants and young children age 6–24 mo on continuous developmental outcomes^1^

	Language		Social-emotional		Executive function		Motor		Fine motor		Gross motor	
	*n* (comp)	Pooled MD (95% CI)	*n* (comp)	Pooled MD (95% CI)	*n* (comp)	Pooled MD (95% CI)	*n* (comp)	Pooled MD (95% CI)	*n* (comp)	Pooled MD (95% CI)	*n* (comp)	Pooled MD (95% CI)
Maternal height, cm	23,010 (11)	*P*-int: 0.857	22,926 (11)	*P*-int: 0.511	8959 (7)	*P*-int: 0.522	22,662 (11)	*P*-int: 0.457	11,533 (8)	*P*-int: 0.069	21,633 (10)	*P*-int: 0.787
<150.1		0.08 (0.01, 0.15)		0.09 (0.03, 0.16)		−0.02 (−0.11, 0.07)		0.09 (0.03, 0.16)		0.04 **(**−0.06, 0.13)		0.09 (0.03, 0.15)
≥150.1		0.08 (0.04, 0.11)		0.07 (0.03, 0.10)		0.01 (−0.05, 0.06)		0.09 (0.05, 0.12)		0.09 (0.04, 0.14)		0.07 (0.04, 0.11)
Maternal BMI, kg/m^2^	19,713 (10)	*P*-int: 0.194	19,626 (10)	*P*-int: 0.885	8712 (7)	*P*-int: 0.888	19,362 (10)	*P*-int: 0.553	8234 (7)	*P*-int: 0.767	18,333 (9)	*P*-int: 0.787
<20		0.10 (0.04, 0.15)		0.09 (0.03, 0.14)		0.01 (−0.06, 0.09)		0.12 (0.06, 0.17)		0.10 (0.01, 0.19)		0.09 (0.03, 0.15)
≥20		0.09 (0.05, 0.14)		0.08 (0.04, 0.12)		0.00 (−0.06, 0.06)		0.07 (0.03, 0.11)		0.09 (0.03, 0.15)		0.07 (0.04, 0.11)
Maternal age, y	24,259 (13)	*P*-int: 0.361	23,289 (11)	*P*-int: 0.666	8870 (7)	*P*-int: 0.470	23,597 (12)	*P*-int: 0.043	12,229 (9)	*P*-int: 0.005	22,575 (11)	*P*-int: 0.610
<25		0.07 (0.03, 0.11)		0.10 (0.05, 0.14)		0.03 (−0.04, 0.09)		0.07 (0.03, 0.11)		0.03 (−0.03, 0.10)		0.06 (0.02, 0.10)
≥25		0.09 (0.05, 0.14)		0.07 (0.02, 0.11)		−0.02 (−0.09, 0.04)		0.10 (0.06, 0.15)		0.14 (0.07, 0.20)		0.06 (0.02, 0.11)
Maternal education	24,416 (13)	*P*-int: 0.411	23,441 (11)	*P*-int: 0.700	8972 (7)	*P*-int: 0.495	23,751 (12)	*P* **-**int: 0.015	12,319 (9)	*P*-int: 0.003	22,725 (11)	*P*-int: 0.104
<Primary		0.07 (0.03, 0.12)		0.05 (0.00, 0.10)		−0.01 (−0.10, 0.08)		0.11 (0.06, 0.16)		0.14 (0.06, 0.21)		0.07 (0.02, 0.11)
≥Primary		0.06 (0.02, 0.10)		0.09 (0.05, 0.13)		0.01 (−0.05, 0.06)		0.06 (0.02, 0.09)		**0**.05**(**−0.01, 0.10)		0.06 (0.02, 0.09)
Maternal depressive symptoms	17,607 (8)	*P*-int: 0.950	17,523 (8)	*P*-int: 0.335	8129 (6)	*P*-int: 0.332	17,302 (8)	*P*-int: 0.823	7433 (6)	*P*-int: 0.647	17,320 (8)	*P*-int: 0.753
<75th percentile		0.06 (0.02, 0.11)		0.08 (0.04, 0.12)		0.02 (−0.03, 0.08)		0.07 (0.03, 0.11)		0.08 (0.01, 0.14)		0.06 (0.03, 0.10)
≥75th percentile		0.06 (−0.01, 0.13)		0.03 (−0.04, 0.10)		−0.03 (−0.12, 0.07)		0.07 (0.01, 0.12)		0.11 (0.03, 0.20)		0.06 (0.00, 0.12)
Child sex	24,561 (13)	*P*-int: 0.424	23,588 (11)	*P*-int: 0.706	9095 (7)	*P*-int: 0.414	23,899 (12)	*P*-int: 0.754	12,460 (9)	*P*-int: 0.162	22,871 (11)	*P*-int: 0.155
Male		0.05 (0.01, 0.10)		0.08 (0.04, 0.13)		0.01 (−0.05, 0.08)		0.08 (0.04, 0.12)		0.07 (0.01, 0.13)		0.07 (0.03, 0.11)
Female		0.09 (0.05, 0.14)		0.07 (0.03, 0.12)		−0.01 (−0.07, 0.05)		0.09 (0.05, 0.13)		0.11 (0.04, 0.17)		0.05 (0.01, 0.09)
Child birth order	24,165 (13)	*P*-int: 0.519	23,201 (11)	*P*-int: 0.979	8811 (7)	*P*-int: 0.285	23,508 (12)	*P*-int: 0.042	12,197 (9)	*P*-int: 0.062	22,479 (11)	*P*-int: 0.145
Firstborn		0.05 (0.00, 0.11)		0.09 (0.03, 0.14)		0.04 (−0.04, 0.12)		0.03 (−0.03, 0.08)		0.04 (−0.04, 0.13)		0.01 (−0.04, 0.06)
Later-born		0.08 (0.04, 0.12)		0.07 (0.03, 0.11)		−0.02 (−0.07, 0.04)		0.11 (0.07, 0.15)		0.12 (0.06, 0.17)		0.08 (0.05, 0.12)
Child stunted	8256 (9)	*P*-int: 0.255	7396 (7)	*P*-int: 0.495	4148 (6)	*P*-int: 0.702	7749 (8)	*P*-int: 0.207	6759 (7)	*P*-int: 0.613	6647 (7)	*P*-int: 0.312
LAZ < −2		0.19 (0.08, 0.30)		0.14 (0.03, 0.25)		−0.04 (−0.19, 0.11)		0.16 (0.06, 0.26)		0.11 (−0.01, 0.23)		0.12 (0.01, 0.22)
LAZ ≥ −2		0.09 (0.03, 0.14)		0.09 (0.04, 0.15)		0.01 (−0.07, 0.08)		0.08 (0.04, 0.13)		0.08 (0.02, 0.13)		0.05 (0.00, 0.09)
Child acute malnutrition	7974 (8)	*P* **-**int:0.034	7412 (7)	*P*-int: 0.349	4161 (6)	*P*-int: 0.166	7765 (8)	*P*-int: 0.131	6774 (7)	*P*-int: 0.992	6662 (7)	*P*-int: 0.417
Acutely malnourished		0.31 (0.17, 0.44)		0.28 (0.12, 0.43)		0.16 (−0.10, 0.43)		0.28 (0.12, 0.44)		0.12 (−0.07, 0.32)		0.11 (−0.06, 0.29)
Not acutely malnourished		0.11 (0.06, 0.16)		0.10 (0.05, 0.16)		−0.01 (−0.07, 0.06)		0.09 (0.05, 0.14)		0.09 (0.04, 0.14)		0.07 (0.02, 0.11)
Child anemia	4231 (5)	*P*-int: 0.650	4228 (5)	*P*-int: 0.834	2662 (4)	*P*-int: 0.567	4119 (5)	*P*-int: 0.369	3029 (4)	*P*-int: 0.084	3003 (4)	*P*-int: 0.658
Hb <110 g/L		0.17 (0.08, 0.26)		0.12 (0.03, 0.22)		0.02 (−0.10, 0.13)		0.16 (0.08, 0.24)		0.13 (0.03, 0.24)		0.08 (−0.01, 0.17)
Hb ≥110 g/L		0.08 (−0.03, 0.20)		0.08 (−0.03, 0.19)		−0.01 (−0.12, 0.10)		0.06 (−0.05, 0.16)		−0.01 (−0.12, 0.10)		0.02 (−0.09, 0.13)
Household SES	24,207 (13)	*P*-int: 0.001	23,263 (11)	*P*-int: 0.982	8894 (7)	*P*-int:0.033	23,572 (12)	*P*-int: 0.077	12,138 (9)	*P*-int: 0.967	22,544 (11)	*P*-int: 0.091
<Study median		0.12 (0.08, 0.17)		0.08 (0.03, 0.12)		0.06 (−0.01, 0.12)		0.11 (0.07, 0.16)		0.09 (0.03, 0.15)		0.09 (0.05, 0.13)
≥Study median		0.03 (−0.01, 0.07)		0.07 (0.03, 0.12)		−0.04 (−0.10, 0.03)		0.05**(**0.01, 0.09)		0.09 (0.02, 0.15)		0.03 (0.00, 0.07)
Household food insecurity	21,412 (11)	*P*-int: 0.627	20,737 (10)	*P*-int: 0.347	8842 (7)	*P*-int: 0.984	21,094 (11)	*P*-int: 0.898	9665 (8)	*P*-int: 0.352	19,992 (10)	*P*-int: 0.951
Moderate to severe		0.10 (0.04, 0.17)		0.07 (0.01, 0.13)		0.01 (−0.07, 0.09)		0.11 (0.05, 0.17)		0.12 (0.04, 0.20)		0.07 (0.01, 0.13)
Mild to none		0.06 (0.03, 0.10)		0.08 (0.04, 0.12)		0.01 (−0.05, 0.06)		0.07 (0.04, 0.11)		0.08 (0.02, 0.14)		0.06 (0.02, 0.09)
Household source water quality	12,608 (9)	*P*-int: 0.988	12,610 (9)	*P*-int: 0.939	3508 (5)	*P*-int: 0.700	12,561 (9)	*P*-int: 0.514	7104 (6)	*P*-int: 0.410	11,513 (8)	*P*-int: 0.883
Unimproved		0.11 (0.03, 0.18)		0.09 (0.01, 0.16)		−0.01 (−0.18, 0.17)		0.17 (0.09, 0.25)		0.08 (−0.05, 0.21)		0.07 (−0.01, 0.15)
Improved		0.10 (0.04, 0.16)		0.11 (0.05, 0.16)		−0.01 (−0.09, 0.06)		0.09 (0.03, 0.15)		0.03 (−0.06, 0.12)		0.08 (0.03, 0.14)
Household sanitation	13,484 (11)	*P*-int: 0.218	12,894 (10)	*P*-int: 0.550	5097 (7)	*P*-int: 0.947	13,187 (11)	*P*-int: 0.193	7937 (8)	*P*-int: 0.172	12,154 (10)	*P*-int: 0.943
Unimproved		0.10 (0.04, 0.15)		0.07 (0.01, 0.13)		−0.04 (−0.14, 0.05)		0.07 (0.02, 0.13)		0.03 (−0.06, 0.11)		0.05 (−0.01, 0.10)
Improved		0.12 (0.06, 0.17)		0.11 (0.05, 0.17)		0.04 (−0.03, 0.11)		0.11 (0.06, 0.16)		0.09 (0.03, 0.15)		0.08 (0.03, 0.12)
Home environment	19,878 (8)	*P*-int: 0.251	19,742 (8)	*P*-int: 0.363	7274 (5)	*P*-int: 0.142	19,514 (8)	*P*-int: 0.565	8074 (5)	*P*-int: 0.860	18,414 (7)	*P*-int: 0.300
<Study median		0.07 (0.01, 0.12)		0.06 (0.00, 0.11)		−0.06 (−0.14, 0.02)		0.05 (−0.01, 0.11)		0.09 (−0.03, 0.20)		0.02 (−0.03, 0.08)
≥Study median		0.05 (0.01, 0.10)		0.04 (0.00, 0.08)		0.03 (−0.03, 0.09)		0.07 (0.04, 0.11)		0.06 (0.00, 0.13)		0.06 (0.02, 0.09)
Season	19,158 (10)	*P*-int: 0.979	18,484 (9)	*P*-int: 0.734	9088 (7)	*P*-int: 0.766	18,843 (10)	*P*-int: 0.827	7403 (7)	*P*-int: 0.993	17,741 (9)	*P*-int: 0.958
Dry		0.09 (0.04, 0.13)		0.10 (0.06, 0.15)		−0.01 (−0.07, 0.05)		0.09 (0.05, 0.14)		0.09 (0.03, 0.14)		0.07 (0.03, 0.11)
Rainy		0.07 (0.02, 0.12)		0.06 (0.01, 0.10)		0.02 (−0.05, 0.09)		0.08 (0.04, 0.12)		0.08 (0.00, 0.16)		0.06 (0.01, 0.10)

1 comp, number of intervention against control group comparisons; Hb, hemoglobin; LAZ, length-for-age *z* score; MD, mean difference in fixed-effects models; *n*, number of individual participants; *P*-int, *P* value for the interaction indicating the difference in effects of small-quantity lipid-based nutrient supplements between the 2 levels of the effect modifier in fixed-effects models; SES, socioeconomic status; SQ-LNS, small-quantity lipid-based nutrient supplement.

**TABLE 6:** Individual-level effect modifiers of effects of SQ-LNSs provided to infants and young children age 6–24 mo on binary developmental outcomes^1^

	Language lowest decile		Social-emotional lowest decile		Executive function lowest decile		Motor lowest decile		Walking without support at 12 mo	
	*n* (comp)	Pooled PR (95% CI)	*n* (comp)	Pooled PR (95% CI)	*n* (comp)	Pooled PR (95% CI)	*n* (comp)	Pooled PR (95% CI)	*n* (comp)	Pooled PR (95% CI)
Maternal height, cm	15,675 (4)	*P*-int: 0.678	9707 (3)	*P*-int: 0.638	5144 (2)	*P*-int: —	9576 (3)	*P*-int: 0.550	6677 (5)	*P*-int: 0.624
<150.1		0.87 (0.70, 1.08)		0.89 (0.73, 1.08)		—		0.87 (0.71, 1.07)		1.19 (1.02, 1.39)
≥150.1		0.86 (0.74, 1.00)		0.86 (0.69, 1.06)		—		0.79 (0.63, 0.98)		1.20 (1.11, 1.29)
Maternal BMI, kg/m^2^	18,852 (9)	*P*-int: 0.149	19,342 (9)	*P*-int: 0.199	7821 (5)	*P*-int: 0.977	19,078 (9)	*P*-int: 0.477	8942 (7)	*P*-int: 0.343
<20		0.83 (0.71, 0.98)		0.88 (0.75, 1.03)		0.91 (0.73, 1.15)		0.83 (0.71, 0.96)		1.11 (1.00, 1.24)
≥20		0.76 (0.67, 0.87)		0.76 (0.66, 0.88)		0.86 (0.69, 1.07)		0.79 (0.69, 0.90)		1.11 (1.03, 1.20)
Maternal age, y	23,678 (11)	*P*-int: 0.832	22,997 (10)	*P*-int: 0.546	7984 (5)	*P*-int: 0.088	23,305 (11)	*P*-int: 0.466	13,609 (9)	*P*-int: 0.394
<25		0.83 (0.73, 0.94)		0.81 (0.71, 0.93)		0.79 (0.63, 1.00)		0.88 (0.77, 1.00)		1.12 (1.05, 1.18)
≥25		0.84 (0.74, 0.95)		0.86 (0.75, 0.98)		1.02 (0.84,1.25)		0.81 (0.71, 0.93)		1.07 (1.00, 1.14)
Maternal education	19,704 (7)	*P*-int: 0.804	18,982 (6)	*P*-int: 0.638	6636 (4)	*P*-int: 0.865	19,293 (7)	*P*-int: 0.225	13,210 (7)	*P*-int: 0.792
<Primary		0.89 (0.77, 1.02)		0.87 (0.75, 1.01)		0.93 (0.72, 1.20)		0.83 (0.72, 0.97)		1.09 (1.02, 1.16)
≥Primary		0.86 (0.74, 0.99)		0.90 (0.77, 1.06)		0.90 (0.72, 1.12)		0.95 (0.81, 1.11)		1.10 (1.04, 1.16)
Maternal depressive symptoms	17,310 (7)	*P*-int: 0.204	17,523 (8)	*P*-int: 0.165	6494 (3)	*P*-int: 0.394	16,120 (6)	*P*-int: 0.649	7718 (5)	*P*-int: 0.543
<75th percentile		0.83 (0.73, 0.94)		0.80 (0.70, 0.91)		0.94 (0.78, 1.15)		0.87 (0.76, 0.99)		1.14 (1.06, 1.23)
≥75th percentile		0.95 (0.80, 1.14)		0.92 (0.76, 1.11)		0.81 (0.58, 1.13)		0.82 (0.67, 1.01)		1.19 (1.04, 1.37)
Child sex	24,262 (12)	*P*-int: 0.255	23,588 (11)	*P*-int: 0.757	8805 (6)	*P*-int: 0.231	23,311 (11)	*P*-int: 0.701	13,841 (10)	*P*-int: 0.952
Male		0.89 (0.79, 1.00)		0.84 (0.74, 0.95)		0.83 (0.66, 1.04)		0.83 (0.73, 0.95)		1.10 (1.03, 1.17)
Female		0.76 (0.67, 0.87)		0.80 (0.70, 0.92)		0.95 (0.80, 1.13)		0.81 (0.71, 0.92)		1.09 (1.03, 1.15)
Child birth order	22,225 (9)	*P*-int: 0.362	22,137 (9)	*P*-int: 0.920	7427 (4)	*P*-int: 0.144	21,209 (8)	*P*-int: 0.015	13,587 (10)	*P*-int: 0.025
Firstborn		0.91 (0.77, 1.09)		0.85 (0.71, 1.01)		0.75 (0.56, 1.02)		1.01 (0.84, 1.21)		1.05 (0.99, 1.11**)**
Later-born		0.80 (0.71, 0.90)		0.82 (0.73, 0.92)		0.97 (0.81, 1.15)		0.76 (0.68, 0.86)		1.16 (1.09, 1.23)
Child stunted	6776 (6)	*P*-int: 0.111	5588 (4)	*P*-int: 0.728	2564 (3)	*P*-int: 0.507	6051 (5)	*P*-int: 0.870	8941 (5)	*P*-int: 0.624
LAZ < −2		0.62 (0.49, 0.79)		0.67 (0.51, 0.89)		1.14 (0.67, 1.93)		0.73 (0.57, 0.93)		1.11 (0.98, 1.26)
LAZ ≥ −2		0.75 (0.63, 0.89)		0.73 (0.59, 0.89)		0.86 (0.64, 1.13)		0.75 (0.62, 0.90)		1.09 (1.03, 1.15)
Child acute malnutrition	4067 (3)	*P*-int: 0.588	3476 (2)	*P*-int: —	—	*P*-int: —	3906 (3)	*P*-int: 0.612	8164 (4)	*P*-int: 0.133
Acutely malnourished		0.67 (0.46, 0.99)		—	—	—		0.71 (0.48, 1.06)		1.19 (1.02, 1.38)
Not acutely malnourished		0.65 (0.53, 0.80)		—	—	—		0.72 (0.58, 0.88)		1.08 (1.03, 1.14)
Child anemia	3110 (4)	*P*-int: 0.847	3107 (4)	*P*-int: 0.881	2662 (4)	*P*-int: 0.464	2998 (4)	*P*-int: 0.237	3013 (4)	*P*-int: 0.526
Hb <110 g/L		0.91 (0.66, 1.25)		0.89 (0.67, 1.18)		0.88 (0.61, 1.27)		0.78 (0.57, 1.06)		1.12 (0.98, 1.27)
Hb ≥110 g/L		0.88 (0.64, 1.21)		0.89 (0.62, 1.26)		1.07 (0.76, 1.52)		1.07 (0.77, 1.49)		1.18 (1.05, 1.33)
Household SES	23,938 (12)	*P*-int: 0.227	23,263 (11)	*P*-int: 0.655	8064 (5)	*P*-int: 0.389	22,985 (11)	*P*-int: 0.878	13,519 (9)	*P*-int: 0.721
<Study median		0.81 (0.72, 0.91)		0.81 (0.72, 0.92)		0.86 (0.70, 1.06)		0.83 (0.74, 0.93)		1.09 (1.02, 1.16)
≥Study median		0.88 (0.76, 1.00)		0.83 (0.71, 0.95)		0.96 (0.78, 1.18)		0.83 (0.72, 0.95)		1.08 (1.03, 1.14)
Household food insecurity	20,532 (9)	*P*-int: 0.772	19,715 (8)	*P*-int: 0.325	8036 (5)	*P*-int: 0.640	19,484 (8)	*P*-int: 0.594	13,076 (7)	*P*-int: 0.812
Moderate to severe		0.78 (0.67, 0.91)		0.81 (0.69, 0.95)		0.91 (0.71, 1.17)		0.78 (0.65, 0.93)		1.10 (1.02, 1.18)
Mild to none		0.85 (0.76, 0.95)		0.81 (0.71, 0.92)		0.87 (0.73, 1.04)		0.85 (0.75, 0.95)		1.09 (1.03, 1.14)
Household source water quality	11,079 (6)	*P*-int: 0.707	11,081 (6)	*P*-int: 0.534	2294 (2)	*P*-int: —	11,034 (6)	*P*-int: 0.723	3205 (5)	*P*-int: 0.190
Unimproved		0.76 (0.62, 0.92)		0.76 (0.61, 0.94)		—		0.72 (0.59, 0.88)		1.17 (0.95, 1.43)
Improved		0.80 (0.66, 0.96)		0.75 (0.61, 0.92)		—		0.82 (0.68, 0.99)		1.09 (0.98, 1.21)
Household sanitation	4971 (3)	*P*-int: 0.821	7706 (4)	*P*-int: 0.304	1455 (2)	*P*-int: —	7500 (4)	*P*-int: 0.228	8584 (5)	*P*-int: 0.336
Unimproved		0.85 (0.65, 1.10)		0.76 (0.61, 0.97)		—		0.98 (0.79, 1.22)		1.07 (0.99, 1.16)
Improved		0.75 (0.60, 0.94)		0.88 (0.71, 1.09)		—		0.80 (0.63, 1.00)		1.07 (1.00, 1.15)
Home environment	19,878 (8)	*P*-int: 0.592	19,742 (8)	*P*-int: 0.863	6636 (4)	*P*-int: 0.287	19,514 (8)	*P*-int: 0.766	8353 (6)	*P*-int: 0.458
<Study median		0.86 (0.75, 0.99)		0.85 (0.74, 0.98)		1.02 (0.82, 1.27)		0.86 (0.75, 0.99)		1.11 (1.00, 1.23)
≥Study median		0.89 (0.77, 1.03)		0.89 (0.77, 1.02)		0.84 (0.67, 1.05)		0.85 (0.72, 0.99)		1.15 (1.07, 1.24)
Season	18,846 (9)	*P*-int: 0.524	16,656 (6)	*P*-int: 0.584	6749 (3)	*P*-int: 0.819	17,934 (8)	*P*-int: 0.839	9043 (7)	*P*-int: 0.845
Dry		0.82 (0.72, 0.94)		0.75 (0.64, 0.88)		0.87 (0.71, 1.07)		0.80 (0.71, 0.91)		1.17 (1.08, 1.27)
Rainy		0.83 (0.72, 0.96)		0.84 (0.72, 0.98)		0.93 (0.72, 1.19)		0.85 (0.73, 1.00)		1.11 (1.02, 1.20)

1 comp, number of intervention against control group comparisons; Hb, hemoglobin; LAZ, length-for-age *z* score; *n*, number of individual participants; *P*-int, *P* value for the interaction indicating the difference in effects of small-quantity lipid-based nutrient supplements between the 2 levels of the effect modifier in fixed-effects models; PR, prevalence ratio in fixed-effects models; SES, socioeconomic status; SQ-LNS, small-quantity lipid-based nutrient supplement.

**FIGURE 7:**
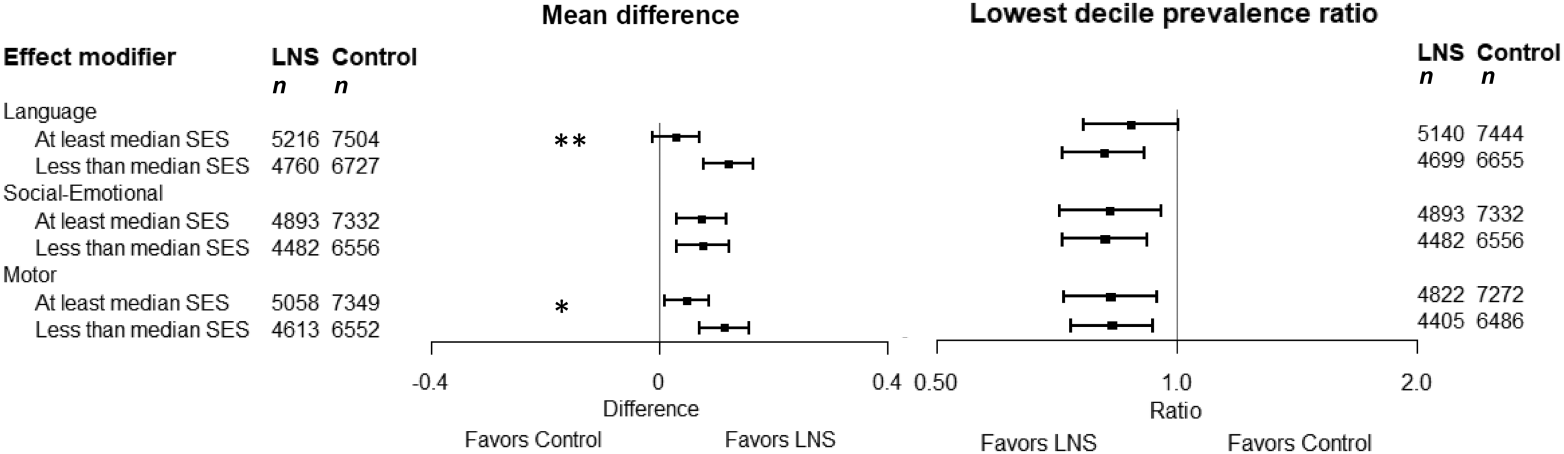
Pooled effects of SQ-LNSs on 6 primary developmental outcomes stratified by individual-level household SES. Individual study estimates for interaction effect were generated from log-binomial regression controlling for baseline measure when available and with clustered observations using robust SEs for cluster-randomized trials. Pooled subgroup estimates and statistical testing of the pooled interaction term were generated using inverse-variance weighting fixed effects. **P-Int < 0.01, *P-Int < 0.1 *P*-int, *P* value for the interaction indicating the difference in effects of small-quantity lipid-based nutrient supplements between the 2 levels of the effect modifier in fixed-effects models. LNS, lipid-based nutrient supplement; SES, socioeconomic status.

Household socioeconomic status (SES; above or below the study median) modified the effect of SQ-LNSs on mean language, motor, and executive function scores (Table 5, **Figure 7**). Effects of SQ-LNSs on these scores were larger among children in low-SES households (0.06–0.12 SD) than among children in high-SES households (−0.04 to 0.05 SD). For the percentage of children in the lowest decile of scores, there was no significant effect modification by household SES with regard to prevalence ratios. However, for language there was a greater percentage point reduction in low scores among children in the low-SES group (3 percentage points) than in the high-SES group (1 percentage point) (Supplemental Figure 8C).

**FIGURE 8:**
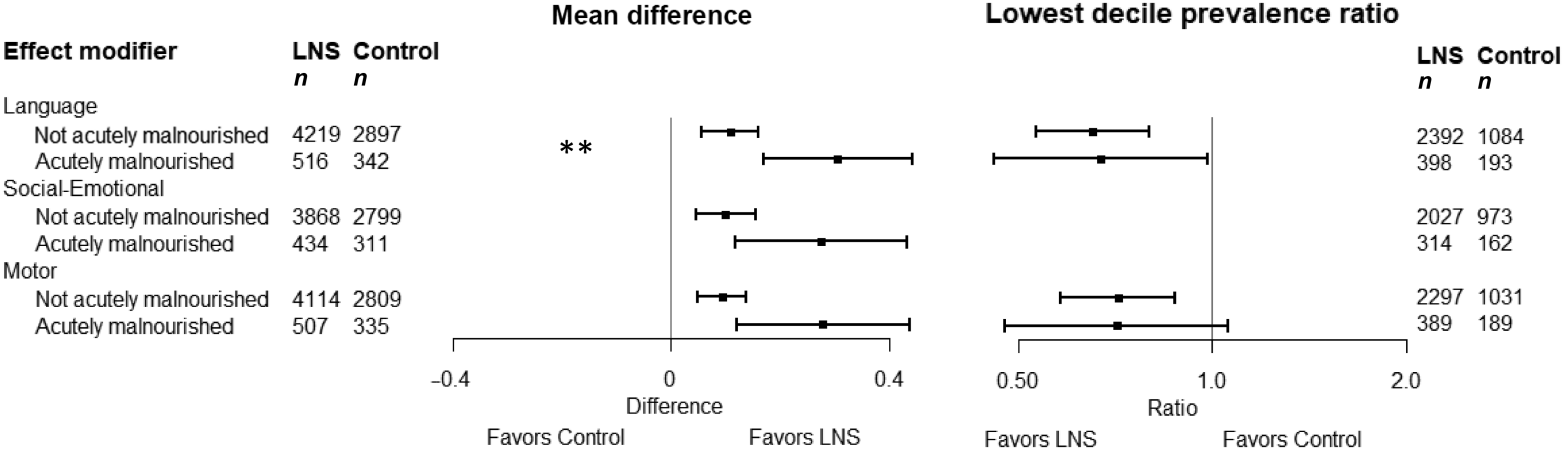
Pooled effects of SQ-LNSs on 6 primary developmental outcomes stratified by individual-level child baseline acute malnutrition. Individual study estimates for interaction effect were generated from log-binomial regression controlling for baseline measure when available and with clustered observations using robust SEs for cluster-randomized trials. Pooled subgroup estimates and statistical testing of the pooled interaction term were generated using inverse-variance weighting fixed effects. **P-Int < 0.05 *P*-int, *P* value for the interaction indicating the difference in effects of small-quantity lipid-based nutrient supplements between the 2 levels of the effect modifier in fixed-effects models. LNS, lipid-based nutrient supplement.

Child baseline acute malnutrition [weight-for-length *z* score (WLZ) < −2 SD or midupper arm circumference (MUAC) < 125 mm] modified the effect of SQ-LNSs on mean language scores (Table 5, **Figure 8**). The effect of SQ-LNSs on mean language score was significantly larger among children who were malnourished when they began receiving SQ-LNSs (0.30 SD) than among those who were not (0.11 SD). Social-emotional and motor scores showed a similar pattern of greater effect sizes among acutely malnourished children (0.27 SD for both outcomes compared with 0.09–0.10 SD among children who were not malnourished); however, the interaction tests were not statistically significant.

**FIGURE 9:**
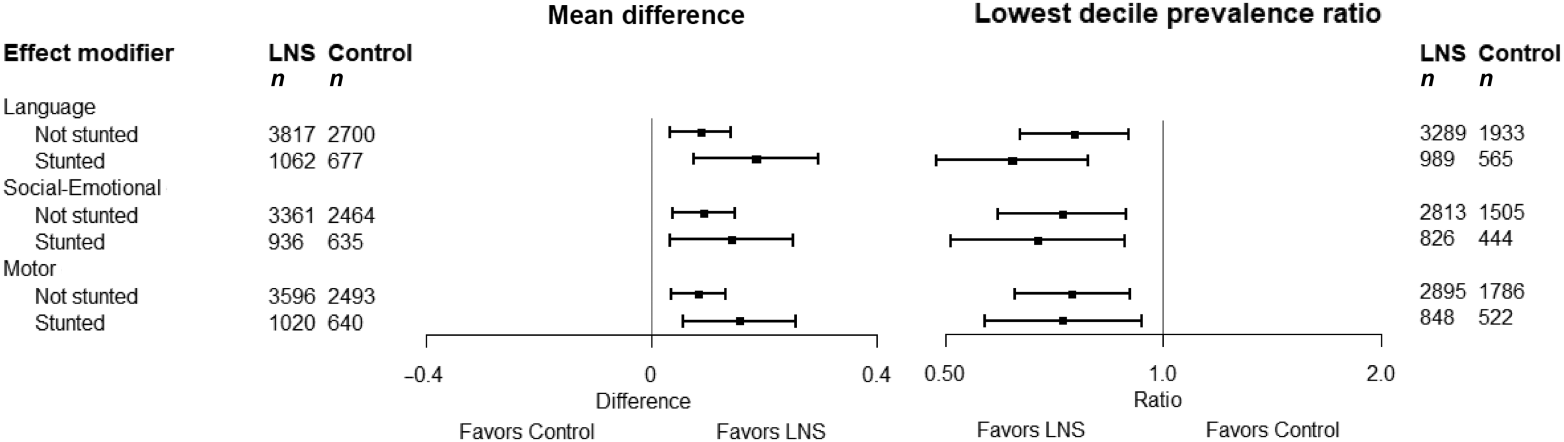
Pooled effects of SQ-LNSs on 6 primary developmental outcomes stratified by individual-level child baseline stunting. Individual study estimates for interaction effect were generated from log-binomial regression controlling for baseline measure when available and with clustered observations using robust SEs for cluster-randomized trials. Pooled subgroup estimates and statistical testing of the pooled interaction term were generated using inverse-variance weighting fixed effects. LNS, lipid-based nutrient supplement.

Child baseline stunting (LAZ < −2 SD) modified the effect of SQ-LNSs on the prevalence difference of children in the lowest decile of language scores. There was a 7 percentage point reduction of children in the lowest decile of language scores among children who were stunted when they began receiving SQ-LNSs (95% CI: −0.11, −0.04), compared with a 3 percentage point reduction (95% CI: −0.04, −0.01) (Supplemental Figure 7C8) among those who were not stunted. Social-emotional and motor scores showed similar trends of greater effect sizes among stunted children (**Figure 9**); however, no interactions between baseline stunting and intervention group were significant for these or any other outcomes.

Maternal education, maternal age, and child birth order modified the effects of SQ-LNSs on mean motor and fine motor scores (Table 5). Greater effects of SQ-LNSs on these scores were found among children of mothers with lower education (0.11–0.14 SD) than those with higher education (0.05–0.06 SD), among children of older mothers (0.10–0.14 SD) than younger mothers (0.03–0.07 SD), and among later-born children (i.e., those born after the firstborn child; 0.11–0.12 SD) than firstborn children (0.03–0.04 SD). Maternal education also modified the effects of SQ-LNSs with respect to the prevalence difference for scoring in the lowest decile of motor scores, with greater reductions among children of mothers with lower education (Supplemental Figure 7I4). Birth order also modified the effect of SQ-LNSs on this adverse motor outcome, with a greater reduction among later-born children (24% reduction compared with 1% increase among firstborn children), and on walking without support at 12 mo, with a greater positive effect among later-born children (16% increase compared with 5% increase among firstborn children) (Table 6). Maternal age also modified the effect of SQ-LNSs on the prevalence of children in the lowest decile of executive function scores. In contrast to the pattern for motor scores, for executive function greater effects with respect to both the prevalence ratio and the prevalence difference were found among children of younger mothers than among children of older mothers (Table 6; Supplemental Figure 7M3, N3). Maternal education, maternal age, and birth order did not modify the effects of SQ-LNSs on any other developmental outcomes.

Maternal height and child baseline anemia modified the effect of SQ-LNSs on mean fine motor scores, but no other outcomes. There were greater effects of SQ-LNSs on fine motor scores among children of taller mothers (0.09 SD) than children of shorter mothers (0.04 SD) and children who were anemic at baseline (0.13 SD) than children who were not (−0.01 SD) (Table 5).

## Discussion

In this IPD meta-analysis of 13 randomized controlled trials in 9 countries with a total sample size of >30,000 children, SQ-LNSs provided to infants and young children 6–24 mo of age increased mean language, social-emotional, and motor scores by 0.06–0.09 SD and led to a relative reduction of 16%–19% in adverse developmental outcomes (1–2 percentage point difference). The quality of the evidence for all outcomes was high. The effects of SQ-LNSs on developmental outcomes did not significantly differ by most study-level characteristics including region (although most studies were conducted in Sub-Saharan Africa), malaria prevalence, water quality, supplementation duration, frequency of contact, or mean compliance with SQ-LNSs, indicating that these aspects of context and program delivery did not explain differences in effect sizes across these 13 trials. However, effects of SQ-LNSs on language, social-emotional, and motor development were larger among study populations with a higher stunting burden and effects on motor development were larger in sites with higher prevalence of child anemia. At the individual level, greater effects of SQ-LNSs were found on language among children who were stunted or acutely malnourished when they started receiving SQ-LNSs; on language, motor, and executive function among households with lower SES; and on motor and fine motor development among later-born children, children of older mothers, and children of mothers with lower education. Children of taller mothers and children who were anemic at baseline also showed greater effects on fine motor scores, whereas children of younger mothers showed greater effects on executive function scores.

### Main effects

Our findings of significant positive effects of SQ-LNSs on developmental outcomes in the range of 0.06–0.09 SD, which would be analogous to ∼1–1.5 IQ points on an IQ test, are consistent with a previous meta-analysis of SQ-LNSs by Tam et al. (7), which reported slightly larger effect sizes of 0.12–0.13 SD (∼1.8–2 IQ points). Effect sizes in the range of 0.06–0.09 SD are probably more accurate for a general population, because our analysis included a larger number of trials (13 compared with 6) and children (∼30,000 compared with ∼3500). However, we found effect sizes closer to their estimates in sites with a higher stunting burden (0.11–0.13 SD), and the trials included in the Tam et al. meta-analysis tended to have a high stunting burden. These effect sizes are also consistent with previous meta-analyses of child nutritional supplementation trials in LMICs. A meta-analysis by Larson and Yousafzai (48) of 23 nutritional supplementation randomized controlled trials among children age 0–2 y indicated an effect size of 0.08 SD for cognitive/mental development. Another meta-analysis by Prado et al. (49) of effects of interventions from pregnancy to 5 y on linear growth and development showed an effect of child nutritional supplementation of 0.13 SD on language (13 studies), 0.09 SD on motor (27 studies), and 0.09 SD on social-emotional development (13 studies).

These previous meta-analyses pooled effects across studies that provided a wide range of different types of nutritional supplements, including single micronutrients, as well as multiple micronutrients with and without macronutrients. Few previous meta-analyses of specific types of nutritional supplements have been able to calculate pooled effects on developmental outcomes, owing to small numbers of studies and differences in measurement and reporting of outcomes in this domain. For example, a 2020 Cochrane review of home fortification of children's foods with multiple micronutrient powders was not able to calculate pooled effects on developmental outcomes (50). Therefore, it is difficult to compare the effects of SQ-LNSs with the effects of other types of nutritional supplements. However, in Prado et al. (49), pooled effect sizes for language, social-emotional, and motor scores were similar in trials that provided multiple micronutrients with macronutrients (0.09–0.10 SD; 10–17 trials) and in trials that provided multiple micronutrients without macronutrients (0.08–0.11 SD; 5–11 trials). This suggests that the multiple micronutrients in SQ-LNSs may be key ingredients for effects on developmental outcomes. However, further research is needed to understand how SQ-LNS compares with other types of nutritional supplements designed to fill nutrient gaps in children's diets, such as micronutrient powders and fortified blended foods, with regard to effects on child development. One difference between SQ-LNSs and other products that contain both macro- and micronutrients is that SQ-LNSs contain substantial amounts of essential fatty acids, important for brain development, whereas other energy-containing supplements may not.

Our study is the first meta-analysis that we know of to report effects of child nutritional supplementation on reducing the prevalence of children in the lowest decile of developmental scores. We used the lowest decile of scores as a proxy for children who may be at the greatest risk of developmental delay. Typically, distributions of developmental assessment scores in LMIC settings have a left tail that is larger than the right tail, comprised of children who score substantially lower than their age- and sex-matched peers and may be developmentally delayed. In most of the studies in this IPD meta-analysis (in 9 of 13 comparisons for language, 10 of 12 for motor, and 8 of 11 for social-emotional scores), a greater proportion of children scored <2 SD below the mean (overall ∼3%) than >2 SD above the mean (<1%). The finding of significant reductions in the lowest decile of scores shows that SQ-LNS not only shifts the mean of the distribution, but also improves outcomes among children in the lower tail of the distribution who may be at particular risk of developmental delay. Attaining developmental skills that are appropriate for the child's age is likely to facilitate further advances in development because many skills developed at later stages build on those that were learned previously. For example, acquiring the skill of walking without support is a catalyst for change in multiple domains of development (51). Both the reduction in the percentage of children in the lowest decile of scores and the increase in the percentage of children walking without support with SQ-LNSs could be important for supporting healthier developmental trajectories in the population. The percentage of children walking without support in the SQ-LNS groups (45% overall compared with 39% in control groups) is closer to the prevalence in the WHO Multi-Center Growth Reference study, which showed that in a healthy group of children, 50% were walking without support at age 12 mo (52). Further research is needed to understand the longer-term effects of SQ-LNSs on developmental outcomes and whether the observed positive effects at 12–24 mo are sustained into later childhood. Two of the trials in this IPD meta-analysis conducted follow-up assessments at age 3–6 y. One follow-up study in Bangladesh found significantly higher composite cognitive scores (+0.13 SD) in the SQ-LNS group (53), whereas the other follow-up study in Ghana showed reduced social-emotional difficulties (−0.12 SD) in the SQ-LNS group, with greater effects among children with lower-quality home environments (−0.22 SD) (54).

Among the 6 trials (7 comparisons) that assessed executive function, no overall effects of SQ-LNSs were found. Executive function is the cognitive control of attention, self-regulation, and emotion, including the ability to plan and monitor actions, self-regulate actions and emotions, focus and sustain attention, and maintain information in short-term memory (55). These skills and the neural structures that underlie them (the prefrontal cortex and other connected cortical and subcortical structures) experience their peak rate of development in later childhood and adolescence (56). Therefore, 6–24 mo of age may be too early for nutritional supplementation to have a measurable effect on the development of executive function. It is also possible that the A not B task might not be the most robust assessment of executive function at this age. Future studies should consider other measures of executive function. Future studies should also consider the age of child assessments when deciding whether to monitor the acquisition of the WHO motor milestones, given that across these 13 studies, variance was found at 12 mo, but ceiling effects were present at age 18 mo.

Comparing the all-trials analysis with the child-LNS-only analysis, we did not find evidence that maternal LNS provided an added benefit for developmental outcomes compared with child SQ-LNS only; however, only 4 trials included maternal LNS arms (29, 37, 40, 43). This absence of an additive effect is consistent with the 2 trials that directly compared arms providing maternal plus child LNS with child SQ-LNS only, which did not find differences between these arms in developmental outcomes (29, 37). This is also consistent with previous meta-analyses, which have found positive effects of child but not maternal nutritional supplementation on developmental outcomes (47, 48). In our “separation of multicomponent arms” sensitivity analysis, which limited comparisons to pairs of arms with the same nonnutrition components, and also excluded the maternal LNS trials/arms, results were nearly identical to those of the all-trials analysis. The consistency across sensitivity analyses indicates that the all-trials analysis, which includes a larger sample size and broader group of trials, presents a valid estimate of the causal effect of child SQ-LNSs.

### Study-level effect modification

The child stunting burden in the study sample was the most consistent effect modifier across developmental outcomes (5 of the 8 developmental outcomes with sufficient data to analyze this effect modifier). Stunted growth has been consistently associated with poor developmental outcomes (57), therefore stunted children may have greater potential to benefit from SQ-LNSs. Within the control groups in this IPD meta-analysis, stunted children scored significantly lower than nonstunted children in language and motor scores in all trials, and in social-emotional scores in all trials except 1. Recent evidence suggests that stunted growth does not cause delayed neurodevelopment, but instead is a sensitive marker of an environment that constrains growth and development through partly overlapping causes (49, 58). High child anemia prevalence, which also modified effects of SQ-LNSs on motor outcomes, may also be a marker of a high-risk environment in which children have greater potential to benefit from SQ-LNSs. The findings from this IPD meta-analysis show that inadequate dietary intake is one of the shared causes of faltering in both linear growth and development, as well as anemia, and that SQ-LNS has positive effects on all of these outcomes (12, 59). However, patterns of effect modification were different; greater effects on linear growth were not found in studies with a higher stunting burden. Linear growth may be less malleable to recovery through intervention after early growth restriction (e.g., in utero and from birth to 6 mo of age), whereas development may be more responsive to postnatal intervention because brain plasticity continues throughout childhood (1).

Apart from stunting and anemia prevalence, we did not find significant effect modification by other study-level characteristics. In all study-level subgroups, effect sizes were consistently in the expected direction favoring SQ-LNS groups. This suggests that effects are evident across a range of contexts from low to high malaria prevalence, water quality, supplementation duration, frequency of contact, and average compliance with SQ-LNSs. However, in the study-level meta-regression analyses, we had limited power to detect significant associations between study-level effect modifiers and effect sizes owing to small sample sizes (a maximum of *n* = 13 intervention against control group comparisons). For example, the relative reduction in the prevalence of children in the lowest decile of language scores was 22% among studies with higher malaria prevalence compared with 7% among studies with lower malaria prevalence. With additional studies and thus power, it is possible that such differences could reach significance. One exception was that the effect of SQ-LNSs on executive function significantly differed between studies with a low and studies with a high prevalence of improved sanitation. However, given the lack of overall effect of SQ-LNSs on executive function and given that CIs around the executive function estimates in both subgroups (high and low prevalence of improved sanitation) included 0, it is likely that there was no true effect on executive function in either subgroup.

### Individual-level effect modification

Just as the study-level effect modification analysis showed that populations in higher-risk environments, as indicated by higher prevalence of child stunting and anemia, had greater potential to benefit from SQ-LNSs in developmental outcomes, the individual-level effect modification analysis consistently showed that certain subgroups of children who may be in higher-risk circumstances had a greater potential to benefit from SQ-LNSs, including children who were stunted, anemic, acutely malnourished, in low-SES households, later-born, and whose mothers were older and less educated. As previously discussed for stunted children, in our pooled control group data, children in all of these categories, except children of older mothers, had lower language and motor scores and therefore had greater room for improvement in developmental skills. Iron deficiency anemia, acute malnutrition, low SES, and low maternal education are associated with poor development (60, 61). Later-born children may have access to fewer household and caregiving resources because they are competing with older siblings, which may negatively affect their development. Although it is not clear why children of older mothers might be at higher risk, older mothers tended to be less educated and their children tended to be later-born, thus these subgroups overlapped (46% of older mothers completed primary school, whereas 59% of younger mothers completed primary school; 84% of older mothers’ children were later born, whereas 46% of younger mothers’ children were later born; 75% of lower educated mothers’ children were later born, whereas 56% of higher educated mothers’ children were later born).

Although the findings generally show that children in higher-risk environments have greater potential to benefit in developmental outcomes from SQ-LNSs, it is somewhat surprising that indicators of maternal depressive symptoms and the home environment did not modify effects of SQ-LNSs in this IPD meta-analysis. Similarly to stunted and malnourished children, children of mothers with depressive symptoms and children in low-quality home environments also tend to have poor developmental scores (62, 63). Three SQ-LNS trials have found that children in lower-quality home environments show greater benefits of SQ-LNSs on developmental outcomes (38, 40, 54). One of these was a follow-up study at age 4–6 y that was not included in this IPD meta-analysis. However, findings from this IPD meta-analysis suggest that effects of SQ-LNSs on development are generalizable regardless of maternal depressive symptoms and home environment, at least across the range represented in these studies. Similarly, lack of effect modification based on child sex, household food insecurity, water quality, sanitation, maternal BMI, and season suggests that effects on developmental outcomes are generalizable across these characteristics.

### Strengths and limitations of this IPD meta-analysis

This IPD meta-analysis had many strengths. A substantial number of high-quality trials that provided similar types of SQ-LNS products to children age 6–24 mo were included. Investigators from all but 1 of the eligible studies participated and the sample size was very large. The availability of IPD allowed harmonization of calculation of developmental outcomes across trials, and enabled incorporation of cluster-randomized trials using robust SEs and allowing for study-specific intracluster correlations. The 13 study sites were highly diverse in terms of sample characteristics and study designs, which provided heterogeneity for exploration of study-level effect modifiers. The consistency of findings across fixed- and random-effects models and sensitivity analyses strengthens confidence in the conclusions.

This IPD meta-analysis also had limitations. We were unable to calculate pooled estimates for effects on general nonverbal cognitive skills because only 2 trials measured this domain (21, 29). Although we were able to harmonize the calculation of developmental assessment scores across trials, different tools were used in different trials and we did not have an external reference to standardize scores. Thus, although all developmental scores were calculated in units of SD based on the within-study distribution, if SDs varied across studies, the point value of 1 SD could be larger in one trial than another, and interpretation of effect sizes would be different across trials. For example, among the 6 studies that used a 10-item A not B task to assess executive function, SDs ranged from 1.4 to 2.5; and among the 5 studies that used a 100-word vocabulary checklist to assess language, SDs ranged from 18.9 to 23.5. Ongoing efforts to develop a standardized scale of developmental scores will greatly improve future meta-analyses of developmental outcomes (64). Future research should also use developmental assessments directly observed by blinded assessors, at least in a subset of children, to address the potential risk of bias when parent-report developmental assessment tools are used in trials in which the intervention precludes participant blinding.

Another limitation was the limited diversity of geographical region. The majority of studies were conducted in Sub-Saharan Africa, with only 1 country representing the WHO Southeast Asia Region (Bangladesh) and 1 country representing Latin America and the Caribbean (Haiti). In addition, not all trials were included in all analyses because some trials did not measure some outcomes (e.g., executive function) or effect modifiers (e.g., baseline child stunting, maternal depressive symptoms, home environment). For study-level effect modifiers, statistical power was constrained by the limited number of trials. For individual-level effect modifiers, although we made every effort to standardize definitions and cutoffs for potential effect modifiers, there was variation across trials in the methods used to collect information on certain characteristics, such as household food insecurity and socioeconomic status. We examined multiple effect modifiers and numerous outcomes, so several of the significant *P*-for-interaction values are likely due to chance. As stated in the Methods, we did not adjust for multiple hypothesis testing because developmental outcomes are interrelated and the effect modification analyses are inherently exploratory. Lastly, caution is needed when interpreting the effect modification results because many of the potential effect modifiers are interrelated and also may be confounded by unmeasured variables. Thus, attribution of the relative potential to benefit from or respond to SQ-LNSs to a particular characteristic may not be warranted.

### Programmatic implications

Our findings suggest that if policy-makers and program planners implement SQ-LNS distribution to children age 6–24 mo, they can expect modest, but potentially important, developmental gains among children in the population, particularly in areas with high child stunting burden. If the goal of a policy or program is to target not only developmental outcomes, but also child growth, iron deficiency, anemia, and mortality, then SQ-LNSs should be considered. To our knowledge, SQ-LNS is the only child nutrition intervention that has been documented in meta-analyses to have positive effects on all of these outcomes (12, 59, 65). As aforementioned, few previous meta-analyses of specific types of nutritional supplements have been able to calculate pooled effects on developmental outcomes.

However, if the primary goal of a policy or program is to improve developmental outcomes, investment is needed not only in nutrition but also in other aspects of nurturing care, especially responsive care and learning opportunities (66). For all developmental domains, interventions that promote responsive care and learning opportunities have effect sizes 4–5 times larger (analogous to ∼5–7 IQ points) than those for nutritional supplementation alone (49). Integration of SQ-LNSs with such programs should be considered. One advantage of integrating nutrition with caregiving interventions could be incentivizing participation in parenting groups or home visits through provision of SQ-LNSs, thereby increasing coverage (67). Another advantage of integration is building on existing contact points between community front-line workers and families with young children, thus potentially reducing implementation costs (68). The cost of SQ-LNSs is estimated at $0.07–0.14 per child per day not including distribution costs (which may be the bulk of program costs), depending on scale and location of production (69, 70). Further research is needed on the costs of programs promoting responsive care and learning opportunities targeting young children in LMICs and integration of such programs with nutrition programs (71).

We consistently found that certain groups of children in higher-risk environments showed greater benefits in developmental outcomes from SQ-LNSs, such as children from low-SES households. This suggests that implementing SQ-LNS programs will promote equity, which is at the core of achieving the Sustainable Development Goals and ensuring that no child is left behind. However, we also found that children from the lower-risk groups (e.g., those from higher-SES households) showed positive effects. Similarly, although the reduction in the prevalence of children in the lowest decile showed gains from SQ-LNSs for the most marginalized children, the significant shifts in mean scores showed that children across the full distribution benefited from SQ-LNSs. We recommend that decisions regarding targeting specific communities or households be based on the wider body of evidence on all outcomes, including nutritional status and growth, not only developmental outcomes.

Our findings also have implications for programs designed for community management of acute malnutrition (CMAM). Many CMAM programs provide large-quantity LNSs (∼1000–1500 kcal/d) to children who meet the cutoffs for severe acute malnutrition (WLZ < −3 SD or MUAC <115 mm), according to WHO guidelines (72). Medium-quantity LNSs (∼250–500 kcal/d) are typically used to treat moderate acute malnutrition (MAM; −3 < WLZ < −2 SD or 115 < MUAC < 125 mm); however, the coverage of MAM treatment is low. Most evidence for the efficacy of MAM treatment has focused on child survival and recovery, rather than developmental outcomes (73). We found that provision of SQ-LNSs to children who experienced MAM at baseline increased developmental scores by 0.3 SD, which is the largest effect size observed in any subgroup, analogous to ∼5 IQ points, and >3 times larger than the overall effect of SQ-LNSs. This suggests that these children have high potential to benefit from LNS distribution programs in developmental outcomes, and that investment in such programs will advance not only child survival, but also fulfillment of developmental potential among the most vulnerable.

### Conclusions

SQ-LNSs can fill nutrient gaps in children's diets in key nutrients that are necessary for brain development. Given that provision of SQ-LNSs has been documented in meta-analyses to positively affect not only nutritional status and growth, but also child survival and development, it is one of the few interventions that is known to be effective to address multiple pillars of the UN's Global Strategy for Women's, Children's and Adolescents’ Health (2016–2030), which targets 3 pillars of survival (ending preventable deaths), thriving (ensuring health and well-being), and transformation (expanding enabling environments).

## Supplementary Material

nqab277_Supplemental_Files

## Data Availability

Data described in the article, code book, and analytic code will not be made available because they are compiled from 14 different trials, and access is under the control of the investigators of each of those trials.

## Notes

Supplemental Figures 1–8 and Supplemental Tables 1–6 are available from the “Supplementary data” link in the online posting of the article and from the same link in the online table of contents at https://academic.oup.com/ajcn/.

Present address for HND: Social Impact, Inc., 2300 Clarendon Blvd, Arlington, VA 22201.

Published in a supplement to *The American Journal of Clinical Nutrition*. Publication costs for this supplement were defrayed in part by the payment of page charges. The Guest Editor for this supplement was Chris Sudfeld who reports no disclosures. The Supplement Coordinator for the supplement publication was Kathryn Dewey, University of California, Davis, Davis, CA. Kathryn Dewey has received funding from the Bill & Melinda Gates Foundation and the US Agency for International Development (via the Food and Nutrition Technical Assistance Project) for research on lipid-based nutrient supplements. The opinions expressed in this publication are those of the authors and are not attributable to the sponsors or the publisher, Editor, or Editorial Board of *The American Journal of Clinical Nutrition*.

Abbreviations used: CDI, MacArthur-Bates Communicative Development Inventory; CMAM, community management of acute malnutrition; GRADE, Grading of Recommendations Assessment, Development and Evaluation; IPD, individual participant data; IYCF, infant and young child feeding; LAZ, length-for-age *z* score; LMIC, low- and middle-income country; LNS, lipid-based nutrient supplement; MAM, moderate acute malnutrition; MDAT, Malawi Developmental Assessment Tool; MUAC, midupper arm circumference; SES, socioeconomic status; SQ, small-quantity; WASH, water, sanitation, and hygiene; WLZ, weight-for-length *z* score.
