# Supplementary material for: Small-quantity lipid-based nutrient supplements for children age 6–24 months: a systematic review and individual participant data meta-analysis of effects on developmental outcomes and effect modifiers
Source: Am J Clin Nutr. 2021 Sep 29;114(Suppl 1):43S–67S. doi: 10.1093/ajcn/nqab277 (PMC8560311; doi:10.1093/ajcn/nqab277)
Supplement: nqab277_Supplemental_Files [file nqab277_supplemental_files.zip › 3_LNS_IPD_Supplemental_Figures_TOC_2021-04-01.docx]

**Online Supplemental Material**

Small-quantity lipid-based nutrient supplements for children age 6-24 months: a systematic review and individual participant data meta-analysis of effects on developmental outcomes and effect modifiers

Prado *et al.* (2021)

**Table of Contents: Supplemental Figures**

Supplemental Figure 1. Summary risk of bias as a percentage of all included studies for the effects of SQ-LNS on developmental outcomes

Supplemental Figure 2. Sensitivity analyses of main effects of SQ-LNS on developmental outcomes

Supplemental Figure 3. Forest plots for all main effects of SQ-LNS on developmental outcomes

Supplemental Figure 4. Forest plots for effects of SQ-LNS on developmental outcomes stratified by implementation within existing programs versus by a research team.

Supplemental Figure 5. Forest plots for effects of SQ-LNS on developmental outcomes stratified by the extent of social and behavior change communication on IYCF that was provided.

Supplemental Figure 6. Forest plots for effects of SQ-LNS on developmental outcomes stratified by study-level effect modifiers

Supplemental Figure 7. Forest plots for effects of SQ-LNS on developmental outcomes stratified by individual-level maternal and child effect modifiers

Supplemental Figure 8. Forest plots for effects of SQ-LNS on developmental outcomes stratified by all individual-level household effect modifiers
