# Supplementary material for: Small-quantity lipid-based nutrient supplements for children age 6–24 months: a systematic review and individual participant data meta-analysis of effects on developmental outcomes and effect modifiers
Source: Am J Clin Nutr. 2021 Sep 29;114(Suppl 1):43S–67S. doi: 10.1093/ajcn/nqab277 (PMC8560311; doi:10.1093/ajcn/nqab277)

Online supplemental material: Small-quantity lipid-based nutrient supplements for children age 6-24 months: a systematic review and individual participant data meta-analysis of effects on developmental outcomes and effect modifiers. Prado et al. (2021)

Supplemental Figure 1: Summary risk of bias as a percentage of all included studies for the effects of SQ-LNS on developmental outcomes

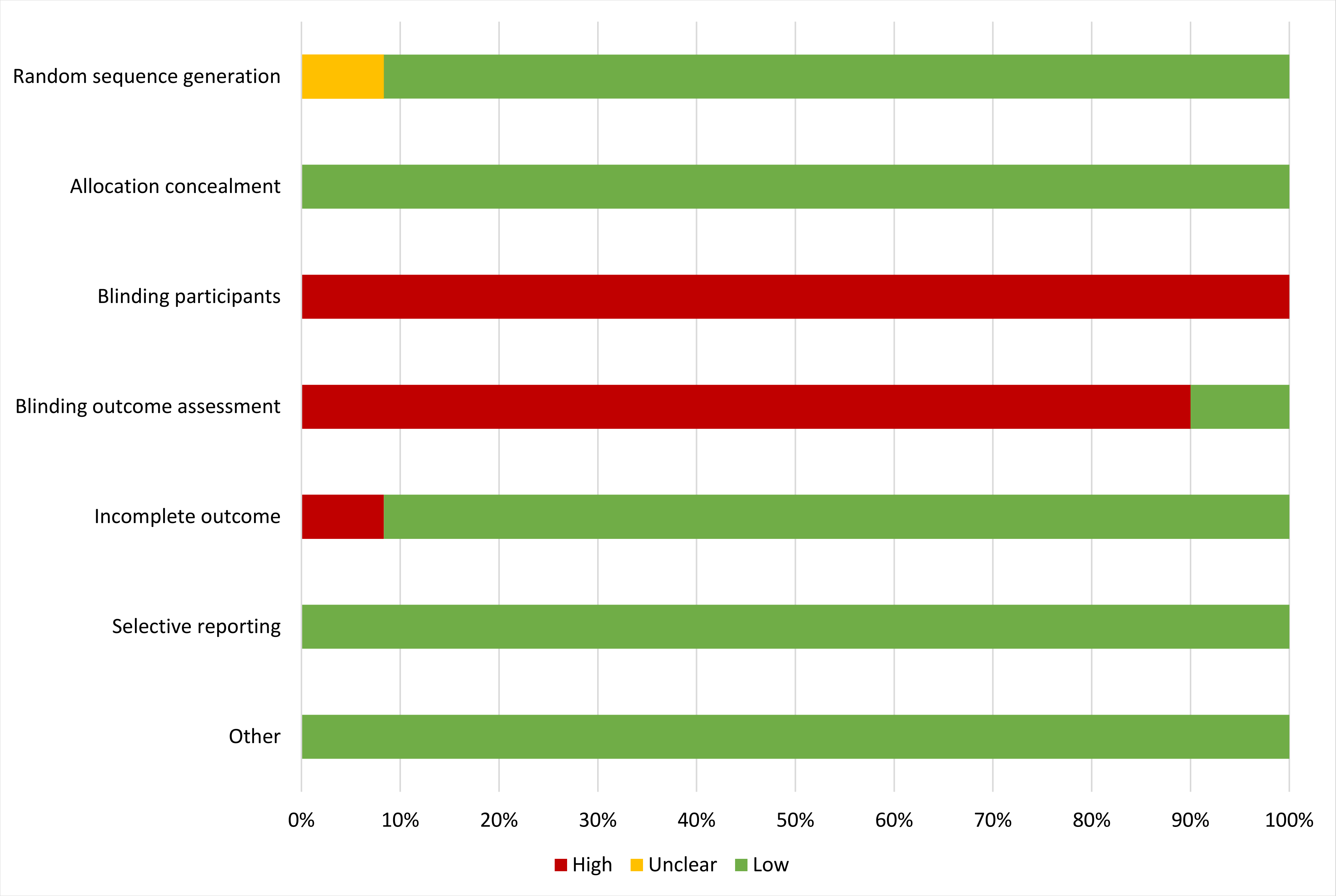

Supplement: nqab277_Supplemental_Files [file nqab277_supplemental_files.zip › ipdd_suppfig1_20210401.pdf]
