# Supplementary material for: Small-quantity lipid-based nutrient supplements for children age 6–24 months: a systematic review and individual participant data meta-analysis of effects on developmental outcomes and effect modifiers
Source: Am J Clin Nutr. 2021 Sep 29;114(Suppl 1):43S–67S. doi: 10.1093/ajcn/nqab277 (PMC8560311; doi:10.1093/ajcn/nqab277)

Supplemental figure 2: Sensitivity analyses of main effects of SQ-LNS on developmental outcomes

Contents

Supplemental figure 2A: Mean differences for continuous outcomes **2**

Supplemental figure 2B: Prevalence ratios for dichotomous outcomes **3**

Supplemental figure 2C: Prevalence differences for dichotomous outcomes **4**

Supplemental figure 2D: Prevalence ratios for 12 mo milestone outcomes **5**

Supplemental figure 2E: Prevalence differences for 12 mo milestone outcomes **6**

Supplemental figure 2F: Prevalence ratios for 18 mo milestone outcomes **7**

Supplemental figure 2G: Prevalence differences for 18 mo milestone outcomes **8**

These figures show the pooled estimates of intervention effects by different pooling methods and different sensitivity analyses. For continuous outcomes, the intervention effect is measured by the difference in mean of the LNS group minus control. For dichotomous outcomes analyzed via prevalence ratios, the effect estimate is the prevalence in the LNS group divided by the prevalence in the control group. For dichotomous outcomes analyzed via prevalence differences, the effect estimate is the prevalence in the LNS group minus the prevalence in the control group. The labels on the left y-axis indicate which outcome is assessed. The different columns correspond to sensitivity analyses in which intervention group categorization differs. All-trial analysis includes all trials; Child-LNS-only excludes trial arms that provided both maternal and child LNS; Multi-component analysis separates comparisons within trials that included multi-component interventions, so that the SQ-LNS vs. no SQ-LNS comparisons were conducted separately between pairs of arms that included the same non-nutrition components (e.g. SQ-LNS+WASH vs. WASH; SQ-LNS vs. Control); Passive arms excluded analysis excludes passive control arms.

Supplemental figure 2A: Mean differences for continuous outcomes

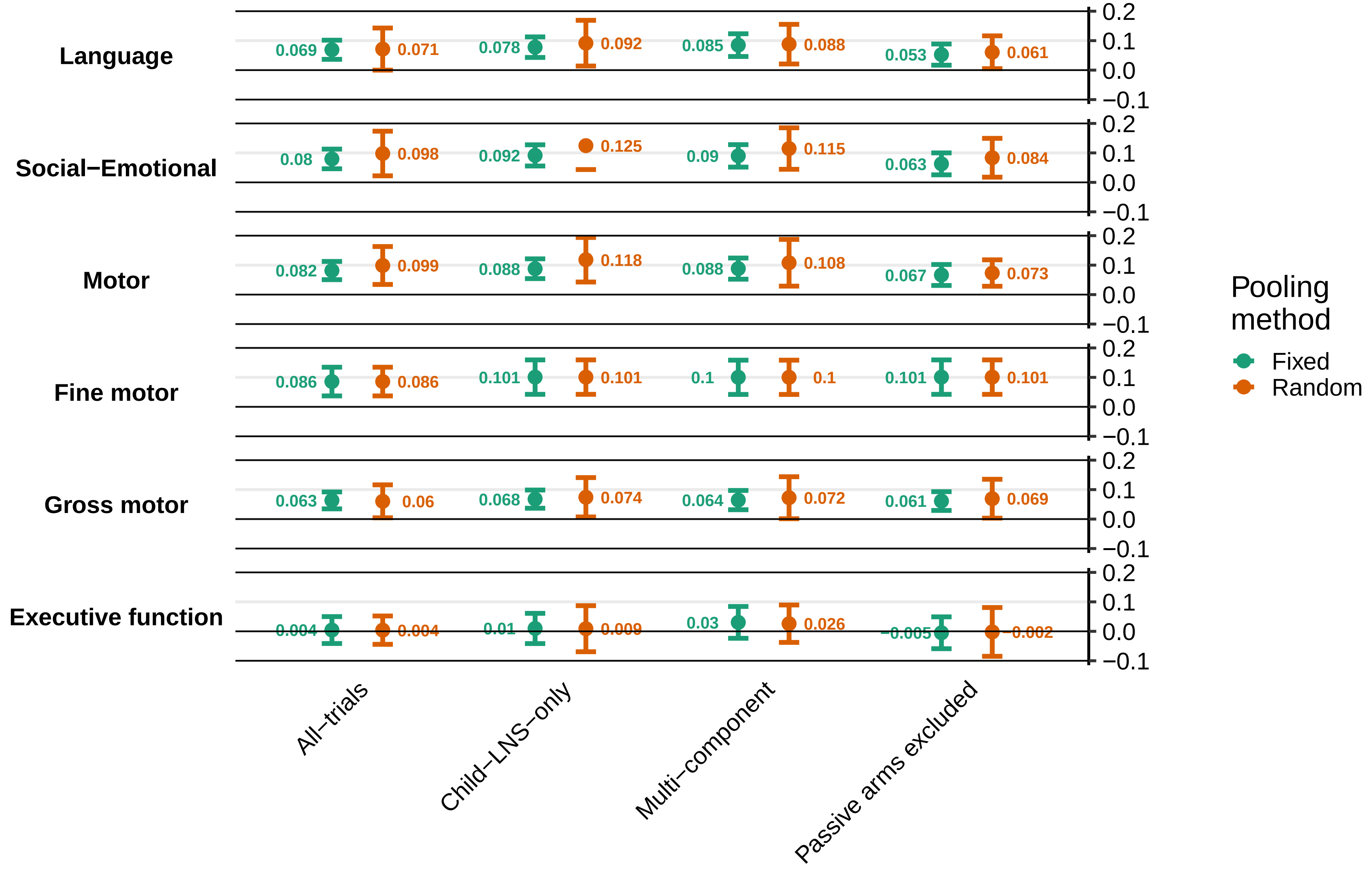

Supplemental figure 2B: Prevalence ratios for dichotomous outcomes

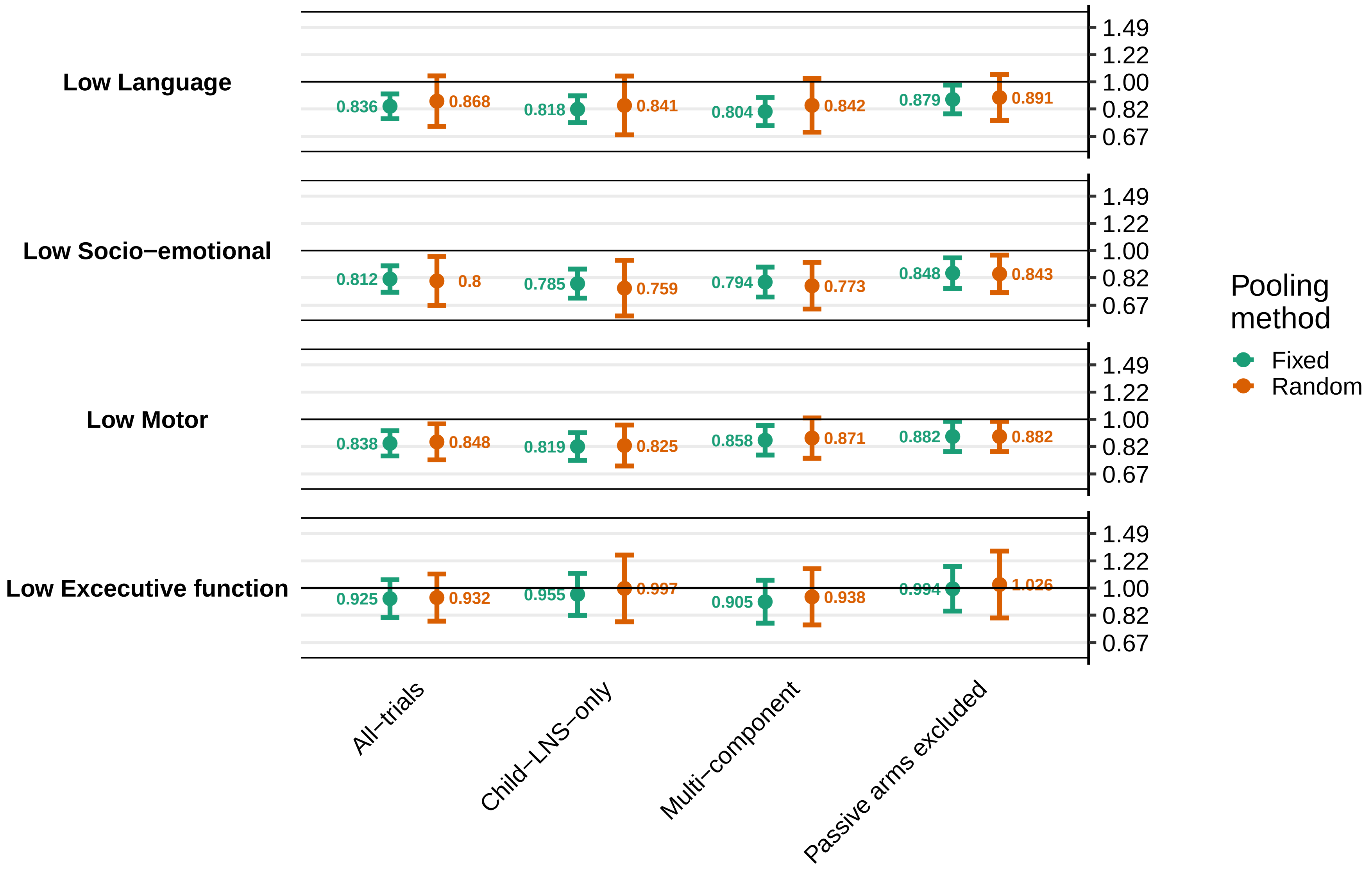

Supplemental figure 2C: Prevalence differences for dichotomous outcomes

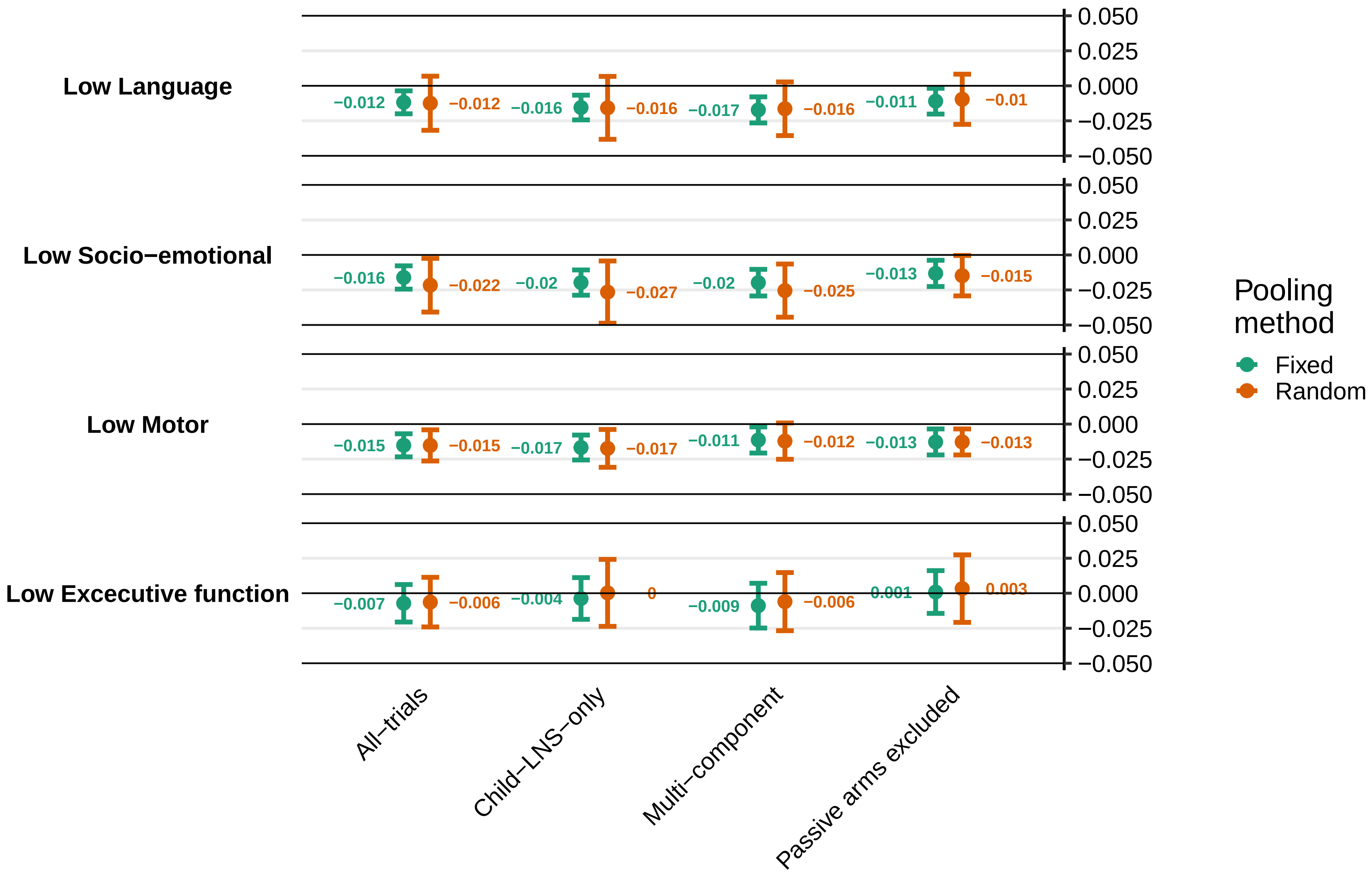

Supplemental figure 2D: Prevalence ratios for 12 mo milestone outcomes

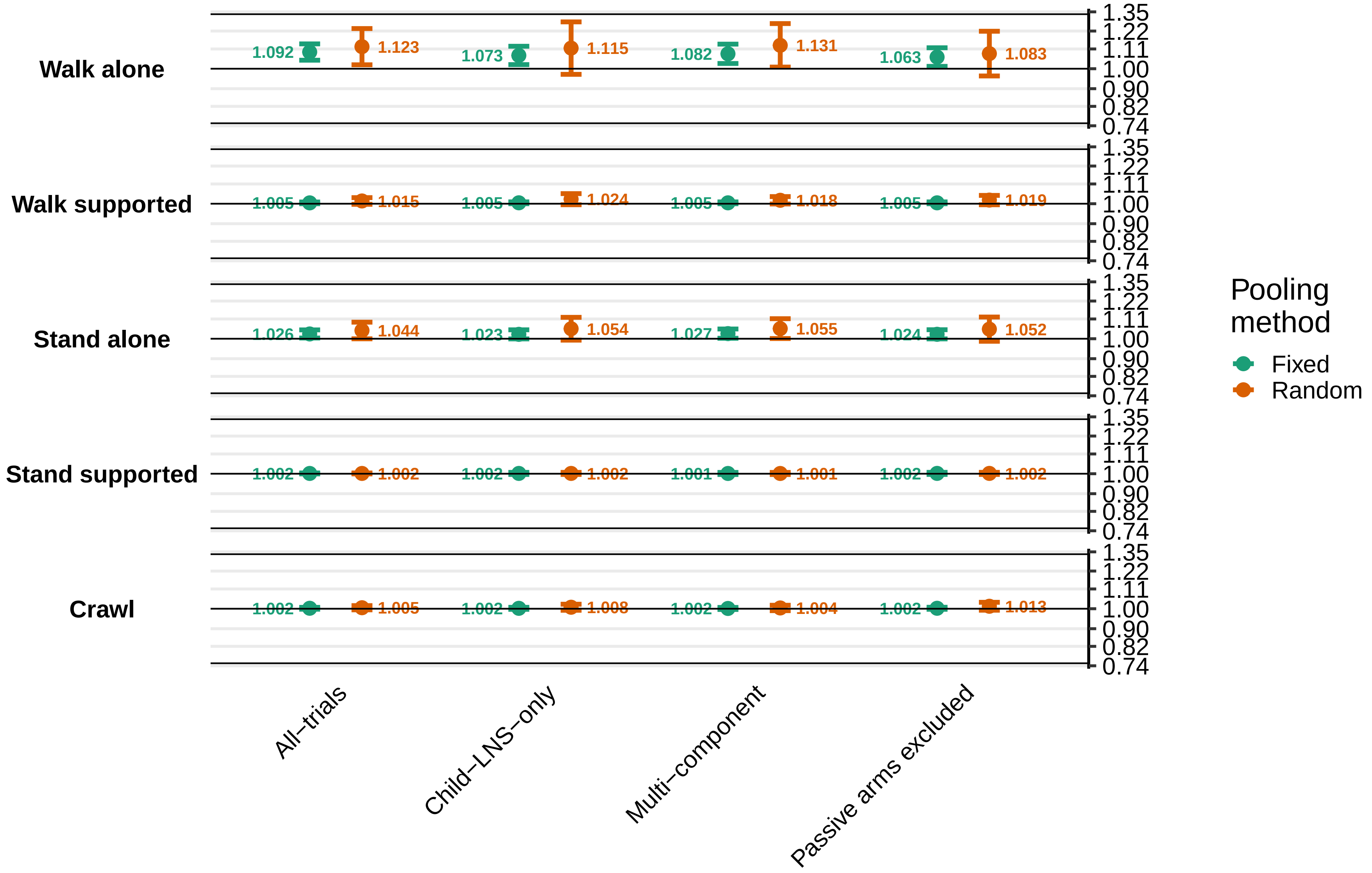

Supplemental figure 2E: Prevalence differences for 12 mo milestone outcomes

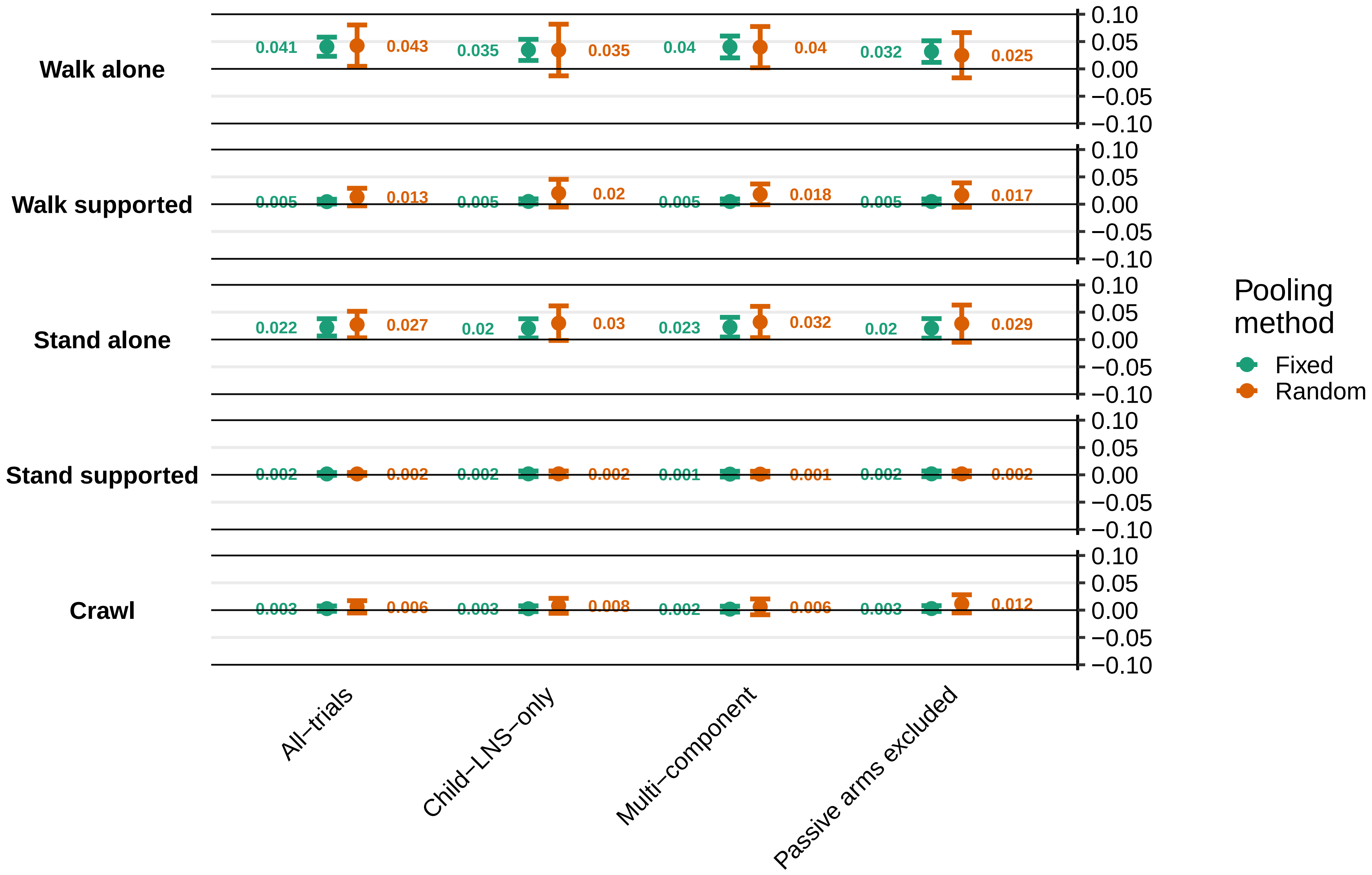

Supplemental figure 2F: Prevalence ratios for 18 mo milestone outcomes

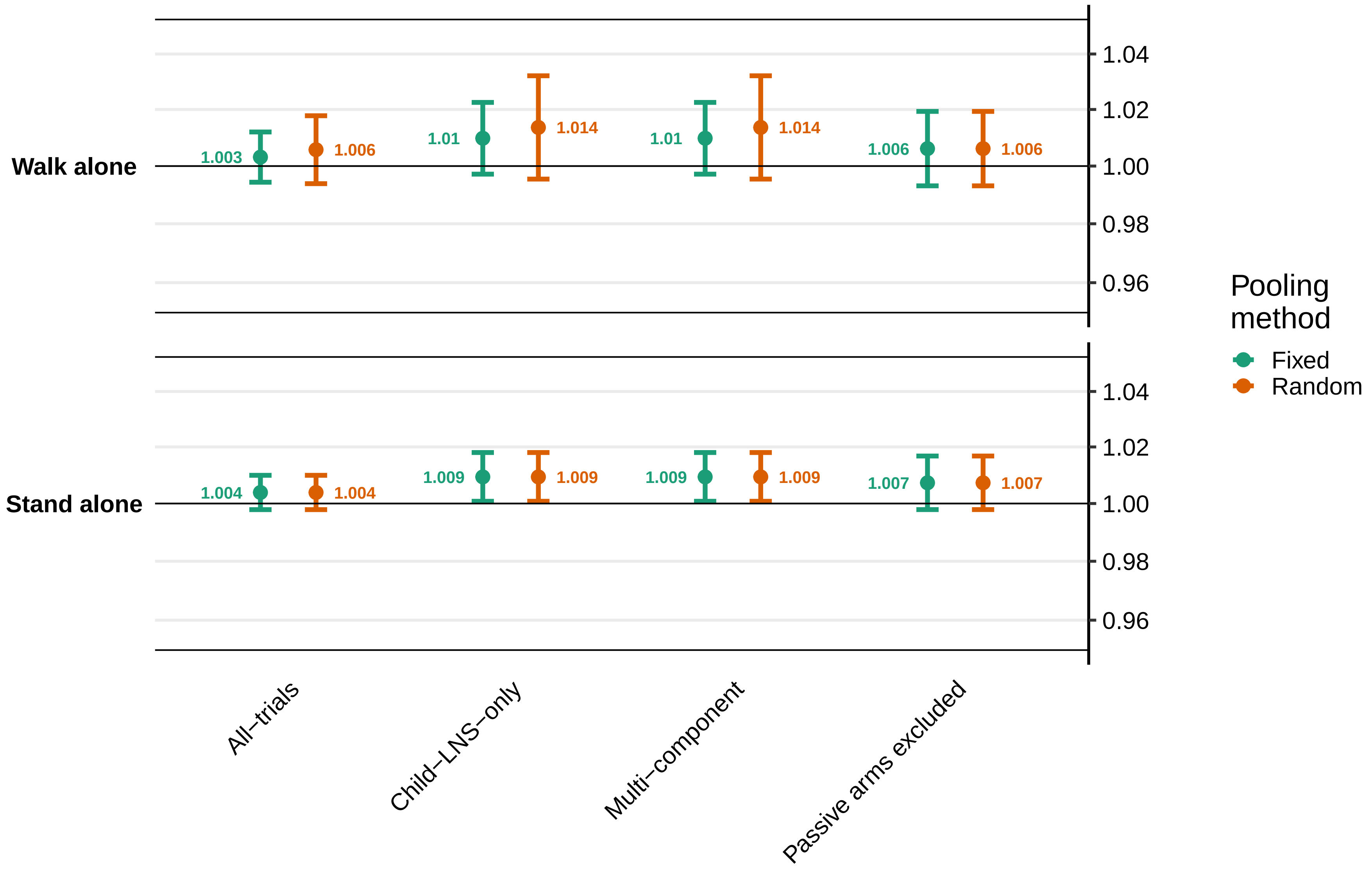

Supplemental figure 2G: Prevalence differences for 18 mo milestone outcomes

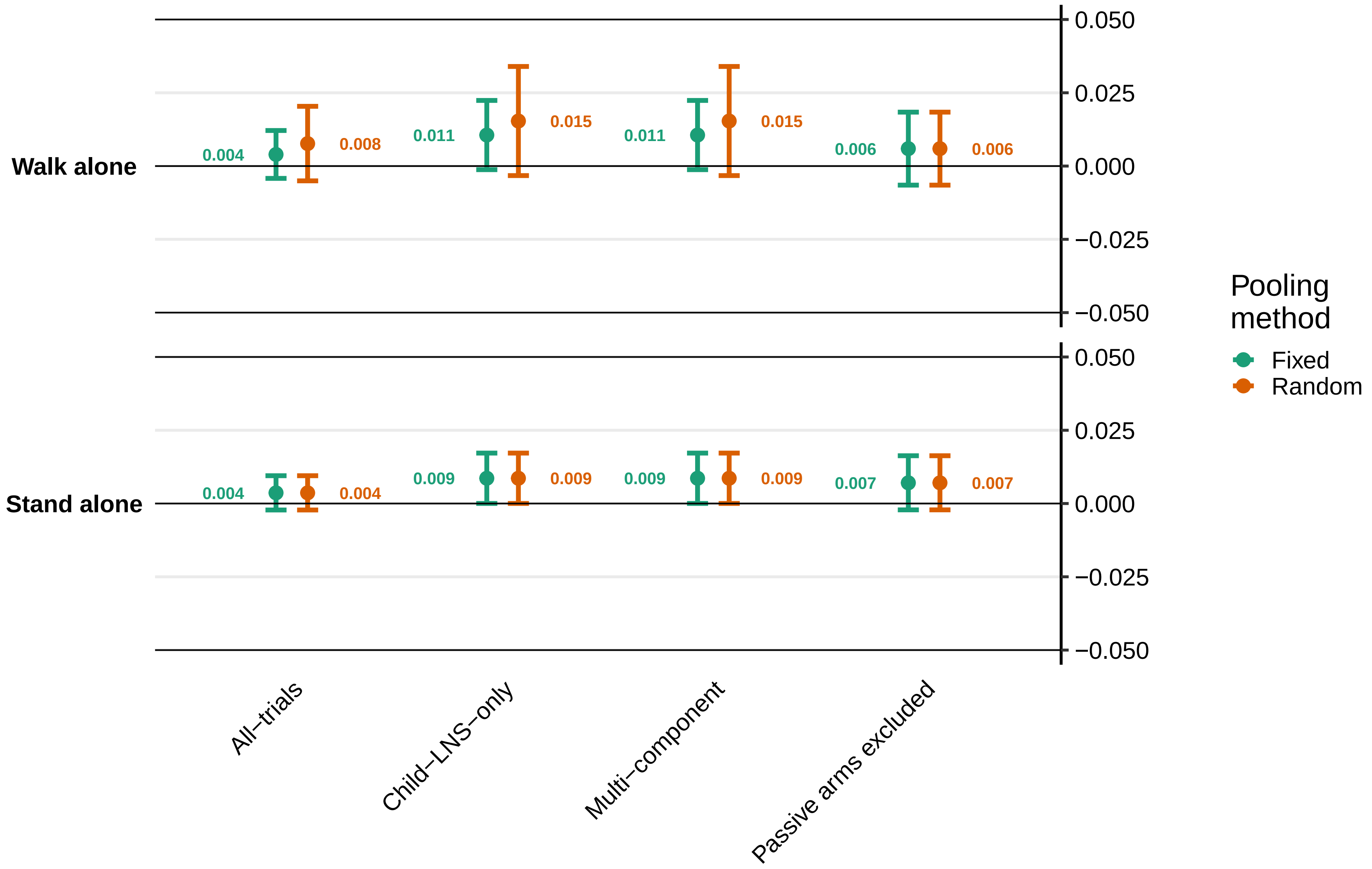

Supplement: nqab277_Supplemental_Files [file nqab277_supplemental_files.zip › ipdd_suppfig2_20210401.pdf]
