# Supplementary material for: Small-quantity lipid-based nutrient supplements for children age 6–24 months: a systematic review and individual participant data meta-analysis of effects on developmental outcomes and effect modifiers
Source: Am J Clin Nutr. 2021 Sep 29;114(Suppl 1):43S–67S. doi: 10.1093/ajcn/nqab277 (PMC8560311; doi:10.1093/ajcn/nqab277)

# Supplemental figure 3: Forest plots for all main effects of SQ-LNS on developmental outcomes

## Contents


**Supplemental figure 3A: Mean difference in language z-score** 3

**Supplemental figure 3B: Language lowest decile prevalence ratio** 4

**Supplemental figure 3C: Language lowest decile prevalence difference** 5

**Supplemental figure 3D: Mean difference in social-emotional z-score** 6

**Supplemental figure 3E: Social-emotional lowest decile prevalence ratio** 7

**Supplemental figure 3F: Social-emotional lowest decile prevalence difference** 8

**Supplemental figure 3G: Mean difference in motor z-score** 9

**Supplemental figure 3H: Motor lowest decile prevalence ratio** 10

**Supplemental figure 3I: Motor lowest decile prevalence difference** 11

**Supplemental figure 3J: Mean difference in gross motor z-score** 12

**Supplemental figure 3K: Mean difference in fine motor z-score** 13

**Supplemental figure 3L: Mean difference in executive function z-score** 14

**Supplemental figure 3M: Executive function lowest decile prevalence ratio** 15

**Supplemental figure 3N: Executive function lowest decile prevalence difference** 16

**Supplemental figure 3O: 12-mo walking without support prevalence ratio** 17

**Supplemental figure 3P: 12-mo walking without support prevalence difference** 18

**Supplemental figure 3Q: 12-mo walking with support prevalence ratio** 19

**Supplemental figure 3R: 12-mo walking with support prevalence difference** 20

**Supplemental figure 3S: 12-mo standing without support prevalence ratio** 21

**Supplemental figure 3T: 12-mo standing without support prevalence difference** 22

**Supplemental figure 3U: 12-mo standing with support prevalence ratio** 23

**Supplemental figure 3V: 12-mo standing with support prevalence difference** 24

**Supplemental figure 3W: 12-mo crawling prevalence ratio** 25

**Supplemental figure 3X: 12-mo crawling prevalence difference** 26

**Supplemental figure 3Y: 18-mo walking without support prevalence ratio** 27

**Supplemental figure 3Z: 18-mo walking without support prevalence difference** 28

**Supplemental figure 3AA: 18-mo walking with support prevalence ratio** **29**

**Supplemental figure 3AB: 18-mo walking with support prevalence difference** **30**

These figures are forest plots showing the study-level estimates of intervention effect with the pooled estimate in the bottom summary rows. Individual study estimates were generated from log-binomial regression for dichotomous outcomes and simple linear regression for continuous outcomes; controlling for baseline measure when available and with clustered observations using robust standard errors for cluster-randomized trials. Pooled estimates were generated using inverse variance weighting in both fixed and random effects models. For continuous outcomes the intervention effect is measured by the difference in mean of the LNS group minus control. For dichotomous outcomes analyzed via prevalence ratios the effect estimate is the prevalence in the LNS group divided by the prevalence in the control group. For dichotomous outcomes analyzed via prevalence differences the effect estimate is the prevalence in the LNS group minus the prevalence in the control group.

The labels on the left y-axis correspond to trial level information. The values on the right indicate the study level effect estimate, confidence interval, and weighting for deriving the pooled estimate.

Figures showing individual trial estimates for the SHINE trial are split by comparison to reflect the cross-over design. For calculating the pooled estimates shown in these figures, the trial is analyzed with LNS intervention arms combined and non-LNS intervention arms combined.

# Supplemental figure 3A: Mean difference in language z-score

| Country | Trial | Tool | LNS N | Control N | MD (95% CI) | Fixed W | Random W |
|---|---|---|---|---|---|---|---|
| Bangladesh | JiVitA−4 (21) | BSID−III | 445 | 143 | −0.08 (−0.29, 0.12) | 0.03 | 0.06 |
| Bangladesh | RDNS (22) | CDI | 1663 | 814 | 0.17 (0.07, 0.26) | 0.11 | 0.09 |
| Bangladesh | WASH−B (23) | CDI | 1109 | 3353 | 0.09 (0.02, 0.17) | 0.20 | 0.10 |
| Burkina Faso | iLiNS−Zinc (24) | DMC | 746 | 375 | 0.37 (0.21, 0.53) | 0.04 | 0.07 |
| Ghana | GHANA (25) | | | | | | |
| Ghana | iLiNS−DYAD−G (26) | CDI | 331 | 658 | 0.01 (−0.12, 0.14) | 0.06 | 0.08 |
| Haiti | HAITI (27) | Total sounds | 150 | 149 | −0.13 (−0.35, 0.10) | 0.02 | 0.05 |
| Kenya | WASH−B (28) | EASQ | 1362 | 4745 | 0.02 (−0.04, 0.08) | 0.30 | 0.11 |
| Madagascar | MAHAY (29) | ASQI | 1613 | 1604 | −0.11 (−0.30, 0.07) | 0.03 | 0.07 |
| Malawi | iLiNS−DYAD−M (30) | CDI | 215 | 439 | 0.01 (−0.15, 0.18) | 0.04 | 0.07 |
| Malawi | iLiNS−DOSE (31) | CDI | 645 | 221 | 0.05 (−0.11, 0.20) | 0.05 | 0.08 |
| Mali | PROMIS CS (32) | DMC | 927 | 944 | 0.15 (−0.02, 0.31) | 0.04 | 0.07 |
| Zimbabwe | SHINE (HIV−) (33), LNS vs SOC | CDI | 381 | 373 | 0.03 (−0.15, 0.21) | 0.07 | 0.09 |
| Zimbabwe | LNS+WSH vs WSH | CDI | 436 | 408 | 0.13 (−0.02, 0.29) | 0.07 | 0.09 |
| Zimbabwe | SHINE (HIV+) (34), LNS vs SOC | CDI | 66 | 68 | 0.06 (−0.29, 0.40) | 0.02 | 0.05 |
| Zimbabwe | LNS+WSH vs WSH | CDI | 99 | 79 | 0.37 (0.07, 0.67) | 0.02 | 0.05 |
| | **I² = 0.64, Tau² = 0.01** | | **10188** | **14373** | | | |
| | **Fixed** | | | | **0.07 (0.04, 0.10)** | | |
| | **Random** | | | | **0.07 (0.00, 0.14)** | | |

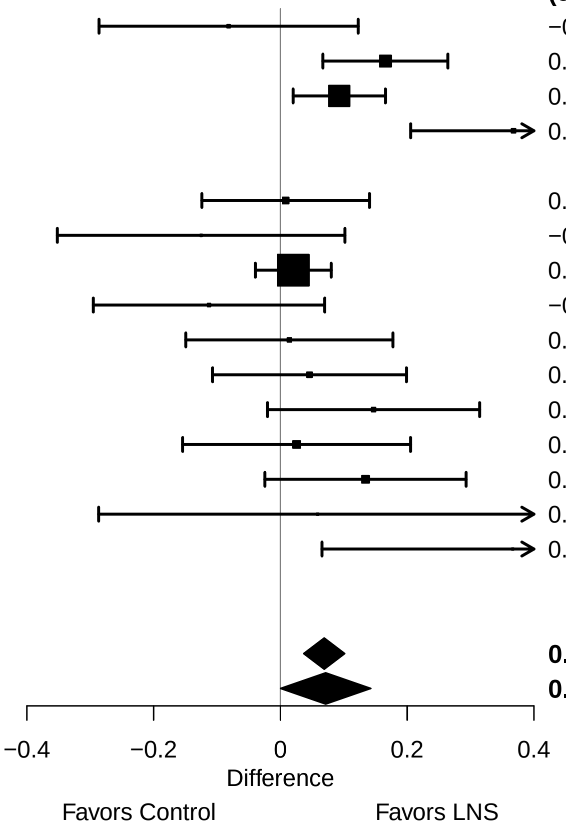

## Supplemental figure 3B: Language lowest decile prevalence ratio

| Country | Trial | Tool | LNS N | Control N | PR (95% CI) | Fixed W | Random W |
|---|---|---|---|---|---|---|---|
| Bangladesh | JiVitA−4 (21) | BSID−III | 445 | 143 | 0.86 (0.50, 1.49) | 0.03 | 0.06 |
| Bangladesh | RDNS (22) | CDI | 1663 | 814 | 0.72 (0.57, 0.91) | 0.15 | 0.10 |
| Bangladesh | WASH−B (23) | CDI | 1109 | 3353 | 0.81 (0.65, 1.00) | 0.18 | 0.10 |
| Burkina Faso | iLiNS−Zinc (24) | DMC | 746 | 375 | 0.46 (0.33, 0.64) | 0.08 | 0.09 |
| Ghana | GHANA (25) | | | | | | |
| Ghana | iLiNS−DYAD−G (26) | CDI | 331 | 658 | 1.09 (0.74, 1.61) | 0.05 | 0.08 |
| Haiti | HAITI (27) | Total sounds | 150 | 149 | 2.75 (1.28, 5.93) | 0.01 | 0.04 |
| Kenya | WASH−B (28) | EASQ | 1362 | 4745 | 0.95 (0.78, 1.16) | 0.21 | 0.11 |
| Madagascar | MAHAY (29) | ASQI | 1613 | 1604 | 1.17 (0.75, 1.83) | 0.04 | 0.07 |
| Malawi | iLiNS−DYAD−M (30) | CDI | 215 | 439 | 0.87 (0.53, 1.43) | 0.03 | 0.06 |
| Malawi | iLiNS−DOSE (31) | CDI | 645 | 221 | 0.95 (0.61, 1.50) | 0.04 | 0.07 |
| Mali | PROMIS CS (32) | DMC | 927 | 944 | 0.77 (0.56, 1.07) | 0.08 | 0.09 |
| Zimbabwe | SHINE (HIV−) (33), LNS vs SOC | CDI | 381 | 373 | 1.05 (0.67, 1.65) | 0.08 | 0.09 |
| Zimbabwe | LNS+WSH vs WSH | CDI | 436 | 408 | 0.77 (0.49, 1.20) | 0.08 | 0.09 |
| Zimbabwe | SHINE (HIV+) (34), LNS vs SOC | CDI | 66 | 68 | 1.29 (0.54, 3.06) | 0.02 | 0.05 |
| Zimbabwe | LNS+WSH vs WSH | CDI | 99 | 79 | 0.32 (0.11, 0.92) | 0.02 | 0.05 |
| | **I² = 0.61, Tau² = 0.07** | | **10188** | **14373** | | | |
| | **Fixed** | | | | **0.84 (0.76, 0.92)** | | |
| | **Random** | | | | **0.87 (0.72, 1.04)** | | |

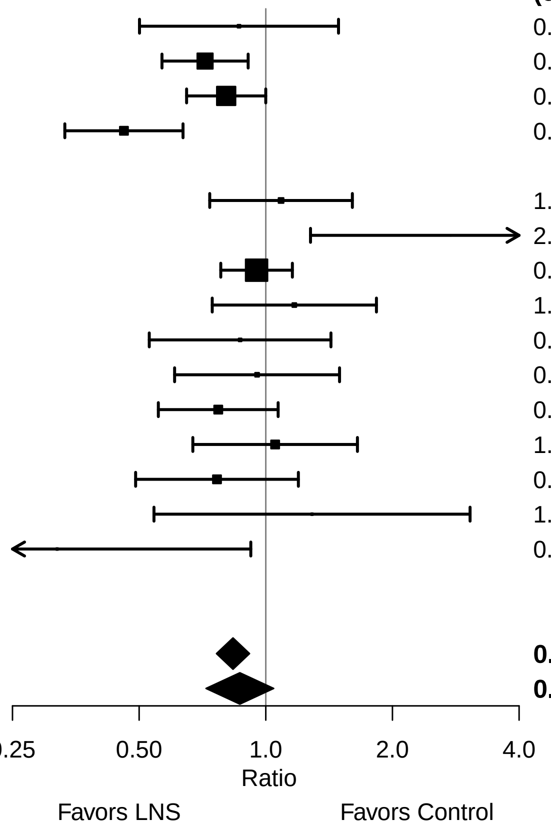

# Supplemental figure 3C: Language lowest decile prevalence difference

| Country | Trial | Tool | LNS N | Control N | PD (95% CI) | Fixed W | Random W |
|---|---|---|---|---|---|---|---|
| Bangladesh | JiVitA−4 (21) | BSID−III | 445 | 143 | −0.02 (−0.07, 0.04) | 0.02 | 0.05 |
| Bangladesh | RDNS (22) | CDI | 1663 | 814 | −0.04 (−0.06, −0.01) | 0.10 | 0.09 |
| Bangladesh | WASH−B (23) | CDI | 1109 | 3353 | −0.02 (−0.04, 0.00) | 0.18 | 0.10 |
| Burkina Faso | iLiNS−Zinc (24) | DMC | 746 | 375 | −0.08 (−0.13, −0.04) | 0.04 | 0.07 |
| Ghana | GHANA (25) | | | | | | |
| Ghana | iLiNS−DYAD−G (26) | CDI | 331 | 658 | 0.01 (−0.03, 0.05) | 0.04 | 0.07 |
| Haiti | HAITI (27) | Total sounds | 150 | 149 | 0.10 (0.03, 0.16) | 0.02 | 0.05 |
| Kenya | WASH−B (28) | EASQ | 1362 | 4745 | 0.00 (−0.02, 0.01) | 0.21 | 0.10 |
| Madagascar | MAHAY (29) | ASQI | 1613 | 1604 | 0.02 (−0.01, 0.04) | 0.16 | 0.10 |
| Malawi | iLiNS−DYAD−M (30) | CDI | 215 | 439 | −0.01 (−0.06, 0.03) | 0.03 | 0.07 |
| Malawi | iLiNS−DOSE (31) | CDI | 645 | 221 | 0.00 (−0.05, 0.04) | 0.03 | 0.07 |
| Mali | PROMIS CS (32) | DMC | 927 | 944 | −0.03 (−0.05, 0.00) | 0.09 | 0.09 |
| Zimbabwe | SHINE (HIV−) (33), LNS vs SOC | CDI | 381 | 373 | 0.01 (−0.04, 0.05) | 0.08 | 0.09 |
| Zimbabwe | LNS+WSH vs WSH | CDI | 436 | 408 | −0.03 (−0.06, 0.01) | 0.08 | 0.09 |
| Zimbabwe | SHINE (HIV+) (34), LNS vs SOC | CDI | 66 | 68 | 0.03 (−0.08, 0.15) | 0.01 | 0.05 |
| Zimbabwe | LNS+WSH vs WSH | CDI | 99 | 79 | −0.09 (−0.17, 0.00) | 0.01 | 0.05 |
| | **I² = 0.66, Tau² = 0.00** | | **10188** | **14373** | | | |
| | **Fixed** | | | | **−0.01 (−0.02, 0.00)** | | |
| | **Random** | | | | **−0.01 (−0.03, 0.01)** | | |

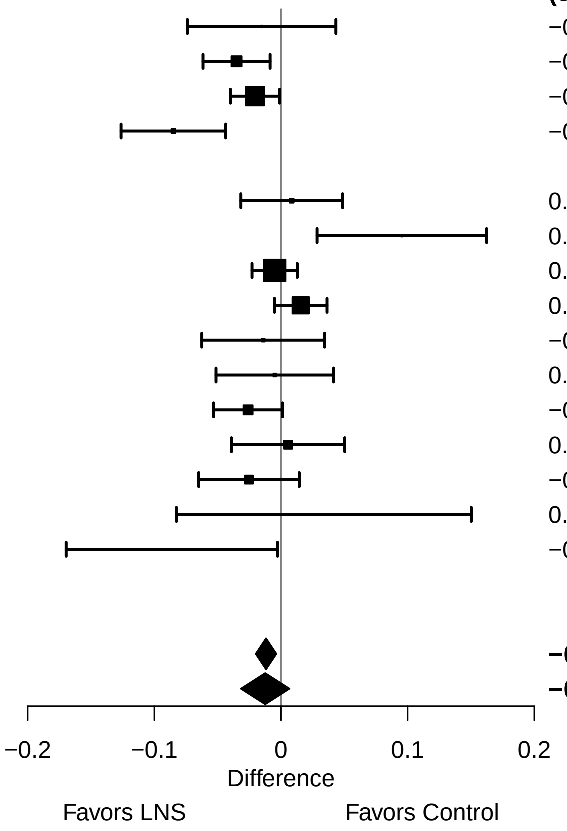

## Supplemental figure 3D: Mean difference in social-emotional z-score

| Country | Trial | Tool | LNS N | Control N | MD (95% CI) | Fixed W | Random W |
|---|---|---|---|---|---|---|---|
| Bangladesh | JiVitA−4 (21) | | | | | | |
| Bangladesh | RDNS (22) | DMC | 1657 | 815 | 0.06 (−0.04, 0.16) | 0.12 | 0.11 |
| Bangladesh | WASH−B (23) | EASQ | 1066 | 3266 | 0.12 (0.05, 0.20) | 0.21 | 0.11 |
| Burkina Faso | iLiNS−Zinc (24) | DMC | 746 | 375 | 0.36 (0.18, 0.53) | 0.04 | 0.08 |
| Ghana | GHANA (25) | | | | | | |
| Ghana | iLiNS−DYAD−G (26) | PSED | 332 | 657 | 0.01 (−0.12, 0.14) | 0.06 | 0.09 |
| Haiti | HAITI (27) | | | | | | |
| Kenya | WASH−B (28) | EASQ | 1362 | 4745 | 0.01 (−0.05, 0.08) | 0.27 | 0.12 |
| Madagascar | MAHAY (29) | ASQI | 1613 | 1604 | −0.07 (−0.26, 0.11) | 0.03 | 0.07 |
| Malawi | iLiNS−DYAD−M (30) | PSED | 215 | 438 | 0.01 (−0.15, 0.18) | 0.04 | 0.08 |
| Malawi | iLiNS−DOSE (31) | PSED | 644 | 221 | 0.03 (−0.12, 0.18) | 0.05 | 0.08 |
| Mali | PROMIS CS (32) | DMC | 927 | 944 | 0.15 (−0.02, 0.31) | 0.04 | 0.08 |
| Zimbabwe | SHINE (HIV−) (33), LNS vs SOC | MDAT | 395 | 382 | 0.07 (−0.07, 0.21) | 0.11 | 0.11 |
| Zimbabwe | LNS+WSH vs WSH | MDAT | 446 | 417 | 0.17 (0.02, 0.31) | 0.11 | 0.11 |
| Zimbabwe | SHINE (HIV+) (34), LNS vs SOC | MDAT | 67 | 68 | 0.24 (0.00, 0.48) | 0.03 | 0.07 |
| Zimbabwe | LNS+WSH vs WSH | MDAT | 103 | 83 | 0.44 (0.14, 0.73) | 0.03 | 0.07 |
| | **I² = 0.66, Tau² = 0.01** | | **9573** | **14015** | | | |
| | **Fixed** | | | | **0.08 (0.05, 0.11)** | | |
| | **Random** | | | | **0.10 (0.02, 0.17)** | | |

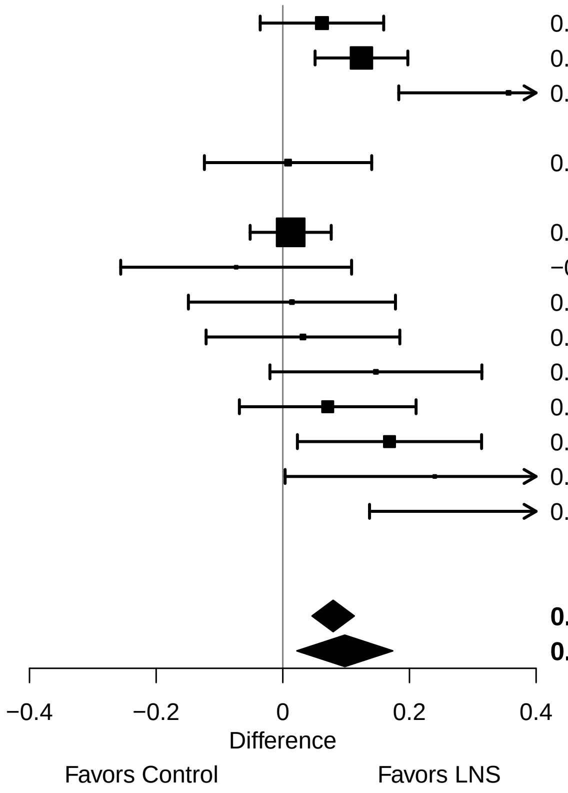

# Supplemental figure 3E: Social-emotional lowest decile prevalence ratio

| Country | Trial | Tool | LNS N | Control N | PR (95% CI) | Fixed W | Random W |
|---|---|---|---|---|---|---|---|
| Bangladesh | JiVitA−4 (21) | | | | | | |
| Bangladesh | RDNS (22) | DMC | 1657 | 815 | 0.84 (0.66, 1.07) | 0.16 | 0.12 |
| Bangladesh | WASH−B (23) | EASQ | 1066 | 3266 | 0.81 (0.64, 1.03) | 0.17 | 0.12 |
| Burkina Faso | iLiNS−Zinc (24) | DMC | 746 | 375 | 0.41 (0.28, 0.62) | 0.06 | 0.08 |
| Ghana | GHANA (25) | | | | | | |
| Ghana | iLiNS−DYAD−G (26) | PSED | 332 | 657 | 0.99 (0.67, 1.47) | 0.06 | 0.09 |
| Haiti | HAITI (27) | | | | | | |
| Kenya | WASH−B (28) | EASQ | 1362 | 4745 | 0.87 (0.71, 1.08) | 0.21 | 0.12 |
| Madagascar | MAHAY (29) | ASQI | 1613 | 1604 | 1.15 (0.75, 1.78) | 0.05 | 0.08 |
| Malawi | iLiNS−DYAD−M (30) | PSED | 215 | 438 | 1.00 (0.61, 1.61) | 0.04 | 0.07 |
| Malawi | iLiNS−DOSE (31) | PSED | 644 | 221 | 1.08 (0.68, 1.72) | 0.04 | 0.07 |
| Mali | PROMIS CS (32) | DMC | 927 | 944 | 0.77 (0.56, 1.07) | 0.09 | 0.10 |
| Zimbabwe | SHINE (HIV−) (33), LNS vs SOC | MDAT | 395 | 382 | 0.86 (0.56, 1.33) | 0.10 | 0.10 |
| Zimbabwe | LNS+WSH vs WSH | MDAT | 446 | 417 | 0.62 (0.41, 0.93) | 0.10 | 0.10 |
| Zimbabwe | SHINE (HIV+) (34), LNS vs SOC | MDAT | 67 | 68 | 0.68 (0.22, 2.04) | 0.02 | 0.05 |
| Zimbabwe | LNS+WSH vs WSH | MDAT | 103 | 83 | 0.33 (0.15, 0.76) | 0.02 | 0.05 |
| | **I² = 0.54, Tau² = 0.06** | | **9573** | **14015** | | | |
| | **Fixed** | | | | **0.81 (0.74, 0.89)** | | |
| | **Random** | | | | **0.80 (0.67, 0.96)** | | |

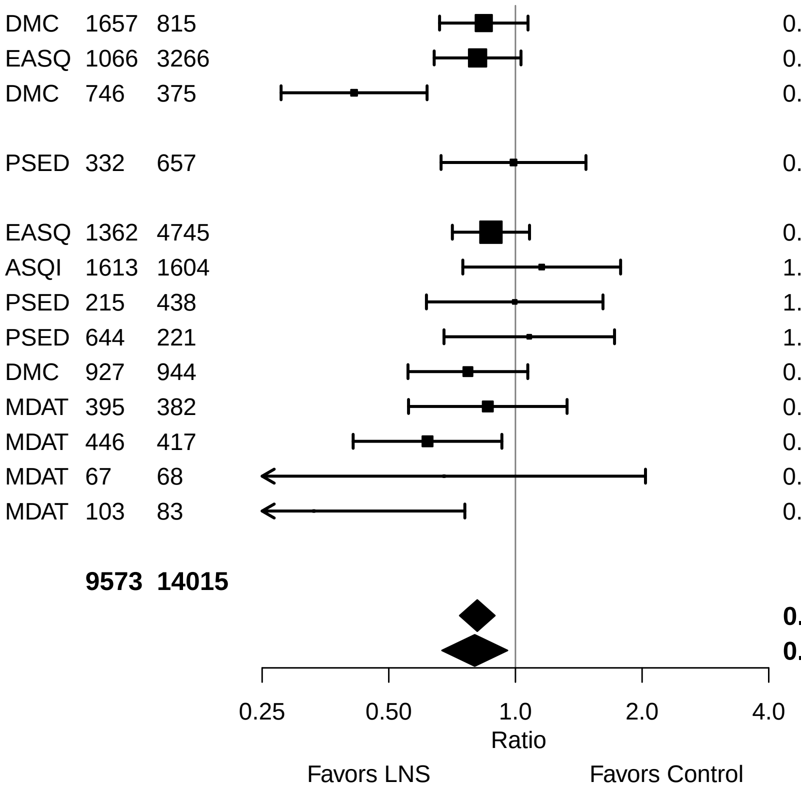

# Supplemental figure 3F: Social-emotional lowest decile prevalence difference

| Country | Trial | Tool | LNS N | Control N | PD (95% CI) | Fixed W | Random W |
|---|---|---|---|---|---|---|---|
| Bangladesh | JiVitA−4 (21) | | | | | | |
| Bangladesh | RDNS (22) | DMC | 1657 | 815 | −0.02 (−0.04, 0.01) | 0.10 | 0.10 |
| Bangladesh | WASH−B (23) | EASQ | 1066 | 3266 | −0.02 (−0.04, 0.00) | 0.18 | 0.11 |
| Burkina Faso | iLiNS−Zinc (24) | DMC | 746 | 375 | −0.10 (−0.14, −0.06) | 0.04 | 0.08 |
| Ghana | GHANA (25) | | | | | | |
| Ghana | iLiNS−DYAD−G (26) | PSED | 332 | 657 | 0.00 (−0.04, 0.04) | 0.04 | 0.08 |
| Haiti | HAITI (27) | | | | | | |
| Kenya | WASH−B (28) | EASQ | 1362 | 4745 | −0.01 (−0.03, 0.00) | 0.23 | 0.12 |
| Madagascar | MAHAY (29) | ASQI | 1613 | 1604 | 0.01 (−0.01, 0.04) | 0.16 | 0.11 |
| Malawi | iLiNS−DYAD−M (30) | PSED | 215 | 438 | 0.00 (−0.05, 0.05) | 0.03 | 0.07 |
| Malawi | iLiNS−DOSE (31) | PSED | 644 | 221 | 0.01 (−0.04, 0.05) | 0.03 | 0.07 |
| Mali | PROMIS CS (32) | DMC | 927 | 944 | −0.03 (−0.05, 0.00) | 0.09 | 0.10 |
| Zimbabwe | SHINE (HIV−) (33), LNS vs SOC | MDAT | 395 | 382 | −0.01 (−0.05, 0.03) | 0.08 | 0.10 |
| Zimbabwe | LNS+WSH vs WSH | MDAT | 446 | 417 | −0.05 (−0.10, −0.01) | 0.08 | 0.10 |
| Zimbabwe | SHINE (HIV+) (34), LNS vs SOC | MDAT | 67 | 68 | −0.03 (−0.12, 0.06) | 0.01 | 0.05 |
| Zimbabwe | LNS+WSH vs WSH | MDAT | 103 | 83 | −0.14 (−0.24, −0.04) | 0.01 | 0.05 |
| | **$I^2$ = 0.68, $Tau^2$ = 0.00** | | **9573** | **14015** | | | |
| | **Fixed** | | | | **−0.02 (−0.02, −0.01)** | | |
| | **Random** | | | | **−0.02 (−0.04, 0.00)** | | |

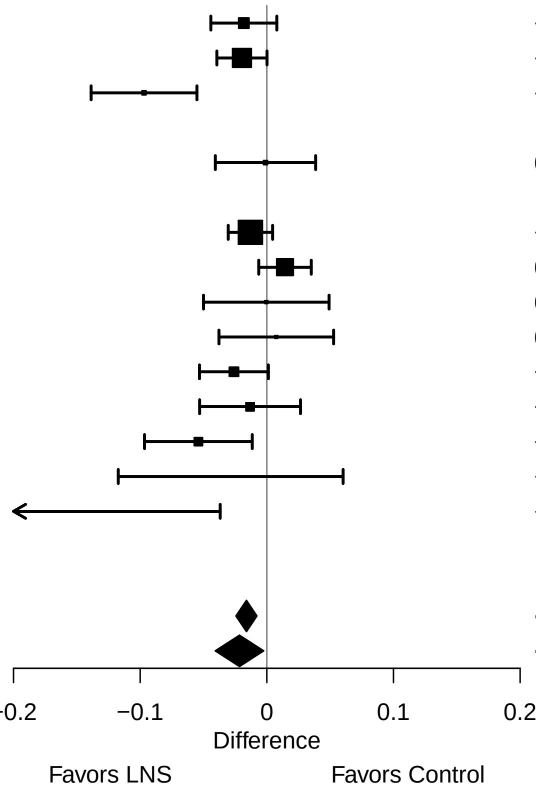

## Supplemental figure 3G: Mean difference in motor z-score

| Country | Trial | Tool | LNS N | Control N | MD (95% CI) | Fixed W | Random W |
|---|---|---|---|---|---|---|---|
| Bangladesh | JiVitA−4 (21) | BSID−III | 445 | 143 | −0.02 (−0.17, 0.14) | 0.04 | 0.07 |
| Bangladesh | RDNS (22) | DMC | 1556 | 753 | 0.10 (0.03, 0.17) | 0.19 | 0.11 |
| Bangladesh | WASH−B (23) | EASQ | 1074 | 3279 | 0.10 (0.04, 0.17) | 0.21 | 0.12 |
| Burkina Faso | iLiNS−Zinc (24) | DMC | 746 | 375 | 0.40 (0.24, 0.57) | 0.04 | 0.07 |
| Ghana | GHANA (25) | | | | | | |
| Ghana | iLiNS−DYAD−G (26) | KDI | 302 | 601 | 0.03 (−0.11, 0.16) | 0.05 | 0.08 |
| Haiti | HAITI (27) | | | | | | |
| Kenya | WASH−B (28) | EASQ | 1362 | 4745 | 0.01 (−0.05, 0.07) | 0.23 | 0.12 |
| Madagascar | MAHAY (29) | ASQI | 1613 | 1604 | 0.07 (−0.13, 0.27) | 0.02 | 0.06 |
| Malawi | iLiNS−DYAD−M (30) | KDI | 214 | 436 | 0.03 (−0.13, 0.18) | 0.04 | 0.07 |
| Malawi | iLiNS−DOSE (31) | KDI | 646 | 221 | 0.05 (−0.10, 0.20) | 0.04 | 0.08 |
| Mali | PROMIS CS (32) | DMC | 902 | 921 | 0.13 (−0.04, 0.30) | 0.03 | 0.07 |
| Zimbabwe | SHINE (HIV−) (33), LNS vs SOC | MDAT | 395 | 382 | 0.00 (−0.16, 0.15) | 0.08 | 0.10 |
| Zimbabwe | LNS+WSH vs WSH | MDAT | 446 | 417 | 0.20 (0.05, 0.35) | 0.08 | 0.10 |
| Zimbabwe | SHINE (HIV+) (34), LNS vs SOC | MDAT | 67 | 68 | 0.04 (−0.30, 0.39) | 0.02 | 0.05 |
| Zimbabwe | LNS+WSH vs WSH | MDAT | 103 | 83 | 0.49 (0.23, 0.74) | 0.02 | 0.05 |
| | **I² = 0.60, Tau² = 0.01** | | **9871** | **14028** | | | |
| | **Fixed** | | | | **0.08 (0.05, 0.11)** | | |
| | **Random** | | | | **0.10 (0.03, 0.16)** | | |

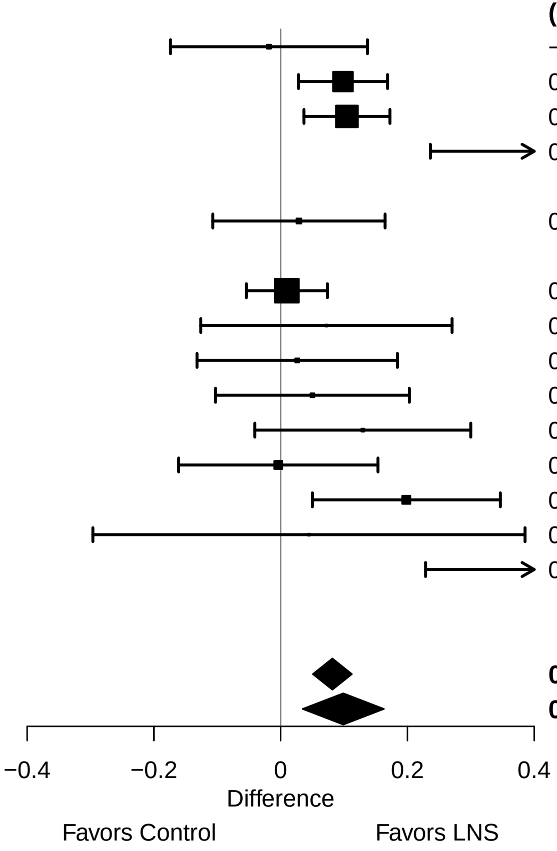

## Supplemental figure 3H: Motor lowest decile prevalence ratio

| Country | Trial | Tool | LNS N | Control N | PR (95% CI) | Fixed W | Random W |
|---|---|---|---|---|---|---|---|
| Bangladesh | JiVitA−4 (21) | BSID−III | 445 | 143 | 1.26 (0.77, 2.07) | 0.03 | 0.05 |
| Bangladesh | RDNS (22) | DMC | 1556 | 753 | 0.85 (0.67, 1.07) | 0.15 | 0.13 |
| Bangladesh | WASH−B (23) | EASQ | 1074 | 3279 | 0.82 (0.66, 1.01) | 0.19 | 0.14 |
| Burkina Faso | iLiNS−Zinc (24) | DMC | 746 | 375 | 0.49 (0.36, 0.67) | 0.09 | 0.10 |
| Ghana | GHANA (25) | | | | | | |
| Ghana | iLiNS−DYAD−G (26) | KDI | 302 | 601 | 1.13 (0.76, 1.70) | 0.05 | 0.07 |
| Haiti | HAITI (27) | | | | | | |
| Kenya | WASH−B (28) | EASQ | 1362 | 4745 | 0.86 (0.70, 1.05) | 0.21 | 0.14 |
| Madagascar | MAHAY (29) | ASQI | 1613 | 1604 | 0.91 (0.58, 1.44) | 0.04 | 0.06 |
| Malawi | iLiNS−DYAD−M (30) | KDI | 214 | 436 | 0.84 (0.51, 1.40) | 0.03 | 0.05 |
| Malawi | iLiNS−DOSE (31) | KDI | 646 | 221 | 1.08 (0.67, 1.71) | 0.04 | 0.06 |
| Mali | PROMIS CS (32) | DMC | 902 | 921 | 0.81 (0.56, 1.18) | 0.06 | 0.08 |
| Zimbabwe | SHINE (HIV−) (33), LNS vs SOC | MDAT | 395 | 382 | 1.08 (0.67, 1.73) | 0.09 | 0.10 |
| Zimbabwe | LNS+WSH vs WSH | MDAT | 446 | 417 | 0.82 (0.54, 1.24) | 0.09 | 0.10 |
| Zimbabwe | SHINE (HIV+) (34), LNS vs SOC | MDAT | 67 | 68 | 1.16 (0.44, 3.08) | 0.02 | 0.03 |
| Zimbabwe | LNS+WSH vs WSH | MDAT | 103 | 83 | 0.40 (0.17, 0.95) | 0.02 | 0.03 |
| | **I² = 0.40, Tau² = 0.02** | | **9871** | **14028** | | | |
| | **Fixed** | | | | **0.84 (0.76, 0.92)** | | |
| | **Random** | | | | **0.85 (0.74, 0.97)** | | |

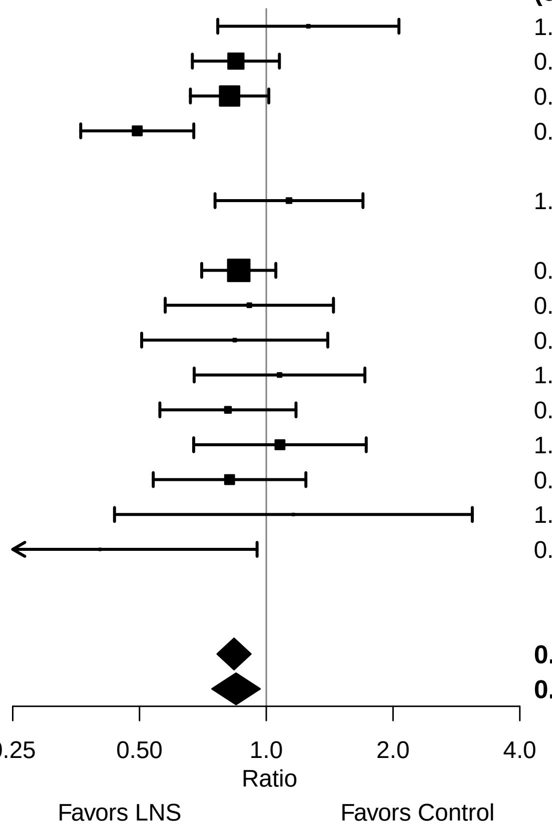

## Supplemental figure 3I: Motor lowest decile prevalence difference

| Country | Trial | Tool | LNS N | Control N | PD (95% CI) | Fixed W | Random W |
|---|---|---|---|---|---|---|---|
| Bangladesh | JiVitA−4 (21) | BSID−III | 445 | 143 | 0.02 (−0.03, 0.08) | 0.02 | 0.04 |
| Bangladesh | RDNS (22) | DMC | 1556 | 753 | −0.02 (−0.04, 0.01) | 0.09 | 0.10 |
| Bangladesh | WASH−B (23) | EASQ | 1074 | 3279 | −0.02 (−0.04, 0.00) | 0.17 | 0.14 |
| Burkina Faso | iLiNS−Zinc (24) | DMC | 746 | 375 | −0.08 (−0.12, −0.04) | 0.04 | 0.06 |
| Ghana | GHANA (25) | | | | | | |
| Ghana | iLiNS−DYAD−G (26) | KDI | 302 | 601 | 0.01 (−0.03, 0.06) | 0.04 | 0.05 |
| Haiti | HAITI (27) | | | | | | |
| Kenya | WASH−B (28) | EASQ | 1362 | 4745 | −0.01 (−0.03, 0.00) | 0.22 | 0.16 |
| Madagascar | MAHAY (29) | ASQI | 1613 | 1604 | −0.01 (−0.03, 0.01) | 0.16 | 0.14 |
| Malawi | iLiNS−DYAD−M (30) | KDI | 214 | 436 | −0.02 (−0.06, 0.03) | 0.03 | 0.04 |
| Malawi | iLiNS−DOSE (31) | KDI | 646 | 221 | 0.01 (−0.04, 0.05) | 0.03 | 0.05 |
| Mali | PROMIS CS (32) | DMC | 902 | 921 | −0.02 (−0.05, 0.01) | 0.09 | 0.10 |
| Zimbabwe | SHINE (HIV−) (33), LNS vs SOC | MDAT | 395 | 382 | 0.01 (−0.03, 0.05) | 0.08 | 0.09 |
| Zimbabwe | LNS+WSH vs WSH | MDAT | 446 | 417 | −0.02 (−0.06, 0.02) | 0.08 | 0.09 |
| Zimbabwe | SHINE (HIV+) (34), LNS vs SOC | MDAT | 67 | 68 | 0.02 (−0.09, 0.12) | 0.02 | 0.02 |
| Zimbabwe | LNS+WSH vs WSH | MDAT | 103 | 83 | −0.09 (−0.17, 0.00) | 0.02 | 0.02 |
| | **I² = 0.25, Tau² = 0.00** | | **9871** | **14028** | | | |
| | **Fixed** | | | | **−0.02 (−0.02, −0.01)** | | |
| | **Random** | | | | **−0.02 (−0.03, 0.00)** | | |

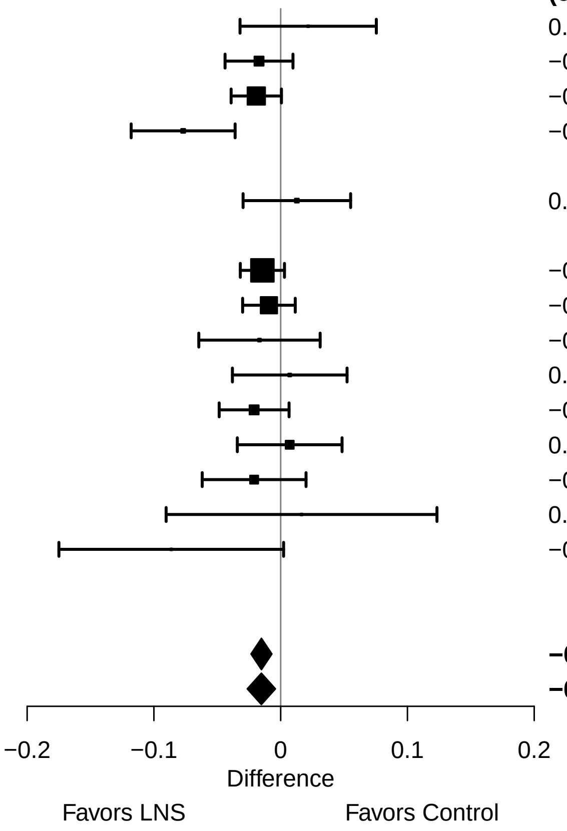

# Supplemental figure 3J: Mean difference in gross motor z-score

| Country | Trial | Tool | LNS N | Control N | MD (95% CI) | Fixed W | Random W |
|---|---|---|---|---|---|---|---|
| Bangladesh | JiVitA−4 (21) | BSID−III | 445 | 143 | −0.10 (−0.25, 0.05) | 0.04 | 0.07 |
| Bangladesh | RDNS (22) | DMC | 1570 | 758 | 0.08 (0.02, 0.15) | 0.22 | 0.13 |
| Bangladesh | WASH−B (23) | EASQ | 1074 | 3279 | 0.10 (0.04, 0.17) | 0.18 | 0.12 |
| Burkina Faso | iLiNS−Zinc (24) | DMC | | | | | |
| Ghana | GHANA (25) | | | | | | |
| Ghana | iLiNS−DYAD−G (26) | KDI | 302 | 601 | 0.01 (−0.13, 0.15) | 0.04 | 0.08 |
| Haiti | HAITI (27) | | | | | | |
| Kenya | WASH−B (28) | EASQ | 1362 | 4745 | 0.01 (−0.05, 0.07) | 0.20 | 0.13 |
| Madagascar | MAHAY (29) | ASQI | 1613 | 1604 | 0.08 (−0.08, 0.23) | 0.04 | 0.07 |
| Malawi | iLiNS−DYAD−M (30) | KDI | 214 | 436 | −0.03 (−0.19, 0.13) | 0.03 | 0.07 |
| Malawi | iLiNS−DOSE (31) | KDI | 646 | 221 | 0.00 (−0.15, 0.16) | 0.04 | 0.07 |
| Mali | PROMIS CS (32) | DMC | 940 | 957 | 0.09 (0.00, 0.17) | 0.13 | 0.11 |
| Zimbabwe | SHINE (HIV−) (33), LNS vs SOC | MDAT | 395 | 382 | 0.03 (−0.13, 0.18) | 0.07 | 0.09 |
| Zimbabwe | LNS+WSH vs WSH | MDAT | 446 | 417 | 0.16 (0.00, 0.32) | 0.07 | 0.09 |
| Zimbabwe | SHINE (HIV+) (34), LNS vs SOC | MDAT | 67 | 68 | 0.14 (−0.14, 0.42) | 0.02 | 0.06 |
| Zimbabwe | LNS+WSH vs WSH | MDAT | 103 | 83 | 0.48 (0.24, 0.72) | 0.02 | 0.06 |
| | **I² = 0.52, Tau² = 0.01** | | **9177** | **13694** | | | |
| | **Fixed** | | | | **0.06 (0.03, 0.09)** | | |
| | **Random** | | | | **0.06 (0.00, 0.12)** | | |

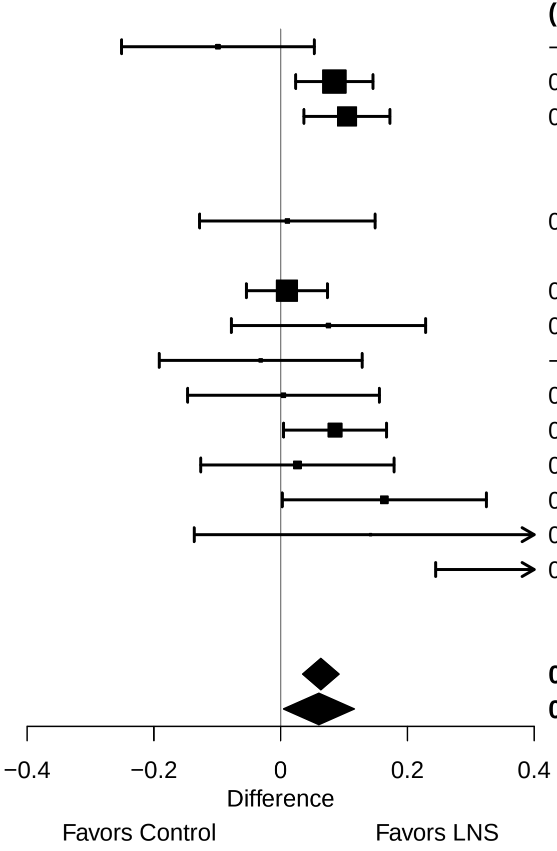

## Supplemental figure 3K: Mean difference in fine motor z-score

| Country | Trial | Tool | LNS N | Control N | MD (95% CI) | Fixed W | Random W |
|---|---|---|---|---|---|---|---|
| Bangladesh | JiVitA−4 (21) | BSID−III | 445 | 143 | 0.14 (−0.05, 0.34) | 0.06 | 0.06 |
| Bangladesh | RDNS (22) | DMC | 1641 | 804 | 0.10 (−0.01, 0.20) | 0.21 | 0.21 |
| Bangladesh | WASH−B (23) | EASQ | | | | | |
| Burkina Faso | iLiNS−Zinc (24) | DMC | | | | | |
| Ghana | GHANA (25) | | | | | | |
| Ghana | iLiNS−DYAD−G (26) | KDI | 302 | 601 | 0.04 (−0.09, 0.17) | 0.14 | 0.14 |
| Haiti | HAITI (27) | | | | | | |
| Kenya | WASH−B (28) | EASQ | | | | | |
| Madagascar | MAHAY (29) | ASQI | 1613 | 1604 | 0.05 (−0.17, 0.26) | 0.05 | 0.05 |
| Malawi | iLiNS−DYAD−M (30) | KDI | 214 | 436 | 0.08 (−0.08, 0.24) | 0.10 | 0.10 |
| Malawi | iLiNS−DOSE (31) | KDI | 646 | 221 | 0.09 (−0.06, 0.24) | 0.10 | 0.10 |
| Mali | PROMIS CS (32) | DMC | 905 | 924 | 0.11 (−0.11, 0.32) | 0.05 | 0.05 |
| Zimbabwe | SHINE (HIV−) (33), LNS vs SOC | MDAT | 395 | 382 | −0.03 (−0.18, 0.11) | 0.23 | 0.23 |
| Zimbabwe | LNS+WSH vs WSH | MDAT | 446 | 417 | 0.17 (0.03, 0.31) | 0.23 | 0.23 |
| Zimbabwe | SHINE (HIV+) (34), LNS vs SOC | MDAT | 67 | 68 | −0.04 (−0.40, 0.31) | 0.05 | 0.05 |
| Zimbabwe | LNS+WSH vs WSH | MDAT | 103 | 83 | 0.31 (0.03, 0.59) | 0.05 | 0.05 |
| | **I² = 0.00, Tau² = 0.00** | | **6777** | **5683** | | | |
| | **Fixed** | | | | **0.09 (0.04, 0.13)** | | |
| | **Random** | | | | **0.09 (0.04, 0.13)** | | |

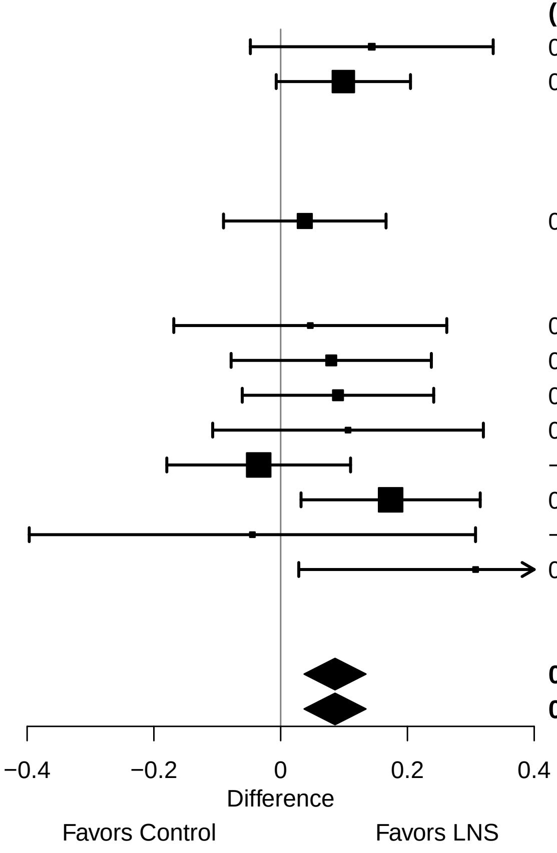

## Supplemental figure 3L: Mean difference in executive function z-score

| Country | Trial | Tool | LNS N | Control N | MD (95% CI) | Fixed W | Random W |
|---|---|---|---|---|---|---|---|
| Bangladesh | JiVitA−4 (21) | | | | | | |
| Bangladesh | RDNS (22) | A not B task | 533 | 266 | 0.10 (−0.02, 0.22) | 0.14 | 0.15 |
| Bangladesh | WASH−B (23) | A not B task | 1097 | 3327 | 0.00 (−0.07, 0.07) | 0.43 | 0.40 |
| Burkina Faso | iLiNS−Zinc (24) | | | | | | |
| Ghana | GHANA (25) | | | | | | |
| Ghana | iLiNS−DYAD−G (26) | A not B task | 290 | 576 | 0.02 (−0.12, 0.17) | 0.10 | 0.11 |
| Haiti | HAITI (27) | | | | | | |
| Kenya | WASH−B (28) | | | | | | |
| Madagascar | MAHAY (29) | | | | | | |
| Malawi | iLiNS−DYAD−M (30) | A not B task | 182 | 369 | −0.09 (−0.27, 0.09) | 0.07 | 0.07 |
| Malawi | iLiNS−DOSE (31) | A not B task | 475 | 164 | −0.11 (−0.29, 0.07) | 0.07 | 0.07 |
| Mali | PROMIS CS (32) | | | | | | |
| Zimbabwe | SHINE (HIV−) (33), LNS vs SOC | A not B task | 370 | 357 | −0.05 (−0.21, 0.10) | 0.15 | 0.16 |
| Zimbabwe | LNS+WSH vs WSH | A not B task | 417 | 382 | 0.12 (−0.05, 0.29) | 0.15 | 0.16 |
| Zimbabwe | SHINE (HIV+) (34), LNS vs SOC | A not B task | 63 | 57 | −0.16 (−0.49, 0.16) | 0.04 | 0.05 |
| Zimbabwe | LNS+WSH vs WSH | A not B task | 94 | 76 | −0.05 (−0.35, 0.25) | 0.04 | 0.05 |
| | **$I^2$ = 0.04, $Tau^2$ = 0.00** | | **3521** | **5574** | | | |
| | **Fixed** | | | | **0.00 (−0.04, 0.05)** | | |
| | **Random** | | | | **0.00 (−0.04, 0.05)** | | |

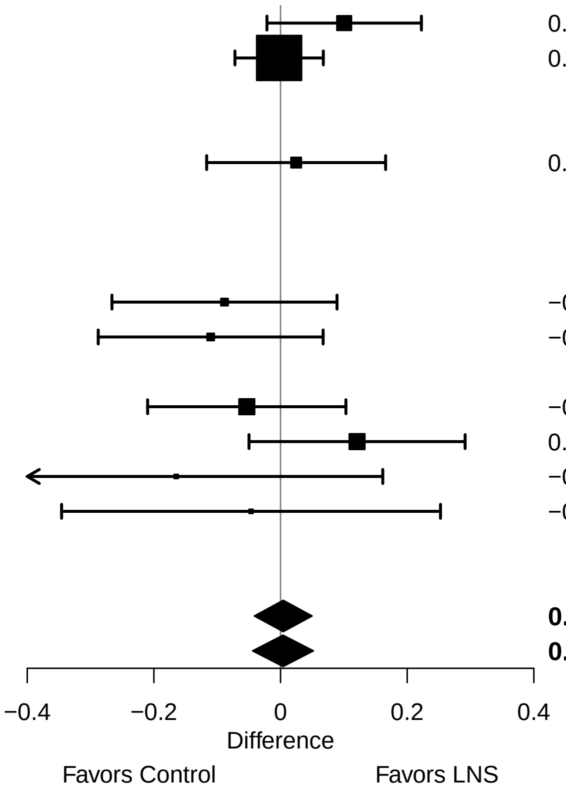

# Supplemental figure 3M: Executive function lowest decile prevalence ratio

| Country | Trial | Tool | LNS N | Control N | PR (95% CI) | Fixed W | Random W |
|---|---|---|---|---|---|---|---|
| Bangladesh | JiVitA−4 (21) | | | | | | |
| Bangladesh | RDNS (22) | A not B task | 533 | 266 | 0.88 (0.60, 1.29) | 0.13 | 0.15 |
| Bangladesh | WASH−B (23) | A not B task | 1097 | 3327 | 0.95 (0.77, 1.18) | 0.41 | 0.31 |
| Burkina Faso | iLiNS−Zinc (24) | | | | | | |
| Ghana | GHANA (25) | | | | | | |
| Ghana | iLiNS−DYAD−G (26) | A not B task | 290 | 576 | 0.78 (0.50, 1.20) | 0.10 | 0.12 |
| Haiti | HAITI (27) | | | | | | |
| Kenya | WASH−B (28) | | | | | | |
| Madagascar | MAHAY (29) | | | | | | |
| Malawi | iLiNS−DYAD−M (30) | A not B task | 182 | 369 | 0.81 (0.47, 1.41) | 0.06 | 0.08 |
| Malawi | iLiNS−DOSE (31) | A not B task | 475 | 164 | 1.50 (0.82, 2.73) | 0.05 | 0.07 |
| Mali | PROMIS CS (32) | | | | | | |
| Zimbabwe | SHINE (HIV−) (33), LNS vs SOC | A not B task | 370 | 357 | 0.82 (0.53, 1.26) | 0.20 | 0.20 |
| Zimbabwe | LNS+WSH vs WSH | A not B task | 417 | 382 | 0.78 (0.50, 1.21) | 0.20 | 0.20 |
| Zimbabwe | SHINE (HIV+) (34), LNS vs SOC | A not B task | 63 | 57 | 1.09 (0.37, 3.22) | 0.05 | 0.06 |
| Zimbabwe | LNS+WSH vs WSH | A not B task | 94 | 76 | 2.10 (0.92, 4.82) | 0.05 | 0.06 |
| | **$I^2$ = 0.16, $Tau^2$ = 0.01** | | **3521** | **5574** | | | |
| | **Fixed** | | | | **0.93 (0.81, 1.06)** | | |
| | **Random** | | | | **0.93 (0.78, 1.11)** | | |

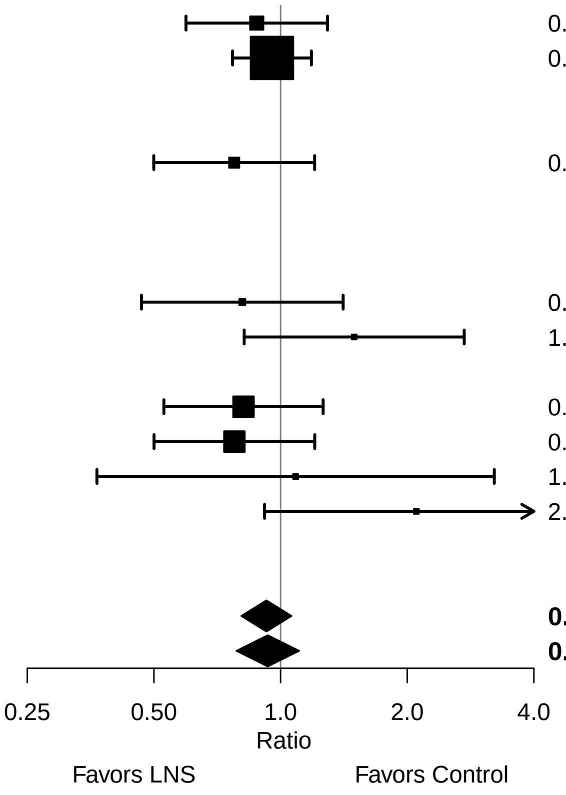

# Supplemental figure 3N: Executive function lowest decile prevalence difference

| Country | Trial | Tool | LNS N | Control N | PD (95% CI) | Fixed W | Random W |
|---|---|---|---|---|---|---|---|
| Bangladesh | JiVitA−4 (21) | | | | | | |
| Bangladesh | RDNS (22) | A not B task | 533 | 266 | −0.01 (−0.06, 0.03) | 0.09 | 0.12 |
| Bangladesh | WASH−B (23) | A not B task | 1097 | 3327 | 0.00 (−0.02, 0.02) | 0.44 | 0.30 |
| Burkina Faso | iLiNS−Zinc (24) | | | | | | |
| Ghana | GHANA (25) | | | | | | |
| Ghana | iLiNS−DYAD−G (26) | A not B task | 290 | 576 | −0.02 (−0.07, 0.02) | 0.10 | 0.13 |
| Haiti | HAITI (27) | | | | | | |
| Kenya | WASH−B (28) | | | | | | |
| Madagascar | MAHAY (29) | | | | | | |
| Malawi | iLiNS−DYAD−M (30) | A not B task | 182 | 369 | −0.02 (−0.07, 0.03) | 0.07 | 0.09 |
| Malawi | iLiNS−DOSE (31) | A not B task | 475 | 164 | 0.04 (−0.01, 0.09) | 0.08 | 0.10 |
| Mali | PROMIS CS (32) | | | | | | |
| Zimbabwe | SHINE (HIV−) (33), LNS vs SOC | A not B task | 370 | 357 | −0.02 (−0.06, 0.02) | 0.19 | 0.20 |
| Zimbabwe | LNS+WSH vs WSH | A not B task | 417 | 382 | −0.03 (−0.07, 0.02) | 0.19 | 0.20 |
| Zimbabwe | SHINE (HIV+) (34), LNS vs SOC | A not B task | 63 | 57 | 0.01 (−0.10, 0.11) | 0.04 | 0.06 |
| Zimbabwe | LNS+WSH vs WSH | A not B task | 94 | 76 | 0.07 (−0.02, 0.16) | 0.04 | 0.06 |
| | **I² = 0.21, Tau² = 0.00** | | **3521** | **5574** | | | |
| | **Fixed** | | | | **−0.01 (−0.02, 0.01)** | | |
| | **Random** | | | | **−0.01 (−0.02, 0.01)** | | |

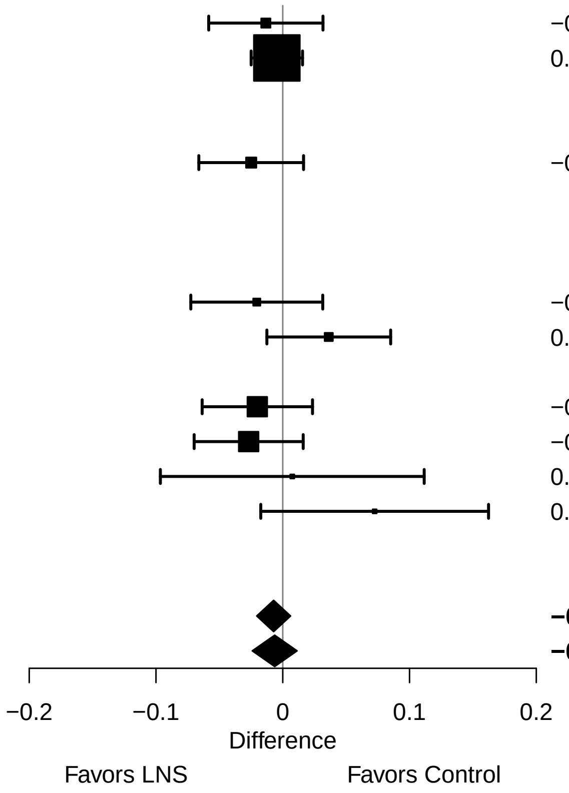

## Supplemental figure 3O: 12-mo walking without support prevalence ratio

| Country | Trial | LNS N | Control N | PR (95% CI) | Fixed W | Random W |
|---|---|---|---|---|---|---|
| Bangladesh | JiVitA−4 (21) | 3014 | 1363 | 1.05 (0.99, 1.12) | 0.46 | 0.16 |
| Bangladesh | RDNS (22) | 1628 | 790 | 1.28 (1.07, 1.53) | 0.06 | 0.11 |
| Bangladesh | WASH−B (23) | 497 | 1474 | 1.20 (1.05, 1.38) | 0.10 | 0.13 |
| Burkina Faso | iLiNS−Zinc (24) | | | | | |
| Ghana | GHANA (25) | 90 | 87 | 1.67 (1.08, 2.58) | 0.01 | 0.04 |
| Ghana | iLiNS−DYAD−G (26) | 327 | 663 | 1.15 (1.01, 1.31) | 0.11 | 0.13 |
| Haiti | HAITI (27) | 83 | 79 | 1.25 (0.86, 1.81) | 0.01 | 0.05 |
| Kenya | WASH−B (28) | 447 | 1604 | 0.97 (0.85, 1.10) | 0.12 | 0.13 |
| Madagascar | MAHAY (29) | | | | | |
| Malawi | iLiNS−DYAD−M (30) | 208 | 426 | 1.20 (1.03, 1.40) | 0.08 | 0.12 |
| Malawi | iLiNS−DOSE (31) | 602 | 200 | 1.04 (0.84, 1.30) | 0.04 | 0.09 |
| Mali | PROMIS CS (32) | 118 | 151 | 0.70 (0.44, 1.11) | 0.01 | 0.03 |
| Zimbabwe | SHINE (HIV−) (33) | | | | | |
| Zimbabwe | SHINE (HIV+) (34) | | | | | |
| | **$I^2$ = 0.55, $Tau^2$ = 0.01** | **7014** | **6837** | | | |
| | **Fixed** | | | **1.09 (1.05, 1.14)** | | |
| | **Random** | | | **1.12 (1.02, 1.24)** | | |

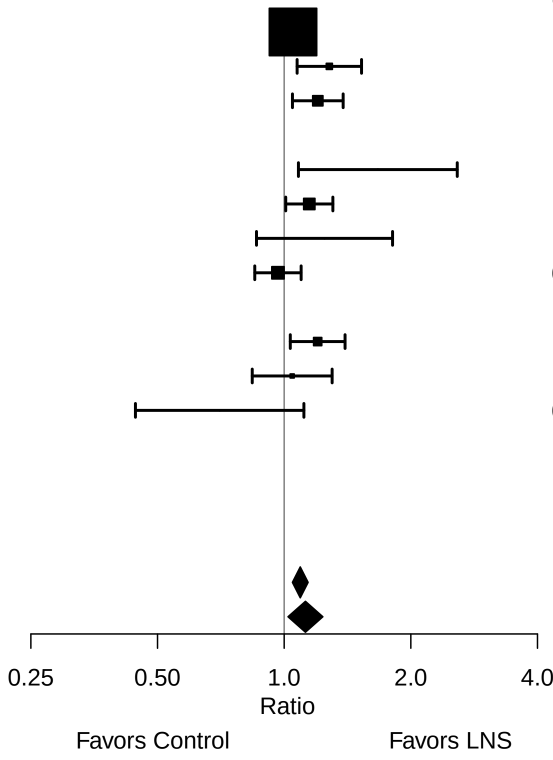

# Supplemental figure 3P: 12-mo walking without support prevalence difference

| Country | Trial | LNS N | Control N | PD (95% CI) | Fixed W | Random W |
|---|---|---|---|---|---|---|
| Bangladesh | JiVitA−4 (21) | 3014 | 1363 | 0.03 (−0.01, 0.06) | 0.30 | 0.14 |
| Bangladesh | RDNS (22) | 1628 | 790 | 0.07 (0.03, 0.11) | 0.21 | 0.14 |
| Bangladesh | WASH−B (23) | 497 | 1474 | 0.05 (0.01, 0.10) | 0.14 | 0.13 |
| Burkina Faso | iLiNS−Zinc (24) | | | | | |
| Ghana | GHANA (25) | 90 | 87 | 0.17 (0.03, 0.31) | 0.02 | 0.05 |
| Ghana | iLiNS−DYAD−G (26) | 327 | 663 | 0.07 (0.00, 0.14) | 0.07 | 0.11 |
| Haiti | HAITI (27) | 83 | 79 | 0.09 (−0.06, 0.24) | 0.01 | 0.04 |
| Kenya | WASH−B (28) | 447 | 1604 | −0.01 (−0.06, 0.04) | 0.12 | 0.12 |
| Madagascar | MAHAY (29) | | | | | |
| Malawi | iLiNS−DYAD−M (30) | 208 | 426 | 0.10 (0.02, 0.18) | 0.05 | 0.09 |
| Malawi | iLiNS−DOSE (31) | 602 | 200 | 0.02 (−0.06, 0.09) | 0.05 | 0.10 |
| Mali | PROMIS CS (32) | 118 | 151 | −0.10 (−0.21, 0.01) | 0.03 | 0.07 |
| Zimbabwe | SHINE (HIV−) (33) | | | | | |
| Zimbabwe | SHINE (HIV+) (34) | | | | | |
| | **I² = 0.57, Tau² = 0.00** | **7014** | **6837** | | | |
| | **Fixed** | | | **0.04 (0.02, 0.06)** | | |
| | **Random** | | | **0.04 (0.00, 0.08)** | | |

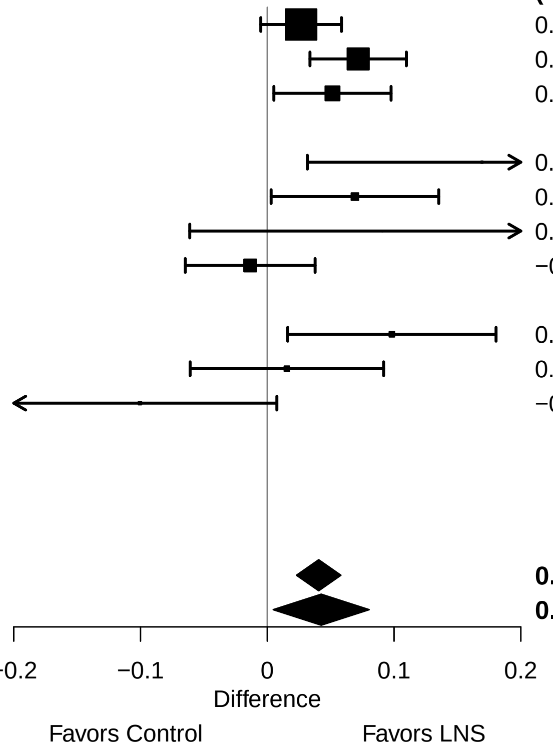

# Supplemental figure 3Q: 12-mo walking with support prevalence ratio

| Country | Trial | LNS N | Control N | PR (95% CI) | Fixed W | Random W |
|---|---|---|---|---|---|---|
| Bangladesh | JiVitA−4 (21) | 3131 | 1418 | 1.00 (1.00, 1.01) | 0.85 | 0.28 |
| Bangladesh | RDNS (22) | 1546 | 746 | 1.06 (1.01, 1.11) | 0.01 | 0.10 |
| Bangladesh | WASH−B (23) | 496 | 1470 | 0.99 (0.94, 1.05) | 0.01 | 0.08 |
| Burkina Faso | iLiNS−Zinc (24) | | | | | |
| Ghana | GHANA (25) | 90 | 87 | 1.16 (0.97, 1.39) | 0.00 | 0.01 |
| Ghana | iLiNS−DYAD−G (26) | 327 | 662 | 1.00 (0.99, 1.01) | 0.10 | 0.24 |
| Haiti | HAITI (27) | | | | | |
| Kenya | WASH−B (28) | 447 | 1604 | 1.03 (1.00, 1.06) | 0.02 | 0.16 |
| Madagascar | MAHAY (29) | | | | | |
| Malawi | iLiNS−DYAD−M (30) | 208 | 426 | 1.02 (0.97, 1.07) | 0.01 | 0.08 |
| Malawi | iLiNS−DOSE (31) | 602 | 200 | 1.05 (0.95, 1.16) | 0.00 | 0.03 |
| Mali | PROMIS CS (32) | 118 | 151 | 0.93 (0.76, 1.13) | 0.00 | 0.01 |
| Zimbabwe | SHINE (HIV−) (33) | | | | | |
| Zimbabwe | SHINE (HIV+) (34) | | | | | |
| | **I² = 0.41, Tau² = 0.00** | **6965** | **6764** | | | |
| | **Fixed** | | | **1.00 (1.00, 1.01)** | | |
| | **Random** | | | **1.01 (1.00, 1.03)** | | |

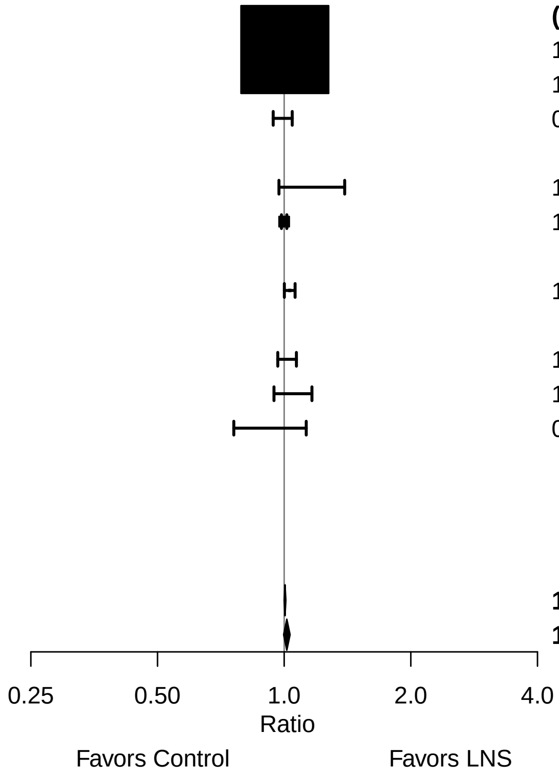

# Supplemental figure 3R: 12-mo walking with support prevalence difference

| Country | Trial | LNS N | Control N | PD (95% CI) | Fixed W | Random W |
|---|---|---|---|---|---|---|
| Bangladesh | JiVitA−4 (21) | 3131 | 1418 | 0.00 (0.00, 0.01) | 0.84 | 0.26 |
| Bangladesh | RDNS (22) | 1546 | 746 | 0.05 (0.01, 0.08) | 0.02 | 0.12 |
| Bangladesh | WASH−B (23) | 496 | 1470 | −0.01 (−0.05, 0.03) | 0.01 | 0.10 |
| Burkina Faso | iLiNS−Zinc (24) | | | | | |
| Ghana | GHANA (25) | 90 | 87 | 0.11 (−0.02, 0.24) | 0.00 | 0.01 |
| Ghana | iLiNS−DYAD−G (26) | 327 | 662 | 0.00 (−0.01, 0.01) | 0.09 | 0.22 |
| Haiti | HAITI (27) | | | | | |
| Kenya | WASH−B (28) | 447 | 1604 | 0.03 (0.00, 0.05) | 0.03 | 0.15 |
| Madagascar | MAHAY (29) | | | | | |
| Malawi | iLiNS−DYAD−M (30) | 208 | 426 | 0.01 (−0.03, 0.06) | 0.01 | 0.08 |
| Malawi | iLiNS−DOSE (31) | 602 | 200 | 0.03 (−0.04, 0.11) | 0.00 | 0.04 |
| Mali | PROMIS CS (32) | 118 | 151 | −0.06 (−0.16, 0.05) | 0.00 | 0.02 |
| Zimbabwe | SHINE (HIV−) (33) | | | | | |
| Zimbabwe | SHINE (HIV+) (34) | | | | | |
| | **I² = 0.42, Tau² = 0.00** | **6965** | **6764** | | | |
| | **Fixed** | | | **0.00 (0.00, 0.01)** | | |
| | **Random** | | | **0.01 (0.00, 0.03)** | | |

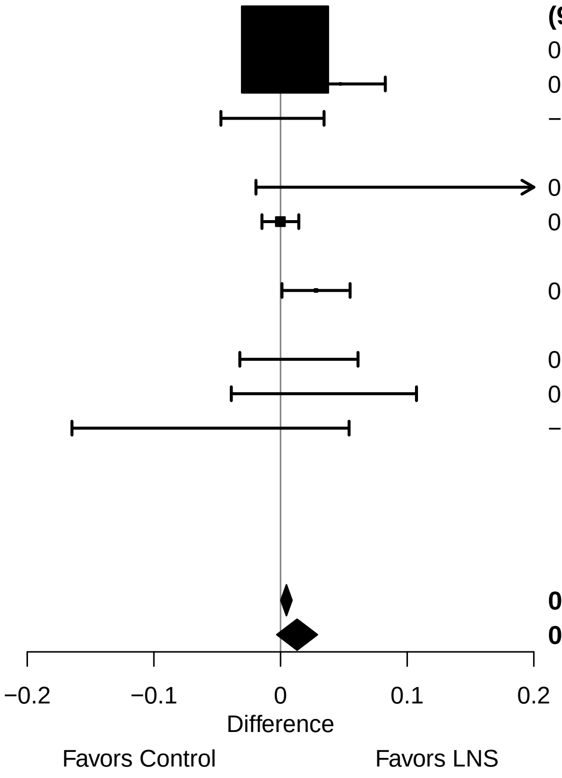

## Supplemental figure 3S: 12-mo standing without support prevalence ratio

| Country | Trial | LNS N | Control N | PR (95% CI) | Fixed W | Random W |
|---|---|---|---|---|---|---|
| Bangladesh | JiVitA−4 (21) | 3067 | 1384 | 1.00 (0.97, 1.03) | 0.48 | 0.18 |
| Bangladesh | RDNS (22) | 1550 | 767 | 1.22 (1.13, 1.33) | 0.07 | 0.12 |
| Bangladesh | WASH−B (23) | 499 | 1474 | 1.07 (0.96, 1.18) | 0.04 | 0.09 |
| Burkina Faso | iLiNS−Zinc (24) | | | | | |
| Ghana | GHANA (25) | 90 | 87 | 1.17 (0.89, 1.54) | 0.01 | 0.02 |
| Ghana | iLiNS−DYAD−G (26) | 323 | 654 | 0.99 (0.92, 1.07) | 0.08 | 0.12 |
| Haiti | HAITI (27) | 132 | 108 | 1.01 (0.83, 1.23) | 0.01 | 0.04 |
| Kenya | WASH−B (28) | 447 | 1604 | 1.00 (0.95, 1.06) | 0.14 | 0.15 |
| Madagascar | MAHAY (29) | | | | | |
| Malawi | iLiNS−DYAD−M (30) | 208 | 426 | 1.06 (0.99, 1.13) | 0.10 | 0.13 |
| Malawi | iLiNS−DOSE (31) | 602 | 200 | 1.04 (0.95, 1.14) | 0.06 | 0.11 |
| Mali | PROMIS CS (32) | 118 | 151 | 0.95 (0.78, 1.16) | 0.01 | 0.04 |
| Zimbabwe | SHINE (HIV−) (33) | | | | | |
| Zimbabwe | SHINE (HIV+) (34) | | | | | |
| | **$I^2$ = 0.65, $Tau^2$ = 0.00** | **7036** | **6855** | | | |
| | **Fixed** | | | **1.03 (1.00, 1.05)** | | |
| | **Random** | | | **1.04 (1.00, 1.09)** | | |

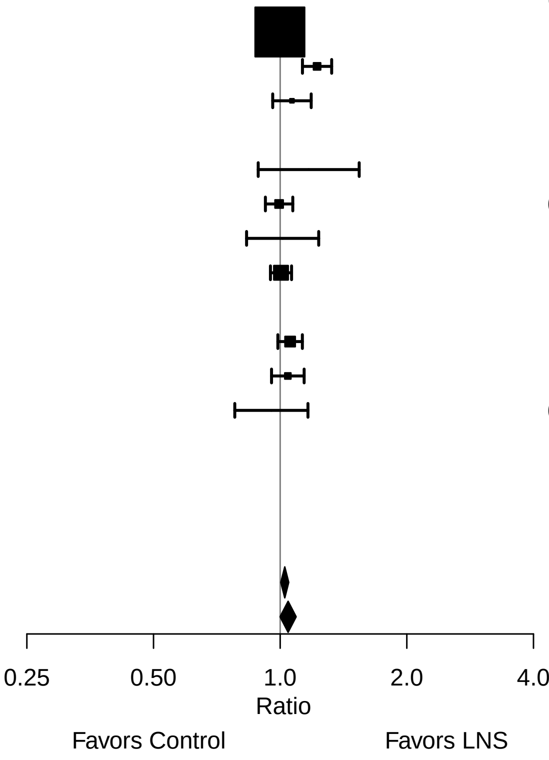

# Supplemental figure 3T: 12-mo standing without support prevalence difference

| Country | Trial | LNS N | Control N | PD (95% CI) | Fixed W | Random W |
|---|---|---|---|---|---|---|
| Bangladesh | JiVitA−4 (21) | 3067 | 1384 | 0.00 (−0.03, 0.02) | 0.40 | 0.21 |
| Bangladesh | RDNS (22) | 1550 | 767 | 0.10 (0.06, 0.15) | 0.13 | 0.14 |
| Bangladesh | WASH−B (23) | 499 | 1474 | 0.03 (−0.02, 0.08) | 0.10 | 0.12 |
| Burkina Faso | iLiNS−Zinc (24) | | | | | |
| Ghana | GHANA (25) | 90 | 87 | 0.08 (−0.06, 0.23) | 0.01 | 0.02 |
| Ghana | iLiNS−DYAD−G (26) | 323 | 654 | 0.00 (−0.06, 0.05) | 0.08 | 0.11 |
| Haiti | HAITI (27) | 132 | 108 | 0.01 (−0.12, 0.13) | 0.02 | 0.03 |
| Kenya | WASH−B (28) | 447 | 1604 | 0.00 (−0.04, 0.05) | 0.12 | 0.13 |
| Madagascar | MAHAY (29) | | | | | |
| Malawi | iLiNS−DYAD−M (30) | 208 | 426 | 0.05 (−0.01, 0.10) | 0.08 | 0.11 |
| Malawi | iLiNS−DOSE (31) | 602 | 200 | 0.03 (−0.04, 0.10) | 0.05 | 0.09 |
| Mali | PROMIS CS (32) | 118 | 151 | −0.03 (−0.15, 0.09) | 0.02 | 0.04 |
| Zimbabwe | SHINE (HIV−) (33) | | | | | |
| Zimbabwe | SHINE (HIV+) (34) | | | | | |
| | **$I^2$ = 0.56, $Tau^2$ = 0.00** | **7036** | **6855** | | | |
| | **Fixed** | | | **0.02 (0.01, 0.04)** | | |
| | **Random** | | | **0.03 (0.00, 0.05)** | | |

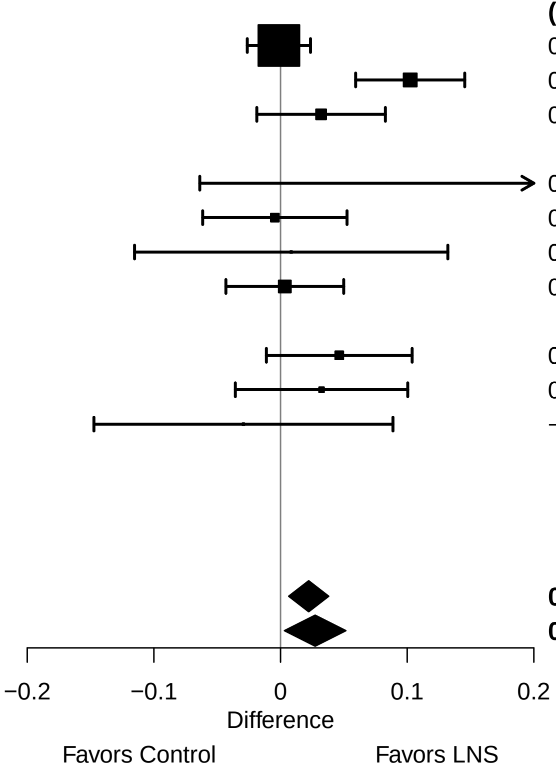

# Supplemental figure 3U: 12-mo standing with support prevalence ratio

| Country | Trial | LNS N | Control N | PR (95% CI) | Fixed W | Random W |
|---|---|---|---|---|---|---|
| Bangladesh | JiVitA−4 (21) | 3121 | 1409 | 1.00 (0.99, 1.01) | 0.16 | 0.16 |
| Bangladesh | RDNS (22) | 1624 | 783 | 1.00 (0.99, 1.02) | 0.04 | 0.04 |
| Bangladesh | WASH−B (23) | 497 | 1472 | 1.00 (0.98, 1.02) | 0.01 | 0.01 |
| Burkina Faso | iLiNS−Zinc (24) | | | | | |
| Ghana | GHANA (25) | 90 | 87 | 1.01 (0.93, 1.10) | 0.00 | 0.00 |
| Ghana | iLiNS−DYAD−G (26) | 329 | 670 | 1.00 (1.00, 1.00) | 0.77 | 0.77 |
| Haiti | HAITI (27) | | | | | |
| Kenya | WASH−B (28) | 447 | 1604 | 1.02 (0.99, 1.05) | 0.01 | 0.01 |
| Madagascar | MAHAY (29) | | | | | |
| Malawi | iLiNS−DYAD−M (30) | 208 | 426 | 1.01 (0.98, 1.04) | 0.01 | 0.01 |
| Malawi | iLiNS−DOSE (31) | 602 | 200 | 1.02 (0.99, 1.05) | 0.01 | 0.01 |
| Mali | PROMIS CS (32) | 118 | 151 | 1.03 (0.94, 1.12) | 0.00 | 0.00 |
| Zimbabwe | SHINE (HIV−) (33) | | | | | |
| Zimbabwe | SHINE (HIV+) (34) | | | | | |
| | **I² = 0.00, Tau² = 0.00** | **7036** | **6802** | | | |
| | **Fixed** | | | **1.00 (1.00, 1.00)** | | |
| | **Random** | | | **1.00 (1.00, 1.00)** | | |

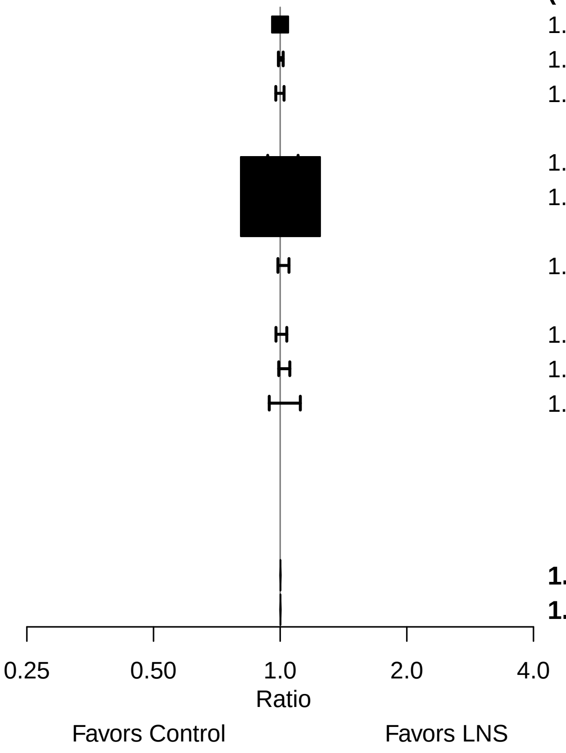

# Supplemental figure 3V: 12-mo standing with support prevalence difference

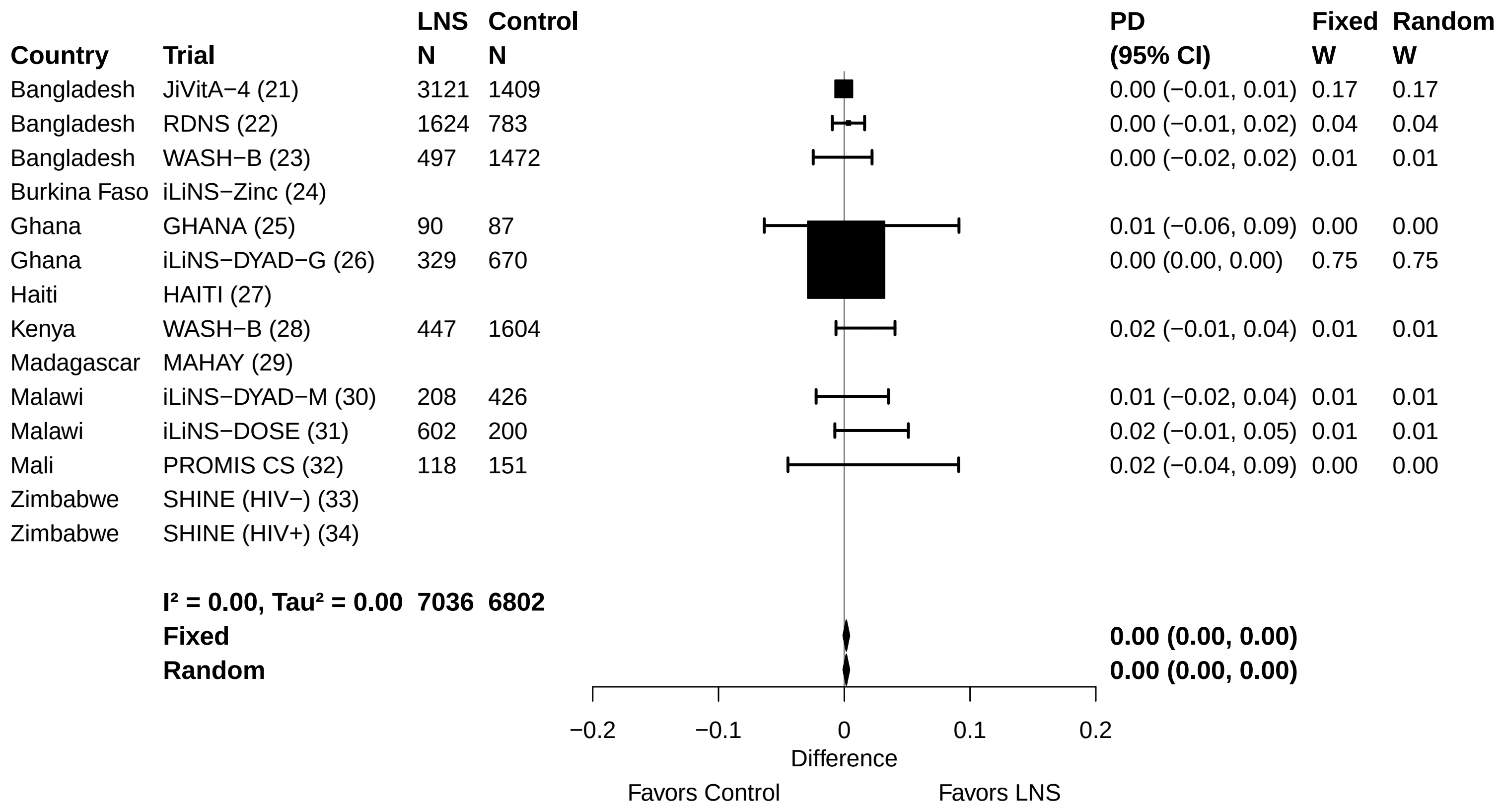

# Supplemental figure 3W: 12-mo crawling prevalence ratio

| Country | Trial | LNS N | Control N | PR (95% CI) | Fixed W | Random W |
|---|---|---|---|---|---|---|
| Bangladesh | JiVitA−4 (21) | 2992 | 1369 | 1.00 (1.00, 1.01) | 0.73 | 0.30 |
| Bangladesh | RDNS (22) | 1494 | 742 | 1.08 (1.02, 1.14) | 0.01 | 0.04 |
| Bangladesh | WASH−B (23) | 500 | 1480 | 1.03 (0.97, 1.08) | 0.01 | 0.04 |
| Burkina Faso | iLiNS−Zinc (24) | | | | | |
| Ghana | GHANA (25) | | | | | |
| Ghana | iLiNS−DYAD−G (26) | 328 | 665 | 1.01 (0.99, 1.02) | 0.12 | 0.21 |
| Haiti | HAITI (27) | 83 | 79 | 1.03 (0.94, 1.14) | 0.00 | 0.01 |
| Kenya | WASH−B (28) | 447 | 1604 | 1.01 (0.99, 1.02) | 0.08 | 0.18 |
| Madagascar | MAHAY (29) | | | | | |
| Malawi | iLiNS−DYAD−M (30) | 208 | 426 | 0.99 (0.97, 1.01) | 0.04 | 0.13 |
| Malawi | iLiNS−DOSE (31) | 602 | 200 | 0.99 (0.95, 1.03) | 0.02 | 0.06 |
| Mali | PROMIS CS (32) | 118 | 151 | 1.01 (0.95, 1.07) | 0.01 | 0.03 |
| Zimbabwe | SHINE (HIV−) (33) | | | | | |
| Zimbabwe | SHINE (HIV+) (34) | | | | | |
| | **$I^2$ = 0.26, $Tau^2$ = 0.00** | **6772** | **6716** | | | |
| | **Fixed** | | | **1.00 (1.00, 1.01)** | | |
| | **Random** | | | **1.01 (0.99, 1.02)** | | |

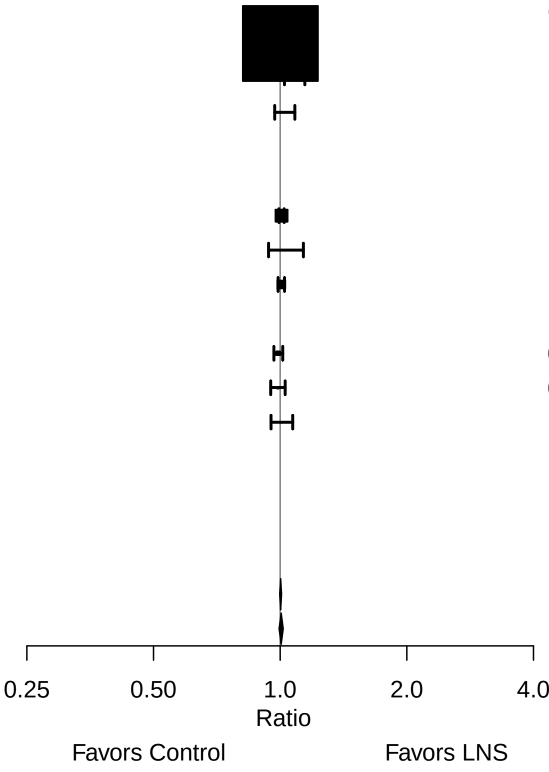

## Supplemental figure 3X: 12-mo crawling prevalence difference

| Country | Trial | LNS N | Control N | PD (95% CI) | Fixed W | Random W |
|---|---|---|---|---|---|---|
| Bangladesh | JiVitA−4 (21) | 2992 | 1369 | 0.00 (0.00, 0.01) | 0.70 | 0.26 |
| Bangladesh | RDNS (22) | 1494 | 742 | 0.06 (0.02, 0.10) | 0.01 | 0.06 |
| Bangladesh | WASH−B (23) | 500 | 1480 | 0.02 (−0.02, 0.06) | 0.01 | 0.06 |
| Burkina Faso | iLiNS−Zinc (24) | | | | | |
| Ghana | GHANA (25) | | | | | |
| Ghana | iLiNS−DYAD−G (26) | 328 | 665 | 0.01 (−0.01, 0.02) | 0.12 | 0.20 |
| Haiti | HAITI (27) | 83 | 79 | 0.03 (−0.06, 0.12) | 0.00 | 0.02 |
| Kenya | WASH−B (28) | 447 | 1604 | 0.01 (−0.01, 0.02) | 0.09 | 0.18 |
| Madagascar | MAHAY (29) | | | | | |
| Malawi | iLiNS−DYAD−M (30) | 208 | 426 | −0.01 (−0.03, 0.01) | 0.04 | 0.13 |
| Malawi | iLiNS−DOSE (31) | 602 | 200 | −0.01 (−0.05, 0.03) | 0.02 | 0.07 |
| Mali | PROMIS CS (32) | 118 | 151 | 0.01 (−0.05, 0.06) | 0.01 | 0.04 |
| Zimbabwe | SHINE (HIV−) (33) | | | | | |
| Zimbabwe | SHINE (HIV+) (34) | | | | | |
| | **$I^2$ = 0.22, $Tau^2$ = 0.00** | **6772** | **6716** | | | |
| | **Fixed** | | | **0.00 (0.00, 0.01)** | | |
| | **Random** | | | **0.01 (−0.01, 0.02)** | | |

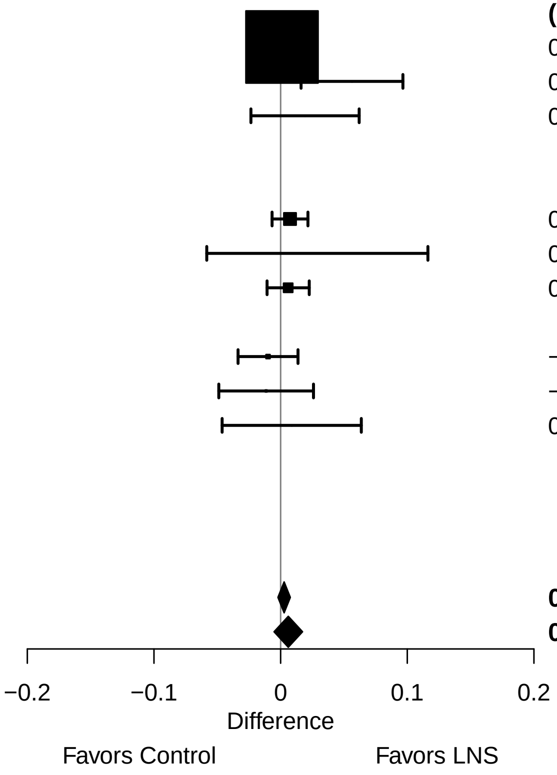

## Supplemental figure 3Y: 18-mo walking without support prevalence ratio

| Country | Trial | LNS N | Control N | PR (95% CI) | Fixed W | Random W |
|---|---|---|---|---|---|---|
| Bangladesh | JiVitA−4 (21) | | | | | |
| Bangladesh | RDNS (22) | 1654 | 816 | 1.00 (0.99, 1.02) | 0.38 | 0.32 |
| Bangladesh | WASH−B (23) | | | | | |
| Burkina Faso | iLiNS−Zinc (24) | 763 | 342 | 1.06 (1.01, 1.11) | 0.03 | 0.06 |
| Ghana | GHANA (25) | | | | | |
| Ghana | iLiNS−DYAD−G (26) | 348 | 693 | 0.99 (0.98, 1.01) | 0.38 | 0.32 |
| Haiti | HAITI (27) | 43 | 60 | 1.02 (0.98, 1.05) | 0.07 | 0.11 |
| Kenya | WASH−B (28) | | | | | |
| Madagascar | MAHAY (29) | | | | | |
| Malawi | iLiNS−DYAD−M (30) | 219 | 441 | 1.01 (0.98, 1.04) | 0.08 | 0.12 |
| Malawi | iLiNS−DOSE (31) | 619 | 222 | 1.00 (0.96, 1.04) | 0.05 | 0.07 |
| Mali | PROMIS CS (32) | 111 | 106 | 1.04 (0.93, 1.16) | 0.01 | 0.01 |
| Zimbabwe | SHINE (HIV−) (33) | | | | | |
| Zimbabwe | SHINE (HIV+) (34) | | | | | |
| | **$I^2$ = 0.28, $Tau^2$ = 0.00** | **3757** | **2680** | | | |
| | **Fixed** | | | **1.00 (0.99, 1.01)** | | |
| | **Random** | | | **1.01 (0.99, 1.02)** | | |

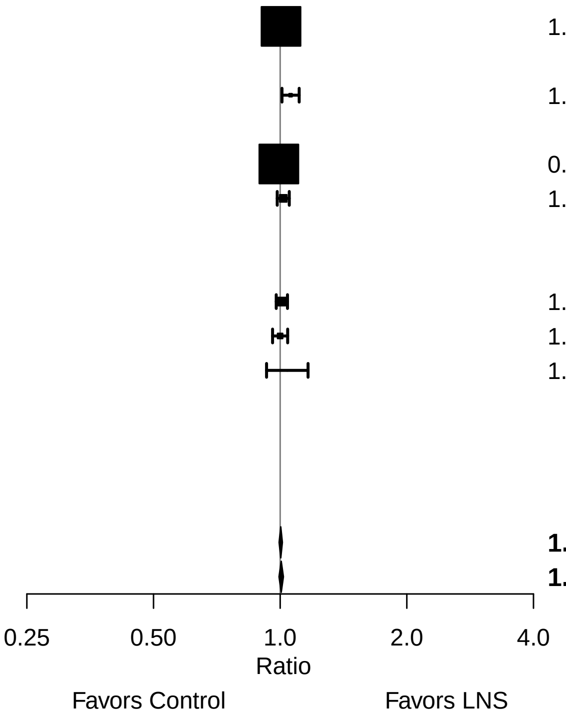

## Supplemental figure 3Z: 18-mo walking without support prevalence difference

| Country | Trial | LNS N | Control N | PD (95% CI) | Fixed W | Random W |
|---|---|---|---|---|---|---|
| Bangladesh | JiVitA−4 (21) | | | | | |
| Bangladesh | RDNS (22) | 1654 | 816 | 0.00 (−0.01, 0.02) | 0.42 | 0.29 |
| Bangladesh | WASH−B (23) | | | | | |
| Burkina Faso | iLiNS−Zinc (24) | 763 | 342 | 0.05 (0.01, 0.09) | 0.05 | 0.09 |
| Ghana | GHANA (25) | | | | | |
| Ghana | iLiNS−DYAD−G (26) | 348 | 693 | −0.01 (−0.02, 0.01) | 0.34 | 0.27 |
| Haiti | HAITI (27) | 43 | 60 | 0.02 (−0.02, 0.05) | 0.06 | 0.11 |
| Kenya | WASH−B (28) | | | | | |
| Madagascar | MAHAY (29) | | | | | |
| Malawi | iLiNS−DYAD−M (30) | 219 | 441 | 0.01 (−0.02, 0.04) | 0.08 | 0.13 |
| Malawi | iLiNS−DOSE (31) | 619 | 222 | 0.00 (−0.04, 0.04) | 0.04 | 0.09 |
| Mali | PROMIS CS (32) | 111 | 106 | 0.03 (−0.06, 0.12) | 0.01 | 0.02 |
| Zimbabwe | SHINE (HIV−) (33) | | | | | |
| Zimbabwe | SHINE (HIV+) (34) | | | | | |
| | **I² = 0.40, Tau² = 0.00** | **3757** | **2680** | | | |
| | **Fixed** | | | **0.00 (0.00, 0.01)** | | |
| | **Random** | | | **0.01 (−0.01, 0.02)** | | |

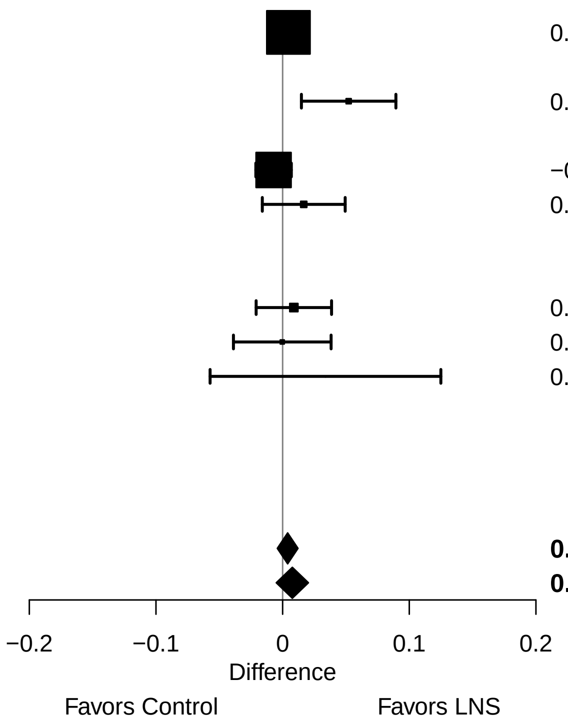

# Supplemental figure 3AA: 18-mo walking with support prevalence ratio

| Country | Trial | LNS N | Control N | PR (95% CI) | Fixed W | Random W |
|---|---|---|---|---|---|---|
| Bangladesh | JiVitA−4 (21) | | | | | |
| Bangladesh | RDNS (22) | 1654 | 816 | 1.01 (1.00, 1.02) | 0.28 | 0.28 |
| Bangladesh | WASH−B (23) | | | | | |
| Burkina Faso | iLiNS−Zinc (24) | 763 | 342 | 1.02 (1.00, 1.04) | 0.09 | 0.09 |
| Ghana | GHANA (25) | | | | | |
| Ghana | iLiNS−DYAD−G (26) | 348 | 693 | 1.00 (0.99, 1.01) | 0.41 | 0.41 |
| Haiti | HAITI (27) | 43 | 60 | 1.02 (0.98, 1.05) | 0.03 | 0.03 |
| Kenya | WASH−B (28) | | | | | |
| Madagascar | MAHAY (29) | | | | | |
| Malawi | iLiNS−DYAD−M (30) | 219 | 441 | 1.00 (0.98, 1.02) | 0.09 | 0.09 |
| Malawi | iLiNS−DOSE (31) | 619 | 222 | 1.00 (0.98, 1.02) | 0.08 | 0.08 |
| Mali | PROMIS CS (32) | 111 | 106 | 1.02 (0.97, 1.07) | 0.02 | 0.02 |
| Zimbabwe | SHINE (HIV−) (33) | | | | | |
| Zimbabwe | SHINE (HIV+) (34) | | | | | |
| | **$I^2$ = 0.00, $Tau^2$ = 0.00** | **3757** | **2680** | | | |
| | **Fixed** | | | **1.00 (1.00, 1.01)** | | |
| | **Random** | | | **1.00 (1.00, 1.01)** | | |

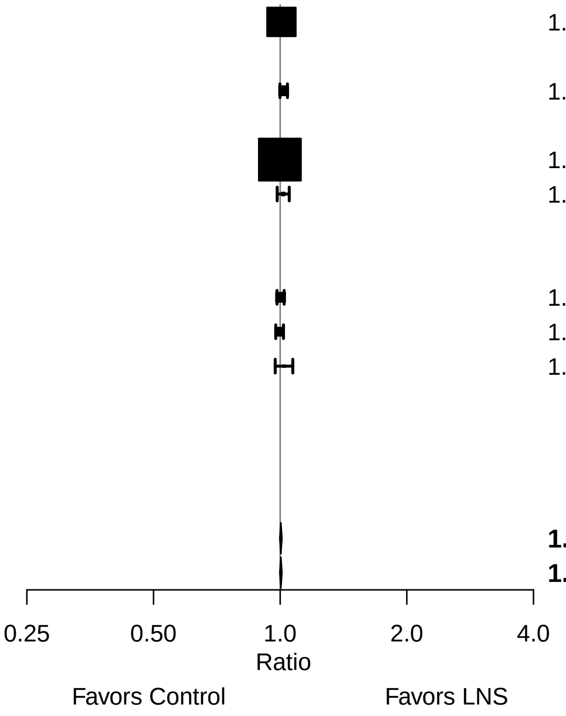

## Supplemental figure 3AB: 18-mo walking with support prevalence difference

| Country | Trial | LNS N | Control N | PD (95% CI) | Fixed W | Random W |
|---|---|---|---|---|---|---|
| Bangladesh | JiVitA−4 (21) | | | | | |
| Bangladesh | RDNS (22) | 1654 | 816 | 0.01 (0.00, 0.02) | 0.33 | 0.33 |
| Bangladesh | WASH−B (23) | | | | | |
| Burkina Faso | iLiNS−Zinc (24) | 763 | 342 | 0.02 (0.00, 0.04) | 0.06 | 0.06 |
| Ghana | GHANA (25) | | | | | |
| Ghana | iLiNS−DYAD−G (26) | 348 | 693 | 0.00 (−0.01, 0.01) | 0.39 | 0.39 |
| Haiti | HAITI (27) | 43 | 60 | 0.02 (−0.02, 0.05) | 0.03 | 0.03 |
| Kenya | WASH−B (28) | | | | | |
| Madagascar | MAHAY (29) | | | | | |
| Malawi | iLiNS−DYAD−M (30) | 219 | 441 | 0.00 (−0.02, 0.02) | 0.09 | 0.09 |
| Malawi | iLiNS−DOSE (31) | 619 | 222 | 0.00 (−0.02, 0.02) | 0.08 | 0.08 |
| Mali | PROMIS CS (32) | 111 | 106 | 0.02 (−0.03, 0.07) | 0.01 | 0.01 |
| Zimbabwe | SHINE (HIV−) (33) | | | | | |
| Zimbabwe | SHINE (HIV+) (34) | | | | | |
| | **$I^2$ = 0.00, $Tau^2$ = 0.00** | **3757** | **2680** | | | |
| | **Fixed** | | | **0.00 (0.00, 0.01)** | | |
| | **Random** | | | **0.00 (0.00, 0.01)** | | |

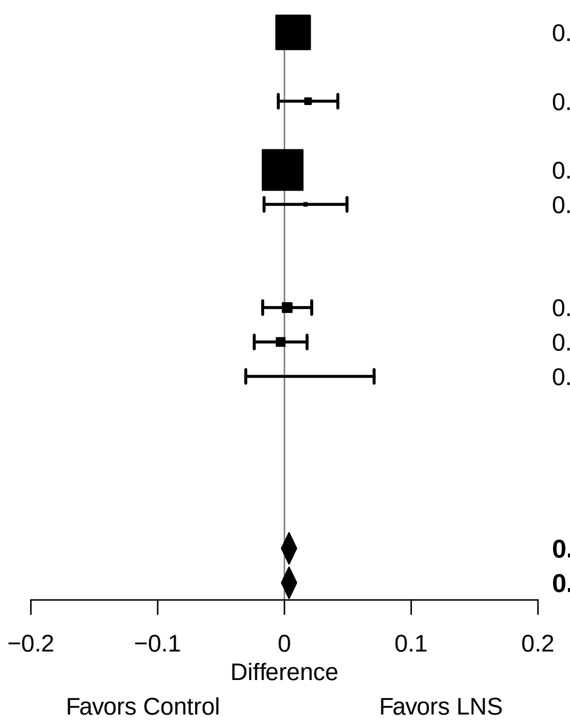

Supplement: nqab277_Supplemental_Files [file nqab277_supplemental_files.zip › ipdd_suppfig3_20210707.pdf]
