# Supplementary material for: Small-quantity lipid-based nutrient supplements for children age 6–24 months: a systematic review and individual participant data meta-analysis of effects on developmental outcomes and effect modifiers
Source: Am J Clin Nutr. 2021 Sep 29;114(Suppl 1):43S–67S. doi: 10.1093/ajcn/nqab277 (PMC8560311; doi:10.1093/ajcn/nqab277)

Supplemental figure 4: Forest plots for effects of SQ-LNS on developmental outcomes stratified by study implementation within an existing program vs not program-based

Contents

Supplemental figure 4A: Language lowest decile prevalence ratio **2**
4A1: Stratified by Study implementation 2

Supplemental figure 4B: Social-emotional lowest decile prevalence ratio **3**
4B1: Stratified by Study implementation 3

Supplemental figure 4C: Motor lowest decile prevalence ratio **4**
4C1: Stratified by Study implementation 4

This figure shows intervention effects stratified by study implementation. The figure shows the study-level estimates along with the corresponding pooled estimate grouped by category. For dichotomous outcomes analyzed via prevalence ratios, the effect estimate is theprevalence in the LNS group divided by the prevalence in the control group.
The labels on the left y-axis correspond to trial level information. The values on the right indicate the study level effect estimate, confidence interval, and weighting for deriving the pooled estimates.

Figures showing individual trial estimates for the SHINE trial are split by comparison to reflect the cross-over design. For calculating the pooled estimates shown in these figures, the trial is analyzed with LNS intervention arms combined and non-LNS intervention arms combined.

Supplemental figure 4A: Language lowest decile prevalence ratio

4A1: Stratified by Study implementation

Country	Trial	Tool	N	N	PR (95% CI)	W
**Study implementation − Program−based**						
Bangladesh	RDNS (22)	CDI	1663	814	0.72 (0.57, 0.91)	0.23
Haiti	HAITI (27)	Total sounds	150	150	2.75 (1.26, 5.98)	0.09
Madagascar	MAHAY (29)	ASQI	1613	1604	1.17 (0.75, 1.83)	0.16
Mali	PROMIS CS (32)	DMC	927	944	0.77 (0.56, 1.07)	0.20
Zimbabwe	SHINE (HIV−) (33), LNS vs SOC	CDI	381	373	1.05 (0.67, 1.65)	0.21
Zimbabwe	LNS+WSH vs WSH	CDI	436	408	0.77 (0.49, 1.20)	0.21
Zimbabwe	SHINE (HIV+) (34), LNS vs SOC	CDI	66	68	1.29 (0.54, 3.06)	0.11
Zimbabwe	LNS+WSH vs WSH	CDI	99	79	0.32 (0.10, 0.98)	0.11
**I² = 0.63, Tau² = 0.15**			**5335**	**4440**	**0.95 (0.66, 1.37)**	
**Study implementation − Not program−based**						
Bangladesh	JiVitA−4 (21)	BSID−III	445	143	0.86 (0.50, 1.49)	0.10
Bangladesh	WASH−B (23)	CDI	1109	3353	0.81 (0.65, 1.00)	0.18
Burkina Faso	iLiNS−Zinc (24)	DMC	746	375	0.46 (0.33, 0.64)	0.15
Ghana	GHANA (25)					
Ghana	iLiNS−DYAD−G (26)	CDI	331	658	1.09 (0.74, 1.61)	0.14
Kenya	WASH−B (28)	EASQ	1362	4745	0.95 (0.78, 1.16)	0.19
Malawi	iLiNS−DYAD−M (30)	CDI	215	439	0.87 (0.53, 1.43)	0.11
Malawi	iLiNS−DOSE (31)	CDI	645	221	0.95 (0.61, 1.50)	0.12
**I² = 0.65, Tau² = 0.05**			**4853**	**9934**	**0.82 (0.66, 1.02)**	

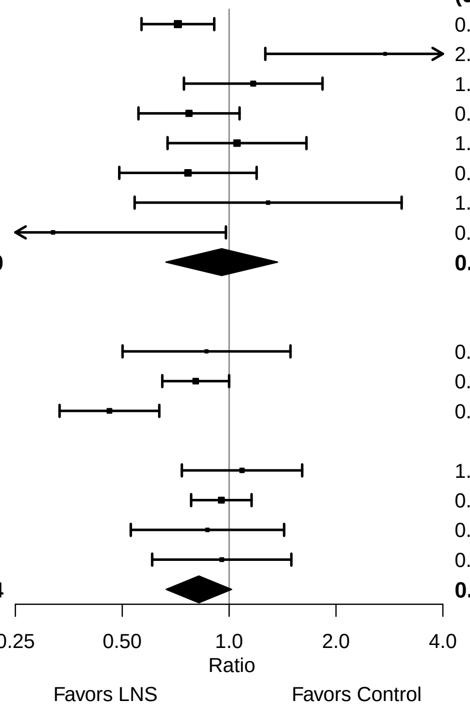

Supplemental figure 4B: Social-emotional lowest decile prevalence ratio

4B1: Stratified by Study implementation

Country	Trial	Tool	N	N	PR (95% CI)	W
**Study implementation − Program−based**						
Bangladesh	RDNS (22)	DMC	1657	815	0.84 (0.66, 1.07)	0.26
Haiti	HAITI (27)					
Madagascar	MAHAY (29)	ASQI	1613	1604	1.15 (0.75, 1.78)	0.18
Mali	PROMIS CS (32)	DMC	927	944	0.77 (0.56, 1.07)	0.22
Zimbabwe	SHINE (HIV−) (33), LNS vs SOC	MDAT	395	382	0.86 (0.56, 1.33)	0.23
Zimbabwe	LNS+WSH vs WSH	MDAT	446	417	0.62 (0.41, 0.93)	0.23
Zimbabwe	SHINE (HIV+) (34), LNS vs SOC	MDAT	67	68	0.68 (0.22, 2.04)	0.10
Zimbabwe	LNS+WSH vs WSH	MDAT	103	83	0.33 (0.15, 0.76)	0.10
**I^2 = 0.42, Tau^2 = 0.05**			**5208**	**4313**	**0.78 (0.61, 1.01)**	
**Study implementation − Not program−based**						
Bangladesh	JiVitA−4 (21)					
Bangladesh	WASH−B (23)	EASQ	1066	3266	0.81 (0.64, 1.03)	0.21
Burkina Faso	iLiNS−Zinc (24)	DMC	746	375	0.41 (0.28, 0.62)	0.15
Ghana	GHANA (25)					
Ghana	iLiNS−DYAD−G (26)	PSED	332	657	0.99 (0.67, 1.47)	0.15
Kenya	WASH−B (28)	EASQ	1362	4745	0.87 (0.71, 1.08)	0.22
Malawi	iLiNS−DYAD−M (30)	PSED	215	438	1.00 (0.61, 1.61)	0.13
Malawi	iLiNS−DOSE (31)	PSED	644	221	1.08 (0.68, 1.72)	0.13
**I^2 = 0.65, Tau^2 = 0.08**			**4365**	**9702**	**0.82 (0.62, 1.07)**	

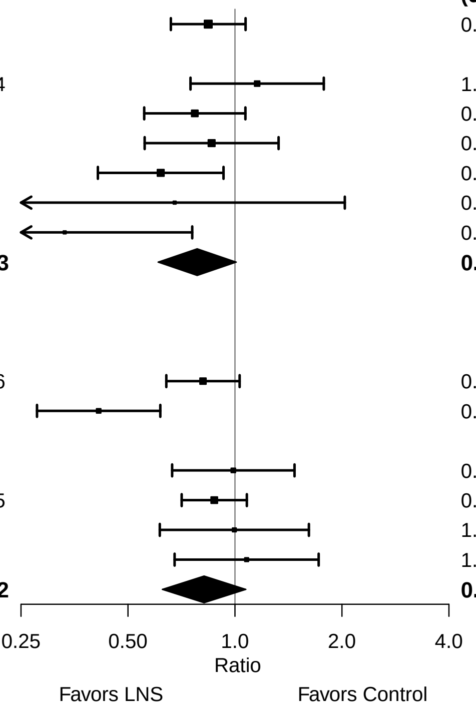

Supplemental figure 4C: Motor lowest decile prevalence ratio

4C1: Stratified by Study implementation

Study implementation − Program−based

Country	Trial	Tool	N	N	PR (95% CI)	W
Bangladesh	RDNS (22)	DMC	1556	753	0.85 (0.67, 1.07)	0.32
Haiti	HAITI (27)					
Madagascar	MAHAY (29)	ASQI	1613	1604	0.91 (0.58, 1.44)	0.15
Mali	PROMIS CS (32)	DMC	902	921	0.81 (0.56, 1.18)	0.20
Zimbabwe	SHINE (HIV−) (33), LNS vs SOC	MDAT	395	382	1.08 (0.67, 1.73)	0.24
Zimbabwe	LNS+WSH vs WSH	MDAT	446	417	0.82 (0.54, 1.24)	0.24
Zimbabwe	SHINE (HIV+) (34), LNS vs SOC	MDAT	67	68	1.16 (0.45, 3.02)	0.09
Zimbabwe	LNS+WSH vs WSH	MDAT	103	83	0.40 (0.16, 1.03)	0.09
**I² = 0.00, Tau² = 0.00**			**5082**	**4228**	**0.85 (0.73, 1.00)**	

Study implementation − Not program−based

Country	Trial	Tool	N	N	PR (95% CI)	W
Bangladesh	JiVitA−4 (21)	BSID−III	445	143	1.26 (0.77, 2.07)	0.09
Bangladesh	WASH−B (23)	EASQ	1074	3279	0.82 (0.66, 1.01)	0.22
Burkina Faso	iLiNS−Zinc (24)	DMC	746	375	0.49 (0.36, 0.67)	0.16
Ghana	GHANA (25)					
Ghana	iLiNS−DYAD−G (26)	KDI	302	601	1.13 (0.76, 1.70)	0.12
Kenya	WASH−B (28)	EASQ	1362	4745	0.86 (0.70, 1.05)	0.23
Malawi	iLiNS−DYAD−M (30)	KDI	214	436	0.84 (0.51, 1.40)	0.08
Malawi	iLiNS−DOSE (31)	KDI	646	221	1.08 (0.67, 1.71)	0.10
**I² = 0.65, Tau² = 0.06**			**4789**	**9800**	**0.86 (0.69, 1.08)**	

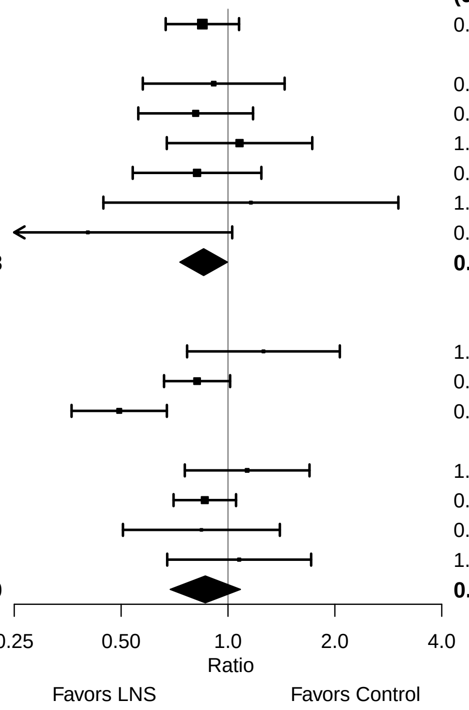

Supplement: nqab277_Supplemental_Files [file nqab277_supplemental_files.zip › ipdd_suppfig4_20210707.pdf]
