# Supplementary material for: Small-quantity lipid-based nutrient supplements for children age 6–24 months: a systematic review and individual participant data meta-analysis of effects on developmental outcomes and effect modifiers
Source: Am J Clin Nutr. 2021 Sep 29;114(Suppl 1):43S–67S. doi: 10.1093/ajcn/nqab277 (PMC8560311; doi:10.1093/ajcn/nqab277)

Supplemental figure 5: Forest plots for effects of SQ-LNS on developmental outcomes stratified by extent of social and behavioral change communication (SBCC) for infant and young child feeding (IYCF) provided in the study

Contents

Supplemental figure 5A: Language lowest decile prevalence ratio **2**
5A1: Stratified by Study SBCC for IYCF 2

Supplemental figure 5B: Social-emotional lowest decile prevalence ratio **3**
5B1: Stratified by Study SBCC for IYCF 3

Supplemental figure 5C: Motor lowest decile prevalence ratio **4**
5C1: Stratified by Study SBCC for IYCF 4

This figure shows intervention effects stratified by study SBCC for IYCF. The figure shows the study-level estimates along with the corresponding pooled estimate grouped by category. For dichotomous outcomes analyzed via prevalence ratios, the effect estimate is the prevalence in the LNS group divided by the prevalence in the control group.
The labels on the left y-axis correspond to trial level information. The values on the right indicate the study level effect estimate, confidence interval, and weighting for deriving the pooled estimates.

Figures showing individual trial estimates for the SHINE trial are split by comparison to reflect the cross-over design. For calculating the pooled estimates shown in these figures, the trial is analyzed with LNS intervention arms combined and non-LNS intervention arms combined.

Supplemental figure 5A: Language lowest decile prevalence ratio

5A1: Stratified by Study SBCC for IYCF

Country	Trial	N	N	PR (95% CI)	W
**Study SBCC for IYCF − Minimal**					
Bangladesh	RDNS (22)	1663	814	0.72 (0.57, 0.91)	0.23
Burkina Faso	iLiNS−Zinc (24)	746	375	0.46 (0.33, 0.64)	0.20
Ghana	GHANA (25)				
Ghana	iLiNS−DYAD−G (26)	331	658	1.09 (0.74, 1.61)	0.18
Haiti	HAITI (27)	150	149	2.75 (1.28, 5.93)	0.09
Malawi	iLiNS−DYAD−M (30)	215	439	0.87 (0.53, 1.43)	0.15
Malawi	iLiNS−DOSE (31)	645	221	0.95 (0.61, 1.50)	0.16
**I² = 0.80, Tau² = 0.25**		**3750**	**2656**	**0.91 (0.58, 1.42)**	
**Study SBCC for IYCF − Expanded in LNS arm(s) only**					
Kenya	WASH−B (28)	1362	4745	0.95 (0.78, 1.16)	0.31
Zimbabwe	SHINE (HIV−) (33), LNS vs SOC	381	373	1.05 (0.67, 1.65)	0.26
Zimbabwe	LNS+WSH vs WSH	436	408	0.77 (0.49, 1.20)	0.26
Zimbabwe	SHINE (HIV+) (34), LNS vs SOC	66	68	1.29 (0.54, 3.06)	0.13
Zimbabwe	LNS+WSH vs WSH	99	79	0.32 (0.11, 0.92)	0.13
**I² = 0.00, Tau² = 0.00**		**3453**	**9026**	**0.88 (0.77, 1.00)**	
**Study SBCC for IYCF − Expanded in LNS & control arms**					
Bangladesh	JiVitA−4 (21)	445	143	0.86 (0.50, 1.49)	0.29
Madagascar	MAHAY (29)	1613	821	1.25 (0.73, 2.15)	0.29
Mali	PROMIS CS (32)	927	944	0.77 (0.56, 1.07)	0.42
**I² = 0.10, Tau² = 0.01**		**2985**	**1908**	**0.88 (0.67, 1.15)**	

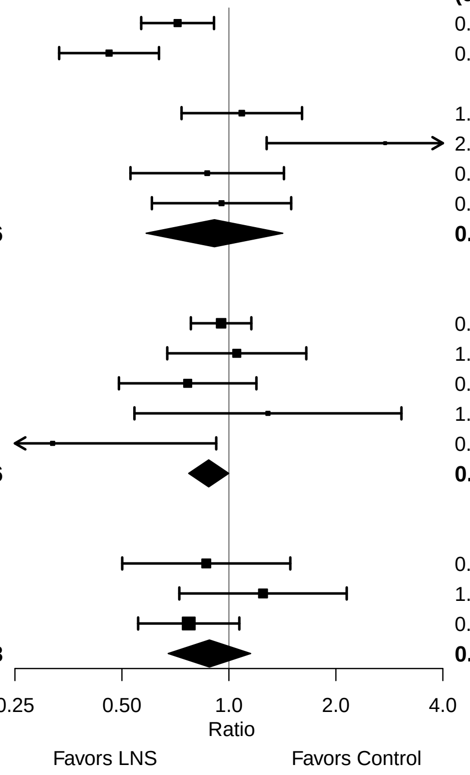

Supplemental figure 5B: Social-emotional lowest decile prevalence ratio

5B1: Stratified by Study SBCC for IYCF

Country	Trial	N	N	PR (95% CI)	W
**Study SBCC for IYCF − Minimal**					
Bangladesh	RDNS (22)	1657	815	0.84 (0.66, 1.07)	0.27
Burkina Faso	iLiNS−Zinc (24)	746	375	0.41 (0.28, 0.62)	0.20
Ghana	GHANA (25)				
Ghana	iLiNS−DYAD−G (26)	332	657	0.99 (0.67, 1.47)	0.20
Haiti	HAITI (27)				
Malawi	iLiNS−DYAD−M (30)	215	438	1.00 (0.61, 1.61)	0.17
Malawi	iLiNS−DOSE (31)	644	221	1.08 (0.68, 1.72)	0.17
**I² = 0.72, Tau² = 0.11**		**3594**	**2506**	**0.81 (0.58, 1.14)**	
**Study SBCC for IYCF − Expanded in LNS arm(s) only**					
Kenya	WASH−B (28)	1362	4745	0.87 (0.71, 1.08)	0.31
Zimbabwe	SHINE (HIV−) (33), LNS vs SOC	395	382	0.86 (0.56, 1.33)	0.27
Zimbabwe	LNS+WSH vs WSH	446	417	0.62 (0.41, 0.93)	0.27
Zimbabwe	SHINE (HIV+) (34), LNS vs SOC	67	68	0.68 (0.22, 2.04)	0.13
Zimbabwe	LNS+WSH vs WSH	103	83	0.33 (0.15, 0.76)	0.13
**I² = 0.37, Tau² = 0.03**		**3439**	**8961**	**0.76 (0.60, 0.95)**	
**Study SBCC for IYCF − Expanded in LNS & control arms**					
Bangladesh	JiVitA−4 (21)				
Madagascar	MAHAY (29)	1613	821	1.29 (0.74, 2.28)	0.38
Mali	PROMIS CS (32)	927	944	0.77 (0.56, 1.07)	0.62
**I² = 0.59, Tau² = 0.08**		**2540**	**1765**	**0.95 (0.58, 1.56)**	

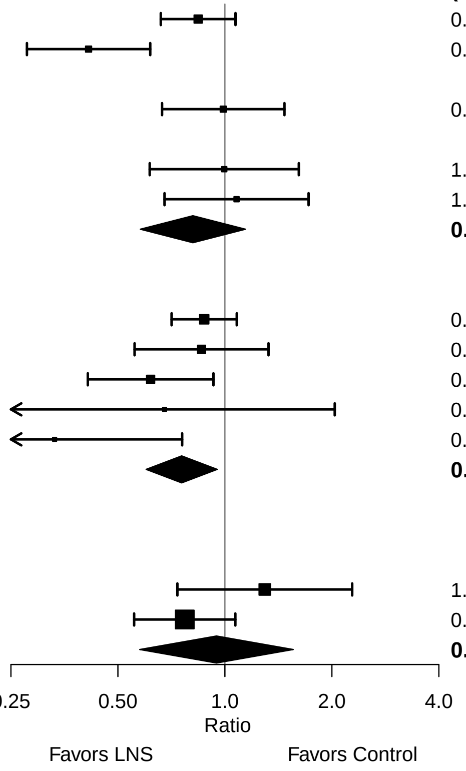

Supplemental figure 5C: Motor lowest decile prevalence ratio

5C1: Stratified by Study SBCC for IYCF

Country	Trial	N	N	PR (95% CI)	W
**Study SBCC for IYCF − Minimal**					
Bangladesh	RDNS (22)	1556	753	0.85 (0.67, 1.07)	0.31
Burkina Faso	iLiNS−Zinc (24)	746	375	0.49 (0.36, 0.67)	0.24
Ghana	GHANA (25)				
Ghana	iLiNS−DYAD−G (26)	302	601	1.13 (0.76, 1.70)	0.18
Haiti	HAITI (27)				
Malawi	iLiNS−DYAD−M (30)	214	436	0.84 (0.51, 1.40)	0.13
Malawi	iLiNS−DOSE (31)	646	221	1.08 (0.67, 1.71)	0.14
**I² = 0.71, Tau² = 0.08**		**3464**	**2386**	**0.83 (0.61, 1.11)**	
**Study SBCC for IYCF − Expanded in LNS arm(s) only**					
Kenya	WASH−B (28)	1362	4745	0.86 (0.70, 1.05)	0.35
Zimbabwe	SHINE (HIV−) (33), LNS vs SOC	395	382	1.08 (0.67, 1.73)	0.24
Zimbabwe	LNS+WSH vs WSH	446	417	0.82 (0.54, 1.24)	0.24
Zimbabwe	SHINE (HIV+) (34), LNS vs SOC	67	68	1.16 (0.44, 3.08)	0.08
Zimbabwe	LNS+WSH vs WSH	103	83	0.40 (0.17, 0.95)	0.08
**I² = 0.00, Tau² = 0.00**		**3447**	**8974**	**0.85 (0.74, 0.96)**	
**Study SBCC for IYCF − Expanded in LNS & control arms**					
Bangladesh	JiVitA−4 (21)	445	143	1.26 (0.77, 2.07)	0.31
Madagascar	MAHAY (29)	1613	821	0.97 (0.52, 1.80)	0.22
Mali	PROMIS CS (32)	902	921	0.81 (0.56, 1.18)	0.47
**I² = 0.00, Tau² = 0.00**		**2960**	**1885**	**0.95 (0.73, 1.25)**	

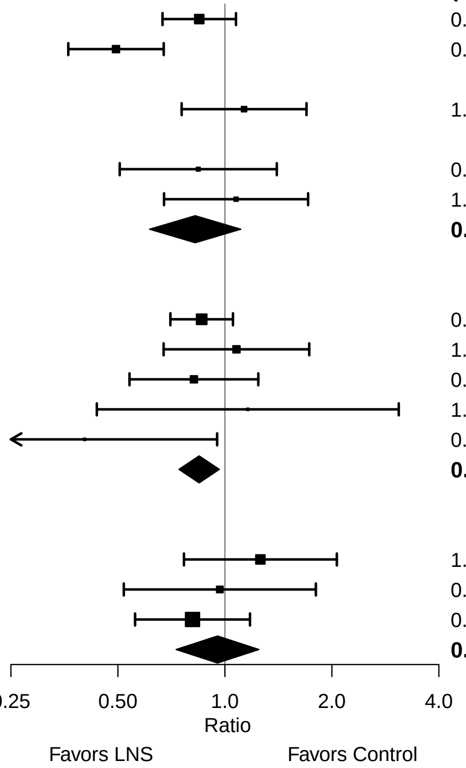

Supplement: nqab277_Supplemental_Files [file nqab277_supplemental_files.zip › ipdd_suppfig5_20210707.pdf]
