# Supplementary material for: Small-quantity lipid-based nutrient supplements for children age 6–24 months: a systematic review and individual participant data meta-analysis of effects on developmental outcomes and effect modifiers
Source: Am J Clin Nutr. 2021 Sep 29;114(Suppl 1):43S–67S. doi: 10.1093/ajcn/nqab277 (PMC8560311; doi:10.1093/ajcn/nqab277)

Supplemental figure 6: Forest plots for effects of SQ-LNS on developmental outcomes stratified by study-level effect modifiers

Contents

Supplemental figure 6A: Mean difference in language z-score **6**
6A1: Stratified by Geographic region . . . 6
6A2: Stratified by Stunting burden . . . 7
6A3: Stratified by Malaria prevalence . . . 8
6A4: Stratified by Anemia prevalence . . . 9
6A5: Stratified by Source water quality . . . 10
6A6: Stratified by Sanitation . . . 11
6A7: Stratified by Supplement duration . . . 12
6A8: Stratified by Frequency of contact . . . 13
6A9: Stratified by Average SQ-LNS compliance . . . 14

Supplemental figure 6B: Language lowest decile prevalence ratio **15**
6B1: Stratified by Geographic region . . . 15
6B2: Stratified by Stunting burden . . . 16
6B3: Stratified by Malaria prevalence . . . 17
6B4: Stratified by Anemia prevalence . . . 18
6B5: Stratified by Source water quality . . . 19
6B6: Stratified by Sanitation . . . 20
6B7: Stratified by Supplement duration . . . 21
6B8: Stratified by Frequency of contact . . . 22
6B9: Stratified by Average SQ-LNS compliance . . . 23

Supplemental figure 6C: Language lowest decile prevalence difference **24**
6C1: Stratified by Geographic region . . . 24
6C2: Stratified by Stunting burden . . . 25
6C3: Stratified by Malaria prevalence . . . 26
6C4: Stratified by Anemia prevalence . . . 27
6C5: Stratified by Source water quality . . . 28
6C6: Stratified by Sanitation . . . 29
6C7: Stratified by Supplement duration . . . 30

6C8: Stratified by Frequency of contact . . . 31
6C9: Stratified by Average SQ-LNS compliance . . . 32

Supplemental figure 6D: Mean difference in social-emotional z-score **33**
6D1: Stratified by Geographic region (insufficient comparisons) . . . 33
6D2: Stratified by Stunting burden . . . 34
6D3: Stratified by Malaria prevalence . . . 35
6D4: Stratified by Anemia prevalence . . . 36
6D5: Stratified by Source water quality . . . 37
6D6: Stratified by Sanitation . . . 38
6D7: Stratified by Supplement duration . . . 39
6D8: Stratified by Frequency of contact . . . 40
6D9: Stratified by Average SQ-LNS compliance . . . 41

Supplemental figure 6E: Social-emotional lowest decile prevalence ratio **42**
6E1: Stratified by Geographic region (insufficient comparisons) . . . 42
6E2: Stratified by Stunting burden . . . 43
6E3: Stratified by Malaria prevalence . . . 44
6E4: Stratified by Anemia prevalence . . . 45
6E5: Stratified by Source water quality . . . 46
6E6: Stratified by Sanitation . . . 47
6E7: Stratified by Supplement duration . . . 48
6E8: Stratified by Frequency of contact . . . 49
6E9: Stratified by Average SQ-LNS compliance . . . 50

Supplemental figure 6F: Social-emotional lowest decile prevalence difference **51**
6F1: Stratified by Geographic region (insufficient comparisons) . . . 51
6F2: Stratified by Stunting burden . . . 52
6F3: Stratified by Malaria prevalence . . . 53
6F4: Stratified by Anemia prevalence . . . 54
6F5: Stratified by Source water quality . . . 55
6F6: Stratified by Sanitation . . . 56
6F7: Stratified by Supplement duration . . . 57
6F8: Stratified by Frequency of contact . . . 58
6F9: Stratified by Average SQ-LNS compliance . . . 59

Supplemental figure 6G: Mean difference in motor z-score **60**
6G1: Stratified by Geographic region . . . 60
6G2: Stratified by Stunting burden . . . 61
6G3: Stratified by Malaria prevalence . . . 62
6G4: Stratified by Anemia prevalence . . . 63
6G5: Stratified by Source water quality . . . 64

6G6: Stratified by Sanitation . . . 65
6G7: Stratified by Supplement duration . . . 66
6G8: Stratified by Frequency of contact . . . 67
6G9: Stratified by Average SQ-LNS compliance . . . 68

Supplemental figure 6H: Motor lowest decile prevalence ratio 69
6H1: Stratified by Geographic region . . . 69
6H2: Stratified by Stunting burden . . . 70
6H3: Stratified by Malaria prevalence . . . 71
6H4: Stratified by Anemia prevalence . . . 72
6H5: Stratified by Source water quality . . . 73
6H6: Stratified by Sanitation . . . 74
6H7: Stratified by Supplement duration . . . 75
6H8: Stratified by Frequency of contact . . . 76
6H9: Stratified by Average SQ-LNS compliance . . . 77

Supplemental figure 6I: Motor lowest decile prevalence difference 78
6I1: Stratified by Geographic region . . . 78
6I2: Stratified by Stunting burden . . . 79
6I3: Stratified by Malaria prevalence . . . 80
6I4: Stratified by Anemia prevalence . . . 81
6I5: Stratified by Source water quality . . . 82
6I6: Stratified by Sanitation . . . 83
6I7: Stratified by Supplement duration . . . 84
6I8: Stratified by Frequency of contact . . . 85
6I9: Stratified by Average SQ-LNS compliance . . . 86

Supplemental figure 6J: Mean difference in gross motor z-score 87
6J1: Stratified by Geographic region . . . 87
6J2: Stratified by Stunting burden . . . 88
6J3: Stratified by Malaria prevalence . . . 89
6J4: Stratified by Anemia prevalence . . . 90
6J5: Stratified by Source water quality . . . 91
6J6: Stratified by Sanitation . . . 92
6J7: Stratified by Supplement duration . . . 93
6J8: Stratified by Frequency of contact . . . 94
6J9: Stratified by Average SQ-LNS compliance . . . 95

Supplemental figure 6K: Mean difference in fine motor z-score 96
6K1: Stratified by Geographic region (insufficient comparisons) . . . 96
6K2: Stratified by Stunting burden (insufficient comparisons) . . . 97
6K3: Stratified by Malaria prevalence . . . 98

6K4: Stratified by Anemia prevalence . . . 99
6K5: Stratified by Source water quality . . . 100
6K6: Stratified by Sanitation . . . 101
6K7: Stratified by Supplement duration (insufficient comparisons) . . . 102
6K8: Stratified by Frequency of contact . . . 103
6K9: Stratified by Average SQ-LNS compliance (insufficient comparisons) . . . 104

Supplemental figure 6L: Mean difference in executive function z-score 105
6L1: Stratified by Geographic region (insufficient comparisons) . . . 105
6L2: Stratified by Stunting burden (insufficient comparisons) . . . 106
6L3: Stratified by Malaria prevalence . . . 107
6L4: Stratified by Anemia prevalence (insufficient comparisons) . . . 108
6L5: Stratified by Source water quality (insufficient comparisons) . . . 109
6L6: Stratified by Sanitation . . . 110
6L7: Stratified by Supplement duration (insufficient comparisons) . . . 111
6L8: Stratified by Frequency of contact . . . 112
6L9: Stratified by Average SQ-LNS compliance (insufficient comparisons) . . . 113

Supplemental figure 6M: Executive function lowest decile prevalence ratio 114
6M1: Stratified by Geographic region (insufficient comparisons) . . . 114
6M2: Stratified by Stunting burden (insufficient comparisons) . . . 115
6M3: Stratified by Malaria prevalence . . . 116
6M4: Stratified by Anemia prevalence (insufficient comparisons) . . . 117
6M5: Stratified by Source water quality (insufficient comparisons) . . . 118
6M6: Stratified by Sanitation . . . 119
6M7: Stratified by Supplement duration (insufficient comparisons) . . . 120
6M8: Stratified by Frequency of contact . . . 121
6M9: Stratified by Average SQ-LNS compliance (insufficient comparisons) . . . 122

Supplemental figure 6N: Executive function lowest decile prevalence difference 123
6N1: Stratified by Geographic region (insufficient comparisons) . . . 123
6N2: Stratified by Stunting burden (insufficient comparisons) . . . 124
6N3: Stratified by Malaria prevalence . . . 125
6N4: Stratified by Anemia prevalence (insufficient comparisons) . . . 126
6N5: Stratified by Source water quality (insufficient comparisons) . . . 127
6N6: Stratified by Sanitation . . . 128
6N7: Stratified by Supplement duration (insufficient comparisons) . . . 129
6N8: Stratified by Frequency of contact . . . 130
6N9: Stratified by Average SQ-LNS compliance (insufficient comparisons) . . . 131

Supplemental figure 6O: 12-mo walking without support prevalence ratio 132
6O1: Stratified by Geographic region . . . 132

6O2: Stratified by Stunting burden . . . 133
6O3: Stratified by Malaria prevalence . . . 134
6O4: Stratified by Anemia prevalence . . . 135
6O5: Stratified by Source water quality (insufficient comparisons) . . . 136
6O6: Stratified by Sanitation . . . 137
6O7: Stratified by Supplement duration . . . 138
6O8: Stratified by Frequency of contact . . . 139
6O9: Stratified by Average SQ-LNS compliance . . . 140

Supplemental figure 6P: 12-mo walking without support prevalence difference 141
6P1: Stratified by Geographic region . . . 141
6P2: Stratified by Stunting burden . . . 142
6P3: Stratified by Malaria prevalence . . . 143
6P4: Stratified by Anemia prevalence . . . 144
6P5: Stratified by Source water quality (insufficient comparisons) . . . 145
6P6: Stratified by Sanitation . . . 146
6P7: Stratified by Supplement duration . . . 147
6P8: Stratified by Frequency of contact . . . 148
6P9: Stratified by Average SQ-LNS compliance . . . 149

These figures are forest plots showing the study-level effect modification of intervention effects. Each figure shows the study-level estimates along with the corresponding pooled estimate grouped by study-level effect modifier category. For definitions of effect modifiers, see Box 1 in the main paper.
Individual study estimates were generated from log-binomial regression for dichotomous outcomes and simple linear regression for continuous outcomes; controlling for baseline measure when available and with clustered observations using robust standard errors for cluster-randomized trials. Pooled sub-group estimates were generated using inverse-variance weighting random effects. P-value for the difference was estimated using random effects meta-regression with the indicated effect modifier as the predictor of intervention effect size; stratified pooled estimates are presented for each strata. For dichotomous outcomes analyzed via prevalence ratios, the effect estimate is the prevalence in the LNS group divided by the prevalence in the control group. For dichotomous outcomes analyzed via prevalence differences, the effect estimate is the prevalence in the LNS group minus the prevalence in the control group. The labels on the left y-axis correspond to trial level information. The values on the right indicate the study level effect estimate, confidence interval, and weighting for deriving the pooled estimates.

Figures showing individual trial estimates for the SHINE trial are split by comparison to reflect the cross-over design. For calculating the pooled estimates shown in these figures, the trial is analyzed with LNS intervention arms combined and non-LNS intervention arms combined. Except for when sanitation or water quality are the effect modifier then the LNS +WSH vs WSH comparison is excluded.

6O2: Stratified by Stunting burden . . . 133
6O3: Stratified by Malaria prevalence . . . 134
6O4: Stratified by Anemia prevalence . . . 135
6O5: Stratified by Source water quality (insufficient comparisons) . . . 136
6O6: Stratified by Sanitation . . . 137
6O7: Stratified by Supplement duration . . . 138
6O8: Stratified by Frequency of contact . . . 139
6O9: Stratified by Average SQ-LNS compliance . . . 140

Supplemental figure 6P: 12-mo walking without support prevalence difference **141**
6P1: Stratified by Geographic region . . . 141
6P2: Stratified by Stunting burden . . . 142
6P3: Stratified by Malaria prevalence . . . 143
6P4: Stratified by Anemia prevalence . . . 144
6P5: Stratified by Source water quality (insufficient comparisons) . . . 145
6P6: Stratified by Sanitation . . . 146
6P7: Stratified by Supplement duration . . . 147
6P8: Stratified by Frequency of contact . . . 148
6P9: Stratified by Average SQ-LNS compliance . . . 149

Supplemental figure 6A: Mean difference in language z-score

6A1: Stratified by Geographic region

Geographic region
(p−diff = 0.978)

Country	Trial	Tool	N	N	MD (95% CI)	W
**Geographic region − SEAR**						
Bangladesh	JiVitA−4 (21)	BSID−III	445	143	−0.08 (−0.29, 0.12)	0.22
Bangladesh	RDNS (22)	CDI	1663	814	0.17 (0.07, 0.26)	0.37
Bangladesh	WASH−B (23)	CDI	1109	3353	0.09 (0.02, 0.17)	0.41
**I² = 0.58, Tau² = 0.01**			**3217**	**4310**	**0.08 (−0.04, 0.21)**	
**Geographic region − AFR**						
Burkina Faso	iLiNS−Zinc (24)	DMC	746	375	0.37 (0.21, 0.53)	0.10
Ghana	GHANA (25)					
Ghana	iLiNS−DYAD−G (26)	CDI	331	658	0.01 (−0.12, 0.14)	0.12
Kenya	WASH−B (28)	EASQ	1362	4745	0.02 (−0.04, 0.08)	0.16
Madagascar	MAHAY (29)	ASQI	1613	1604	−0.11 (−0.30, 0.07)	0.09
Malawi	iLiNS−DYAD−M (30)	CDI	215	439	0.01 (−0.15, 0.18)	0.10
Malawi	iLiNS−DOSE (31)	CDI	645	221	0.05 (−0.11, 0.20)	0.11
Mali	PROMIS CS (32)	DMC	927	944	0.15 (−0.02, 0.31)	0.10
Zimbabwe	SHINE (HIV−) (33), LNS vs SOC	CDI	381	373	0.03 (−0.15, 0.21)	0.13
Zimbabwe	LNS+WSH vs WSH	CDI	436	408	0.13 (−0.02, 0.29)	0.13
Zimbabwe	SHINE (HIV+) (34), LNS vs SOC	CDI	66	68	0.06 (−0.29, 0.40)	0.08
Zimbabwe	LNS+WSH vs WSH	CDI	99	79	0.37 (0.07, 0.67)	0.08
**I² = 0.66, Tau² = 0.01**			**6821**	**9914**	**0.08 (−0.01, 0.17)**	

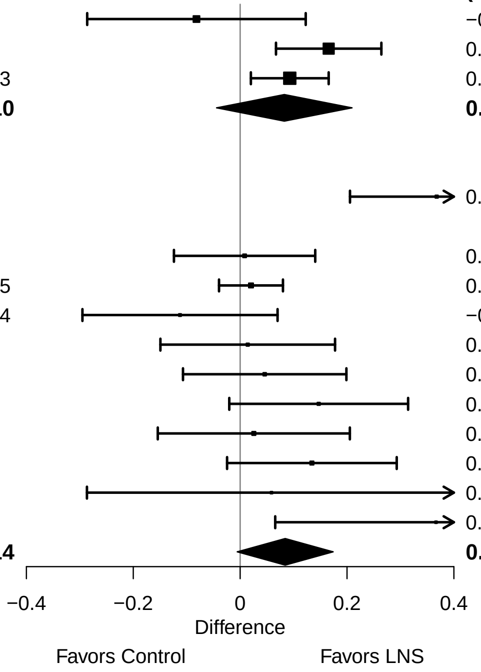

Supplemental figure 6A: Mean difference in language z-score

6A2: Stratified by Stunting burden

Stunting burden
(p−diff = 0.077)

Country	Trial	Tool	N	N	MD (95% CI)	W
**Stunting burden − Less than 35%**						
Ghana	GHANA (25)					
Ghana	iLiNS−DYAD−G (26)	CDI	331	658	0.01 (−0.12, 0.14)	0.26
Haiti	HAITI (27)	Total sounds	150	149	−0.13 (−0.35, 0.10)	0.17
Kenya	WASH−B (28)	EASQ	1362	4745	0.02 (−0.04, 0.08)	0.34
Malawi	iLiNS−DYAD−M (30)	CDI	215	439	0.01 (−0.15, 0.18)	0.23
**I² = 0.00, Tau² = 0.00**			**2058**	**5991**	**0.01 (−0.04, 0.06)**	
**Stunting burden − More than 35%**						
Bangladesh	JiVitA−4 (21)	BSID−III	445	143	−0.08 (−0.29, 0.12)	0.09
Bangladesh	RDNS (22)	CDI	1663	814	0.17 (0.07, 0.26)	0.14
Bangladesh	WASH−B (23)	CDI	1109	3353	0.09 (0.02, 0.17)	0.15
Burkina Faso	iLiNS−Zinc (24)	DMC	746	375	0.37 (0.21, 0.53)	0.11
Madagascar	MAHAY (29)	ASQI	1613	1604	−0.11 (−0.30, 0.07)	0.10
Malawi	iLiNS−DOSE (31)	CDI	645	221	0.05 (−0.11, 0.20)	0.11
Mali	PROMIS CS (32)	DMC	927	944	0.15 (−0.02, 0.31)	0.10
Zimbabwe	SHINE (HIV−) (33), LNS vs SOC	CDI	381	373	0.03 (−0.15, 0.21)	0.13
Zimbabwe	LNS+WSH vs WSH	CDI	436	408	0.13 (−0.02, 0.29)	0.13
Zimbabwe	SHINE (HIV+) (34), LNS vs SOC	CDI	66	68	0.06 (−0.29, 0.40)	0.08
Zimbabwe	LNS+WSH vs WSH	CDI	99	79	0.37 (0.07, 0.67)	0.08
**I² = 0.65, Tau² = 0.01**			**8130**	**8382**	**0.11 (0.02, 0.20)**	

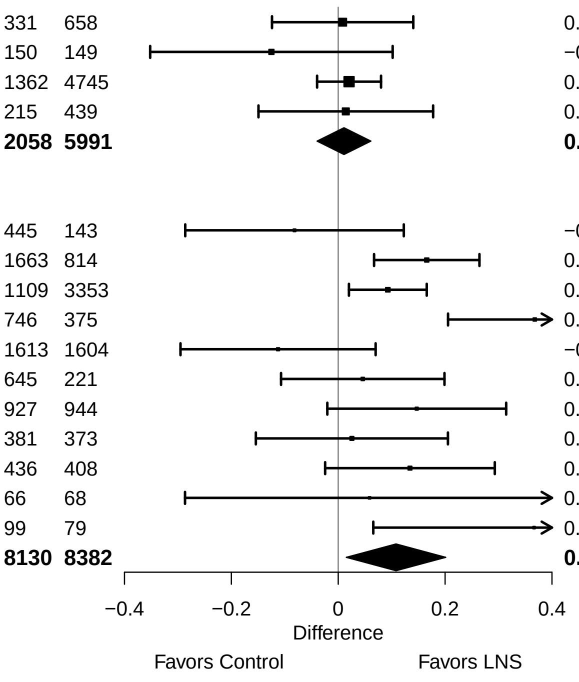

Supplemental figure 6A: Mean difference in language z-score

6A3: Stratified by Malaria prevalence

Malaria prevalence
(p−diff = 0.365)

Country	Trial	Tool	N	N	MD (95% CI)	W
**Malaria prevalence − Less than 10%**						
Bangladesh	JiVitA−4 (21)	BSID−III	445	143	−0.08 (−0.29, 0.12)	0.10
Bangladesh	RDNS (22)	CDI	1663	814	0.17 (0.07, 0.26)	0.15
Bangladesh	WASH−B (23)	CDI	1109	3353	0.09 (0.02, 0.17)	0.17
Haiti	HAITI (27)	Total sounds	150	149	−0.13 (−0.35, 0.10)	0.09
Kenya	WASH−B (28)	EASQ	1362	4745	0.02 (−0.04, 0.08)	0.17
Madagascar	MAHAY (29)	ASQI	1613	1604	−0.11 (−0.30, 0.07)	0.11
Zimbabwe	SHINE (HIV−) (33), LNS vs SOC	CDI	381	373	0.03 (−0.15, 0.21)	0.14
Zimbabwe	LNS+WSH vs WSH	CDI	436	408	0.13 (−0.02, 0.29)	0.14
Zimbabwe	SHINE (HIV+) (34), LNS vs SOC	CDI	66	68	0.06 (−0.29, 0.40)	0.09
Zimbabwe	LNS+WSH vs WSH	CDI	99	79	0.37 (0.07, 0.67)	0.09
**I² = 0.60, Tau² = 0.01**			**7324**	**11736**	**0.05 (−0.03, 0.13)**	
**Malaria prevalence − At least 10%**						
Burkina Faso	iLiNS−Zinc (24)	DMC	746	375	0.37 (0.21, 0.53)	0.19
Ghana	GHANA (25)					
Ghana	iLiNS−DYAD−G (26)	CDI	331	658	0.01 (−0.12, 0.14)	0.22
Malawi	iLiNS−DYAD−M (30)	CDI	215	439	0.01 (−0.15, 0.18)	0.19
Malawi	iLiNS−DOSE (31)	CDI	645	221	0.05 (−0.11, 0.20)	0.20
Mali	PROMIS CS (32)	DMC	927	944	0.15 (−0.02, 0.31)	0.19
**I² = 0.72, Tau² = 0.02**			**2864**	**2637**	**0.11 (−0.02, 0.25)**	

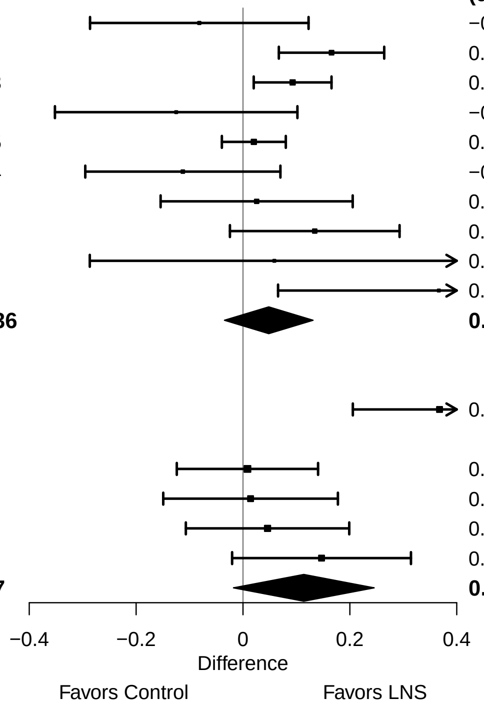

Supplemental figure 6A: Mean difference in language z-score

6A4: Stratified by Anemia prevalence

Anemia prevalence
(p−diff = 0.530)

Country	Trial	Tool	N	N	MD (95% CI)	W
**Anemia prevalence − High**						
Burkina Faso	iLiNS−Zinc (24)	DMC	746	375	0.37 (0.21, 0.53)	0.21
Ghana	GHANA (25)					
Haiti	HAITI (27)	Total sounds	150	149	−0.13 (−0.35, 0.10)	0.16
Malawi	iLiNS−DYAD−M (30)	CDI	215	439	0.01 (−0.15, 0.18)	0.21
Malawi	iLiNS−DOSE (31)	CDI	645	221	0.05 (−0.11, 0.20)	0.22
Mali	PROMIS CS (32)	DMC	927	944	0.15 (−0.02, 0.31)	0.21
**I² = 0.75, Tau² = 0.02**			**2683**	**2128**	**0.10 (−0.06, 0.26)**	
**Anemia prevalence − Moderate**						
Bangladesh	JiVitA−4 (21)	BSID−III	445	143	−0.08 (−0.29, 0.12)	0.09
Bangladesh	RDNS (22)	CDI	1663	814	0.17 (0.07, 0.26)	0.15
Bangladesh	WASH−B (23)	CDI	1109	3353	0.09 (0.02, 0.17)	0.16
Ghana	iLiNS−DYAD−G (26)	CDI	331	658	0.01 (−0.12, 0.14)	0.13
Kenya	WASH−B (28)	EASQ	1362	4745	0.02 (−0.04, 0.08)	0.16
Madagascar	MAHAY (29)	ASQI	1613	1604	−0.11 (−0.30, 0.07)	0.10
Zimbabwe	SHINE (HIV−) (33), LNS vs SOC	CDI	381	373	0.03 (−0.15, 0.21)	0.13
Zimbabwe	LNS+WSH vs WSH	CDI	436	408	0.13 (−0.02, 0.29)	0.13
Zimbabwe	SHINE (HIV+) (34), LNS vs SOC	CDI	66	68	0.06 (−0.29, 0.40)	0.08
Zimbabwe	LNS+WSH vs WSH	CDI	99	79	0.37 (0.07, 0.67)	0.08
**I² = 0.55, Tau² = 0.01**			**7505**	**12245**	**0.06 (−0.01, 0.13)**	

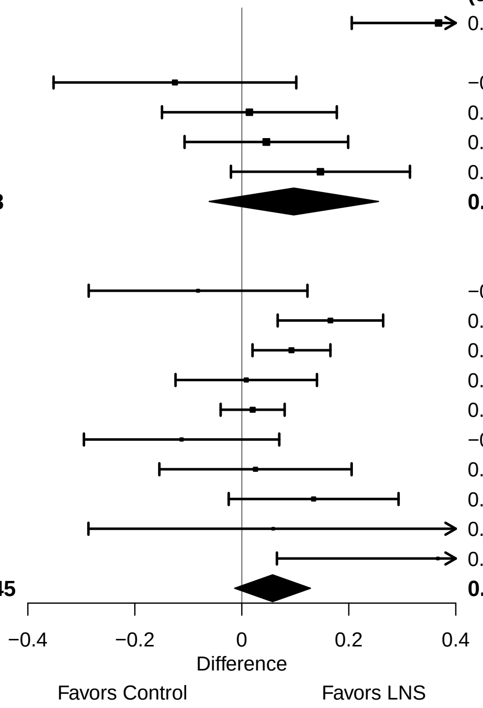

Supplemental figure 6A: Mean difference in language z-score

6A5: Stratified by Source water quality

Source water quality
(p−diff = 0.536)

Country	Trial	Tool	N	N	MD (95% CI)	W
**Source water quality − Improved**						
Bangladesh	JiVitA−4 (21)	BSID−III	445	143	−0.08 (−0.29, 0.12)	0.11
Bangladesh	RDNS (22)	CDI	1663	814	0.17 (0.07, 0.26)	0.18
Bangladesh	WASH−B (23)	CDI	548	1099	0.17 (0.06, 0.29)	0.17
Ghana	GHANA (25)					
Ghana	iLiNS−DYAD−G (26)	CDI	331	658	0.01 (−0.12, 0.14)	0.16
Haiti	HAITI (27)	Total sounds	150	149	−0.13 (−0.35, 0.10)	0.10
Malawi	iLiNS−DYAD−M (30)	CDI	215	439	0.01 (−0.15, 0.18)	0.14
Malawi	iLiNS−DOSE (31)	CDI	645	221	0.05 (−0.11, 0.20)	0.14
**I^2 = 0.54, Tau^2 = 0.01**			**3997**	**3523**	**0.05 (−0.03, 0.14)**	
**Source water quality − Unimproved**						
Burkina Faso	iLiNS−Zinc (24)	DMC	746	375	0.37 (0.21, 0.53)	0.18
Kenya	WASH−B (28)	EASQ	649	2073	0.05 (−0.05, 0.15)	0.24
Madagascar	MAHAY (29)	ASQI	1613	1604	−0.11 (−0.30, 0.07)	0.16
Mali	PROMIS CS (32)	DMC	927	944	0.15 (−0.02, 0.31)	0.18
Zimbabwe	SHINE (HIV−) (33)	CDI	381	373	0.03 (−0.15, 0.21)	0.17
Zimbabwe	SHINE (HIV+) (34)	CDI	66	68	0.06 (−0.29, 0.40)	0.08
**I^2 = 0.72, Tau^2 = 0.02**			**4382**	**5437**	**0.09 (−0.04, 0.22)**	

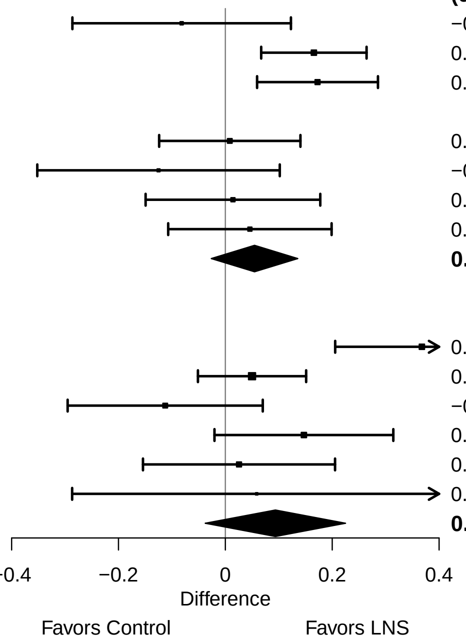

Supplemental figure 6A: Mean difference in language z-score

6A6: Stratified by Sanitation

Sanitation
(p−diff = 0.991)

Sanitation − Improved

Country	Trial	Tool	N	N	MD (95% CI)	W
Bangladesh	JiVitA−4 (21)	BSID−III	445	143	−0.08 (−0.29, 0.12)	0.13
Bangladesh	RDNS (22)	CDI	1663	814	0.17 (0.07, 0.26)	0.21
Bangladesh	WASH−B (23)	CDI	548	1099	0.17 (0.06, 0.29)	0.20
Ghana	GHANA (25)					
Ghana	iLiNS−DYAD−G (26)	CDI	331	658	0.01 (−0.12, 0.14)	0.18
Haiti	HAITI (27)	Total sounds	150	149	−0.13 (−0.35, 0.10)	0.12
Mali	PROMIS CS (32)	DMC	927	944	0.15 (−0.02, 0.31)	0.16
**I² = 0.59, Tau² = 0.01**			**4064**	**3807**	**0.07 (−0.03, 0.17)**	

Sanitation − Unimproved

Country	Trial	Tool	N	N	MD (95% CI)	W
Burkina Faso	iLiNS−Zinc (24)	DMC	746	375	0.37 (0.21, 0.53)	0.15
Kenya	WASH−B (28)	EASQ	649	2073	0.05 (−0.05, 0.15)	0.20
Madagascar	MAHAY (29)	ASQI	1613	1604	−0.11 (−0.30, 0.07)	0.14
Malawi	iLiNS−DYAD−M (30)	CDI	215	439	0.01 (−0.15, 0.18)	0.15
Malawi	iLiNS−DOSE (31)	CDI	645	221	0.05 (−0.11, 0.20)	0.16
Zimbabwe	SHINE (HIV−) (33)	CDI	381	373	0.03 (−0.15, 0.21)	0.14
Zimbabwe	SHINE (HIV+) (34)	CDI	66	68	0.06 (−0.29, 0.40)	0.06
**I² = 0.66, Tau² = 0.01**			**4315**	**5153**	**0.07 (−0.04, 0.18)**	

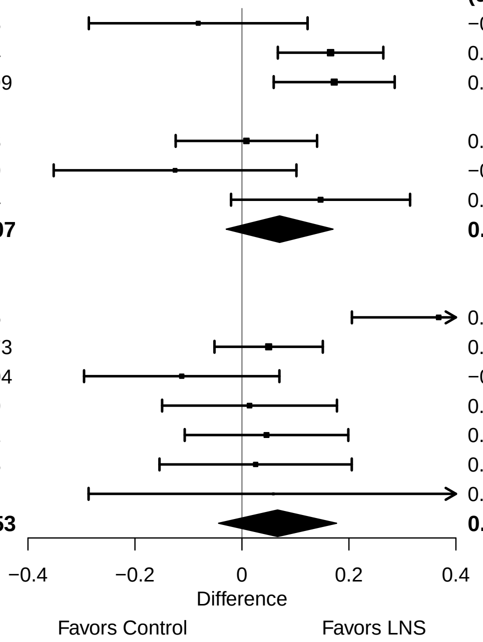

Supplemental figure 6A: Mean difference in language z-score

6A7: Stratified by Supplement duration

Supplement duration
(p−diff = 0.524)

Country	Trial	Tool	N	N	MD (95% CI)	W
**Supplement duration − 12m or less**						
Bangladesh	JiVitA−4 (21)	BSID−III	445	143	−0.08 (−0.29, 0.12)	0.10
Burkina Faso	iLiNS−Zinc (24)	DMC	746	375	0.37 (0.21, 0.53)	0.12
Ghana	GHANA (25)					
Ghana	iLiNS−DYAD−G (26)	CDI	331	658	0.01 (−0.12, 0.14)	0.13
Haiti	HAITI (27)	Total sounds	150	149	−0.13 (−0.35, 0.10)	0.09
Madagascar	MAHAY (29)	ASQI	1613	1604	−0.11 (−0.30, 0.07)	0.11
Malawi	iLiNS−DYAD−M (30)	CDI	215	439	0.01 (−0.15, 0.18)	0.12
Malawi	iLiNS−DOSE (31)	CDI	645	221	0.05 (−0.11, 0.20)	0.12
Zimbabwe	SHINE (HIV−) (33), LNS vs SOC	CDI	381	373	0.03 (−0.15, 0.21)	0.14
Zimbabwe	LNS+WSH vs WSH	CDI	436	408	0.13 (−0.02, 0.29)	0.14
Zimbabwe	SHINE (HIV+) (34), LNS vs SOC	CDI	66	68	0.06 (−0.29, 0.40)	0.09
Zimbabwe	LNS+WSH vs WSH	CDI	99	79	0.37 (0.07, 0.67)	0.09
**I² = 0.68, Tau² = 0.02**			**5127**	**4517**	**0.05 (−0.05, 0.16)**	
**Supplement duration − > 12m**						
Bangladesh	RDNS (22)	CDI	1663	814	0.17 (0.07, 0.26)	0.25
Bangladesh	WASH−B (23)	CDI	1109	3353	0.09 (0.02, 0.17)	0.27
Kenya	WASH−B (28)	EASQ	1362	4745	0.02 (−0.04, 0.08)	0.28
Mali	PROMIS CS (32)	DMC	927	944	0.15 (−0.02, 0.31)	0.19
**I² = 0.59, Tau² = 0.00**			**5061**	**9856**	**0.09 (0.02, 0.16)**	

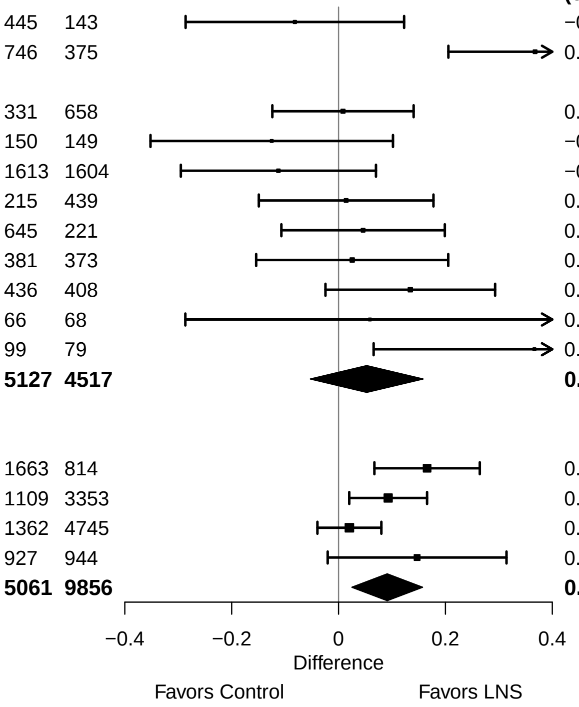

Supplemental figure 6A: Mean difference in language z-score

6A8: Stratified by Frequency of contact

Frequency of contact
(p−diff = 0.853)

Country	Trial	Tool	N	N	MD (95% CI)	W
**Frequency of contact − Monthly**						
Bangladesh	RDNS (22)	CDI	1663	814	0.17 (0.07, 0.26)	0.18
Haiti	HAITI (27)	Total sounds	150	149	−0.13 (−0.35, 0.10)	0.10
Kenya	WASH−B (28)	EASQ	1362	4745	0.02 (−0.04, 0.08)	0.20
Madagascar	MAHAY (29)	ASQI	1613	1604	−0.11 (−0.30, 0.07)	0.12
Mali	PROMIS CS (32)	DMC	927	944	0.15 (−0.02, 0.31)	0.13
Zimbabwe	SHINE (HIV−) (33), LNS vs SOC	CDI	381	373	0.03 (−0.15, 0.21)	0.16
Zimbabwe	LNS+WSH vs WSH	CDI	436	408	0.13 (−0.02, 0.29)	0.16
Zimbabwe	SHINE (HIV+) (34), LNS vs SOC	CDI	66	68	0.06 (−0.29, 0.40)	0.10
Zimbabwe	LNS+WSH vs WSH	CDI	99	79	0.37 (0.07, 0.67)	0.10
**I² = 0.62, Tau² = 0.01**			**6697**	**9184**	**0.07 (−0.03, 0.16)**	
**Frequency of contact − Weekly**						
Bangladesh	JiVitA−4 (21)	BSID−III	445	143	−0.08 (−0.29, 0.12)	0.13
Bangladesh	WASH−B (23)	CDI	1109	3353	0.09 (0.02, 0.17)	0.22
Burkina Faso	iLiNS−Zinc (24)	DMC	746	375	0.37 (0.21, 0.53)	0.16
Ghana	GHANA (25)					
Ghana	iLiNS−DYAD−G (26)	CDI	331	658	0.01 (−0.12, 0.14)	0.18
Malawi	iLiNS−DYAD−M (30)	CDI	215	439	0.01 (−0.15, 0.18)	0.15
Malawi	iLiNS−DOSE (31)	CDI	645	221	0.05 (−0.11, 0.20)	0.16
**I² = 0.70, Tau² = 0.02**			**3491**	**5189**	**0.08 (−0.04, 0.20)**	

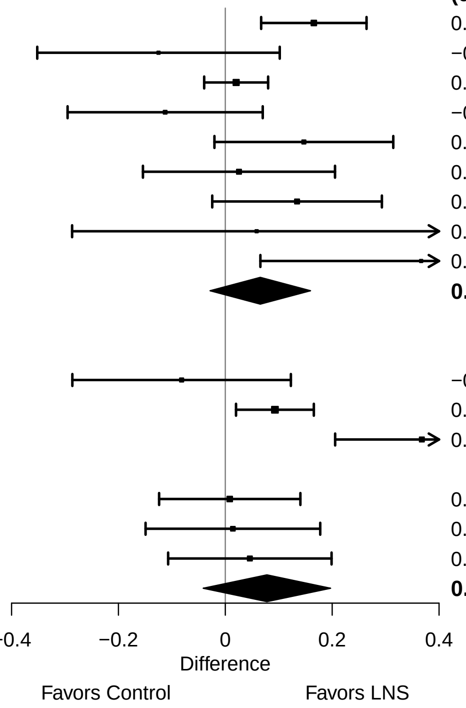

Supplemental figure 6A: Mean difference in language z-score

6A9: Stratified by Average SQ-LNS compliance

Average SQ−LNS compliance
(p−diff = 0.918)

Country	Trial	Tool	N	N	MD (95% CI)	W
**Average SQ−LNS compliance − Low**						
Ghana	iLiNS−DYAD−G (26)	CDI	331	658	0.01 (−0.12, 0.14)	0.19
Malawi	iLiNS−DYAD−M (30)	CDI	215	439	0.01 (−0.15, 0.18)	0.16
Malawi	iLiNS−DOSE (31)	CDI	645	221	0.05 (−0.11, 0.20)	0.17
Mali	PROMIS CS (32)	DMC	927	944	0.15 (−0.02, 0.31)	0.16
Zimbabwe	SHINE (HIV−) (33), LNS vs SOC	CDI	381	373	0.03 (−0.15, 0.21)	0.20
Zimbabwe	LNS+WSH vs WSH	CDI	436	408	0.13 (−0.02, 0.29)	0.20
Zimbabwe	SHINE (HIV+) (34), LNS vs SOC	CDI	66	68	0.06 (−0.29, 0.40)	0.12
Zimbabwe	LNS+WSH vs WSH	CDI	99	79	0.37 (0.07, 0.67)	0.12
**I² = 0.00, Tau² = 0.00**			**3100**	**3190**	**0.07 (0.01, 0.13)**	
**Average SQ−LNS compliance − High**						
Bangladesh	JiVitA−4 (21)	BSID−III	445	143	−0.08 (−0.29, 0.12)	0.12
Bangladesh	RDNS (22)	CDI	1663	814	0.17 (0.07, 0.26)	0.20
Bangladesh	WASH−B (23)	CDI	1109	3353	0.09 (0.02, 0.17)	0.21
Burkina Faso	iLiNS−Zinc (24)	DMC	746	375	0.37 (0.21, 0.53)	0.15
Ghana	GHANA (25)					
Haiti	HAITI (27)	Total sounds	150	149	−0.13 (−0.35, 0.10)	0.10
Kenya	WASH−B (28)	EASQ	1362	4745	0.02 (−0.04, 0.08)	0.22
**I² = 0.80, Tau² = 0.02**			**5475**	**9579**	**0.08 (−0.05, 0.22)**	

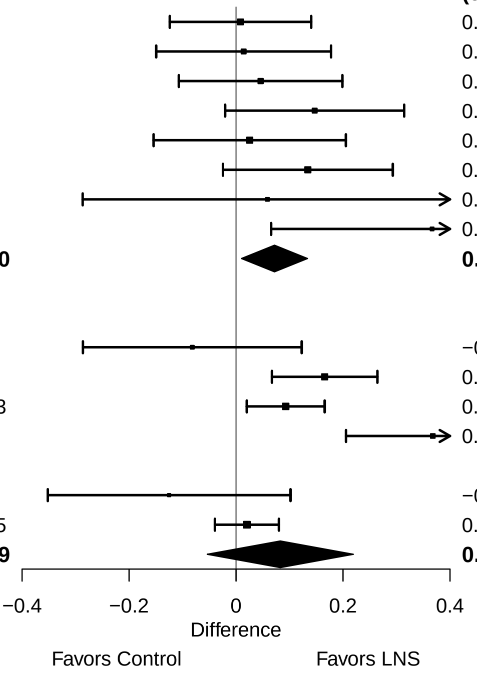

Supplemental figure 6B: Language lowest decile prevalence ratio

6B1: Stratified by Geographic region

Geographic region
(p−diff = 0.624)

Country	Trial	Tool	N	N	PR (95% CI)	W
**Geographic region − SEAR**						
Bangladesh	JiVitA−4 (21)	BSID−III	445	143	0.86 (0.50, 1.49)	0.16
Bangladesh	RDNS (22)	CDI	1663	814	0.72 (0.57, 0.91)	0.41
Bangladesh	WASH−B (23)	CDI	1109	3353	0.81 (0.65, 1.00)	0.43
**I² = 0.00, Tau² = 0.00**			**3217**	**4310**	**0.77 (0.66, 0.90)**	
**Geographic region − AFR**						
Burkina Faso	iLiNS−Zinc (24)	DMC	746	375	0.46 (0.33, 0.64)	0.13
Ghana	GHANA (25)					
Ghana	iLiNS−DYAD−G (26)	CDI	331	658	1.09 (0.74, 1.61)	0.11
Kenya	WASH−B (28)	EASQ	1362	4745	0.95 (0.78, 1.16)	0.20
Madagascar	MAHAY (29)	ASQI	1613	1604	1.17 (0.75, 1.83)	0.09
Malawi	iLiNS−DYAD−M (30)	CDI	215	439	0.87 (0.53, 1.43)	0.08
Malawi	iLiNS−DOSE (31)	CDI	645	221	0.95 (0.61, 1.50)	0.09
Mali	PROMIS CS (32)	DMC	927	944	0.77 (0.56, 1.07)	0.13
Zimbabwe	SHINE (HIV−) (33), LNS vs SOC	CDI	381	373	1.05 (0.67, 1.65)	0.13
Zimbabwe	LNS+WSH vs WSH	CDI	436	408	0.77 (0.49, 1.20)	0.13
Zimbabwe	SHINE (HIV+) (34), LNS vs SOC	CDI	66	68	1.29 (0.54, 3.06)	0.05
Zimbabwe	LNS+WSH vs WSH	CDI	99	79	0.32 (0.11, 0.92)	0.05
**I² = 0.59, Tau² = 0.04**			**6821**	**9914**	**0.84 (0.70, 1.02)**	

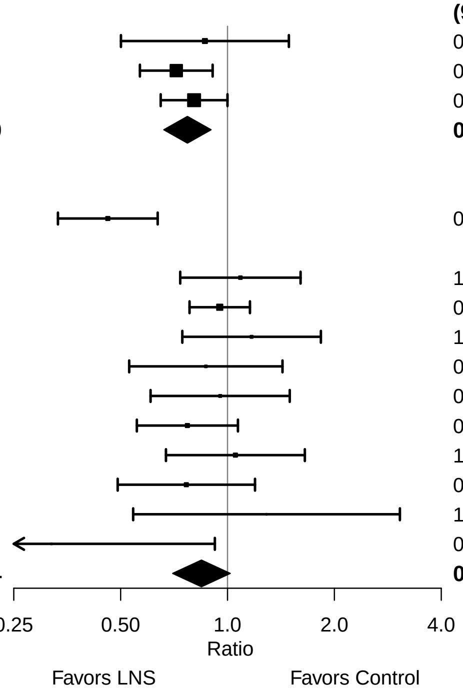

Supplemental figure 6B: Language lowest decile prevalence ratio

6B2: Stratified by Stunting burden

Stunting burden
(p−diff = 0.046)

Country	Trial		Tool	N	N	PR (95% CI)	W
**Stunting burden − Less than 35%**							
Ghana	GHANA (25)						
Ghana	iLiNS−DYAD−G (26)		CDI	331	658	1.09 (0.74, 1.61)	0.27
Haiti	HAITI (27)		Total sounds	150	149	2.75 (1.28, 5.93)	0.14
Kenya	WASH−B (28)		EASQ	1362	4745	0.95 (0.78, 1.16)	0.37
Malawi	iLiNS−DYAD−M (30)		CDI	215	439	0.87 (0.53, 1.43)	0.22
**I² = 0.59, Tau² = 0.16**				**2058**	**5991**	**1.16 (0.73, 1.83)**	
**Stunting burden − More than 35%**							
Bangladesh	JiVitA−4 (21)		BSID−III	445	143	0.86 (0.50, 1.49)	0.08
Bangladesh	RDNS (22)		CDI	1663	814	0.72 (0.57, 0.91)	0.14
Bangladesh	WASH−B (23)		CDI	1109	3353	0.81 (0.65, 1.00)	0.14
Burkina Faso	iLiNS−Zinc (24)		DMC	746	375	0.46 (0.33, 0.64)	0.12
Madagascar	MAHAY (29)		ASQI	1613	1604	1.17 (0.75, 1.83)	0.10
Malawi	iLiNS−DOSE (31)		CDI	645	221	0.95 (0.61, 1.50)	0.10
Mali	PROMIS CS (32)		DMC	927	944	0.77 (0.56, 1.07)	0.12
Zimbabwe	SHINE (HIV−) (33),	LNS vs SOC	CDI	381	373	1.05 (0.67, 1.65)	0.12
Zimbabwe		LNS+WSH vs WSH	CDI	436	408	0.77 (0.49, 1.20)	0.12
Zimbabwe	SHINE (HIV+) (34),	LNS vs SOC	CDI	66	68	1.29 (0.54, 3.06)	0.06
Zimbabwe		LNS+WSH vs WSH	CDI	99	79	0.32 (0.11, 0.92)	0.06
**I² = 0.49, Tau² = 0.03**				**8130**	**8382**	**0.78 (0.66, 0.92)**	

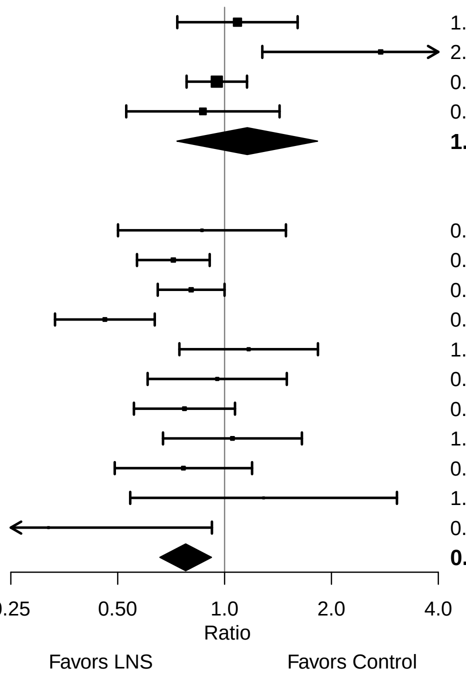

Supplemental figure 6B: Language lowest decile prevalence ratio

6B3: Stratified by Malaria prevalence

Malaria prevalence
(p−diff = 0.322)

Malaria prevalence − Less than 10%

**Country**	**Trial**	**Tool**	**N**	**N**	**PR (95% CI)**	**W**
Bangladesh	JiVitA−4 (21)	BSID−III	445	143	0.86 (0.50, 1.49)	0.10
Bangladesh	RDNS (22)	CDI	1663	814	0.72 (0.57, 0.91)	0.16
Bangladesh	WASH−B (23)	CDI	1109	3353	0.81 (0.65, 1.00)	0.17
Haiti	HAITI (27)	Total sounds	150	149	2.75 (1.28, 5.93)	0.06
Kenya	WASH−B (28)	EASQ	1362	4745	0.95 (0.78, 1.16)	0.17
Madagascar	MAHAY (29)	ASQI	1613	1604	1.17 (0.75, 1.83)	0.11
Zimbabwe	SHINE (HIV−) (33), LNS vs SOC	CDI	381	373	1.05 (0.67, 1.65)	0.14
Zimbabwe	LNS+WSH vs WSH	CDI	436	408	0.77 (0.49, 1.20)	0.14
Zimbabwe	SHINE (HIV+) (34), LNS vs SOC	CDI	66	68	1.29 (0.54, 3.06)	0.07
Zimbabwe	LNS+WSH vs WSH	CDI	99	79	0.32 (0.11, 0.92)	0.07
**I² = 0.52, Tau² = 0.08**			**7324**	**11736**	**0.93 (0.73, 1.18)**	

Malaria prevalence − At least 10%

**Country**	**Trial**	**Tool**	**N**	**N**	**PR (95% CI)**	**W**
Burkina Faso	iLiNS−Zinc (24)	DMC	746	375	0.46 (0.33, 0.64)	0.23
Ghana	GHANA (25)					
Ghana	iLiNS−DYAD−G (26)	CDI	331	658	1.09 (0.74, 1.61)	0.20
Malawi	iLiNS−DYAD−M (30)	CDI	215	439	0.87 (0.53, 1.43)	0.17
Malawi	iLiNS−DOSE (31)	CDI	645	221	0.95 (0.61, 1.50)	0.18
Mali	PROMIS CS (32)	DMC	927	944	0.77 (0.56, 1.07)	0.22
**I² = 0.71, Tau² = 0.08**			**2864**	**2637**	**0.78 (0.58, 1.05)**	

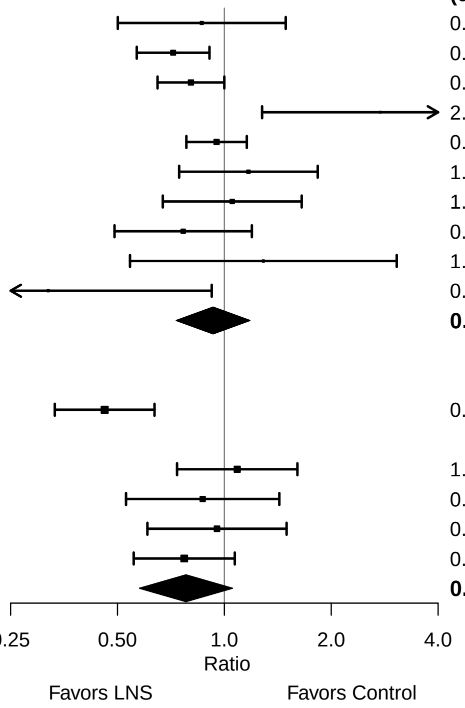

Supplemental figure 6B: Language lowest decile prevalence ratio

6B4: Stratified by Anemia prevalence

Anemia prevalence
(p−diff = 0.644)

Country	Trial		Tool	N	N	PR (95% CI)	W
**Anemia prevalence − High**							
Burkina Faso	iLiNS−Zinc (24)		DMC	746	375	0.46 (0.33, 0.64)	0.25
Ghana	GHANA (25)						
Haiti	HAITI (27)		Total sounds	150	149	2.75 (1.28, 5.93)	0.11
Malawi	iLiNS−DYAD−M (30)		CDI	215	439	0.87 (0.53, 1.43)	0.18
Malawi	iLiNS−DOSE (31)		CDI	645	221	0.95 (0.61, 1.50)	0.20
Mali	PROMIS CS (32)		DMC	927	944	0.77 (0.56, 1.07)	0.25
**I² = 0.81, Tau² = 0.32**				**2683**	**2128**	**0.90 (0.53, 1.55)**	
**Anemia prevalence − Moderate**							
Bangladesh	JiVitA−4 (21)		BSID−III	445	143	0.86 (0.50, 1.49)	0.09
Bangladesh	RDNS (22)		CDI	1663	814	0.72 (0.57, 0.91)	0.15
Bangladesh	WASH−B (23)		CDI	1109	3353	0.81 (0.65, 1.00)	0.16
Ghana	iLiNS−DYAD−G (26)		CDI	331	658	1.09 (0.74, 1.61)	0.12
Kenya	WASH−B (28)		EASQ	1362	4745	0.95 (0.78, 1.16)	0.16
Madagascar	MAHAY (29)		ASQI	1613	1604	1.17 (0.75, 1.83)	0.11
Zimbabwe	SHINE (HIV−) (33),	LNS vs SOC	CDI	381	373	1.05 (0.67, 1.65)	0.14
Zimbabwe		LNS+WSH vs WSH	CDI	436	408	0.77 (0.49, 1.20)	0.14
Zimbabwe	SHINE (HIV+) (34),	LNS vs SOC	CDI	66	68	1.29 (0.54, 3.06)	0.07
Zimbabwe		LNS+WSH vs WSH	CDI	99	79	0.32 (0.11, 0.92)	0.07
**I² = 0.03, Tau² = 0.00**				**7505**	**12245**	**0.87 (0.78, 0.97)**	

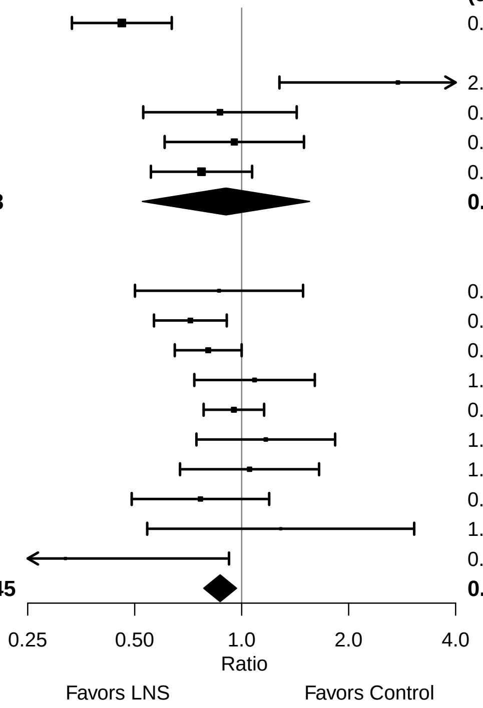

Supplemental figure 6B: Language lowest decile prevalence ratio

6B5: Stratified by Source water quality

Source water quality
(p−diff = 0.737)

Country	Trial	Tool	N	N	PR (95% CI)	W
**Source water quality − Improved**						
Bangladesh	JiVitA−4 (21)	BSID−III	445	143	0.86 (0.50, 1.49)	0.12
Bangladesh	RDNS (22)	CDI	1663	814	0.72 (0.57, 0.91)	0.19
Bangladesh	WASH−B (23)	CDI	548	1099	0.64 (0.46, 0.89)	0.17
Ghana	GHANA (25)					
Ghana	iLiNS−DYAD−G (26)	CDI	331	658	1.09 (0.74, 1.61)	0.16
Haiti	HAITI (27)	Total sounds	150	149	2.75 (1.28, 5.93)	0.08
Malawi	iLiNS−DYAD−M (30)	CDI	215	439	0.87 (0.53, 1.43)	0.13
Malawi	iLiNS−DOSE (31)	CDI	645	221	0.95 (0.61, 1.50)	0.14
**I² = 0.61, Tau² = 0.12**			**3997**	**3523**	**0.93 (0.68, 1.26)**	
**Source water quality − Unimproved**						
Burkina Faso	iLiNS−Zinc (24)	DMC	746	375	0.46 (0.33, 0.64)	0.20
Kenya	WASH−B (28)	EASQ	649	2073	0.96 (0.72, 1.28)	0.21
Madagascar	MAHAY (29)	ASQI	1613	1604	1.17 (0.75, 1.83)	0.16
Mali	PROMIS CS (32)	DMC	927	944	0.77 (0.56, 1.07)	0.19
Zimbabwe	SHINE (HIV−) (33)	CDI	381	373	1.05 (0.67, 1.65)	0.16
Zimbabwe	SHINE (HIV+) (34)	CDI	66	68	1.29 (0.54, 3.06)	0.08
**I² = 0.72, Tau² = 0.09**			**4382**	**5437**	**0.85 (0.63, 1.15)**	

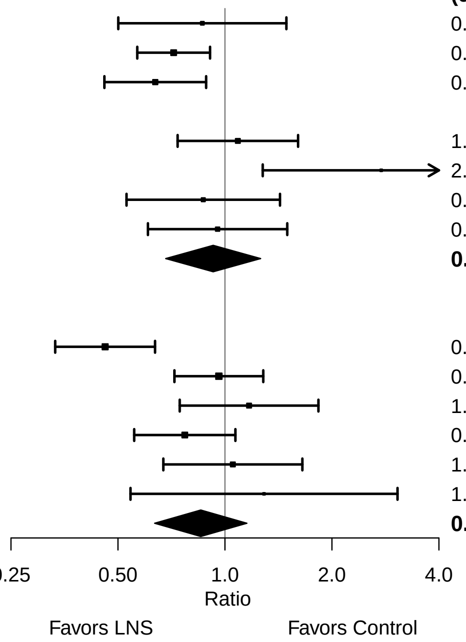

Supplemental figure 6B: Language lowest decile prevalence ratio

6B6: Stratified by Sanitation

Sanitation
(p−diff = 0.994)

Country	Trial	Tool	N	N	PR (95% CI)	W
**Sanitation − Improved**						
Bangladesh	JiVitA−4 (21)	BSID−III	445	143	0.86 (0.50, 1.49)	0.14
Bangladesh	RDNS (22)	CDI	1663	814	0.72 (0.57, 0.91)	0.22
Bangladesh	WASH−B (23)	CDI	548	1099	0.64 (0.46, 0.89)	0.19
Ghana	GHANA (25)					
Ghana	iLiNS−DYAD−G (26)	CDI	331	658	1.09 (0.74, 1.61)	0.17
Haiti	HAITI (27)	Total sounds	150	149	2.75 (1.28, 5.93)	0.09
Mali	PROMIS CS (32)	DMC	927	944	0.77 (0.56, 1.07)	0.19
**I² = 0.67, Tau² = 0.17**			**4064**	**3807**	**0.91 (0.63, 1.33)**	
**Sanitation − Unimproved**						
Burkina Faso	iLiNS−Zinc (24)	DMC	746	375	0.46 (0.33, 0.64)	0.18
Kenya	WASH−B (28)	EASQ	649	2073	0.96 (0.72, 1.28)	0.18
Madagascar	MAHAY (29)	ASQI	1613	1604	1.17 (0.75, 1.83)	0.14
Malawi	iLiNS−DYAD−M (30)	CDI	215	439	0.87 (0.53, 1.43)	0.13
Malawi	iLiNS−DOSE (31)	CDI	645	221	0.95 (0.61, 1.50)	0.14
Zimbabwe	SHINE (HIV−) (33)	CDI	381	373	1.05 (0.67, 1.65)	0.14
Zimbabwe	SHINE (HIV+) (34)	CDI	66	68	1.29 (0.54, 3.06)	0.07
**I² = 0.67, Tau² = 0.07**			**4315**	**5153**	**0.88 (0.68, 1.14)**	

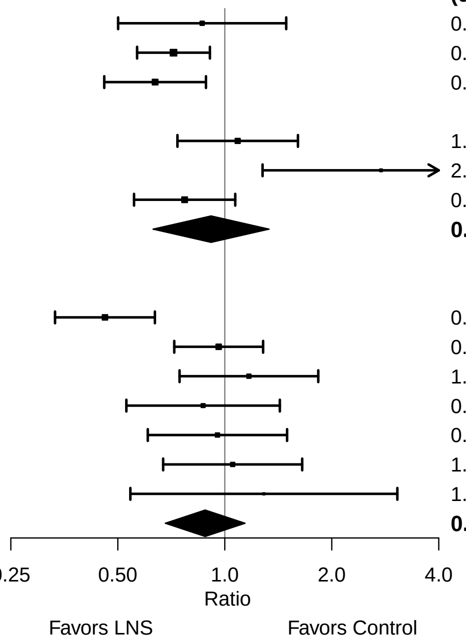

Supplemental figure 6B: Language lowest decile prevalence ratio

6B7: Stratified by Supplement duration

Supplement duration
(p−diff = 0.545)

Country	Trial	Tool	N	N	PR (95% CI)	W
**Supplement duration − 12m or less**						
Bangladesh	JiVitA−4 (21)	BSID−III	445	143	0.86 (0.50, 1.49)	0.10
Burkina Faso	iLiNS−Zinc (24)	DMC	746	375	0.46 (0.33, 0.64)	0.15
Ghana	GHANA (25)					
Ghana	iLiNS−DYAD−G (26)	CDI	331	658	1.09 (0.74, 1.61)	0.13
Haiti	HAITI (27)	Total sounds	150	149	2.75 (1.28, 5.93)	0.07
Madagascar	MAHAY (29)	ASQI	1613	1604	1.17 (0.75, 1.83)	0.12
Malawi	iLiNS−DYAD−M (30)	CDI	215	439	0.87 (0.53, 1.43)	0.11
Malawi	iLiNS−DOSE (31)	CDI	645	221	0.95 (0.61, 1.50)	0.12
Zimbabwe	SHINE (HIV−) (33), LNS vs SOC	CDI	381	373	1.05 (0.67, 1.65)	0.15
Zimbabwe	LNS+WSH vs WSH	CDI	436	408	0.77 (0.49, 1.20)	0.15
Zimbabwe	SHINE (HIV+) (34), LNS vs SOC	CDI	66	68	1.29 (0.54, 3.06)	0.08
Zimbabwe	LNS+WSH vs WSH	CDI	99	79	0.32 (0.11, 0.92)	0.08
**I² = 0.70, Tau² = 0.13**			**5127**	**4517**	**0.92 (0.69, 1.23)**	
**Supplement duration − > 12m**						
Bangladesh	RDNS (22)	CDI	1663	814	0.72 (0.57, 0.91)	0.25
Bangladesh	WASH−B (23)	CDI	1109	3353	0.81 (0.65, 1.00)	0.26
Kenya	WASH−B (28)	EASQ	1362	4745	0.95 (0.78, 1.16)	0.27
Mali	PROMIS CS (32)	DMC	927	944	0.77 (0.56, 1.07)	0.22
**I² = 0.16, Tau² = 0.00**			**5061**	**9856**	**0.82 (0.73, 0.93)**	

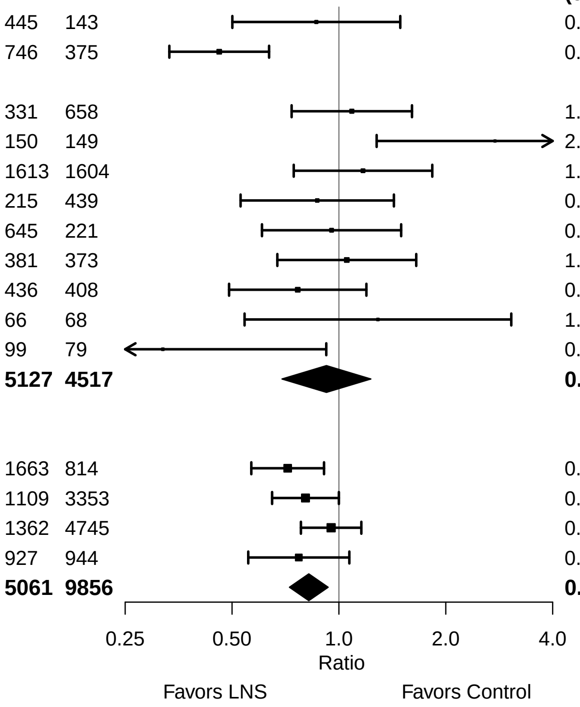

Supplemental figure 6B: Language lowest decile prevalence ratio

6B8: Stratified by Frequency of contact

Frequency of contact
(p−diff = 0.370)

Country	Trial	Tool	N	N	PR (95% CI)	W
**Frequency of contact − Monthly**						
Bangladesh	RDNS (22)	CDI	1663	814	0.72 (0.57, 0.91)	0.19
Haiti	HAITI (27)	Total sounds	150	149	2.75 (1.28, 5.93)	0.07
Kenya	WASH−B (28)	EASQ	1362	4745	0.95 (0.78, 1.16)	0.20
Madagascar	MAHAY (29)	ASQI	1613	1604	1.17 (0.75, 1.83)	0.13
Mali	PROMIS CS (32)	DMC	927	944	0.77 (0.56, 1.07)	0.16
Zimbabwe	SHINE (HIV−) (33), LNS vs SOC	CDI	381	373	1.05 (0.67, 1.65)	0.16
Zimbabwe	LNS+WSH vs WSH	CDI	436	408	0.77 (0.49, 1.20)	0.16
Zimbabwe	SHINE (HIV+) (34), LNS vs SOC	CDI	66	68	1.29 (0.54, 3.06)	0.08
Zimbabwe	LNS+WSH vs WSH	CDI	99	79	0.32 (0.11, 0.92)	0.08
**I² = 0.59, Tau² = 0.11**			**6697**	**9184**	**0.95 (0.71, 1.26)**	
**Frequency of contact − Weekly**						
Bangladesh	JiVitA−4 (21)	BSID−III	445	143	0.86 (0.50, 1.49)	0.13
Bangladesh	WASH−B (23)	CDI	1109	3353	0.81 (0.65, 1.00)	0.22
Burkina Faso	iLiNS−Zinc (24)	DMC	746	375	0.46 (0.33, 0.64)	0.19
Ghana	GHANA (25)					
Ghana	iLiNS−DYAD−G (26)	CDI	331	658	1.09 (0.74, 1.61)	0.17
Malawi	iLiNS−DYAD−M (30)	CDI	215	439	0.87 (0.53, 1.43)	0.14
Malawi	iLiNS−DOSE (31)	CDI	645	221	0.95 (0.61, 1.50)	0.15
**I² = 0.65, Tau² = 0.06**			**3491**	**5189**	**0.79 (0.62, 1.02)**	

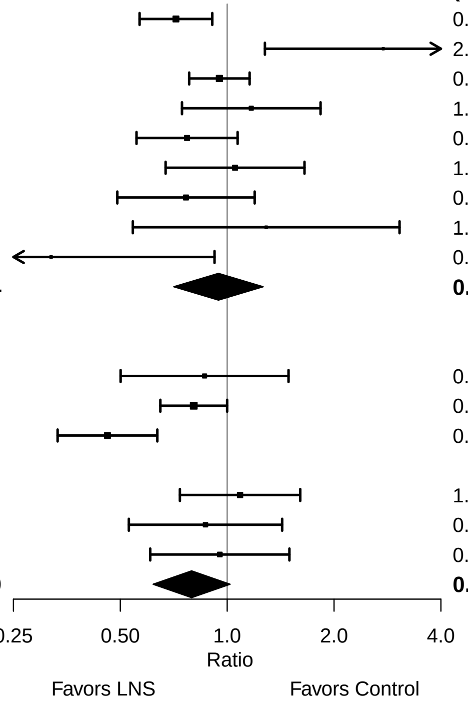

Supplemental figure 6B: Language lowest decile prevalence ratio

6B9: Stratified by Average SQ-LNS compliance

Average SQ−LNS compliance
(p−diff = 0.616)

Country	Trial	Tool	N	N	PR (95% CI)	W
**Average SQ−LNS compliance − Low**						
Ghana	iLiNS−DYAD−G (26)	CDI	331	658	1.09 (0.74, 1.61)	0.18
Malawi	iLiNS−DYAD−M (30)	CDI	215	439	0.87 (0.53, 1.43)	0.15
Malawi	iLiNS−DOSE (31)	CDI	645	221	0.95 (0.61, 1.50)	0.16
Mali	PROMIS CS (32)	DMC	927	944	0.77 (0.56, 1.07)	0.20
Zimbabwe	SHINE (HIV−) (33), LNS vs SOC	CDI	381	373	1.05 (0.67, 1.65)	0.20
Zimbabwe	LNS+WSH vs WSH	CDI	436	408	0.77 (0.49, 1.20)	0.20
Zimbabwe	SHINE (HIV+) (34), LNS vs SOC	CDI	66	68	1.29 (0.54, 3.06)	0.11
Zimbabwe	LNS+WSH vs WSH	CDI	99	79	0.32 (0.11, 0.92)	0.11
**I² = 0.00, Tau² = 0.00**			**3100**	**3190**	**0.88 (0.75, 1.04)**	
**Average SQ−LNS compliance − High**						
Bangladesh	JiVitA−4 (21)	BSID−III	445	143	0.86 (0.50, 1.49)	0.12
Bangladesh	RDNS (22)	CDI	1663	814	0.72 (0.57, 0.91)	0.20
Bangladesh	WASH−B (23)	CDI	1109	3353	0.81 (0.65, 1.00)	0.21
Burkina Faso	iLiNS−Zinc (24)	DMC	746	375	0.46 (0.33, 0.64)	0.18
Ghana	GHANA (25)					
Haiti	HAITI (27)	Total sounds	150	149	2.75 (1.28, 5.93)	0.08
Kenya	WASH−B (28)	EASQ	1362	4745	0.95 (0.78, 1.16)	0.21
**I² = 0.80, Tau² = 0.25**			**5475**	**9579**	**0.86 (0.56, 1.33)**	

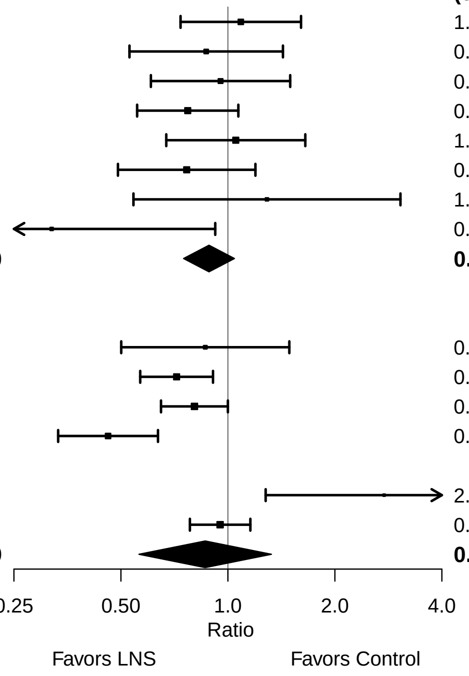

Supplemental figure 6C: Language lowest decile prevalence difference

6C1: Stratified by Geographic region

Geographic region
(p−diff = 0.484)

Country	Trial	Tool	N	N	PD (95% CI)	W
**Geographic region − SEAR**						
Bangladesh	JiVitA−4 (21)	BSID−III	445	143	−0.02 (−0.07, 0.04)	0.16
Bangladesh	RDNS (22)	CDI	1663	814	−0.04 (−0.06, −0.01)	0.38
Bangladesh	WASH−B (23)	CDI	1109	3353	−0.02 (−0.04, 0.00)	0.46
**I² = 0.00, Tau² = 0.00**			**3217**	**4310**	**−0.02 (−0.04, −0.01)**	
**Geographic region − AFR**						
Burkina Faso	iLiNS−Zinc (24)	DMC	746	375	−0.08 (−0.13, −0.04)	0.09
Ghana	GHANA (25)					
Ghana	iLiNS−DYAD−G (26)	CDI	331	658	0.01 (−0.03, 0.05)	0.10
Kenya	WASH−B (28)	EASQ	1362	4745	0.00 (−0.02, 0.01)	0.17
Madagascar	MAHAY (29)	ASQI	1613	1604	0.02 (−0.01, 0.04)	0.16
Malawi	iLiNS−DYAD−M (30)	CDI	215	439	−0.01 (−0.06, 0.03)	0.08
Malawi	iLiNS−DOSE (31)	CDI	645	221	0.00 (−0.05, 0.04)	0.08
Mali	PROMIS CS (32)	DMC	927	944	−0.03 (−0.05, 0.00)	0.14
Zimbabwe	SHINE (HIV−) (33), LNS vs SOC	CDI	381	373	0.01 (−0.04, 0.05)	0.13
Zimbabwe	LNS+WSH vs WSH	CDI	436	408	−0.03 (−0.06, 0.01)	0.13
Zimbabwe	SHINE (HIV+) (34), LNS vs SOC	CDI	66	68	0.03 (−0.08, 0.15)	0.05
Zimbabwe	LNS+WSH vs WSH	CDI	99	79	−0.09 (−0.17, 0.00)	0.05
**I² = 0.63, Tau² = 0.00**			**6821**	**9914**	**−0.01 (−0.03, 0.00)**	

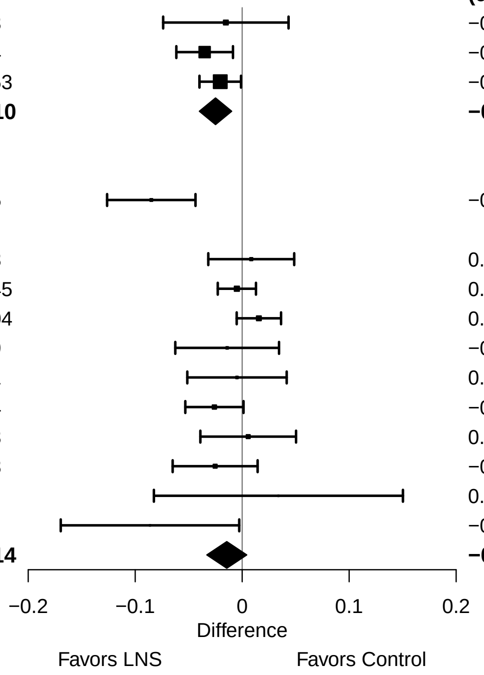

Supplemental figure 6C: Language lowest decile prevalence difference

6C2: Stratified by Stunting burden

Stunting burden
(p−diff = 0.073)

Country	Trial	Tool	N	N	PD (95% CI)	W
**Stunting burden − Less than 35%**						
Ghana	GHANA (25)					
Ghana	iLiNS−DYAD−G (26)	CDI	331	658	0.01 (−0.03, 0.05)	0.26
Haiti	HAITI (27)	Total sounds	150	149	0.10 (0.03, 0.16)	0.16
Kenya	WASH−B (28)	EASQ	1362	4745	0.00 (−0.02, 0.01)	0.35
Malawi	iLiNS−DYAD−M (30)	CDI	215	439	−0.01 (−0.06, 0.03)	0.23
**I² = 0.65, Tau² = 0.00**			**2058**	**5991**	**0.02 (−0.03, 0.06)**	
**Stunting burden − More than 35%**						
Bangladesh	JiVitA−4 (21)	BSID−III	445	143	−0.02 (−0.07, 0.04)	0.08
Bangladesh	RDNS (22)	CDI	1663	814	−0.04 (−0.06, −0.01)	0.13
Bangladesh	WASH−B (23)	CDI	1109	3353	−0.02 (−0.04, 0.00)	0.14
Burkina Faso	iLiNS−Zinc (24)	DMC	746	375	−0.08 (−0.13, −0.04)	0.10
Madagascar	MAHAY (29)	ASQI	1613	1604	0.02 (−0.01, 0.04)	0.14
Malawi	iLiNS−DOSE (31)	CDI	645	221	0.00 (−0.05, 0.04)	0.10
Mali	PROMIS CS (32)	DMC	927	944	−0.03 (−0.05, 0.00)	0.13
Zimbabwe	SHINE (HIV−) (33), LNS vs SOC	CDI	381	373	0.01 (−0.04, 0.05)	0.12
Zimbabwe	LNS+WSH vs WSH	CDI	436	408	−0.03 (−0.06, 0.01)	0.12
Zimbabwe	SHINE (HIV+) (34), LNS vs SOC	CDI	66	68	0.03 (−0.08, 0.15)	0.07
Zimbabwe	LNS+WSH vs WSH	CDI	99	79	−0.09 (−0.17, 0.00)	0.07
**I² = 0.65, Tau² = 0.00**			**8130**	**8382**	**−0.02 (−0.04, 0.00)**	

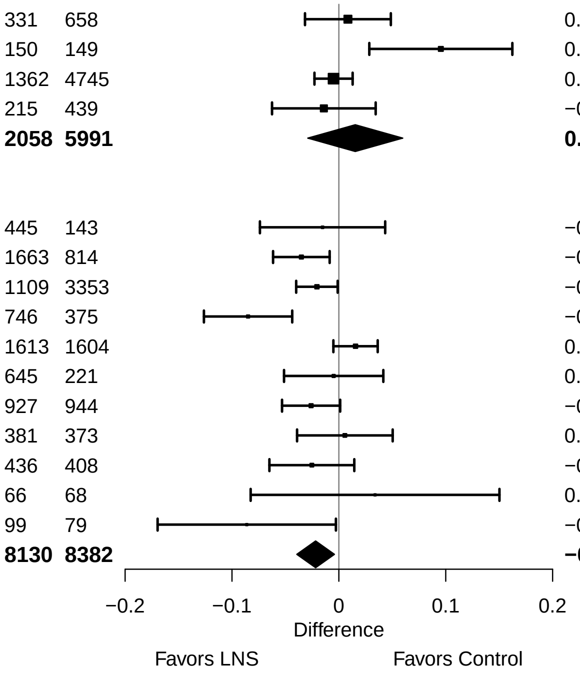

Supplemental figure 6C: Language lowest decile prevalence difference

6C3: Stratified by Malaria prevalence

Malaria prevalence
(p−diff = 0.289)

Country	Trial	Tool	N	N	PD (95% CI)	W
**Malaria prevalence − Less than 10%**						
Bangladesh	JiVitA−4 (21)	BSID−III	445	143	−0.02 (−0.07, 0.04)	0.09
Bangladesh	RDNS (22)	CDI	1663	814	−0.04 (−0.06, −0.01)	0.15
Bangladesh	WASH−B (23)	CDI	1109	3353	−0.02 (−0.04, 0.00)	0.16
Haiti	HAITI (27)	Total sounds	150	149	0.10 (0.03, 0.16)	0.08
Kenya	WASH−B (28)	EASQ	1362	4745	0.00 (−0.02, 0.01)	0.16
Madagascar	MAHAY (29)	ASQI	1613	1604	0.02 (−0.01, 0.04)	0.16
Zimbabwe	SHINE (HIV−) (33), LNS vs SOC	CDI	381	373	0.01 (−0.04, 0.05)	0.14
Zimbabwe	LNS+WSH vs WSH	CDI	436	408	−0.03 (−0.06, 0.01)	0.14
Zimbabwe	SHINE (HIV+) (34), LNS vs SOC	CDI	66	68	0.03 (−0.08, 0.15)	0.07
Zimbabwe	LNS+WSH vs WSH	CDI	99	79	−0.09 (−0.17, 0.00)	0.07
**I² = 0.66, Tau² = 0.00**			**7324**	**11736**	**−0.01 (−0.03, 0.02)**	
**Malaria prevalence − At least 10%**						
Burkina Faso	iLiNS−Zinc (24)	DMC	746	375	−0.08 (−0.13, −0.04)	0.20
Ghana	GHANA (25)					
Ghana	iLiNS−DYAD−G (26)	CDI	331	658	0.01 (−0.03, 0.05)	0.20
Malawi	iLiNS−DYAD−M (30)	CDI	215	439	−0.01 (−0.06, 0.03)	0.18
Malawi	iLiNS−DOSE (31)	CDI	645	221	0.00 (−0.05, 0.04)	0.18
Mali	PROMIS CS (32)	DMC	927	944	−0.03 (−0.05, 0.00)	0.24
**I² = 0.66, Tau² = 0.00**			**2864**	**2637**	**−0.02 (−0.06, 0.01)**	

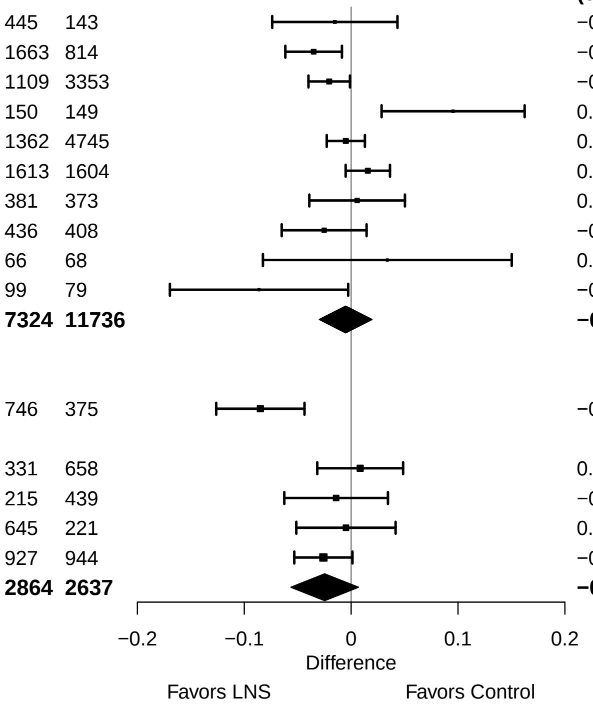

Supplemental figure 6C: Language lowest decile prevalence difference

6C4: Stratified by Anemia prevalence

Anemia prevalence
(p−diff = 0.762)

Country	Trial	Tool	N	N	PD (95% CI)	W
**Anemia prevalence − High**						
Burkina Faso	iLiNS−Zinc (24)	DMC	746	375	−0.08 (−0.13, −0.04)	0.21
Ghana	GHANA (25)					
Haiti	HAITI (27)	Total sounds	150	149	0.10 (0.03, 0.16)	0.14
Malawi	iLiNS−DYAD−M (30)	CDI	215	439	−0.01 (−0.06, 0.03)	0.19
Malawi	iLiNS−DOSE (31)	CDI	645	221	0.00 (−0.05, 0.04)	0.20
Mali	PROMIS CS (32)	DMC	927	944	−0.03 (−0.05, 0.00)	0.26
**I² = 0.81, Tau² = 0.00**			**2683**	**2128**	**−0.01 (−0.07, 0.04)**	
**Anemia prevalence − Moderate**						
Bangladesh	JiVitA−4 (21)	BSID−III	445	143	−0.02 (−0.07, 0.04)	0.08
Bangladesh	RDNS (22)	CDI	1663	814	−0.04 (−0.06, −0.01)	0.14
Bangladesh	WASH−B (23)	CDI	1109	3353	−0.02 (−0.04, 0.00)	0.15
Ghana	iLiNS−DYAD−G (26)	CDI	331	658	0.01 (−0.03, 0.05)	0.11
Kenya	WASH−B (28)	EASQ	1362	4745	0.00 (−0.02, 0.01)	0.15
Madagascar	MAHAY (29)	ASQI	1613	1604	0.02 (−0.01, 0.04)	0.15
Zimbabwe	SHINE (HIV−) (33), LNS vs SOC	CDI	381	373	0.01 (−0.04, 0.05)	0.13
Zimbabwe	LNS+WSH vs WSH	CDI	436	408	−0.03 (−0.06, 0.01)	0.13
Zimbabwe	SHINE (HIV+) (34), LNS vs SOC	CDI	66	68	0.03 (−0.08, 0.15)	0.07
Zimbabwe	LNS+WSH vs WSH	CDI	99	79	−0.09 (−0.17, 0.00)	0.07
**I² = 0.42, Tau² = 0.00**			**7505**	**12245**	**−0.01 (−0.02, 0.00)**	

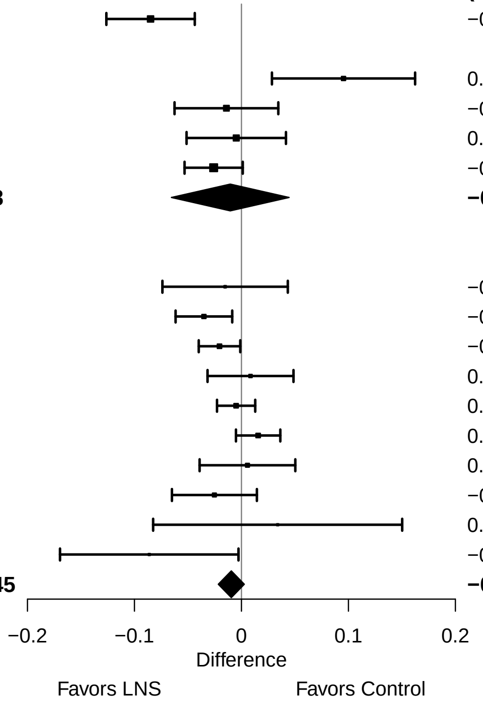

Supplemental figure 6C: Language lowest decile prevalence difference

6C5: Stratified by Source water quality

Source water quality
(p−diff = 0.748)

Country	Trial	Tool	N	N	PD (95% CI)	W
**Source water quality − Improved**						
Bangladesh	JiVitA−4 (21)	BSID−III	445	143	−0.02 (−0.07, 0.04)	0.11
Bangladesh	RDNS (22)	CDI	1663	814	−0.04 (−0.06, −0.01)	0.18
Bangladesh	WASH−B (23)	CDI	548	1099	−0.04 (−0.07, −0.01)	0.18
Ghana	GHANA (25)					
Ghana	iLiNS−DYAD−G (26)	CDI	331	658	0.01 (−0.03, 0.05)	0.15
Haiti	HAITI (27)	Total sounds	150	149	0.10 (0.03, 0.16)	0.10
Malawi	iLiNS−DYAD−M (30)	CDI	215	439	−0.01 (−0.06, 0.03)	0.13
Malawi	iLiNS−DOSE (31)	CDI	645	221	0.00 (−0.05, 0.04)	0.14
**I² = 0.65, Tau² = 0.00**			**3997**	**3523**	**−0.01 (−0.04, 0.02)**	
**Source water quality − Unimproved**						
Burkina Faso	iLiNS−Zinc (24)	DMC	746	375	−0.08 (−0.13, −0.04)	0.17
Kenya	WASH−B (28)	EASQ	649	2073	0.00 (−0.03, 0.02)	0.20
Madagascar	MAHAY (29)	ASQI	1613	1604	0.02 (−0.01, 0.04)	0.22
Mali	PROMIS CS (32)	DMC	927	944	−0.03 (−0.05, 0.00)	0.20
Zimbabwe	SHINE (HIV−) (33)	CDI	381	373	0.01 (−0.04, 0.05)	0.16
Zimbabwe	SHINE (HIV+) (34)	CDI	66	68	0.03 (−0.08, 0.15)	0.05
**I² = 0.76, Tau² = 0.00**			**4382**	**5437**	**−0.01 (−0.05, 0.02)**	

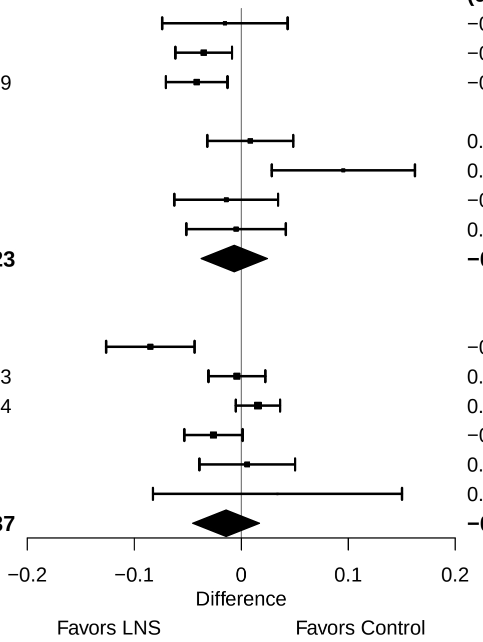

Supplemental figure 6C: Language lowest decile prevalence difference

6C6: Stratified by Sanitation

Sanitation
(p−diff = 0.989)

Country	Trial	Tool	N	N	PD (95% CI)	W
**Sanitation − Improved**						
Bangladesh	JiVitA−4 (21)	BSID−III	445	143	−0.02 (−0.07, 0.04)	0.13
Bangladesh	RDNS (22)	CDI	1663	814	−0.04 (−0.06, −0.01)	0.20
Bangladesh	WASH−B (23)	CDI	548	1099	−0.04 (−0.07, −0.01)	0.20
Ghana	GHANA (25)					
Ghana	iLiNS−DYAD−G (26)	CDI	331	658	0.01 (−0.03, 0.05)	0.17
Haiti	HAITI (27)	Total sounds	150	149	0.10 (0.03, 0.16)	0.11
Mali	PROMIS CS (32)	DMC	927	944	−0.03 (−0.05, 0.00)	0.20
**I² = 0.70, Tau² = 0.00**			**4064**	**3807**	**−0.01 (−0.05, 0.03)**	
**Sanitation − Unimproved**						
Burkina Faso	iLiNS−Zinc (24)	DMC	746	375	−0.08 (−0.13, −0.04)	0.15
Kenya	WASH−B (28)	EASQ	649	2073	0.00 (−0.03, 0.02)	0.18
Madagascar	MAHAY (29)	ASQI	1613	1604	0.02 (−0.01, 0.04)	0.20
Malawi	iLiNS−DYAD−M (30)	CDI	215	439	−0.01 (−0.06, 0.03)	0.14
Malawi	iLiNS−DOSE (31)	CDI	645	221	0.00 (−0.05, 0.04)	0.14
Zimbabwe	SHINE (HIV−) (33)	CDI	381	373	0.01 (−0.04, 0.05)	0.14
Zimbabwe	SHINE (HIV+) (34)	CDI	66	68	0.03 (−0.08, 0.15)	0.05
**I² = 0.68, Tau² = 0.00**			**4315**	**5153**	**−0.01 (−0.04, 0.02)**	

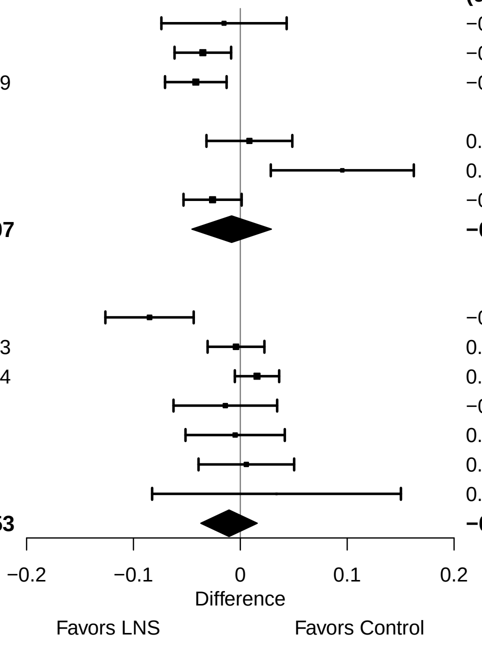

Supplemental figure 6C: Language lowest decile prevalence difference

6C7: Stratified by Supplement duration

Supplement duration
(p−diff = 0.443)

Country	Trial		Tool	N	N	PD (95% CI)	W
**Supplement duration − 12m or less**							
Bangladesh	JiVitA−4 (21)		BSID−III	445	143	−0.02 (−0.07, 0.04)	0.09
Burkina Faso	iLiNS−Zinc (24)		DMC	746	375	−0.08 (−0.13, −0.04)	0.12
Ghana	GHANA (25)						
Ghana	iLiNS−DYAD−G (26)		CDI	331	658	0.01 (−0.03, 0.05)	0.12
Haiti	HAITI (27)		Total sounds	150	149	0.10 (0.03, 0.16)	0.08
Madagascar	MAHAY (29)		ASQI	1613	1604	0.02 (−0.01, 0.04)	0.16
Malawi	iLiNS−DYAD−M (30)		CDI	215	439	−0.01 (−0.06, 0.03)	0.11
Malawi	iLiNS−DOSE (31)		CDI	645	221	0.00 (−0.05, 0.04)	0.11
Zimbabwe	SHINE (HIV−) (33),	LNS vs SOC	CDI	381	373	0.01 (−0.04, 0.05)	0.14
Zimbabwe		LNS+WSH vs WSH	CDI	436	408	−0.03 (−0.06, 0.01)	0.14
Zimbabwe	SHINE (HIV+) (34),	LNS vs SOC	CDI	66	68	0.03 (−0.08, 0.15)	0.08
Zimbabwe		LNS+WSH vs WSH	CDI	99	79	−0.09 (−0.17, 0.00)	0.08
**I² = 0.72, Tau² = 0.00**				**5127**	**4517**	**−0.01 (−0.04, 0.02)**	
**Supplement duration − > 12m**							
Bangladesh	RDNS (22)		CDI	1663	814	−0.04 (−0.06, −0.01)	0.24
Bangladesh	WASH−B (23)		CDI	1109	3353	−0.02 (−0.04, 0.00)	0.26
Kenya	WASH−B (28)		EASQ	1362	4745	0.00 (−0.02, 0.01)	0.26
Mali	PROMIS CS (32)		DMC	927	944	−0.03 (−0.05, 0.00)	0.24
**I² = 0.26, Tau² = 0.00**				**5061**	**9856**	**−0.02 (−0.03, −0.01)**	

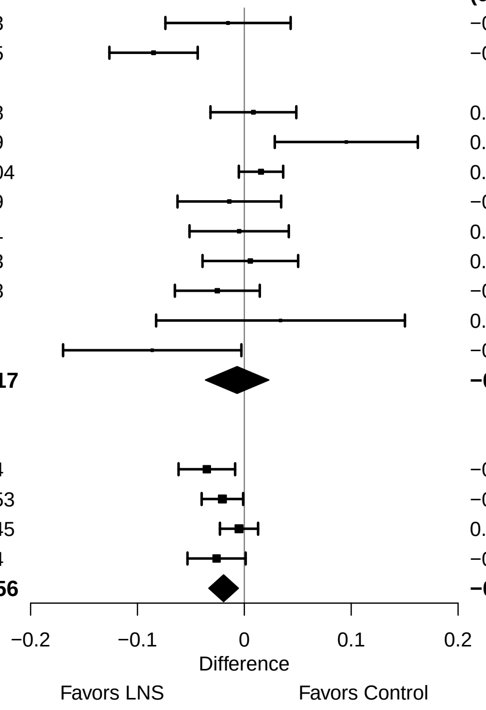

Supplemental figure 6C: Language lowest decile prevalence difference

6C8: Stratified by Frequency of contact

Frequency of contact
(p−diff = 0.340)

Frequency of contact − Monthly

Country	Trial	Tool	N	N	PD (95% CI)	W
Bangladesh	RDNS (22)	CDI	1663	814	−0.04 (−0.06, −0.01)	0.16
Haiti	HAITI (27)	Total sounds	150	149	0.10 (0.03, 0.16)	0.08
Kenya	WASH−B (28)	EASQ	1362	4745	0.00 (−0.02, 0.01)	0.18
Madagascar	MAHAY (29)	ASQI	1613	1604	0.02 (−0.01, 0.04)	0.17
Mali	PROMIS CS (32)	DMC	927	944	−0.03 (−0.05, 0.00)	0.16
Zimbabwe	SHINE (HIV−) (33), LNS vs SOC	CDI	381	373	0.01 (−0.04, 0.05)	0.16
Zimbabwe	LNS+WSH vs WSH	CDI	436	408	−0.03 (−0.06, 0.01)	0.16
Zimbabwe	SHINE (HIV+) (34), LNS vs SOC	CDI	66	68	0.03 (−0.08, 0.15)	0.08
Zimbabwe	LNS+WSH vs WSH	CDI	99	79	−0.09 (−0.17, 0.00)	0.08
**I² = 0.71, Tau² = 0.00**			**6697**	**9184**	**0.00 (−0.03, 0.03)**	

Frequency of contact − Weekly

Country	Trial	Tool	N	N	PD (95% CI)	W
Bangladesh	JiVitA−4 (21)	BSID−III	445	143	−0.02 (−0.07, 0.04)	0.13
Bangladesh	WASH−B (23)	CDI	1109	3353	−0.02 (−0.04, 0.00)	0.23
Burkina Faso	iLiNS−Zinc (24)	DMC	746	375	−0.08 (−0.13, −0.04)	0.17
Ghana	GHANA (25)					
Ghana	iLiNS−DYAD−G (26)	CDI	331	658	0.01 (−0.03, 0.05)	0.17
Malawi	iLiNS−DYAD−M (30)	CDI	215	439	−0.01 (−0.06, 0.03)	0.15
Malawi	iLiNS−DOSE (31)	CDI	645	221	0.00 (−0.05, 0.04)	0.15
**I² = 0.58, Tau² = 0.00**			**3491**	**5189**	**−0.02 (−0.05, 0.00)**	

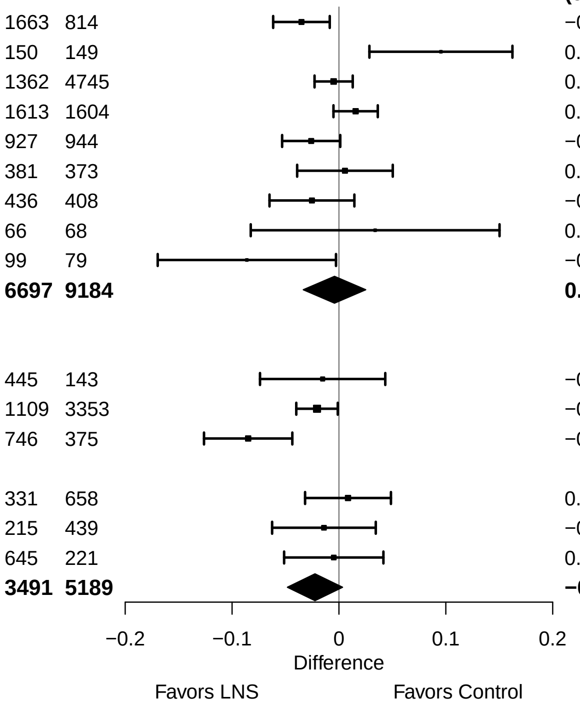

Supplemental figure 6C: Language lowest decile prevalence difference

6C9: Stratified by Average SQ-LNS compliance

Average SQ−LNS compliance
(p−diff = 0.770)

Country	Trial	Tool	N	N	PD (95% CI)	W
**Average SQ−LNS compliance − Low**						
Ghana	iLiNS−DYAD−G (26)	CDI	331	658	0.01 (−0.03, 0.05)	0.17
Malawi	iLiNS−DYAD−M (30)	CDI	215	439	−0.01 (−0.06, 0.03)	0.15
Malawi	iLiNS−DOSE (31)	CDI	645	221	0.00 (−0.05, 0.04)	0.16
Mali	PROMIS CS (32)	DMC	927	944	−0.03 (−0.05, 0.00)	0.21
Zimbabwe	SHINE (HIV−) (33), LNS vs SOC	CDI	381	373	0.01 (−0.04, 0.05)	0.20
Zimbabwe	LNS+WSH vs WSH	CDI	436	408	−0.03 (−0.06, 0.01)	0.20
Zimbabwe	SHINE (HIV+) (34), LNS vs SOC	CDI	66	68	0.03 (−0.08, 0.15)	0.11
Zimbabwe	LNS+WSH vs WSH	CDI	99	79	−0.09 (−0.17, 0.00)	0.11
**I² = 0.00, Tau² = 0.00**			**3100**	**3190**	**−0.01 (−0.03, 0.00)**	
**Average SQ−LNS compliance − High**						
Bangladesh	JiVitA−4 (21)	BSID−III	445	143	−0.02 (−0.07, 0.04)	0.12
Bangladesh	RDNS (22)	CDI	1663	814	−0.04 (−0.06, −0.01)	0.20
Bangladesh	WASH−B (23)	CDI	1109	3353	−0.02 (−0.04, 0.00)	0.21
Burkina Faso	iLiNS−Zinc (24)	DMC	746	375	−0.08 (−0.13, −0.04)	0.16
Ghana	GHANA (25)					
Haiti	HAITI (27)	Total sounds	150	149	0.10 (0.03, 0.16)	0.10
Kenya	WASH−B (28)	EASQ	1362	4745	0.00 (−0.02, 0.01)	0.22
**I² = 0.80, Tau² = 0.00**			**5475**	**9579**	**−0.01 (−0.06, 0.03)**	

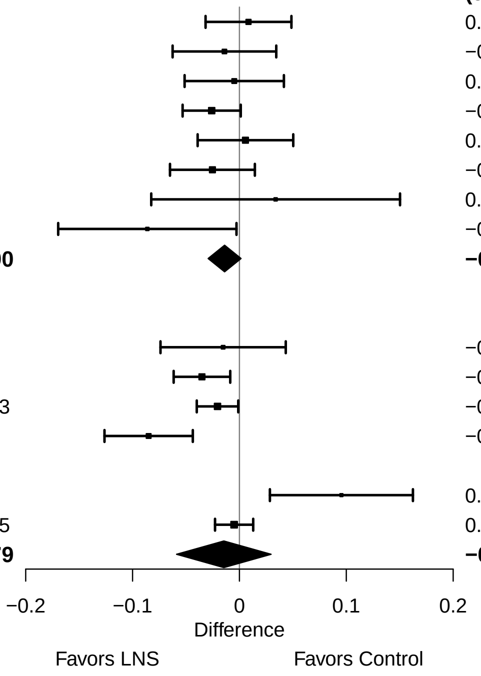

Supplemental figure 6D: Mean difference in social-emotional z-score

6D1: Stratified by Geographic region (insufficient comparisons)

Supplemental figure 6D: Mean difference in social-emotional z-score

6D2: Stratified by Stunting burden

Stunting burden
(p−diff = 0.066)

Country	Trial	Tool	N	N	MD (95% CI)	W
**Stunting burden − Less than 35%**						
Ghana	GHANA (25)					
Ghana	iLiNS−DYAD−G (26)	PSED	332	657	0.01 (−0.12, 0.14)	0.32
Haiti	HAITI (27)					
Kenya	WASH−B (28)	EASQ	1362	4745	0.01 (−0.05, 0.08)	0.41
Malawi	iLiNS−DYAD−M (30)	PSED	215	438	0.01 (−0.15, 0.18)	0.28
**I² = 0.00, Tau² = 0.00**			**1909**	**5840**	**0.01 (−0.04, 0.07)**	
**Stunting burden − More than 35%**						
Bangladesh	JiVitA−4 (21)					
Bangladesh	RDNS (22)	DMC	1657	815	0.06 (−0.04, 0.16)	0.15
Bangladesh	WASH−B (23)	EASQ	1066	3266	0.12 (0.05, 0.20)	0.16
Burkina Faso	iLiNS−Zinc (24)	DMC	746	375	0.36 (0.18, 0.53)	0.11
Madagascar	MAHAY (29)	ASQI	1613	1604	−0.07 (−0.26, 0.11)	0.10
Malawi	iLiNS−DOSE (31)	PSED	644	221	0.03 (−0.12, 0.18)	0.12
Mali	PROMIS CS (32)	DMC	927	944	0.15 (−0.02, 0.31)	0.11
Zimbabwe	SHINE (HIV−) (33), LNS vs SOC	MDAT	395	382	0.07 (−0.07, 0.21)	0.15
Zimbabwe	LNS+WSH vs WSH	MDAT	446	417	0.17 (0.02, 0.31)	0.15
Zimbabwe	SHINE (HIV+) (34), LNS vs SOC	MDAT	67	68	0.24 (0.00, 0.48)	0.10
Zimbabwe	LNS+WSH vs WSH	MDAT	103	83	0.44 (0.14, 0.73)	0.10
**I² = 0.65, Tau² = 0.01**			**7664**	**8175**	**0.13 (0.04, 0.23)**	

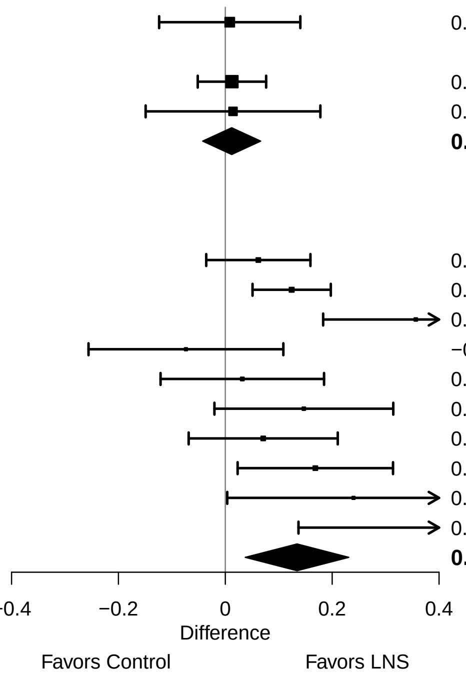

Supplemental figure 6D: Mean difference in social-emotional z-score

6D3: Stratified by Malaria prevalence

Malaria prevalence
(p−diff = 0.863)

Malaria prevalence − Less than 10%

Country	Trial		Tool	N	N	MD (95% CI)	W
Bangladesh	JiVitA−4 (21)						
Bangladesh	RDNS (22)		DMC	1657	815	0.06 (−0.04, 0.16)	0.18
Bangladesh	WASH−B (23)		EASQ	1066	3266	0.12 (0.05, 0.20)	0.20
Haiti	HAITI (27)						
Kenya	WASH−B (28)		EASQ	1362	4745	0.01 (−0.05, 0.08)	0.20
Madagascar	MAHAY (29)		ASQI	1613	1604	−0.07 (−0.26, 0.11)	0.13
Zimbabwe	SHINE (HIV−) (33),	LNS vs SOC	MDAT	395	382	0.07 (−0.07, 0.21)	0.18
Zimbabwe		LNS+WSH vs WSH	MDAT	446	417	0.17 (0.02, 0.31)	0.18
Zimbabwe	SHINE (HIV+) (34),	LNS vs SOC	MDAT	67	68	0.24 (0.00, 0.48)	0.12
Zimbabwe		LNS+WSH vs WSH	MDAT	103	83	0.44 (0.14, 0.73)	0.12
**I² = 0.70, Tau² = 0.01**				**6709**	**11380**	**0.09 (−0.01, 0.20)**	

Malaria prevalence − At least 10%

Country	Trial	Tool	N	N	MD (95% CI)	W
Burkina Faso	iLiNS−Zinc (24)	DMC	746	375	0.36 (0.18, 0.53)	0.19
Ghana	GHANA (25)					
Ghana	iLiNS−DYAD−G (26)	PSED	332	657	0.01 (−0.12, 0.14)	0.22
Malawi	iLiNS−DYAD−M (30)	PSED	215	438	0.01 (−0.15, 0.18)	0.19
Malawi	iLiNS−DOSE (31)	PSED	644	221	0.03 (−0.12, 0.18)	0.20
Mali	PROMIS CS (32)	DMC	927	944	0.15 (−0.02, 0.31)	0.19
**I² = 0.68, Tau² = 0.01**			**2864**	**2635**	**0.11 (−0.02, 0.23)**	

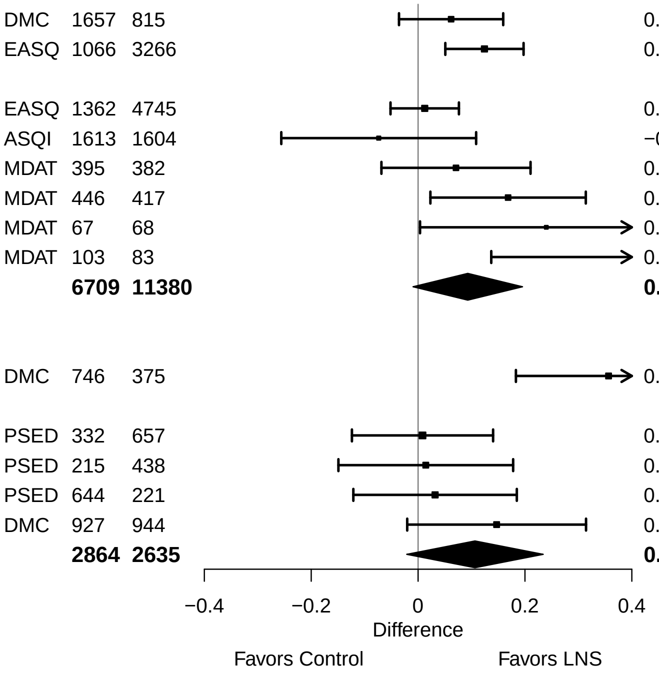

Supplemental figure 6D: Mean difference in social-emotional z-score

6D4: Stratified by Anemia prevalence

Anemia prevalence
(p−diff = 0.496)

Country	Trial	Tool	N	N	MD (95% CI)	W
**Anemia prevalence − High**						
Burkina Faso	iLiNS−Zinc (24)	DMC	746	375	0.36 (0.18, 0.53)	0.24
Ghana	GHANA (25)					
Haiti	HAITI (27)					
Malawi	iLiNS−DYAD−M (30)	PSED	215	438	0.01 (−0.15, 0.18)	0.25
Malawi	iLiNS−DOSE (31)	PSED	644	221	0.03 (−0.12, 0.18)	0.26
Mali	PROMIS CS (32)	DMC	927	944	0.15 (−0.02, 0.31)	0.25
**I² = 0.70, Tau² = 0.02**			**2532**	**1978**	**0.13 (−0.02, 0.29)**	
**Anemia prevalence − Moderate**						
Bangladesh	JiVitA−4 (21)					
Bangladesh	RDNS (22)	DMC	1657	815	0.06 (−0.04, 0.16)	0.16
Bangladesh	WASH−B (23)	EASQ	1066	3266	0.12 (0.05, 0.20)	0.17
Ghana	iLiNS−DYAD−G (26)	PSED	332	657	0.01 (−0.12, 0.14)	0.14
Kenya	WASH−B (28)	EASQ	1362	4745	0.01 (−0.05, 0.08)	0.17
Madagascar	MAHAY (29)	ASQI	1613	1604	−0.07 (−0.26, 0.11)	0.11
Zimbabwe	SHINE (HIV−) (33), LNS vs SOC	MDAT	395	382	0.07 (−0.07, 0.21)	0.15
Zimbabwe	LNS+WSH vs WSH	MDAT	446	417	0.17 (0.02, 0.31)	0.15
Zimbabwe	SHINE (HIV+) (34), LNS vs SOC	MDAT	67	68	0.24 (0.00, 0.48)	0.10
Zimbabwe	LNS+WSH vs WSH	MDAT	103	83	0.44 (0.14, 0.73)	0.10
**I² = 0.66, Tau² = 0.01**			**7041**	**12037**	**0.08 (−0.01, 0.17)**	

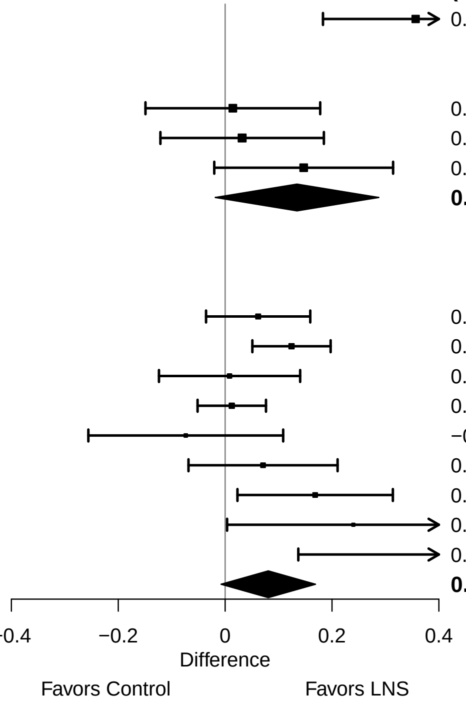

Supplemental figure 6D: Mean difference in social-emotional z-score

6D5: Stratified by Source water quality

Source water quality
(p−diff = 0.681)

Country	Trial	Tool	N	N	MD (95% CI)	W
**Source water quality − Improved**						
Bangladesh	JiVitA−4 (21)					
Bangladesh	RDNS (22)	DMC	1657	815	0.06 (−0.04, 0.16)	0.23
Bangladesh	WASH−B (23)	EASQ	528	1099	0.22 (0.11, 0.33)	0.22
Ghana	GHANA (25)					
Ghana	iLiNS−DYAD−G (26)	PSED	332	657	0.01 (−0.12, 0.14)	0.20
Haiti	HAITI (27)					
Malawi	iLiNS−DYAD−M (30)	PSED	215	438	0.01 (−0.15, 0.18)	0.17
Malawi	iLiNS−DOSE (31)	PSED	644	221	0.03 (−0.12, 0.18)	0.18
**I² = 0.54, Tau² = 0.00**			**3376**	**3230**	**0.08 (0.00, 0.16)**	
**Source water quality − Unimproved**						
Burkina Faso	iLiNS−Zinc (24)	DMC	746	375	0.36 (0.18, 0.53)	0.16
Kenya	WASH−B (28)	EASQ	649	2073	−0.03 (−0.12, 0.07)	0.23
Madagascar	MAHAY (29)	ASQI	1613	1604	−0.07 (−0.26, 0.11)	0.15
Mali	PROMIS CS (32)	DMC	927	944	0.15 (−0.02, 0.31)	0.16
Zimbabwe	SHINE (HIV−) (33)	MDAT	395	382	0.07 (−0.07, 0.21)	0.19
Zimbabwe	SHINE (HIV+) (34)	MDAT	67	68	0.24 (0.00, 0.48)	0.12
**I² = 0.74, Tau² = 0.02**			**4397**	**5446**	**0.11 (−0.02, 0.24)**	

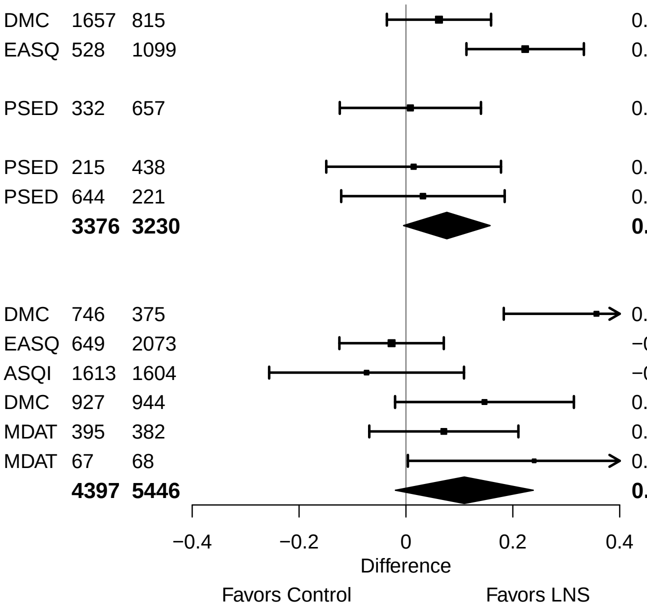

Supplemental figure 6D: Mean difference in social-emotional z-score

6D6: Stratified by Sanitation

Sanitation
(p−diff = 0.660)

**Sanitation − Improved**					**MD**	
**Country**	**Trial**	**Tool**	**N**	**N**	**(95% CI)**	**W**
Bangladesh	JiVitA−4 (21)					
Bangladesh	RDNS (22)	DMC	1657	815	0.06 (−0.04, 0.16)	0.28
Bangladesh	WASH−B (23)	EASQ	528	1099	0.22 (0.11, 0.33)	0.27
Ghana	GHANA (25)					
Ghana	iLiNS−DYAD−G (26)	PSED	332	657	0.01 (−0.12, 0.14)	0.24
Haiti	HAITI (27)					
Mali	PROMIS CS (32)	DMC	927	944	0.15 (−0.02, 0.31)	0.20
**I² = 0.60, Tau² = 0.01**			**3444**	**3515**	**0.11 (0.02, 0.20)**	
**Sanitation − Unimproved**						
Burkina Faso	iLiNS−Zinc (24)	DMC	746	375	0.36 (0.18, 0.53)	0.13
Kenya	WASH−B (28)	EASQ	649	2073	−0.03 (−0.12, 0.07)	0.19
Madagascar	MAHAY (29)	ASQI	1613	1604	−0.07 (−0.26, 0.11)	0.13
Malawi	iLiNS−DYAD−M (30)	PSED	215	438	0.01 (−0.15, 0.18)	0.14
Malawi	iLiNS−DOSE (31)	PSED	644	221	0.03 (−0.12, 0.18)	0.15
Zimbabwe	SHINE (HIV−) (33)	MDAT	395	382	0.07 (−0.07, 0.21)	0.16
Zimbabwe	SHINE (HIV+) (34)	MDAT	67	68	0.24 (0.00, 0.48)	0.10
**I² = 0.68, Tau² = 0.02**			**4329**	**5161**	**0.08 (−0.03, 0.19)**	

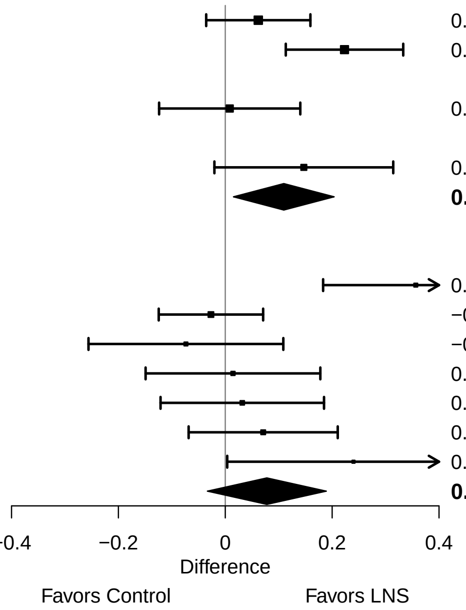

Supplemental figure 6D: Mean difference in social-emotional z-score

6D7: Stratified by Supplement duration

Supplement duration
(p−diff = 0.702)

Country	Trial		Tool	N	N	MD (95% CI)	W
**Supplement duration − 12m or less**							
Bangladesh	JiVitA−4 (21)						
Burkina Faso	iLiNS−Zinc (24)		DMC	746	375	0.36 (0.18, 0.53)	0.13
Ghana	GHANA (25)						
Ghana	iLiNS−DYAD−G (26)		PSED	332	657	0.01 (−0.12, 0.14)	0.16
Haiti	HAITI (27)						
Madagascar	MAHAY (29)		ASQI	1613	1604	−0.07 (−0.26, 0.11)	0.13
Malawi	iLiNS−DYAD−M (30)		PSED	215	438	0.01 (−0.15, 0.18)	0.14
Malawi	iLiNS−DOSE (31)		PSED	644	221	0.03 (−0.12, 0.18)	0.15
Zimbabwe	SHINE (HIV−) (33),	LNS vs SOC	MDAT	395	382	0.07 (−0.07, 0.21)	0.18
Zimbabwe		LNS+WSH vs WSH	MDAT	446	417	0.17 (0.02, 0.31)	0.18
Zimbabwe	SHINE (HIV+) (34),	LNS vs SOC	MDAT	67	68	0.24 (0.00, 0.48)	0.12
Zimbabwe		LNS+WSH vs WSH	MDAT	103	83	0.44 (0.14, 0.73)	0.12
**I² = 0.73, Tau² = 0.02**				**4561**	**4245**	**0.11 (−0.01, 0.24)**	
**Supplement duration − > 12m**							
Bangladesh	RDNS (22)		DMC	1657	815	0.06 (−0.04, 0.16)	0.25
Bangladesh	WASH−B (23)		EASQ	1066	3266	0.12 (0.05, 0.20)	0.27
Kenya	WASH−B (28)		EASQ	1362	4745	0.01 (−0.05, 0.08)	0.28
Mali	PROMIS CS (32)		DMC	927	944	0.15 (−0.02, 0.31)	0.19
**I² = 0.50, Tau² = 0.00**				**5012**	**9770**	**0.07 (0.01, 0.13)**	

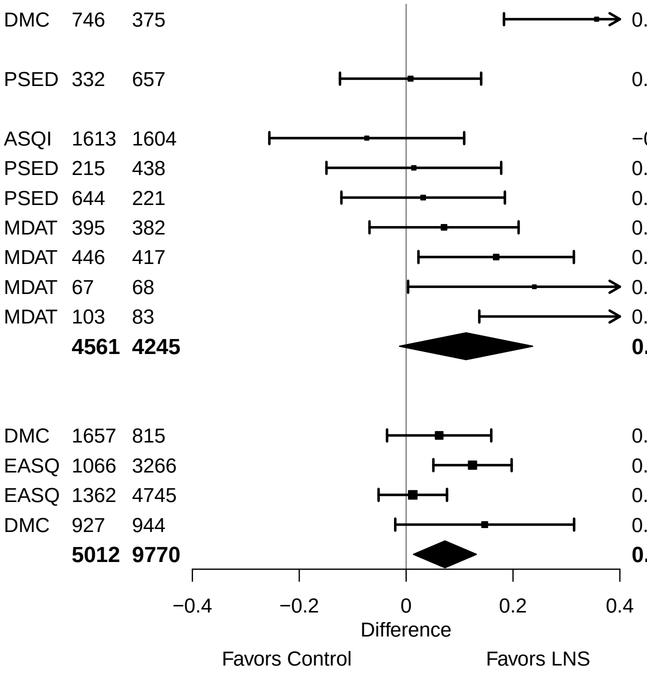

Supplemental figure 6D: Mean difference in social-emotional z-score

6D8: Stratified by Frequency of contact

Frequency of contact
(p−diff = 0.897)

Country	Trial	Tool	N	N	MD (95% CI)	W
**Frequency of contact − Monthly**						
Bangladesh	RDNS (22)	DMC	1657	815	0.06 (−0.04, 0.16)	0.19
Haiti	HAITI (27)					
Kenya	WASH−B (28)	EASQ	1362	4745	0.01 (−0.05, 0.08)	0.21
Madagascar	MAHAY (29)	ASQI	1613	1604	−0.07 (−0.26, 0.11)	0.13
Mali	PROMIS CS (32)	DMC	927	944	0.15 (−0.02, 0.31)	0.14
Zimbabwe	SHINE (HIV−) (33), LNS vs SOC	MDAT	395	382	0.07 (−0.07, 0.21)	0.19
Zimbabwe	LNS+WSH vs WSH	MDAT	446	417	0.17 (0.02, 0.31)	0.19
Zimbabwe	SHINE (HIV+) (34), LNS vs SOC	MDAT	67	68	0.24 (0.00, 0.48)	0.13
Zimbabwe	LNS+WSH vs WSH	MDAT	103	83	0.44 (0.14, 0.73)	0.13
**I² = 0.68, Tau² = 0.01**			**6570**	**9058**	**0.09 (−0.01, 0.20)**	
**Frequency of contact − Weekly**						
Bangladesh	JiVitA−4 (21)					
Bangladesh	WASH−B (23)	EASQ	1066	3266	0.12 (0.05, 0.20)	0.26
Burkina Faso	iLiNS−Zinc (24)	DMC	746	375	0.36 (0.18, 0.53)	0.17
Ghana	GHANA (25)					
Ghana	iLiNS−DYAD−G (26)	PSED	332	657	0.01 (−0.12, 0.14)	0.21
Malawi	iLiNS−DYAD−M (30)	PSED	215	438	0.01 (−0.15, 0.18)	0.18
Malawi	iLiNS−DOSE (31)	PSED	644	221	0.03 (−0.12, 0.18)	0.19
**I² = 0.68, Tau² = 0.01**			**3003**	**4957**	**0.10 (−0.02, 0.23)**	

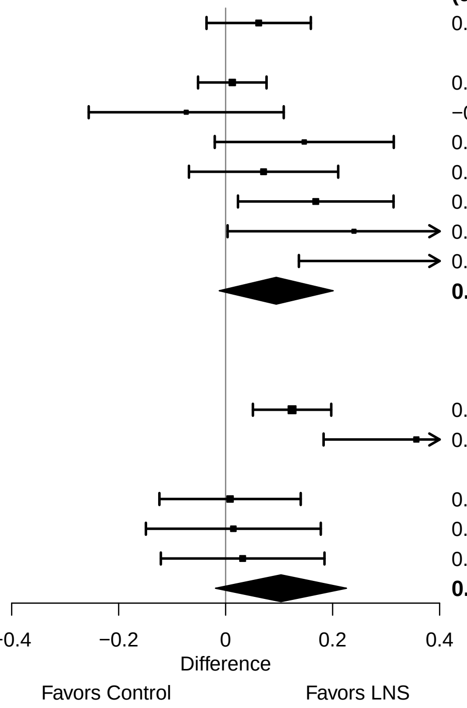

Supplemental figure 6D: Mean difference in social-emotional z-score

6D9: Stratified by Average SQ-LNS compliance

Average SQ−LNS compliance
(p−diff = 0.863)

Country	Trial	Tool	N	N	MD (95% CI)	W
**Average SQ−LNS compliance − Low**						
Ghana	iLiNS−DYAD−G (26)	PSED	332	657	0.01 (−0.12, 0.14)	0.18
Malawi	iLiNS−DYAD−M (30)	PSED	215	438	0.01 (−0.15, 0.18)	0.16
Malawi	iLiNS−DOSE (31)	PSED	644	221	0.03 (−0.12, 0.18)	0.17
Mali	PROMIS CS (32)	DMC	927	944	0.15 (−0.02, 0.31)	0.15
Zimbabwe	SHINE (HIV−) (33), LNS vs SOC	MDAT	395	382	0.07 (−0.07, 0.21)	0.21
Zimbabwe	LNS+WSH vs WSH	MDAT	446	417	0.17 (0.02, 0.31)	0.21
Zimbabwe	SHINE (HIV+) (34), LNS vs SOC	MDAT	67	68	0.24 (0.00, 0.48)	0.13
Zimbabwe	LNS+WSH vs WSH	MDAT	103	83	0.44 (0.14, 0.73)	0.13
**I² = 0.53, Tau² = 0.01**			**3129**	**3210**	**0.10 (0.01, 0.20)**	
**Average SQ−LNS compliance − High**						
Bangladesh	JiVitA−4 (21)					
Bangladesh	RDNS (22)	DMC	1657	815	0.06 (−0.04, 0.16)	0.26
Bangladesh	WASH−B (23)	EASQ	1066	3266	0.12 (0.05, 0.20)	0.28
Burkina Faso	iLiNS−Zinc (24)	DMC	746	375	0.36 (0.18, 0.53)	0.18
Ghana	GHANA (25)					
Haiti	HAITI (27)					
Kenya	WASH−B (28)	EASQ	1362	4745	0.01 (−0.05, 0.08)	0.29
**I² = 0.81, Tau² = 0.02**			**4831**	**9201**	**0.12 (−0.02, 0.26)**	

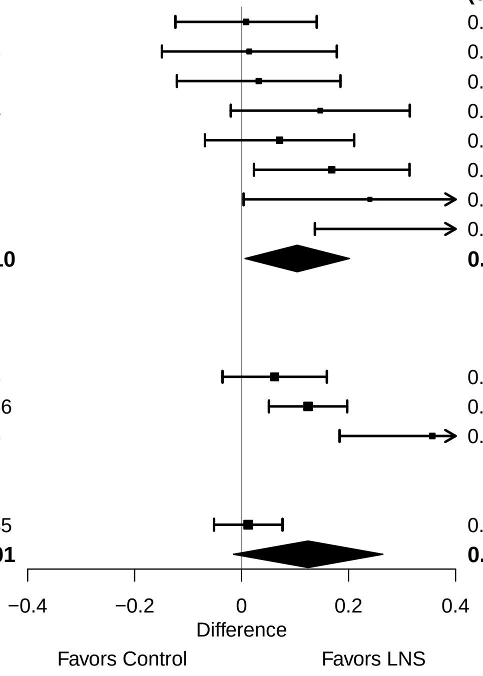

Supplemental figure 6E: Social-emotional lowest decile prevalence ratio

6E1: Stratified by Geographic region (insufficient comparisons)

Supplemental figure 6E: Social-emotional lowest decile prevalence ratio

6E2: Stratified by Stunting burden

Stunting burden
(p−diff = 0.228)

Country	Trial	Tool	N	N	PR (95% CI)	W
**Stunting burden − Less than 35%**						
Ghana	GHANA (25)					
Ghana	iLiNS−DYAD−G (26)	PSED	332	657	0.99 (0.67, 1.47)	0.31
Haiti	HAITI (27)					
Kenya	WASH−B (28)	EASQ	1362	4745	0.87 (0.71, 1.08)	0.44
Malawi	iLiNS−DYAD−M (30)	PSED	215	438	1.00 (0.61, 1.61)	0.26
**I² = 0.00, Tau² = 0.00**			**1909**	**5840**	**0.91 (0.77, 1.08)**	
**Stunting burden − More than 35%**						
Bangladesh	JiVitA−4 (21)					
Bangladesh	RDNS (22)	DMC	1657	815	0.84 (0.66, 1.07)	0.16
Bangladesh	WASH−B (23)	EASQ	1066	3266	0.81 (0.64, 1.03)	0.16
Burkina Faso	iLiNS−Zinc (24)	DMC	746	375	0.41 (0.28, 0.62)	0.12
Madagascar	MAHAY (29)	ASQI	1613	1604	1.15 (0.75, 1.78)	0.11
Malawi	iLiNS−DOSE (31)	PSED	644	221	1.08 (0.68, 1.72)	0.10
Mali	PROMIS CS (32)	DMC	927	944	0.77 (0.56, 1.07)	0.14
Zimbabwe	SHINE (HIV−) (33), LNS vs SOC	MDAT	395	382	0.86 (0.56, 1.33)	0.14
Zimbabwe	LNS+WSH vs WSH	MDAT	446	417	0.62 (0.41, 0.93)	0.14
Zimbabwe	SHINE (HIV+) (34), LNS vs SOC	MDAT	67	68	0.68 (0.22, 2.04)	0.07
Zimbabwe	LNS+WSH vs WSH	MDAT	103	83	0.33 (0.15, 0.76)	0.07
**I² = 0.63, Tau² = 0.08**			**7664**	**8175**	**0.75 (0.59, 0.95)**	

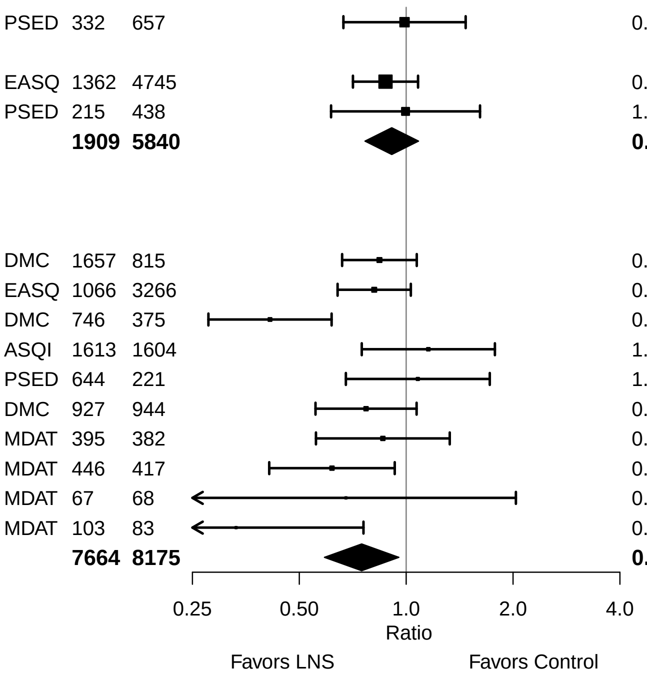

Supplemental figure 6E: Social-emotional lowest decile prevalence ratio

6E3: Stratified by Malaria prevalence

Malaria prevalence
(p−diff = 0.902)

Country	Trial		Tool	N	N	PR (95% CI)	W
**Malaria prevalence − Less than 10%**							
Bangladesh	JiVitA−4 (21)						
Bangladesh	RDNS (22)		DMC	1657	815	0.84 (0.66, 1.07)	0.20
Bangladesh	WASH−B (23)		EASQ	1066	3266	0.81 (0.64, 1.03)	0.20
Haiti	HAITI (27)						
Kenya	WASH−B (28)		EASQ	1362	4745	0.87 (0.71, 1.08)	0.21
Madagascar	MAHAY (29)		ASQI	1613	1604	1.15 (0.75, 1.78)	0.14
Zimbabwe	SHINE (HIV−) (33),	LNS vs SOC	MDAT	395	382	0.86 (0.56, 1.33)	0.18
Zimbabwe		LNS+WSH vs WSH	MDAT	446	417	0.62 (0.41, 0.93)	0.18
Zimbabwe	SHINE (HIV+) (34),	LNS vs SOC	MDAT	67	68	0.68 (0.22, 2.04)	0.08
Zimbabwe		LNS+WSH vs WSH	MDAT	103	83	0.33 (0.15, 0.76)	0.08
**I² = 0.33, Tau² = 0.03**				**6709**	**11380**	**0.81 (0.67, 0.98)**	
**Malaria prevalence − At least 10%**							
Burkina Faso	iLiNS−Zinc (24)		DMC	746	375	0.41 (0.28, 0.62)	0.21
Ghana	GHANA (25)						
Ghana	iLiNS−DYAD−G (26)		PSED	332	657	0.99 (0.67, 1.47)	0.21
Malawi	iLiNS−DYAD−M (30)		PSED	215	438	1.00 (0.61, 1.61)	0.17
Malawi	iLiNS−DOSE (31)		PSED	644	221	1.08 (0.68, 1.72)	0.18
Mali	PROMIS CS (32)		DMC	927	944	0.77 (0.56, 1.07)	0.24
**I² = 0.71, Tau² = 0.11**				**2864**	**2635**	**0.80 (0.56, 1.12)**	

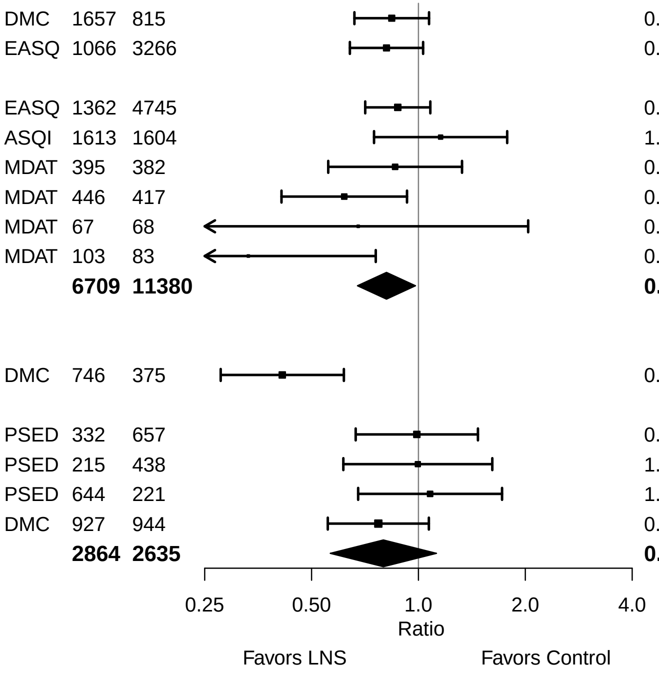

Supplemental figure 6E: Social-emotional lowest decile prevalence ratio

6E4: Stratified by Anemia prevalence

Anemia prevalence
(p−diff = 0.534)

Country	Trial	Tool	N	N	PR (95% CI)	W
**Anemia prevalence − High**						
Burkina Faso	iLiNS−Zinc (24)	DMC	746	375	0.41 (0.28, 0.62)	0.26
Ghana	GHANA (25)					
Haiti	HAITI (27)					
Malawi	iLiNS−DYAD−M (30)	PSED	215	438	1.00 (0.61, 1.61)	0.22
Malawi	iLiNS−DOSE (31)	PSED	644	221	1.08 (0.68, 1.72)	0.22
Mali	PROMIS CS (32)	DMC	927	944	0.77 (0.56, 1.07)	0.30
**I^2 = 0.75, Tau^2 = 0.14**			**2532**	**1978**	**0.76 (0.49, 1.15)**	
**Anemia prevalence − Moderate**						
Bangladesh	JiVitA−4 (21)					
Bangladesh	RDNS (22)	DMC	1657	815	0.84 (0.66, 1.07)	0.17
Bangladesh	WASH−B (23)	EASQ	1066	3266	0.81 (0.64, 1.03)	0.17
Ghana	iLiNS−DYAD−G (26)	PSED	332	657	0.99 (0.67, 1.47)	0.13
Kenya	WASH−B (28)	EASQ	1362	4745	0.87 (0.71, 1.08)	0.18
Madagascar	MAHAY (29)	ASQI	1613	1604	1.15 (0.75, 1.78)	0.12
Zimbabwe	SHINE (HIV−) (33), LNS vs SOC	MDAT	395	382	0.86 (0.56, 1.33)	0.15
Zimbabwe	LNS+WSH vs WSH	MDAT	446	417	0.62 (0.41, 0.93)	0.15
Zimbabwe	SHINE (HIV+) (34), LNS vs SOC	MDAT	67	68	0.68 (0.22, 2.04)	0.07
Zimbabwe	LNS+WSH vs WSH	MDAT	103	83	0.33 (0.15, 0.76)	0.07
**I^2 = 0.27, Tau^2 = 0.02**			**7041**	**12037**	**0.83 (0.71, 0.98)**	

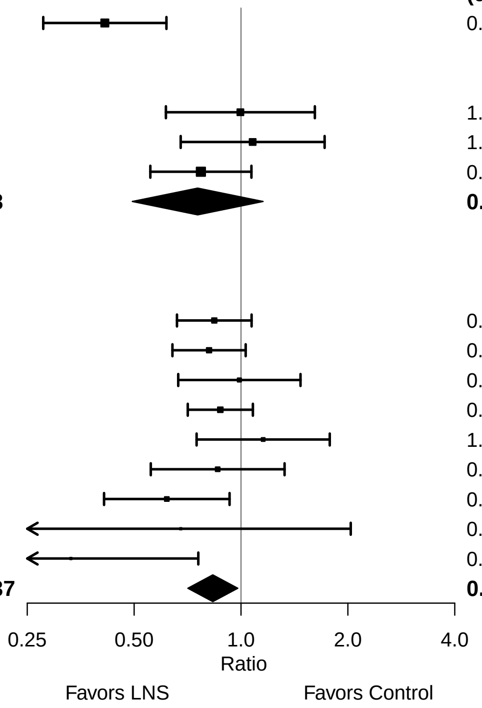

Supplemental figure 6E: Social-emotional lowest decile prevalence ratio

6E5: Stratified by Source water quality

Source water quality
(p−diff = 0.394)

Source water quality − Improved

Country	Trial	Tool	N	N	PR (95% CI)	W
Bangladesh	JiVitA−4 (21)					
Bangladesh	RDNS (22)	DMC	1657	815	0.84 (0.66, 1.07)	0.28
Bangladesh	WASH−B (23)	EASQ	528	1099	0.70 (0.50, 0.99)	0.22
Ghana	GHANA (25)					
Ghana	iLiNS−DYAD−G (26)	PSED	332	657	0.99 (0.67, 1.47)	0.19
Haiti	HAITI (27)					
Malawi	iLiNS−DYAD−M (30)	PSED	215	438	1.00 (0.61, 1.61)	0.15
Malawi	iLiNS−DOSE (31)	PSED	644	221	1.08 (0.68, 1.72)	0.16
**I² = 0.00, Tau² = 0.00**			**3376**	**3230**	**0.87 (0.74, 1.02)**	

Source water quality − Unimproved

Country	Trial	Tool	N	N	PR (95% CI)	W
Burkina Faso	iLiNS−Zinc (24)	DMC	746	375	0.41 (0.28, 0.62)	0.19
Kenya	WASH−B (28)	EASQ	649	2073	0.85 (0.60, 1.21)	0.22
Madagascar	MAHAY (29)	ASQI	1613	1604	1.15 (0.75, 1.78)	0.18
Mali	PROMIS CS (32)	DMC	927	944	0.77 (0.56, 1.07)	0.23
Zimbabwe	SHINE (HIV−) (33)	MDAT	395	382	0.86 (0.56, 1.33)	0.18
Zimbabwe	SHINE (HIV+) (34)	MDAT				
**I² = 0.70, Tau² = 0.10**			**4330**	**5378**	**0.77 (0.55, 1.06)**	

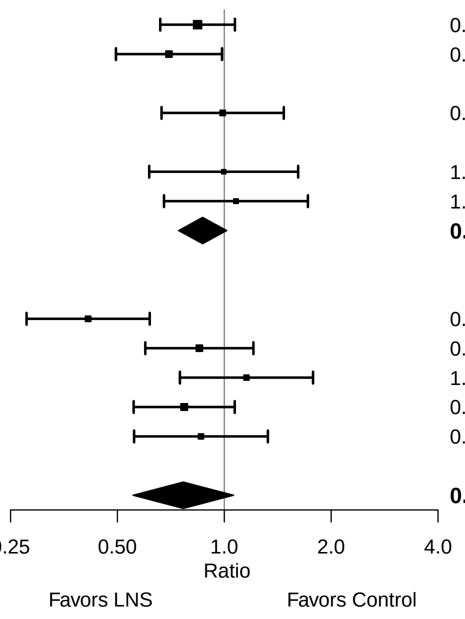

Supplemental figure 6E: Social-emotional lowest decile prevalence ratio

6E6: Stratified by Sanitation

Sanitation
(p−diff = 0.888)

Country	Trial	Tool	N	N	PR (95% CI)	W
**Sanitation − Improved**						
Bangladesh	JiVitA−4 (21)					
Bangladesh	RDNS (22)	DMC	1657	815	0.84 (0.66, 1.07)	0.30
Bangladesh	WASH−B (23)	EASQ	528	1099	0.70 (0.50, 0.99)	0.24
Ghana	GHANA (25)					
Ghana	iLiNS−DYAD−G (26)	PSED	332	657	0.99 (0.67, 1.47)	0.21
Haiti	HAITI (27)					
Mali	PROMIS CS (32)	DMC	927	944	0.77 (0.56, 1.07)	0.25
**I² = 0.00, Tau² = 0.00**			**3444**	**3515**	**0.81 (0.70, 0.95)**	
**Sanitation − Unimproved**						
Burkina Faso	iLiNS−Zinc (24)	DMC	746	375	0.41 (0.28, 0.62)	0.18
Kenya	WASH−B (28)	EASQ	649	2073	0.85 (0.60, 1.21)	0.20
Madagascar	MAHAY (29)	ASQI	1613	1604	1.15 (0.75, 1.78)	0.16
Malawi	iLiNS−DYAD−M (30)	PSED	215	438	1.00 (0.61, 1.61)	0.14
Malawi	iLiNS−DOSE (31)	PSED	644	221	1.08 (0.68, 1.72)	0.15
Zimbabwe	SHINE (HIV−) (33)	MDAT	395	382	0.86 (0.56, 1.33)	0.16
Zimbabwe	SHINE (HIV+) (34)	MDAT				
**I² = 0.68, Tau² = 0.09**			**4262**	**5093**	**0.84 (0.62, 1.14)**	

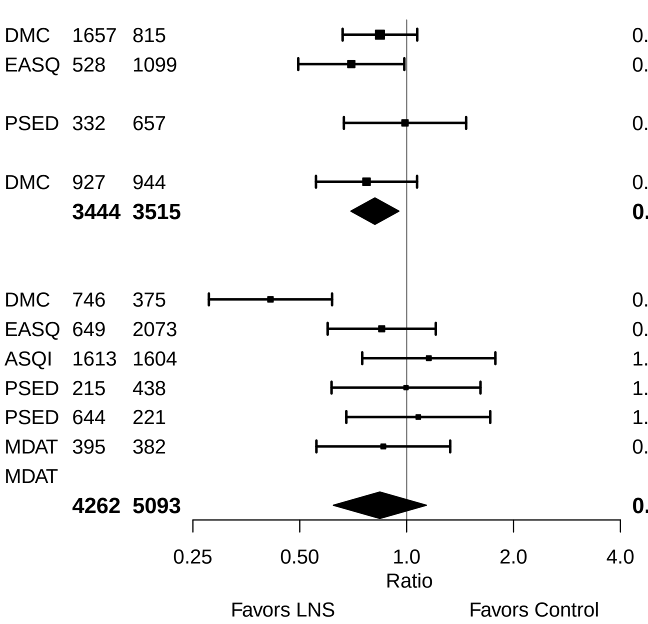

Supplemental figure 6E: Social-emotional lowest decile prevalence ratio

6E7: Stratified by Supplement duration

Supplement duration
(p−diff = 0.731)

Country	Trial	Tool	N	N	PR (95% CI)	W
**Supplement duration − 12m or less**						
Bangladesh	JiVitA−4 (21)					
Burkina Faso	iLiNS−Zinc (24)	DMC	746	375	0.41 (0.28, 0.62)	0.16
Ghana	GHANA (25)					
Ghana	iLiNS−DYAD−G (26)	PSED	332	657	0.99 (0.67, 1.47)	0.16
Haiti	HAITI (27)					
Madagascar	MAHAY (29)	ASQI	1613	1604	1.15 (0.75, 1.78)	0.14
Malawi	iLiNS−DYAD−M (30)	PSED	215	438	1.00 (0.61, 1.61)	0.13
Malawi	iLiNS−DOSE (31)	PSED	644	221	1.08 (0.68, 1.72)	0.13
Zimbabwe	SHINE (HIV−) (33), LNS vs SOC	MDAT	395	382	0.86 (0.56, 1.33)	0.19
Zimbabwe	LNS+WSH vs WSH	MDAT	446	417	0.62 (0.41, 0.93)	0.19
Zimbabwe	SHINE (HIV+) (34), LNS vs SOC	MDAT	67	68	0.68 (0.22, 2.04)	0.09
Zimbabwe	LNS+WSH vs WSH	MDAT	103	83	0.33 (0.15, 0.76)	0.09
**I² = 0.71, Tau² = 0.13**			**4561**	**4245**	**0.78 (0.57, 1.06)**	
**Supplement duration − > 12m**						
Bangladesh	RDNS (22)	DMC	1657	815	0.84 (0.66, 1.07)	0.26
Bangladesh	WASH−B (23)	EASQ	1066	3266	0.81 (0.64, 1.03)	0.26
Kenya	WASH−B (28)	EASQ	1362	4745	0.87 (0.71, 1.08)	0.27
Mali	PROMIS CS (32)	DMC	927	944	0.77 (0.56, 1.07)	0.22
**I² = 0.00, Tau² = 0.00**			**5012**	**9770**	**0.83 (0.74, 0.94)**	

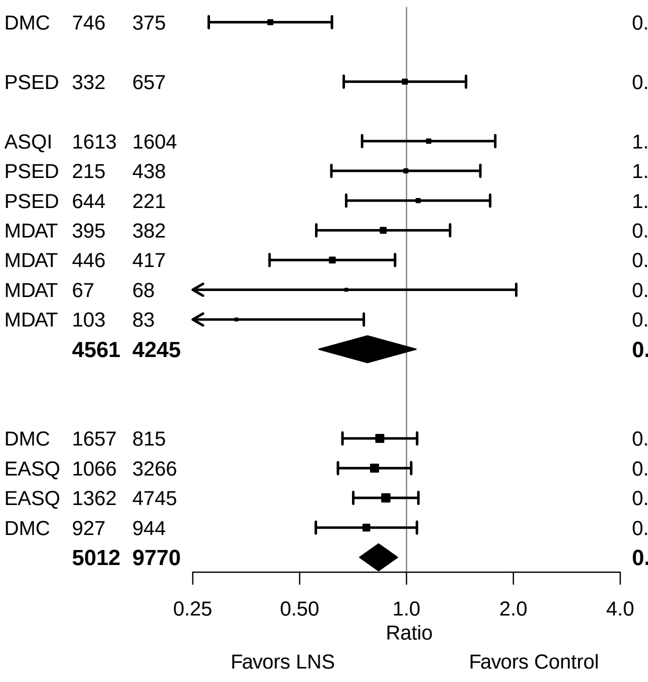

Supplemental figure 6E: Social-emotional lowest decile prevalence ratio

6E8: Stratified by Frequency of contact

Frequency of contact
(p−diff = 0.991)

Country	Trial	Tool	N	N	PR (95% CI)	W
**Frequency of contact − Monthly**						
Bangladesh	RDNS (22)	DMC	1657	815	0.84 (0.66, 1.07)	0.20
Haiti	HAITI (27)					
Kenya	WASH−B (28)	EASQ	1362	4745	0.87 (0.71, 1.08)	0.21
Madagascar	MAHAY (29)	ASQI	1613	1604	1.15 (0.75, 1.78)	0.14
Mali	PROMIS CS (32)	DMC	927	944	0.77 (0.56, 1.07)	0.17
Zimbabwe	SHINE (HIV−) (33), LNS vs SOC	MDAT	395	382	0.86 (0.56, 1.33)	0.18
Zimbabwe	LNS+WSH vs WSH	MDAT	446	417	0.62 (0.41, 0.93)	0.18
Zimbabwe	SHINE (HIV+) (34), LNS vs SOC	MDAT	67	68	0.68 (0.22, 2.04)	0.09
Zimbabwe	LNS+WSH vs WSH	MDAT	103	83	0.33 (0.15, 0.76)	0.09
**I² = 0.34, Tau² = 0.03**			**6570**	**9058**	**0.81 (0.66, 0.98)**	
**Frequency of contact − Weekly**						
Bangladesh	JiVitA−4 (21)					
Bangladesh	WASH−B (23)	EASQ	1066	3266	0.81 (0.64, 1.03)	0.27
Burkina Faso	iLiNS−Zinc (24)	DMC	746	375	0.41 (0.28, 0.62)	0.20
Ghana	GHANA (25)					
Ghana	iLiNS−DYAD−G (26)	PSED	332	657	0.99 (0.67, 1.47)	0.20
Malawi	iLiNS−DYAD−M (30)	PSED	215	438	1.00 (0.61, 1.61)	0.16
Malawi	iLiNS−DOSE (31)	PSED	644	221	1.08 (0.68, 1.72)	0.17
**I² = 0.71, Tau² = 0.11**			**3003**	**4957**	**0.81 (0.57, 1.13)**	

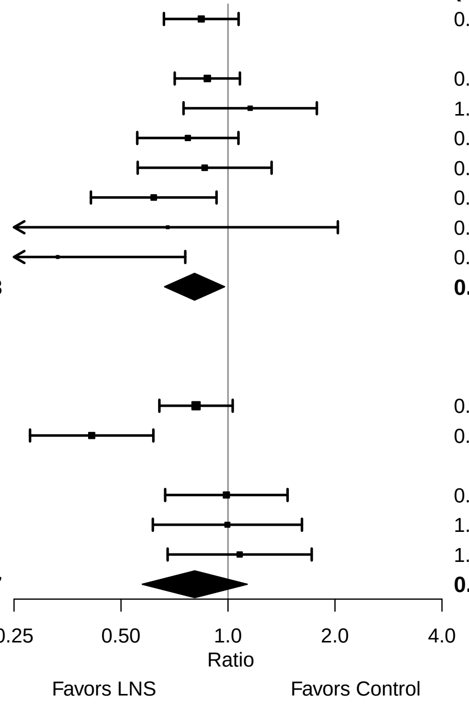

Supplemental figure 6E: Social-emotional lowest decile prevalence ratio

6E9: Stratified by Average SQ-LNS compliance

Average SQ−LNS compliance
(p−diff = 0.575)

Country	Trial		Tool	N	N	PR (95% CI)	W
**Average SQ−LNS compliance − Low**							
Ghana	iLiNS−DYAD−G (26)		PSED	332	657	0.99 (0.67, 1.47)	0.18
Malawi	iLiNS−DYAD−M (30)		PSED	215	438	1.00 (0.61, 1.61)	0.15
Malawi	iLiNS−DOSE (31)		PSED	644	221	1.08 (0.68, 1.72)	0.15
Mali	PROMIS CS (32)		DMC	927	944	0.77 (0.56, 1.07)	0.21
Zimbabwe	SHINE (HIV−) (33),	LNS vs SOC	MDAT	395	382	0.86 (0.56, 1.33)	0.22
Zimbabwe		LNS+WSH vs WSH	MDAT	446	417	0.62 (0.41, 0.93)	0.22
Zimbabwe	SHINE (HIV+) (34),	LNS vs SOC	MDAT	67	68	0.68 (0.22, 2.04)	0.10
Zimbabwe		LNS+WSH vs WSH	MDAT	103	83	0.33 (0.15, 0.76)	0.10
**I² = 0.34, Tau² = 0.03**				**3129**	**3210**	**0.82 (0.65, 1.02)**	
**Average SQ−LNS compliance − High**							
Bangladesh	JiVitA−4 (21)						
Bangladesh	RDNS (22)		DMC	1657	815	0.84 (0.66, 1.07)	0.26
Bangladesh	WASH−B (23)		EASQ	1066	3266	0.81 (0.64, 1.03)	0.27
Burkina Faso	iLiNS−Zinc (24)		DMC	746	375	0.41 (0.28, 0.62)	0.19
Ghana	GHANA (25)						
Haiti	HAITI (27)						
Kenya	WASH−B (28)		EASQ	1362	4745	0.87 (0.71, 1.08)	0.28
**I² = 0.73, Tau² = 0.09**				**4831**	**9201**	**0.73 (0.52, 1.01)**	

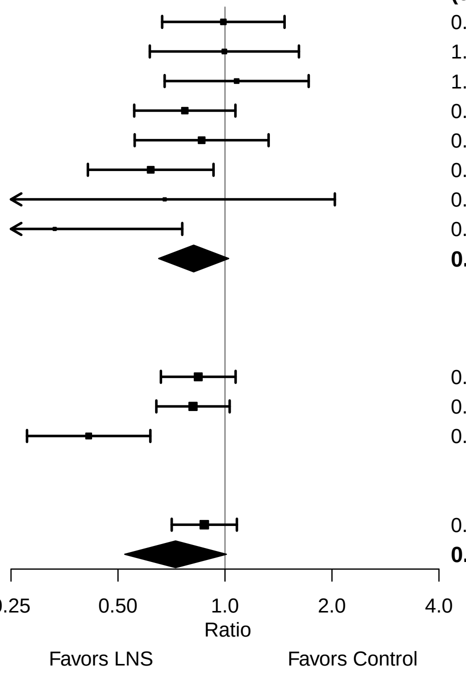

Supplemental figure 6F: Social-emotional lowest decile prevalence difference

6F1: Stratified by Geographic region (insufficient comparisons)

Supplemental figure 6F: Social-emotional lowest decile prevalence difference

6F2: Stratified by Stunting burden

Stunting burden
(p−diff = 0.324)

Country	Trial	Tool	N	N	PD (95% CI)	W
**Stunting burden − Less than 35%**						
Ghana	GHANA (25)					
Ghana	iLiNS−DYAD−G (26)	PSED	332	657	0.00 (−0.04, 0.04)	0.31
Haiti	HAITI (27)					
Kenya	WASH−B (28)	EASQ	1362	4745	−0.01 (−0.03, 0.00)	0.43
Malawi	iLiNS−DYAD−M (30)	PSED	215	438	0.00 (−0.05, 0.05)	0.26
**I² = 0.00, Tau² = 0.00**			**1909**	**5840**	**−0.01 (−0.03, 0.01)**	
**Stunting burden − More than 35%**						
Bangladesh	JiVitA−4 (21)					
Bangladesh	RDNS (22)	DMC	1657	815	−0.02 (−0.04, 0.01)	0.14
Bangladesh	WASH−B (23)	EASQ	1066	3266	−0.02 (−0.04, 0.00)	0.15
Burkina Faso	iLiNS−Zinc (24)	DMC	746	375	−0.10 (−0.14, −0.06)	0.11
Madagascar	MAHAY (29)	ASQI	1613	1604	0.01 (−0.01, 0.04)	0.15
Malawi	iLiNS−DOSE (31)	PSED	644	221	0.01 (−0.04, 0.05)	0.10
Mali	PROMIS CS (32)	DMC	927	944	−0.03 (−0.05, 0.00)	0.14
Zimbabwe	SHINE (HIV−) (33), LNS vs SOC	MDAT	395	382	−0.01 (−0.05, 0.03)	0.13
Zimbabwe	LNS+WSH vs WSH	MDAT	446	417	−0.05 (−0.10, −0.01)	0.13
Zimbabwe	SHINE (HIV+) (34), LNS vs SOC	MDAT	67	68	−0.03 (−0.12, 0.06)	0.07
Zimbabwe	LNS+WSH vs WSH	MDAT	103	83	−0.14 (−0.24, −0.04)	0.07
**I² = 0.77, Tau² = 0.00**			**7664**	**8175**	**−0.03 (−0.05, 0.00)**	

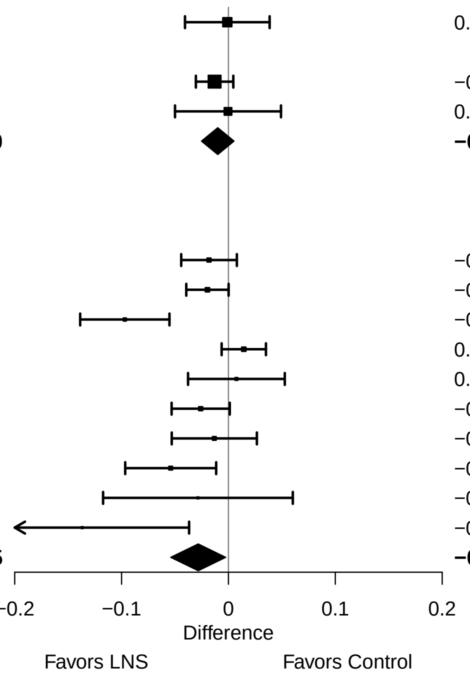

Supplemental figure 6F: Social-emotional lowest decile prevalence difference

6F3: Stratified by Malaria prevalence

Malaria prevalence
(p−diff = 0.802)

Country	Trial		Tool	N	N	PD (95% CI)	W
**Malaria prevalence − Less than 10%**							
Bangladesh	JiVitA−4 (21)						
Bangladesh	RDNS (22)		DMC	1657	815	−0.02 (−0.04, 0.01)	0.18
Bangladesh	WASH−B (23)		EASQ	1066	3266	−0.02 (−0.04, 0.00)	0.19
Haiti	HAITI (27)						
Kenya	WASH−B (28)		EASQ	1362	4745	−0.01 (−0.03, 0.00)	0.20
Madagascar	MAHAY (29)		ASQI	1613	1604	0.01 (−0.01, 0.04)	0.19
Zimbabwe	SHINE (HIV−) (33),	LNS vs SOC	MDAT	395	382	−0.01 (−0.05, 0.03)	0.17
Zimbabwe		LNS+WSH vs WSH	MDAT	446	417	−0.05 (−0.10, −0.01)	0.17
Zimbabwe	SHINE (HIV+) (34),	LNS vs SOC	MDAT	67	68	−0.03 (−0.12, 0.06)	0.08
Zimbabwe		LNS+WSH vs WSH	MDAT	103	83	−0.14 (−0.24, −0.04)	0.08
**I² = 0.64, Tau² = 0.00**				**6709**	**11380**	**−0.02 (−0.04, 0.00)**	
**Malaria prevalence − At least 10%**							
Burkina Faso	iLiNS−Zinc (24)		DMC	746	375	−0.10 (−0.14, −0.06)	0.20
Ghana	GHANA (25)						
Ghana	iLiNS−DYAD−G (26)		PSED	332	657	0.00 (−0.04, 0.04)	0.20
Malawi	iLiNS−DYAD−M (30)		PSED	215	438	0.00 (−0.05, 0.05)	0.17
Malawi	iLiNS−DOSE (31)		PSED	644	221	0.01 (−0.04, 0.05)	0.18
Mali	PROMIS CS (32)		DMC	927	944	−0.03 (−0.05, 0.00)	0.25
**I² = 0.75, Tau² = 0.00**				**2864**	**2635**	**−0.02 (−0.06, 0.01)**	

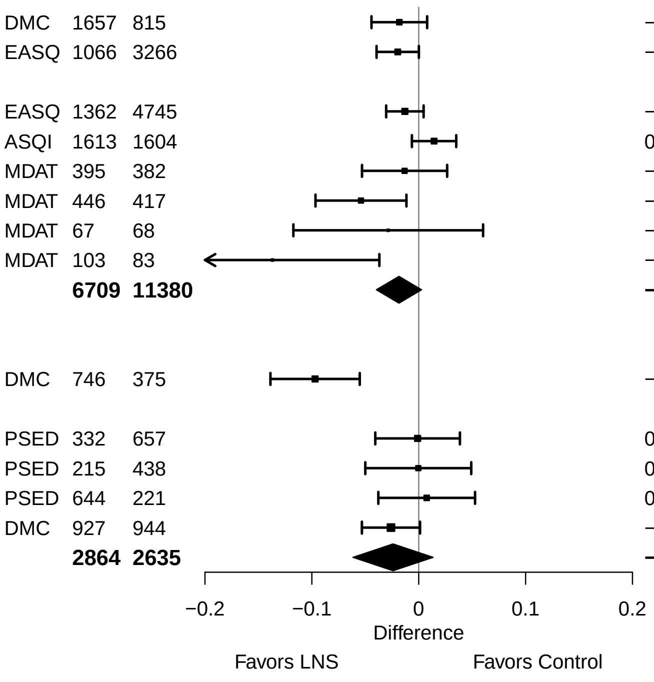

Supplemental figure 6F: Social-emotional lowest decile prevalence difference

6F4: Stratified by Anemia prevalence

Anemia prevalence
(p−diff = 0.492)

Country	Trial	Tool	N	N	PD (95% CI)	W
**Anemia prevalence − High**						
Burkina Faso	iLiNS−Zinc (24)	DMC	746	375	−0.10 (−0.14, −0.06)	0.24
Ghana	GHANA (25)					
Haiti	HAITI (27)					
Malawi	iLiNS−DYAD−M (30)	PSED	215	438	0.00 (−0.05, 0.05)	0.21
Malawi	iLiNS−DOSE (31)	PSED	644	221	0.01 (−0.04, 0.05)	0.23
Mali	PROMIS CS (32)	DMC	927	944	−0.03 (−0.05, 0.00)	0.31
**I² = 0.78, Tau² = 0.00**			**2532**	**1978**	**−0.03 (−0.08, 0.02)**	
**Anemia prevalence − Moderate**						
Bangladesh	JiVitA−4 (21)					
Bangladesh	RDNS (22)	DMC	1657	815	−0.02 (−0.04, 0.01)	0.15
Bangladesh	WASH−B (23)	EASQ	1066	3266	−0.02 (−0.04, 0.00)	0.17
Ghana	iLiNS−DYAD−G (26)	PSED	332	657	0.00 (−0.04, 0.04)	0.12
Kenya	WASH−B (28)	EASQ	1362	4745	−0.01 (−0.03, 0.00)	0.17
Madagascar	MAHAY (29)	ASQI	1613	1604	0.01 (−0.01, 0.04)	0.17
Zimbabwe	SHINE (HIV−) (33), LNS vs SOC	MDAT	395	382	−0.01 (−0.05, 0.03)	0.15
Zimbabwe	LNS+WSH vs WSH	MDAT	446	417	−0.05 (−0.10, −0.01)	0.15
Zimbabwe	SHINE (HIV+) (34), LNS vs SOC	MDAT	67	68	−0.03 (−0.12, 0.06)	0.07
Zimbabwe	LNS+WSH vs WSH	MDAT	103	83	−0.14 (−0.24, −0.04)	0.07
**I² = 0.58, Tau² = 0.00**			**7041**	**12037**	**−0.02 (−0.03, 0.00)**	

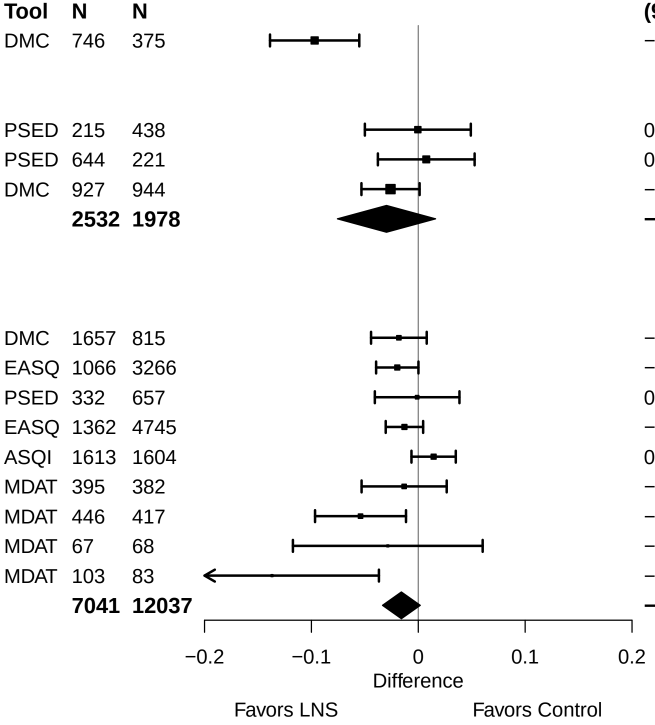

Supplemental figure 6F: Social-emotional lowest decile prevalence difference

6F5: Stratified by Source water quality

Source water quality
(p−diff = 0.587)

Source water quality − Improved

**Country**	**Trial**	**Tool**	**N**	**N**	**PD (95% CI)**	**W**
Bangladesh	JiVitA−4 (21)					
Bangladesh	RDNS (22)	DMC	1657	815	−0.02 (−0.04, 0.01)	0.25
Bangladesh	WASH−B (23)	EASQ	528	1099	−0.04 (−0.07, −0.01)	0.22
Ghana	GHANA (25)					
Ghana	iLiNS−DYAD−G (26)	PSED	332	657	0.00 (−0.04, 0.04)	0.19
Haiti	HAITI (27)					
Malawi	iLiNS−DYAD−M (30)	PSED	215	438	0.00 (−0.05, 0.05)	0.16
Malawi	iLiNS−DOSE (31)	PSED	644	221	0.01 (−0.04, 0.05)	0.17
**I² = 0.07, Tau² = 0.00**			**3376**	**3230**	**−0.02 (−0.03, 0.00)**	

Source water quality − Unimproved

**Country**	**Trial**	**Tool**	**N**	**N**	**PD (95% CI)**	**W**
Burkina Faso	iLiNS−Zinc (24)	DMC	746	375	−0.10 (−0.14, −0.06)	0.16
Kenya	WASH−B (28)	EASQ	649	2073	−0.01 (−0.04, 0.01)	0.22
Madagascar	MAHAY (29)	ASQI	1613	1604	0.01 (−0.01, 0.04)	0.24
Mali	PROMIS CS (32)	DMC	927	944	−0.03 (−0.05, 0.00)	0.21
Zimbabwe	SHINE (HIV−) (33)	MDAT	395	382	−0.01 (−0.05, 0.03)	0.17
Zimbabwe	SHINE (HIV+) (34)	MDAT				
**I² = 0.83, Tau² = 0.00**			**4330**	**5378**	**−0.03 (−0.06, 0.01)**	

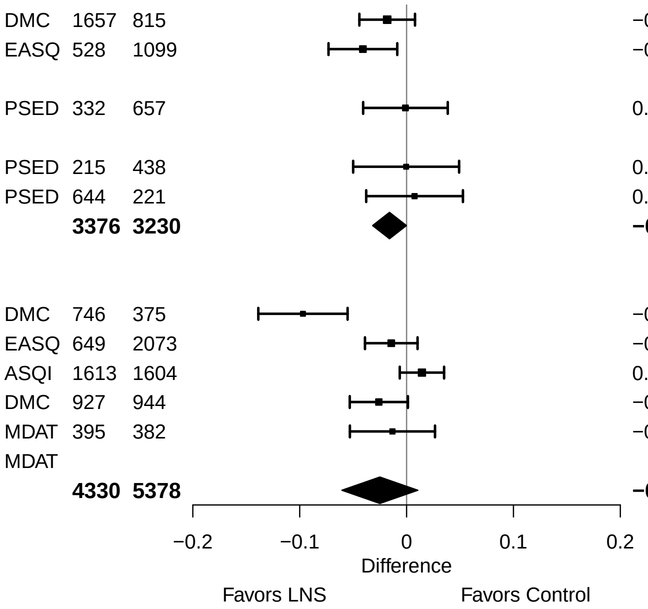

Supplemental figure 6F: Social-emotional lowest decile prevalence difference

6F6: Stratified by Sanitation

Sanitation
(p−diff = 0.739)

Country	Trial	Tool	N	N	PD (95% CI)	W
**Sanitation − Improved**						
Bangladesh	JiVitA−4 (21)					
Bangladesh	RDNS (22)	DMC	1657	815	−0.02 (−0.04, 0.01)	0.27
Bangladesh	WASH−B (23)	EASQ	528	1099	−0.04 (−0.07, −0.01)	0.25
Ghana	GHANA (25)					
Ghana	iLiNS−DYAD−G (26)	PSED	332	657	0.00 (−0.04, 0.04)	0.21
Haiti	HAITI (27)					
Mali	PROMIS CS (32)	DMC	927	944	−0.03 (−0.05, 0.00)	0.27
**I² = 0.00, Tau² = 0.00**			**3444**	**3515**	**−0.02 (−0.04, −0.01)**	
**Sanitation − Unimproved**						
Burkina Faso	iLiNS−Zinc (24)	DMC	746	375	−0.10 (−0.14, −0.06)	0.15
Kenya	WASH−B (28)	EASQ	649	2073	−0.01 (−0.04, 0.01)	0.21
Madagascar	MAHAY (29)	ASQI	1613	1604	0.01 (−0.01, 0.04)	0.22
Malawi	iLiNS−DYAD−M (30)	PSED	215	438	0.00 (−0.05, 0.05)	0.13
Malawi	iLiNS−DOSE (31)	PSED	644	221	0.01 (−0.04, 0.05)	0.14
Zimbabwe	SHINE (HIV−) (33)	MDAT	395	382	−0.01 (−0.05, 0.03)	0.16
Zimbabwe	SHINE (HIV+) (34)	MDAT				
**I² = 0.78, Tau² = 0.00**			**4262**	**5093**	**−0.02 (−0.05, 0.02)**	

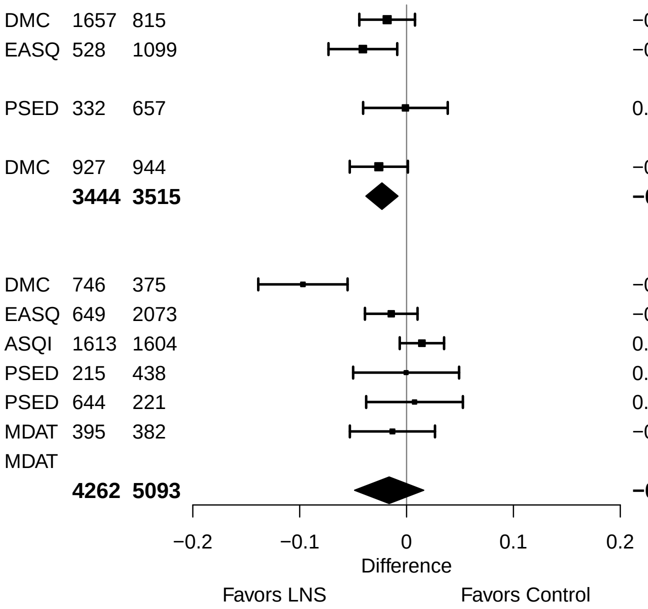

Supplemental figure 6F: Social-emotional lowest decile prevalence difference

6F7: Stratified by Supplement duration

Supplement duration
(p−diff = 0.815)

Country	Trial	Tool	N	N	PD (95% CI)	W
**Supplement duration − 12m or less**						
Bangladesh	JiVitA−4 (21)					
Burkina Faso	iLiNS−Zinc (24)	DMC	746	375	−0.10 (−0.14, −0.06)	0.14
Ghana	GHANA (25)					
Ghana	iLiNS−DYAD−G (26)	PSED	332	657	0.00 (−0.04, 0.04)	0.15
Haiti	HAITI (27)					
Madagascar	MAHAY (29)	ASQI	1613	1604	0.01 (−0.01, 0.04)	0.20
Malawi	iLiNS−DYAD−M (30)	PSED	215	438	0.00 (−0.05, 0.05)	0.12
Malawi	iLiNS−DOSE (31)	PSED	644	221	0.01 (−0.04, 0.05)	0.13
Zimbabwe	SHINE (HIV−) (33), LNS vs SOC	MDAT	395	382	−0.01 (−0.05, 0.03)	0.17
Zimbabwe	LNS+WSH vs WSH	MDAT	446	417	−0.05 (−0.10, −0.01)	0.17
Zimbabwe	SHINE (HIV+) (34), LNS vs SOC	MDAT	67	68	−0.03 (−0.12, 0.06)	0.09
Zimbabwe	LNS+WSH vs WSH	MDAT	103	83	−0.14 (−0.24, −0.04)	0.09
**I² = 0.80, Tau² = 0.00**			**4561**	**4245**	**−0.03 (−0.06, 0.01)**	
**Supplement duration − > 12m**						
Bangladesh	RDNS (22)	DMC	1657	815	−0.02 (−0.04, 0.01)	0.24
Bangladesh	WASH−B (23)	EASQ	1066	3266	−0.02 (−0.04, 0.00)	0.26
Kenya	WASH−B (28)	EASQ	1362	4745	−0.01 (−0.03, 0.00)	0.27
Mali	PROMIS CS (32)	DMC	927	944	−0.03 (−0.05, 0.00)	0.23
**I² = 0.00, Tau² = 0.00**			**5012**	**9770**	**−0.02 (−0.03, −0.01)**	

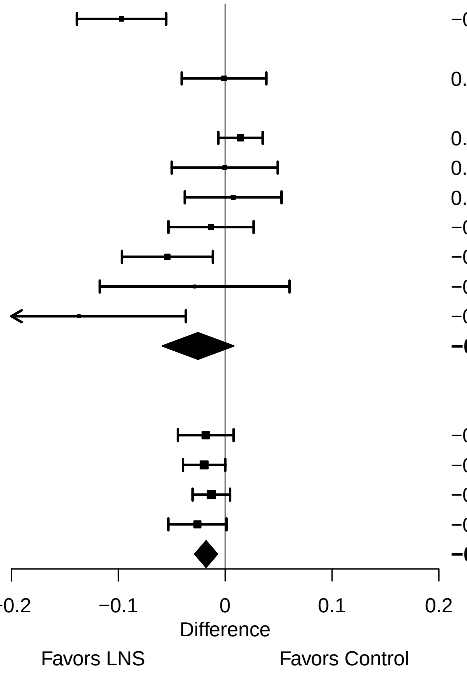

Supplemental figure 6F: Social-emotional lowest decile prevalence difference

6F8: Stratified by Frequency of contact

Frequency of contact
(p−diff = 0.919)

Country	Trial	Tool	N	N	PD (95% CI)	W
**Frequency of contact − Monthly**						
Bangladesh	RDNS (22)	DMC	1657	815	−0.02 (−0.04, 0.01)	0.18
Haiti	HAITI (27)					
Kenya	WASH−B (28)	EASQ	1362	4745	−0.01 (−0.03, 0.00)	0.20
Madagascar	MAHAY (29)	ASQI	1613	1604	0.01 (−0.01, 0.04)	0.19
Mali	PROMIS CS (32)	DMC	927	944	−0.03 (−0.05, 0.00)	0.18
Zimbabwe	SHINE (HIV−) (33), LNS vs SOC	MDAT	395	382	−0.01 (−0.05, 0.03)	0.17
Zimbabwe	LNS+WSH vs WSH	MDAT	446	417	−0.05 (−0.10, −0.01)	0.17
Zimbabwe	SHINE (HIV+) (34), LNS vs SOC	MDAT	67	68	−0.03 (−0.12, 0.06)	0.08
Zimbabwe	LNS+WSH vs WSH	MDAT	103	83	−0.14 (−0.24, −0.04)	0.08
**I² = 0.65, Tau² = 0.00**			**6570**	**9058**	**−0.02 (−0.04, 0.00)**	
**Frequency of contact − Weekly**						
Bangladesh	JiVitA−4 (21)					
Bangladesh	WASH−B (23)	EASQ	1066	3266	−0.02 (−0.04, 0.00)	0.27
Burkina Faso	iLiNS−Zinc (24)	DMC	746	375	−0.10 (−0.14, −0.06)	0.19
Ghana	GHANA (25)					
Ghana	iLiNS−DYAD−G (26)	PSED	332	657	0.00 (−0.04, 0.04)	0.20
Malawi	iLiNS−DYAD−M (30)	PSED	215	438	0.00 (−0.05, 0.05)	0.16
Malawi	iLiNS−DOSE (31)	PSED	644	221	0.01 (−0.04, 0.05)	0.18
**I² = 0.75, Tau² = 0.00**			**3003**	**4957**	**−0.02 (−0.06, 0.01)**	

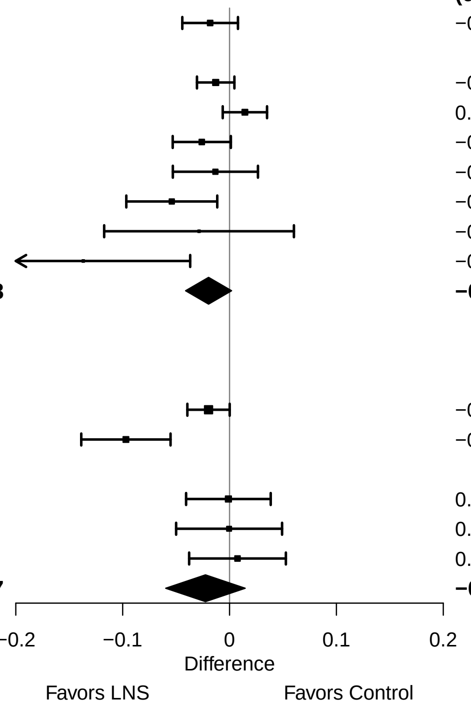

Supplemental figure 6F: Social-emotional lowest decile prevalence difference

6F9: Stratified by Average SQ-LNS compliance

Average SQ−LNS compliance
(p−diff = 0.563)

Country	Trial	Tool	N	N	PD (95% CI)	W
**Average SQ−LNS compliance − Low**						
Ghana	iLiNS−DYAD−G (26)	PSED	332	657	0.00 (−0.04, 0.04)	0.17
Malawi	iLiNS−DYAD−M (30)	PSED	215	438	0.00 (−0.05, 0.05)	0.14
Malawi	iLiNS−DOSE (31)	PSED	644	221	0.01 (−0.04, 0.05)	0.16
Mali	PROMIS CS (32)	DMC	927	944	−0.03 (−0.05, 0.00)	0.22
Zimbabwe	SHINE (HIV−) (33), LNS vs SOC	MDAT	395	382	−0.01 (−0.05, 0.03)	0.21
Zimbabwe	LNS+WSH vs WSH	MDAT	446	417	−0.05 (−0.10, −0.01)	0.21
Zimbabwe	SHINE (HIV+) (34), LNS vs SOC	MDAT	67	68	−0.03 (−0.12, 0.06)	0.10
Zimbabwe	LNS+WSH vs WSH	MDAT	103	83	−0.14 (−0.24, −0.04)	0.10
**I² = 0.35, Tau² = 0.00**			**3129**	**3210**	**−0.02 (−0.04, 0.00)**	
**Average SQ−LNS compliance − High**						
Bangladesh	JiVitA−4 (21)					
Bangladesh	RDNS (22)	DMC	1657	815	−0.02 (−0.04, 0.01)	0.25
Bangladesh	WASH−B (23)	EASQ	1066	3266	−0.02 (−0.04, 0.00)	0.28
Burkina Faso	iLiNS−Zinc (24)	DMC	746	375	−0.10 (−0.14, −0.06)	0.19
Ghana	GHANA (25)					
Haiti	HAITI (27)					
Kenya	WASH−B (28)	EASQ	1362	4745	−0.01 (−0.03, 0.00)	0.28
**I² = 0.78, Tau² = 0.00**			**4831**	**9201**	**−0.03 (−0.07, 0.00)**	

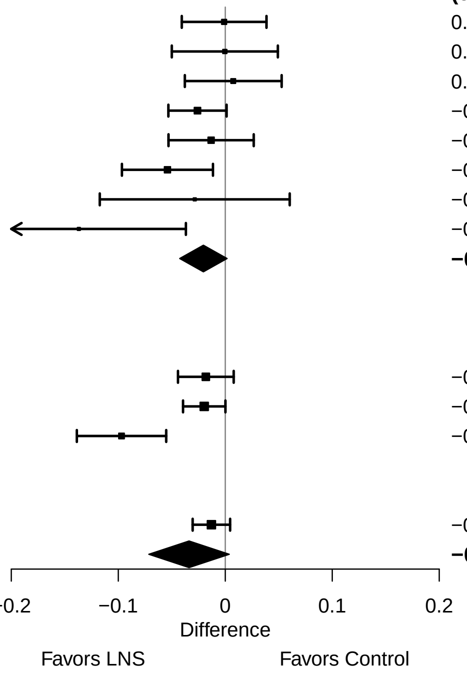

Supplemental figure 6G: Mean difference in motor z-score

6G1: Stratified by Geographic region

Geographic region
(p−diff = 0.591)

Geographic region − SEAR

**Country**	**Trial**	**Tool**	**N**	**N**	**MD (95% CI)**	**W**
Bangladesh	JiVitA−4 (21)	BSID−III	445	143	−0.02 (−0.17, 0.14)	0.25
Bangladesh	RDNS (22)	DMC	1556	753	0.10 (0.03, 0.17)	0.38
Bangladesh	WASH−B (23)	EASQ	1074	3279	0.10 (0.04, 0.17)	0.38
**I² = 0.05, Tau² = 0.00**			**3075**	**4175**	**0.09 (0.04, 0.14)**	

Geographic region − AFR

Burkina Faso	iLiNS−Zinc (24)	DMC	746	375	0.40 (0.24, 0.57)	0.10
Ghana	GHANA (25)					
Ghana	iLiNS−DYAD−G (26)	KDI	302	601	0.03 (−0.11, 0.16)	0.12
Kenya	WASH−B (28)	EASQ	1362	4745	0.01 (−0.05, 0.07)	0.17
Madagascar	MAHAY (29)	ASQI	1613	1604	0.07 (−0.13, 0.27)	0.08
Malawi	iLiNS−DYAD−M (30)	KDI	214	436	0.03 (−0.13, 0.18)	0.11
Malawi	iLiNS−DOSE (31)	KDI	646	221	0.05 (−0.10, 0.20)	0.11
Mali	PROMIS CS (32)	DMC	902	921	0.13 (−0.04, 0.30)	0.10
Zimbabwe	SHINE (HIV−) (33), LNS vs SOC	MDAT	395	382	0.00 (−0.16, 0.15)	0.14
Zimbabwe	LNS+WSH vs WSH	MDAT	446	417	0.20 (0.05, 0.35)	0.14
Zimbabwe	SHINE (HIV+) (34), LNS vs SOC	MDAT	67	68	0.04 (−0.30, 0.39)	0.08
Zimbabwe	LNS+WSH vs WSH	MDAT	103	83	0.49 (0.23, 0.74)	0.08
**I² = 0.68, Tau² = 0.01**			**6796**	**9853**	**0.11 (0.03, 0.20)**	

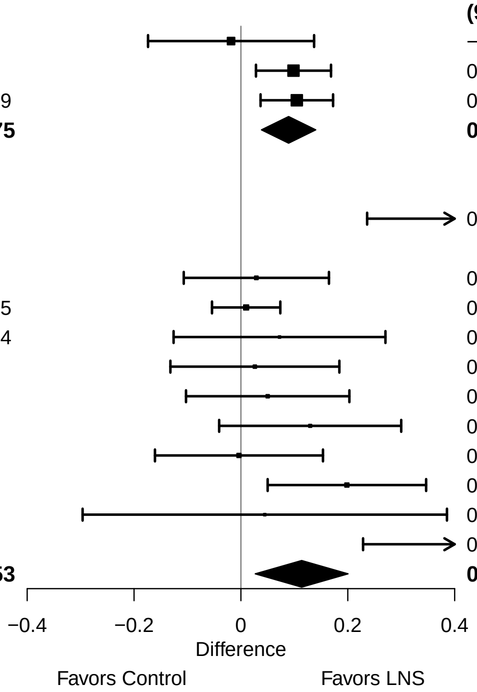

Supplemental figure 6G: Mean difference in motor z-score

6G2: Stratified by Stunting burden

Stunting burden
(p−diff = 0.045)

Country	Trial	Tool	N	N	MD (95% CI)	W
**Stunting burden − Less than 35%**						
Ghana	GHANA (25)					
Ghana	iLiNS−DYAD−G (26)	KDI	302	601	0.03 (−0.11, 0.16)	0.30
Haiti	HAITI (27)					
Kenya	WASH−B (28)	EASQ	1362	4745	0.01 (−0.05, 0.07)	0.43
Malawi	iLiNS−DYAD−M (30)	KDI	214	436	0.03 (−0.13, 0.18)	0.27
**I² = 0.00, Tau² = 0.00**			**1878**	**5782**	**0.01 (−0.04, 0.07)**	
**Stunting burden − More than 35%**						
Bangladesh	JiVitA−4 (21)	BSID−III	445	143	−0.02 (−0.17, 0.14)	0.10
Bangladesh	RDNS (22)	DMC	1556	753	0.10 (0.03, 0.17)	0.16
Bangladesh	WASH−B (23)	EASQ	1074	3279	0.10 (0.04, 0.17)	0.16
Burkina Faso	iLiNS−Zinc (24)	DMC	746	375	0.40 (0.24, 0.57)	0.10
Madagascar	MAHAY (29)	ASQI	1613	1604	0.07 (−0.13, 0.27)	0.08
Malawi	iLiNS−DOSE (31)	KDI	646	221	0.05 (−0.10, 0.20)	0.10
Mali	PROMIS CS (32)	DMC	902	921	0.13 (−0.04, 0.30)	0.09
Zimbabwe	SHINE (HIV−) (33), LNS vs SOC	MDAT	395	382	0.00 (−0.16, 0.15)	0.13
Zimbabwe	LNS+WSH vs WSH	MDAT	446	417	0.20 (0.05, 0.35)	0.13
Zimbabwe	SHINE (HIV+) (34), LNS vs SOC	MDAT	67	68	0.04 (−0.30, 0.39)	0.07
Zimbabwe	LNS+WSH vs WSH	MDAT	103	83	0.49 (0.23, 0.74)	0.07
**I² = 0.57, Tau² = 0.01**			**7993**	**8246**	**0.13 (0.05, 0.21)**	

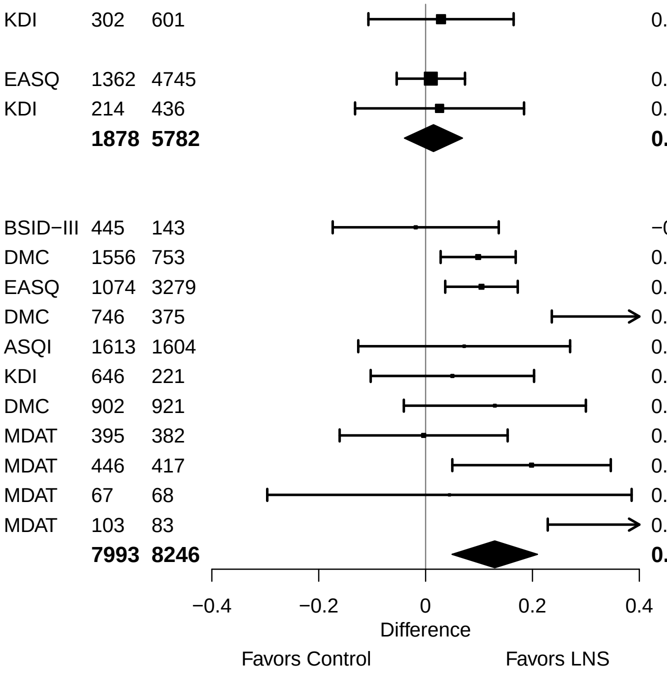

Supplemental figure 6G: Mean difference in motor z-score

6G3: Stratified by Malaria prevalence

Malaria prevalence
(p−diff = 0.557)

Country	Trial	Tool	N	N	MD (95% CI)	W
**Malaria prevalence − Less than 10%**						
Bangladesh	JiVitA−4 (21)	BSID−III	445	143	−0.02 (−0.17, 0.14)	0.12
Bangladesh	RDNS (22)	DMC	1556	753	0.10 (0.03, 0.17)	0.18
Bangladesh	WASH−B (23)	EASQ	1074	3279	0.10 (0.04, 0.17)	0.18
Haiti	HAITI (27)					
Kenya	WASH−B (28)	EASQ	1362	4745	0.01 (−0.05, 0.07)	0.19
Madagascar	MAHAY (29)	ASQI	1613	1604	0.07 (−0.13, 0.27)	0.09
Zimbabwe	SHINE (HIV−) (33), LNS vs SOC	MDAT	395	382	0.00 (−0.16, 0.15)	0.15
Zimbabwe	LNS+WSH vs WSH	MDAT	446	417	0.20 (0.05, 0.35)	0.15
Zimbabwe	SHINE (HIV+) (34), LNS vs SOC	MDAT	67	68	0.04 (−0.30, 0.39)	0.08
Zimbabwe	LNS+WSH vs WSH	MDAT	103	83	0.49 (0.23, 0.74)	0.08
**I² = 0.47, Tau² = 0.00**			**7061**	**11474**	**0.08 (0.02, 0.14)**	
**Malaria prevalence − At least 10%**						
Burkina Faso	iLiNS−Zinc (24)	DMC	746	375	0.40 (0.24, 0.57)	0.19
Ghana	GHANA (25)					
Ghana	iLiNS−DYAD−G (26)	KDI	302	601	0.03 (−0.11, 0.16)	0.22
Malawi	iLiNS−DYAD−M (30)	KDI	214	436	0.03 (−0.13, 0.18)	0.20
Malawi	iLiNS−DOSE (31)	KDI	646	221	0.05 (−0.10, 0.20)	0.20
Mali	PROMIS CS (32)	DMC	902	921	0.13 (−0.04, 0.30)	0.18
**I² = 0.73, Tau² = 0.02**			**2810**	**2554**	**0.12 (−0.01, 0.26)**	

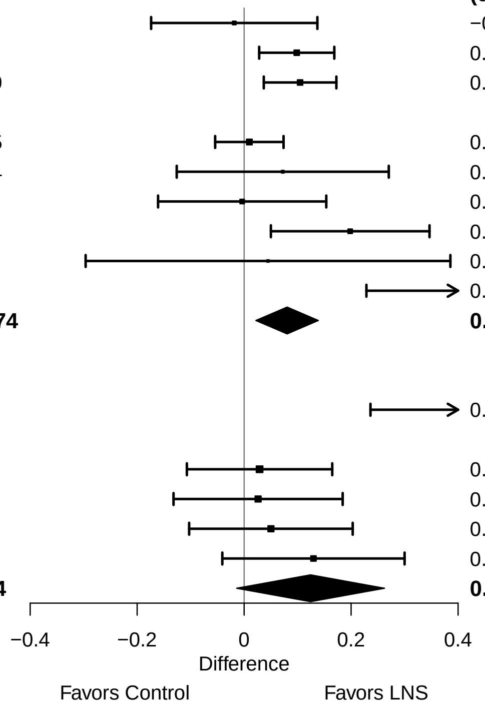

Supplemental figure 6G: Mean difference in motor z-score

6G4: Stratified by Anemia prevalence

Anemia prevalence
(p−diff = 0.269)

Country	Trial	Tool	N	N	MD (95% CI)	W
**Anemia prevalence − High**						
Burkina Faso	iLiNS−Zinc (24)	DMC	746	375	0.40 (0.24, 0.57)	0.25
Ghana	GHANA (25)					
Haiti	HAITI (27)					
Malawi	iLiNS−DYAD−M (30)	KDI	214	436	0.03 (−0.13, 0.18)	0.25
Malawi	iLiNS−DOSE (31)	KDI	646	221	0.05 (−0.10, 0.20)	0.26
Mali	PROMIS CS (32)	DMC	902	921	0.13 (−0.04, 0.30)	0.24
**I² = 0.77, Tau² = 0.02**			**2508**	**1953**	**0.15 (−0.02, 0.32)**	
**Anemia prevalence − Moderate**						
Bangladesh	JiVitA−4 (21)	BSID−III	445	143	−0.02 (−0.17, 0.14)	0.10
Bangladesh	RDNS (22)	DMC	1556	753	0.10 (0.03, 0.17)	0.16
Bangladesh	WASH−B (23)	EASQ	1074	3279	0.10 (0.04, 0.17)	0.16
Ghana	iLiNS−DYAD−G (26)	KDI	302	601	0.03 (−0.11, 0.16)	0.12
Kenya	WASH−B (28)	EASQ	1362	4745	0.01 (−0.05, 0.07)	0.16
Madagascar	MAHAY (29)	ASQI	1613	1604	0.07 (−0.13, 0.27)	0.08
Zimbabwe	SHINE (HIV−) (33), LNS vs SOC	MDAT	395	382	0.00 (−0.16, 0.15)	0.13
Zimbabwe	LNS+WSH vs WSH	MDAT	446	417	0.20 (0.05, 0.35)	0.13
Zimbabwe	SHINE (HIV+) (34), LNS vs SOC	MDAT	67	68	0.04 (−0.30, 0.39)	0.07
Zimbabwe	LNS+WSH vs WSH	MDAT	103	83	0.49 (0.23, 0.74)	0.07
**I² = 0.40, Tau² = 0.00**			**7363**	**12075**	**0.07 (0.02, 0.13)**	

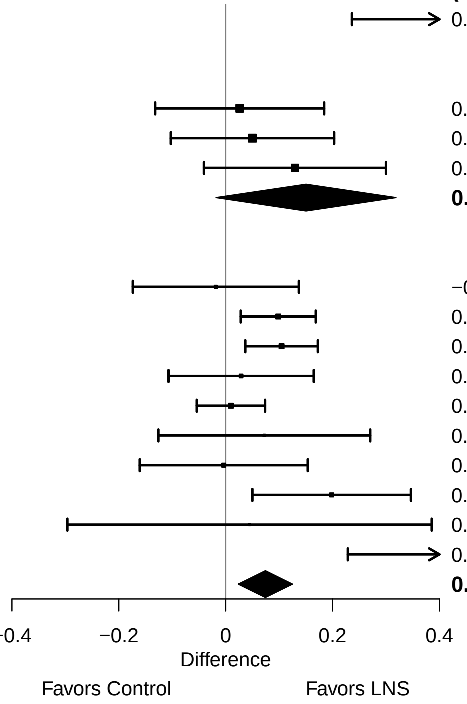

Supplemental figure 6G: Mean difference in motor z-score

6G5: Stratified by Source water quality

Source water quality
(p−diff = 0.530)

Source water quality − Improved

Country	Trial	Tool	N	N	MD (95% CI)	W
Bangladesh	JiVitA−4 (21)	BSID−III	445	143	−0.02 (−0.17, 0.14)	0.14
Bangladesh	RDNS (22)	DMC	1556	753	0.10 (0.03, 0.17)	0.23
Bangladesh	WASH−B (23)	EASQ	528	1099	0.19 (0.09, 0.30)	0.19
Ghana	GHANA (25)					
Ghana	iLiNS−DYAD−G (26)	KDI	302	601	0.03 (−0.11, 0.16)	0.16
Haiti	HAITI (27)					
Malawi	iLiNS−DYAD−M (30)	KDI	214	436	0.03 (−0.13, 0.18)	0.14
Malawi	iLiNS−DOSE (31)	KDI	646	221	0.05 (−0.10, 0.20)	0.14
**I² = 0.32, Tau² = 0.00**			**3691**	**3253**	**0.08 (0.02, 0.14)**	

Source water quality − Unimproved

Country	Trial	Tool	N	N	MD (95% CI)	W
Burkina Faso	iLiNS−Zinc (24)	DMC	746	375	0.40 (0.24, 0.57)	0.18
Kenya	WASH−B (28)	EASQ	649	2073	0.03 (−0.06, 0.13)	0.26
Madagascar	MAHAY (29)	ASQI	1613	1604	0.07 (−0.13, 0.27)	0.14
Mali	PROMIS CS (32)	DMC	902	921	0.13 (−0.04, 0.30)	0.17
Zimbabwe	SHINE (HIV−) (33)	MDAT	395	382	0.00 (−0.16, 0.15)	0.18
Zimbabwe	SHINE (HIV+) (34)	MDAT	67	68	0.04 (−0.30, 0.39)	0.07
**I² = 0.70, Tau² = 0.01**			**4372**	**5423**	**0.12 (−0.01, 0.24)**	

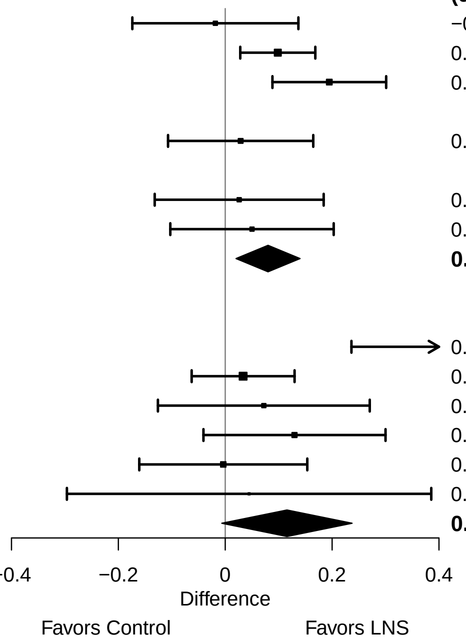

Supplemental figure 6G: Mean difference in motor z-score

6G6: Stratified by Sanitation

Sanitation
(p−diff = 0.963)

Country	Trial	Tool	N	N	MD (95% CI)	W
**Sanitation − Improved**						
Bangladesh	JiVitA−4 (21)	BSID−III	445	143	−0.02 (−0.17, 0.14)	0.17
Bangladesh	RDNS (22)	DMC	1556	753	0.10 (0.03, 0.17)	0.27
Bangladesh	WASH−B (23)	EASQ	528	1099	0.19 (0.09, 0.30)	0.22
Ghana	GHANA (25)					
Ghana	iLiNS−DYAD−G (26)	KDI	302	601	0.03 (−0.11, 0.16)	0.19
Haiti	HAITI (27)					
Mali	PROMIS CS (32)	DMC	902	921	0.13 (−0.04, 0.30)	0.15
**I² = 0.38, Tau² = 0.00**			**3733**	**3517**	**0.10 (0.03, 0.17)**	
**Sanitation − Unimproved**						
Burkina Faso	iLiNS−Zinc (24)	DMC	746	375	0.40 (0.24, 0.57)	0.15
Kenya	WASH−B (28)	EASQ	649	2073	0.03 (−0.06, 0.13)	0.22
Madagascar	MAHAY (29)	ASQI	1613	1604	0.07 (−0.13, 0.27)	0.12
Malawi	iLiNS−DYAD−M (30)	KDI	214	436	0.03 (−0.13, 0.18)	0.15
Malawi	iLiNS−DOSE (31)	KDI	646	221	0.05 (−0.10, 0.20)	0.16
Zimbabwe	SHINE (HIV−) (33)	MDAT	395	382	0.00 (−0.16, 0.15)	0.15
Zimbabwe	SHINE (HIV+) (34)	MDAT	67	68	0.04 (−0.30, 0.39)	0.05
**I² = 0.65, Tau² = 0.01**			**4330**	**5159**	**0.09 (−0.02, 0.19)**	

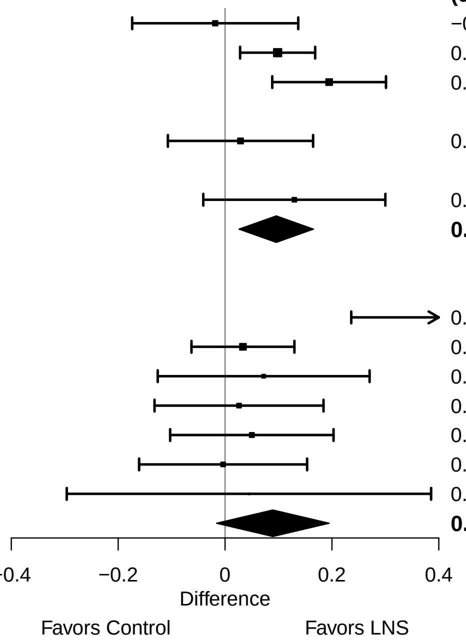

Supplemental figure 6G: Mean difference in motor z-score

6G7: Stratified by Supplement duration

Supplement duration
(p−diff = 0.621)

Country	Trial	Tool	N	N	MD (95% CI)	W
**Supplement duration − 12m or less**						
Bangladesh	JiVitA−4 (21)	BSID−III	445	143	−0.02 (−0.17, 0.14)	0.13
Burkina Faso	iLiNS−Zinc (24)	DMC	746	375	0.40 (0.24, 0.57)	0.12
Ghana	GHANA (25)					
Ghana	iLiNS−DYAD−G (26)	KDI	302	601	0.03 (−0.11, 0.16)	0.14
Haiti	HAITI (27)					
Madagascar	MAHAY (29)	ASQI	1613	1604	0.07 (−0.13, 0.27)	0.10
Malawi	iLiNS−DYAD−M (30)	KDI	214	436	0.03 (−0.13, 0.18)	0.13
Malawi	iLiNS−DOSE (31)	KDI	646	221	0.05 (−0.10, 0.20)	0.13
Zimbabwe	SHINE (HIV−) (33), LNS vs SOC	MDAT	395	382	0.00 (−0.16, 0.15)	0.16
Zimbabwe	LNS+WSH vs WSH	MDAT	446	417	0.20 (0.05, 0.35)	0.16
Zimbabwe	SHINE (HIV+) (34), LNS vs SOC	MDAT	67	68	0.04 (−0.30, 0.39)	0.09
Zimbabwe	LNS+WSH vs WSH	MDAT	103	83	0.49 (0.23, 0.74)	0.09
**I² = 0.67, Tau² = 0.01**			**4977**	**4330**	**0.11 (0.01, 0.22)**	
**Supplement duration − > 12m**						
Bangladesh	RDNS (22)	DMC	1556	753	0.10 (0.03, 0.17)	0.28
Bangladesh	WASH−B (23)	EASQ	1074	3279	0.10 (0.04, 0.17)	0.28
Kenya	WASH−B (28)	EASQ	1362	4745	0.01 (−0.05, 0.07)	0.28
Mali	PROMIS CS (32)	DMC	902	921	0.13 (−0.04, 0.30)	0.17
**I² = 0.45, Tau² = 0.00**			**4894**	**9698**	**0.07 (0.02, 0.13)**	

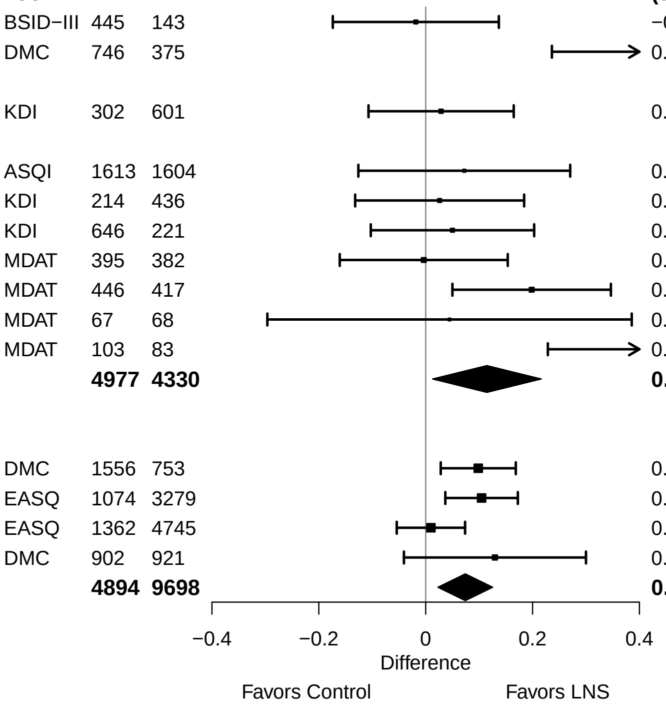

Supplemental figure 6G: Mean difference in motor z-score

6G8: Stratified by Frequency of contact

Frequency of contact
(p−diff = 0.932)

Country	Trial	Tool	N	N	MD (95% CI)	W
**Frequency of contact − Monthly**						
Bangladesh	RDNS (22)	DMC	1556	753	0.10 (0.03, 0.17)	0.23
Haiti	HAITI (27)					
Kenya	WASH−B (28)	EASQ	1362	4745	0.01 (−0.05, 0.07)	0.23
Madagascar	MAHAY (29)	ASQI	1613	1604	0.07 (−0.13, 0.27)	0.12
Mali	PROMIS CS (32)	DMC	902	921	0.13 (−0.04, 0.30)	0.14
Zimbabwe	SHINE (HIV−) (33), LNS vs SOC	MDAT	395	382	0.00 (−0.16, 0.15)	0.19
Zimbabwe	LNS+WSH vs WSH	MDAT	446	417	0.20 (0.05, 0.35)	0.19
Zimbabwe	SHINE (HIV+) (34), LNS vs SOC	MDAT	67	68	0.04 (−0.30, 0.39)	0.10
Zimbabwe	LNS+WSH vs WSH	MDAT	103	83	0.49 (0.23, 0.74)	0.10
**I² = 0.47, Tau² = 0.00**			**6444**	**8973**	**0.09 (0.03, 0.16)**	
**Frequency of contact − Weekly**						
Bangladesh	JiVitA−4 (21)	BSID−III	445	143	−0.02 (−0.17, 0.14)	0.15
Bangladesh	WASH−B (23)	EASQ	1074	3279	0.10 (0.04, 0.17)	0.23
Burkina Faso	iLiNS−Zinc (24)	DMC	746	375	0.40 (0.24, 0.57)	0.14
Ghana	GHANA (25)					
Ghana	iLiNS−DYAD−G (26)	KDI	302	601	0.03 (−0.11, 0.16)	0.17
Malawi	iLiNS−DYAD−M (30)	KDI	214	436	0.03 (−0.13, 0.18)	0.15
Malawi	iLiNS−DOSE (31)	KDI	646	221	0.05 (−0.10, 0.20)	0.15
**I² = 0.71, Tau² = 0.02**			**3427**	**5055**	**0.10 (−0.02, 0.21)**	

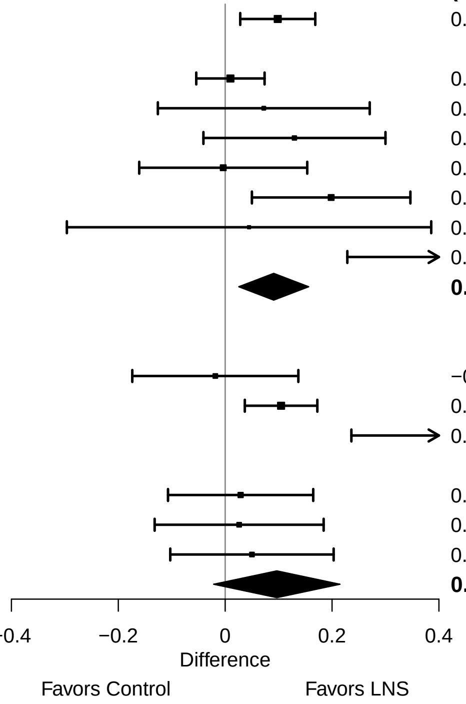

Supplemental figure 6G: Mean difference in motor z-score

6G9: Stratified by Average SQ-LNS compliance

Average SQ−LNS compliance
(p−diff = 0.898)

Country	Trial	Tool	N	N	MD (95% CI)	W
**Average SQ−LNS compliance − Low**						
Ghana	iLiNS−DYAD−G (26)	KDI	302	601	0.03 (−0.11, 0.16)	0.18
Malawi	iLiNS−DYAD−M (30)	KDI	214	436	0.03 (−0.13, 0.18)	0.16
Malawi	iLiNS−DOSE (31)	KDI	646	221	0.05 (−0.10, 0.20)	0.17
Mali	PROMIS CS (32)	DMC	902	921	0.13 (−0.04, 0.30)	0.15
Zimbabwe	SHINE (HIV−) (33), LNS vs SOC	MDAT	395	382	0.00 (−0.16, 0.15)	0.21
Zimbabwe	LNS+WSH vs WSH	MDAT	446	417	0.20 (0.05, 0.35)	0.21
Zimbabwe	SHINE (HIV+) (34), LNS vs SOC	MDAT	67	68	0.04 (−0.30, 0.39)	0.12
Zimbabwe	LNS+WSH vs WSH	MDAT	103	83	0.49 (0.23, 0.74)	0.12
**I² = 0.14, Tau² = 0.00**			**3075**	**3129**	**0.09 (0.02, 0.16)**	
**Average SQ−LNS compliance − High**						
Bangladesh	JiVitA−4 (21)	BSID−III	445	143	−0.02 (−0.17, 0.14)	0.16
Bangladesh	RDNS (22)	DMC	1556	753	0.10 (0.03, 0.17)	0.23
Bangladesh	WASH−B (23)	EASQ	1074	3279	0.10 (0.04, 0.17)	0.23
Burkina Faso	iLiNS−Zinc (24)	DMC	746	375	0.40 (0.24, 0.57)	0.15
Ghana	GHANA (25)					
Haiti	HAITI (27)					
Kenya	WASH−B (28)	EASQ	1362	4745	0.01 (−0.05, 0.07)	0.23
**I² = 0.81, Tau² = 0.02**			**5183**	**9295**	**0.11 (−0.03, 0.25)**	

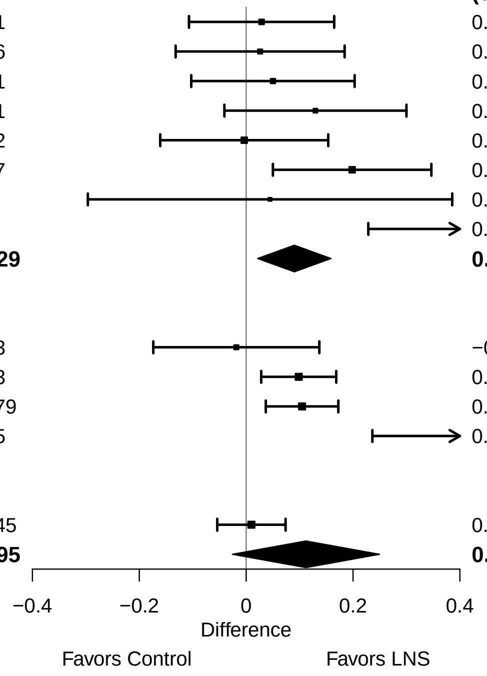

Supplemental figure 6H: Motor lowest decile prevalence ratio

6H1: Stratified by Geographic region

Geographic region
(p−diff = 0.610)

Country	Trial	Tool	N	N	PR (95% CI)	W
**Geographic region − SEAR**						
Bangladesh	JiVitA−4 (21)	BSID−III	445	143	1.26 (0.77, 2.07)	0.17
Bangladesh	RDNS (22)	DMC	1556	753	0.85 (0.67, 1.07)	0.40
Bangladesh	WASH−B (23)	EASQ	1074	3279	0.82 (0.66, 1.01)	0.43
**I² = 0.20, Tau² = 0.01**			**3075**	**4175**	**0.88 (0.72, 1.08)**	
**Geographic region − AFR**						
Burkina Faso	iLiNS−Zinc (24)	DMC	746	375	0.49 (0.36, 0.67)	0.14
Ghana	GHANA (25)					
Ghana	iLiNS−DYAD−G (26)	KDI	302	601	1.13 (0.76, 1.70)	0.10
Kenya	WASH−B (28)	EASQ	1362	4745	0.86 (0.70, 1.05)	0.21
Madagascar	MAHAY (29)	ASQI	1613	1604	0.91 (0.58, 1.44)	0.09
Malawi	iLiNS−DYAD−M (30)	KDI	214	436	0.84 (0.51, 1.40)	0.07
Malawi	iLiNS−DOSE (31)	KDI	646	221	1.08 (0.67, 1.71)	0.08
Mali	PROMIS CS (32)	DMC	902	921	0.81 (0.56, 1.18)	0.11
Zimbabwe	SHINE (HIV−) (33), LNS vs SOC	MDAT	395	382	1.08 (0.67, 1.73)	0.14
Zimbabwe	LNS+WSH vs WSH	MDAT	446	417	0.82 (0.54, 1.24)	0.14
Zimbabwe	SHINE (HIV+) (34), LNS vs SOC	MDAT	67	68	1.16 (0.44, 3.08)	0.05
Zimbabwe	LNS+WSH vs WSH	MDAT	103	83	0.40 (0.17, 0.95)	0.05
**I² = 0.48, Tau² = 0.03**			**6796**	**9853**	**0.83 (0.70, 0.98)**	

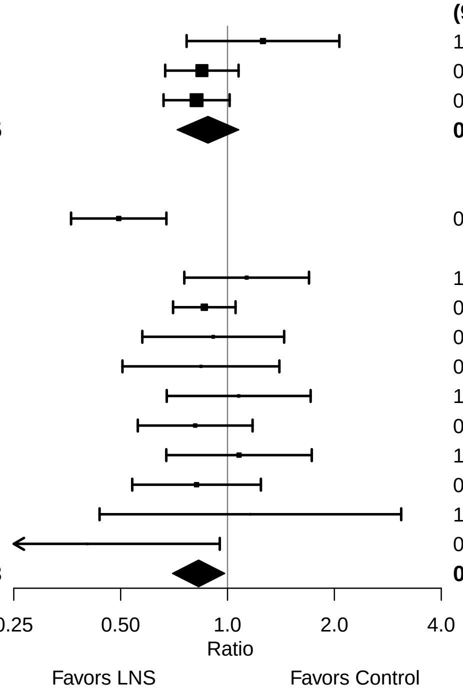

Supplemental figure 6H: Motor lowest decile prevalence ratio

6H2: Stratified by Stunting burden

Stunting burden
(p−diff = 0.466)

Country	Trial	Tool	N	N	PR (95% CI)	W
**Stunting burden − Less than 35%**						
Ghana	GHANA (25)					
Ghana	iLiNS−DYAD−G (26)	KDI	302	601	1.13 (0.76, 1.70)	0.27
Haiti	HAITI (27)					
Kenya	WASH−B (28)	EASQ	1362	4745	0.86 (0.70, 1.05)	0.54
Malawi	iLiNS−DYAD−M (30)	KDI	214	436	0.84 (0.51, 1.40)	0.19
**I² = 0.00, Tau² = 0.00**			**1878**	**5782**	**0.90 (0.76, 1.07)**	
**Stunting burden − More than 35%**						
Bangladesh	JiVitA−4 (21)	BSID−III	445	143	1.26 (0.77, 2.07)	0.07
Bangladesh	RDNS (22)	DMC	1556	753	0.85 (0.67, 1.07)	0.17
Bangladesh	WASH−B (23)	EASQ	1074	3279	0.82 (0.66, 1.01)	0.18
Burkina Faso	iLiNS−Zinc (24)	DMC	746	375	0.49 (0.36, 0.67)	0.13
Madagascar	MAHAY (29)	ASQI	1613	1604	0.91 (0.58, 1.44)	0.08
Malawi	iLiNS−DOSE (31)	KDI	646	221	1.08 (0.67, 1.71)	0.08
Mali	PROMIS CS (32)	DMC	902	921	0.81 (0.56, 1.18)	0.11
Zimbabwe	SHINE (HIV−) (33), LNS vs SOC	MDAT	395	382	1.08 (0.67, 1.73)	0.13
Zimbabwe	LNS+WSH vs WSH	MDAT	446	417	0.82 (0.54, 1.24)	0.13
Zimbabwe	SHINE (HIV+) (34), LNS vs SOC	MDAT	67	68	1.16 (0.44, 3.08)	0.05
Zimbabwe	LNS+WSH vs WSH	MDAT	103	83	0.40 (0.17, 0.95)	0.05
**I² = 0.49, Tau² = 0.03**			**7993**	**8246**	**0.83 (0.70, 0.98)**	

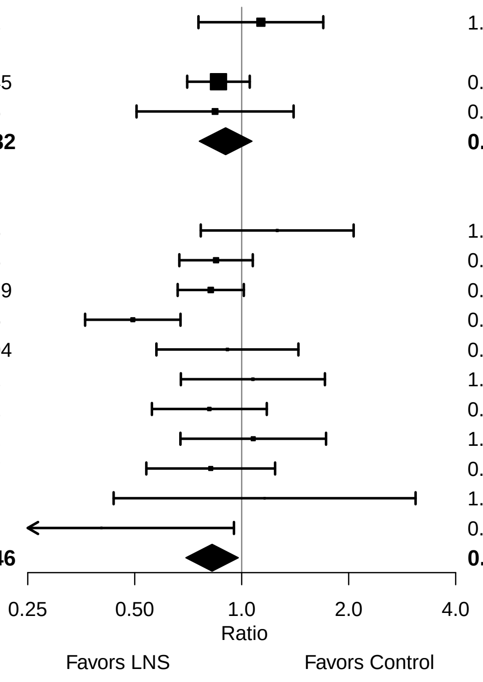

Supplemental figure 6H: Motor lowest decile prevalence ratio

6H3: Stratified by Malaria prevalence

Malaria prevalence
(p−diff = 0.491)

Country	Trial	Tool	N	N	PR (95% CI)	W
**Malaria prevalence − Less than 10%**						
Bangladesh	JiVitA−4 (21)	BSID−III	445	143	1.26 (0.77, 2.07)	0.08
Bangladesh	RDNS (22)	DMC	1556	753	0.85 (0.67, 1.07)	0.19
Bangladesh	WASH−B (23)	EASQ	1074	3279	0.82 (0.66, 1.01)	0.21
Haiti	HAITI (27)					
Kenya	WASH−B (28)	EASQ	1362	4745	0.86 (0.70, 1.05)	0.22
Madagascar	MAHAY (29)	ASQI	1613	1604	0.91 (0.58, 1.44)	0.09
Zimbabwe	SHINE (HIV−) (33), LNS vs SOC	MDAT	395	382	1.08 (0.67, 1.73)	0.15
Zimbabwe	LNS+WSH vs WSH	MDAT	446	417	0.82 (0.54, 1.24)	0.15
Zimbabwe	SHINE (HIV+) (34), LNS vs SOC	MDAT	67	68	1.16 (0.44, 3.08)	0.05
Zimbabwe	LNS+WSH vs WSH	MDAT	103	83	0.40 (0.17, 0.95)	0.05
**I² = 0.00, Tau² = 0.00**			**7061**	**11474**	**0.87 (0.78, 0.97)**	
**Malaria prevalence − At least 10%**						
Burkina Faso	iLiNS−Zinc (24)	DMC	746	375	0.49 (0.36, 0.67)	0.27
Ghana	GHANA (25)					
Ghana	iLiNS−DYAD−G (26)	KDI	302	601	1.13 (0.76, 1.70)	0.20
Malawi	iLiNS−DYAD−M (30)	KDI	214	436	0.84 (0.51, 1.40)	0.14
Malawi	iLiNS−DOSE (31)	KDI	646	221	1.08 (0.67, 1.71)	0.16
Mali	PROMIS CS (32)	DMC	902	921	0.81 (0.56, 1.18)	0.22
**I² = 0.71, Tau² = 0.08**			**2810**	**2554**	**0.82 (0.60, 1.10)**	

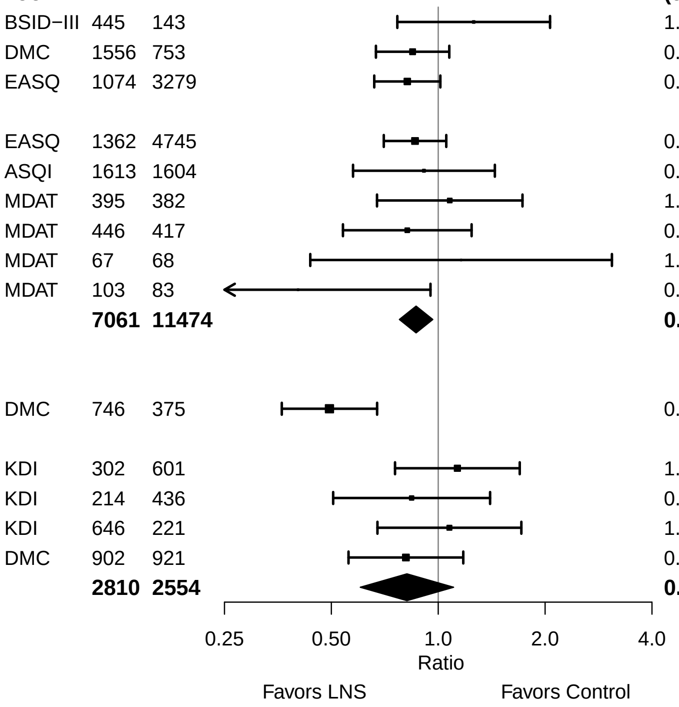

Supplemental figure 6H: Motor lowest decile prevalence ratio

6H4: Stratified by Anemia prevalence

Anemia prevalence
(p−diff = 0.093)

Country	Trial	Tool	N	N	PR (95% CI)	W
**Anemia prevalence − High**						
Burkina Faso	iLiNS−Zinc (24)	DMC	746	375	0.49 (0.36, 0.67)	0.34
Ghana	GHANA (25)					
Haiti	HAITI (27)					
Malawi	iLiNS−DYAD−M (30)	KDI	214	436	0.84 (0.51, 1.40)	0.18
Malawi	iLiNS−DOSE (31)	KDI	646	221	1.08 (0.67, 1.71)	0.20
Mali	PROMIS CS (32)	DMC	902	921	0.81 (0.56, 1.18)	0.28
**I² = 0.68, Tau² = 0.07**			**2508**	**1953**	**0.75 (0.54, 1.05)**	
**Anemia prevalence − Moderate**						
Bangladesh	JiVitA−4 (21)	BSID−III	445	143	1.26 (0.77, 2.07)	0.07
Bangladesh	RDNS (22)	DMC	1556	753	0.85 (0.67, 1.07)	0.18
Bangladesh	WASH−B (23)	EASQ	1074	3279	0.82 (0.66, 1.01)	0.19
Ghana	iLiNS−DYAD−G (26)	KDI	302	601	1.13 (0.76, 1.70)	0.10
Kenya	WASH−B (28)	EASQ	1362	4745	0.86 (0.70, 1.05)	0.20
Madagascar	MAHAY (29)	ASQI	1613	1604	0.91 (0.58, 1.44)	0.08
Zimbabwe	SHINE (HIV−) (33), LNS vs SOC	MDAT	395	382	1.08 (0.67, 1.73)	0.13
Zimbabwe	LNS+WSH vs WSH	MDAT	446	417	0.82 (0.54, 1.24)	0.13
Zimbabwe	SHINE (HIV+) (34), LNS vs SOC	MDAT	67	68	1.16 (0.44, 3.08)	0.05
Zimbabwe	LNS+WSH vs WSH	MDAT	103	83	0.40 (0.17, 0.95)	0.05
**I² = 0.00, Tau² = 0.00**			**7363**	**12075**	**0.88 (0.79, 0.98)**	

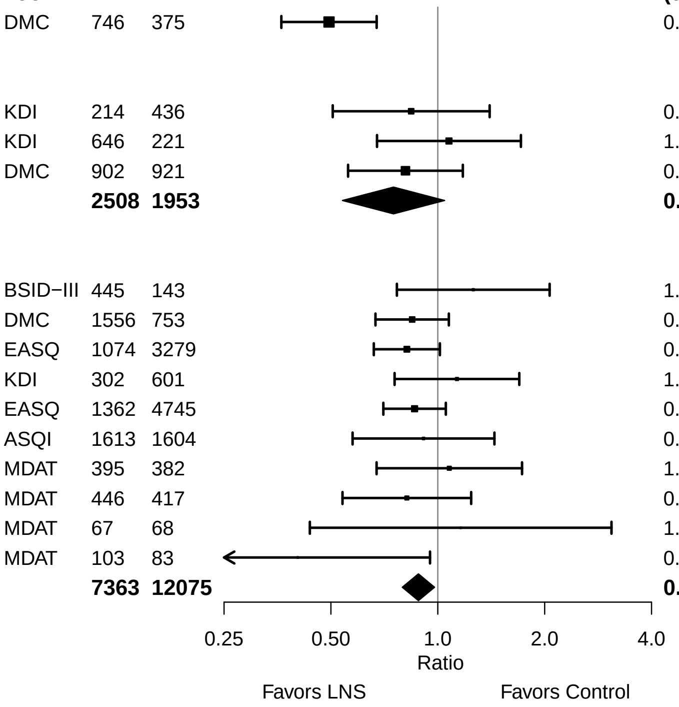

Supplemental figure 6H: Motor lowest decile prevalence ratio

6H5: Stratified by Source water quality

Source water quality
(p−diff = 0.303)

Source water quality − Improved

Country	Trial	Tool	N	N	PR (95% CI)	W
Bangladesh	JiVitA−4 (21)	BSID−III	445	143	1.26 (0.77, 2.07)	0.12
Bangladesh	RDNS (22)	DMC	1556	753	0.85 (0.67, 1.07)	0.27
Bangladesh	WASH−B (23)	EASQ	528	1099	0.81 (0.58, 1.13)	0.20
Ghana	GHANA (25)					
Ghana	iLiNS−DYAD−G (26)	KDI	302	601	1.13 (0.76, 1.70)	0.16
Haiti	HAITI (27)					
Malawi	iLiNS−DYAD−M (30)	KDI	214	436	0.84 (0.51, 1.40)	0.12
Malawi	iLiNS−DOSE (31)	KDI	646	221	1.08 (0.67, 1.71)	0.13
**I² = 0.00, Tau² = 0.00**			**3691**	**3253**	**0.93 (0.80, 1.08)**	

Source water quality − Unimproved

Country	Trial	Tool	N	N	PR (95% CI)	W
Burkina Faso	iLiNS−Zinc (24)	DMC	746	375	0.49 (0.36, 0.67)	0.24
Kenya	WASH−B (28)	EASQ	649	2073	0.96 (0.71, 1.31)	0.24
Madagascar	MAHAY (29)	ASQI	1613	1604	0.91 (0.58, 1.44)	0.15
Mali	PROMIS CS (32)	DMC	902	921	0.81 (0.56, 1.18)	0.19
Zimbabwe	SHINE (HIV−) (33)	MDAT	395	382	1.08 (0.67, 1.73)	0.14
Zimbabwe	SHINE (HIV+) (34)	MDAT	67	68	1.16 (0.44, 3.08)	0.04
**I² = 0.62, Tau² = 0.05**			**4372**	**5423**	**0.82 (0.64, 1.05)**	

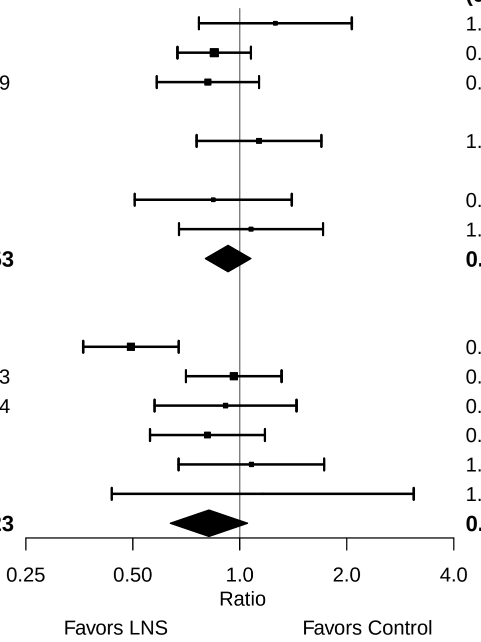

Supplemental figure 6H: Motor lowest decile prevalence ratio

6H6: Stratified by Sanitation

Sanitation
(p−diff = 0.599)

Country	Trial	Tool	N	N	PR (95% CI)	W
**Sanitation − Improved**						
Bangladesh	JiVitA−4 (21)	BSID−III	445	143	1.26 (0.77, 2.07)	0.13
Bangladesh	RDNS (22)	DMC	1556	753	0.85 (0.67, 1.07)	0.29
Bangladesh	WASH−B (23)	EASQ	528	1099	0.81 (0.58, 1.13)	0.22
Ghana	GHANA (25)					
Ghana	iLiNS−DYAD−G (26)	KDI	302	601	1.13 (0.76, 1.70)	0.17
Haiti	HAITI (27)					
Mali	PROMIS CS (32)	DMC	902	921	0.81 (0.56, 1.18)	0.19
**I² = 0.00, Tau² = 0.00**			**3733**	**3517**	**0.90 (0.77, 1.05)**	
**Sanitation − Unimproved**						
Burkina Faso	iLiNS−Zinc (24)	DMC	746	375	0.49 (0.36, 0.67)	0.22
Kenya	WASH−B (28)	EASQ	649	2073	0.96 (0.71, 1.31)	0.22
Madagascar	MAHAY (29)	ASQI	1613	1604	0.91 (0.58, 1.44)	0.14
Malawi	iLiNS−DYAD−M (30)	KDI	214	436	0.84 (0.51, 1.40)	0.12
Malawi	iLiNS−DOSE (31)	KDI	646	221	1.08 (0.67, 1.71)	0.13
Zimbabwe	SHINE (HIV−) (33)	MDAT	395	382	1.08 (0.67, 1.73)	0.13
Zimbabwe	SHINE (HIV+) (34)	MDAT	67	68	1.16 (0.44, 3.08)	0.04
**I² = 0.59, Tau² = 0.04**			**4330**	**5159**	**0.85 (0.68, 1.08)**	

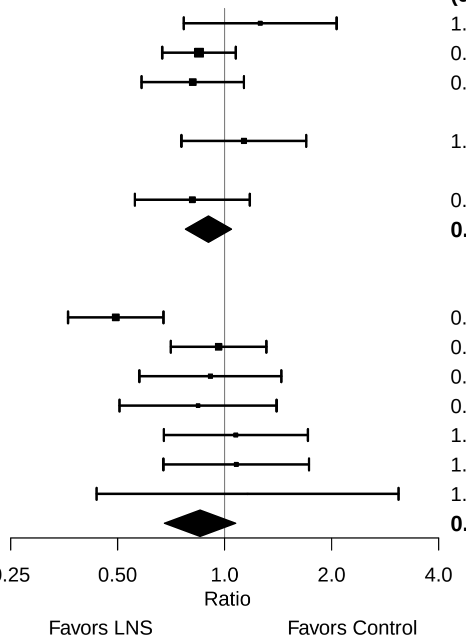

Supplemental figure 6H: Motor lowest decile prevalence ratio

6H7: Stratified by Supplement duration

Supplement duration
(p−diff = 0.839)

Country	Trial		Tool	N	N	PR (95% CI)	W
**Supplement duration − 12m or less**							
Bangladesh	JiVitA−4 (21)		BSID−III	445	143	1.26 (0.77, 2.07)	0.10
Burkina Faso	iLiNS−Zinc (24)		DMC	746	375	0.49 (0.36, 0.67)	0.19
Ghana	GHANA (25)						
Ghana	iLiNS−DYAD−G (26)		KDI	302	601	1.13 (0.76, 1.70)	0.14
Haiti	HAITI (27)						
Madagascar	MAHAY (29)		ASQI	1613	1604	0.91 (0.58, 1.44)	0.11
Malawi	iLiNS−DYAD−M (30)		KDI	214	436	0.84 (0.51, 1.40)	0.10
Malawi	iLiNS−DOSE (31)		KDI	646	221	1.08 (0.67, 1.71)	0.11
Zimbabwe	SHINE (HIV−) (33),	LNS vs SOC	MDAT	395	382	1.08 (0.67, 1.73)	0.18
Zimbabwe		LNS+WSH vs WSH	MDAT	446	417	0.82 (0.54, 1.24)	0.18
Zimbabwe	SHINE (HIV+) (34),	LNS vs SOC	MDAT	67	68	1.16 (0.44, 3.08)	0.06
Zimbabwe		LNS+WSH vs WSH	MDAT	103	83	0.40 (0.17, 0.95)	0.06
**I² = 0.61, Tau² = 0.05**				**4977**	**4330**	**0.87 (0.69, 1.08)**	
**Supplement duration − > 12m**							
Bangladesh	RDNS (22)		DMC	1556	753	0.85 (0.67, 1.07)	0.26
Bangladesh	WASH−B (23)		EASQ	1074	3279	0.82 (0.66, 1.01)	0.28
Kenya	WASH−B (28)		EASQ	1362	4745	0.86 (0.70, 1.05)	0.29
Mali	PROMIS CS (32)		DMC	902	921	0.81 (0.56, 1.18)	0.16
**I² = 0.00, Tau² = 0.00**				**4894**	**9698**	**0.84 (0.74, 0.94)**	

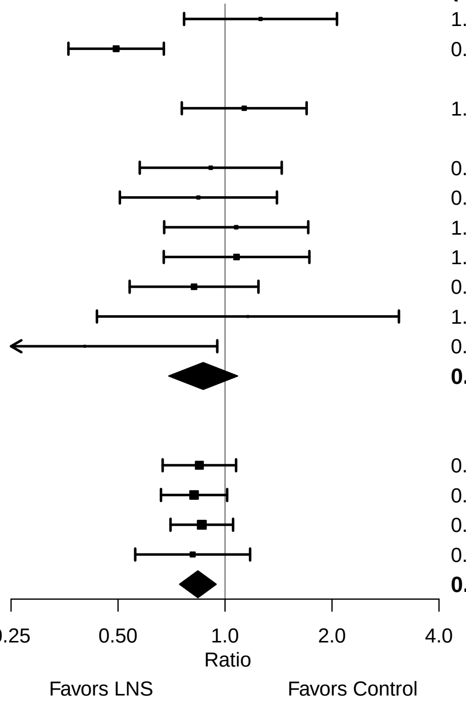

Supplemental figure 6H: Motor lowest decile prevalence ratio

6H8: Stratified by Frequency of contact

Frequency of contact
(p−diff = 0.977)

Country	Trial		Tool	N	N	PR (95% CI)	W
**Frequency of contact − Monthly**							
Bangladesh	RDNS (22)		DMC	1556	753	0.85 (0.67, 1.07)	0.23
Haiti	HAITI (27)						
Kenya	WASH−B (28)		EASQ	1362	4745	0.86 (0.70, 1.05)	0.26
Madagascar	MAHAY (29)		ASQI	1613	1604	0.91 (0.58, 1.44)	0.11
Mali	PROMIS CS (32)		DMC	902	921	0.81 (0.56, 1.18)	0.15
Zimbabwe	SHINE (HIV−) (33),	LNS vs SOC	MDAT	395	382	1.08 (0.67, 1.73)	0.18
Zimbabwe		LNS+WSH vs WSH	MDAT	446	417	0.82 (0.54, 1.24)	0.18
Zimbabwe	SHINE (HIV+) (34),	LNS vs SOC	MDAT	67	68	1.16 (0.44, 3.08)	0.06
Zimbabwe		LNS+WSH vs WSH	MDAT	103	83	0.40 (0.17, 0.95)	0.06
**I² = 0.00, Tau² = 0.00**				**6444**	**8973**	**0.86 (0.76, 0.97)**	
**Frequency of contact − Weekly**							
Bangladesh	JiVitA−4 (21)		BSID−III	445	143	1.26 (0.77, 2.07)	0.11
Bangladesh	WASH−B (23)		EASQ	1074	3279	0.82 (0.66, 1.01)	0.29
Burkina Faso	iLiNS−Zinc (24)		DMC	746	375	0.49 (0.36, 0.67)	0.21
Ghana	GHANA (25)						
Ghana	iLiNS−DYAD−G (26)		KDI	302	601	1.13 (0.76, 1.70)	0.15
Malawi	iLiNS−DYAD−M (30)		KDI	214	436	0.84 (0.51, 1.40)	0.11
Malawi	iLiNS−DOSE (31)		KDI	646	221	1.08 (0.67, 1.71)	0.12
**I² = 0.71, Tau² = 0.08**				**3427**	**5055**	**0.87 (0.66, 1.15)**	

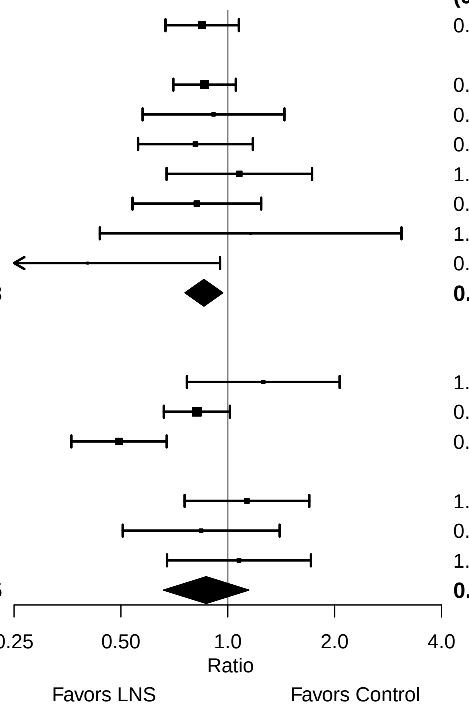

Supplemental figure 6H: Motor lowest decile prevalence ratio

6H9: Stratified by Average SQ-LNS compliance

Average SQ−LNS compliance
(p−diff = 0.330)

Average SQ−LNS compliance − Low

Country	Trial	Tool	N	N	PR (95% CI)	W
Ghana	iLiNS−DYAD−G (26)	KDI	302	601	1.13 (0.76, 1.70)	0.18
Malawi	iLiNS−DYAD−M (30)	KDI	214	436	0.84 (0.51, 1.40)	0.13
Malawi	iLiNS−DOSE (31)	KDI	646	221	1.08 (0.67, 1.71)	0.15
Mali	PROMIS CS (32)	DMC	902	921	0.81 (0.56, 1.18)	0.20
Zimbabwe	SHINE (HIV−) (33), LNS vs SOC	MDAT	395	382	1.08 (0.67, 1.73)	0.24
Zimbabwe	LNS+WSH vs WSH	MDAT	446	417	0.82 (0.54, 1.24)	0.24
Zimbabwe	SHINE (HIV+) (34), LNS vs SOC	MDAT	67	68	1.16 (0.44, 3.08)	0.09
Zimbabwe	LNS+WSH vs WSH	MDAT	103	83	0.40 (0.17, 0.95)	0.09
**I² = 0.00, Tau² = 0.00**			**3075**	**3129**	**0.92 (0.78, 1.09)**	

Average SQ−LNS compliance − High

Country	Trial	Tool	N	N	PR (95% CI)	W
Bangladesh	JiVitA−4 (21)	BSID−III	445	143	1.26 (0.77, 2.07)	0.10
Bangladesh	RDNS (22)	DMC	1556	753	0.85 (0.67, 1.07)	0.23
Bangladesh	WASH−B (23)	EASQ	1074	3279	0.82 (0.66, 1.01)	0.24
Burkina Faso	iLiNS−Zinc (24)	DMC	746	375	0.49 (0.36, 0.67)	0.18
Ghana	GHANA (25)					
Haiti	HAITI (27)					
Kenya	WASH−B (28)	EASQ	1362	4745	0.86 (0.70, 1.05)	0.25
**I² = 0.70, Tau² = 0.07**			**5183**	**9295**	**0.80 (0.61, 1.05)**	

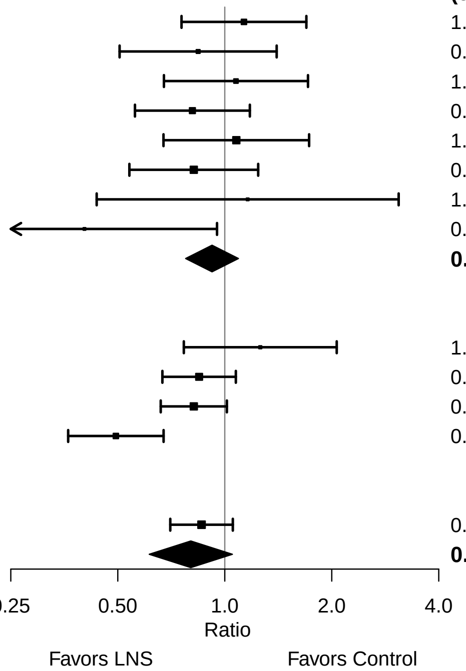

Supplemental figure 6I: Motor lowest decile prevalence difference

6I1: Stratified by Geographic region

Geographic region
(p−diff = 0.995)

Country	Trial	Tool	N	N	PD (95% CI)	W
**Geographic region − SEAR**						
Bangladesh	JiVitA−4 (21)	BSID−III	445	143	0.02 (−0.03, 0.08)	0.13
Bangladesh	RDNS (22)	DMC	1556	753	−0.02 (−0.04, 0.01)	0.37
Bangladesh	WASH−B (23)	EASQ	1074	3279	−0.02 (−0.04, 0.00)	0.50
**I² = 0.00, Tau² = 0.00**			**3075**	**4175**	**−0.02 (−0.03, 0.00)**	
**Geographic region − AFR**						
Burkina Faso	iLiNS−Zinc (24)	DMC	746	375	−0.08 (−0.12, −0.04)	0.08
Ghana	GHANA (25)					
Ghana	iLiNS−DYAD−G (26)	KDI	302	601	0.01 (−0.03, 0.06)	0.08
Kenya	WASH−B (28)	EASQ	1362	4745	−0.01 (−0.03, 0.00)	0.22
Madagascar	MAHAY (29)	ASQI	1613	1604	−0.01 (−0.03, 0.01)	0.19
Malawi	iLiNS−DYAD−M (30)	KDI	214	436	−0.02 (−0.06, 0.03)	0.06
Malawi	iLiNS−DOSE (31)	KDI	646	221	0.01 (−0.04, 0.05)	0.07
Mali	PROMIS CS (32)	DMC	902	921	−0.02 (−0.05, 0.01)	0.14
Zimbabwe	SHINE (HIV−) (33), LNS vs SOC	MDAT	395	382	0.01 (−0.03, 0.05)	0.13
Zimbabwe	LNS+WSH vs WSH	MDAT	446	417	−0.02 (−0.06, 0.02)	0.13
Zimbabwe	SHINE (HIV+) (34), LNS vs SOC	MDAT	67	68	0.02 (−0.09, 0.12)	0.03
Zimbabwe	LNS+WSH vs WSH	MDAT	103	83	−0.09 (−0.17, 0.00)	0.03
**I² = 0.37, Tau² = 0.00**			**6796**	**9853**	**−0.02 (−0.03, 0.00)**	

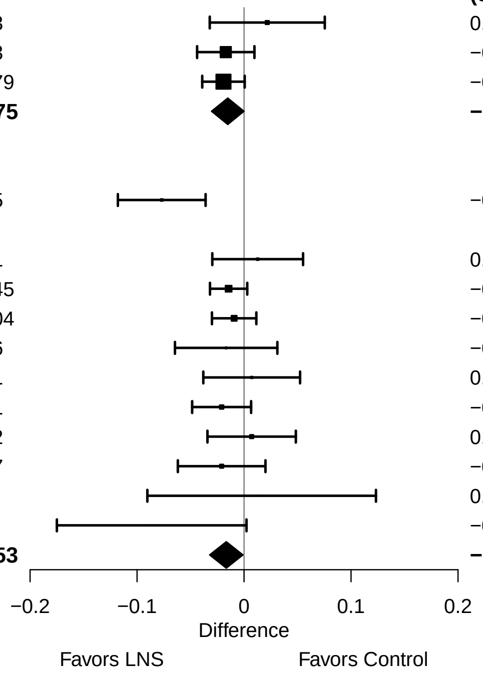

Supplemental figure 6I: Motor lowest decile prevalence difference

6I2: Stratified by Stunting burden

Stunting burden
(p−diff = 0.537)

Country	Trial	Tool	N	N	PD (95% CI)	W
**Stunting burden − Less than 35%**						
Ghana	GHANA (25)					
Ghana	iLiNS−DYAD−G (26)	KDI	302	601	0.01 (−0.03, 0.06)	0.21
Haiti	HAITI (27)					
Kenya	WASH−B (28)	EASQ	1362	4745	−0.01 (−0.03, 0.00)	0.61
Malawi	iLiNS−DYAD−M (30)	KDI	214	436	−0.02 (−0.06, 0.03)	0.17
**I² = 0.00, Tau² = 0.00**			**1878**	**5782**	**−0.01 (−0.03, 0.00)**	
**Stunting burden − More than 35%**						
Bangladesh	JiVitA−4 (21)	BSID−III	445	143	0.02 (−0.03, 0.08)	0.05
Bangladesh	RDNS (22)	DMC	1556	753	−0.02 (−0.04, 0.01)	0.14
Bangladesh	WASH−B (23)	EASQ	1074	3279	−0.02 (−0.04, 0.00)	0.19
Burkina Faso	iLiNS−Zinc (24)	DMC	746	375	−0.08 (−0.12, −0.04)	0.08
Madagascar	MAHAY (29)	ASQI	1613	1604	−0.01 (−0.03, 0.01)	0.18
Malawi	iLiNS−DOSE (31)	KDI	646	221	0.01 (−0.04, 0.05)	0.07
Mali	PROMIS CS (32)	DMC	902	921	−0.02 (−0.05, 0.01)	0.13
Zimbabwe	SHINE (HIV−) (33), LNS vs SOC	MDAT	395	382	0.01 (−0.03, 0.05)	0.13
Zimbabwe	LNS+WSH vs WSH	MDAT	446	417	−0.02 (−0.06, 0.02)	0.13
Zimbabwe	SHINE (HIV+) (34), LNS vs SOC	MDAT	67	68	0.02 (−0.09, 0.12)	0.03
Zimbabwe	LNS+WSH vs WSH	MDAT	103	83	−0.09 (−0.17, 0.00)	0.03
**I² = 0.38, Tau² = 0.00**			**7993**	**8246**	**−0.02 (−0.03, 0.00)**	

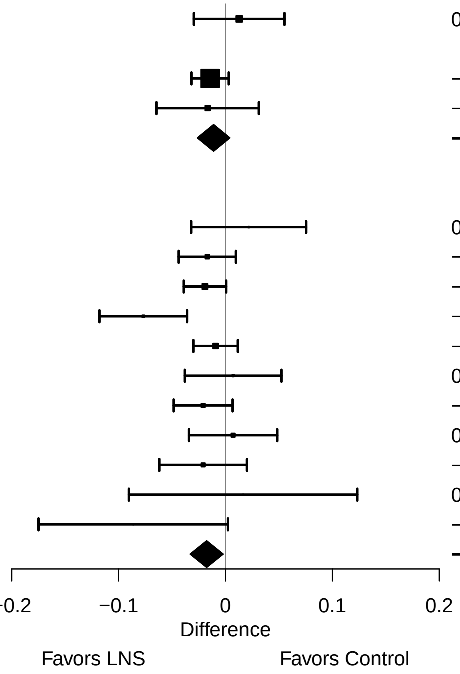

Supplemental figure 6I: Motor lowest decile prevalence difference

6I3: Stratified by Malaria prevalence

Malaria prevalence
(p−diff = 0.476)

Country	Trial	Tool	N	N	PD (95% CI)	W
**Malaria prevalence − Less than 10%**						
Bangladesh	JiVitA−4 (21)	BSID−III	445	143	0.02 (−0.03, 0.08)	0.05
Bangladesh	RDNS (22)	DMC	1556	753	−0.02 (−0.04, 0.01)	0.15
Bangladesh	WASH−B (23)	EASQ	1074	3279	−0.02 (−0.04, 0.00)	0.20
Haiti	HAITI (27)					
Kenya	WASH−B (28)	EASQ	1362	4745	−0.01 (−0.03, 0.00)	0.23
Madagascar	MAHAY (29)	ASQI	1613	1604	−0.01 (−0.03, 0.01)	0.20
Zimbabwe	SHINE (HIV−) (33), LNS vs SOC	MDAT	395	382	0.01 (−0.03, 0.05)	0.13
Zimbabwe	LNS+WSH vs WSH	MDAT	446	417	−0.02 (−0.06, 0.02)	0.13
Zimbabwe	SHINE (HIV+) (34), LNS vs SOC	MDAT	67	68	0.02 (−0.09, 0.12)	0.04
Zimbabwe	LNS+WSH vs WSH	MDAT	103	83	−0.09 (−0.17, 0.00)	0.04
**I² = 0.00, Tau² = 0.00**			**7061**	**11474**	**−0.01 (−0.02, 0.00)**	
**Malaria prevalence − At least 10%**						
Burkina Faso	iLiNS−Zinc (24)	DMC	746	375	−0.08 (−0.12, −0.04)	0.19
Ghana	GHANA (25)					
Ghana	iLiNS−DYAD−G (26)	KDI	302	601	0.01 (−0.03, 0.06)	0.18
Malawi	iLiNS−DYAD−M (30)	KDI	214	436	−0.02 (−0.06, 0.03)	0.15
Malawi	iLiNS−DOSE (31)	KDI	646	221	0.01 (−0.04, 0.05)	0.16
Mali	PROMIS CS (32)	DMC	902	921	−0.02 (−0.05, 0.01)	0.33
**I² = 0.64, Tau² = 0.00**			**2810**	**2554**	**−0.02 (−0.05, 0.01)**	

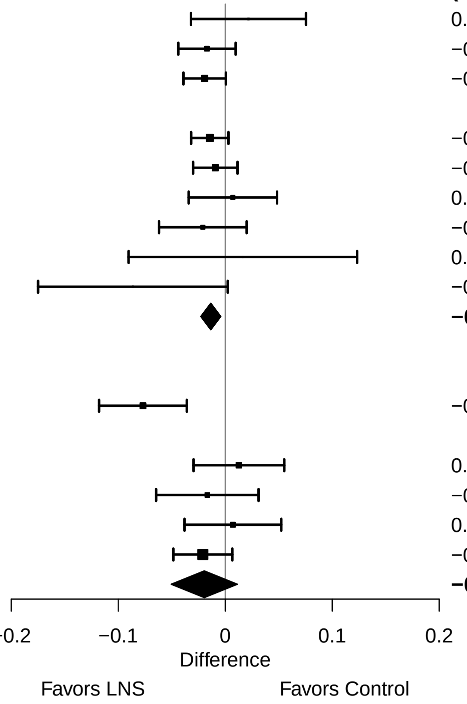

Supplemental figure 6I: Motor lowest decile prevalence difference

6I4: Stratified by Anemia prevalence

Anemia prevalence
(p−diff = 0.163)

Country	Trial	Tool	N	N	PD (95% CI)	W
**Anemia prevalence − High**						
Burkina Faso	iLiNS−Zinc (24)	DMC	746	375	−0.08 (−0.12, −0.04)	0.23
Ghana	GHANA (25)					
Haiti	HAITI (27)					
Malawi	iLiNS−DYAD−M (30)	KDI	214	436	−0.02 (−0.06, 0.03)	0.18
Malawi	iLiNS−DOSE (31)	KDI	646	221	0.01 (−0.04, 0.05)	0.20
Mali	PROMIS CS (32)	DMC	902	921	−0.02 (−0.05, 0.01)	0.40
**I² = 0.64, Tau² = 0.00**			**2508**	**1953**	**−0.03 (−0.06, 0.01)**	
**Anemia prevalence − Moderate**						
Bangladesh	JiVitA−4 (21)	BSID−III	445	143	0.02 (−0.03, 0.08)	0.05
Bangladesh	RDNS (22)	DMC	1556	753	−0.02 (−0.04, 0.01)	0.14
Bangladesh	WASH−B (23)	EASQ	1074	3279	−0.02 (−0.04, 0.00)	0.19
Ghana	iLiNS−DYAD−G (26)	KDI	302	601	0.01 (−0.03, 0.06)	0.07
Kenya	WASH−B (28)	EASQ	1362	4745	−0.01 (−0.03, 0.00)	0.21
Madagascar	MAHAY (29)	ASQI	1613	1604	−0.01 (−0.03, 0.01)	0.18
Zimbabwe	SHINE (HIV−) (33), LNS vs SOC	MDAT	395	382	0.01 (−0.03, 0.05)	0.12
Zimbabwe	LNS+WSH vs WSH	MDAT	446	417	−0.02 (−0.06, 0.02)	0.12
Zimbabwe	SHINE (HIV+) (34), LNS vs SOC	MDAT	67	68	0.02 (−0.09, 0.12)	0.03
Zimbabwe	LNS+WSH vs WSH	MDAT	103	83	−0.09 (−0.17, 0.00)	0.03
**I² = 0.00, Tau² = 0.00**			**7363**	**12075**	**−0.01 (−0.02, 0.00)**	

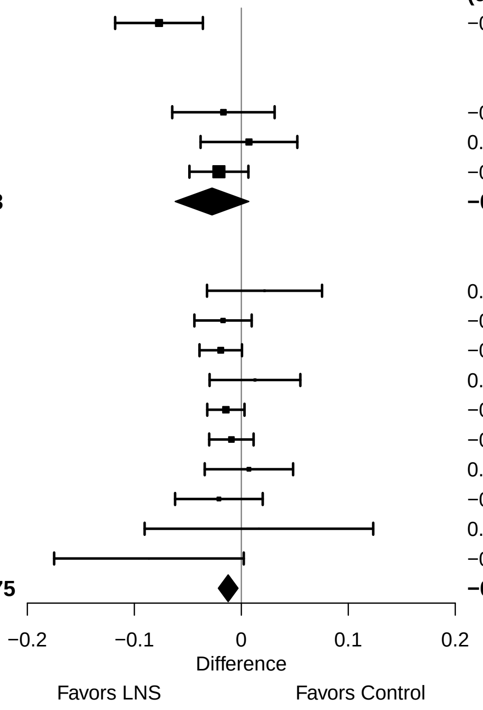

Supplemental figure 6I: Motor lowest decile prevalence difference

6I5: Stratified by Source water quality

Source water quality
(p−diff = 0.444)

Source water quality − Improved

Country	Trial	Tool	N	N	PD (95% CI)	W
Bangladesh	JiVitA−4 (21)	BSID−III	445	143	0.02 (−0.03, 0.08)	0.10
Bangladesh	RDNS (22)	DMC	1556	753	−0.02 (−0.04, 0.01)	0.27
Bangladesh	WASH−B (23)	EASQ	528	1099	−0.02 (−0.05, 0.01)	0.22
Ghana	GHANA (25)					
Ghana	iLiNS−DYAD−G (26)	KDI	302	601	0.01 (−0.03, 0.06)	0.15
Haiti	HAITI (27)					
Malawi	iLiNS−DYAD−M (30)	KDI	214	436	−0.02 (−0.06, 0.03)	0.12
Malawi	iLiNS−DOSE (31)	KDI	646	221	0.01 (−0.04, 0.05)	0.14
**I² = 0.00, Tau² = 0.00**			**3691**	**3253**	**−0.01 (−0.02, 0.01)**	

Source water quality − Unimproved

Country	Trial	Tool	N	N	PD (95% CI)	W
Burkina Faso	iLiNS−Zinc (24)	DMC	746	375	−0.08 (−0.12, −0.04)	0.13
Kenya	WASH−B (28)	EASQ	649	2073	0.00 (−0.03, 0.02)	0.23
Madagascar	MAHAY (29)	ASQI	1613	1604	−0.01 (−0.03, 0.01)	0.28
Mali	PROMIS CS (32)	DMC	902	921	−0.02 (−0.05, 0.01)	0.21
Zimbabwe	SHINE (HIV−) (33)	MDAT	395	382	0.01 (−0.03, 0.05)	0.13
Zimbabwe	SHINE (HIV+) (34)	MDAT	67	68	0.02 (−0.09, 0.12)	0.03
**I² = 0.56, Tau² = 0.00**			**4372**	**5423**	**−0.02 (−0.04, 0.01)**	

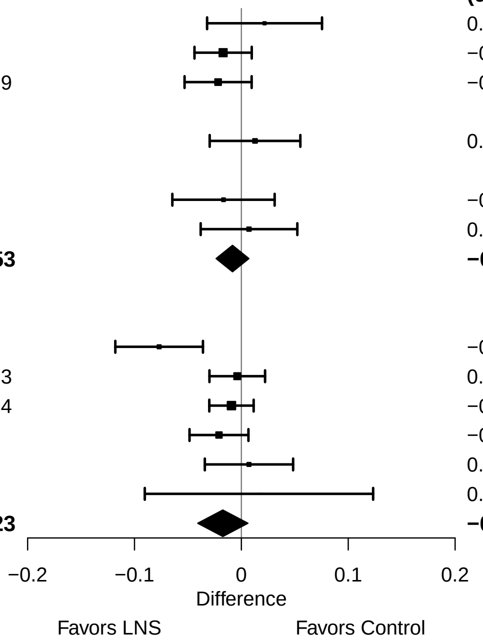

Supplemental figure 6I: Motor lowest decile prevalence difference

6I6: Stratified by Sanitation

Sanitation
(p−diff = 0.871)

Country	Trial	Tool	N	N	PD (95% CI)	W
**Sanitation − Improved**						
Bangladesh	JiVitA−4 (21)	BSID−III	445	143	0.02 (−0.03, 0.08)	0.10
Bangladesh	RDNS (22)	DMC	1556	753	−0.02 (−0.04, 0.01)	0.27
Bangladesh	WASH−B (23)	EASQ	528	1099	−0.02 (−0.05, 0.01)	0.22
Ghana	GHANA (25)					
Ghana	iLiNS−DYAD−G (26)	KDI	302	601	0.01 (−0.03, 0.06)	0.15
Haiti	HAITI (27)					
Mali	PROMIS CS (32)	DMC	902	921	−0.02 (−0.05, 0.01)	0.26
**I² = 0.00, Tau² = 0.00**			**3733**	**3517**	**−0.01 (−0.03, 0.00)**	
**Sanitation − Unimproved**						
Burkina Faso	iLiNS−Zinc (24)	DMC	746	375	−0.08 (−0.12, −0.04)	0.13
Kenya	WASH−B (28)	EASQ	649	2073	0.00 (−0.03, 0.02)	0.23
Madagascar	MAHAY (29)	ASQI	1613	1604	−0.01 (−0.03, 0.01)	0.28
Malawi	iLiNS−DYAD−M (30)	KDI	214	436	−0.02 (−0.06, 0.03)	0.10
Malawi	iLiNS−DOSE (31)	KDI	646	221	0.01 (−0.04, 0.05)	0.11
Zimbabwe	SHINE (HIV−) (33)	MDAT	395	382	0.01 (−0.03, 0.05)	0.13
Zimbabwe	SHINE (HIV+) (34)	MDAT	67	68	0.02 (−0.09, 0.12)	0.03
**I² = 0.50, Tau² = 0.00**			**4330**	**5159**	**−0.01 (−0.04, 0.01)**	

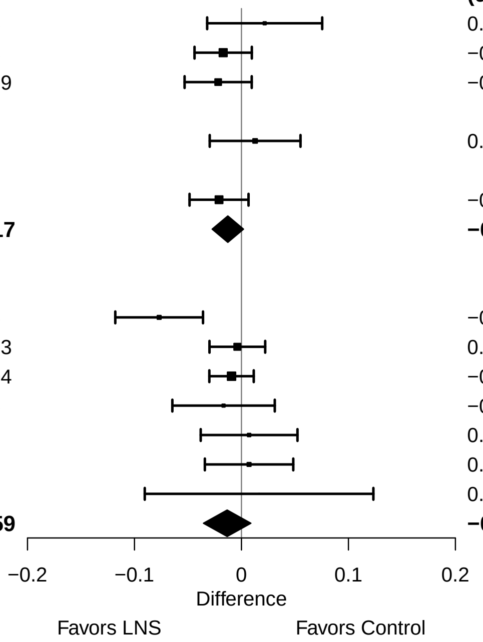

Supplemental figure 6I: Motor lowest decile prevalence difference

6I7: Stratified by Supplement duration

Supplement duration
(p−diff = 0.553)

Country	Trial		Tool	N	N	PD (95% CI)	W
**Supplement duration − 12m or less**							
Bangladesh	JiVitA−4 (21)		BSID−III	445	143	0.02 (−0.03, 0.08)	0.07
Burkina Faso	iLiNS−Zinc (24)		DMC	746	375	−0.08 (−0.12, −0.04)	0.12
Ghana	GHANA (25)						
Ghana	iLiNS−DYAD−G (26)		KDI	302	601	0.01 (−0.03, 0.06)	0.11
Haiti	HAITI (27)						
Madagascar	MAHAY (29)		ASQI	1613	1604	−0.01 (−0.03, 0.01)	0.27
Malawi	iLiNS−DYAD−M (30)		KDI	214	436	−0.02 (−0.06, 0.03)	0.09
Malawi	iLiNS−DOSE (31)		KDI	646	221	0.01 (−0.04, 0.05)	0.10
Zimbabwe	SHINE (HIV−) (33),	LNS vs SOC	MDAT	395	382	0.01 (−0.03, 0.05)	0.19
Zimbabwe		LNS+WSH vs WSH	MDAT	446	417	−0.02 (−0.06, 0.02)	0.19
Zimbabwe	SHINE (HIV+) (34),	LNS vs SOC	MDAT	67	68	0.02 (−0.09, 0.12)	0.05
Zimbabwe		LNS+WSH vs WSH	MDAT	103	83	−0.09 (−0.17, 0.00)	0.05
**I^2 = 0.51, Tau^2 = 0.00**				**4977**	**4330**	**−0.01 (−0.03, 0.01)**	
**Supplement duration − > 12m**							
Bangladesh	RDNS (22)		DMC	1556	753	−0.02 (−0.04, 0.01)	0.21
Bangladesh	WASH−B (23)		EASQ	1074	3279	−0.02 (−0.04, 0.00)	0.28
Kenya	WASH−B (28)		EASQ	1362	4745	−0.01 (−0.03, 0.00)	0.31
Mali	PROMIS CS (32)		DMC	902	921	−0.02 (−0.05, 0.01)	0.20
**I^2 = 0.00, Tau^2 = 0.00**				**4894**	**9698**	**−0.02 (−0.03, −0.01)**	

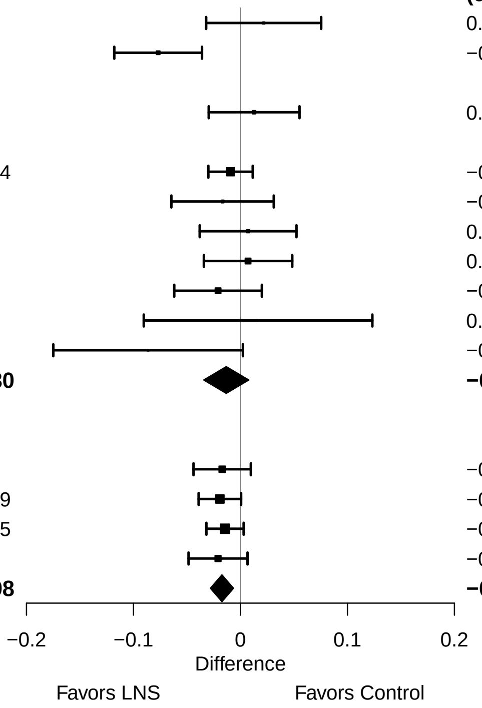

Supplemental figure 6I: Motor lowest decile prevalence difference

6I8: Stratified by Frequency of contact

Frequency of contact
(p−diff = 0.780)

Country	Trial	Tool	N	N	PD (95% CI)	W
**Frequency of contact − Monthly**						
Bangladesh	RDNS (22)	DMC	1556	753	−0.02 (−0.04, 0.01)	0.17
Haiti	HAITI (27)					
Kenya	WASH−B (28)	EASQ	1362	4745	−0.01 (−0.03, 0.00)	0.26
Madagascar	MAHAY (29)	ASQI	1613	1604	−0.01 (−0.03, 0.01)	0.22
Mali	PROMIS CS (32)	DMC	902	921	−0.02 (−0.05, 0.01)	0.16
Zimbabwe	SHINE (HIV−) (33), LNS vs SOC	MDAT	395	382	0.01 (−0.03, 0.05)	0.15
Zimbabwe	LNS+WSH vs WSH	MDAT	446	417	−0.02 (−0.06, 0.02)	0.15
Zimbabwe	SHINE (HIV+) (34), LNS vs SOC	MDAT	67	68	0.02 (−0.09, 0.12)	0.04
Zimbabwe	LNS+WSH vs WSH	MDAT	103	83	−0.09 (−0.17, 0.00)	0.04
**I² = 0.00, Tau² = 0.00**			**6444**	**8973**	**−0.01 (−0.02, 0.00)**	
**Frequency of contact − Weekly**						
Bangladesh	JiVitA−4 (21)	BSID−III	445	143	0.02 (−0.03, 0.08)	0.10
Bangladesh	WASH−B (23)	EASQ	1074	3279	−0.02 (−0.04, 0.00)	0.37
Burkina Faso	iLiNS−Zinc (24)	DMC	746	375	−0.08 (−0.12, −0.04)	0.15
Ghana	GHANA (25)					
Ghana	iLiNS−DYAD−G (26)	KDI	302	601	0.01 (−0.03, 0.06)	0.14
Malawi	iLiNS−DYAD−M (30)	KDI	214	436	−0.02 (−0.06, 0.03)	0.12
Malawi	iLiNS−DOSE (31)	KDI	646	221	0.01 (−0.04, 0.05)	0.13
**I² = 0.62, Tau² = 0.00**			**3427**	**5055**	**−0.01 (−0.04, 0.01)**	

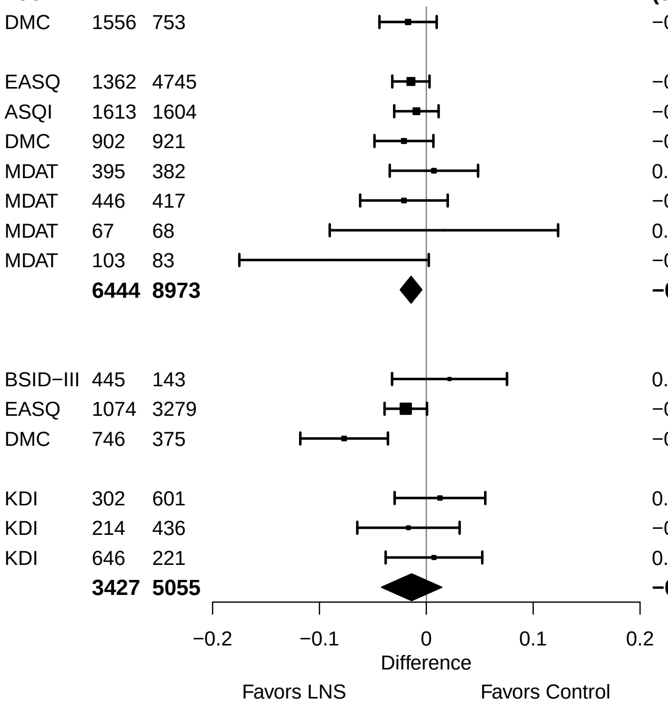

Supplemental figure 6I: Motor lowest decile prevalence difference

6I9: Stratified by Average SQ-LNS compliance

Average SQ−LNS compliance
(p−diff = 0.342)

Country	Trial		Tool	N	N	PD (95% CI)	W
**Average SQ−LNS compliance − Low**							
Ghana	iLiNS−DYAD−G (26)		KDI	302	601	0.01 (−0.03, 0.06)	0.15
Malawi	iLiNS−DYAD−M (30)		KDI	214	436	−0.02 (−0.06, 0.03)	0.13
Malawi	iLiNS−DOSE (31)		KDI	646	221	0.01 (−0.04, 0.05)	0.14
Mali	PROMIS CS (32)		DMC	902	921	−0.02 (−0.05, 0.01)	0.26
Zimbabwe	SHINE (HIV−) (33),	LNS vs SOC	MDAT	395	382	0.01 (−0.03, 0.05)	0.24
Zimbabwe		LNS+WSH vs WSH	MDAT	446	417	−0.02 (−0.06, 0.02)	0.24
Zimbabwe	SHINE (HIV+) (34),	LNS vs SOC	MDAT	67	68	0.02 (−0.09, 0.12)	0.08
Zimbabwe		LNS+WSH vs WSH	MDAT	103	83	−0.09 (−0.17, 0.00)	0.08
**I² = 0.00, Tau² = 0.00**				**3075**	**3129**	**−0.01 (−0.03, 0.01)**	
**Average SQ−LNS compliance − High**							
Bangladesh	JiVitA−4 (21)		BSID−III	445	143	0.02 (−0.03, 0.08)	0.09
Bangladesh	RDNS (22)		DMC	1556	753	−0.02 (−0.04, 0.01)	0.21
Bangladesh	WASH−B (23)		EASQ	1074	3279	−0.02 (−0.04, 0.00)	0.27
Burkina Faso	iLiNS−Zinc (24)		DMC	746	375	−0.08 (−0.12, −0.04)	0.13
Ghana	GHANA (25)						
Haiti	HAITI (27)						
Kenya	WASH−B (28)		EASQ	1362	4745	−0.01 (−0.03, 0.00)	0.29
**I² = 0.61, Tau² = 0.00**				**5183**	**9295**	**−0.02 (−0.05, 0.01)**	

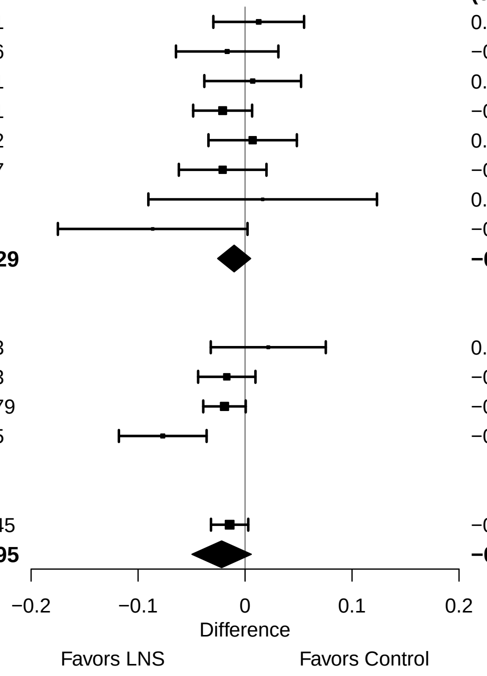

Supplemental figure 6J: Mean difference in gross motor z-score

6J1: Stratified by Geographic region

Geographic region
(p−diff = 0.929)

Country	Trial		Tool	N	N	MD (95% CI)	W
**Geographic region − SEAR**							
Bangladesh	JiVitA−4 (21)		BSID−III	445	143	−0.10 (−0.25, 0.05)	0.22
Bangladesh	RDNS (22)		DMC	1570	758	0.08 (0.02, 0.15)	0.40
Bangladesh	WASH−B (23)		EASQ	1074	3279	0.10 (0.04, 0.17)	0.38
**I² = 0.66, Tau² = 0.01**				**3089**	**4180**	**0.05 (−0.07, 0.16)**	
**Geographic region − AFR**							
Burkina Faso	iLiNS−Zinc (24)		DMC				
Ghana	GHANA (25)						
Ghana	iLiNS−DYAD−G (26)		KDI	302	601	0.01 (−0.13, 0.15)	0.12
Kenya	WASH−B (28)		EASQ	1362	4745	0.01 (−0.05, 0.07)	0.19
Madagascar	MAHAY (29)		ASQI	1613	1604	0.08 (−0.08, 0.23)	0.10
Malawi	iLiNS−DYAD−M (30)		KDI	214	436	−0.03 (−0.19, 0.13)	0.10
Malawi	iLiNS−DOSE (31)		KDI	646	221	0.00 (−0.15, 0.16)	0.11
Mali	PROMIS CS (32)		DMC	940	957	0.09 (0.00, 0.17)	0.17
Zimbabwe	SHINE (HIV−) (33),	LNS vs SOC	MDAT	395	382	0.03 (−0.13, 0.18)	0.14
Zimbabwe		LNS+WSH vs WSH	MDAT	446	417	0.16 (0.00, 0.32)	0.14
Zimbabwe	SHINE (HIV+) (34),	LNS vs SOC	MDAT	67	68	0.14 (−0.14, 0.42)	0.08
Zimbabwe		LNS+WSH vs WSH	MDAT	103	83	0.48 (0.24, 0.72)	0.08
**I² = 0.50, Tau² = 0.01**				**6088**	**9514**	**0.06 (−0.01, 0.14)**	

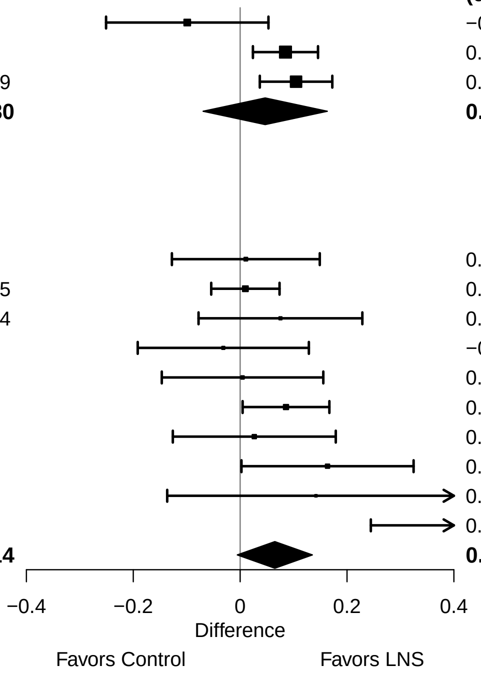

Supplemental figure 6J: Mean difference in gross motor z-score

6J2: Stratified by Stunting burden

Stunting burden
(p−diff = 0.014)

Stunting burden − Less than 35%

**Country**	**Trial**	**Tool**	**N**	**N**	**MD (95% CI)**	**W**
Ghana	GHANA (25)					
Ghana	iLiNS−DYAD−G (26)	KDI	302	601	0.01 (−0.13, 0.15)	0.29
Haiti	HAITI (27)					
Kenya	WASH−B (28)	EASQ	1362	4745	0.01 (−0.05, 0.07)	0.46
Malawi	iLiNS−DYAD−M (30)	KDI	214	436	−0.03 (−0.19, 0.13)	0.25
**I² = 0.00, Tau² = 0.00**			**1878**	**5782**	**0.01 (−0.05, 0.06)**	

Stunting burden − More than 35%

Country	Trial	Tool	N	N	MD (95% CI)	W
Bangladesh	JiVitA−4 (21)	BSID−III	445	143	−0.10 (−0.25, 0.05)	0.10
Bangladesh	RDNS (22)	DMC	1570	758	0.08 (0.02, 0.15)	0.18
Bangladesh	WASH−B (23)	EASQ	1074	3279	0.10 (0.04, 0.17)	0.17
Burkina Faso	iLiNS−Zinc (24)	DMC				
Madagascar	MAHAY (29)	ASQI	1613	1604	0.08 (−0.08, 0.23)	0.10
Malawi	iLiNS−DOSE (31)	KDI	646	221	0.00 (−0.15, 0.16)	0.10
Mali	PROMIS CS (32)	DMC	940	957	0.09 (0.00, 0.17)	0.16
Zimbabwe	SHINE (HIV−) (33), LNS vs SOC	MDAT	395	382	0.03 (−0.13, 0.18)	0.13
Zimbabwe	LNS+WSH vs WSH	MDAT	446	417	0.16 (0.00, 0.32)	0.13
Zimbabwe	SHINE (HIV+) (34), LNS vs SOC	MDAT	67	68	0.14 (−0.14, 0.42)	0.08
Zimbabwe	LNS+WSH vs WSH	MDAT	103	83	0.48 (0.24, 0.72)	0.08
**I² = 0.51, Tau² = 0.01**			**7299**	**7912**	**0.08 (0.01, 0.16)**	

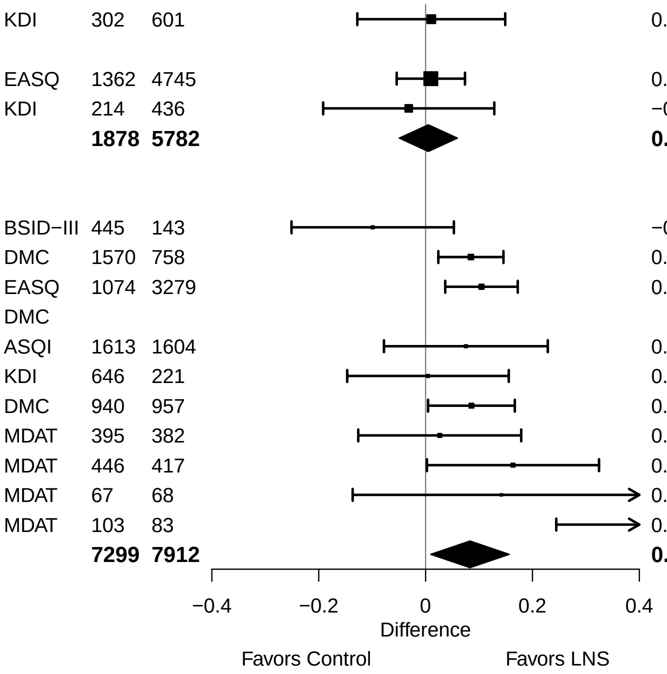

Supplemental figure 6J: Mean difference in gross motor z-score

6J3: Stratified by Malaria prevalence

Malaria prevalence
(p−diff = 0.387)

Country	Trial	Tool	N	N	MD (95% CI)	W
**Malaria prevalence − Less than 10%**						
Bangladesh	JiVitA−4 (21)	BSID−III	445	143	−0.10 (−0.25, 0.05)	0.11
Bangladesh	RDNS (22)	DMC	1570	758	0.08 (0.02, 0.15)	0.19
Bangladesh	WASH−B (23)	EASQ	1074	3279	0.10 (0.04, 0.17)	0.18
Haiti	HAITI (27)					
Kenya	WASH−B (28)	EASQ	1362	4745	0.01 (−0.05, 0.07)	0.19
Madagascar	MAHAY (29)	ASQI	1613	1604	0.08 (−0.08, 0.23)	0.11
Zimbabwe	SHINE (HIV−) (33), LNS vs SOC	MDAT	395	382	0.03 (−0.13, 0.18)	0.14
Zimbabwe	LNS+WSH vs WSH	MDAT	446	417	0.16 (0.00, 0.32)	0.14
Zimbabwe	SHINE (HIV+) (34), LNS vs SOC	MDAT	67	68	0.14 (−0.14, 0.42)	0.08
Zimbabwe	LNS+WSH vs WSH	MDAT	103	83	0.48 (0.24, 0.72)	0.08
**I² = 0.66, Tau² = 0.01**			**7075**	**11479**	**0.08 (−0.01, 0.17)**	
**Malaria prevalence − At least 10%**						
Burkina Faso	iLiNS−Zinc (24)	DMC				
Ghana	GHANA (25)					
Ghana	iLiNS−DYAD−G (26)	KDI	302	601	0.01 (−0.13, 0.15)	0.24
Malawi	iLiNS−DYAD−M (30)	KDI	214	436	−0.03 (−0.19, 0.13)	0.20
Malawi	iLiNS−DOSE (31)	KDI	646	221	0.00 (−0.15, 0.16)	0.22
Mali	PROMIS CS (32)	DMC	940	957	0.09 (0.00, 0.17)	0.34
**I² = 0.00, Tau² = 0.00**			**2102**	**2215**	**0.04 (−0.02, 0.10)**	

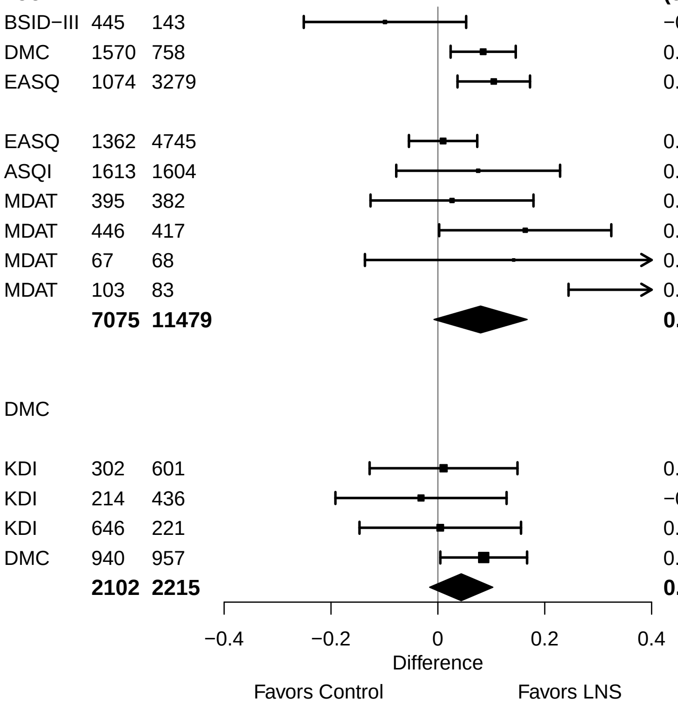

Supplemental figure 6J: Mean difference in gross motor z-score

6J4: Stratified by Anemia prevalence

Anemia prevalence
(p−diff = 0.558)

**Anemia prevalence − High**<br>**Country**	**Trial**	**Tool**	**N**	**N**	**MD (95% CI)**	**W**
Burkina Faso	iLiNS−Zinc (24)	DMC				
Ghana	GHANA (25)					
Haiti	HAITI (27)					
Malawi	iLiNS−DYAD−M (30)	KDI	214	436	−0.03 (−0.19, 0.13)	0.27
Malawi	iLiNS−DOSE (31)	KDI	646	221	0.00 (−0.15, 0.16)	0.28
Mali	PROMIS CS (32)	DMC	940	957	0.09 (0.00, 0.17)	0.45
**I² = 0.04, Tau² = 0.00**			**1800**	**1614**	**0.05 (−0.02, 0.12)**	
**Anemia prevalence − Moderate**						
Bangladesh	JiVitA−4 (21)	BSID−III	445	143	−0.10 (−0.25, 0.05)	0.10
Bangladesh	RDNS (22)	DMC	1570	758	0.08 (0.02, 0.15)	0.17
Bangladesh	WASH−B (23)	EASQ	1074	3279	0.10 (0.04, 0.17)	0.17
Ghana	iLiNS−DYAD−G (26)	KDI	302	601	0.01 (−0.13, 0.15)	0.11
Kenya	WASH−B (28)	EASQ	1362	4745	0.01 (−0.05, 0.07)	0.17
Madagascar	MAHAY (29)	ASQI	1613	1604	0.08 (−0.08, 0.23)	0.09
Zimbabwe	SHINE (HIV−) (33), LNS vs SOC	MDAT	395	382	0.03 (−0.13, 0.18)	0.13
Zimbabwe	LNS+WSH vs WSH	MDAT	446	417	0.16 (0.00, 0.32)	0.13
Zimbabwe	SHINE (HIV+) (34), LNS vs SOC	MDAT	67	68	0.14 (−0.14, 0.42)	0.07
Zimbabwe	LNS+WSH vs WSH	MDAT	103	83	0.48 (0.24, 0.72)	0.07
**I² = 0.62, Tau² = 0.01**			**7377**	**12080**	**0.07 (0.00, 0.15)**	

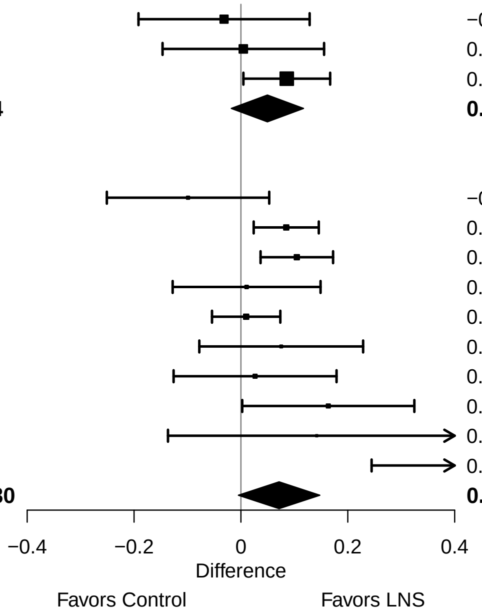

Supplemental figure 6J: Mean difference in gross motor z-score

6J5: Stratified by Source water quality

Source water quality
(p−diff = 0.777)

Country	Trial	Tool	N	N	MD (95% CI)	W
**Source water quality − Improved**						
Bangladesh	JiVitA−4 (21)	BSID−III	445	143	−0.10 (−0.25, 0.05)	0.12
Bangladesh	RDNS (22)	DMC	1570	758	0.08 (0.02, 0.15)	0.33
Bangladesh	WASH−B (23)	EASQ	528	1099	0.19 (0.09, 0.30)	0.19
Ghana	GHANA (25)					
Ghana	iLiNS−DYAD−G (26)	KDI	302	601	0.01 (−0.13, 0.15)	0.13
Haiti	HAITI (27)					
Malawi	iLiNS−DYAD−M (30)	KDI	214	436	−0.03 (−0.19, 0.13)	0.11
Malawi	iLiNS−DOSE (31)	KDI	646	221	0.00 (−0.15, 0.16)	0.12
**I² = 0.62, Tau² = 0.01**			**3705**	**3258**	**0.04 (−0.04, 0.12)**	
**Source water quality − Unimproved**						
Burkina Faso	iLiNS−Zinc (24)	DMC				
Kenya	WASH−B (28)	EASQ	649	2073	0.03 (−0.06, 0.13)	0.29
Madagascar	MAHAY (29)	ASQI	1613	1604	0.08 (−0.08, 0.23)	0.15
Mali	PROMIS CS (32)	DMC	940	957	0.09 (0.00, 0.17)	0.35
Zimbabwe	SHINE (HIV−) (33)	MDAT	395	382	0.03 (−0.13, 0.18)	0.15
Zimbabwe	SHINE (HIV+) (34)	MDAT	67	68	0.14 (−0.14, 0.42)	0.05
**I² = 0.00, Tau² = 0.00**			**3664**	**5084**	**0.06 (0.01, 0.12)**	

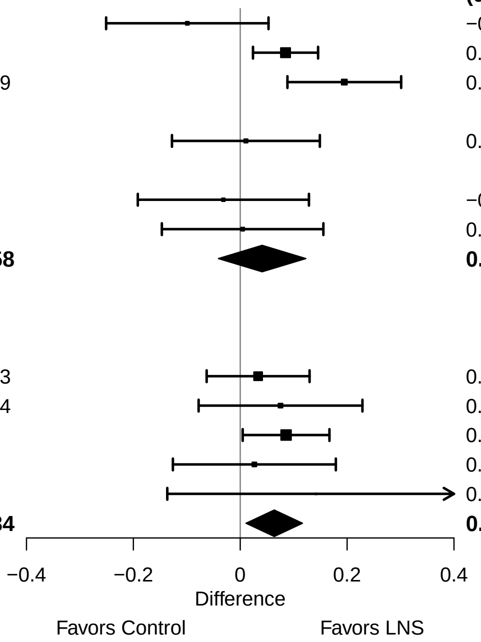

Supplemental figure 6J: Mean difference in gross motor z-score

6J6: Stratified by Sanitation

Sanitation
(p−diff = 0.276)

Sanitation − Improved

Country	Trial	Tool	N	N	MD (95% CI)	W
Bangladesh	JiVitA−4 (21)	BSID−III	445	143	−0.10 (−0.25, 0.05)	0.11
Bangladesh	RDNS (22)	DMC	1570	758	0.08 (0.02, 0.15)	0.32
Bangladesh	WASH−B (23)	EASQ	528	1099	0.19 (0.09, 0.30)	0.19
Ghana	GHANA (25)					
Ghana	iLiNS−DYAD−G (26)	KDI	302	601	0.01 (−0.13, 0.15)	0.13
Haiti	HAITI (27)					
Mali	PROMIS CS (32)	DMC	940	957	0.09 (0.00, 0.17)	0.25
**I² = 0.63, Tau² = 0.01**			**3785**	**3558**	**0.07 (−0.02, 0.16)**	

Sanitation − Unimproved

Country	Trial	Tool	N	N	MD (95% CI)	W
Burkina Faso	iLiNS−Zinc (24)	DMC				
Kenya	WASH−B (28)	EASQ	649	2073	0.03 (−0.06, 0.13)	0.30
Madagascar	MAHAY (29)	ASQI	1613	1604	0.08 (−0.08, 0.23)	0.16
Malawi	iLiNS−DYAD−M (30)	KDI	214	436	−0.03 (−0.19, 0.13)	0.15
Malawi	iLiNS−DOSE (31)	KDI	646	221	0.00 (−0.15, 0.16)	0.16
Zimbabwe	SHINE (HIV−) (33)	MDAT	395	382	0.03 (−0.13, 0.18)	0.16
Zimbabwe	SHINE (HIV+) (34)	MDAT	67	68	0.14 (−0.14, 0.42)	0.06
**I² = 0.00, Tau² = 0.00**			**3584**	**4784**	**0.03 (−0.03, 0.09)**	

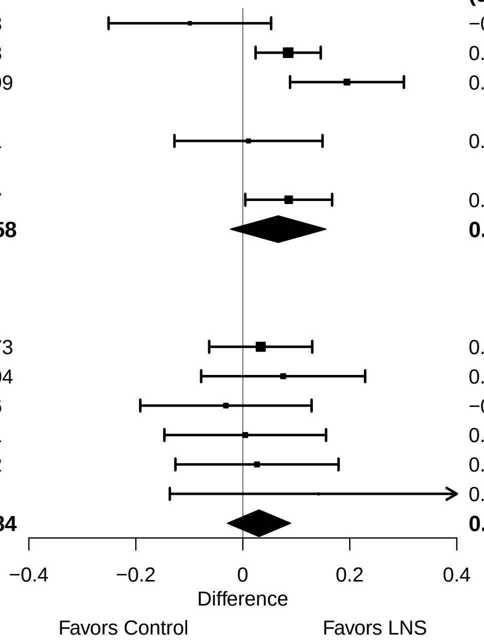

Supplemental figure 6J: Mean difference in gross motor z-score

6J7: Stratified by Supplement duration

Supplement duration
(p−diff = 0.643)

Supplement duration − 12m or less

Country	Trial	Tool	N	N	MD (95% CI)	W
Bangladesh	JiVitA−4 (21)	BSID−III	445	143	−0.10 (−0.25, 0.05)	0.14
Burkina Faso	iLiNS−Zinc (24)	DMC				
Ghana	GHANA (25)					
Ghana	iLiNS−DYAD−G (26)	KDI	302	601	0.01 (−0.13, 0.15)	0.15
Haiti	HAITI (27)					
Madagascar	MAHAY (29)	ASQI	1613	1604	0.08 (−0.08, 0.23)	0.14
Malawi	iLiNS−DYAD−M (30)	KDI	214	436	−0.03 (−0.19, 0.13)	0.13
Malawi	iLiNS−DOSE (31)	KDI	646	221	0.00 (−0.15, 0.16)	0.14
Zimbabwe	SHINE (HIV−) (33), LNS vs SOC	MDAT	395	382	0.03 (−0.13, 0.18)	0.18
Zimbabwe	LNS+WSH vs WSH	MDAT	446	417	0.16 (0.00, 0.32)	0.18
Zimbabwe	SHINE (HIV+) (34), LNS vs SOC	MDAT	67	68	0.14 (−0.14, 0.42)	0.11
Zimbabwe	LNS+WSH vs WSH	MDAT	103	83	0.48 (0.24, 0.72)	0.11
**I^2 = 0.61, Tau^2 = 0.01**			**4231**	**3955**	**0.05 (−0.05, 0.15)**	

Supplement duration − > 12m

Country	Trial	Tool	N	N	MD (95% CI)	W
Bangladesh	RDNS (22)	DMC	1570	758	0.08 (0.02, 0.15)	0.26
Bangladesh	WASH−B (23)	EASQ	1074	3279	0.10 (0.04, 0.17)	0.25
Kenya	WASH−B (28)	EASQ	1362	4745	0.01 (−0.05, 0.07)	0.26
Mali	PROMIS CS (32)	DMC	940	957	0.09 (0.00, 0.17)	0.23
**I^2 = 0.37, Tau^2 = 0.00**			**4946**	**9739**	**0.07 (0.03, 0.11)**	

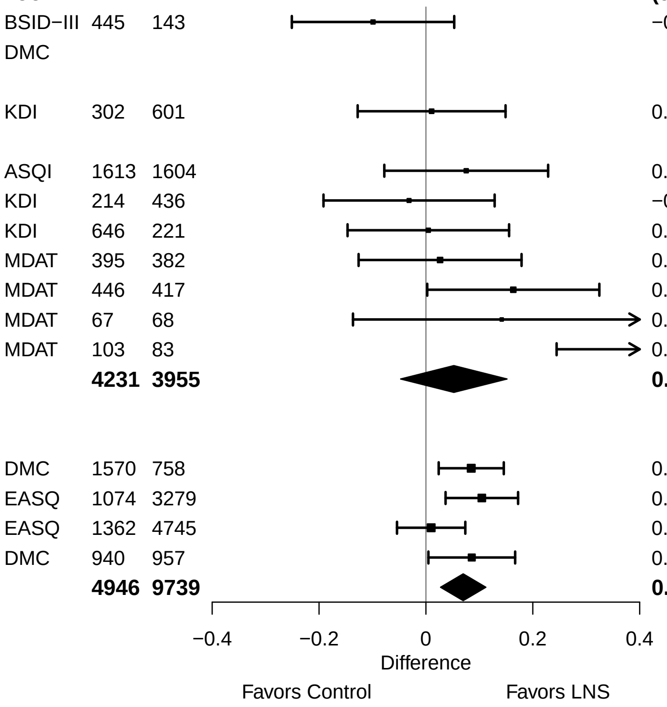

Supplemental figure 6J: Mean difference in gross motor z-score

6J8: Stratified by Frequency of contact

Frequency of contact
(p−diff = 0.150)

Country	Trial	Tool	N	N	MD (95% CI)	W
**Frequency of contact − Monthly**						
Bangladesh	RDNS (22)	DMC	1570	758	0.08 (0.02, 0.15)	0.22
Haiti	HAITI (27)					
Kenya	WASH−B (28)	EASQ	1362	4745	0.01 (−0.05, 0.07)	0.21
Madagascar	MAHAY (29)	ASQI	1613	1604	0.08 (−0.08, 0.23)	0.12
Mali	PROMIS CS (32)	DMC	940	957	0.09 (0.00, 0.17)	0.19
Zimbabwe	SHINE (HIV−) (33), LNS vs SOC	MDAT	395	382	0.03 (−0.13, 0.18)	0.16
Zimbabwe	LNS+WSH vs WSH	MDAT	446	417	0.16 (0.00, 0.32)	0.16
Zimbabwe	SHINE (HIV+) (34), LNS vs SOC	MDAT	67	68	0.14 (−0.14, 0.42)	0.09
Zimbabwe	LNS+WSH vs WSH	MDAT	103	83	0.48 (0.24, 0.72)	0.09
**I² = 0.59, Tau² = 0.01**			**6496**	**9014**	**0.10 (0.02, 0.17)**	
**Frequency of contact − Weekly**						
Bangladesh	JiVitA−4 (21)	BSID−III	445	143	−0.10 (−0.25, 0.05)	0.17
Bangladesh	WASH−B (23)	EASQ	1074	3279	0.10 (0.04, 0.17)	0.30
Burkina Faso	iLiNS−Zinc (24)	DMC				
Ghana	GHANA (25)					
Ghana	iLiNS−DYAD−G (26)	KDI	302	601	0.01 (−0.13, 0.15)	0.19
Malawi	iLiNS−DYAD−M (30)	KDI	214	436	−0.03 (−0.19, 0.13)	0.16
Malawi	iLiNS−DOSE (31)	KDI	646	221	0.00 (−0.15, 0.16)	0.17
**I² = 0.49, Tau² = 0.00**			**2681**	**4680**	**0.02 (−0.05, 0.09)**	

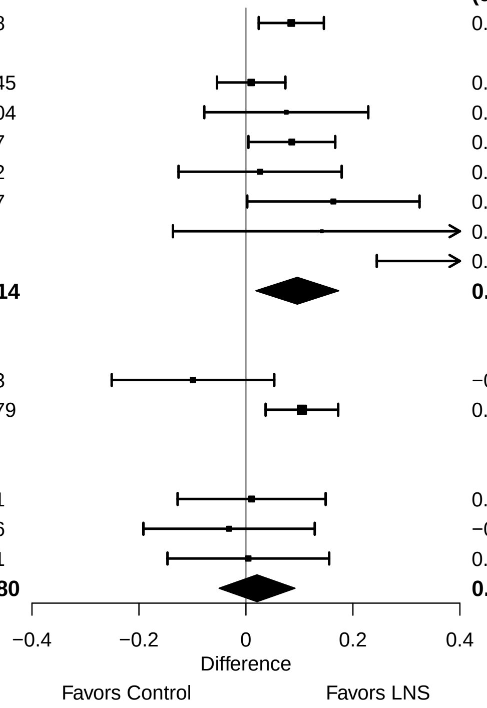

Supplemental figure 6J: Mean difference in gross motor z-score

6J9: Stratified by Average SQ-LNS compliance

Average SQ−LNS compliance
(p−diff = 0.525)

Average SQ−LNS compliance − Low

Country	Trial	Tool	N	N	MD (95% CI)	W
Ghana	iLiNS−DYAD−G (26)	KDI	302	601	0.01 (−0.13, 0.15)	0.16
Malawi	iLiNS−DYAD−M (30)	KDI	214	436	−0.03 (−0.19, 0.13)	0.14
Malawi	iLiNS−DOSE (31)	KDI	646	221	0.00 (−0.15, 0.16)	0.15
Mali	PROMIS CS (32)	DMC	940	957	0.09 (0.00, 0.17)	0.23
Zimbabwe	SHINE (HIV−) (33), LNS vs SOC	MDAT	395	382	0.03 (−0.13, 0.18)	0.19
Zimbabwe	LNS+WSH vs WSH	MDAT	446	417	0.16 (0.00, 0.32)	0.19
Zimbabwe	SHINE (HIV+) (34), LNS vs SOC	MDAT	67	68	0.14 (−0.14, 0.42)	0.12
Zimbabwe	LNS+WSH vs WSH	MDAT	103	83	0.48 (0.24, 0.72)	0.12
**I² = 0.56, Tau² = 0.01**			**3113**	**3165**	**0.08 (−0.02, 0.18)**	

Average SQ−LNS compliance − High

Country	Trial	Tool	N	N	MD (95% CI)	W
Bangladesh	JiVitA−4 (21)	BSID−III	445	143	−0.10 (−0.25, 0.05)	0.17
Bangladesh	RDNS (22)	DMC	1570	758	0.08 (0.02, 0.15)	0.28
Bangladesh	WASH−B (23)	EASQ	1074	3279	0.10 (0.04, 0.17)	0.27
Burkina Faso	iLiNS−Zinc (24)	DMC				
Ghana	GHANA (25)					
Haiti	HAITI (27)					
Kenya	WASH−B (28)	EASQ	1362	4745	0.01 (−0.05, 0.07)	0.28
**I² = 0.66, Tau² = 0.00**			**4451**	**8925**	**0.04 (−0.04, 0.12)**	

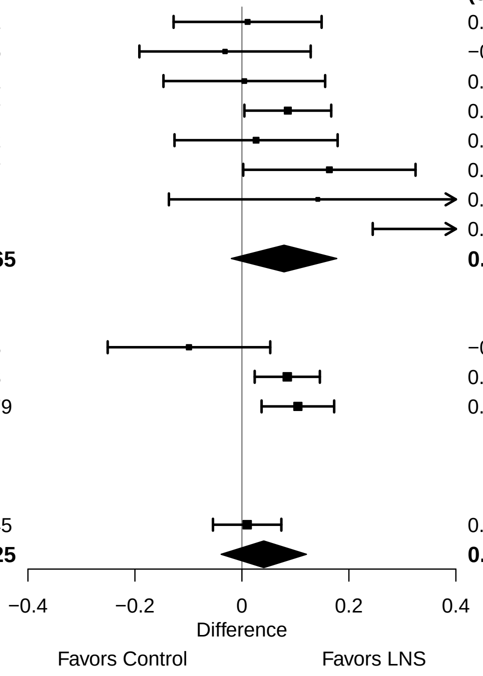

Supplemental figure 6K: Mean difference in fine motor z-score

6K1: Stratified by Geographic region (insufficient comparisons)

Supplemental figure 6K: Mean difference in fine motor z-score

6K2: Stratified by Stunting burden (insufficient comparisons)

Supplemental figure 6K: Mean difference in fine motor z-score

6K3: Stratified by Malaria prevalence

Malaria prevalence
(p−diff = 0.626)

Country	Trial	Tool	N	N	MD (95% CI)	W
**Malaria prevalence − Less than 10%**						
Bangladesh	JiVitA−4 (21)	BSID−III	445	143	0.14 (−0.05, 0.34)	0.11
Bangladesh	RDNS (22)	DMC	1641	804	0.10 (−0.01, 0.20)	0.35
Bangladesh	WASH−B (23)	EASQ				
Haiti	HAITI (27)					
Kenya	WASH−B (28)	EASQ				
Madagascar	MAHAY (29)	ASQI	1613	1604	0.05 (−0.17, 0.26)	0.08
Zimbabwe	SHINE (HIV−) (33), LNS vs SOC	MDAT	395	382	−0.03 (−0.18, 0.11)	0.38
Zimbabwe	LNS+WSH vs WSH	MDAT	446	417	0.17 (0.03, 0.31)	0.38
Zimbabwe	SHINE (HIV+) (34), LNS vs SOC	MDAT	67	68	−0.04 (−0.40, 0.31)	0.08
Zimbabwe	LNS+WSH vs WSH	MDAT	103	83	0.31 (0.03, 0.59)	0.08
**I^2 = 0.00, Tau^2 = 0.00**			**4710**	**3501**	**0.10 (0.03, 0.16)**	
**Malaria prevalence − At least 10%**						
Burkina Faso	iLiNS−Zinc (24)	DMC				
Ghana	GHANA (25)					
Ghana	iLiNS−DYAD−G (26)	KDI	302	601	0.04 (−0.09, 0.17)	0.36
Malawi	iLiNS−DYAD−M (30)	KDI	214	436	0.08 (−0.08, 0.24)	0.24
Malawi	iLiNS−DOSE (31)	KDI	646	221	0.09 (−0.06, 0.24)	0.26
Mali	PROMIS CS (32)	DMC	905	924	0.11 (−0.11, 0.32)	0.13
**I^2 = 0.00, Tau^2 = 0.00**			**2067**	**2182**	**0.07 (−0.01, 0.15)**	

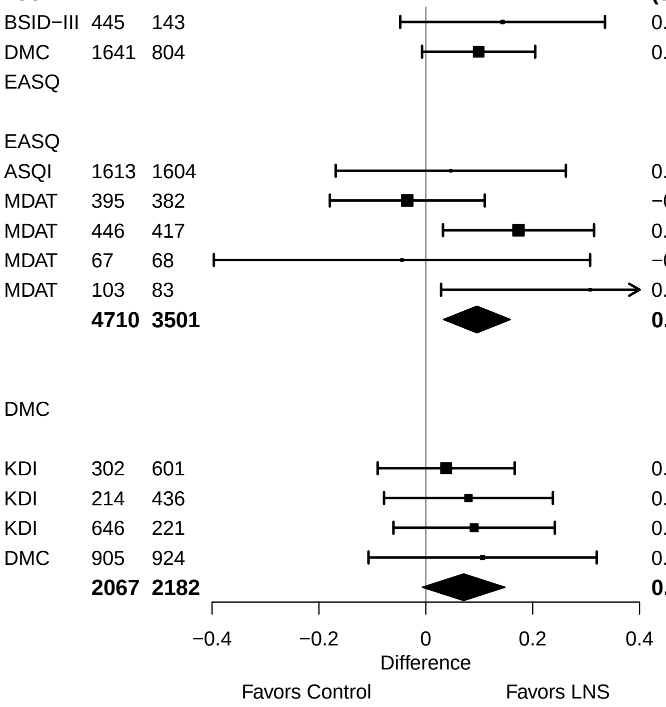

Supplemental figure 6K: Mean difference in fine motor z-score

6K4: Stratified by Anemia prevalence

Anemia prevalence
(p−diff = 0.928)

Country	Trial	Tool	N	N	MD (95% CI)	W
**Anemia prevalence − High**						
Burkina Faso	iLiNS−Zinc (24)	DMC				
Ghana	GHANA (25)					
Haiti	HAITI (27)					
Malawi	iLiNS−DYAD−M (30)	KDI	214	436	0.08 (−0.08, 0.24)	0.38
Malawi	iLiNS−DOSE (31)	KDI	646	221	0.09 (−0.06, 0.24)	0.41
Mali	PROMIS CS (32)	DMC	905	924	0.11 (−0.11, 0.32)	0.21
**I² = 0.00, Tau² = 0.00**			**1765**	**1581**	**0.09 (−0.01, 0.19)**	
**Anemia prevalence − Moderate**						
Bangladesh	JiVitA−4 (21)	BSID−III	445	143	0.14 (−0.05, 0.34)	0.09
Bangladesh	RDNS (22)	DMC	1641	804	0.10 (−0.01, 0.20)	0.28
Bangladesh	WASH−B (23)	EASQ				
Ghana	iLiNS−DYAD−G (26)	KDI	302	601	0.04 (−0.09, 0.17)	0.19
Kenya	WASH−B (28)	EASQ				
Madagascar	MAHAY (29)	ASQI	1613	1604	0.05 (−0.17, 0.26)	0.07
Zimbabwe	SHINE (HIV−) (33), LNS vs SOC	MDAT	395	382	−0.03 (−0.18, 0.11)	0.31
Zimbabwe	LNS+WSH vs WSH	MDAT	446	417	0.17 (0.03, 0.31)	0.31
Zimbabwe	SHINE (HIV+) (34), LNS vs SOC	MDAT	67	68	−0.04 (−0.40, 0.31)	0.06
Zimbabwe	LNS+WSH vs WSH	MDAT	103	83	0.31 (0.03, 0.59)	0.06
**I² = 0.00, Tau² = 0.00**			**5012**	**4102**	**0.08 (0.03, 0.14)**	

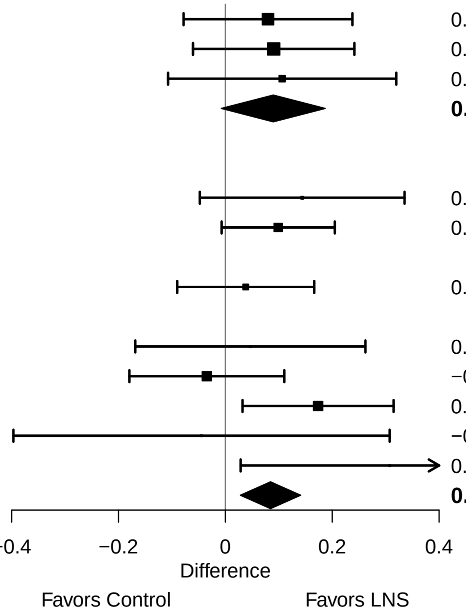

Supplemental figure 6K: Mean difference in fine motor z-score

6K5: Stratified by Source water quality

Source water quality
(p−diff = 0.234)

Country	Trial	Tool	N	N	MD (95% CI)	W
**Source water quality − Improved**						
Bangladesh	JiVitA−4 (21)	BSID−III	445	143	0.14 (−0.05, 0.34)	0.10
Bangladesh	RDNS (22)	DMC	1641	804	0.10 (−0.01, 0.20)	0.34
Bangladesh	WASH−B (23)	EASQ				
Ghana	GHANA (25)					
Ghana	iLiNS−DYAD−G (26)	KDI	302	601	0.04 (−0.09, 0.17)	0.23
Haiti	HAITI (27)					
Malawi	iLiNS−DYAD−M (30)	KDI	214	436	0.08 (−0.08, 0.24)	0.15
Malawi	iLiNS−DOSE (31)	KDI	646	221	0.09 (−0.06, 0.24)	0.17
**I² = 0.00, Tau² = 0.00**			**3248**	**2205**	**0.09 (0.02, 0.15)**	
**Source water quality − Unimproved**						
Burkina Faso	iLiNS−Zinc (24)	DMC				
Kenya	WASH−B (28)	EASQ				
Madagascar	MAHAY (29)	ASQI	1613	1604	0.05 (−0.17, 0.26)	0.22
Mali	PROMIS CS (32)	DMC	905	924	0.11 (−0.11, 0.32)	0.22
Zimbabwe	SHINE (HIV−) (33)	MDAT	395	382	−0.03 (−0.18, 0.11)	0.48
Zimbabwe	SHINE (HIV+) (34)	MDAT	67	68	−0.04 (−0.40, 0.31)	0.08
**I² = 0.00, Tau² = 0.00**			**2980**	**2978**	**0.01 (−0.09, 0.11)**	

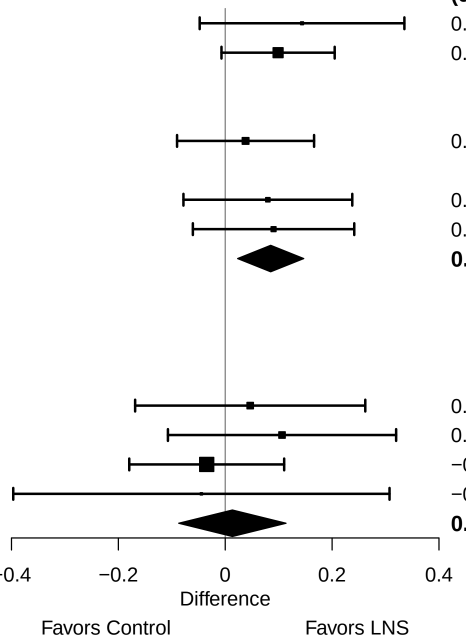

Supplemental figure 6K: Mean difference in fine motor z-score

6K6: Stratified by Sanitation

Sanitation
(p−diff = 0.364)

Country	Trial	Tool	N	N	MD (95% CI)	W
**Sanitation − Improved**						
Bangladesh	JiVitA−4 (21)	BSID−III	445	143	0.14 (−0.05, 0.34)	0.14
Bangladesh	RDNS (22)	DMC	1641	804	0.10 (−0.01, 0.20)	0.45
Bangladesh	WASH−B (23)	EASQ				
Ghana	GHANA (25)					
Ghana	iLiNS−DYAD−G (26)	KDI	302	601	0.04 (−0.09, 0.17)	0.31
Haiti	HAITI (27)					
Mali	PROMIS CS (32)	DMC	905	924	0.11 (−0.11, 0.32)	0.11
**I² = 0.00, Tau² = 0.00**			**3293**	**2472**	**0.09 (0.02, 0.16)**	
**Sanitation − Unimproved**						
Burkina Faso	iLiNS−Zinc (24)	DMC				
Kenya	WASH−B (28)	EASQ				
Madagascar	MAHAY (29)	ASQI	1613	1604	0.05 (−0.17, 0.26)	0.13
Malawi	iLiNS−DYAD−M (30)	KDI	214	436	0.08 (−0.08, 0.24)	0.25
Malawi	iLiNS−DOSE (31)	KDI	646	221	0.09 (−0.06, 0.24)	0.27
Zimbabwe	SHINE (HIV−) (33)	MDAT	395	382	−0.03 (−0.18, 0.11)	0.30
Zimbabwe	SHINE (HIV+) (34)	MDAT	67	68	−0.04 (−0.40, 0.31)	0.05
**I² = 0.00, Tau² = 0.00**			**2935**	**2711**	**0.04 (−0.04, 0.12)**	

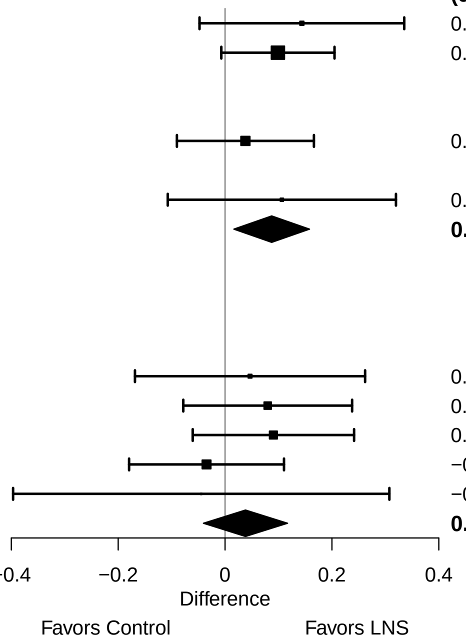

Supplemental figure 6K: Mean difference in fine motor z-score

6K7: Stratified by Supplement duration (insufficient comparisons)

Supplemental figure 6K: Mean difference in fine motor z-score

6K8: Stratified by Frequency of contact

Frequency of contact
(p−diff = 0.791)

Frequency of contact − Monthly

Country	Trial	Tool	N	N	MD (95% CI)	W
Bangladesh	RDNS (22)	DMC	1641	804	0.10 (−0.01, 0.20)	0.36
Haiti	HAITI (27)					
Kenya	WASH−B (28)	EASQ				
Madagascar	MAHAY (29)	ASQI	1613	1604	0.05 (−0.17, 0.26)	0.09
Mali	PROMIS CS (32)	DMC	905	924	0.11 (−0.11, 0.32)	0.09
Zimbabwe	SHINE (HIV−) (33), LNS vs SOC	MDAT	395	382	−0.03 (−0.18, 0.11)	0.39
Zimbabwe	LNS+WSH vs WSH	MDAT	446	417	0.17 (0.03, 0.31)	0.39
Zimbabwe	SHINE (HIV+) (34), LNS vs SOC	MDAT	67	68	−0.04 (−0.40, 0.31)	0.08
Zimbabwe	LNS+WSH vs WSH	MDAT	103	83	0.31 (0.03, 0.59)	0.08
**I² = 0.00, Tau² = 0.00**			**5170**	**4282**	**0.09 (0.03, 0.15)**	

Frequency of contact − Weekly

Country	Trial	Tool	N	N	MD (95% CI)	W
Bangladesh	JiVitA−4 (21)	BSID−III	445	143	0.14 (−0.05, 0.34)	0.16
Bangladesh	WASH−B (23)	EASQ				
Burkina Faso	iLiNS−Zinc (24)	DMC				
Ghana	GHANA (25)					
Ghana	iLiNS−DYAD−G (26)	KDI	302	601	0.04 (−0.09, 0.17)	0.35
Malawi	iLiNS−DYAD−M (30)	KDI	214	436	0.08 (−0.08, 0.24)	0.23
Malawi	iLiNS−DOSE (31)	KDI	646	221	0.09 (−0.06, 0.24)	0.25
**I² = 0.00, Tau² = 0.00**			**1607**	**1401**	**0.08 (0.00, 0.15)**	

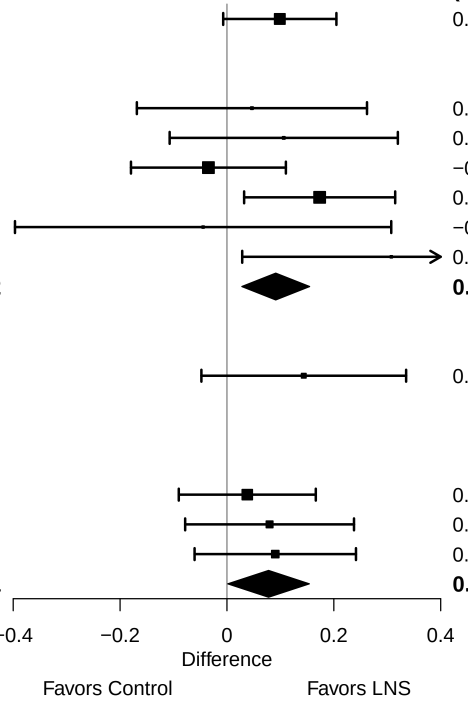

Supplemental figure 6K: Mean difference in fine motor z-score

6K9: Stratified by Average SQ-LNS compliance (insufficient comparisons)

Supplemental figure 6L: Mean difference in executive function z-score

6L1: Stratified by Geographic region (insufficient comparisons)

Supplemental figure 6L: Mean difference in executive function z-score

6L2: Stratified by Stunting burden (insufficient comparisons)

Supplemental figure 6L: Mean difference in executive function z-score

6L3: Stratified by Malaria prevalence

Malaria prevalence
(p−diff = 0.242)

Country	Trial	Tool	N	N	MD (95% CI)	W
**Malaria prevalence − Less than 10%**						
Bangladesh	JiVitA−4 (21)					
Bangladesh	RDNS (22)	A not B task	533	266	0.10 (−0.02, 0.22)	0.20
Bangladesh	WASH−B (23)	A not B task	1097	3327	0.00 (−0.07, 0.07)	0.53
Haiti	HAITI (27)					
Kenya	WASH−B (28)					
Madagascar	MAHAY (29)					
Zimbabwe	SHINE (HIV−) (33), LNS vs SOC	A not B task	370	357	−0.05 (−0.21, 0.10)	0.21
Zimbabwe	LNS+WSH vs WSH	A not B task	417	382	0.12 (−0.05, 0.29)	0.21
Zimbabwe	SHINE (HIV+) (34), LNS vs SOC	A not B task	63	57	−0.16 (−0.49, 0.16)	0.06
Zimbabwe	LNS+WSH vs WSH	A not B task	94	76	−0.05 (−0.35, 0.25)	0.06
**I² = 0.06, Tau² = 0.00**			**2574**	**4465**	**0.02 (−0.04, 0.08)**	
**Malaria prevalence − At least 10%**						
Burkina Faso	iLiNS−Zinc (24)					
Ghana	GHANA (25)					
Ghana	iLiNS−DYAD−G (26)	A not B task	290	576	0.02 (−0.12, 0.17)	0.44
Malawi	iLiNS−DYAD−M (30)	A not B task	182	369	−0.09 (−0.27, 0.09)	0.28
Malawi	iLiNS−DOSE (31)	A not B task	475	164	−0.11 (−0.29, 0.07)	0.28
Mali	PROMIS CS (32)					
**I² = 0.00, Tau² = 0.00**			**947**	**1109**	**−0.04 (−0.14, 0.05)**	

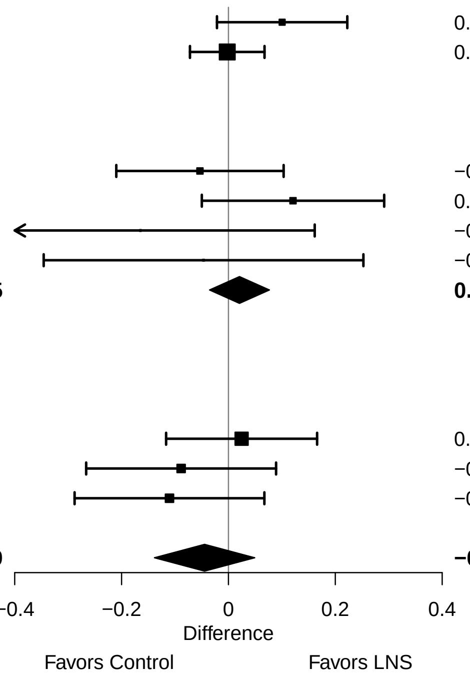

Supplemental figure 6L: Mean difference in executive function z-score

6L4: Stratified by Anemia prevalence (insufficient comparisons)

Supplemental figure 6L: Mean difference in executive function z-score

6L5: Stratified by Source water quality (insufficient comparisons)

Supplemental figure 6L: Mean difference in executive function z-score

6L6: Stratified by Sanitation

Sanitation
(p−diff = 0.012)

Country	Trial	Tool	N	N	MD (95% CI)	W
**Sanitation − Improved**						
Bangladesh	JiVitA−4 (21)					
Bangladesh	RDNS (22)	A not B task	533	266	0.10 (−0.02, 0.22)	0.32
Bangladesh	WASH−B (23)	A not B task	541	1099	0.05 (−0.05, 0.15)	0.42
Ghana	GHANA (25)					
Ghana	iLiNS−DYAD−G (26)	A not B task	290	576	0.02 (−0.12, 0.17)	0.26
Haiti	HAITI (27)					
Mali	PROMIS CS (32)					
**I² = 0.00, Tau² = 0.00**			**1364**	**1941**	**0.06 (−0.01, 0.13)**	
**Sanitation − Unimproved**						
Burkina Faso	iLiNS−Zinc (24)					
Kenya	WASH−B (28)					
Madagascar	MAHAY (29)					
Malawi	iLiNS−DYAD−M (30)	A not B task	182	369	−0.09 (−0.27, 0.09)	0.28
Malawi	iLiNS−DOSE (31)	A not B task	475	164	−0.11 (−0.29, 0.07)	0.28
Zimbabwe	SHINE (HIV−) (33)	A not B task	370	357	−0.05 (−0.21, 0.10)	0.35
Zimbabwe	SHINE (HIV+) (34)	A not B task	63	57	−0.16 (−0.49, 0.16)	0.09
**I² = 0.00, Tau² = 0.00**			**1090**	**947**	**−0.09 (−0.18, 0.01)**	

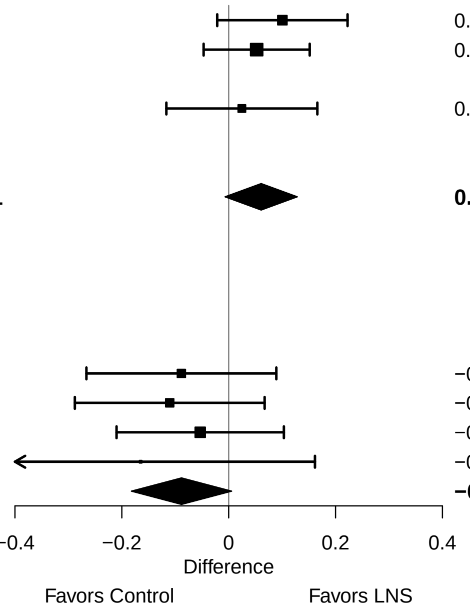

Supplemental figure 6L: Mean difference in executive function z-score

6L7: Stratified by Supplement duration (insufficient comparisons)

Supplemental figure 6L: Mean difference in executive function z-score

6L8: Stratified by Frequency of contact

Frequency of contact
(p−diff = 0.192)

Country	Trial	Tool	N	N	MD (95% CI)	W
**Frequency of contact − Monthly**						
Bangladesh	RDNS (22)	A not B task	533	266	0.10 (−0.02, 0.22)	0.42
Haiti	HAITI (27)					
Kenya	WASH−B (28)					
Madagascar	MAHAY (29)					
Mali	PROMIS CS (32)					
Zimbabwe	SHINE (HIV−) (33), LNS vs SOC	A not B task	370	357	−0.05 (−0.21, 0.10)	0.45
Zimbabwe	LNS+WSH vs WSH	A not B task	417	382	0.12 (−0.05, 0.29)	0.45
Zimbabwe	SHINE (HIV+) (34), LNS vs SOC	A not B task	63	57	−0.16 (−0.49, 0.16)	0.13
Zimbabwe	LNS+WSH vs WSH	A not B task	94	76	−0.05 (−0.35, 0.25)	0.13
**I² = 0.15, Tau² = 0.00**			**1477**	**1138**	**0.04 (−0.05, 0.13)**	
**Frequency of contact − Weekly**						
Bangladesh	JiVitA−4 (21)					
Bangladesh	WASH−B (23)	A not B task	1097	3327	0.00 (−0.07, 0.07)	0.61
Burkina Faso	iLiNS−Zinc (24)					
Ghana	GHANA (25)					
Ghana	iLiNS−DYAD−G (26)	A not B task	290	576	0.02 (−0.12, 0.17)	0.17
Malawi	iLiNS−DYAD−M (30)	A not B task	182	369	−0.09 (−0.27, 0.09)	0.11
Malawi	iLiNS−DOSE (31)	A not B task	475	164	−0.11 (−0.29, 0.07)	0.11
**I² = 0.00, Tau² = 0.00**			**2044**	**4436**	**−0.02 (−0.07, 0.04)**	

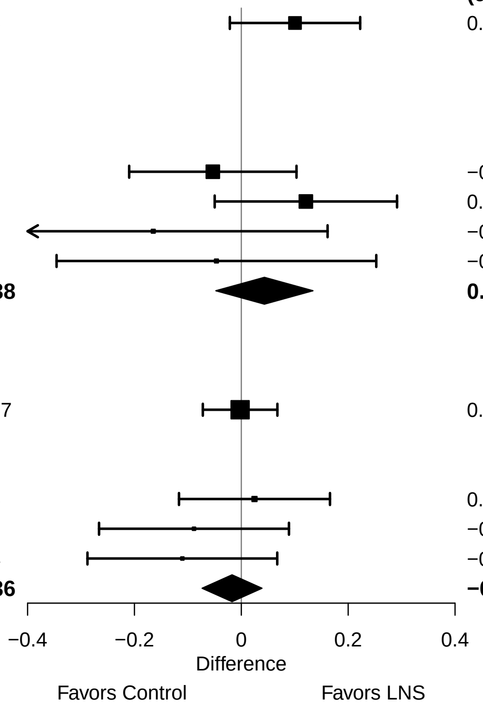

Supplemental figure 6L: Mean difference in executive function z-score

6L9: Stratified by Average SQ-LNS compliance (insufficient comparisons)

Supplemental figure 6M: Executive function lowest decile prevalence ratio

6M1: Stratified by Geographic region (insufficient comparisons)

Supplemental figure 6M: Executive function lowest decile prevalence ratio

6M2: Stratified by Stunting burden (insufficient comparisons)

Supplemental figure 6M: Executive function lowest decile prevalence ratio

6M3: Stratified by Malaria prevalence

Malaria prevalence
(p−diff = 0.990)

Malaria prevalence − Less than 10%

Country	Trial	Tool	N	N	PR (95% CI)	W
Bangladesh	JiVitA−4 (21)					
Bangladesh	RDNS (22)	A not B task	533	266	0.88 (0.60, 1.29)	0.21
Bangladesh	WASH−B (23)	A not B task	1097	3327	0.95 (0.77, 1.18)	0.42
Haiti	HAITI (27)					
Kenya	WASH−B (28)					
Madagascar	MAHAY (29)					
Zimbabwe	SHINE (HIV−) (33), LNS vs SOC	A not B task	370	357	0.82 (0.53, 1.26)	0.28
Zimbabwe	LNS+WSH vs WSH	A not B task	417	382	0.78 (0.50, 1.21)	0.28
Zimbabwe	SHINE (HIV+) (34), LNS vs SOC	A not B task	63	57	1.09 (0.37, 3.22)	0.09
Zimbabwe	LNS+WSH vs WSH	A not B task	94	76	2.10 (0.92, 4.82)	0.09
**I² = 0.22, Tau² = 0.02**			**2574**	**4465**	**0.94 (0.75, 1.16)**	

Malaria prevalence − At least 10%

Country	Trial	Tool	N	N	PR (95% CI)	W
Burkina Faso	iLiNS−Zinc (24)					
Ghana	GHANA (25)					
Ghana	iLiNS−DYAD−G (26)	A not B task	290	576	0.78 (0.50, 1.20)	0.44
Malawi	iLiNS−DYAD−M (30)	A not B task	182	369	0.81 (0.47, 1.41)	0.30
Malawi	iLiNS−DOSE (31)	A not B task	475	164	1.50 (0.82, 2.73)	0.26
Mali	PROMIS CS (32)					
**I² = 0.39, Tau² = 0.05**			**947**	**1109**	**0.95 (0.64, 1.41)**	

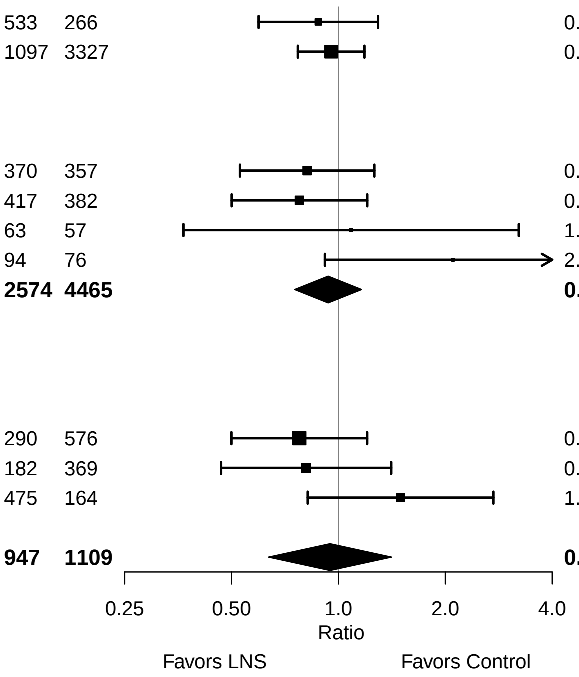

Supplemental figure 6M: Executive function lowest decile prevalence ratio

6M4: Stratified by Anemia prevalence (insufficient comparisons)

Supplemental figure 6M: Executive function lowest decile prevalence ratio

6M5: Stratified by Source water quality (insufficient comparisons)

Supplemental figure 6M: Executive function lowest decile prevalence ratio

6M6: Stratified by Sanitation

Sanitation
(p−diff = 0.286)

Country	Trial	Tool	N	N	PR (95% CI)	W
**Sanitation − Improved**						
Bangladesh	JiVitA−4 (21)					
Bangladesh	RDNS (22)	A not B task	533	266	0.88 (0.60, 1.29)	0.28
Bangladesh	WASH−B (23)	A not B task	541	1099	0.74 (0.56, 1.00)	0.50
Ghana	GHANA (25)					
Ghana	iLiNS−DYAD−G (26)	A not B task	290	576	0.78 (0.50, 1.20)	0.22
Haiti	HAITI (27)					
Mali	PROMIS CS (32)					
**I² = 0.00, Tau² = 0.00**			**1364**	**1941**	**0.79 (0.64, 0.97)**	
**Sanitation − Unimproved**						
Burkina Faso	iLiNS−Zinc (24)					
Kenya	WASH−B (28)					
Madagascar	MAHAY (29)					
Malawi	iLiNS−DYAD−M (30)	A not B task	182	369	0.81 (0.47, 1.41)	0.27
Malawi	iLiNS−DOSE (31)	A not B task	475	164	1.50 (0.82, 2.73)	0.23
Zimbabwe	SHINE (HIV−) (33)	A not B task	370	357	0.82 (0.53, 1.26)	0.43
Zimbabwe	SHINE (HIV+) (34)	A not B task	63	57	1.09 (0.37, 3.22)	0.07
**I² = 0.01, Tau² = 0.00**			**1090**	**947**	**0.95 (0.72, 1.27)**	

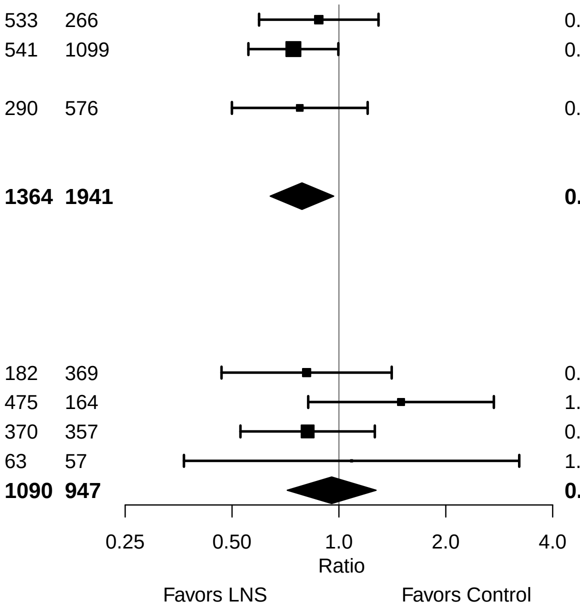

Supplemental figure 6M: Executive function lowest decile prevalence ratio

6M7: Stratified by Supplement duration (insufficient comparisons)

Supplemental figure 6M: Executive function lowest decile prevalence ratio

6M8: Stratified by Frequency of contact

Frequency of contact
(p−diff = 0.724)

Country	Trial	Tool	N	N	PR (95% CI)	W
**Frequency of contact − Monthly**						
Bangladesh	RDNS (22)	A not B task	533	266	0.88 (0.60, 1.29)	0.36
Haiti	HAITI (27)					
Kenya	WASH−B (28)					
Madagascar	MAHAY (29)					
Mali	PROMIS CS (32)					
Zimbabwe	SHINE (HIV−) (33), LNS vs SOC	A not B task	370	357	0.82 (0.53, 1.26)	0.49
Zimbabwe	LNS+WSH vs WSH	A not B task	417	382	0.78 (0.50, 1.21)	0.49
Zimbabwe	SHINE (HIV+) (34), LNS vs SOC	A not B task	63	57	1.09 (0.37, 3.22)	0.15
Zimbabwe	LNS+WSH vs WSH	A not B task	94	76	2.10 (0.92, 4.82)	0.15
**I² = 0.46, Tau² = 0.07**			**1477**	**1138**	**0.96 (0.66, 1.41)**	
**Frequency of contact − Weekly**						
Bangladesh	JiVitA−4 (21)					
Bangladesh	WASH−B (23)	A not B task	1097	3327	0.95 (0.77, 1.18)	0.52
Burkina Faso	iLiNS−Zinc (24)					
Ghana	GHANA (25)					
Ghana	iLiNS−DYAD−G (26)	A not B task	290	576	0.78 (0.50, 1.20)	0.21
Malawi	iLiNS−DYAD−M (30)	A not B task	182	369	0.81 (0.47, 1.41)	0.14
Malawi	iLiNS−DOSE (31)	A not B task	475	164	1.50 (0.82, 2.73)	0.12
**I² = 0.09, Tau² = 0.01**			**2044**	**4436**	**0.94 (0.77, 1.16)**	

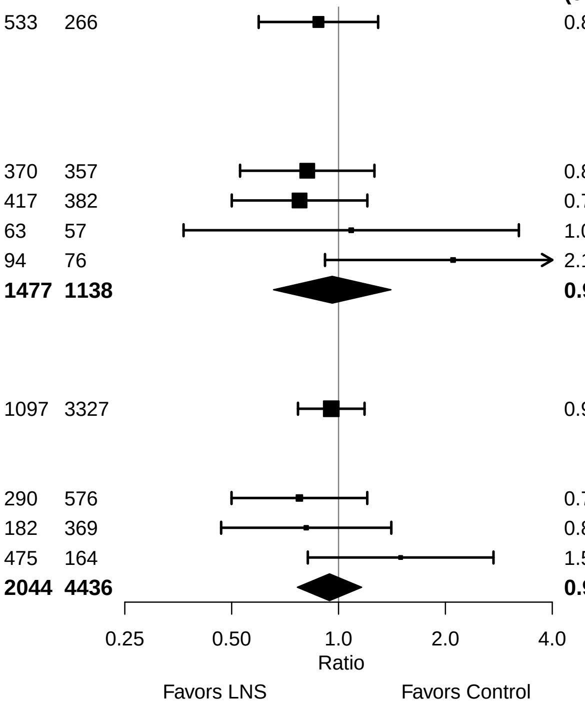

Supplemental figure 6M: Executive function lowest decile prevalence ratio

6M9: Stratified by Average SQ-LNS compliance (insufficient comparisons)

Supplemental figure 6N: Executive function lowest decile prevalence difference

6N1: Stratified by Geographic region (insufficient comparisons)

Supplemental figure 6N: Executive function lowest decile prevalence difference

6N2: Stratified by Stunting burden (insufficient comparisons)

Supplemental figure 6N: Executive function lowest decile prevalence difference

6N3: Stratified by Malaria prevalence

Malaria prevalence
(p−diff = 0.865)

Malaria prevalence − Less than 10%

**Country**	**Trial**		**Tool**	**N**	**N**	**PD (95% CI)**	**W**
Bangladesh	JiVitA−4 (21)						
Bangladesh	RDNS (22)		A not B task	533	266	−0.01 (−0.06, 0.03)	0.17
Bangladesh	WASH−B (23)		A not B task	1097	3327	0.00 (−0.02, 0.02)	0.44
Haiti	HAITI (27)						
Kenya	WASH−B (28)						
Madagascar	MAHAY (29)						
Zimbabwe	SHINE (HIV−) (33),	LNS vs SOC	A not B task	370	357	−0.02 (−0.06, 0.02)	0.30
Zimbabwe		LNS+WSH vs WSH	A not B task	417	382	−0.03 (−0.07, 0.02)	0.30
Zimbabwe	SHINE (HIV+) (34),	LNS vs SOC	A not B task	63	57	0.01 (−0.10, 0.11)	0.09
Zimbabwe		LNS+WSH vs WSH	A not B task	94	76	0.07 (−0.02, 0.16)	0.09
**I² = 0.15, Tau² = 0.00**				**2574**	**4465**	**−0.01 (−0.03, 0.01)**	

Malaria prevalence − At least 10%

**Country**	**Trial**	**Tool**	**N**	**N**	**PD (95% CI)**	**W**
Burkina Faso	iLiNS−Zinc (24)					
Ghana	GHANA (25)					
Ghana	iLiNS−DYAD−G (26)	A not B task	290	576	−0.02 (−0.07, 0.02)	0.40
Malawi	iLiNS−DYAD−M (30)	A not B task	182	369	−0.02 (−0.07, 0.03)	0.28
Malawi	iLiNS−DOSE (31)	A not B task	475	164	0.04 (−0.01, 0.09)	0.31
Mali	PROMIS CS (32)					
**I² = 0.50, Tau² = 0.00**			**947**	**1109**	**0.00 (−0.04, 0.03)**	

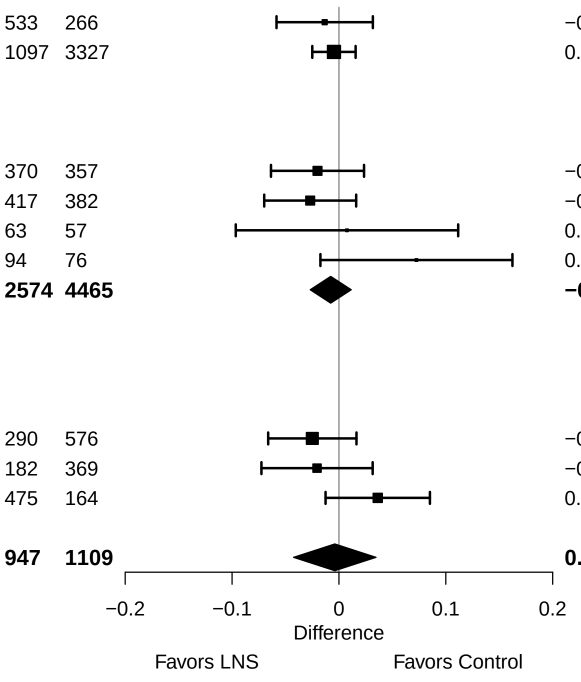

Supplemental figure 6N: Executive function lowest decile prevalence difference

6N4: Stratified by Anemia prevalence (insufficient comparisons)

Supplemental figure 6N: Executive function lowest decile prevalence difference

6N5: Stratified by Source water quality (insufficient comparisons)

Supplemental figure 6N: Executive function lowest decile prevalence difference

6N6: Stratified by Sanitation

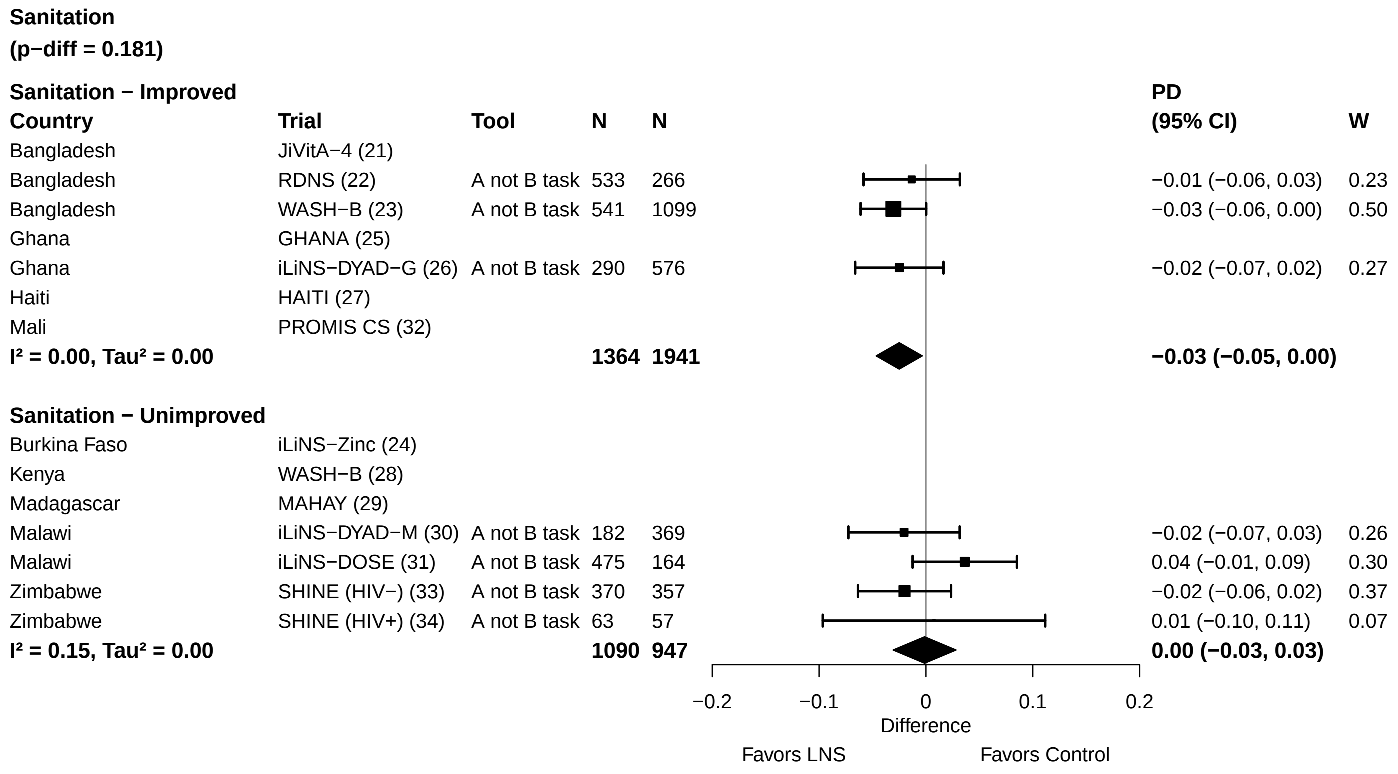

Supplemental figure 6N: Executive function lowest decile prevalence difference

6N7: Stratified by Supplement duration (insufficient comparisons)

Supplemental figure 6N: Executive function lowest decile prevalence difference

6N8: Stratified by Frequency of contact

Frequency of contact
(p−diff = 0.609)

Frequency of contact − Monthly

Country	Trial	Tool	N	N	PD (95% CI)	W
Bangladesh	RDNS (22)	A not B task	533	266	−0.01 (−0.06, 0.03)	0.31
Haiti	HAITI (27)					
Kenya	WASH−B (28)					
Madagascar	MAHAY (29)					
Mali	PROMIS CS (32)					
Zimbabwe	SHINE (HIV−) (33), LNS vs SOC	A not B task	370	357	−0.02 (−0.06, 0.02)	0.53
Zimbabwe	LNS+WSH vs WSH	A not B task	417	382	−0.03 (−0.07, 0.02)	0.53
Zimbabwe	SHINE (HIV+) (34), LNS vs SOC	A not B task	63	57	0.01 (−0.10, 0.11)	0.16
Zimbabwe	LNS+WSH vs WSH	A not B task	94	76	0.07 (−0.02, 0.16)	0.16
**I² = 0.40, Tau² = 0.00**			**1477**	**1138**	**−0.01 (−0.04, 0.03)**	

Frequency of contact − Weekly

Country	Trial	Tool	N	N	PD (95% CI)	W
Bangladesh	JiVitA−4 (21)					
Bangladesh	WASH−B (23)	A not B task	1097	3327	0.00 (−0.02, 0.02)	0.47
Burkina Faso	iLiNS−Zinc (24)					
Ghana	GHANA (25)					
Ghana	iLiNS−DYAD−G (26)	A not B task	290	576	−0.02 (−0.07, 0.02)	0.21
Malawi	iLiNS−DYAD−M (30)	A not B task	182	369	−0.02 (−0.07, 0.03)	0.15
Malawi	iLiNS−DOSE (31)	A not B task	475	164	0.04 (−0.01, 0.09)	0.17
**I² = 0.25, Tau² = 0.00**			**2044**	**4436**	**0.00 (−0.03, 0.02)**	

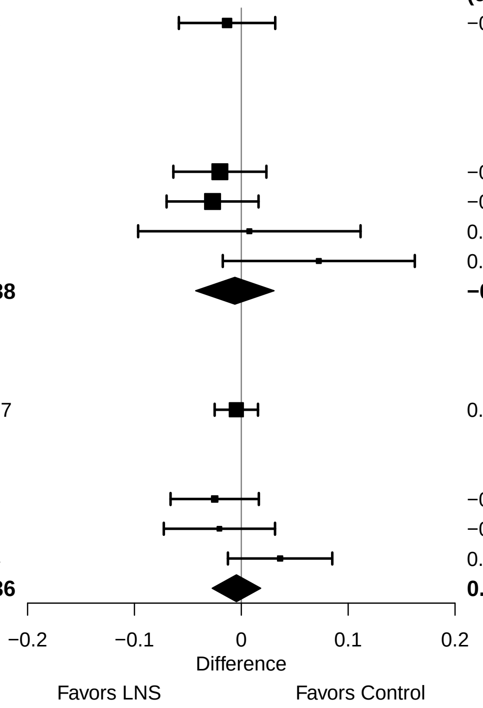

Supplemental figure 6N: Executive function lowest decile prevalence difference

6N9: Stratified by Average SQ-LNS compliance (insufficient comparisons)

Supplemental figure 6O: 12-mo walking without support prevalence ratio

6O1: Stratified by Geographic region

Geographic region
(p−diff = 0.525)

Country	Trial	N	N	PR (95% CI)	W
**Geographic region − SEAR**					
Bangladesh	JiVitA−4 (21)	3014	1363	1.05 (0.99, 1.12)	0.39
Bangladesh	RDNS (22)	1628	790	1.28 (1.07, 1.53)	0.28
Bangladesh	WASH−B (23)	497	1474	1.20 (1.05, 1.38)	0.32
**I² = 0.69, Tau² = 0.01**		**5139**	**3627**	**1.15 (1.02, 1.29)**	
**Geographic region − AFR**					
Burkina Faso	iLiNS−Zinc (24)				
Ghana	GHANA (25)	90	87	1.67 (1.08, 2.58)	0.08
Ghana	iLiNS−DYAD−G (26)	327	663	1.15 (1.01, 1.31)	0.23
Kenya	WASH−B (28)	447	1604	0.97 (0.85, 1.10)	0.23
Madagascar	MAHAY (29)				
Malawi	iLiNS−DYAD−M (30)	208	426	1.20 (1.03, 1.40)	0.22
Malawi	iLiNS−DOSE (31)	602	200	1.04 (0.84, 1.30)	0.17
Mali	PROMIS CS (32)	118	151	0.70 (0.44, 1.11)	0.07
Zimbabwe	SHINE (HIV−) (33)				
Zimbabwe	SHINE (HIV+) (34)				
**I² = 0.62, Tau² = 0.04**		**1792**	**3131**	**1.09 (0.91, 1.30)**	

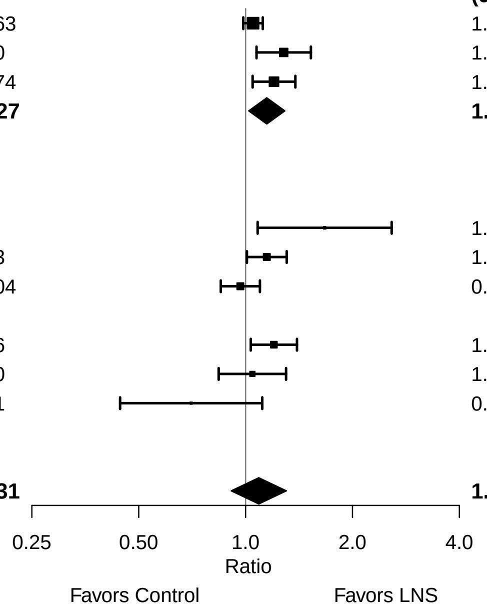

Supplemental figure 6O: 12-mo walking without support prevalence ratio

6O2: Stratified by Stunting burden

Stunting burden
(p−diff = 0.711)

Country	Trial	N	N	PR (95% CI)	W
**Stunting burden − Less than 35%**					
Ghana	GHANA (25)	90	87	1.67 (1.08, 2.58)	0.08
Ghana	iLiNS−DYAD−G (26)	327	663	1.15 (1.01, 1.31)	0.28
Haiti	HAITI (27)	83	79	1.25 (0.86, 1.81)	0.10
Kenya	WASH−B (28)	447	1604	0.97 (0.85, 1.10)	0.28
Malawi	iLiNS−DYAD−M (30)	208	426	1.20 (1.03, 1.40)	0.26
**I² = 0.59, Tau² = 0.01**		**1155**	**2859**	**1.15 (1.00, 1.33)**	
**Stunting burden − More than 35%**					
Bangladesh	JiVitA−4 (21)	3014	1363	1.05 (0.99, 1.12)	0.31
Bangladesh	RDNS (22)	1628	790	1.28 (1.07, 1.53)	0.21
Bangladesh	WASH−B (23)	497	1474	1.20 (1.05, 1.38)	0.24
Burkina Faso	iLiNS−Zinc (24)				
Madagascar	MAHAY (29)				
Malawi	iLiNS−DOSE (31)	602	200	1.04 (0.84, 1.30)	0.17
Mali	PROMIS CS (32)	118	151	0.70 (0.44, 1.11)	0.07
Zimbabwe	SHINE (HIV−) (33)				
Zimbabwe	SHINE (HIV+) (34)				
**I² = 0.60, Tau² = 0.02**		**5859**	**3978**	**1.09 (0.94, 1.27)**	

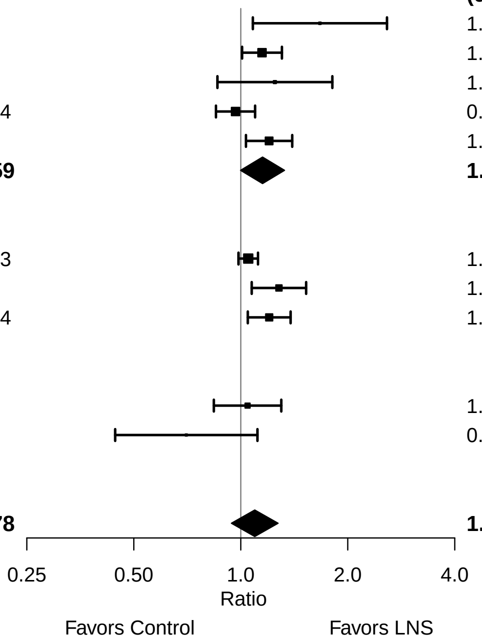

Supplemental figure 6O: 12-mo walking without support prevalence ratio

6O3: Stratified by Malaria prevalence

Malaria prevalence
(p−diff = 0.768)

Country	Trial	N	N	PR (95% CI)	W
**Malaria prevalence − Less than 10%**					
Bangladesh	JiVitA−4 (21)	3014	1363	1.05 (0.99, 1.12)	0.28
Bangladesh	RDNS (22)	1628	790	1.28 (1.07, 1.53)	0.19
Bangladesh	WASH−B (23)	497	1474	1.20 (1.05, 1.38)	0.22
Haiti	HAITI (27)	83	79	1.25 (0.86, 1.81)	0.08
Kenya	WASH−B (28)	447	1604	0.97 (0.85, 1.10)	0.23
Madagascar	MAHAY (29)				
Zimbabwe	SHINE (HIV−) (33)				
Zimbabwe	SHINE (HIV+) (34)				
**I² = 0.61, Tau² = 0.01**		**5669**	**5310**	**1.11 (1.00, 1.23)**	
**Malaria prevalence − At least 10%**					
Burkina Faso	iLiNS−Zinc (24)				
Ghana	GHANA (25)	90	87	1.67 (1.08, 2.58)	0.09
Ghana	iLiNS−DYAD−G (26)	327	663	1.15 (1.01, 1.31)	0.32
Malawi	iLiNS−DYAD−M (30)	208	426	1.20 (1.03, 1.40)	0.29
Malawi	iLiNS−DOSE (31)	602	200	1.04 (0.84, 1.30)	0.22
Mali	PROMIS CS (32)	118	151	0.70 (0.44, 1.11)	0.08
**I² = 0.52, Tau² = 0.05**		**1345**	**1527**	**1.12 (0.90, 1.41)**	

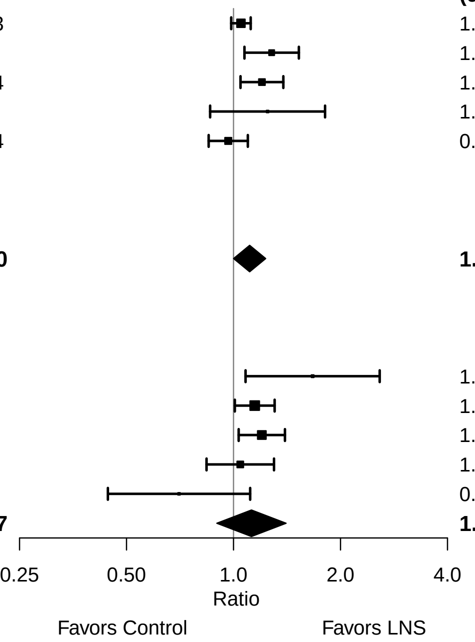

Supplemental figure 6O: 12-mo walking without support prevalence ratio

6O4: Stratified by Anemia prevalence

Anemia prevalence
(p−diff = 0.720)

**Anemia prevalence − High**				**PR**	
**Country**	**Trial**	**N**	**N**	**(95% CI)**	**W**
Burkina Faso	iLiNS−Zinc (24)				
Ghana	GHANA (25)	90	87	1.67 (1.08, 2.58)	0.11
Haiti	HAITI (27)	83	79	1.25 (0.86, 1.81)	0.14
Malawi	iLiNS−DYAD−M (30)	208	426	1.20 (1.03, 1.40)	0.37
Malawi	iLiNS−DOSE (31)	602	200	1.04 (0.84, 1.30)	0.27
Mali	PROMIS CS (32)	118	151	0.70 (0.44, 1.11)	0.10
**I² = 0.53, Tau² = 0.05**		**1101**	**943**	**1.14 (0.89, 1.45)**	
**Anemia prevalence − Moderate**					
Bangladesh	JiVitA−4 (21)	3014	1363	1.05 (0.99, 1.12)	0.24
Bangladesh	RDNS (22)	1628	790	1.28 (1.07, 1.53)	0.16
Bangladesh	WASH−B (23)	497	1474	1.20 (1.05, 1.38)	0.19
Ghana	iLiNS−DYAD−G (26)	327	663	1.15 (1.01, 1.31)	0.20
Kenya	WASH−B (28)	447	1604	0.97 (0.85, 1.10)	0.20
Madagascar	MAHAY (29)				
Zimbabwe	SHINE (HIV−) (33)				
Zimbabwe	SHINE (HIV+) (34)				
**I² = 0.62, Tau² = 0.01**		**5913**	**5894**	**1.11 (1.01, 1.22)**	

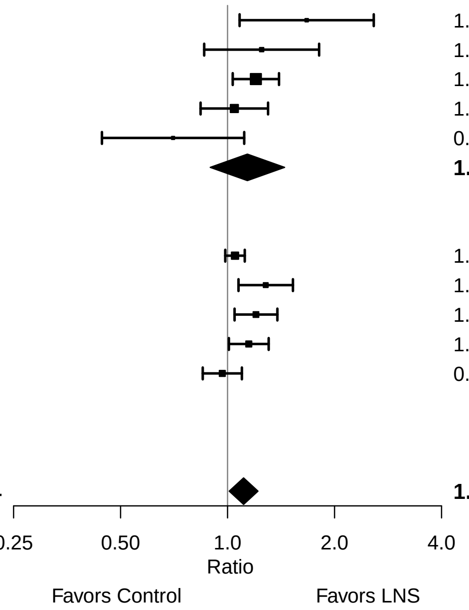

Supplemental figure 6O: 12-mo walking without support prevalence ratio

6O5: Stratified by Source water quality (insufficient comparisons)

Supplemental figure 6O: 12-mo walking without support prevalence ratio

6O6: Stratified by Sanitation

Sanitation
(p−diff = 0.631)

Country	Trial	N	N	PR (95% CI)	W
**Sanitation − Improved**					
Bangladesh	JiVitA−4 (21)	3014	1363	1.05 (0.99, 1.12)	0.29
Bangladesh	RDNS (22)	1628	790	1.28 (1.07, 1.53)	0.18
Bangladesh	WASH−B (23)	240	484	1.19 (0.94, 1.51)	0.13
Ghana	GHANA (25)	90	87	1.67 (1.08, 2.58)	0.05
Ghana	iLiNS−DYAD−G (26)	327	663	1.15 (1.01, 1.31)	0.22
Haiti	HAITI (27)	83	79	1.25 (0.86, 1.81)	0.07
Mali	PROMIS CS (32)	118	151	0.70 (0.44, 1.11)	0.05
**I² = 0.55, Tau² = 0.02**		**5500**	**3617**	**1.15 (1.00, 1.33)**	
**Sanitation − Unimproved**					
Burkina Faso	iLiNS−Zinc (24)				
Kenya	WASH−B (28)	202	706	1.00 (0.81, 1.22)	0.31
Madagascar	MAHAY (29)				
Malawi	iLiNS−DYAD−M (30)	208	426	1.20 (1.03, 1.40)	0.40
Malawi	iLiNS−DOSE (31)	602	200	1.04 (0.84, 1.30)	0.29
Zimbabwe	SHINE (HIV−) (33)				
Zimbabwe	SHINE (HIV+) (34)				
**I² = 0.18, Tau² = 0.00**		**1012**	**1332**	**1.10 (0.98, 1.24)**	

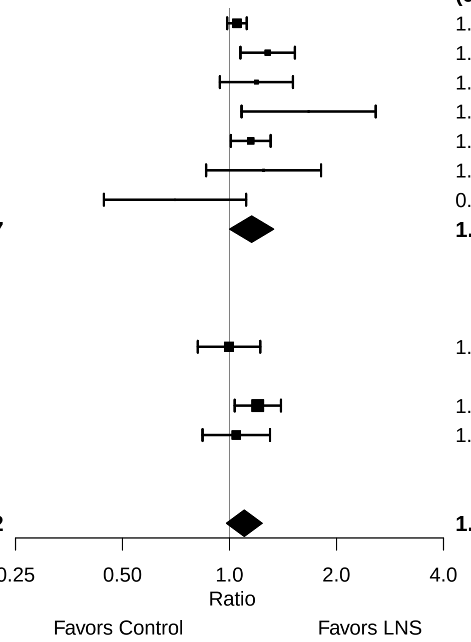

Supplemental figure 6O: 12-mo walking without support prevalence ratio

6O7: Stratified by Supplement duration

Supplement duration
(p−diff = 0.618)

Country	Trial	N	N	PR (95% CI)	W
**Supplement duration − 12m or less**					
Bangladesh	JiVitA−4 (21)	3014	1363	1.05 (0.99, 1.12)	0.27
Burkina Faso	iLiNS−Zinc (24)				
Ghana	GHANA (25)	90	87	1.67 (1.08, 2.58)	0.06
Ghana	iLiNS−DYAD−G (26)	327	663	1.15 (1.01, 1.31)	0.22
Haiti	HAITI (27)	83	79	1.25 (0.86, 1.81)	0.08
Madagascar	MAHAY (29)				
Malawi	iLiNS−DYAD−M (30)	208	426	1.20 (1.03, 1.40)	0.21
Malawi	iLiNS−DOSE (31)	602	200	1.04 (0.84, 1.30)	0.15
Zimbabwe	SHINE (HIV−) (33)				
Zimbabwe	SHINE (HIV+) (34)				
**I² = 0.37, Tau² = 0.00**		**4324**	**2818**	**1.13 (1.03, 1.23)**	
**Supplement duration − > 12m**					
Bangladesh	RDNS (22)	1628	790	1.28 (1.07, 1.53)	0.27
Bangladesh	WASH−B (23)	497	1474	1.20 (1.05, 1.38)	0.32
Kenya	WASH−B (28)	447	1604	0.97 (0.85, 1.10)	0.33
Mali	PROMIS CS (32)	118	151	0.70 (0.44, 1.11)	0.09
**I² = 0.75, Tau² = 0.04**		**2690**	**4019**	**1.06 (0.85, 1.34)**	

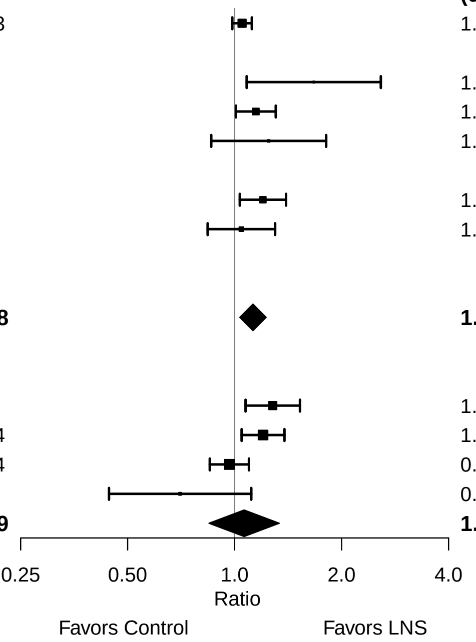

Supplemental figure 6O: 12-mo walking without support prevalence ratio

6O8: Stratified by Frequency of contact

Frequency of contact
(p−diff = 0.433)

Country	Trial	N	N	PR (95% CI)	W
**Frequency of contact − Monthly**					
Bangladesh	RDNS (22)	1628	790	1.28 (1.07, 1.53)	0.34
Haiti	HAITI (27)	83	79	1.25 (0.86, 1.81)	0.15
Kenya	WASH−B (28)	447	1604	0.97 (0.85, 1.10)	0.41
Madagascar	MAHAY (29)				
Mali	PROMIS CS (32)	118	151	0.70 (0.44, 1.11)	0.11
Zimbabwe	SHINE (HIV−) (33)				
Zimbabwe	SHINE (HIV+) (34)				
**I² = 0.71, Tau² = 0.04**		**2276**	**2624**	**1.05 (0.83, 1.34)**	
**Frequency of contact − Weekly**					
Bangladesh	JiVitA−4 (21)	3014	1363	1.05 (0.99, 1.12)	0.24
Bangladesh	WASH−B (23)	497	1474	1.20 (1.05, 1.38)	0.19
Burkina Faso	iLiNS−Zinc (24)				
Ghana	GHANA (25)	90	87	1.67 (1.08, 2.58)	0.06
Ghana	iLiNS−DYAD−G (26)	327	663	1.15 (1.01, 1.31)	0.20
Malawi	iLiNS−DYAD−M (30)	208	426	1.20 (1.03, 1.40)	0.18
Malawi	iLiNS−DOSE (31)	602	200	1.04 (0.84, 1.30)	0.14
**I² = 0.46, Tau² = 0.00**		**4738**	**4213**	**1.14 (1.05, 1.23)**	

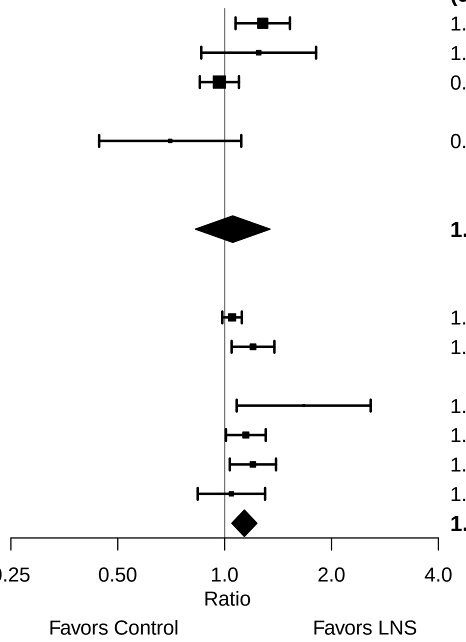

Supplemental figure 6O: 12-mo walking without support prevalence ratio

6O9: Stratified by Average SQ-LNS compliance

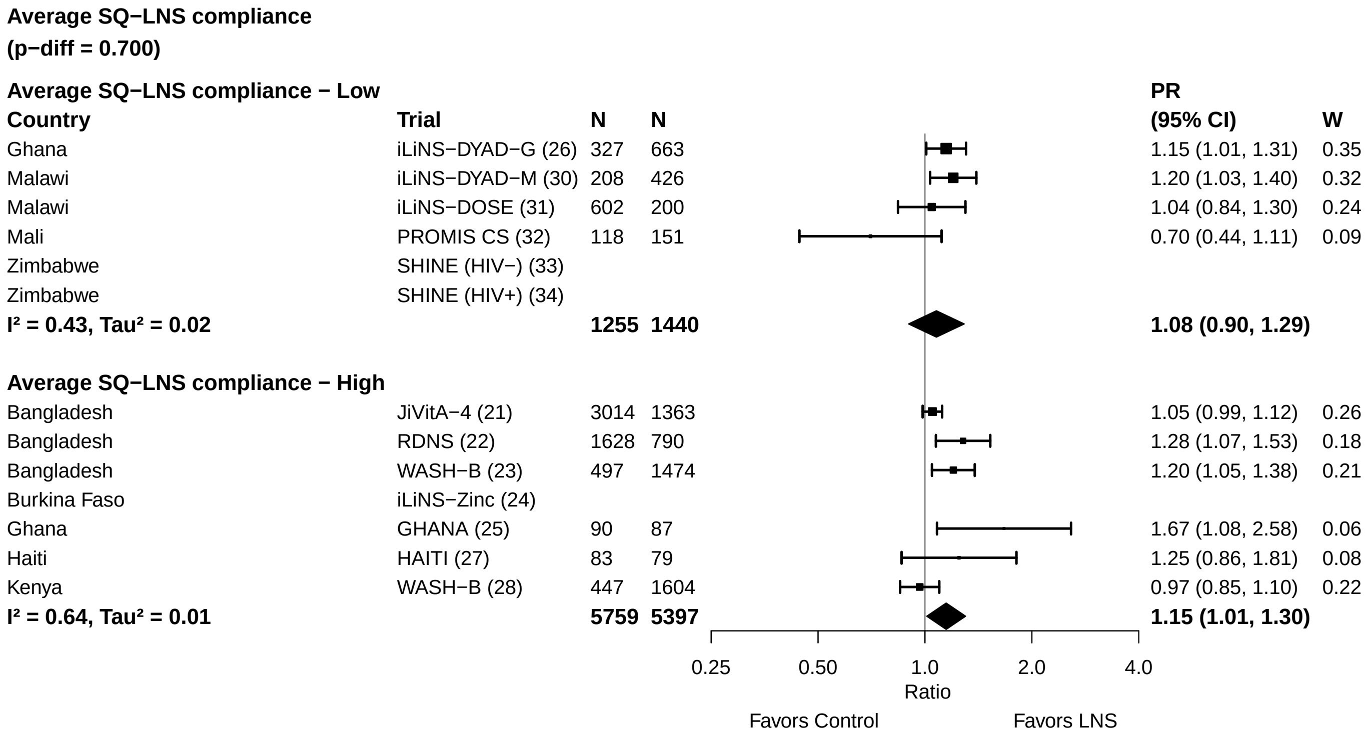

Supplemental figure 6P: 12-mo walking without support prevalence difference

6P1: Stratified by Geographic region

Geographic region
(p−diff = 0.639)

Country	Trial	N	N	PD (95% CI)	W
**Geographic region − SEAR**					
Bangladesh	JiVitA−4 (21)	3014	1363	0.03 (−0.01, 0.06)	0.35
Bangladesh	RDNS (22)	1628	790	0.07 (0.03, 0.11)	0.34
Bangladesh	WASH−B (23)	497	1474	0.05 (0.01, 0.10)	0.32
**I² = 0.38, Tau² = 0.00**		**5139**	**3627**	**0.05 (0.02, 0.08)**	
**Geographic region − AFR**					
Burkina Faso	iLiNS−Zinc (24)				
Ghana	GHANA (25)	90	87	0.17 (0.03, 0.31)	0.10
Ghana	iLiNS−DYAD−G (26)	327	663	0.07 (0.00, 0.14)	0.20
Kenya	WASH−B (28)	447	1604	−0.01 (−0.06, 0.04)	0.22
Madagascar	MAHAY (29)				
Malawi	iLiNS−DYAD−M (30)	208	426	0.10 (0.02, 0.18)	0.17
Malawi	iLiNS−DOSE (31)	602	200	0.02 (−0.06, 0.09)	0.18
Mali	PROMIS CS (32)	118	151	−0.10 (−0.21, 0.01)	0.13
Zimbabwe	SHINE (HIV−) (33)				
Zimbabwe	SHINE (HIV+) (34)				
**I² = 0.69, Tau² = 0.01**		**1792**	**3131**	**0.04 (−0.03, 0.10)**	

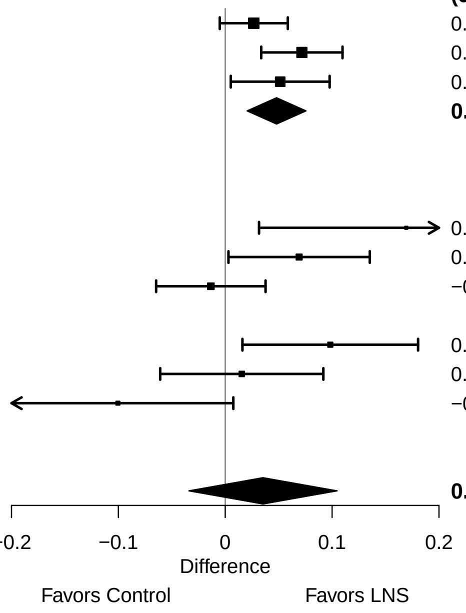

Supplemental figure 6P: 12-mo walking without support prevalence difference

6P2: Stratified by Stunting burden

Stunting burden
(p−diff = 0.316)

Country	Trial	N	N	PD (95% CI)	W
**Stunting burden − Less than 35%**					
Ghana	GHANA (25)	90	87	0.17 (0.03, 0.31)	0.12
Ghana	iLiNS−DYAD−G (26)	327	663	0.07 (0.00, 0.14)	0.26
Haiti	HAITI (27)	83	79	0.09 (−0.06, 0.24)	0.11
Kenya	WASH−B (28)	447	1604	−0.01 (−0.06, 0.04)	0.30
Malawi	iLiNS−DYAD−M (30)	208	426	0.10 (0.02, 0.18)	0.22
**I² = 0.62, Tau² = 0.00**		**1155**	**2859**	**0.07 (0.01, 0.12)**	
**Stunting burden − More than 35%**					
Bangladesh	JiVitA−4 (21)	3014	1363	0.03 (−0.01, 0.06)	0.25
Bangladesh	RDNS (22)	1628	790	0.07 (0.03, 0.11)	0.24
Bangladesh	WASH−B (23)	497	1474	0.05 (0.01, 0.10)	0.22
Burkina Faso	iLiNS−Zinc (24)				
Madagascar	MAHAY (29)				
Malawi	iLiNS−DOSE (31)	602	200	0.02 (−0.06, 0.09)	0.17
Mali	PROMIS CS (32)	118	151	−0.10 (−0.21, 0.01)	0.12
Zimbabwe	SHINE (HIV−) (33)				
Zimbabwe	SHINE (HIV+) (34)				
**I² = 0.62, Tau² = 0.00**		**5859**	**3978**	**0.03 (−0.02, 0.08)**	

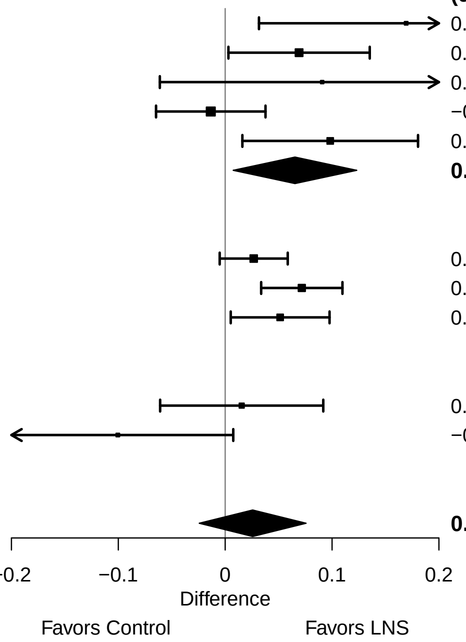

Supplemental figure 6P: 12-mo walking without support prevalence difference

6P3: Stratified by Malaria prevalence

Malaria prevalence
(p−diff = 0.814)

Malaria prevalence − Less than 10%

**Country**	**Trial**	**N**	**N**	**PD (95% CI)**	**W**
Bangladesh	JiVitA−4 (21)	3014	1363	0.03 (−0.01, 0.06)	0.25
Bangladesh	RDNS (22)	1628	790	0.07 (0.03, 0.11)	0.24
Bangladesh	WASH−B (23)	497	1474	0.05 (0.01, 0.10)	0.22
Haiti	HAITI (27)	83	79	0.09 (−0.06, 0.24)	0.08
Kenya	WASH−B (28)	447	1604	−0.01 (−0.06, 0.04)	0.21
Madagascar	MAHAY (29)				
Zimbabwe	SHINE (HIV−) (33)				
Zimbabwe	SHINE (HIV+) (34)				
**I² = 0.51, Tau² = 0.00**		**5669**	**5310**	**0.04 (0.01, 0.07)**	

Malaria prevalence − At least 10%

**Country**	**Trial**	**N**	**N**	**PD (95% CI)**	**W**
Burkina Faso	iLiNS−Zinc (24)				
Ghana	GHANA (25)	90	87	0.17 (0.03, 0.31)	0.12
Ghana	iLiNS−DYAD−G (26)	327	663	0.07 (0.00, 0.14)	0.26
Malawi	iLiNS−DYAD−M (30)	208	426	0.10 (0.02, 0.18)	0.22
Malawi	iLiNS−DOSE (31)	602	200	0.02 (−0.06, 0.09)	0.23
Mali	PROMIS CS (32)	118	151	−0.10 (−0.21, 0.01)	0.17
**I² = 0.69, Tau² = 0.01**		**1345**	**1527**	**0.05 (−0.04, 0.13)**	

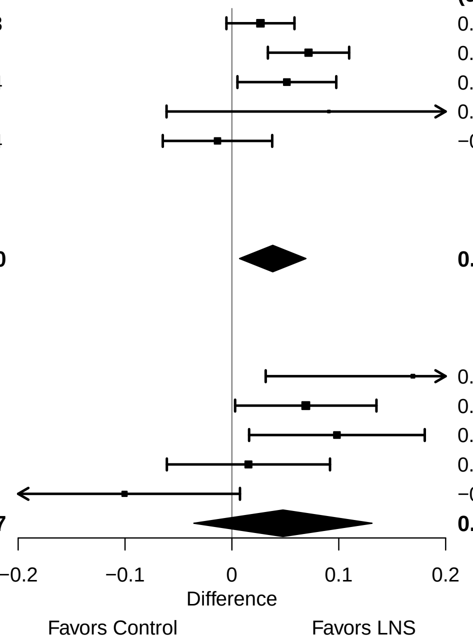

Supplemental figure 6P: 12-mo walking without support prevalence difference

6P4: Stratified by Anemia prevalence

Anemia prevalence
(p−diff = 0.903)

Country	Trial	N	N	PD (95% CI)	W
**Anemia prevalence − High**					
Burkina Faso	iLiNS−Zinc (24)				
Ghana	GHANA (25)	90	87	0.17 (0.03, 0.31)	0.14
Haiti	HAITI (27)	83	79	0.09 (−0.06, 0.24)	0.13
Malawi	iLiNS−DYAD−M (30)	208	426	0.10 (0.02, 0.18)	0.26
Malawi	iLiNS−DOSE (31)	602	200	0.02 (−0.06, 0.09)	0.27
Mali	PROMIS CS (32)	118	151	−0.10 (−0.21, 0.01)	0.20
**I² = 0.68, Tau² = 0.01**		**1101**	**943**	**0.05 (−0.04, 0.14)**	
**Anemia prevalence − Moderate**					
Bangladesh	JiVitA−4 (21)	3014	1363	0.03 (−0.01, 0.06)	0.22
Bangladesh	RDNS (22)	1628	790	0.07 (0.03, 0.11)	0.21
Bangladesh	WASH−B (23)	497	1474	0.05 (0.01, 0.10)	0.20
Ghana	iLiNS−DYAD−G (26)	327	663	0.07 (0.00, 0.14)	0.17
Kenya	WASH−B (28)	447	1604	−0.01 (−0.06, 0.04)	0.19
Madagascar	MAHAY (29)				
Zimbabwe	SHINE (HIV−) (33)				
Zimbabwe	SHINE (HIV+) (34)				
**I² = 0.53, Tau² = 0.00**		**5913**	**5894**	**0.04 (0.01, 0.07)**	

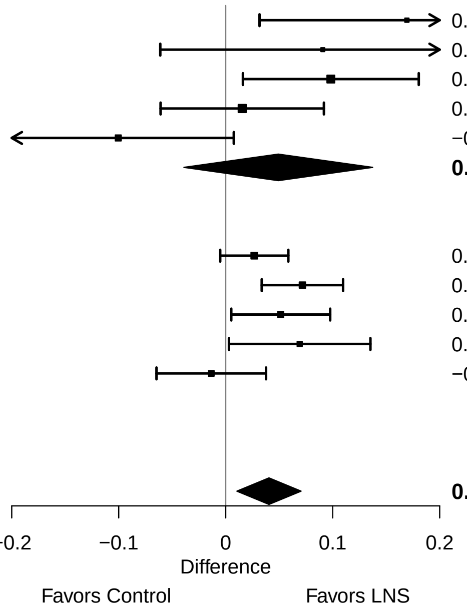

Supplemental figure 6P: 12-mo walking without support prevalence difference

6P5: Stratified by Source water quality (insufficient comparisons)

Supplemental figure 6P: 12-mo walking without support prevalence difference

6P6: Stratified by Sanitation

Sanitation
(p−diff = 0.733)

Country	Trial	N	N	PD (95% CI)	W
**Sanitation − Improved**					
Bangladesh	JiVitA−4 (21)	3014	1363	0.03 (−0.01, 0.06)	0.23
Bangladesh	RDNS (22)	1628	790	0.07 (0.03, 0.11)	0.22
Bangladesh	WASH−B (23)	240	484	0.05 (−0.02, 0.12)	0.16
Ghana	GHANA (25)	90	87	0.17 (0.03, 0.31)	0.07
Ghana	iLiNS−DYAD−G (26)	327	663	0.07 (0.00, 0.14)	0.16
Haiti	HAITI (27)	83	79	0.09 (−0.06, 0.24)	0.06
Mali	PROMIS CS (32)	118	151	−0.10 (−0.21, 0.01)	0.10
**I² = 0.57, Tau² = 0.00**		**5500**	**3617**	**0.05 (0.00, 0.10)**	
**Sanitation − Unimproved**					
Burkina Faso	iLiNS−Zinc (24)				
Kenya	WASH−B (28)	202	706	0.00 (−0.08, 0.08)	0.34
Madagascar	MAHAY (29)				
Malawi	iLiNS−DYAD−M (30)	208	426	0.10 (0.02, 0.18)	0.32
Malawi	iLiNS−DOSE (31)	602	200	0.02 (−0.06, 0.09)	0.34
Zimbabwe	SHINE (HIV−) (33)				
Zimbabwe	SHINE (HIV+) (34)				
**I² = 0.41, Tau² = 0.00**		**1012**	**1332**	**0.04 (−0.02, 0.10)**	

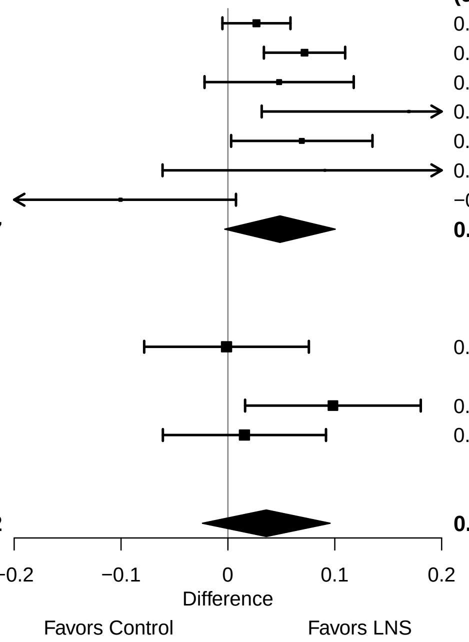

Supplemental figure 6P: 12-mo walking without support prevalence difference

6P7: Stratified by Supplement duration

Supplement duration
(p−diff = 0.221)

Country	Trial	N	N	PD (95% CI)	W
**Supplement duration − 12m or less**					
Bangladesh	JiVitA−4 (21)	3014	1363	0.03 (−0.01, 0.06)	0.27
Burkina Faso	iLiNS−Zinc (24)				
Ghana	GHANA (25)	90	87	0.17 (0.03, 0.31)	0.10
Ghana	iLiNS−DYAD−G (26)	327	663	0.07 (0.00, 0.14)	0.20
Haiti	HAITI (27)	83	79	0.09 (−0.06, 0.24)	0.08
Madagascar	MAHAY (29)				
Malawi	iLiNS−DYAD−M (30)	208	426	0.10 (0.02, 0.18)	0.17
Malawi	iLiNS−DOSE (31)	602	200	0.02 (−0.06, 0.09)	0.18
Zimbabwe	SHINE (HIV−) (33)				
Zimbabwe	SHINE (HIV+) (34)				
**I² = 0.33, Tau² = 0.00**		**4324**	**2818**	**0.06 (0.02, 0.09)**	
**Supplement duration − > 12m**					
Bangladesh	RDNS (22)	1628	790	0.07 (0.03, 0.11)	0.30
Bangladesh	WASH−B (23)	497	1474	0.05 (0.01, 0.10)	0.28
Kenya	WASH−B (28)	447	1604	−0.01 (−0.06, 0.04)	0.27
Mali	PROMIS CS (32)	118	151	−0.10 (−0.21, 0.01)	0.15
**I² = 0.78, Tau² = 0.00**		**2690**	**4019**	**0.01 (−0.06, 0.08)**	

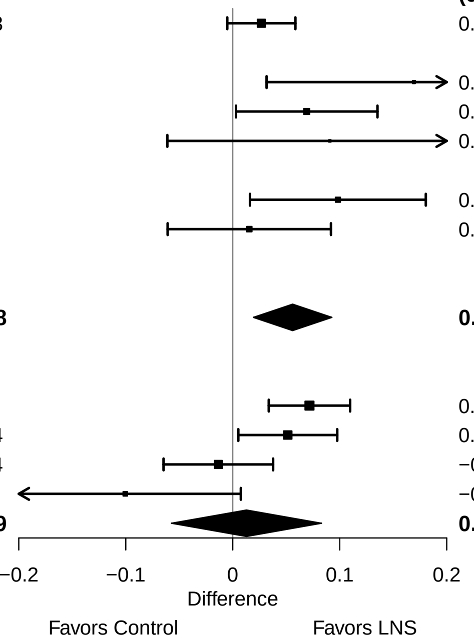

Supplemental figure 6P: 12-mo walking without support prevalence difference

6P8: Stratified by Frequency of contact

Frequency of contact
(p−diff = 0.235)

Country	Trial	N	N	PD (95% CI)	W
**Frequency of contact − Monthly**					
Bangladesh	RDNS (22)	1628	790	0.07 (0.03, 0.11)	0.37
Haiti	HAITI (27)	83	79	0.09 (−0.06, 0.24)	0.12
Kenya	WASH−B (28)	447	1604	−0.01 (−0.06, 0.04)	0.33
Madagascar	MAHAY (29)				
Mali	PROMIS CS (32)	118	151	−0.10 (−0.21, 0.01)	0.18
Zimbabwe	SHINE (HIV−) (33)				
Zimbabwe	SHINE (HIV+) (34)				
**I² = 0.78, Tau² = 0.00**		**2276**	**2624**	**0.01 (−0.07, 0.09)**	
**Frequency of contact − Weekly**					
Bangladesh	JiVitA−4 (21)	3014	1363	0.03 (−0.01, 0.06)	0.23
Bangladesh	WASH−B (23)	497	1474	0.05 (0.01, 0.10)	0.21
Burkina Faso	iLiNS−Zinc (24)				
Ghana	GHANA (25)	90	87	0.17 (0.03, 0.31)	0.08
Ghana	iLiNS−DYAD−G (26)	327	663	0.07 (0.00, 0.14)	0.17
Malawi	iLiNS−DYAD−M (30)	208	426	0.10 (0.02, 0.18)	0.15
Malawi	iLiNS−DOSE (31)	602	200	0.02 (−0.06, 0.09)	0.16
**I² = 0.30, Tau² = 0.00**		**4738**	**4213**	**0.05 (0.02, 0.09)**	

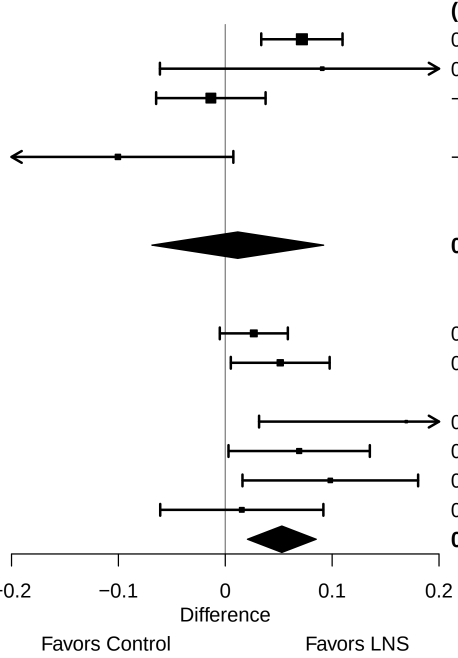

Supplemental figure 6P: 12-mo walking without support prevalence difference

6P9: Stratified by Average SQ-LNS compliance

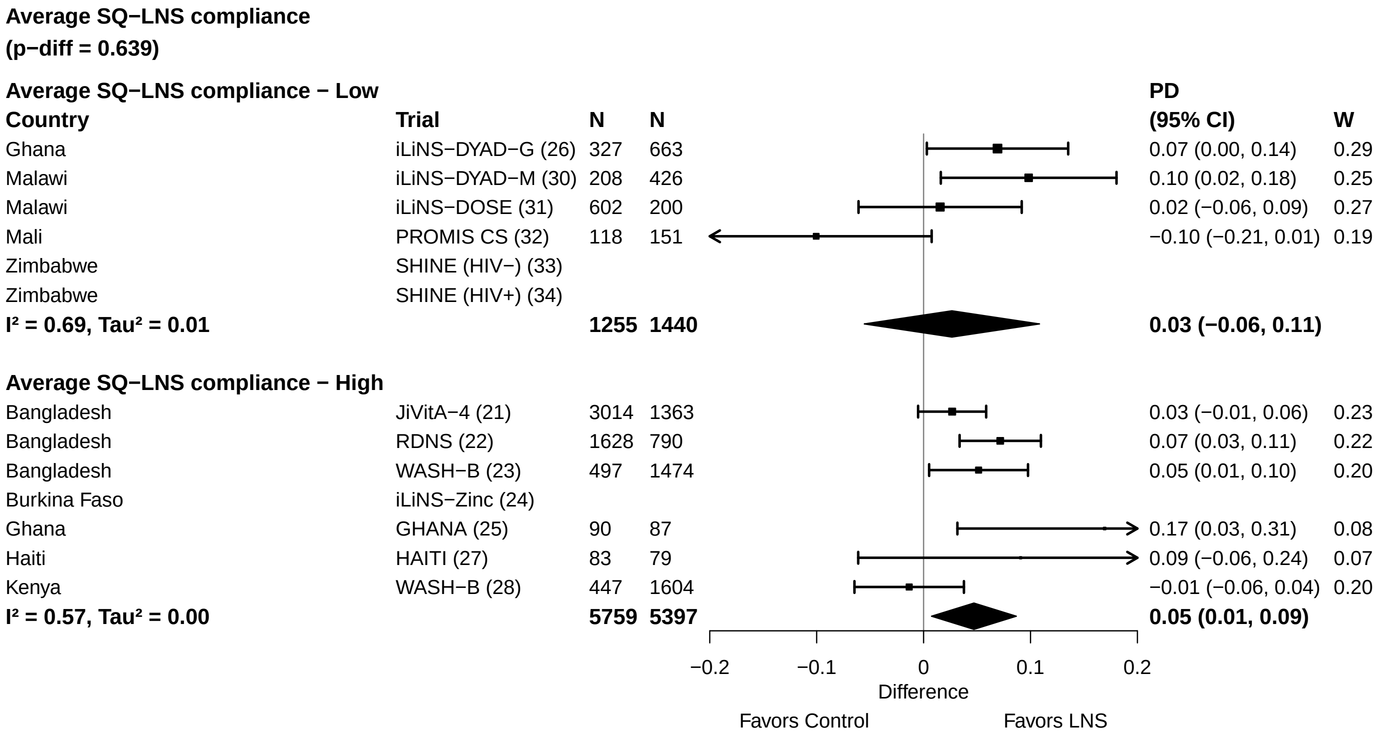

Supplement: nqab277_Supplemental_Files [file nqab277_supplemental_files.zip › ipdd_suppfig6_20210707.pdf]
