# Supplementary material for: Small-quantity lipid-based nutrient supplements for children age 6–24 months: a systematic review and individual participant data meta-analysis of effects on developmental outcomes and effect modifiers
Source: Am J Clin Nutr. 2021 Sep 29;114(Suppl 1):43S–67S. doi: 10.1093/ajcn/nqab277 (PMC8560311; doi:10.1093/ajcn/nqab277)

Online supplemental material: Small-quantity lipid-based nutrient supplements for children age 6-24 months: a systematic review and individual participant data meta-analysis of effects on developmental outcomes and effect modifiers. Prado et al. (2021)

# Supplemental figure 7 : Forest plots for effects of SQ-LNS on developmental outcomes stratified by individual-level maternal and child effect modifiers

## Contents


**Supplemental figure 7A: Mean difference in language z-score 5**
7A1: Stratified by Maternal height . . . 5
7A2: Stratified by Maternal BMI . . . 6
7A3: Stratified by Maternal age . . . 7
7A4: Stratified by Maternal education . . . 8
7A5: Stratified by Maternal depressive symptoms . . . 9
7A6: Stratified by Child sex . . . 10
7A7: Stratified by Child birth order . . . 11
7A8: Stratified by Child baseline stunting . . . 12
7A9: Stratified by Child baseline acute malnutrition . . . 13
7A10: Stratified by Child baseline anemia . . . 14

**Supplemental figure 7B: Language lowest decile prevalence ratio 15**
7B1: Stratified by Maternal height . . . 15
7B2: Stratified by Maternal BMI . . . 16
7B3: Stratified by Maternal age . . . 17
7B4: Stratified by Maternal education . . . 18
7B5: Stratified by Maternal depressive symptoms . . . 19
7B6: Stratified by Child sex . . . 20
7B7: Stratified by Child birth order . . . 21
7B8: Stratified by Child baseline stunting . . . 22
7B9: Stratified by Child baseline acute malnutrition . . . 23
7B10: Stratified by Child baseline anemia . . . 24

**Supplemental figure 7C: Language lowest decile prevalence difference 25**
7C1: Stratified by Maternal height . . . 25
7C2: Stratified by Maternal BMI . . . 26
7C3: Stratified by Maternal age . . . 27
7C4: Stratified by Maternal education . . . 28
7C5: Stratified by Maternal depressive symptoms . . . 29
7C6: Stratified by Child sex . . . 30
7C7: Stratified by Child birth order . . . 31
7C8: Stratified by Child baseline stunting . . . 32
7C9: Stratified by Child baseline acute malnutrition . . . 33
7C10: Stratified by Child baseline anemia . . . 34

**Supplemental figure 7D: Mean difference in social-emotional z-score 35**
7D1: Stratified by Maternal height . . . 35
7D2: Stratified by Maternal BMI . . . 36
7D3: Stratified by Maternal age . . . 37
7D4: Stratified by Maternal education . . . 38

7D5: Stratified by Maternal depressive symptoms . . . 39
7D6: Stratified by Child sex . . . 40
7D7: Stratified by Child birth order . . . 41
7D8: Stratified by Child baseline stunting . . . 42
7D9: Stratified by Child baseline acute malnutrition . . . 43
7D10: Stratified by Child baseline anemia . . . 44

**Supplemental figure 7E: Social-emotional lowest decile prevalence ratio** **45**
7E1: Stratified by Maternal height . . . 45
7E2: Stratified by Maternal BMI . . . 46
7E3: Stratified by Maternal age . . . 47
7E4: Stratified by Maternal education . . . 48
7E5: Stratified by Maternal depressive symptoms . . . 49
7E6: Stratified by Child sex . . . 50
7E7: Stratified by Child birth order . . . 51
7E8: Stratified by Child baseline stunting . . . 52
7E9: Stratified by Child baseline acute malnutrition (insufficient comparisons) . . . 53
7E10: Stratified by Child baseline anemia . . . 54

**Supplemental figure 7F: Social-emotional lowest decile prevalence difference** **55**
7F1: Stratified by Maternal height . . . 55
7F2: Stratified by Maternal BMI . . . 56
7F3: Stratified by Maternal age . . . 57
7F4: Stratified by Maternal education . . . 58
7F5: Stratified by Maternal depressive symptoms . . . 59
7F6: Stratified by Child sex . . . 60
7F7: Stratified by Child birth order . . . 61
7F8: Stratified by Child baseline stunting . . . 62
7F9: Stratified by Child baseline acute malnutrition (insufficient comparisons) . . . 63
7F10: Stratified by Child baseline anemia . . . 64

**Supplemental figure 7G: Mean difference in motor z-score** **65**
7G1: Stratified by Maternal height . . . 65
7G2: Stratified by Maternal BMI . . . 66
7G3: Stratified by Maternal age . . . 67
7G4: Stratified by Maternal education . . . 68
7G5: Stratified by Maternal depressive symptoms . . . 69
7G6: Stratified by Child sex . . . 70
7G7: Stratified by Child birth order . . . 71
7G8: Stratified by Child baseline stunting . . . 72
7G9: Stratified by Child baseline acute malnutrition . . . 73
7G10: Stratified by Child baseline anemia . . . 74

**Supplemental figure 7H: Motor lowest decile prevalence ratio** **75**
7H1: Stratified by Maternal height . . . 75
7H2: Stratified by Maternal BMI . . . 76
7H3: Stratified by Maternal age . . . 77
7H4: Stratified by Maternal education . . . 78
7H5: Stratified by Maternal depressive symptoms . . . 79
7H6: Stratified by Child sex . . . 80
7H7: Stratified by Child birth order . . . 81

7H8: Stratified by Child baseline stunting . . . 82
7H9: Stratified by Child baseline acute malnutrition . . . 83
7H10: Stratified by Child baseline anemia . . . 84

**Supplemental figure 7I: Motor lowest decile prevalence difference** **85**
7I1: Stratified by Maternal height . . . 85
7I2: Stratified by Maternal BMI . . . 86
7I3: Stratified by Maternal age . . . 87
7I4: Stratified by Maternal education . . . 88
7I5: Stratified by Maternal depressive symptoms . . . 89
7I6: Stratified by Child sex . . . 90
7I7: Stratified by Child birth order . . . 91
7I8: Stratified by Child baseline stunting . . . 92
7I9: Stratified by Child baseline acute malnutrition . . . 93
7I10: Stratified by Child baseline anemia . . . 94

**Supplemental figure 7J: Mean difference in gross motor z-score** **95**
7J1: Stratified by Maternal height . . . 95
7J2: Stratified by Maternal BMI . . . 96
7J3: Stratified by Maternal age . . . 97
7J4: Stratified by Maternal education . . . 98
7J5: Stratified by Maternal depressive symptoms . . . 99
7J6: Stratified by Child sex . . . 100
7J7: Stratified by Child birth order . . . 101
7J8: Stratified by Child baseline stunting . . . 102
7J9: Stratified by Child baseline acute malnutrition . . . 103
7J10: Stratified by Child baseline anemia . . . 104

**Supplemental figure 7K: Mean difference in fine motor z-score** **105**
7K1: Stratified by Maternal height . . . 105
7K2: Stratified by Maternal BMI . . . 106
7K3: Stratified by Maternal age . . . 107
7K4: Stratified by Maternal education . . . 108
7K5: Stratified by Maternal depressive symptoms . . . 109
7K6: Stratified by Child sex . . . 110
7K7: Stratified by Child birth order . . . 111
7K8: Stratified by Child baseline stunting . . . 112
7K9: Stratified by Child baseline acute malnutrition . . . 113
7K10: Stratified by Child baseline anemia . . . 114

**Supplemental figure 7L: Mean difference in executive function z-score** **115**
7L1: Stratified by Maternal height . . . 115
7L2: Stratified by Maternal BMI . . . 116
7L3: Stratified by Maternal age . . . 117
7L4: Stratified by Maternal education . . . 118
7L5: Stratified by Maternal depressive symptoms . . . 119
7L6: Stratified by Child sex . . . 120
7L7: Stratified by Child birth order . . . 121
7L8: Stratified by Child baseline stunting . . . 122
7L9: Stratified by Child baseline acute malnutrition . . . 123
7L10: Stratified by Child baseline anemia . . . 124

**Supplemental figure 7M: Executive function lowest decile prevalence ratio** **125**
7M1: Stratified by Maternal height (insufficient comparisons) . . . 125
7M2: Stratified by Maternal BMI . . . 126
7M3: Stratified by Maternal age . . . 127
7M4: Stratified by Maternal education . . . 128
7M5: Stratified by Maternal depressive symptoms . . . 129
7M6: Stratified by Child sex . . . 130
7M7: Stratified by Child birth order . . . 131
7M8: Stratified by Child baseline stunting . . . 132
7M9: Stratified by Child baseline acute malnutrition (insufficient comparisons) . . . 133
7M10: Stratified by Child baseline anemia . . . 134

**Supplemental figure 7N: Executive function lowest decile prevalence difference** **135**
7N1: Stratified by Maternal height (insufficient comparisons) . . . 135
7N2: Stratified by Maternal BMI . . . 136
7N3: Stratified by Maternal age . . . 137
7N4: Stratified by Maternal education . . . 138
7N5: Stratified by Maternal depressive symptoms . . . 139
7N6: Stratified by Child sex . . . 140
7N7: Stratified by Child birth order . . . 141
7N8: Stratified by Child baseline stunting . . . 142
7N9: Stratified by Child baseline acute malnutrition (insufficient comparisons) . . . 143
7N10: Stratified by Child baseline anemia . . . 144

**Supplemental figure 7O: 12-mo walking without support prevalence ratio** **145**
7O1: Stratified by Maternal height . . . 145
7O2: Stratified by Maternal BMI . . . 146
7O3: Stratified by Maternal age . . . 147
7O4: Stratified by Maternal education . . . 148
7O5: Stratified by Maternal depressive symptoms . . . 149
7O6: Stratified by Child sex . . . 150
7O7: Stratified by Child birth order . . . 151
7O8: Stratified by Child baseline stunting . . . 152
7O9: Stratified by Child baseline acute malnutrition . . . 153
7O10: Stratified by Child baseline anemia . . . 154

**Supplemental figure 7P: 12-mo walking without support prevalence difference** **155**
7P1: Stratified by Maternal height . . . 155
7P2: Stratified by Maternal BMI . . . 156
7P3: Stratified by Maternal age . . . 157
7P4: Stratified by Maternal education . . . 158
7P5: Stratified by Maternal depressive symptoms . . . 159
7P6: Stratified by Child sex . . . 160
7P7: Stratified by Child birth order . . . 161
7P8: Stratified by Child baseline stunting . . . 162
7P9: Stratified by Child baseline acute malnutrition . . . 163
7P10: Stratified by Child baseline anemia . . . 164

Supplemental figure 7: Forest plots for effects of SQ-LNS on developmental outcomes stratified by individual-level maternal and child effect modifiers

These figures are forest plots showing the individual-level effect modification of intervention effects. Each figure has the estimates of intervention effect stratified within study by individual-level effect modifier category. For definitions of effect modifiers, see Box 1 in the main paper. Individual study estimates were generated from log-binomial regression for dichotomous outcomes and simple linear regression for continuous outcomes; controlling for baseline measure when available and with clustered observations using robust standard errors for cluster-randomized trials. Pooled interaction term and sub-group estimates were generated using inverse-variance weighting fixed and random effects. For continuous outcomes analyzed via mean differences, the effect estimate is the mean in the LNS group minus the mean in the control group. For dichotomous outcomes analyzed via prevalence ratios, the effect estimate is the prevalence in the LNS group divided by the prevalence in the control group. For dichotomous outcomes analyzed via prevalence differences, the effect estimate is the prevalence in the LNS group minus the prevalence in the control group.

The labels on the far left correspond to trial level information. In the middle left and on the right the values indicate the study level effect estimate, confidence interval, and weighting for deriving the pooled estimates is shown by subgroup.

# Supplemental figure 7A: Mean difference in language z-score

## 7A1: Stratified by Maternal height

**P−for−interaction = 0.857**
**Difference in MDs = −0.01 (−0.09, 0.07)**

| Country | Trial | Tool | At least 150.1 cm: LNS N | Control N | Control Mean | MD (95% CI) | Fixed W | Random W | Less than 150.1 cm: LNS N | Control N | Control Mean | MD (95% CI) | Fixed W | Random W |
|---|---|---|---|---|---|---|---|---|---|---|---|---|---|---|
| Bangladesh | JiVitA−4 (21) | BSID−III | | | | | | | | | | | | |
| Bangladesh | RDNS (22) | CDI | 868 | 431 | −0.10 | 0.25 (0.10, 0.39) | 0.07 | 0.09 | 735 | 358 | −0.13 | 0.07 (−0.06, 0.20) | 0.27 | 0.26 |
| Bangladesh | WASH−B (23) | CDI | 589 | 1812 | 0.02 | 0.08 (−0.01, 0.17) | 0.18 | 0.12 | 515 | 1499 | −0.06 | 0.11 (0.01, 0.21) | 0.46 | 0.33 |
| Burkina Faso | iLiNS−Zinc (24) | DMC | 727 | 365 | −0.25 | 0.38 (0.22, 0.55) | 0.05 | 0.08 | 17 | 10 | 0.06 | −0.14 (−0.97, 0.69) | 0.01 | 0.01 |
| Ghana | GHANA (25) | | | | | | | | | | | | | |
| Ghana | iLiNS−DYAD−G (26) | CDI | 310 | 612 | 0.01 | 0.00 (−0.13, 0.14) | 0.07 | 0.10 | 16 | 35 | −0.23 | 0.23 (−0.33, 0.79) | 0.02 | 0.03 |
| Haiti | HAITI (27) | Total sounds | | | | | | | | | | | | |
| Kenya | WASH−B (28) | EASQ | 1237 | 4372 | 0.01 | 0.01 (−0.06, 0.08) | 0.32 | 0.13 | 51 | 175 | −0.21 | 0.34 (0.03, 0.65) | 0.05 | 0.08 |
| Madagascar | MAHAY (29) | ASQI | 956 | 986 | 0.06 | −0.07 (−0.27, 0.13) | 0.04 | 0.07 | 553 | 538 | 0.08 | −0.18 (−0.40, 0.04) | 0.10 | 0.14 |
| Malawi | iLiNS−DYAD−M (30) | CDI | 187 | 378 | 0.02 | 0.01 (−0.17, 0.18) | 0.05 | 0.08 | 28 | 58 | −0.15 | 0.07 (−0.38, 0.51) | 0.02 | 0.04 |
| Malawi | iLiNS−DOSE (31) | CDI | 542 | 185 | −0.05 | 0.06 (−0.11, 0.22) | 0.05 | 0.08 | 102 | 35 | −0.01 | 0.04 (−0.36, 0.44) | 0.03 | 0.05 |
| Mali | PROMIS CS (32) | DMC | 889 | 911 | −0.06 | 0.14 (−0.02, 0.31) | 0.05 | 0.08 | 32 | 27 | −0.27 | 0.39 (−0.15, 0.92) | 0.02 | 0.03 |
| Zimbabwe | SHINE (HIV−) (33) | CDI | 766 | 745 | −0.05 | 0.09 (−0.03, 0.21) | 0.10 | 0.10 | 32 | 21 | −0.12 | −0.08 (−0.67, 0.51) | 0.01 | 0.02 |
| Zimbabwe | SHINE (HIV+) (34) | CDI | 153 | 136 | −0.11 | 0.22 (−0.02, 0.45) | 0.03 | 0.06 | 10 | 6 | −0.39 | 0.69 (−0.31, 1.68) | 0.00 | 0.01 |
| | | | **7224** | **10933** | | **I² = 0.64, Tau² = 0.01** | | | **2091** | **2762** | | **I² = 0.18, Tau² = 0.00** | | |
| **Fixed** | | | | | | **0.08 (0.04, 0.11)** | | | | | | **0.08 (0.01, 0.15)** | | |
| **Random** | | | | | | **0.10 (0.03, 0.18)** | | | | | | **0.08 (−0.01, 0.18)** | | |

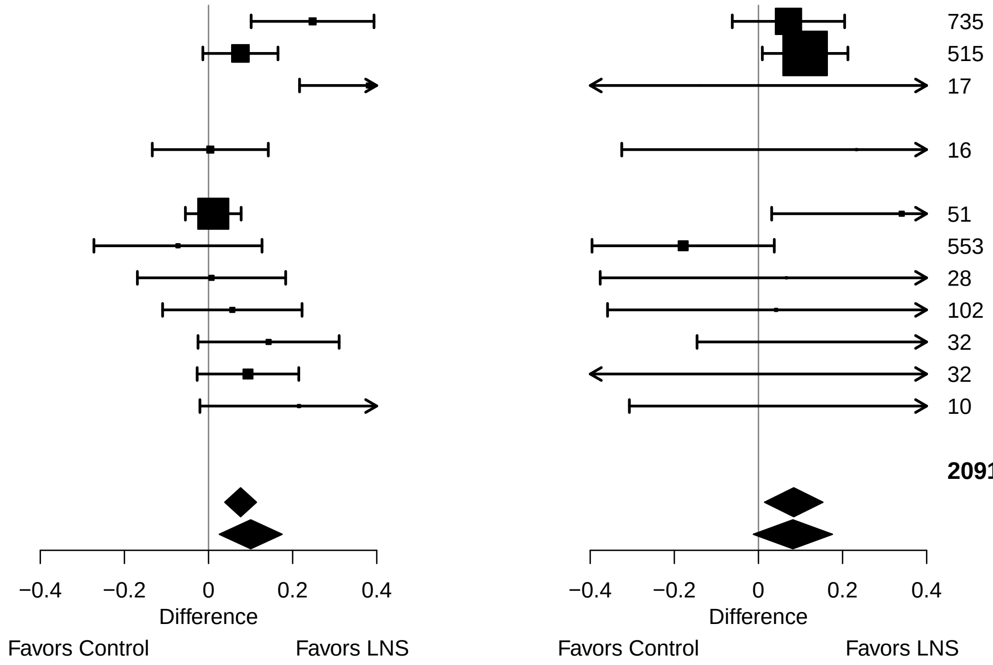

## Supplemental figure 7A: Mean difference in language z-score

## 7A2: Stratified by Maternal BMI

**P−for−interaction = 0.194**
**Difference in MDs = −0.04 (−0.11, 0.02)**

| | | | At least 20 kg/m² | | | | | | Less than 20 kg/m² | | | | | |
|---|---|---|---|---|---|---|---|---|---|---|---|---|---|---|
| Country | Trial | Tool | LNS N | Control N | Control Mean | MD (95% CI) | Fixed W | Random W | LNS N | Control N | Control Mean | MD (95% CI) | Fixed W | Random W |
| Bangladesh | JiVitA−4 (21) | BSID−III | | | | | | | | | | | | |
| Bangladesh | RDNS (22) | CDI | 730 | 334 | −0.13 | 0.26 (0.13, 0.40) | 0.09 | 0.11 | 873 | 455 | −0.11 | 0.09 (−0.02, 0.19) | 0.25 | 0.15 |
| Bangladesh | WASH−B (23) | CDI | 498 | 1529 | 0.06 | 0.11 (0.00, 0.21) | 0.16 | 0.14 | 606 | 1781 | −0.08 | 0.08 (−0.01, 0.18) | 0.31 | 0.15 |
| Burkina Faso | iLiNS−Zinc (24) | DMC | 480 | 225 | −0.16 | 0.32 (0.13, 0.51) | 0.04 | 0.08 | 264 | 150 | −0.37 | 0.43 (0.23, 0.63) | 0.07 | 0.11 |
| Ghana | GHANA (25) | | | | | | | | | | | | | |
| Ghana | iLiNS−DYAD−G (26) | CDI | 291 | 531 | 0.00 | 0.03 (−0.12, 0.17) | 0.08 | 0.11 | 35 | 116 | −0.04 | −0.10 (−0.48, 0.27) | 0.02 | 0.06 |
| Haiti | HAITI (27) | Total sounds | | | | | | | | | | | | |
| Kenya | WASH−B (28) | EASQ | 1031 | 3535 | 0.01 | 0.01 (−0.06, 0.08) | 0.36 | 0.17 | 257 | 1012 | −0.04 | 0.07 (−0.07, 0.20) | 0.15 | 0.14 |
| Madagascar | MAHAY (29) | ASQI | | | | | | | | | | | | |
| Malawi | iLiNS−DYAD−M (30) | CDI | 127 | 261 | −0.01 | −0.01 (−0.23, 0.21) | 0.04 | 0.07 | 87 | 175 | −0.01 | 0.07 (−0.17, 0.32) | 0.05 | 0.09 |
| Malawi | iLiNS−DOSE (31) | CDI | 479 | 158 | −0.14 | 0.18 (0.00, 0.35) | 0.05 | 0.08 | 163 | 61 | 0.21 | −0.27 (−0.57, 0.04) | 0.03 | 0.08 |
| Mali | PROMIS CS (32) | DMC | 683 | 664 | −0.07 | 0.18 (0.00, 0.37) | 0.05 | 0.08 | 238 | 274 | −0.07 | 0.06 (−0.13, 0.25) | 0.08 | 0.11 |
| Zimbabwe | SHINE (HIV−) (33) | CDI | 581 | 569 | −0.03 | 0.08 (−0.05, 0.20) | 0.10 | 0.12 | 96 | 93 | −0.13 | 0.21 (−0.08, 0.51) | 0.03 | 0.08 |
| Zimbabwe | SHINE (HIV+) (34) | CDI | 121 | 110 | −0.11 | 0.23 (−0.02, 0.48) | 0.03 | 0.05 | 23 | 22 | −0.51 | 0.37 (−0.21, 0.95) | 0.01 | 0.03 |
| | | | **5021** | **7916** | | **I² = 0.58, Tau² = 0.01** | | | **2642** | **4139** | | **I² = 0.54, Tau² = 0.02** | | |
| **Fixed** | | | | | | **0.09 (0.05, 0.14)** | | | | | | **0.10 (0.04, 0.15)** | | |
| **Random** | | | | | | **0.12 (0.06, 0.19)** | | | | | | **0.10 (−0.01, 0.21)** | | |

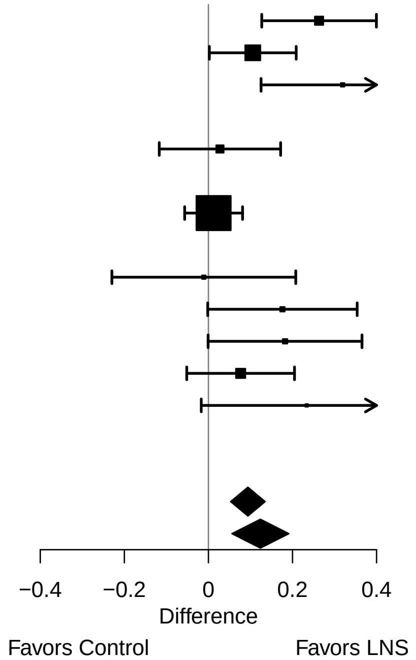


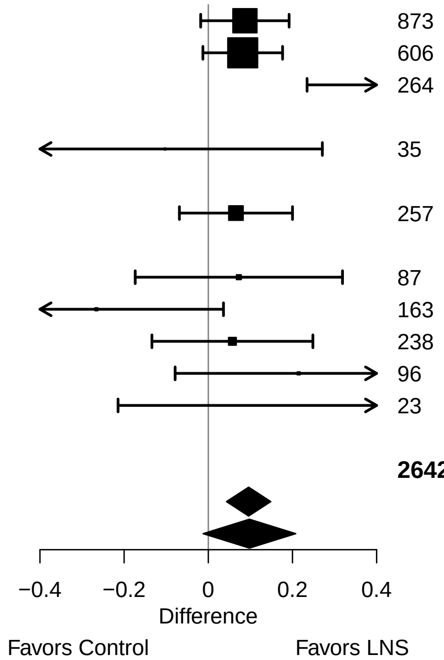

## Supplemental figure 7A: Mean difference in language z-score

## 7A3: Stratified by Maternal age

**P−for−interaction = 0.361**
**Difference in MDs = −0.03 (−0.08, 0.03)**

| | | | At least 25 y | | | | | | Less than 25 y | | | | | |
|---|---|---|---|---|---|---|---|---|---|---|---|---|---|---|
| **Country** | **Trial** | **Tool** | **LNS N** | **Control N** | **Control Mean** | **MD (95% CI)** | **Fixed W** | **Random W** | **LNS N** | **Control N** | **Control Mean** | **MD (95% CI)** | **Fixed W** | **Random W** |
| Bangladesh | JiVitA−4 (21) | BSID−III | 200 | 68 | 0.06 | −0.14 (−0.42, 0.14) | 0.03 | 0.06 | 242 | 74 | 0.05 | −0.03 (−0.27, 0.20) | 0.03 | 0.05 |
| Bangladesh | RDNS (22) | CDI | 453 | 220 | −0.22 | 0.24 (0.03, 0.45) | 0.05 | 0.07 | 1210 | 594 | −0.07 | 0.14 (0.04, 0.24) | 0.17 | 0.15 |
| Bangladesh | WASH−B (23) | CDI | 477 | 1475 | −0.04 | 0.05 (−0.08, 0.17) | 0.14 | 0.10 | 627 | 1863 | −0.01 | 0.12 (0.04, 0.20) | 0.27 | 0.18 |
| Burkina Faso | iLiNS−Zinc (24) | DMC | 421 | 232 | −0.29 | 0.47 (0.33, 0.62) | 0.11 | 0.09 | 321 | 141 | −0.16 | 0.20 (−0.09, 0.49) | 0.02 | 0.03 |
| Ghana | GHANA (25) | | | | | | | | | | | | | |
| Ghana | iLiNS−DYAD−G (26) | CDI | 211 | 405 | 0.05 | 0.11 (−0.07, 0.28) | 0.07 | 0.08 | 120 | 253 | −0.08 | −0.17 (−0.37, 0.03) | 0.04 | 0.06 |
| Haiti | HAITI (27) | Total sounds | 102 | 103 | 0.06 | −0.16 (−0.43, 0.11) | 0.03 | 0.06 | 47 | 45 | 0.05 | −0.03 (−0.44, 0.38) | 0.01 | 0.02 |
| Kenya | WASH−B (28) | EASQ | 798 | 2622 | 0.02 | 0.01 (−0.08, 0.10) | 0.25 | 0.10 | 549 | 2092 | −0.03 | 0.03 (−0.05, 0.12) | 0.24 | 0.18 |
| Madagascar | MAHAY (29) | ASQI | 852 | 901 | 0.03 | −0.09 (−0.28, 0.10) | 0.06 | 0.08 | 759 | 703 | 0.08 | −0.13 (−0.33, 0.06) | 0.04 | 0.07 |
| Malawi | iLiNS−DYAD−M (30) | CDI | 103 | 225 | 0.02 | 0.09 (−0.15, 0.33) | 0.04 | 0.06 | 112 | 214 | −0.03 | −0.05 (−0.27, 0.17) | 0.04 | 0.06 |
| Malawi | iLiNS−DOSE (31) | CDI | 345 | 124 | −0.05 | 0.01 (−0.19, 0.22) | 0.05 | 0.07 | 288 | 90 | 0.00 | 0.08 (−0.15, 0.31) | 0.03 | 0.05 |
| Mali | PROMIS CS (32) | DMC | 492 | 492 | −0.07 | 0.17 (−0.02, 0.36) | 0.06 | 0.08 | 435 | 452 | −0.07 | 0.12 (−0.07, 0.31) | 0.05 | 0.07 |
| Zimbabwe | SHINE (HIV−) (33) | CDI | 414 | 384 | 0.06 | 0.10 (−0.06, 0.25) | 0.09 | 0.09 | 315 | 310 | −0.18 | 0.10 (−0.08, 0.27) | 0.05 | 0.08 |
| Zimbabwe | SHINE (HIV+) (34) | CDI | 122 | 107 | −0.08 | 0.20 (−0.08, 0.47) | 0.03 | 0.06 | 29 | 26 | −0.22 | 0.25 (−0.32, 0.82) | 0.01 | 0.01 |
| | | | **4990** | **7358** | | **I² = 0.73, Tau² = 0.02** | | | **5054** | **6857** | | **I² = 0.31, Tau² = 0.00** | | |
| **Fixed** | | | | | | **0.09 (0.05, 0.14)** | | | | | | **0.07 (0.03, 0.11)** | | |
| **Random** | | | | | | **0.09 (0.00, 0.18)** | | | | | | **0.05 (0.00, 0.11)** | | |

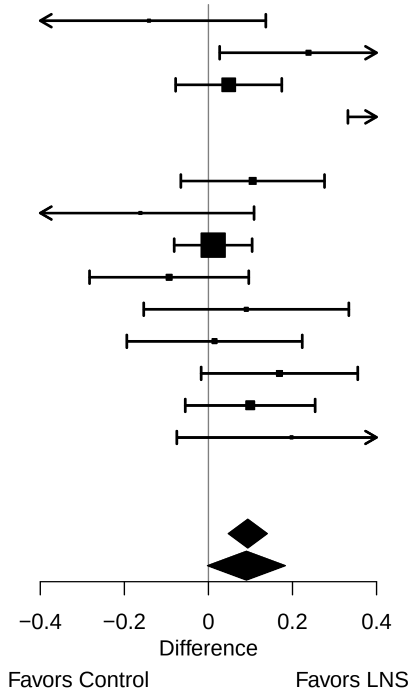


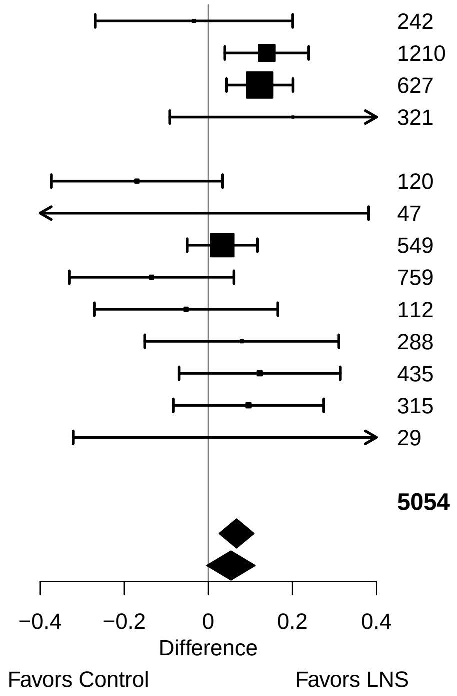

### Supplemental figure 7A: Mean difference in language z-score

## 7A4: Stratified by Maternal education

**P−for−interaction = 0.411**
**Difference in MDs = 0.03 (−0.04, 0.09)**

| Country | Trial | Tool | LNS N | Control N | Control Mean | Primary or greater MD (95% CI) | Fixed W | Random W | LNS N | Control N | Control Mean | Incomplete or no formal MD (95% CI) | Fixed W | Random W |
|---|---|---|---|---|---|---|---|---|---|---|---|---|---|---|
| Bangladesh | JiVitA−4 (21) | BSID−III | 292 | 92 | 0.25 | −0.15 (−0.41, 0.11) | 0.02 | 0.06 | 152 | 51 | −0.27 | 0.03 (−0.20, 0.25) | 0.04 | 0.07 |
| Bangladesh | RDNS (22) | CDI | 1245 | 588 | −0.03 | 0.17 (0.07, 0.27) | 0.15 | 0.13 | 418 | 226 | −0.33 | 0.13 (−0.03, 0.30) | 0.08 | 0.10 |
| Bangladesh | WASH−B (23) | CDI | 781 | 2406 | 0.08 | 0.12 (0.04, 0.20) | 0.25 | 0.14 | 328 | 947 | −0.28 | 0.05 (−0.08, 0.18) | 0.12 | 0.12 |
| Burkina Faso | iLiNS−Zinc (24) | DMC | 32 | 9 | 0.06 | 0.10 (−0.48, 0.67) | 0.00 | 0.02 | 712 | 366 | −0.25 | 0.37 (0.21, 0.54) | 0.08 | 0.10 |
| Ghana | GHANA (25) | | | | | | | | | | | | | |
| Ghana | iLiNS−DYAD−G (26) | CDI | 256 | 519 | 0.01 | −0.03 (−0.17, 0.12) | 0.07 | 0.10 | 75 | 139 | −0.05 | 0.13 (−0.16, 0.43) | 0.03 | 0.05 |
| Haiti | HAITI (27) | Total sounds | 130 | 126 | 0.11 | −0.18 (−0.42, 0.07) | 0.03 | 0.06 | 20 | 23 | −0.13 | 0.17 (−0.40, 0.74) | 0.01 | 0.02 |
| Kenya | WASH−B (28) | EASQ | 658 | 2258 | 0.15 | 0.01 (−0.07, 0.08) | 0.27 | 0.14 | 702 | 2484 | −0.15 | 0.03 (−0.05, 0.11) | 0.34 | 0.16 |
| Madagascar | MAHAY (29) | ASQI | 339 | 416 | 0.43 | −0.25 (−0.49, −0.01) | 0.03 | 0.06 | 1274 | 1188 | −0.08 | −0.04 (−0.22, 0.13) | 0.07 | 0.09 |
| Malawi | iLiNS−DYAD−M (30) | CDI | 33 | 69 | 0.17 | 0.21 (−0.16, 0.57) | 0.01 | 0.03 | 181 | 367 | −0.03 | −0.02 (−0.20, 0.16) | 0.07 | 0.09 |
| Malawi | iLiNS−DOSE (31) | CDI | 148 | 52 | 0.11 | 0.07 (−0.24, 0.38) | 0.02 | 0.04 | 486 | 162 | −0.07 | 0.04 (−0.14, 0.22) | 0.07 | 0.09 |
| Mali | PROMIS CS (32) | DMC | 100 | 94 | 0.17 | −0.03 (−0.37, 0.30) | 0.01 | 0.04 | 826 | 850 | −0.10 | 0.17 (0.00, 0.33) | 0.08 | 0.10 |
| Zimbabwe | SHINE (HIV−) (33) | CDI | 742 | 715 | −0.04 | 0.11 (−0.01, 0.23) | 0.10 | 0.12 | 27 | 24 | −0.12 | −0.42 (−1.04, 0.20) | 0.01 | 0.01 |
| Zimbabwe | SHINE (HIV+) (34) | CDI | 145 | 126 | −0.09 | 0.22 (−0.01, 0.46) | 0.03 | 0.06 | 9 | 8 | −0.18 | −0.09 (−1.06, 0.88) | 0.00 | 0.01 |
| | | | **4901** | **7470** | | **I² = 0.53, Tau² = 0.01** | | | **5210** | **6835** | | **I² = 0.45, Tau² = 0.01** | | |
| **Fixed** | | | | | | **0.06 (0.02, 0.10)** | | | | | | **0.07 (0.03, 0.12)** | | |
| **Random** | | | | | | **0.04 (−0.04, 0.11)** | | | | | | **0.08 (0.01, 0.15)** | | |

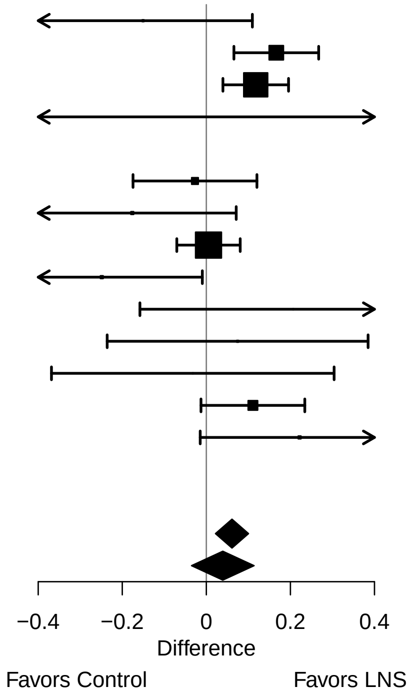


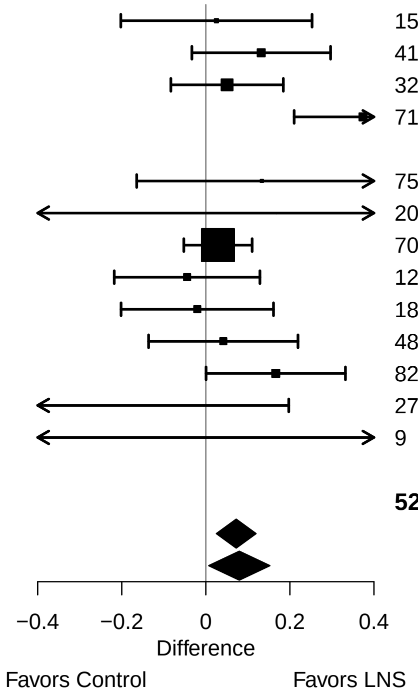

## Supplemental figure 7A: Mean difference in language z-score

### 7A5: Stratified by Maternal depressive symptoms

**P−for−interaction = 0.950**
**Difference in MDs = 0.00 (−0.07, 0.08)**

| Country | Trial | Tool | LNS N | Control N | Control Mean | Less than 75th percentile MD (95% CI) | Fixed W | Random W | LNS N | Control N | Control Mean | At least 75th percentile MD (95% CI) | Fixed W | Random W |
|---|---|---|---|---|---|---|---|---|---|---|---|---|---|---|
| Bangladesh | JiVitA−4 (21) | BSID−III | | | | | | | | | | | | |
| Bangladesh | RDNS (22) | CDI | 1081 | 471 | −0.06 | 0.18 (0.06, 0.31) | 0.11 | 0.14 | 520 | 288 | −0.21 | 0.11 (−0.03, 0.25) | 0.23 | 0.23 |
| Bangladesh | WASH−B (23) | CDI | 856 | 2410 | 0.01 | 0.11 (0.02, 0.19) | 0.25 | 0.17 | 232 | 859 | −0.12 | 0.02 (−0.13, 0.17) | 0.20 | 0.20 |
| Burkina Faso | iLiNS−Zinc (24) | DMC | | | | | | | | | | | | |
| Ghana | GHANA (25) | | | | | | | | | | | | | |
| Ghana | iLiNS−DYAD−G (26) | CDI | 241 | 435 | 0.00 | −0.01 (−0.17, 0.15) | 0.07 | 0.12 | 78 | 202 | 0.00 | 0.10 (−0.16, 0.36) | 0.07 | 0.07 |
| Haiti | HAITI (27) | Total sounds | | | | | | | | | | | | |
| Kenya | WASH−B (28) | EASQ | 960 | 3317 | 0.01 | 0.01 (−0.06, 0.08) | 0.37 | 0.18 | 317 | 1156 | 0.11 | 0.02 (−0.11, 0.14) | 0.29 | 0.29 |
| Madagascar | MAHAY (29) | ASQI | 615 | 667 | 0.08 | −0.17 (−0.43, 0.10) | 0.02 | 0.07 | 268 | 242 | 0.03 | −0.01 (−0.28, 0.27) | 0.06 | 0.06 |
| Malawi | iLiNS−DYAD−M (30) | CDI | 153 | 302 | −0.01 | 0.01 (−0.19, 0.20) | 0.05 | 0.10 | 47 | 112 | 0.00 | 0.11 (−0.25, 0.48) | 0.03 | 0.03 |
| Malawi | iLiNS−DOSE (31) | CDI | | | | | | | | | | | | |
| Mali | PROMIS CS (32) | DMC | | | | | | | | | | | | |
| Zimbabwe | SHINE (HIV−) (33) | CDI | 566 | 552 | −0.02 | 0.06 (−0.07, 0.19) | 0.10 | 0.14 | 190 | 173 | −0.12 | 0.21 (0.00, 0.42) | 0.10 | 0.10 |
| Zimbabwe | SHINE (HIV+) (34) | CDI | 127 | 107 | −0.22 | 0.35 (0.11, 0.60) | 0.03 | 0.08 | 32 | 31 | 0.32 | −0.32 (−0.91, 0.26) | 0.01 | 0.01 |
| | | | **4599** | **8261** | | **I² = 0.57, Tau² = 0.01** | | | **1684** | **3063** | | **I² = 0.00, Tau² = 0.00** | | |
| **Fixed** | | | | | | **0.06 (0.02, 0.11)** | | | | | | **0.06 (−0.01, 0.13)** | | |
| **Random** | | | | | | **0.07 (−0.02, 0.16)** | | | | | | **0.06 (−0.01, 0.13)** | | |

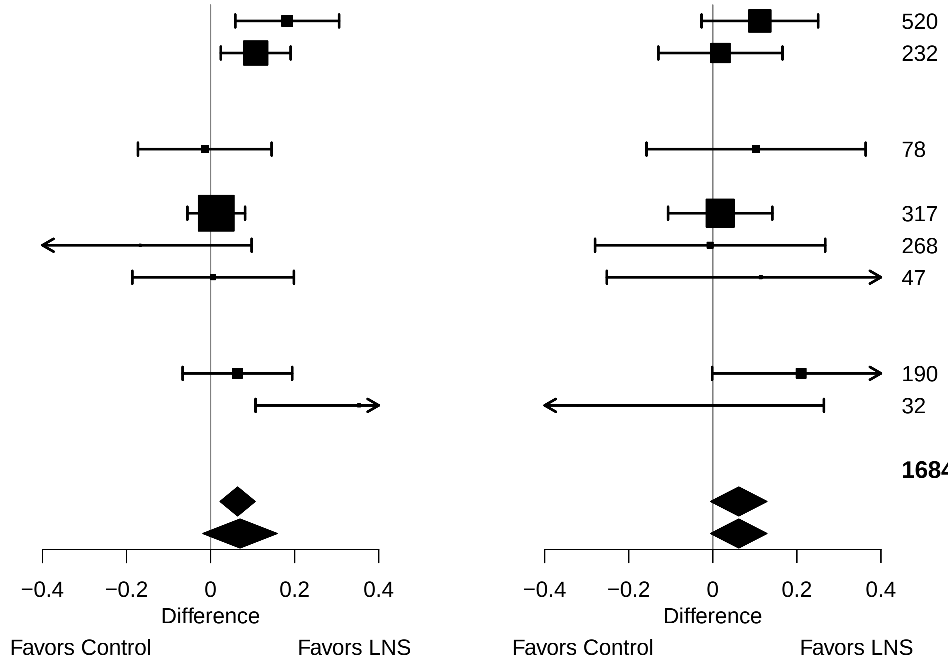

## Supplemental figure 7A: Mean difference in language z-score

### 7A6: Stratified by Child sex

**P−for−interaction = 0.424**
**Difference in MDs = 0.02 (−0.03, 0.08)**

**Male**

| Country | Trial | Tool | LNS N | Control N | Control Mean | MD (95% CI) | Fixed W | Random W |
|---|---|---|---|---|---|---|---|---|
| Bangladesh | JiVitA−4 (21) | BSID−III | 231 | 70 | 0.04 | −0.05 (−0.35, 0.25) | 0.02 | 0.03 |
| Bangladesh | RDNS (22) | CDI | 833 | 411 | −0.04 | 0.05 (−0.07, 0.18) | 0.13 | 0.13 |
| Bangladesh | WASH−B (23) | CDI | 545 | 1685 | −0.02 | 0.09 (−0.01, 0.19) | 0.19 | 0.15 |
| Burkina Faso | iLiNS−Zinc (24) | DMC | 372 | 193 | −0.21 | 0.32 (0.10, 0.55) | 0.04 | 0.05 |
| Ghana | GHANA (25) | | | | | | | |
| Ghana | iLiNS−DYAD−G (26) | CDI | 164 | 306 | 0.03 | −0.08 (−0.28, 0.11) | 0.05 | 0.06 |
| Haiti | HAITI (27) | Total sounds | 71 | 60 | 0.04 | −0.08 (−0.43, 0.28) | 0.02 | 0.02 |
| Kenya | WASH−B (28) | EASQ | 682 | 2260 | 0.00 | −0.01 (−0.10, 0.08) | 0.25 | 0.17 |
| Madagascar | MAHAY (29) | ASQI | 795 | 787 | 0.04 | −0.08 (−0.26, 0.10) | 0.06 | 0.07 |
| Malawi | iLiNS−DYAD−M (30) | CDI | 105 | 205 | −0.03 | 0.10 (−0.13, 0.33) | 0.04 | 0.05 |
| Malawi | iLiNS−DOSE (31) | CDI | 320 | 117 | −0.05 | 0.07 (−0.15, 0.28) | 0.04 | 0.06 |
| Mali | PROMIS CS (32) | DMC | 469 | 514 | −0.08 | 0.17 (0.00, 0.33) | 0.07 | 0.08 |
| Zimbabwe | SHINE (HIV−) (33) | CDI | 412 | 378 | −0.07 | 0.14 (−0.03, 0.31) | 0.07 | 0.08 |
| Zimbabwe | SHINE (HIV+) (34) | CDI | 82 | 76 | −0.08 | 0.15 (−0.18, 0.48) | 0.02 | 0.03 |
| | | | **5081** | **7062** | | **I² = 0.26, Tau² = 0.00** | | |
| **Fixed** | | | | | | **0.05 (0.01, 0.10)** | | |
| **Random** | | | | | | **0.06 (0.00, 0.12)** | | |

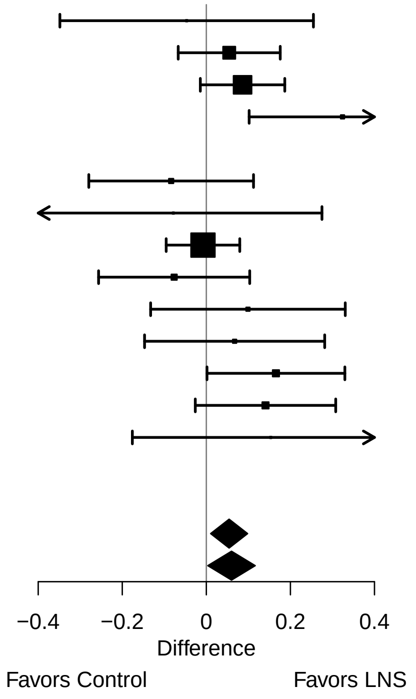


**Female**

| Country | Trial | LNS N | Control N | Control Mean | MD (95% CI) | Fixed W | Random W |
|---|---|---|---|---|---|---|---|
| Bangladesh | JiVitA−4 (21) | 214 | 73 | 0.09 | −0.12 (−0.34, 0.10) | 0.04 | 0.07 |
| Bangladesh | RDNS (22) | 830 | 403 | −0.18 | 0.28 (0.14, 0.42) | 0.10 | 0.09 |
| Bangladesh | WASH−B (23) | 564 | 1668 | −0.03 | 0.10 (0.01, 0.19) | 0.21 | 0.10 |
| Burkina Faso | iLiNS−Zinc (24) | 374 | 182 | −0.28 | 0.41 (0.25, 0.58) | 0.07 | 0.08 |
| Ghana | GHANA (25) | | | | | | |
| Ghana | iLiNS−DYAD−G (26) | 167 | 352 | −0.03 | 0.10 (−0.08, 0.27) | 0.06 | 0.08 |
| Haiti | HAITI (27) | 79 | 89 | 0.09 | −0.17 (−0.47, 0.13) | 0.02 | 0.05 |
| Kenya | WASH−B (28) | 680 | 2485 | −0.01 | 0.05 (−0.04, 0.13) | 0.26 | 0.10 |
| Madagascar | MAHAY (29) | 818 | 817 | 0.07 | −0.15 (−0.36, 0.06) | 0.04 | 0.07 |
| Malawi | iLiNS−DYAD−M (30) | 110 | 234 | 0.02 | −0.06 (−0.30, 0.17) | 0.03 | 0.07 |
| Malawi | iLiNS−DOSE (31) | 325 | 104 | −0.02 | 0.02 (−0.20, 0.24) | 0.04 | 0.07 |
| Mali | PROMIS CS (32) | 458 | 430 | −0.07 | 0.13 (−0.08, 0.33) | 0.04 | 0.07 |
| Zimbabwe | SHINE (HIV−) (33) | 405 | 403 | −0.01 | 0.03 (−0.14, 0.20) | 0.07 | 0.08 |
| Zimbabwe | SHINE (HIV+) (34) | 83 | 71 | −0.19 | 0.35 (0.04, 0.65) | 0.02 | 0.05 |
| | | **5107** | **7311** | | **I² = 0.69, Tau² = 0.02** | | |
| **Fixed** | | | | | **0.09 (0.05, 0.14)** | | |
| **Random** | | | | | **0.08 (−0.01, 0.18)** | | |

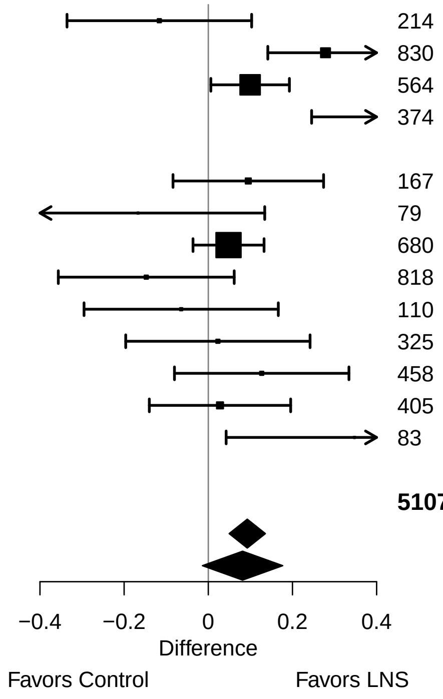

## Supplemental figure 7A: Mean difference in language z-score

### 7A7: Stratified by Child birth order

**P−for−interaction = 0.519**
**Difference in MDs = −0.02 (−0.09, 0.04)**

| Country | Trial | Tool | Later born LNS N | Later born Control N | Later born Control Mean | Later born MD (95% CI) | Later born Fixed W | Later born Random W | Firstborn LNS N | Firstborn Control N | Firstborn Control Mean | Firstborn MD (95% CI) | Firstborn Fixed W | Firstborn Random W |
|---|---|---|---|---|---|---|---|---|---|---|---|---|---|---|
| Bangladesh | JiVitA−4 (21) | BSID−III | 111 | 31 | −0.08 | −0.14 (−0.60, 0.32) | 0.01 | 0.02 | 333 | 111 | 0.11 | −0.06 (−0.29, 0.17) | 0.06 | 0.08 |
| Bangladesh | RDNS (22) | CDI | 974 | 507 | −0.21 | 0.24 (0.13, 0.35) | 0.13 | 0.10 | 688 | 307 | 0.06 | 0.04 (−0.12, 0.20) | 0.12 | 0.11 |
| Bangladesh | WASH−B (23) | CDI | 693 | 2187 | −0.07 | 0.08 (−0.02, 0.17) | 0.15 | 0.11 | 395 | 1060 | 0.09 | 0.10 (0.00, 0.21) | 0.27 | 0.13 |
| Burkina Faso | iLiNS−Zinc (24) | DMC | 587 | 290 | −0.25 | 0.41 (0.25, 0.57) | 0.06 | 0.08 | 159 | 85 | −0.21 | 0.22 (−0.14, 0.57) | 0.02 | 0.05 |
| Ghana | GHANA (25) | | | | | | | | | | | | | |
| Ghana | iLiNS−DYAD−G (26) | CDI | 224 | 445 | 0.04 | 0.05 (−0.11, 0.22) | 0.06 | 0.08 | 107 | 213 | −0.10 | −0.08 (−0.30, 0.13) | 0.07 | 0.09 |
| Haiti | HAITI (27) | Total sounds | 89 | 99 | −0.01 | −0.06 (−0.33, 0.22) | 0.02 | 0.05 | 61 | 50 | 0.22 | −0.27 (−0.67, 0.12) | 0.02 | 0.04 |
| Kenya | WASH−B (28) | EASQ | 1071 | 3767 | 0.00 | 0.01 (−0.06, 0.08) | 0.30 | 0.12 | 289 | 974 | −0.03 | 0.07 (−0.05, 0.19) | 0.22 | 0.12 |
| Madagascar | MAHAY (29) | ASQI | 1162 | 1118 | 0.01 | −0.10 (−0.28, 0.08) | 0.05 | 0.07 | 433 | 465 | 0.16 | −0.14 (−0.38, 0.09) | 0.06 | 0.08 |
| Malawi | iLiNS−DYAD−M (30) | CDI | 170 | 350 | 0.03 | 0.03 (−0.15, 0.22) | 0.04 | 0.07 | 45 | 87 | −0.14 | −0.04 (−0.37, 0.29) | 0.03 | 0.06 |
| Malawi | iLiNS−DOSE (31) | CDI | 424 | 151 | −0.09 | 0.05 (−0.14, 0.24) | 0.04 | 0.07 | 134 | 41 | 0.18 | 0.03 (−0.31, 0.37) | 0.03 | 0.05 |
| Mali | PROMIS CS (32) | DMC | 770 | 811 | −0.07 | 0.16 (−0.01, 0.34) | 0.05 | 0.08 | 142 | 122 | −0.05 | 0.08 (−0.25, 0.40) | 0.03 | 0.06 |
| Zimbabwe | SHINE (HIV−) (33) | CDI | 587 | 562 | 0.06 | 0.06 (−0.08, 0.20) | 0.08 | 0.09 | 194 | 185 | −0.34 | 0.19 (−0.03, 0.40) | 0.07 | 0.09 |
| Zimbabwe | SHINE (HIV+) (34) | CDI | 144 | 114 | −0.06 | 0.14 (−0.13, 0.41) | 0.02 | 0.05 | 18 | 29 | −0.42 | 0.77 (0.28, 1.27) | 0.01 | 0.03 |
| | | | **7006** | **10432** | | **I² = 0.66, Tau² = 0.01** | | | **2998** | **3729** | | **I² = 0.38, Tau² = 0.02** | | |
| **Fixed** | | | | | | **0.08 (0.04, 0.12)** | | | | | | **0.05 (0.00, 0.11)** | | |
| **Random** | | | | | | **0.09 (0.01, 0.16)** | | | | | | **0.05 (−0.05, 0.14)** | | |

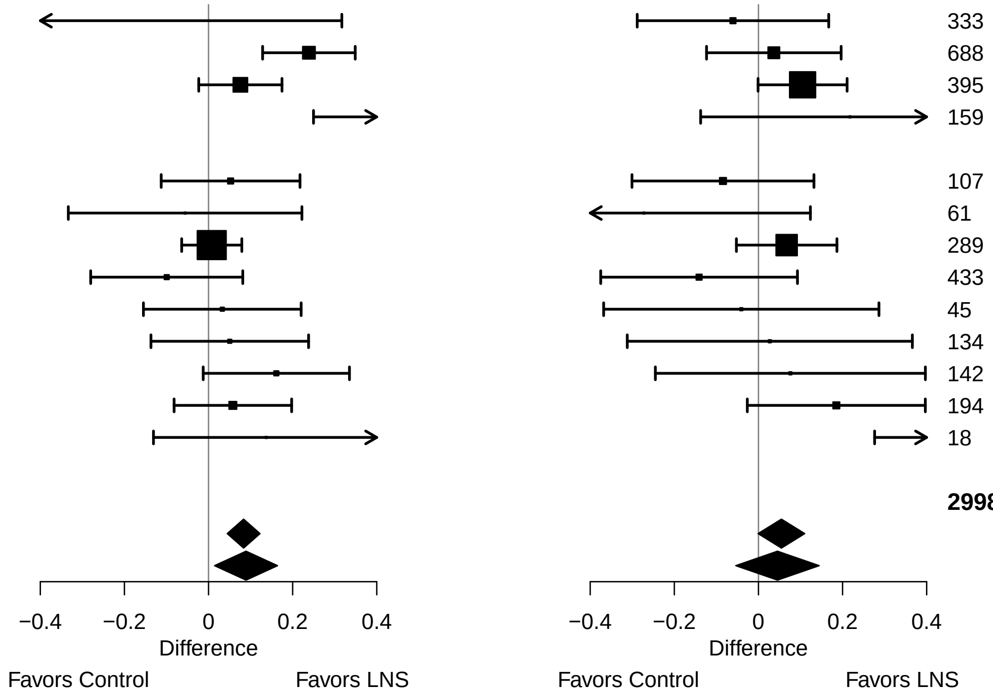

## Supplemental figure 7A: Mean difference in language z-score

## 7A8: Stratified by Child baseline stunting

**P−for−interaction = 0.255**
**Difference in MDs = 0.07 (−0.05, 0.20)**

| | | | No | | | | | | Yes | | | | | |
|---|---|---|---|---|---|---|---|---|---|---|---|---|---|---|
| **Country** | **Trial** | **Tool** | **LNS N** | **Control N** | **Control Mean** | **MD (95% CI)** | **Fixed W** | **Random W** | **LNS N** | **Control N** | **Control Mean** | **MD (95% CI)** | **Fixed W** | **Random W** |
| Bangladesh | JiVitA−4 (21) | BSID−III | 327 | 114 | 0.15 | −0.08 (−0.33, 0.17) | 0.04 | 0.08 | 116 | 29 | −0.30 | 0.03 (−0.32, 0.38) | 0.10 | 0.10 |
| Bangladesh | RDNS (22) | CDI | 1246 | 573 | −0.03 | 0.13 (0.04, 0.22) | 0.35 | 0.16 | 355 | 185 | −0.38 | 0.26 (0.01, 0.51) | 0.19 | 0.19 |
| Bangladesh | WASH−B (23) | CDI | | | | | | | | | | | | |
| Burkina Faso | iLiNS−Zinc (24) | DMC | 576 | 294 | −0.18 | 0.37 (0.17, 0.57) | 0.07 | 0.11 | 170 | 80 | −0.47 | 0.37 (0.13, 0.60) | 0.22 | 0.22 |
| Ghana | GHANA (25) | | | | | | | | | | | | | |
| Ghana | iLiNS−DYAD−G (26) | CDI | 279 | 548 | 0.04 | −0.03 (−0.18, 0.11) | 0.14 | 0.13 | 28 | 63 | −0.28 | 0.21 (−0.26, 0.68) | 0.05 | 0.05 |
| Haiti | HAITI (27) | Total sounds | 137 | 132 | 0.09 | −0.11 (−0.35, 0.13) | 0.05 | 0.09 | 12 | 17 | −0.08 | −0.35 (−1.09, 0.38) | 0.02 | 0.02 |
| Kenya | WASH−B (28) | EASQ | | | | | | | | | | | | |
| Madagascar | MAHAY (29) | ASQI | | | | | | | | | | | | |
| Malawi | iLiNS−DYAD−M (30) | CDI | 153 | 323 | 0.04 | 0.00 (−0.19, 0.19) | 0.08 | 0.11 | 48 | 97 | −0.25 | 0.24 (−0.12, 0.61) | 0.09 | 0.09 |
| Malawi | iLiNS−DOSE (31) | CDI | 464 | 147 | 0.07 | −0.02 (−0.20, 0.16) | 0.09 | 0.11 | 181 | 74 | −0.24 | 0.15 (−0.12, 0.43) | 0.16 | 0.16 |
| Mali | PROMIS CS (32) | DMC | | | | | | | | | | | | |
| Zimbabwe | SHINE (HIV−) (33) | CDI | 523 | 482 | −0.01 | 0.16 (0.01, 0.30) | 0.14 | 0.13 | 119 | 100 | −0.25 | −0.03 (−0.34, 0.28) | 0.13 | 0.13 |
| Zimbabwe | SHINE (HIV+) (34) | CDI | 112 | 87 | −0.04 | 0.24 (−0.03, 0.51) | 0.04 | 0.08 | 33 | 32 | −0.19 | 0.20 (−0.38, 0.79) | 0.04 | 0.04 |
| | | | **3817** | **2700** | | **$I^2$ = 0.60, $Tau^2$ = 0.01** | | | **1062** | **677** | | **$I^2$ = 0.00, $Tau^2$ = 0.00** | | |
| **Fixed** | | | | | | **0.09 (0.03, 0.14)** | | | | | | **0.19 (0.08, 0.30)** | | |
| **Random** | | | | | | **0.07 (−0.03, 0.18)** | | | | | | **0.19 (0.08, 0.30)** | | |

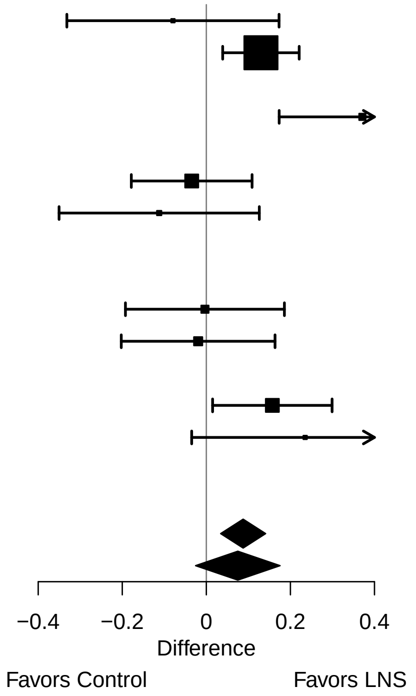


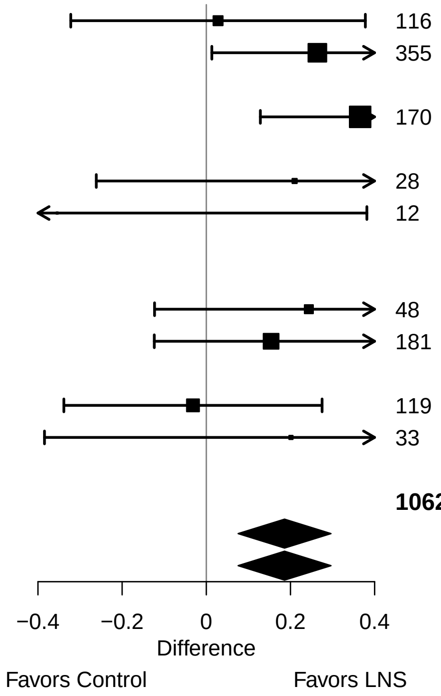

## Supplemental figure 7A: Mean difference in language z-score

## 7A9: Stratified by Child baseline acute malnutrition

**P−for−interaction = 0.034**
**Difference in MDs = 0.16 (0.01, 0.31)**

| | | | No | | | | | | Yes | | | | | |
|---|---|---|---|---|---|---|---|---|---|---|---|---|---|---|
| **Country** | **Trial** | **Tool** | **LNS N** | **Control N** | **Control Mean** | **MD (95% CI)** | **Fixed W** | **Random W** | **LNS N** | **Control N** | **Control Mean** | **MD (95% CI)** | **Fixed W** | **Random W** |
| Bangladesh | JiVitA−4 (21) | BSID−III | 359 | 112 | 0.14 | −0.14 (−0.39, 0.12) | 0.04 | 0.08 | 84 | 31 | −0.21 | 0.12 (−0.16, 0.40) | 0.23 | 0.23 |
| Bangladesh | RDNS (22) | CDI | 1477 | 706 | −0.08 | 0.16 (0.08, 0.25) | 0.35 | 0.17 | 124 | 53 | −0.56 | 0.26 (−0.24, 0.75) | 0.08 | 0.08 |
| Bangladesh | WASH−B (23) | CDI | | | | | | | | | | | | |
| Burkina Faso | iLiNS−Zinc (24) | DMC | 556 | 266 | −0.14 | 0.34 (0.17, 0.52) | 0.09 | 0.12 | 190 | 109 | −0.49 | 0.40 (0.18, 0.61) | 0.40 | 0.40 |
| Ghana | GHANA (25) | | | | | | | | | | | | | |
| Ghana | iLiNS−DYAD−G (26) | CDI | 289 | 555 | 0.01 | −0.03 (−0.17, 0.12) | 0.13 | 0.14 | 19 | 56 | 0.00 | 0.28 (−0.18, 0.75) | 0.09 | 0.09 |
| Haiti | HAITI (27) | Total sounds | | | | | | | | | | | | |
| Kenya | WASH−B (28) | EASQ | | | | | | | | | | | | |
| Madagascar | MAHAY (29) | ASQI | | | | | | | | | | | | |
| Malawi | iLiNS−DYAD−M (30) | CDI | 187 | 386 | 0.02 | 0.02 (−0.15, 0.20) | 0.09 | 0.12 | 14 | 37 | −0.45 | 0.33 (−0.26, 0.93) | 0.05 | 0.05 |
| Malawi | iLiNS−DOSE (31) | CDI | 608 | 206 | −0.02 | 0.04 (−0.12, 0.20) | 0.11 | 0.13 | 37 | 15 | −0.21 | 0.15 (−0.48, 0.78) | 0.05 | 0.05 |
| Mali | PROMIS CS (32) | DMC | | | | | | | | | | | | |
| Zimbabwe | SHINE (HIV−) (33) | CDI | 608 | 553 | −0.03 | 0.10 (−0.03, 0.23) | 0.15 | 0.15 | 36 | 34 | −0.43 | 0.44 (−0.03, 0.91) | 0.09 | 0.09 |
| Zimbabwe | SHINE (HIV+) (34) | CDI | 135 | 113 | −0.05 | 0.22 (−0.03, 0.47) | 0.04 | 0.09 | 12 | 7 | −0.65 | 0.64 (−0.29, 1.58) | 0.02 | 0.02 |
| | | | **4219** | **2897** | | **I² = 0.61, Tau² = 0.01** | | | **516** | **342** | | **I² = 0.00, Tau² = 0.00** | | |
| **Fixed** | | | | | | **0.11 (0.06, 0.16)** | | | | | | **0.31 (0.17, 0.44)** | | |
| **Random** | | | | | | **0.10 (0.00, 0.19)** | | | | | | **0.31 (0.17, 0.44)** | | |

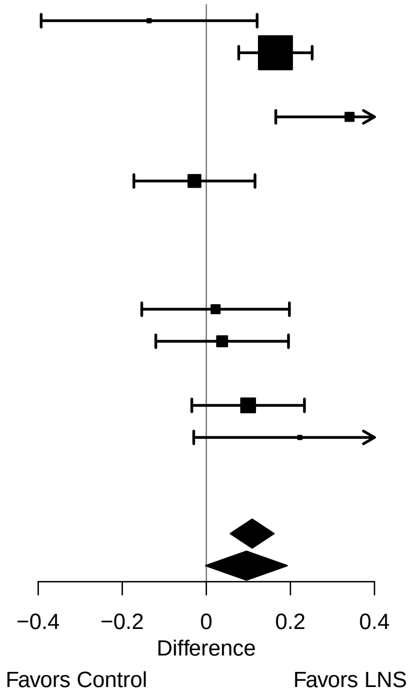


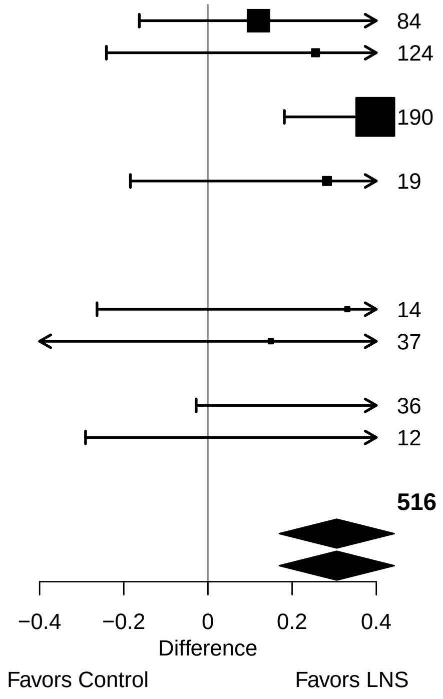

## Supplemental figure 7A: Mean difference in language z-score

## 7A10: Stratified by Child baseline anemia

**P−for−interaction = 0.650**
**Difference in MDs = −0.04 (−0.19, 0.12)**

| Country | Trial | Tool | Not anemic: LNS N | Not anemic: Control N | Not anemic: Control Mean | Not anemic: MD (95% CI) | Not anemic: Fixed W | Not anemic: Random W | Anemic: LNS N | Anemic: Control N | Anemic: Control Mean | Anemic: MD (95% CI) | Anemic: Fixed W | Anemic: Random W |
|---|---|---|---|---|---|---|---|---|---|---|---|---|---|---|
| Bangladesh | JiVitA−4 (21) | BSID−III | | | | | | | | | | | | |
| Bangladesh | RDNS (22) | CDI | 212 | 96 | −0.24 | 0.28 (−0.02, 0.58) | 0.14 | 0.18 | 318 | 160 | −0.21 | 0.30 (0.08, 0.51) | 0.17 | 0.19 |
| Bangladesh | WASH−B (23) | CDI | | | | | | | | | | | | |
| Burkina Faso | iLiNS−Zinc (24) | DMC | 59 | 34 | −0.35 | 0.48 (0.08, 0.87) | 0.08 | 0.14 | 687 | 341 | −0.23 | 0.36 (0.19, 0.52) | 0.30 | 0.23 |
| Ghana | GHANA (25) | | | | | | | | | | | | | |
| Ghana | iLiNS−DYAD−G (26) | CDI | 192 | 351 | 0.05 | −0.10 (−0.27, 0.07) | 0.41 | 0.27 | 90 | 204 | −0.02 | 0.07 (−0.19, 0.32) | 0.12 | 0.17 |
| Haiti | HAITI (27) | Total sounds | | | | | | | | | | | | |
| Kenya | WASH−B (28) | EASQ | | | | | | | | | | | | |
| Madagascar | MAHAY (29) | ASQI | | | | | | | | | | | | |
| Malawi | iLiNS−DYAD−M (30) | CDI | 66 | 147 | −0.06 | 0.20 (−0.12, 0.51) | 0.13 | 0.18 | 138 | 275 | 0.02 | −0.03 (−0.23, 0.16) | 0.21 | 0.21 |
| Malawi | iLiNS−DOSE (31) | CDI | 224 | 94 | −0.01 | 0.10 (−0.12, 0.33) | 0.24 | 0.23 | 417 | 126 | −0.06 | 0.03 (−0.18, 0.24) | 0.19 | 0.20 |
| Mali | PROMIS CS (32) | DMC | | | | | | | | | | | | |
| Zimbabwe | SHINE (HIV−) (33) | CDI | | | | | | | | | | | | |
| Zimbabwe | SHINE (HIV+) (34) | CDI | | | | | | | | | | | | |
| | | | **753** | **722** | | **I² = 0.61, Tau² = 0.02** | | | **1650** | **1106** | | **I² = 0.69, Tau² = 0.02** | | |
| **Fixed** | | | | | | **0.08 (−0.03, 0.20)** | | | | | | **0.17 (0.08, 0.26)** | | |
| **Random** | | | | | | **0.15 (−0.04, 0.33)** | | | | | | **0.15 (−0.01, 0.30)** | | |

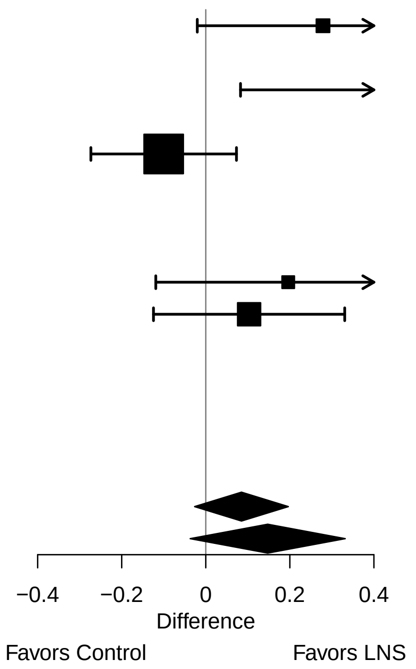


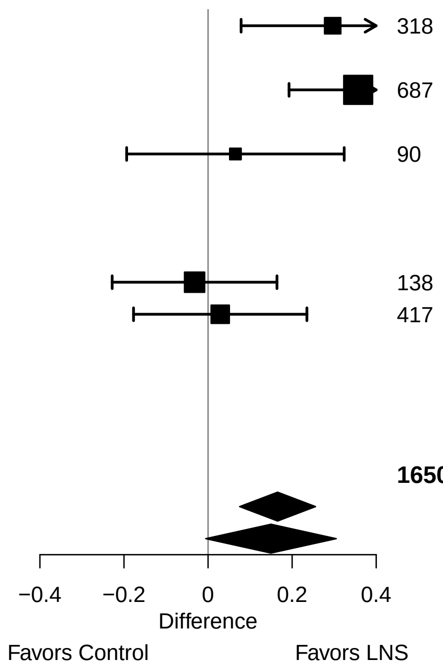

# Supplemental figure 7B: Language lowest decile prevalence ratio

## 7B1: Stratified by Maternal height

**P−for−interaction = 0.678**
**Ratio of PRs = 1.06 (0.81, 1.39)**

| | | | At least 150.1 cm | | | | | | Less than 150.1 cm | | | | | |
|---|---|---|---|---|---|---|---|---|---|---|---|---|---|---|
| **Country** | **Trial** | **Tool** | **LNS N** | **Control N** | **Control Prevalence** | **PR (95% CI)** | **Fixed W** | **Random W** | **LNS N** | **Control N** | **Control Prevalence** | **PR (95% CI)** | **Fixed W** | **Random W** |
| Bangladesh | JiVitA−4 (21) | BSID−III | | | | | | | | | | | | |
| Bangladesh | RDNS (22) | CDI | 868 | 431 | 12.5 | 0.58 (0.40, 0.84) | 0.16 | 0.23 | 735 | 358 | 12.0 | 0.93 (0.65, 1.32) | 0.36 | 0.36 |
| Bangladesh | WASH−B (23) | CDI | 589 | 1812 | 10.2 | 0.82 (0.62, 1.08) | 0.27 | 0.28 | 515 | 1499 | 10.8 | 0.77 (0.56, 1.07) | 0.42 | 0.42 |
| Burkina Faso | iLiNS−Zinc (24) | DMC | | | | | | | | | | | | |
| Ghana | GHANA (25) | | | | | | | | | | | | | |
| Ghana | iLiNS−DYAD−G (26) | CDI | | | | | | | | | | | | |
| Haiti | HAITI (27) | Total sounds | | | | | | | | | | | | |
| Kenya | WASH−B (28) | EASQ | 1237 | 4372 | 9.7 | 0.96 (0.78, 1.18) | 0.50 | 0.33 | 51 | 175 | 15.4 | 0.64 (0.26, 1.58) | 0.05 | 0.05 |
| Madagascar | MAHAY (29) | ASQI | 956 | 986 | 8.7 | 1.18 (0.69, 2.00) | 0.07 | 0.16 | 553 | 538 | 9.9 | 1.14 (0.68, 1.91) | 0.17 | 0.17 |
| Malawi | iLiNS−DYAD−M (30) | CDI | | | | | | | | | | | | |
| Malawi | iLiNS−DOSE (31) | CDI | | | | | | | | | | | | |
| Mali | PROMIS CS (32) | DMC | | | | | | | | | | | | |
| Zimbabwe | SHINE (HIV−) (33) | CDI | | | | | | | | | | | | |
| Zimbabwe | SHINE (HIV+) (34) | CDI | | | | | | | | | | | | |
| | | | **3650** | **7601** | | **I² = 0.57, Tau² = 0.04** | | | **1854** | **2570** | | **I² = 0.00, Tau² = 0.00** | | |
| **Fixed** | | | | | | **0.86 (0.74, 1.00)** | | | | | | **0.87 (0.70, 1.08)** | | |
| **Random** | | | | | | **0.84 (0.65, 1.10)** | | | | | | **0.87 (0.70, 1.08)** | | |

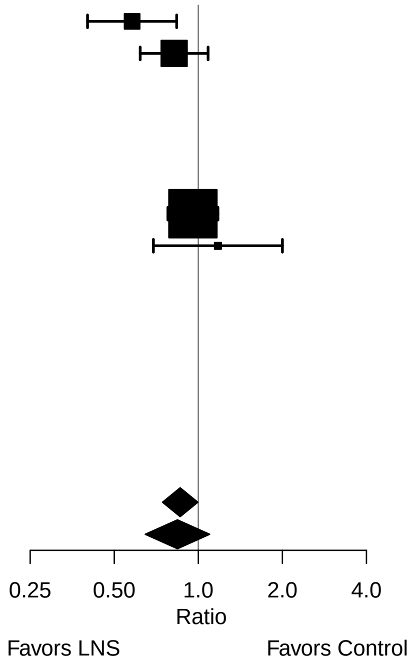


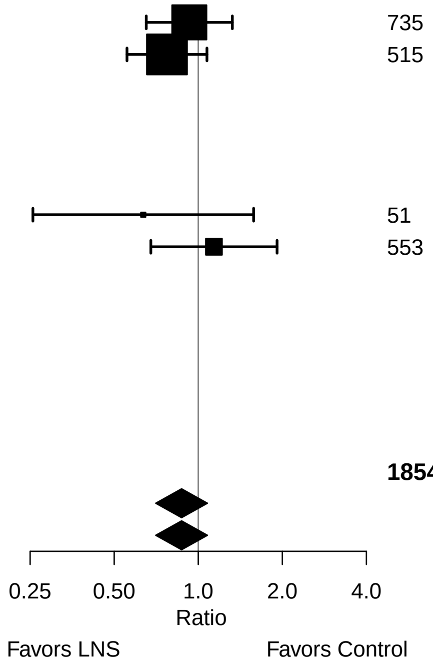

## Supplemental figure 7B: Language lowest decile prevalence ratio

## 7B2: Stratified by Maternal BMI

**P−for−interaction = 0.149**
**Ratio of PRs = 1.18 (0.94, 1.47)**

| | | | At least 20 kg/m² | | | | | | Less than 20 kg/m² | | | | | |
|---|---|---|---|---|---|---|---|---|---|---|---|---|---|---|
| **Country** | **Trial** | **Tool** | **LNS N** | **Control N** | **Control Prevalence** | **PR (95% CI)** | **Fixed W** | **Random W** | **LNS N** | **Control N** | **Control Prevalence** | **PR (95% CI)** | **Fixed W** | **Random W** |
| Bangladesh | JiVitA−4 (21) | BSID−III | | | | | | | | | | | | |
| Bangladesh | RDNS (22) | CDI | 730 | 334 | 12.0 | 0.54 (0.36, 0.80) | 0.12 | 0.15 | 873 | 455 | 12.5 | 0.90 (0.66, 1.21) | 0.27 | 0.20 |
| Bangladesh | WASH−B (23) | CDI | 498 | 1529 | 9.5 | 0.63 (0.44, 0.91) | 0.14 | 0.16 | 606 | 1781 | 11.2 | 0.91 (0.70, 1.18) | 0.36 | 0.21 |
| Burkina Faso | iLiNS−Zinc (24) | DMC | 480 | 225 | 12.4 | 0.57 (0.36, 0.91) | 0.09 | 0.12 | 264 | 150 | 20.7 | 0.37 (0.21, 0.63) | 0.09 | 0.15 |
| Ghana | GHANA (25) | | | | | | | | | | | | | |
| Ghana | iLiNS−DYAD−G (26) | CDI | | | | | | | | | | | | |
| Haiti | HAITI (27) | Total sounds | | | | | | | | | | | | |
| Kenya | WASH−B (28) | EASQ | 1031 | 3535 | 10.0 | 0.94 (0.75, 1.18) | 0.37 | 0.24 | 257 | 1012 | 9.8 | 0.91 (0.61, 1.38) | 0.15 | 0.18 |
| Madagascar | MAHAY (29) | ASQI | | | | | | | | | | | | |
| Malawi | iLiNS−DYAD−M (30) | CDI | | | | | | | | | | | | |
| Malawi | iLiNS−DOSE (31) | CDI | | | | | | | | | | | | |
| Mali | PROMIS CS (32) | DMC | 683 | 664 | 11.9 | 0.66 (0.45, 0.98) | 0.12 | 0.15 | 238 | 274 | 9.9 | 1.07 (0.67, 1.70) | 0.11 | 0.17 |
| Zimbabwe | SHINE (HIV−) (33) | CDI | 581 | 569 | 10.9 | 0.93 (0.67, 1.31) | 0.16 | 0.18 | 96 | 93 | 15.1 | 0.42 (0.17, 1.00) | 0.03 | 0.09 |
| Zimbabwe | SHINE (HIV+) (34) | CDI | | | | | | | | | | | | |
| | | | **4003** | **6856** | | **I² = 0.54, Tau² = 0.03** | | | **2334** | **3765** | | **I² = 0.62, Tau² = 0.13** | | |
| **Fixed** | | | | | | **0.76 (0.67, 0.87)** | | | | | | **0.83 (0.71, 0.98)** | | |
| **Random** | | | | | | **0.72 (0.59, 0.88)** | | | | | | **0.76 (0.54, 1.06)** | | |

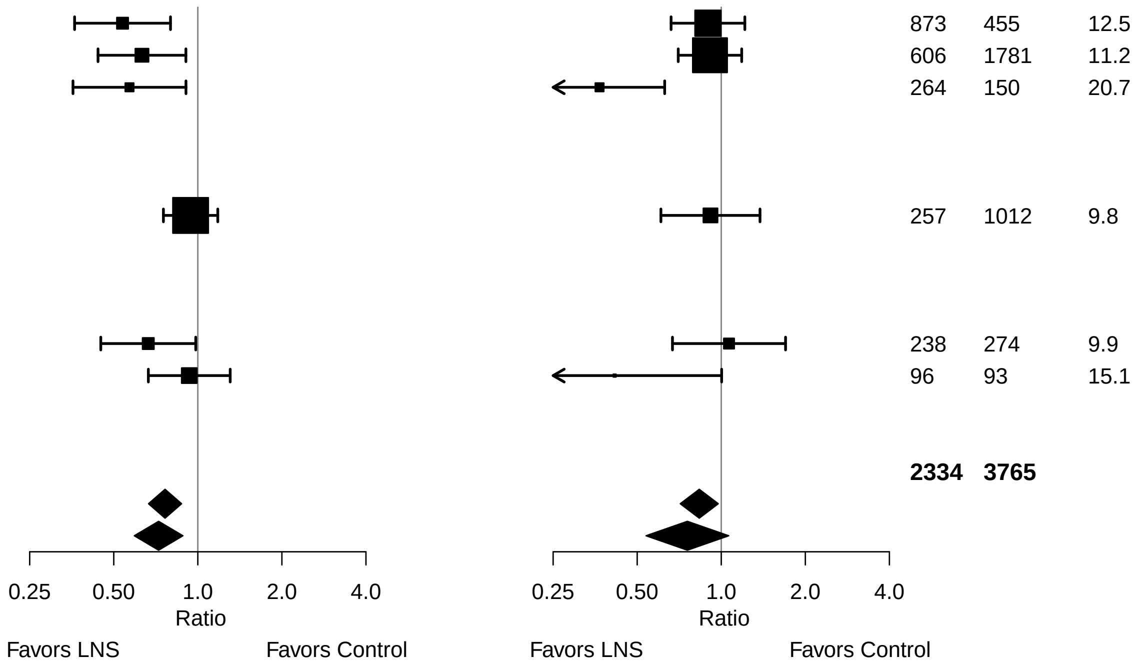

## Supplemental figure 7B: Language lowest decile prevalence ratio

## 7B3: Stratified by Maternal age

**P−for−interaction = 0.832**
**Ratio of PRs = 1.02 (0.86, 1.20)**

| Country | Trial | Tool | At least 25 y: LNS N | Control N | Control Prevalence | PR (95% CI) | Fixed W | Random W | Less than 25 y: LNS N | Control N | Control Prevalence | PR (95% CI) | Fixed W | Random W |
|---|---|---|---|---|---|---|---|---|---|---|---|---|---|---|
| Bangladesh | JiVitA−4 (21) | BSID−III | 200 | 68 | 11.8 | 0.89 (0.48, 1.66) | 0.04 | 0.04 | 242 | 74 | 10.8 | 0.84 (0.38, 1.85) | 0.03 | 0.05 |
| Bangladesh | RDNS (22) | CDI | 453 | 220 | 13.6 | 0.83 (0.57, 1.20) | 0.12 | 0.12 | 1210 | 594 | 12.0 | 0.67 (0.51, 0.88) | 0.21 | 0.13 |
| Bangladesh | WASH−B (23) | CDI | 477 | 1475 | 11.5 | 0.90 (0.66, 1.22) | 0.18 | 0.18 | 627 | 1863 | 9.8 | 0.73 (0.54, 0.99) | 0.17 | 0.13 |
| Burkina Faso | iLiNS−Zinc (24) | DMC | 421 | 232 | 15.1 | 0.52 (0.33, 0.83) | 0.08 | 0.08 | 321 | 141 | 17.0 | 0.38 (0.22, 0.67) | 0.05 | 0.08 |
| Ghana | GHANA (25) | | | | | | | | | | | | | |
| Ghana | iLiNS−DYAD−G (26) | CDI | 211 | 405 | 10.4 | 0.82 (0.49, 1.39) | 0.06 | 0.06 | 120 | 253 | 8.7 | 1.63 (0.90, 2.95) | 0.05 | 0.07 |
| Haiti | HAITI (27) | Total sounds | | | | | | | | | | | | |
| Kenya | WASH−B (28) | EASQ | 798 | 2622 | 9.9 | 1.01 (0.76, 1.35) | 0.19 | 0.19 | 549 | 2092 | 10.3 | 0.89 (0.66, 1.19) | 0.18 | 0.13 |
| Madagascar | MAHAY (29) | ASQI | 852 | 901 | 9.8 | 1.14 (0.72, 1.82) | 0.08 | 0.08 | 759 | 703 | 8.5 | 1.20 (0.72, 2.01) | 0.06 | 0.09 |
| Malawi | iLiNS−DYAD−M (30) | CDI | 103 | 225 | 14.7 | 0.66 (0.34, 1.29) | 0.04 | 0.04 | 112 | 214 | 6.5 | 1.36 (0.63, 2.97) | 0.03 | 0.05 |
| Malawi | iLiNS−DOSE (31) | CDI | 345 | 124 | 11.3 | 0.92 (0.52, 1.65) | 0.05 | 0.05 | 288 | 90 | 8.9 | 1.02 (0.48, 2.16) | 0.03 | 0.05 |
| Mali | PROMIS CS (32) | DMC | 492 | 492 | 11.0 | 0.65 (0.42, 1.00) | 0.09 | 0.09 | 435 | 452 | 11.7 | 0.90 (0.61, 1.34) | 0.10 | 0.11 |
| Zimbabwe | SHINE (HIV−) (33) | CDI | 414 | 384 | 9.9 | 0.76 (0.48, 1.19) | 0.08 | 0.08 | 315 | 310 | 12.9 | 0.96 (0.63, 1.47) | 0.09 | 0.10 |
| Zimbabwe | SHINE (HIV+) (34) | CDI | | | | | | | | | | | | |
| | | | **4766** | **7148** | | **I² = 0.00, Tau² = 0.00** | | | **4978** | **6786** | | **I² = 0.50, Tau² = 0.07** | | |
| **Fixed** | | | | | | **0.84 (0.74, 0.95)** | | | | | | **0.83 (0.73, 0.94)** | | |
| **Random** | | | | | | **0.84 (0.74, 0.95)** | | | | | | **0.87 (0.70, 1.07)** | | |

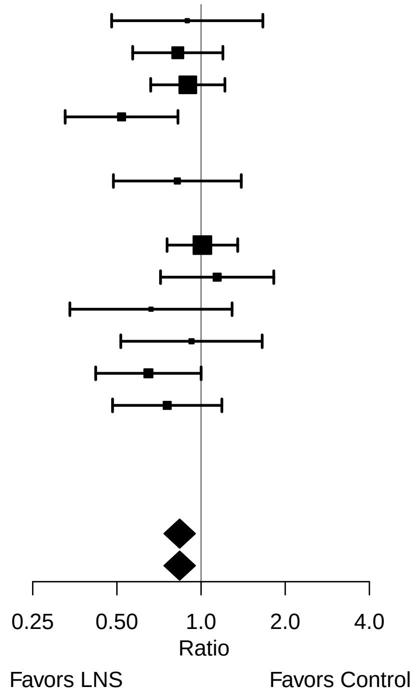


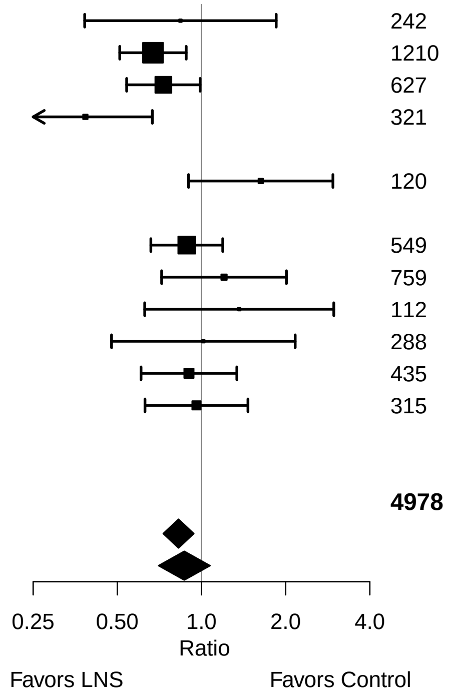

## Supplemental figure 7B: Language lowest decile prevalence ratio

### 7B4: Stratified by Maternal education

**P−for−interaction = 0.804**
**Ratio of PRs = 0.97 (0.79, 1.20)**

| Country | Trial | Tool | LNS N | Control N | Control Prevalence | Primary or greater PR (95% CI) | Fixed W | Random W | LNS N | Control N | Control Prevalence | Incomplete or no formal PR (95% CI) | Fixed W | Random W |
|---|---|---|---|---|---|---|---|---|---|---|---|---|---|---|
| Bangladesh | JiVitA−4 (21) | BSID−III | 292 | 92 | 8.7 | 0.87 (0.38, 1.98) | 0.03 | 0.08 | 152 | 51 | 15.7 | 0.88 (0.43, 1.81) | 0.04 | 0.04 |
| Bangladesh | RDNS (22) | CDI | 1245 | 588 | 9.9 | 0.67 (0.49, 0.90) | 0.23 | 0.20 | 418 | 226 | 19.0 | 0.83 (0.59, 1.17) | 0.16 | 0.16 |
| Bangladesh | WASH−B (23) | CDI | 781 | 2406 | 8.9 | 0.87 (0.69, 1.09) | 0.40 | 0.22 | 328 | 947 | 14.8 | 0.70 (0.49, 1.00) | 0.15 | 0.15 |
| Burkina Faso | iLiNS−Zinc (24) | DMC | | | | | | | | | | | | |
| Ghana | GHANA (25) | | | | | | | | | | | | | |
| Ghana | iLiNS−DYAD−G (26) | CDI | 256 | 519 | 9.4 | 0.99 (0.62, 1.58) | 0.10 | 0.16 | 75 | 139 | 10.8 | 1.36 (0.66, 2.81) | 0.04 | 0.04 |
| Haiti | HAITI (27) | Total sounds | | | | | | | | | | | | |
| Kenya | WASH−B (28) | EASQ | 658 | 2258 | 6.9 | 0.84 (0.60, 1.16) | 0.20 | 0.20 | 702 | 2484 | 13.0 | 1.02 (0.81, 1.28) | 0.37 | 0.37 |
| Madagascar | MAHAY (29) | ASQI | 339 | 416 | 3.6 | 2.62 (1.24, 5.52) | 0.04 | 0.10 | 1274 | 1188 | 11.2 | 1.00 (0.63, 1.57) | 0.09 | 0.09 |
| Malawi | iLiNS−DYAD−M (30) | CDI | | | | | | | | | | | | |
| Malawi | iLiNS−DOSE (31) | CDI | | | | | | | | | | | | |
| Mali | PROMIS CS (32) | DMC | 100 | 94 | 7.4 | 1.21 (0.35, 4.19) | 0.01 | 0.05 | 826 | 850 | 11.8 | 0.74 (0.52, 1.05) | 0.16 | 0.16 |
| Zimbabwe | SHINE (HIV−) (33) | CDI | | | | | | | | | | | | |
| Zimbabwe | SHINE (HIV+) (34) | CDI | | | | | | | | | | | | |
| | | | **3671** | **6373** | | **I² = 0.50, Tau² = 0.08** | | | **3775** | **5885** | | **I² = 0.00, Tau² = 0.00** | | |
| **Fixed** | | | | | | **0.86 (0.74, 0.99)** | | | | | | **0.89 (0.77, 1.02)** | | |
| **Random** | | | | | | **0.94 (0.70, 1.26)** | | | | | | **0.89 (0.77, 1.02)** | | |

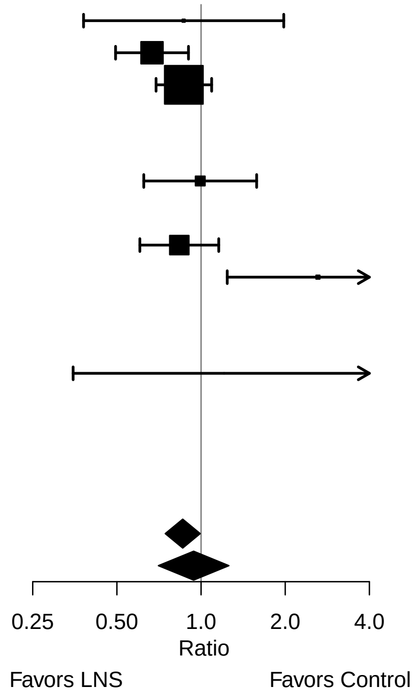


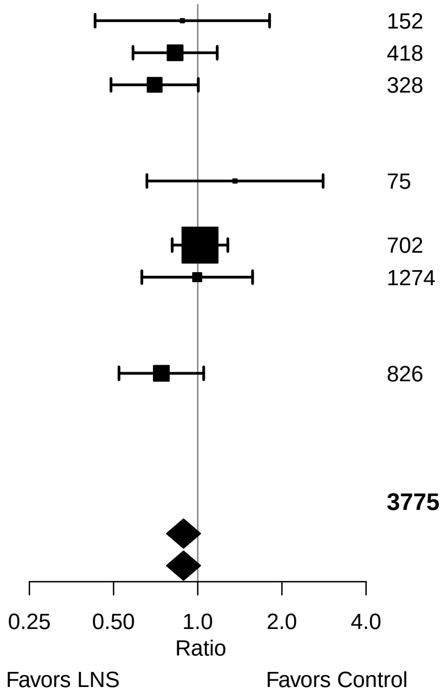

## Supplemental figure 7B: Language lowest decile prevalence ratio

### 7B5: Stratified by Maternal depressive symptoms

**P−for−interaction = 0.204**
**Ratio of PRs = 0.86 (0.69, 1.08)**

| Country | Trial | Tool | LNS N | Control N | Control Prevalence | Less than 75th percentile PR (95% CI) | Fixed W | Random W | LNS N | Control N | Control Prevalence | At least 75th percentile PR (95% CI) | Fixed W | Random W |
|---|---|---|---|---|---|---|---|---|---|---|---|---|---|---|
| Bangladesh | JiVitA−4 (21) | BSID−III | | | | | | | | | | | | |
| Bangladesh | RDNS (22) | CDI | 1081 | 471 | 11.9 | 0.65 (0.46, 0.92) | 0.14 | 0.15 | 520 | 288 | 13.5 | 0.87 (0.62, 1.21) | 0.28 | 0.28 |
| Bangladesh | WASH−B (23) | CDI | 856 | 2410 | 10.2 | 0.68 (0.52, 0.90) | 0.23 | 0.22 | 232 | 859 | 11.3 | 1.18 (0.80, 1.76) | 0.20 | 0.20 |
| Burkina Faso | iLiNS−Zinc (24) | DMC | | | | | | | | | | | | |
| Ghana | GHANA (25) | | | | | | | | | | | | | |
| Ghana | iLiNS−DYAD−G (26) | CDI | 241 | 435 | 10.8 | 1.00 (0.64, 1.57) | 0.08 | 0.10 | 78 | 202 | 7.4 | 1.38 (0.61, 3.13) | 0.05 | 0.05 |
| Haiti | HAITI (27) | Total sounds | | | | | | | | | | | | |
| Kenya | WASH−B (28) | EASQ | 960 | 3317 | 9.5 | 0.90 (0.72, 1.12) | 0.34 | 0.29 | 317 | 1156 | 8.7 | 1.06 (0.74, 1.52) | 0.25 | 0.25 |
| Madagascar | MAHAY (29) | ASQI | 615 | 667 | 8.8 | 1.29 (0.70, 2.35) | 0.05 | 0.06 | 268 | 242 | 12.0 | 0.72 (0.37, 1.40) | 0.07 | 0.07 |
| Malawi | iLiNS−DYAD−M (30) | CDI | 153 | 302 | 9.9 | 0.92 (0.50, 1.68) | 0.05 | 0.06 | 47 | 112 | 12.5 | 0.85 (0.33, 2.23) | 0.03 | 0.03 |
| Malawi | iLiNS−DOSE (31) | CDI | | | | | | | | | | | | |
| Mali | PROMIS CS (32) | DMC | | | | | | | | | | | | |
| Zimbabwe | SHINE (HIV−) (33) | CDI | 566 | 552 | 10.3 | 0.89 (0.60, 1.31) | 0.11 | 0.13 | 190 | 173 | 14.5 | 0.69 (0.41, 1.17) | 0.12 | 0.12 |
| Zimbabwe | SHINE (HIV+) (34) | CDI | | | | | | | | | | | | |
| | | | **4472** | **8154** | | **I² = 0.15, Tau² = 0.01** | | | **1652** | **3032** | | **I² = 0.00, Tau² = 0.00** | | |
| **Fixed** | | | | | | **0.83 (0.73, 0.94)** | | | | | | **0.95 (0.80, 1.14)** | | |
| **Random** | | | | | | **0.83 (0.72, 0.96)** | | | | | | **0.95 (0.80, 1.14)** | | |

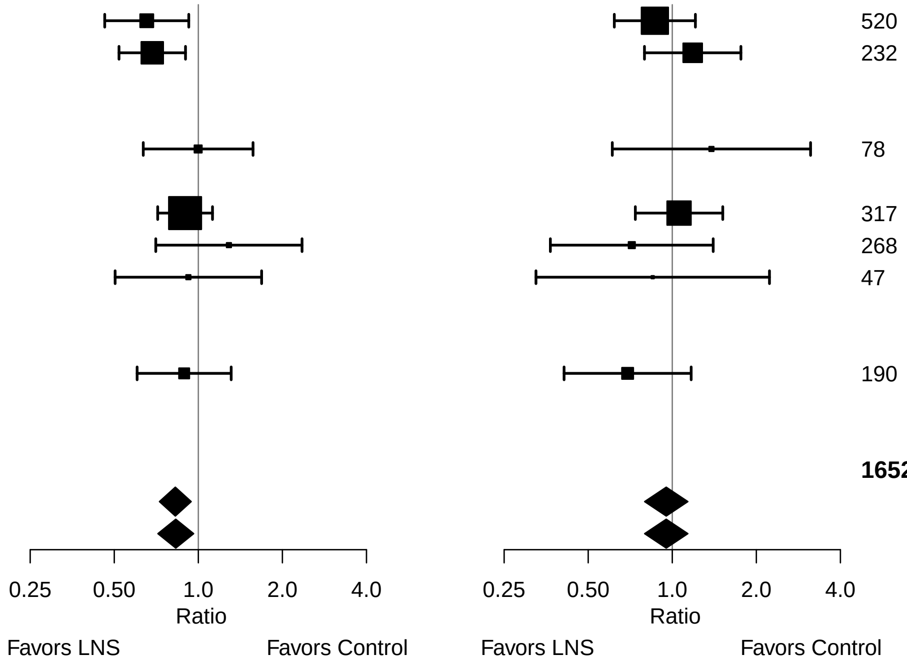

## Supplemental figure 7B: Language lowest decile prevalence ratio

## 7B6: Stratified by Child sex

**P−for−interaction = 0.255**
**Ratio of PRs = 0.91 (0.77, 1.07)**

| Country | Trial | Tool | LNS N | Control N | Control Prevalence | Male PR (95% CI) | Fixed W | Random W | LNS N | Control N | Control Prevalence | Female PR (95% CI) | Fixed W | Random W |
|---|---|---|---|---|---|---|---|---|---|---|---|---|---|---|
| Bangladesh | JiVitA−4 (21) | BSID−III | 231 | 70 | 14.3 | 0.64 (0.30, 1.34) | 0.03 | 0.04 | 214 | 73 | 8.2 | 1.25 (0.65, 2.42) | 0.04 | 0.05 |
| Bangladesh | RDNS (22) | CDI | 833 | 411 | 10.5 | 1.01 (0.72, 1.42) | 0.13 | 0.12 | 830 | 403 | 14.4 | 0.50 (0.35, 0.73) | 0.12 | 0.11 |
| Bangladesh | WASH−B (23) | CDI | 545 | 1685 | 11.5 | 0.80 (0.60, 1.06) | 0.18 | 0.15 | 564 | 1668 | 9.6 | 0.81 (0.59, 1.12) | 0.17 | 0.13 |
| Burkina Faso | iLiNS−Zinc (24) | DMC | 372 | 193 | 12.4 | 0.48 (0.30, 0.76) | 0.07 | 0.08 | 374 | 182 | 19.2 | 0.44 (0.30, 0.67) | 0.11 | 0.10 |
| Ghana | GHANA (25) | | | | | | | | | | | | | |
| Ghana | iLiNS−DYAD−G (26) | CDI | 164 | 306 | 8.8 | 1.45 (0.85, 2.48) | 0.05 | 0.06 | 167 | 352 | 10.5 | 0.80 (0.44, 1.43) | 0.05 | 0.07 |
| Haiti | HAITI (27) | Total sounds | | | | | | | | | | | | |
| Kenya | WASH−B (28) | EASQ | 682 | 2260 | 10.7 | 1.03 (0.80, 1.32) | 0.23 | 0.17 | 680 | 2485 | 9.6 | 0.86 (0.62, 1.17) | 0.17 | 0.13 |
| Madagascar | MAHAY (29) | ASQI | 795 | 787 | 8.9 | 1.13 (0.70, 1.84) | 0.06 | 0.07 | 818 | 817 | 9.5 | 1.20 (0.71, 2.04) | 0.06 | 0.08 |
| Malawi | iLiNS−DYAD−M (30) | CDI | 105 | 205 | 8.8 | 0.76 (0.33, 1.76) | 0.02 | 0.03 | 110 | 234 | 12.4 | 0.95 (0.52, 1.76) | 0.05 | 0.06 |
| Malawi | iLiNS−DOSE (31) | CDI | 320 | 117 | 11.1 | 0.87 (0.47, 1.61) | 0.04 | 0.05 | 325 | 104 | 9.6 | 1.06 (0.54, 2.07) | 0.04 | 0.05 |
| Mali | PROMIS CS (32) | DMC | 469 | 514 | 10.7 | 0.76 (0.51, 1.12) | 0.09 | 0.10 | 458 | 430 | 12.1 | 0.78 (0.52, 1.16) | 0.11 | 0.11 |
| Zimbabwe | SHINE (HIV−) (33) | CDI | 412 | 378 | 11.6 | 0.92 (0.62, 1.37) | 0.09 | 0.10 | 405 | 403 | 9.9 | 0.87 (0.52, 1.46) | 0.06 | 0.08 |
| Zimbabwe | SHINE (HIV+) (34) | CDI | 82 | 76 | 13.2 | 0.74 (0.29, 1.88) | 0.02 | 0.02 | 83 | 71 | 11.3 | 0.64 (0.24, 1.72) | 0.02 | 0.03 |
| | | | **5010** | **7002** | | **$I^2$ = 0.28, $Tau^2$ = 0.02** | | | **5028** | **7222** | | **$I^2$ = 0.43, $Tau^2$ = 0.04** | | |
| **Fixed** | | | | | | **0.89 (0.79, 1.00)** | | | | | | **0.76 (0.67, 0.87)** | | |
| **Random** | | | | | | **0.88 (0.76, 1.02)** | | | | | | **0.78 (0.65, 0.93)** | | |

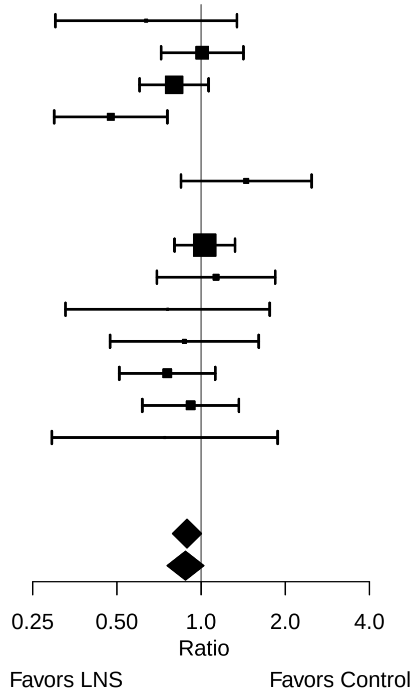


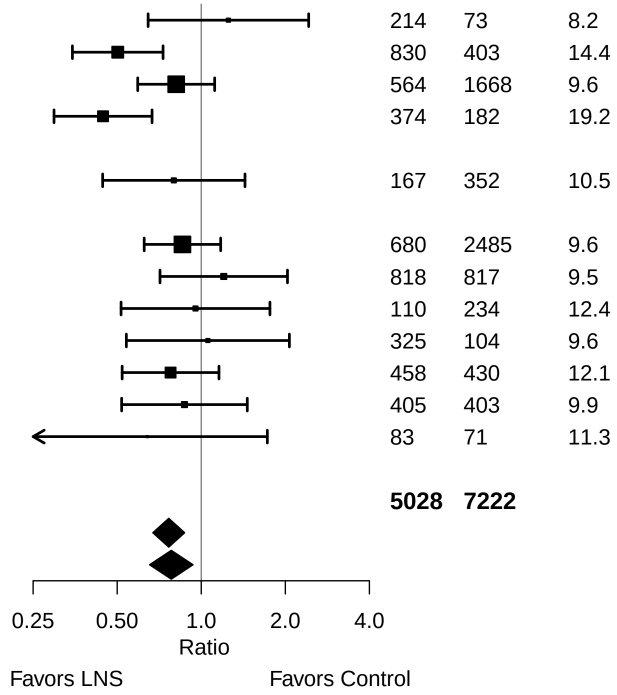

### Supplemental figure 7B: Language lowest decile prevalence ratio

## 7B7: Stratified by Child birth order

**P−for−interaction = 0.362**
**Ratio of PRs = 1.10 (0.90, 1.35)**

| Country | Trial | Tool | LNS N | Control N | Control Prevalence | Later born PR (95% CI) | Fixed W | Random W | LNS N | Control N | Control Prevalence | Firstborn PR (95% CI) | Fixed W | Random W |
|---|---|---|---|---|---|---|---|---|---|---|---|---|---|---|
| Bangladesh | JiVitA−4 (21) | BSID−III | | | | | | | | | | | | |
| Bangladesh | RDNS (22) | CDI | 974 | 507 | 14.0 | 0.70 (0.53, 0.92) | 0.17 | 0.15 | 688 | 307 | 9.8 | 0.79 (0.51, 1.22) | 0.16 | 0.15 |
| Bangladesh | WASH−B (23) | CDI | 693 | 2187 | 11.6 | 0.75 (0.57, 0.99) | 0.18 | 0.15 | 395 | 1060 | 8.3 | 0.91 (0.63, 1.32) | 0.23 | 0.17 |
| Burkina Faso | iLiNS−Zinc (24) | DMC | 587 | 290 | 15.5 | 0.47 (0.31, 0.72) | 0.08 | 0.10 | 159 | 85 | 16.5 | 0.42 (0.20, 0.89) | 0.05 | 0.08 |
| Ghana | GHANA (25) | | | | | | | | | | | | | |
| Ghana | iLiNS−DYAD−G (26) | CDI | 224 | 445 | 10.1 | 0.88 (0.53, 1.46) | 0.05 | 0.08 | 107 | 213 | 8.9 | 1.57 (0.83, 2.97) | 0.08 | 0.10 |
| Haiti | HAITI (27) | Total sounds | | | | | | | | | | | | |
| Kenya | WASH−B (28) | EASQ | 1071 | 3767 | 9.8 | 0.97 (0.77, 1.24) | 0.24 | 0.17 | 289 | 974 | 11.4 | 0.88 (0.57, 1.36) | 0.16 | 0.15 |
| Madagascar | MAHAY (29) | ASQI | 1162 | 1118 | 9.1 | 1.20 (0.75, 1.91) | 0.06 | 0.08 | 433 | 465 | 9.7 | 1.07 (0.61, 1.89) | 0.10 | 0.11 |
| Malawi | iLiNS−DYAD−M (30) | CDI | 170 | 350 | 11.7 | 0.65 (0.36, 1.19) | 0.04 | 0.06 | 45 | 87 | 6.9 | 2.26 (0.81, 6.31) | 0.03 | 0.05 |
| Malawi | iLiNS−DOSE (31) | CDI | | | | | | | | | | | | |
| Mali | PROMIS CS (32) | DMC | 770 | 811 | 11.2 | 0.75 (0.53, 1.07) | 0.11 | 0.12 | 142 | 122 | 9.0 | 1.09 (0.52, 2.31) | 0.05 | 0.08 |
| Zimbabwe | SHINE (HIV−) (33) | CDI | 587 | 562 | 9.4 | 0.94 (0.62, 1.42) | 0.08 | 0.10 | 194 | 185 | 15.7 | 0.79 (0.49, 1.27) | 0.13 | 0.13 |
| Zimbabwe | SHINE (HIV+) (34) | CDI | | | | | | | | | | | | |
| | | | **6238** | **10037** | | **I² = 0.43, Tau² = 0.03** | | | **2452** | **3498** | | **I² = 0.29, Tau² = 0.06** | | |
| **Fixed** | | | | | | **0.80 (0.71, 0.90)** | | | | | | **0.91 (0.77, 1.09)** | | |
| **Random** | | | | | | **0.79 (0.67, 0.93)** | | | | | | **0.93 (0.73, 1.19)** | | |

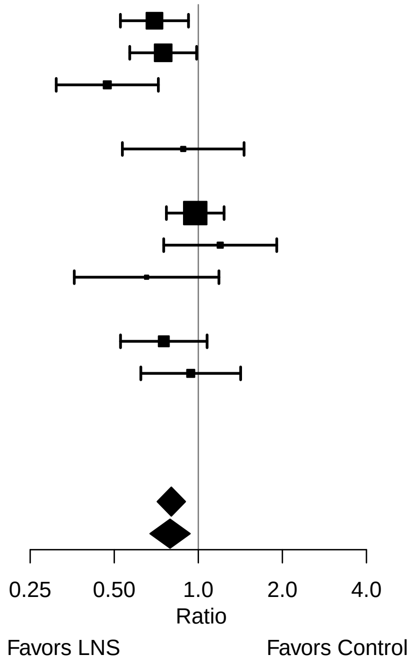


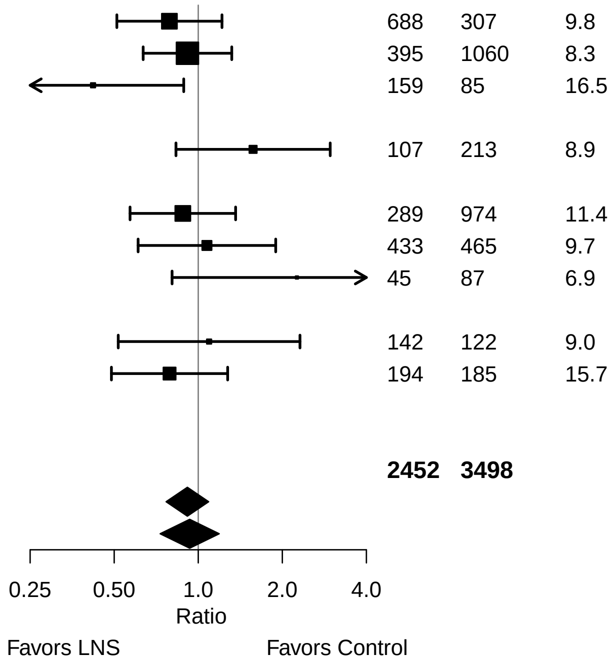

### Supplemental figure 7B: Language lowest decile prevalence ratio

## 7B8: Stratified by Child baseline stunting

**P−for−interaction = 0.111**
**Ratio of PRs = 0.79 (0.59, 1.06)**

| | | | No | | | | | | Yes | | | | | |
|---|---|---|---|---|---|---|---|---|---|---|---|---|---|---|
| **Country** | **Trial** | **Tool** | **LNS N** | **Control N** | **Control Prevalence** | **PR (95% CI)** | **Fixed W** | **Random W** | **LNS N** | **Control N** | **Control Prevalence** | **PR (95% CI)** | **Fixed W** | **Random W** |
| Bangladesh | JiVitA−4 (21) | BSID−III | 327 | 114 | 8.8 | 0.91 (0.42, 1.94) | 0.05 | 0.05 | 116 | 29 | 20.7 | 0.67 (0.35, 1.25) | 0.15 | 0.16 |
| Bangladesh | RDNS (22) | CDI | 1246 | 573 | 10.6 | 0.75 (0.58, 0.99) | 0.41 | 0.39 | 355 | 185 | 18.4 | 0.69 (0.46, 1.03) | 0.37 | 0.30 |
| Bangladesh | WASH−B (23) | CDI | | | | | | | | | | | | |
| Burkina Faso | iLiNS−Zinc (24) | DMC | 576 | 294 | 12.6 | 0.55 (0.38, 0.80) | 0.21 | 0.21 | 170 | 80 | 27.5 | 0.30 (0.16, 0.56) | 0.15 | 0.16 |
| Ghana | GHANA (25) | | | | | | | | | | | | | |
| Ghana | iLiNS−DYAD−G (26) | CDI | | | | | | | | | | | | |
| Haiti | HAITI (27) | Total sounds | | | | | | | | | | | | |
| Kenya | WASH−B (28) | EASQ | | | | | | | | | | | | |
| Madagascar | MAHAY (29) | ASQI | | | | | | | | | | | | |
| Malawi | iLiNS−DYAD−M (30) | CDI | 153 | 323 | 8.4 | 1.02 (0.54, 1.91) | 0.07 | 0.08 | 48 | 97 | 19.6 | 0.64 (0.27, 1.49) | 0.08 | 0.10 |
| Malawi | iLiNS−DOSE (31) | CDI | 464 | 147 | 8.2 | 1.11 (0.60, 2.05) | 0.08 | 0.08 | 181 | 74 | 14.9 | 0.82 (0.42, 1.60) | 0.13 | 0.14 |
| Mali | PROMIS CS (32) | DMC | | | | | | | | | | | | |
| Zimbabwe | SHINE (HIV−) (33) | CDI | 523 | 482 | 10.0 | 0.77 (0.51, 1.16) | 0.18 | 0.18 | 119 | 100 | 16.0 | 0.74 (0.37, 1.47) | 0.12 | 0.14 |
| Zimbabwe | SHINE (HIV+) (34) | CDI | | | | | | | | | | | | |
| | | | **3289** | **1933** | | **I² = 0.05, Tau² = 0.00** | | | **989** | **565** | | **I² = 0.23, Tau² = 0.03** | | |
| **Fixed** | | | | | | **0.75 (0.63, 0.89)** | | | | | | **0.62 (0.49, 0.79)** | | |
| **Random** | | | | | | **0.76 (0.63, 0.90)** | | | | | | **0.61 (0.46, 0.82)** | | |

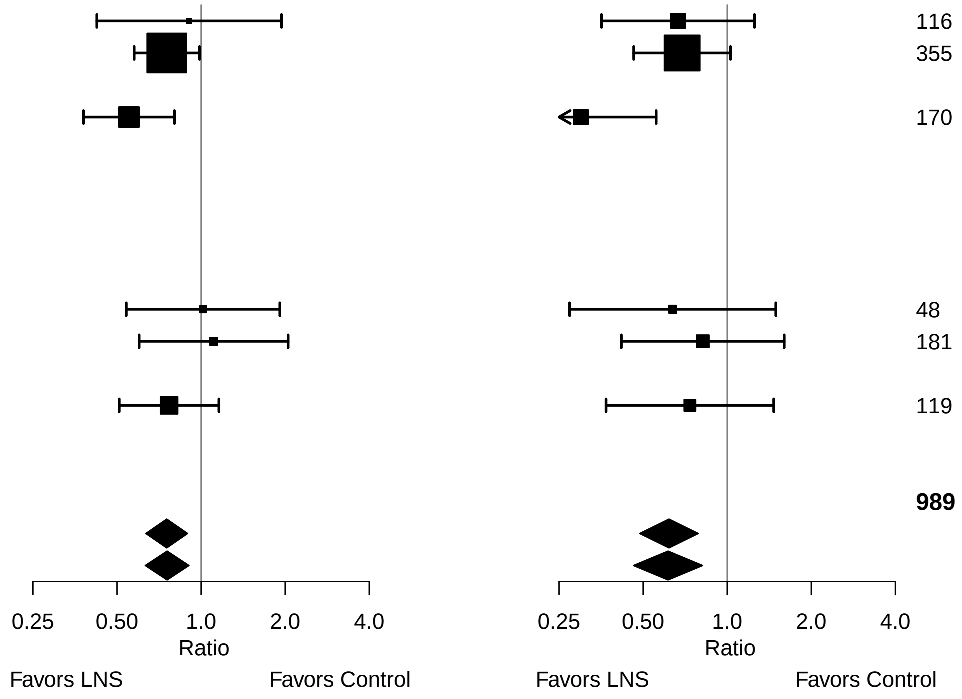

**Supplemental figure 7B: Language lowest decile prevalence ratio**

## 7B9: Stratified by Child baseline acute malnutrition

**P−for−interaction = 0.588**
**Ratio of PRs = 1.13 (0.73, 1.76)**

| Country | Trial | Tool | No: LNS N | No: Control N | No: Control Prevalence | No: PR (95% CI) | No: Fixed W | No: Random W | Yes: LNS N | Yes: Control N | Yes: Control Prevalence | Yes: PR (95% CI) | Yes: Fixed W | Yes: Random W |
|---|---|---|---|---|---|---|---|---|---|---|---|---|---|---|
| Bangladesh | JiVitA−4 (21) | BSID−III | 359 | 112 | 9.8 | 0.88 (0.43, 1.80) | 0.08 | 0.20 | 84 | 31 | 16.1 | 0.81 (0.33, 2.00) | 0.18 | 0.18 |
| Bangladesh | RDNS (22) | CDI | 1477 | 706 | 11.9 | 0.71 (0.56, 0.90) | 0.73 | 0.48 | 124 | 53 | 20.8 | 0.78 (0.42, 1.44) | 0.39 | 0.39 |
| Bangladesh | WASH−B (23) | CDI | | | | | | | | | | | | |
| Burkina Faso | iLiNS−Zinc (24) | DMC | 556 | 266 | 13.5 | 0.43 (0.27, 0.67) | 0.19 | 0.32 | 190 | 109 | 21.1 | 0.55 (0.31, 0.98) | 0.44 | 0.44 |
| Ghana | GHANA (25) | | | | | | | | | | | | | |
| Ghana | iLiNS−DYAD−G (26) | CDI | | | | | | | | | | | | |
| Haiti | HAITI (27) | Total sounds | | | | | | | | | | | | |
| Kenya | WASH−B (28) | EASQ | | | | | | | | | | | | |
| Madagascar | MAHAY (29) | ASQI | | | | | | | | | | | | |
| Malawi | iLiNS−DYAD−M (30) | CDI | | | | | | | | | | | | |
| Malawi | iLiNS−DOSE (31) | CDI | | | | | | | | | | | | |
| Mali | PROMIS CS (32) | DMC | | | | | | | | | | | | |
| Zimbabwe | SHINE (HIV−) (33) | CDI | | | | | | | | | | | | |
| Zimbabwe | SHINE (HIV+) (34) | CDI | | | | | | | | | | | | |
| | | | **2392** | **1084** | | **I² = 0.56, Tau² = 0.07** | | | **398** | **193** | | **I² = 0.00, Tau² = 0.00** | | |
| **Fixed** | | | | | | **0.65 (0.53, 0.80)** | | | | | | **0.67 (0.46, 0.99)** | | |
| **Random** | | | | | | **0.63 (0.43, 0.93)** | | | | | | **0.67 (0.46, 0.99)** | | |

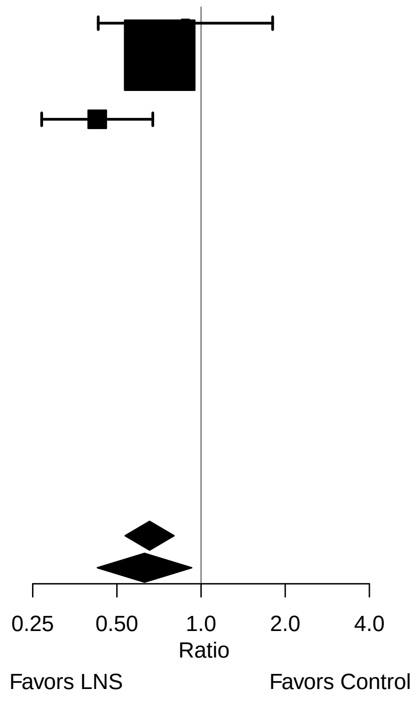


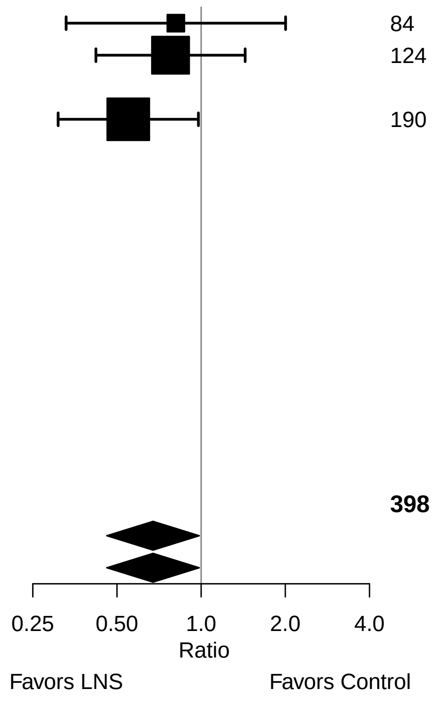

**Supplemental figure 7B: Language lowest decile prevalence ratio**

## 7B10: Stratified by Child baseline anemia

**P−for−interaction = 0.847**
**Ratio of PRs = 1.05 (0.65, 1.68)**

| Country | Trial | Tool | LNS N | Control N | Control Prevalence | Not anemic PR (95% CI) | Fixed W | Random W | LNS N | Control N | Control Prevalence | Anemic PR (95% CI) | Fixed W | Random W |
|---|---|---|---|---|---|---|---|---|---|---|---|---|---|---|
| Bangladesh | JiVitA−4 (21) | BSID−III | | | | | | | | | | | | |
| Bangladesh | RDNS (22) | CDI | 212 | 96 | 14.6 | 0.71 (0.43, 1.17) | 0.39 | 0.35 | 318 | 160 | 12.5 | 0.70 (0.37, 1.33) | 0.25 | 0.25 |
| Bangladesh | WASH−B (23) | CDI | | | | | | | | | | | | |
| Burkina Faso | iLiNS−Zinc (24) | DMC | | | | | | | | | | | | |
| Ghana | GHANA (25) | | | | | | | | | | | | | |
| Ghana | iLiNS−DYAD−G (26) | CDI | 192 | 351 | 7.4 | 1.41 (0.81, 2.45) | 0.32 | 0.31 | 90 | 204 | 10.8 | 0.93 (0.44, 1.93) | 0.19 | 0.19 |
| Haiti | HAITI (27) | Total sounds | | | | | | | | | | | | |
| Kenya | WASH−B (28) | EASQ | | | | | | | | | | | | |
| Madagascar | MAHAY (29) | ASQI | | | | | | | | | | | | |
| Malawi | iLiNS−DYAD−M (30) | CDI | 66 | 147 | 14.3 | 0.53 (0.21, 1.35) | 0.11 | 0.15 | 138 | 275 | 9.1 | 1.04 (0.55, 1.96) | 0.25 | 0.25 |
| Malawi | iLiNS−DOSE (31) | CDI | 224 | 94 | 9.6 | 0.84 (0.39, 1.80) | 0.17 | 0.20 | 417 | 126 | 11.1 | 0.99 (0.56, 1.75) | 0.32 | 0.32 |
| Mali | PROMIS CS (32) | DMC | | | | | | | | | | | | |
| Zimbabwe | SHINE (HIV−) (33) | CDI | | | | | | | | | | | | |
| Zimbabwe | SHINE (HIV+) (34) | CDI | | | | | | | | | | | | |
| | | | **694** | **688** | | **I² = 0.34, Tau² = 0.05** | | | **963** | **765** | | **I² = 0.00, Tau² = 0.00** | | |
| **Fixed** | | | | | | **0.88 (0.64, 1.21)** | | | | | | **0.91 (0.66, 1.25)** | | |
| **Random** | | | | | | **0.87 (0.59, 1.29)** | | | | | | **0.91 (0.66, 1.25)** | | |

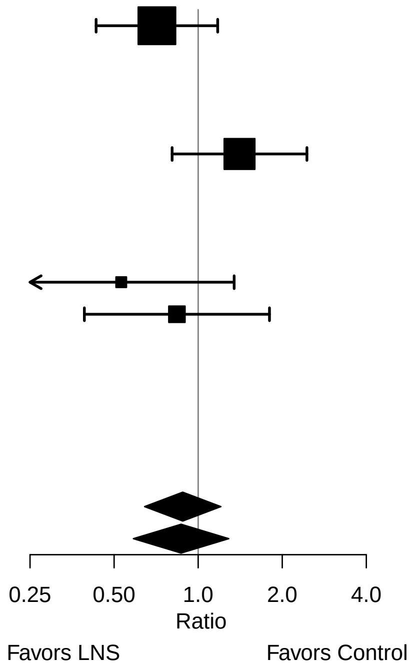


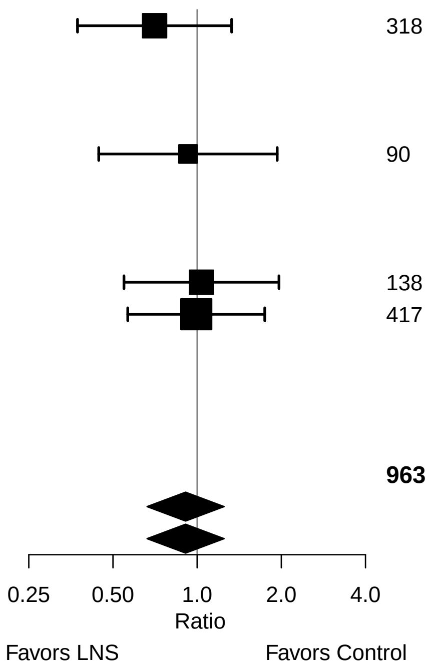

# Supplemental figure 7C: Language lowest decile prevalence difference

## 7C1: Stratified by Maternal height

**P−for−interaction = 0.813**
**Difference in PDs = 0.00 (−0.02, 0.03)**

| Country | Trial | Tool | LNS N | Control N | Control Prevalence | At least 150.1 cm PD (95% CI) | Fixed W | Random W | LNS N | Control N | Control Prevalence | Less than 150.1 cm PD (95% CI) | Fixed W | Random W |
|---|---|---|---|---|---|---|---|---|---|---|---|---|---|---|
| Bangladesh | JiVitA−4 (21) | BSID−III | | | | | | | | | | | | |
| Bangladesh | RDNS (22) | CDI | 868 | 431 | 12.5 | −0.05 (−0.09, −0.02) | 0.14 | 0.23 | 735 | 358 | 12.0 | −0.01 (−0.05, 0.03) | 0.27 | 0.27 |
| Bangladesh | WASH−B (23) | CDI | 589 | 1812 | 10.2 | −0.02 (−0.04, 0.01) | 0.31 | 0.29 | 515 | 1499 | 10.8 | −0.02 (−0.05, 0.00) | 0.53 | 0.53 |
| Burkina Faso | iLiNS−Zinc (24) | DMC | | | | | | | | | | | | |
| Ghana | GHANA (25) | | | | | | | | | | | | | |
| Ghana | iLiNS−DYAD−G (26) | CDI | | | | | | | | | | | | |
| Haiti | HAITI (27) | Total sounds | | | | | | | | | | | | |
| Kenya | WASH−B (28) | EASQ | 1237 | 4372 | 9.7 | 0.00 (−0.02, 0.02) | 0.48 | 0.32 | 51 | 175 | 15.4 | −0.06 (−0.16, 0.04) | 0.05 | 0.05 |
| Madagascar | MAHAY (29) | ASQI | 956 | 986 | 8.7 | 0.02 (−0.04, 0.07) | 0.07 | 0.16 | 553 | 538 | 9.9 | 0.01 (−0.04, 0.07) | 0.16 | 0.16 |
| Malawi | iLiNS−DYAD−M (30) | CDI | | | | | | | | | | | | |
| Malawi | iLiNS−DOSE (31) | CDI | | | | | | | | | | | | |
| Mali | PROMIS CS (32) | DMC | | | | | | | | | | | | |
| Zimbabwe | SHINE (HIV−) (33) | CDI | | | | | | | | | | | | |
| Zimbabwe | SHINE (HIV+) (34) | CDI | | | | | | | | | | | | |
| | | | **3650** | **7601** | | **$I^2$ = 0.57, $Tau^2$ = 0.00** | | | **1854** | **2570** | | **$I^2$ = 0.00, $Tau^2$ = 0.00** | | |
| **Fixed** | | | | | | **−0.01 (−0.03, 0.00)** | | | | | | **−0.02 (−0.04, 0.01)** | | |
| **Random** | | | | | | **−0.02 (−0.04, 0.01)** | | | | | | **−0.02 (−0.04, 0.01)** | | |

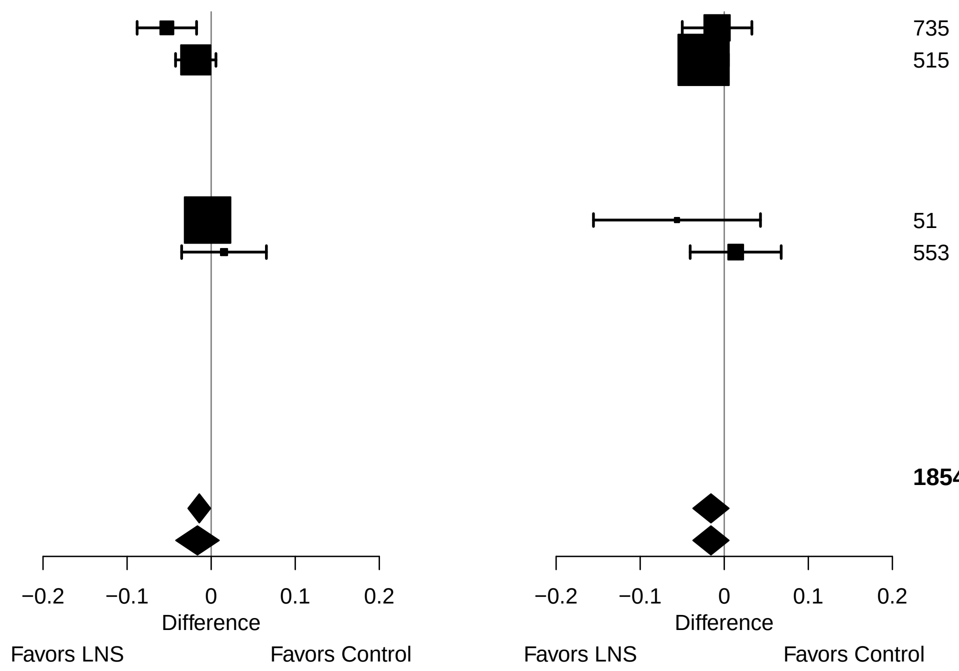

## Supplemental figure 7C: Language lowest decile prevalence difference

### 7C2: Stratified by Maternal BMI

**P−for−interaction = 0.303**
**Difference in PDs = 0.01 (−0.01, 0.03)**

| Country | Trial | Tool | At least 20 kg/m²: LNS N | Control N | Control Prevalence | PD (95% CI) | Fixed W | Random W | Less than 20 kg/m²: LNS N | Control N | Control Prevalence | PD (95% CI) | Fixed W | Random W |
|---|---|---|---|---|---|---|---|---|---|---|---|---|---|---|
| Bangladesh | JiVitA−4 (21) | BSID−III | | | | | | | | | | | | |
| Bangladesh | RDNS (22) | CDI | 730 | 334 | 12.0 | −0.06 (−0.09, −0.02) | 0.12 | 0.15 | 873 | 455 | 12.5 | −0.01 (−0.05, 0.02) | 0.21 | 0.19 |
| Bangladesh | WASH−B (23) | CDI | 498 | 1529 | 9.5 | −0.04 (−0.06, −0.01) | 0.27 | 0.23 | 606 | 1781 | 11.2 | −0.01 (−0.04, 0.02) | 0.36 | 0.20 |
| Burkina Faso | iLiNS−Zinc (24) | DMC | 480 | 225 | 12.4 | −0.05 (−0.10, −0.01) | 0.07 | 0.10 | 264 | 150 | 20.7 | −0.13 (−0.19, −0.07) | 0.07 | 0.15 |
| Ghana | GHANA (25) | | | | | | | | | | | | | |
| Ghana | iLiNS−DYAD−G (26) | CDI | | | | | | | | | | | | |
| Haiti | HAITI (27) | Total sounds | | | | | | | | | | | | |
| Kenya | WASH−B (28) | EASQ | 1031 | 3535 | 10.0 | −0.01 (−0.03, 0.02) | 0.33 | 0.25 | 257 | 1012 | 9.8 | −0.01 (−0.05, 0.03) | 0.19 | 0.18 |
| Madagascar | MAHAY (29) | ASQI | | | | | | | | | | | | |
| Malawi | iLiNS−DYAD−M (30) | CDI | | | | | | | | | | | | |
| Malawi | iLiNS−DOSE (31) | CDI | | | | | | | | | | | | |
| Mali | PROMIS CS (32) | DMC | 683 | 664 | 11.9 | −0.04 (−0.08, 0.00) | 0.10 | 0.13 | 238 | 274 | 9.9 | 0.01 (−0.04, 0.05) | 0.12 | 0.17 |
| Zimbabwe | SHINE (HIV−) (33) | CDI | 581 | 569 | 10.9 | −0.01 (−0.04, 0.03) | 0.12 | 0.15 | 96 | 93 | 15.1 | −0.09 (−0.17, 0.00) | 0.04 | 0.12 |
| Zimbabwe | SHINE (HIV+) (34) | CDI | | | | | | | | | | | | |
| | | | **4003** | **6856** | | **I² = 0.48, Tau² = 0.00** | | | **2334** | **3765** | | **I² = 0.71, Tau² = 0.00** | | |
| **Fixed** | | | | | | **−0.03 (−0.04, −0.01)** | | | | | | **−0.02 (−0.04, 0.00)** | | |
| **Random** | | | | | | **−0.03 (−0.05, −0.01)** | | | | | | **−0.03 (−0.08, 0.01)** | | |

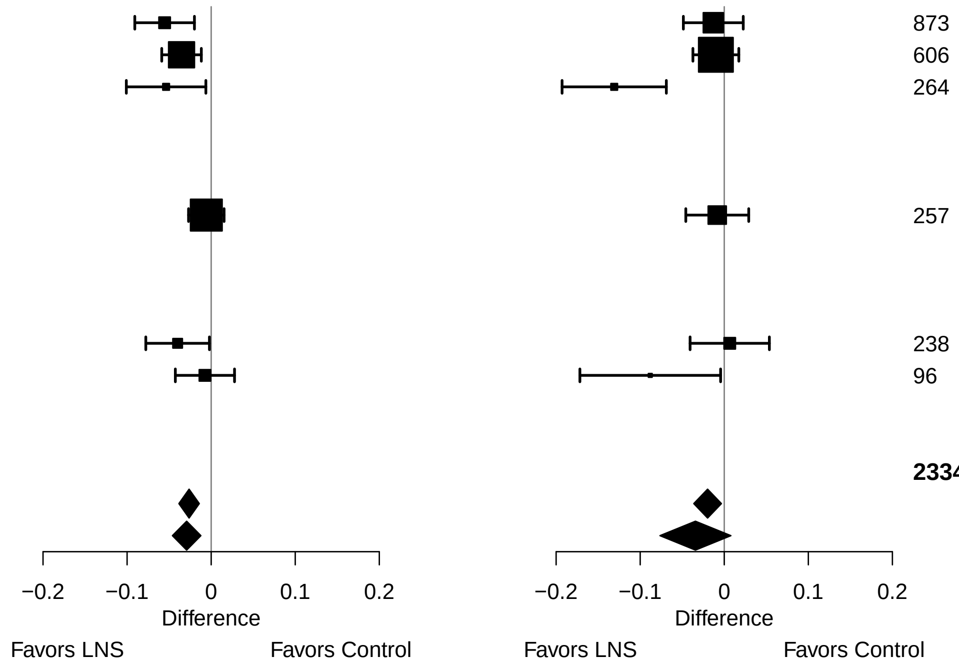

## Supplemental figure 7C: Language lowest decile prevalence difference

### 7C3: Stratified by Maternal age

**P−for−interaction = 0.820**
**Difference in PDs = 0.00 (−0.02, 0.02)**

| Country | Trial | Tool | At least 25 y: LNS N | At least 25 y: Control N | At least 25 y: Control Prevalence | At least 25 y: PD (95% CI) | At least 25 y: Fixed W | At least 25 y: Random W | Less than 25 y: LNS N | Less than 25 y: Control N | Less than 25 y: Control Prevalence | Less than 25 y: PD (95% CI) | Less than 25 y: Fixed W | Less than 25 y: Random W |
|---|---|---|---|---|---|---|---|---|---|---|---|---|---|---|
| Bangladesh | JiVitA−4 (21) | BSID−III | 200 | 68 | 11.8 | −0.01 (−0.08, 0.06) | 0.03 | 0.03 | 242 | 74 | 10.8 | −0.02 (−0.10, 0.07) | 0.02 | 0.05 |
| Bangladesh | RDNS (22) | CDI | 453 | 220 | 13.6 | −0.02 (−0.07, 0.02) | 0.08 | 0.08 | 1210 | 594 | 12.0 | −0.04 (−0.07, −0.01) | 0.20 | 0.14 |
| Bangladesh | WASH−B (23) | CDI | 477 | 1475 | 11.5 | −0.01 (−0.04, 0.02) | 0.17 | 0.17 | 627 | 1863 | 9.8 | −0.03 (−0.05, 0.00) | 0.27 | 0.15 |
| Burkina Faso | iLiNS−Zinc (24) | DMC | 421 | 232 | 15.1 | −0.07 (−0.13, −0.02) | 0.06 | 0.06 | 321 | 141 | 17.0 | −0.10 (−0.18, −0.03) | 0.03 | 0.05 |
| Ghana | GHANA (25) | | | | | | | | | | | | | |
| Ghana | iLiNS−DYAD−G (26) | CDI | 211 | 405 | 10.4 | −0.02 (−0.07, 0.03) | 0.07 | 0.07 | 120 | 253 | 8.7 | 0.05 (−0.01, 0.12) | 0.03 | 0.06 |
| Haiti | HAITI (27) | Total sounds | | | | | | | | | | | | |
| Kenya | WASH−B (28) | EASQ | 798 | 2622 | 9.9 | 0.00 (−0.03, 0.03) | 0.20 | 0.20 | 549 | 2092 | 10.3 | −0.01 (−0.04, 0.02) | 0.19 | 0.14 |
| Madagascar | MAHAY (29) | ASQI | 852 | 901 | 9.8 | 0.01 (−0.03, 0.06) | 0.07 | 0.07 | 759 | 703 | 8.5 | 0.02 (−0.03, 0.07) | 0.06 | 0.09 |
| Malawi | iLiNS−DYAD−M (30) | CDI | 103 | 225 | 14.7 | −0.05 (−0.13, 0.03) | 0.03 | 0.03 | 112 | 214 | 6.5 | 0.02 (−0.04, 0.08) | 0.04 | 0.07 |
| Malawi | iLiNS−DOSE (31) | CDI | 345 | 124 | 11.3 | −0.01 (−0.07, 0.05) | 0.04 | 0.04 | 288 | 90 | 8.9 | 0.00 (−0.07, 0.07) | 0.03 | 0.06 |
| Mali | PROMIS CS (32) | DMC | 492 | 492 | 11.0 | −0.04 (−0.08, 0.00) | 0.12 | 0.12 | 435 | 452 | 11.7 | −0.01 (−0.06, 0.03) | 0.08 | 0.10 |
| Zimbabwe | SHINE (HIV−) (33) | CDI | 414 | 384 | 9.9 | −0.02 (−0.06, 0.01) | 0.12 | 0.12 | 315 | 310 | 12.9 | −0.01 (−0.06, 0.05) | 0.05 | 0.08 |
| Zimbabwe | SHINE (HIV+) (34) | CDI | | | | | | | | | | | | |
| | | | **4766** | **7148** | | **I² = 0.00, Tau² = 0.00** | | | **4978** | **6786** | | **I² = 0.44, Tau² = 0.00** | | |
| **Fixed** | | | | | | **−0.02 (−0.03, 0.00)** | | | | | | **−0.02 (−0.03, −0.01)** | | |
| **Random** | | | | | | **−0.02 (−0.03, 0.00)** | | | | | | **−0.01 (−0.03, 0.01)** | | |

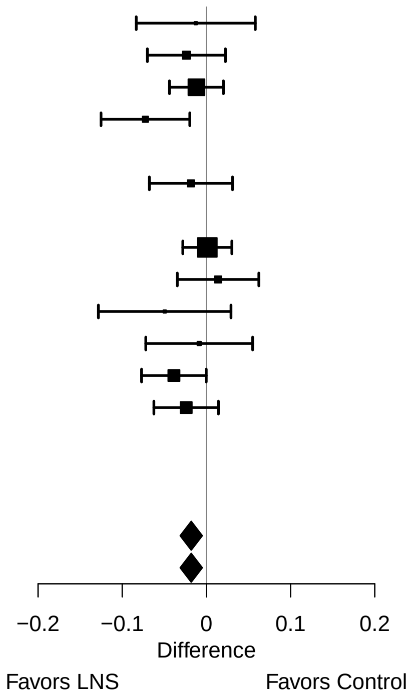


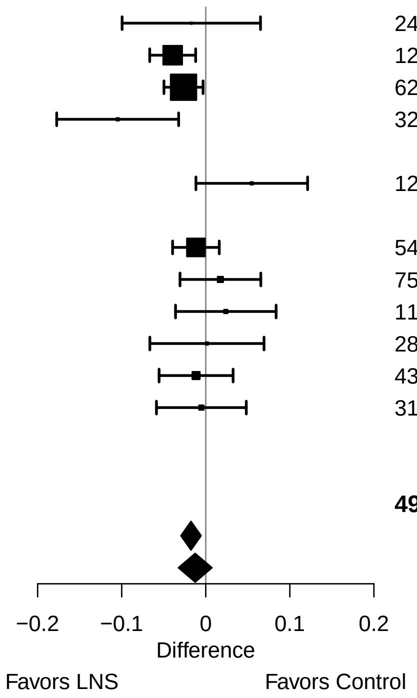

## Supplemental figure 7C: Language lowest decile prevalence difference

### 7C4: Stratified by Maternal education

**P−for−interaction = 0.116**
**Difference in PDs = −0.02 (−0.04, 0.00)**

| Country | Trial | Tool | LNS N | Control N | Control Prevalence | Primary or greater PD (95% CI) | Fixed W | Random W | LNS N | Control N | Control Prevalence | Incomplete or no formal PD (95% CI) | Fixed W | Random W |
|---|---|---|---|---|---|---|---|---|---|---|---|---|---|---|
| Bangladesh | JiVitA−4 (21) | BSID−III | 292 | 92 | 8.7 | −0.01 (−0.08, 0.06) | 0.02 | 0.07 | 152 | 51 | 15.7 | −0.02 (−0.13, 0.09) | 0.02 | 0.02 |
| Bangladesh | RDNS (22) | CDI | 1245 | 588 | 9.9 | −0.03 (−0.06, −0.01) | 0.20 | 0.20 | 418 | 226 | 19.0 | −0.03 (−0.10, 0.03) | 0.07 | 0.07 |
| Bangladesh | WASH−B (23) | CDI | 781 | 2406 | 8.9 | −0.01 (−0.03, 0.01) | 0.35 | 0.23 | 328 | 947 | 14.8 | −0.04 (−0.08, 0.00) | 0.18 | 0.18 |
| Burkina Faso | iLiNS−Zinc (24) | DMC | | | | | | | | | | | | |
| Ghana | GHANA (25) | | | | | | | | | | | | | |
| Ghana | iLiNS−DYAD−G (26) | CDI | 256 | 519 | 9.4 | 0.00 (−0.04, 0.04) | 0.06 | 0.12 | 75 | 139 | 10.8 | 0.04 (−0.05, 0.13) | 0.03 | 0.03 |
| Haiti | HAITI (27) | Total sounds | | | | | | | | | | | | |
| Kenya | WASH−B (28) | EASQ | 658 | 2258 | 6.9 | −0.01 (−0.03, 0.01) | 0.31 | 0.23 | 702 | 2484 | 13.0 | 0.00 (−0.03, 0.03) | 0.32 | 0.32 |
| Madagascar | MAHAY (29) | ASQI | 339 | 416 | 3.6 | 0.06 (0.01, 0.11) | 0.05 | 0.11 | 1274 | 1188 | 11.2 | 0.00 (−0.05, 0.05) | 0.11 | 0.11 |
| Malawi | iLiNS−DYAD−M (30) | CDI | | | | | | | | | | | | |
| Malawi | iLiNS−DOSE (31) | CDI | | | | | | | | | | | | |
| Mali | PROMIS CS (32) | DMC | 100 | 94 | 7.4 | 0.02 (−0.09, 0.12) | 0.01 | 0.03 | 826 | 850 | 11.8 | −0.03 (−0.06, 0.00) | 0.25 | 0.25 |
| Zimbabwe | SHINE (HIV−) (33) | CDI | | | | | | | | | | | | |
| Zimbabwe | SHINE (HIV+) (34) | CDI | | | | | | | | | | | | |
| | | | **3671** | **6373** | | **I² = 0.50, Tau² = 0.00** | | | **3775** | **5885** | | **I² = 0.00, Tau² = 0.00** | | |
| **Fixed** | | | | | | **−0.01 (−0.02, 0.00)** | | | | | | **−0.02 (−0.03, 0.00)** | | |
| **Random** | | | | | | **−0.01 (−0.03, 0.01)** | | | | | | **−0.02 (−0.03, 0.00)** | | |

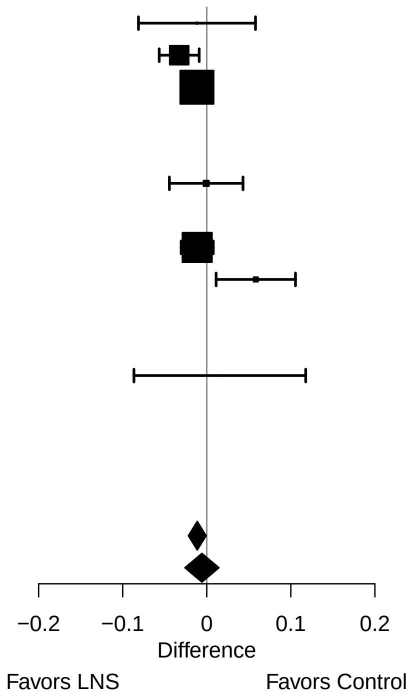


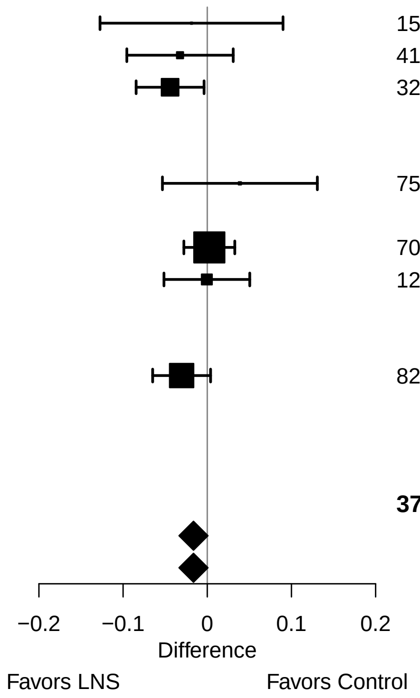

## Supplemental figure 7C: Language lowest decile prevalence difference

### 7C5: Stratified by Maternal depressive symptoms

**−for−interaction = 0.461**
**ifference in PDs = −0.01 (−0.03, 0.01)**

| | | | | | | Less than 75th percentile | | | | | | At least 75th percentile | | |
|---|---|---|---|---|---|---|---|---|---|---|---|---|---|---|
| **ountry** | **Trial** | **Tool** | **LNS N** | **Control N** | **Control Prevalence** | **PD (95% CI)** | **Fixed W** | **Random W** | **LNS N** | **Control N** | **Control Prevalence** | **PD (95% CI)** | **Fixed W** | **Random W** |
| angladesh | JiVitA−4 (21) | BSID−III | | | | | | | | | | | | |
| angladesh | RDNS (22) | CDI | 1081 | 471 | 11.9 | −0.04 (−0.08, −0.01) | 0.11 | 0.11 | 520 | 288 | 13.5 | −0.02 (−0.06, 0.02) | 0.21 | 0.21 |
| angladesh | WASH−B (23) | CDI | 856 | 2410 | 10.2 | −0.03 (−0.05, −0.01) | 0.31 | 0.31 | 232 | 859 | 11.3 | 0.02 (−0.03, 0.07) | 0.15 | 0.15 |
| urkina Faso | iLiNS−Zinc (24) | DMC | | | | | | | | | | | | |
| hana | GHANA (25) | | | | | | | | | | | | | |
| hana | iLiNS−DYAD−G (26) | CDI | 241 | 435 | 10.8 | 0.00 (−0.05, 0.05) | 0.06 | 0.06 | 78 | 202 | 7.4 | 0.03 (−0.04, 0.10) | 0.07 | 0.07 |
| aiti | HAITI (27) | Total sounds | | | | | | | | | | | | |
| enya | WASH−B (28) | EASQ | 960 | 3317 | 9.5 | −0.01 (−0.03, 0.01) | 0.35 | 0.35 | 317 | 1156 | 8.7 | 0.00 (−0.03, 0.04) | 0.36 | 0.36 |
| ladagascar | MAHAY (29) | ASQI | 615 | 667 | 8.8 | 0.03 (−0.03, 0.08) | 0.04 | 0.04 | 268 | 242 | 12.0 | −0.03 (−0.10, 0.04) | 0.08 | 0.08 |
| lalawi | iLiNS−DYAD−M (30) | CDI | 153 | 302 | 9.9 | −0.01 (−0.07, 0.05) | 0.04 | 0.04 | 47 | 112 | 12.5 | −0.02 (−0.13, 0.09) | 0.03 | 0.03 |
| lalawi | iLiNS−DOSE (31) | CDI | | | | | | | | | | | | |
| lali | PROMIS CS (32) | DMC | | | | | | | | | | | | |
| imbabwe | SHINE (HIV−) (33) | CDI | 566 | 552 | 10.3 | −0.01 (−0.05, 0.03) | 0.10 | 0.10 | 190 | 173 | 14.5 | −0.04 (−0.11, 0.02) | 0.10 | 0.10 |
| imbabwe | SHINE (HIV+) (34) | CDI | | | | | | | | | | | | |
| | | | **4472** | **8154** | | **I² = 0.12, Tau² = 0.00** | | | **1652** | **3032** | | **I² = 0.00, Tau² = 0.00** | | |
| **ixed** | | | | | | **−0.02 (−0.03, −0.01)** | | | | | | **0.00 (−0.02, 0.02)** | | |
| **andom** | | | | | | **−0.02 (−0.03, −0.01)** | | | | | | **0.00 (−0.02, 0.02)** | | |

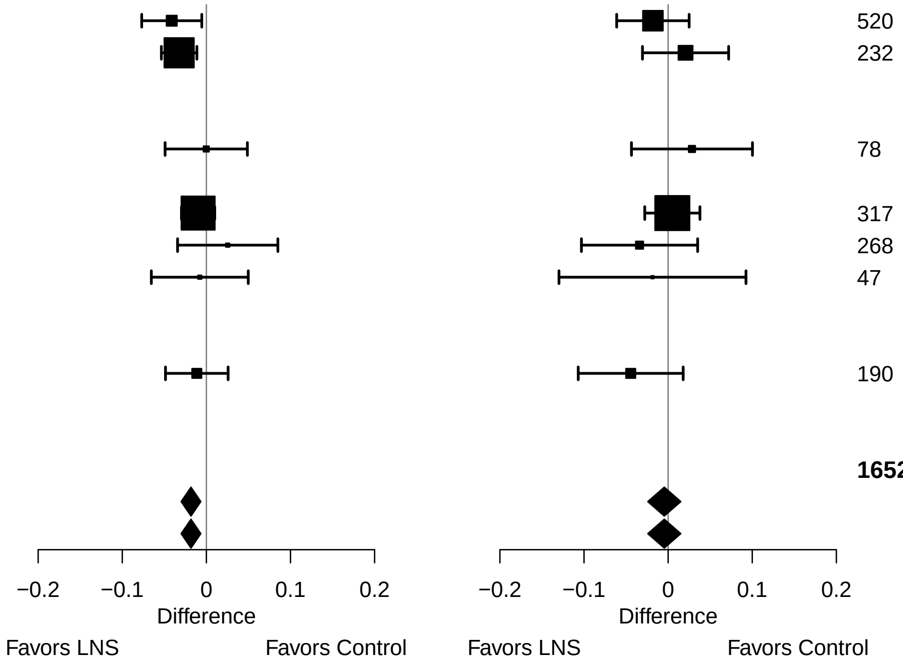

**Supplemental figure 7C: Language lowest decile prevalence difference**

## 7C6: Stratified by Child sex

**P−for−interaction = 0.199**
**Difference in PDs = −0.01 (−0.03, 0.01)**

| Country | Trial | Tool | LNS N | Control N | Control Prevalence | Male PD (95% CI) | Fixed W | Random W | LNS N | Control N | Control Prevalence | Female PD (95% CI) | Fixed W | Random W |
|---|---|---|---|---|---|---|---|---|---|---|---|---|---|---|
| Bangladesh | JiVitA−4 (21) | BSID−III | 231 | 70 | 14.3 | −0.05 (−0.15, 0.05) | 0.01 | 0.02 | 214 | 73 | 8.2 | 0.02 (−0.04, 0.08) | 0.05 | 0.07 |
| Bangladesh | RDNS (22) | CDI | 833 | 411 | 10.5 | 0.00 (−0.03, 0.04) | 0.11 | 0.11 | 830 | 403 | 14.4 | −0.07 (−0.11, −0.04) | 0.12 | 0.11 |
| Bangladesh | WASH−B (23) | CDI | 545 | 1685 | 11.5 | −0.02 (−0.05, 0.00) | 0.19 | 0.15 | 564 | 1668 | 9.6 | −0.02 (−0.04, 0.01) | 0.22 | 0.13 |
| Burkina Faso | iLiNS−Zinc (24) | DMC | 372 | 193 | 12.4 | −0.07 (−0.10, −0.03) | 0.11 | 0.11 | 374 | 182 | 19.2 | −0.11 (−0.16, −0.05) | 0.05 | 0.08 |
| Ghana | GHANA (25) | | | | | | | | | | | | | |
| Ghana | iLiNS−DYAD−G (26) | CDI | 164 | 306 | 8.8 | 0.04 (−0.02, 0.10) | 0.04 | 0.06 | 167 | 352 | 10.5 | −0.02 (−0.08, 0.03) | 0.05 | 0.07 |
| Haiti | HAITI (27) | Total sounds | | | | | | | | | | | | |
| Kenya | WASH−B (28) | EASQ | 682 | 2260 | 10.7 | 0.00 (−0.02, 0.03) | 0.19 | 0.15 | 680 | 2485 | 9.6 | −0.01 (−0.04, 0.01) | 0.22 | 0.13 |
| Madagascar | MAHAY (29) | ASQI | 795 | 787 | 8.9 | 0.01 (−0.03, 0.06) | 0.07 | 0.08 | 818 | 817 | 9.5 | 0.02 (−0.04, 0.07) | 0.05 | 0.07 |
| Malawi | iLiNS−DYAD−M (30) | CDI | 105 | 205 | 8.8 | −0.02 (−0.09, 0.04) | 0.03 | 0.05 | 110 | 234 | 12.4 | −0.01 (−0.08, 0.07) | 0.03 | 0.05 |
| Malawi | iLiNS−DOSE (31) | CDI | 320 | 117 | 11.1 | −0.01 (−0.08, 0.05) | 0.04 | 0.05 | 325 | 104 | 9.6 | 0.01 (−0.06, 0.07) | 0.03 | 0.06 |
| Mali | PROMIS CS (32) | DMC | 469 | 514 | 10.7 | −0.03 (−0.06, 0.01) | 0.11 | 0.11 | 458 | 430 | 12.1 | −0.03 (−0.07, 0.02) | 0.08 | 0.10 |
| Zimbabwe | SHINE (HIV−) (33) | CDI | 412 | 378 | 11.6 | −0.01 (−0.05, 0.03) | 0.07 | 0.08 | 405 | 403 | 9.9 | −0.01 (−0.06, 0.03) | 0.07 | 0.09 |
| Zimbabwe | SHINE (HIV+) (34) | CDI | 82 | 76 | 13.2 | −0.03 (−0.14, 0.07) | 0.01 | 0.02 | 83 | 71 | 11.3 | −0.04 (−0.13, 0.05) | 0.02 | 0.04 |
| | | | **5010** | **7002** | | **$I^2$ = 0.30, $Tau^2$ = 0.00** | | | **5028** | **7222** | | **$I^2$ = 0.52, $Tau^2$ = 0.00** | | |
| **Fixed** | | | | | | **−0.01 (−0.03, 0.00)** | | | | | | **−0.02 (−0.04, −0.01)** | | |
| **Random** | | | | | | **−0.01 (−0.03, 0.00)** | | | | | | **−0.02 (−0.04, 0.00)** | | |

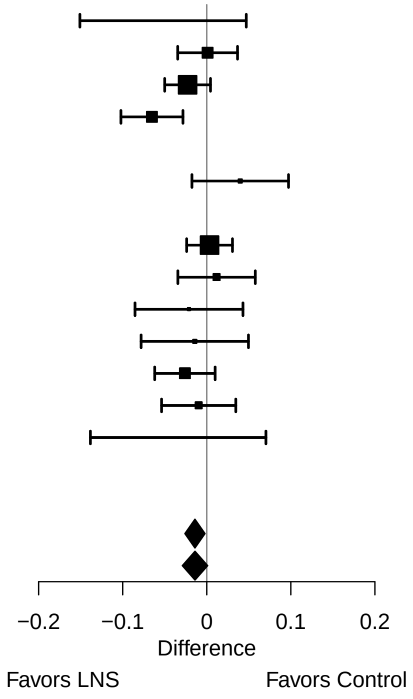


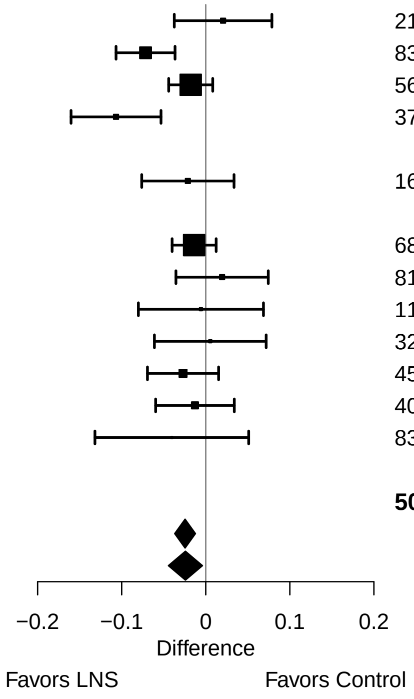

## Supplemental figure 7C: Language lowest decile prevalence difference

### 7C7: Stratified by Child birth order

**P−for−interaction = 0.300**
**Difference in PDs = 0.01 (−0.01, 0.03)**

| Country | Trial | Tool | LNS N | Control N | Control Prevalence | Later born PD (95% CI) | Fixed W | Random W | LNS N | Control N | Control Prevalence | Firstborn PD (95% CI) | Fixed W | Random W |
|---|---|---|---|---|---|---|---|---|---|---|---|---|---|---|
| Bangladesh | JiVitA−4 (21) | BSID−III | | | | | | | | | | | | |
| Bangladesh | RDNS (22) | CDI | 974 | 507 | 14.0 | −0.04 (−0.08, −0.01) | 0.11 | 0.12 | 688 | 307 | 9.8 | −0.02 (−0.06, 0.02) | 0.18 | 0.16 |
| Bangladesh | WASH−B (23) | CDI | 693 | 2187 | 11.6 | −0.03 (−0.05, 0.00) | 0.20 | 0.16 | 395 | 1060 | 8.3 | −0.01 (−0.04, 0.02) | 0.35 | 0.19 |
| Burkina Faso | iLiNS−Zinc (24) | DMC | 587 | 290 | 15.5 | −0.08 (−0.13, −0.03) | 0.05 | 0.08 | 159 | 85 | 16.5 | −0.10 (−0.18, −0.01) | 0.04 | 0.07 |
| Ghana | GHANA (25) | | | | | | | | | | | | | |
| Ghana | iLiNS−DYAD−G (26) | CDI | 224 | 445 | 10.1 | −0.01 (−0.06, 0.04) | 0.06 | 0.08 | 107 | 213 | 8.9 | 0.05 (−0.02, 0.12) | 0.06 | 0.09 |
| Haiti | HAITI (27) | Total sounds | | | | | | | | | | | | |
| Kenya | WASH−B (28) | EASQ | 1071 | 3767 | 9.8 | 0.00 (−0.03, 0.02) | 0.25 | 0.17 | 289 | 974 | 11.4 | −0.01 (−0.06, 0.03) | 0.14 | 0.14 |
| Madagascar | MAHAY (29) | ASQI | 1162 | 1118 | 9.1 | 0.02 (−0.03, 0.06) | 0.06 | 0.09 | 433 | 465 | 9.7 | 0.01 (−0.05, 0.06) | 0.09 | 0.11 |
| Malawi | iLiNS−DYAD−M (30) | CDI | 170 | 350 | 11.7 | −0.04 (−0.10, 0.02) | 0.04 | 0.07 | 45 | 87 | 6.9 | 0.09 (−0.02, 0.19) | 0.02 | 0.05 |
| Malawi | iLiNS−DOSE (31) | CDI | | | | | | | | | | | | |
| Mali | PROMIS CS (32) | DMC | 770 | 811 | 11.2 | −0.03 (−0.06, 0.01) | 0.12 | 0.13 | 142 | 122 | 9.0 | 0.01 (−0.06, 0.08) | 0.06 | 0.09 |
| Zimbabwe | SHINE (HIV−) (33) | CDI | 587 | 562 | 9.4 | −0.01 (−0.04, 0.03) | 0.09 | 0.11 | 194 | 185 | 15.7 | −0.03 (−0.10, 0.04) | 0.06 | 0.09 |
| Zimbabwe | SHINE (HIV+) (34) | CDI | | | | | | | | | | | | |
| | | | **6238** | **10037** | | **I² = 0.45, Tau² = 0.00** | | | **2452** | **3498** | | **I² = 0.30, Tau² = 0.00** | | |
| **Fixed** | | | | | | **−0.02 (−0.03, −0.01)** | | | | | | **−0.01 (−0.02, 0.01)** | | |
| **Random** | | | | | | **−0.02 (−0.04, −0.01)** | | | | | | **−0.01 (−0.03, 0.02)** | | |

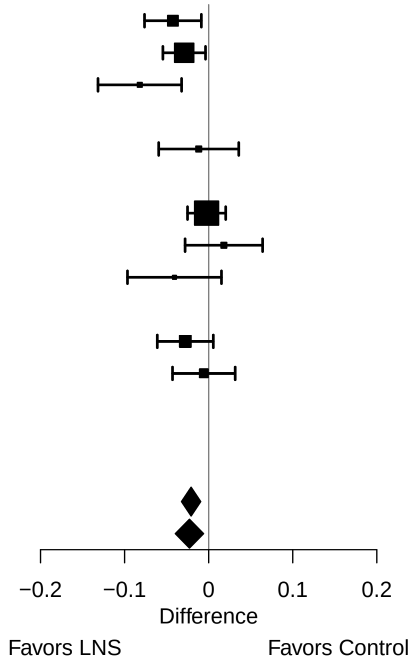


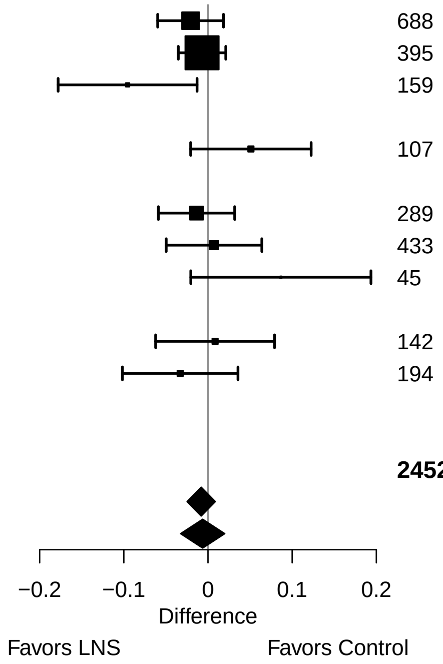

**Supplemental figure 7C: Language lowest decile prevalence difference**

## 7C8: Stratified by Child baseline stunting

**P−for−interaction = 0.021**
**Difference in PDs = −0.05 (−0.09, −0.01)**

| Country | Trial | Tool | No: LNS N | No: Control N | No: Control Prevalence | No: PD (95% CI) | No: Fixed W | No: Random W | Yes: LNS N | Yes: Control N | Yes: Control Prevalence | Yes: PD (95% CI) | Yes: Fixed W | Yes: Random W |
|---|---|---|---|---|---|---|---|---|---|---|---|---|---|---|
| Bangladesh | JiVitA−4 (21) | BSID−III | 327 | 114 | 8.8 | −0.01 (−0.07, 0.06) | 0.06 | 0.07 | 116 | 29 | 20.7 | −0.07 (−0.18, 0.05) | 0.10 | 0.13 |
| Bangladesh | RDNS (22) | CDI | 1246 | 573 | 10.6 | −0.03 (−0.05, 0.00) | 0.38 | 0.32 | 355 | 185 | 18.4 | −0.06 (−0.12, 0.01) | 0.35 | 0.27 |
| Bangladesh | WASH−B (23) | CDI | | | | | | | | | | | | |
| Burkina Faso | iLiNS−Zinc (24) | DMC | 576 | 294 | 12.6 | −0.06 (−0.09, −0.02) | 0.20 | 0.20 | 170 | 80 | 27.5 | −0.19 (−0.29, −0.10) | 0.15 | 0.16 |
| Ghana | GHANA (25) | | | | | | | | | | | | | |
| Ghana | iLiNS−DYAD−G (26) | CDI | | | | | | | | | | | | |
| Haiti | HAITI (27) | Total sounds | | | | | | | | | | | | |
| Kenya | WASH−B (28) | EASQ | | | | | | | | | | | | |
| Madagascar | MAHAY (29) | ASQI | | | | | | | | | | | | |
| Malawi | iLiNS−DYAD−M (30) | CDI | 153 | 323 | 8.4 | 0.00 (−0.05, 0.05) | 0.08 | 0.10 | 48 | 97 | 19.6 | −0.07 (−0.20, 0.06) | 0.08 | 0.10 |
| Malawi | iLiNS−DOSE (31) | CDI | 464 | 147 | 8.2 | 0.01 (−0.04, 0.06) | 0.09 | 0.10 | 181 | 74 | 14.9 | −0.03 (−0.12, 0.06) | 0.17 | 0.18 |
| Mali | PROMIS CS (32) | DMC | | | | | | | | | | | | |
| Zimbabwe | SHINE (HIV−) (33) | CDI | 523 | 482 | 10.0 | −0.02 (−0.06, 0.01) | 0.19 | 0.20 | 119 | 100 | 16.0 | −0.04 (−0.14, 0.05) | 0.15 | 0.17 |
| Zimbabwe | SHINE (HIV+) (34) | CDI | | | | | | | | | | | | |
| | | | **3289** | **1933** | | **I² = 0.15, Tau² = 0.00** | | | **989** | **565** | | **I² = 0.34, Tau² = 0.00** | | |
| **Fixed** | | | | | | **−0.03 (−0.04, −0.01)** | | | | | | **−0.07 (−0.11, −0.04)** | | |
| **Random** | | | | | | **−0.02 (−0.04, −0.01)** | | | | | | **−0.07 (−0.12, −0.03)** | | |

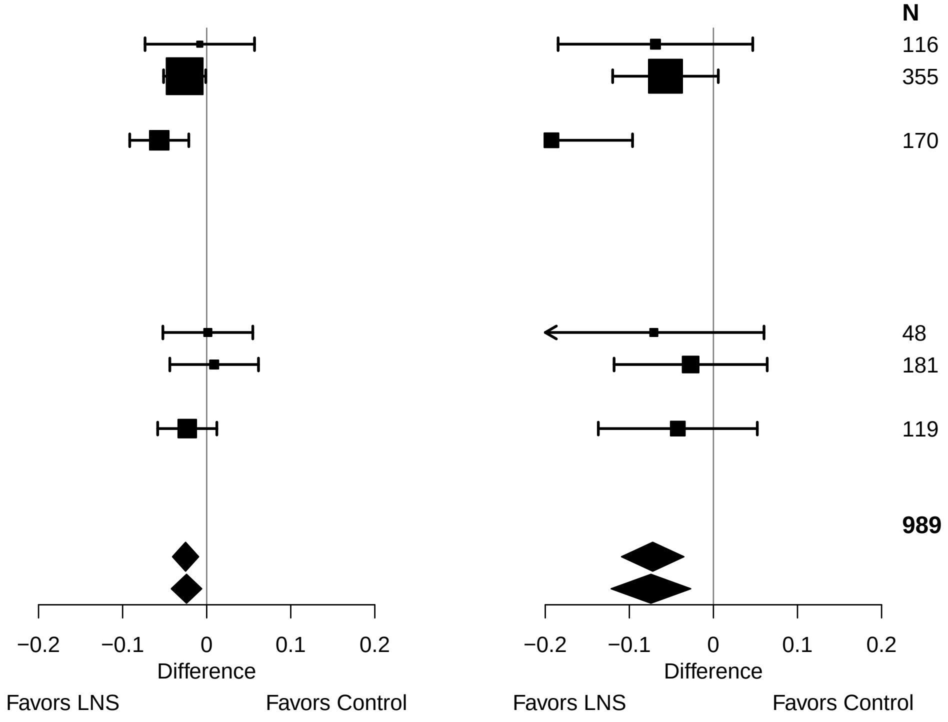

## Supplemental figure 7C: Language lowest decile prevalence difference

## 7C9: Stratified by Child baseline acute malnutrition

**P−for−interaction = 0.660**
**Difference in PDs = −0.02 (−0.09, 0.06)**

**No**

| Country | Trial | Tool | LNS N | Control N | Control Prevalence | PD (95% CI) | Fixed W | Random W |
|---|---|---|---|---|---|---|---|---|
| Bangladesh | JiVitA−4 (21) | BSID−III | 359 | 112 | 9.8 | −0.01 (−0.08, 0.06) | 0.08 | 0.18 |
| Bangladesh | RDNS (22) | CDI | 1477 | 706 | 11.9 | −0.03 (−0.06, −0.01) | 0.66 | 0.47 |
| Bangladesh | WASH−B (23) | CDI | | | | | | |
| Burkina Faso | iLiNS−Zinc (24) | DMC | 556 | 266 | 13.5 | −0.08 (−0.12, −0.04) | 0.26 | 0.35 |
| Ghana | GHANA (25) | | | | | | | |
| Ghana | iLiNS−DYAD−G (26) | CDI | | | | | | |
| Haiti | HAITI (27) | Total sounds | | | | | | |
| Kenya | WASH−B (28) | EASQ | | | | | | |
| Madagascar | MAHAY (29) | ASQI | | | | | | |
| Malawi | iLiNS−DYAD−M (30) | CDI | | | | | | |
| Malawi | iLiNS−DOSE (31) | CDI | | | | | | |
| Mali | PROMIS CS (32) | DMC | | | | | | |
| Zimbabwe | SHINE (HIV−) (33) | CDI | | | | | | |
| Zimbabwe | SHINE (HIV+) (34) | CDI | | | | | | |
| | | | **2392** | **1084** | | **I² = 0.56, Tau² = 0.00** | | |
| **Fixed** | | | | | | **−0.04 (−0.06, −0.02)** | | |
| **Random** | | | | | | **−0.05 (−0.08, −0.01)** | | |

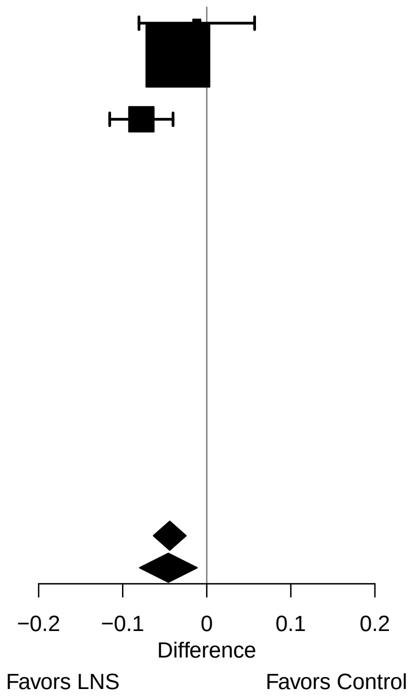


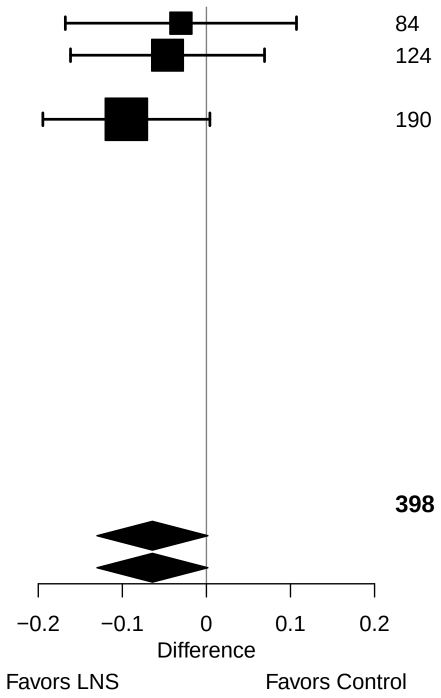


**Yes**

| Country | Trial | LNS N | Control N | Control Prevalence | PD (95% CI) | Fixed W | Random W |
|---|---|---|---|---|---|---|---|
| Bangladesh | JiVitA−4 (21) | 84 | 31 | 16.1 | −0.03 (−0.17, 0.11) | 0.23 | 0.23 |
| Bangladesh | RDNS (22) | 124 | 53 | 20.8 | −0.05 (−0.16, 0.07) | 0.33 | 0.33 |
| Burkina Faso | iLiNS−Zinc (24) | 190 | 109 | 21.1 | −0.10 (−0.19, 0.00) | 0.44 | 0.44 |
| | | **398** | **193** | | **I² = 0.00, Tau² = 0.00** | | |
| **Fixed** | | | | | **−0.06 (−0.13, 0.00)** | | |
| **Random** | | | | | **−0.06 (−0.13, 0.00)** | | |

## Supplemental figure 7C: Language lowest decile prevalence difference

## 7C10: Stratified by Child baseline anemia

**P−for−interaction = 0.698**
**Difference in PDs = 0.01 (−0.04, 0.06)**

| Country | Trial | Tool | Not anemic: LNS N | Control N | Control Prevalence | PD (95% CI) | Fixed W | Random W | Anemic: LNS N | Control N | Control Prevalence | PD (95% CI) | Fixed W | Random W |
|---|---|---|---|---|---|---|---|---|---|---|---|---|---|---|
| Bangladesh | JiVitA−4 (21) | BSID−III | | | | | | | | | | | | |
| Bangladesh | RDNS (22) | CDI | 212 | 96 | 14.6 | −0.04 (−0.11, 0.02) | 0.23 | 0.25 | 318 | 160 | 12.5 | −0.04 (−0.11, 0.04) | 0.21 | 0.21 |
| Bangladesh | WASH−B (23) | CDI | | | | | | | | | | | | |
| Burkina Faso | iLiNS−Zinc (24) | DMC | | | | | | | | | | | | |
| Ghana | GHANA (25) | | | | | | | | | | | | | |
| Ghana | iLiNS−DYAD−G (26) | CDI | 192 | 351 | 7.4 | 0.03 (−0.02, 0.08) | 0.43 | 0.36 | 90 | 204 | 10.8 | −0.01 (−0.08, 0.07) | 0.19 | 0.19 |
| Haiti | HAITI (27) | Total sounds | | | | | | | | | | | | |
| Kenya | WASH−B (28) | EASQ | | | | | | | | | | | | |
| Madagascar | MAHAY (29) | ASQI | | | | | | | | | | | | |
| Malawi | iLiNS−DYAD−M (30) | CDI | 66 | 147 | 14.3 | −0.07 (−0.16, 0.03) | 0.11 | 0.15 | 138 | 275 | 9.1 | 0.00 (−0.06, 0.06) | 0.32 | 0.32 |
| Malawi | iLiNS−DOSE (31) | CDI | 224 | 94 | 9.6 | −0.02 (−0.08, 0.05) | 0.23 | 0.25 | 417 | 126 | 11.1 | 0.00 (−0.06, 0.06) | 0.28 | 0.28 |
| Mali | PROMIS CS (32) | DMC | | | | | | | | | | | | |
| Zimbabwe | SHINE (HIV−) (33) | CDI | | | | | | | | | | | | |
| Zimbabwe | SHINE (HIV+) (34) | CDI | | | | | | | | | | | | |
| | | | **694** | **688** | | **I² = 0.38, Tau² = 0.00** | | | **963** | **765** | | **I² = 0.00, Tau² = 0.00** | | |
| **Fixed** | | | | | | **−0.01 (−0.04, 0.02)** | | | | | | **−0.01 (−0.04, 0.02)** | | |
| **Random** | | | | | | **−0.01 (−0.05, 0.03)** | | | | | | **−0.01 (−0.04, 0.02)** | | |

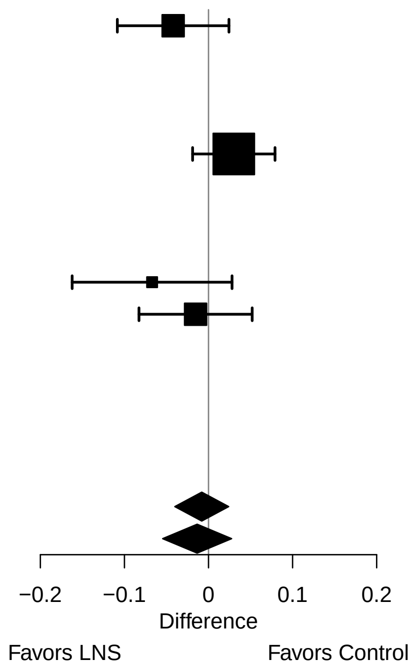


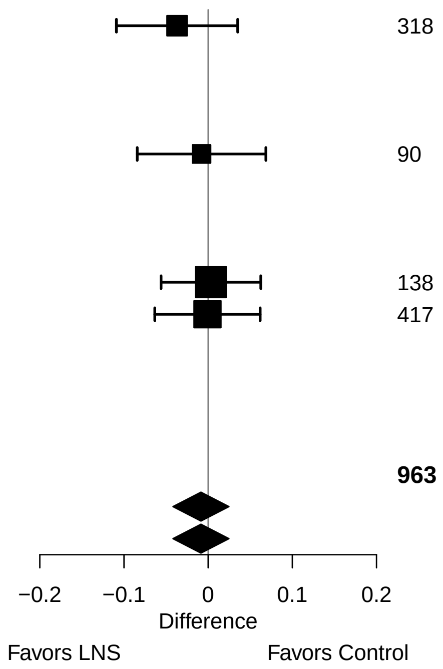

# Supplemental figure 7D: Mean difference in social-emotional z-score

## 7D1: Stratified by Maternal height

**P−for−interaction = 0.511**
**Difference in MDs = 0.03 (−0.05, 0.10)**

| | | | At least 150.1 cm | | | | | | Less than 150.1 cm | | | | | |
|---|---|---|---|---|---|---|---|---|---|---|---|---|---|---|
| **Country** | **Trial** | **Tool** | **LNS N** | **Control N** | **Control Mean** | **MD (95% CI)** | **Fixed W** | **Random W** | **LNS N** | **Control N** | **Control Mean** | **MD (95% CI)** | **Fixed W** | **Random W** |
| Bangladesh | JiVitA−4 (21) | | | | | | | | | | | | | |
| Bangladesh | RDNS (22) | DMC | 865 | 432 | 0.00 | 0.09 (−0.05, 0.24) | 0.07 | 0.09 | 732 | 358 | −0.08 | 0.04 (−0.06, 0.14) | 0.41 | 0.24 |
| Bangladesh | WASH−B (23) | EASQ | 567 | 1768 | 0.03 | 0.11 (0.01, 0.20) | 0.15 | 0.11 | 494 | 1458 | −0.10 | 0.14 (0.03, 0.24) | 0.36 | 0.24 |
| Burkina Faso | iLiNS−Zinc (24) | DMC | 727 | 365 | −0.23 | 0.36 (0.18, 0.53) | 0.04 | 0.08 | 17 | 10 | −0.30 | 0.32 (−0.34, 0.98) | 0.01 | 0.03 |
| Ghana | GHANA (25) | | | | | | | | | | | | | |
| Ghana | iLiNS−DYAD−G (26) | PSED | 311 | 611 | 0.00 | 0.00 (−0.14, 0.14) | 0.07 | 0.09 | 16 | 35 | −0.11 | −0.04 (−0.61, 0.54) | 0.01 | 0.04 |
| Haiti | HAITI (27) | | | | | | | | | | | | | |
| Kenya | WASH−B (28) | EASQ | 1237 | 4372 | 0.01 | −0.01 (−0.08, 0.05) | 0.33 | 0.13 | 51 | 175 | −0.15 | 0.49 (0.18, 0.80) | 0.04 | 0.09 |
| Madagascar | MAHAY (29) | ASQI | 956 | 986 | 0.03 | −0.05 (−0.24, 0.15) | 0.03 | 0.07 | 553 | 538 | 0.10 | −0.13 (−0.34, 0.07) | 0.09 | 0.15 |
| Malawi | iLiNS−DYAD−M (30) | PSED | 187 | 377 | 0.01 | −0.01 (−0.19, 0.16) | 0.04 | 0.08 | 28 | 58 | −0.14 | 0.22 (−0.22, 0.66) | 0.02 | 0.06 |
| Malawi | iLiNS−DOSE (31) | PSED | 541 | 185 | 0.03 | 0.01 (−0.16, 0.17) | 0.05 | 0.08 | 102 | 35 | −0.25 | 0.11 (−0.29, 0.51) | 0.02 | 0.07 |
| Mali | PROMIS CS (32) | DMC | 889 | 911 | −0.06 | 0.14 (−0.02, 0.31) | 0.05 | 0.08 | 32 | 27 | −0.27 | 0.39 (−0.15, 0.92) | 0.01 | 0.04 |
| Zimbabwe | SHINE (HIV−) (33) | MDAT | 788 | 762 | −0.07 | 0.13 (0.03, 0.24) | 0.12 | 0.11 | 34 | 22 | −0.04 | 0.10 (−0.47, 0.67) | 0.01 | 0.04 |
| Zimbabwe | SHINE (HIV+) (34) | MDAT | 158 | 140 | −0.15 | 0.32 (0.12, 0.51) | 0.04 | 0.07 | 10 | 6 | −0.30 | 0.70 (−0.18, 1.59) | 0.00 | 0.02 |
| | | | **7226** | **10909** | | **$I^2$ = 0.65, $Tau^2$ = 0.01** | | | **2069** | **2722** | | **$I^2$ = 0.40, $Tau^2$ = 0.01** | | |
| **Fixed** | | | | | | **0.07 (0.03, 0.10)** | | | | | | **0.09 (0.03, 0.16)** | | |
| **Random** | | | | | | **0.09 (0.02, 0.17)** | | | | | | **0.13 (0.01, 0.24)** | | |

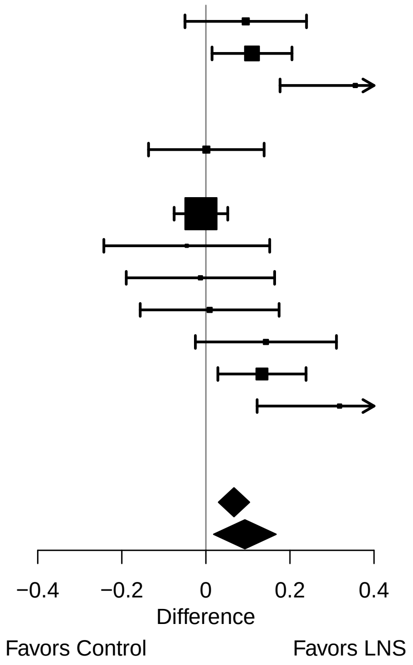


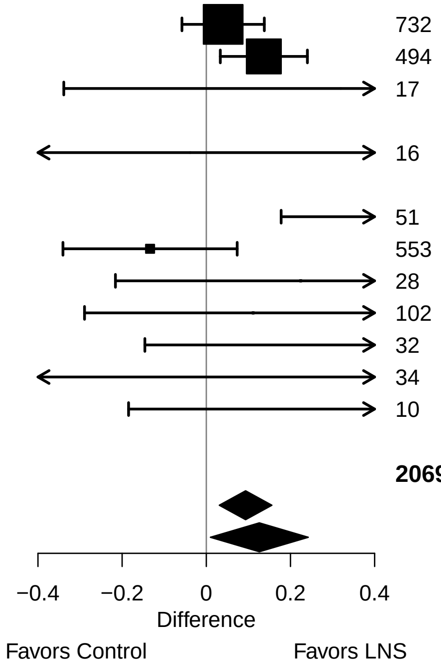

**Supplemental figure 7D: Mean difference in social-emotional z-score**

## 7D2: Stratified by Maternal BMI

**P−for−interaction = 0.885**
**Difference in MDs = −0.01 (−0.07, 0.06)**

| Country | Trial | Tool | At least 20 kg/m² LNS N | Control N | Control Mean | MD (95% CI) | Fixed W | Random W | Less than 20 kg/m² LNS N | Control N | Control Mean | MD (95% CI) | Fixed W | Random W |
|---|---|---|---|---|---|---|---|---|---|---|---|---|---|---|
| Bangladesh | JiVitA−4 (21) | | | | | | | | | | | | | |
| Bangladesh | RDNS (22) | DMC | 726 | 335 | 0.05 | 0.05 (−0.09, 0.18) | 0.09 | 0.11 | 871 | 455 | −0.11 | 0.08 (−0.04, 0.20) | 0.22 | 0.17 |
| Bangladesh | WASH−B (23) | EASQ | 479 | 1493 | 0.03 | 0.09 (−0.02, 0.19) | 0.17 | 0.14 | 582 | 1732 | −0.08 | 0.15 (0.04, 0.26) | 0.28 | 0.18 |
| Burkina Faso | iLiNS−Zinc (24) | DMC | 480 | 225 | −0.16 | 0.30 (0.11, 0.50) | 0.04 | 0.07 | 264 | 150 | −0.35 | 0.42 (0.16, 0.69) | 0.05 | 0.07 |
| Ghana | GHANA (25) | | | | | | | | | | | | | |
| Ghana | iLiNS−DYAD−G (26) | PSED | 292 | 530 | 0.01 | −0.01 (−0.15, 0.14) | 0.08 | 0.10 | 35 | 116 | −0.04 | 0.01 (−0.34, 0.37) | 0.03 | 0.05 |
| Haiti | HAITI (27) | | | | | | | | | | | | | |
| Kenya | WASH−B (28) | EASQ | 1031 | 3535 | 0.01 | 0.01 (−0.06, 0.08) | 0.32 | 0.16 | 257 | 1012 | −0.02 | −0.01 (−0.14, 0.12) | 0.19 | 0.16 |
| Madagascar | MAHAY (29) | ASQI | | | | | | | | | | | | |
| Malawi | iLiNS−DYAD−M (30) | PSED | 127 | 260 | −0.05 | 0.12 (−0.09, 0.34) | 0.04 | 0.06 | 87 | 175 | 0.05 | −0.16 (−0.41, 0.09) | 0.05 | 0.08 |
| Malawi | iLiNS−DOSE (31) | PSED | 478 | 158 | 0.05 | 0.01 (−0.17, 0.19) | 0.05 | 0.08 | 163 | 61 | −0.20 | 0.05 (−0.24, 0.34) | 0.04 | 0.07 |
| Mali | PROMIS CS (32) | DMC | 683 | 664 | −0.07 | 0.18 (0.00, 0.37) | 0.05 | 0.08 | 238 | 274 | −0.07 | 0.06 (−0.13, 0.25) | 0.09 | 0.11 |
| Zimbabwe | SHINE (HIV−) (33) | MDAT | 601 | 581 | −0.07 | 0.17 (0.06, 0.29) | 0.12 | 0.12 | 100 | 96 | −0.15 | 0.18 (−0.09, 0.44) | 0.05 | 0.07 |
| Zimbabwe | SHINE (HIV+) (34) | MDAT | 124 | 113 | −0.14 | 0.31 (0.09, 0.52) | 0.04 | 0.07 | 25 | 23 | −0.24 | 0.25 (−0.26, 0.76) | 0.01 | 0.03 |
| | | | **5021** | **7894** | | **I² = 0.52, Tau² = 0.01** | | | **2622** | **4094** | | **I² = 0.39, Tau² = 0.01** | | |
| **Fixed** | | | | | | **0.08 (0.04, 0.12)** | | | | | | **0.09 (0.03, 0.14)** | | |
| **Random** | | | | | | **0.11 (0.04, 0.17)** | | | | | | **0.09 (0.00, 0.17)** | | |

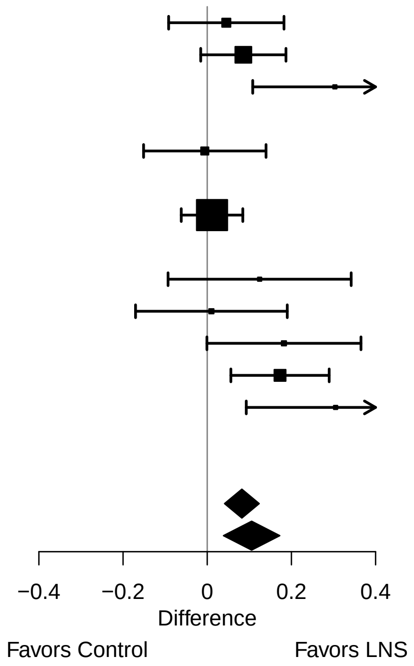


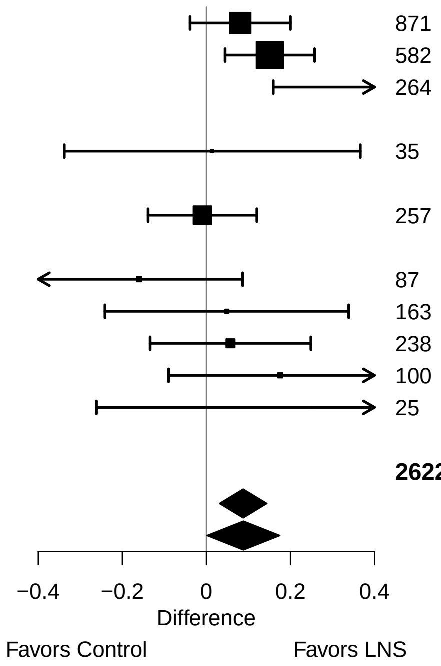

**Supplemental figure 7D: Mean difference in social-emotional z-score**

## 7D3: Stratified by Maternal age

**P−for−interaction = 0.666**
**Difference in MDs = 0.01 (−0.05, 0.07)**

| | | | At least 25 y | | | | | | Less than 25 y | | | | | |
|---|---|---|---|---|---|---|---|---|---|---|---|---|---|---|
| **Country** | **Trial** | **Tool** | **LNS N** | **Control N** | **Control Mean** | **MD (95% CI)** | **Fixed W** | **Random W** | **LNS N** | **Control N** | **Control Mean** | **MD (95% CI)** | **Fixed W** | **Random W** |
| Bangladesh | JiVitA−4 (21) | | | | | | | | | | | | | |
| Bangladesh | RDNS (22) | DMC | 450 | 221 | −0.08 | 0.04 (−0.17, 0.25) | 0.05 | 0.08 | 1207 | 594 | −0.01 | 0.07 (−0.03, 0.17) | 0.21 | 0.16 |
| Bangladesh | WASH−B (23) | EASQ | 458 | 1442 | −0.01 | −0.02 (−0.13, 0.09) | 0.19 | 0.11 | 603 | 1809 | −0.04 | 0.23 (0.13, 0.32) | 0.21 | 0.17 |
| Burkina Faso | iLiNS−Zinc (24) | DMC | 421 | 232 | −0.30 | 0.43 (0.23, 0.62) | 0.05 | 0.08 | 321 | 141 | −0.12 | 0.23 (0.04, 0.42) | 0.06 | 0.08 |
| Ghana | GHANA (25) | | | | | | | | | | | | | |
| Ghana | iLiNS−DYAD−G (26) | PSED | 212 | 404 | 0.00 | 0.04 (−0.13, 0.21) | 0.07 | 0.09 | 120 | 253 | −0.01 | −0.05 (−0.25, 0.16) | 0.05 | 0.07 |
| Haiti | HAITI (27) | | | | | | | | | | | | | |
| Kenya | WASH−B (28) | EASQ | 798 | 2622 | 0.02 | −0.02 (−0.10, 0.07) | 0.29 | 0.12 | 549 | 2092 | −0.03 | 0.06 (−0.04, 0.15) | 0.22 | 0.17 |
| Madagascar | MAHAY (29) | ASQI | 852 | 901 | 0.03 | −0.05 (−0.23, 0.14) | 0.06 | 0.09 | 759 | 703 | 0.05 | −0.10 (−0.31, 0.11) | 0.05 | 0.07 |
| Malawi | iLiNS−DYAD−M (30) | PSED | 103 | 224 | −0.03 | 0.04 (−0.19, 0.27) | 0.04 | 0.08 | 112 | 214 | 0.02 | −0.01 (−0.25, 0.22) | 0.04 | 0.06 |
| Malawi | iLiNS−DOSE (31) | PSED | 346 | 124 | −0.05 | 0.06 (−0.15, 0.27) | 0.05 | 0.08 | 286 | 90 | 0.00 | 0.00 (−0.23, 0.24) | 0.04 | 0.06 |
| Mali | PROMIS CS (32) | DMC | 492 | 492 | −0.07 | 0.17 (−0.02, 0.36) | 0.06 | 0.09 | 435 | 452 | −0.07 | 0.12 (−0.07, 0.31) | 0.05 | 0.08 |
| Zimbabwe | SHINE (HIV−) (33) | MDAT | 427 | 389 | −0.06 | 0.17 (0.03, 0.32) | 0.09 | 0.10 | 326 | 321 | −0.10 | 0.11 (−0.05, 0.28) | 0.07 | 0.09 |
| Zimbabwe | SHINE (HIV+) (34) | MDAT | 126 | 111 | −0.25 | 0.43 (0.22, 0.64) | 0.05 | 0.08 | 29 | 26 | −0.01 | 0.03 (−0.58, 0.65) | 0.01 | 0.01 |
| | | | **4685** | **7162** | | **I² = 0.72, Tau² = 0.02** | | | **4747** | **6695** | | **I² = 0.41, Tau² = 0.00** | | |
| **Fixed** | | | | | | **0.07 (0.02, 0.11)** | | | | | | **0.10 (0.05, 0.14)** | | |
| **Random** | | | | | | **0.11 (0.01, 0.21)** | | | | | | **0.09 (0.02, 0.15)** | | |

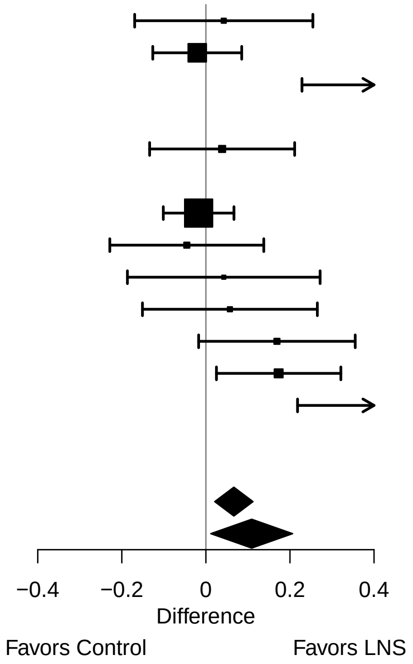


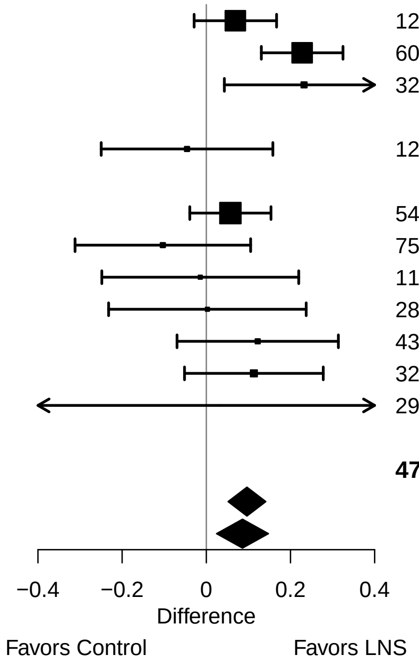

### Supplemental figure 7D: Mean difference in social-emotional z-score

## 7D4: Stratified by Maternal education

**P−for−interaction = 0.700**
**Difference in MDs = −0.01 (−0.08, 0.05)**

| Country | Trial | Tool | LNS N | Control N | Control Mean | Primary or greater MD (95% CI) | Fixed W | Random W | LNS N | Control N | Control Mean | Incomplete or no formal MD (95% CI) | Fixed W | Random W |
|---|---|---|---|---|---|---|---|---|---|---|---|---|---|---|
| Bangladesh | JiVitA−4 (21) | | | | | | | | | | | | | |
| Bangladesh | RDNS (22) | DMC | 1241 | 589 | 0.05 | 0.06 (−0.05, 0.16) | 0.15 | 0.12 | 416 | 226 | −0.25 | 0.04 (−0.12, 0.21) | 0.09 | 0.11 |
| Bangladesh | WASH−B (23) | EASQ | 750 | 2339 | 0.03 | 0.17 (0.08, 0.25) | 0.25 | 0.13 | 316 | 927 | −0.18 | 0.03 (−0.09, 0.15) | 0.16 | 0.14 |
| Burkina Faso | iLiNS−Zinc (24) | DMC | 32 | 9 | −0.42 | 0.52 (−0.09, 1.13) | 0.00 | 0.03 | 712 | 366 | −0.23 | 0.36 (0.18, 0.53) | 0.08 | 0.10 |
| Ghana | GHANA (25) | | | | | | | | | | | | | |
| Ghana | iLiNS−DYAD−G (26) | PSED | 257 | 518 | −0.01 | −0.03 (−0.17, 0.12) | 0.08 | 0.11 | 75 | 139 | 0.02 | 0.13 (−0.18, 0.43) | 0.03 | 0.05 |
| Haiti | HAITI (27) | | | | | | | | | | | | | |
| Kenya | WASH−B (28) | EASQ | 658 | 2258 | 0.10 | 0.04 (−0.04, 0.12) | 0.25 | 0.13 | 702 | 2484 | −0.09 | −0.02 (−0.10, 0.07) | 0.34 | 0.18 |
| Madagascar | MAHAY (29) | ASQI | 339 | 416 | 0.32 | −0.28 (−0.50, −0.07) | 0.04 | 0.09 | 1274 | 1188 | −0.06 | 0.01 (−0.18, 0.19) | 0.07 | 0.09 |
| Malawi | iLiNS−DYAD−M (30) | PSED | 33 | 69 | 0.01 | 0.24 (−0.16, 0.65) | 0.01 | 0.05 | 181 | 366 | −0.01 | −0.03 (−0.21, 0.15) | 0.07 | 0.10 |
| Malawi | iLiNS−DOSE (31) | PSED | 148 | 52 | −0.02 | −0.06 (−0.38, 0.26) | 0.02 | 0.06 | 485 | 162 | −0.03 | 0.06 (−0.12, 0.24) | 0.07 | 0.10 |
| Mali | PROMIS CS (32) | DMC | 100 | 94 | 0.17 | −0.03 (−0.37, 0.30) | 0.02 | 0.06 | 826 | 850 | −0.10 | 0.17 (0.00, 0.33) | 0.09 | 0.11 |
| Zimbabwe | SHINE (HIV−) (33) | MDAT | 763 | 731 | −0.08 | 0.15 (0.04, 0.26) | 0.15 | 0.12 | 29 | 25 | 0.18 | −0.24 (−0.78, 0.30) | 0.01 | 0.02 |
| Zimbabwe | SHINE (HIV+) (34) | MDAT | 149 | 130 | −0.19 | 0.37 (0.16, 0.57) | 0.04 | 0.09 | 9 | 8 | −0.22 | 0.11 (−0.82, 1.04) | 0.00 | 0.01 |
| | | | **4470** | **7205** | | **I² = 0.68, Tau² = 0.02** | | | **5025** | **6741** | | **I² = 0.47, Tau² = 0.01** | | |
| **Fixed** | | | | | | **0.09 (0.05, 0.13)** | | | | | | **0.05 (0.00, 0.10)** | | |
| **Random** | | | | | | **0.08 (−0.03, 0.19)** | | | | | | **0.07 (−0.01, 0.14)** | | |

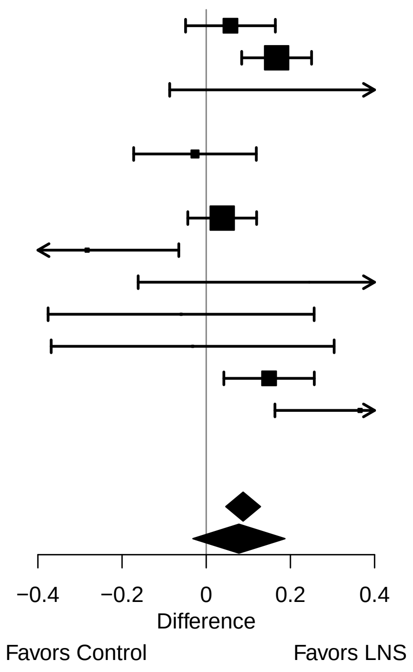


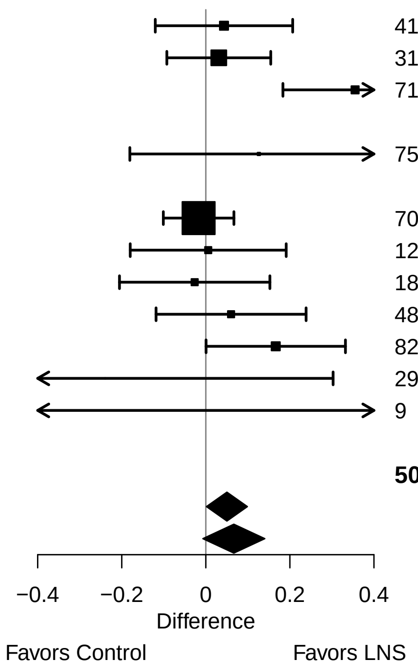

## Supplemental figure 7D: Mean difference in social-emotional z-score

### 7D5: Stratified by Maternal depressive symptoms

**P−for−interaction = 0.335**
**Difference in MDs = 0.04 (−0.04, 0.11)**

| | | | | | | **Less than 75th percentile** | | | | | | **At least 75th percentile** | | |
|---|---|---|---|---|---|---|---|---|---|---|---|---|---|---|
| **Country** | **Trial** | **Tool** | **LNS N** | **Control N** | **Control Mean** | **MD (95% CI)** | **Fixed W** | **Random W** | **LNS N** | **Control N** | **Control Mean** | **MD (95% CI)** | **Fixed W** | **Random W** |
| Bangladesh | JiVitA−4 (21) | | | | | | | | | | | | | |
| Bangladesh | RDNS (22) | DMC | 1075 | 472 | 0.03 | 0.05 (−0.06, 0.16) | 0.15 | 0.15 | 520 | 288 | −0.11 | 0.05 (−0.12, 0.22) | 0.16 | 0.16 |
| Bangladesh | WASH−B (23) | EASQ | 822 | 2356 | −0.02 | 0.13 (0.04, 0.21) | 0.24 | 0.17 | 223 | 830 | −0.08 | 0.10 (−0.05, 0.24) | 0.22 | 0.22 |
| Burkina Faso | iLiNS−Zinc (24) | DMC | | | | | | | | | | | | |
| Ghana | GHANA (25) | | | | | | | | | | | | | |
| Ghana | iLiNS−DYAD−G (26) | PSED | 242 | 435 | 0.00 | 0.08 (−0.08, 0.24) | 0.07 | 0.11 | 78 | 201 | −0.05 | −0.14 (−0.40, 0.11) | 0.07 | 0.07 |
| Haiti | HAITI (27) | | | | | | | | | | | | | |
| Kenya | WASH−B (28) | EASQ | 960 | 3317 | 0.00 | −0.01 (−0.08, 0.07) | 0.31 | 0.17 | 317 | 1156 | 0.14 | 0.05 (−0.08, 0.17) | 0.30 | 0.30 |
| Madagascar | MAHAY (29) | ASQI | 615 | 667 | −0.02 | −0.02 (−0.25, 0.22) | 0.03 | 0.08 | 268 | 242 | 0.08 | −0.12 (−0.37, 0.13) | 0.07 | 0.07 |
| Malawi | iLiNS−DYAD−M (30) | PSED | 153 | 302 | 0.09 | −0.01 (−0.20, 0.18) | 0.05 | 0.09 | 47 | 111 | −0.23 | 0.05 (−0.28, 0.38) | 0.04 | 0.04 |
| Malawi | iLiNS−DOSE (31) | PSED | | | | | | | | | | | | |
| Mali | PROMIS CS (32) | DMC | | | | | | | | | | | | |
| Zimbabwe | SHINE (HIV−) (33) | MDAT | 583 | 560 | −0.07 | 0.19 (0.08, 0.30) | 0.13 | 0.15 | 197 | 180 | −0.08 | 0.01 (−0.20, 0.22) | 0.11 | 0.11 |
| Zimbabwe | SHINE (HIV+) (34) | MDAT | 131 | 110 | −0.17 | 0.38 (0.15, 0.62) | 0.03 | 0.08 | 33 | 32 | −0.06 | 0.01 (−0.49, 0.51) | 0.02 | 0.02 |
| | | | **4581** | **8219** | | **I² = 0.63, Tau² = 0.01** | | | **1683** | **3040** | | **I² = 0.00, Tau² = 0.00** | | |
| **Fixed** | | | | | | **0.08 (0.04, 0.12)** | | | | | | **0.03 (−0.04, 0.10)** | | |
| **Random** | | | | | | **0.09 (0.01, 0.17)** | | | | | | **0.03 (−0.04, 0.10)** | | |

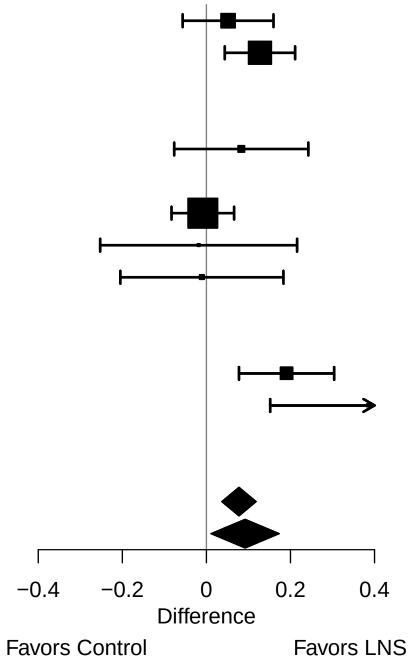


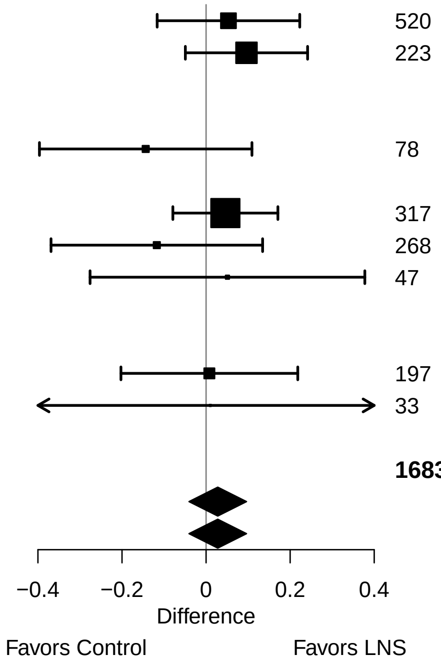

### Supplemental figure 7D: Mean difference in social-emotional z-score

## 7D6: Stratified by Child sex

**P−for−interaction = 0.706**
**Difference in MDs = −0.01 (−0.06, 0.04)**

| Country | Trial | Tool | LNS N | Control N | Control Mean | Male MD (95% CI) | Fixed W | Random W | LNS N | Control N | Control Mean | Female MD (95% CI) | Fixed W | Random W |
|---|---|---|---|---|---|---|---|---|---|---|---|---|---|---|
| Bangladesh | JiVitA−4 (21) | | | | | | | | | | | | | |
| Bangladesh | RDNS (22) | DMC | 830 | 411 | 0.02 | −0.02 (−0.12, 0.08) | 0.18 | 0.12 | 827 | 404 | −0.08 | 0.15 (0.01, 0.29) | 0.10 | 0.11 |
| Bangladesh | WASH−B (23) | EASQ | 522 | 1635 | −0.04 | 0.16 (0.05, 0.27) | 0.15 | 0.12 | 544 | 1631 | −0.02 | 0.09 (−0.01, 0.19) | 0.22 | 0.12 |
| Burkina Faso | iLiNS−Zinc (24) | DMC | 372 | 193 | −0.22 | 0.34 (0.20, 0.48) | 0.09 | 0.10 | 374 | 182 | −0.25 | 0.38 (0.12, 0.63) | 0.03 | 0.07 |
| Ghana | GHANA (25) | | | | | | | | | | | | | |
| Ghana | iLiNS−DYAD−G (26) | PSED | 164 | 306 | 0.01 | −0.04 (−0.22, 0.15) | 0.05 | 0.08 | 168 | 351 | −0.02 | 0.05 (−0.14, 0.24) | 0.06 | 0.09 |
| Haiti | HAITI (27) | | | | | | | | | | | | | |
| Kenya | WASH−B (28) | EASQ | 682 | 2260 | 0.00 | 0.00 (−0.08, 0.09) | 0.23 | 0.13 | 680 | 2485 | 0.00 | 0.02 (−0.06, 0.11) | 0.28 | 0.13 |
| Madagascar | MAHAY (29) | ASQI | 795 | 787 | 0.00 | 0.00 (−0.19, 0.19) | 0.05 | 0.08 | 818 | 817 | 0.07 | −0.14 (−0.34, 0.05) | 0.05 | 0.09 |
| Malawi | iLiNS−DYAD−M (30) | PSED | 105 | 205 | −0.02 | 0.07 (−0.17, 0.31) | 0.03 | 0.06 | 110 | 233 | 0.01 | −0.04 (−0.26, 0.19) | 0.04 | 0.08 |
| Malawi | iLiNS−DOSE (31) | PSED | 320 | 117 | 0.05 | −0.07 (−0.29, 0.15) | 0.04 | 0.07 | 324 | 104 | −0.11 | 0.15 (−0.07, 0.36) | 0.04 | 0.08 |
| Mali | PROMIS CS (32) | DMC | 469 | 514 | −0.08 | 0.17 (0.00, 0.33) | 0.07 | 0.09 | 458 | 430 | −0.07 | 0.13 (−0.08, 0.33) | 0.05 | 0.08 |
| Zimbabwe | SHINE (HIV−) (33) | MDAT | 424 | 388 | −0.10 | 0.20 (0.05, 0.34) | 0.09 | 0.10 | 417 | 411 | −0.02 | 0.05 (−0.09, 0.19) | 0.11 | 0.11 |
| Zimbabwe | SHINE (HIV+) (34) | MDAT | 84 | 79 | −0.12 | 0.24 (−0.04, 0.51) | 0.02 | 0.05 | 86 | 72 | −0.26 | 0.48 (0.16, 0.80) | 0.02 | 0.05 |
| | | | **4767** | **6895** | | **I² = 0.68, Tau² = 0.01** | | | **4806** | **7120** | | **I² = 0.52, Tau² = 0.01** | | |
| **Fixed** | | | | | | **0.08 (0.04, 0.13)** | | | | | | **0.07 (0.03, 0.12)** | | |
| **Random** | | | | | | **0.09 (0.02, 0.17)** | | | | | | **0.10 (0.01, 0.19)** | | |

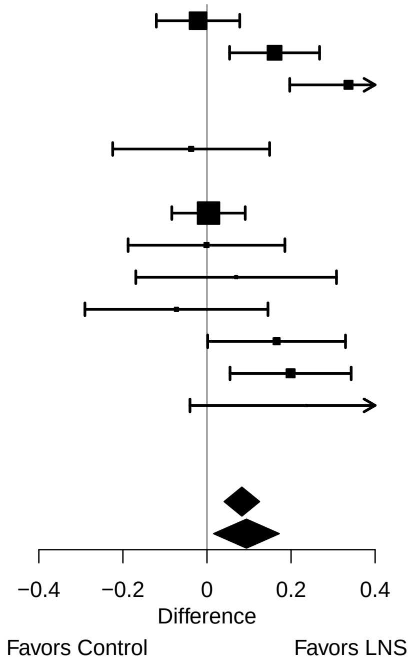


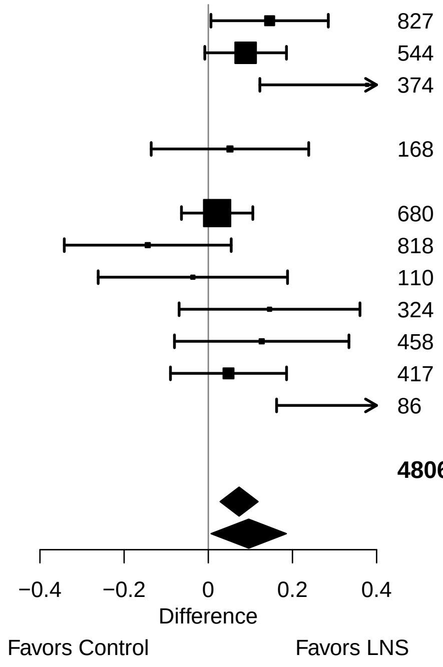

## Supplemental figure 7D: Mean difference in social-emotional z-score

### 7D7: Stratified by Child birth order

**P−for−interaction = 0.979**
**Difference in MDs = 0.00 (−0.06, 0.07)**

| Country | Trial | Tool | LNS N | Control N | Control Mean | Later born MD (95% CI) | Fixed W | Random W | LNS N | Control N | Control Mean | Firstborn MD (95% CI) | Fixed W | Random W |
|---|---|---|---|---|---|---|---|---|---|---|---|---|---|---|
| Bangladesh | JiVitA−4 (21) | | | | | | | | | | | | | |
| Bangladesh | RDNS (22) | DMC | 969 | 508 | −0.05 | 0.08 (−0.04, 0.20) | 0.10 | 0.10 | 687 | 307 | 0.00 | 0.03 (−0.10, 0.16) | 0.20 | 0.16 |
| Bangladesh | WASH−B (23) | EASQ | 669 | 2134 | −0.07 | 0.08 (0.00, 0.17) | 0.20 | 0.12 | 377 | 1031 | 0.07 | 0.14 (0.02, 0.26) | 0.24 | 0.17 |
| Burkina Faso | iLiNS−Zinc (24) | DMC | 587 | 290 | −0.27 | 0.42 (0.25, 0.60) | 0.05 | 0.08 | 159 | 85 | −0.12 | 0.11 (−0.15, 0.38) | 0.05 | 0.07 |
| Ghana | GHANA (25) | | | | | | | | | | | | | |
| Ghana | iLiNS−DYAD−G (26) | PSED | 225 | 444 | 0.02 | −0.05 (−0.21, 0.12) | 0.05 | 0.09 | 107 | 213 | −0.06 | 0.12 (−0.10, 0.34) | 0.07 | 0.09 |
| Haiti | HAITI (27) | | | | | | | | | | | | | |
| Kenya | WASH−B (28) | EASQ | 1071 | 3767 | 0.00 | −0.01 (−0.08, 0.06) | 0.30 | 0.12 | 289 | 974 | −0.01 | 0.08 (−0.06, 0.21) | 0.18 | 0.15 |
| Madagascar | MAHAY (29) | ASQI | 1162 | 1118 | 0.00 | −0.04 (−0.22, 0.15) | 0.04 | 0.08 | 433 | 465 | 0.09 | −0.14 (−0.38, 0.11) | 0.06 | 0.08 |
| Malawi | iLiNS−DYAD−M (30) | PSED | 170 | 349 | 0.01 | 0.07 (−0.11, 0.25) | 0.04 | 0.08 | 45 | 87 | −0.07 | −0.18 (−0.53, 0.18) | 0.03 | 0.05 |
| Malawi | iLiNS−DOSE (31) | PSED | 425 | 151 | −0.02 | 0.04 (−0.14, 0.22) | 0.04 | 0.08 | 133 | 41 | 0.05 | −0.02 (−0.39, 0.34) | 0.03 | 0.04 |
| Mali | PROMIS CS (32) | DMC | 770 | 811 | −0.07 | 0.16 (−0.01, 0.34) | 0.05 | 0.08 | 142 | 122 | −0.05 | 0.08 (−0.25, 0.40) | 0.03 | 0.05 |
| Zimbabwe | SHINE (HIV−) (33) | MDAT | 602 | 574 | −0.02 | 0.10 (−0.03, 0.22) | 0.10 | 0.10 | 203 | 191 | −0.20 | 0.19 (0.02, 0.37) | 0.11 | 0.12 |
| Zimbabwe | SHINE (HIV+) (34) | MDAT | 149 | 118 | −0.14 | 0.29 (0.08, 0.49) | 0.04 | 0.07 | 18 | 29 | −0.29 | 0.73 (0.16, 1.31) | 0.01 | 0.02 |
| | | | **6799** | **10264** | | **I² = 0.65, Tau² = 0.01** | | | **2593** | **3545** | | **I² = 0.26, Tau² = 0.01** | | |
| **Fixed** | | | | | | **0.07 (0.03, 0.11)** | | | | | | **0.09 (0.03, 0.14)** | | |
| **Random** | | | | | | **0.10 (0.02, 0.18)** | | | | | | **0.08 (0.00, 0.16)** | | |

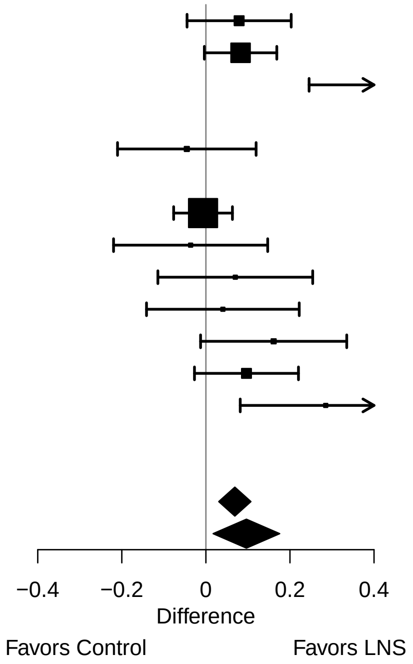


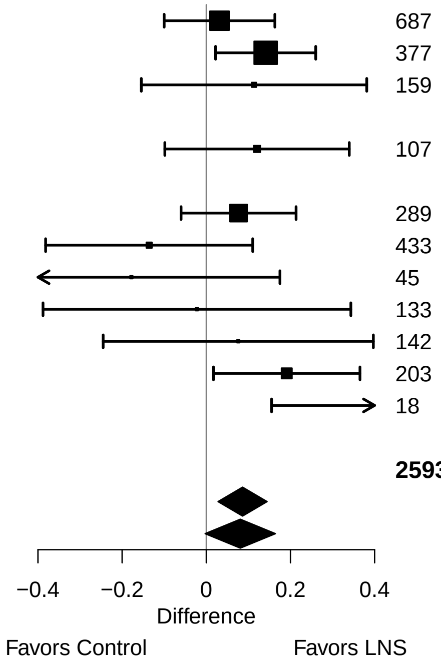

**Supplemental figure 7D: Mean difference in social-emotional z-score**

## 7D8: Stratified by Child baseline stunting

**P−for−interaction = 0.495**
**Difference in MDs = 0.04 (−0.08, 0.16)**

| | | | No | | | | | | Yes | | | | | |
|---|---|---|---|---|---|---|---|---|---|---|---|---|---|---|
| **Country** | **Trial** | **Tool** | **LNS N** | **Control N** | **Control Mean** | **MD (95% CI)** | **Fixed W** | **Random W** | **LNS N** | **Control N** | **Control Mean** | **MD (95% CI)** | **Fixed W** | **Random W** |
| Bangladesh | JiVitA−4 (21) | | | | | | | | | | | | | |
| Bangladesh | RDNS (22) | DMC | 1241 | 574 | 0.06 | 0.01 (−0.08, 0.10) | 0.36 | 0.19 | 354 | 185 | −0.29 | 0.18 (−0.03, 0.40) | 0.25 | 0.24 |
| Bangladesh | WASH−B (23) | EASQ | | | | | | | | | | | | |
| Burkina Faso | iLiNS−Zinc (24) | DMC | 576 | 294 | −0.16 | 0.35 (0.19, 0.52) | 0.11 | 0.14 | 170 | 80 | −0.50 | 0.37 (0.06, 0.68) | 0.12 | 0.13 |
| Ghana | GHANA (25) | | | | | | | | | | | | | |
| Ghana | iLiNS−DYAD−G (26) | PSED | 280 | 547 | 0.00 | 0.00 (−0.14, 0.15) | 0.14 | 0.16 | 28 | 63 | 0.02 | 0.09 (−0.32, 0.51) | 0.07 | 0.07 |
| Haiti | HAITI (27) | | | | | | | | | | | | | |
| Kenya | WASH−B (28) | EASQ | | | | | | | | | | | | |
| Madagascar | MAHAY (29) | ASQI | | | | | | | | | | | | |
| Malawi | iLiNS−DYAD−M (30) | PSED | 153 | 323 | 0.03 | 0.01 (−0.19, 0.20) | 0.08 | 0.13 | 48 | 96 | −0.06 | 0.02 (−0.31, 0.35) | 0.11 | 0.11 |
| Malawi | iLiNS−DOSE (31) | PSED | 464 | 147 | −0.04 | 0.07 (−0.11, 0.26) | 0.09 | 0.13 | 180 | 74 | 0.02 | −0.06 (−0.33, 0.21) | 0.17 | 0.17 |
| Mali | PROMIS CS (32) | DMC | | | | | | | | | | | | |
| Zimbabwe | SHINE (HIV−) (33) | MDAT | 532 | 490 | −0.02 | 0.15 (0.02, 0.28) | 0.17 | 0.16 | 122 | 105 | −0.26 | 0.12 (−0.11, 0.35) | 0.22 | 0.21 |
| Zimbabwe | SHINE (HIV+) (34) | MDAT | 115 | 89 | −0.18 | 0.35 (0.08, 0.62) | 0.04 | 0.09 | 34 | 32 | −0.34 | 0.42 (−0.02, 0.86) | 0.06 | 0.07 |
| | | | **3361** | **2464** | | **I² = 0.68, Tau² = 0.02** | | | **936** | **635** | | **I² = 0.08, Tau² = 0.00** | | |
| **Fixed** | | | | | | **0.09 (0.04, 0.15)** | | | | | | **0.14 (0.03, 0.25)** | | |
| **Random** | | | | | | **0.12 (0.01, 0.23)** | | | | | | **0.14 (0.03, 0.26)** | | |

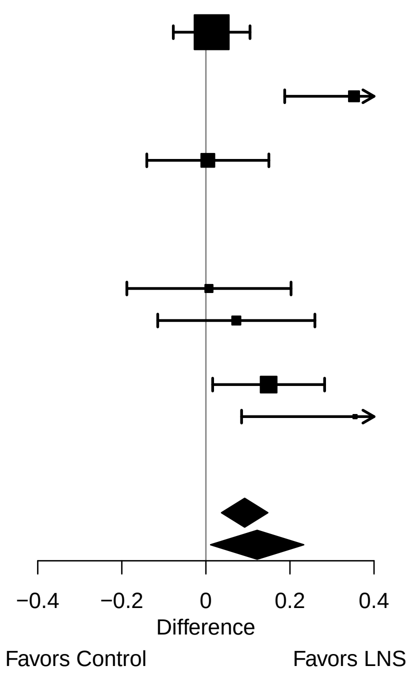


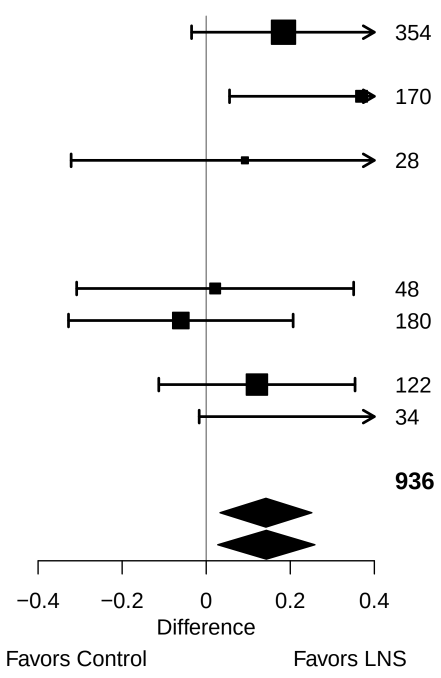

**Supplemental figure 7D: Mean difference in social-emotional z-score**

## 7D9: Stratified by Child baseline acute malnutrition

**P−for−interaction = 0.349**
**Difference in MDs = 0.08 (−0.08, 0.24)**

| Country | Trial | Tool | No: LNS N | No: Control N | No: Control Mean | No: MD (95% CI) | No: Fixed W | No: Random W | Yes: LNS N | Yes: Control N | Yes: Control Mean | Yes: MD (95% CI) | Yes: Fixed W | Yes: Random W |
|---|---|---|---|---|---|---|---|---|---|---|---|---|---|---|
| Bangladesh | JiVitA−4 (21) | | | | | | | | | | | | | |
| Bangladesh | RDNS (22) | DMC | 1471 | 707 | 0.00 | 0.06 (−0.04, 0.16) | 0.30 | 0.17 | 124 | 53 | −0.38 | 0.12 (−0.38, 0.62) | 0.10 | 0.14 |
| Bangladesh | WASH−B (23) | EASQ | | | | | | | | | | | | |
| Burkina Faso | iLiNS−Zinc (24) | DMC | 556 | 266 | −0.15 | 0.31 (0.11, 0.51) | 0.07 | 0.13 | 190 | 109 | −0.44 | 0.46 (0.24, 0.68) | 0.51 | 0.30 |
| Ghana | GHANA (25) | | | | | | | | | | | | | |
| Ghana | iLiNS−DYAD−G (26) | PSED | 290 | 554 | 0.01 | −0.01 (−0.15, 0.13) | 0.15 | 0.15 | 19 | 56 | −0.10 | 0.26 (−0.26, 0.78) | 0.09 | 0.13 |
| Haiti | HAITI (27) | | | | | | | | | | | | | |
| Kenya | WASH−B (28) | EASQ | | | | | | | | | | | | |
| Madagascar | MAHAY (29) | ASQI | | | | | | | | | | | | |
| Malawi | iLiNS−DYAD−M (30) | PSED | 187 | 386 | 0.01 | 0.03 (−0.14, 0.21) | 0.10 | 0.14 | 14 | 36 | 0.02 | −0.29 (−0.90, 0.31) | 0.07 | 0.10 |
| Malawi | iLiNS−DOSE (31) | PSED | 607 | 206 | −0.03 | 0.04 (−0.12, 0.20) | 0.12 | 0.14 | 37 | 15 | 0.13 | −0.10 (−0.69, 0.49) | 0.07 | 0.11 |
| Mali | PROMIS CS (32) | DMC | | | | | | | | | | | | |
| Zimbabwe | SHINE (HIV−) (33) | MDAT | 620 | 566 | −0.05 | 0.13 (0.01, 0.25) | 0.20 | 0.16 | 36 | 34 | −0.18 | 0.39 (−0.06, 0.84) | 0.12 | 0.16 |
| Zimbabwe | SHINE (HIV+) (34) | MDAT | 137 | 114 | −0.27 | 0.46 (0.24, 0.68) | 0.06 | 0.12 | 14 | 8 | 0.28 | −0.32 (−1.09, 0.46) | 0.04 | 0.07 |
| | | | **3868** | **2799** | | **I² = 0.68, Tau² = 0.02** | | | **434** | **311** | | **I² = 0.42, Tau² = 0.03** | | |
| **Fixed** | | | | | | **0.10 (0.05, 0.16)** | | | | | | **0.28 (0.12, 0.43)** | | |
| **Random** | | | | | | **0.13 (0.01, 0.25)** | | | | | | **0.18 (−0.04, 0.41)** | | |

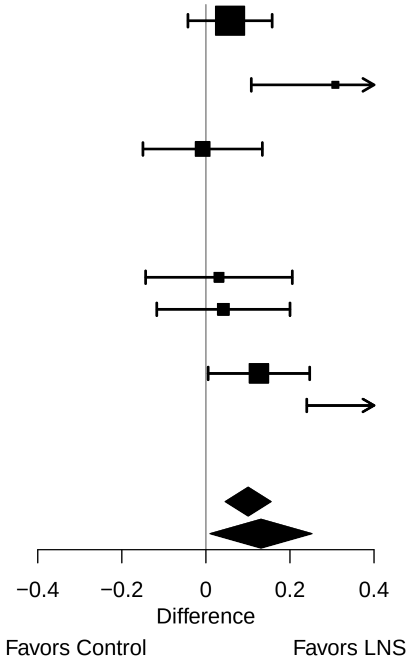


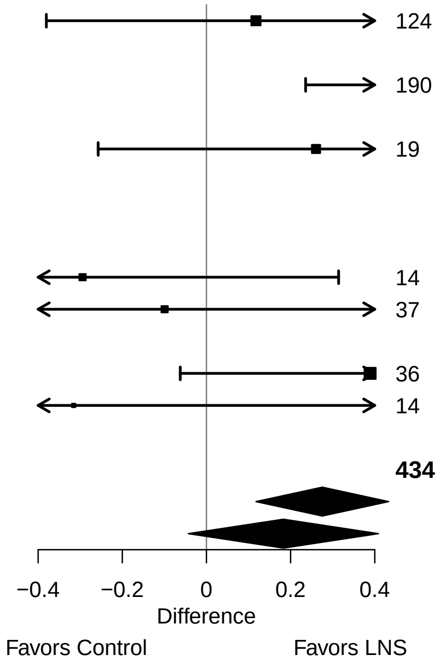

## Supplemental figure 7D: Mean difference in social-emotional z-score

## 7D10: Stratified by Child baseline anemia

**P−for−interaction = 0.834**
**Difference in MDs = −0.02 (−0.17, 0.14)**

| Country | Trial | Tool | Not anemic: LNS N | Not anemic: Control N | Not anemic: Control Mean | Not anemic: MD (95% CI) | Not anemic: Fixed W | Not anemic: Random W | Anemic: LNS N | Anemic: Control N | Anemic: Control Mean | Anemic: MD (95% CI) | Anemic: Fixed W | Anemic: Random W |
|---|---|---|---|---|---|---|---|---|---|---|---|---|---|---|
| Bangladesh | JiVitA−4 (21) | | | | | | | | | | | | | |
| Bangladesh | RDNS (22) | DMC | 212 | 96 | −0.11 | 0.15 (−0.11, 0.42) | 0.18 | 0.20 | 316 | 160 | −0.10 | 0.18 (−0.03, 0.39) | 0.19 | 0.20 |
| Bangladesh | WASH−B (23) | EASQ | | | | | | | | | | | | |
| Burkina Faso | iLiNS−Zinc (24) | DMC | 59 | 34 | −0.26 | 0.50 (0.13, 0.87) | 0.09 | 0.13 | 687 | 341 | −0.23 | 0.34 (0.15, 0.54) | 0.23 | 0.21 |
| Ghana | GHANA (25) | | | | | | | | | | | | | |
| Ghana | iLiNS−DYAD−G (26) | PSED | 193 | 350 | 0.00 | 0.03 (−0.14, 0.21) | 0.38 | 0.28 | 90 | 204 | 0.02 | −0.08 (−0.32, 0.16) | 0.15 | 0.17 |
| Haiti | HAITI (27) | | | | | | | | | | | | | |
| Kenya | WASH−B (28) | EASQ | | | | | | | | | | | | |
| Madagascar | MAHAY (29) | ASQI | | | | | | | | | | | | |
| Malawi | iLiNS−DYAD−M (30) | PSED | 66 | 147 | 0.05 | 0.02 (−0.27, 0.30) | 0.15 | 0.18 | 138 | 275 | −0.04 | 0.01 (−0.19, 0.22) | 0.21 | 0.20 |
| Malawi | iLiNS−DOSE (31) | PSED | 223 | 94 | 0.05 | −0.02 (−0.26, 0.22) | 0.21 | 0.22 | 417 | 126 | −0.08 | 0.08 (−0.12, 0.28) | 0.22 | 0.21 |
| Mali | PROMIS CS (32) | DMC | | | | | | | | | | | | |
| Zimbabwe | SHINE (HIV−) (33) | MDAT | | | | | | | | | | | | |
| Zimbabwe | SHINE (HIV+) (34) | MDAT | | | | | | | | | | | | |
| | | | **753** | **721** | | **I² = 0.37, Tau² = 0.02** | | | **1648** | **1106** | | **I² = 0.56, Tau² = 0.01** | | |
| **Fixed** | | | | | | **0.08 (−0.03, 0.19)** | | | | | | **0.12 (0.03, 0.22)** | | |
| **Random** | | | | | | **0.10 (−0.06, 0.26)** | | | | | | **0.12 (−0.03, 0.26)** | | |

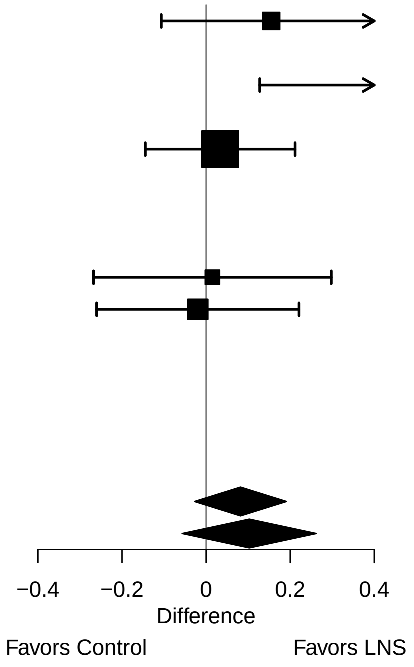


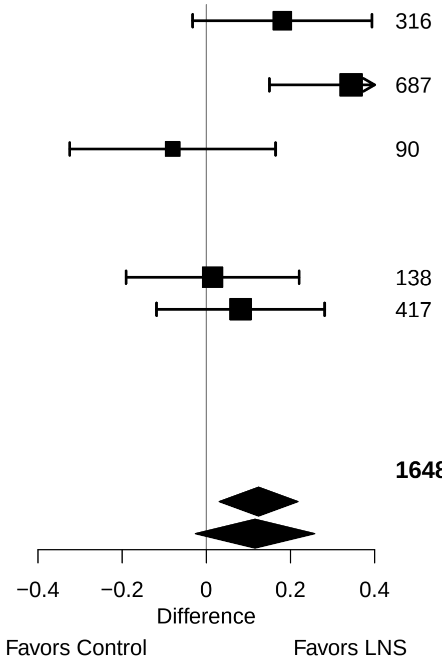

# Supplemental figure 7E: Social-emotional lowest decile prevalence ratio

## 7E1: Stratified by Maternal height

**P−for−interaction = 0.638**
**Ratio of PRs = 1.06 (0.82, 1.38)**

| Country | Trial | Tool | At least 150.1 cm: LNS N | Control N | Control Prevalence | PR (95% CI) | Fixed W | Random W | Less than 150.1 cm: LNS N | Control N | Control Prevalence | PR (95% CI) | Fixed W | Random W |
|---|---|---|---|---|---|---|---|---|---|---|---|---|---|---|
| Bangladesh | JiVitA−4 (21) | | | | | | | | | | | | | |
| Bangladesh | RDNS (22) | DMC | 865 | 432 | 10.6 | 0.76 (0.53, 1.08) | 0.36 | 0.36 | 732 | 358 | 12.3 | 0.92 (0.68, 1.25) | 0.43 | 0.43 |
| Bangladesh | WASH−B (23) | EASQ | 567 | 1768 | 8.9 | 0.83 (0.60, 1.14) | 0.43 | 0.43 | 494 | 1458 | 12.2 | 0.80 (0.59, 1.07) | 0.46 | 0.46 |
| Burkina Faso | iLiNS−Zinc (24) | DMC | | | | | | | | | | | | |
| Ghana | GHANA (25) | | | | | | | | | | | | | |
| Ghana | iLiNS−DYAD−G (26) | PSED | | | | | | | | | | | | |
| Haiti | HAITI (27) | | | | | | | | | | | | | |
| Kenya | WASH−B (28) | EASQ | | | | | | | | | | | | |
| Madagascar | MAHAY (29) | ASQI | 956 | 986 | 9.4 | 1.12 (0.70, 1.78) | 0.21 | 0.21 | 553 | 538 | 8.4 | 1.19 (0.66, 2.16) | 0.11 | 0.11 |
| Malawi | iLiNS−DYAD−M (30) | PSED | | | | | | | | | | | | |
| Malawi | iLiNS−DOSE (31) | PSED | | | | | | | | | | | | |
| Mali | PROMIS CS (32) | DMC | | | | | | | | | | | | |
| Zimbabwe | SHINE (HIV−) (33) | MDAT | | | | | | | | | | | | |
| Zimbabwe | SHINE (HIV+) (34) | MDAT | | | | | | | | | | | | |
| | | | **2388** | **3186** | | **I² = 0.00, Tau² = 0.00** | | | **1779** | **2354** | | **I² = 0.00, Tau² = 0.00** | | |
| **Fixed** | | | | | | **0.86 (0.69, 1.06)** | | | | | | **0.89 (0.73, 1.08)** | | |
| **Random** | | | | | | **0.86 (0.69, 1.06)** | | | | | | **0.89 (0.73, 1.08)** | | |

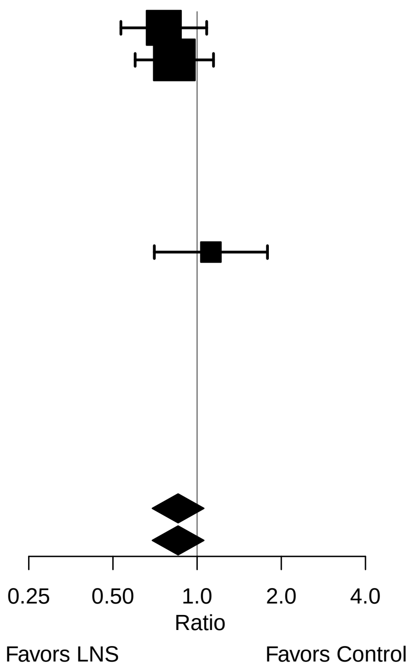


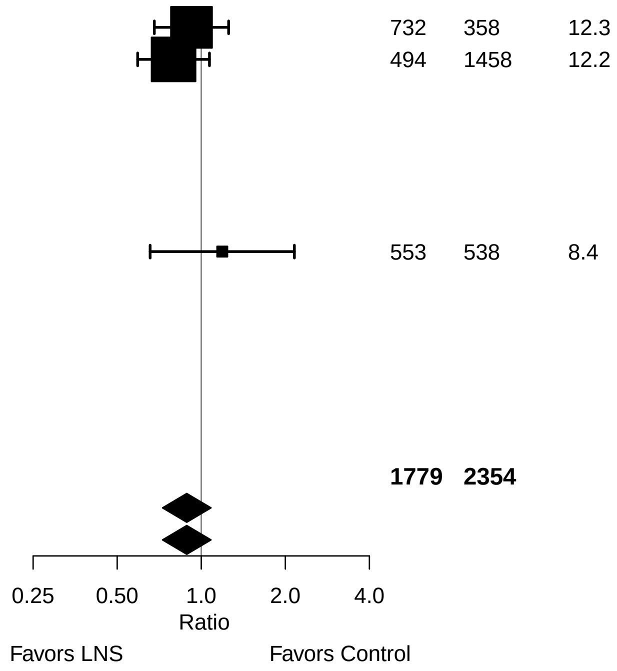

**Supplemental figure 7E: Social-emotional lowest decile prevalence ratio**

## 7E2: Stratified by Maternal BMI

**P−for−interaction = 0.199**
**Ratio of PRs = 1.16 (0.93, 1.45)**

| Country | Trial | Tool | At least 20 kg/m² LNS N | Control N | Control Prevalence | PR (95% CI) | Fixed W | Random W | Less than 20 kg/m² LNS N | Control N | Control Prevalence | PR (95% CI) | Fixed W | Random W |
|---|---|---|---|---|---|---|---|---|---|---|---|---|---|---|
| Bangladesh | JiVitA−4 (21) | | | | | | | | | | | | | |
| Bangladesh | RDNS (22) | DMC | 726 | 335 | 9.0 | 0.86 (0.54, 1.38) | 0.09 | 0.09 | 871 | 455 | 13.2 | 0.84 (0.61, 1.16) | 0.25 | 0.17 |
| Bangladesh | WASH−B (23) | EASQ | 479 | 1493 | 9.1 | 0.80 (0.54, 1.18) | 0.13 | 0.13 | 582 | 1732 | 11.5 | 0.82 (0.61, 1.10) | 0.29 | 0.17 |
| Burkina Faso | iLiNS−Zinc (24) | DMC | 480 | 225 | 14.7 | 0.44 (0.27, 0.71) | 0.09 | 0.09 | 264 | 150 | 19.3 | 0.39 (0.20, 0.75) | 0.06 | 0.11 |
| Ghana | GHANA (25) | | | | | | | | | | | | | |
| Ghana | iLiNS−DYAD−G (26) | PSED | | | | | | | | | | | | |
| Haiti | HAITI (27) | | | | | | | | | | | | | |
| Kenya | WASH−B (28) | EASQ | 1031 | 3535 | 10.0 | 0.85 (0.66, 1.10) | 0.31 | 0.29 | 257 | 1012 | 10.5 | 1.08 (0.73, 1.58) | 0.17 | 0.16 |
| Madagascar | MAHAY (29) | ASQI | | | | | | | | | | | | |
| Malawi | iLiNS−DYAD−M (30) | PSED | 127 | 260 | 12.7 | 0.74 (0.40, 1.39) | 0.05 | 0.05 | 87 | 175 | 6.9 | 1.68 (0.75, 3.73) | 0.04 | 0.09 |
| Malawi | iLiNS−DOSE (31) | PSED | 478 | 158 | 8.9 | 1.02 (0.57, 1.81) | 0.06 | 0.06 | 163 | 61 | 9.8 | 1.43 (0.61, 3.35) | 0.04 | 0.08 |
| Mali | PROMIS CS (32) | DMC | 683 | 664 | 11.9 | 0.66 (0.45, 0.98) | 0.13 | 0.13 | 238 | 274 | 9.9 | 1.07 (0.67, 1.70) | 0.12 | 0.14 |
| Zimbabwe | SHINE (HIV−) (33) | MDAT | 601 | 581 | 11.5 | 0.74 (0.50, 1.07) | 0.14 | 0.14 | 100 | 96 | 17.7 | 0.45 (0.20, 1.02) | 0.04 | 0.08 |
| Zimbabwe | SHINE (HIV+) (34) | MDAT | | | | | | | | | | | | |
| | | | **4605** | **7251** | | **I² = 0.07, Tau² = 0.00** | | | **2562** | **3955** | | **I² = 0.51, Tau² = 0.12** | | |
| **Fixed** | | | | | | **0.76 (0.66, 0.88)** | | | | | | **0.88 (0.75, 1.03)** | | |
| **Random** | | | | | | **0.76 (0.65, 0.88)** | | | | | | **0.87 (0.64, 1.19)** | | |

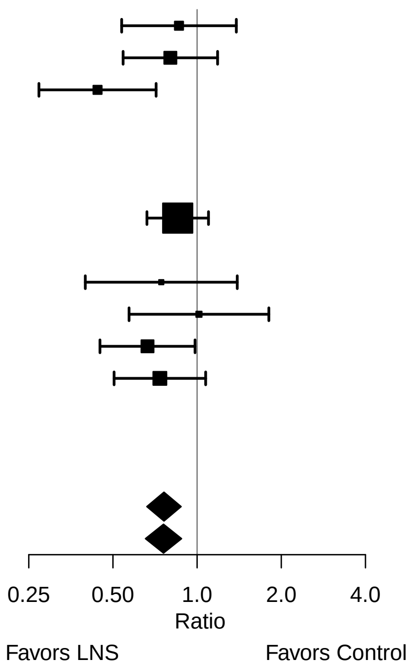


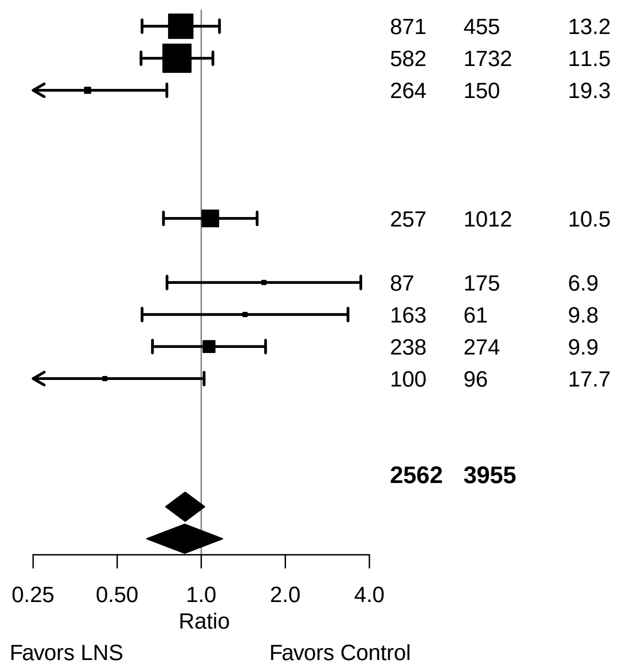

## Supplemental figure 7E: Social-emotional lowest decile prevalence ratio

### 7E3: Stratified by Maternal age

**P−for−interaction = 0.546**
**Ratio of PRs = 0.95 (0.79, 1.13)**

| Country | Trial | Tool | LNS N | Control N | Control Prevalence | At least 25 y PR (95% CI) | Fixed W | Random W | LNS N | Control N | Control Prevalence | Less than 25 y PR (95% CI) | Fixed W | Random W |
|---|---|---|---|---|---|---|---|---|---|---|---|---|---|---|
| Bangladesh | JiVitA−4 (21) | | | | | | | | | | | | | |
| Bangladesh | RDNS (22) | DMC | 450 | 221 | 11.8 | 1.06 (0.67, 1.66) | 0.09 | 0.10 | 1207 | 594 | 11.3 | 0.76 (0.58, 0.99) | 0.25 | 0.25 |
| Bangladesh | WASH−B (23) | EASQ | 458 | 1442 | 11.2 | 1.00 (0.73, 1.37) | 0.18 | 0.13 | 603 | 1809 | 10.0 | 0.66 (0.47, 0.94) | 0.15 | 0.15 |
| Burkina Faso | iLiNS−Zinc (24) | DMC | 421 | 232 | 19.8 | 0.36 (0.21, 0.62) | 0.06 | 0.09 | 321 | 141 | 11.3 | 0.58 (0.28, 1.17) | 0.04 | 0.04 |
| Ghana | GHANA (25) | | | | | | | | | | | | | |
| Ghana | iLiNS−DYAD−G (26) | PSED | 212 | 404 | 10.6 | 1.15 (0.73, 1.82) | 0.08 | 0.10 | 120 | 253 | 9.1 | 0.64 (0.28, 1.45) | 0.03 | 0.03 |
| Haiti | HAITI (27) | | | | | | | | | | | | | |
| Kenya | WASH−B (28) | EASQ | 798 | 2622 | 10.1 | 0.85 (0.65, 1.10) | 0.26 | 0.14 | 549 | 2092 | 10.7 | 0.90 (0.66, 1.24) | 0.18 | 0.18 |
| Madagascar | MAHAY (29) | ASQI | 852 | 901 | 8.8 | 1.23 (0.79, 1.91) | 0.09 | 0.10 | 759 | 703 | 10.0 | 1.06 (0.64, 1.75) | 0.07 | 0.07 |
| Malawi | iLiNS−DYAD−M (30) | PSED | 103 | 224 | 10.3 | 0.95 (0.47, 1.91) | 0.04 | 0.07 | 112 | 214 | 10.3 | 1.04 (0.54, 2.03) | 0.04 | 0.04 |
| Malawi | iLiNS−DOSE (31) | PSED | 346 | 124 | 10.5 | 1.05 (0.58, 1.90) | 0.05 | 0.08 | 286 | 90 | 6.7 | 1.47 (0.63, 3.43) | 0.03 | 0.03 |
| Mali | PROMIS CS (32) | DMC | 492 | 492 | 11.0 | 0.65 (0.42, 1.00) | 0.09 | 0.10 | 435 | 452 | 11.7 | 0.90 (0.61, 1.34) | 0.12 | 0.12 |
| Zimbabwe | SHINE (HIV−) (33) | MDAT | 427 | 389 | 12.6 | 0.56 (0.34, 0.91) | 0.07 | 0.09 | 326 | 321 | 13.1 | 0.75 (0.48, 1.18) | 0.09 | 0.09 |
| Zimbabwe | SHINE (HIV+) (34) | MDAT | | | | | | | | | | | | |
| | | | **4559** | **7051** | | **I² = 0.56, Tau² = 0.08** | | | **4718** | **6669** | | **I² = 0.00, Tau² = 0.00** | | |
| **Fixed** | | | | | | **0.86 (0.75, 0.98)** | | | | | | **0.81 (0.71, 0.93)** | | |
| **Random** | | | | | | **0.84 (0.67, 1.06)** | | | | | | **0.81 (0.71, 0.93)** | | |

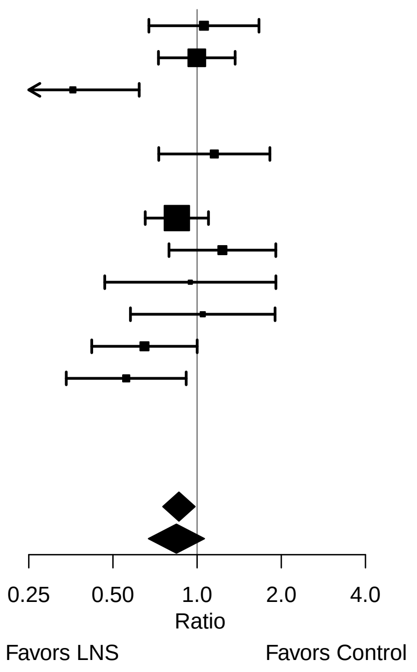


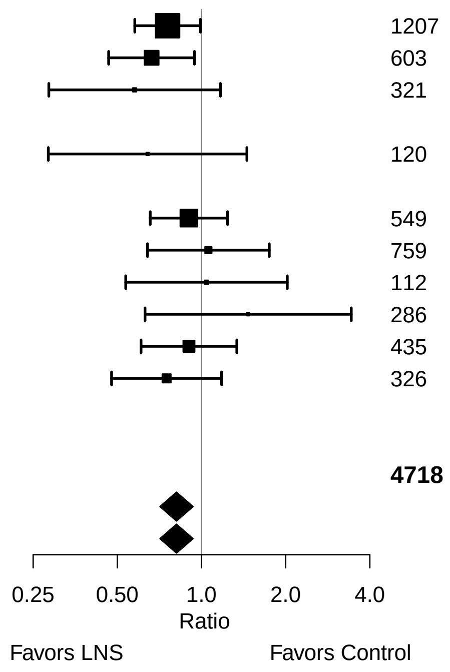

**Supplemental figure 7E: Social-emotional lowest decile prevalence ratio**

## 7E4: Stratified by Maternal education

**P−for−interaction = 0.638**
**Ratio of PRs = 0.95 (0.76, 1.18)**

| Country | Trial | Tool | LNS N | Control N | Control Prevalence | Primary or greater PR (95% CI) | Fixed W | Random W | LNS N | Control N | Control Prevalence | Incomplete or no formal PR (95% CI) | Fixed W | Random W |
|---|---|---|---|---|---|---|---|---|---|---|---|---|---|---|
| Bangladesh | JiVitA−4 (21) | | | | | | | | | | | | | |
| Bangladesh | RDNS (22) | DMC | 1241 | 589 | 9.3 | 0.77 (0.55, 1.07) | 0.24 | 0.23 | 416 | 226 | 16.8 | 1.00 (0.70, 1.44) | 0.17 | 0.17 |
| Bangladesh | WASH−B (23) | EASQ | 750 | 2339 | 9.1 | 0.79 (0.57, 1.10) | 0.24 | 0.23 | 316 | 927 | 14.1 | 0.83 (0.61, 1.13) | 0.23 | 0.23 |
| Burkina Faso | iLiNS−Zinc (24) | DMC | | | | | | | | | | | | |
| Ghana | GHANA (25) | | | | | | | | | | | | | |
| Ghana | iLiNS−DYAD−G (26) | PSED | 257 | 518 | 10.0 | 1.05 (0.67, 1.63) | 0.13 | 0.16 | 75 | 139 | 10.1 | 0.79 (0.32, 1.98) | 0.03 | 0.03 |
| Haiti | HAITI (27) | | | | | | | | | | | | | |
| Kenya | WASH−B (28) | EASQ | 658 | 2258 | 7.4 | 0.90 (0.68, 1.21) | 0.31 | 0.26 | 702 | 2484 | 13.0 | 0.87 (0.66, 1.15) | 0.28 | 0.28 |
| Madagascar | MAHAY (29) | ASQI | 339 | 416 | 5.8 | 1.84 (0.98, 3.47) | 0.06 | 0.10 | 1274 | 1188 | 10.5 | 1.02 (0.66, 1.59) | 0.11 | 0.11 |
| Malawi | iLiNS−DYAD−M (30) | PSED | | | | | | | | | | | | |
| Malawi | iLiNS−DOSE (31) | PSED | | | | | | | | | | | | |
| Mali | PROMIS CS (32) | DMC | 100 | 94 | 7.4 | 1.21 (0.35, 4.19) | 0.02 | 0.03 | 826 | 850 | 11.8 | 0.74 (0.52, 1.05) | 0.18 | 0.18 |
| Zimbabwe | SHINE (HIV−) (33) | MDAT | | | | | | | | | | | | |
| Zimbabwe | SHINE (HIV+) (34) | MDAT | | | | | | | | | | | | |
| | | | **3345** | **6214** | | **I² = 0.29, Tau² = 0.03** | | | **3609** | **5814** | | **I² = 0.00, Tau² = 0.00** | | |
| **Fixed** | | | | | | **0.90 (0.77, 1.06)** | | | | | | **0.87 (0.75, 1.01)** | | |
| **Random** | | | | | | **0.93 (0.75, 1.16)** | | | | | | **0.87 (0.75, 1.01)** | | |

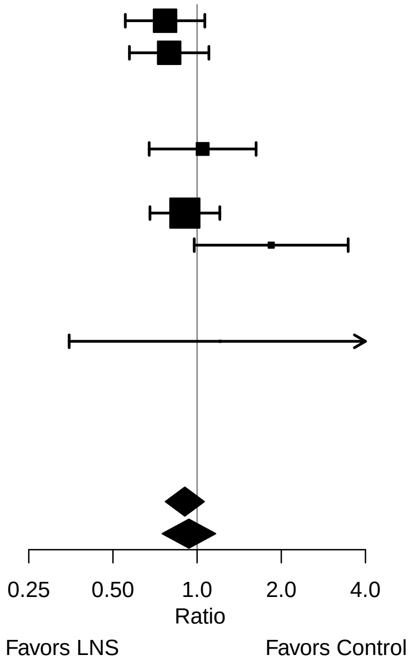


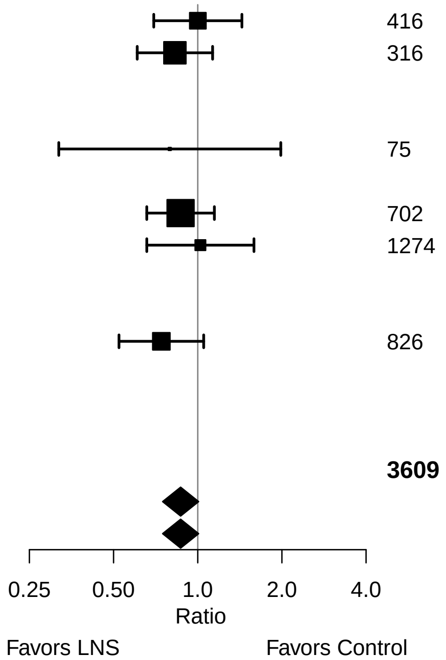

## Supplemental figure 7E: Social-emotional lowest decile prevalence ratio

### 7E5: Stratified by Maternal depressive symptoms

**P−for−interaction = 0.165**
**Ratio of PRs = 0.86 (0.69, 1.07)**

| | | | Less than 75th percentile | | | | | | At least 75th percentile | | | | | |
|---|---|---|---|---|---|---|---|---|---|---|---|---|---|---|
| **Country** | **Trial** | **Tool** | **LNS N** | **Control N** | **Control Prevalence** | **PR (95% CI)** | **Fixed W** | **Random W** | **LNS N** | **Control N** | **Control Prevalence** | **PR (95% CI)** | **Fixed W** | **Random W** |
| Bangladesh | JiVitA−4 (21) | | | | | | | | | | | | | |
| Bangladesh | RDNS (22) | DMC | 1075 | 472 | 10.8 | 0.74 (0.56, 0.97) | 0.21 | 0.21 | 520 | 288 | 12.2 | 1.01 (0.69, 1.50) | 0.24 | 0.24 |
| Bangladesh | WASH−B (23) | EASQ | 822 | 2356 | 9.6 | 0.81 (0.61, 1.07) | 0.20 | 0.20 | 223 | 830 | 13.1 | 0.89 (0.60, 1.31) | 0.24 | 0.24 |
| Burkina Faso | iLiNS−Zinc (24) | DMC | | | | | | | | | | | | |
| Ghana | GHANA (25) | | | | | | | | | | | | | |
| Ghana | iLiNS−DYAD−G (26) | PSED | 242 | 435 | 11.0 | 0.79 (0.48, 1.28) | 0.07 | 0.07 | 78 | 201 | 9.0 | 1.29 (0.60, 2.74) | 0.06 | 0.06 |
| Haiti | HAITI (27) | | | | | | | | | | | | | |
| Kenya | WASH−B (28) | EASQ | 960 | 3317 | 10.0 | 0.94 (0.74, 1.18) | 0.30 | 0.30 | 317 | 1156 | 7.7 | 0.74 (0.44, 1.23) | 0.14 | 0.14 |
| Madagascar | MAHAY (29) | ASQI | 615 | 667 | 10.5 | 0.85 (0.48, 1.51) | 0.05 | 0.05 | 268 | 242 | 9.1 | 1.03 (0.57, 1.85) | 0.10 | 0.10 |
| Malawi | iLiNS−DYAD−M (30) | PSED | 153 | 302 | 9.9 | 0.72 (0.37, 1.40) | 0.04 | 0.04 | 47 | 111 | 9.0 | 1.89 (0.80, 4.49) | 0.05 | 0.05 |
| Malawi | iLiNS−DOSE (31) | PSED | | | | | | | | | | | | |
| Mali | PROMIS CS (32) | DMC | | | | | | | | | | | | |
| Zimbabwe | SHINE (HIV−) (33) | MDAT | 583 | 560 | 11.1 | 0.70 (0.48, 1.02) | 0.11 | 0.11 | 197 | 180 | 16.7 | 0.64 (0.39, 1.04) | 0.15 | 0.15 |
| Zimbabwe | SHINE (HIV+) (34) | MDAT | 131 | 110 | 13.6 | 0.34 (0.14, 0.82) | 0.02 | 0.02 | 33 | 32 | 15.6 | 0.97 (0.32, 2.92) | 0.03 | 0.03 |
| | | | **4581** | **8219** | | **I² = 0.00, Tau² = 0.00** | | | **1683** | **3040** | | **I² = 0.00, Tau² = 0.00** | | |
| **Fixed** | | | | | | **0.80 (0.70, 0.91)** | | | | | | **0.92 (0.76, 1.11)** | | |
| **Random** | | | | | | **0.80 (0.70, 0.91)** | | | | | | **0.92 (0.76, 1.11)** | | |

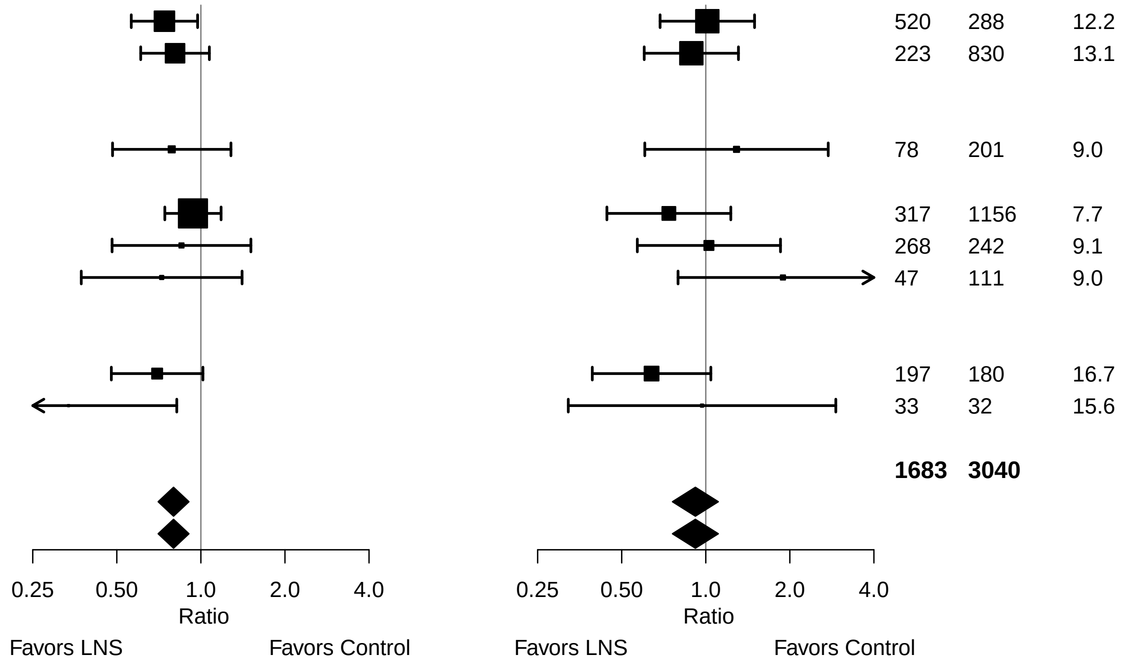

## Supplemental figure 7E: Social-emotional lowest decile prevalence ratio

### 7E6: Stratified by Child sex

**P−for−interaction = 0.757**
**Ratio of PRs = 1.03 (0.87, 1.21)**

| Country | Trial | Tool | Male LNS N | Male Control N | Male Control Prevalence | Male PR (95% CI) | Male Fixed W | Male Random W | Female LNS N | Female Control N | Female Control Prevalence | Female PR (95% CI) | Female Fixed W | Female Random W |
|---|---|---|---|---|---|---|---|---|---|---|---|---|---|---|
| Bangladesh | JiVitA−4 (21) | | | | | | | | | | | | | |
| Bangladesh | RDNS (22) | DMC | 830 | 411 | 9.0 | 1.03 (0.73, 1.45) | 0.13 | 0.11 | 827 | 404 | 13.9 | 0.72 (0.51, 0.99) | 0.17 | 0.13 |
| Bangladesh | WASH−B (23) | EASQ | 522 | 1635 | 10.4 | 0.77 (0.56, 1.08) | 0.14 | 0.11 | 544 | 1631 | 10.6 | 0.85 (0.61, 1.19) | 0.16 | 0.13 |
| Burkina Faso | iLiNS−Zinc (24) | DMC | 372 | 193 | 13.5 | 0.44 (0.27, 0.70) | 0.07 | 0.09 | 374 | 182 | 19.8 | 0.39 (0.22, 0.69) | 0.06 | 0.08 |
| Ghana | GHANA (25) | | | | | | | | | | | | | |
| Ghana | iLiNS−DYAD−G (26) | PSED | 164 | 306 | 7.8 | 1.32 (0.73, 2.39) | 0.04 | 0.08 | 168 | 351 | 12.0 | 0.80 (0.46, 1.37) | 0.06 | 0.08 |
| Haiti | HAITI (27) | | | | | | | | | | | | | |
| Kenya | WASH−B (28) | EASQ | 682 | 2260 | 10.8 | 0.96 (0.76, 1.22) | 0.27 | 0.13 | 680 | 2485 | 9.9 | 0.77 (0.55, 1.09) | 0.16 | 0.13 |
| Madagascar | MAHAY (29) | ASQI | 795 | 787 | 11.1 | 0.92 (0.60, 1.42) | 0.08 | 0.10 | 818 | 817 | 7.6 | 1.48 (0.90, 2.43) | 0.08 | 0.09 |
| Malawi | iLiNS−DYAD−M (30) | PSED | 105 | 205 | 12.2 | 0.62 (0.29, 1.34) | 0.03 | 0.06 | 110 | 233 | 8.6 | 1.48 (0.78, 2.82) | 0.04 | 0.07 |
| Malawi | iLiNS−DOSE (31) | PSED | 320 | 117 | 6.8 | 1.74 (0.83, 3.61) | 0.03 | 0.06 | 324 | 104 | 12.5 | 0.69 (0.37, 1.28) | 0.05 | 0.07 |
| Mali | PROMIS CS (32) | DMC | 469 | 514 | 10.7 | 0.76 (0.51, 1.12) | 0.10 | 0.10 | 458 | 430 | 12.1 | 0.78 (0.52, 1.16) | 0.12 | 0.11 |
| Zimbabwe | SHINE (HIV−) (33) | MDAT | 424 | 388 | 13.1 | 0.66 (0.43, 1.01) | 0.09 | 0.10 | 417 | 411 | 10.7 | 0.76 (0.48, 1.21) | 0.09 | 0.10 |
| Zimbabwe | SHINE (HIV+) (34) | MDAT | 84 | 79 | 20.3 | 0.35 (0.15, 0.82) | 0.02 | 0.05 | 86 | 72 | 9.7 | 0.60 (0.17, 2.16) | 0.01 | 0.02 |
| | | | **4767** | **6895** | | **$I^2$ = 0.55, $Tau^2$ = 0.10** | | | **4806** | **7120** | | **$I^2$ = 0.40, $Tau^2$ = 0.05** | | |
| **Fixed** | | | | | | **0.84 (0.74, 0.95)** | | | | | | **0.80 (0.70, 0.92)** | | |
| **Random** | | | | | | **0.81 (0.64, 1.03)** | | | | | | **0.80 (0.66, 0.98)** | | |

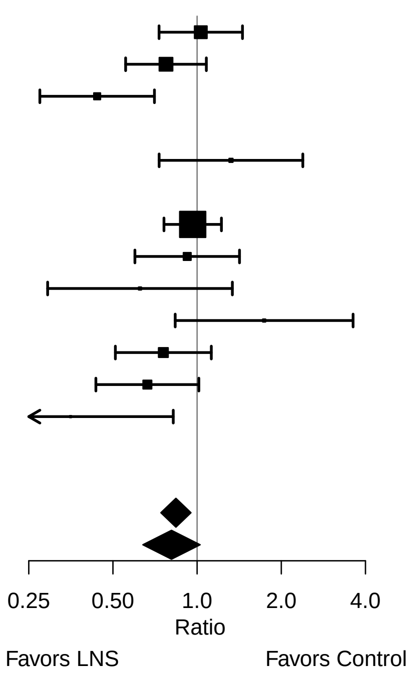


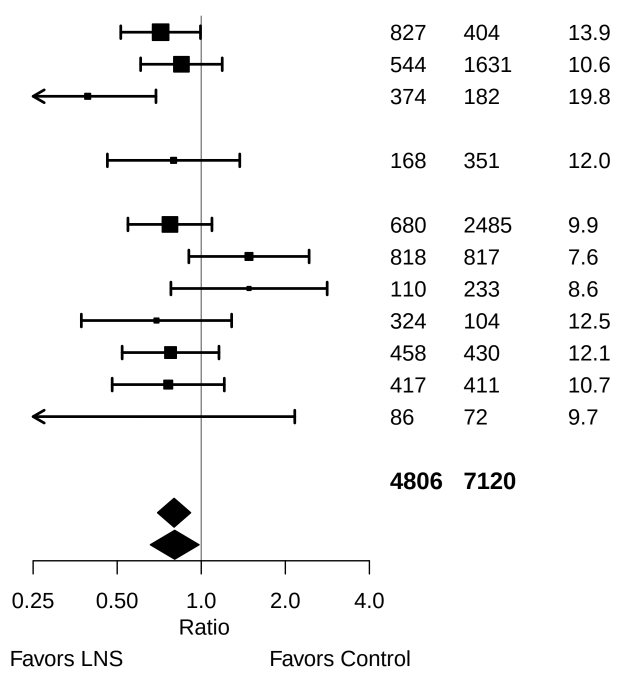

## Supplemental figure 7E: Social-emotional lowest decile prevalence ratio

### 7E7: Stratified by Child birth order

**P−for−interaction = 0.920**
**Ratio of PRs = 1.01 (0.83, 1.24)**

| Country | Trial | Tool | LNS N | Control N | Control Prevalence | Later born PR (95% CI) | Fixed W | Random W | LNS N | Control N | Control Prevalence | Firstborn PR (95% CI) | Fixed W | Random W |
|---|---|---|---|---|---|---|---|---|---|---|---|---|---|---|
| Bangladesh | JiVitA−4 (21) | | | | | | | | | | | | | |
| Bangladesh | RDNS (22) | DMC | 969 | 508 | 12.4 | 0.82 (0.59, 1.12) | 0.13 | 0.12 | 687 | 307 | 9.8 | 0.91 (0.65, 1.26) | 0.29 | 0.29 |
| Bangladesh | WASH−B (23) | EASQ | 669 | 2134 | 10.8 | 0.88 (0.67, 1.16) | 0.17 | 0.13 | 377 | 1031 | 9.0 | 0.76 (0.50, 1.18) | 0.17 | 0.17 |
| Burkina Faso | iLiNS−Zinc (24) | DMC | 587 | 290 | 17.9 | 0.34 (0.23, 0.52) | 0.07 | 0.10 | 159 | 85 | 11.8 | 0.80 (0.34, 1.87) | 0.04 | 0.04 |
| Ghana | GHANA (25) | | | | | | | | | | | | | |
| Ghana | iLiNS−DYAD−G (26) | PSED | 225 | 444 | 9.7 | 1.24 (0.79, 1.95) | 0.06 | 0.10 | 107 | 213 | 10.8 | 0.52 (0.22, 1.24) | 0.04 | 0.04 |
| Haiti | HAITI (27) | | | | | | | | | | | | | |
| Kenya | WASH−B (28) | EASQ | 1071 | 3767 | 10.4 | 0.89 (0.71, 1.11) | 0.26 | 0.14 | 289 | 974 | 10.1 | 0.83 (0.53, 1.29) | 0.16 | 0.16 |
| Madagascar | MAHAY (29) | ASQI | 1162 | 1118 | 8.9 | 1.17 (0.74, 1.84) | 0.06 | 0.10 | 433 | 465 | 10.8 | 1.05 (0.59, 1.88) | 0.09 | 0.09 |
| Malawi | iLiNS−DYAD−M (30) | PSED | 170 | 349 | 10.9 | 0.81 (0.46, 1.43) | 0.04 | 0.08 | 45 | 87 | 8.0 | 1.93 (0.72, 5.17) | 0.03 | 0.03 |
| Malawi | iLiNS−DOSE (31) | PSED | | | | | | | | | | | | |
| Mali | PROMIS CS (32) | DMC | 770 | 811 | 11.2 | 0.75 (0.53, 1.07) | 0.10 | 0.11 | 142 | 122 | 9.0 | 1.09 (0.52, 2.31) | 0.06 | 0.06 |
| Zimbabwe | SHINE (HIV−) (33) | MDAT | 602 | 574 | 11.5 | 0.74 (0.52, 1.05) | 0.10 | 0.12 | 203 | 191 | 14.7 | 0.60 (0.36, 1.02) | 0.11 | 0.11 |
| Zimbabwe | SHINE (HIV+) (34) | MDAT | | | | | | | | | | | | |
| | | | **6225** | **9995** | | **I² = 0.66, Tau² = 0.09** | | | **2442** | **3475** | | **I² = 0.00, Tau² = 0.00** | | |
| **Fixed** | | | | | | **0.82 (0.73, 0.92)** | | | | | | **0.85 (0.71, 1.01)** | | |
| **Random** | | | | | | **0.80 (0.64, 1.01)** | | | | | | **0.85 (0.71, 1.01)** | | |

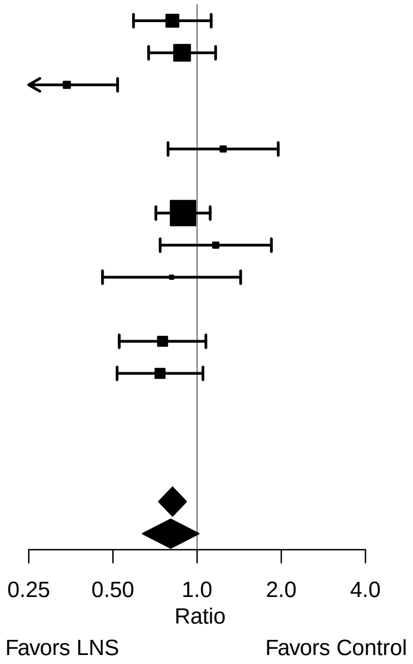


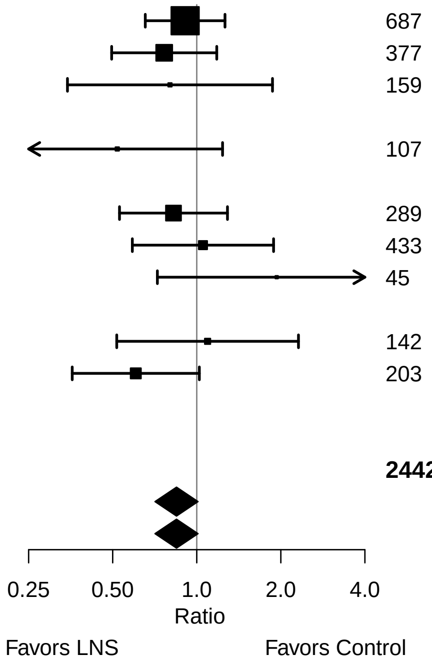

## Supplemental figure 7E: Social-emotional lowest decile prevalence ratio

## 7E8: Stratified by Child baseline stunting

**P−for−interaction = 0.728**
**Ratio of PRs = 0.94 (0.67, 1.32)**

| Country | Trial | Tool | No: LNS N | No: Control N | No: Control Prevalence | No: PR (95% CI) | No: Fixed W | No: Random W | Yes: LNS N | Yes: Control N | Yes: Control Prevalence | Yes: PR (95% CI) | Yes: Fixed W | Yes: Random W |
|---|---|---|---|---|---|---|---|---|---|---|---|---|---|---|
| Bangladesh | JiVitA−4 (21) | | | | | | | | | | | | | |
| Bangladesh | RDNS (22) | DMC | 1241 | 574 | 8.9 | 0.95 (0.70, 1.29) | 0.45 | 0.29 | 354 | 185 | 18.9 | 0.67 (0.44, 1.02) | 0.45 | 0.36 |
| Bangladesh | WASH−B (23) | EASQ | | | | | | | | | | | | |
| Burkina Faso | iLiNS−Zinc (24) | DMC | 576 | 294 | 13.3 | 0.41 (0.26, 0.63) | 0.22 | 0.25 | 170 | 80 | 27.5 | 0.43 (0.24, 0.75) | 0.24 | 0.26 |
| Ghana | GHANA (25) | | | | | | | | | | | | | |
| Ghana | iLiNS−DYAD−G (26) | PSED | | | | | | | | | | | | |
| Haiti | HAITI (27) | | | | | | | | | | | | | |
| Kenya | WASH−B (28) | EASQ | | | | | | | | | | | | |
| Madagascar | MAHAY (29) | ASQI | | | | | | | | | | | | |
| Malawi | iLiNS−DYAD−M (30) | PSED | | | | | | | | | | | | |
| Malawi | iLiNS−DOSE (31) | PSED | 464 | 147 | 8.8 | 1.10 (0.61, 1.98) | 0.12 | 0.21 | 180 | 74 | 10.8 | 1.08 (0.50, 2.33) | 0.13 | 0.17 |
| Mali | PROMIS CS (32) | DMC | | | | | | | | | | | | |
| Zimbabwe | SHINE (HIV−) (33) | MDAT | 532 | 490 | 10.8 | 0.59 (0.38, 0.91) | 0.22 | 0.25 | 122 | 105 | 14.3 | 0.86 (0.45, 1.65) | 0.18 | 0.21 |
| Zimbabwe | SHINE (HIV+) (34) | MDAT | | | | | | | | | | | | |
| | | | **2813** | **1505** | | **I² = 0.76, Tau² = 0.15** | | | **826** | **444** | | **I² = 0.33, Tau² = 0.05** | | |
| **Fixed** | | | | | | **0.73 (0.59, 0.89)** | | | | | | **0.67 (0.51, 0.89)** | | |
| **Random** | | | | | | **0.70 (0.45, 1.09)** | | | | | | **0.68 (0.48, 0.98)** | | |

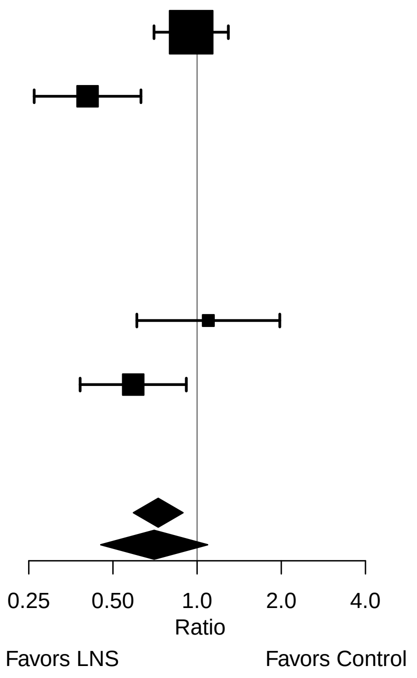


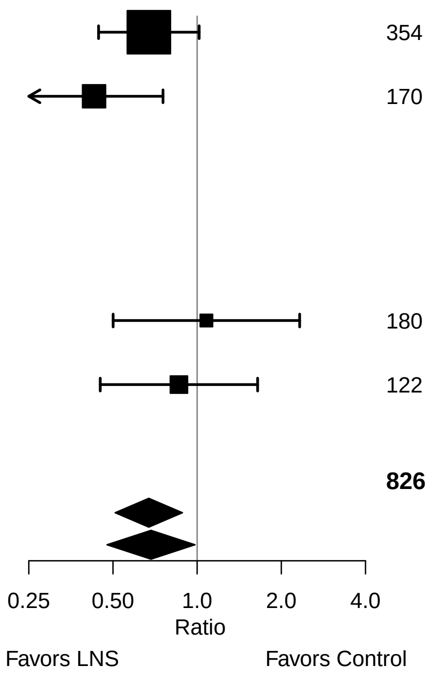

**Supplemental figure 7E: Social-emotional lowest decile prevalence ratio**

## 7E9: Stratified by Child baseline acute malnutrition (insufficient comparisons)

**Supplemental figure 7E: Social-emotional lowest decile prevalence ratio**

## 7E10: Stratified by Child baseline anemia

**P−for−interaction = 0.881**
**Ratio of PRs = 1.04 (0.63, 1.70)**

| Country | Trial | Tool | LNS N | Control N | Control Prevalence | Not anemic PR (95% CI) | Fixed W | Random W | LNS N | Control N | Control Prevalence | Anemic PR (95% CI) | Fixed W | Random W |
|---|---|---|---|---|---|---|---|---|---|---|---|---|---|---|
| Bangladesh | JiVitA−4 (21) | | | | | | | | | | | | | |
| Bangladesh | RDNS (22) | DMC | 212 | 96 | 14.6 | 0.49 (0.23, 1.01) | 0.23 | 0.24 | 316 | 160 | 12.5 | 0.76 (0.48, 1.21) | 0.37 | 0.37 |
| Bangladesh | WASH−B (23) | EASQ | | | | | | | | | | | | |
| Burkina Faso | iLiNS−Zinc (24) | DMC | | | | | | | | | | | | |
| Ghana | GHANA (25) | | | | | | | | | | | | | |
| Ghana | iLiNS−DYAD−G (26) | PSED | 193 | 350 | 10.3 | 1.01 (0.60, 1.69) | 0.46 | 0.41 | 90 | 204 | 9.8 | 0.91 (0.42, 1.98) | 0.13 | 0.13 |
| Haiti | HAITI (27) | | | | | | | | | | | | | |
| Kenya | WASH−B (28) | EASQ | | | | | | | | | | | | |
| Madagascar | MAHAY (29) | ASQI | | | | | | | | | | | | |
| Malawi | iLiNS−DYAD−M (30) | PSED | 66 | 147 | 8.2 | 0.93 (0.34, 2.53) | 0.12 | 0.14 | 138 | 275 | 10.9 | 1.06 (0.60, 1.88) | 0.25 | 0.25 |
| Malawi | iLiNS−DOSE (31) | PSED | 223 | 94 | 7.4 | 1.32 (0.59, 2.99) | 0.19 | 0.21 | 417 | 126 | 11.1 | 0.93 (0.53, 1.64) | 0.25 | 0.25 |
| Mali | PROMIS CS (32) | DMC | | | | | | | | | | | | |
| Zimbabwe | SHINE (HIV−) (33) | MDAT | | | | | | | | | | | | |
| Zimbabwe | SHINE (HIV+) (34) | MDAT | | | | | | | | | | | | |
| | | | **694** | **687** | | **I² = 0.21, Tau² = 0.04** | | | **961** | **765** | | **I² = 0.00, Tau² = 0.00** | | |
| **Fixed** | | | | | | **0.89 (0.62, 1.26)** | | | | | | **0.89 (0.67, 1.18)** | | |
| **Random** | | | | | | **0.88 (0.59, 1.32)** | | | | | | **0.89 (0.67, 1.18)** | | |

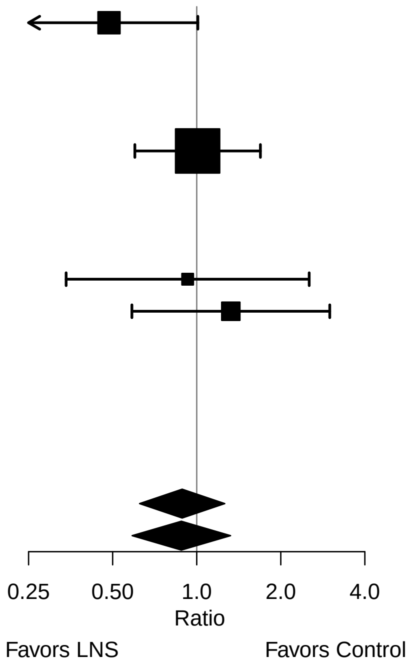


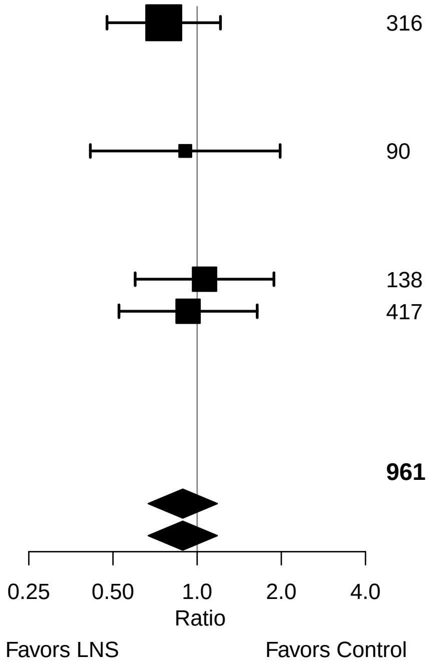

# Supplemental figure 7F: Social-emotional lowest decile prevalence difference

## 7F1: Stratified by Maternal height

**P−for−interaction = 0.920**
**Difference in PDs = 0.00 (−0.02, 0.03)**

| | | | At least 150.1 cm | | | | | | Less than 150.1 cm | | | | | |
|---|---|---|---|---|---|---|---|---|---|---|---|---|---|---|
| **Country** | **Trial** | **Tool** | **LNS N** | **Control N** | **Control Prevalence** | **PD (95% CI)** | **Fixed W** | **Random W** | **LNS N** | **Control N** | **Control Prevalence** | **PD (95% CI)** | **Fixed W** | **Random W** |
| Bangladesh | JiVitA−4 (21) | | | | | | | | | | | | | |
| Bangladesh | RDNS (22) | DMC | 865 | 432 | 10.6 | −0.03 (−0.06, 0.01) | 0.29 | 0.29 | 732 | 358 | 12.3 | −0.01 (−0.05, 0.03) | 0.36 | 0.36 |
| Bangladesh | WASH−B (23) | EASQ | 567 | 1768 | 8.9 | −0.02 (−0.04, 0.01) | 0.55 | 0.55 | 494 | 1458 | 12.2 | −0.02 (−0.06, 0.01) | 0.49 | 0.49 |
| Burkina Faso | iLiNS−Zinc (24) | DMC | | | | | | | | | | | | |
| Ghana | GHANA (25) | | | | | | | | | | | | | |
| Ghana | iLiNS−DYAD−G (26) | PSED | | | | | | | | | | | | |
| Haiti | HAITI (27) | | | | | | | | | | | | | |
| Kenya | WASH−B (28) | EASQ | | | | | | | | | | | | |
| Madagascar | MAHAY (29) | ASQI | 956 | 986 | 9.4 | 0.01 (−0.04, 0.06) | 0.16 | 0.16 | 553 | 538 | 8.4 | 0.02 (−0.04, 0.07) | 0.15 | 0.15 |
| Malawi | iLiNS−DYAD−M (30) | PSED | | | | | | | | | | | | |
| Malawi | iLiNS−DOSE (31) | PSED | | | | | | | | | | | | |
| Mali | PROMIS CS (32) | DMC | | | | | | | | | | | | |
| Zimbabwe | SHINE (HIV−) (33) | MDAT | | | | | | | | | | | | |
| Zimbabwe | SHINE (HIV+) (34) | MDAT | | | | | | | | | | | | |
| | | | **2388** | **3186** | | **I² = 0.00, Tau² = 0.00** | | | **1779** | **2354** | | **I² = 0.00, Tau² = 0.00** | | |
| **Fixed** | | | | | | **−0.01 (−0.03, 0.00)** | | | | | | **−0.01 (−0.03, 0.01)** | | |
| **Random** | | | | | | **−0.01 (−0.03, 0.00)** | | | | | | **−0.01 (−0.03, 0.01)** | | |

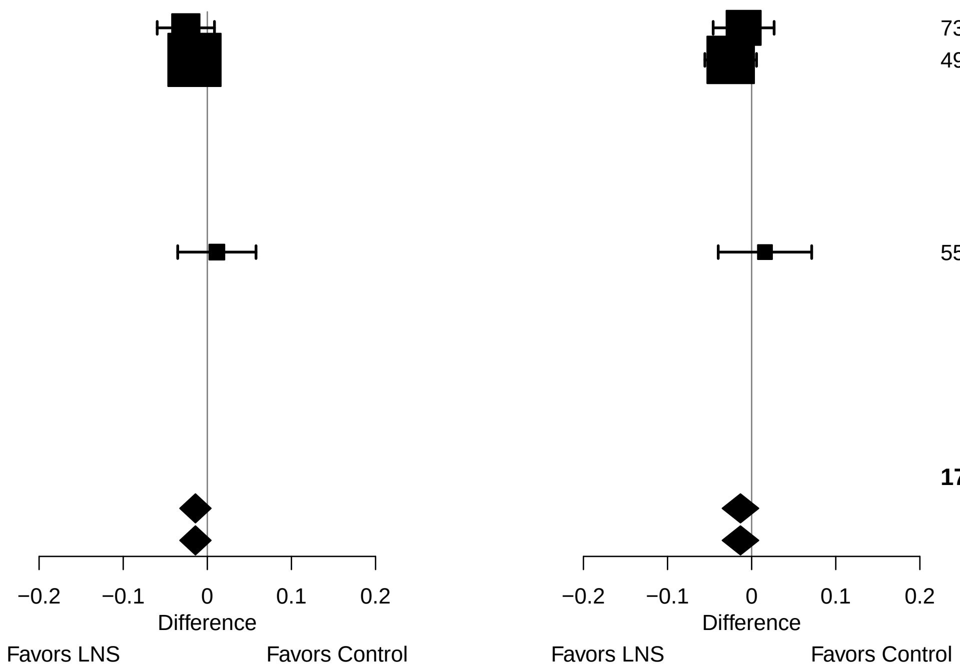

**Supplemental figure 7F: Social-emotional lowest decile prevalence difference**

## 7F2: Stratified by Maternal BMI

**P−for−interaction = 0.455**
**Difference in PDs = 0.01 (−0.01, 0.03)**

| | | | At least 20 kg/m² | | | | | | Less than 20 kg/m² | | | | | |
|---|---|---|---|---|---|---|---|---|---|---|---|---|---|---|
| **Country** | **Trial** | **Tool** | **LNS N** | **Control N** | **Control Prevalence** | **PD (95% CI)** | **Fixed W** | **Random W** | **LNS N** | **Control N** | **Control Prevalence** | **PD (95% CI)** | **Fixed W** | **Random W** |
| Bangladesh | JiVitA−4 (21) | | | | | | | | | | | | | |
| Bangladesh | RDNS (22) | DMC | 726 | 335 | 9.0 | −0.01 (−0.05, 0.03) | 0.10 | 0.10 | 871 | 455 | 13.2 | −0.02 (−0.06, 0.02) | 0.19 | 0.16 |
| Bangladesh | WASH−B (23) | EASQ | 479 | 1493 | 9.1 | −0.02 (−0.05, 0.01) | 0.18 | 0.18 | 582 | 1732 | 11.5 | −0.02 (−0.05, 0.01) | 0.34 | 0.17 |
| Burkina Faso | iLiNS−Zinc (24) | DMC | 480 | 225 | 14.7 | −0.08 (−0.14, −0.02) | 0.04 | 0.04 | 264 | 150 | 19.3 | −0.12 (−0.20, −0.03) | 0.04 | 0.10 |
| Ghana | GHANA (25) | | | | | | | | | | | | | |
| Ghana | iLiNS−DYAD−G (26) | PSED | | | | | | | | | | | | |
| Haiti | HAITI (27) | | | | | | | | | | | | | |
| Kenya | WASH−B (28) | EASQ | 1031 | 3535 | 10.0 | −0.01 (−0.04, 0.01) | 0.34 | 0.34 | 257 | 1012 | 10.5 | 0.01 (−0.03, 0.05) | 0.16 | 0.15 |
| Madagascar | MAHAY (29) | ASQI | | | | | | | | | | | | |
| Malawi | iLiNS−DYAD−M (30) | PSED | 127 | 260 | 12.7 | −0.03 (−0.10, 0.04) | 0.04 | 0.04 | 87 | 175 | 6.9 | 0.05 (−0.02, 0.12) | 0.06 | 0.11 |
| Malawi | iLiNS−DOSE (31) | PSED | 478 | 158 | 8.9 | 0.00 (−0.05, 0.05) | 0.06 | 0.06 | 163 | 61 | 9.8 | 0.04 (−0.06, 0.14) | 0.03 | 0.08 |
| Mali | PROMIS CS (32) | DMC | 683 | 664 | 11.9 | −0.04 (−0.08, 0.00) | 0.12 | 0.12 | 238 | 274 | 9.9 | 0.01 (−0.04, 0.05) | 0.14 | 0.15 |
| Zimbabwe | SHINE (HIV−) (33) | MDAT | 601 | 581 | 11.5 | −0.03 (−0.07, 0.01) | 0.12 | 0.12 | 100 | 96 | 17.7 | −0.10 (−0.19, 0.00) | 0.03 | 0.08 |
| Zimbabwe | SHINE (HIV+) (34) | MDAT | | | | | | | | | | | | |
| | | | **4605** | **7251** | | **I² = 0.00, Tau² = 0.00** | | | **2562** | **3955** | | **I² = 0.52, Tau² = 0.00** | | |
| **Fixed** | | | | | | **−0.02 (−0.04, −0.01)** | | | | | | **−0.01 (−0.03, 0.00)** | | |
| **Random** | | | | | | **−0.02 (−0.04, −0.01)** | | | | | | **−0.02 (−0.05, 0.02)** | | |

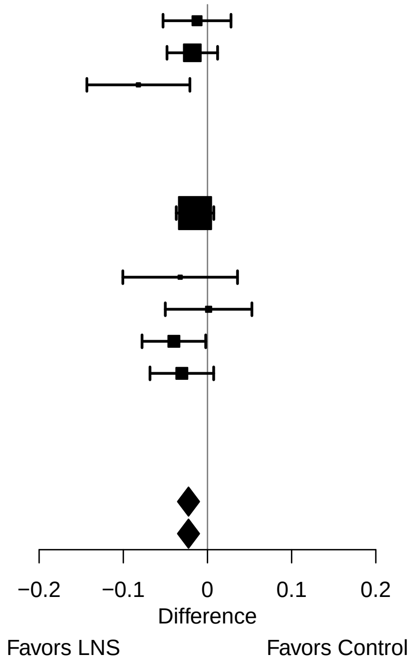


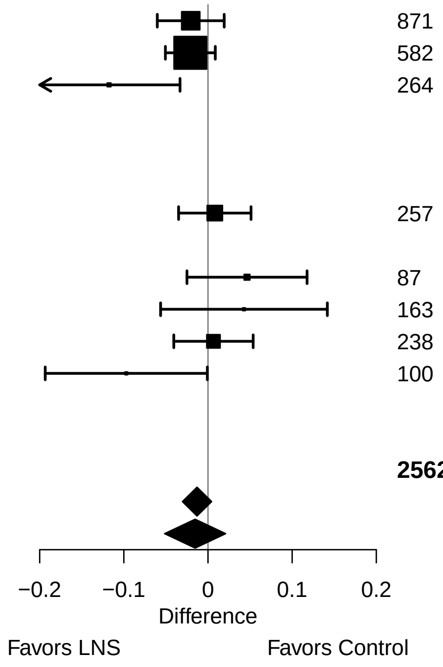

## Supplemental figure 7F: Social-emotional lowest decile prevalence difference

### 7F3: Stratified by Maternal age

**P−for−interaction = 0.701**
**Difference in PDs = 0.00 (−0.02, 0.01)**

| | | | At least 25 y | | | | | | Less than 25 y | | | | | |
|---|---|---|---|---|---|---|---|---|---|---|---|---|---|---|
| Country | Trial | Tool | LNS N | Control N | Control Prevalence | PD (95% CI) | Fixed W | Random W | LNS N | Control N | Control Prevalence | PD (95% CI) | Fixed W | Random W |
| Bangladesh | JiVitA−4 (21) | | | | | | | | | | | | | |
| Bangladesh | RDNS (22) | DMC | 450 | 221 | 11.8 | 0.01 (−0.05, 0.06) | 0.06 | 0.09 | 1207 | 594 | 11.3 | −0.03 (−0.05, 0.00) | 0.22 | 0.22 |
| Bangladesh | WASH−B (23) | EASQ | 458 | 1442 | 11.2 | 0.00 (−0.04, 0.03) | 0.14 | 0.12 | 603 | 1809 | 10.0 | −0.03 (−0.06, −0.01) | 0.24 | 0.24 |
| Burkina Faso | iLiNS−Zinc (24) | DMC | 421 | 232 | 19.8 | −0.13 (−0.19, −0.06) | 0.04 | 0.08 | 321 | 141 | 11.3 | −0.05 (−0.11, 0.02) | 0.04 | 0.04 |
| Ghana | GHANA (25) | | | | | | | | | | | | | |
| Ghana | iLiNS−DYAD−G (26) | PSED | 212 | 404 | 10.6 | 0.02 (−0.04, 0.07) | 0.06 | 0.09 | 120 | 253 | 9.1 | −0.03 (−0.09, 0.03) | 0.05 | 0.05 |
| Haiti | HAITI (27) | | | | | | | | | | | | | |
| Kenya | WASH−B (28) | EASQ | 798 | 2622 | 10.1 | −0.02 (−0.04, 0.01) | 0.33 | 0.14 | 549 | 2092 | 10.7 | −0.01 (−0.04, 0.02) | 0.17 | 0.17 |
| Madagascar | MAHAY (29) | ASQI | 852 | 901 | 8.8 | 0.02 (−0.02, 0.06) | 0.09 | 0.11 | 759 | 703 | 10.0 | 0.01 (−0.05, 0.06) | 0.06 | 0.06 |
| Malawi | iLiNS−DYAD−M (30) | PSED | 103 | 224 | 10.3 | −0.01 (−0.08, 0.06) | 0.03 | 0.07 | 112 | 214 | 10.3 | 0.00 (−0.07, 0.07) | 0.03 | 0.03 |
| Malawi | iLiNS−DOSE (31) | PSED | 346 | 124 | 10.5 | 0.00 (−0.06, 0.07) | 0.04 | 0.08 | 286 | 90 | 6.7 | 0.03 (−0.04, 0.10) | 0.04 | 0.04 |
| Mali | PROMIS CS (32) | DMC | 492 | 492 | 11.0 | −0.04 (−0.08, 0.00) | 0.12 | 0.12 | 435 | 452 | 11.7 | −0.01 (−0.06, 0.03) | 0.09 | 0.09 |
| Zimbabwe | SHINE (HIV−) (33) | MDAT | 427 | 389 | 12.6 | −0.06 (−0.10, −0.01) | 0.09 | 0.11 | 326 | 321 | 13.1 | −0.03 (−0.09, 0.02) | 0.06 | 0.06 |
| Zimbabwe | SHINE (HIV+) (34) | MDAT | | | | | | | | | | | | |
| | | | **4559** | **7051** | | **I² = 0.57, Tau² = 0.00** | | | **4718** | **6669** | | **I² = 0.00, Tau² = 0.00** | | |
| **Fixed** | | | | | | **−0.02 (−0.03, 0.00)** | | | | | | **−0.02 (−0.03, −0.01)** | | |
| **Random** | | | | | | **−0.02 (−0.04, 0.01)** | | | | | | **−0.02 (−0.03, −0.01)** | | |

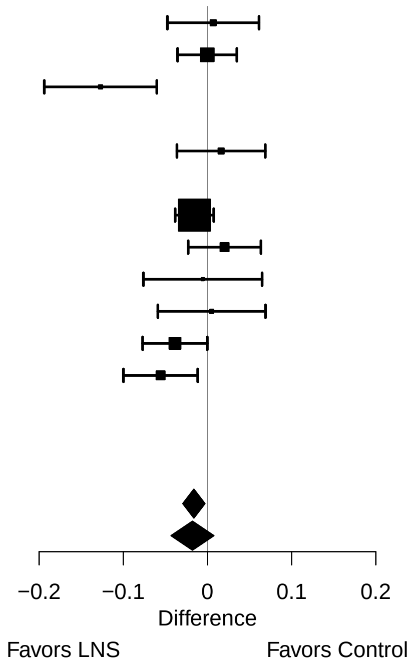


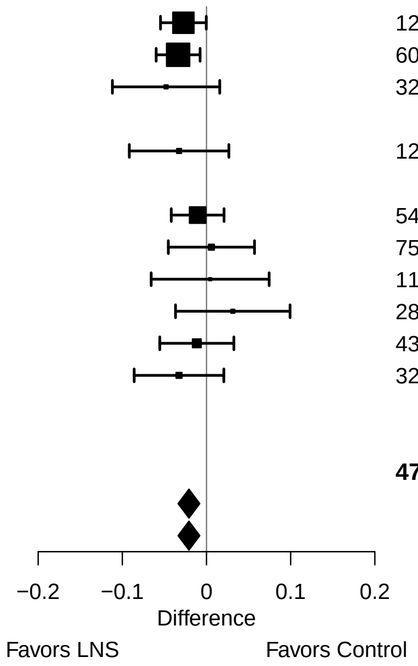

### Supplemental figure 7F: Social-emotional lowest decile prevalence difference

## 7F4: Stratified by Maternal education

**P−for−interaction = 0.125**
**Difference in PDs = −0.02 (−0.04, 0.00)**

| Country | Trial | Tool | LNS N | Control N | Control Prevalence | Primary or greater PD (95% CI) | Fixed W | Random W | LNS N | Control N | Control Prevalence | Incomplete or no formal PD (95% CI) | Fixed W | Random W |
|---|---|---|---|---|---|---|---|---|---|---|---|---|---|---|
| Bangladesh | JiVitA−4 (21) | | | | | | | | | | | | | |
| Bangladesh | RDNS (22) | DMC | 1241 | 589 | 9.3 | −0.02 (−0.05, 0.01) | 0.20 | 0.22 | 416 | 226 | 16.8 | 0.00 (−0.06, 0.06) | 0.08 | 0.08 |
| Bangladesh | WASH−B (23) | EASQ | 750 | 2339 | 9.1 | −0.02 (−0.04, 0.01) | 0.26 | 0.25 | 316 | 927 | 14.1 | −0.02 (−0.06, 0.01) | 0.20 | 0.20 |
| Burkina Faso | iLiNS−Zinc (24) | DMC | | | | | | | | | | | | |
| Ghana | GHANA (25) | | | | | | | | | | | | | |
| Ghana | iLiNS−DYAD−G (26) | PSED | 257 | 518 | 10.0 | 0.00 (−0.04, 0.05) | 0.08 | 0.11 | 75 | 139 | 10.1 | −0.02 (−0.10, 0.06) | 0.04 | 0.04 |
| Haiti | HAITI (27) | | | | | | | | | | | | | |
| Kenya | WASH−B (28) | EASQ | 658 | 2258 | 7.4 | −0.01 (−0.03, 0.01) | 0.40 | 0.30 | 702 | 2484 | 13.0 | −0.02 (−0.05, 0.02) | 0.29 | 0.29 |
| Madagascar | MAHAY (29) | ASQI | 339 | 416 | 5.8 | 0.05 (−0.01, 0.10) | 0.05 | 0.09 | 1274 | 1188 | 10.5 | 0.00 (−0.04, 0.05) | 0.14 | 0.14 |
| Malawi | iLiNS−DYAD−M (30) | PSED | | | | | | | | | | | | |
| Malawi | iLiNS−DOSE (31) | PSED | | | | | | | | | | | | |
| Mali | PROMIS CS (32) | DMC | 100 | 94 | 7.4 | 0.02 (−0.09, 0.12) | 0.01 | 0.03 | 826 | 850 | 11.8 | −0.03 (−0.06, 0.00) | 0.25 | 0.25 |
| Zimbabwe | SHINE (HIV−) (33) | MDAT | | | | | | | | | | | | |
| Zimbabwe | SHINE (HIV+) (34) | MDAT | | | | | | | | | | | | |
| | | | **3345** | **6214** | | **I² = 0.22, Tau² = 0.00** | | | **3609** | **5814** | | **I² = 0.00, Tau² = 0.00** | | |
| **Fixed** | | | | | | **−0.01 (−0.02, 0.00)** | | | | | | **−0.02 (−0.04, 0.00)** | | |
| **Random** | | | | | | **−0.01 (−0.02, 0.01)** | | | | | | **−0.02 (−0.04, 0.00)** | | |

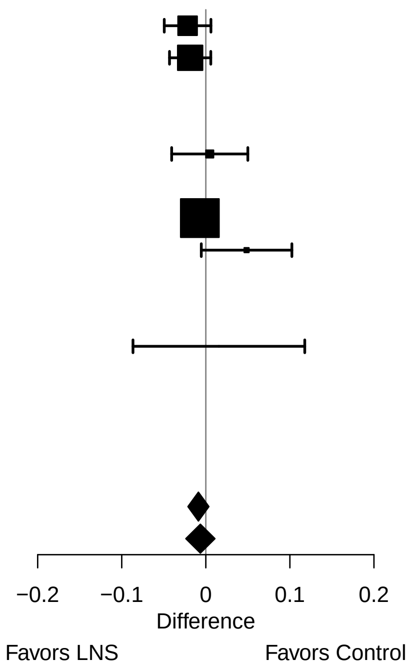


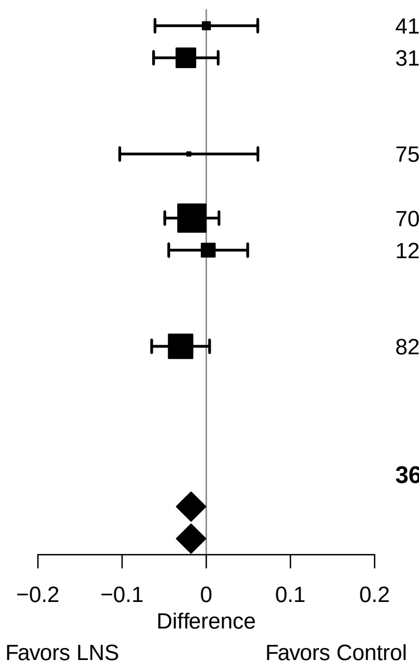

## Supplemental figure 7F: Social-emotional lowest decile prevalence difference

### 7F5: Stratified by Maternal depressive symptoms

**P−for−interaction = 0.525**
**Difference in PDs = −0.01 (−0.03, 0.01)**

| Country | Trial | Tool | Less than 75th percentile: LNS N | Control N | Control Prevalence | PD (95% CI) | Fixed W | Random W | At least 75th percentile: LNS N | Control N | Control Prevalence | PD (95% CI) | Fixed W | Random W |
|---|---|---|---|---|---|---|---|---|---|---|---|---|---|---|
| Bangladesh | JiVitA−4 (21) | | | | | | | | | | | | | |
| Bangladesh | RDNS (22) | DMC | 1075 | 472 | 10.8 | −0.03 (−0.05, 0.00) | 0.20 | 0.20 | 520 | 288 | 12.2 | 0.00 (−0.05, 0.05) | 0.16 | 0.16 |
| Bangladesh | WASH−B (23) | EASQ | 822 | 2356 | 9.6 | −0.02 (−0.04, 0.00) | 0.24 | 0.24 | 223 | 830 | 13.1 | −0.01 (−0.06, 0.03) | 0.16 | 0.16 |
| Burkina Faso | iLiNS−Zinc (24) | DMC | | | | | | | | | | | | |
| Ghana | GHANA (25) | | | | | | | | | | | | | |
| Ghana | iLiNS−DYAD−G (26) | PSED | 242 | 435 | 11.0 | −0.02 (−0.07, 0.02) | 0.06 | 0.06 | 78 | 201 | 9.0 | 0.03 (−0.05, 0.10) | 0.06 | 0.06 |
| Haiti | HAITI (27) | | | | | | | | | | | | | |
| Kenya | WASH−B (28) | EASQ | 960 | 3317 | 10.0 | −0.01 (−0.03, 0.02) | 0.28 | 0.28 | 317 | 1156 | 7.7 | −0.02 (−0.05, 0.01) | 0.38 | 0.38 |
| Madagascar | MAHAY (29) | ASQI | 615 | 667 | 10.5 | −0.02 (−0.07, 0.04) | 0.04 | 0.04 | 268 | 242 | 9.1 | 0.00 (−0.05, 0.06) | 0.12 | 0.12 |
| Malawi | iLiNS−DYAD−M (30) | PSED | 153 | 302 | 9.9 | −0.03 (−0.08, 0.03) | 0.04 | 0.04 | 47 | 111 | 9.0 | 0.08 (−0.03, 0.19) | 0.03 | 0.03 |
| Malawi | iLiNS−DOSE (31) | PSED | | | | | | | | | | | | |
| Mali | PROMIS CS (32) | DMC | | | | | | | | | | | | |
| Zimbabwe | SHINE (HIV−) (33) | MDAT | 583 | 560 | 11.1 | −0.03 (−0.07, 0.00) | 0.11 | 0.11 | 197 | 180 | 16.7 | −0.06 (−0.13, 0.01) | 0.08 | 0.08 |
| Zimbabwe | SHINE (HIV+) (34) | MDAT | 131 | 110 | 13.6 | −0.09 (−0.16, −0.02) | 0.03 | 0.03 | 33 | 32 | 15.6 | 0.00 (−0.17, 0.17) | 0.01 | 0.01 |
| | | | **4581** | **8219** | | **I² = 0.00, Tau² = 0.00** | | | **1683** | **3040** | | **I² = 0.00, Tau² = 0.00** | | |
| **Fixed** | | | | | | **−0.02 (−0.03, −0.01)** | | | | | | **−0.01 (−0.03, 0.01)** | | |
| **Random** | | | | | | **−0.02 (−0.03, −0.01)** | | | | | | **−0.01 (−0.03, 0.01)** | | |

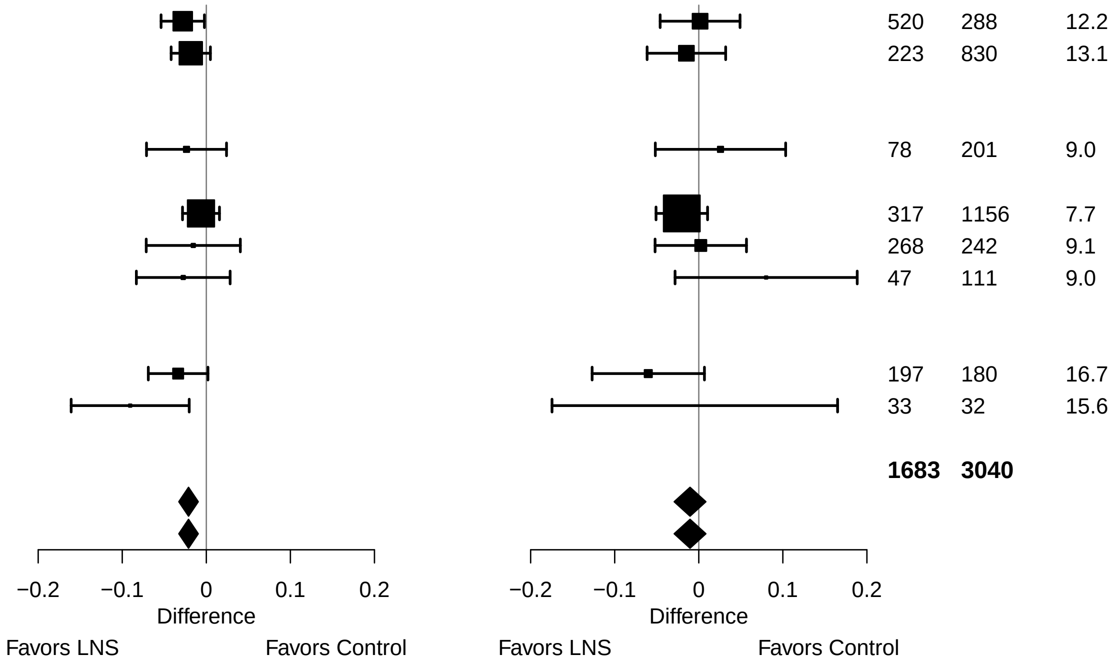

## Supplemental figure 7F: Social-emotional lowest decile prevalence difference

## 7F6: Stratified by Child sex

**P−for−interaction = 0.852**
**Difference in PDs = 0.00 (−0.02, 0.02)**

| Country | Trial | Tool | Male LNS N | Male Control N | Male Control Prevalence | Male PD (95% CI) | Male Fixed W | Male Random W | Female LNS N | Female Control N | Female Control Prevalence | Female PD (95% CI) | Female Fixed W | Female Random W |
|---|---|---|---|---|---|---|---|---|---|---|---|---|---|---|
| Bangladesh | JiVitA−4 (21) | | | | | | | | | | | | | |
| Bangladesh | RDNS (22) | DMC | 830 | 411 | 9.0 | 0.00 (−0.03, 0.03) | 0.15 | 0.11 | 827 | 404 | 13.9 | −0.04 (−0.08, 0.00) | 0.11 | 0.11 |
| Bangladesh | WASH−B (23) | EASQ | 522 | 1635 | 10.4 | −0.02 (−0.05, 0.01) | 0.18 | 0.12 | 544 | 1631 | 10.6 | −0.02 (−0.05, 0.02) | 0.18 | 0.13 |
| Burkina Faso | iLiNS−Zinc (24) | DMC | 372 | 193 | 13.5 | −0.08 (−0.12, −0.03) | 0.08 | 0.10 | 374 | 182 | 19.8 | −0.12 (−0.19, −0.05) | 0.03 | 0.06 |
| Ghana | GHANA (25) | | | | | | | | | | | | | |
| Ghana | iLiNS−DYAD−G (26) | PSED | 164 | 306 | 7.8 | 0.03 (−0.03, 0.08) | 0.05 | 0.08 | 168 | 351 | 12.0 | −0.02 (−0.08, 0.03) | 0.05 | 0.08 |
| Haiti | HAITI (27) | | | | | | | | | | | | | |
| Kenya | WASH−B (28) | EASQ | 682 | 2260 | 10.8 | 0.00 (−0.03, 0.02) | 0.23 | 0.12 | 680 | 2485 | 9.9 | −0.02 (−0.05, 0.01) | 0.24 | 0.14 |
| Madagascar | MAHAY (29) | ASQI | 795 | 787 | 11.1 | −0.01 (−0.05, 0.04) | 0.07 | 0.09 | 818 | 817 | 7.6 | 0.04 (−0.01, 0.08) | 0.08 | 0.10 |
| Malawi | iLiNS−DYAD−M (30) | PSED | 105 | 205 | 12.2 | −0.05 (−0.12, 0.03) | 0.03 | 0.06 | 110 | 233 | 8.6 | 0.04 (−0.03, 0.11) | 0.04 | 0.07 |
| Malawi | iLiNS−DOSE (31) | PSED | 320 | 117 | 6.8 | 0.05 (−0.01, 0.12) | 0.03 | 0.07 | 324 | 104 | 12.5 | −0.04 (−0.10, 0.03) | 0.04 | 0.07 |
| Mali | PROMIS CS (32) | DMC | 469 | 514 | 10.7 | −0.03 (−0.06, 0.01) | 0.11 | 0.11 | 458 | 430 | 12.1 | −0.03 (−0.07, 0.02) | 0.10 | 0.11 |
| Zimbabwe | SHINE (HIV−) (33) | MDAT | 424 | 388 | 13.1 | −0.04 (−0.09, 0.00) | 0.06 | 0.09 | 417 | 411 | 10.7 | −0.03 (−0.07, 0.02) | 0.10 | 0.11 |
| Zimbabwe | SHINE (HIV+) (34) | MDAT | 84 | 79 | 20.3 | −0.13 (−0.22, −0.04) | 0.02 | 0.05 | 86 | 72 | 9.7 | −0.04 (−0.14, 0.07) | 0.02 | 0.03 |
| | | | **4767** | **6895** | | **I² = 0.59, Tau² = 0.00** | | | **4806** | **7120** | | **I² = 0.43, Tau² = 0.00** | | |
| **Fixed** | | | | | | **−0.02 (−0.03, −0.01)** | | | | | | **−0.02 (−0.03, −0.01)** | | |
| **Random** | | | | | | **−0.02 (−0.05, 0.00)** | | | | | | **−0.02 (−0.04, 0.00)** | | |

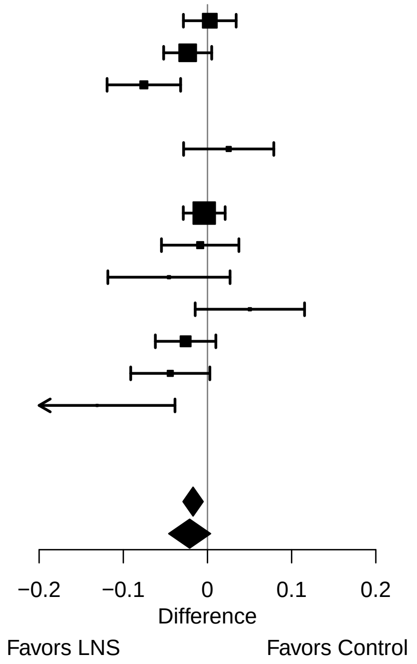


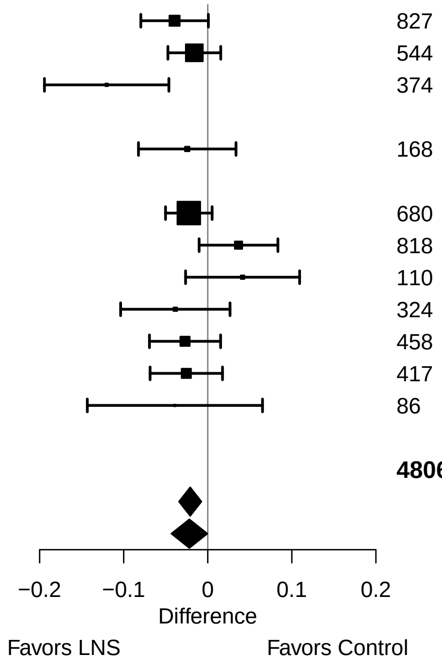

**Supplemental figure 7F: Social-emotional lowest decile prevalence difference**

## 7F7: Stratified by Child birth order

**P−for−interaction = 0.909**
**Difference in PDs = 0.00 (−0.02, 0.02)**

| Country | Trial | Tool | Later born LNS N | Later born Control N | Later born Control Prevalence | Later born PD (95% CI) | Later born Fixed W | Later born Random W | Firstborn LNS N | Firstborn Control N | Firstborn Control Prevalence | Firstborn PD (95% CI) | Firstborn Fixed W | Firstborn Random W |
|---|---|---|---|---|---|---|---|---|---|---|---|---|---|---|
| Bangladesh | JiVitA−4 (21) | | | | | | | | | | | | | |
| Bangladesh | RDNS (22) | DMC | 969 | 508 | 12.4 | −0.02 (−0.06, 0.01) | 0.09 | 0.11 | 687 | 307 | 9.8 | −0.01 (−0.04, 0.02) | 0.27 | 0.27 |
| Bangladesh | WASH−B (23) | EASQ | 669 | 2134 | 10.8 | −0.01 (−0.04, 0.01) | 0.18 | 0.13 | 377 | 1031 | 9.0 | −0.02 (−0.05, 0.01) | 0.25 | 0.25 |
| Burkina Faso | iLiNS−Zinc (24) | DMC | 587 | 290 | 17.9 | −0.12 (−0.17, −0.07) | 0.05 | 0.09 | 159 | 85 | 11.8 | −0.02 (−0.12, 0.07) | 0.03 | 0.03 |
| Ghana | GHANA (25) | | | | | | | | | | | | | |
| Ghana | iLiNS−DYAD−G (26) | PSED | 225 | 444 | 9.7 | 0.02 (−0.03, 0.07) | 0.05 | 0.10 | 107 | 213 | 10.8 | −0.05 (−0.12, 0.01) | 0.06 | 0.06 |
| Haiti | HAITI (27) | | | | | | | | | | | | | |
| Kenya | WASH−B (28) | EASQ | 1071 | 3767 | 10.4 | −0.01 (−0.03, 0.01) | 0.29 | 0.14 | 289 | 974 | 10.1 | −0.02 (−0.06, 0.02) | 0.18 | 0.18 |
| Madagascar | MAHAY (29) | ASQI | 1162 | 1118 | 8.9 | 0.01 (−0.03, 0.06) | 0.07 | 0.10 | 433 | 465 | 10.8 | 0.01 (−0.06, 0.07) | 0.06 | 0.06 |
| Malawi | iLiNS−DYAD−M (30) | PSED | 170 | 349 | 10.9 | −0.02 (−0.08, 0.03) | 0.04 | 0.09 | 45 | 87 | 8.0 | 0.08 (−0.04, 0.19) | 0.02 | 0.02 |
| Malawi | iLiNS−DOSE (31) | PSED | | | | | | | | | | | | |
| Mali | PROMIS CS (32) | DMC | 770 | 811 | 11.2 | −0.03 (−0.06, 0.01) | 0.12 | 0.12 | 142 | 122 | 9.0 | 0.01 (−0.06, 0.08) | 0.05 | 0.05 |
| Zimbabwe | SHINE (HIV−) (33) | MDAT | 602 | 574 | 11.5 | −0.03 (−0.07, 0.00) | 0.11 | 0.12 | 203 | 191 | 14.7 | −0.06 (−0.12, 0.00) | 0.07 | 0.07 |
| Zimbabwe | SHINE (HIV+) (34) | MDAT | | | | | | | | | | | | |
| | | | **6225** | **9995** | | **I² = 0.61, Tau² = 0.00** | | | **2442** | **3475** | | **I² = 0.00, Tau² = 0.00** | | |
| **Fixed** | | | | | | **−0.02 (−0.03, −0.01)** | | | | | | **−0.02 (−0.03, 0.00)** | | |
| **Random** | | | | | | **−0.02 (−0.05, 0.00)** | | | | | | **−0.02 (−0.03, 0.00)** | | |

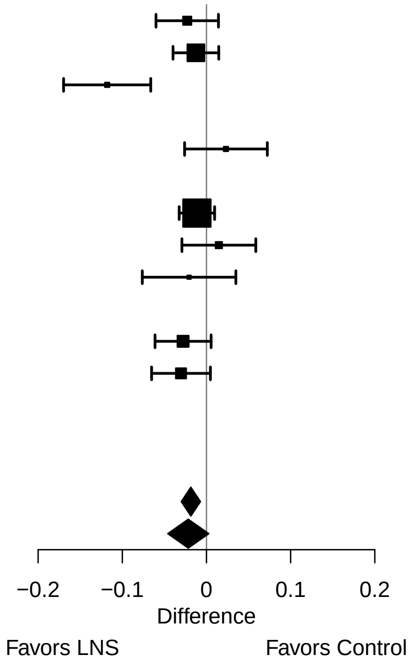


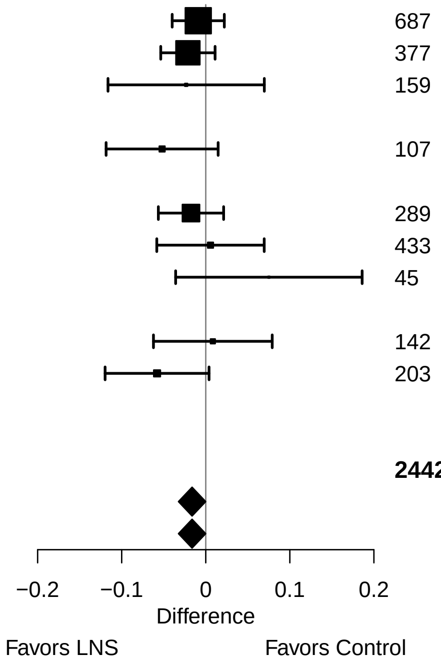

**Supplemental figure 7F: Social-emotional lowest decile prevalence difference**

## 7F8: Stratified by Child baseline stunting

**P−for−interaction = 0.202**
**Difference in PDs = −0.03 (−0.08, 0.02)**

| | | | | | | No | | | | | | Yes | | |
|---|---|---|---|---|---|---|---|---|---|---|---|---|---|---|
| **Country** | **Trial** | **Tool** | **LNS N** | **Control N** | **Control Prevalence** | **PD (95% CI)** | **Fixed W** | **Random W** | **LNS N** | **Control N** | **Control Prevalence** | **PD (95% CI)** | **Fixed W** | **Random W** |
| Bangladesh | JiVitA−4 (21) | | | | | | | | | | | | | |
| Bangladesh | RDNS (22) | DMC | 1241 | 574 | 8.9 | 0.00 (−0.03, 0.02) | 0.48 | 0.31 | 354 | 185 | 18.9 | −0.06 (−0.13, 0.01) | 0.36 | 0.28 |
| Bangladesh | WASH−B (23) | EASQ | | | | | | | | | | | | |
| Burkina Faso | iLiNS−Zinc (24) | DMC | 576 | 294 | 13.3 | −0.08 (−0.13, −0.03) | 0.14 | 0.22 | 170 | 80 | 27.5 | −0.16 (−0.25, −0.06) | 0.19 | 0.23 |
| Ghana | GHANA (25) | | | | | | | | | | | | | |
| Ghana | iLiNS−DYAD−G (26) | PSED | | | | | | | | | | | | |
| Haiti | HAITI (27) | | | | | | | | | | | | | |
| Kenya | WASH−B (28) | EASQ | | | | | | | | | | | | |
| Madagascar | MAHAY (29) | ASQI | | | | | | | | | | | | |
| Malawi | iLiNS−DYAD−M (30) | PSED | | | | | | | | | | | | |
| Malawi | iLiNS−DOSE (31) | PSED | 464 | 147 | 8.8 | 0.01 (−0.05, 0.06) | 0.12 | 0.20 | 180 | 74 | 10.8 | 0.01 (−0.08, 0.09) | 0.23 | 0.24 |
| Mali | PROMIS CS (32) | DMC | | | | | | | | | | | | |
| Zimbabwe | SHINE (HIV−) (33) | MDAT | 532 | 490 | 10.8 | −0.04 (−0.08, −0.01) | 0.26 | 0.27 | 122 | 105 | 14.3 | −0.02 (−0.11, 0.07) | 0.22 | 0.24 |
| Zimbabwe | SHINE (HIV+) (34) | MDAT | | | | | | | | | | | | |
| | | | **2813** | **1505** | | **$I^2$ = 0.68, $Tau^2$ = 0.00** | | | **826** | **444** | | **$I^2$ = 0.59, $Tau^2$ = 0.00** | | |
| **Fixed** | | | | | | **−0.02 (−0.04, −0.01)** | | | | | | **−0.05 (−0.10, −0.01)** | | |
| **Random** | | | | | | **−0.03 (−0.07, 0.01)** | | | | | | **−0.06 (−0.13, 0.01)** | | |

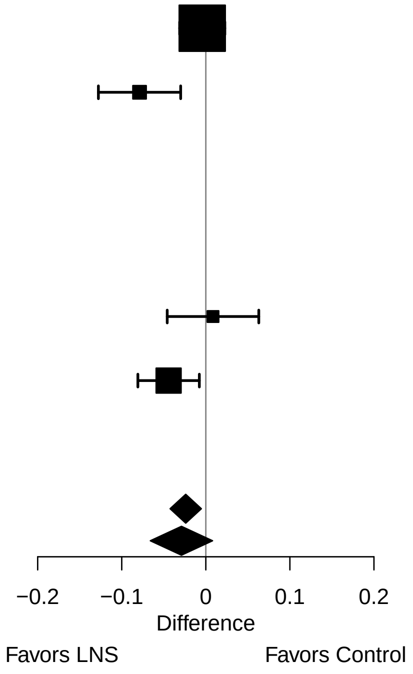


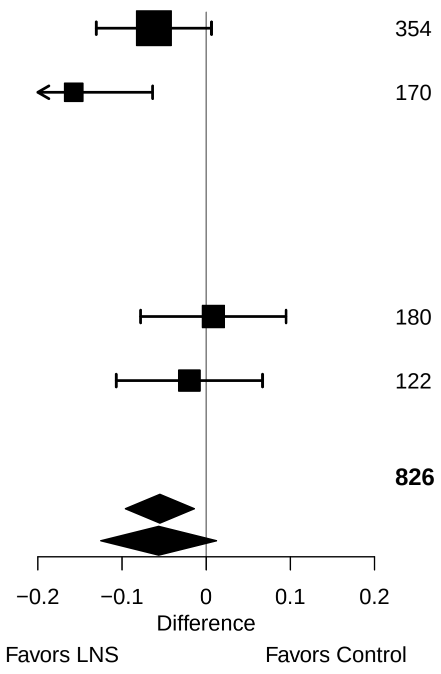

**Supplemental figure 7F: Social-emotional lowest decile prevalence difference**

## 7F9: Stratified by Child baseline acute malnutrition (insufficient comparisons)

## Supplemental figure 7F: Social-emotional lowest decile prevalence difference

### 7F10: Stratified by Child baseline anemia

**P−for−interaction = 0.952**
**Difference in PDs = 0.00 (−0.05, 0.05)**

| Country | Trial | Tool | LNS N | Control N | Control Prevalence | Not anemic PD (95% CI) | Fixed W | Random W | LNS N | Control N | Control Prevalence | Anemic PD (95% CI) | Fixed W | Random W |
|---|---|---|---|---|---|---|---|---|---|---|---|---|---|---|
| Bangladesh | JiVitA−4 (21) | | | | | | | | | | | | | |
| Bangladesh | RDNS (22) | DMC | 212 | 96 | 14.6 | −0.08 (−0.16, 0.01) | 0.17 | 0.19 | 316 | 160 | 12.5 | −0.03 (−0.08, 0.02) | 0.35 | 0.35 |
| Bangladesh | WASH−B (23) | EASQ | | | | | | | | | | | | |
| Burkina Faso | iLiNS−Zinc (24) | DMC | | | | | | | | | | | | |
| Ghana | GHANA (25) | | | | | | | | | | | | | |
| Ghana | iLiNS−DYAD−G (26) | PSED | 193 | 350 | 10.3 | 0.00 (−0.05, 0.05) | 0.40 | 0.37 | 90 | 204 | 9.8 | −0.01 (−0.08, 0.06) | 0.18 | 0.18 |
| Haiti | HAITI (27) | | | | | | | | | | | | | |
| Kenya | WASH−B (28) | EASQ | | | | | | | | | | | | |
| Madagascar | MAHAY (29) | ASQI | | | | | | | | | | | | |
| Malawi | iLiNS−DYAD−M (30) | PSED | 66 | 147 | 8.2 | −0.01 (−0.08, 0.07) | 0.19 | 0.20 | 138 | 275 | 10.9 | 0.01 (−0.06, 0.07) | 0.23 | 0.23 |
| Malawi | iLiNS−DOSE (31) | PSED | 223 | 94 | 7.4 | 0.02 (−0.05, 0.09) | 0.24 | 0.25 | 417 | 126 | 11.1 | −0.01 (−0.07, 0.05) | 0.25 | 0.25 |
| Mali | PROMIS CS (32) | DMC | | | | | | | | | | | | |
| Zimbabwe | SHINE (HIV−) (33) | MDAT | | | | | | | | | | | | |
| Zimbabwe | SHINE (HIV+) (34) | MDAT | | | | | | | | | | | | |
| | | | **694** | **687** | | **$I^2$ = 0.14, $Tau^2$ = 0.00** | | | **961** | **765** | | **$I^2$ = 0.00, $Tau^2$ = 0.00** | | |
| **Fixed** | | | | | | **−0.01 (−0.04, 0.03)** | | | | | | **−0.01 (−0.04, 0.02)** | | |
| **Random** | | | | | | **−0.01 (−0.05, 0.03)** | | | | | | **−0.01 (−0.04, 0.02)** | | |

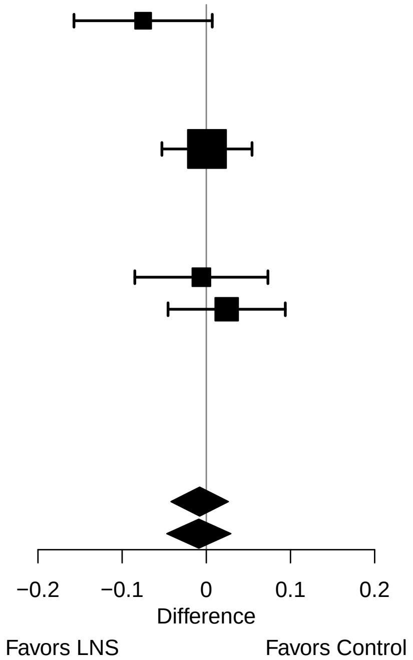


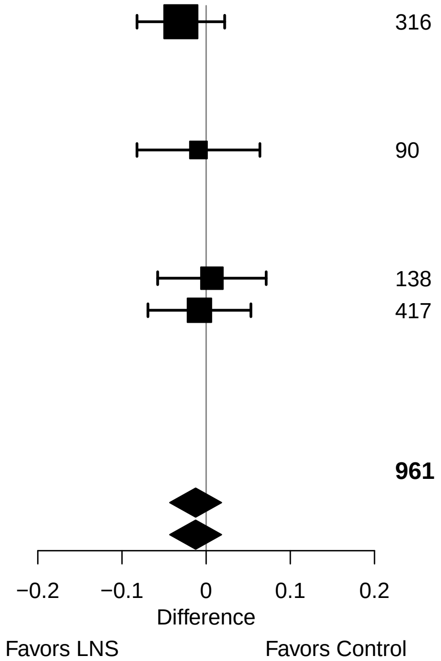

# Supplemental figure 7G: Mean difference in motor z-score

## 7G1: Stratified by Maternal height

**P−for−interaction = 0.457**
**Difference in MDs = −0.03 (−0.10, 0.05)**

| | | | At least 150.1 cm | | | | | | Less than 150.1 cm | | | | | |
|---|---|---|---|---|---|---|---|---|---|---|---|---|---|---|
| **Country** | **Trial** | **Tool** | **LNS N** | **Control N** | **Control Mean** | **MD (95% CI)** | **Fixed W** | **Random W** | **LNS N** | **Control N** | **Control Mean** | **MD (95% CI)** | **Fixed W** | **Random W** |
| Bangladesh | JiVitA−4 (21) | BSID−III | | | | | | | | | | | | |
| Bangladesh | RDNS (22) | DMC | 818 | 399 | −0.04 | 0.16 (0.08, 0.24) | 0.20 | 0.12 | 684 | 333 | −0.06 | 0.04 (−0.06, 0.15) | 0.36 | 0.36 |
| Bangladesh | WASH−B (23) | EASQ | 568 | 1773 | 0.04 | 0.09 (−0.02, 0.19) | 0.12 | 0.11 | 501 | 1467 | −0.09 | 0.13 (0.03, 0.23) | 0.42 | 0.42 |
| Burkina Faso | iLiNS−Zinc (24) | DMC | 727 | 365 | −0.25 | 0.40 (0.23, 0.57) | 0.04 | 0.08 | 17 | 10 | −0.63 | 0.39 (−0.48, 1.26) | 0.01 | 0.01 |
| Ghana | GHANA (25) | | | | | | | | | | | | | |
| Ghana | iLiNS−DYAD−G (26) | KDI | 284 | 557 | 0.02 | 0.01 (−0.13, 0.14) | 0.07 | 0.09 | 13 | 34 | −0.46 | 0.30 (−0.62, 1.23) | 0.00 | 0.00 |
| Haiti | HAITI (27) | | | | | | | | | | | | | |
| Kenya | WASH−B (28) | EASQ | 1237 | 4372 | 0.01 | 0.00 (−0.07, 0.06) | 0.29 | 0.13 | 51 | 175 | −0.21 | 0.27 (−0.01, 0.56) | 0.05 | 0.05 |
| Madagascar | MAHAY (29) | ASQI | 956 | 986 | −0.05 | 0.09 (−0.13, 0.30) | 0.03 | 0.06 | 553 | 538 | 0.06 | 0.03 (−0.19, 0.25) | 0.08 | 0.08 |
| Malawi | iLiNS−DYAD−M (30) | KDI | 186 | 376 | −0.03 | 0.04 (−0.13, 0.20) | 0.04 | 0.08 | 28 | 57 | 0.16 | −0.04 (−0.49, 0.42) | 0.02 | 0.02 |
| Malawi | iLiNS−DOSE (31) | KDI | 543 | 185 | −0.01 | 0.04 (−0.13, 0.20) | 0.04 | 0.08 | 102 | 35 | −0.17 | 0.10 (−0.30, 0.50) | 0.03 | 0.03 |
| Mali | PROMIS CS (32) | DMC | 865 | 889 | −0.07 | 0.14 (−0.03, 0.31) | 0.04 | 0.08 | 32 | 26 | −0.01 | −0.04 (−0.60, 0.53) | 0.01 | 0.01 |
| Zimbabwe | SHINE (HIV−) (33) | MDAT | 788 | 762 | −0.06 | 0.11 (0.00, 0.22) | 0.10 | 0.11 | 34 | 22 | −0.15 | 0.11 (−0.35, 0.57) | 0.02 | 0.02 |
| Zimbabwe | SHINE (HIV+) (34) | MDAT | 158 | 140 | −0.14 | 0.28 (0.06, 0.51) | 0.02 | 0.06 | 10 | 6 | −0.36 | 0.42 (−0.76, 1.60) | 0.00 | 0.00 |
| | | | **7130** | **10804** | | **I² = 0.67, Tau² = 0.01** | | | **2025** | **2703** | | **I² = 0.00, Tau² = 0.00** | | |
| **Fixed** | | | | | | **0.09 (0.05, 0.12)** | | | | | | **0.09 (0.03, 0.16)** | | |
| **Random** | | | | | | **0.11 (0.04, 0.18)** | | | | | | **0.09 (0.03, 0.16)** | | |

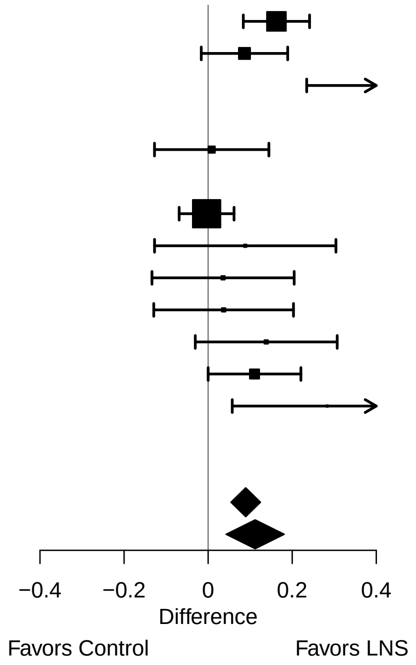


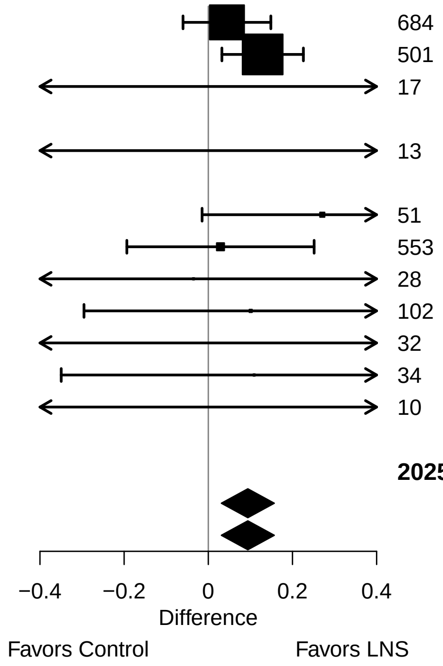

## Supplemental figure 7G: Mean difference in motor z-score

## 7G2: Stratified by Maternal BMI

**P−for−interaction = 0.553**
**Difference in MDs = 0.02 (−0.05, 0.09)**

| | | | At least 20 kg/m² | | | | | | Less than 20 kg/m² | | | | | |
|---|---|---|---|---|---|---|---|---|---|---|---|---|---|---|
| **Country** | **Trial** | **Tool** | **LNS N** | **Control N** | **Control Mean** | **MD (95% CI)** | **Fixed W** | **Random W** | **LNS N** | **Control N** | **Control Mean** | **MD (95% CI)** | **Fixed W** | **Random W** |
| Bangladesh | JiVitA−4 (21) | BSID−III | | | | | | | | | | | | |
| Bangladesh | RDNS (22) | DMC | 689 | 306 | −0.01 | 0.10 (−0.01, 0.20) | 0.13 | 0.12 | 813 | 426 | −0.08 | 0.11 (0.03, 0.20) | 0.36 | 0.20 |
| Bangladesh | WASH−B (23) | EASQ | 478 | 1502 | 0.05 | 0.09 (−0.02, 0.19) | 0.14 | 0.13 | 591 | 1737 | −0.09 | 0.12 (0.03, 0.22) | 0.28 | 0.19 |
| Burkina Faso | iLiNS−Zinc (24) | DMC | 480 | 225 | −0.13 | 0.33 (0.14, 0.52) | 0.05 | 0.08 | 264 | 150 | −0.46 | 0.49 (0.23, 0.75) | 0.04 | 0.08 |
| Ghana | GHANA (25) | | | | | | | | | | | | | |
| Ghana | iLiNS−DYAD−G (26) | KDI | 262 | 485 | 0.00 | 0.04 (−0.10, 0.19) | 0.07 | 0.10 | 35 | 106 | −0.05 | −0.10 (−0.50, 0.29) | 0.02 | 0.04 |
| Haiti | HAITI (27) | | | | | | | | | | | | | |
| Kenya | WASH−B (28) | EASQ | 1031 | 3535 | 0.01 | −0.02 (−0.09, 0.05) | 0.33 | 0.15 | 257 | 1012 | −0.04 | 0.12 (−0.02, 0.27) | 0.13 | 0.15 |
| Madagascar | MAHAY (29) | ASQI | | | | | | | | | | | | |
| Malawi | iLiNS−DYAD−M (30) | KDI | 126 | 258 | −0.04 | 0.06 (−0.16, 0.28) | 0.03 | 0.07 | 87 | 175 | 0.05 | −0.03 (−0.24, 0.19) | 0.05 | 0.10 |
| Malawi | iLiNS−DOSE (31) | KDI | 480 | 158 | 0.01 | 0.02 (−0.16, 0.19) | 0.05 | 0.09 | 163 | 61 | −0.16 | 0.13 (−0.19, 0.45) | 0.03 | 0.06 |
| Mali | PROMIS CS (32) | DMC | 665 | 653 | −0.08 | 0.18 (0.00, 0.36) | 0.05 | 0.09 | 232 | 262 | −0.04 | 0.00 (−0.22, 0.22) | 0.05 | 0.10 |
| Zimbabwe | SHINE (HIV−) (33) | MDAT | 601 | 581 | −0.05 | 0.13 (0.01, 0.25) | 0.11 | 0.12 | 100 | 96 | −0.22 | 0.21 (−0.08, 0.51) | 0.03 | 0.07 |
| Zimbabwe | SHINE (HIV+) (34) | MDAT | 124 | 113 | −0.11 | 0.36 (0.13, 0.60) | 0.03 | 0.06 | 25 | 23 | −0.31 | −0.13 (−0.68, 0.42) | 0.01 | 0.02 |
| | | | **4936** | **7816** | | **I² = 0.62, Tau² = 0.01** | | | **2567** | **4048** | | **I² = 0.31, Tau² = 0.01** | | |
| **Fixed** | | | | | | **0.07 (0.03, 0.11)** | | | | | | **0.12 (0.06, 0.17)** | | |
| **Random** | | | | | | **0.11 (0.04, 0.18)** | | | | | | **0.11 (0.03, 0.20)** | | |

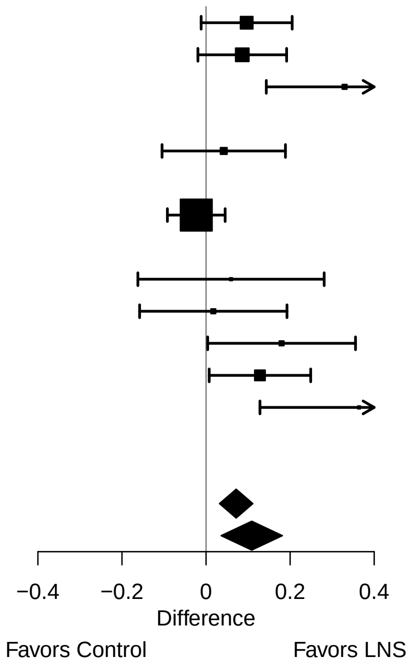


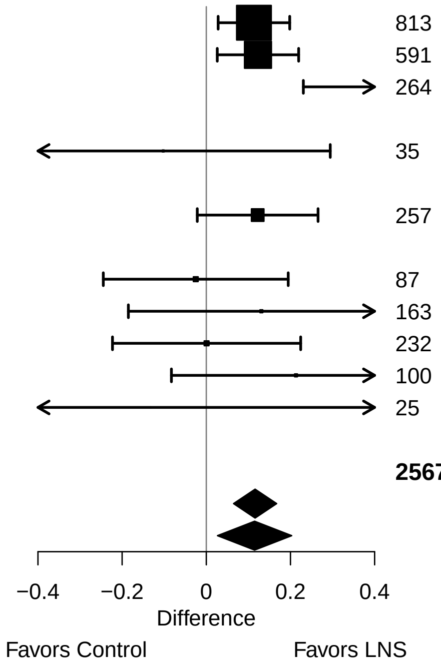

## Supplemental figure 7G: Mean difference in motor z-score

## 7G3: Stratified by Maternal age

**P−for−interaction = 0.043**
**Difference in MDs = −0.06 (−0.11, 0.00)**

| | | | At least 25 y | | | | | | | Less than 25 y | | | | | |
|---|---|---|---|---|---|---|---|---|---|---|---|---|---|---|---|
| **Country** | **Trial** | **Tool** | **LNS N** | **Control N** | **Control Mean** | **MD (95% CI)** | **Fixed W** | **Random W** | **LNS N** | **Control N** | **Control Mean** | **MD (95% CI)** | **Fixed W** | **Random W** |
| Bangladesh | JiVitA−4 (21) | BSID−III | 200 | 68 | 0.06 | −0.06 (−0.27, 0.15) | 0.04 | 0.07 | 242 | 74 | 0.01 | −0.02 (−0.30, 0.27) | 0.02 | 0.02 |
| Bangladesh | RDNS (22) | DMC | 424 | 200 | −0.11 | 0.15 (0.00, 0.30) | 0.08 | 0.09 | 1132 | 553 | −0.02 | 0.08 (0.00, 0.15) | 0.31 | 0.31 |
| Bangladesh | WASH−B (23) | EASQ | 466 | 1450 | −0.02 | 0.09 (−0.03, 0.20) | 0.14 | 0.10 | 603 | 1815 | −0.03 | 0.11 (0.03, 0.20) | 0.23 | 0.23 |
| Burkina Faso | iLiNS−Zinc (24) | DMC | 421 | 232 | −0.33 | 0.53 (0.36, 0.70) | 0.06 | 0.08 | 321 | 141 | −0.14 | 0.20 (−0.04, 0.44) | 0.03 | 0.03 |
| Ghana | GHANA (25) | | | | | | | | | | | | | |
| Ghana | iLiNS−DYAD−G (26) | KDI | 192 | 378 | −0.01 | 0.11 (−0.06, 0.27) | 0.07 | 0.09 | 110 | 223 | 0.01 | −0.11 (−0.35, 0.13) | 0.03 | 0.03 |
| Haiti | HAITI (27) | | | | | | | | | | | | | |
| Kenya | WASH−B (28) | EASQ | 798 | 2622 | 0.04 | −0.02 (−0.10, 0.05) | 0.30 | 0.11 | 549 | 2092 | −0.05 | 0.05 (−0.05, 0.16) | 0.16 | 0.16 |
| Madagascar | MAHAY (29) | ASQI | 852 | 901 | −0.09 | 0.08 (−0.12, 0.29) | 0.04 | 0.07 | 759 | 703 | 0.04 | 0.06 (−0.16, 0.27) | 0.04 | 0.04 |
| Malawi | iLiNS−DYAD−M (30) | KDI | 102 | 224 | 0.01 | 0.12 (−0.10, 0.34) | 0.04 | 0.07 | 112 | 212 | −0.01 | −0.06 (−0.29, 0.17) | 0.03 | 0.03 |
| Malawi | iLiNS−DOSE (31) | KDI | 346 | 124 | −0.08 | 0.09 (−0.12, 0.30) | 0.04 | 0.07 | 288 | 90 | 0.04 | −0.02 (−0.25, 0.21) | 0.03 | 0.03 |
| Mali | PROMIS CS (32) | DMC | 480 | 479 | −0.08 | 0.21 (0.04, 0.38) | 0.06 | 0.08 | 422 | 442 | −0.05 | 0.04 (−0.17, 0.25) | 0.04 | 0.04 |
| Zimbabwe | SHINE (HIV−) (33) | MDAT | 427 | 389 | −0.03 | 0.18 (0.03, 0.32) | 0.09 | 0.09 | 326 | 321 | −0.11 | 0.05 (−0.10, 0.20) | 0.08 | 0.08 |
| Zimbabwe | SHINE (HIV+) (34) | MDAT | 126 | 111 | −0.12 | 0.28 (0.03, 0.52) | 0.03 | 0.06 | 29 | 26 | −0.20 | 0.40 (−0.24, 1.03) | 0.00 | 0.00 |
| | | | **4834** | **7178** | | **I² = 0.74, Tau² = 0.02** | | | **4893** | **6692** | | **I² = 0.00, Tau² = 0.00** | | |
| **Fixed** | | | | | | **0.10 (0.06, 0.15)** | | | | | | **0.07 (0.03, 0.11)** | | |
| **Random** | | | | | | **0.14 (0.06, 0.23)** | | | | | | **0.07 (0.03, 0.11)** | | |

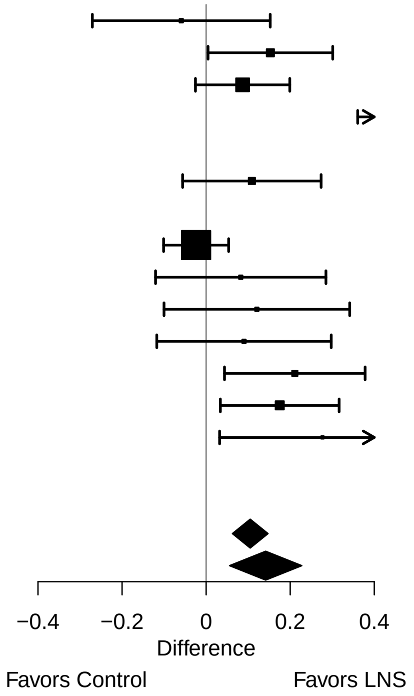


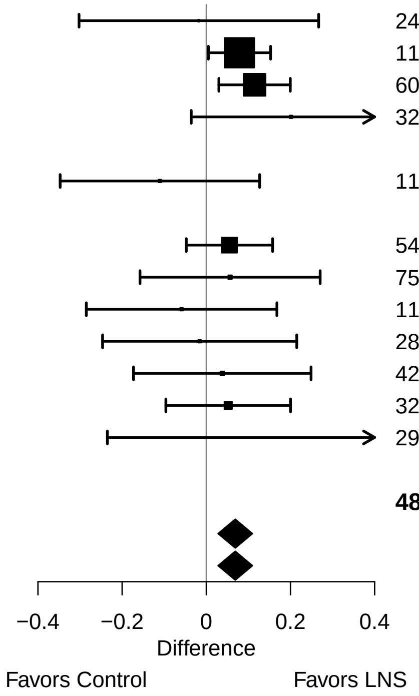

## Supplemental figure 7G: Mean difference in motor z-score

### 7G4: Stratified by Maternal education

**P−for−interaction = 0.015**
**Difference in MDs = 0.09 (0.02, 0.16)**

| Country | Trial | Tool | Primary or greater: LNS N | Control N | Control Mean | MD (95% CI) | Fixed W | Random W | Incomplete or no formal: LNS N | Control N | Control Mean | MD (95% CI) | Fixed W | Random W |
|---|---|---|---|---|---|---|---|---|---|---|---|---|---|---|
| Bangladesh | JiVitA−4 (21) | BSID−III | 292 | 92 | 0.20 | −0.14 (−0.32, 0.05) | 0.04 | 0.08 | 152 | 51 | −0.31 | 0.18 (−0.14, 0.51) | 0.02 | 0.05 |
| Bangladesh | RDNS (22) | DMC | 1166 | 547 | 0.03 | 0.08 (0.02, 0.15) | 0.29 | 0.14 | 390 | 206 | −0.24 | 0.12 (−0.05, 0.28) | 0.09 | 0.11 |
| Bangladesh | WASH−B (23) | EASQ | 755 | 2344 | 0.04 | 0.08 (0.00, 0.17) | 0.21 | 0.14 | 319 | 935 | −0.17 | 0.16 (0.04, 0.29) | 0.16 | 0.13 |
| Burkina Faso | iLiNS−Zinc (24) | DMC | 32 | 9 | −0.22 | 0.41 (−0.18, 1.00) | 0.00 | 0.01 | 712 | 366 | −0.26 | 0.40 (0.23, 0.57) | 0.09 | 0.10 |
| Ghana | GHANA (25) | | | | | | | | | | | | | |
| Ghana | iLiNS−DYAD−G (26) | KDI | 235 | 467 | 0.02 | −0.02 (−0.18, 0.13) | 0.06 | 0.10 | 67 | 134 | −0.09 | 0.22 (−0.09, 0.53) | 0.03 | 0.05 |
| Haiti | HAITI (27) | | | | | | | | | | | | | |
| Kenya | WASH−B (28) | EASQ | 658 | 2258 | 0.11 | −0.01 (−0.10, 0.08) | 0.17 | 0.13 | 702 | 2484 | −0.11 | 0.02 (−0.07, 0.12) | 0.29 | 0.15 |
| Madagascar | MAHAY (29) | ASQI | 339 | 416 | 0.27 | −0.13 (−0.33, 0.08) | 0.03 | 0.07 | 1274 | 1188 | −0.14 | 0.15 (−0.05, 0.36) | 0.06 | 0.09 |
| Malawi | iLiNS−DYAD−M (30) | KDI | 33 | 67 | 0.06 | −0.09 (−0.42, 0.25) | 0.01 | 0.04 | 180 | 366 | −0.02 | 0.04 (−0.14, 0.22) | 0.08 | 0.10 |
| Malawi | iLiNS−DOSE (31) | KDI | 148 | 52 | 0.24 | 0.00 (−0.26, 0.27) | 0.02 | 0.05 | 487 | 162 | −0.12 | 0.06 (−0.12, 0.25) | 0.08 | 0.10 |
| Mali | PROMIS CS (32) | DMC | 99 | 93 | 0.06 | 0.09 (−0.28, 0.46) | 0.01 | 0.03 | 802 | 828 | −0.08 | 0.13 (−0.03, 0.30) | 0.09 | 0.11 |
| Zimbabwe | SHINE (HIV−) (33) | MDAT | 763 | 731 | −0.06 | 0.13 (0.02, 0.24) | 0.12 | 0.12 | 29 | 25 | 0.05 | −0.43 (−0.96, 0.09) | 0.01 | 0.02 |
| Zimbabwe | SHINE (HIV+) (34) | MDAT | 149 | 130 | −0.13 | 0.34 (0.13, 0.55) | 0.03 | 0.07 | 9 | 8 | −0.06 | −0.60 (−1.94, 0.74) | 0.00 | 0.00 |
| | | | **4669** | **7206** | | **I² = 0.50, Tau² = 0.01** | | | **5123** | **6753** | | **I² = 0.49, Tau² = 0.01** | | |
| **Fixed** | | | | | | **0.06 (0.02, 0.09)** | | | | | | **0.11 (0.06, 0.16)** | | |
| **Random** | | | | | | **0.05 (−0.03, 0.12)** | | | | | | **0.12 (0.04, 0.21)** | | |

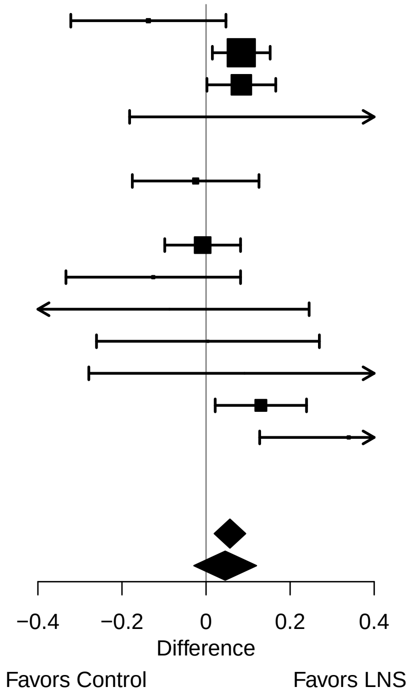


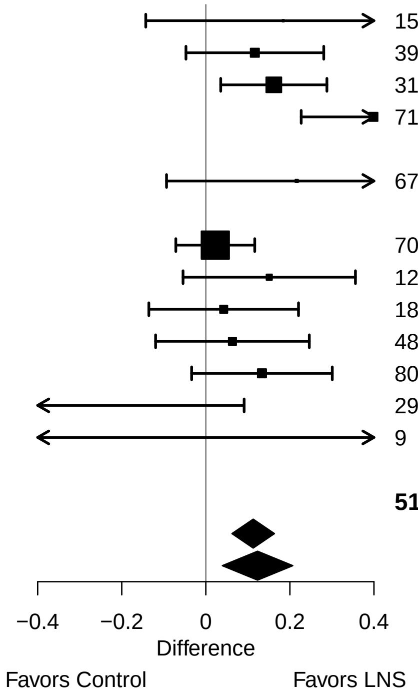

## Supplemental figure 7G: Mean difference in motor z-score

## 7G5: Stratified by Maternal depressive symptoms

**P−for−interaction = 0.823**
**Difference in MDs = −0.01 (−0.07, 0.06)**

| | | | Less than 75th percentile | | | | | | At least 75th percentile | | | | | |
|---|---|---|---|---|---|---|---|---|---|---|---|---|---|---|
| **Country** | **Trial** | **Tool** | **LNS N** | **Control N** | **Control Mean** | **MD (95% CI)** | **Fixed W** | **Random W** | **LNS N** | **Control N** | **Control Mean** | **MD (95% CI)** | **Fixed W** | **Random W** |
| Bangladesh | JiVitA−4 (21) | BSID−III | | | | | | | | | | | | |
| Bangladesh | RDNS (22) | DMC | 1008 | 435 | −0.03 | 0.12 (0.02, 0.23) | 0.15 | 0.16 | 489 | 267 | −0.08 | 0.06 (−0.04, 0.16) | 0.33 | 0.32 |
| Bangladesh | WASH−B (23) | EASQ | 832 | 2358 | −0.01 | 0.11 (0.04, 0.19) | 0.30 | 0.20 | 221 | 838 | −0.08 | 0.07 (−0.07, 0.22) | 0.15 | 0.15 |
| Burkina Faso | iLiNS−Zinc (24) | DMC | | | | | | | | | | | | |
| Ghana | GHANA (25) | | | | | | | | | | | | | |
| Ghana | iLiNS−DYAD−G (26) | KDI | 226 | 393 | 0.00 | −0.05 (−0.21, 0.12) | 0.06 | 0.10 | 67 | 190 | −0.03 | 0.30 (0.04, 0.57) | 0.05 | 0.05 |
| Haiti | HAITI (27) | | | | | | | | | | | | | |
| Kenya | WASH−B (28) | EASQ | 960 | 3317 | 0.04 | −0.02 (−0.09, 0.06) | 0.30 | 0.20 | 317 | 1156 | 0.09 | 0.03 (−0.08, 0.13) | 0.32 | 0.31 |
| Madagascar | MAHAY (29) | ASQI | 615 | 667 | 0.02 | 0.11 (−0.13, 0.34) | 0.03 | 0.06 | 268 | 242 | −0.08 | 0.11 (−0.22, 0.43) | 0.03 | 0.03 |
| Malawi | iLiNS−DYAD−M (30) | KDI | 152 | 299 | −0.04 | 0.07 (−0.11, 0.26) | 0.04 | 0.09 | 47 | 112 | 0.07 | −0.14 (−0.48, 0.21) | 0.03 | 0.03 |
| Malawi | iLiNS−DOSE (31) | KDI | | | | | | | | | | | | |
| Mali | PROMIS CS (32) | DMC | | | | | | | | | | | | |
| Zimbabwe | SHINE (HIV−) (33) | MDAT | 583 | 560 | −0.04 | 0.08 (−0.05, 0.21) | 0.10 | 0.13 | 197 | 180 | −0.10 | 0.18 (−0.01, 0.38) | 0.09 | 0.09 |
| Zimbabwe | SHINE (HIV+) (34) | MDAT | 131 | 110 | −0.19 | 0.37 (0.10, 0.64) | 0.02 | 0.05 | 33 | 32 | 0.07 | −0.14 (−0.65, 0.37) | 0.01 | 0.01 |
| | | | **4507** | **8139** | | **I² = 0.52, Tau² = 0.00** | | | **1639** | **3017** | | **I² = 0.02, Tau² = 0.00** | | |
| **Fixed** | | | | | | **0.07 (0.03, 0.11)** | | | | | | **0.07 (0.01, 0.12)** | | |
| **Random** | | | | | | **0.08 (0.01, 0.15)** | | | | | | **0.07 (0.01, 0.13)** | | |

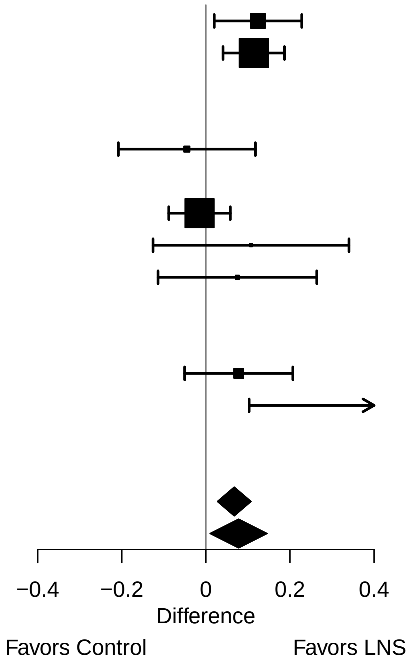


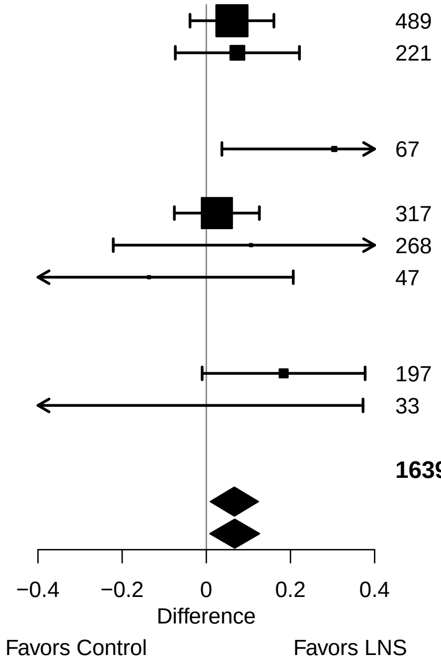

## Supplemental figure 7G: Mean difference in motor z-score

## 7G6: Stratified by Child sex

**P−for−interaction = 0.754**
**Difference in MDs = 0.01 (−0.04, 0.06)**

| Country | Trial | Tool | Male LNS N | Male Control N | Male Control Mean | Male MD (95% CI) | Male Fixed W | Male Random W | Female LNS N | Female Control N | Female Control Mean | Female MD (95% CI) | Female Fixed W | Female Random W |
|---|---|---|---|---|---|---|---|---|---|---|---|---|---|---|
| Bangladesh | JiVitA−4 (21) | BSID−III | 231 | 70 | 0.14 | −0.18 (−0.39, 0.03) | 0.04 | 0.06 | 214 | 73 | −0.10 | 0.14 (−0.06, 0.34) | 0.04 | 0.08 |
| Bangladesh | RDNS (22) | DMC | 782 | 386 | −0.02 | 0.05 (−0.04, 0.14) | 0.21 | 0.14 | 774 | 367 | −0.06 | 0.15 (0.06, 0.24) | 0.22 | 0.11 |
| Bangladesh | WASH−B (23) | EASQ | 528 | 1646 | −0.02 | 0.08 (−0.02, 0.18) | 0.17 | 0.13 | 546 | 1633 | −0.03 | 0.13 (0.04, 0.23) | 0.19 | 0.11 |
| Burkina Faso | iLiNS−Zinc (24) | DMC | 372 | 193 | −0.20 | 0.32 (0.13, 0.52) | 0.05 | 0.07 | 374 | 182 | −0.32 | 0.48 (0.29, 0.67) | 0.04 | 0.08 |
| Ghana | GHANA (25) | | | | | | | | | | | | | |
| Ghana | iLiNS−DYAD−G (26) | KDI | 150 | 280 | −0.05 | 0.14 (−0.04, 0.33) | 0.05 | 0.07 | 152 | 321 | 0.03 | −0.08 (−0.28, 0.12) | 0.04 | 0.08 |
| Haiti | HAITI (27) | | | | | | | | | | | | | |
| Kenya | WASH−B (28) | EASQ | 682 | 2260 | −0.01 | 0.03 (−0.05, 0.12) | 0.22 | 0.14 | 680 | 2485 | 0.00 | −0.01 (−0.09, 0.07) | 0.24 | 0.11 |
| Madagascar | MAHAY (29) | ASQI | 795 | 787 | −0.07 | 0.13 (−0.06, 0.33) | 0.04 | 0.07 | 818 | 817 | −0.01 | 0.01 (−0.20, 0.23) | 0.03 | 0.07 |
| Malawi | iLiNS−DYAD−M (30) | KDI | 104 | 204 | −0.07 | 0.23 (−0.01, 0.46) | 0.03 | 0.05 | 110 | 232 | 0.05 | −0.16 (−0.37, 0.05) | 0.04 | 0.07 |
| Malawi | iLiNS−DOSE (31) | KDI | 321 | 117 | −0.01 | 0.01 (−0.21, 0.23) | 0.04 | 0.06 | 325 | 104 | −0.07 | 0.09 (−0.12, 0.31) | 0.04 | 0.07 |
| Mali | PROMIS CS (32) | DMC | 456 | 501 | −0.06 | 0.13 (−0.06, 0.31) | 0.05 | 0.07 | 446 | 420 | −0.07 | 0.13 (−0.07, 0.33) | 0.04 | 0.08 |
| Zimbabwe | SHINE (HIV−) (33) | MDAT | 424 | 388 | −0.06 | 0.12 (−0.02, 0.27) | 0.08 | 0.10 | 417 | 411 | −0.04 | 0.08 (−0.08, 0.25) | 0.06 | 0.09 |
| Zimbabwe | SHINE (HIV+) (34) | MDAT | 84 | 79 | −0.11 | 0.22 (−0.08, 0.52) | 0.02 | 0.04 | 86 | 72 | −0.22 | 0.40 (0.08, 0.72) | 0.02 | 0.05 |
| | | | **4929** | **6911** | | **I² = 0.38, Tau² = 0.01** | | | **4942** | **7117** | | **I² = 0.70, Tau² = 0.02** | | |
| **Fixed** | | | | | | **0.08 (0.04, 0.12)** | | | | | | **0.09 (0.05, 0.13)** | | |
| **Random** | | | | | | **0.09 (0.03, 0.16)** | | | | | | **0.11 (0.01, 0.20)** | | |

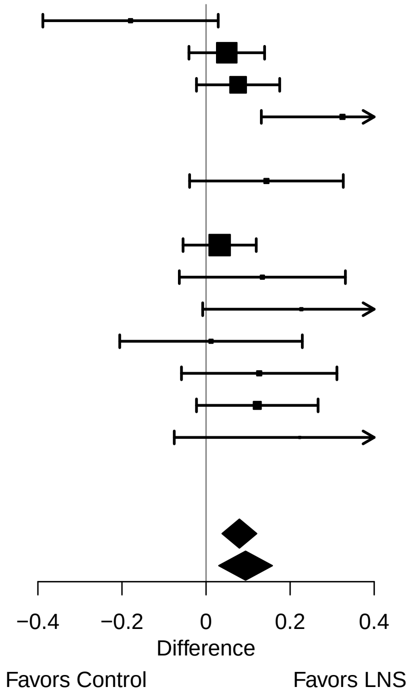


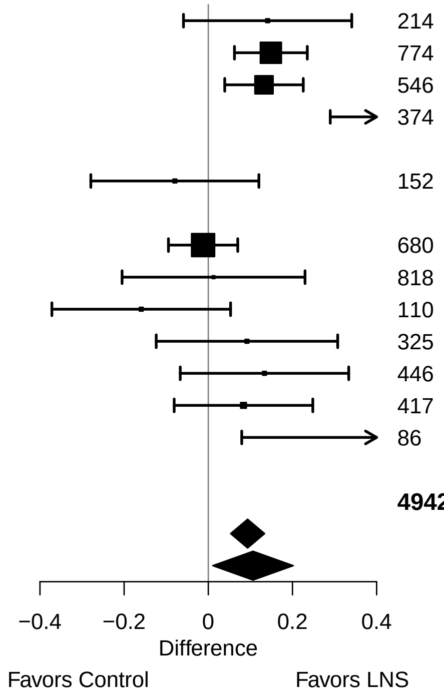

## Supplemental figure 7G: Mean difference in motor z-score

## 7G7: Stratified by Child birth order

**P−for−interaction = 0.042**
**Difference in MDs = −0.07 (−0.13, 0.00)**

| Country | Trial | Tool | Later born LNS N | Later born Control N | Later born Control Mean | Later born MD (95% CI) | Later born Fixed W | Later born Random W | Firstborn LNS N | Firstborn Control N | Firstborn Control Mean | Firstborn MD (95% CI) | Firstborn Fixed W | Firstborn Random W |
|---|---|---|---|---|---|---|---|---|---|---|---|---|---|---|
| Bangladesh | JiVitA−4 (21) | BSID−III | 111 | 31 | −0.26 | 0.12 (−0.22, 0.46) | 0.01 | 0.03 | 333 | 111 | 0.08 | −0.04 (−0.21, 0.13) | 0.10 | 0.11 |
| Bangladesh | RDNS (22) | DMC | 909 | 469 | −0.09 | 0.16 (0.06, 0.26) | 0.15 | 0.12 | 646 | 284 | 0.04 | 0.00 (−0.13, 0.13) | 0.19 | 0.16 |
| Bangladesh | WASH−B (23) | EASQ | 675 | 2149 | −0.03 | 0.11 (0.02, 0.19) | 0.19 | 0.12 | 379 | 1028 | 0.02 | 0.07 (−0.04, 0.18) | 0.25 | 0.18 |
| Burkina Faso | iLiNS−Zinc (24) | DMC | 587 | 290 | −0.29 | 0.47 (0.31, 0.63) | 0.06 | 0.08 | 159 | 85 | −0.18 | 0.16 (−0.22, 0.54) | 0.02 | 0.03 |
| Ghana | GHANA (25) | | | | | | | | | | | | | |
| Ghana | iLiNS−DYAD−G (26) | KDI | 203 | 406 | −0.04 | 0.07 (−0.10, 0.24) | 0.05 | 0.08 | 99 | 195 | 0.06 | −0.05 (−0.27, 0.16) | 0.07 | 0.08 |
| Haiti | HAITI (27) | | | | | | | | | | | | | |
| Kenya | WASH−B (28) | EASQ | 1071 | 3767 | −0.02 | 0.01 (−0.06, 0.08) | 0.28 | 0.13 | 289 | 974 | 0.07 | 0.01 (−0.14, 0.16) | 0.14 | 0.13 |
| Madagascar | MAHAY (29) | ASQI | 1162 | 1118 | −0.09 | 0.08 (−0.12, 0.28) | 0.03 | 0.07 | 433 | 465 | 0.06 | 0.08 (−0.15, 0.31) | 0.06 | 0.07 |
| Malawi | iLiNS−DYAD−M (30) | KDI | 169 | 349 | −0.02 | 0.03 (−0.15, 0.20) | 0.04 | 0.07 | 45 | 85 | 0.04 | 0.03 (−0.31, 0.37) | 0.03 | 0.04 |
| Malawi | iLiNS−DOSE (31) | KDI | 425 | 151 | −0.07 | 0.11 (−0.08, 0.30) | 0.04 | 0.07 | 134 | 41 | 0.16 | −0.09 (−0.42, 0.25) | 0.03 | 0.04 |
| Mali | PROMIS CS (32) | DMC | 749 | 786 | −0.06 | 0.17 (0.00, 0.35) | 0.04 | 0.08 | 139 | 123 | −0.03 | −0.10 (−0.41, 0.21) | 0.03 | 0.05 |
| Zimbabwe | SHINE (HIV−) (33) | MDAT | 602 | 574 | −0.01 | 0.13 (−0.01, 0.26) | 0.08 | 0.10 | 203 | 191 | −0.17 | 0.04 (−0.16, 0.24) | 0.08 | 0.09 |
| Zimbabwe | SHINE (HIV+) (34) | MDAT | 149 | 118 | −0.10 | 0.22 (−0.02, 0.46) | 0.02 | 0.05 | 18 | 29 | −0.35 | 0.84 (0.30, 1.38) | 0.01 | 0.02 |
| | | | **6812** | **10208** | | **$I^2$ = 0.66, $Tau^2$ = 0.01** | | | **2877** | **3611** | | **$I^2$ = 0.11, $Tau^2$ = 0.00** | | |
| **Fixed** | | | | | | **0.11 (0.07, 0.15)** | | | | | | **0.03 (−0.03, 0.08)** | | |
| **Random** | | | | | | **0.13 (0.06, 0.20)** | | | | | | **0.03 (−0.04, 0.10)** | | |

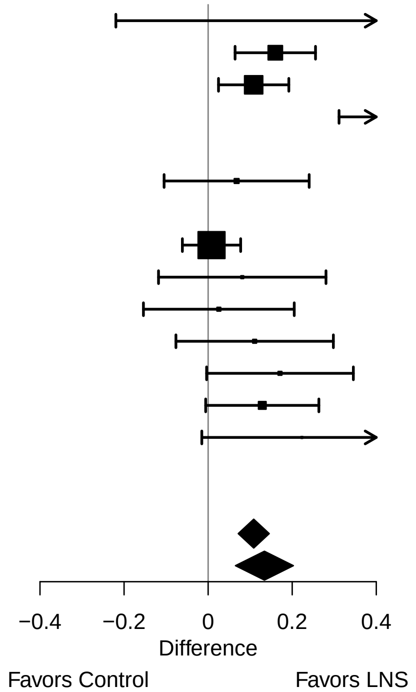


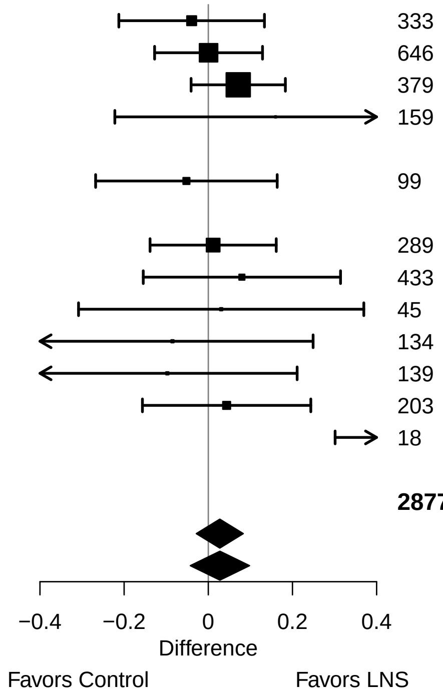

## Supplemental figure 7G: Mean difference in motor z-score

## 7G8: Stratified by Child baseline stunting

**P−for−interaction = 0.207**
**Difference in MDs = 0.07 (−0.04, 0.18)**

| | | | No | | | | | | | Yes | | | | | | |
|---|---|---|---|---|---|---|---|---|---|---|---|---|---|---|---|---|
| **Country** | **Trial** | **Tool** | **LNS N** | **Control N** | **Control Mean** | **MD (95% CI)** | **Fixed W** | **Random W** | **LNS N** | **Control N** | **Control Mean** | **MD (95% CI)** | **Fixed W** | **Random W** |
| Bangladesh | JiVitA−4 (21) | BSID−III | 327 | 114 | 0.06 | −0.02 (−0.20, 0.16) | 0.07 | 0.11 | 116 | 29 | −0.14 | 0.03 (−0.24, 0.30) | 0.14 | 0.15 |
| Bangladesh | RDNS (22) | DMC | 1170 | 535 | −0.01 | 0.10 (0.03, 0.17) | 0.44 | 0.19 | 327 | 166 | −0.18 | 0.12 (−0.06, 0.29) | 0.34 | 0.19 |
| Bangladesh | WASH−B (23) | EASQ | | | | | | | | | | | | |
| Burkina Faso | iLiNS−Zinc (24) | DMC | 576 | 294 | −0.08 | 0.33 (0.15, 0.50) | 0.07 | 0.11 | 170 | 80 | −0.90 | 0.69 (0.32, 1.07) | 0.07 | 0.11 |
| Ghana | GHANA (25) | | | | | | | | | | | | | |
| Ghana | iLiNS−DYAD−G (26) | KDI | 259 | 504 | 0.04 | −0.02 (−0.16, 0.12) | 0.12 | 0.14 | 22 | 57 | −0.36 | 0.36 (−0.28, 1.00) | 0.02 | 0.05 |
| Haiti | HAITI (27) | | | | | | | | | | | | | |
| Kenya | WASH−B (28) | EASQ | | | | | | | | | | | | |
| Madagascar | MAHAY (29) | ASQI | | | | | | | | | | | | |
| Malawi | iLiNS−DYAD−M (30) | KDI | 152 | 320 | 0.08 | −0.02 (−0.20, 0.15) | 0.08 | 0.12 | 48 | 97 | −0.24 | 0.13 (−0.26, 0.53) | 0.06 | 0.10 |
| Malawi | iLiNS−DOSE (31) | KDI | 465 | 147 | 0.05 | 0.06 (−0.11, 0.23) | 0.08 | 0.12 | 181 | 74 | −0.20 | −0.03 (−0.35, 0.28) | 0.10 | 0.13 |
| Mali | PROMIS CS (32) | DMC | | | | | | | | | | | | |
| Zimbabwe | SHINE (HIV−) (33) | MDAT | 532 | 490 | 0.02 | 0.08 (−0.06, 0.22) | 0.11 | 0.14 | 122 | 105 | −0.25 | 0.08 (−0.15, 0.30) | 0.20 | 0.17 |
| Zimbabwe | SHINE (HIV+) (34) | MDAT | 115 | 89 | −0.05 | 0.27 (0.01, 0.54) | 0.03 | 0.07 | 34 | 32 | −0.42 | 0.51 (0.12, 0.90) | 0.07 | 0.11 |
| | | | **3596** | **2493** | | **I² = 0.50, Tau² = 0.01** | | | **1020** | **640** | | **I² = 0.52, Tau² = 0.03** | | |
| **Fixed** | | | | | | **0.08 (0.04, 0.13)** | | | | | | **0.16 (0.06, 0.26)** | | |
| **Random** | | | | | | **0.09 (0.00, 0.17)** | | | | | | **0.20 (0.03, 0.37)** | | |

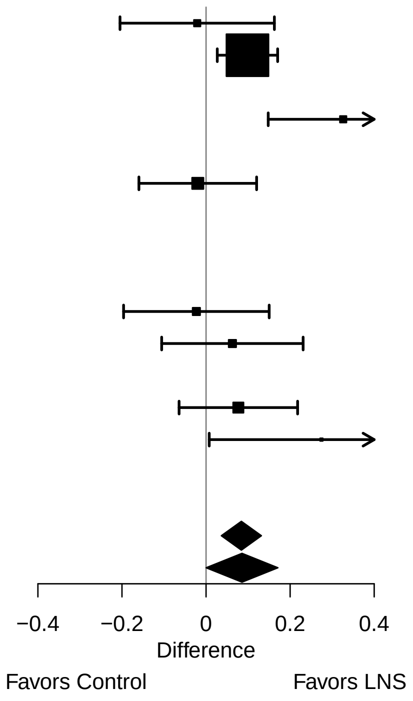


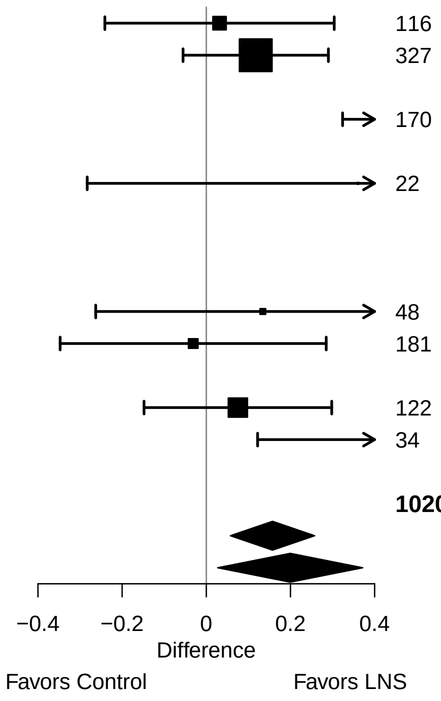

## Supplemental figure 7G: Mean difference in motor z-score

### 7G9: Stratified by Child baseline acute malnutrition

**P−for−interaction = 0.131**
**Difference in MDs = 0.12 (−0.04, 0.28)**

| | | | No | | | | | | Yes | | | | | |
|---|---|---|---|---|---|---|---|---|---|---|---|---|---|---|
| **Country** | **Trial** | **Tool** | **LNS N** | **Control N** | **Control Mean** | **MD (95% CI)** | **Fixed W** | **Random W** | **LNS N** | **Control N** | **Control Mean** | **MD (95% CI)** | **Fixed W** | **Random W** |
| Bangladesh | JiVitA−4 (21) | BSID−III | 359 | 112 | 0.10 | −0.06 (−0.24, 0.11) | 0.06 | 0.11 | 84 | 31 | −0.27 | 0.12 (−0.27, 0.51) | 0.16 | 0.16 |
| Bangladesh | RDNS (22) | DMC | 1382 | 653 | −0.03 | 0.11 (0.04, 0.18) | 0.45 | 0.19 | 115 | 49 | −0.29 | 0.11 (−0.25, 0.47) | 0.19 | 0.18 |
| Bangladesh | WASH−B (23) | EASQ | | | | | | | | | | | | |
| Burkina Faso | iLiNS−Zinc (24) | DMC | 556 | 266 | −0.03 | 0.28 (0.11, 0.45) | 0.07 | 0.11 | 190 | 109 | −0.83 | 0.65 (0.36, 0.94) | 0.30 | 0.22 |
| Ghana | GHANA (25) | | | | | | | | | | | | | |
| Ghana | iLiNS−DYAD−G (26) | KDI | 265 | 509 | 0.02 | 0.04 (−0.11, 0.18) | 0.10 | 0.13 | 17 | 52 | −0.20 | −0.34 (−0.94, 0.27) | 0.07 | 0.09 |
| Haiti | HAITI (27) | | | | | | | | | | | | | |
| Kenya | WASH−B (28) | EASQ | | | | | | | | | | | | |
| Madagascar | MAHAY (29) | ASQI | | | | | | | | | | | | |
| Malawi | iLiNS−DYAD−M (30) | KDI | 186 | 383 | 0.02 | 0.02 (−0.15, 0.18) | 0.07 | 0.12 | 14 | 37 | −0.25 | 0.06 (−0.75, 0.88) | 0.04 | 0.06 |
| Malawi | iLiNS−DOSE (31) | KDI | 609 | 206 | 0.00 | 0.05 (−0.10, 0.20) | 0.09 | 0.12 | 37 | 15 | −0.49 | −0.09 (−0.96, 0.77) | 0.03 | 0.05 |
| Mali | PROMIS CS (32) | DMC | | | | | | | | | | | | |
| Zimbabwe | SHINE (HIV−) (33) | MDAT | 620 | 566 | −0.03 | 0.07 (−0.06, 0.20) | 0.12 | 0.14 | 36 | 34 | −0.10 | 0.24 (−0.19, 0.67) | 0.13 | 0.15 |
| Zimbabwe | SHINE (HIV+) (34) | MDAT | 137 | 114 | −0.13 | 0.33 (0.10, 0.57) | 0.04 | 0.08 | 14 | 8 | −0.34 | 0.50 (−0.12, 1.12) | 0.07 | 0.09 |
| | | | **4114** | **2809** | | **I² = 0.48, Tau² = 0.01** | | | **507** | **335** | | **I² = 0.47, Tau² = 0.04** | | |
| **Fixed** | | | | | | **0.09 (0.05, 0.14)** | | | | | | **0.28 (0.12, 0.44)** | | |
| **Random** | | | | | | **0.09 (0.01, 0.18)** | | | | | | **0.23 (0.01, 0.44)** | | |

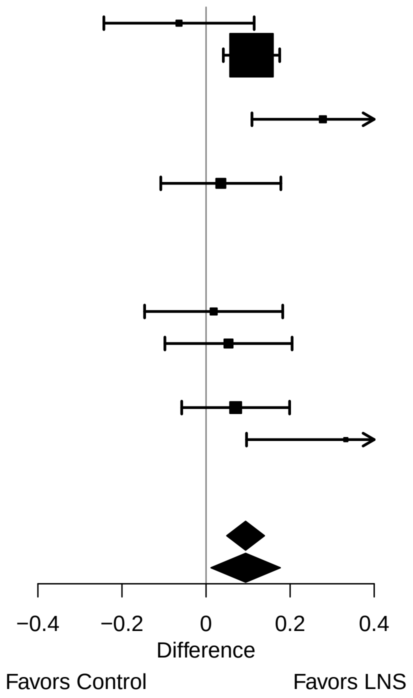


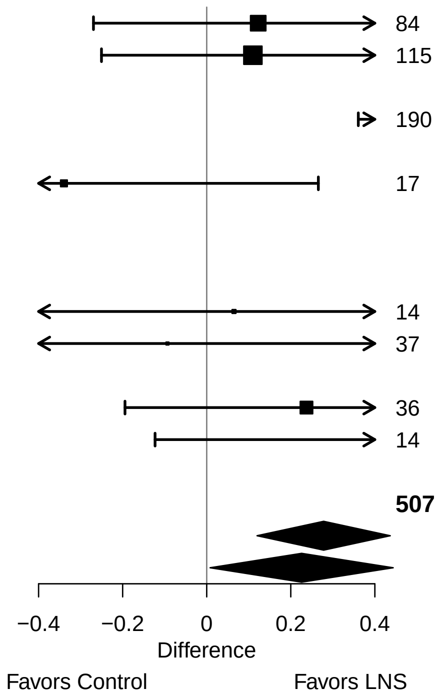

## Supplemental figure 7G: Mean difference in motor z-score

## 7G10: Stratified by Child baseline anemia

**P−for−interaction = 0.369**
**Difference in MDs = 0.07 (−0.08, 0.21)**

Not anemic

| Country | Trial | Tool | LNS N | Control N | Control Mean | MD (95% CI) | Fixed W | Random W |
|---|---|---|---|---|---|---|---|---|
| Bangladesh | JiVitA−4 (21) | BSID−III | | | | | | |
| Bangladesh | RDNS (22) | DMC | 198 | 91 | −0.16 | 0.19 (−0.03, 0.41) | 0.23 | 0.21 |
| Bangladesh | WASH−B (23) | EASQ | | | | | | |
| Burkina Faso | iLiNS−Zinc (24) | DMC | 59 | 34 | −0.25 | 0.51 (0.18, 0.85) | 0.10 | 0.16 |
| Ghana | GHANA (25) | | | | | | | |
| Ghana | iLiNS−DYAD−G (26) | KDI | 180 | 321 | 0.02 | −0.07 (−0.26, 0.12) | 0.30 | 0.23 |
| Haiti | HAITI (27) | | | | | | | |
| Kenya | WASH−B (28) | EASQ | | | | | | |
| Madagascar | MAHAY (29) | ASQI | | | | | | |
| Malawi | iLiNS−DYAD−M (30) | KDI | 65 | 146 | 0.02 | −0.05 (−0.33, 0.22) | 0.15 | 0.19 |
| Malawi | iLiNS−DOSE (31) | KDI | 224 | 94 | 0.07 | −0.04 (−0.26, 0.18) | 0.23 | 0.21 |
| Mali | PROMIS CS (32) | DMC | | | | | | |
| Zimbabwe | SHINE (HIV−) (33) | MDAT | | | | | | |
| Zimbabwe | SHINE (HIV+) (34) | MDAT | | | | | | |
| | | | **726** | **686** | | **I² = 0.66, Tau² = 0.04** | | |
| **Fixed** | | | | | | **0.06 (−0.05, 0.16)** | | |
| **Random** | | | | | | **0.09 (−0.12, 0.30)** | | |

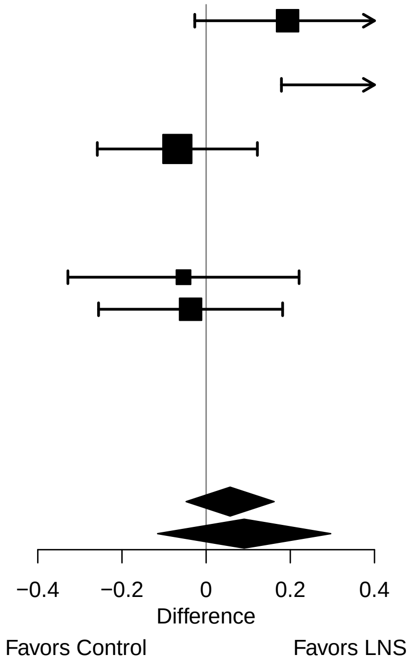


Anemic

| Trial | LNS N | Control N | Control Mean | MD (95% CI) | Fixed W | Random W |
|---|---|---|---|---|---|---|
| RDNS (22) | 303 | 151 | −0.06 | 0.12 (0.00, 0.24) | 0.42 | 0.28 |
| iLiNS−Zinc (24) | 687 | 341 | −0.26 | 0.39 (0.21, 0.58) | 0.18 | 0.20 |
| iLiNS−DYAD−G (26) | 81 | 189 | −0.06 | 0.20 (−0.04, 0.44) | 0.11 | 0.15 |
| iLiNS−DYAD−M (30) | 138 | 273 | 0.01 | 0.03 (−0.16, 0.23) | 0.16 | 0.19 |
| iLiNS−DOSE (31) | 418 | 126 | −0.12 | 0.12 (−0.09, 0.33) | 0.14 | 0.18 |
| | **1627** | **1080** | | **I² = 0.52, Tau² = 0.01** | | |
| **Fixed** | | | | **0.16 (0.08, 0.24)** | | |
| **Random** | | | | **0.17 (0.05, 0.29)** | | |

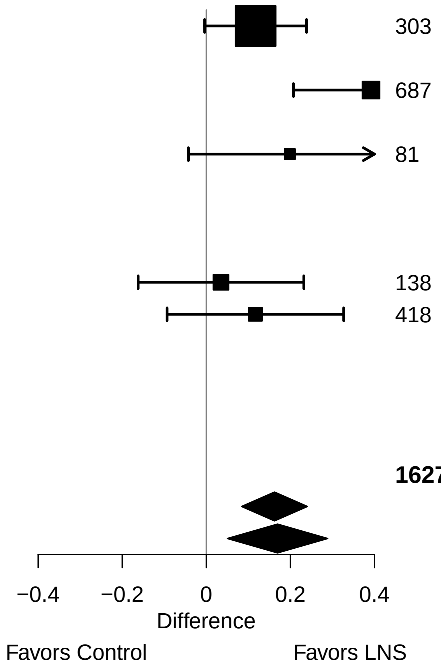

# Supplemental figure 7H: Motor lowest decile prevalence ratio

## 7H1: Stratified by Maternal height

**P−for−interaction = 0.550**
**Ratio of PRs = 1.09 (0.82, 1.45)**

| | | | At least 150.1 cm | | | | | | Less than 150.1 cm | | | | | |
|---|---|---|---|---|---|---|---|---|---|---|---|---|---|---|
| **Country** | **Trial** | **Tool** | **LNS N** | **Control N** | **Control Prevalence** | **PR (95% CI)** | **Fixed W** | **Random W** | **LNS N** | **Control N** | **Control Prevalence** | **PR (95% CI)** | **Fixed W** | **Random W** |
| Bangladesh | JiVitA−4 (21) | BSID−III | | | | | | | | | | | | |
| Bangladesh | RDNS (22) | DMC | 818 | 399 | 10.3 | 0.79 (0.56, 1.11) | 0.40 | 0.40 | 684 | 333 | 12.9 | 0.85 (0.62, 1.16) | 0.43 | 0.43 |
| Bangladesh | WASH−B (23) | EASQ | 568 | 1773 | 9.4 | 0.73 (0.52, 1.03) | 0.42 | 0.42 | 501 | 1467 | 11.9 | 0.87 (0.64, 1.18) | 0.46 | 0.46 |
| Burkina Faso | iLiNS−Zinc (24) | DMC | | | | | | | | | | | | |
| Ghana | GHANA (25) | | | | | | | | | | | | | |
| Ghana | iLiNS−DYAD−G (26) | KDI | | | | | | | | | | | | |
| Haiti | HAITI (27) | | | | | | | | | | | | | |
| Kenya | WASH−B (28) | EASQ | | | | | | | | | | | | |
| Madagascar | MAHAY (29) | ASQI | 956 | 986 | 10.2 | 0.95 (0.56, 1.60) | 0.17 | 0.17 | 553 | 538 | 9.1 | 0.93 (0.52, 1.68) | 0.12 | 0.12 |
| Malawi | iLiNS−DYAD−M (30) | KDI | | | | | | | | | | | | |
| Malawi | iLiNS−DOSE (31) | KDI | | | | | | | | | | | | |
| Mali | PROMIS CS (32) | DMC | | | | | | | | | | | | |
| Zimbabwe | SHINE (HIV−) (33) | MDAT | | | | | | | | | | | | |
| Zimbabwe | SHINE (HIV+) (34) | MDAT | | | | | | | | | | | | |
| | | | **2342** | **3158** | | **I² = 0.00, Tau² = 0.00** | | | **1738** | **2338** | | **I² = 0.00, Tau² = 0.00** | | |
| **Fixed** | | | | | | **0.79 (0.63, 0.98)** | | | | | | **0.87 (0.71, 1.07)** | | |
| **Random** | | | | | | **0.79 (0.63, 0.98)** | | | | | | **0.87 (0.71, 1.07)** | | |

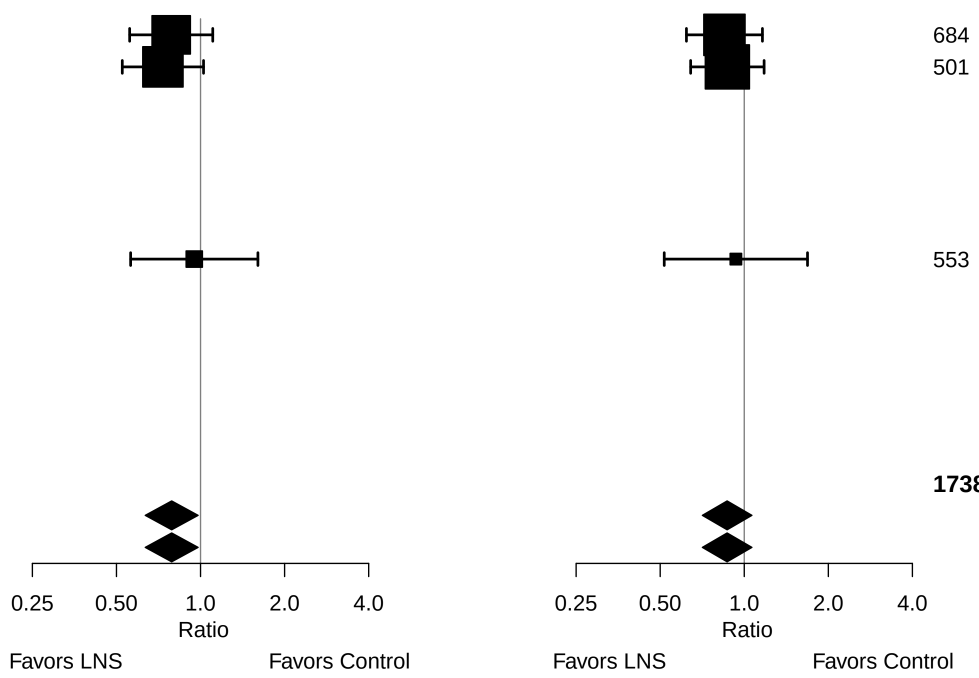

## Supplemental figure 7H: Motor lowest decile prevalence ratio

### 7H2: Stratified by Maternal BMI

**P−for−interaction = 0.477**
**Ratio of PRs = 1.08 (0.87, 1.34)**

| Country | Trial | Tool | LNS N | Control N | Control Prevalence | At least 20 kg/m² PR (95% CI) | Fixed W | Random W | LNS N | Control N | Control Prevalence | Less than 20 kg/m² PR (95% CI) | Fixed W | Random W |
|---|---|---|---|---|---|---|---|---|---|---|---|---|---|---|
| Bangladesh | JiVitA−4 (21) | BSID−III | | | | | | | | | | | | |
| Bangladesh | RDNS (22) | DMC | 689 | 306 | 10.1 | 0.77 (0.54, 1.12) | 0.13 | 0.13 | 813 | 426 | 12.4 | 0.86 (0.65, 1.13) | 0.31 | 0.26 |
| Bangladesh | WASH−B (23) | EASQ | 478 | 1502 | 9.7 | 0.75 (0.53, 1.07) | 0.14 | 0.14 | 591 | 1737 | 11.2 | 0.84 (0.63, 1.13) | 0.28 | 0.24 |
| Burkina Faso | iLiNS−Zinc (24) | DMC | 480 | 225 | 12.0 | 0.56 (0.36, 0.86) | 0.09 | 0.09 | 264 | 150 | 20.0 | 0.45 (0.28, 0.75) | 0.10 | 0.11 |
| Ghana | GHANA (25) | | | | | | | | | | | | | |
| Ghana | iLiNS−DYAD−G (26) | KDI | | | | | | | | | | | | |
| Haiti | HAITI (27) | | | | | | | | | | | | | |
| Kenya | WASH−B (28) | EASQ | 1031 | 3535 | 10.2 | 0.88 (0.70, 1.11) | 0.34 | 0.34 | 257 | 1012 | 9.9 | 0.79 (0.50, 1.24) | 0.11 | 0.13 |
| Madagascar | MAHAY (29) | ASQI | | | | | | | | | | | | |
| Malawi | iLiNS−DYAD−M (30) | KDI | 126 | 258 | 10.9 | 0.80 (0.41, 1.56) | 0.04 | 0.04 | 87 | 175 | 10.3 | 0.89 (0.40, 1.97) | 0.04 | 0.05 |
| Malawi | iLiNS−DOSE (31) | KDI | 480 | 158 | 8.9 | 1.03 (0.58, 1.84) | 0.05 | 0.05 | 163 | 61 | 11.5 | 1.12 (0.50, 2.51) | 0.04 | 0.05 |
| Mali | PROMIS CS (32) | DMC | 665 | 653 | 11.5 | 0.67 (0.43, 1.04) | 0.09 | 0.09 | 232 | 262 | 10.3 | 1.21 (0.72, 2.04) | 0.09 | 0.10 |
| Zimbabwe | SHINE (HIV−) (33) | MDAT | 601 | 581 | 10.5 | 0.81 (0.55, 1.20) | 0.11 | 0.11 | 100 | 96 | 14.6 | 0.75 (0.35, 1.64) | 0.04 | 0.05 |
| Zimbabwe | SHINE (HIV+) (34) | MDAT | | | | | | | | | | | | |
| | | | **4550** | **7218** | | **I² = 0.00, Tau² = 0.00** | | | **2507** | **3919** | | **I² = 0.17, Tau² = 0.01** | | |
| **Fixed** | | | | | | **0.79 (0.69, 0.90)** | | | | | | **0.83 (0.71, 0.96)** | | |
| **Random** | | | | | | **0.79 (0.69, 0.90)** | | | | | | **0.82 (0.68, 0.99)** | | |

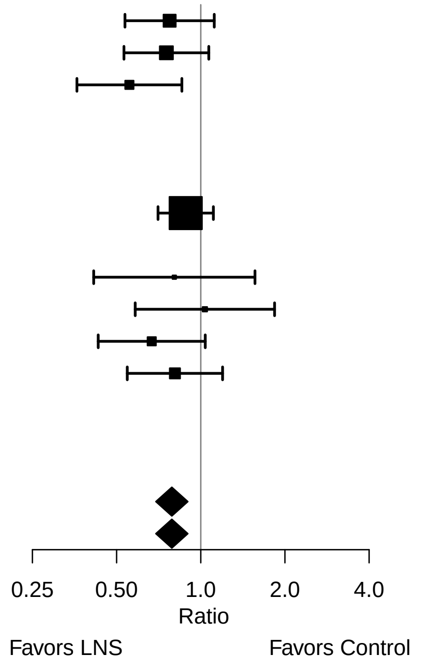


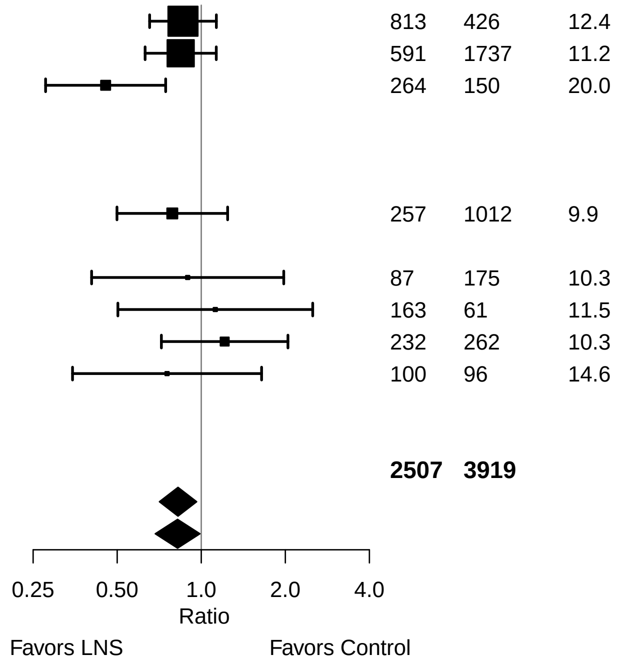

## Supplemental figure 7H: Motor lowest decile prevalence ratio

### 7H3: Stratified by Maternal age

**P−for−interaction = 0.466**
**Ratio of PRs = 1.07 (0.89, 1.28)**

| Country | Trial | Tool | LNS N | Control N | Control Prevalence | At least 25 y PR (95% CI) | Fixed W | Random W | LNS N | Control N | Control Prevalence | Less than 25 y PR (95% CI) | Fixed W | Random W |
|---|---|---|---|---|---|---|---|---|---|---|---|---|---|---|
| Bangladesh | JiVitA−4 (21) | BSID−III | 200 | 68 | 8.8 | 1.13 (0.53, 2.42) | 0.03 | 0.04 | 242 | 74 | 6.8 | 1.65 (0.64, 4.29) | 0.02 | 0.02 |
| Bangladesh | RDNS (22) | DMC | 424 | 200 | 13.0 | 0.82 (0.50, 1.34) | 0.07 | 0.08 | 1132 | 553 | 10.5 | 0.86 (0.61, 1.20) | 0.16 | 0.16 |
| Bangladesh | WASH−B (23) | EASQ | 466 | 1450 | 10.6 | 0.89 (0.63, 1.27) | 0.14 | 0.13 | 603 | 1815 | 10.7 | 0.76 (0.57, 1.01) | 0.22 | 0.22 |
| Burkina Faso | iLiNS−Zinc (24) | DMC | 421 | 232 | 17.2 | 0.40 (0.26, 0.61) | 0.10 | 0.10 | 321 | 141 | 11.3 | 0.74 (0.43, 1.28) | 0.06 | 0.06 |
| Ghana | GHANA (25) | | | | | | | | | | | | | |
| Ghana | iLiNS−DYAD−G (26) | KDI | 192 | 378 | 10.1 | 0.98 (0.58, 1.66) | 0.06 | 0.08 | 110 | 223 | 9.0 | 1.42 (0.75, 2.70) | 0.04 | 0.04 |
| Haiti | HAITI (27) | | | | | | | | | | | | | |
| Kenya | WASH−B (28) | EASQ | 798 | 2622 | 10.1 | 0.86 (0.66, 1.11) | 0.25 | 0.18 | 549 | 2092 | 10.6 | 0.86 (0.62, 1.19) | 0.17 | 0.17 |
| Madagascar | MAHAY (29) | ASQI | 852 | 901 | 11.3 | 0.90 (0.57, 1.43) | 0.08 | 0.09 | 759 | 703 | 9.4 | 0.93 (0.53, 1.61) | 0.06 | 0.06 |
| Malawi | iLiNS−DYAD−M (30) | KDI | 102 | 224 | 8.9 | 0.88 (0.40, 1.93) | 0.03 | 0.04 | 112 | 212 | 12.3 | 0.80 (0.41, 1.56) | 0.04 | 0.04 |
| Malawi | iLiNS−DOSE (31) | KDI | 346 | 124 | 8.9 | 1.11 (0.58, 2.12) | 0.04 | 0.05 | 288 | 90 | 10.0 | 1.01 (0.50, 2.05) | 0.04 | 0.04 |
| Mali | PROMIS CS (32) | DMC | 480 | 479 | 10.9 | 0.77 (0.52, 1.14) | 0.11 | 0.11 | 422 | 442 | 11.3 | 0.86 (0.51, 1.45) | 0.07 | 0.07 |
| Zimbabwe | SHINE (HIV−) (33) | MDAT | 427 | 389 | 10.5 | 0.87 (0.55, 1.37) | 0.08 | 0.09 | 326 | 321 | 11.5 | 0.96 (0.65, 1.42) | 0.12 | 0.12 |
| Zimbabwe | SHINE (HIV+) (34) | MDAT | | | | | | | | | | | | |
| | | | **4708** | **7067** | | **I² = 0.29, Tau² = 0.02** | | | **4864** | **6666** | | **I² = 0.00, Tau² = 0.00** | | |
| **Fixed** | | | | | | **0.81 (0.71, 0.93)** | | | | | | **0.88 (0.77, 1.00)** | | |
| **Random** | | | | | | **0.82 (0.69, 0.96)** | | | | | | **0.88 (0.77, 1.00)** | | |

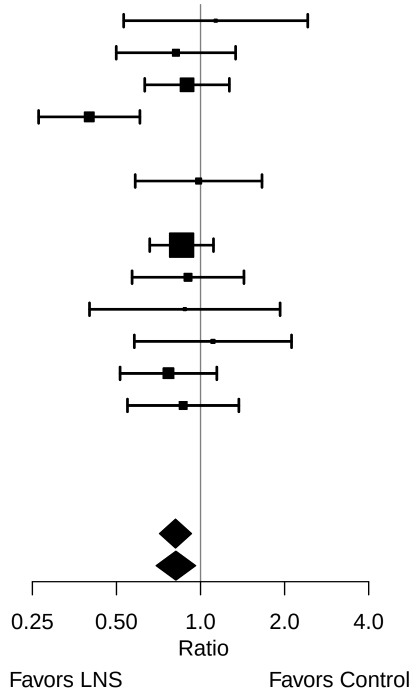


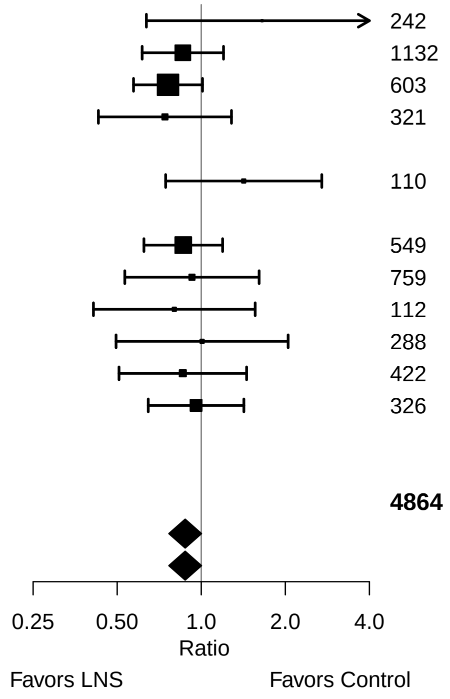

## Supplemental figure 7H: Motor lowest decile prevalence ratio

## 7H4: Stratified by Maternal education

**P−for−interaction = 0.225**
**Ratio of PRs = 0.87 (0.69, 1.09)**

| Country | Trial | Tool | Primary or greater: LNS N | Control N | Control Prevalence | PR (95% CI) | Fixed W | Random W | Incomplete or no formal: LNS N | Control N | Control Prevalence | PR (95% CI) | Fixed W | Random W |
|---|---|---|---|---|---|---|---|---|---|---|---|---|---|---|
| Bangladesh | JiVitA−4 (21) | BSID−III | 292 | 92 | 5.4 | 1.76 (0.79, 3.96) | 0.04 | 0.04 | 152 | 51 | 13.7 | 0.91 (0.44, 1.87) | 0.04 | 0.04 |
| Bangladesh | RDNS (22) | DMC | 1166 | 547 | 7.5 | 0.94 (0.65, 1.35) | 0.19 | 0.19 | 390 | 206 | 20.9 | 0.80 (0.55, 1.16) | 0.16 | 0.16 |
| Bangladesh | WASH−B (23) | EASQ | 755 | 2344 | 9.7 | 0.86 (0.66, 1.11) | 0.36 | 0.36 | 319 | 935 | 12.7 | 0.74 (0.50, 1.10) | 0.14 | 0.14 |
| Burkina Faso | iLiNS−Zinc (24) | DMC | | | | | | | | | | | | |
| Ghana | GHANA (25) | | | | | | | | | | | | | |
| Ghana | iLiNS−DYAD−G (26) | KDI | 235 | 467 | 8.8 | 1.31 (0.83, 2.07) | 0.12 | 0.12 | 67 | 134 | 12.7 | 0.71 (0.29, 1.71) | 0.03 | 0.03 |
| Haiti | HAITI (27) | | | | | | | | | | | | | |
| Kenya | WASH−B (28) | EASQ | 658 | 2258 | 7.5 | 0.81 (0.57, 1.14) | 0.20 | 0.20 | 702 | 2484 | 12.8 | 0.90 (0.70, 1.15) | 0.37 | 0.37 |
| Madagascar | MAHAY (29) | ASQI | 339 | 416 | 5.8 | 1.12 (0.63, 2.02) | 0.07 | 0.07 | 1274 | 1188 | 12.1 | 0.85 (0.53, 1.38) | 0.10 | 0.10 |
| Malawi | iLiNS−DYAD−M (30) | KDI | | | | | | | | | | | | |
| Malawi | iLiNS−DOSE (31) | KDI | | | | | | | | | | | | |
| Mali | PROMIS CS (32) | DMC | 99 | 93 | 8.6 | 0.94 (0.37, 2.41) | 0.03 | 0.03 | 802 | 828 | 11.4 | 0.80 (0.55, 1.16) | 0.16 | 0.16 |
| Zimbabwe | SHINE (HIV−) (33) | MDAT | | | | | | | | | | | | |
| Zimbabwe | SHINE (HIV+) (34) | MDAT | | | | | | | | | | | | |
| | | | **3544** | **6217** | | **I² = 0.00, Tau² = 0.00** | | | **3706** | **5826** | | **I² = 0.00, Tau² = 0.00** | | |
| **Fixed** | | | | | | **0.95 (0.81, 1.11)** | | | | | | **0.83 (0.72, 0.97)** | | |
| **Random** | | | | | | **0.95 (0.81, 1.11)** | | | | | | **0.83 (0.72, 0.97)** | | |

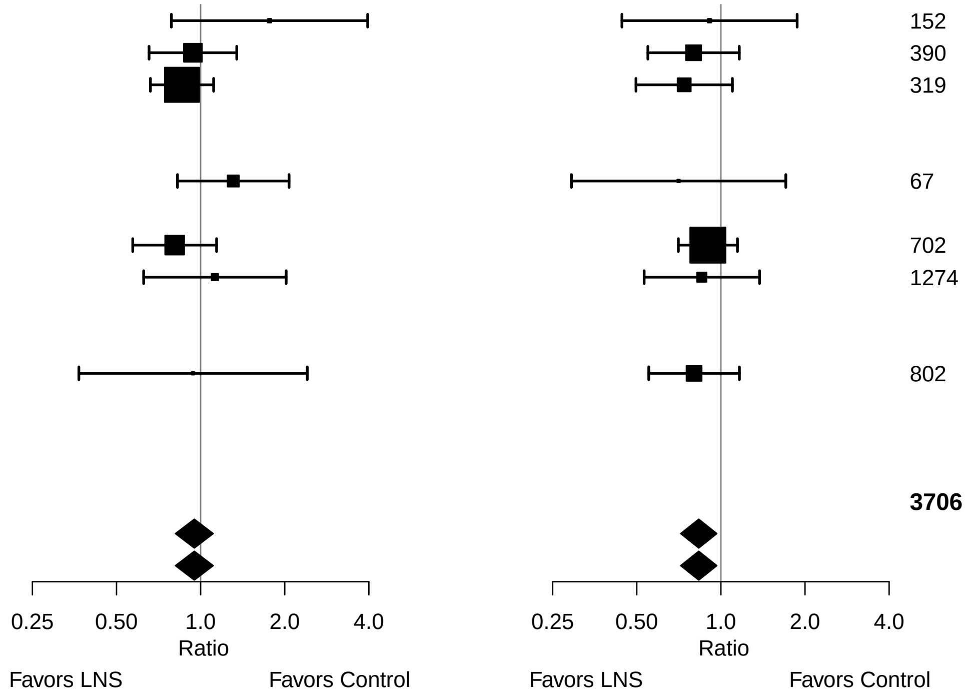

## Supplemental figure 7H: Motor lowest decile prevalence ratio

## 7H5: Stratified by Maternal depressive symptoms

**P−for−interaction = 0.649**
**Ratio of PRs = 1.06 (0.83, 1.34)**

| | | | Less than 75th percentile | | | | | | | At least 75th percentile | | | | | |
|---|---|---|---|---|---|---|---|---|---|---|---|---|---|---|---|
| **Country** | **Trial** | **Tool** | **LNS N** | **Control N** | **Control Prevalence** | **PR (95% CI)** | **Fixed W** | **Random W** | **LNS N** | **Control N** | **Control Prevalence** | **PR (95% CI)** | **Fixed W** | **Random W** |
| Bangladesh | JiVitA−4 (21) | BSID−III | | | | | | | | | | | | |
| Bangladesh | RDNS (22) | DMC | 1008 | 435 | 10.8 | 0.80 (0.58, 1.10) | 0.18 | 0.18 | 489 | 267 | 12.0 | 0.82 (0.52, 1.28) | 0.22 | 0.22 |
| Bangladesh | WASH−B (23) | EASQ | 832 | 2358 | 10.3 | 0.79 (0.62, 1.01) | 0.31 | 0.31 | 221 | 838 | 11.3 | 0.88 (0.58, 1.34) | 0.25 | 0.25 |
| Burkina Faso | iLiNS−Zinc (24) | DMC | | | | | | | | | | | | |
| Ghana | GHANA (25) | | | | | | | | | | | | | |
| Ghana | iLiNS−DYAD−G (26) | KDI | | | | | | | | | | | | |
| Haiti | HAITI (27) | | | | | | | | | | | | | |
| Kenya | WASH−B (28) | EASQ | 960 | 3317 | 8.3 | 0.98 (0.76, 1.26) | 0.29 | 0.29 | 317 | 1156 | 10.3 | 0.77 (0.50, 1.18) | 0.24 | 0.24 |
| Madagascar | MAHAY (29) | ASQI | 615 | 667 | 10.9 | 0.73 (0.39, 1.37) | 0.05 | 0.05 | 268 | 242 | 11.6 | 0.87 (0.40, 1.91) | 0.07 | 0.07 |
| Malawi | iLiNS−DYAD−M (30) | KDI | 152 | 299 | 11.7 | 0.73 (0.40, 1.34) | 0.05 | 0.05 | 47 | 112 | 8.0 | 1.32 (0.47, 3.74) | 0.04 | 0.04 |
| Malawi | iLiNS−DOSE (31) | KDI | | | | | | | | | | | | |
| Mali | PROMIS CS (32) | DMC | | | | | | | | | | | | |
| Zimbabwe | SHINE (HIV−) (33) | MDAT | 583 | 560 | 8.9 | 1.06 (0.72, 1.55) | 0.12 | 0.12 | 197 | 180 | 16.1 | 0.72 (0.44, 1.19) | 0.18 | 0.18 |
| Zimbabwe | SHINE (HIV+) (34) | MDAT | | | | | | | | | | | | |
| | | | **4150** | **7636** | | **I² = 0.00, Tau² = 0.00** | | | **1539** | **2795** | | **I² = 0.00, Tau² = 0.00** | | |
| **Fixed** | | | | | | **0.87 (0.76, 0.99)** | | | | | | **0.82 (0.67, 1.01)** | | |
| **Random** | | | | | | **0.87 (0.76, 0.99)** | | | | | | **0.82 (0.67, 1.01)** | | |

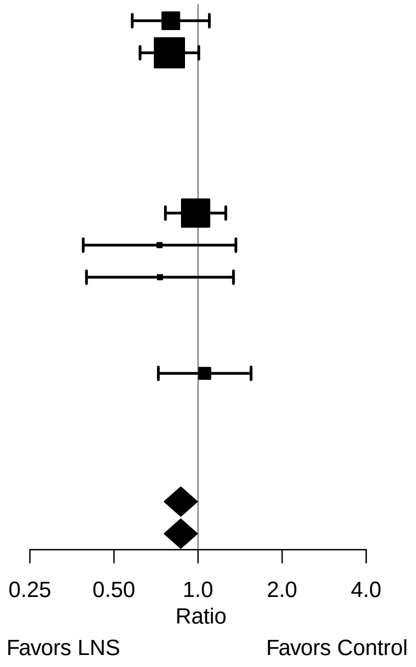


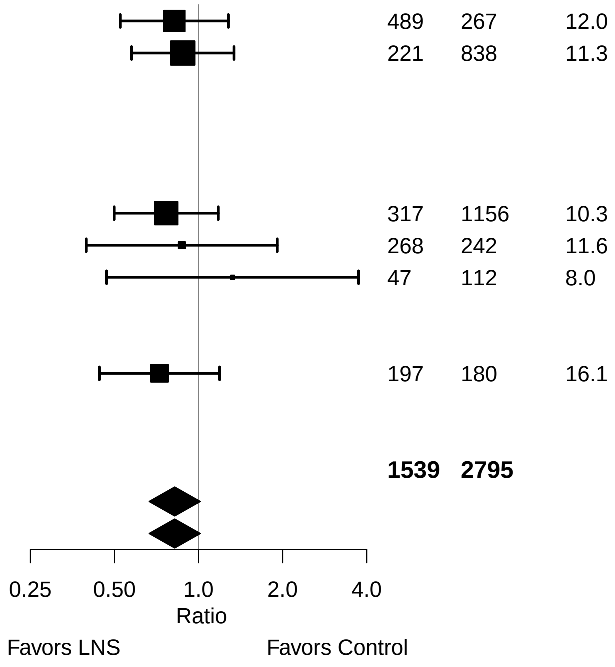

## Supplemental figure 7H: Motor lowest decile prevalence ratio

## 7H6: Stratified by Child sex

**P−for−interaction = 0.701**
**Ratio of PRs = 0.97 (0.81, 1.15)**

| Country | Trial | Tool | LNS N | Control N | Control Prevalence | Male PR (95% CI) | Fixed W | Random W | LNS N | Control N | Control Prevalence | Female PR (95% CI) | Fixed W | Random W |
|---|---|---|---|---|---|---|---|---|---|---|---|---|---|---|
| Bangladesh | JiVitA−4 (21) | BSID−III | | | | | | | | | | | | |
| Bangladesh | RDNS (22) | DMC | 782 | 386 | 10.4 | 0.96 (0.66, 1.40) | 0.12 | 0.12 | 774 | 367 | 12.0 | 0.74 (0.53, 1.04) | 0.15 | 0.13 |
| Bangladesh | WASH−B (23) | EASQ | 528 | 1646 | 14.1 | 0.90 (0.69, 1.17) | 0.24 | 0.24 | 546 | 1633 | 7.0 | 0.68 (0.46, 1.00) | 0.11 | 0.11 |
| Burkina Faso | iLiNS−Zinc (24) | DMC | 372 | 193 | 14.0 | 0.54 (0.37, 0.78) | 0.12 | 0.12 | 374 | 182 | 16.5 | 0.45 (0.30, 0.68) | 0.10 | 0.11 |
| Ghana | GHANA (25) | | | | | | | | | | | | | |
| Ghana | iLiNS−DYAD−G (26) | KDI | 150 | 280 | 8.6 | 1.01 (0.53, 1.93) | 0.04 | 0.04 | 152 | 321 | 10.6 | 1.24 (0.74, 2.08) | 0.06 | 0.08 |
| Haiti | HAITI (27) | | | | | | | | | | | | | |
| Kenya | WASH−B (28) | EASQ | 682 | 2260 | 10.0 | 0.89 (0.66, 1.20) | 0.18 | 0.18 | 680 | 2485 | 10.6 | 0.83 (0.64, 1.08) | 0.25 | 0.16 |
| Madagascar | MAHAY (29) | ASQI | 795 | 787 | 11.1 | 0.83 (0.52, 1.33) | 0.07 | 0.07 | 818 | 817 | 9.9 | 1.00 (0.57, 1.75) | 0.05 | 0.07 |
| Malawi | iLiNS−DYAD−M (30) | KDI | 104 | 204 | 13.2 | 0.44 (0.19, 1.02) | 0.02 | 0.02 | 110 | 232 | 8.2 | 1.44 (0.74, 2.81) | 0.04 | 0.06 |
| Malawi | iLiNS−DOSE (31) | KDI | 321 | 117 | 10.3 | 1.03 (0.55, 1.93) | 0.04 | 0.04 | 325 | 104 | 8.7 | 1.14 (0.56, 2.30) | 0.03 | 0.05 |
| Mali | PROMIS CS (32) | DMC | 456 | 501 | 10.6 | 0.83 (0.51, 1.36) | 0.07 | 0.07 | 446 | 420 | 11.7 | 0.79 (0.50, 1.23) | 0.08 | 0.10 |
| Zimbabwe | SHINE (HIV−) (33) | MDAT | 424 | 388 | 9.8 | 0.92 (0.58, 1.45) | 0.08 | 0.08 | 417 | 411 | 10.9 | 0.94 (0.63, 1.41) | 0.10 | 0.11 |
| Zimbabwe | SHINE (HIV+) (34) | MDAT | 84 | 79 | 13.9 | 0.60 (0.23, 1.53) | 0.02 | 0.02 | 86 | 72 | 11.1 | 0.73 (0.30, 1.78) | 0.02 | 0.04 |
| | | | **4698** | **6841** | | **I² = 0.00, Tau² = 0.00** | | | **4728** | **7044** | | **I² = 0.39, Tau² = 0.04** | | |
| **Fixed** | | | | | | **0.83 (0.73, 0.95)** | | | | | | **0.81 (0.71, 0.92)** | | |
| **Random** | | | | | | **0.83 (0.73, 0.95)** | | | | | | **0.83 (0.69, 0.99)** | | |

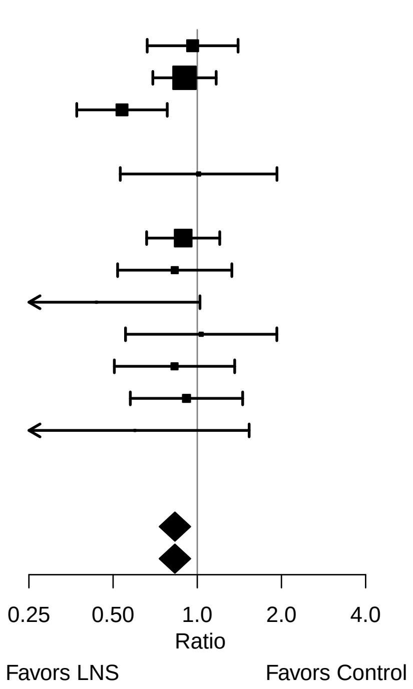


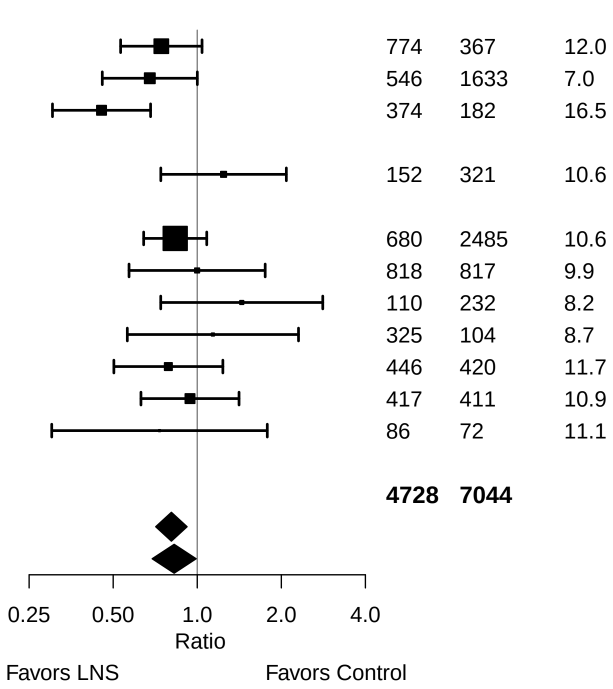

## Supplemental figure 7H: Motor lowest decile prevalence ratio

## 7H7: Stratified by Child birth order

**P−for−interaction = 0.015**
**Ratio of PRs = 1.29 (1.05, 1.59)**

| Country | Trial | Tool | LNS N | Control N | Control Prevalence | Later born PR (95% CI) | Fixed W | Random W | LNS N | Control N | Control Prevalence | Firstborn PR (95% CI) | Fixed W | Random W |
|---|---|---|---|---|---|---|---|---|---|---|---|---|---|---|
| Bangladesh | JiVitA−4 (21) | BSID−III | | | | | | | | | | | | |
| Bangladesh | RDNS (22) | DMC | 909 | 469 | 12.4 | 0.73 (0.54, 0.98) | 0.16 | 0.14 | 646 | 284 | 9.2 | 1.10 (0.72, 1.68) | 0.18 | 0.18 |
| Bangladesh | WASH−B (23) | EASQ | 675 | 2149 | 10.5 | 0.77 (0.58, 1.03) | 0.17 | 0.15 | 379 | 1028 | 10.2 | 0.90 (0.64, 1.27) | 0.28 | 0.28 |
| Burkina Faso | iLiNS−Zinc (24) | DMC | 587 | 290 | 16.6 | 0.38 (0.26, 0.56) | 0.09 | 0.12 | 159 | 85 | 10.6 | 1.13 (0.56, 2.29) | 0.06 | 0.06 |
| Ghana | GHANA (25) | | | | | | | | | | | | | |
| Ghana | iLiNS−DYAD−G (26) | KDI | 203 | 406 | 11.1 | 1.07 (0.67, 1.70) | 0.06 | 0.10 | 99 | 195 | 6.7 | 1.36 (0.60, 3.08) | 0.05 | 0.05 |
| Haiti | HAITI (27) | | | | | | | | | | | | | |
| Kenya | WASH−B (28) | EASQ | 1071 | 3767 | 10.6 | 0.85 (0.68, 1.08) | 0.26 | 0.16 | 289 | 974 | 9.1 | 0.91 (0.56, 1.48) | 0.14 | 0.14 |
| Madagascar | MAHAY (29) | ASQI | 1162 | 1118 | 10.9 | 0.89 (0.56, 1.43) | 0.06 | 0.10 | 433 | 465 | 9.9 | 0.91 (0.49, 1.68) | 0.09 | 0.09 |
| Malawi | iLiNS−DYAD−M (30) | KDI | | | | | | | | | | | | |
| Malawi | iLiNS−DOSE (31) | KDI | | | | | | | | | | | | |
| Mali | PROMIS CS (32) | DMC | 749 | 786 | 11.1 | 0.77 (0.54, 1.11) | 0.11 | 0.12 | 139 | 123 | 8.9 | 1.21 (0.55, 2.63) | 0.05 | 0.05 |
| Zimbabwe | SHINE (HIV−) (33) | MDAT | 602 | 574 | 9.6 | 0.83 (0.56, 1.25) | 0.09 | 0.11 | 203 | 191 | 14.1 | 1.05 (0.66, 1.67) | 0.15 | 0.15 |
| Zimbabwe | SHINE (HIV+) (34) | MDAT | | | | | | | | | | | | |
| | | | **5958** | **9559** | | **$I^2$ = 0.56, $Tau^2$ = 0.05** | | | **2347** | **3345** | | **$I^2$ = 0.00, $Tau^2$ = 0.00** | | |
| **Fixed** | | | | | | **0.76 (0.68, 0.86)** | | | | | | **1.01 (0.84, 1.21)** | | |
| **Random** | | | | | | **0.76 (0.62, 0.92)** | | | | | | **1.01 (0.84, 1.21)** | | |

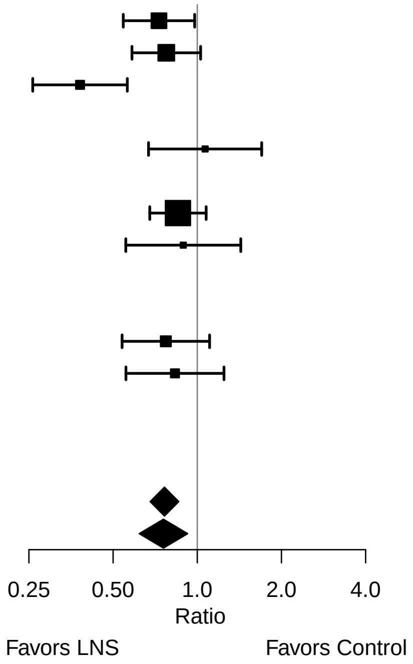


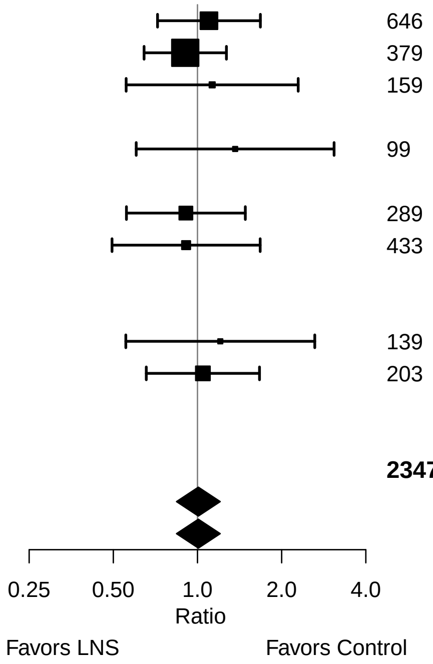

## Supplemental figure 7H: Motor lowest decile prevalence ratio

## 7H8: Stratified by Child baseline stunting

**P−for−interaction = 0.870**
**Ratio of PRs = 0.97 (0.72, 1.32)**

| Country | Trial | Tool | LNS N | Control N | Control Prevalence | No: PR (95% CI) | Fixed W | Random W | LNS N | Control N | Control Prevalence | Yes: PR (95% CI) | Fixed W | Random W |
|---|---|---|---|---|---|---|---|---|---|---|---|---|---|---|
| Bangladesh | JiVitA−4 (21) | BSID−III | | | | | | | | | | | | |
| Bangladesh | RDNS (22) | DMC | 1170 | 535 | 10.1 | 0.83 (0.62, 1.11) | 0.40 | 0.25 | 327 | 166 | 15.1 | 0.75 (0.49, 1.15) | 0.35 | 0.30 |
| Bangladesh | WASH−B (23) | EASQ | | | | | | | | | | | | |
| Burkina Faso | iLiNS−Zinc (24) | DMC | 576 | 294 | 11.6 | 0.41 (0.28, 0.58) | 0.26 | 0.23 | 170 | 80 | 28.7 | 0.59 (0.37, 0.95) | 0.29 | 0.27 |
| Ghana | GHANA (25) | | | | | | | | | | | | | |
| Ghana | iLiNS−DYAD−G (26) | KDI | | | | | | | | | | | | |
| Haiti | HAITI (27) | | | | | | | | | | | | | |
| Kenya | WASH−B (28) | EASQ | | | | | | | | | | | | |
| Madagascar | MAHAY (29) | ASQI | | | | | | | | | | | | |
| Malawi | iLiNS−DYAD−M (30) | KDI | 152 | 320 | 8.4 | 0.94 (0.49, 1.80) | 0.08 | 0.15 | 48 | 97 | 14.4 | 0.87 (0.36, 2.11) | 0.08 | 0.10 |
| Malawi | iLiNS−DOSE (31) | KDI | 465 | 147 | 8.2 | 0.92 (0.49, 1.73) | 0.08 | 0.16 | 181 | 74 | 12.2 | 1.41 (0.71, 2.81) | 0.13 | 0.16 |
| Mali | PROMIS CS (32) | DMC | | | | | | | | | | | | |
| Zimbabwe | SHINE (HIV−) (33) | MDAT | 532 | 490 | 8.2 | 1.17 (0.76, 1.81) | 0.18 | 0.21 | 122 | 105 | 19.0 | 0.52 (0.27, 0.97) | 0.15 | 0.18 |
| Zimbabwe | SHINE (HIV+) (34) | MDAT | | | | | | | | | | | | |
| | | | **2895** | **1786** | | **I² = 0.76, Tau² = 0.12** | | | **848** | **522** | | **I² = 0.28, Tau² = 0.04** | | |
| **Fixed** | | | | | | **0.75 (0.62, 0.90)** | | | | | | **0.73 (0.57, 0.93)** | | |
| **Random** | | | | | | **0.78 (0.54, 1.14)** | | | | | | **0.74 (0.54, 1.01)** | | |

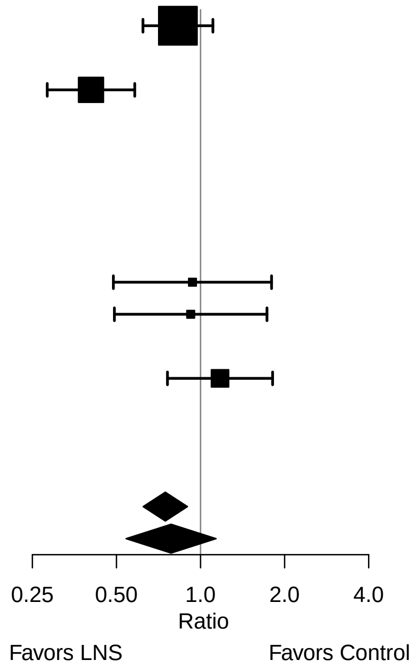


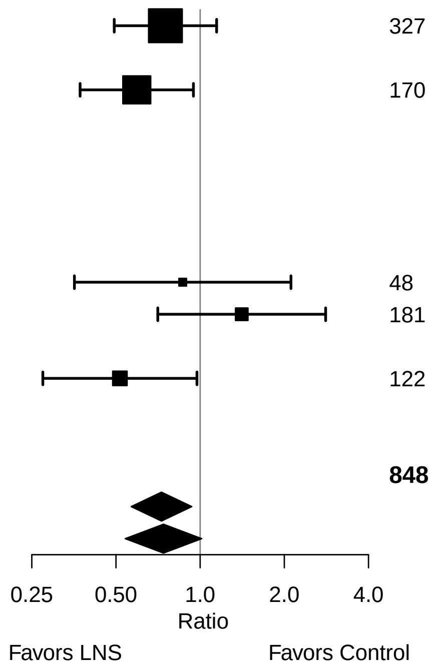

## Supplemental figure 7H: Motor lowest decile prevalence ratio

## 7H9: Stratified by Child baseline acute malnutrition

**P−for−interaction = 0.612**
**Ratio of PRs = 1.14 (0.69, 1.88)**

| Country | Trial | Tool | LNS N | Control N | Control Prevalence | No: PR (95% CI) | Fixed W | Random W | LNS N | Control N | Control Prevalence | Yes: PR (95% CI) | Fixed W | Random W |
|---|---|---|---|---|---|---|---|---|---|---|---|---|---|---|
| Bangladesh | JiVitA−4 (21) | BSID−III | 359 | 112 | 5.4 | 1.87 (0.86, 4.05) | 0.07 | 0.28 | 84 | 31 | 19.4 | 0.68 (0.28, 1.61) | 0.21 | 0.25 |
| Bangladesh | RDNS (22) | DMC | 1382 | 653 | 11.2 | 0.75 (0.59, 0.96) | 0.70 | 0.37 | 115 | 49 | 12.2 | 1.35 (0.57, 3.20) | 0.21 | 0.25 |
| Bangladesh | WASH−B (23) | EASQ | | | | | | | | | | | | |
| Burkina Faso | iLiNS−Zinc (24) | DMC | 556 | 266 | 10.2 | 0.46 (0.30, 0.71) | 0.23 | 0.35 | 190 | 109 | 27.5 | 0.57 (0.34, 0.97) | 0.58 | 0.50 |
| Ghana | GHANA (25) | | | | | | | | | | | | | |
| Ghana | iLiNS−DYAD−G (26) | KDI | | | | | | | | | | | | |
| Haiti | HAITI (27) | | | | | | | | | | | | | |
| Kenya | WASH−B (28) | EASQ | | | | | | | | | | | | |
| Madagascar | MAHAY (29) | ASQI | | | | | | | | | | | | |
| Malawi | iLiNS−DYAD−M (30) | KDI | | | | | | | | | | | | |
| Malawi | iLiNS−DOSE (31) | KDI | | | | | | | | | | | | |
| Mali | PROMIS CS (32) | DMC | | | | | | | | | | | | |
| Zimbabwe | SHINE (HIV−) (33) | MDAT | | | | | | | | | | | | |
| Zimbabwe | SHINE (HIV+) (34) | MDAT | | | | | | | | | | | | |
| | | | **2297** | **1031** | | **I² = 0.80, Tau² = 0.40** | | | **389** | **189** | | **I² = 0.28, Tau² = 0.05** | | |
| **Fixed** | | | | | | **0.72 (0.58, 0.88)** | | | | | | **0.71 (0.48, 1.06)** | | |
| **Random** | | | | | | **0.82 (0.38, 1.77)** | | | | | | **0.74 (0.45, 1.21)** | | |

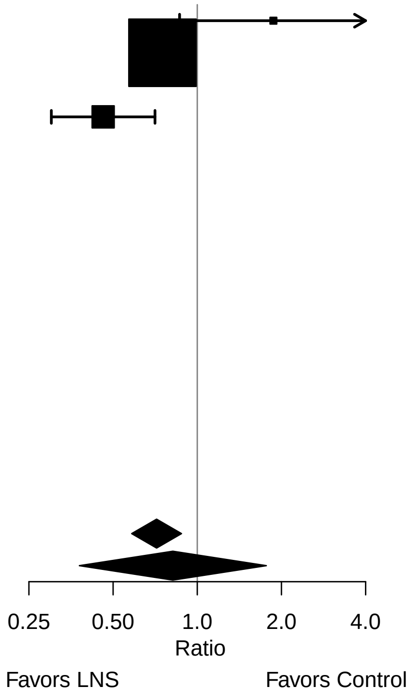


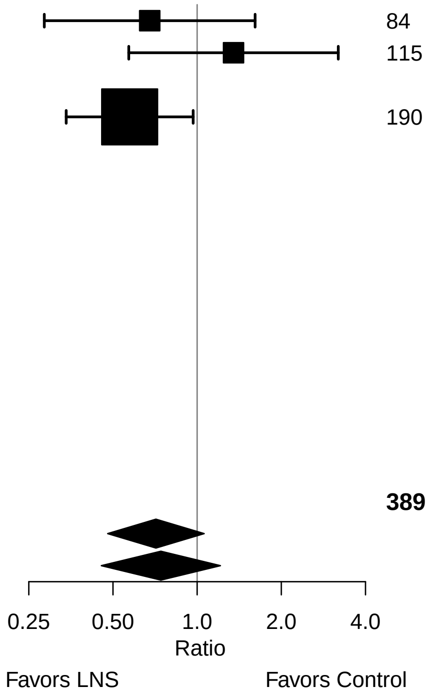

## Supplemental figure 7H: Motor lowest decile prevalence ratio

## 7H10: Stratified by Child baseline anemia

**P−for−interaction = 0.237**
**Ratio of PRs = 0.74 (0.46, 1.21)**

| Country | Trial | Tool | LNS N | Control N | Control Prevalence | Not anemic PR (95% CI) | Fixed W | Random W | LNS N | Control N | Control Prevalence | Anemic PR (95% CI) | Fixed W | Random W |
|---|---|---|---|---|---|---|---|---|---|---|---|---|---|---|
| Bangladesh | JiVitA−4 (21) | BSID−III | | | | | | | | | | | | |
| Bangladesh | RDNS (22) | DMC | 198 | 91 | 15.4 | 0.56 (0.30, 1.03) | 0.30 | 0.29 | 303 | 151 | 12.6 | 0.66 (0.38, 1.14) | 0.31 | 0.31 |
| Bangladesh | WASH−B (23) | EASQ | | | | | | | | | | | | |
| Burkina Faso | iLiNS−Zinc (24) | DMC | | | | | | | | | | | | |
| Ghana | GHANA (25) | | | | | | | | | | | | | |
| Ghana | iLiNS−DYAD−G (26) | KDI | 180 | 321 | 8.7 | 1.59 (0.96, 2.64) | 0.43 | 0.34 | 81 | 189 | 12.7 | 0.58 (0.25, 1.37) | 0.13 | 0.13 |
| Haiti | HAITI (27) | | | | | | | | | | | | | |
| Kenya | WASH−B (28) | EASQ | | | | | | | | | | | | |
| Madagascar | MAHAY (29) | ASQI | | | | | | | | | | | | |
| Malawi | iLiNS−DYAD−M (30) | KDI | 65 | 146 | 7.5 | 1.23 (0.47, 3.17) | 0.12 | 0.17 | 138 | 273 | 11.7 | 0.80 (0.44, 1.48) | 0.26 | 0.26 |
| Malawi | iLiNS−DOSE (31) | KDI | 224 | 94 | 7.4 | 1.14 (0.50, 2.62) | 0.16 | 0.20 | 418 | 126 | 11.1 | 1.01 (0.58, 1.78) | 0.30 | 0.30 |
| Mali | PROMIS CS (32) | DMC | | | | | | | | | | | | |
| Zimbabwe | SHINE (HIV−) (33) | MDAT | | | | | | | | | | | | |
| Zimbabwe | SHINE (HIV+) (34) | MDAT | | | | | | | | | | | | |
| | | | **667** | **652** | | **I² = 0.56, Tau² = 0.11** | | | **940** | **739** | | **I² = 0.00, Tau² = 0.00** | | |
| **Fixed** | | | | | | **1.07 (0.77, 1.49)** | | | | | | **0.78 (0.57, 1.06)** | | |
| **Random** | | | | | | **1.05 (0.65, 1.70)** | | | | | | **0.78 (0.57, 1.06)** | | |

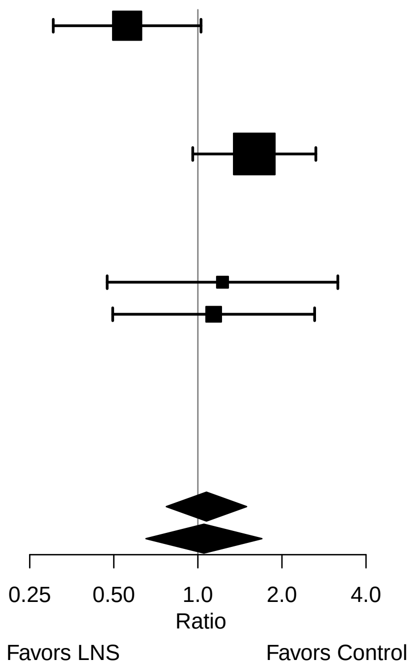


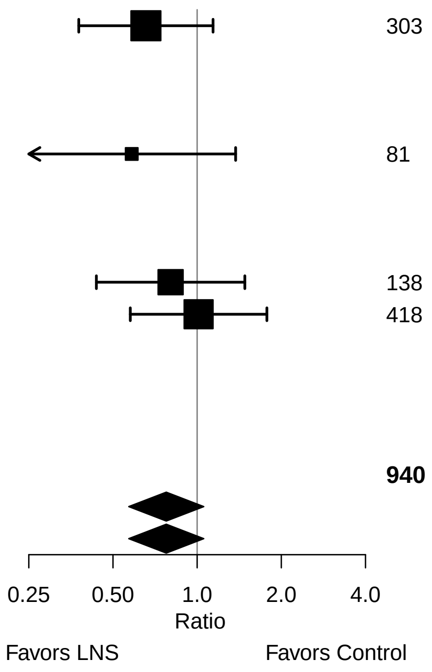

# Supplemental figure 7I: Motor lowest decile prevalence difference

## 7I1: Stratified by Maternal height

**P−for−interaction = 0.752**
**Difference in PDs = 0.00 (−0.02, 0.03)**

| Country | Trial | Tool | At least 150.1 cm: LNS N | Control N | Control Prevalence | PD (95% CI) | Fixed W | Random W | Less than 150.1 cm: LNS N | Control N | Control Prevalence | PD (95% CI) | Fixed W | Random W |
|---|---|---|---|---|---|---|---|---|---|---|---|---|---|---|
| Bangladesh | JiVitA−4 (21) | BSID−III | | | | | | | | | | | | |
| Bangladesh | RDNS (22) | DMC | 818 | 399 | 10.3 | −0.02 (−0.05, 0.01) | 0.35 | 0.35 | 684 | 333 | 12.9 | −0.02 (−0.06, 0.02) | 0.34 | 0.34 |
| Bangladesh | WASH−B (23) | EASQ | 568 | 1773 | 9.4 | −0.02 (−0.05, 0.00) | 0.53 | 0.53 | 501 | 1467 | 11.9 | −0.02 (−0.05, 0.02) | 0.48 | 0.48 |
| Burkina Faso | iLiNS−Zinc (24) | DMC | | | | | | | | | | | | |
| Ghana | GHANA (25) | | | | | | | | | | | | | |
| Ghana | iLiNS−DYAD−G (26) | KDI | | | | | | | | | | | | |
| Haiti | HAITI (27) | | | | | | | | | | | | | |
| Kenya | WASH−B (28) | EASQ | | | | | | | | | | | | |
| Madagascar | MAHAY (29) | ASQI | 956 | 986 | 10.2 | −0.01 (−0.06, 0.05) | 0.12 | 0.12 | 553 | 538 | 9.1 | −0.01 (−0.06, 0.05) | 0.18 | 0.18 |
| Malawi | iLiNS−DYAD−M (30) | KDI | | | | | | | | | | | | |
| Malawi | iLiNS−DOSE (31) | KDI | | | | | | | | | | | | |
| Mali | PROMIS CS (32) | DMC | | | | | | | | | | | | |
| Zimbabwe | SHINE (HIV−) (33) | MDAT | | | | | | | | | | | | |
| Zimbabwe | SHINE (HIV+) (34) | MDAT | | | | | | | | | | | | |
| | | | **2342** | **3158** | | **I² = 0.00, Tau² = 0.00** | | | **1738** | **2338** | | **I² = 0.00, Tau² = 0.00** | | |
| **Fixed** | | | | | | **−0.02 (−0.04, 0.00)** | | | | | | **−0.02 (−0.04, 0.01)** | | |
| **Random** | | | | | | **−0.02 (−0.04, 0.00)** | | | | | | **−0.02 (−0.04, 0.01)** | | |

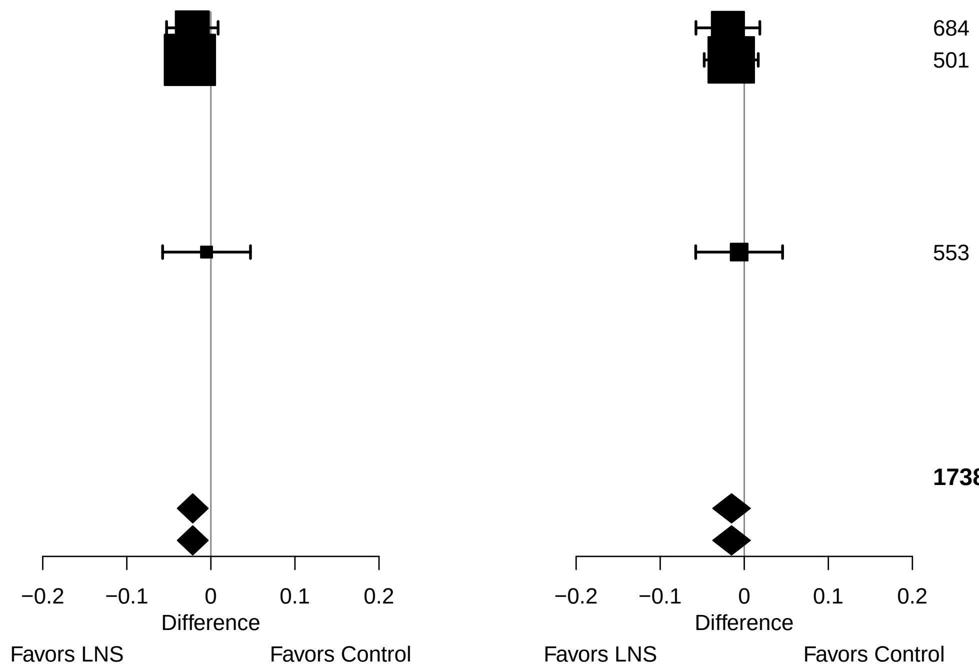

## Supplemental figure 7I: Motor lowest decile prevalence difference

## 7I2: Stratified by Maternal BMI

**P−for−interaction = 0.721**
**Difference in PDs = 0.00 (−0.02, 0.03)**

| Country | Trial | Tool | LNS N | Control N | Control Prevalence | At least 20 kg/m² PD (95% CI) | Fixed W | Random W | LNS N | Control N | Control Prevalence | Less than 20 kg/m² PD (95% CI) | Fixed W | Random W |
|---|---|---|---|---|---|---|---|---|---|---|---|---|---|---|
| Bangladesh | JiVitA−4 (21) | BSID−III | | | | | | | | | | | | |
| Bangladesh | RDNS (22) | DMC | 689 | 306 | 10.1 | −0.02 (−0.06, 0.01) | 0.13 | 0.13 | 813 | 426 | 12.4 | −0.02 (−0.05, 0.01) | 0.26 | 0.24 |
| Bangladesh | WASH−B (23) | EASQ | 478 | 1502 | 9.7 | −0.02 (−0.05, 0.00) | 0.19 | 0.19 | 591 | 1737 | 11.2 | −0.02 (−0.05, 0.01) | 0.33 | 0.28 |
| Burkina Faso | iLiNS−Zinc (24) | DMC | 480 | 225 | 12.0 | −0.05 (−0.09, −0.01) | 0.08 | 0.08 | 264 | 150 | 20.0 | −0.11 (−0.19, −0.03) | 0.05 | 0.06 |
| Ghana | GHANA (25) | | | | | | | | | | | | | |
| Ghana | iLiNS−DYAD−G (26) | KDI | | | | | | | | | | | | |
| Haiti | HAITI (27) | | | | | | | | | | | | | |
| Kenya | WASH−B (28) | EASQ | 1031 | 3535 | 10.2 | −0.01 (−0.03, 0.01) | 0.31 | 0.31 | 257 | 1012 | 9.9 | −0.02 (−0.06, 0.02) | 0.18 | 0.19 |
| Madagascar | MAHAY (29) | ASQI | | | | | | | | | | | | |
| Malawi | iLiNS−DYAD−M (30) | KDI | 126 | 258 | 10.9 | −0.02 (−0.09, 0.04) | 0.03 | 0.03 | 87 | 175 | 10.3 | −0.01 (−0.09, 0.07) | 0.05 | 0.06 |
| Malawi | iLiNS−DOSE (31) | KDI | 480 | 158 | 8.9 | 0.00 (−0.05, 0.05) | 0.05 | 0.05 | 163 | 61 | 11.5 | 0.01 (−0.08, 0.11) | 0.03 | 0.04 |
| Mali | PROMIS CS (32) | DMC | 665 | 653 | 11.5 | −0.04 (−0.08, 0.00) | 0.09 | 0.09 | 232 | 262 | 10.3 | 0.02 (−0.04, 0.08) | 0.08 | 0.09 |
| Zimbabwe | SHINE (HIV−) (33) | MDAT | 601 | 581 | 10.5 | −0.02 (−0.06, 0.02) | 0.10 | 0.10 | 100 | 96 | 14.6 | −0.04 (−0.13, 0.06) | 0.03 | 0.04 |
| Zimbabwe | SHINE (HIV+) (34) | MDAT | | | | | | | | | | | | |
| | | | **4550** | **7218** | | **I² = 0.00, Tau² = 0.00** | | | **2507** | **3919** | | **I² = 0.09, Tau² = 0.00** | | |
| **Fixed** | | | | | | **−0.02 (−0.03, −0.01)** | | | | | | **−0.02 (−0.04, 0.00)** | | |
| **Random** | | | | | | **−0.02 (−0.03, −0.01)** | | | | | | **−0.02 (−0.04, 0.00)** | | |

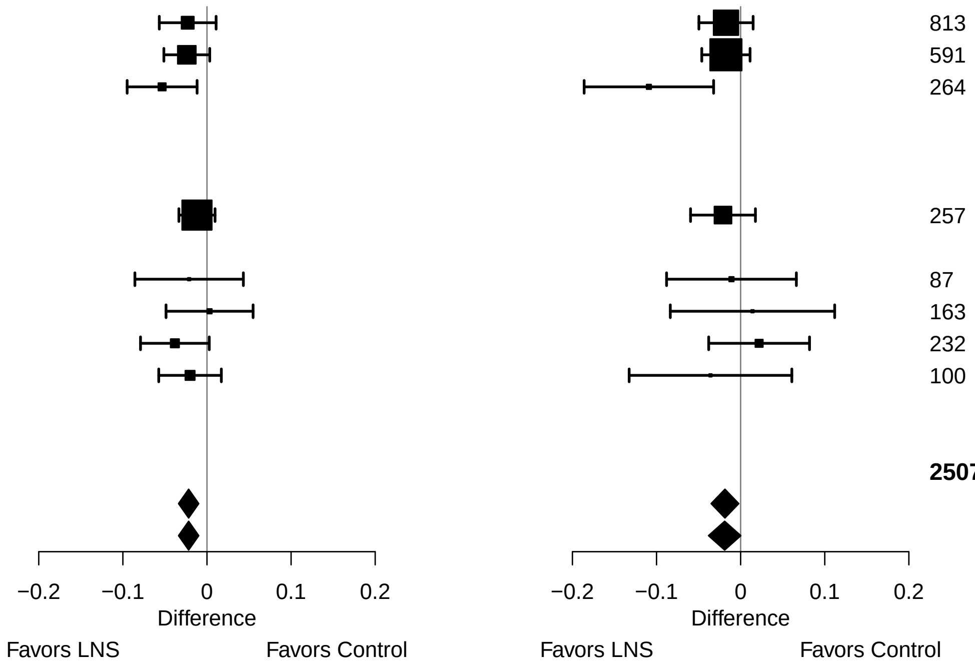

## Supplemental figure 7I: Motor lowest decile prevalence difference

### 7I3: Stratified by Maternal age

**P−for−interaction = 0.555**
**Difference in PDs = 0.01 (−0.01, 0.02)**

| | | | At least 25 y | | | | | | Less than 25 y | | | | | |
|---|---|---|---|---|---|---|---|---|---|---|---|---|---|---|
| Country | Trial | Tool | LNS N | Control N | Control Prevalence | PD (95% CI) | Fixed W | Random W | LNS N | Control N | Control Prevalence | PD (95% CI) | Fixed W | Random W |
| Bangladesh | JiVitA−4 (21) | BSID−III | 200 | 68 | 8.8 | 0.01 (−0.06, 0.08) | 0.03 | 0.05 | 242 | 74 | 6.8 | 0.04 (−0.03, 0.12) | 0.03 | 0.03 |
| Bangladesh | RDNS (22) | DMC | 424 | 200 | 13.0 | −0.02 (−0.08, 0.03) | 0.05 | 0.07 | 1132 | 553 | 10.5 | −0.01 (−0.05, 0.02) | 0.15 | 0.15 |
| Bangladesh | WASH−B (23) | EASQ | 466 | 1450 | 10.6 | −0.01 (−0.04, 0.02) | 0.14 | 0.12 | 603 | 1815 | 10.7 | −0.03 (−0.05, 0.00) | 0.28 | 0.28 |
| Burkina Faso | iLiNS−Zinc (24) | DMC | 421 | 232 | 17.2 | −0.10 (−0.14, −0.06) | 0.10 | 0.11 | 321 | 141 | 11.3 | −0.03 (−0.09, 0.03) | 0.05 | 0.05 |
| Ghana | GHANA (25) | | | | | | | | | | | | | |
| Ghana | iLiNS−DYAD−G (26) | KDI | 192 | 378 | 10.1 | 0.00 (−0.05, 0.05) | 0.06 | 0.08 | 110 | 223 | 9.0 | 0.04 (−0.03, 0.11) | 0.03 | 0.03 |
| Haiti | HAITI (27) | | | | | | | | | | | | | |
| Kenya | WASH−B (28) | EASQ | 798 | 2622 | 10.1 | −0.01 (−0.04, 0.01) | 0.28 | 0.15 | 549 | 2092 | 10.6 | −0.01 (−0.05, 0.02) | 0.18 | 0.18 |
| Madagascar | MAHAY (29) | ASQI | 852 | 901 | 11.3 | −0.01 (−0.06, 0.04) | 0.07 | 0.08 | 759 | 703 | 9.4 | −0.01 (−0.06, 0.04) | 0.07 | 0.07 |
| Malawi | iLiNS−DYAD−M (30) | KDI | 102 | 224 | 8.9 | −0.01 (−0.08, 0.05) | 0.04 | 0.06 | 112 | 212 | 12.3 | −0.02 (−0.10, 0.05) | 0.03 | 0.03 |
| Malawi | iLiNS−DOSE (31) | KDI | 346 | 124 | 8.9 | 0.01 (−0.05, 0.07) | 0.04 | 0.07 | 288 | 90 | 10.0 | 0.00 (−0.07, 0.07) | 0.03 | 0.03 |
| Mali | PROMIS CS (32) | DMC | 480 | 479 | 10.9 | −0.03 (−0.06, 0.01) | 0.12 | 0.11 | 422 | 442 | 11.3 | −0.02 (−0.07, 0.04) | 0.06 | 0.06 |
| Zimbabwe | SHINE (HIV−) (33) | MDAT | 427 | 389 | 10.5 | −0.01 (−0.06, 0.03) | 0.08 | 0.09 | 326 | 321 | 11.5 | 0.00 (−0.05, 0.04) | 0.08 | 0.08 |
| Zimbabwe | SHINE (HIV+) (34) | MDAT | | | | | | | | | | | | |
| | | | **4708** | **7067** | | **I² = 0.48, Tau² = 0.00** | | | **4864** | **6666** | | **I² = 0.00, Tau² = 0.00** | | |
| **Fixed** | | | | | | **−0.02 (−0.03, −0.01)** | | | | | | **−0.01 (−0.03, 0.00)** | | |
| **Random** | | | | | | **−0.02 (−0.04, 0.00)** | | | | | | **−0.01 (−0.03, 0.00)** | | |

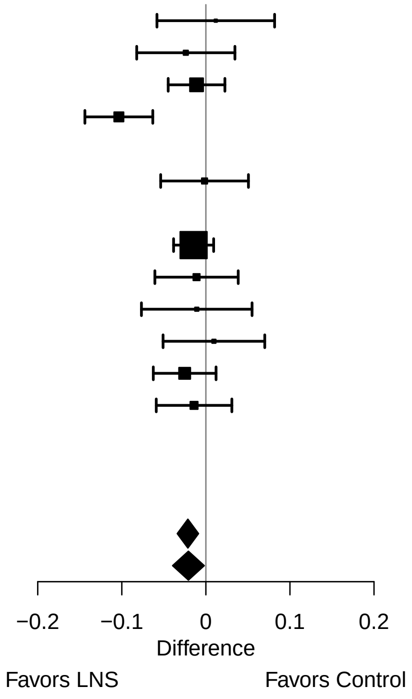


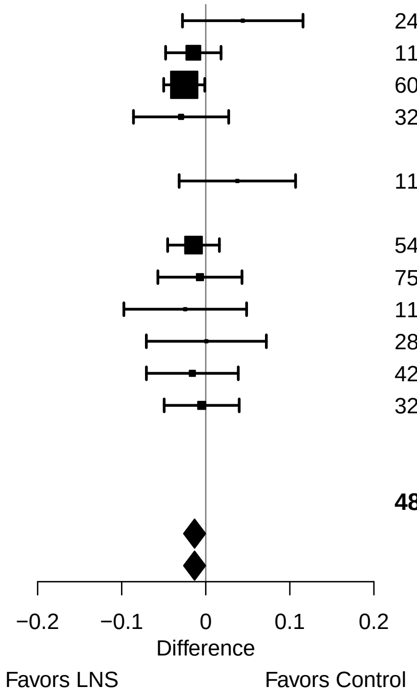

## Supplemental figure 7I: Motor lowest decile prevalence difference

## 7I4: Stratified by Maternal education

**P−for−interaction = 0.095**
**Difference in PDs = −0.02 (−0.04, 0.00)**

| Country | Trial | Tool | LNS N | Control N | Control Prevalence | Primary or greater PD (95% CI) | Fixed W | Random W | LNS N | Control N | Control Prevalence | Incomplete or no formal PD (95% CI) | Fixed W | Random W |
|---|---|---|---|---|---|---|---|---|---|---|---|---|---|---|
| Bangladesh | JiVitA−4 (21) | BSID−III | 292 | 92 | 5.4 | 0.04 (−0.01, 0.09) | 0.05 | 0.05 | 152 | 51 | 13.7 | −0.01 (−0.11, 0.08) | 0.03 | 0.03 |
| Bangladesh | RDNS (22) | DMC | 1166 | 547 | 7.5 | 0.00 (−0.03, 0.02) | 0.20 | 0.20 | 390 | 206 | 20.9 | −0.04 (−0.11, 0.03) | 0.06 | 0.06 |
| Bangladesh | WASH−B (23) | EASQ | 755 | 2344 | 9.7 | −0.01 (−0.04, 0.01) | 0.28 | 0.28 | 319 | 935 | 12.7 | −0.03 (−0.07, 0.01) | 0.19 | 0.19 |
| Burkina Faso | iLiNS−Zinc (24) | DMC | | | | | | | | | | | | |
| Ghana | GHANA (25) | | | | | | | | | | | | | |
| Ghana | iLiNS−DYAD−G (26) | KDI | 235 | 467 | 8.8 | 0.03 (−0.02, 0.07) | 0.06 | 0.06 | 67 | 134 | 12.7 | −0.04 (−0.13, 0.06) | 0.03 | 0.03 |
| Haiti | HAITI (27) | | | | | | | | | | | | | |
| Kenya | WASH−B (28) | EASQ | 658 | 2258 | 7.5 | −0.01 (−0.04, 0.01) | 0.28 | 0.28 | 702 | 2484 | 12.8 | −0.01 (−0.04, 0.02) | 0.36 | 0.36 |
| Madagascar | MAHAY (29) | ASQI | 339 | 416 | 5.8 | 0.01 (−0.03, 0.04) | 0.11 | 0.11 | 1274 | 1188 | 12.1 | −0.02 (−0.07, 0.04) | 0.11 | 0.11 |
| Malawi | iLiNS−DYAD−M (30) | KDI | | | | | | | | | | | | |
| Malawi | iLiNS−DOSE (31) | KDI | | | | | | | | | | | | |
| Mali | PROMIS CS (32) | DMC | 99 | 93 | 8.6 | −0.01 (−0.08, 0.07) | 0.02 | 0.02 | 802 | 828 | 11.4 | −0.02 (−0.06, 0.02) | 0.22 | 0.22 |
| Zimbabwe | SHINE (HIV−) (33) | MDAT | | | | | | | | | | | | |
| Zimbabwe | SHINE (HIV+) (34) | MDAT | | | | | | | | | | | | |
| | | | **3544** | **6217** | | **I² = 0.09, Tau² = 0.00** | | | **3706** | **5826** | | **I² = 0.00, Tau² = 0.00** | | |
| **Fixed** | | | | | | **0.00 (−0.02, 0.01)** | | | | | | **−0.02 (−0.04, 0.00)** | | |
| **Random** | | | | | | **0.00 (−0.02, 0.01)** | | | | | | **−0.02 (−0.04, 0.00)** | | |

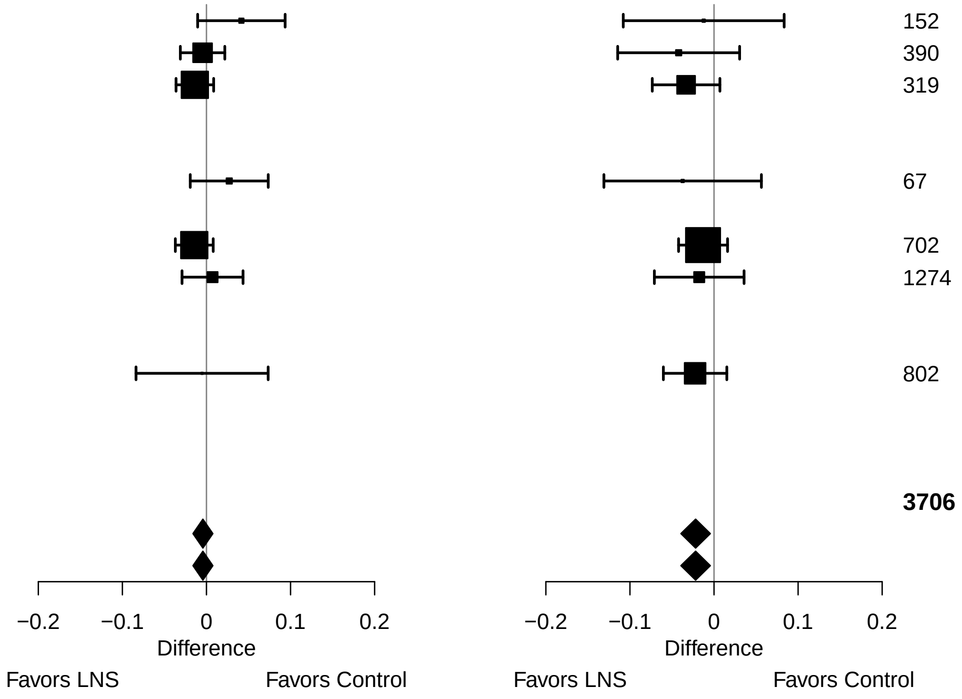

## Supplemental figure 7I: Motor lowest decile prevalence difference

## 7I5: Stratified by Maternal depressive symptoms

**P−for−interaction = 0.603**
**Difference in PDs = 0.01 (−0.02, 0.03)**

| | | | Less than 75th percentile | | | | | | At least 75th percentile | | | | | |
|---|---|---|---|---|---|---|---|---|---|---|---|---|---|---|
| **Country** | **Trial** | **Tool** | **LNS N** | **Control N** | **Control Prevalence** | **PD (95% CI)** | **Fixed W** | **Random W** | **LNS N** | **Control N** | **Control Prevalence** | **PD (95% CI)** | **Fixed W** | **Random W** |
| Bangladesh | JiVitA−4 (21) | BSID−III | | | | | | | | | | | | |
| Bangladesh | RDNS (22) | DMC | 1008 | 435 | 10.8 | −0.02 (−0.05, 0.01) | 0.14 | 0.14 | 489 | 267 | 12.0 | −0.02 (−0.07, 0.03) | 0.19 | 0.19 |
| Bangladesh | WASH−B (23) | EASQ | 832 | 2358 | 10.3 | −0.02 (−0.04, 0.00) | 0.33 | 0.33 | 221 | 838 | 11.3 | −0.01 (−0.06, 0.03) | 0.24 | 0.24 |
| Burkina Faso | iLiNS−Zinc (24) | DMC | | | | | | | | | | | | |
| Ghana | GHANA (25) | | | | | | | | | | | | | |
| Ghana | iLiNS−DYAD−G (26) | KDI | | | | | | | | | | | | |
| Haiti | HAITI (27) | | | | | | | | | | | | | |
| Kenya | WASH−B (28) | EASQ | 960 | 3317 | 8.3 | 0.00 (−0.02, 0.02) | 0.34 | 0.34 | 317 | 1156 | 10.3 | −0.02 (−0.06, 0.01) | 0.36 | 0.36 |
| Madagascar | MAHAY (29) | ASQI | 615 | 667 | 10.9 | −0.03 (−0.09, 0.03) | 0.04 | 0.04 | 268 | 242 | 11.6 | −0.01 (−0.10, 0.07) | 0.06 | 0.06 |
| Malawi | iLiNS−DYAD−M (30) | KDI | 152 | 299 | 11.7 | −0.03 (−0.09, 0.03) | 0.04 | 0.04 | 47 | 112 | 8.0 | 0.03 (−0.07, 0.12) | 0.05 | 0.05 |
| Malawi | iLiNS−DOSE (31) | KDI | | | | | | | | | | | | |
| Mali | PROMIS CS (32) | DMC | | | | | | | | | | | | |
| Zimbabwe | SHINE (HIV−) (33) | MDAT | 583 | 560 | 8.9 | 0.01 (−0.03, 0.04) | 0.11 | 0.11 | 197 | 180 | 16.1 | −0.04 (−0.11, 0.03) | 0.09 | 0.09 |
| Zimbabwe | SHINE (HIV+) (34) | MDAT | | | | | | | | | | | | |
| | | | **4150** | **7636** | | **I² = 0.00, Tau² = 0.00** | | | **1539** | **2795** | | **I² = 0.00, Tau² = 0.00** | | |
| **Fixed** | | | | | | **−0.01 (−0.02, 0.00)** | | | | | | **−0.02 (−0.04, 0.00)** | | |
| **Random** | | | | | | **−0.01 (−0.02, 0.00)** | | | | | | **−0.02 (−0.04, 0.00)** | | |

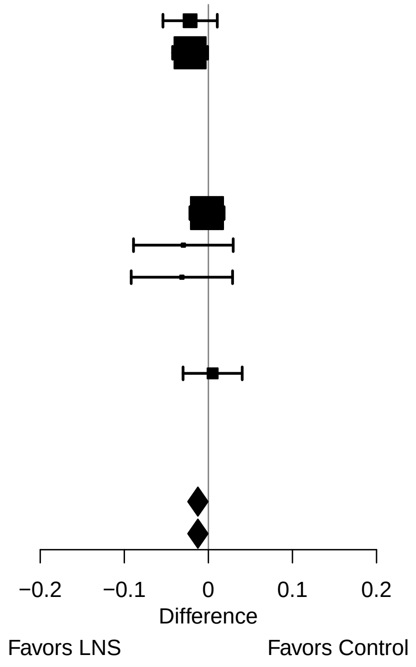


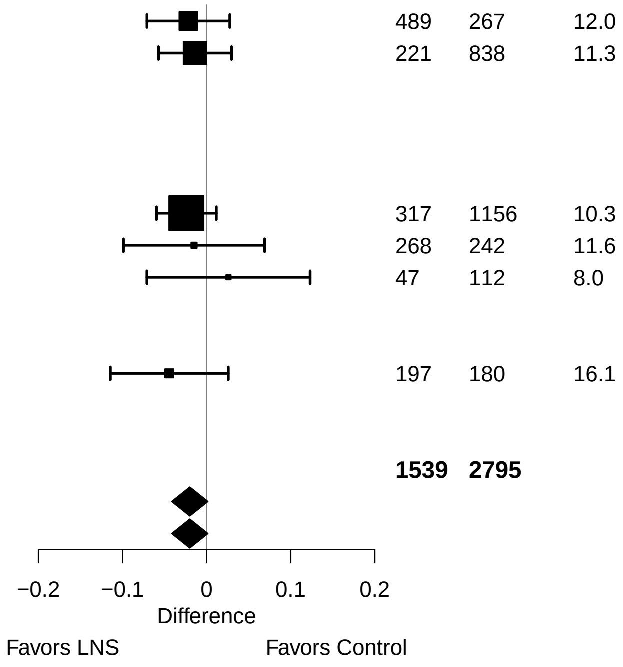

## Supplemental figure 7I: Motor lowest decile prevalence difference

## 7I6: Stratified by Child sex

**P−for−interaction = 0.917**
**Difference in PDs = 0.00 (−0.02, 0.02)**

| Country | Trial | Tool | LNS N | Control N | Control Prevalence | Male PD (95% CI) | Fixed W | Random W | LNS N | Control N | Control Prevalence | Female PD (95% CI) | Fixed W | Random W |
|---|---|---|---|---|---|---|---|---|---|---|---|---|---|---|
| Bangladesh | JiVitA−4 (21) | BSID−III | | | | | | | | | | | | |
| Bangladesh | RDNS (22) | DMC | 782 | 386 | 10.4 | 0.00 (−0.04, 0.03) | 0.12 | 0.12 | 774 | 367 | 12.0 | −0.03 (−0.06, 0.00) | 0.12 | 0.12 |
| Bangladesh | WASH−B (23) | EASQ | 528 | 1646 | 14.1 | −0.01 (−0.05, 0.02) | 0.15 | 0.15 | 546 | 1633 | 7.0 | −0.02 (−0.04, 0.00) | 0.30 | 0.16 |
| Burkina Faso | iLiNS−Zinc (24) | DMC | 372 | 193 | 14.0 | −0.06 (−0.10, −0.03) | 0.11 | 0.11 | 374 | 182 | 16.5 | −0.09 (−0.14, −0.04) | 0.06 | 0.09 |
| Ghana | GHANA (25) | | | | | | | | | | | | | |
| Ghana | iLiNS−DYAD−G (26) | KDI | 150 | 280 | 8.6 | 0.00 (−0.05, 0.06) | 0.06 | 0.06 | 152 | 321 | 10.6 | 0.03 (−0.04, 0.09) | 0.04 | 0.06 |
| Haiti | HAITI (27) | | | | | | | | | | | | | |
| Kenya | WASH−B (28) | EASQ | 682 | 2260 | 10.0 | −0.01 (−0.04, 0.02) | 0.22 | 0.22 | 680 | 2485 | 10.6 | −0.02 (−0.04, 0.01) | 0.22 | 0.15 |
| Madagascar | MAHAY (29) | ASQI | 795 | 787 | 11.1 | −0.02 (−0.07, 0.03) | 0.08 | 0.08 | 818 | 817 | 9.9 | 0.00 (−0.06, 0.06) | 0.04 | 0.07 |
| Malawi | iLiNS−DYAD−M (30) | KDI | 104 | 204 | 13.2 | −0.07 (−0.15, 0.00) | 0.03 | 0.03 | 110 | 232 | 8.2 | 0.04 (−0.03, 0.10) | 0.03 | 0.06 |
| Malawi | iLiNS−DOSE (31) | KDI | 321 | 117 | 10.3 | 0.00 (−0.06, 0.07) | 0.04 | 0.04 | 325 | 104 | 8.7 | 0.01 (−0.05, 0.08) | 0.03 | 0.06 |
| Mali | PROMIS CS (32) | DMC | 456 | 501 | 10.6 | −0.02 (−0.06, 0.03) | 0.08 | 0.08 | 446 | 420 | 11.7 | −0.02 (−0.07, 0.02) | 0.06 | 0.09 |
| Zimbabwe | SHINE (HIV−) (33) | MDAT | 424 | 388 | 9.8 | −0.01 (−0.05, 0.04) | 0.09 | 0.09 | 417 | 411 | 10.9 | −0.01 (−0.05, 0.04) | 0.07 | 0.10 |
| Zimbabwe | SHINE (HIV+) (34) | MDAT | 84 | 79 | 13.9 | −0.06 (−0.15, 0.04) | 0.02 | 0.02 | 86 | 72 | 11.1 | −0.03 (−0.11, 0.05) | 0.02 | 0.04 |
| | | | **4698** | **6841** | | **I² = 0.03, Tau² = 0.00** | | | **4728** | **7044** | | **I² = 0.36, Tau² = 0.00** | | |
| **Fixed** | | | | | | **−0.02 (−0.03, −0.01)** | | | | | | **−0.02 (−0.03, −0.01)** | | |
| **Random** | | | | | | **−0.02 (−0.03, −0.01)** | | | | | | **−0.02 (−0.04, 0.00)** | | |

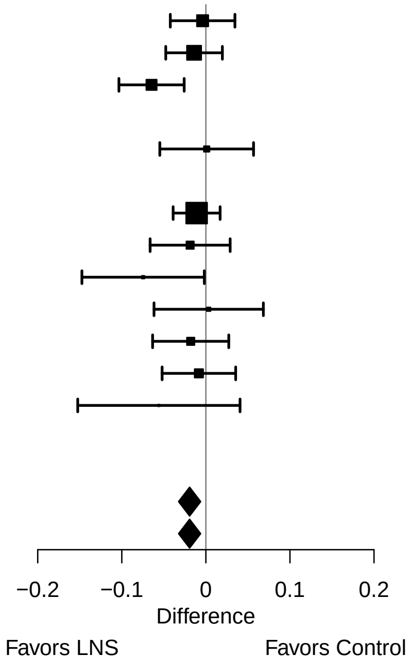


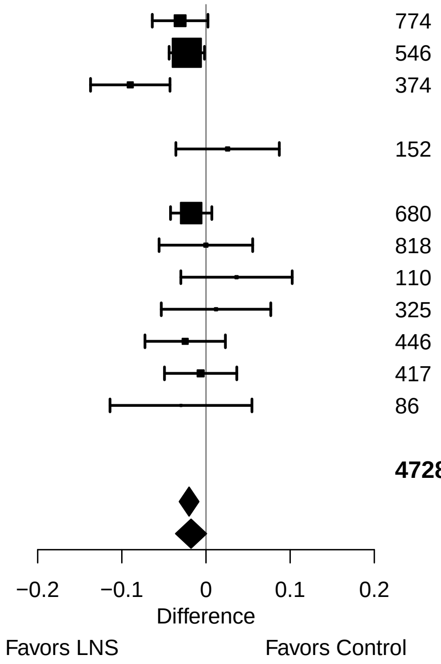

## Supplemental figure 7I: Motor lowest decile prevalence difference

### 7I7: Stratified by Child birth order

**P−for−interaction = 0.035**
**Difference in PDs = 0.02 (0.00, 0.04)**

| Country | Trial | Tool | Later born LNS N | Later born Control N | Later born Control Prevalence | Later born PD (95% CI) | Later born Fixed W | Later born Random W | Firstborn LNS N | Firstborn Control N | Firstborn Control Prevalence | Firstborn PD (95% CI) | Firstborn Fixed W | Firstborn Random W |
|---|---|---|---|---|---|---|---|---|---|---|---|---|---|---|
| Bangladesh | JiVitA−4 (21) | BSID−III | | | | | | | | | | | | |
| Bangladesh | RDNS (22) | DMC | 909 | 469 | 12.4 | −0.03 (−0.06, 0.00) | 0.14 | 0.14 | 646 | 284 | 9.2 | 0.01 (−0.03, 0.05) | 0.19 | 0.19 |
| Bangladesh | WASH−B (23) | EASQ | 675 | 2149 | 10.5 | −0.02 (−0.05, 0.00) | 0.22 | 0.15 | 379 | 1028 | 10.2 | −0.01 (−0.04, 0.02) | 0.30 | 0.30 |
| Burkina Faso | iLiNS−Zinc (24) | DMC | 587 | 290 | 16.6 | −0.10 (−0.14, −0.06) | 0.08 | 0.12 | 159 | 85 | 10.6 | 0.01 (−0.07, 0.09) | 0.05 | 0.05 |
| Ghana | GHANA (25) | | | | | | | | | | | | | |
| Ghana | iLiNS−DYAD−G (26) | KDI | 203 | 406 | 11.1 | 0.01 (−0.05, 0.06) | 0.04 | 0.09 | 99 | 195 | 6.7 | 0.02 (−0.04, 0.09) | 0.08 | 0.08 |
| Haiti | HAITI (27) | | | | | | | | | | | | | |
| Kenya | WASH−B (28) | EASQ | 1071 | 3767 | 10.6 | −0.02 (−0.04, 0.01) | 0.25 | 0.16 | 289 | 974 | 9.1 | −0.01 (−0.05, 0.03) | 0.18 | 0.18 |
| Madagascar | MAHAY (29) | ASQI | 1162 | 1118 | 10.9 | −0.01 (−0.06, 0.04) | 0.05 | 0.10 | 433 | 465 | 9.9 | −0.01 (−0.07, 0.05) | 0.09 | 0.09 |
| Malawi | iLiNS−DYAD−M (30) | KDI | | | | | | | | | | | | |
| Malawi | iLiNS−DOSE (31) | KDI | | | | | | | | | | | | |
| Mali | PROMIS CS (32) | DMC | 749 | 786 | 11.1 | −0.03 (−0.06, 0.01) | 0.11 | 0.13 | 139 | 123 | 8.9 | 0.02 (−0.06, 0.10) | 0.05 | 0.05 |
| Zimbabwe | SHINE (HIV−) (33) | MDAT | 602 | 574 | 9.6 | −0.02 (−0.05, 0.02) | 0.10 | 0.13 | 203 | 191 | 14.1 | 0.01 (−0.06, 0.07) | 0.07 | 0.07 |
| Zimbabwe | SHINE (HIV+) (34) | MDAT | | | | | | | | | | | | |
| | | | **5958** | **9559** | | **I² = 0.61, Tau² = 0.00** | | | **2347** | **3345** | | **I² = 0.00, Tau² = 0.00** | | |
| **Fixed** | | | | | | **−0.03 (−0.04, −0.02)** | | | | | | **0.00 (−0.02, 0.02)** | | |
| **Random** | | | | | | **−0.03 (−0.05, −0.01)** | | | | | | **0.00 (−0.02, 0.02)** | | |

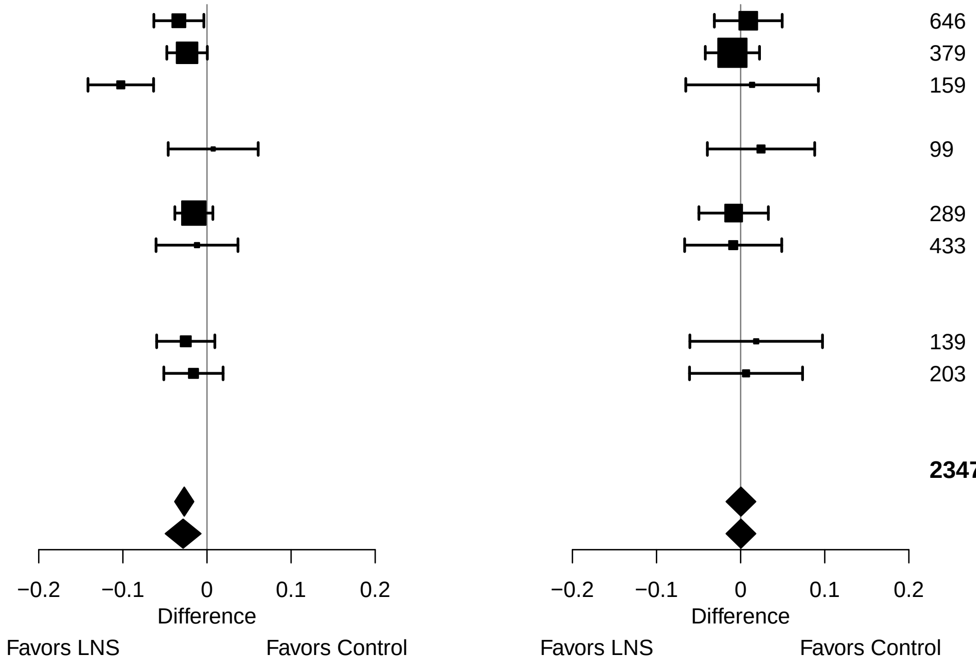

## Supplemental figure 7I: Motor lowest decile prevalence difference

## 7I8: Stratified by Child baseline stunting

**P−for−interaction = 0.226**
**Difference in PDs = −0.03 (−0.07, 0.02)**

| | | | No | | | | | | Yes | | | | | |
|---|---|---|---|---|---|---|---|---|---|---|---|---|---|---|
| **Country** | **Trial** | **Tool** | **LNS N** | **Control N** | **Control Prevalence** | **PD (95% CI)** | **Fixed W** | **Random W** | **LNS N** | **Control N** | **Control Prevalence** | **PD (95% CI)** | **Fixed W** | **Random W** |
| Bangladesh | JiVitA−4 (21) | BSID−III | | | | | | | | | | | | |
| Bangladesh | RDNS (22) | DMC | 1170 | 535 | 10.1 | −0.02 (−0.04, 0.01) | 0.32 | 0.24 | 327 | 166 | 15.1 | −0.04 (−0.09, 0.02) | 0.47 | 0.33 |
| Bangladesh | WASH−B (23) | EASQ | | | | | | | | | | | | |
| Burkina Faso | iLiNS−Zinc (24) | DMC | 576 | 294 | 11.6 | −0.07 (−0.09, −0.04) | 0.34 | 0.24 | 170 | 80 | 28.7 | −0.12 (−0.24, 0.00) | 0.10 | 0.14 |
| Ghana | GHANA (25) | | | | | | | | | | | | | |
| Ghana | iLiNS−DYAD−G (26) | KDI | | | | | | | | | | | | |
| Haiti | HAITI (27) | | | | | | | | | | | | | |
| Kenya | WASH−B (28) | EASQ | | | | | | | | | | | | |
| Madagascar | MAHAY (29) | ASQI | | | | | | | | | | | | |
| Malawi | iLiNS−DYAD−M (30) | KDI | 152 | 320 | 8.4 | −0.01 (−0.06, 0.05) | 0.08 | 0.15 | 48 | 97 | 14.4 | −0.02 (−0.14, 0.10) | 0.10 | 0.14 |
| Malawi | iLiNS−DOSE (31) | KDI | 465 | 147 | 8.2 | −0.01 (−0.06, 0.04) | 0.09 | 0.16 | 181 | 74 | 12.2 | 0.05 (−0.05, 0.15) | 0.15 | 0.18 |
| Mali | PROMIS CS (32) | DMC | | | | | | | | | | | | |
| Zimbabwe | SHINE (HIV−) (33) | MDAT | 532 | 490 | 8.2 | 0.01 (−0.02, 0.05) | 0.16 | 0.20 | 122 | 105 | 19.0 | −0.09 (−0.18, 0.00) | 0.18 | 0.21 |
| Zimbabwe | SHINE (HIV+) (34) | MDAT | | | | | | | | | | | | |
| | | | **2895** | **1786** | | **I² = 0.75, Tau² = 0.00** | | | **848** | **522** | | **I² = 0.35, Tau² = 0.00** | | |
| **Fixed** | | | | | | **−0.03 (−0.04, −0.01)** | | | | | | **−0.04 (−0.08, 0.00)** | | |
| **Random** | | | | | | **−0.02 (−0.05, 0.01)** | | | | | | **−0.04 (−0.09, 0.01)** | | |

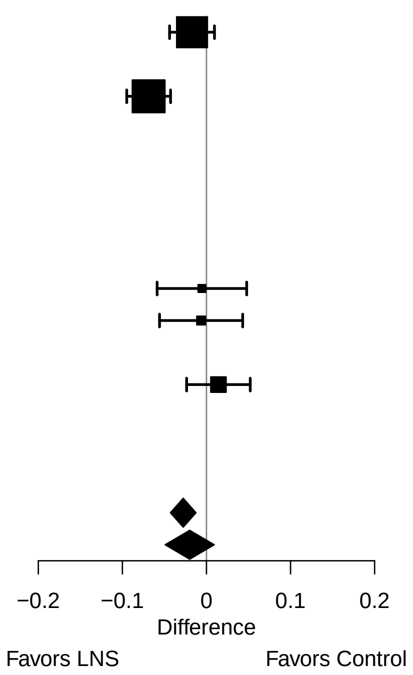


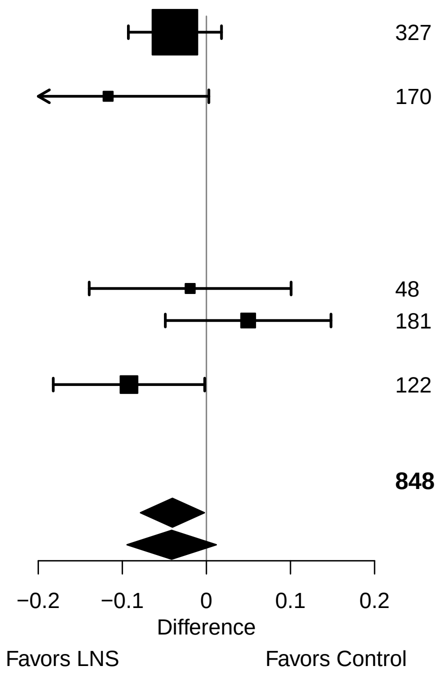

## Supplemental figure 7I: Motor lowest decile prevalence difference

### 7I9: Stratified by Child baseline acute malnutrition

**P−for−interaction = 0.540**
**Difference in PDs = −0.02 (−0.09, 0.05)**

| | | | No | | | | | | Yes | | | | | |
|---|---|---|---|---|---|---|---|---|---|---|---|---|---|---|
| **Country** | **Trial** | **Tool** | **LNS N** | **Control N** | **Control Prevalence** | **PD (95% CI)** | **Fixed W** | **Random W** | **LNS N** | **Control N** | **Control Prevalence** | **PD (95% CI)** | **Fixed W** | **Random W** |
| Bangladesh | JiVitA−4 (21) | BSID−III | 359 | 112 | 5.4 | 0.05 (0.00, 0.10) | 0.14 | 0.30 | 84 | 31 | 19.4 | −0.06 (−0.21, 0.09) | 0.22 | 0.27 |
| Bangladesh | RDNS (22) | DMC | 1382 | 653 | 11.2 | −0.03 (−0.05, 0.00) | 0.58 | 0.36 | 115 | 49 | 12.2 | 0.04 (−0.07, 0.16) | 0.37 | 0.36 |
| Bangladesh | WASH−B (23) | EASQ | | | | | | | | | | | | |
| Burkina Faso | iLiNS−Zinc (24) | DMC | 556 | 266 | 10.2 | −0.05 (−0.09, −0.02) | 0.28 | 0.34 | 190 | 109 | 27.5 | −0.12 (−0.23, −0.01) | 0.41 | 0.38 |
| Ghana | GHANA (25) | | | | | | | | | | | | | |
| Ghana | iLiNS−DYAD−G (26) | KDI | | | | | | | | | | | | |
| Haiti | HAITI (27) | | | | | | | | | | | | | |
| Kenya | WASH−B (28) | EASQ | | | | | | | | | | | | |
| Madagascar | MAHAY (29) | ASQI | | | | | | | | | | | | |
| Malawi | iLiNS−DYAD−M (30) | KDI | | | | | | | | | | | | |
| Malawi | iLiNS−DOSE (31) | KDI | | | | | | | | | | | | |
| Mali | PROMIS CS (32) | DMC | | | | | | | | | | | | |
| Zimbabwe | SHINE (HIV−) (33) | MDAT | | | | | | | | | | | | |
| Zimbabwe | SHINE (HIV+) (34) | MDAT | | | | | | | | | | | | |
| | | | **2297** | **1031** | | **I² = 0.82, Tau² = 0.00** | | | **389** | **189** | | **I² = 0.50, Tau² = 0.00** | | |
| **Fixed** | | | | | | **−0.03 (−0.04, −0.01)** | | | | | | **−0.05 (−0.12, 0.02)** | | |
| **Random** | | | | | | **−0.01 (−0.07, 0.04)** | | | | | | **−0.05 (−0.14, 0.05)** | | |

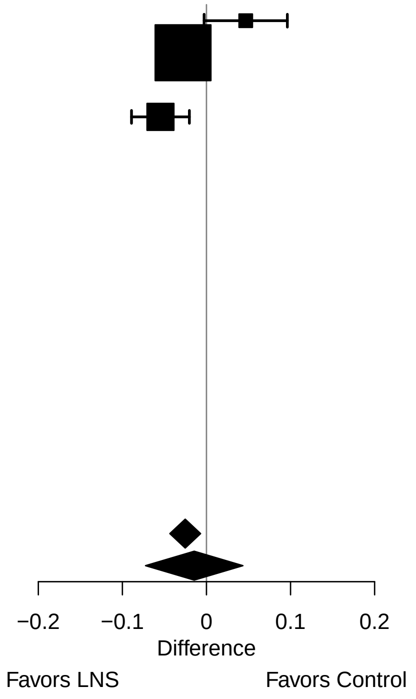


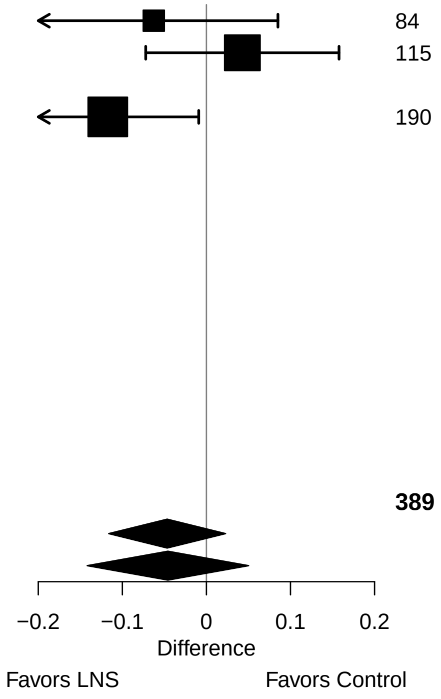

### Supplemental figure 7I: Motor lowest decile prevalence difference

## 7I10: Stratified by Child baseline anemia

**P−for−interaction = 0.177**
**Difference in PDs = −0.03 (−0.08, 0.02)**

| Country | Trial | Tool | LNS N | Control N | Control Prevalence | Not anemic PD (95% CI) | Fixed W | Random W | LNS N | Control N | Control Prevalence | Anemic PD (95% CI) | Fixed W | Random W |
|---|---|---|---|---|---|---|---|---|---|---|---|---|---|---|
| Bangladesh | JiVitA−4 (21) | BSID−III | | | | | | | | | | | | |
| Bangladesh | RDNS (22) | DMC | 198 | 91 | 15.4 | −0.07 (−0.14, 0.00) | 0.22 | 0.24 | 303 | 151 | 12.6 | −0.04 (−0.10, 0.01) | 0.34 | 0.34 |
| Bangladesh | WASH−B (23) | EASQ | | | | | | | | | | | | |
| Burkina Faso | iLiNS−Zinc (24) | DMC | | | | | | | | | | | | |
| Ghana | GHANA (25) | | | | | | | | | | | | | |
| Ghana | iLiNS−DYAD−G (26) | KDI | 180 | 321 | 8.7 | 0.05 (0.00, 0.11) | 0.36 | 0.29 | 81 | 189 | 12.7 | −0.05 (−0.13, 0.03) | 0.15 | 0.15 |
| Haiti | HAITI (27) | | | | | | | | | | | | | |
| Kenya | WASH−B (28) | EASQ | | | | | | | | | | | | |
| Madagascar | MAHAY (29) | ASQI | | | | | | | | | | | | |
| Malawi | iLiNS−DYAD−M (30) | KDI | 65 | 146 | 7.5 | 0.02 (−0.06, 0.10) | 0.17 | 0.21 | 138 | 273 | 11.7 | −0.02 (−0.09, 0.04) | 0.25 | 0.25 |
| Malawi | iLiNS−DOSE (31) | KDI | 224 | 94 | 7.4 | 0.01 (−0.06, 0.08) | 0.25 | 0.26 | 418 | 126 | 11.1 | 0.00 (−0.06, 0.06) | 0.26 | 0.26 |
| Mali | PROMIS CS (32) | DMC | | | | | | | | | | | | |
| Zimbabwe | SHINE (HIV−) (33) | MDAT | | | | | | | | | | | | |
| Zimbabwe | SHINE (HIV+) (34) | MDAT | | | | | | | | | | | | |
| | | | **667** | **652** | | **I² = 0.55, Tau² = 0.00** | | | **940** | **739** | | **I² = 0.00, Tau² = 0.00** | | |
| **Fixed** | | | | | | **0.01 (−0.02, 0.04)** | | | | | | **−0.03 (−0.06, 0.00)** | | |
| **Random** | | | | | | **0.01 (−0.04, 0.06)** | | | | | | **−0.03 (−0.06, 0.00)** | | |

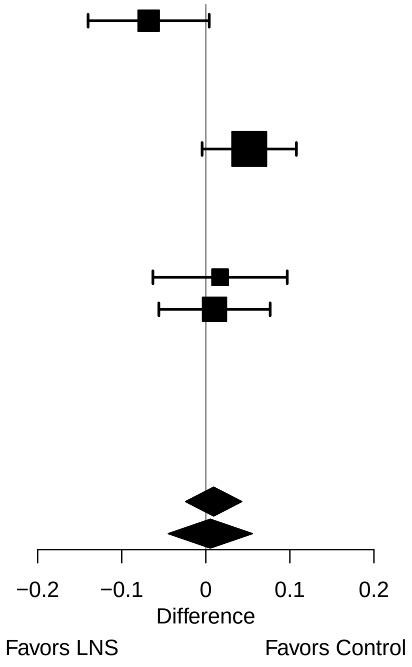


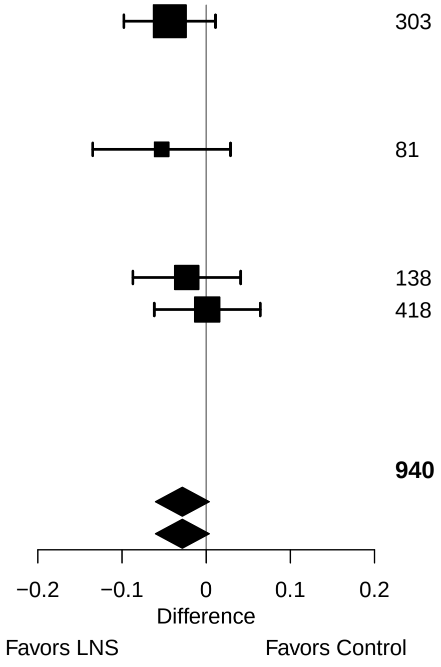

# Supplemental figure 7J: Mean difference in gross motor z-score

## 7J1: Stratified by Maternal height

**P−for−interaction = 0.787**
**Difference in MDs = −0.01 (−0.09, 0.06)**

| Country | Trial | Tool | LNS N | Control N | Control Mean | At least 150.1 cm MD (95% CI) | Fixed W | Random W | LNS N | Control N | Control Mean | Less than 150.1 cm MD (95% CI) | Fixed W | Random W |
|---|---|---|---|---|---|---|---|---|---|---|---|---|---|---|
| Bangladesh | JiVitA−4 (21) | BSID−III | | | | | | | | | | | | |
| Bangladesh | RDNS (22) | DMC | 824 | 399 | −0.04 | 0.15 (0.08, 0.22) | 0.22 | 0.15 | 691 | 337 | −0.04 | 0.03 (−0.08, 0.13) | 0.35 | 0.35 |
| Bangladesh | WASH−B (23) | EASQ | 568 | 1773 | 0.04 | 0.09 (−0.02, 0.19) | 0.10 | 0.12 | 501 | 1467 | −0.09 | 0.13 (0.03, 0.23) | 0.41 | 0.41 |
| Burkina Faso | iLiNS−Zinc (24) | DMC | | | | | | | | | | | | |
| Ghana | GHANA (25) | | | | | | | | | | | | | |
| Ghana | iLiNS−DYAD−G (26) | KDI | 284 | 557 | 0.02 | −0.02 (−0.16, 0.13) | 0.05 | 0.08 | 13 | 34 | −0.28 | 0.44 (−0.17, 1.05) | 0.01 | 0.01 |
| Haiti | HAITI (27) | | | | | | | | | | | | | |
| Kenya | WASH−B (28) | EASQ | 1237 | 4372 | 0.01 | 0.00 (−0.07, 0.06) | 0.25 | 0.15 | 51 | 175 | −0.21 | 0.27 (−0.01, 0.56) | 0.05 | 0.05 |
| Madagascar | MAHAY (29) | ASQI | 956 | 986 | −0.01 | 0.07 (−0.10, 0.23) | 0.04 | 0.07 | 553 | 538 | −0.01 | 0.08 (−0.12, 0.28) | 0.10 | 0.10 |
| Malawi | iLiNS−DYAD−M (30) | KDI | 186 | 376 | −0.01 | 0.00 (−0.17, 0.17) | 0.04 | 0.07 | 28 | 57 | 0.19 | −0.23 (−0.67, 0.21) | 0.02 | 0.02 |
| Malawi | iLiNS−DOSE (31) | KDI | 543 | 185 | 0.03 | −0.02 (−0.18, 0.15) | 0.04 | 0.07 | 102 | 35 | −0.16 | 0.09 (−0.28, 0.47) | 0.03 | 0.03 |
| Mali | PROMIS CS (32) | DMC | 902 | 924 | −0.04 | 0.09 (0.00, 0.17) | 0.16 | 0.13 | 32 | 27 | −0.09 | 0.17 (−0.42, 0.76) | 0.01 | 0.01 |
| Zimbabwe | SHINE (HIV−) (33) | MDAT | 788 | 762 | −0.05 | 0.10 (−0.02, 0.21) | 0.08 | 0.11 | 34 | 22 | −0.14 | 0.18 (−0.22, 0.59) | 0.02 | 0.02 |
| Zimbabwe | SHINE (HIV+) (34) | MDAT | 158 | 140 | −0.17 | 0.34 (0.14, 0.54) | 0.03 | 0.05 | 10 | 6 | −0.12 | 0.02 (−0.77, 0.80) | 0.01 | 0.01 |
| | | | **6446** | **10474** | | **I² = 0.56, Tau² = 0.00** | | | **2015** | **2698** | | **I² = 0.00, Tau² = 0.00** | | |
| **Fixed** | | | | | | **0.07 (0.04, 0.11)** | | | | | | **0.09 (0.03, 0.15)** | | |
| **Random** | | | | | | **0.07 (0.02, 0.13)** | | | | | | **0.09 (0.03, 0.15)** | | |

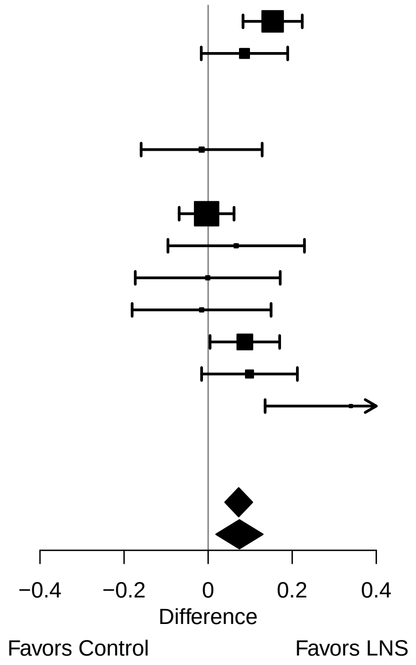


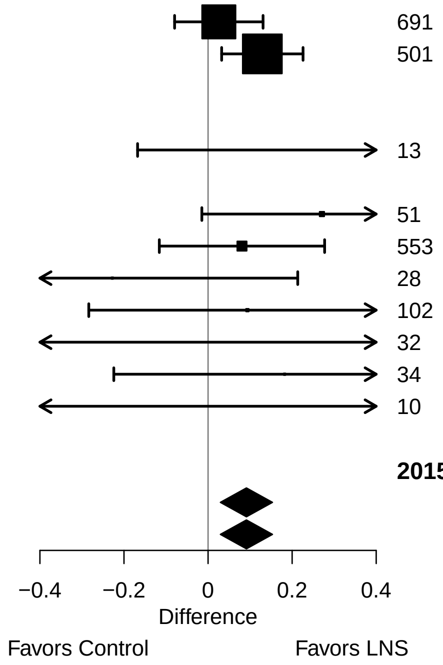

### Supplemental figure 7J: Mean difference in gross motor z-score

## 7J2: Stratified by Maternal BMI

**P−for−interaction = 0.642**
**Difference in MDs = 0.01 (−0.05, 0.08)**

| | | | At least 20 kg/m² | | | | | | Less than 20 kg/m² | | | | | |
|---|---|---|---|---|---|---|---|---|---|---|---|---|---|---|
| **Country** | **Trial** | **Tool** | **LNS N** | **Control N** | **Control Mean** | **MD (95% CI)** | **Fixed W** | **Random W** | **LNS N** | **Control N** | **Control Mean** | **MD (95% CI)** | **Fixed W** | **Random W** |
| Bangladesh | JiVitA−4 (21) | BSID−III | | | | | | | | | | | | |
| Bangladesh | RDNS (22) | DMC | 693 | 308 | −0.01 | 0.09 (0.00, 0.18) | 0.17 | 0.15 | 822 | 428 | −0.06 | 0.09 (0.02, 0.17) | 0.40 | 0.40 |
| Bangladesh | WASH−B (23) | EASQ | 478 | 1502 | 0.05 | 0.09 (−0.02, 0.19) | 0.13 | 0.14 | 591 | 1737 | −0.09 | 0.12 (0.03, 0.22) | 0.24 | 0.24 |
| Burkina Faso | iLiNS−Zinc (24) | DMC | | | | | | | | | | | | |
| Ghana | GHANA (25) | | | | | | | | | | | | | |
| Ghana | iLiNS−DYAD−G (26) | KDI | 262 | 485 | 0.01 | 0.02 (−0.13, 0.17) | 0.06 | 0.09 | 35 | 106 | −0.03 | −0.09 (−0.48, 0.30) | 0.01 | 0.01 |
| Haiti | HAITI (27) | | | | | | | | | | | | | |
| Kenya | WASH−B (28) | EASQ | 1031 | 3535 | 0.01 | −0.02 (−0.09, 0.05) | 0.30 | 0.18 | 257 | 1012 | −0.04 | 0.12 (−0.02, 0.27) | 0.11 | 0.11 |
| Madagascar | MAHAY (29) | ASQI | | | | | | | | | | | | |
| Malawi | iLiNS−DYAD−M (30) | KDI | 126 | 258 | −0.05 | 0.00 (−0.23, 0.23) | 0.03 | 0.05 | 87 | 175 | 0.12 | −0.08 (−0.28, 0.13) | 0.05 | 0.05 |
| Malawi | iLiNS−DOSE (31) | KDI | 480 | 158 | 0.02 | 0.01 (−0.16, 0.18) | 0.05 | 0.08 | 163 | 61 | −0.06 | −0.01 (−0.33, 0.31) | 0.02 | 0.02 |
| Mali | PROMIS CS (32) | DMC | 693 | 676 | −0.07 | 0.12 (0.02, 0.22) | 0.14 | 0.14 | 241 | 275 | 0.01 | 0.01 (−0.12, 0.15) | 0.12 | 0.12 |
| Zimbabwe | SHINE (HIV−) (33) | MDAT | 601 | 581 | −0.04 | 0.11 (−0.02, 0.24) | 0.09 | 0.11 | 100 | 96 | −0.18 | 0.19 (−0.07, 0.44) | 0.03 | 0.03 |
| Zimbabwe | SHINE (HIV+) (34) | MDAT | 124 | 113 | −0.13 | 0.35 (0.13, 0.58) | 0.03 | 0.05 | 25 | 23 | −0.42 | 0.27 (−0.16, 0.69) | 0.01 | 0.01 |
| | | | **4488** | **7616** | | **I² = 0.49, Tau² = 0.00** | | | **2321** | **3913** | | **I² = 0.00, Tau² = 0.00** | | |
| **Fixed** | | | | | | **0.06 (0.02, 0.10)** | | | | | | **0.09 (0.04, 0.13)** | | |
| **Random** | | | | | | **0.07 (0.01, 0.13)** | | | | | | **0.09 (0.04, 0.13)** | | |

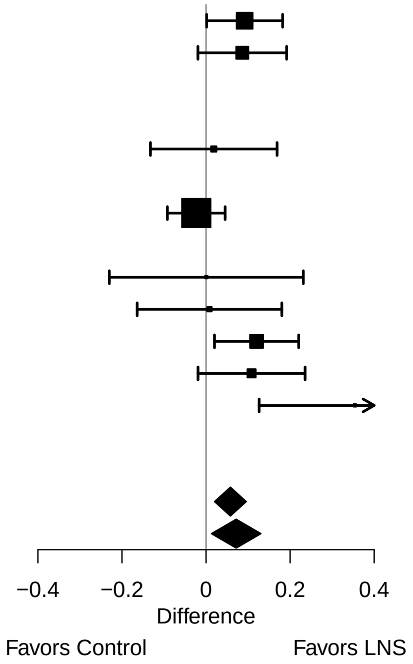


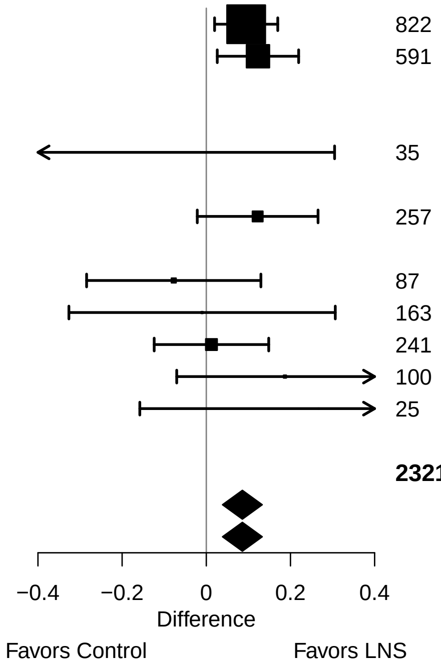

## Supplemental figure 7J: Mean difference in gross motor z-score

### 7J3: Stratified by Maternal age

**P−for−interaction = 0.610**
**Difference in MDs = −0.02 (−0.07, 0.04)**

| Country | Trial | Tool | LNS N | Control N | Control Mean | At least 25 y MD (95% CI) | Fixed W | Random W | LNS N | Control N | Control Mean | Less than 25 y MD (95% CI) | Fixed W | Random W |
|---|---|---|---|---|---|---|---|---|---|---|---|---|---|---|
| Bangladesh | JiVitA−4 (21) | BSID−III | 200 | 68 | 0.08 | −0.07 (−0.29, 0.14) | 0.04 | 0.06 | 242 | 74 | 0.12 | −0.15 (−0.44, 0.14) | 0.02 | 0.02 |
| Bangladesh | RDNS (22) | DMC | 426 | 202 | −0.12 | 0.12 (−0.03, 0.26) | 0.08 | 0.10 | 1144 | 556 | 0.00 | 0.07 (0.00, 0.15) | 0.30 | 0.26 |
| Bangladesh | WASH−B (23) | EASQ | 466 | 1450 | −0.02 | 0.09 (−0.03, 0.20) | 0.14 | 0.13 | 603 | 1815 | −0.03 | 0.11 (0.03, 0.20) | 0.22 | 0.21 |
| Burkina Faso | iLiNS−Zinc (24) | DMC | | | | | | | | | | | | |
| Ghana | GHANA (25) | | | | | | | | | | | | | |
| Ghana | iLiNS−DYAD−G (26) | KDI | 192 | 378 | −0.02 | 0.07 (−0.10, 0.24) | 0.06 | 0.08 | 110 | 223 | 0.02 | −0.10 (−0.33, 0.14) | 0.03 | 0.03 |
| Haiti | HAITI (27) | | | | | | | | | | | | | |
| Kenya | WASH−B (28) | EASQ | 798 | 2622 | 0.04 | −0.02 (−0.10, 0.05) | 0.30 | 0.18 | 549 | 2092 | −0.05 | 0.05 (−0.05, 0.16) | 0.15 | 0.15 |
| Madagascar | MAHAY (29) | ASQI | 852 | 901 | −0.09 | 0.09 (−0.08, 0.25) | 0.07 | 0.08 | 759 | 703 | 0.03 | 0.06 (−0.11, 0.23) | 0.06 | 0.06 |
| Malawi | iLiNS−DYAD−M (30) | KDI | 102 | 224 | 0.06 | −0.05 (−0.28, 0.18) | 0.03 | 0.05 | 112 | 212 | −0.03 | −0.01 (−0.24, 0.21) | 0.03 | 0.04 |
| Malawi | iLiNS−DOSE (31) | KDI | 346 | 124 | −0.06 | 0.06 (−0.15, 0.26) | 0.04 | 0.06 | 288 | 90 | 0.08 | −0.07 (−0.29, 0.16) | 0.03 | 0.04 |
| Mali | PROMIS CS (32) | DMC | 495 | 500 | −0.08 | 0.15 (0.03, 0.27) | 0.12 | 0.12 | 445 | 457 | 0.00 | 0.02 (−0.11, 0.14) | 0.10 | 0.10 |
| Zimbabwe | SHINE (HIV−) (33) | MDAT | 427 | 389 | −0.02 | 0.15 (0.00, 0.30) | 0.08 | 0.10 | 326 | 321 | −0.11 | 0.09 (−0.06, 0.25) | 0.07 | 0.07 |
| Zimbabwe | SHINE (HIV+) (34) | MDAT | 126 | 111 | −0.15 | 0.31 (0.09, 0.54) | 0.04 | 0.05 | 29 | 26 | −0.27 | 0.63 (−0.01, 1.26) | 0.00 | 0.00 |
| | | | **4430** | **6969** | | **$I^2$ = 0.38, $Tau^2$ = 0.00** | | | **4607** | **6569** | | **$I^2$ = 0.06, $Tau^2$ = 0.00** | | |
| **Fixed** | | | | | | **0.06 (0.02, 0.11)** | | | | | | **0.06 (0.02, 0.10)** | | |
| **Random** | | | | | | **0.08 (0.02, 0.13)** | | | | | | **0.06 (0.01, 0.10)** | | |

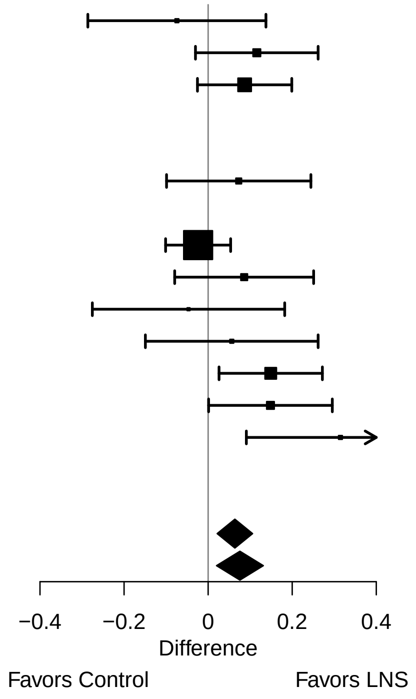


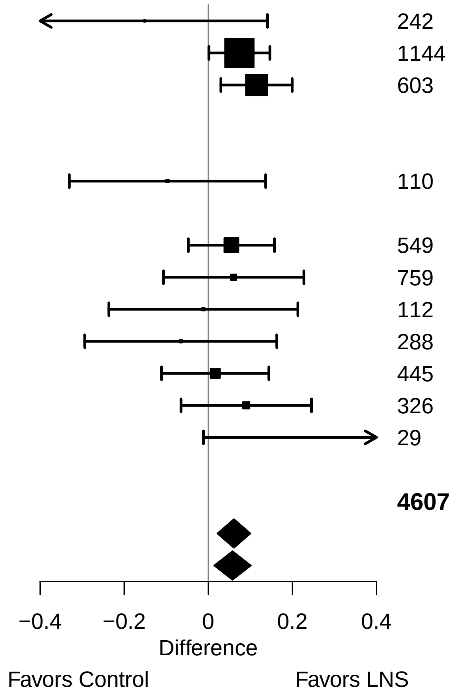

## Supplemental figure 7J: Mean difference in gross motor z-score

## 7J4: Stratified by Maternal education

**P−for−interaction = 0.104**
**Difference in MDs = 0.06 (−0.01, 0.13)**

| | | | Primary or greater | | | | | | | Incomplete or no formal | | | | | |
|---|---|---|---|---|---|---|---|---|---|---|---|---|---|---|---|
| **Country** | **Trial** | **Tool** | **LNS N** | **Control N** | **Control Mean** | **MD (95% CI)** | **Fixed W** | **Random W** | **LNS N** | **Control N** | **Control Mean** | **MD (95% CI)** | **Fixed W** | **Random W** |
| Bangladesh | JiVitA−4 (21) | BSID−III | 292 | 92 | 0.24 | −0.19 (−0.37, −0.01) | 0.04 | 0.09 | 152 | 51 | −0.19 | 0.06 (−0.29, 0.40) | 0.02 | 0.02 |
| Bangladesh | RDNS (22) | DMC | 1178 | 549 | 0.03 | 0.09 (0.02, 0.15) | 0.31 | 0.14 | 392 | 209 | −0.19 | 0.05 (−0.11, 0.21) | 0.08 | 0.08 |
| Bangladesh | WASH−B (23) | EASQ | 755 | 2344 | 0.04 | 0.08 (0.00, 0.17) | 0.20 | 0.13 | 319 | 935 | −0.17 | 0.16 (0.04, 0.29) | 0.13 | 0.13 |
| Burkina Faso | iLiNS−Zinc (24) | DMC | | | | | | | | | | | | |
| Ghana | GHANA (25) | | | | | | | | | | | | | |
| Ghana | iLiNS−DYAD−G (26) | KDI | 235 | 467 | −0.01 | 0.00 (−0.15, 0.15) | 0.06 | 0.10 | 67 | 134 | 0.00 | 0.04 (−0.28, 0.36) | 0.02 | 0.02 |
| Haiti | HAITI (27) | | | | | | | | | | | | | |
| Kenya | WASH−B (28) | EASQ | 658 | 2258 | 0.11 | −0.01 (−0.10, 0.08) | 0.16 | 0.13 | 702 | 2484 | −0.11 | 0.02 (−0.07, 0.12) | 0.23 | 0.23 |
| Madagascar | MAHAY (29) | ASQI | 339 | 416 | 0.20 | −0.07 (−0.26, 0.11) | 0.04 | 0.08 | 1274 | 1188 | −0.12 | 0.13 (−0.02, 0.29) | 0.08 | 0.08 |
| Malawi | iLiNS−DYAD−M (30) | KDI | 33 | 67 | 0.06 | −0.04 (−0.40, 0.31) | 0.01 | 0.04 | 180 | 366 | 0.01 | −0.04 (−0.21, 0.14) | 0.06 | 0.06 |
| Malawi | iLiNS−DOSE (31) | KDI | 148 | 52 | 0.22 | −0.04 (−0.29, 0.20) | 0.02 | 0.06 | 487 | 162 | −0.07 | 0.02 (−0.16, 0.21) | 0.06 | 0.06 |
| Mali | PROMIS CS (32) | DMC | 103 | 96 | 0.11 | −0.04 (−0.34, 0.25) | 0.02 | 0.05 | 836 | 861 | −0.06 | 0.10 (0.02, 0.18) | 0.30 | 0.30 |
| Zimbabwe | SHINE (HIV−) (33) | MDAT | 763 | 731 | −0.05 | 0.12 (0.01, 0.24) | 0.11 | 0.12 | 29 | 25 | −0.02 | −0.26 (−0.74, 0.22) | 0.01 | 0.01 |
| Zimbabwe | SHINE (HIV+) (34) | MDAT | 149 | 130 | −0.16 | 0.37 (0.17, 0.57) | 0.03 | 0.08 | 9 | 8 | −0.12 | 0.04 (−0.57, 0.65) | 0.01 | 0.01 |
| | | | **4653** | **7202** | | **I² = 0.60, Tau² = 0.01** | | | **4447** | **6423** | | **I² = 0.00, Tau² = 0.00** | | |
| **Fixed** | | | | | | **0.06 (0.02, 0.09)** | | | | | | **0.07 (0.02, 0.11)** | | |
| **Random** | | | | | | **0.04 (−0.04, 0.12)** | | | | | | **0.07 (0.02, 0.11)** | | |

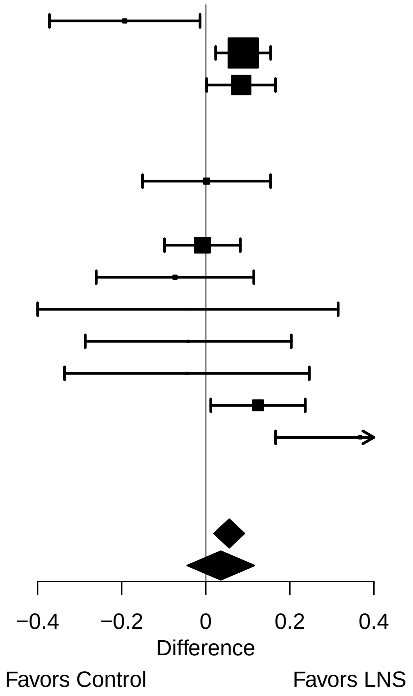


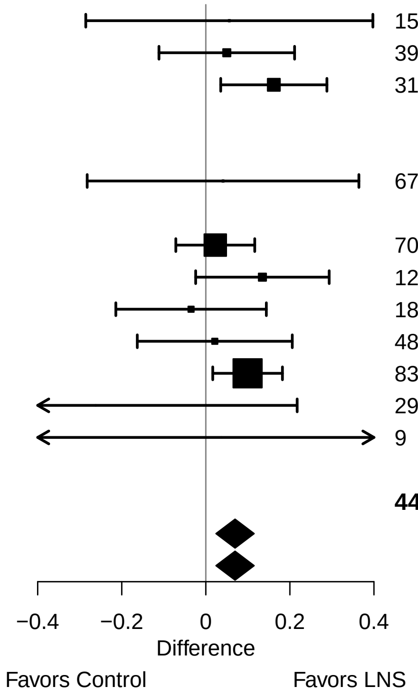

## Supplemental figure 7J: Mean difference in gross motor z-score

### 7J5: Stratified by Maternal depressive symptoms

**P−for−interaction = 0.753**
**Difference in MDs = −0.01 (−0.08, 0.06)**

| | | | Less than 75th percentile | | | | | | | At least 75th percentile | | | | | |
|---|---|---|---|---|---|---|---|---|---|---|---|---|---|---|---|
| **Country** | **Trial** | **Tool** | **LNS N** | **Control N** | **Control Mean** | **MD (95% CI)** | **Fixed W** | **Random W** | **LNS N** | **Control N** | **Control Mean** | **MD (95% CI)** | **Fixed W** | **Random W** |
| Bangladesh | JiVitA−4 (21) | BSID−III | | | | | | | | | | | | |
| Bangladesh | RDNS (22) | DMC | 1018 | 436 | −0.01 | 0.10 (0.00, 0.20) | 0.16 | 0.15 | 493 | 270 | −0.07 | 0.05 (−0.06, 0.15) | 0.30 | 0.30 |
| Bangladesh | WASH−B (23) | EASQ | 832 | 2358 | −0.01 | 0.11 (0.04, 0.19) | 0.29 | 0.16 | 221 | 838 | −0.08 | 0.07 (−0.07, 0.22) | 0.15 | 0.15 |
| Burkina Faso | iLiNS−Zinc (24) | DMC | | | | | | | | | | | | |
| Ghana | GHANA (25) | | | | | | | | | | | | | |
| Ghana | iLiNS−DYAD−G (26) | KDI | 226 | 393 | 0.00 | −0.04 (−0.21, 0.12) | 0.05 | 0.11 | 67 | 190 | −0.03 | 0.16 (−0.10, 0.42) | 0.05 | 0.05 |
| Haiti | HAITI (27) | | | | | | | | | | | | | |
| Kenya | WASH−B (28) | EASQ | 960 | 3317 | 0.04 | −0.02 (−0.09, 0.06) | 0.29 | 0.16 | 317 | 1156 | 0.09 | 0.03 (−0.08, 0.13) | 0.33 | 0.33 |
| Madagascar | MAHAY (29) | ASQI | 615 | 667 | 0.05 | 0.09 (−0.08, 0.27) | 0.05 | 0.11 | 268 | 242 | −0.05 | 0.12 (−0.14, 0.39) | 0.05 | 0.05 |
| Malawi | iLiNS−DYAD−M (30) | KDI | 152 | 299 | −0.01 | −0.01 (−0.20, 0.18) | 0.04 | 0.10 | 47 | 112 | 0.04 | −0.13 (−0.50, 0.25) | 0.02 | 0.02 |
| Malawi | iLiNS−DOSE (31) | KDI | | | | | | | | | | | | |
| Mali | PROMIS CS (32) | DMC | | | | | | | | | | | | |
| Zimbabwe | SHINE (HIV−) (33) | MDAT | 583 | 560 | −0.06 | 0.08 (−0.05, 0.21) | 0.09 | 0.13 | 197 | 180 | −0.02 | 0.18 (−0.01, 0.37) | 0.09 | 0.09 |
| Zimbabwe | SHINE (HIV+) (34) | MDAT | 131 | 110 | −0.24 | 0.43 (0.19, 0.68) | 0.03 | 0.08 | 33 | 32 | 0.13 | −0.19 (−0.77, 0.40) | 0.01 | 0.01 |
| | | | **4517** | **8140** | | **I² = 0.62, Tau² = 0.01** | | | **1643** | **3020** | | **I² = 0.00, Tau² = 0.00** | | |
| **Fixed** | | | | | | **0.06 (0.03, 0.10)** | | | | | | **0.06 (0.00, 0.12)** | | |
| **Random** | | | | | | **0.08 (−0.01, 0.17)** | | | | | | **0.06 (0.00, 0.12)** | | |

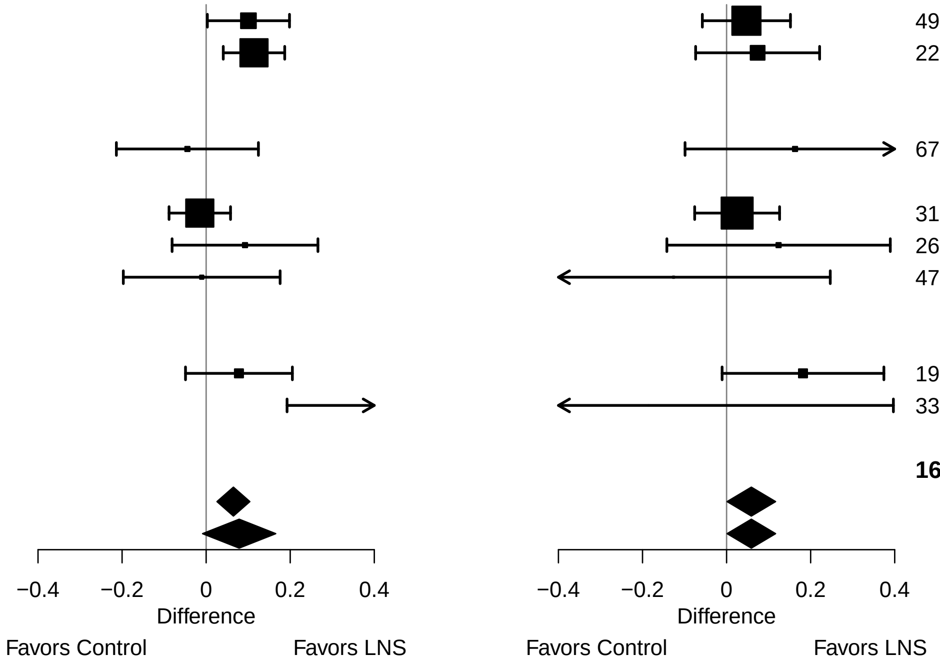

**Supplemental figure 7J: Mean difference in gross motor z-score**

## 7J6: Stratified by Child sex

**P−for−interaction = 0.155**
**Difference in MDs = −0.04 (−0.09, 0.01)**

**Male**

| Country | Trial | Tool | LNS N | Control N | Control Mean | MD (95% CI) | Fixed W | Random W |
|---|---|---|---|---|---|---|---|---|
| Bangladesh | JiVitA−4 (21) | BSID−III | 231 | 70 | 0.19 | −0.24 (−0.44, −0.03) | 0.04 | 0.06 |
| Bangladesh | RDNS (22) | DMC | 790 | 390 | −0.03 | 0.06 (−0.03, 0.14) | 0.22 | 0.15 |
| Bangladesh | WASH−B (23) | EASQ | 528 | 1646 | −0.02 | 0.08 (−0.02, 0.18) | 0.16 | 0.13 |
| Burkina Faso | iLiNS−Zinc (24) | DMC | | | | | | |
| Ghana | GHANA (25) | | | | | | | |
| Ghana | iLiNS−DYAD−G (26) | KDI | 150 | 280 | −0.04 | 0.11 (−0.09, 0.30) | 0.04 | 0.06 |
| Haiti | HAITI (27) | | | | | | | |
| Kenya | WASH−B (28) | EASQ | 682 | 2260 | −0.01 | 0.03 (−0.05, 0.12) | 0.20 | 0.15 |
| Madagascar | MAHAY (29) | ASQI | 795 | 787 | −0.08 | 0.16 (0.00, 0.33) | 0.06 | 0.08 |
| Malawi | iLiNS−DYAD−M (30) | KDI | 104 | 204 | −0.05 | 0.17 (−0.07, 0.41) | 0.03 | 0.05 |
| Malawi | iLiNS−DOSE (31) | KDI | 321 | 117 | −0.01 | 0.02 (−0.19, 0.23) | 0.03 | 0.06 |
| Mali | PROMIS CS (32) | DMC | 473 | 522 | −0.07 | 0.14 (0.03, 0.25) | 0.13 | 0.13 |
| Zimbabwe | SHINE (HIV−) (33) | MDAT | 424 | 388 | −0.05 | 0.10 (−0.04, 0.24) | 0.07 | 0.09 |
| Zimbabwe | SHINE (HIV+) (34) | MDAT | 84 | 79 | −0.11 | 0.22 (−0.06, 0.49) | 0.02 | 0.04 |
| | | | **4582** | **6743** | | **I² = 0.33, Tau² = 0.00** | | |
| **Fixed** | | | | | | **0.07 (0.03, 0.11)** | | |
| **Random** | | | | | | **0.07 (0.02, 0.13)** | | |

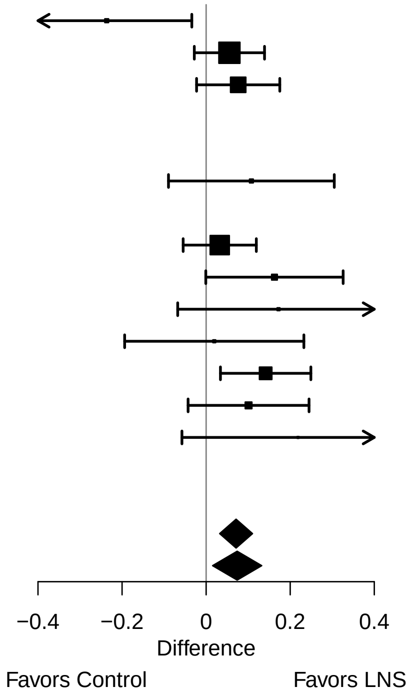


**Female**

| Country | Trial | LNS N | Control N | Control Mean | MD (95% CI) | Fixed W | Random W |
|---|---|---|---|---|---|---|---|
| Bangladesh | JiVitA−4 (21) | 214 | 73 | −0.02 | 0.04 (−0.18, 0.25) | 0.03 | 0.07 |
| Bangladesh | RDNS (22) | 780 | 368 | −0.04 | 0.11 (0.03, 0.20) | 0.21 | 0.12 |
| Bangladesh | WASH−B (23) | 546 | 1633 | −0.03 | 0.13 (0.04, 0.23) | 0.18 | 0.12 |
| Burkina Faso | iLiNS−Zinc (24) | | | | | | |
| Ghana | GHANA (25) | | | | | | |
| Ghana | iLiNS−DYAD−G (26) | 152 | 321 | 0.03 | −0.08 (−0.28, 0.11) | 0.04 | 0.08 |
| Haiti | HAITI (27) | | | | | | |
| Kenya | WASH−B (28) | 680 | 2485 | 0.00 | −0.01 (−0.09, 0.07) | 0.23 | 0.13 |
| Madagascar | MAHAY (29) | 818 | 817 | 0.00 | −0.01 (−0.18, 0.16) | 0.06 | 0.09 |
| Malawi | iLiNS−DYAD−M (30) | 110 | 232 | 0.07 | −0.22 (−0.43, −0.01) | 0.03 | 0.07 |
| Malawi | iLiNS−DOSE (31) | 325 | 104 | 0.01 | −0.01 (−0.23, 0.20) | 0.03 | 0.07 |
| Mali | PROMIS CS (32) | 467 | 435 | −0.01 | 0.02 (−0.09, 0.14) | 0.11 | 0.11 |
| Zimbabwe | SHINE (HIV−) (33) | 417 | 411 | −0.04 | 0.10 (−0.07, 0.26) | 0.06 | 0.09 |
| Zimbabwe | SHINE (HIV+) (34) | 86 | 72 | −0.26 | 0.48 (0.16, 0.79) | 0.02 | 0.05 |
| | | **4595** | **6951** | | **I² = 0.57, Tau² = 0.01** | | |
| **Fixed** | | | | | **0.05 (0.01, 0.09)** | | |
| **Random** | | | | | **0.04 (−0.04, 0.12)** | | |

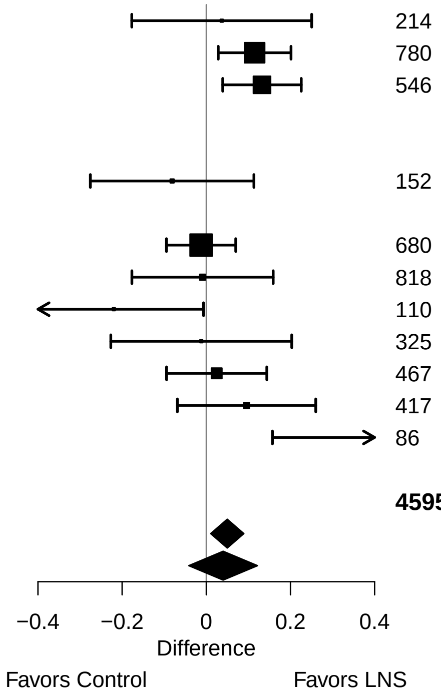

## Supplemental figure 7J: Mean difference in gross motor z-score

## 7J7: Stratified by Child birth order

**P−for−interaction = 0.145**
**Difference in MDs = −0.05 (−0.12, 0.02)**

| Country | Trial | Tool | Later born LNS N | Later born Control N | Later born Control Mean | Later born MD (95% CI) | Later born Fixed W | Later born Random W | Firstborn LNS N | Firstborn Control N | Firstborn Control Mean | Firstborn MD (95% CI) | Firstborn Fixed W | Firstborn Random W |
|---|---|---|---|---|---|---|---|---|---|---|---|---|---|---|
| Bangladesh | JiVitA−4 (21) | BSID−III | 111 | 31 | −0.15 | 0.03 (−0.36, 0.41) | 0.01 | 0.01 | 333 | 111 | 0.14 | −0.12 (−0.28, 0.04) | 0.11 | 0.12 |
| Bangladesh | RDNS (22) | DMC | 916 | 471 | −0.09 | 0.13 (0.03, 0.23) | 0.13 | 0.14 | 653 | 287 | 0.05 | 0.01 (−0.10, 0.12) | 0.24 | 0.16 |
| Bangladesh | WASH−B (23) | EASQ | 675 | 2149 | −0.03 | 0.11 (0.02, 0.19) | 0.18 | 0.17 | 379 | 1028 | 0.02 | 0.07 (−0.04, 0.18) | 0.22 | 0.15 |
| Burkina Faso | iLiNS−Zinc (24) | DMC | | | | | | | | | | | | |
| Ghana | GHANA (25) | | | | | | | | | | | | | |
| Ghana | iLiNS−DYAD−G (26) | KDI | 203 | 406 | −0.05 | 0.09 (−0.09, 0.27) | 0.04 | 0.05 | 99 | 195 | 0.10 | −0.16 (−0.37, 0.06) | 0.06 | 0.08 |
| Haiti | HAITI (27) | | | | | | | | | | | | | |
| Kenya | WASH−B (28) | EASQ | 1071 | 3767 | −0.02 | 0.01 (−0.06, 0.08) | 0.26 | 0.22 | 289 | 974 | 0.07 | 0.01 (−0.14, 0.16) | 0.12 | 0.12 |
| Madagascar | MAHAY (29) | ASQI | 1162 | 1118 | −0.08 | 0.07 (−0.08, 0.22) | 0.05 | 0.06 | 433 | 465 | 0.04 | 0.11 (−0.09, 0.30) | 0.07 | 0.09 |
| Malawi | iLiNS−DYAD−M (30) | KDI | 169 | 349 | 0.01 | −0.04 (−0.23, 0.14) | 0.04 | 0.04 | 45 | 85 | 0.04 | 0.02 (−0.30, 0.34) | 0.03 | 0.05 |
| Malawi | iLiNS−DOSE (31) | KDI | 425 | 151 | −0.06 | 0.07 (−0.12, 0.25) | 0.04 | 0.04 | 134 | 41 | 0.21 | −0.11 (−0.42, 0.21) | 0.03 | 0.05 |
| Mali | PROMIS CS (32) | DMC | 780 | 819 | −0.04 | 0.12 (0.03, 0.21) | 0.16 | 0.16 | 145 | 126 | −0.03 | −0.07 (−0.32, 0.17) | 0.05 | 0.07 |
| Zimbabwe | SHINE (HIV−) (33) | MDAT | 602 | 574 | 0.00 | 0.10 (−0.03, 0.24) | 0.07 | 0.08 | 203 | 191 | −0.15 | 0.08 (−0.13, 0.29) | 0.06 | 0.09 |
| Zimbabwe | SHINE (HIV+) (34) | MDAT | 149 | 118 | −0.14 | 0.29 (0.08, 0.50) | 0.03 | 0.03 | 18 | 29 | −0.30 | 0.73 (0.15, 1.31) | 0.01 | 0.02 |
| | | | **6263** | **9953** | | **I² = 0.18, Tau² = 0.00** | | | **2731** | **3532** | | **I² = 0.30, Tau² = 0.01** | | |
| **Fixed** | | | | | | **0.08 (0.05, 0.12)** | | | | | | **0.01 (−0.04, 0.06)** | | |
| **Random** | | | | | | **0.08 (0.04, 0.12)** | | | | | | **0.01 (−0.07, 0.09)** | | |

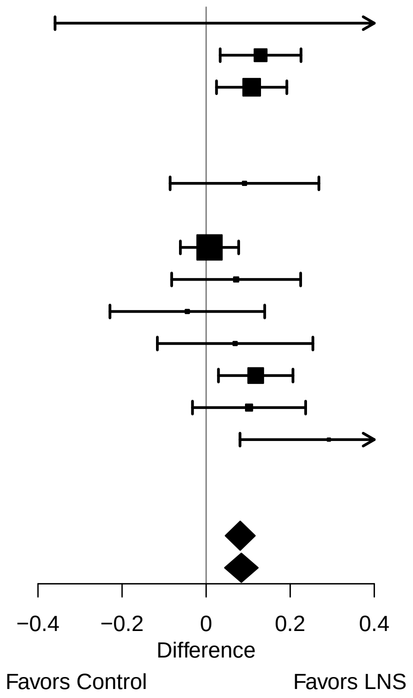


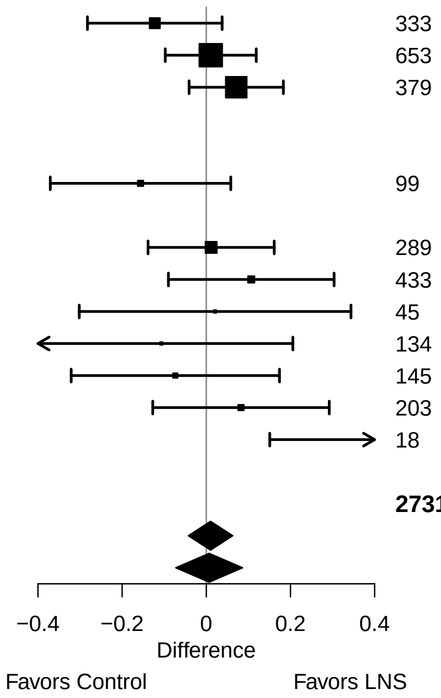

## Supplemental figure 7J: Mean difference in gross motor z-score

## 7J8: Stratified by Child baseline stunting

**P−for−interaction = 0.312**
**Difference in MDs = 0.06 (−0.05, 0.17)**

| Country | Trial | Tool | No: LNS N | No: Control N | No: Control Mean | No: MD (95% CI) | No: Fixed W | No: Random W | Yes: LNS N | Yes: Control N | Yes: Control Mean | Yes: MD (95% CI) | Yes: Fixed W | Yes: Random W |
|---|---|---|---|---|---|---|---|---|---|---|---|---|---|---|
| Bangladesh | JiVitA−4 (21) | BSID−III | 327 | 114 | 0.14 | −0.13 (−0.29, 0.04) | 0.07 | 0.13 | 116 | 29 | −0.16 | 0.04 (−0.31, 0.39) | 0.08 | 0.13 |
| Bangladesh | RDNS (22) | DMC | 1180 | 537 | 0.00 | 0.08 (0.02, 0.14) | 0.57 | 0.22 | 331 | 168 | −0.14 | 0.09 (−0.08, 0.26) | 0.37 | 0.21 |
| Bangladesh | WASH−B (23) | EASQ | | | | | | | | | | | | |
| Burkina Faso | iLiNS−Zinc (24) | DMC | | | | | | | | | | | | |
| Ghana | GHANA (25) | | | | | | | | | | | | | |
| Ghana | iLiNS−DYAD−G (26) | KDI | 259 | 504 | 0.03 | −0.03 (−0.18, 0.11) | 0.09 | 0.15 | 22 | 57 | −0.28 | 0.32 (−0.23, 0.88) | 0.03 | 0.07 |
| Haiti | HAITI (27) | | | | | | | | | | | | | |
| Kenya | WASH−B (28) | EASQ | | | | | | | | | | | | |
| Madagascar | MAHAY (29) | ASQI | | | | | | | | | | | | |
| Malawi | iLiNS−DYAD−M (30) | KDI | 152 | 320 | 0.09 | −0.06 (−0.23, 0.12) | 0.07 | 0.13 | 48 | 97 | −0.17 | 0.01 (−0.39, 0.42) | 0.06 | 0.11 |
| Malawi | iLiNS−DOSE (31) | KDI | 465 | 147 | 0.13 | −0.04 (−0.20, 0.13) | 0.07 | 0.13 | 181 | 74 | −0.26 | 0.04 (−0.27, 0.35) | 0.11 | 0.14 |
| Mali | PROMIS CS (32) | DMC | | | | | | | | | | | | |
| Zimbabwe | SHINE (HIV−) (33) | MDAT | 532 | 490 | 0.01 | 0.09 (−0.05, 0.24) | 0.10 | 0.15 | 122 | 105 | −0.19 | 0.00 (−0.20, 0.21) | 0.25 | 0.19 |
| Zimbabwe | SHINE (HIV+) (34) | MDAT | 115 | 89 | −0.11 | 0.30 (0.04, 0.55) | 0.03 | 0.08 | 34 | 32 | −0.44 | 0.66 (0.34, 0.99) | 0.10 | 0.14 |
| | | | **3030** | **2201** | | **I² = 0.54, Tau² = 0.01** | | | **854** | **562** | | **I² = 0.54, Tau² = 0.03** | | |
| **Fixed** | | | | | | **0.05 (0.00, 0.09)** | | | | | | **0.12 (0.01, 0.22)** | | |
| **Random** | | | | | | **0.02 (−0.07, 0.11)** | | | | | | **0.15 (−0.03, 0.32)** | | |

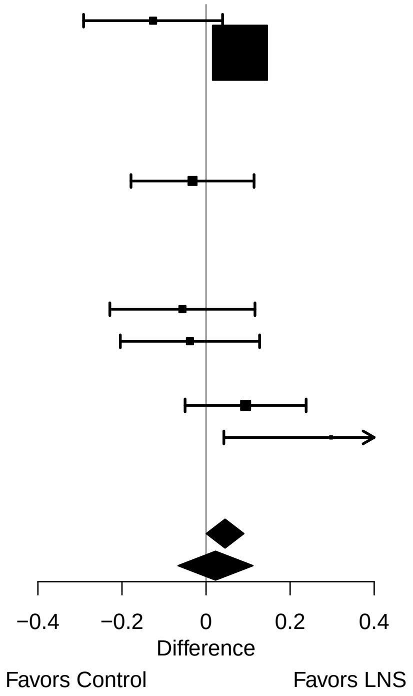


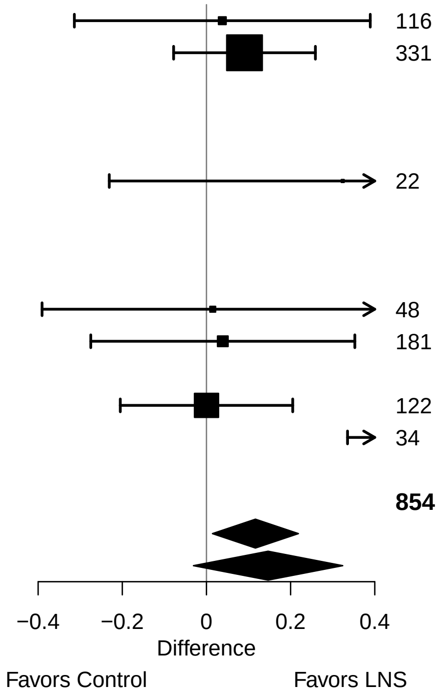

## Supplemental figure 7J: Mean difference in gross motor z-score

## 7J9: Stratified by Child baseline acute malnutrition

**P−for−interaction = 0.417**
**Difference in MDs = 0.08 (−0.11, 0.26)**

| Country | Trial | Tool | No: LNS N | No: Control N | No: Control Mean | No: MD (95% CI) | No: Fixed W | No: Random W | Yes: LNS N | Yes: Control N | Yes: Control Mean | Yes: MD (95% CI) | Yes: Fixed W | Yes: Random W |
|---|---|---|---|---|---|---|---|---|---|---|---|---|---|---|
| Bangladesh | JiVitA−4 (21) | BSID−III | 359 | 112 | 0.15 | −0.14 (−0.34, 0.05) | 0.05 | 0.12 | 84 | 31 | −0.16 | 0.04 (−0.31, 0.40) | 0.24 | 0.24 |
| Bangladesh | RDNS (22) | DMC | 1396 | 657 | −0.02 | 0.09 (0.03, 0.15) | 0.54 | 0.18 | 115 | 49 | −0.23 | 0.05 (−0.34, 0.44) | 0.21 | 0.21 |
| Bangladesh | WASH−B (23) | EASQ | | | | | | | | | | | | |
| Burkina Faso | iLiNS−Zinc (24) | DMC | | | | | | | | | | | | |
| Ghana | GHANA (25) | | | | | | | | | | | | | |
| Ghana | iLiNS−DYAD−G (26) | KDI | 265 | 509 | 0.02 | 0.00 (−0.15, 0.15) | 0.09 | 0.14 | 17 | 52 | −0.17 | −0.08 (−0.50, 0.34) | 0.18 | 0.18 |
| Haiti | HAITI (27) | | | | | | | | | | | | | |
| Kenya | WASH−B (28) | EASQ | | | | | | | | | | | | |
| Madagascar | MAHAY (29) | ASQI | | | | | | | | | | | | |
| Malawi | iLiNS−DYAD−M (30) | KDI | 186 | 383 | 0.03 | −0.03 (−0.19, 0.14) | 0.07 | 0.14 | 14 | 37 | −0.21 | −0.01 (−0.86, 0.83) | 0.04 | 0.04 |
| Malawi | iLiNS−DOSE (31) | KDI | 609 | 206 | 0.03 | 0.01 (−0.14, 0.16) | 0.08 | 0.14 | 37 | 15 | −0.40 | −0.14 (−0.93, 0.65) | 0.05 | 0.05 |
| Mali | PROMIS CS (32) | DMC | | | | | | | | | | | | |
| Zimbabwe | SHINE (HIV−) (33) | MDAT | 620 | 566 | −0.02 | 0.07 (−0.06, 0.19) | 0.12 | 0.16 | 36 | 34 | −0.11 | 0.34 (−0.07, 0.75) | 0.19 | 0.19 |
| Zimbabwe | SHINE (HIV+) (34) | MDAT | 137 | 114 | −0.17 | 0.38 (0.17, 0.60) | 0.04 | 0.11 | 14 | 8 | −0.54 | 0.55 (−0.03, 1.13) | 0.09 | 0.09 |
| | | | **3572** | **2547** | | **I² = 0.64, Tau² = 0.02** | | | **317** | **226** | | **I² = 0.00, Tau² = 0.00** | | |
| **Fixed** | | | | | | **0.07 (0.02, 0.11)** | | | | | | **0.11 (−0.06, 0.29)** | | |
| **Random** | | | | | | **0.05 (−0.06, 0.16)** | | | | | | **0.11 (−0.06, 0.29)** | | |

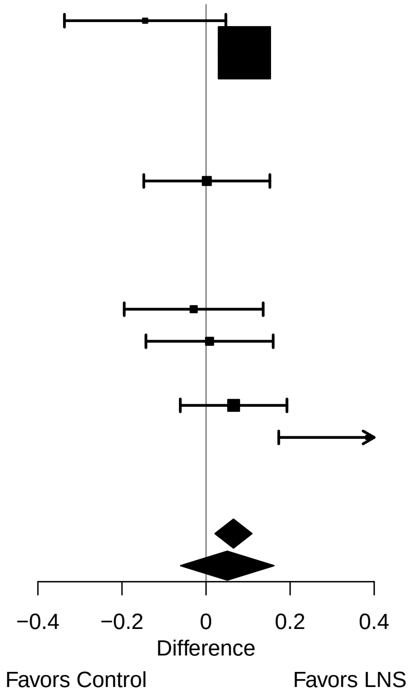


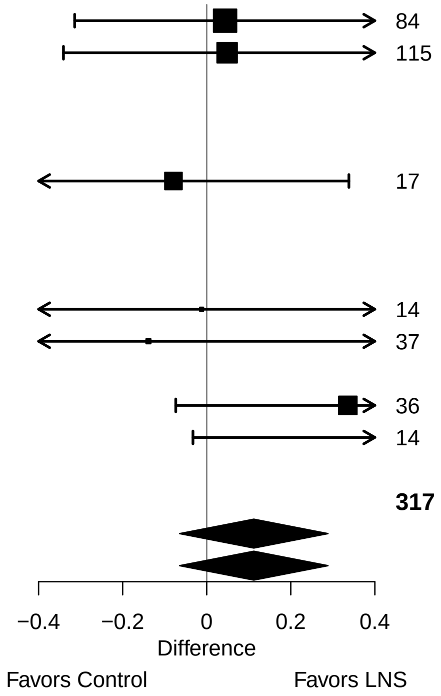

## Supplemental figure 7J: Mean difference in gross motor z-score

## 7J10: Stratified by Child baseline anemia

**P−for−interaction = 0.658**
**Difference in MDs = 0.03 (−0.12, 0.19)**

| | | | Not anemic | | | | | | Anemic | | | | | |
|---|---|---|---|---|---|---|---|---|---|---|---|---|---|---|
| Country | Trial | Tool | LNS N | Control N | Control Mean | MD (95% CI) | Fixed W | Random W | LNS N | Control N | Control Mean | MD (95% CI) | Fixed W | Random W |
| Bangladesh | JiVitA−4 (21) | BSID−III | | | | | | | | | | | | |
| Bangladesh | RDNS (22) | DMC | 202 | 91 | −0.16 | 0.19 (−0.03, 0.40) | 0.27 | 0.27 | 304 | 151 | −0.08 | 0.14 (0.02, 0.27) | 0.50 | 0.46 |
| Bangladesh | WASH−B (23) | EASQ | | | | | | | | | | | | |
| Burkina Faso | iLiNS−Zinc (24) | DMC | | | | | | | | | | | | |
| Ghana | GHANA (25) | | | | | | | | | | | | | |
| Ghana | iLiNS−DYAD−G (26) | KDI | 180 | 321 | 0.03 | −0.04 (−0.23, 0.14) | 0.34 | 0.33 | 81 | 189 | −0.09 | 0.12 (−0.14, 0.38) | 0.12 | 0.13 |
| Haiti | HAITI (27) | | | | | | | | | | | | | |
| Kenya | WASH−B (28) | EASQ | | | | | | | | | | | | |
| Madagascar | MAHAY (29) | ASQI | | | | | | | | | | | | |
| Malawi | iLiNS−DYAD−M (30) | KDI | 65 | 146 | −0.01 | 0.01 (−0.28, 0.29) | 0.15 | 0.15 | 138 | 273 | 0.04 | −0.07 (−0.27, 0.12) | 0.20 | 0.21 |
| Malawi | iLiNS−DOSE (31) | KDI | 224 | 94 | 0.13 | −0.05 (−0.27, 0.17) | 0.25 | 0.25 | 418 | 126 | −0.10 | 0.06 (−0.15, 0.26) | 0.19 | 0.20 |
| Mali | PROMIS CS (32) | DMC | | | | | | | | | | | | |
| Zimbabwe | SHINE (HIV−) (33) | MDAT | | | | | | | | | | | | |
| Zimbabwe | SHINE (HIV+) (34) | MDAT | | | | | | | | | | | | |
| | | | **671** | **652** | | **I² = 0.08, Tau² = 0.00** | | | **941** | **739** | | **I² = 0.11, Tau² = 0.00** | | |
| **Fixed** | | | | | | **0.02 (−0.09, 0.13)** | | | | | | **0.08 (−0.01, 0.17)** | | |
| **Random** | | | | | | **0.02 (−0.09, 0.14)** | | | | | | **0.08 (−0.02, 0.17)** | | |

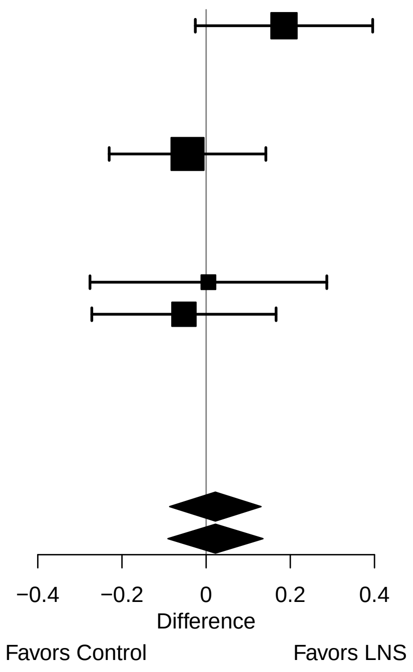


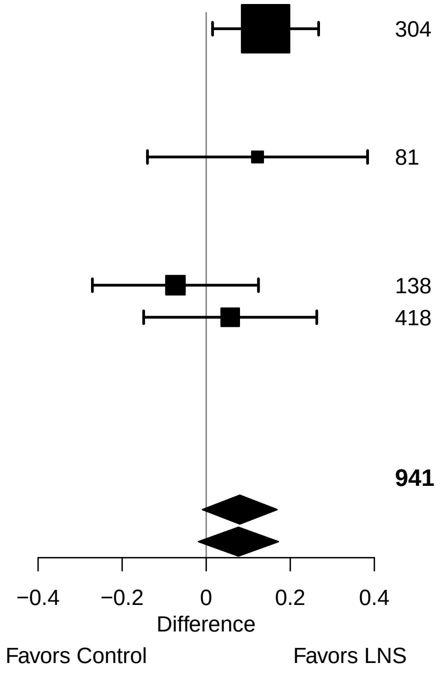

# Supplemental figure 7K: Mean difference in fine motor z-score

## 7K1: Stratified by Maternal height

**P−for−interaction = 0.069**
**Difference in MDs = −0.10 (−0.20, 0.01)**

| | | | At least 150.1 cm | | | | | | | Less than 150.1 cm | | | | | |
|---|---|---|---|---|---|---|---|---|---|---|---|---|---|---|---|
| **Country** | **Trial** | **Tool** | **LNS N** | **Control N** | **Control Mean** | **MD (95% CI)** | **Fixed W** | **Random W** | **LNS N** | **Control N** | **Control Mean** | **MD (95% CI)** | **Fixed W** | **Random W** |
| Bangladesh | JiVitA−4 (21) | BSID−III | | | | | | | | | | | | |
| Bangladesh | RDNS (22) | DMC | 857 | 430 | −0.05 | 0.16 (0.03, 0.30) | 0.16 | 0.16 | 725 | 350 | −0.06 | 0.05 (−0.07, 0.16) | 0.65 | 0.65 |
| Bangladesh | WASH−B (23) | EASQ | | | | | | | | | | | | |
| Burkina Faso | iLiNS−Zinc (24) | DMC | | | | | | | | | | | | |
| Ghana | GHANA (25) | | | | | | | | | | | | | |
| Ghana | iLiNS−DYAD−G (26) | KDI | 284 | 557 | 0.03 | 0.03 (−0.10, 0.16) | 0.19 | 0.19 | 13 | 34 | −0.44 | 0.09 (−0.93, 1.11) | 0.01 | 0.01 |
| Haiti | HAITI (27) | | | | | | | | | | | | | |
| Kenya | WASH−B (28) | EASQ | | | | | | | | | | | | |
| Madagascar | MAHAY (29) | ASQI | 956 | 986 | −0.07 | 0.08 (−0.16, 0.32) | 0.05 | 0.05 | 553 | 538 | 0.10 | −0.03 (−0.26, 0.19) | 0.18 | 0.18 |
| Malawi | iLiNS−DYAD−M (30) | KDI | 186 | 376 | −0.03 | 0.06 (−0.11, 0.23) | 0.10 | 0.10 | 28 | 57 | 0.08 | 0.21 (−0.25, 0.67) | 0.04 | 0.04 |
| Malawi | iLiNS−DOSE (31) | KDI | 543 | 185 | −0.05 | 0.09 (−0.08, 0.25) | 0.11 | 0.11 | 102 | 35 | −0.13 | 0.09 (−0.31, 0.49) | 0.05 | 0.05 |
| Mali | PROMIS CS (32) | DMC | 868 | 892 | −0.06 | 0.12 (−0.09, 0.33) | 0.06 | 0.06 | 32 | 26 | 0.12 | −0.25 (−0.82, 0.33) | 0.03 | 0.03 |
| Zimbabwe | SHINE (HIV−) (33) | MDAT | 788 | 762 | −0.04 | 0.09 (−0.01, 0.19) | 0.27 | 0.27 | 34 | 22 | −0.10 | −0.03 (−0.53, 0.47) | 0.03 | 0.03 |
| Zimbabwe | SHINE (HIV+) (34) | MDAT | 158 | 140 | −0.06 | 0.13 (−0.10, 0.36) | 0.06 | 0.06 | 10 | 6 | −0.49 | 0.71 (−0.66, 2.09) | 0.00 | 0.00 |
| | | | **4640** | **4328** | | **I² = 0.00, Tau² = 0.00** | | | **1497** | **1068** | | **I² = 0.00, Tau² = 0.00** | | |
| **Fixed** | | | | | | **0.09 (0.04, 0.14)** | | | | | | **0.04 (−0.06, 0.13)** | | |
| **Random** | | | | | | **0.09 (0.04, 0.14)** | | | | | | **0.04 (−0.06, 0.13)** | | |

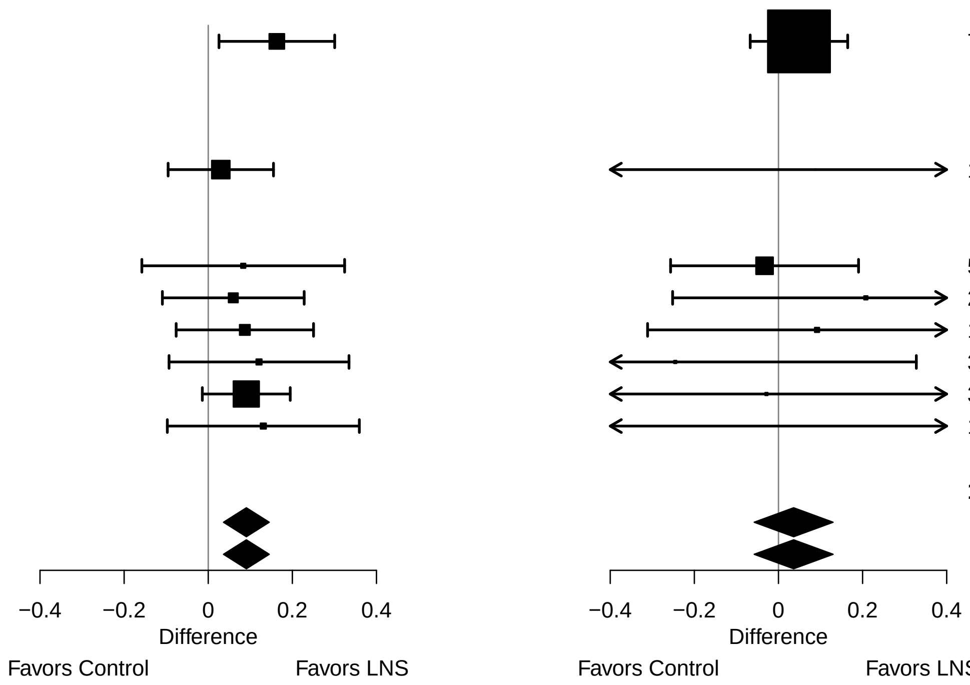

## Supplemental figure 7K: Mean difference in fine motor z-score

## 7K2: Stratified by Maternal BMI

**P−for−interaction = 0.767**
**Difference in MDs = −0.02 (−0.12, 0.09)**

| Country | Trial | Tool | At least 20 kg/m² LNS N | Control N | Control Mean | MD (95% CI) | Fixed W | Random W | Less than 20 kg/m² LNS N | Control N | Control Mean | MD (95% CI) | Fixed W | Random W |
|---|---|---|---|---|---|---|---|---|---|---|---|---|---|---|
| Bangladesh | JiVitA−4 (21) | BSID−III | | | | | | | | | | | | |
| Bangladesh | RDNS (22) | DMC | 722 | 330 | 0.01 | 0.09 (−0.05, 0.23) | 0.18 | 0.18 | 860 | 450 | −0.11 | 0.12 (0.00, 0.25) | 0.52 | 0.52 |
| Bangladesh | WASH−B (23) | EASQ | | | | | | | | | | | | |
| Burkina Faso | iLiNS−Zinc (24) | DMC | | | | | | | | | | | | |
| Ghana | GHANA (25) | | | | | | | | | | | | | |
| Ghana | iLiNS−DYAD−G (26) | KDI | 262 | 485 | 0.01 | 0.04 (−0.09, 0.18) | 0.19 | 0.19 | 35 | 106 | −0.04 | −0.02 (−0.40, 0.35) | 0.06 | 0.06 |
| Haiti | HAITI (27) | | | | | | | | | | | | | |
| Kenya | WASH−B (28) | EASQ | | | | | | | | | | | | |
| Madagascar | MAHAY (29) | ASQI | | | | | | | | | | | | |
| Malawi | iLiNS−DYAD−M (30) | KDI | 126 | 258 | −0.01 | 0.10 (−0.11, 0.31) | 0.08 | 0.08 | 87 | 175 | −0.03 | 0.05 (−0.20, 0.29) | 0.13 | 0.13 |
| Malawi | iLiNS−DOSE (31) | KDI | 480 | 158 | 0.00 | 0.03 (−0.15, 0.20) | 0.12 | 0.12 | 163 | 61 | −0.22 | 0.25 (−0.06, 0.55) | 0.09 | 0.09 |
| Mali | PROMIS CS (32) | DMC | 668 | 655 | −0.05 | 0.15 (−0.07, 0.37) | 0.08 | 0.08 | 232 | 263 | −0.06 | −0.01 (−0.29, 0.28) | 0.10 | 0.10 |
| Zimbabwe | SHINE (HIV−) (33) | MDAT | 601 | 581 | −0.04 | 0.11 (0.00, 0.22) | 0.28 | 0.28 | 100 | 96 | −0.17 | 0.19 (−0.11, 0.49) | 0.09 | 0.09 |
| Zimbabwe | SHINE (HIV+) (34) | MDAT | 124 | 113 | −0.06 | 0.24 (0.00, 0.48) | 0.06 | 0.06 | 25 | 23 | −0.08 | −0.43 (−1.04, 0.17) | 0.02 | 0.02 |
| | | | **2983** | **2580** | | **I² = 0.00, Tau² = 0.00** | | | **1502** | **1174** | | **I² = 0.00, Tau² = 0.00** | | |
| **Fixed** | | | | | | **0.09 (0.03, 0.15)** | | | | | | **0.10 (0.01, 0.19)** | | |
| **Random** | | | | | | **0.09 (0.03, 0.15)** | | | | | | **0.10 (0.01, 0.19)** | | |

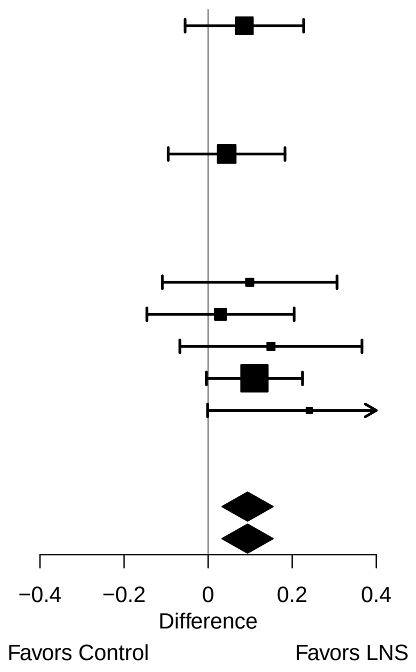


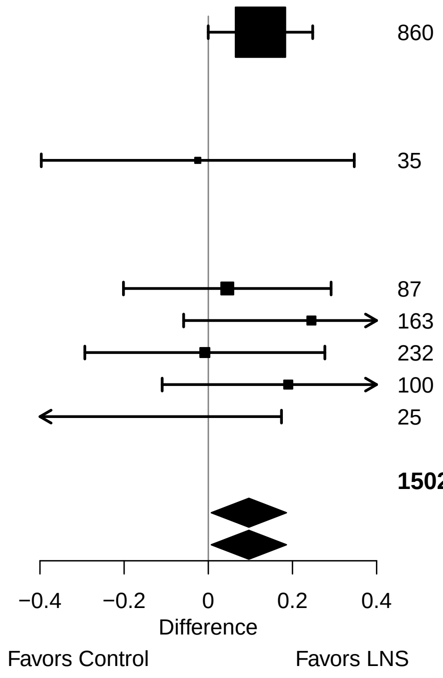

## Supplemental figure 7K: Mean difference in fine motor z-score

### 7K3: Stratified by Maternal age

P−for−interaction = 0.005
Difference in MDs = −0.10 (−0.17, −0.03)

| Country | Trial | Tool | At least 25 y: LNS N | Control N | Control Mean | MD (95% CI) | Fixed W | Random W | Less than 25 y: LNS N | Control N | Control Mean | MD (95% CI) | Fixed W | Random W |
|---|---|---|---|---|---|---|---|---|---|---|---|---|---|---|
| Bangladesh | JiVitA−4 (21) | BSID−III | 200 | 68 | 0.00 | −0.01 (−0.30, 0.28) | 0.05 | 0.05 | 242 | 74 | −0.19 | 0.25 (−0.03, 0.52) | 0.05 | 0.05 |
| Bangladesh | RDNS (22) | DMC | 451 | 218 | −0.06 | 0.17 (−0.01, 0.36) | 0.12 | 0.12 | 1190 | 586 | −0.05 | 0.07 (−0.03, 0.17) | 0.40 | 0.40 |
| Bangladesh | WASH−B (23) | EASQ | | | | | | | | | | | | |
| Burkina Faso | iLiNS−Zinc (24) | DMC | | | | | | | | | | | | |
| Ghana | GHANA (25) | | | | | | | | | | | | | |
| Ghana | iLiNS−DYAD−G (26) | KDI | 192 | 378 | 0.00 | 0.10 (−0.06, 0.25) | 0.17 | 0.17 | 110 | 223 | 0.00 | −0.06 (−0.28, 0.16) | 0.08 | 0.08 |
| Haiti | HAITI (27) | | | | | | | | | | | | | |
| Kenya | WASH−B (28) | EASQ | | | | | | | | | | | | |
| Madagascar | MAHAY (29) | ASQI | 852 | 901 | −0.07 | 0.05 (−0.17, 0.27) | 0.08 | 0.08 | 759 | 703 | 0.04 | 0.04 (−0.20, 0.27) | 0.07 | 0.07 |
| Malawi | iLiNS−DYAD−M (30) | KDI | 102 | 224 | −0.06 | 0.29 (0.07, 0.50) | 0.09 | 0.09 | 112 | 212 | 0.01 | −0.11 (−0.35, 0.12) | 0.07 | 0.07 |
| Malawi | iLiNS−DOSE (31) | KDI | 346 | 124 | −0.08 | 0.11 (−0.10, 0.31) | 0.10 | 0.10 | 288 | 90 | −0.02 | 0.05 (−0.18, 0.28) | 0.07 | 0.07 |
| Mali | PROMIS CS (32) | DMC | 482 | 480 | −0.04 | 0.17 (−0.04, 0.38) | 0.09 | 0.09 | 423 | 444 | −0.06 | 0.03 (−0.22, 0.28) | 0.06 | 0.06 |
| Zimbabwe | SHINE (HIV−) (33) | MDAT | 427 | 389 | −0.03 | 0.15 (0.01, 0.28) | 0.22 | 0.22 | 326 | 321 | −0.06 | 0.00 (−0.15, 0.14) | 0.19 | 0.19 |
| Zimbabwe | SHINE (HIV+) (34) | MDAT | 126 | 111 | −0.05 | 0.15 (−0.09, 0.39) | 0.07 | 0.07 | 29 | 26 | −0.05 | 0.00 (−0.65, 0.65) | 0.01 | 0.01 |
| | | | **3178** | **2893** | | **I² = 0.00, Tau² = 0.00** | | | **3479** | **2679** | | **I² = 0.00, Tau² = 0.00** | | |
| **Fixed** | | | | | | **0.14 (0.07, 0.20)** | | | | | | **0.03 (−0.03, 0.10)** | | |
| **Random** | | | | | | **0.14 (0.07, 0.20)** | | | | | | **0.03 (−0.03, 0.10)** | | |

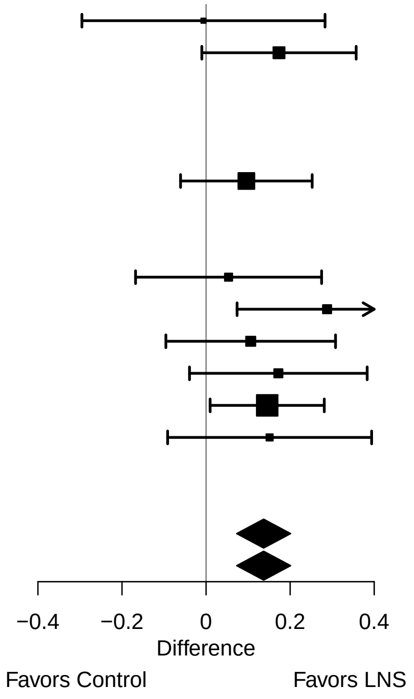


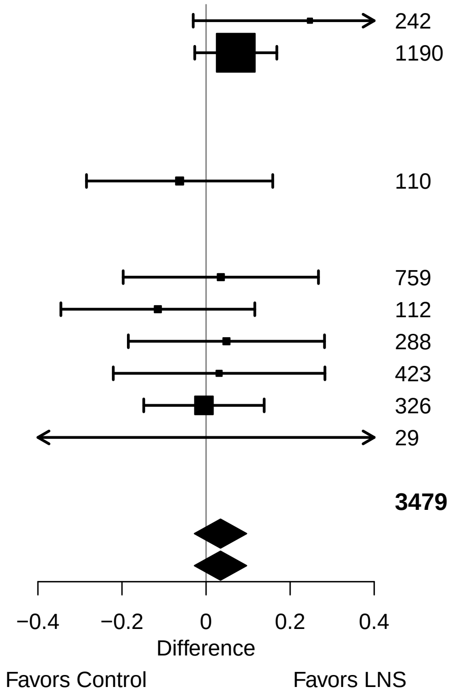

## Supplemental figure 7K: Mean difference in fine motor z-score

## 7K4: Stratified by Maternal education

**P−for−interaction = 0.003**
**Difference in MDs = 0.15 (0.05, 0.25)**

| | | | Primary or greater | | | | | | | Incomplete or no formal | | | | | |
|---|---|---|---|---|---|---|---|---|---|---|---|---|---|---|---|
| **Country** | **Trial** | **Tool** | **LNS N** | **Control N** | **Control Mean** | **MD (95% CI)** | **Fixed W** | **Random W** | **LNS N** | **Control N** | **Control Mean** | **MD (95% CI)** | **Fixed W** | **Random W** |
| Bangladesh | JiVitA−4 (21) | BSID−III | 292 | 92 | 0.05 | 0.04 (−0.17, 0.24) | 0.07 | 0.07 | 152 | 51 | −0.39 | 0.34 (0.03, 0.64) | 0.06 | 0.09 |
| Bangladesh | RDNS (22) | DMC | 1227 | 582 | 0.03 | 0.06 (−0.03, 0.16) | 0.31 | 0.31 | 414 | 222 | −0.26 | 0.17 (0.00, 0.35) | 0.19 | 0.16 |
| Bangladesh | WASH−B (23) | EASQ | | | | | | | | | | | | |
| Burkina Faso | iLiNS−Zinc (24) | DMC | | | | | | | | | | | | |
| Ghana | GHANA (25) | | | | | | | | | | | | | |
| Ghana | iLiNS−DYAD−G (26) | KDI | 235 | 467 | 0.03 | −0.03 (−0.18, 0.11) | 0.14 | 0.14 | 67 | 134 | −0.12 | 0.29 (0.02, 0.56) | 0.08 | 0.11 |
| Haiti | HAITI (27) | | | | | | | | | | | | | |
| Kenya | WASH−B (28) | EASQ | | | | | | | | | | | | |
| Madagascar | MAHAY (29) | ASQI | 339 | 416 | 0.27 | −0.14 (−0.36, 0.08) | 0.06 | 0.06 | 1274 | 1188 | −0.13 | 0.12 (−0.10, 0.35) | 0.12 | 0.13 |
| Malawi | iLiNS−DYAD−M (30) | KDI | 33 | 67 | 0.04 | −0.12 (−0.49, 0.25) | 0.02 | 0.02 | 180 | 366 | −0.03 | 0.11 (−0.06, 0.29) | 0.20 | 0.17 |
| Malawi | iLiNS−DOSE (31) | KDI | 148 | 52 | 0.19 | 0.05 (−0.24, 0.35) | 0.03 | 0.03 | 487 | 162 | −0.13 | 0.10 (−0.08, 0.27) | 0.19 | 0.16 |
| Mali | PROMIS CS (32) | DMC | 100 | 93 | 0.00 | 0.15 (−0.26, 0.55) | 0.02 | 0.02 | 804 | 831 | −0.06 | 0.10 (−0.11, 0.31) | 0.13 | 0.14 |
| Zimbabwe | SHINE (HIV−) (33) | MDAT | 763 | 731 | −0.04 | 0.10 (−0.01, 0.20) | 0.28 | 0.28 | 29 | 25 | 0.13 | −0.49 (−1.04, 0.06) | 0.02 | 0.04 |
| Zimbabwe | SHINE (HIV+) (34) | MDAT | 149 | 130 | −0.06 | 0.18 (−0.03, 0.40) | 0.06 | 0.06 | 9 | 8 | 0.04 | −0.83 (−2.38, 0.73) | 0.00 | 0.00 |
| | | | **3286** | **2630** | | **I² = 0.00, Tau² = 0.00** | | | **3416** | **2987** | | **I² = 0.19, Tau² = 0.01** | | |
| **Fixed** | | | | | | **0.05 (−0.01, 0.10)** | | | | | | **0.14 (0.06, 0.21)** | | |
| **Random** | | | | | | **0.05 (−0.01, 0.10)** | | | | | | **0.13 (0.02, 0.24)** | | |

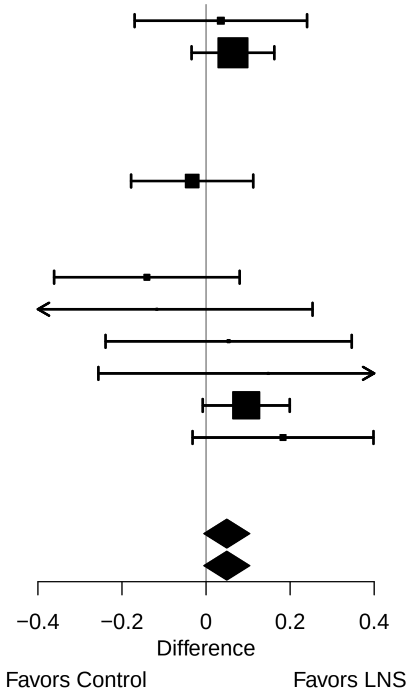


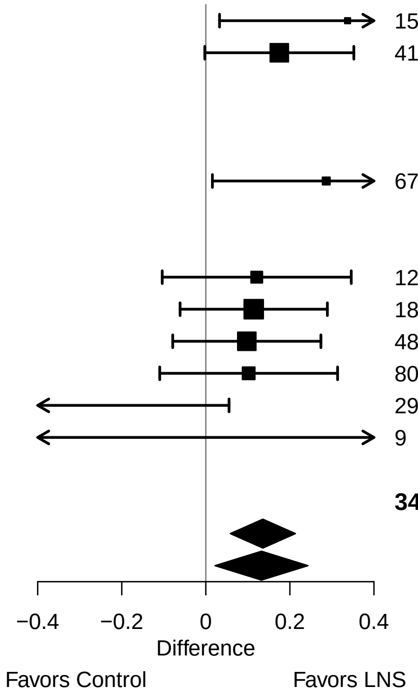

## Supplemental figure 7K: Mean difference in fine motor z-score

## 7K5: Stratified by Maternal depressive symptoms

**P−for−interaction = 0.647**
**Difference in MDs = −0.02 (−0.11, 0.07)**

| | | | Less than 75th percentile | | | | | | At least 75th percentile | | | | | |
|---|---|---|---|---|---|---|---|---|---|---|---|---|---|---|
| **Country** | **Trial** | **Tool** | **LNS N** | **Control N** | **Control Mean** | **MD (95% CI)** | **Fixed W** | **Random W** | **LNS N** | **Control N** | **Control Mean** | **MD (95% CI)** | **Fixed W** | **Random W** |
| Bangladesh | JiVitA−4 (21) | BSID−III | | | | | | | | | | | | |
| Bangladesh | RDNS (22) | DMC | 1067 | 469 | −0.03 | 0.11 (−0.02, 0.23) | 0.27 | 0.27 | 512 | 281 | −0.13 | 0.12 (0.00, 0.24) | 0.53 | 0.47 |
| Bangladesh | WASH−B (23) | EASQ | | | | | | | | | | | | |
| Burkina Faso | iLiNS−Zinc (24) | DMC | | | | | | | | | | | | |
| Ghana | GHANA (25) | | | | | | | | | | | | | |
| Ghana | iLiNS−DYAD−G (26) | KDI | 226 | 393 | 0.00 | −0.02 (−0.17, 0.13) | 0.19 | 0.19 | 67 | 190 | 0.00 | 0.32 (0.06, 0.57) | 0.11 | 0.13 |
| Haiti | HAITI (27) | | | | | | | | | | | | | |
| Kenya | WASH−B (28) | EASQ | | | | | | | | | | | | |
| Madagascar | MAHAY (29) | ASQI | 615 | 667 | −0.02 | 0.09 (−0.19, 0.37) | 0.05 | 0.05 | 268 | 242 | −0.08 | 0.06 (−0.28, 0.39) | 0.07 | 0.08 |
| Malawi | iLiNS−DYAD−M (30) | KDI | 152 | 299 | −0.07 | 0.15 (−0.04, 0.35) | 0.12 | 0.12 | 47 | 112 | 0.08 | −0.14 (−0.46, 0.18) | 0.07 | 0.08 |
| Malawi | iLiNS−DOSE (31) | KDI | | | | | | | | | | | | |
| Mali | PROMIS CS (32) | DMC | | | | | | | | | | | | |
| Zimbabwe | SHINE (HIV−) (33) | MDAT | 583 | 560 | 0.00 | 0.06 (−0.06, 0.18) | 0.31 | 0.31 | 197 | 180 | −0.17 | 0.12 (−0.08, 0.31) | 0.19 | 0.21 |
| Zimbabwe | SHINE (HIV+) (34) | MDAT | 131 | 110 | −0.07 | 0.18 (−0.07, 0.44) | 0.06 | 0.06 | 33 | 32 | −0.03 | −0.03 (−0.54, 0.47) | 0.03 | 0.03 |
| | | | **2774** | **2498** | | **I² = 0.00, Tau² = 0.00** | | | **1124** | **1037** | | **I² = 0.05, Tau² = 0.00** | | |
| **Fixed** | | | | | | **0.08 (0.01, 0.14)** | | | | | | **0.11 (0.03, 0.20)** | | |
| **Random** | | | | | | **0.08 (0.01, 0.14)** | | | | | | **0.11 (0.02, 0.21)** | | |

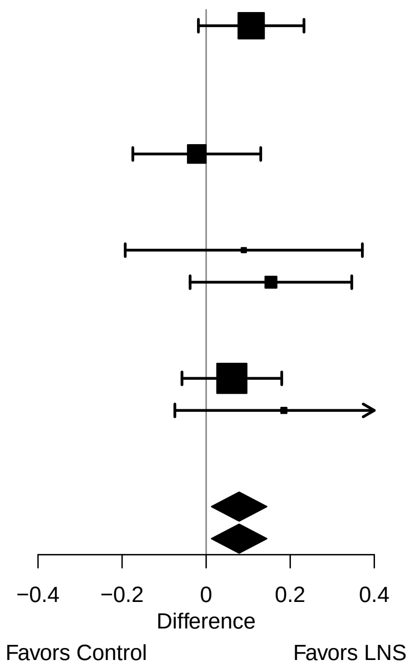


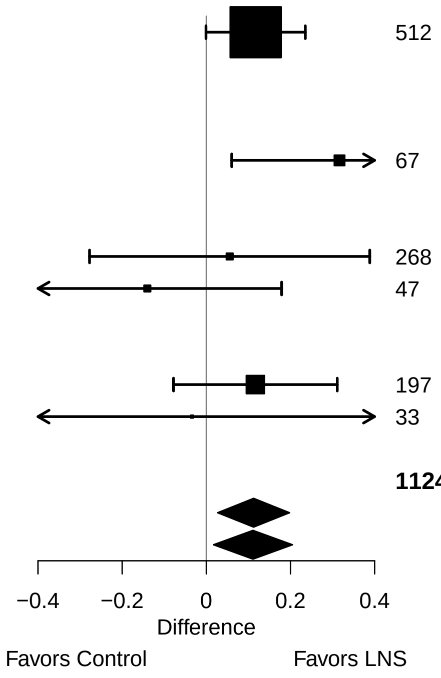

## Supplemental figure 7K: Mean difference in fine motor z-score

## 7K6: Stratified by Child sex

**P−for−interaction = 0.162**
**Difference in MDs = 0.05 (−0.02, 0.11)**

| | | | Male | | | | | | Female | | | | | |
|---|---|---|---|---|---|---|---|---|---|---|---|---|---|---|
| **Country** | **Trial** | **Tool** | **LNS N** | **Control N** | **Control Mean** | **MD (95% CI)** | **Fixed W** | **Random W** | **LNS N** | **Control N** | **Control Mean** | **MD (95% CI)** | **Fixed W** | **Random W** |
| Bangladesh | JiVitA−4 (21) | BSID−III | 231 | 70 | −0.01 | 0.02 (−0.26, 0.30) | 0.04 | 0.04 | 214 | 73 | −0.20 | 0.27 (0.07, 0.47) | 0.10 | 0.11 |
| Bangladesh | RDNS (22) | DMC | 819 | 404 | 0.00 | 0.01 (−0.10, 0.11) | 0.33 | 0.33 | 822 | 400 | −0.10 | 0.19 (0.05, 0.33) | 0.21 | 0.17 |
| Bangladesh | WASH−B (23) | EASQ | | | | | | | | | | | | |
| Burkina Faso | iLiNS−Zinc (24) | DMC | | | | | | | | | | | | |
| Ghana | GHANA (25) | | | | | | | | | | | | | |
| Ghana | iLiNS−DYAD−G (26) | KDI | 150 | 280 | −0.04 | 0.14 (−0.03, 0.31) | 0.12 | 0.12 | 152 | 321 | 0.03 | −0.05 (−0.24, 0.14) | 0.11 | 0.12 |
| Haiti | HAITI (27) | | | | | | | | | | | | | |
| Kenya | WASH−B (28) | EASQ | | | | | | | | | | | | |
| Madagascar | MAHAY (29) | ASQI | 795 | 787 | −0.03 | 0.06 (−0.15, 0.27) | 0.08 | 0.08 | 818 | 817 | −0.02 | 0.03 (−0.21, 0.27) | 0.07 | 0.08 |
| Malawi | iLiNS−DYAD−M (30) | KDI | 104 | 204 | −0.06 | 0.21 (−0.02, 0.45) | 0.06 | 0.06 | 110 | 232 | 0.01 | −0.05 (−0.26, 0.17) | 0.09 | 0.10 |
| Malawi | iLiNS−DOSE (31) | KDI | 321 | 117 | 0.00 | 0.00 (−0.22, 0.22) | 0.07 | 0.07 | 325 | 104 | −0.14 | 0.19 (−0.02, 0.40) | 0.10 | 0.10 |
| Mali | PROMIS CS (32) | DMC | 457 | 501 | −0.03 | 0.06 (−0.17, 0.29) | 0.07 | 0.07 | 448 | 423 | −0.08 | 0.16 (−0.07, 0.39) | 0.08 | 0.09 |
| Zimbabwe | SHINE (HIV−) (33) | MDAT | 424 | 388 | −0.06 | 0.12 (−0.02, 0.25) | 0.19 | 0.19 | 417 | 411 | −0.01 | 0.03 (−0.12, 0.18) | 0.18 | 0.16 |
| Zimbabwe | SHINE (HIV+) (34) | MDAT | 84 | 79 | −0.07 | 0.15 (−0.19, 0.48) | 0.03 | 0.03 | 86 | 72 | −0.09 | 0.19 (−0.09, 0.47) | 0.05 | 0.06 |
| | | | **3385** | **2830** | | **$I^2$ = 0.00, $Tau^2$ = 0.00** | | | **3392** | **2853** | | **$I^2$ = 0.27, $Tau^2$ = 0.00** | | |
| **Fixed** | | | | | | **0.07 (0.01, 0.13)** | | | | | | **0.11 (0.04, 0.17)** | | |
| **Random** | | | | | | **0.07 (0.01, 0.13)** | | | | | | **0.11 (0.03, 0.18)** | | |

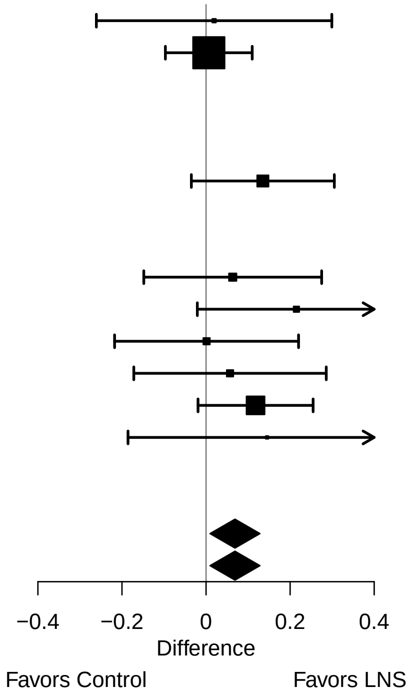


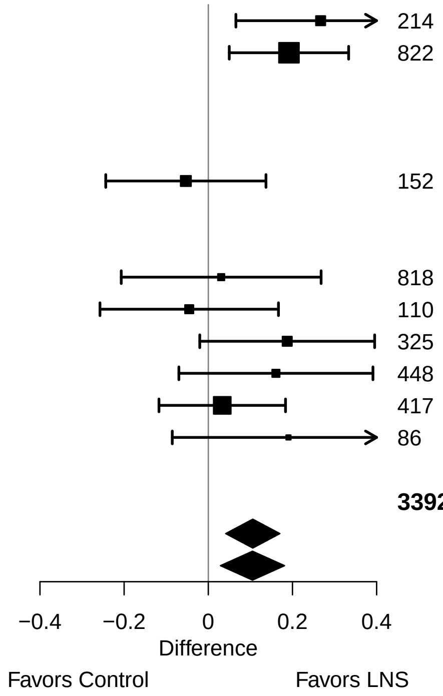

## Supplemental figure 7K: Mean difference in fine motor z-score

### 7K7: Stratified by Child birth order

**P−for−interaction = 0.062**
**Difference in MDs = −0.08 (−0.17, 0.00)**

| Country | Trial | Tool | Later born LNS N | Later born Control N | Later born Control Mean | Later born MD (95% CI) | Later born Fixed W | Later born Random W | Firstborn LNS N | Firstborn Control N | Firstborn Control Mean | Firstborn MD (95% CI) | Firstborn Fixed W | Firstborn Random W |
|---|---|---|---|---|---|---|---|---|---|---|---|---|---|---|
| Bangladesh | JiVitA−4 (21) | BSID−III | 111 | 31 | −0.35 | 0.23 (−0.18, 0.64) | 0.02 | 0.02 | 333 | 111 | −0.05 | 0.14 (−0.07, 0.34) | 0.16 | 0.16 |
| Bangladesh | RDNS (22) | DMC | 961 | 505 | −0.08 | 0.17 (0.05, 0.28) | 0.27 | 0.27 | 679 | 299 | 0.00 | 0.00 (−0.17, 0.16) | 0.24 | 0.24 |
| Bangladesh | WASH−B (23) | EASQ | | | | | | | | | | | | |
| Burkina Faso | iLiNS−Zinc (24) | DMC | | | | | | | | | | | | |
| Ghana | GHANA (25) | | | | | | | | | | | | | |
| Ghana | iLiNS−DYAD−G (26) | KDI | 203 | 406 | −0.01 | 0.02 (−0.14, 0.18) | 0.13 | 0.13 | 99 | 195 | 0.01 | 0.07 (−0.15, 0.28) | 0.15 | 0.15 |
| Haiti | HAITI (27) | | | | | | | | | | | | | |
| Kenya | WASH−B (28) | EASQ | | | | | | | | | | | | |
| Madagascar | MAHAY (29) | ASQI | 1162 | 1118 | −0.07 | 0.07 (−0.16, 0.29) | 0.07 | 0.07 | 433 | 465 | 0.07 | 0.03 (−0.21, 0.27) | 0.11 | 0.11 |
| Malawi | iLiNS−DYAD−M (30) | KDI | 169 | 349 | −0.04 | 0.09 (−0.09, 0.27) | 0.10 | 0.10 | 45 | 85 | 0.03 | 0.03 (−0.32, 0.39) | 0.05 | 0.05 |
| Malawi | iLiNS−DOSE (31) | KDI | 425 | 151 | −0.07 | 0.13 (−0.06, 0.31) | 0.10 | 0.10 | 134 | 41 | 0.06 | −0.03 (−0.39, 0.34) | 0.05 | 0.05 |
| Mali | PROMIS CS (32) | DMC | 751 | 789 | −0.05 | 0.14 (−0.09, 0.36) | 0.07 | 0.07 | 140 | 123 | −0.03 | −0.07 (−0.38, 0.24) | 0.07 | 0.07 |
| Zimbabwe | SHINE (HIV−) (33) | MDAT | 602 | 574 | −0.01 | 0.12 (−0.01, 0.25) | 0.20 | 0.20 | 203 | 191 | −0.14 | −0.02 (−0.22, 0.19) | 0.15 | 0.15 |
| Zimbabwe | SHINE (HIV+) (34) | MDAT | 149 | 118 | −0.02 | 0.08 (−0.17, 0.32) | 0.05 | 0.05 | 18 | 29 | −0.27 | 0.65 (0.15, 1.16) | 0.03 | 0.03 |
| | | | **4533** | **4041** | | **I² = 0.00, Tau² = 0.00** | | | **2084** | **1539** | | **I² = 0.00, Tau² = 0.00** | | |
| **Fixed** | | | | | | **0.12 (0.06, 0.17)** | | | | | | **0.04 (−0.04, 0.13)** | | |
| **Random** | | | | | | **0.12 (0.06, 0.17)** | | | | | | **0.04 (−0.04, 0.13)** | | |

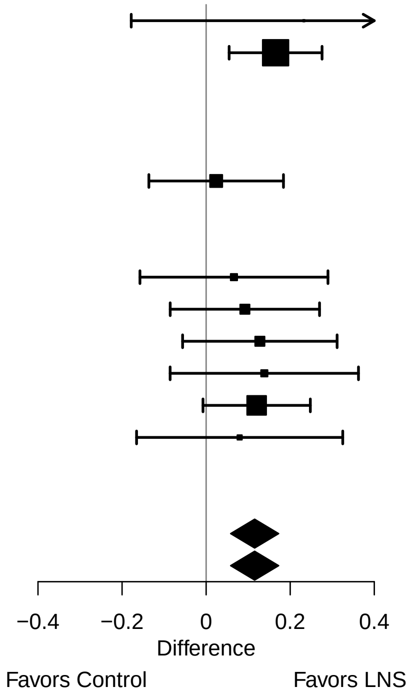


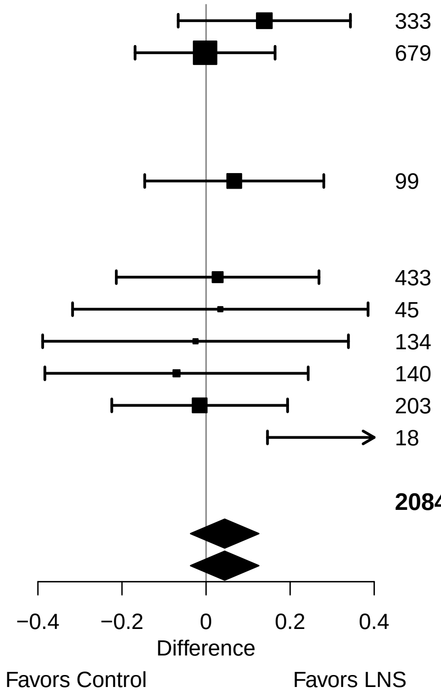

## Supplemental figure 7K: Mean difference in fine motor z-score

## 7K8: Stratified by Child baseline stunting

**P−for−interaction = 0.613**
**Difference in MDs = 0.03 (−0.09, 0.15)**

| Country | Trial | Tool | No: LNS N | No: Control N | No: Control Mean | No: MD (95% CI) | No: Fixed W | No: Random W | Yes: LNS N | Yes: Control N | Yes: Control Mean | Yes: MD (95% CI) | Yes: Fixed W | Yes: Random W |
|---|---|---|---|---|---|---|---|---|---|---|---|---|---|---|
| Bangladesh | JiVitA−4 (21) | BSID−III | 327 | 114 | −0.12 | 0.19 (−0.04, 0.42) | 0.06 | 0.06 | 116 | 29 | −0.05 | 0.01 (−0.30, 0.31) | 0.15 | 0.15 |
| Bangladesh | RDNS (22) | DMC | 1231 | 567 | −0.01 | 0.10 (0.00, 0.19) | 0.34 | 0.34 | 348 | 182 | −0.24 | 0.16 (−0.08, 0.40) | 0.24 | 0.24 |
| Bangladesh | WASH−B (23) | EASQ | | | | | | | | | | | | |
| Burkina Faso | iLiNS−Zinc (24) | DMC | | | | | | | | | | | | |
| Ghana | GHANA (25) | | | | | | | | | | | | | |
| Ghana | iLiNS−DYAD−G (26) | KDI | 259 | 504 | 0.04 | 0.00 (−0.13, 0.14) | 0.17 | 0.17 | 22 | 57 | −0.28 | 0.24 (−0.38, 0.85) | 0.04 | 0.04 |
| Haiti | HAITI (27) | | | | | | | | | | | | | |
| Kenya | WASH−B (28) | EASQ | | | | | | | | | | | | |
| Madagascar | MAHAY (29) | ASQI | | | | | | | | | | | | |
| Malawi | iLiNS−DYAD−M (30) | KDI | 152 | 320 | 0.06 | 0.02 (−0.16, 0.20) | 0.10 | 0.10 | 48 | 97 | −0.25 | 0.21 (−0.17, 0.59) | 0.10 | 0.10 |
| Malawi | iLiNS−DOSE (31) | KDI | 465 | 147 | −0.06 | 0.15 (−0.01, 0.32) | 0.11 | 0.11 | 181 | 74 | −0.08 | −0.08 (−0.39, 0.23) | 0.15 | 0.15 |
| Mali | PROMIS CS (32) | DMC | | | | | | | | | | | | |
| Zimbabwe | SHINE (HIV−) (33) | MDAT | 532 | 490 | 0.02 | 0.03 (−0.10, 0.16) | 0.18 | 0.18 | 122 | 105 | −0.24 | 0.17 (−0.06, 0.40) | 0.26 | 0.26 |
| Zimbabwe | SHINE (HIV+) (34) | MDAT | 115 | 89 | 0.04 | 0.17 (−0.08, 0.42) | 0.05 | 0.05 | 34 | 32 | −0.25 | 0.15 (−0.29, 0.59) | 0.07 | 0.07 |
| | | | **3081** | **2231** | | **I² = 0.00, Tau² = 0.00** | | | **871** | **576** | | **I² = 0.00, Tau² = 0.00** | | |
| **Fixed** | | | | | | **0.08 (0.02, 0.13)** | | | | | | **0.11 (−0.01, 0.23)** | | |
| **Random** | | | | | | **0.08 (0.02, 0.13)** | | | | | | **0.11 (−0.01, 0.23)** | | |

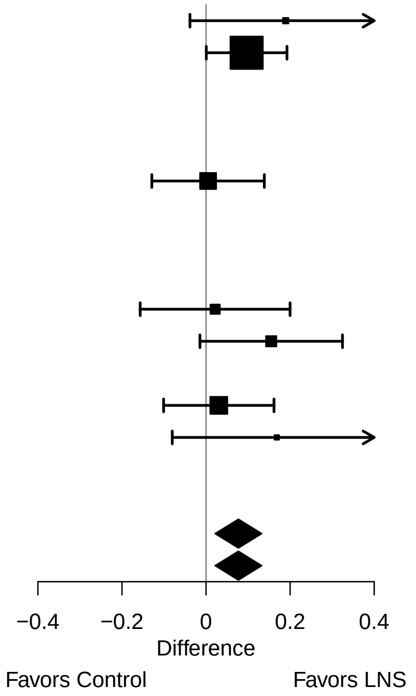


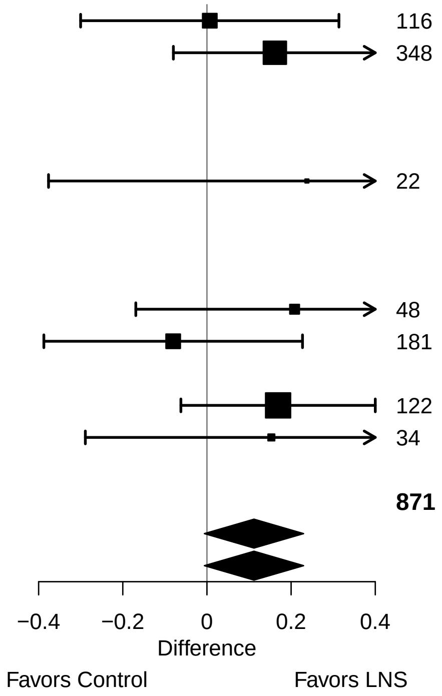

## Supplemental figure 7K: Mean difference in fine motor z-score

## 7K9: Stratified by Child baseline acute malnutrition

**P−for−interaction = 0.992**
**Difference in MDs = 0.00 (−0.18, 0.18)**

| | | | No | | | | | | Yes | | | | | |
|---|---|---|---|---|---|---|---|---|---|---|---|---|---|---|
| **Country** | **Trial** | **Tool** | **LNS N** | **Control N** | **Control Mean** | **MD (95% CI)** | **Fixed W** | **Random W** | **LNS N** | **Control N** | **Control Mean** | **MD (95% CI)** | **Fixed W** | **Random W** |
| Bangladesh | JiVitA−4 (21) | BSID−III | 359 | 112 | −0.04 | 0.12 (−0.09, 0.32) | 0.06 | 0.06 | 84 | 31 | −0.36 | 0.21 (−0.14, 0.56) | 0.31 | 0.31 |
| Bangladesh | RDNS (22) | DMC | 1455 | 697 | −0.03 | 0.10 (0.02, 0.18) | 0.38 | 0.38 | 124 | 53 | −0.48 | 0.33 (−0.18, 0.85) | 0.15 | 0.15 |
| Bangladesh | WASH−B (23) | EASQ | | | | | | | | | | | | |
| Burkina Faso | iLiNS−Zinc (24) | DMC | | | | | | | | | | | | |
| Ghana | GHANA (25) | | | | | | | | | | | | | |
| Ghana | iLiNS−DYAD−G (26) | KDI | 265 | 509 | 0.02 | 0.06 (−0.08, 0.19) | 0.15 | 0.15 | 17 | 52 | −0.14 | −0.44 (−1.15, 0.26) | 0.08 | 0.08 |
| Haiti | HAITI (27) | | | | | | | | | | | | | |
| Kenya | WASH−B (28) | EASQ | | | | | | | | | | | | |
| Madagascar | MAHAY (29) | ASQI | | | | | | | | | | | | |
| Malawi | iLiNS−DYAD−M (30) | KDI | 186 | 383 | 0.00 | 0.06 (−0.11, 0.23) | 0.09 | 0.09 | 14 | 37 | −0.21 | 0.13 (−0.51, 0.76) | 0.10 | 0.10 |
| Malawi | iLiNS−DOSE (31) | KDI | 609 | 206 | −0.04 | 0.09 (−0.06, 0.24) | 0.11 | 0.11 | 37 | 15 | −0.43 | 0.02 (−0.81, 0.86) | 0.06 | 0.06 |
| Mali | PROMIS CS (32) | DMC | | | | | | | | | | | | |
| Zimbabwe | SHINE (HIV−) (33) | MDAT | 620 | 566 | −0.02 | 0.06 (−0.06, 0.18) | 0.17 | 0.17 | 36 | 34 | −0.05 | 0.02 (−0.40, 0.43) | 0.22 | 0.22 |
| Zimbabwe | SHINE (HIV+) (34) | MDAT | 137 | 114 | −0.04 | 0.17 (−0.06, 0.40) | 0.05 | 0.05 | 14 | 8 | 0.02 | 0.26 (−0.39, 0.91) | 0.09 | 0.09 |
| | | | **3631** | **2587** | | **$I^2$ = 0.00, $Tau^2$ = 0.00** | | | **326** | **230** | | **$I^2$ = 0.00, $Tau^2$ = 0.00** | | |
| **Fixed** | | | | | | **0.09 (0.04, 0.14)** | | | | | | **0.12 (−0.07, 0.32)** | | |
| **Random** | | | | | | **0.09 (0.04, 0.14)** | | | | | | **0.12 (−0.07, 0.32)** | | |

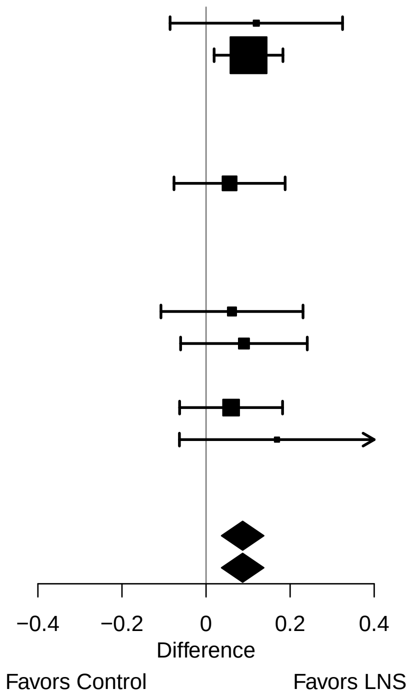


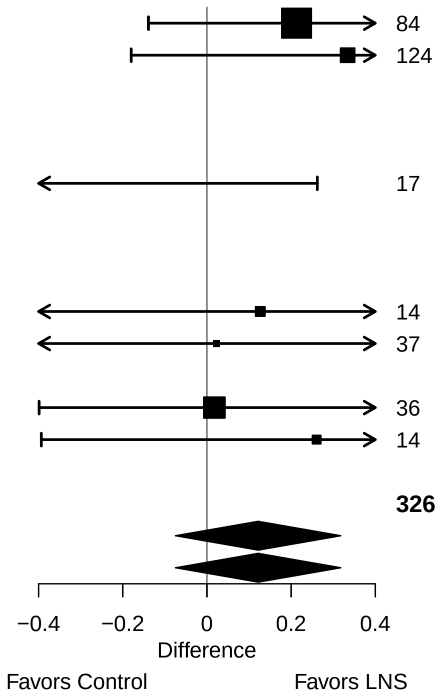

### Supplemental figure 7K: Mean difference in fine motor z-score

## 7K10: Stratified by Child baseline anemia

**P−for−interaction = 0.084**
**Difference in MDs = 0.14 (−0.02, 0.29)**

| | | | Not anemic | | | | | | Anemic | | | | | |
|---|---|---|---|---|---|---|---|---|---|---|---|---|---|---|
| **Country** | **Trial** | **Tool** | **LNS N** | **Control N** | **Control Mean** | **MD (95% CI)** | **Fixed W** | **Random W** | **LNS N** | **Control N** | **Control Mean** | **MD (95% CI)** | **Fixed W** | **Random W** |
| Bangladesh | JiVitA−4 (21) | BSID−III | | | | | | | | | | | | |
| Bangladesh | RDNS (22) | DMC | 206 | 96 | −0.10 | 0.15 (−0.08, 0.39) | 0.23 | 0.23 | 315 | 157 | −0.06 | 0.07 (−0.10, 0.25) | 0.32 | 0.32 |
| Bangladesh | WASH−B (23) | EASQ | | | | | | | | | | | | |
| Burkina Faso | iLiNS−Zinc (24) | DMC | | | | | | | | | | | | |
| Ghana | GHANA (25) | | | | | | | | | | | | | |
| Ghana | iLiNS−DYAD−G (26) | KDI | 180 | 321 | 0.01 | −0.07 (−0.25, 0.11) | 0.38 | 0.38 | 81 | 189 | −0.01 | 0.20 (−0.03, 0.44) | 0.18 | 0.18 |
| Haiti | HAITI (27) | | | | | | | | | | | | | |
| Kenya | WASH−B (28) | EASQ | | | | | | | | | | | | |
| Madagascar | MAHAY (29) | ASQI | | | | | | | | | | | | |
| Malawi | iLiNS−DYAD−M (30) | KDI | 65 | 146 | 0.04 | −0.12 (−0.39, 0.16) | 0.15 | 0.15 | 138 | 273 | −0.03 | 0.14 (−0.05, 0.34) | 0.26 | 0.26 |
| Malawi | iLiNS−DOSE (31) | KDI | 224 | 94 | −0.01 | 0.00 (−0.22, 0.22) | 0.24 | 0.24 | 418 | 126 | −0.11 | 0.15 (−0.05, 0.36) | 0.24 | 0.24 |
| Mali | PROMIS CS (32) | DMC | | | | | | | | | | | | |
| Zimbabwe | SHINE (HIV−) (33) | MDAT | | | | | | | | | | | | |
| Zimbabwe | SHINE (HIV+) (34) | MDAT | | | | | | | | | | | | |
| | | | **675** | **657** | | **$I^2$ = 0.00, Tau² = 0.00** | | | **952** | **745** | | **$I^2$ = 0.00, Tau² = 0.00** | | |
| **Fixed** | | | | | | **−0.01 (−0.12, 0.10)** | | | | | | **0.13 (0.03, 0.24)** | | |
| **Random** | | | | | | **−0.01 (−0.12, 0.10)** | | | | | | **0.13 (0.03, 0.24)** | | |

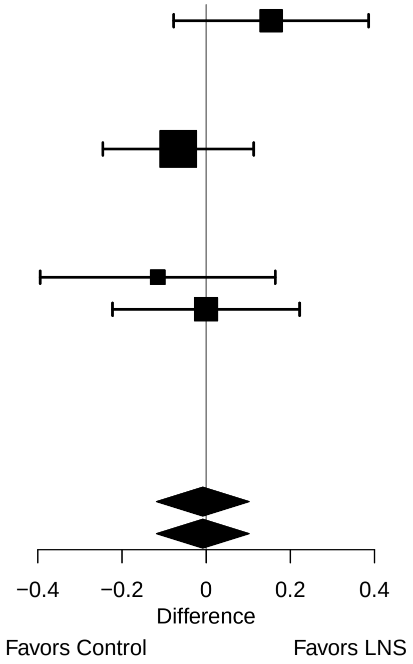


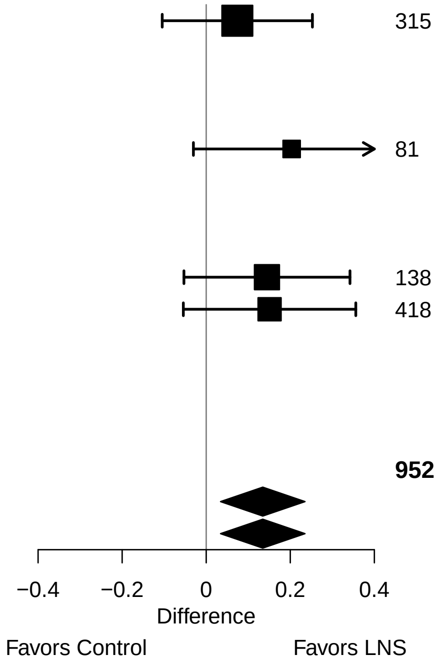

# Supplemental figure 7L: Mean difference in executive function z-score

## 7L1: Stratified by Maternal height

**P−for−interaction = 0.522**
**Difference in MDs = −0.04 (−0.15, 0.08)**

| | | | At least 150.1 cm | | | | | | Less than 150.1 cm | | | | | |
|---|---|---|---|---|---|---|---|---|---|---|---|---|---|---|
| **Country** | **Trial** | **Tool** | **LNS N** | **Control N** | **Control Mean** | **MD (95% CI)** | **Fixed W** | **Random W** | **LNS N** | **Control N** | **Control Mean** | **MD (95% CI)** | **Fixed W** | **Random W** |
| Bangladesh | JiVitA−4 (21) | | | | | | | | | | | | | |
| Bangladesh | RDNS (22) | A not B task | 289 | 136 | −0.06 | 0.07 (−0.08, 0.23) | 0.12 | 0.12 | 222 | 120 | −0.11 | 0.16 (−0.08, 0.40) | 0.14 | 0.14 |
| Bangladesh | WASH−B (23) | A not B task | 585 | 1796 | 0.02 | 0.02 (−0.07, 0.12) | 0.33 | 0.33 | 507 | 1489 | 0.00 | −0.04 (−0.15, 0.06) | 0.74 | 0.74 |
| Burkina Faso | iLiNS−Zinc (24) | | | | | | | | | | | | | |
| Ghana | GHANA (25) | | | | | | | | | | | | | |
| Ghana | iLiNS−DYAD−G (26) | A not B task | 273 | 535 | 0.01 | 0.00 (−0.14, 0.15) | 0.14 | 0.14 | 12 | 31 | −0.19 | 0.25 (−0.44, 0.94) | 0.02 | 0.02 |
| Haiti | HAITI (27) | | | | | | | | | | | | | |
| Kenya | WASH−B (28) | | | | | | | | | | | | | |
| Madagascar | MAHAY (29) | | | | | | | | | | | | | |
| Malawi | iLiNS−DYAD−M (30) | A not B task | 158 | 315 | 0.06 | −0.10 (−0.29, 0.08) | 0.08 | 0.08 | 24 | 52 | −0.16 | 0.02 (−0.51, 0.56) | 0.03 | 0.03 |
| Malawi | iLiNS−DOSE (31) | A not B task | 398 | 142 | 0.07 | −0.07 (−0.26, 0.12) | 0.08 | 0.08 | 76 | 21 | 0.21 | −0.38 (−0.88, 0.11) | 0.03 | 0.03 |
| Mali | PROMIS CS (32) | | | | | | | | | | | | | |
| Zimbabwe | SHINE (HIV−) (33) | A not B task | 737 | 703 | −0.02 | 0.05 (−0.07, 0.17) | 0.19 | 0.19 | 32 | 22 | −0.09 | −0.07 (−0.54, 0.40) | 0.04 | 0.04 |
| Zimbabwe | SHINE (HIV+) (34) | A not B task | 145 | 124 | 0.05 | −0.08 (−0.31, 0.14) | 0.06 | 0.06 | 10 | 5 | −0.11 | 0.29 (−0.81, 1.39) | 0.01 | 0.01 |
| | | | **2585** | **3751** | | **I² = 0.00, Tau² = 0.00** | | | **883** | **1740** | | **I² = 0.00, Tau² = 0.00** | | |
| **Fixed** | | | | | | **0.01 (−0.05, 0.06)** | | | | | | **−0.02 (−0.11, 0.07)** | | |
| **Random** | | | | | | **0.01 (−0.05, 0.06)** | | | | | | **−0.02 (−0.11, 0.07)** | | |

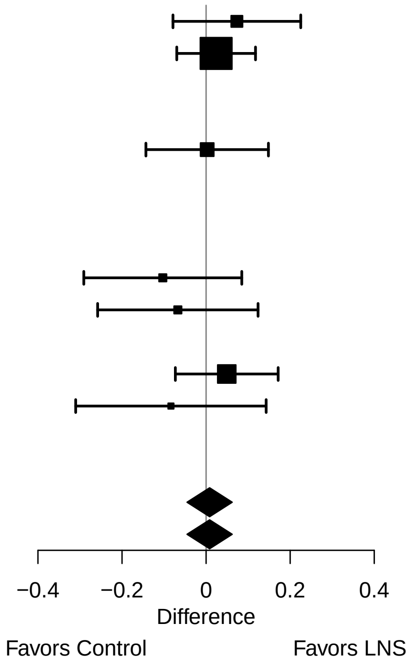


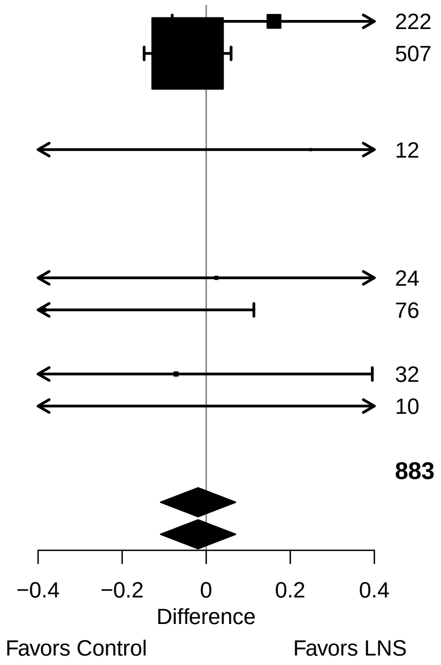

## Supplemental figure 7L: Mean difference in executive function z-score

## 7L2: Stratified by Maternal BMI

**P−for−interaction = 0.888**
**Difference in MDs = 0.01 (−0.09, 0.11)**

| Country | Trial | Tool | At least 20 kg/m²: LNS N | Control N | Control Mean | MD (95% CI) | Fixed W | Random W | Less than 20 kg/m²: LNS N | Control N | Control Mean | MD (95% CI) | Fixed W | Random W |
|---|---|---|---|---|---|---|---|---|---|---|---|---|---|---|
| Bangladesh | JiVitA−4 (21) | | | | | | | | | | | | | |
| Bangladesh | RDNS (22) | A not B task | 226 | 103 | −0.03 | 0.09 (−0.13, 0.32) | 0.07 | 0.07 | 285 | 153 | −0.12 | 0.12 (−0.05, 0.29) | 0.18 | 0.18 |
| Bangladesh | WASH−B (23) | A not B task | 495 | 1517 | 0.01 | 0.02 (−0.08, 0.12) | 0.36 | 0.36 | 597 | 1767 | 0.01 | −0.03 (−0.13, 0.06) | 0.60 | 0.60 |
| Burkina Faso | iLiNS−Zinc (24) | | | | | | | | | | | | | |
| Ghana | GHANA (25) | | | | | | | | | | | | | |
| Ghana | iLiNS−DYAD−G (26) | A not B task | 251 | 464 | 0.01 | −0.01 (−0.16, 0.15) | 0.15 | 0.15 | 34 | 102 | −0.09 | 0.13 (−0.26, 0.53) | 0.03 | 0.03 |
| Haiti | HAITI (27) | | | | | | | | | | | | | |
| Kenya | WASH−B (28) | | | | | | | | | | | | | |
| Madagascar | MAHAY (29) | | | | | | | | | | | | | |
| Malawi | iLiNS−DYAD−M (30) | A not B task | 108 | 221 | 0.06 | −0.11 (−0.34, 0.11) | 0.07 | 0.07 | 74 | 146 | −0.02 | −0.04 (−0.33, 0.25) | 0.06 | 0.06 |
| Malawi | iLiNS−DOSE (31) | A not B task | 360 | 119 | 0.10 | −0.13 (−0.34, 0.08) | 0.08 | 0.08 | 112 | 44 | 0.05 | −0.06 (−0.41, 0.29) | 0.04 | 0.04 |
| Mali | PROMIS CS (32) | | | | | | | | | | | | | |
| Zimbabwe | SHINE (HIV−) (33) | A not B task | 563 | 539 | 0.00 | 0.03 (−0.10, 0.16) | 0.22 | 0.22 | 93 | 87 | −0.14 | 0.19 (−0.09, 0.47) | 0.07 | 0.07 |
| Zimbabwe | SHINE (HIV+) (34) | A not B task | 115 | 99 | 0.06 | 0.01 (−0.24, 0.27) | 0.05 | 0.05 | 22 | 21 | −0.14 | −0.05 (−0.68, 0.58) | 0.01 | 0.01 |
| | | | **2118** | **3062** | | **I² = 0.00, Tau² = 0.00** | | | **1217** | **2320** | | **I² = 0.00, Tau² = 0.00** | | |
| **Fixed** | | | | | | **0.00 (−0.06, 0.06)** | | | | | | **0.01 (−0.06, 0.09)** | | |
| **Random** | | | | | | **0.00 (−0.06, 0.06)** | | | | | | **0.01 (−0.06, 0.09)** | | |

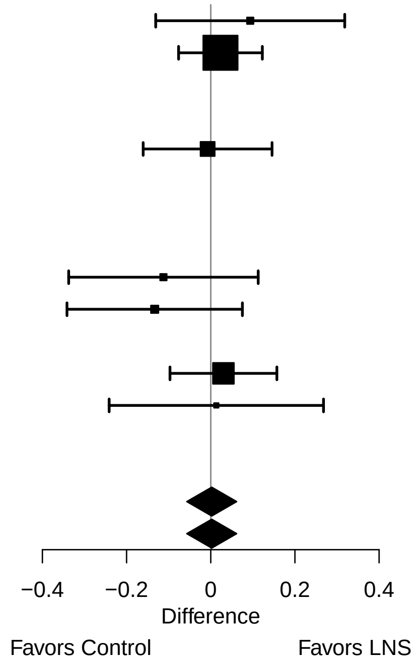


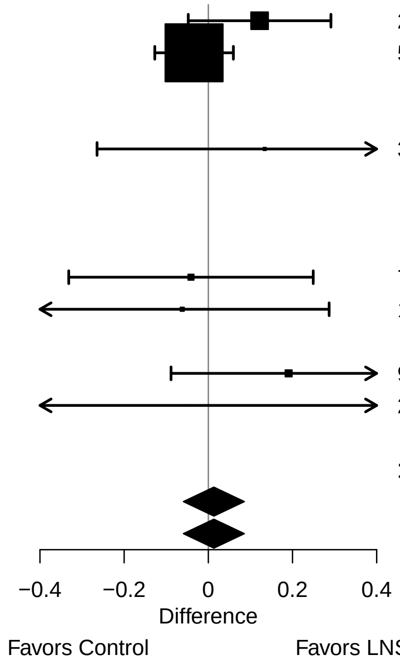

### Supplemental figure 7L: Mean difference in executive function z-score

## 7L3: Stratified by Maternal age

**P−for−interaction = 0.470**
**Difference in MDs = 0.04 (−0.06, 0.13)**

| | | | At least 25 y | | | | | | Less than 25 y | | | | | |
|---|---|---|---|---|---|---|---|---|---|---|---|---|---|---|
| **Country** | **Trial** | **Tool** | **LNS N** | **Control N** | **Control Mean** | **MD (95% CI)** | **Fixed W** | **Random W** | **LNS N** | **Control N** | **Control Mean** | **MD (95% CI)** | **Fixed W** | **Random W** |
| Bangladesh | JiVitA−4 (21) | | | | | | | | | | | | | |
| Bangladesh | RDNS (22) | A not B task | 146 | 80 | −0.16 | 0.19 (−0.09, 0.47) | 0.05 | 0.09 | 387 | 186 | −0.03 | 0.06 (−0.07, 0.19) | 0.25 | 0.25 |
| Bangladesh | WASH−B (23) | A not B task | 471 | 1469 | 0.02 | −0.05 (−0.15, 0.05) | 0.42 | 0.26 | 621 | 1844 | −0.01 | 0.04 (−0.07, 0.14) | 0.41 | 0.41 |
| Burkina Faso | iLiNS−Zinc (24) | | | | | | | | | | | | | |
| Ghana | GHANA (25) | | | | | | | | | | | | | |
| Ghana | iLiNS−DYAD−G (26) | A not B task | 185 | 362 | −0.06 | 0.08 (−0.09, 0.26) | 0.14 | 0.16 | 105 | 214 | 0.08 | −0.08 (−0.31, 0.16) | 0.07 | 0.07 |
| Haiti | HAITI (27) | | | | | | | | | | | | | |
| Kenya | WASH−B (28) | | | | | | | | | | | | | |
| Madagascar | MAHAY (29) | | | | | | | | | | | | | |
| Malawi | iLiNS−DYAD−M (30) | A not B task | 89 | 190 | 0.06 | −0.26 (−0.51, −0.01) | 0.07 | 0.11 | 93 | 179 | −0.01 | 0.08 (−0.18, 0.33) | 0.06 | 0.06 |
| Malawi | iLiNS−DOSE (31) | A not B task | 253 | 86 | 0.04 | −0.14 (−0.40, 0.11) | 0.07 | 0.10 | 212 | 73 | 0.17 | −0.13 (−0.38, 0.13) | 0.06 | 0.06 |
| Mali | PROMIS CS (32) | | | | | | | | | | | | | |
| Zimbabwe | SHINE (HIV−) (33) | A not B task | 399 | 358 | −0.03 | 0.05 (−0.10, 0.20) | 0.19 | 0.19 | 306 | 300 | −0.02 | 0.04 (−0.15, 0.23) | 0.12 | 0.12 |
| Zimbabwe | SHINE (HIV+) (34) | A not B task | 115 | 97 | 0.05 | −0.07 (−0.36, 0.21) | 0.05 | 0.09 | 27 | 23 | 0.21 | −0.03 (−0.44, 0.38) | 0.02 | 0.02 |
| | | | **1658** | **2642** | | **I² = 0.35, Tau² = 0.01** | | | **1751** | **2819** | | **I² = 0.00, Tau² = 0.00** | | |
| **Fixed** | | | | | | **−0.02 (−0.09, 0.04)** | | | | | | **0.03 (−0.04, 0.09)** | | |
| **Random** | | | | | | **−0.02 (−0.12, 0.07)** | | | | | | **0.03 (−0.04, 0.09)** | | |

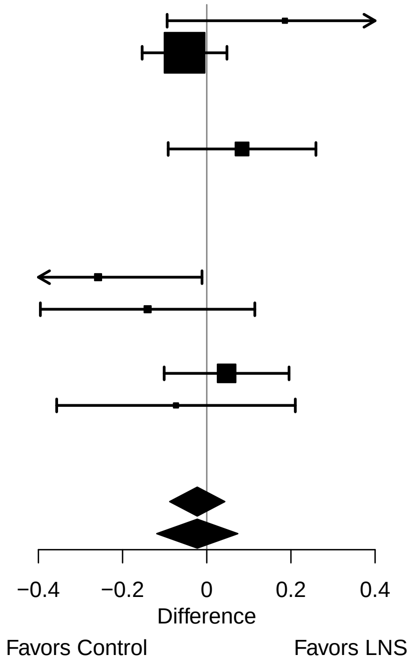


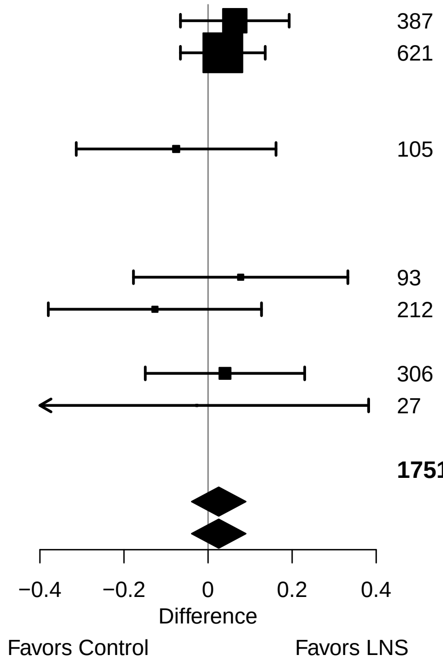

### Supplemental figure 7L: Mean difference in executive function z-score

## 7L4: Stratified by Maternal education

**P−for−interaction = 0.495**
**Difference in MDs = 0.04 (−0.08, 0.16)**

| Country | Trial | Tool | Primary or greater: LNS N | Primary or greater: Control N | Primary or greater: Control Mean | Primary or greater: MD (95% CI) | Primary or greater: Fixed W | Primary or greater: Random W | Incomplete or no formal: LNS N | Incomplete or no formal: Control N | Incomplete or no formal: Control Mean | Incomplete or no formal: MD (95% CI) | Incomplete or no formal: Fixed W | Incomplete or no formal: Random W |
|---|---|---|---|---|---|---|---|---|---|---|---|---|---|---|
| Bangladesh | JiVitA−4 (21) | | | | | | | | | | | | | |
| Bangladesh | RDNS (22) | A not B task | 394 | 191 | −0.01 | 0.08 (−0.08, 0.25) | 0.11 | 0.11 | 139 | 75 | −0.22 | 0.14 (−0.18, 0.45) | 0.08 | 0.08 |
| Bangladesh | WASH−B (23) | A not B task | 776 | 2386 | 0.03 | −0.02 (−0.10, 0.06) | 0.47 | 0.47 | 321 | 941 | −0.07 | 0.04 (−0.10, 0.18) | 0.42 | 0.42 |
| Burkina Faso | iLiNS−Zinc (24) | | | | | | | | | | | | | |
| Ghana | GHANA (25) | | | | | | | | | | | | | |
| Ghana | iLiNS−DYAD−G (26) | A not B task | 223 | 449 | 0.03 | 0.01 (−0.15, 0.17) | 0.12 | 0.12 | 67 | 127 | −0.13 | 0.09 (−0.22, 0.39) | 0.09 | 0.09 |
| Haiti | HAITI (27) | | | | | | | | | | | | | |
| Kenya | WASH−B (28) | | | | | | | | | | | | | |
| Madagascar | MAHAY (29) | | | | | | | | | | | | | |
| Malawi | iLiNS−DYAD−M (30) | A not B task | 26 | 59 | −0.14 | −0.27 (−0.73, 0.18) | 0.01 | 0.01 | 155 | 307 | 0.06 | −0.07 (−0.26, 0.12) | 0.21 | 0.21 |
| Malawi | iLiNS−DOSE (31) | A not B task | 121 | 43 | 0.09 | −0.05 (−0.38, 0.28) | 0.03 | 0.03 | 345 | 116 | 0.10 | −0.17 (−0.38, 0.05) | 0.17 | 0.17 |
| Mali | PROMIS CS (32) | | | | | | | | | | | | | |
| Zimbabwe | SHINE (HIV−) (33) | A not B task | 716 | 679 | −0.02 | 0.06 (−0.06, 0.18) | 0.21 | 0.21 | 27 | 23 | −0.12 | −0.04 (−0.73, 0.65) | 0.02 | 0.02 |
| Zimbabwe | SHINE (HIV+) (34) | A not B task | 137 | 114 | 0.03 | −0.04 (−0.28, 0.21) | 0.05 | 0.05 | 8 | 7 | 0.28 | −0.20 (−1.18, 0.79) | 0.01 | 0.01 |
| | | | **2393** | **3921** | | **I² = 0.00, Tau² = 0.00** | | | **1062** | **1596** | | **I² = 0.00, Tau² = 0.00** | | |
| **Fixed** | | | | | | **0.01 (−0.05, 0.06)** | | | | | | **−0.01 (−0.10, 0.08)** | | |
| **Random** | | | | | | **0.01 (−0.05, 0.06)** | | | | | | **−0.01 (−0.10, 0.08)** | | |

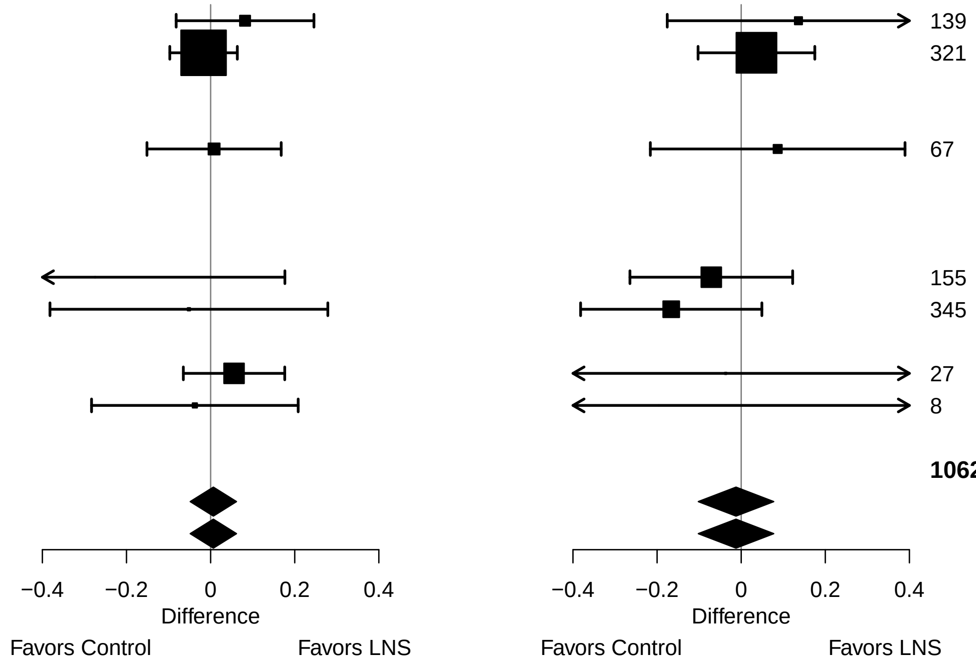

### Supplemental figure 7L: Mean difference in executive function z-score

## 7L5: Stratified by Maternal depressive symptoms

**P−for−interaction = 0.332**
**Difference in MDs = 0.06 (−0.06, 0.17)**

| | | | | | | Less than 75th percentile | | | | | | At least 75th percentile | | |
|---|---|---|---|---|---|---|---|---|---|---|---|---|---|---|
| **Country** | **Trial** | **Tool** | **LNS N** | **Control N** | **Control Mean** | **MD (95% CI)** | **Fixed W** | **Random W** | **LNS N** | **Control N** | **Control Mean** | **MD (95% CI)** | **Fixed W** | **Random W** |
| Bangladesh | JiVitA−4 (21) | | | | | | | | | | | | | |
| Bangladesh | RDNS (22) | A not B task | 345 | 150 | −0.06 | 0.12 (−0.05, 0.28) | 0.11 | 0.11 | 166 | 100 | −0.08 | 0.12 (−0.18, 0.41) | 0.11 | 0.11 |
| Bangladesh | WASH−B (23) | A not B task | 847 | 2392 | 0.01 | 0.01 (−0.07, 0.09) | 0.52 | 0.52 | 230 | 852 | −0.03 | −0.07 (−0.23, 0.09) | 0.38 | 0.38 |
| Burkina Faso | iLiNS−Zinc (24) | | | | | | | | | | | | | |
| Ghana | GHANA (25) | | | | | | | | | | | | | |
| Ghana | iLiNS−DYAD−G (26) | A not B task | 216 | 373 | −0.03 | 0.03 (−0.14, 0.20) | 0.11 | 0.11 | 66 | 185 | 0.09 | 0.01 (−0.26, 0.28) | 0.13 | 0.13 |
| Haiti | HAITI (27) | | | | | | | | | | | | | |
| Kenya | WASH−B (28) | | | | | | | | | | | | | |
| Madagascar | MAHAY (29) | | | | | | | | | | | | | |
| Malawi | iLiNS−DYAD−M (30) | A not B task | 130 | 254 | 0.03 | −0.07 (−0.29, 0.15) | 0.06 | 0.06 | 42 | 93 | 0.00 | −0.07 (−0.42, 0.28) | 0.08 | 0.08 |
| Malawi | iLiNS−DOSE (31) | A not B task | | | | | | | | | | | | |
| Mali | PROMIS CS (32) | | | | | | | | | | | | | |
| Zimbabwe | SHINE (HIV−) (33) | A not B task | 542 | 520 | −0.06 | 0.05 (−0.09, 0.20) | 0.15 | 0.15 | 186 | 164 | 0.07 | 0.01 (−0.18, 0.21) | 0.25 | 0.25 |
| Zimbabwe | SHINE (HIV+) (34) | A not B task | 122 | 96 | 0.05 | −0.03 (−0.29, 0.22) | 0.05 | 0.05 | 29 | 29 | 0.18 | −0.28 (−0.75, 0.18) | 0.04 | 0.04 |
| | | | **2202** | **3785** | | **I² = 0.00, Tau² = 0.00** | | | **719** | **1423** | | **I² = 0.00, Tau² = 0.00** | | |
| **Fixed** | | | | | | **0.02 (−0.03, 0.08)** | | | | | | **−0.03 (−0.12, 0.07)** | | |
| **Random** | | | | | | **0.02 (−0.03, 0.08)** | | | | | | **−0.03 (−0.12, 0.07)** | | |

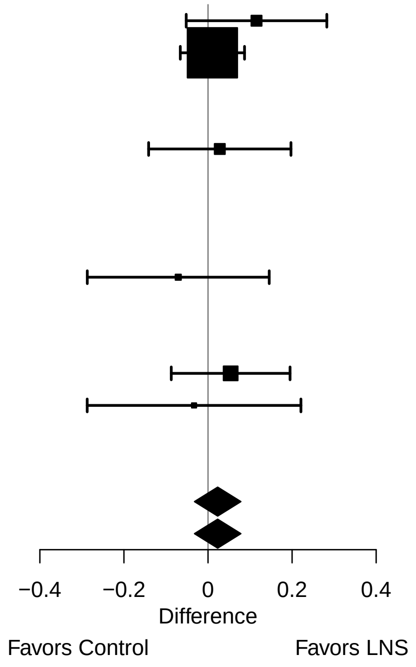


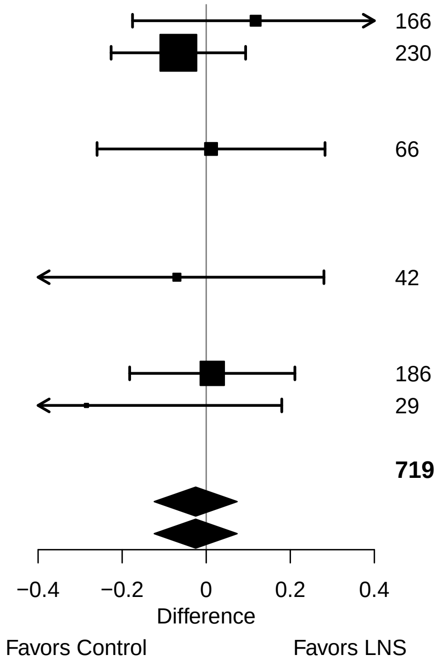

## Supplemental figure 7L: Mean difference in executive function z-score

### 7L6: Stratified by Child sex

**P−for−interaction = 0.414**
**Difference in MDs = −0.04 (−0.12, 0.05)**

| Country | Trial | Tool | LNS N | Control N | Control Mean | Male MD (95% CI) | Fixed W | Random W | LNS N | Control N | Control Mean | Female MD (95% CI) | Fixed W | Random W |
|---|---|---|---|---|---|---|---|---|---|---|---|---|---|---|
| Bangladesh | JiVitA−4 (21) | | | | | | | | | | | | | |
| Bangladesh | RDNS (22) | A not B task | 261 | 133 | 0.05 | −0.08 (−0.26, 0.10) | 0.13 | 0.13 | 272 | 133 | −0.19 | 0.28 (0.09, 0.47) | 0.11 | 0.15 |
| Bangladesh | WASH−B (23) | A not B task | 540 | 1671 | 0.00 | 0.02 (−0.08, 0.12) | 0.44 | 0.41 | 557 | 1656 | 0.01 | −0.02 (−0.12, 0.07) | 0.44 | 0.24 |
| Burkina Faso | iLiNS−Zinc (24) | | | | | | | | | | | | | |
| Ghana | GHANA (25) | | | | | | | | | | | | | |
| Ghana | iLiNS−DYAD−G (26) | A not B task | 145 | 273 | −0.04 | 0.13 (−0.08, 0.34) | 0.10 | 0.10 | 145 | 303 | 0.02 | −0.08 (−0.27, 0.12) | 0.11 | 0.14 |
| Haiti | HAITI (27) | | | | | | | | | | | | | |
| Kenya | WASH−B (28) | | | | | | | | | | | | | |
| Madagascar | MAHAY (29) | | | | | | | | | | | | | |
| Malawi | iLiNS−DYAD−M (30) | A not B task | 92 | 176 | 0.00 | −0.01 (−0.27, 0.24) | 0.06 | 0.07 | 90 | 193 | 0.05 | −0.16 (−0.41, 0.08) | 0.07 | 0.11 |
| Malawi | iLiNS−DOSE (31) | A not B task | 235 | 93 | 0.12 | −0.16 (−0.41, 0.08) | 0.07 | 0.07 | 240 | 71 | 0.04 | −0.05 (−0.31, 0.21) | 0.06 | 0.10 |
| Mali | PROMIS CS (32) | | | | | | | | | | | | | |
| Zimbabwe | SHINE (HIV−) (33) | A not B task | 399 | 364 | −0.06 | 0.11 (−0.04, 0.27) | 0.17 | 0.18 | 388 | 375 | 0.02 | −0.03 (−0.18, 0.11) | 0.18 | 0.18 |
| Zimbabwe | SHINE (HIV+) (34) | A not B task | 74 | 68 | 0.05 | −0.10 (−0.43, 0.23) | 0.04 | 0.04 | 83 | 65 | 0.06 | −0.09 (−0.42, 0.23) | 0.04 | 0.07 |
| | | | **1746** | **2778** | | **I² = 0.05, Tau² = 0.00** | | | **1775** | **2796** | | **I² = 0.46, Tau² = 0.01** | | |
| **Fixed** | | | | | | **0.01 (−0.05, 0.08)** | | | | | | **−0.01 (−0.07, 0.05)** | | |
| **Random** | | | | | | **0.01 (−0.05, 0.08)** | | | | | | **−0.01 (−0.11, 0.09)** | | |

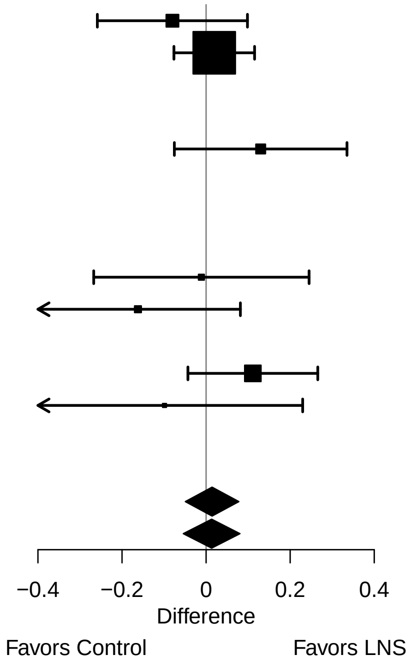


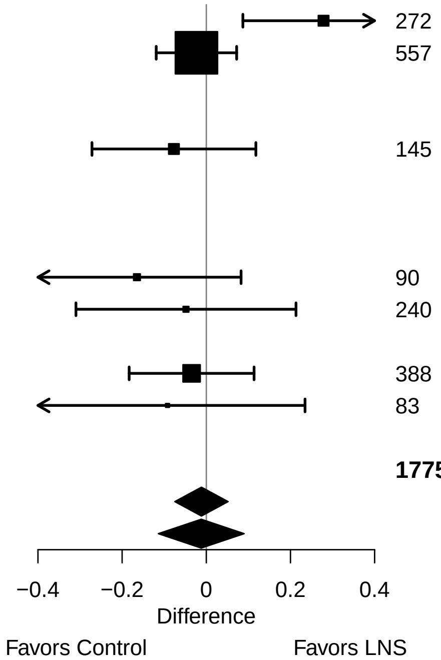

**Supplemental figure 7L: Mean difference in executive function z-score**

## 7L7: Stratified by Child birth order

**P−for−interaction = 0.285**
**Difference in MDs = 0.05 (−0.04, 0.15)**

| | | | Later born | | | | | | Firstborn | | | | | |
|---|---|---|---|---|---|---|---|---|---|---|---|---|---|---|
| **Country** | **Trial** | **Tool** | **LNS N** | **Control N** | **Control Mean** | **MD (95% CI)** | **Fixed W** | **Random W** | **LNS N** | **Control N** | **Control Mean** | **MD (95% CI)** | **Fixed W** | **Random W** |
| Bangladesh | JiVitA−4 (21) | | | | | | | | | | | | | |
| Bangladesh | RDNS (22) | A not B task | 320 | 163 | −0.14 | 0.19 (0.01, 0.38) | 0.09 | 0.11 | 213 | 103 | 0.05 | −0.05 (−0.26, 0.17) | 0.14 | 0.14 |
| Bangladesh | WASH−B (23) | A not B task | 683 | 2174 | 0.02 | −0.05 (−0.13, 0.03) | 0.46 | 0.34 | 394 | 1049 | −0.01 | 0.06 (−0.06, 0.17) | 0.48 | 0.48 |
| Burkina Faso | iLiNS−Zinc (24) | | | | | | | | | | | | | |
| Ghana | GHANA (25) | | | | | | | | | | | | | |
| Ghana | iLiNS−DYAD−G (26) | A not B task | 195 | 390 | −0.02 | 0.03 (−0.15, 0.20) | 0.10 | 0.13 | 95 | 186 | 0.03 | 0.02 (−0.22, 0.27) | 0.11 | 0.11 |
| Haiti | HAITI (27) | | | | | | | | | | | | | |
| Kenya | WASH−B (28) | | | | | | | | | | | | | |
| Madagascar | MAHAY (29) | | | | | | | | | | | | | |
| Malawi | iLiNS−DYAD−M (30) | A not B task | 145 | 297 | 0.05 | −0.09 (−0.29, 0.11) | 0.08 | 0.10 | 37 | 70 | −0.09 | −0.07 (−0.47, 0.33) | 0.04 | 0.04 |
| Malawi | iLiNS−DOSE (31) | A not B task | 313 | 109 | 0.05 | −0.10 (−0.32, 0.13) | 0.06 | 0.08 | 97 | 33 | 0.02 | 0.12 (−0.24, 0.48) | 0.05 | 0.05 |
| Mali | PROMIS CS (32) | | | | | | | | | | | | | |
| Zimbabwe | SHINE (HIV−) (33) | A not B task | 568 | 530 | −0.03 | 0.03 (−0.11, 0.17) | 0.16 | 0.17 | 186 | 178 | −0.02 | 0.09 (−0.11, 0.28) | 0.17 | 0.17 |
| Zimbabwe | SHINE (HIV+) (34) | A not B task | 137 | 106 | 0.02 | −0.10 (−0.34, 0.15) | 0.05 | 0.07 | 17 | 23 | 0.31 | −0.04 (−0.64, 0.56) | 0.02 | 0.02 |
| | | | **2361** | **3769** | | **I² = 0.21, Tau² = 0.00** | | | **1039** | **1642** | | **I² = 0.00, Tau² = 0.00** | | |
| **Fixed** | | | | | | **−0.02 (−0.07, 0.04)** | | | | | | **0.04 (−0.04, 0.12)** | | |
| **Random** | | | | | | **−0.01 (−0.08, 0.06)** | | | | | | **0.04 (−0.04, 0.12)** | | |

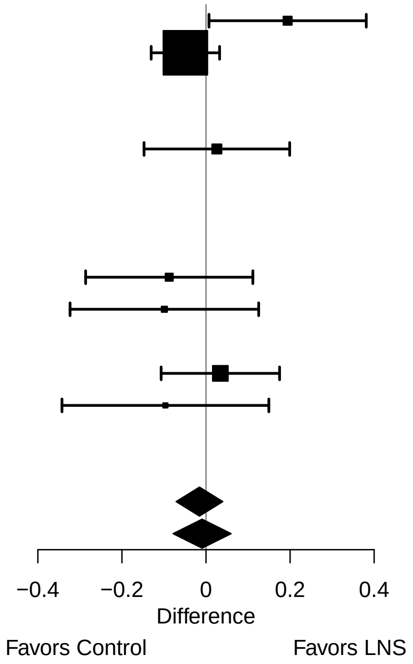


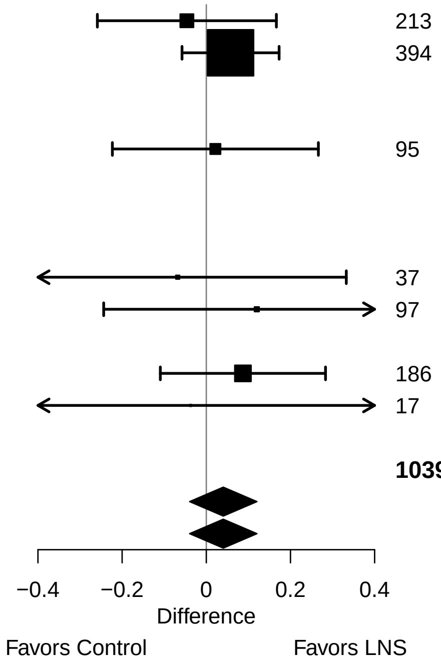

## Supplemental figure 7L: Mean difference in executive function z-score

## 7L8: Stratified by Child baseline stunting

**P−for−interaction = 0.702**
**Difference in MDs = −0.03 (−0.20, 0.13)**

| Country | Trial | Tool | No: LNS N | No: Control N | No: Control Mean | No: MD (95% CI) | No: Fixed W | No: Random W | Yes: LNS N | Yes: Control N | Yes: Control Mean | Yes: MD (95% CI) | Yes: Fixed W | Yes: Random W |
|---|---|---|---|---|---|---|---|---|---|---|---|---|---|---|
| Bangladesh | JiVitA−4 (21) | | | | | | | | | | | | | |
| Bangladesh | RDNS (22) | A not B task | 404 | 187 | −0.02 | 0.07 (−0.08, 0.22) | 0.23 | 0.21 | 107 | 62 | −0.22 | 0.24 (−0.12, 0.59) | 0.17 | 0.18 |
| Bangladesh | WASH−B (23) | A not B task | | | | | | | | | | | | |
| Burkina Faso | iLiNS−Zinc (24) | | | | | | | | | | | | | |
| Ghana | GHANA (25) | | | | | | | | | | | | | |
| Ghana | iLiNS−DYAD−G (26) | A not B task | 248 | 485 | −0.01 | 0.04 (−0.12, 0.19) | 0.21 | 0.20 | 22 | 52 | 0.12 | −0.18 (−0.72, 0.36) | 0.08 | 0.09 |
| Haiti | HAITI (27) | | | | | | | | | | | | | |
| Kenya | WASH−B (28) | | | | | | | | | | | | | |
| Madagascar | MAHAY (29) | | | | | | | | | | | | | |
| Malawi | iLiNS−DYAD−M (30) | A not B task | 129 | 278 | 0.07 | −0.13 (−0.33, 0.08) | 0.12 | 0.14 | 43 | 76 | −0.06 | −0.10 (−0.49, 0.30) | 0.14 | 0.15 |
| Malawi | iLiNS−DOSE (31) | A not B task | 353 | 111 | 0.14 | −0.22 (−0.43, 0.00) | 0.11 | 0.13 | 122 | 53 | −0.05 | 0.14 (−0.17, 0.46) | 0.22 | 0.21 |
| Mali | PROMIS CS (32) | | | | | | | | | | | | | |
| Zimbabwe | SHINE (HIV−) (33) | A not B task | 497 | 459 | −0.05 | 0.08 (−0.05, 0.22) | 0.28 | 0.23 | 116 | 93 | 0.03 | −0.22 (−0.50, 0.05) | 0.29 | 0.26 |
| Zimbabwe | SHINE (HIV+) (34) | A not B task | 109 | 84 | 0.06 | −0.07 (−0.36, 0.22) | 0.06 | 0.08 | 31 | 27 | 0.02 | −0.21 (−0.67, 0.24) | 0.11 | 0.12 |
| | | | **1740** | **1604** | | **I² = 0.39, Tau² = 0.01** | | | **441** | **363** | | **I² = 0.21, Tau² = 0.01** | | |
| **Fixed** | | | | | | **0.01 (−0.07, 0.08)** | | | | | | **−0.04 (−0.19, 0.11)** | | |
| **Random** | | | | | | **−0.01 (−0.11, 0.08)** | | | | | | **−0.04 (−0.21, 0.12)** | | |

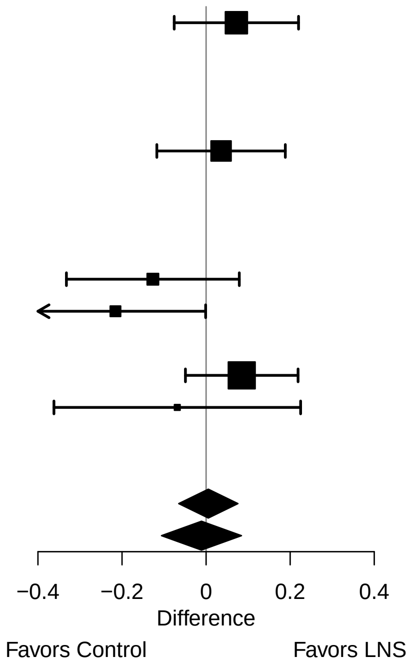


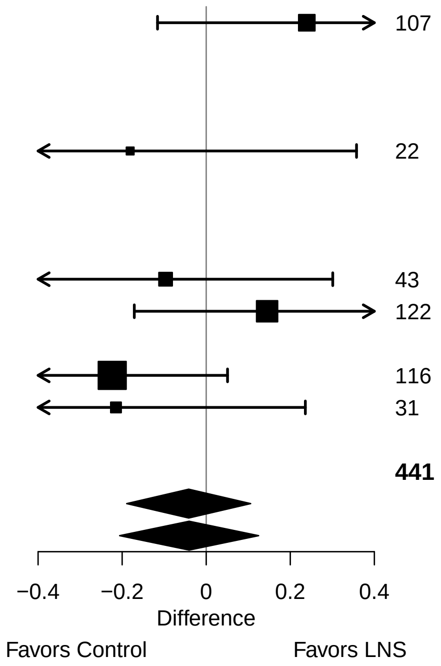

## Supplemental figure 7L: Mean difference in executive function z-score

## 7L9: Stratified by Child baseline acute malnutrition

**P−for−interaction = 0.166**
**Difference in MDs = 0.19 (−0.08, 0.47)**

| | | | No | | | | | | Yes | | | | | |
|---|---|---|---|---|---|---|---|---|---|---|---|---|---|---|
| **Country** | **Trial** | **Tool** | **LNS N** | **Control N** | **Control Mean** | **MD (95% CI)** | **Fixed W** | **Random W** | **LNS N** | **Control N** | **Control Mean** | **MD (95% CI)** | **Fixed W** | **Random W** |
| Bangladesh | JiVitA−4 (21) | | | | | | | | | | | | | |
| Bangladesh | RDNS (22) | A not B task | 475 | 238 | −0.04 | 0.10 (−0.02, 0.23) | 0.26 | 0.22 | 36 | 12 | −0.67 | 0.53 (−0.08, 1.15) | 0.18 | 0.18 |
| Bangladesh | WASH−B (23) | A not B task | | | | | | | | | | | | |
| Burkina Faso | iLiNS−Zinc (24) | | | | | | | | | | | | | |
| Ghana | GHANA (25) | | | | | | | | | | | | | |
| Ghana | iLiNS−DYAD−G (26) | A not B task | 256 | 490 | −0.02 | 0.05 (−0.10, 0.21) | 0.17 | 0.18 | 15 | 47 | 0.18 | −0.41 (−0.97, 0.15) | 0.22 | 0.21 |
| Haiti | HAITI (27) | | | | | | | | | | | | | |
| Kenya | WASH−B (28) | | | | | | | | | | | | | |
| Madagascar | MAHAY (29) | | | | | | | | | | | | | |
| Malawi | iLiNS−DYAD−M (30) | A not B task | 162 | 332 | 0.04 | −0.14 (−0.33, 0.05) | 0.11 | 0.14 | 10 | 25 | 0.06 | 0.23 (−0.50, 0.95) | 0.13 | 0.14 |
| Malawi | iLiNS−DOSE (31) | A not B task | 453 | 156 | 0.09 | −0.12 (−0.30, 0.06) | 0.12 | 0.15 | 22 | 8 | −0.08 | 0.09 (−0.60, 0.78) | 0.14 | 0.16 |
| Mali | PROMIS CS (32) | | | | | | | | | | | | | |
| Zimbabwe | SHINE (HIV−) (33) | A not B task | 581 | 523 | −0.01 | 0.01 (−0.12, 0.13) | 0.26 | 0.22 | 34 | 33 | −0.33 | 0.28 (−0.21, 0.77) | 0.29 | 0.25 |
| Zimbabwe | SHINE (HIV+) (34) | A not B task | 128 | 103 | 0.12 | −0.18 (−0.43, 0.06) | 0.07 | 0.10 | 14 | 8 | −0.83 | 0.86 (−0.44, 2.16) | 0.04 | 0.05 |
| | | | **2055** | **1842** | | **I² = 0.45, Tau² = 0.01** | | | **131** | **133** | | **I² = 0.27, Tau² = 0.04** | | |
| **Fixed** | | | | | | **−0.01 (−0.07, 0.06)** | | | | | | **0.16 (−0.10, 0.43)** | | |
| **Random** | | | | | | **−0.02 (−0.11, 0.07)** | | | | | | **0.17 (−0.14, 0.48)** | | |

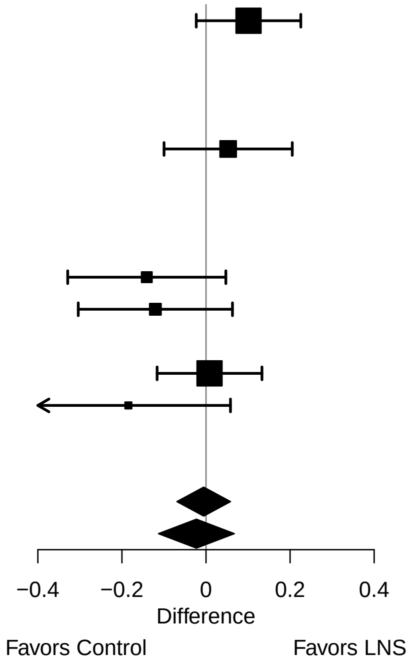


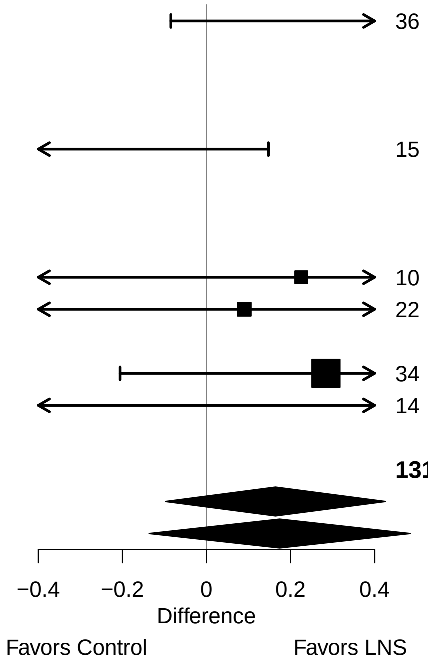

## Supplemental figure 7L: Mean difference in executive function z-score

### 7L10: Stratified by Child baseline anemia

**P−for−interaction = 0.567**
**Difference in MDs = 0.05 (−0.12, 0.22)**

| Country | Trial | Tool | Not anemic LNS N | Not anemic Control N | Not anemic Control Mean | Not anemic MD (95% CI) | Not anemic Fixed W | Not anemic Random W | Anemic LNS N | Anemic Control N | Anemic Control Mean | Anemic MD (95% CI) | Anemic Fixed W | Anemic Random W |
|---|---|---|---|---|---|---|---|---|---|---|---|---|---|---|
| Bangladesh | JiVitA−4 (21) | | | | | | | | | | | | | |
| Bangladesh | RDNS (22) | A not B task | 204 | 93 | −0.05 | 0.09 (−0.09, 0.27) | 0.39 | 0.39 | 306 | 153 | −0.10 | 0.15 (−0.04, 0.34) | 0.33 | 0.31 |
| Bangladesh | WASH−B (23) | A not B task | | | | | | | | | | | | |
| Burkina Faso | iLiNS−Zinc (24) | | | | | | | | | | | | | |
| Ghana | GHANA (25) | | | | | | | | | | | | | |
| Ghana | iLiNS−DYAD−G (26) | A not B task | 172 | 308 | 0.03 | −0.05 (−0.24, 0.14) | 0.33 | 0.33 | 78 | 181 | −0.02 | 0.06 (−0.20, 0.32) | 0.18 | 0.20 |
| Haiti | HAITI (27) | | | | | | | | | | | | | |
| Kenya | WASH−B (28) | | | | | | | | | | | | | |
| Madagascar | MAHAY (29) | | | | | | | | | | | | | |
| Malawi | iLiNS−DYAD−M (30) | A not B task | 55 | 122 | 0.01 | −0.15 (−0.48, 0.18) | 0.11 | 0.11 | 121 | 234 | 0.04 | −0.06 (−0.28, 0.16) | 0.27 | 0.26 |
| Malawi | iLiNS−DOSE (31) | A not B task | 176 | 70 | 0.05 | −0.06 (−0.33, 0.20) | 0.17 | 0.17 | 296 | 93 | 0.10 | −0.14 (−0.38, 0.10) | 0.22 | 0.23 |
| Mali | PROMIS CS (32) | | | | | | | | | | | | | |
| Zimbabwe | SHINE (HIV−) (33) | A not B task | | | | | | | | | | | | |
| Zimbabwe | SHINE (HIV+) (34) | A not B task | | | | | | | | | | | | |
| | | | **607** | **593** | | **I² = 0.00, Tau² = 0.00** | | | **801** | **661** | | **I² = 0.24, Tau² = 0.00** | | |
| **Fixed** | | | | | | **−0.01 (−0.12, 0.10)** | | | | | | **0.02 (−0.10, 0.13)** | | |
| **Random** | | | | | | **−0.01 (−0.12, 0.10)** | | | | | | **0.01 (−0.12, 0.14)** | | |

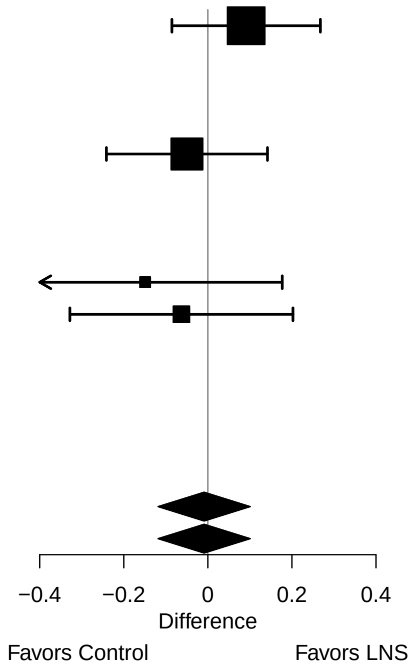


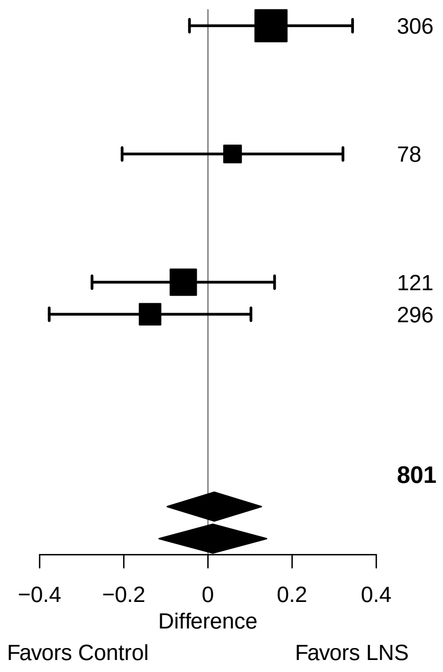

## Supplemental figure 7M: Executive function lowest decile prevalence ratio

### 7M1: Stratified by Maternal height (insufficient comparisons)

## Supplemental figure 7M: Executive function lowest decile prevalence ratio

## 7M2: Stratified by Maternal BMI

**P−for−interaction = 0.977**
**Ratio of PRs = 1.00 (0.72, 1.40)**

| | | | | | | At least 20 $kg/m^2$ | | | | | | Less than 20 $kg/m^2$ | | |
|---|---|---|---|---|---|---|---|---|---|---|---|---|---|---|
| Country | Trial | Tool | LNS N | Control N | Control Prevalence | PR (95% CI) | Fixed W | Random W | LNS N | Control N | Control Prevalence | PR (95% CI) | Fixed W | Random W |
| Bangladesh | JiVitA−4 (21) | | | | | | | | | | | | | |
| Bangladesh | RDNS (22) | A not B task | 226 | 103 | 9.7 | 0.87 (0.43, 1.76) | 0.09 | 0.09 | 285 | 153 | 12.4 | 0.85 (0.51, 1.42) | 0.20 | 0.20 |
| Bangladesh | WASH−B (23) | A not B task | 495 | 1517 | 9.6 | 0.92 (0.67, 1.27) | 0.46 | 0.46 | 597 | 1767 | 10.3 | 0.99 (0.75, 1.31) | 0.67 | 0.67 |
| Burkina Faso | iLiNS−Zinc (24) | | | | | | | | | | | | | |
| Ghana | GHANA (25) | | | | | | | | | | | | | |
| Ghana | iLiNS−DYAD−G (26) | A not B task | | | | | | | | | | | | |
| Haiti | HAITI (27) | | | | | | | | | | | | | |
| Kenya | WASH−B (28) | | | | | | | | | | | | | |
| Madagascar | MAHAY (29) | | | | | | | | | | | | | |
| Malawi | iLiNS−DYAD−M (30) | A not B task | 108 | 221 | 9.5 | 0.97 (0.48, 2.00) | 0.09 | 0.09 | 74 | 146 | 13.0 | 0.62 (0.26, 1.49) | 0.07 | 0.07 |
| Malawi | iLiNS−DOSE (31) | A not B task | | | | | | | | | | | | |
| Mali | PROMIS CS (32) | | | | | | | | | | | | | |
| Zimbabwe | SHINE (HIV−) (33) | A not B task | 563 | 539 | 11.9 | 0.76 (0.53, 1.09) | 0.36 | 0.36 | 93 | 87 | 11.5 | 0.75 (0.31, 1.80) | 0.07 | 0.07 |
| Zimbabwe | SHINE (HIV+) (34) | A not B task | | | | | | | | | | | | |
| | | | **1392** | **2380** | | **$I^2$ = 0.00, $Tau^2$ = 0.00** | | | **1049** | **2153** | | **$I^2$ = 0.00, $Tau^2$ = 0.00** | | |
| **Fixed** | | | | | | **0.86 (0.69, 1.07)** | | | | | | **0.91 (0.73, 1.15)** | | |
| **Random** | | | | | | **0.86 (0.69, 1.07)** | | | | | | **0.91 (0.73, 1.15)** | | |

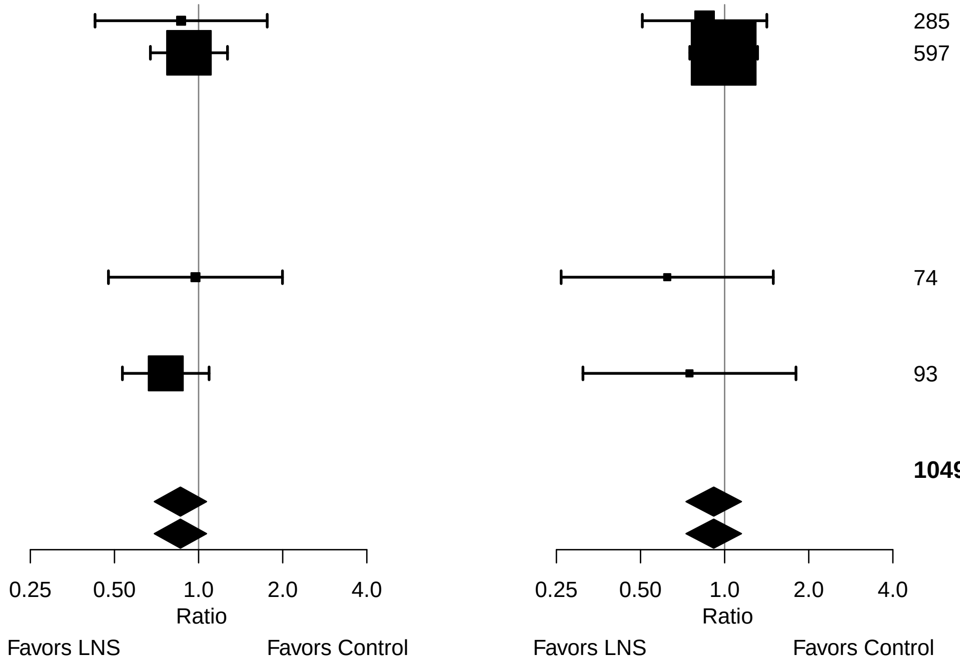

## Supplemental figure 7M: Executive function lowest decile prevalence ratio

# 7M3: Stratified by Maternal age

**P−for−interaction = 0.088**
**Ratio of PRs = 0.76 (0.55, 1.04)**

| | | | At least 25 y | | | | | | Less than 25 y | | | | | |
|---|---|---|---|---|---|---|---|---|---|---|---|---|---|---|
| **Country** | **Trial** | **Tool** | **LNS N** | **Control N** | **Control Prevalence** | **PR (95% CI)** | **Fixed W** | **Random W** | **LNS N** | **Control N** | **Control Prevalence** | **PR (95% CI)** | **Fixed W** | **Random W** |
| Bangladesh | JiVitA−4 (21) | | | | | | | | | | | | | |
| Bangladesh | RDNS (22) | A not B task | 146 | 80 | 13.8 | 0.75 (0.33, 1.68) | 0.06 | 0.09 | 387 | 186 | 9.7 | 0.96 (0.59, 1.57) | 0.21 | 0.21 |
| Bangladesh | WASH−B (23) | A not B task | 471 | 1469 | 9.7 | 1.21 (0.91, 1.61) | 0.50 | 0.39 | 621 | 1844 | 10.4 | 0.79 (0.56, 1.11) | 0.45 | 0.45 |
| Burkina Faso | iLiNS−Zinc (24) | | | | | | | | | | | | | |
| Ghana | GHANA (25) | | | | | | | | | | | | | |
| Ghana | iLiNS−DYAD−G (26) | A not B task | 185 | 362 | 12.2 | 0.76 (0.44, 1.29) | 0.14 | 0.18 | 105 | 214 | 9.3 | 0.82 (0.37, 1.79) | 0.08 | 0.08 |
| Haiti | HAITI (27) | | | | | | | | | | | | | |
| Kenya | WASH−B (28) | | | | | | | | | | | | | |
| Madagascar | MAHAY (29) | | | | | | | | | | | | | |
| Malawi | iLiNS−DYAD−M (30) | A not B task | 89 | 190 | 7.9 | 1.57 (0.75, 3.27) | 0.07 | 0.10 | 93 | 179 | 14.0 | 0.38 (0.15, 0.97) | 0.06 | 0.06 |
| Malawi | iLiNS−DOSE (31) | A not B task | | | | | | | | | | | | |
| Mali | PROMIS CS (32) | | | | | | | | | | | | | |
| Zimbabwe | SHINE (HIV−) (33) | A not B task | 399 | 358 | 11.7 | 0.81 (0.53, 1.25) | 0.22 | 0.24 | 306 | 300 | 11.3 | 0.81 (0.48, 1.35) | 0.20 | 0.20 |
| Zimbabwe | SHINE (HIV+) (34) | A not B task | | | | | | | | | | | | |
| | | | **1290** | **2459** | | **I² = 0.28, Tau² = 0.02** | | | **1512** | **2723** | | **I² = 0.00, Tau² = 0.00** | | |
| **Fixed** | | | | | | **1.02 (0.84, 1.25)** | | | | | | **0.79 (0.63, 1.00)** | | |
| **Random** | | | | | | **1.00 (0.77, 1.28)** | | | | | | **0.79 (0.63, 1.00)** | | |

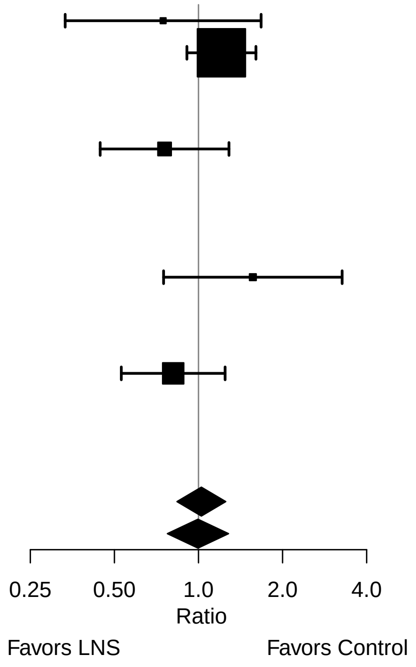


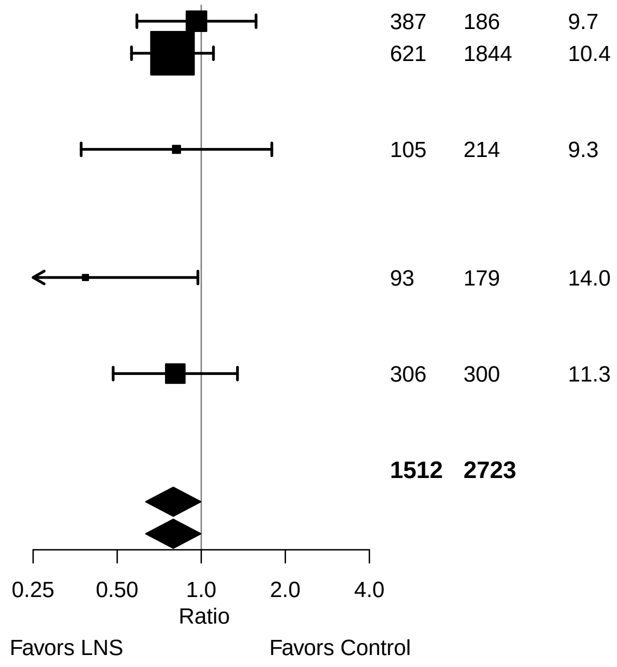

## Supplemental figure 7M: Executive function lowest decile prevalence ratio

## 7M4: Stratified by Maternal education

**P−for−interaction = 0.865**
**Ratio of PRs = 1.03 (0.73, 1.46)**

| Country | Trial | Tool | LNS N | Control N | Control Prevalence | Primary or greater PR (95% CI) | Fixed W | Random W | LNS N | Control N | Control Prevalence | Incomplete or no formal PR (95% CI) | Fixed W | Random W |
|---|---|---|---|---|---|---|---|---|---|---|---|---|---|---|
| Bangladesh | JiVitA−4 (21) | | | | | | | | | | | | | |
| Bangladesh | RDNS (22) | A not B task | 394 | 191 | 9.4 | 0.78 (0.44, 1.38) | 0.15 | 0.15 | 139 | 75 | 14.7 | 1.08 (0.61, 1.91) | 0.20 | 0.20 |
| Bangladesh | WASH−B (23) | A not B task | 776 | 2386 | 9.3 | 0.91 (0.69, 1.20) | 0.64 | 0.64 | 321 | 941 | 12.1 | 1.03 (0.73, 1.46) | 0.54 | 0.54 |
| Burkina Faso | iLiNS−Zinc (24) | | | | | | | | | | | | | |
| Ghana | GHANA (25) | | | | | | | | | | | | | |
| Ghana | iLiNS−DYAD−G (26) | A not B task | 223 | 449 | 10.0 | 0.81 (0.48, 1.36) | 0.18 | 0.18 | 67 | 127 | 15.0 | 0.70 (0.31, 1.58) | 0.10 | 0.10 |
| Haiti | HAITI (27) | | | | | | | | | | | | | |
| Kenya | WASH−B (28) | | | | | | | | | | | | | |
| Madagascar | MAHAY (29) | | | | | | | | | | | | | |
| Malawi | iLiNS−DYAD−M (30) | A not B task | 26 | 59 | 10.2 | 1.89 (0.63, 5.64) | 0.04 | 0.04 | 155 | 307 | 11.1 | 0.64 (0.33, 1.23) | 0.16 | 0.16 |
| Malawi | iLiNS−DOSE (31) | A not B task | | | | | | | | | | | | |
| Mali | PROMIS CS (32) | | | | | | | | | | | | | |
| Zimbabwe | SHINE (HIV−) (33) | A not B task | | | | | | | | | | | | |
| Zimbabwe | SHINE (HIV+) (34) | A not B task | | | | | | | | | | | | |
| | | | **1419** | **3085** | | **I² = 0.00, Tau² = 0.00** | | | **682** | **1450** | | **I² = 0.00, Tau² = 0.00** | | |
| **Fixed** | | | | | | **0.90 (0.72, 1.12)** | | | | | | **0.93 (0.72, 1.20)** | | |
| **Random** | | | | | | **0.90 (0.72, 1.12)** | | | | | | **0.93 (0.72, 1.20)** | | |

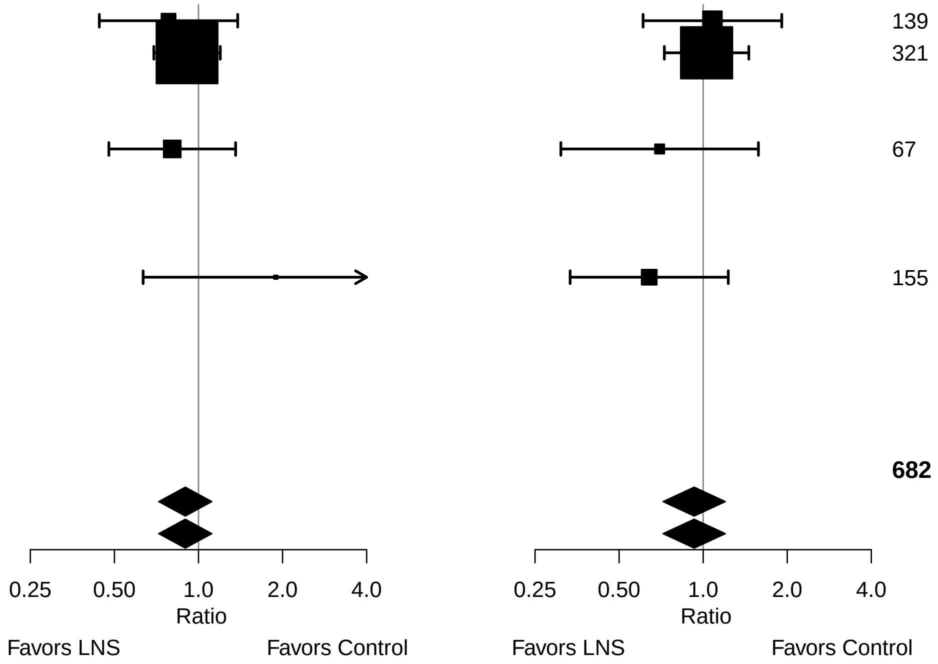

## Supplemental figure 7M: Executive function lowest decile prevalence ratio

### 7M5: Stratified by Maternal depressive symptoms

**P−for−interaction = 0.394**
**Ratio of PRs = 1.18 (0.80, 1.75)**

| | | | Less than 75th percentile | | | | | | At least 75th percentile | | | | | |
|---|---|---|---|---|---|---|---|---|---|---|---|---|---|---|
| **Country** | **Trial** | **Tool** | **LNS N** | **Control N** | **Control Prevalence** | **PR (95% CI)** | **Fixed W** | **Random W** | **LNS N** | **Control N** | **Control Prevalence** | **PR (95% CI)** | **Fixed W** | **Random W** |
| Bangladesh | JiVitA−4 (21) | | | | | | | | | | | | | |
| Bangladesh | RDNS (22) | A not B task | 345 | 150 | 10.0 | 0.93 (0.56, 1.55) | 0.14 | 0.14 | 166 | 100 | 12.0 | 0.80 (0.34, 1.89) | 0.15 | 0.17 |
| Bangladesh | WASH−B (23) | A not B task | 847 | 2392 | 9.4 | 0.97 (0.75, 1.26) | 0.56 | 0.56 | 230 | 852 | 12.1 | 0.97 (0.64, 1.48) | 0.62 | 0.57 |
| Burkina Faso | iLiNS−Zinc (24) | | | | | | | | | | | | | |
| Ghana | GHANA (25) | | | | | | | | | | | | | |
| Ghana | iLiNS−DYAD−G (26) | A not B task | | | | | | | | | | | | |
| Haiti | HAITI (27) | | | | | | | | | | | | | |
| Kenya | WASH−B (28) | | | | | | | | | | | | | |
| Madagascar | MAHAY (29) | | | | | | | | | | | | | |
| Malawi | iLiNS−DYAD−M (30) | A not B task | | | | | | | | | | | | |
| Malawi | iLiNS−DOSE (31) | A not B task | | | | | | | | | | | | |
| Mali | PROMIS CS (32) | | | | | | | | | | | | | |
| Zimbabwe | SHINE (HIV−) (33) | A not B task | 542 | 520 | 11.5 | 0.90 (0.63, 1.28) | 0.29 | 0.29 | 186 | 164 | 11.6 | 0.51 (0.26, 1.02) | 0.23 | 0.26 |
| Zimbabwe | SHINE (HIV+) (34) | A not B task | | | | | | | | | | | | |
| | | | **1734** | **3062** | | **I² = 0.00, Tau² = 0.00** | | | **582** | **1116** | | **I² = 0.18, Tau² = 0.02** | | |
| **Fixed** | | | | | | **0.94 (0.78, 1.15)** | | | | | | **0.81 (0.58, 1.13)** | | |
| **Random** | | | | | | **0.94 (0.78, 1.15)** | | | | | | **0.79 (0.55, 1.16)** | | |

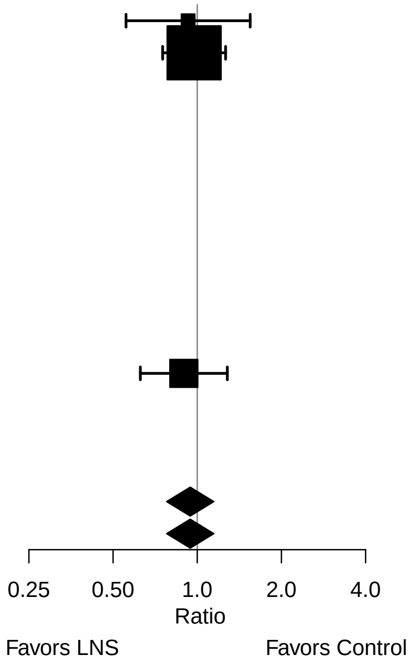


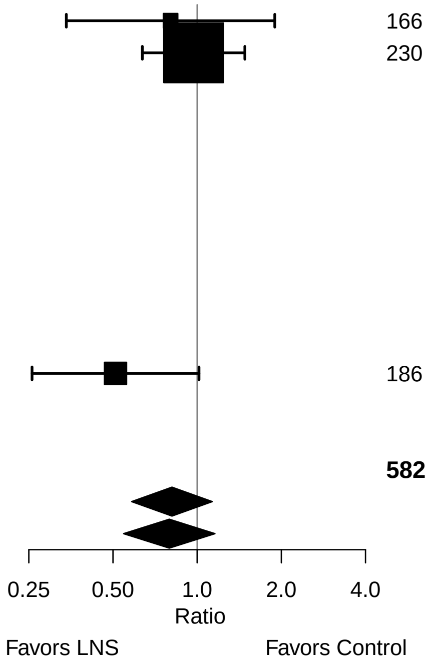

## Supplemental figure 7M: Executive function lowest decile prevalence ratio

## 7M6: Stratified by Child sex

**P−for−interaction = 0.231**
**Ratio of PRs = 1.19 (0.90, 1.57)**

| Country | Trial | Tool | LNS N | Control N | Control Prevalence | Male PR (95% CI) | Fixed W | Random W | LNS N | Control N | Control Prevalence | Female PR (95% CI) | Fixed W | Random W |
|---|---|---|---|---|---|---|---|---|---|---|---|---|---|---|
| Bangladesh | JiVitA−4 (21) | | | | | | | | | | | | | |
| Bangladesh | RDNS (22) | A not B task | 261 | 133 | 6.8 | 1.42 (0.68, 2.94) | 0.09 | 0.14 | 272 | 133 | 15.0 | 0.64 (0.41, 0.98) | 0.16 | 0.16 |
| Bangladesh | WASH−B (23) | A not B task | 540 | 1671 | 9.8 | 0.87 (0.64, 1.20) | 0.51 | 0.31 | 557 | 1656 | 10.5 | 1.03 (0.78, 1.34) | 0.42 | 0.42 |
| Burkina Faso | iLiNS−Zinc (24) | | | | | | | | | | | | | |
| Ghana | GHANA (25) | | | | | | | | | | | | | |
| Ghana | iLiNS−DYAD−G (26) | A not B task | 145 | 273 | 11.0 | 0.63 (0.32, 1.25) | 0.11 | 0.15 | 145 | 303 | 11.2 | 0.92 (0.52, 1.64) | 0.09 | 0.09 |
| Haiti | HAITI (27) | | | | | | | | | | | | | |
| Kenya | WASH−B (28) | | | | | | | | | | | | | |
| Madagascar | MAHAY (29) | | | | | | | | | | | | | |
| Malawi | iLiNS−DYAD−M (30) | A not B task | 92 | 176 | 12.5 | 0.61 (0.27, 1.37) | 0.08 | 0.12 | 90 | 193 | 9.3 | 1.07 (0.50, 2.29) | 0.05 | 0.05 |
| Malawi | iLiNS−DOSE (31) | A not B task | 235 | 93 | 7.5 | 1.41 (0.63, 3.15) | 0.08 | 0.12 | 240 | 71 | 7.0 | 1.60 (0.64, 4.00) | 0.04 | 0.04 |
| Mali | PROMIS CS (32) | | | | | | | | | | | | | |
| Zimbabwe | SHINE (HIV−) (33) | A not B task | 399 | 364 | 8.8 | 0.51 (0.28, 0.94) | 0.14 | 0.17 | 388 | 375 | 14.1 | 0.98 (0.68, 1.42) | 0.23 | 0.23 |
| Zimbabwe | SHINE (HIV+) (34) | A not B task | | | | | | | | | | | | | |
| | | | **1672** | **2710** | | **I² = 0.33, Tau² = 0.06** | | | **1692** | **2731** | | **I² = 0.00, Tau² = 0.00** | | |
| **Fixed** | | | | | | **0.83 (0.66, 1.04)** | | | | | | **0.95 (0.80, 1.13)** | | |
| **Random** | | | | | | **0.82 (0.60, 1.14)** | | | | | | **0.95 (0.80, 1.13)** | | |

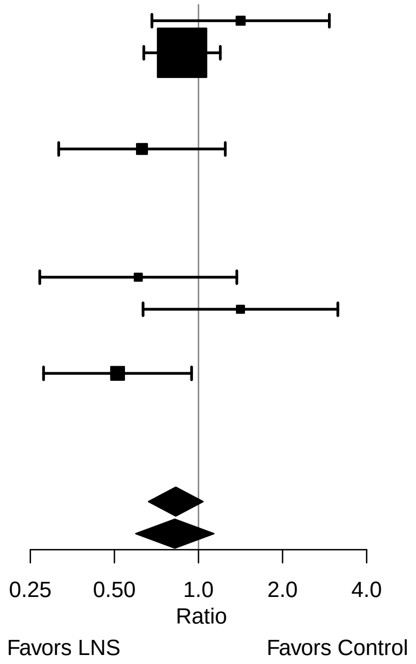


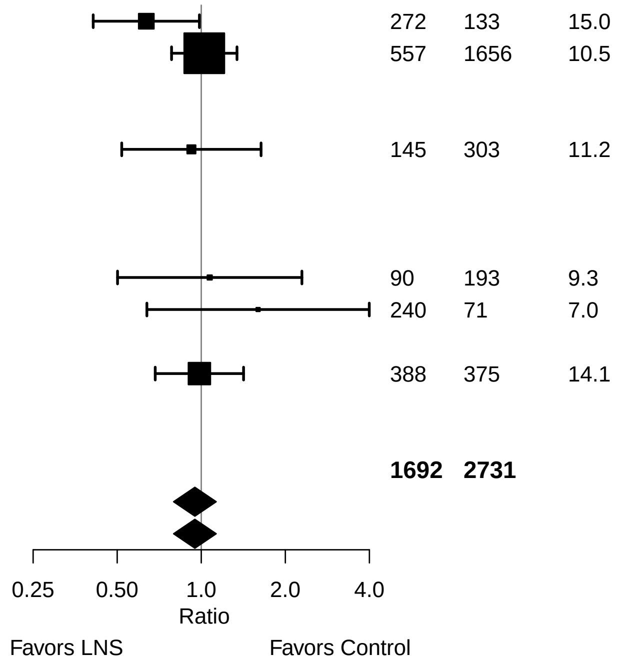

## Supplemental figure 7M: Executive function lowest decile prevalence ratio

### 7M7: Stratified by Child birth order

**P−for−interaction = 0.144**
**Ratio of PRs = 0.78 (0.56, 1.09)**

| Country | Trial | Tool | Later born LNS N | Later born Control N | Later born Control Prevalence | Later born PR (95% CI) | Later born Fixed W | Later born Random W | Firstborn LNS N | Firstborn Control N | Firstborn Control Prevalence | Firstborn PR (95% CI) | Firstborn Fixed W | Firstborn Random W |
|---|---|---|---|---|---|---|---|---|---|---|---|---|---|---|
| Bangladesh | JiVitA−4 (21) | | | | | | | | | | | | | |
| Bangladesh | RDNS (22) | A not B task | 320 | 163 | 12.9 | 0.68 (0.41, 1.12) | 0.12 | 0.15 | 213 | 103 | 7.8 | 1.39 (0.64, 3.00) | 0.15 | 0.16 |
| Bangladesh | WASH−B (23) | A not B task | 683 | 2174 | 9.5 | 1.14 (0.89, 1.45) | 0.51 | 0.43 | 394 | 1049 | 10.3 | 0.71 (0.47, 1.10) | 0.49 | 0.46 |
| Burkina Faso | iLiNS−Zinc (24) | | | | | | | | | | | | | |
| Ghana | GHANA (25) | | | | | | | | | | | | | |
| Ghana | iLiNS−DYAD−G (26) | A not B task | 195 | 390 | 10.8 | 0.90 (0.54, 1.51) | 0.11 | 0.14 | 95 | 186 | 11.8 | 0.53 (0.22, 1.27) | 0.12 | 0.13 |
| Haiti | HAITI (27) | | | | | | | | | | | | | |
| Kenya | WASH−B (28) | | | | | | | | | | | | | |
| Madagascar | MAHAY (29) | | | | | | | | | | | | | |
| Malawi | iLiNS−DYAD−M (30) | A not B task | | | | | | | | | | | | |
| Malawi | iLiNS−DOSE (31) | A not B task | | | | | | | | | | | | |
| Mali | PROMIS CS (32) | | | | | | | | | | | | | |
| Zimbabwe | SHINE (HIV−) (33) | A not B task | 568 | 530 | 11.7 | 0.84 (0.60, 1.19) | 0.26 | 0.28 | 186 | 178 | 11.8 | 0.68 (0.37, 1.25) | 0.24 | 0.25 |
| Zimbabwe | SHINE (HIV+) (34) | A not B task | | | | | | | | | | | | |
| | | | **1766** | **3257** | | **I² = 0.32, Tau² = 0.01** | | | **888** | **1516** | | **I² = 0.06, Tau² = 0.01** | | |
| **Fixed** | | | | | | **0.97 (0.81, 1.15)** | | | | | | **0.75 (0.56, 1.02)** | | |
| **Random** | | | | | | **0.94 (0.76, 1.16)** | | | | | | **0.76 (0.55, 1.04)** | | |

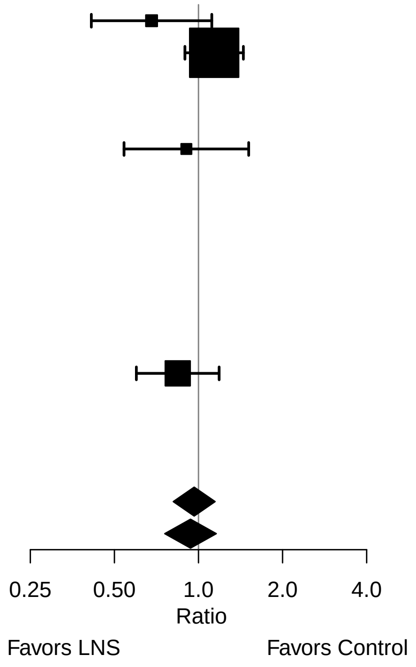


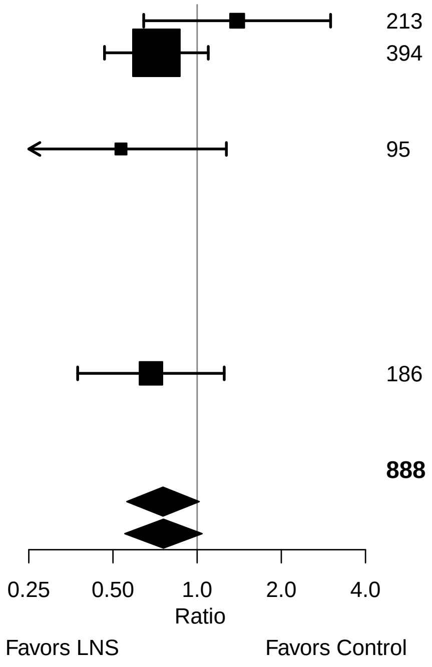

## Supplemental figure 7M: Executive function lowest decile prevalence ratio

## 7M8: Stratified by Child baseline stunting

**P−for−interaction = 0.507**
**Ratio of PRs = 1.23 (0.67, 2.28)**

| Country | Trial | Tool | LNS N | Control N | Control Prevalence | No PR (95% CI) | Fixed W | Random W | LNS N | Control N | Control Prevalence | Yes PR (95% CI) | Fixed W | Random W |
|---|---|---|---|---|---|---|---|---|---|---|---|---|---|---|
| Bangladesh | JiVitA−4 (21) | | | | | | | | | | | | | |
| Bangladesh | RDNS (22) | A not B task | 404 | 187 | 10.7 | 0.83 (0.52, 1.34) | 0.36 | 0.36 | 107 | 62 | 11.3 | 0.99 (0.34, 2.90) | 0.24 | 0.24 |
| Bangladesh | WASH−B (23) | A not B task | | | | | | | | | | | | |
| Burkina Faso | iLiNS−Zinc (24) | | | | | | | | | | | | | |
| Ghana | GHANA (25) | | | | | | | | | | | | | |
| Ghana | iLiNS−DYAD−G (26) | A not B task | | | | | | | | | | | | |
| Haiti | HAITI (27) | | | | | | | | | | | | | |
| Kenya | WASH−B (28) | | | | | | | | | | | | | |
| Madagascar | MAHAY (29) | | | | | | | | | | | | | |
| Malawi | iLiNS−DYAD−M (30) | A not B task | | | | | | | | | | | | |
| Malawi | iLiNS−DOSE (31) | A not B task | 353 | 111 | 5.4 | 2.10 (0.91, 4.81) | 0.12 | 0.25 | 122 | 53 | 11.3 | 0.87 (0.34, 2.19) | 0.33 | 0.33 |
| Mali | PROMIS CS (32) | | | | | | | | | | | | | |
| Zimbabwe | SHINE (HIV−) (33) | A not B task | 497 | 459 | 11.5 | 0.71 (0.48, 1.06) | 0.53 | 0.39 | 116 | 93 | 9.7 | 1.51 (0.68, 3.38) | 0.43 | 0.43 |
| Zimbabwe | SHINE (HIV+) (34) | A not B task | | | | | | | | | | | | |
| | | | **1254** | **757** | | **I² = 0.62, Tau² = 0.21** | | | **345** | **208** | | **I² = 0.00, Tau² = 0.00** | | |
| **Fixed** | | | | | | **0.86 (0.64, 1.13)** | | | | | | **1.14 (0.67, 1.93)** | | |
| **Random** | | | | | | **0.99 (0.54, 1.81)** | | | | | | **1.14 (0.67, 1.93)** | | |

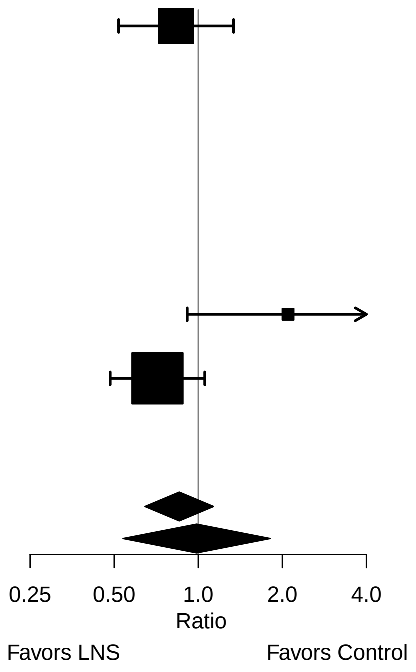


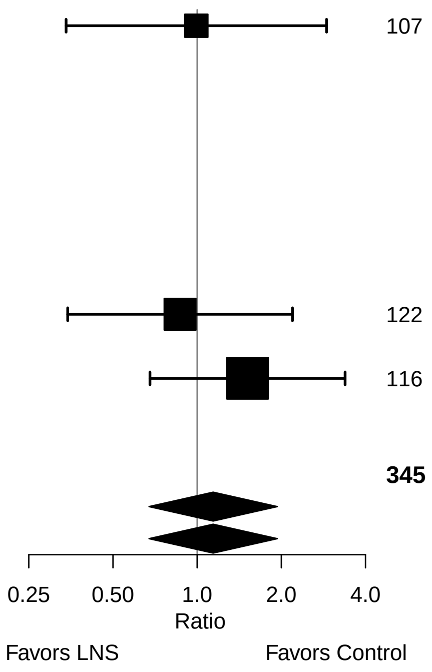

**Supplemental figure 7M: Executive function lowest decile prevalence ratio**

## 7M9: Stratified by Child baseline acute malnutrition (insufficient comparisons)

## Supplemental figure 7M: Executive function lowest decile prevalence ratio

### 7M10: Stratified by Child baseline anemia

**P−for−interaction = 0.464**
**Ratio of PRs = 0.81 (0.47, 1.41)**

| Country | Trial | Tool | LNS N | Control N | Control Prevalence | Not anemic PR (95% CI) | Fixed W | Random W | LNS N | Control N | Control Prevalence | Anemic PR (95% CI) | Fixed W | Random W |
|---|---|---|---|---|---|---|---|---|---|---|---|---|---|---|
| Bangladesh | JiVitA−4 (21) | | | | | | | | | | | | | |
| Bangladesh | RDNS (22) | A not B task | 204 | 93 | 7.5 | 1.24 (0.65, 2.37) | 0.29 | 0.29 | 306 | 153 | 13.1 | 0.73 (0.40, 1.32) | 0.37 | 0.32 |
| Bangladesh | WASH−B (23) | A not B task | | | | | | | | | | | | |
| Burkina Faso | iLiNS−Zinc (24) | | | | | | | | | | | | | |
| Ghana | GHANA (25) | | | | | | | | | | | | | |
| Ghana | iLiNS−DYAD−G (26) | A not B task | 172 | 308 | 10.1 | 1.10 (0.64, 1.88) | 0.42 | 0.42 | 78 | 181 | 12.7 | 0.50 (0.20, 1.28) | 0.15 | 0.19 |
| Haiti | HAITI (27) | | | | | | | | | | | | | |
| Kenya | WASH−B (28) | | | | | | | | | | | | | |
| Madagascar | MAHAY (29) | | | | | | | | | | | | | |
| Malawi | iLiNS−DYAD−M (30) | A not B task | 55 | 122 | 13.1 | 0.69 (0.27, 1.80) | 0.14 | 0.14 | 121 | 234 | 9.4 | 0.97 (0.49, 1.93) | 0.28 | 0.27 |
| Malawi | iLiNS−DOSE (31) | A not B task | 176 | 70 | 8.6 | 1.13 (0.46, 2.74) | 0.16 | 0.16 | 296 | 93 | 6.5 | 1.78 (0.77, 4.11) | 0.19 | 0.22 |
| Mali | PROMIS CS (32) | | | | | | | | | | | | | |
| Zimbabwe | SHINE (HIV−) (33) | A not B task | | | | | | | | | | | | |
| Zimbabwe | SHINE (HIV+) (34) | A not B task | | | | | | | | | | | | |
| | | | **607** | **593** | | **I² = 0.00, Tau² = 0.00** | | | **801** | **661** | | **I² = 0.34, Tau² = 0.09** | | |
| **Fixed** | | | | | | **1.07 (0.76, 1.52)** | | | | | | **0.88 (0.61, 1.27)** | | |
| **Random** | | | | | | **1.07 (0.76, 1.52)** | | | | | | **0.89 (0.55, 1.44)** | | |

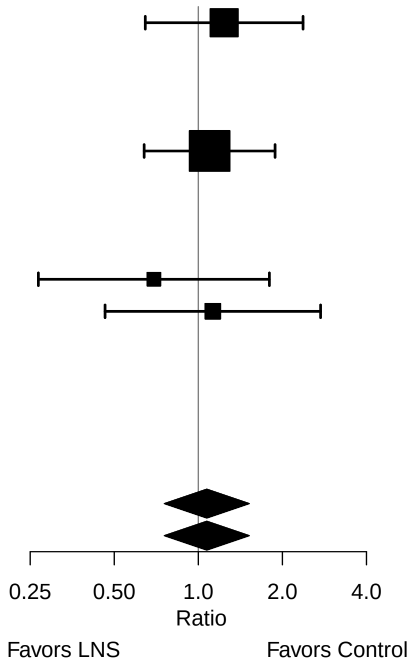


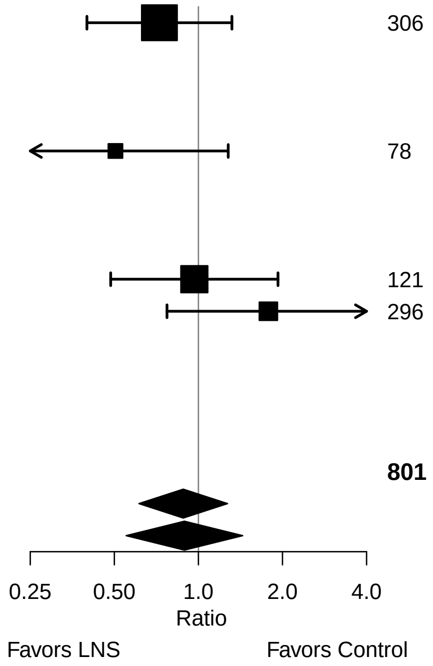

## Supplemental figure 7N: Executive function lowest decile prevalence difference

### 7N1: Stratified by Maternal height (insufficient comparisons)

## Supplemental figure 7N: Executive function lowest decile prevalence difference

## 7N2: Stratified by Maternal BMI

**P−for−interaction = 0.955**
**Difference in PDs = 0.00 (−0.03, 0.03)**

| Country | Trial | Tool | At least 20 kg/m² LNS N | Control N | Control Prevalence | PD (95% CI) | Fixed W | Random W | Less than 20 kg/m² LNS N | Control N | Control Prevalence | PD (95% CI) | Fixed W | Random W |
|---|---|---|---|---|---|---|---|---|---|---|---|---|---|---|
| Bangladesh | JiVitA−4 (21) | | | | | | | | | | | | | |
| Bangladesh | RDNS (22) | A not B task | 226 | 103 | 9.7 | −0.01 (−0.08, 0.05) | 0.10 | 0.10 | 285 | 153 | 12.4 | −0.02 (−0.08, 0.04) | 0.16 | 0.16 |
| Bangladesh | WASH−B (23) | A not B task | 495 | 1517 | 9.6 | −0.01 (−0.04, 0.02) | 0.51 | 0.51 | 597 | 1767 | 10.3 | 0.00 (−0.03, 0.03) | 0.70 | 0.70 |
| Burkina Faso | iLiNS−Zinc (24) | | | | | | | | | | | | | |
| Ghana | GHANA (25) | | | | | | | | | | | | | |
| Ghana | iLiNS−DYAD−G (26) | A not B task | | | | | | | | | | | | |
| Haiti | HAITI (27) | | | | | | | | | | | | | |
| Kenya | WASH−B (28) | | | | | | | | | | | | | |
| Madagascar | MAHAY (29) | | | | | | | | | | | | | |
| Malawi | iLiNS−DYAD−M (30) | A not B task | 108 | 221 | 9.5 | 0.00 (−0.07, 0.06) | 0.09 | 0.09 | 74 | 146 | 13.0 | −0.05 (−0.14, 0.04) | 0.07 | 0.07 |
| Malawi | iLiNS−DOSE (31) | A not B task | | | | | | | | | | | | |
| Mali | PROMIS CS (32) | | | | | | | | | | | | | |
| Zimbabwe | SHINE (HIV−) (33) | A not B task | 563 | 539 | 11.9 | −0.03 (−0.07, 0.01) | 0.29 | 0.29 | 93 | 87 | 11.5 | −0.03 (−0.12, 0.06) | 0.07 | 0.07 |
| Zimbabwe | SHINE (HIV+) (34) | A not B task | | | | | | | | | | | | |
| | | | **1392** | **2380** | | **I² = 0.00, Tau² = 0.00** | | | **1049** | **2153** | | **I² = 0.00, Tau² = 0.00** | | |
| **Fixed** | | | | | | **−0.01 (−0.03, 0.01)** | | | | | | **−0.01 (−0.03, 0.01)** | | |
| **Random** | | | | | | **−0.01 (−0.03, 0.01)** | | | | | | **−0.01 (−0.03, 0.01)** | | |

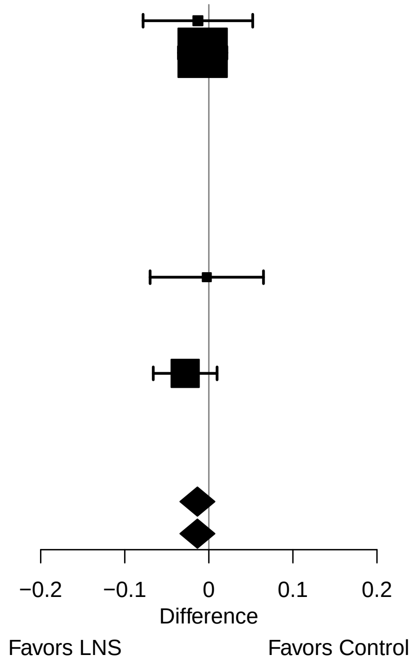


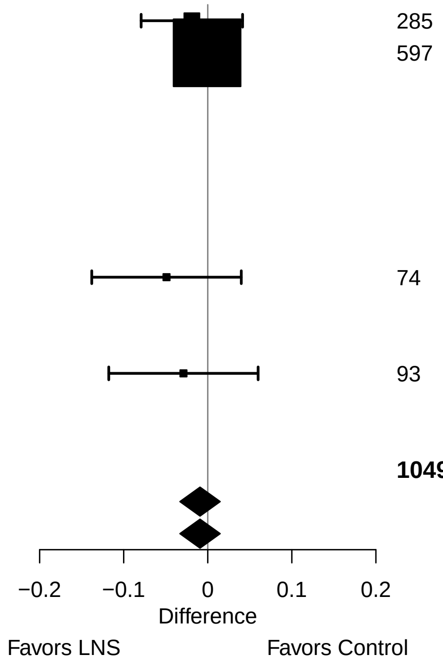

## Supplemental figure 7N: Executive function lowest decile prevalence difference

### 7N3: Stratified by Maternal age

**P−for−interaction = 0.066**
**Difference in PDs = −0.03 (−0.06, 0.00)**

| Country | Trial | Tool | LNS N | Control N | Control Prevalence | At least 25 y PD (95% CI) | Fixed W | Random W | LNS N | Control N | Control Prevalence | Less than 25 y PD (95% CI) | Fixed W | Random W |
|---|---|---|---|---|---|---|---|---|---|---|---|---|---|---|
| Bangladesh | JiVitA−4 (21) | | | | | | | | | | | | | |
| Bangladesh | RDNS (22) | A not B task | 146 | 80 | 13.8 | −0.03 (−0.14, 0.07) | 0.05 | 0.06 | 387 | 186 | 9.7 | 0.00 (−0.05, 0.04) | 0.19 | 0.19 |
| Bangladesh | WASH−B (23) | A not B task | 471 | 1469 | 9.7 | 0.02 (−0.01, 0.05) | 0.48 | 0.40 | 621 | 1844 | 10.4 | −0.02 (−0.05, 0.01) | 0.48 | 0.48 |
| Burkina Faso | iLiNS−Zinc (24) | | | | | | | | | | | | | |
| Ghana | GHANA (25) | | | | | | | | | | | | | |
| Ghana | iLiNS−DYAD−G (26) | A not B task | 185 | 362 | 12.2 | −0.03 (−0.09, 0.03) | 0.16 | 0.18 | 105 | 214 | 9.3 | −0.02 (−0.08, 0.05) | 0.10 | 0.10 |
| Haiti | HAITI (27) | | | | | | | | | | | | | |
| Kenya | WASH−B (28) | | | | | | | | | | | | | |
| Madagascar | MAHAY (29) | | | | | | | | | | | | | |
| Malawi | iLiNS−DYAD−M (30) | A not B task | 89 | 190 | 7.9 | 0.04 (−0.03, 0.12) | 0.09 | 0.11 | 93 | 179 | 14.0 | −0.09 (−0.16, −0.01) | 0.07 | 0.07 |
| Malawi | iLiNS−DOSE (31) | A not B task | | | | | | | | | | | | |
| Mali | PROMIS CS (32) | | | | | | | | | | | | | |
| Zimbabwe | SHINE (HIV−) (33) | A not B task | 399 | 358 | 11.7 | −0.02 (−0.07, 0.02) | 0.23 | 0.24 | 306 | 300 | 11.3 | −0.02 (−0.07, 0.03) | 0.16 | 0.16 |
| Zimbabwe | SHINE (HIV+) (34) | A not B task | | | | | | | | | | | | |
| | | | **1290** | **2459** | | **I² = 0.25, Tau² = 0.00** | | | **1512** | **2723** | | **I² = 0.00, Tau² = 0.00** | | |
| **Fixed** | | | | | | **0.00 (−0.02, 0.02)** | | | | | | **−0.02 (−0.04, 0.00)** | | |
| **Random** | | | | | | **0.00 (−0.03, 0.03)** | | | | | | **−0.02 (−0.04, 0.00)** | | |

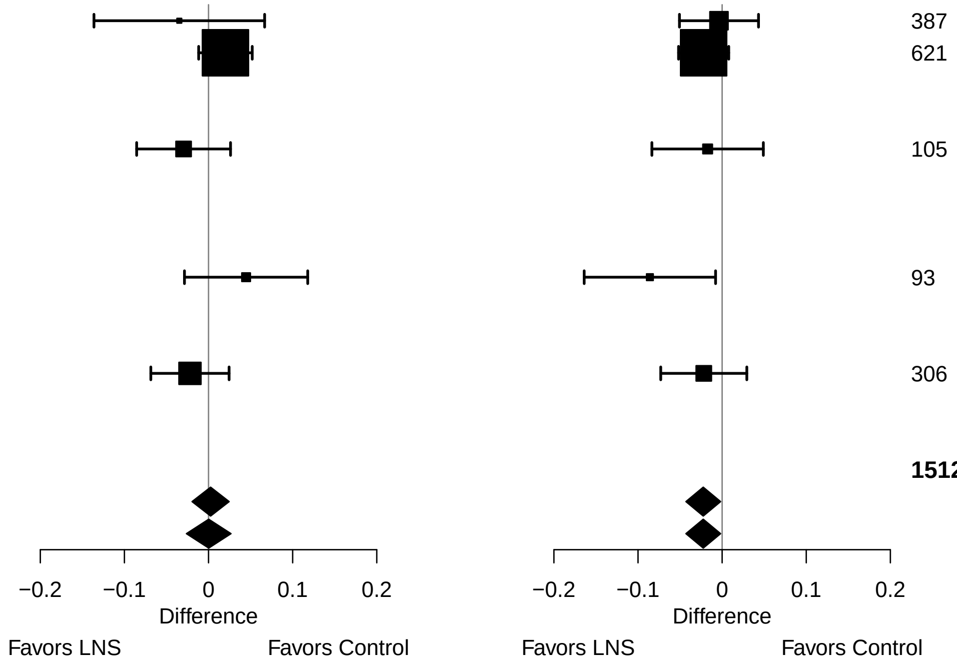

**Supplemental figure 7N: Executive function lowest decile prevalence difference**

## 7N4: Stratified by Maternal education

**P−for−interaction = 0.909**
**Difference in PDs = 0.00 (−0.04, 0.04)**

| Country | Trial | Tool | LNS N | Control N | Control Prevalence | Primary or greater PD (95% CI) | Fixed W | Random W | LNS N | Control N | Control Prevalence | Incomplete or no formal PD (95% CI) | Fixed W | Random W |
|---|---|---|---|---|---|---|---|---|---|---|---|---|---|---|
| Bangladesh | JiVitA−4 (21) | | | | | | | | | | | | | |
| Bangladesh | RDNS (22) | A not B task | 394 | 191 | 9.4 | −0.02 (−0.07, 0.03) | 0.16 | 0.16 | 139 | 75 | 14.7 | 0.01 (−0.07, 0.10) | 0.13 | 0.13 |
| Bangladesh | WASH−B (23) | A not B task | 776 | 2386 | 9.3 | −0.01 (−0.03, 0.02) | 0.65 | 0.65 | 321 | 941 | 12.1 | 0.00 (−0.04, 0.05) | 0.50 | 0.50 |
| Burkina Faso | iLiNS−Zinc (24) | | | | | | | | | | | | | |
| Ghana | GHANA (25) | | | | | | | | | | | | | |
| Ghana | iLiNS−DYAD−G (26) | A not B task | 223 | 449 | 10.0 | −0.02 (−0.07, 0.03) | 0.17 | 0.17 | 67 | 127 | 15.0 | −0.05 (−0.15, 0.06) | 0.09 | 0.09 |
| Haiti | HAITI (27) | | | | | | | | | | | | | |
| Kenya | WASH−B (28) | | | | | | | | | | | | | |
| Madagascar | MAHAY (29) | | | | | | | | | | | | | |
| Malawi | iLiNS−DYAD−M (30) | A not B task | 26 | 59 | 10.2 | 0.09 (−0.06, 0.25) | 0.02 | 0.02 | 155 | 307 | 11.1 | −0.04 (−0.10, 0.02) | 0.28 | 0.28 |
| Malawi | iLiNS−DOSE (31) | A not B task | | | | | | | | | | | | |
| Mali | PROMIS CS (32) | | | | | | | | | | | | | |
| Zimbabwe | SHINE (HIV−) (33) | A not B task | | | | | | | | | | | | |
| Zimbabwe | SHINE (HIV+) (34) | A not B task | | | | | | | | | | | | |
| | | | **1419** | **3085** | | **I² = 0.00, Tau² = 0.00** | | | **682** | **1450** | | **I² = 0.00, Tau² = 0.00** | | |
| **Fixed** | | | | | | **−0.01 (−0.03, 0.01)** | | | | | | **−0.01 (−0.04, 0.02)** | | |
| **Random** | | | | | | **−0.01 (−0.03, 0.01)** | | | | | | **−0.01 (−0.04, 0.02)** | | |

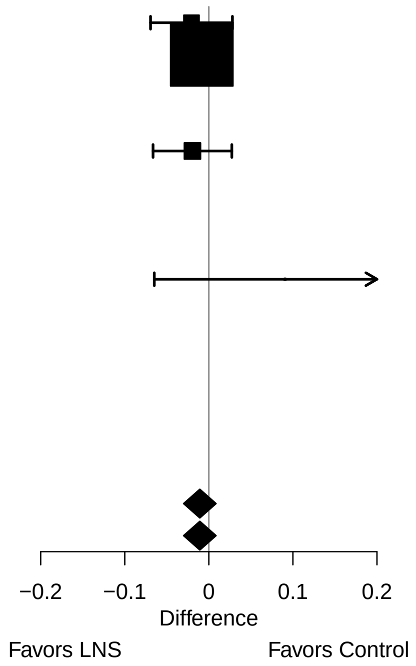


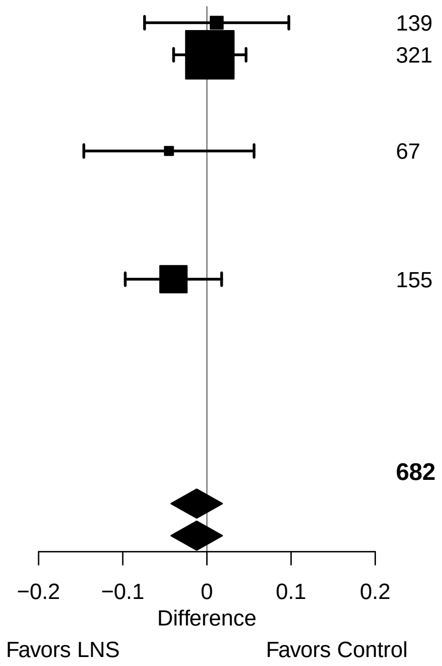

## Supplemental figure 7N: Executive function lowest decile prevalence difference

### 7N5: Stratified by Maternal depressive symptoms

**P−for−interaction = 0.401**
**Difference in PDs = 0.02 (−0.02, 0.06)**

| Country | Trial | Tool | LNS N | Control N | Control Prevalence | Less than 75th percentile PD (95% CI) | Fixed W | Random W | LNS N | Control N | Control Prevalence | At least 75th percentile PD (95% CI) | Fixed W | Random W |
|---|---|---|---|---|---|---|---|---|---|---|---|---|---|---|
| Bangladesh | JiVitA−4 (21) | | | | | | | | | | | | | |
| Bangladesh | RDNS (22) | A not B task | 345 | 150 | 10.0 | −0.01 (−0.06, 0.04) | 0.15 | 0.15 | 166 | 100 | 12.0 | −0.02 (−0.12, 0.07) | 0.13 | 0.13 |
| Bangladesh | WASH−B (23) | A not B task | 847 | 2392 | 9.4 | 0.00 (−0.03, 0.02) | 0.62 | 0.62 | 230 | 852 | 12.1 | 0.00 (−0.05, 0.05) | 0.49 | 0.49 |
| Burkina Faso | iLiNS−Zinc (24) | | | | | | | | | | | | | |
| Ghana | GHANA (25) | | | | | | | | | | | | | |
| Ghana | iLiNS−DYAD−G (26) | A not B task | | | | | | | | | | | | |
| Haiti | HAITI (27) | | | | | | | | | | | | | |
| Kenya | WASH−B (28) | | | | | | | | | | | | | |
| Madagascar | MAHAY (29) | | | | | | | | | | | | | |
| Malawi | iLiNS−DYAD−M (30) | A not B task | | | | | | | | | | | | |
| Malawi | iLiNS−DOSE (31) | A not B task | | | | | | | | | | | | |
| Mali | PROMIS CS (32) | | | | | | | | | | | | | |
| Zimbabwe | SHINE (HIV−) (33) | A not B task | 542 | 520 | 11.5 | −0.01 (−0.05, 0.03) | 0.23 | 0.23 | 186 | 164 | 11.6 | −0.06 (−0.11, 0.00) | 0.38 | 0.38 |
| Zimbabwe | SHINE (HIV+) (34) | A not B task | | | | | | | | | | | | |
| | | | **1734** | **3062** | | **I² = 0.00, Tau² = 0.00** | | | **582** | **1116** | | **I² = 0.00, Tau² = 0.00** | | |
| **Fixed** | | | | | | **−0.01 (−0.02, 0.01)** | | | | | | **−0.03 (−0.06, 0.01)** | | |
| **Random** | | | | | | **−0.01 (−0.02, 0.01)** | | | | | | **−0.03 (−0.06, 0.01)** | | |

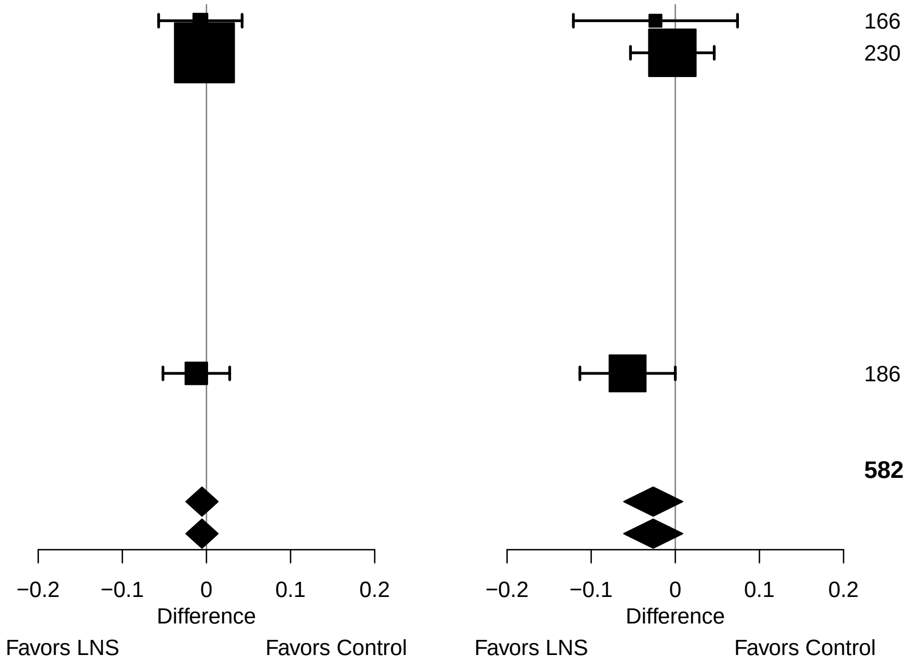

## Supplemental figure 7N: Executive function lowest decile prevalence difference

### 7N6: Stratified by Child sex

**P−for−interaction = 0.267**
**Difference in PDs = 0.02 (−0.01, 0.04)**

| Country | Trial | Tool | Male LNS N | Male Control N | Male Control Prevalence | Male PD (95% CI) | Male Fixed W | Male Random W | Female LNS N | Female Control N | Female Control Prevalence | Female PD (95% CI) | Female Fixed W | Female Random W |
|---|---|---|---|---|---|---|---|---|---|---|---|---|---|---|
| Bangladesh | JiVitA−4 (21) | | | | | | | | | | | | | |
| Bangladesh | RDNS (22) | A not B task | 261 | 133 | 6.8 | 0.03 (−0.03, 0.08) | 0.11 | 0.15 | 272 | 133 | 15.0 | −0.05 (−0.11, 0.00) | 0.14 | 0.15 |
| Bangladesh | WASH−B (23) | A not B task | 540 | 1671 | 9.8 | −0.01 (−0.04, 0.02) | 0.44 | 0.30 | 557 | 1656 | 10.5 | 0.00 (−0.03, 0.03) | 0.47 | 0.43 |
| Burkina Faso | iLiNS−Zinc (24) | | | | | | | | | | | | | |
| Ghana | GHANA (25) | | | | | | | | | | | | | |
| Ghana | iLiNS−DYAD−G (26) | A not B task | 145 | 273 | 11.0 | −0.04 (−0.10, 0.02) | 0.10 | 0.13 | 145 | 303 | 11.2 | −0.01 (−0.07, 0.05) | 0.10 | 0.11 |
| Haiti | HAITI (27) | | | | | | | | | | | | | |
| Kenya | WASH−B (28) | | | | | | | | | | | | | |
| Madagascar | MAHAY (29) | | | | | | | | | | | | | |
| Malawi | iLiNS−DYAD−M (30) | A not B task | 92 | 176 | 12.5 | −0.05 (−0.13, 0.03) | 0.06 | 0.09 | 90 | 193 | 9.3 | 0.01 (−0.07, 0.08) | 0.07 | 0.08 |
| Malawi | iLiNS−DOSE (31) | A not B task | 235 | 93 | 7.5 | 0.03 (−0.04, 0.10) | 0.07 | 0.10 | 240 | 71 | 7.0 | 0.04 (−0.04, 0.12) | 0.06 | 0.07 |
| Mali | PROMIS CS (32) | | | | | | | | | | | | | |
| Zimbabwe | SHINE (HIV−) (33) | A not B task | 399 | 364 | 8.8 | −0.04 (−0.08, 0.00) | 0.23 | 0.23 | 388 | 375 | 14.1 | 0.00 (−0.05, 0.05) | 0.15 | 0.16 |
| Zimbabwe | SHINE (HIV+) (34) | A not B task | | | | | | | | | | | | |
| | | | **1672** | **2710** | | **I² = 0.32, Tau² = 0.00** | | | **1692** | **2731** | | **I² = 0.04, Tau² = 0.00** | | |
| **Fixed** | | | | | | **−0.02 (−0.04, 0.00)** | | | | | | **0.00 (−0.02, 0.01)** | | |
| **Random** | | | | | | **−0.02 (−0.04, 0.01)** | | | | | | **−0.01 (−0.03, 0.02)** | | |

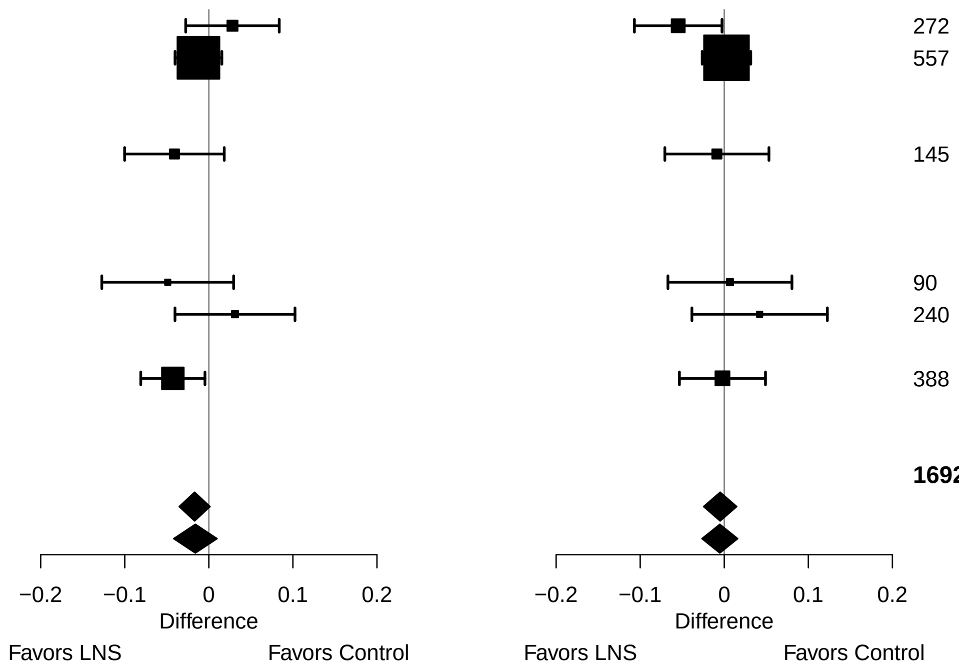

## Supplemental figure 7N: Executive function lowest decile prevalence difference

## 7N7: Stratified by Child birth order

**P−for−interaction = 0.116**
**Difference in PDs = −0.02 (−0.06, 0.01)**

| Country | Trial | Tool | LNS N | Control N | Control Prevalence | Later born PD (95% CI) | Fixed W | Random W | LNS N | Control N | Control Prevalence | Firstborn PD (95% CI) | Fixed W | Random W |
|---|---|---|---|---|---|---|---|---|---|---|---|---|---|---|
| Bangladesh | JiVitA−4 (21) | | | | | | | | | | | | | |
| Bangladesh | RDNS (22) | A not B task | 320 | 163 | 12.9 | −0.04 (−0.09, 0.01) | 0.13 | 0.16 | 213 | 103 | 7.8 | 0.03 (−0.04, 0.10) | 0.15 | 0.17 |
| Bangladesh | WASH−B (23) | A not B task | 683 | 2174 | 9.5 | 0.01 (−0.01, 0.04) | 0.52 | 0.43 | 394 | 1049 | 10.3 | −0.03 (−0.06, 0.01) | 0.54 | 0.46 |
| Burkina Faso | iLiNS−Zinc (24) | | | | | | | | | | | | | |
| Ghana | GHANA (25) | | | | | | | | | | | | | |
| Ghana | iLiNS−DYAD−G (26) | A not B task | 195 | 390 | 10.8 | −0.01 (−0.06, 0.04) | 0.12 | 0.15 | 95 | 186 | 11.8 | −0.06 (−0.13, 0.02) | 0.12 | 0.15 |
| Haiti | HAITI (27) | | | | | | | | | | | | | |
| Kenya | WASH−B (28) | | | | | | | | | | | | | |
| Madagascar | MAHAY (29) | | | | | | | | | | | | | |
| Malawi | iLiNS−DYAD−M (30) | A not B task | | | | | | | | | | | | |
| Malawi | iLiNS−DOSE (31) | A not B task | | | | | | | | | | | | |
| Mali | PROMIS CS (32) | | | | | | | | | | | | | |
| Zimbabwe | SHINE (HIV−) (33) | A not B task | 568 | 530 | 11.7 | −0.02 (−0.06, 0.02) | 0.24 | 0.26 | 186 | 178 | 11.8 | −0.04 (−0.10, 0.02) | 0.19 | 0.22 |
| Zimbabwe | SHINE (HIV+) (34) | A not B task | | | | | | | | | | | | |
| | | | **1766** | **3257** | | **I² = 0.32, Tau² = 0.00** | | | **888** | **1516** | | **I² = 0.14, Tau² = 0.00** | | |
| **Fixed** | | | | | | **0.00 (−0.02, 0.01)** | | | | | | **−0.03 (−0.05, 0.00)** | | |
| **Random** | | | | | | **−0.01 (−0.03, 0.02)** | | | | | | **−0.02 (−0.05, 0.01)** | | |

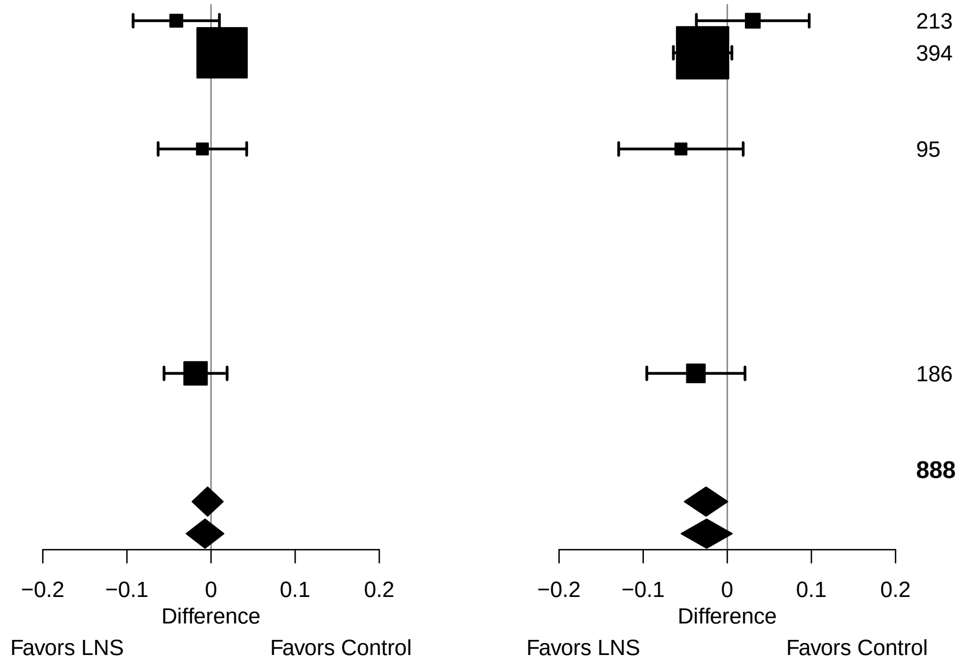

## Supplemental figure 7N: Executive function lowest decile prevalence difference

### 7N8: Stratified by Child baseline stunting

**P−for−interaction = 0.577**
**Difference in PDs = 0.02 (−0.04, 0.08)**

| Country | Trial | Tool | LNS N | Control N | Control Prevalence | No PD (95% CI) | Fixed W | Random W | LNS N | Control N | Control Prevalence | Yes PD (95% CI) | Fixed W | Random W |
|---|---|---|---|---|---|---|---|---|---|---|---|---|---|---|
| Bangladesh | JiVitA−4 (21) | | | | | | | | | | | | | |
| Bangladesh | RDNS (22) | A not B task | 404 | 187 | 10.7 | −0.02 (−0.06, 0.03) | 0.33 | 0.34 | 107 | 62 | 11.3 | 0.00 (−0.12, 0.12) | 0.24 | 0.24 |
| Bangladesh | WASH−B (23) | A not B task | | | | | | | | | | | | |
| Burkina Faso | iLiNS−Zinc (24) | | | | | | | | | | | | | |
| Ghana | GHANA (25) | | | | | | | | | | | | | |
| Ghana | iLiNS−DYAD−G (26) | A not B task | | | | | | | | | | | | |
| Haiti | HAITI (27) | | | | | | | | | | | | | |
| Kenya | WASH−B (28) | | | | | | | | | | | | | |
| Madagascar | MAHAY (29) | | | | | | | | | | | | | |
| Malawi | iLiNS−DYAD−M (30) | A not B task | | | | | | | | | | | | |
| Malawi | iLiNS−DOSE (31) | A not B task | 353 | 111 | 5.4 | 0.06 (0.00, 0.12) | 0.18 | 0.28 | 122 | 53 | 11.3 | −0.01 (−0.11, 0.08) | 0.36 | 0.36 |
| Mali | PROMIS CS (32) | | | | | | | | | | | | | |
| Zimbabwe | SHINE (HIV−) (33) | A not B task | 497 | 459 | 11.5 | −0.03 (−0.07, 0.01) | 0.49 | 0.38 | 116 | 93 | 9.7 | 0.05 (−0.04, 0.14) | 0.40 | 0.40 |
| Zimbabwe | SHINE (HIV+) (34) | A not B task | | | | | | | | | | | | |
| | | | **1254** | **757** | | **I² = 0.67, Tau² = 0.00** | | | **345** | **208** | | **I² = 0.00, Tau² = 0.00** | | |
| **Fixed** | | | | | | **−0.01 (−0.04, 0.02)** | | | | | | **0.01 (−0.04, 0.07)** | | |
| **Random** | | | | | | **0.00 (−0.06, 0.05)** | | | | | | **0.01 (−0.04, 0.07)** | | |

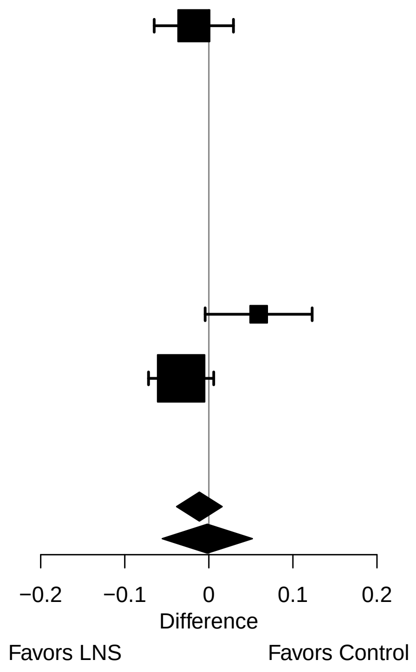


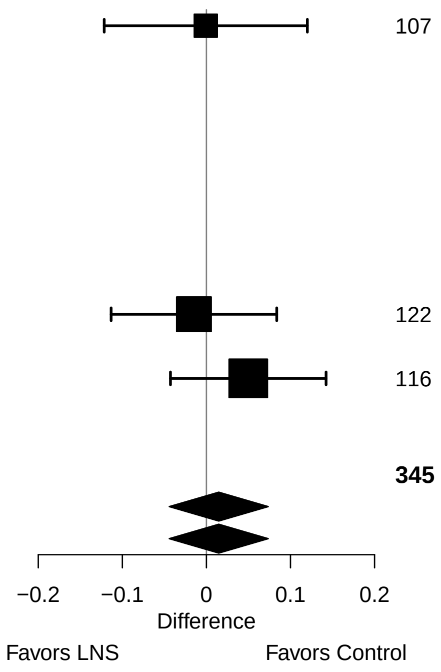

**Supplemental figure 7N: Executive function lowest decile prevalence difference**

## 7N9: Stratified by Child baseline acute malnutrition (insufficient comparisons)

## Supplemental figure 7N: Executive function lowest decile prevalence difference

## 7N10: Stratified by Child baseline anemia

**P−for−interaction = 0.432**
**Difference in PDs = −0.02 (−0.07, 0.03)**

| Country | Trial | Tool | LNS N | Control N | Control Prevalence | Not anemic PD (95% CI) | Fixed W | Random W | LNS N | Control N | Control Prevalence | Anemic PD (95% CI) | Fixed W | Random W |
|---|---|---|---|---|---|---|---|---|---|---|---|---|---|---|
| Bangladesh | JiVitA−4 (21) | | | | | | | | | | | | | |
| Bangladesh | RDNS (22) | A not B task | 204 | 93 | 7.5 | 0.02 (−0.04, 0.07) | 0.38 | 0.38 | 306 | 153 | 13.1 | −0.04 (−0.11, 0.03) | 0.26 | 0.26 |
| Bangladesh | WASH−B (23) | A not B task | | | | | | | | | | | | |
| Burkina Faso | iLiNS−Zinc (24) | | | | | | | | | | | | | |
| Ghana | GHANA (25) | | | | | | | | | | | | | |
| Ghana | iLiNS−DYAD−G (26) | A not B task | 172 | 308 | 10.1 | 0.01 (−0.05, 0.07) | 0.34 | 0.34 | 78 | 181 | 12.7 | −0.06 (−0.15, 0.02) | 0.19 | 0.21 |
| Haiti | HAITI (27) | | | | | | | | | | | | | |
| Kenya | WASH−B (28) | | | | | | | | | | | | | |
| Madagascar | MAHAY (29) | | | | | | | | | | | | | |
| Malawi | iLiNS−DYAD−M (30) | A not B task | 55 | 122 | 13.1 | −0.04 (−0.14, 0.06) | 0.10 | 0.10 | 121 | 234 | 9.4 | 0.00 (−0.07, 0.06) | 0.31 | 0.28 |
| Malawi | iLiNS−DOSE (31) | A not B task | 176 | 70 | 8.6 | 0.01 (−0.07, 0.09) | 0.17 | 0.17 | 296 | 93 | 6.5 | 0.05 (−0.02, 0.12) | 0.25 | 0.25 |
| Mali | PROMIS CS (32) | | | | | | | | | | | | | |
| Zimbabwe | SHINE (HIV−) (33) | A not B task | | | | | | | | | | | | |
| Zimbabwe | SHINE (HIV+) (34) | A not B task | | | | | | | | | | | | |
| | | | **607** | **593** | | **I² = 0.00, Tau² = 0.00** | | | **801** | **661** | | **I² = 0.39, Tau² = 0.00** | | |
| **Fixed** | | | | | | **0.01 (−0.03, 0.04)** | | | | | | **−0.01 (−0.04, 0.03)** | | |
| **Random** | | | | | | **0.01 (−0.03, 0.04)** | | | | | | **−0.01 (−0.06, 0.04)** | | |

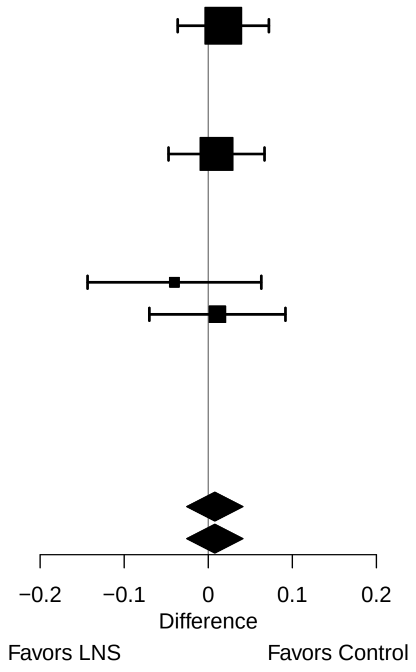


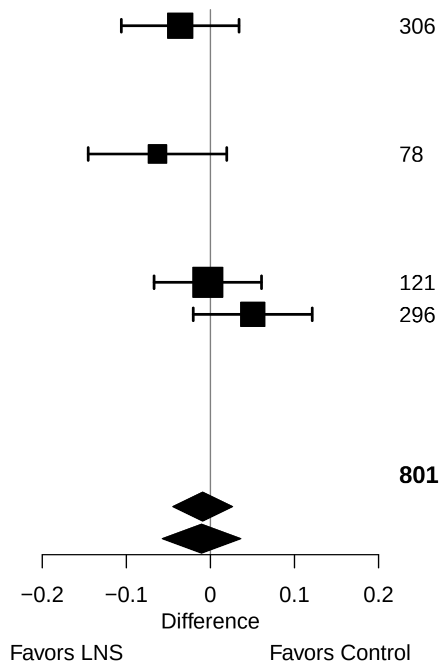

# Supplemental figure 7O: 12-mo walking without support prevalence ratio

## 7O1: Stratified by Maternal height

**P−for−interaction = 0.624**
**Ratio of PRs = 0.96 (0.79, 1.15)**

| | | At least 150.1 cm | | | | | | Less than 150.1 cm | | | | | |
|---|---|---|---|---|---|---|---|---|---|---|---|---|---|
| **Country** | **Trial** | **LNS N** | **Control N** | **Control Prevalence** | **PR (95% CI)** | **Fixed W** | **Random W** | **LNS N** | **Control N** | **Control Prevalence** | **PR (95% CI)** | **Fixed W** | **Random W** |
| Bangladesh | JiVitA−4 (21) | | | | | | | | | | | | |
| Bangladesh | RDNS (22) | 848 | 418 | 74.4 | 1.42 (1.16, 1.73) | 0.15 | 0.16 | 721 | 347 | 76.4 | 1.17 (0.87, 1.56) | 0.27 | 0.27 |
| Bangladesh | WASH−B (23) | 261 | 782 | 73.0 | 1.21 (0.99, 1.47) | 0.16 | 0.16 | 230 | 666 | 76.1 | 1.22 (0.98, 1.52) | 0.46 | 0.46 |
| Burkina Faso | iLiNS−Zinc (24) | | | | | | | | | | | | |
| Ghana | GHANA (25) | | | | | | | | | | | | |
| Ghana | iLiNS−DYAD−G (26) | 308 | 613 | 52.9 | 1.13 (0.99, 1.29) | 0.34 | 0.34 | 14 | 38 | 63.2 | 1.74 (0.99, 3.09) | 0.07 | 0.07 |
| Haiti | HAITI (27) | | | | | | | | | | | | |
| Kenya | WASH−B (28) | | | | | | | | | | | | |
| Madagascar | MAHAY (29) | | | | | | | | | | | | |
| Malawi | iLiNS−DYAD−M (30) | 180 | 367 | 50.4 | 1.20 (1.02, 1.40) | 0.24 | 0.24 | 28 | 56 | 53.6 | 1.15 (0.74, 1.80) | 0.12 | 0.12 |
| Malawi | iLiNS−DOSE (31) | 505 | 164 | 66.5 | 1.10 (0.86, 1.40) | 0.10 | 0.10 | 96 | 35 | 62.9 | 0.84 (0.50, 1.42) | 0.08 | 0.08 |
| Mali | PROMIS CS (32) | | | | | | | | | | | | |
| Zimbabwe | SHINE (HIV−) (33) | | | | | | | | | | | | |
| Zimbabwe | SHINE (HIV+) (34) | | | | | | | | | | | | |
| | | **2102** | **2344** | | **I² = 0.00, Tau² = 0.00** | | | **1089** | **1142** | | **I² = 0.00, Tau² = 0.00** | | |
| **Fixed** | | | | | **1.20 (1.11, 1.29)** | | | | | | **1.19 (1.02, 1.39)** | | |
| **Random** | | | | | **1.20 (1.11, 1.29)** | | | | | | **1.19 (1.02, 1.39)** | | |

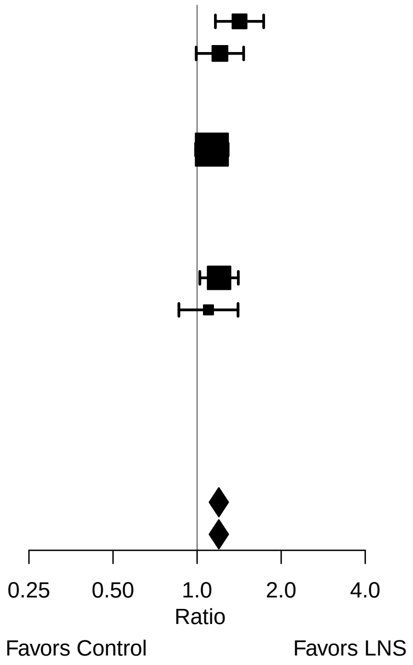


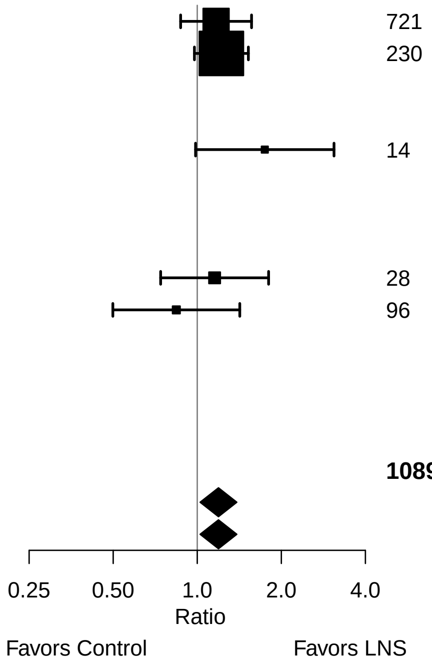

### Supplemental figure 7O: 12-mo walking without support prevalence ratio

## 7O2: Stratified by Maternal BMI

**P−for−interaction = 0.343**
**Ratio of PRs = 0.94 (0.82, 1.07)**

| Country | Trial | At least 20 kg/m²: LNS N | Control N | Control Prevalence | PR (95% CI) | Fixed W | Random W | Less than 20 kg/m²: LNS N | Control N | Control Prevalence | PR (95% CI) | Fixed W | Random W |
|---|---|---|---|---|---|---|---|---|---|---|---|---|---|
| Bangladesh | JiVitA−4 (21) | | | | | | | | | | | | |
| Bangladesh | RDNS (22) | 708 | 327 | 74.6 | 1.57 (1.20, 2.05) | 0.08 | 0.13 | 861 | 438 | 75.8 | 1.08 (0.88, 1.32) | 0.27 | 0.27 |
| Bangladesh | WASH−B (23) | 225 | 637 | 73.0 | 1.30 (1.06, 1.60) | 0.13 | 0.15 | 266 | 810 | 75.7 | 1.13 (0.91, 1.39) | 0.25 | 0.25 |
| Burkina Faso | iLiNS−Zinc (24) | | | | | | | | | | | | |
| Ghana | GHANA (25) | | | | | | | | | | | | |
| Ghana | iLiNS−DYAD−G (26) | 284 | 535 | 52.3 | 1.15 (1.00, 1.32) | 0.29 | 0.18 | 38 | 116 | 58.6 | 1.08 (0.71, 1.64) | 0.06 | 0.06 |
| Haiti | HAITI (27) | | | | | | | | | | | | |
| Kenya | WASH−B (28) | 334 | 1210 | 58.8 | 0.92 (0.79, 1.07) | 0.25 | 0.17 | 96 | 364 | 61.5 | 1.03 (0.77, 1.38) | 0.13 | 0.13 |
| Madagascar | MAHAY (29) | | | | | | | | | | | | |
| Malawi | iLiNS−DYAD−M (30) | 121 | 255 | 51.4 | 1.17 (0.96, 1.43) | 0.14 | 0.16 | 86 | 168 | 50.0 | 1.23 (0.98, 1.54) | 0.22 | 0.22 |
| Malawi | iLiNS−DOSE (31) | 451 | 138 | 63.0 | 1.00 (0.78, 1.28) | 0.09 | 0.14 | 148 | 60 | 71.7 | 1.19 (0.75, 1.89) | 0.05 | 0.05 |
| Mali | PROMIS CS (32) | 83 | 98 | 66.3 | 0.68 (0.41, 1.14) | 0.02 | 0.07 | 34 | 52 | 67.3 | 0.81 (0.41, 1.60) | 0.02 | 0.02 |
| Zimbabwe | SHINE (HIV−) (33) | | | | | | | | | | | | |
| Zimbabwe | SHINE (HIV+) (34) | | | | | | | | | | | | |
| | | **2206** | **3200** | | **I² = 0.69, Tau² = 0.04** | | | **1529** | **2008** | | **I² = 0.00, Tau² = 0.00** | | |
| **Fixed** | | | | | **1.11 (1.03, 1.20)** | | | | | | **1.11 (1.00, 1.24)** | | |
| **Random** | | | | | **1.11 (0.94, 1.32)** | | | | | | **1.11 (1.00, 1.24)** | | |

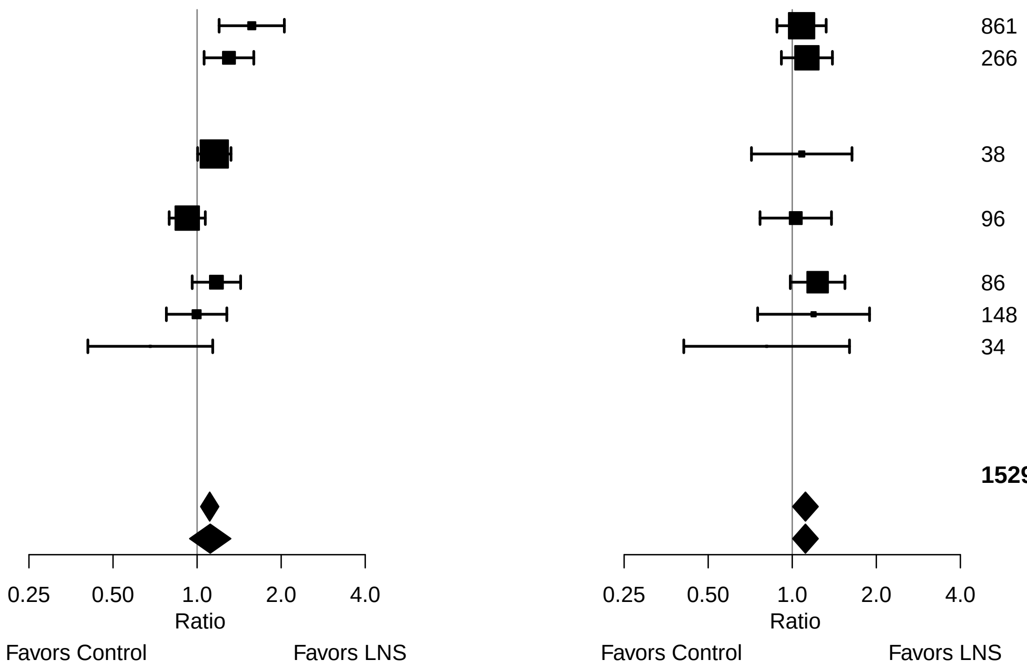

## Supplemental figure 7O: 12-mo walking without support prevalence ratio

### 7O3: Stratified by Maternal age

**P−for−interaction = 0.394**
**Ratio of PRs = 1.04 (0.95, 1.13)**

| Country | Trial | At least 25 y: LNS N | At least 25 y: Control N | At least 25 y: Control Prevalence | At least 25 y: PR (95% CI) | At least 25 y: Fixed W | At least 25 y: Random W | Less than 25 y: LNS N | Less than 25 y: Control N | Less than 25 y: Control Prevalence | Less than 25 y: PR (95% CI) | Less than 25 y: Fixed W | Less than 25 y: Random W |
|---|---|---|---|---|---|---|---|---|---|---|---|---|---|
| Bangladesh | JiVitA−4 (21) | 1273 | 587 | 47.5 | 1.05 (0.96, 1.15) | 0.46 | 0.19 | 1726 | 769 | 45.0 | 1.05 (0.97, 1.14) | 0.51 | 0.18 |
| Bangladesh | RDNS (22) | 447 | 217 | 76.0 | 1.37 (1.07, 1.76) | 0.06 | 0.11 | 1181 | 573 | 73.8 | 1.25 (1.02, 1.53) | 0.08 | 0.13 |
| Bangladesh | WASH−B (23) | 223 | 676 | 74.6 | 0.95 (0.74, 1.22) | 0.06 | 0.11 | 274 | 791 | 74.6 | 1.41 (1.18, 1.67) | 0.11 | 0.14 |
| Burkina Faso | iLiNS−Zinc (24) | | | | | | | | | | | | |
| Ghana | GHANA (25) | | | | | | | | | | | | |
| Ghana | iLiNS−DYAD−G (26) | 209 | 405 | 53.8 | 1.08 (0.91, 1.28) | 0.13 | 0.15 | 118 | 258 | 51.9 | 1.27 (1.05, 1.54) | 0.09 | 0.13 |
| Haiti | HAITI (27) | 60 | 57 | 68.4 | 1.58 (1.00, 2.50) | 0.02 | 0.05 | 22 | 22 | 50.0 | 0.73 (0.36, 1.45) | 0.01 | 0.03 |
| Kenya | WASH−B (28) | 243 | 914 | 61.3 | 1.03 (0.87, 1.22) | 0.13 | 0.15 | 197 | 679 | 57.7 | 0.91 (0.76, 1.10) | 0.10 | 0.14 |
| Madagascar | MAHAY (29) | | | | | | | | | | | | |
| Malawi | iLiNS−DYAD−M (30) | 100 | 213 | 47.9 | 1.06 (0.85, 1.31) | 0.08 | 0.12 | 108 | 213 | 54.5 | 1.36 (1.11, 1.68) | 0.08 | 0.13 |
| Malawi | iLiNS−DOSE (31) | 327 | 111 | 64.9 | 1.11 (0.83, 1.47) | 0.05 | 0.09 | 264 | 83 | 67.5 | 0.99 (0.69, 1.41) | 0.03 | 0.08 |
| Mali | PROMIS CS (32) | 59 | 71 | 62.0 | 0.58 (0.32, 1.06) | 0.01 | 0.03 | 59 | 80 | 70.0 | 0.85 (0.49, 1.45) | 0.01 | 0.04 |
| Zimbabwe | SHINE (HIV−) (33) | | | | | | | | | | | | |
| Zimbabwe | SHINE (HIV+) (34) | | | | | | | | | | | | |
| | | **2941** | **3251** | | **I² = 0.32, Tau² = 0.02** | | | **3949** | **3468** | | **I² = 0.65, Tau² = 0.02** | | |
| **Fixed** | | | | | **1.07 (1.00, 1.14)** | | | | | | **1.12 (1.05, 1.18)** | | |
| **Random** | | | | | **1.08 (0.96, 1.21)** | | | | | | **1.14 (1.01, 1.29)** | | |

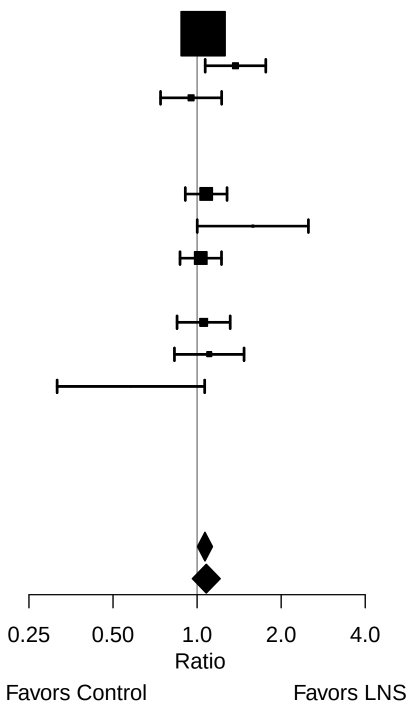


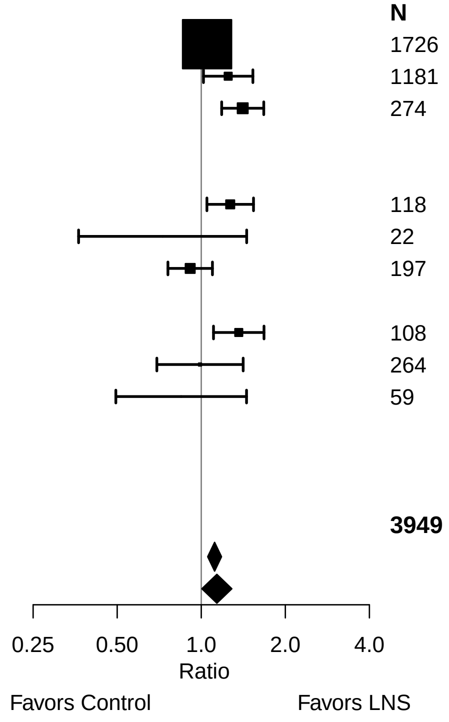

## Supplemental figure 7O: 12-mo walking without support prevalence ratio

### 7O4: Stratified by Maternal education

**P−for−interaction** = **0.792**
**Ratio of PRs =** **0.99 (0.91, 1.08)**

| Country | Trial | Primary or greater: LNS N | Control N | Control Prevalence | PR (95% CI) | Fixed W | Random W | Incomplete or no formal: LNS N | Control N | Control Prevalence | PR (95% CI) | Fixed W | Random W |
|---|---|---|---|---|---|---|---|---|---|---|---|---|---|
| Bangladesh | JiVitA−4 (21) | 1933 | 848 | 43.8 | 1.04 (0.96, 1.12) | 0.53 | 0.32 | 1075 | 512 | 50.0 | 1.07 (0.97, 1.18) | 0.47 | 0.37 |
| Bangladesh | RDNS (22) | 1219 | 574 | 73.9 | 1.32 (1.08, 1.62) | 0.07 | 0.11 | 409 | 216 | 75.9 | 1.14 (0.91, 1.42) | 0.09 | 0.11 |
| Bangladesh | WASH−B (23) | 337 | 1048 | 72.7 | 1.28 (1.11, 1.49) | 0.13 | 0.17 | 160 | 426 | 79.1 | 1.02 (0.76, 1.37) | 0.05 | 0.07 |
| Burkina Faso | iLiNS−Zinc (24) | | | | | | | | | | | | |
| Ghana | GHANA (25) | | | | | | | | | | | | |
| Ghana | iLiNS−DYAD−G (26) | 257 | 524 | 51.5 | 1.12 (0.97, 1.30) | 0.14 | 0.18 | 70 | 139 | 59.0 | 1.25 (0.93, 1.70) | 0.05 | 0.06 |
| Haiti | HAITI (27) | | | | | | | | | | | | |
| Kenya | WASH−B (28) | 217 | 775 | 59.2 | 1.04 (0.87, 1.24) | 0.09 | 0.14 | 229 | 827 | 60.3 | 0.89 (0.72, 1.10) | 0.11 | 0.12 |
| Madagascar | MAHAY (29) | | | | | | | | | | | | |
| Malawi | iLiNS−DYAD−M (30) | 32 | 64 | 42.2 | 0.97 (0.67, 1.41) | 0.02 | 0.04 | 175 | 359 | 52.4 | 1.24 (1.05, 1.46) | 0.17 | 0.18 |
| Malawi | iLiNS−DOSE (31) | 129 | 42 | 61.9 | 1.08 (0.70, 1.67) | 0.02 | 0.03 | 462 | 152 | 67.1 | 1.05 (0.81, 1.36) | 0.07 | 0.08 |
| Mali | PROMIS CS (32) | | | | | | | | | | | | |
| Zimbabwe | SHINE (HIV−) (33) | | | | | | | | | | | | |
| Zimbabwe | SHINE (HIV+) (34) | | | | | | | | | | | | |
| | | **4124** | **3875** | | **I² = 0.44, Tau² = 0.00** | | | **2580** | **2631** | | **I² = 0.16, Tau² = 0.00** | | |
| **Fixed** | | | | | **1.10 (1.04, 1.16)** | | | | | | **1.09 (1.02, 1.16)** | | |
| **Random** | | | | | **1.12 (1.04, 1.21)** | | | | | | **1.09 (1.00, 1.18)** | | |

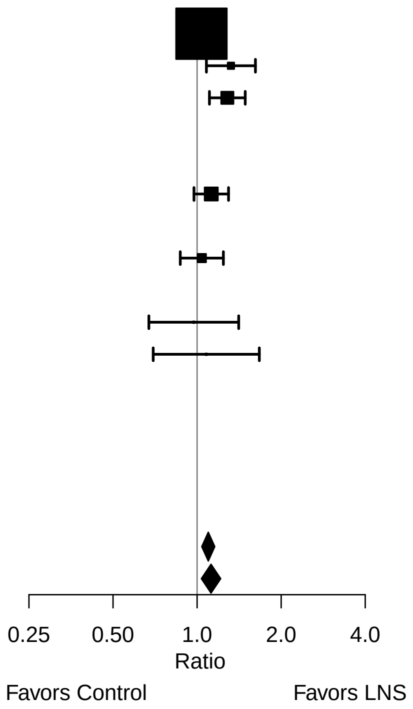


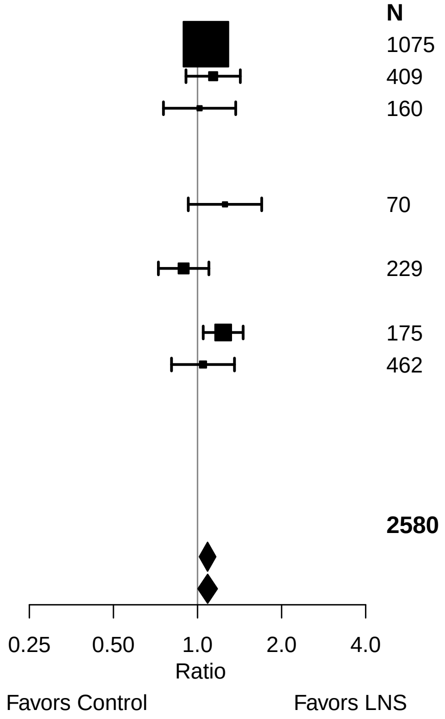

## Supplemental figure 7O: 12-mo walking without support prevalence ratio

### 7O5: Stratified by Maternal depressive symptoms

**P−for−interaction = 0.543**
**Ratio of PRs = 0.95 (0.81, 1.12)**

| | | Less than 75th percentile | | | | | | At least 75th percentile | | | | | |
|---|---|---|---|---|---|---|---|---|---|---|---|---|---|
| **Country** | **Trial** | **LNS N** | **Control N** | **Control Prevalence** | **PR (95% CI)** | **Fixed W** | **Random W** | **LNS N** | **Control N** | **Control Prevalence** | **PR (95% CI)** | **Fixed W** | **Random W** |
| Bangladesh | JiVitA−4 (21) | | | | | | | | | | | | |
| Bangladesh | RDNS (22) | 1058 | 460 | 70.9 | 1.11 (0.89, 1.38) | 0.11 | 0.13 | 508 | 279 | 79.6 | 1.67 (1.26, 2.21) | 0.24 | 0.22 |
| Bangladesh | WASH−B (23) | 389 | 1075 | 73.4 | 1.28 (1.08, 1.50) | 0.20 | 0.21 | 108 | 399 | 77.7 | 0.83 (0.53, 1.29) | 0.10 | 0.15 |
| Burkina Faso | iLiNS−Zinc (24) | | | | | | | | | | | | |
| Ghana | GHANA (25) | | | | | | | | | | | | |
| Ghana | iLiNS−DYAD−G (26) | 242 | 446 | 52.9 | 1.16 (1.00, 1.35) | 0.24 | 0.23 | 75 | 198 | 55.1 | 1.16 (0.89, 1.51) | 0.27 | 0.23 |
| Haiti | HAITI (27) | | | | | | | | | | | | |
| Kenya | WASH−B (28) | 314 | 1098 | 59.6 | 0.98 (0.85, 1.14) | 0.25 | 0.23 | 99 | 373 | 61.1 | 1.01 (0.77, 1.33) | 0.25 | 0.23 |
| Madagascar | MAHAY (29) | | | | | | | | | | | | |
| Malawi | iLiNS−DYAD−M (30) | 149 | 293 | 48.1 | 1.20 (1.02, 1.42) | 0.20 | 0.20 | 47 | 108 | 60.2 | 1.23 (0.85, 1.78) | 0.14 | 0.17 |
| Malawi | iLiNS−DOSE (31) | | | | | | | | | | | | |
| Mali | PROMIS CS (32) | | | | | | | | | | | | |
| Zimbabwe | SHINE (HIV−) (33) | | | | | | | | | | | | |
| Zimbabwe | SHINE (HIV+) (34) | | | | | | | | | | | | |
| | | **2152** | **3372** | | **I² = 0.34, Tau² = 0.00** | | | **837** | **1357** | | **I² = 0.57, Tau² = 0.04** | | |
| **Fixed** | | | | | **1.14 (1.06, 1.23)** | | | | | | **1.19 (1.04, 1.37)** | | |
| **Random** | | | | | **1.14 (1.04, 1.25)** | | | | | | **1.17 (0.94, 1.46)** | | |

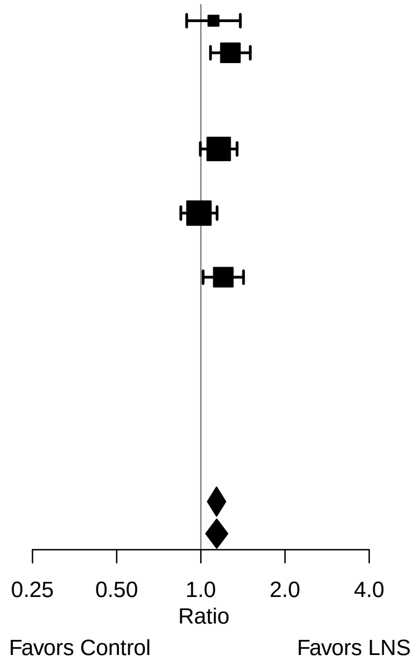


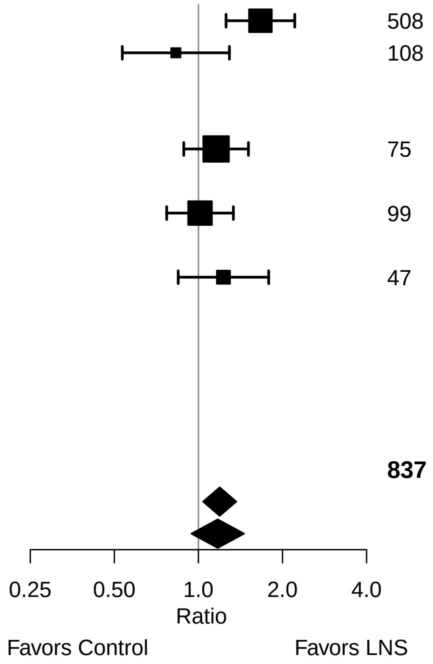

## Supplemental figure 7O: 12-mo walking without support prevalence ratio

## 7O6: Stratified by Child sex

**P−for−interaction = 0.952**
**Ratio of PRs = 1.00 (0.92, 1.09)**

| Country | Trial | Male LNS N | Male Control N | Male Control Prevalence | Male PR (95% CI) | Male Fixed W | Male Random W | Female LNS N | Female Control N | Female Control Prevalence | Female PR (95% CI) | Female Fixed W | Female Random W |
|---|---|---|---|---|---|---|---|---|---|---|---|---|---|
| Bangladesh | JiVitA−4 (21) | 1532 | 659 | 47.3 | 0.99 (0.91, 1.09) | 0.49 | 0.26 | 1482 | 704 | 44.7 | 1.11 (1.02, 1.20) | 0.49 | 0.12 |
| Bangladesh | RDNS (22) | 819 | 396 | 74.5 | 1.17 (0.94, 1.45) | 0.08 | 0.12 | 801 | 394 | 74.4 | 1.40 (1.11, 1.76) | 0.06 | 0.11 |
| Bangladesh | WASH−B (23) | 232 | 692 | 75.9 | 1.27 (1.02, 1.58) | 0.08 | 0.11 | 265 | 782 | 73.4 | 1.15 (0.94, 1.41) | 0.08 | 0.11 |
| Burkina Faso | iLiNS−Zinc (24) | | | | | | | | | | | | |
| Ghana | GHANA (25) | 55 | 40 | 67.5 | 1.29 (0.75, 2.22) | 0.01 | 0.03 | 35 | 47 | 80.9 | 2.24 (1.11, 4.51) | 0.01 | 0.05 |
| Ghana | iLiNS−DYAD−G (26) | 166 | 321 | 53.6 | 1.16 (0.96, 1.39) | 0.12 | 0.14 | 160 | 341 | 52.8 | 1.14 (0.95, 1.37) | 0.10 | 0.12 |
| Haiti | HAITI (27) | 38 | 29 | 69.0 | 1.19 (0.60, 2.35) | 0.01 | 0.02 | 45 | 50 | 60.0 | 1.33 (0.86, 2.06) | 0.02 | 0.08 |
| Kenya | WASH−B (28) | 231 | 764 | 62.7 | 0.97 (0.78, 1.21) | 0.08 | 0.12 | 216 | 840 | 57.0 | 0.97 (0.81, 1.15) | 0.11 | 0.12 |
| Madagascar | MAHAY (29) | | | | | | | | | | | | |
| Malawi | iLiNS−DYAD−M (30) | 105 | 198 | 57.6 | 1.48 (1.19, 1.84) | 0.08 | 0.12 | 103 | 228 | 45.6 | 1.00 (0.81, 1.24) | 0.07 | 0.11 |
| Malawi | iLiNS−DOSE (31) | 310 | 111 | 73.9 | 1.40 (0.99, 1.97) | 0.03 | 0.06 | 292 | 89 | 55.1 | 0.79 (0.60, 1.05) | 0.04 | 0.10 |
| Mali | PROMIS CS (32) | 58 | 83 | 71.1 | 1.07 (0.63, 1.83) | 0.01 | 0.03 | 60 | 68 | 60.3 | 0.42 (0.23, 0.75) | 0.01 | 0.07 |
| Zimbabwe | SHINE (HIV−) (33) | | | | | | | | | | | | |
| Zimbabwe | SHINE (HIV+) (34) | | | | | | | | | | | | |
| | | **3546** | **3293** | | **I² = 0.49, Tau² = 0.01** | | | **3459** | **3543** | | **I² = 0.67, Tau² = 0.10** | | |
| **Fixed** | | | | | **1.10 (1.03, 1.17)** | | | | | | **1.09 (1.03, 1.15)** | | |
| **Random** | | | | | **1.15 (1.05, 1.26)** | | | | | | **1.06 (0.86, 1.33)** | | |

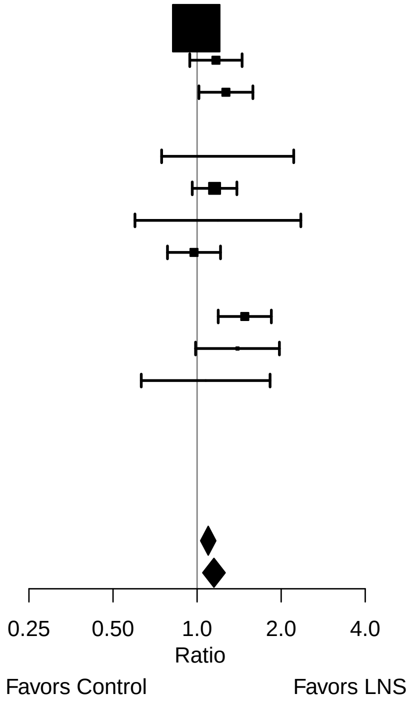


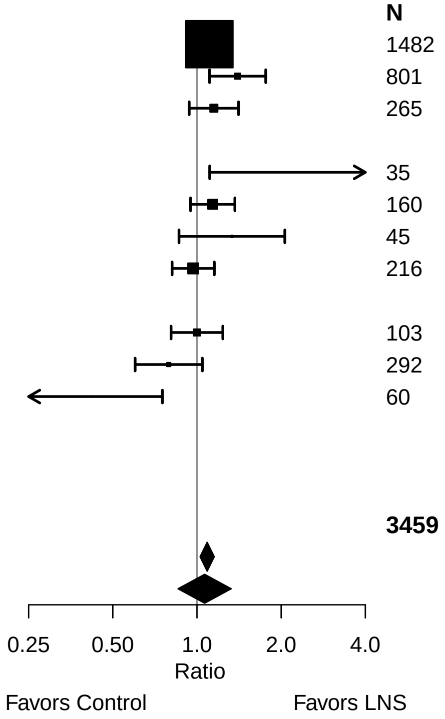

## Supplemental figure 7O: 12-mo walking without support prevalence ratio

# 7O7: Stratified by Child birth order

**P−for−interaction = 0.025**
**Ratio of PRs = 0.90 (0.81, 0.99)**

| | | Later born | | | | | | Firstborn | | | | | |
|---|---|---|---|---|---|---|---|---|---|---|---|---|---|
| **Country** | **Trial** | **LNS N** | **Control N** | **Control Prevalence** | **PR (95% CI)** | **Fixed W** | **Random W** | **LNS N** | **Control N** | **Control Prevalence** | **PR (95% CI)** | **Fixed W** | **Random W** |
| Bangladesh | JiVitA−4 (21) | 643 | 275 | 49.5 | 1.14 (1.00, 1.29) | 0.24 | 0.16 | 2363 | 1081 | 45.1 | 1.03 (0.96, 1.10) | 0.69 | 0.21 |
| Bangladesh | RDNS (22) | 953 | 496 | 75.6 | 1.38 (1.13, 1.67) | 0.10 | 0.12 | 673 | 294 | 72.4 | 1.14 (0.90, 1.45) | 0.06 | 0.13 |
| Bangladesh | WASH−B (23) | 314 | 944 | 74.8 | 1.15 (0.94, 1.40) | 0.10 | 0.12 | 157 | 440 | 74.1 | 1.38 (1.09, 1.74) | 0.06 | 0.13 |
| Burkina Faso | iLiNS−Zinc (24) | | | | | | | | | | | | |
| Ghana | GHANA (25) | 54 | 47 | 83.0 | 2.07 (1.00, 4.28) | 0.01 | 0.02 | 36 | 36 | 69.4 | 1.73 (0.97, 3.09) | 0.01 | 0.04 |
| Ghana | iLiNS−DYAD−G (26) | 222 | 439 | 54.9 | 1.21 (1.03, 1.42) | 0.16 | 0.14 | 105 | 224 | 49.6 | 1.04 (0.83, 1.30) | 0.07 | 0.14 |
| Haiti | HAITI (27) | 57 | 50 | 68.0 | 1.43 (0.87, 2.33) | 0.02 | 0.04 | 26 | 29 | 55.2 | 1.03 (0.58, 1.84) | 0.01 | 0.04 |
| Kenya | WASH−B (28) | 338 | 1296 | 60.2 | 1.02 (0.88, 1.18) | 0.18 | 0.15 | 108 | 306 | 58.2 | 0.80 (0.60, 1.05) | 0.05 | 0.12 |
| Madagascar | MAHAY (29) | | | | | | | | | | | | |
| Malawi | iLiNS−DYAD−M (30) | 166 | 340 | 50.6 | 1.17 (0.99, 1.39) | 0.14 | 0.13 | 42 | 84 | 52.4 | 1.30 (0.94, 1.80) | 0.03 | 0.10 |
| Malawi | iLiNS−DOSE (31) | 388 | 139 | 67.6 | 1.20 (0.92, 1.58) | 0.05 | 0.09 | 124 | 33 | 57.6 | 0.72 (0.45, 1.16) | 0.02 | 0.06 |
| Mali | PROMIS CS (32) | 103 | 134 | 65.7 | 0.65 (0.39, 1.08) | 0.02 | 0.04 | 14 | 14 | 64.3 | 1.00 (0.39, 2.56) | 0.00 | 0.02 |
| Zimbabwe | SHINE (HIV−) (33) | | | | | | | | | | | | |
| Zimbabwe | SHINE (HIV+) (34) | | | | | | | | | | | | |
| | | **3238** | **4160** | | **I² = 0.37, Tau² = 0.01** | | | **3648** | **2541** | | **I² = 0.46, Tau² = 0.02** | | |
| **Fixed** | | | | | **1.16 (1.09, 1.23)** | | | | | | **1.05 (0.99, 1.11)** | | |
| **Random** | | | | | **1.17 (1.05, 1.30)** | | | | | | **1.08 (0.94, 1.24)** | | |

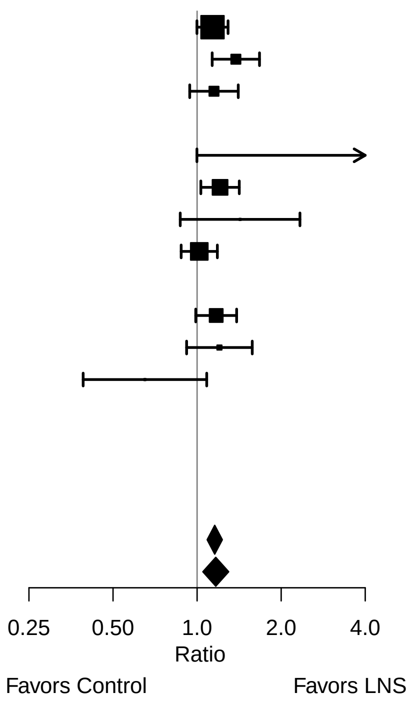


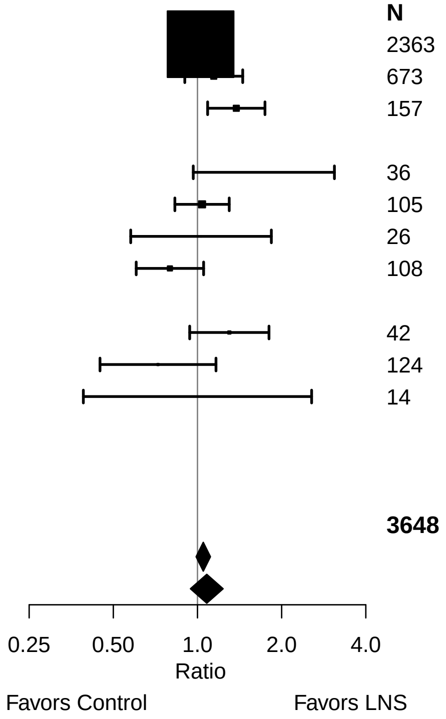

**Supplemental figure 7O: 12-mo walking without support prevalence ratio**

## 7O8: Stratified by Child baseline stunting

**P−for−interaction = 0.624**
**Ratio of PRs = 1.03 (0.90, 1.18)**

| | | No | | | | | | Yes | | | | | |
|---|---|---|---|---|---|---|---|---|---|---|---|---|---|
| **Country** | **Trial** | **LNS N** | **Control N** | **Control Prevalence** | **PR (95% CI)** | **Fixed W** | **Random W** | **LNS N** | **Control N** | **Control Prevalence** | **PR (95% CI)** | **Fixed W** | **Random W** |
| Bangladesh | JiVitA−4 (21) | 2241 | 1036 | 43.9 | 1.06 (0.99, 1.13) | 0.60 | 0.32 | 731 | 298 | 53.7 | 1.05 (0.91, 1.21) | 0.74 | 0.66 |
| Bangladesh | RDNS (22) | 1219 | 559 | 72.1 | 1.23 (1.02, 1.47) | 0.09 | 0.16 | 344 | 179 | 80.4 | 1.46 (0.96, 2.22) | 0.09 | 0.11 |
| Bangladesh | WASH−B (23) | | | | | | | | | | | | |
| Burkina Faso | iLiNS−Zinc (24) | | | | | | | | | | | | |
| Ghana | GHANA (25) | | | | | | | | | | | | |
| Ghana | iLiNS−DYAD−G (26) | 277 | 557 | 52.1 | 1.14 (1.00, 1.31) | 0.15 | 0.21 | 28 | 62 | 69.4 | 1.17 (0.63, 2.17) | 0.04 | 0.05 |
| Haiti | HAITI (27) | | | | | | | | | | | | |
| Kenya | WASH−B (28) | | | | | | | | | | | | |
| Madagascar | MAHAY (29) | | | | | | | | | | | | |
| Malawi | iLiNS−DYAD−M (30) | 148 | 317 | 47.6 | 1.21 (1.03, 1.42) | 0.11 | 0.18 | 50 | 93 | 63.4 | 1.09 (0.71, 1.69) | 0.08 | 0.11 |
| Malawi | iLiNS−DOSE (31) | 424 | 133 | 57.1 | 0.90 (0.71, 1.13) | 0.05 | 0.12 | 178 | 67 | 82.1 | 1.69 (0.97, 2.96) | 0.05 | 0.07 |
| Mali | PROMIS CS (32) | | | | | | | | | | | | |
| Zimbabwe | SHINE (HIV−) (33) | | | | | | | | | | | | |
| Zimbabwe | SHINE (HIV+) (34) | | | | | | | | | | | | |
| | | **4309** | **2602** | | **I² = 0.45, Tau² = 0.01** | | | **1331** | **699** | | **I² = 0.11, Tau² = 0.00** | | |
| **Fixed** | | | | | **1.09 (1.03, 1.15)** | | | | | | **1.11 (0.98, 1.26)** | | |
| **Random** | | | | | **1.11 (1.01, 1.22)** | | | | | | **1.13 (0.98, 1.31)** | | |

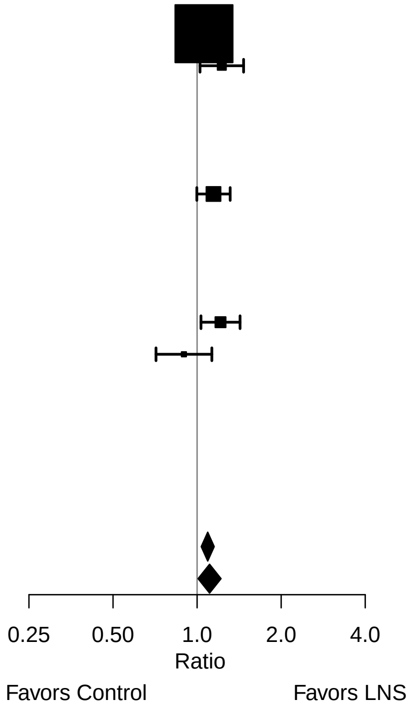


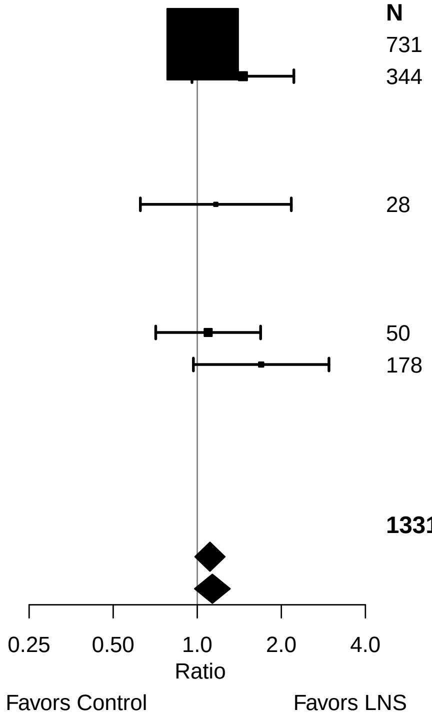

## Supplemental figure 7O: 12-mo walking without support prevalence ratio

## 7O9: Stratified by Child baseline acute malnutrition

P−for−interaction = 0.133
Ratio of PRs = 1.13 (0.96, 1.31)

| Country | Trial | No: LNS N | No: Control N | No: Control Prevalence | No: PR (95% CI) | No: Fixed W | No: Random W | Yes: LNS N | Yes: Control N | Yes: Control Prevalence | Yes: PR (95% CI) | Yes: Fixed W | Yes: Random W |
|---|---|---|---|---|---|---|---|---|---|---|---|---|---|
| Bangladesh | JiVitA−4 (21) | 2423 | 1084 | 44.6 | 1.05 (0.98, 1.12) | 0.65 | 0.47 | 558 | 255 | 53.7 | 1.10 (0.94, 1.29) | 0.87 | 0.38 |
| Bangladesh | RDNS (22) | 1447 | 689 | 73.0 | 1.26 (1.05, 1.51) | 0.09 | 0.14 | 119 | 50 | 90.0 | 1.93 (0.84, 4.45) | 0.03 | 0.18 |
| Bangladesh | WASH−B (23) | | | | | | | | | | | | |
| Burkina Faso | iLiNS−Zinc (24) | | | | | | | | | | | | |
| Ghana | GHANA (25) | | | | | | | | | | | | |
| Ghana | iLiNS−DYAD−G (26) | 284 | 564 | 50.7 | 1.09 (0.95, 1.25) | 0.15 | 0.22 | 23 | 57 | 84.2 | 3.30 (1.61, 6.76) | 0.04 | 0.21 |
| Haiti | HAITI (27) | | | | | | | | | | | | |
| Kenya | WASH−B (28) | | | | | | | | | | | | |
| Madagascar | MAHAY (29) | | | | | | | | | | | | |
| Malawi | iLiNS−DYAD−M (30) | 184 | 374 | 50.3 | 1.17 (1.00, 1.37) | 0.11 | 0.17 | 14 | 39 | 61.5 | 1.30 (0.67, 2.51) | 0.05 | 0.23 |
| Malawi | iLiNS−DOSE (31) | | | | | | | | | | | | |
| Mali | PROMIS CS (32) | | | | | | | | | | | | |
| Zimbabwe | SHINE (HIV−) (33) | | | | | | | | | | | | |
| Zimbabwe | SHINE (HIV+) (34) | | | | | | | | | | | | |
| | | **4338** | **2711** | | **I² = 0.36, Tau² = 0.00** | | | **714** | **401** | | **I² = 0.70, Tau² = 0.15** | | |
| **Fixed** | | | | | **1.08 (1.03, 1.14)** | | | | | | **1.19 (1.02, 1.38)** | | |
| **Random** | | | | | **1.10 (1.02, 1.19)** | | | | | | **1.60 (0.99, 2.58)** | | |

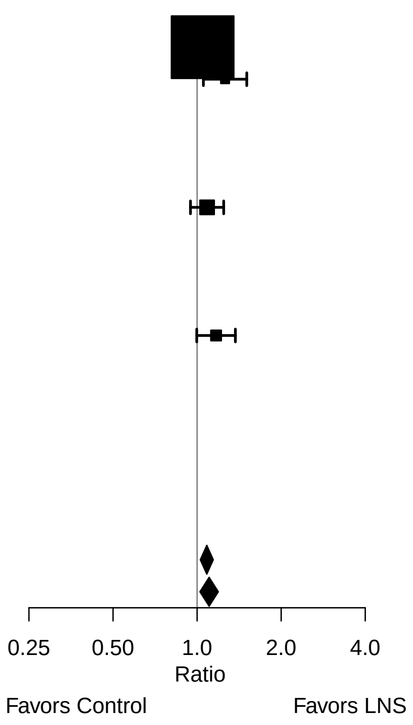


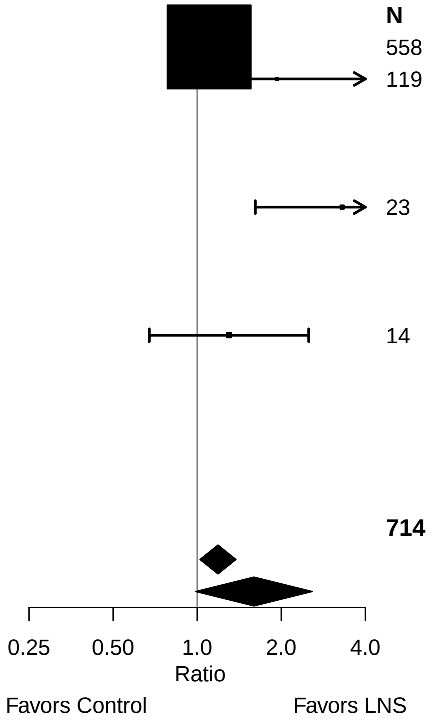

## Supplemental figure 7O: 12-mo walking without support prevalence ratio

## 7O10: Stratified by Child baseline anemia

**P−for−interaction = 0.526**
**Ratio of PRs = 0.94 (0.78, 1.14)**

| Country | Trial | LNS N | Control N | Control Prevalence | Not anemic PR (95% CI) | Fixed W | Random W | LNS N | Control N | Control Prevalence | Anemic PR (95% CI) | Fixed W | Random W |
|---|---|---|---|---|---|---|---|---|---|---|---|---|---|
| Bangladesh | JiVitA−4 (21) | | | | | | | | | | | | |
| Bangladesh | RDNS (22) | 211 | 95 | 71.6 | 1.20 (0.87, 1.65) | 0.14 | 0.14 | 310 | 157 | 74.5 | 1.20 (0.84, 1.72) | 0.13 | 0.16 |
| Bangladesh | WASH−B (23) | | | | | | | | | | | | |
| Burkina Faso | iLiNS−Zinc (24) | | | | | | | | | | | | |
| Ghana | GHANA (25) | | | | | | | | | | | | |
| Ghana | iLiNS−DYAD−G (26) | 185 | 358 | 51.4 | 1.22 (1.04, 1.44) | 0.55 | 0.55 | 91 | 201 | 55.2 | 0.93 (0.70, 1.24) | 0.20 | 0.23 |
| Haiti | HAITI (27) | | | | | | | | | | | | |
| Kenya | WASH−B (28) | | | | | | | | | | | | |
| Madagascar | MAHAY (29) | | | | | | | | | | | | |
| Malawi | iLiNS−DYAD−M (30) | 63 | 143 | 45.5 | 1.05 (0.81, 1.36) | 0.20 | 0.20 | 135 | 266 | 54.1 | 1.28 (1.05, 1.55) | 0.45 | 0.38 |
| Malawi | iLiNS−DOSE (31) | 205 | 84 | 67.9 | 1.20 (0.84, 1.71) | 0.11 | 0.11 | 393 | 116 | 63.8 | 0.96 (0.73, 1.27) | 0.22 | 0.24 |
| Mali | PROMIS CS (32) | | | | | | | | | | | | |
| Zimbabwe | SHINE (HIV−) (33) | | | | | | | | | | | | |
| Zimbabwe | SHINE (HIV+) (34) | | | | | | | | | | | | |
| | | **664** | **680** | | **I² = 0.00, Tau² = 0.00** | | | **929** | **740** | | **I² = 0.35, Tau² = 0.01** | | |
| **Fixed** | | | | | **1.18 (1.05, 1.33)** | | | | | | **1.12 (0.98, 1.27)** | | |
| **Random** | | | | | **1.18 (1.05, 1.33)** | | | | | | **1.10 (0.94, 1.29)** | | |

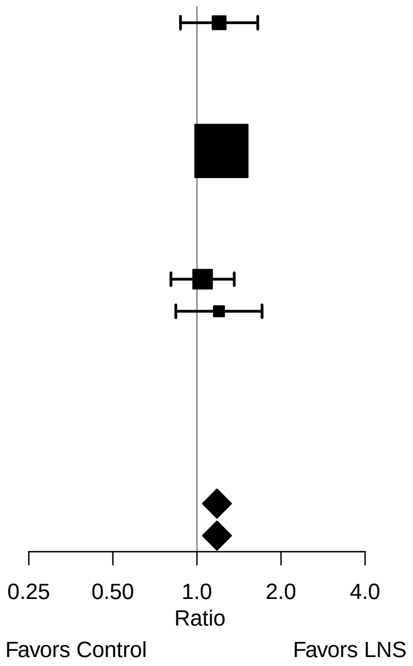


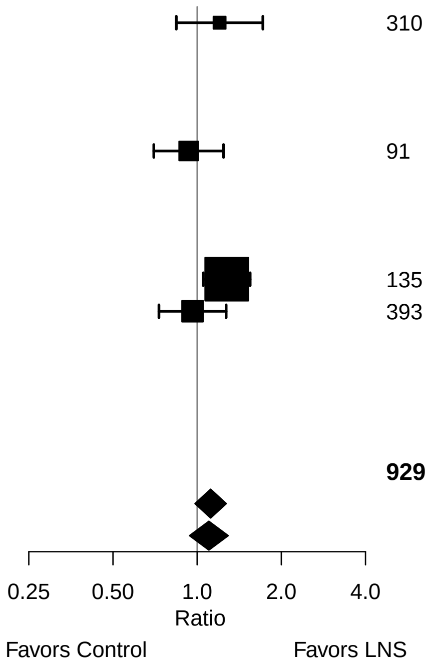

# Supplemental figure 7P: 12-mo walking without support prevalence difference

## 7P1: Stratified by Maternal height

**P−for−interaction = 0.192**
**Difference in PDs = −0.04 (−0.09, 0.02)**

| | | At least 150.1 cm | | | | | | Less than 150.1 cm | | | | | |
|---|---|---|---|---|---|---|---|---|---|---|---|---|---|
| **Country** | **Trial** | **LNS N** | **Control N** | **Control Prevalence** | **PD (95% CI)** | **Fixed W** | **Random W** | **LNS N** | **Control N** | **Control Prevalence** | **PD (95% CI)** | **Fixed W** | **Random W** |
| Bangladesh | JiVitA−4 (21) | | | | | | | | | | | | |
| Bangladesh | RDNS (22) | 848 | 418 | 74.4 | 0.11 (0.05, 0.16) | 0.28 | 0.28 | 721 | 347 | 76.4 | 0.04 (−0.03, 0.11) | 0.38 | 0.38 |
| Bangladesh | WASH−B (23) | 261 | 782 | 73.0 | 0.06 (0.00, 0.12) | 0.26 | 0.26 | 230 | 666 | 76.1 | 0.05 (−0.01, 0.11) | 0.51 | 0.51 |
| Burkina Faso | iLiNS−Zinc (24) | | | | | | | | | | | | |
| Ghana | GHANA (25) | | | | | | | | | | | | |
| Ghana | iLiNS−DYAD−G (26) | 308 | 613 | 52.9 | 0.06 (−0.01, 0.13) | 0.20 | 0.20 | 14 | 38 | 63.2 | 0.27 (−0.03, 0.58) | 0.02 | 0.02 |
| Haiti | HAITI (27) | | | | | | | | | | | | |
| Kenya | WASH−B (28) | | | | | | | | | | | | |
| Madagascar | MAHAY (29) | | | | | | | | | | | | |
| Malawi | iLiNS−DYAD−M (30) | 180 | 367 | 50.4 | 0.10 (0.01, 0.19) | 0.12 | 0.12 | 28 | 56 | 53.6 | 0.07 (−0.16, 0.30) | 0.04 | 0.04 |
| Malawi | iLiNS−DOSE (31) | 505 | 164 | 66.5 | 0.03 (−0.05, 0.12) | 0.13 | 0.13 | 96 | 35 | 62.9 | −0.06 (−0.24, 0.12) | 0.06 | 0.06 |
| Mali | PROMIS CS (32) | | | | | | | | | | | | |
| Zimbabwe | SHINE (HIV−) (33) | | | | | | | | | | | | |
| Zimbabwe | SHINE (HIV+) (34) | | | | | | | | | | | | |
| | | **2102** | **2344** | | **$I^2$ = 0.00, $Tau^2$ = 0.00** | | | **1089** | **1142** | | **$I^2$ = 0.00, $Tau^2$ = 0.00** | | |
| **Fixed** | | | | | **0.07 (0.04, 0.10)** | | | | | | **0.05 (0.00, 0.09)** | | |
| **Random** | | | | | **0.07 (0.04, 0.10)** | | | | | | **0.05 (0.00, 0.09)** | | |

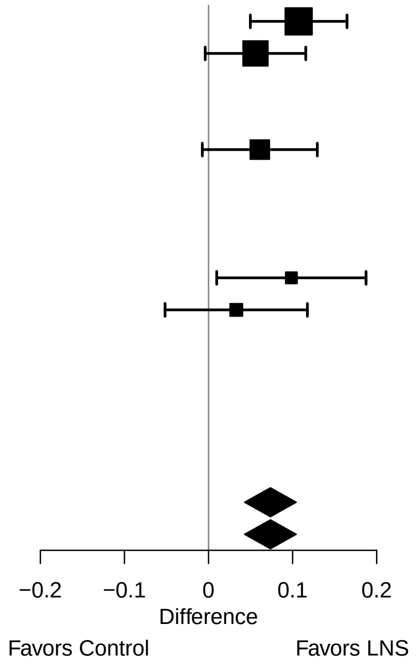


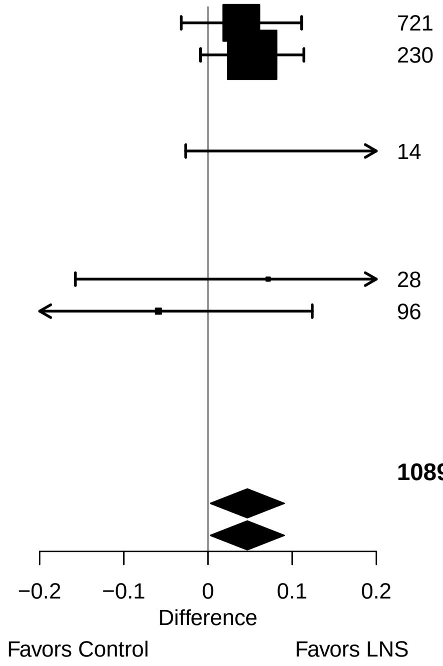

## Supplemental figure 7P: 12-mo walking without support prevalence difference

## 7P2: Stratified by Maternal BMI

**P−for−interaction = 0.058**
**Difference in PDs = −0.05 (−0.09, 0.00)**

| Country | Trial | At least 20 kg/m²: LNS N | Control N | Control Prevalence | PD (95% CI) | Fixed W | Random W | Less than 20 kg/m²: LNS N | Control N | Control Prevalence | PD (95% CI) | Fixed W | Random W |
|---|---|---|---|---|---|---|---|---|---|---|---|---|---|
| Bangladesh | JiVitA−4 (21) | | | | | | | | | | | | |
| Bangladesh | RDNS (22) | 708 | 327 | 74.6 | 0.14 (0.07, 0.22) | 0.15 | 0.15 | 861 | 438 | 75.8 | 0.02 (−0.03, 0.07) | 0.41 | 0.41 |
| Bangladesh | WASH−B (23) | 225 | 637 | 73.0 | 0.08 (0.01, 0.15) | 0.20 | 0.16 | 266 | 810 | 75.7 | 0.03 (−0.02, 0.09) | 0.33 | 0.33 |
| Burkina Faso | iLiNS−Zinc (24) | | | | | | | | | | | | |
| Ghana | GHANA (25) | | | | | | | | | | | | |
| Ghana | iLiNS−DYAD−G (26) | 284 | 535 | 52.3 | 0.07 (0.00, 0.14) | 0.17 | 0.16 | 38 | 116 | 58.6 | 0.03 (−0.15, 0.22) | 0.03 | 0.03 |
| Haiti | HAITI (27) | | | | | | | | | | | | |
| Kenya | WASH−B (28) | 334 | 1210 | 58.8 | −0.03 (−0.09, 0.02) | 0.26 | 0.17 | 96 | 364 | 61.5 | 0.01 (−0.10, 0.13) | 0.08 | 0.08 |
| Madagascar | MAHAY (29) | | | | | | | | | | | | |
| Malawi | iLiNS−DYAD−M (30) | 121 | 255 | 51.4 | 0.08 (−0.02, 0.19) | 0.07 | 0.12 | 86 | 168 | 50.0 | 0.12 (−0.01, 0.25) | 0.06 | 0.06 |
| Malawi | iLiNS−DOSE (31) | 451 | 138 | 63.0 | 0.00 (−0.09, 0.09) | 0.10 | 0.14 | 148 | 60 | 71.7 | 0.05 (−0.09, 0.20) | 0.05 | 0.05 |
| Mali | PROMIS CS (32) | 83 | 98 | 66.3 | −0.11 (−0.25, 0.03) | 0.04 | 0.10 | 34 | 52 | 67.3 | −0.06 (−0.26, 0.13) | 0.03 | 0.03 |
| Zimbabwe | SHINE (HIV−) (33) | | | | | | | | | | | | |
| Zimbabwe | SHINE (HIV+) (34) | | | | | | | | | | | | |
| | | **2206** | **3200** | | **I² = 0.72, Tau² = 0.00** | | | **1529** | **2008** | | **I² = 0.00, Tau² = 0.00** | | |
| **Fixed** | | | | | **0.04 (0.01, 0.07)** | | | | | | **0.03 (0.00, 0.06)** | | |
| **Random** | | | | | **0.04 (−0.02, 0.10)** | | | | | | **0.03 (0.00, 0.06)** | | |

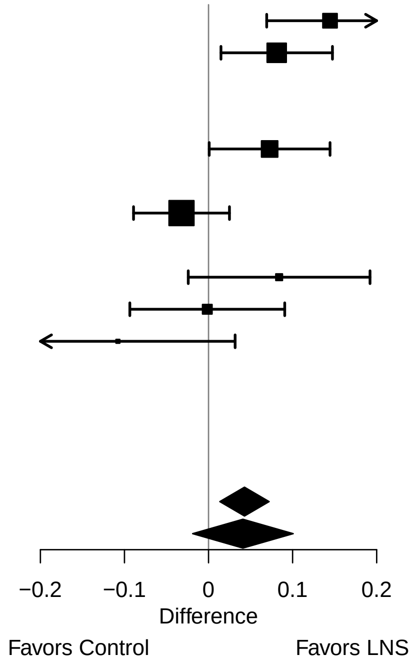


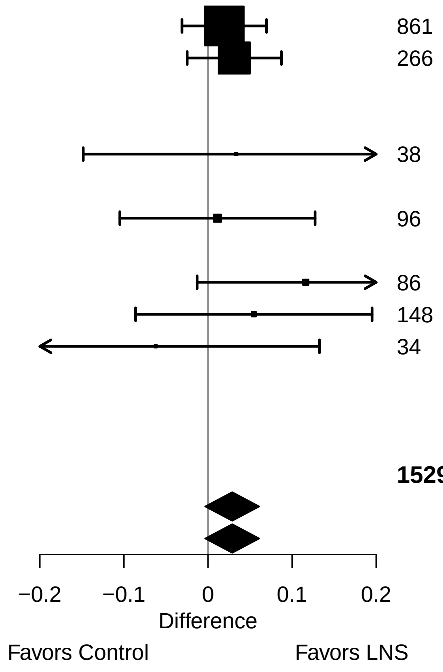

## Supplemental figure 7P: 12-mo walking without support prevalence difference

### 7P3: Stratified by Maternal age

**P−for−interaction = 0.315**
**Difference in PDs = 0.02 (−0.02, 0.06)**

**At least 25 y**

| Country | Trial | LNS N | Control N | Control Prevalence | PD (95% CI) | Fixed W | Random W |
|---|---|---|---|---|---|---|---|
| Bangladesh | JiVitA−4 (21) | 1273 | 587 | 47.5 | 0.03 (−0.02, 0.08) | 0.28 | 0.17 |
| Bangladesh | RDNS (22) | 447 | 217 | 76.0 | 0.09 (0.02, 0.16) | 0.14 | 0.14 |
| Bangladesh | WASH−B (23) | 223 | 676 | 74.6 | −0.01 (−0.07, 0.05) | 0.18 | 0.15 |
| Burkina Faso | iLiNS−Zinc (24) | | | | | | |
| Ghana | GHANA (25) | | | | | | |
| Ghana | iLiNS−DYAD−G (26) | 209 | 405 | 53.8 | 0.04 (−0.05, 0.12) | 0.10 | 0.12 |
| Haiti | HAITI (27) | 60 | 57 | 68.4 | 0.18 (0.01, 0.36) | 0.02 | 0.05 |
| Kenya | WASH−B (28) | 243 | 914 | 61.3 | 0.01 (−0.06, 0.08) | 0.15 | 0.14 |
| Madagascar | MAHAY (29) | | | | | | |
| Malawi | iLiNS−DYAD−M (30) | 100 | 213 | 47.9 | 0.03 (−0.09, 0.15) | 0.05 | 0.08 |
| Malawi | iLiNS−DOSE (31) | 327 | 111 | 64.9 | 0.04 (−0.07, 0.14) | 0.06 | 0.09 |
| Mali | PROMIS CS (32) | 59 | 71 | 62.0 | −0.16 (−0.32, 0.00) | 0.03 | 0.05 |
| Zimbabwe | SHINE (HIV−) (33) | | | | | | |
| Zimbabwe | SHINE (HIV+) (34) | | | | | | |
| | | **2941** | **3251** | | **I² = 0.38, Tau² = 0.00** | | |
| **Fixed** | | | | | **0.03 (0.00, 0.05)** | | |
| **Random** | | | | | **0.03 (−0.02, 0.07)** | | |

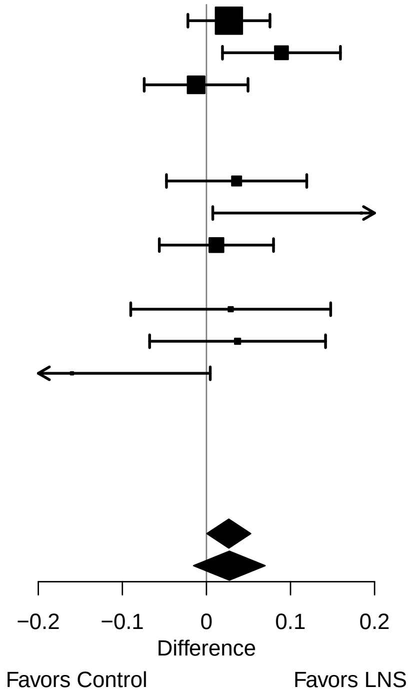


**Less than 25 y**

| Country | Trial | LNS N | Control N | Control Prevalence | PD (95% CI) | Fixed W | Random W |
|---|---|---|---|---|---|---|---|
| Bangladesh | JiVitA−4 (21) | 1726 | 769 | 45.0 | 0.03 (−0.02, 0.07) | 0.31 | 0.17 |
| Bangladesh | RDNS (22) | 1181 | 573 | 73.8 | 0.07 (0.01, 0.12) | 0.19 | 0.15 |
| Bangladesh | WASH−B (23) | 274 | 791 | 74.6 | 0.10 (0.05, 0.16) | 0.21 | 0.16 |
| Burkina Faso | iLiNS−Zinc (24) | | | | | | |
| Ghana | GHANA (25) | | | | | | |
| Ghana | iLiNS−DYAD−G (26) | 118 | 258 | 51.9 | 0.13 (0.02, 0.24) | 0.05 | 0.10 |
| Haiti | HAITI (27) | 22 | 22 | 50.0 | −0.14 (−0.43, 0.16) | 0.01 | 0.03 |
| Kenya | WASH−B (28) | 197 | 679 | 57.7 | −0.04 (−0.11, 0.04) | 0.12 | 0.14 |
| Madagascar | MAHAY (29) | | | | | | |
| Malawi | iLiNS−DYAD−M (30) | 108 | 213 | 54.5 | 0.16 (0.05, 0.28) | 0.05 | 0.10 |
| Malawi | iLiNS−DOSE (31) | 264 | 83 | 67.5 | 0.00 (−0.12, 0.11) | 0.05 | 0.10 |
| Mali | PROMIS CS (32) | 59 | 80 | 70.0 | −0.05 (−0.19, 0.10) | 0.03 | 0.07 |
| Zimbabwe | SHINE (HIV−) (33) | | | | | | |
| Zimbabwe | SHINE (HIV+) (34) | | | | | | |
| | | **3949** | **3468** | | **I² = 0.61, Tau² = 0.00** | | |
| **Fixed** | | | | | **0.05 (0.02, 0.07)** | | |
| **Random** | | | | | **0.05 (0.00, 0.10)** | | |

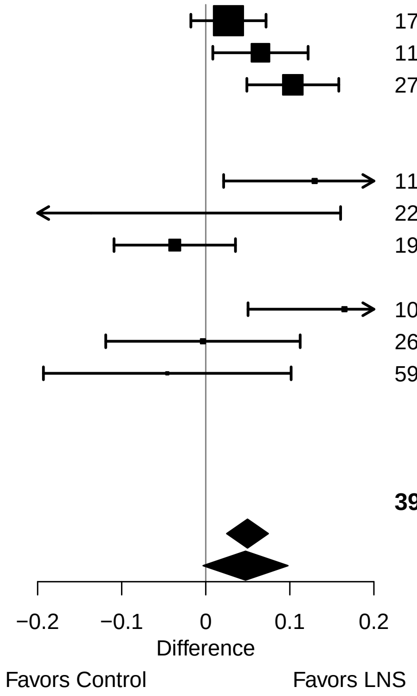

## Supplemental figure 7P: 12-mo walking without support prevalence difference

# 7P4: Stratified by Maternal education

**P−for−interaction = 0.291**
**Difference in PDs = −0.02 (−0.06, 0.02)**

| | | Primary or greater | | | | | | Incomplete or no formal | | | | | |
|---|---|---|---|---|---|---|---|---|---|---|---|---|---|
| **Country** | **Trial** | **LNS N** | **Control N** | **Control Prevalence** | **PD (95% CI)** | **Fixed W** | **Random W** | **LNS N** | **Control N** | **Control Prevalence** | **PD (95% CI)** | **Fixed W** | **Random W** |
| Bangladesh | JiVitA−4 (21) | 1933 | 848 | 43.8 | 0.02 (−0.02, 0.06) | 0.33 | 0.33 | 1075 | 512 | 50.0 | 0.04 (−0.01, 0.09) | 0.28 | 0.22 |
| Bangladesh | RDNS (22) | 1219 | 574 | 73.9 | 0.08 (0.03, 0.14) | 0.18 | 0.18 | 409 | 216 | 75.9 | 0.03 (−0.02, 0.09) | 0.21 | 0.19 |
| Bangladesh | WASH−B (23) | 337 | 1048 | 72.7 | 0.08 (0.03, 0.13) | 0.25 | 0.25 | 160 | 426 | 79.1 | 0.00 (−0.06, 0.07) | 0.18 | 0.17 |
| Burkina Faso | iLiNS−Zinc (24) | | | | | | | | | | | | |
| Ghana | GHANA (25) | | | | | | | | | | | | |
| Ghana | iLiNS−DYAD−G (26) | 257 | 524 | 51.5 | 0.06 (−0.01, 0.13) | 0.10 | 0.10 | 70 | 139 | 59.0 | 0.10 (−0.04, 0.25) | 0.03 | 0.05 |
| Haiti | HAITI (27) | | | | | | | | | | | | |
| Kenya | WASH−B (28) | 217 | 775 | 59.2 | 0.02 (−0.06, 0.09) | 0.10 | 0.10 | 229 | 827 | 60.3 | −0.04 (−0.12, 0.03) | 0.12 | 0.14 |
| Madagascar | MAHAY (29) | | | | | | | | | | | | |
| Malawi | iLiNS−DYAD−M (30) | 32 | 64 | 42.2 | −0.02 (−0.23, 0.20) | 0.01 | 0.01 | 175 | 359 | 52.4 | 0.11 (0.02, 0.20) | 0.09 | 0.11 |
| Malawi | iLiNS−DOSE (31) | 129 | 42 | 61.9 | 0.03 (−0.14, 0.20) | 0.02 | 0.02 | 462 | 152 | 67.1 | 0.02 (−0.07, 0.10) | 0.09 | 0.12 |
| Mali | PROMIS CS (32) | | | | | | | | | | | | |
| Zimbabwe | SHINE (HIV−) (33) | | | | | | | | | | | | |
| Zimbabwe | SHINE (HIV+) (34) | | | | | | | | | | | | |
| | | **4124** | **3875** | | **I² = 0.00, Tau² = 0.00** | | | **2580** | **2631** | | **I² = 0.30, Tau² = 0.00** | | |
| **Fixed** | | | | | **0.05 (0.03, 0.07)** | | | | | | **0.03 (0.00, 0.05)** | | |
| **Random** | | | | | **0.05 (0.03, 0.07)** | | | | | | **0.03 (−0.01, 0.06)** | | |

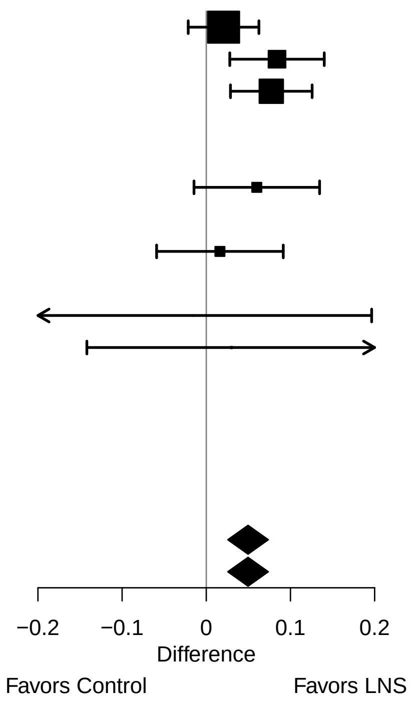


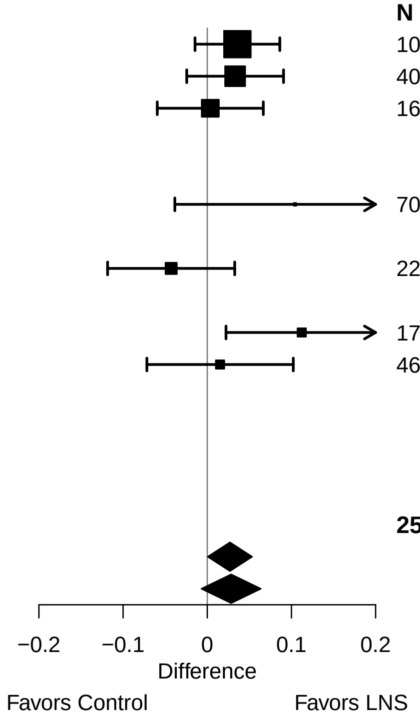

### Supplemental figure 7P: 12-mo walking without support prevalence difference

## 7P5: Stratified by Maternal depressive symptoms

**P−for−interaction = 0.606**
**Difference in PDs = −0.01 (−0.06, 0.04)**

| | | Less than 75th percentile | | | | | | At least 75th percentile | | | | | |
|---|---|---|---|---|---|---|---|---|---|---|---|---|---|
| **Country** | **Trial** | **LNS N** | **Control N** | **Control Prevalence** | **PD (95% CI)** | **Fixed W** | **Random W** | **LNS N** | **Control N** | **Control Prevalence** | **PD (95% CI)** | **Fixed W** | **Random W** |
| Bangladesh | JiVitA−4 (21) | | | | | | | | | | | | |
| Bangladesh | RDNS (22) | 1058 | 460 | 70.9 | 0.03 (−0.03, 0.10) | 0.20 | 0.21 | 508 | 279 | 79.6 | 0.14 (0.07, 0.20) | 0.42 | 0.29 |
| Bangladesh | WASH−B (23) | 389 | 1075 | 73.4 | 0.07 (0.02, 0.12) | 0.33 | 0.28 | 108 | 399 | 77.7 | −0.04 (−0.12, 0.05) | 0.26 | 0.25 |
| Burkina Faso | iLiNS−Zinc (24) | | | | | | | | | | | | |
| Ghana | GHANA (25) | | | | | | | | | | | | |
| Ghana | iLiNS−DYAD−G (26) | 242 | 446 | 52.9 | 0.07 (0.00, 0.15) | 0.14 | 0.16 | 75 | 198 | 55.1 | 0.07 (−0.06, 0.20) | 0.10 | 0.16 |
| Haiti | HAITI (27) | | | | | | | | | | | | |
| Kenya | WASH−B (28) | 314 | 1098 | 59.6 | −0.01 (−0.07, 0.05) | 0.24 | 0.23 | 99 | 373 | 61.1 | 0.01 (−0.10, 0.11) | 0.16 | 0.20 |
| Madagascar | MAHAY (29) | | | | | | | | | | | | |
| Malawi | iLiNS−DYAD−M (30) | 149 | 293 | 48.1 | 0.11 (0.01, 0.20) | 0.09 | 0.12 | 47 | 108 | 60.2 | 0.09 (−0.08, 0.26) | 0.06 | 0.11 |
| Malawi | iLiNS−DOSE (31) | | | | | | | | | | | | |
| Mali | PROMIS CS (32) | | | | | | | | | | | | |
| Zimbabwe | SHINE (HIV−) (33) | | | | | | | | | | | | |
| Zimbabwe | SHINE (HIV+) (34) | | | | | | | | | | | | |
| | | **2152** | **3372** | | **I² = 0.35, Tau² = 0.00** | | | **837** | **1357** | | **I² = 0.65, Tau² = 0.00** | | |
| **Fixed** | | | | | **0.05 (0.02, 0.08)** | | | | | | **0.06 (0.02, 0.10)** | | |
| **Random** | | | | | **0.05 (0.01, 0.09)** | | | | | | **0.05 (−0.01, 0.12)** | | |

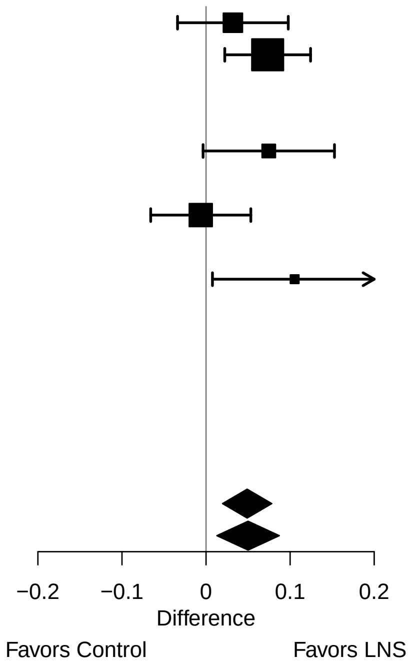


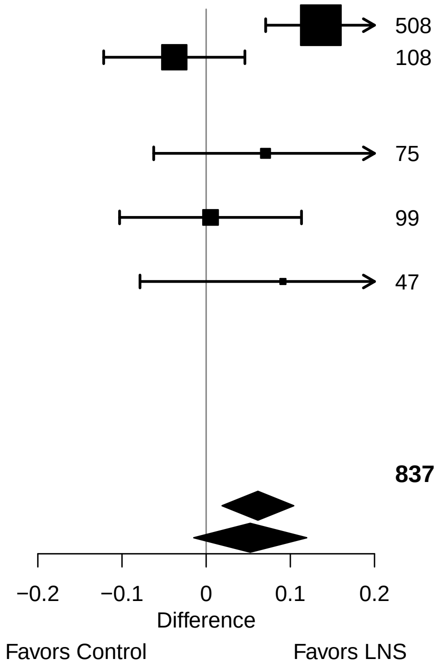

## Supplemental figure 7P: 12-mo walking without support prevalence difference

## 7P6: Stratified by Child sex

**P−for−interaction = 0.816**
**Difference in PDs = 0.00 (−0.03, 0.04)**

| Country | Trial | Male: LNS N | Male: Control N | Male: Control Prevalence | Male: PD (95% CI) | Male: Fixed W | Male: Random W | Female: LNS N | Female: Control N | Female: Control Prevalence | Female: PD (95% CI) | Female: Fixed W | Female: Random W |
|---|---|---|---|---|---|---|---|---|---|---|---|---|---|
| Bangladesh | JiVitA−4 (21) | 1532 | 659 | 47.3 | 0.00 (−0.05, 0.04) | 0.30 | 0.19 | 1482 | 704 | 44.7 | 0.06 (0.01, 0.11) | 0.29 | 0.12 |
| Bangladesh | RDNS (22) | 819 | 396 | 74.5 | 0.04 (−0.02, 0.10) | 0.20 | 0.17 | 801 | 394 | 74.4 | 0.10 (0.04, 0.17) | 0.16 | 0.12 |
| Bangladesh | WASH−B (23) | 232 | 692 | 75.9 | 0.06 (0.00, 0.13) | 0.16 | 0.15 | 265 | 782 | 73.4 | 0.04 (−0.02, 0.10) | 0.19 | 0.12 |
| Burkina Faso | iLiNS−Zinc (24) | | | | | | | | | | | | |
| Ghana | GHANA (25) | 55 | 40 | 67.5 | 0.09 (−0.11, 0.29) | 0.02 | 0.03 | 35 | 47 | 80.9 | 0.24 (0.04, 0.43) | 0.02 | 0.07 |
| Ghana | iLiNS−DYAD−G (26) | 166 | 321 | 53.6 | 0.07 (−0.02, 0.17) | 0.08 | 0.10 | 160 | 341 | 52.8 | 0.07 (−0.03, 0.16) | 0.07 | 0.11 |
| Haiti | HAITI (27) | 38 | 29 | 69.0 | 0.06 (−0.17, 0.29) | 0.01 | 0.02 | 45 | 50 | 60.0 | 0.13 (−0.07, 0.33) | 0.02 | 0.06 |
| Kenya | WASH−B (28) | 231 | 764 | 62.7 | −0.01 (−0.09, 0.07) | 0.10 | 0.12 | 216 | 840 | 57.0 | −0.01 (−0.09, 0.06) | 0.12 | 0.12 |
| Madagascar | MAHAY (29) | | | | | | | | | | | | |
| Malawi | iLiNS−DYAD−M (30) | 105 | 198 | 57.6 | 0.20 (0.09, 0.32) | 0.05 | 0.07 | 103 | 228 | 45.6 | 0.00 (−0.12, 0.12) | 0.05 | 0.10 |
| Malawi | iLiNS−DOSE (31) | 310 | 111 | 73.9 | 0.10 (0.00, 0.21) | 0.06 | 0.09 | 292 | 89 | 55.1 | −0.09 (−0.21, 0.02) | 0.05 | 0.10 |
| Mali | PROMIS CS (32) | 58 | 83 | 71.1 | 0.02 (−0.14, 0.18) | 0.03 | 0.05 | 60 | 68 | 60.3 | −0.23 (−0.38, −0.08) | 0.03 | 0.08 |
| Zimbabwe | SHINE (HIV−) (33) | | | | | | | | | | | | |
| Zimbabwe | SHINE (HIV+) (34) | | | | | | | | | | | | |
| | | **3546** | **3293** | | **I² = 0.41, Tau² = 0.00** | | | **3459** | **3543** | | **I² = 0.70, Tau² = 0.01** | | |
| **Fixed** | | | | | **0.04 (0.02, 0.07)** | | | | | | **0.04 (0.01, 0.06)** | | |
| **Random** | | | | | **0.05 (0.02, 0.09)** | | | | | | **0.03 (−0.05, 0.10)** | | |

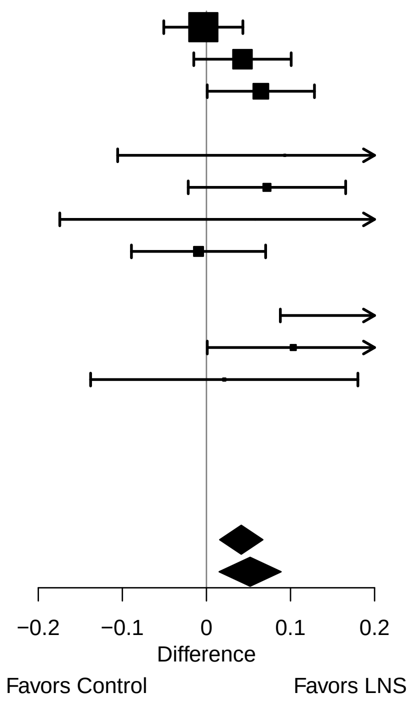


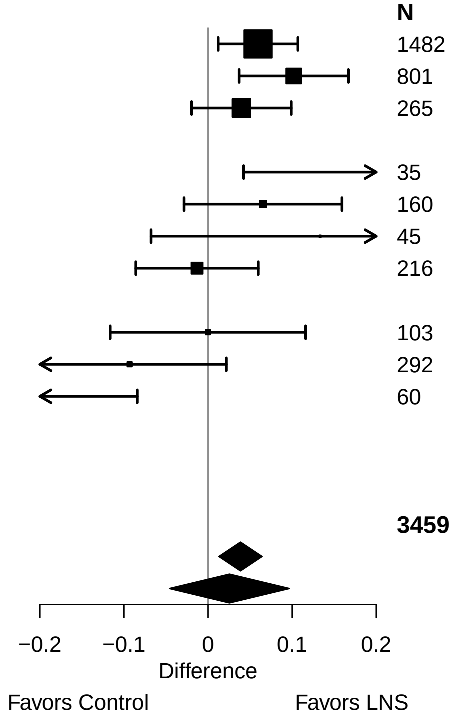

### Supplemental figure 7P: 12-mo walking without support prevalence difference

## 7P7: Stratified by Child birth order

**P−for−interaction = 0.055**
**Difference in PDs = −0.04 (−0.08, 0.00)**

| Country | Trial | LNS N | Control N | Control Prevalence | Later born PD (95% CI) | Fixed W | Random W | LNS N | Control N | Control Prevalence | Firstborn PD (95% CI) | Fixed W | Random W |
|---|---|---|---|---|---|---|---|---|---|---|---|---|---|
| Bangladesh | JiVitA−4 (21) | 643 | 275 | 49.5 | 0.07 (0.00, 0.14) | 0.13 | 0.13 | 2363 | 1081 | 45.1 | 0.01 (−0.03, 0.05) | 0.49 | 0.21 |
| Bangladesh | RDNS (22) | 953 | 496 | 75.6 | 0.09 (0.04, 0.14) | 0.21 | 0.15 | 673 | 294 | 72.4 | 0.04 (−0.03, 0.11) | 0.16 | 0.17 |
| Bangladesh | WASH−B (23) | 314 | 944 | 74.8 | 0.04 (−0.02, 0.09) | 0.19 | 0.14 | 157 | 440 | 74.1 | 0.10 (0.02, 0.17) | 0.13 | 0.16 |
| Burkina Faso | iLiNS−Zinc (24) | | | | | | | | | | | | |
| Ghana | GHANA (25) | 54 | 47 | 83.0 | 0.18 (0.01, 0.35) | 0.02 | 0.04 | 36 | 36 | 69.4 | 0.22 (0.00, 0.45) | 0.02 | 0.04 |
| Ghana | iLiNS−DYAD−G (26) | 222 | 439 | 54.9 | 0.09 (0.01, 0.17) | 0.09 | 0.11 | 105 | 224 | 49.6 | 0.02 (−0.10, 0.14) | 0.06 | 0.11 |
| Haiti | HAITI (27) | 57 | 50 | 68.0 | 0.14 (−0.05, 0.32) | 0.02 | 0.04 | 26 | 29 | 55.2 | 0.01 (−0.26, 0.28) | 0.01 | 0.03 |
| Kenya | WASH−B (28) | 338 | 1296 | 60.2 | 0.01 (−0.05, 0.07) | 0.16 | 0.14 | 108 | 306 | 58.2 | −0.08 (−0.18, 0.01) | 0.08 | 0.13 |
| Madagascar | MAHAY (29) | | | | | | | | | | | | |
| Malawi | iLiNS−DYAD−M (30) | 166 | 340 | 50.6 | 0.08 (−0.01, 0.18) | 0.07 | 0.10 | 42 | 84 | 52.4 | 0.14 (−0.04, 0.33) | 0.02 | 0.06 |
| Malawi | iLiNS−DOSE (31) | 388 | 139 | 67.6 | 0.07 (−0.03, 0.16) | 0.07 | 0.09 | 124 | 33 | 57.6 | −0.12 (−0.30, 0.06) | 0.02 | 0.06 |
| Mali | PROMIS CS (32) | 103 | 134 | 65.7 | −0.12 (−0.25, 0.01) | 0.03 | 0.06 | 14 | 14 | 64.3 | 0.00 (−0.34, 0.34) | 0.01 | 0.02 |
| Zimbabwe | SHINE (HIV−) (33) | | | | | | | | | | | | |
| Zimbabwe | SHINE (HIV+) (34) | | | | | | | | | | | | |
| | | **3238** | **4160** | | **I² = 0.42, Tau² = 0.00** | | | **3648** | **2541** | | **I² = 0.43, Tau² = 0.00** | | |
| **Fixed** | | | | | **0.06 (0.03, 0.08)** | | | | | | **0.02 (0.00, 0.05)** | | |
| **Random** | | | | | **0.06 (0.02, 0.10)** | | | | | | **0.03 (−0.02, 0.08)** | | |

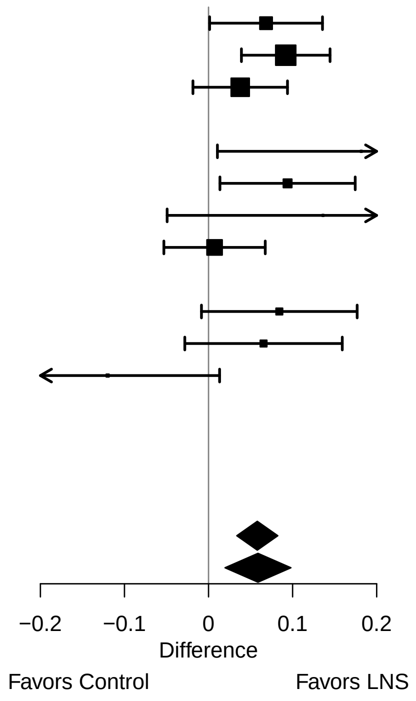


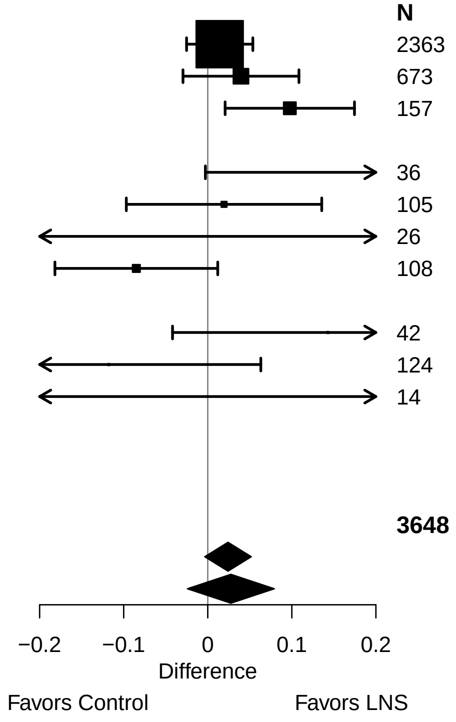

**Supplemental figure 7P: 12-mo walking without support prevalence difference**

## 7P8: Stratified by Child baseline stunting

**P−for−interaction = 0.517**
**Difference in PDs = 0.02 (−0.03, 0.07)**

| Country | Trial | No: LNS N | No: Control N | No: Control Prevalence | No: PD (95% CI) | No: Fixed W | No: Random W | Yes: LNS N | Yes: Control N | Yes: Control Prevalence | Yes: PD (95% CI) | Yes: Fixed W | Yes: Random W |
|---|---|---|---|---|---|---|---|---|---|---|---|---|---|
| Bangladesh | JiVitA−4 (21) | 2241 | 1036 | 43.9 | 0.03 (−0.01, 0.07) | 0.46 | 0.30 | 731 | 298 | 53.7 | 0.02 (−0.05, 0.09) | 0.47 | 0.47 |
| Bangladesh | RDNS (22) | 1219 | 559 | 72.1 | 0.06 (0.01, 0.12) | 0.25 | 0.25 | 344 | 179 | 80.4 | 0.09 (0.00, 0.18) | 0.27 | 0.27 |
| Bangladesh | WASH−B (23) | | | | | | | | | | | | |
| Burkina Faso | iLiNS−Zinc (24) | | | | | | | | | | | | |
| Ghana | GHANA (25) | | | | | | | | | | | | |
| Ghana | iLiNS−DYAD−G (26) | 277 | 557 | 52.1 | 0.07 (0.00, 0.14) | 0.14 | 0.19 | 28 | 62 | 69.4 | 0.05 (−0.16, 0.26) | 0.05 | 0.05 |
| Haiti | HAITI (27) | | | | | | | | | | | | |
| Kenya | WASH−B (28) | | | | | | | | | | | | |
| Madagascar | MAHAY (29) | | | | | | | | | | | | |
| Malawi | iLiNS−DYAD−M (30) | 148 | 317 | 47.6 | 0.11 (0.01, 0.21) | 0.08 | 0.13 | 50 | 93 | 63.4 | 0.03 (−0.13, 0.20) | 0.07 | 0.07 |
| Malawi | iLiNS−DOSE (31) | 424 | 133 | 57.1 | −0.04 (−0.14, 0.05) | 0.08 | 0.13 | 178 | 67 | 82.1 | 0.12 (0.00, 0.25) | 0.14 | 0.14 |
| Mali | PROMIS CS (32) | | | | | | | | | | | | |
| Zimbabwe | SHINE (HIV−) (33) | | | | | | | | | | | | |
| Zimbabwe | SHINE (HIV+) (34) | | | | | | | | | | | | |
| | | **4309** | **2602** | | **I² = 0.38, Tau² = 0.00** | | | **1331** | **699** | | **I² = 0.00, Tau² = 0.00** | | |
| **Fixed** | | | | | **0.05 (0.02, 0.07)** | | | | | | **0.06 (0.01, 0.10)** | | |
| **Random** | | | | | **0.05 (0.01, 0.09)** | | | | | | **0.06 (0.01, 0.10)** | | |

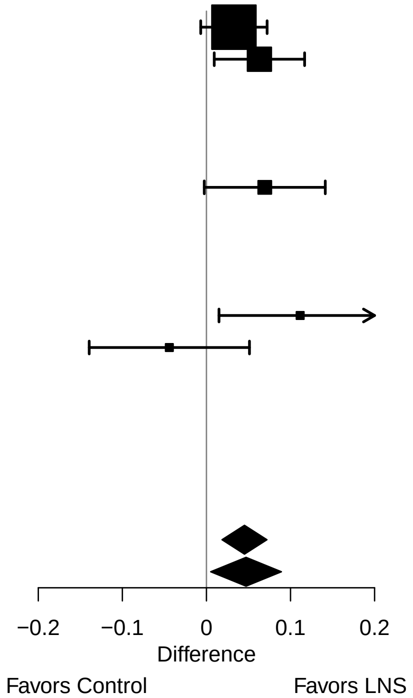


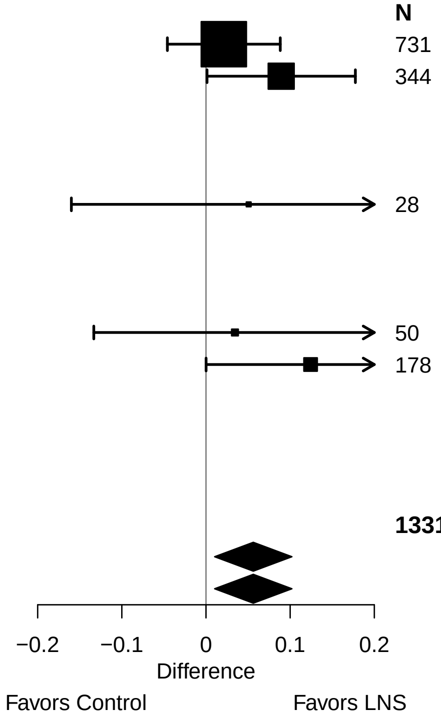

## Supplemental figure 7P: 12-mo walking without support prevalence difference

## 7P9: Stratified by Child baseline acute malnutrition

**P−for−interaction = 0.170**
**Difference in PDs = 0.04 (−0.02, 0.11)**

| Country | Trial | No: LNS N | No: Control N | No: Control Prevalence | No: PD (95% CI) | No: Fixed W | No: Random W | Yes: LNS N | Yes: Control N | Yes: Control Prevalence | Yes: PD (95% CI) | Yes: Fixed W | Yes: Random W |
|---|---|---|---|---|---|---|---|---|---|---|---|---|---|
| Bangladesh | JiVitA−4 (21) | 2423 | 1084 | 44.6 | 0.03 (−0.01, 0.06) | 0.51 | 0.51 | 558 | 255 | 53.7 | 0.05 (−0.03, 0.12) | 0.57 | 0.35 |
| Bangladesh | RDNS (22) | 1447 | 689 | 73.0 | 0.07 (0.02, 0.12) | 0.26 | 0.26 | 119 | 50 | 90.0 | 0.09 (−0.01, 0.20) | 0.31 | 0.32 |
| Bangladesh | WASH−B (23) | | | | | | | | | | | | |
| Burkina Faso | iLiNS−Zinc (24) | | | | | | | | | | | | |
| Ghana | GHANA (25) | | | | | | | | | | | | |
| Ghana | iLiNS−DYAD−G (26) | 284 | 564 | 50.7 | 0.04 (−0.03, 0.11) | 0.14 | 0.14 | 23 | 57 | 84.2 | 0.36 (0.16, 0.56) | 0.08 | 0.21 |
| Haiti | HAITI (27) | | | | | | | | | | | | |
| Kenya | WASH−B (28) | | | | | | | | | | | | |
| Madagascar | MAHAY (29) | | | | | | | | | | | | |
| Malawi | iLiNS−DYAD−M (30) | 184 | 374 | 50.3 | 0.08 (0.00, 0.17) | 0.09 | 0.09 | 14 | 39 | 61.5 | 0.12 (−0.19, 0.42) | 0.03 | 0.13 |
| Malawi | iLiNS−DOSE (31) | | | | | | | | | | | | |
| Mali | PROMIS CS (32) | | | | | | | | | | | | |
| Zimbabwe | SHINE (HIV−) (33) | | | | | | | | | | | | |
| Zimbabwe | SHINE (HIV+) (34) | | | | | | | | | | | | |
| | | **4338** | **2711** | | **I² = 0.00, Tau² = 0.00** | | | **714** | **401** | | **I² = 0.64, Tau² = 0.01** | | |
| **Fixed** | | | | | **0.05 (0.02, 0.07)** | | | | | | **0.09 (0.03, 0.15)** | | |
| **Random** | | | | | **0.05 (0.02, 0.07)** | | | | | | **0.14 (0.00, 0.27)** | | |

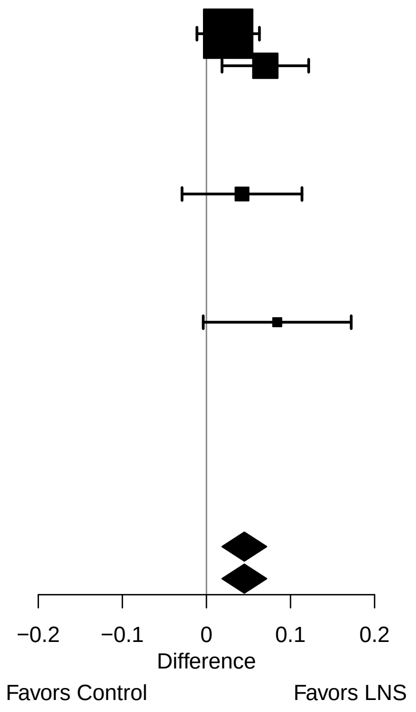


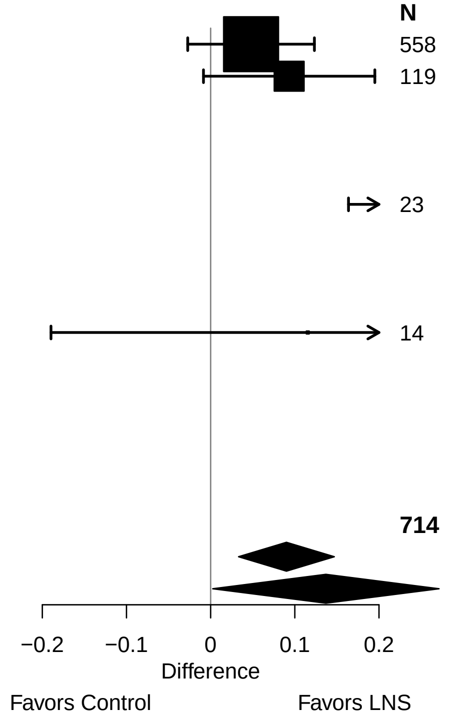

## Supplemental figure 7P: 12-mo walking without support prevalence difference

### 7P10: Stratified by Child baseline anemia

**P−for−interaction = 0.330**
**Difference in PDs = −0.04 (−0.12, 0.04)**

| Country | Trial | LNS N | Control N | Control Prevalence | Not anemic PD (95% CI) | Fixed W | Random W | LNS N | Control N | Control Prevalence | Anemic PD (95% CI) | Fixed W | Random W |
|---|---|---|---|---|---|---|---|---|---|---|---|---|---|
| Bangladesh | JiVitA−4 (21) | | | | | | | | | | | | |
| Bangladesh | RDNS (22) | 211 | 95 | 71.6 | 0.06 (−0.04, 0.15) | 0.31 | 0.31 | 310 | 157 | 74.5 | 0.05 (−0.04, 0.15) | 0.29 | 0.27 |
| Bangladesh | WASH−B (23) | | | | | | | | | | | | |
| Burkina Faso | iLiNS−Zinc (24) | | | | | | | | | | | | |
| Ghana | GHANA (25) | | | | | | | | | | | | |
| Ghana | iLiNS−DYAD−G (26) | 185 | 358 | 51.4 | 0.11 (0.02, 0.20) | 0.37 | 0.37 | 91 | 201 | 55.2 | −0.03 (−0.15, 0.09) | 0.18 | 0.20 |
| Haiti | HAITI (27) | | | | | | | | | | | | |
| Kenya | WASH−B (28) | | | | | | | | | | | | |
| Madagascar | MAHAY (29) | | | | | | | | | | | | |
| Malawi | iLiNS−DYAD−M (30) | 63 | 143 | 45.5 | 0.03 (−0.12, 0.17) | 0.13 | 0.13 | 135 | 266 | 54.1 | 0.13 (0.02, 0.23) | 0.25 | 0.25 |
| Malawi | iLiNS−DOSE (31) | 205 | 84 | 67.9 | 0.06 (−0.06, 0.19) | 0.19 | 0.19 | 393 | 116 | 63.8 | −0.01 (−0.11, 0.09) | 0.28 | 0.27 |
| Mali | PROMIS CS (32) | | | | | | | | | | | | |
| Zimbabwe | SHINE (HIV−) (33) | | | | | | | | | | | | |
| Zimbabwe | SHINE (HIV+) (34) | | | | | | | | | | | | |
| | | **664** | **680** | | **I² = 0.00, Tau² = 0.00** | | | **929** | **740** | | **I² = 0.41, Tau² = 0.00** | | |
| **Fixed** | | | | | **0.07 (0.02, 0.13)** | | | | | | **0.04 (−0.01, 0.09)** | | |
| **Random** | | | | | **0.07 (0.02, 0.13)** | | | | | | **0.04 (−0.03, 0.11)** | | |

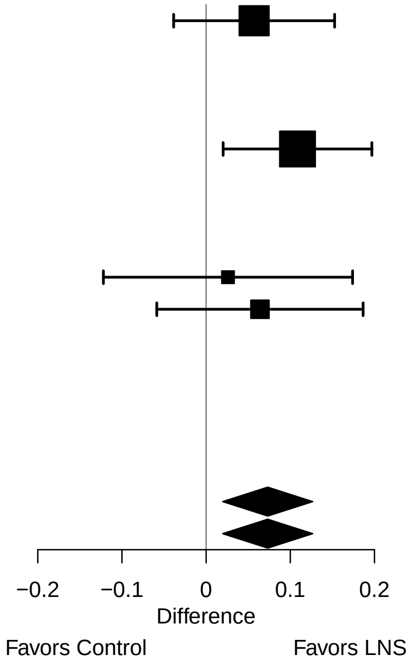


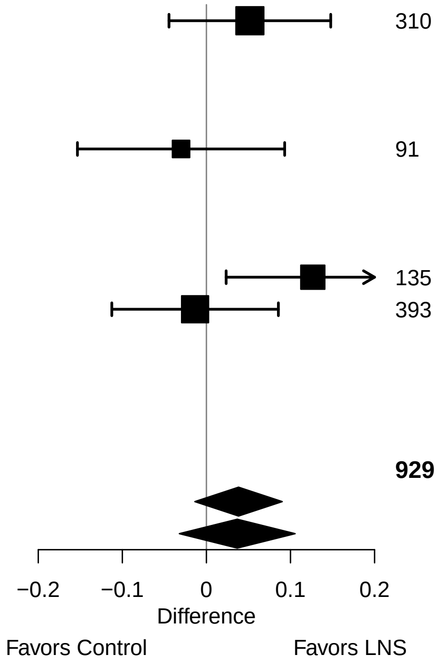

Supplement: nqab277_Supplemental_Files [file nqab277_supplemental_files.zip › ipdd_suppfig7_20210707.pdf]
