# Supplementary material for: Small-quantity lipid-based nutrient supplements for children age 6–24 months: a systematic review and individual participant data meta-analysis of effects on developmental outcomes and effect modifiers
Source: Am J Clin Nutr. 2021 Sep 29;114(Suppl 1):43S–67S. doi: 10.1093/ajcn/nqab277 (PMC8560311; doi:10.1093/ajcn/nqab277)

Online supplemental material: Small-quantity lipid-based nutrient supplements for children age 6-24 months: a systematic review and individual participant data meta-analysis of effects on developmental outcomes and effect modifiers. Prado et al. (2021)

Supplemental figure 8: Forest plots for effects of SQ-LNS on developmental outcomes stratified by all individual-level household effect modifiers

Contents

Supplemental figure 8A: Mean difference in language z-score **4**
8A1: Stratified by Household socio-economic status . . . 4
8A2: Stratified by Household food insecurity . . . 5
8A3: Stratified by Household source water quality . . . 6
8A4: Stratified by Household sanitation . . . 7
8A5: Stratified by Home environment . . . 8
8A6: Stratified by Season at the time of assessment . . . 9

Supplemental figure 8B: Language lowest decile prevalence ratio **10**
8B1: Stratified by Household socio-economic status . . . 10
8B2: Stratified by Household food insecurity . . . 11
8B3: Stratified by Household source water quality . . . 12
8B4: Stratified by Household sanitation . . . 13
8B5: Stratified by Home environment . . . 14
8B6: Stratified by Season at the time of assessment . . . 15

Supplemental figure 8C: Language lowest decile prevalence difference **16**
8C1: Stratified by Household socio-economic status . . . 16
8C2: Stratified by Household food insecurity . . . 17
8C3: Stratified by Household source water quality . . . 18
8C4: Stratified by Household sanitation . . . 19
8C5: Stratified by Home environment . . . 20
8C6: Stratified by Season at the time of assessment . . . 21

Supplemental figure 8D: Mean difference in social-emotional z-score **22**
8D1: Stratified by Household socio-economic status . . . 22
8D2: Stratified by Household food insecurity . . . 23
8D3: Stratified by Household source water quality . . . 24
8D4: Stratified by Household sanitation . . . 25
8D5: Stratified by Home environment . . . 26
8D6: Stratified by Season at the time of assessment . . . 27

Supplemental figure 8E: Social-emotional lowest decile prevalence ratio **28**
8E1: Stratified by Household socio-economic status . . . 28
8E2: Stratified by Household food insecurity . . . 29
8E3: Stratified by Household source water quality . . . 30
8E4: Stratified by Household sanitation . . . 31
8E5: Stratified by Home environment . . . 32
8E6: Stratified by Season at the time of assessment . . . 33

Supplemental figure 8F: Social-emotional lowest decile prevalence difference **34**

8F1: Stratified by Household socio-economic status . . . 34
8F2: Stratified by Household food insecurity . . . 35
8F3: Stratified by Household source water quality . . . 36
8F4: Stratified by Household sanitation . . . 37
8F5: Stratified by Home environment . . . 38
8F6: Stratified by Season at the time of assessment . . . 39

Supplemental figure 8G: Mean difference in motor z-score **40**
8G1: Stratified by Household socio-economic status . . . 40
8G2: Stratified by Household food insecurity . . . 41
8G3: Stratified by Household source water quality . . . 42
8G4: Stratified by Household sanitation . . . 43
8G5: Stratified by Home environment . . . 44
8G6: Stratified by Season at the time of assessment . . . 45

Supplemental figure 8H: Motor lowest decile prevalence ratio **46**
8H1: Stratified by Household socio-economic status . . . 46
8H2: Stratified by Household food insecurity . . . 47
8H3: Stratified by Household source water quality . . . 48
8H4: Stratified by Household sanitation . . . 49
8H5: Stratified by Home environment . . . 50
8H6: Stratified by Season at the time of assessment . . . 51

Supplemental figure 8I: Motor lowest decile prevalence difference **52**
8I1: Stratified by Household socio-economic status . . . 52
8I2: Stratified by Household food insecurity . . . 53
8I3: Stratified by Household source water quality . . . 54
8I4: Stratified by Household sanitation . . . 55
8I5: Stratified by Home environment . . . 56
8I6: Stratified by Season at the time of assessment . . . 57

Supplemental figure 8J: Mean difference in gross motor z-score **58**
8J1: Stratified by Household socio-economic status . . . 58
8J2: Stratified by Household food insecurity . . . 59
8J3: Stratified by Household source water quality . . . 60
8J4: Stratified by Household sanitation . . . 61
8J5: Stratified by Home environment . . . 62
8J6: Stratified by Season at the time of assessment . . . 63

Supplemental figure 8K: Mean difference in fine motor z-score **64**
8K1: Stratified by Household socio-economic status . . . 64
8K2: Stratified by Household food insecurity . . . 65
8K3: Stratified by Household source water quality . . . 66
8K4: Stratified by Household sanitation . . . 67
8K5: Stratified by Home environment . . . 68
8K6: Stratified by Season at the time of assessment . . . 69

Supplemental figure 8L: Mean difference in executive function z-score **70**
8L1: Stratified by Household socio-economic status . . . 70
8L2: Stratified by Household food insecurity . . . 71
8L3: Stratified by Household source water quality . . . 72

8L4: Stratified by Household sanitation . . . 73
8L5: Stratified by Home environment . . . 74
8L6: Stratified by Season at the time of assessment . . . 75

Supplemental figure 8M: Executive function lowest decile prevalence ratio **76**
8M1: Stratified by Household socio-economic status . . . 76
8M2: Stratified by Household food insecurity . . . 77
8M3: Stratified by Household source water quality (insufficient comparisons) . . . 78
8M4: Stratified by Household sanitation (insufficient comparisons) . . . 79
8M5: Stratified by Home environment . . . 80
8M6: Stratified by Season at the time of assessment . . . 81

Supplemental figure 8N: Executive function lowest decile prevalence difference **82**
8N1: Stratified by Household socio-economic status . . . 82
8N2: Stratified by Household food insecurity . . . 83
8N3: Stratified by Household source water quality (insufficient comparisons) . . . 84
8N4: Stratified by Household sanitation (insufficient comparisons) . . . 85
8N5: Stratified by Home environment . . . 86
8N6: Stratified by Season at the time of assessment . . . 87

Supplemental figure 8O: 12-mo walking without support prevalence ratio **88**
8O1: Stratified by Household socio-economic status . . . 88
8O2: Stratified by Household food insecurity . . . 89
8O3: Stratified by Household source water quality . . . 90
8O4: Stratified by Household sanitation . . . 91
8O5: Stratified by Home environment . . . 92
8O6: Stratified by Season at the time of assessment . . . 93

Supplemental figure 8P: 12-mo walking without support prevalence difference **94**
8P1: Stratified by Household socio-economic status . . . 94
8P2: Stratified by Household food insecurity . . . 95
8P3: Stratified by Household source water quality . . . 96
8P4: Stratified by Household sanitation . . . 97
8P5: Stratified by Home environment . . . 98
8P6: Stratified by Season at the time of assessment . . . 99

Supplemental figure 8: Forest plots for effects of SQ-LNS on developmental outcomes stratified by all individual-level household effect modifiers

These figures are forest plots showing the individual-level effect modification of intervention effects. Each figure has the estimates of intervention effect stratified within study by individual-level effect modifier category. For definitions of effect modifiers, see Box 1 in the main paper. Individual study estimates were generated from log-binomial regression for dichotomous outcomes and simple linear regression for continuous outcomes; controlling for baseline measure when available and with clustered observations using robust standard errors for cluster-randomized trials. Pooled interaction term and sub-group estimates were generated using inverse-variance weighting fixed and random effects. For continuous outcomes analyzed via mean differences, the effect estimate is the mean in the LNS group minus the mean in the control group. For dichotomous outcomes analyzed via prevalence ratios, the effect estimate is the prevalence in the LNS group divided by the prevalence in the control group. For dichotomous outcomes analyzed via prevalence differences, the effect estimate is the prevalence in the LNS group minus the prevalence in the control group.

The labels on the far left correspond to trial level information. In the middle left and on the right the values indicate the study level effect estimate, confidence interval, and weighting for deriving the pooled estimates is shown by subgroup.

Supplemental figure 8A: Mean difference in language z-score

8A1: Stratified by Household socio-economic status

P−for−interaction = 0.001
Difference in MDs = 0.10 (0.04, 0.15)

			At least median						Less than median					
**Country**	**Trial**	**Tool**	**LNS N**	**Control N**	**Control Mean**	**MD (95% CI)**	**Fixed W**	**Random W**	**LNS N**	**Control N**	**Control Mean**	**MD (95% CI)**	**Fixed W**	**Random W**
Bangladesh	JiVitA−4 (21)	BSID−III	236	77	0.31	−0.19 (−0.46, 0.08)	0.02	0.05	208	66	−0.23	0.04 (−0.21, 0.29)	0.03	0.06
Bangladesh	RDNS (22)	CDI	834	407	0.02	0.17 (0.01, 0.32)	0.07	0.09	829	407	−0.24	0.17 (0.06, 0.27)	0.16	0.11
Bangladesh	WASH−B (23)	CDI	568	1703	0.15	0.07 (−0.02, 0.16)	0.21	0.13	541	1650	−0.20	0.11 (0.01, 0.21)	0.19	0.11
Burkina Faso	iLiNS−Zinc (24)	DMC	459	163	−0.12	0.24 (0.08, 0.41)	0.06	0.09	287	212	−0.34	0.47 (0.27, 0.67)	0.05	0.07
Ghana	GHANA (25)													
Ghana	iLiNS−DYAD−G (26)	CDI	152	341	0.07	0.08 (−0.11, 0.27)	0.05	0.08	179	315	−0.08	−0.04 (−0.23, 0.15)	0.06	0.08
Haiti	HAITI (27)	Total sounds	76	60	−0.13	0.14 (−0.19, 0.46)	0.02	0.04	61	72	0.09	−0.26 (−0.59, 0.08)	0.02	0.04
Kenya	WASH−B (28)	EASQ	778	2706	0.09	−0.05 (−0.13, 0.02)	0.31	0.14	582	2036	−0.13	0.12 (0.02, 0.21)	0.23	0.12
Madagascar	MAHAY (29)	ASQI	741	845	0.32	−0.15 (−0.34, 0.04)	0.05	0.08	823	724	−0.25	0.00 (−0.19, 0.18)	0.06	0.08
Malawi	iLiNS−DYAD−M (30)	CDI	118	230	0.09	−0.06 (−0.28, 0.16)	0.04	0.06	97	208	−0.11	0.09 (−0.15, 0.34)	0.03	0.06
Malawi	iLiNS−DOSE (31)	CDI	278	94	0.04	−0.01 (−0.25, 0.23)	0.03	0.06	271	96	−0.12	0.13 (−0.10, 0.36)	0.04	0.06
Mali	PROMIS CS (32)	DMC	494	450	0.03	0.05 (−0.14, 0.25)	0.04	0.07	433	494	−0.17	0.23 (0.03, 0.42)	0.05	0.07
Zimbabwe	SHINE (HIV−) (33)	CDI	396	351	0.03	0.06 (−0.09, 0.21)	0.08	0.10	375	383	−0.12	0.12 (−0.04, 0.28)	0.07	0.09
Zimbabwe	SHINE (HIV+) (34)	CDI	86	77	−0.10	0.32 (−0.01, 0.65)	0.02	0.04	74	64	−0.14	0.13 (−0.19, 0.45)	0.02	0.04
			**5216**	**7504**		**I² = 0.53, Tau² = 0.01**			**4760**	**6727**		**I² = 0.48, Tau² = 0.01**		
**Fixed**						**0.03 (−0.01, 0.07)**						**0.12 (0.08, 0.17)**		
**Random**						**0.05 (−0.03, 0.12)**						**0.11 (0.04, 0.19)**		

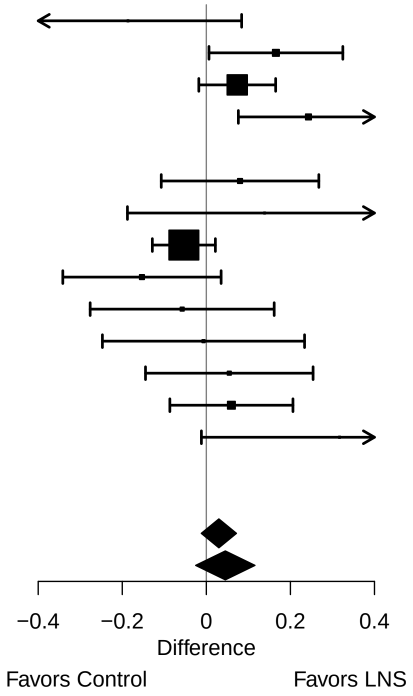

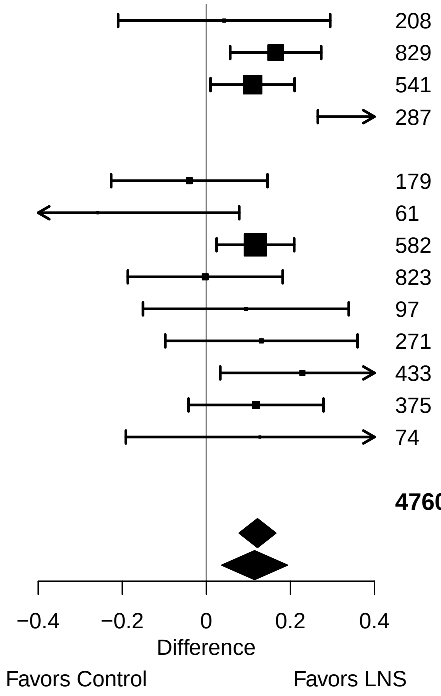

Supplemental figure 8A: Mean difference in language z-score

8A2: Stratified by Household food insecurity

P−for−interaction = 0.627
Difference in MDs = 0.02 (−0.05, 0.09)

Country	Trial	Tool	LNS N	Control N	Control Mean	Mild to secure MD (95% CI)	Fixed W	Random W	LNS N	Control N	Control Mean	Moderate to severe MD (95% CI)	Fixed W	Random W
Bangladesh	JiVitA−4 (21)	BSID−III	329	111	0.16	−0.15 (−0.37, 0.08)	0.03	0.08	115	32	−0.28	0.16 (−0.12, 0.44)	0.05	0.06
Bangladesh	RDNS (22)	CDI	1058	493	−0.02	0.14 (0.01, 0.27)	0.09	0.11	605	321	−0.25	0.19 (0.05, 0.32)	0.20	0.16
Bangladesh	WASH−B (23)	CDI	883	2608	0.03	0.11 (0.03, 0.19)	0.24	0.12	226	745	−0.22	0.00 (−0.16, 0.17)	0.13	0.12
Burkina Faso	iLiNS−Zinc (24)	DMC	389	162	−0.22	0.38 (0.17, 0.58)	0.03	0.09	357	213	−0.27	0.35 (0.18, 0.52)	0.13	0.12
Ghana	GHANA (25)													
Ghana	iLiNS−DYAD−G (26)	CDI	238	453	0.00	0.00 (−0.15, 0.16)	0.06	0.10	92	202	0.00	0.02 (−0.24, 0.28)	0.05	0.07
Haiti	HAITI (27)	Total sounds												
Kenya	WASH−B (28)	EASQ	1214	4244	0.01	0.02 (−0.04, 0.08)	0.38	0.12	146	487	−0.11	0.01 (−0.18, 0.20)	0.10	0.11
Madagascar	MAHAY (29)	ASQI	940	892	0.11	−0.15 (−0.37, 0.07)	0.03	0.08	336	389	−0.15	−0.02 (−0.26, 0.22)	0.06	0.07
Malawi	iLiNS−DYAD−M (30)	CDI	68	118	0.10	−0.25 (−0.55, 0.06)	0.02	0.06	146	315	−0.03	0.11 (−0.08, 0.31)	0.10	0.10
Malawi	iLiNS−DOSE (31)	CDI	133	51	0.17	−0.07 (−0.42, 0.27)	0.01	0.05	403	133	−0.07	0.07 (−0.12, 0.26)	0.10	0.10
Mali	PROMIS CS (32)	DMC												
Zimbabwe	SHINE (HIV−) (33)	CDI	612	576	−0.04	0.14 (0.01, 0.26)	0.09	0.11	136	148	−0.14	−0.07 (−0.34, 0.20)	0.05	0.06
Zimbabwe	SHINE (HIV+) (34)	CDI	114	103	−0.06	0.28 (0.01, 0.56)	0.02	0.07	41	35	−0.22	−0.02 (−0.44, 0.41)	0.02	0.03
			**5978**	**9811**		**I² = 0.66, Tau² = 0.02**			**2603**	**3020**		**I² = 0.35, Tau² = 0.00**		
**Fixed**						**0.06 (0.03, 0.10)**						**0.10 (0.04, 0.17)**		
**Random**						**0.05 (−0.05, 0.16)**						**0.10 (0.02, 0.17)**		

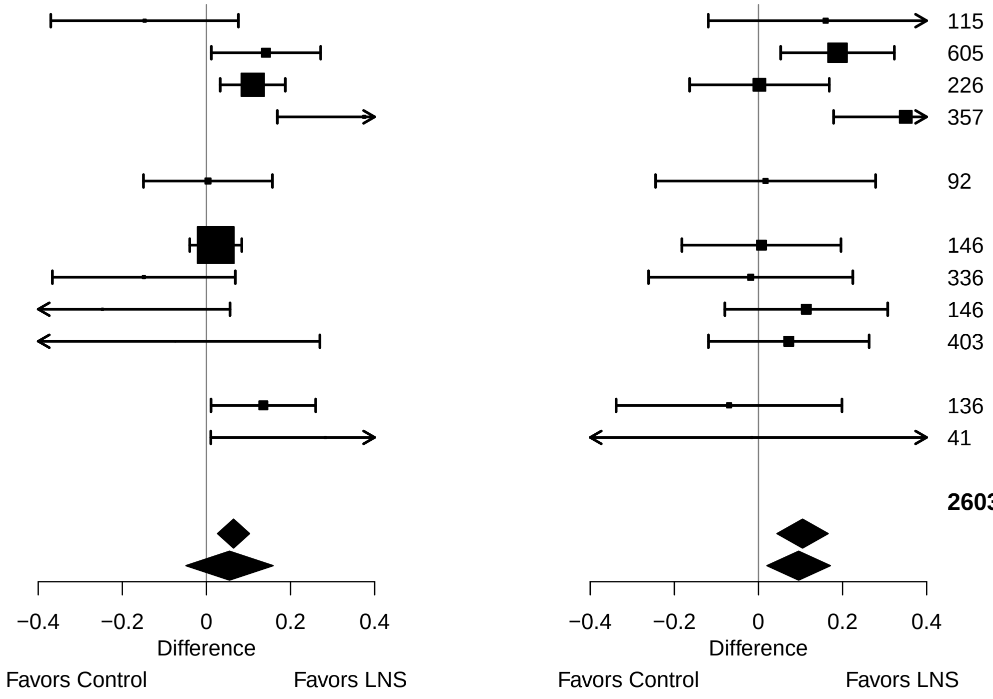

Supplemental figure 8A: Mean difference in language z-score

8A3: Stratified by Household source water quality

P−for−interaction = 0.988
Difference in MDs = 0.00 (−0.11, 0.11)

Country	Trial	Tool	LNS N	Control N	Control Mean	Improved MD (95% CI)	Fixed W	Random W	LNS N	Control N	Control Mean	Unimproved MD (95% CI)	Fixed W	Random W
Bangladesh	JiVitA−4 (21)	BSID−III												
Bangladesh	RDNS (22)	CDI												
Bangladesh	WASH−B (23)	CDI	480	981	−0.08	0.18 (0.07, 0.30)	0.26	0.24	67	117	−0.22	0.09 (−0.23, 0.41)	0.06	0.10
Burkina Faso	iLiNS−Zinc (24)	DMC	220	82	−0.06	0.30 (0.08, 0.52)	0.07	0.08	526	293	−0.30	0.37 (0.20, 0.54)	0.23	0.18
Ghana	GHANA (25)													
Ghana	iLiNS−DYAD−G (26)	CDI												
Haiti	HAITI (27)	Total sounds												
Kenya	WASH−B (28)	EASQ	413	1419	0.01	0.01 (−0.12, 0.15)	0.20	0.19	236	649	−0.03	0.12 (−0.03, 0.27)	0.28	0.19
Madagascar	MAHAY (29)	ASQI	405	430	−0.05	−0.07 (−0.42, 0.29)	0.03	0.03	1134	1133	0.10	−0.14 (−0.32, 0.05)	0.18	0.16
Malawi	iLiNS−DYAD−M (30)	CDI	195	401	0.01	0.00 (−0.17, 0.17)	0.12	0.12	20	36	−0.11	0.14 (−0.38, 0.66)	0.02	0.05
Malawi	iLiNS−DOSE (31)	CDI	514	177	−0.04	0.07 (−0.10, 0.24)	0.12	0.12	44	15	0.06	−0.16 (−0.72, 0.40)	0.02	0.04
Mali	PROMIS CS (32)	DMC	528	552	−0.04	0.12 (−0.09, 0.33)	0.08	0.09	357	378	−0.15	0.17 (−0.05, 0.39)	0.13	0.14
Zimbabwe	SHINE (HIV−) (33)	CDI	220	212	0.00	0.12 (−0.07, 0.31)	0.10	0.10	125	122	−0.10	−0.13 (−0.42, 0.16)	0.07	0.11
Zimbabwe	SHINE (HIV+) (34)	CDI	36	41	−0.18	0.12 (−0.33, 0.57)	0.02	0.02	25	25	−0.16	−0.20 (−0.86, 0.46)	0.01	0.03
			**3011**	**4295**		**I² = 0.13, Tau² = 0.00**			**2534**	**2768**		**I² = 0.62, Tau² = 0.02**		
**Fixed**						**0.10 (0.04, 0.16)**						**0.11 (0.03, 0.18)**		
**Random**						**0.10 (0.04, 0.17)**						**0.08 (−0.05, 0.20)**		

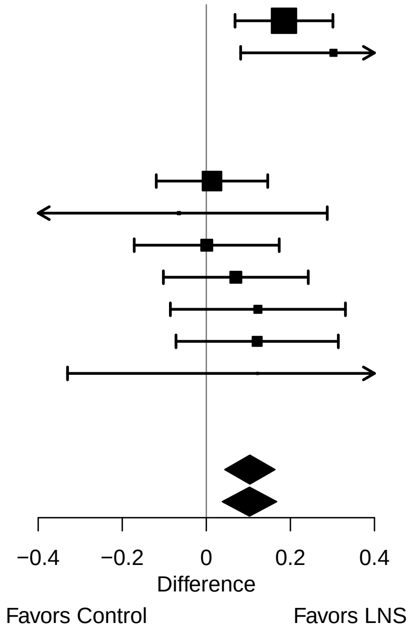

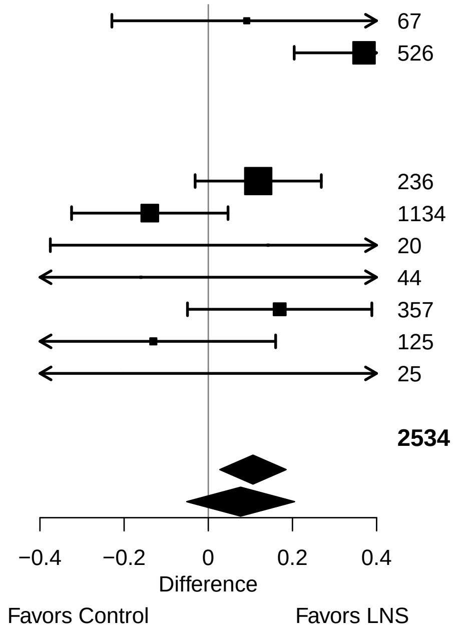

Supplemental figure 8A: Mean difference in language z-score

8A4: Stratified by Household sanitation

P−for−interaction = 0.218
Difference in MDs = −0.06 (−0.16, 0.04)

Country	Trial	Tool	LNS N	Control N	Control Mean	Improved MD (95% CI)	Fixed W	Random W	LNS N	Control N	Control Mean	Unimproved MD (95% CI)	Fixed W	Random W
Bangladesh	JiVitA−4 (21)	BSID−III	353	112	0.12	−0.10 (−0.30, 0.10)	0.08	0.11	91	31	−0.16	−0.03 (−0.45, 0.39)	0.02	0.03
Bangladesh	RDNS (22)	CDI	1179	572	−0.06	0.18 (0.07, 0.29)	0.27	0.18	482	241	−0.22	0.13 (0.00, 0.26)	0.19	0.17
Bangladesh	WASH−B (23)	CDI	492	998	−0.07	0.18 (0.06, 0.29)	0.24	0.18	29	53	−0.36	0.19 (−0.25, 0.64)	0.02	0.03
Burkina Faso	iLiNS−Zinc (24)	DMC	16	11	−0.45	0.77 (0.19, 1.34)	0.01	0.02	730	364	−0.24	0.36 (0.19, 0.52)	0.12	0.13
Ghana	GHANA (25)													
Ghana	iLiNS−DYAD−G (26)	CDI	320	640	−0.01	0.02 (−0.12, 0.15)	0.18	0.16	10	16	0.30	−0.40 (−1.24, 0.43)	0.00	0.01
Haiti	HAITI (27)	Total sounds												
Kenya	WASH−B (28)	EASQ	94	337	0.12	0.13 (−0.09, 0.36)	0.06	0.10	555	1732	−0.02	0.04 (−0.07, 0.15)	0.26	0.19
Madagascar	MAHAY (29)	ASQI												
Malawi	iLiNS−DYAD−M (30)	CDI	19	42	0.19	−0.04 (−0.54, 0.46)	0.01	0.03	196	395	−0.02	0.02 (−0.15, 0.19)	0.11	0.12
Malawi	iLiNS−DOSE (31)	CDI	12	9	0.32	−0.23 (−1.09, 0.62)	0.00	0.01	546	183	−0.05	0.07 (−0.10, 0.24)	0.12	0.13
Mali	PROMIS CS (32)	DMC	687	678	−0.05	0.15 (−0.04, 0.34)	0.09	0.11	211	242	−0.10	0.11 (−0.12, 0.33)	0.07	0.08
Zimbabwe	SHINE (HIV−) (33)	CDI	127	106	−0.03	0.14 (−0.12, 0.40)	0.05	0.08	217	229	−0.04	−0.02 (−0.22, 0.19)	0.08	0.10
Zimbabwe	SHINE (HIV+) (34)	CDI	24	17	−0.06	−0.10 (−0.70, 0.50)	0.01	0.02	37	49	−0.21	0.01 (−0.44, 0.47)	0.02	0.02
			**3323**	**3522**		**I² = 0.36, Tau² = 0.01**			**3104**	**3535**		**I² = 0.34, Tau² = 0.00**		
**Fixed**						**0.12 (0.06, 0.17)**						**0.10 (0.04, 0.15)**		
**Random**						**0.11 (0.02, 0.20)**						**0.10 (0.02, 0.17)**		

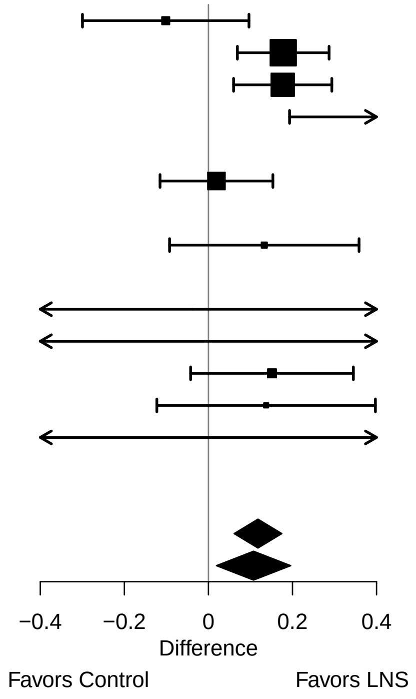

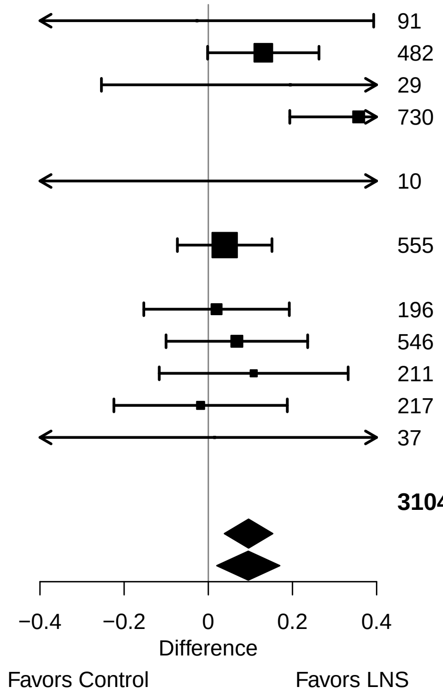

Supplemental figure 8A: Mean difference in language z-score

8A5: Stratified by Home environment

P−for−interaction = 0.251
Difference in MDs = 0.04 (−0.03, 0.11)

Country	Trial	Tool	At least median: LNS N	Control N	Control Mean	MD (95% CI)	Fixed W	Random W	Less than median: LNS N	Control N	Control Mean	MD (95% CI)	Fixed W	Random W
Bangladesh	JiVitA−4 (21)	BSID−III												
Bangladesh	RDNS (22)	CDI	1192	581	0.07	0.16 (0.05, 0.26)	0.17	0.16	468	230	−0.56	0.18 (−0.04, 0.40)	0.06	0.11
Bangladesh	WASH−B (23)	CDI	630	1652	0.23	0.11 (0.03, 0.20)	0.24	0.18	479	1700	−0.27	−0.02 (−0.13, 0.09)	0.25	0.16
Burkina Faso	iLiNS−Zinc (24)	DMC	473	199	0.11	0.17 (0.02, 0.33)	0.07	0.11	272	175	−0.64	0.49 (0.29, 0.69)	0.08	0.12
Ghana	GHANA (25)													
Ghana	iLiNS−DYAD−G (26)	CDI	213	410	0.18	0.01 (−0.15, 0.17)	0.07	0.11	118	248	−0.31	−0.03 (−0.24, 0.19)	0.07	0.12
Haiti	HAITI (27)	Total sounds												
Kenya	WASH−B (28)	EASQ	809	2682	0.20	−0.02 (−0.10, 0.06)	0.30	0.18	552	2060	−0.27	0.05 (−0.04, 0.14)	0.37	0.17
Madagascar	MAHAY (29)	ASQI	843	839	0.21	−0.16 (−0.36, 0.03)	0.04	0.08	770	764	−0.12	−0.06 (−0.26, 0.15)	0.07	0.12
Malawi	iLiNS−DYAD−M (30)	CDI	137	290	0.20	−0.01 (−0.21, 0.19)	0.05	0.09	78	149	−0.40	0.10 (−0.16, 0.36)	0.04	0.10
Malawi	iLiNS−DOSE (31)	CDI	375	135	0.15	0.01 (−0.17, 0.19)	0.05	0.09	269	86	−0.32	0.13 (−0.13, 0.38)	0.05	0.10
Mali	PROMIS CS (32)	DMC												
Zimbabwe	SHINE (HIV−) (33)	CDI												
Zimbabwe	SHINE (HIV+) (34)	CDI												
			**4672**	**6788**		**I² = 0.59, Tau² = 0.01**			**3006**	**5412**		**I² = 0.70, Tau² = 0.02**		
**Fixed**						**0.05 (0.01, 0.10)**						**0.07 (0.01, 0.12)**		
**Random**						**0.05 (−0.03, 0.12)**						**0.10 (−0.02, 0.22)**		

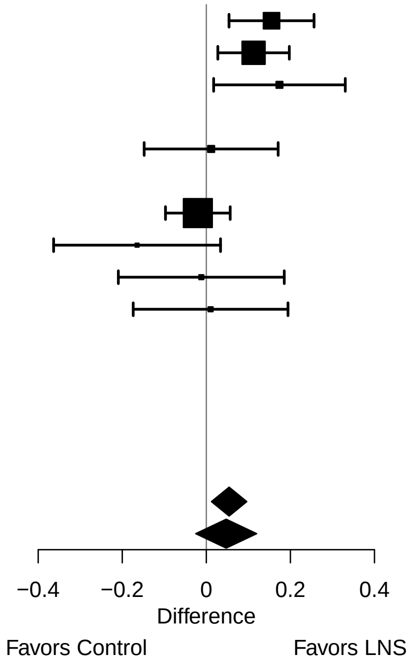

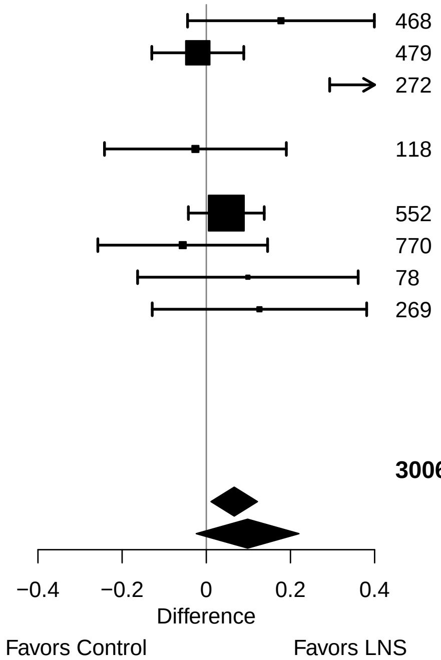

Supplemental figure 8A: Mean difference in language z-score

8A6: Stratified by Season at the time of assessment

P−for−interaction = 0.979
Difference in MDs = 0.00 (−0.07, 0.07)

			Dry						Rainy					
**Country**	**Trial**	**Tool**	**LNS N**	**Control N**	**Control Mean**	**MD (95% CI)**	**Fixed W**	**Random W**	**LNS N**	**Control N**	**Control Mean**	**MD (95% CI)**	**Fixed W**	**Random W**
Bangladesh	JiVitA−4 (21)	BSID−III	251	70	0.11	−0.08 (−0.34, 0.19)	0.03	0.06	194	73	0.02	−0.10 (−0.36, 0.15)	0.03	0.06
Bangladesh	RDNS (22)	CDI	739	359	−0.09	0.17 (0.03, 0.31)	0.12	0.12	924	455	−0.13	0.16 (0.03, 0.29)	0.12	0.14
Bangladesh	WASH−B (23)	CDI	629	1944	0.04	0.06 (−0.03, 0.15)	0.29	0.16	480	1409	−0.11	0.14 (0.02, 0.25)	0.18	0.17
Burkina Faso	iLiNS−Zinc (24)	DMC	514	274	−0.25	0.42 (0.18, 0.66)	0.04	0.07	232	101	−0.24	0.25 (0.03, 0.47)	0.05	0.08
Ghana	GHANA (25)													
Ghana	iLiNS−DYAD−G (26)	CDI	194	388	−0.03	0.10 (−0.07, 0.28)	0.08	0.10	137	270	0.04	−0.13 (−0.33, 0.08)	0.05	0.09
Haiti	HAITI (27)	Total sounds												
Kenya	WASH−B (28)	EASQ	315	1059	−0.18	0.07 (−0.05, 0.20)	0.15	0.13	1046	3682	0.05	0.01 (−0.06, 0.08)	0.44	0.22
Madagascar	MAHAY (29)	ASQI												
Malawi	iLiNS−DYAD−M (30)	CDI	153	311	0.05	−0.01 (−0.21, 0.18)	0.06	0.09	62	127	−0.14	0.09 (−0.20, 0.39)	0.02	0.05
Malawi	iLiNS−DOSE (31)	CDI	394	118	0.02	0.00 (−0.21, 0.21)	0.05	0.08	244	100	−0.08	0.09 (−0.14, 0.32)	0.04	0.07
Mali	PROMIS CS (32)	DMC												
Zimbabwe	SHINE (HIV−) (33)	CDI	582	555	−0.09	0.04 (−0.09, 0.17)	0.13	0.13	235	226	0.07	0.20 (−0.02, 0.41)	0.05	0.08
Zimbabwe	SHINE (HIV+) (34)	CDI	117	106	−0.18	0.25 (−0.02, 0.53)	0.03	0.06	48	41	0.00	0.23 (−0.17, 0.62)	0.01	0.03
			**3888**	**5184**		**I² = 0.38, Tau² = 0.01**			**3602**	**6484**		**I² = 0.43, Tau² = 0.00**		
**Fixed**						**0.09 (0.04, 0.13)**						**0.07 (0.02, 0.12)**		
**Random**						**0.09 (0.02, 0.17)**						**0.09 (0.01, 0.16)**		

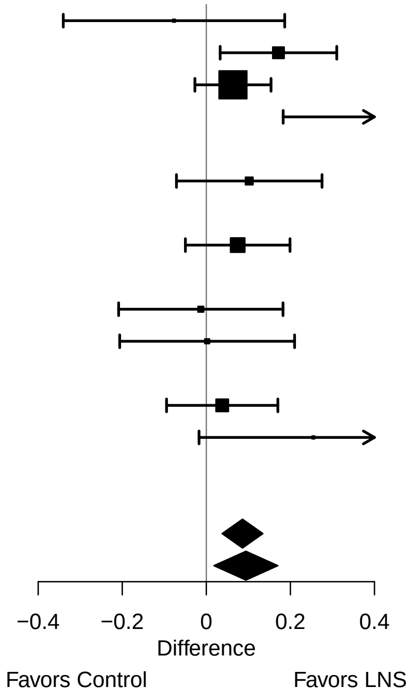

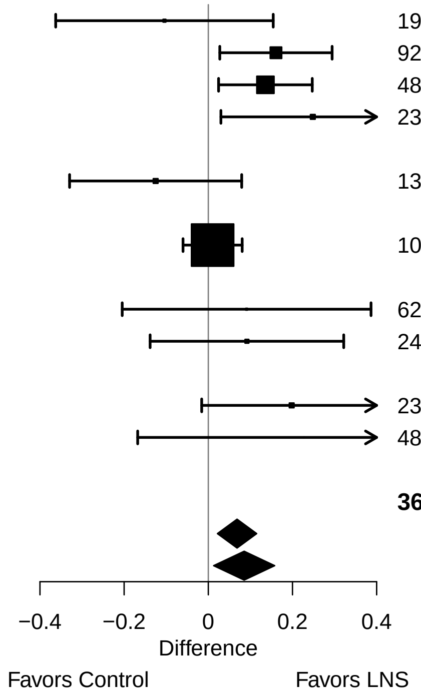

Supplemental figure 8B: Language lowest decile prevalence ratio

8B1: Stratified by Household socio-economic status

P−for−interaction = 0.227

Ratio of PRs = 0.90 (0.75, 1.07)

At least median

Country	Trial	Tool	LNS N	Control N	Control Prevalence	PR (95% CI)	Fixed W	Random W
Bangladesh	JiVitA−4 (21)	BSID−III	236	77	7.8	1.03 (0.43, 2.46)	0.02	0.03
Bangladesh	RDNS (22)	CDI	834	407	9.3	0.65 (0.43, 1.00)	0.10	0.11
Bangladesh	WASH−B (23)	CDI	568	1703	7.8	0.91 (0.66, 1.26)	0.18	0.16
Burkina Faso	iLiNS−Zinc (24)	DMC	459	163	15.3	0.50 (0.32, 0.78)	0.09	0.10
Ghana	GHANA (25)							
Ghana	iLiNS−DYAD−G (26)	CDI	152	341	7.9	1.00 (0.52, 1.91)	0.04	0.06
Haiti	HAITI (27)	Total sounds						
Kenya	WASH−B (28)	EASQ	778	2706	8.5	1.09 (0.84, 1.41)	0.28	0.20
Madagascar	MAHAY (29)	ASQI	741	845	5.0	1.36 (0.72, 2.56)	0.05	0.06
Malawi	iLiNS−DYAD−M (30)	CDI	118	230	9.1	0.74 (0.34, 1.63)	0.03	0.04
Malawi	iLiNS−DOSE (31)	CDI	278	94	9.6	1.09 (0.54, 2.22)	0.04	0.05
Mali	PROMIS CS (32)	DMC	494	450	9.6	0.85 (0.52, 1.39)	0.08	0.09
Zimbabwe	SHINE (HIV−) (33)	CDI	396	351	10.3	0.81 (0.49, 1.34)	0.07	0.09
Zimbabwe	SHINE (HIV+) (34)	CDI	86	77	15.6	0.45 (0.16, 1.24)	0.02	0.02
			**5140**	**7444**		**I² = 0.27, Tau² = 0.02**		
**Fixed**						**0.88 (0.76, 1.00)**		
**Random**						**0.86 (0.73, 1.01)**		

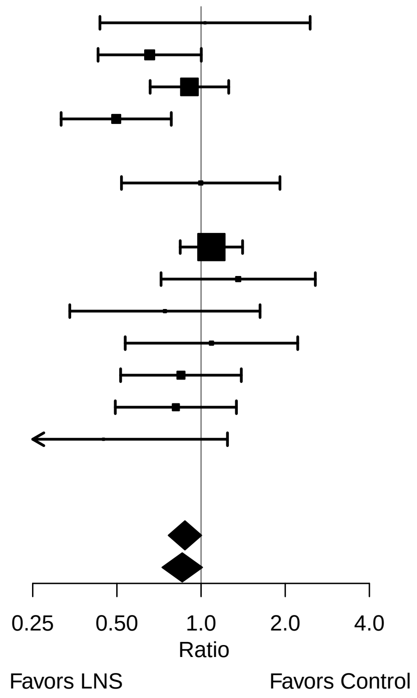

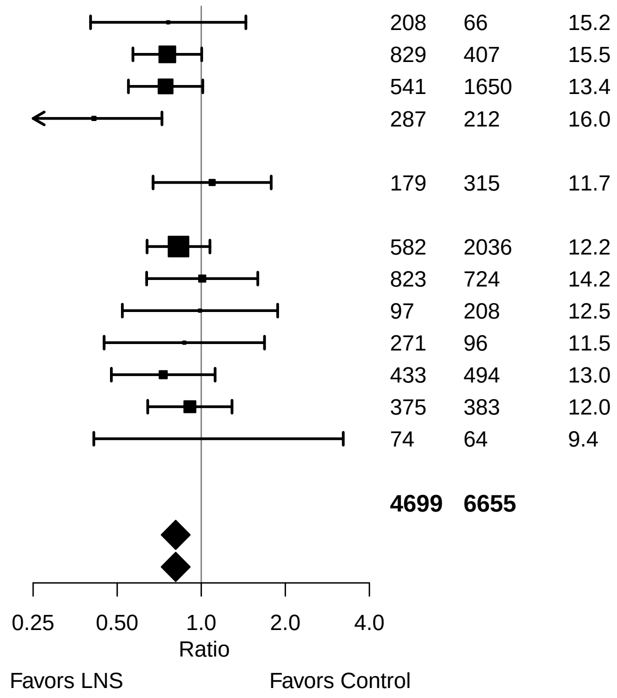

Less than median

Country	Trial	LNS N	Control N	Control Prevalence	PR (95% CI)	Fixed W	Random W
Bangladesh	JiVitA−4 (21)	208	66	15.2	0.76 (0.40, 1.44)	0.03	0.03
Bangladesh	RDNS (22)	829	407	15.5	0.76 (0.57, 1.00)	0.17	0.17
Bangladesh	WASH−B (23)	541	1650	13.4	0.75 (0.55, 1.01)	0.15	0.15
Burkina Faso	iLiNS−Zinc (24)	287	212	16.0	0.41 (0.24, 0.72)	0.04	0.04
Ghana	GHANA (25)						
Ghana	iLiNS−DYAD−G (26)	179	315	11.7	1.09 (0.67, 1.78)	0.06	0.06
Haiti	HAITI (27)						
Kenya	WASH−B (28)	582	2036	12.2	0.83 (0.64, 1.07)	0.21	0.21
Madagascar	MAHAY (29)	823	724	14.2	1.01 (0.64, 1.59)	0.07	0.07
Malawi	iLiNS−DYAD−M (30)	97	208	12.5	0.99 (0.52, 1.88)	0.03	0.03
Malawi	iLiNS−DOSE (31)	271	96	11.5	0.87 (0.45, 1.68)	0.03	0.03
Mali	PROMIS CS (32)	433	494	13.0	0.73 (0.48, 1.12)	0.08	0.08
Zimbabwe	SHINE (HIV−) (33)	375	383	12.0	0.91 (0.64, 1.29)	0.12	0.12
Zimbabwe	SHINE (HIV+) (34)	74	64	9.4	1.15 (0.41, 3.22)	0.01	0.01
		**4699**	**6655**		**I² = 0.00, Tau² = 0.00**		
**Fixed**					**0.81 (0.72, 0.91)**		
**Random**					**0.81 (0.72, 0.91)**		

Supplemental figure 8B: Language lowest decile prevalence ratio

8B2: Stratified by Household food insecurity

P−for−interaction = 0.772
Ratio of PRs = 1.03 (0.85, 1.25)

Country	Trial	Tool	LNS N	Control N	Control Prevalence	Mild to secure PR (95% CI)	Fixed W	Random W	LNS N	Control N	Control Prevalence	Moderate to severe PR (95% CI)	Fixed W	Random W
Bangladesh	JiVitA−4 (21)	BSID−III												
Bangladesh	RDNS (22)	CDI	1058	493	9.9	0.74 (0.54, 1.03)	0.12	0.13	605	321	16.2	0.71 (0.53, 0.97)	0.27	0.19
Bangladesh	WASH−B (23)	CDI	883	2608	9.6	0.80 (0.63, 1.02)	0.22	0.15	226	745	13.7	0.84 (0.54, 1.30)	0.13	0.14
Burkina Faso	iLiNS−Zinc (24)	DMC	389	162	15.4	0.42 (0.28, 0.63)	0.07	0.12	357	213	16.0	0.51 (0.35, 0.74)	0.18	0.16
Ghana	GHANA (25)													
Ghana	iLiNS−DYAD−G (26)	CDI	238	453	8.4	1.45 (0.92, 2.29)	0.06	0.11	92	202	12.9	0.51 (0.22, 1.19)	0.03	0.05
Haiti	HAITI (27)	Total sounds												
Kenya	WASH−B (28)	EASQ	1214	4244	10.0	0.90 (0.74, 1.09)	0.34	0.16	146	487	11.3	1.33 (0.84, 2.12)	0.12	0.13
Madagascar	MAHAY (29)	ASQI	940	892	8.0	1.40 (0.82, 2.41)	0.04	0.10	336	389	12.6	0.92 (0.49, 1.74)	0.06	0.08
Malawi	iLiNS−DYAD−M (30)	CDI	68	118	9.3	1.42 (0.62, 3.25)	0.02	0.06	146	315	10.5	0.72 (0.37, 1.38)	0.06	0.08
Malawi	iLiNS−DOSE (31)	CDI	133	51	9.8	0.77 (0.28, 2.13)	0.01	0.05	403	133	10.5	1.01 (0.57, 1.79)	0.08	0.10
Mali	PROMIS CS (32)	DMC												
Zimbabwe	SHINE (HIV−) (33)	CDI	612	576	10.6	0.82 (0.59, 1.14)	0.12	0.13	136	148	14.2	1.04 (0.54, 1.98)	0.06	0.08
Zimbabwe	SHINE (HIV+) (34)	CDI												
			**5535**	**9597**		**I² = 0.65, Tau² = 0.10**			**2447**	**2953**		**I² = 0.40, Tau² = 0.04**		
**Fixed**						**0.85 (0.76, 0.95)**						**0.78 (0.67, 0.91)**		
**Random**						**0.87 (0.68, 1.13)**						**0.80 (0.65, 0.99)**		

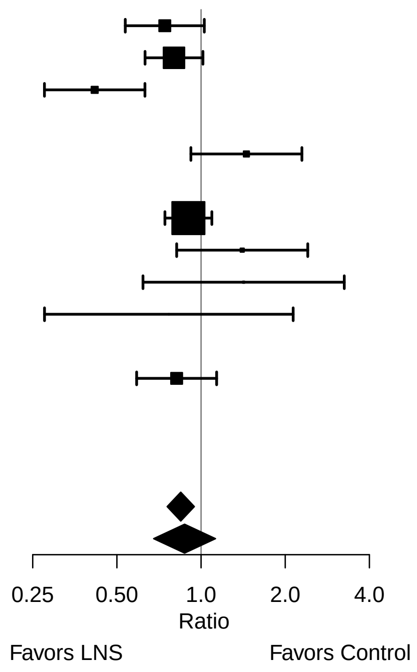

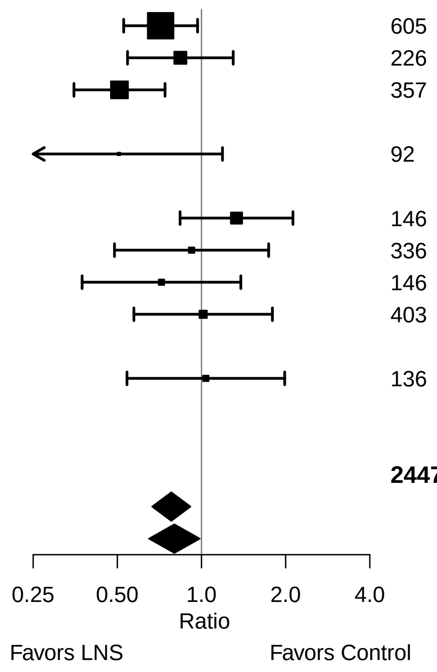

Supplemental figure 8B: Language lowest decile prevalence ratio

8B3: Stratified by Household source water quality

P−for−interaction = 0.707
Ratio of PRs = 0.95 (0.73, 1.24)

Country	Trial	Tool	Improved LNS N	Improved Control N	Improved Control Prevalence	Improved PR (95% CI)	Improved Fixed W	Improved Random W	Unimproved LNS N	Unimproved Control N	Unimproved Control Prevalence	Unimproved PR (95% CI)	Unimproved Fixed W	Unimproved Random W
Bangladesh	JiVitA−4 (21)	BSID−III												
Bangladesh	RDNS (22)	CDI												
Bangladesh	WASH−B (23)	CDI	480	981	10.9	0.65 (0.45, 0.93)	0.26	0.22	67	117	16.2	0.55 (0.21, 1.46)	0.04	0.07
Burkina Faso	iLiNS−Zinc (24)	DMC	220	82	12.2	0.45 (0.26, 0.77)	0.11	0.14	526	293	16.7	0.48 (0.33, 0.69)	0.27	0.22
Ghana	GHANA (25)													
Ghana	iLiNS−DYAD−G (26)	CDI												
Haiti	HAITI (27)	Total sounds												
Kenya	WASH−B (28)	EASQ	413	1419	10.4	1.01 (0.70, 1.44)	0.26	0.22	236	649	10.5	0.89 (0.61, 1.29)	0.26	0.22
Madagascar	MAHAY (29)	ASQI	405	430	12.3	1.18 (0.60, 2.31)	0.07	0.11	1134	1133	8.1	1.17 (0.73, 1.90)	0.16	0.18
Malawi	iLiNS−DYAD−M (30)	CDI												
Malawi	iLiNS−DOSE (31)	CDI												
Mali	PROMIS CS (32)	DMC	528	552	9.8	0.85 (0.55, 1.31)	0.18	0.18	357	378	14.0	0.70 (0.44, 1.11)	0.17	0.18
Zimbabwe	SHINE (HIV−) (33)	CDI	220	212	10.4	0.92 (0.53, 1.59)	0.11	0.14	125	122	12.3	1.17 (0.62, 2.22)	0.09	0.13
Zimbabwe	SHINE (HIV+) (34)	CDI												
			**2266**	**3676**		**I² = 0.43, Tau² = 0.05**			**2445**	**2692**		**I² = 0.59, Tau² = 0.07**		
**Fixed**						**0.80 (0.66, 0.96)**						**0.76 (0.62, 0.92)**		
**Random**						**0.80 (0.61, 1.03)**						**0.78 (0.58, 1.06)**		

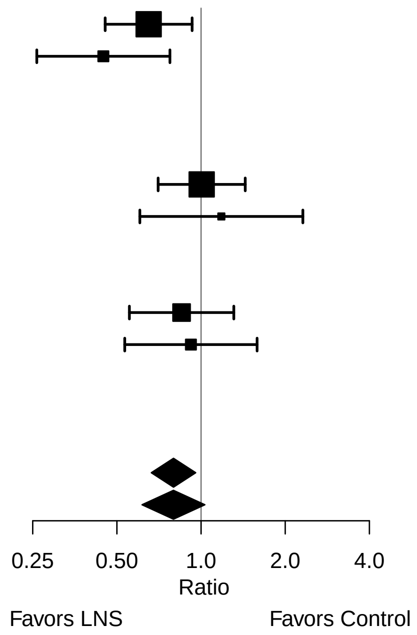

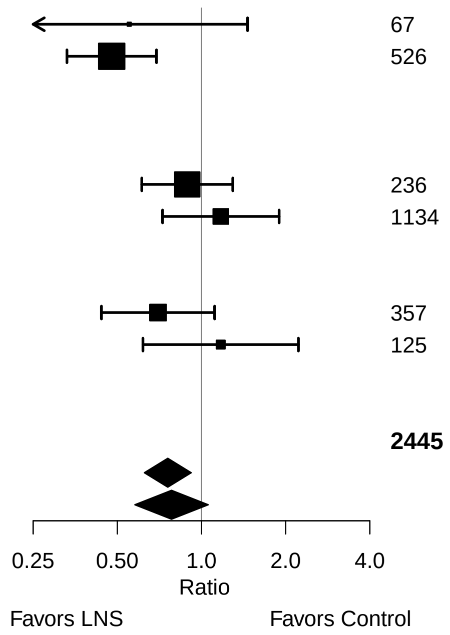

Supplemental figure 8B: Language lowest decile prevalence ratio

8B4: Stratified by Household sanitation

P−for−interaction = 0.821
Ratio of PRs = 1.04 (0.72, 1.50)

Country	Trial	Tool	LNS N	Control N	Control Prevalence	Improved PR (95% CI)	Fixed W	Random W	LNS N	Control N	Control Prevalence	Unimproved PR (95% CI)	Fixed W	Random W
Bangladesh	JiVitA−4 (21)	BSID−III												
Bangladesh	RDNS (22)	CDI	1179	572	10.8	0.73 (0.54, 0.98)	0.57	0.57	482	241	16.2	0.71 (0.48, 1.03)	0.47	0.41
Bangladesh	WASH−B (23)	CDI												
Burkina Faso	iLiNS−Zinc (24)	DMC												
Ghana	GHANA (25)													
Ghana	iLiNS−DYAD−G (26)	CDI												
Haiti	HAITI (27)	Total sounds												
Kenya	WASH−B (28)	EASQ												
Madagascar	MAHAY (29)	ASQI												
Malawi	iLiNS−DYAD−M (30)	CDI												
Malawi	iLiNS−DOSE (31)	CDI												
Mali	PROMIS CS (32)	DMC	687	678	10.5	0.83 (0.57, 1.21)	0.36	0.36	211	242	12.4	0.69 (0.37, 1.27)	0.18	0.24
Zimbabwe	SHINE (HIV−) (33)	CDI	127	106	9.4	0.58 (0.26, 1.33)	0.07	0.07	217	229	11.8	1.21 (0.78, 1.89)	0.35	0.35
Zimbabwe	SHINE (HIV+) (34)	CDI												
			**1993**	**1356**		**I² = 0.00, Tau² = 0.00**			**910**	**712**		**I² = 0.48, Tau² = 0.05**		
**Fixed**						**0.75 (0.60, 0.94)**						**0.85 (0.65, 1.10)**		
**Random**						**0.75 (0.60, 0.94)**						**0.85 (0.59, 1.22)**		

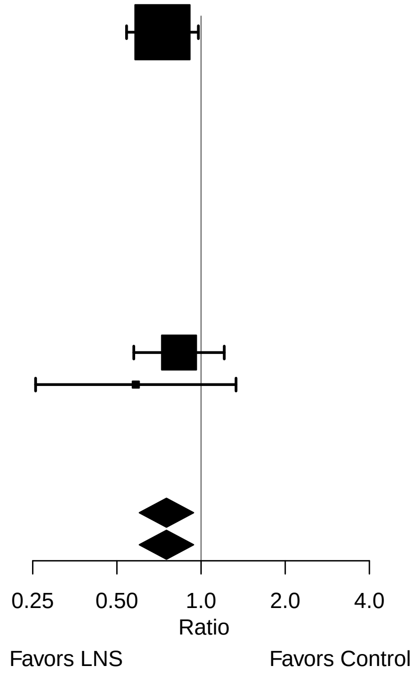

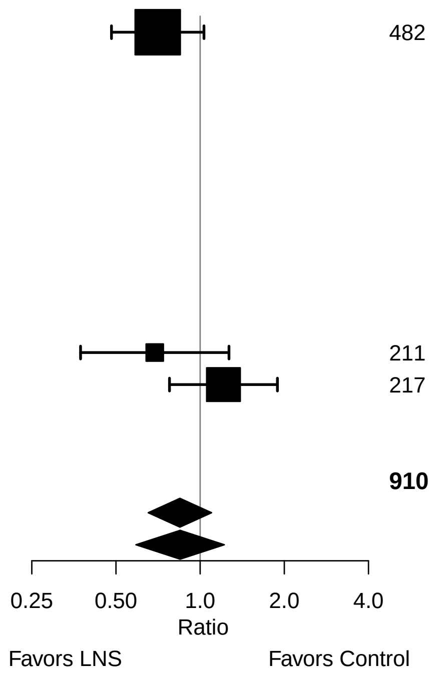

Supplemental figure 8B: Language lowest decile prevalence ratio

8B5: Stratified by Home environment

P−for−interaction = 0.592
Ratio of PRs = 0.95 (0.78, 1.16)

Country	Trial	Tool	LNS N	Control N	Control Prevalence	At least median PR (95% CI)	Fixed W	Random W	LNS N	Control N	Control Prevalence	Less than median PR (95% CI)	Fixed W	Random W
Bangladesh	JiVitA−4 (21)	BSID−III												
Bangladesh	RDNS (22)	CDI	1192	581	9.0	0.60 (0.42, 0.85)	0.18	0.18	468	230	20.4	0.88 (0.58, 1.32)	0.12	0.13
Bangladesh	WASH−B (23)	CDI	630	1652	5.9	0.73 (0.51, 1.05)	0.16	0.16	479	1700	15.1	0.93 (0.71, 1.21)	0.27	0.17
Burkina Faso	iLiNS−Zinc (24)	DMC	473	199	5.5	0.92 (0.60, 1.41)	0.12	0.13	272	175	27.4	0.40 (0.26, 0.63)	0.10	0.12
Ghana	GHANA (25)													
Ghana	iLiNS−DYAD−G (26)	CDI	213	410	6.3	1.04 (0.55, 1.94)	0.05	0.07	118	248	15.3	1.16 (0.71, 1.89)	0.08	0.11
Haiti	HAITI (27)	Total sounds												
Kenya	WASH−B (28)	EASQ	809	2682	6.9	1.11 (0.86, 1.44)	0.32	0.26	552	2060	14.3	0.87 (0.66, 1.15)	0.25	0.17
Madagascar	MAHAY (29)	ASQI	843	839	7.7	1.07 (0.67, 1.71)	0.10	0.11	770	764	10.9	1.24 (0.72, 2.16)	0.06	0.10
Malawi	iLiNS−DYAD−M (30)	CDI	137	290	6.6	1.00 (0.47, 2.16)	0.04	0.05	78	149	18.8	0.75 (0.40, 1.43)	0.05	0.08
Malawi	iLiNS−DOSE (31)	CDI	375	135	5.9	0.81 (0.36, 1.82)	0.03	0.04	269	86	17.4	0.98 (0.58, 1.67)	0.07	0.10
Mali	PROMIS CS (32)	DMC												
Zimbabwe	SHINE (HIV−) (33)	CDI												
Zimbabwe	SHINE (HIV+) (34)	CDI												
			**4672**	**6788**		**I² = 0.30, Tau² = 0.01**			**3006**	**5412**		**I² = 0.53, Tau² = 0.06**		
**Fixed**						**0.89 (0.77, 1.03)**						**0.86 (0.75, 0.99)**		
**Random**						**0.88 (0.74, 1.05)**						**0.86 (0.68, 1.08)**		

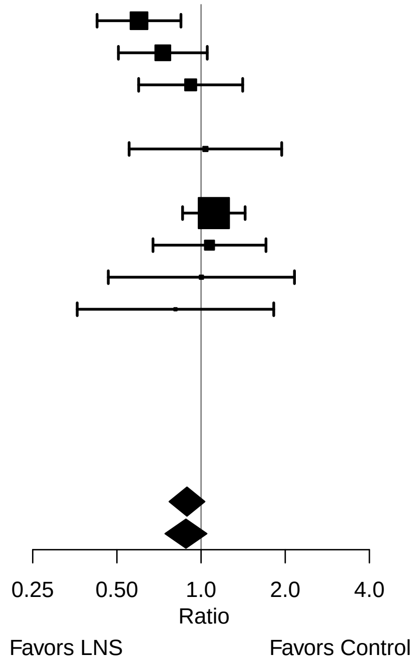

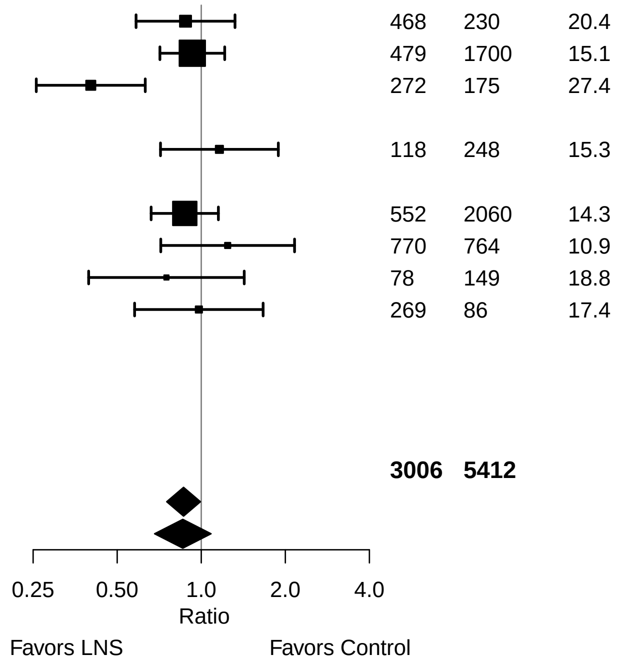

Supplemental figure 8B: Language lowest decile prevalence ratio

8B6: Stratified by Season at the time of assessment

P−for−interaction = 0.524
Ratio of PRs = 0.93 (0.76, 1.15)

Country	Trial	Tool	Dry LNS N	Dry Control N	Dry Control Prevalence	Dry PR (95% CI)	Dry Fixed W	Dry Random W	Rainy LNS N	Rainy Control N	Rainy Control Prevalence	Rainy PR (95% CI)	Rainy Fixed W	Rainy Random W
Bangladesh	JiVitA−4 (21)	BSID−III	251	70	11.4	0.59 (0.26, 1.38)	0.03	0.04	194	73	11.0	1.22 (0.62, 2.41)	0.05	0.08
Bangladesh	RDNS (22)	CDI	739	359	12.0	0.84 (0.61, 1.15)	0.18	0.16	924	455	12.7	0.63 (0.45, 0.87)	0.20	0.18
Bangladesh	WASH−B (23)	CDI	629	1944	9.4	0.92 (0.70, 1.21)	0.24	0.19	480	1409	12.1	0.69 (0.49, 0.96)	0.19	0.17
Burkina Faso	iLiNS−Zinc (24)	DMC	514	274	16.1	0.46 (0.31, 0.69)	0.11	0.12	232	101	14.9	0.46 (0.19, 1.16)	0.02	0.05
Ghana	GHANA (25)													
Ghana	iLiNS−DYAD−G (26)	CDI	194	388	10.1	0.77 (0.44, 1.36)	0.06	0.07	137	270	9.3	1.58 (0.91, 2.74)	0.07	0.11
Haiti	HAITI (27)	Total sounds												
Kenya	WASH−B (28)	EASQ	315	1059	13.5	1.01 (0.69, 1.48)	0.13	0.13	1046	3682	9.2	0.91 (0.72, 1.15)	0.38	0.21
Madagascar	MAHAY (29)	ASQI												
Malawi	iLiNS−DYAD−M (30)	CDI	153	311	10.3	0.95 (0.53, 1.71)	0.05	0.07	62	127	11.8	0.68 (0.26, 1.79)	0.02	0.05
Malawi	iLiNS−DOSE (31)	CDI	394	118	12.7	0.72 (0.41, 1.27)	0.06	0.07	244	100	8.0	1.38 (0.65, 2.94)	0.04	0.07
Mali	PROMIS CS (32)	DMC												
Zimbabwe	SHINE (HIV−) (33)	CDI	582	555	11.4	0.92 (0.65, 1.30)	0.15	0.15	235	226	9.3	0.82 (0.40, 1.69)	0.04	0.08
Zimbabwe	SHINE (HIV+) (34)	CDI												
			**3771**	**5078**		**I² = 0.28, Tau² = 0.02**			**3554**	**6443**		**I² = 0.45, Tau² = 0.05**		
**Fixed**						**0.82 (0.72, 0.94)**						**0.83 (0.72, 0.96)**		
**Random**						**0.81 (0.69, 0.95)**						**0.86 (0.68, 1.09)**		

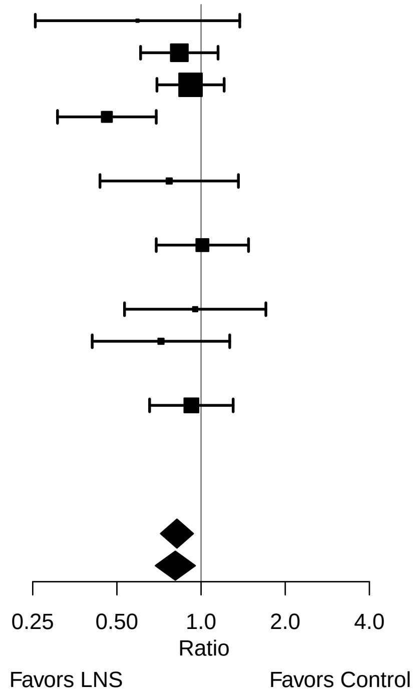

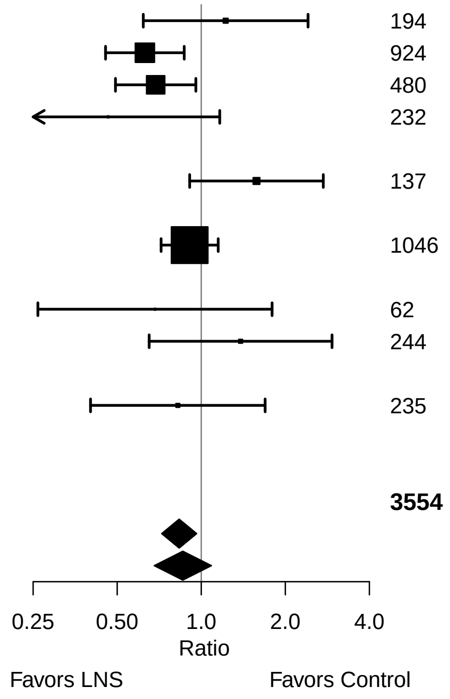

Supplemental figure 8C: Language lowest decile prevalence difference

8C1: Stratified by Household socio-economic status

P−for−interaction = 0.078
Difference in PDs = −0.02 (−0.03, 0.00)

Country	Trial	Tool	LNS N	Control N	Control Prevalence	At least median PD (95% CI)	Fixed W	Random W	LNS N	Control N	Control Prevalence	Less than median PD (95% CI)	Fixed W	Random W
Bangladesh	JiVitA−4 (21)	BSID−III	236	77	7.8	0.00 (−0.07, 0.07)	0.03	0.04	208	66	15.2	−0.04 (−0.13, 0.05)	0.02	0.03
Bangladesh	RDNS (22)	CDI	834	407	9.3	−0.03 (−0.07, 0.00)	0.11	0.12	829	407	15.5	−0.04 (−0.08, 0.00)	0.12	0.12
Bangladesh	WASH−B (23)	CDI	568	1703	7.8	−0.01 (−0.03, 0.02)	0.24	0.17	541	1650	13.4	−0.03 (−0.07, 0.00)	0.17	0.16
Burkina Faso	iLiNS−Zinc (24)	DMC	459	163	15.3	−0.08 (−0.13, −0.02)	0.05	0.06	287	212	16.0	−0.09 (−0.14, −0.05)	0.08	0.08
Ghana	GHANA (25)													
Ghana	iLiNS−DYAD−G (26)	CDI	152	341	7.9	0.00 (−0.05, 0.05)	0.05	0.07	179	315	11.7	0.01 (−0.05, 0.07)	0.05	0.06
Haiti	HAITI (27)	Total sounds												
Kenya	WASH−B (28)	EASQ	778	2706	8.5	0.01 (−0.02, 0.03)	0.23	0.16	582	2036	12.2	−0.02 (−0.05, 0.01)	0.24	0.21
Madagascar	MAHAY (29)	ASQI	741	845	5.0	0.02 (−0.02, 0.05)	0.09	0.10	823	724	14.2	0.00 (−0.06, 0.07)	0.04	0.05
Malawi	iLiNS−DYAD−M (30)	CDI	118	230	9.1	−0.02 (−0.08, 0.04)	0.03	0.05	97	208	12.5	0.00 (−0.08, 0.08)	0.03	0.03
Malawi	iLiNS−DOSE (31)	CDI	278	94	9.6	0.01 (−0.06, 0.08)	0.03	0.04	271	96	11.5	−0.01 (−0.09, 0.06)	0.04	0.04
Mali	PROMIS CS (32)	DMC	494	450	9.6	−0.01 (−0.06, 0.03)	0.07	0.09	433	494	13.0	−0.03 (−0.08, 0.01)	0.09	0.09
Zimbabwe	SHINE (HIV−) (33)	CDI	396	351	10.3	−0.02 (−0.06, 0.03)	0.06	0.08	375	383	12.0	−0.01 (−0.05, 0.03)	0.11	0.12
Zimbabwe	SHINE (HIV+) (34)	CDI	86	77	15.6	−0.09 (−0.19, 0.02)	0.01	0.02	74	64	9.4	0.01 (−0.09, 0.12)	0.02	0.02
			**5140**	**7444**		**I² = 0.28, Tau² = 0.00**			**4699**	**6655**		**I² = 0.10, Tau² = 0.00**		
**Fixed**						**−0.01 (−0.02, 0.00)**						**−0.03 (−0.04, −0.01)**		
**Random**						**−0.01 (−0.03, 0.00)**						**−0.03 (−0.04, −0.01)**		

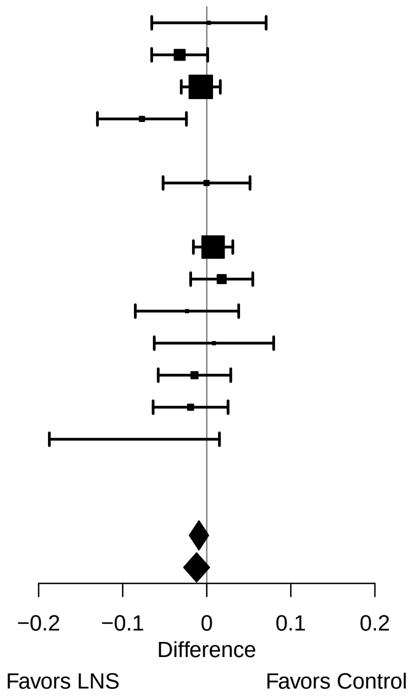

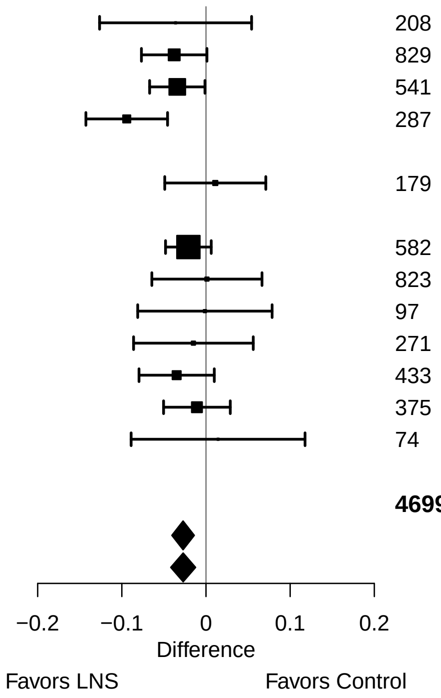

Supplemental figure 8C: Language lowest decile prevalence difference

8C2: Stratified by Household food insecurity

P−for−interaction = 0.269
Difference in PDs = −0.01 (−0.04, 0.01)

Country	Trial	Tool	LNS N	Control N	Control Prevalence	Mild to secure PD (95% CI)	Fixed W	Random W	LNS N	Control N	Control Prevalence	Moderate to severe PD (95% CI)	Fixed W	Random W
Bangladesh	JiVitA−4 (21)	BSID−III												
Bangladesh	RDNS (22)	CDI	1058	493	9.9	−0.03 (−0.06, 0.00)	0.12	0.14	605	321	16.2	−0.05 (−0.09, 0.00)	0.21	0.18
Bangladesh	WASH−B (23)	CDI	883	2608	9.6	−0.02 (−0.04, 0.00)	0.27	0.15	226	745	13.7	−0.02 (−0.07, 0.03)	0.14	0.14
Burkina Faso	iLiNS−Zinc (24)	DMC	389	162	15.4	−0.09 (−0.13, −0.05)	0.06	0.12	357	213	16.0	−0.08 (−0.12, −0.03)	0.18	0.16
Ghana	GHANA (25)													
Ghana	iLiNS−DYAD−G (26)	CDI	238	453	8.4	0.04 (−0.01, 0.08)	0.05	0.11	92	202	12.9	−0.06 (−0.14, 0.01)	0.06	0.08
Haiti	HAITI (27)	Total sounds												
Kenya	WASH−B (28)	EASQ	1214	4244	10.0	−0.01 (−0.03, 0.01)	0.33	0.15	146	487	11.3	0.04 (−0.03, 0.10)	0.08	0.10
Madagascar	MAHAY (29)	ASQI	940	892	8.0	0.03 (−0.02, 0.08)	0.04	0.10	336	389	12.6	−0.01 (−0.09, 0.07)	0.06	0.08
Malawi	iLiNS−DYAD−M (30)	CDI	68	118	9.3	0.04 (−0.05, 0.13)	0.01	0.05	146	315	10.5	−0.03 (−0.09, 0.03)	0.11	0.12
Malawi	iLiNS−DOSE (31)	CDI	133	51	9.8	−0.02 (−0.11, 0.07)	0.01	0.05	403	133	10.5	0.00 (−0.06, 0.06)	0.10	0.11
Mali	PROMIS CS (32)	DMC												
Zimbabwe	SHINE (HIV−) (33)	CDI	612	576	10.6	−0.02 (−0.05, 0.01)	0.10	0.13	136	148	14.2	0.01 (−0.09, 0.10)	0.04	0.05
Zimbabwe	SHINE (HIV+) (34)	CDI												
			**5535**	**9597**		**I² = 0.66, Tau² = 0.00**			**2447**	**2953**		**I² = 0.32, Tau² = 0.00**		
**Fixed**						**−0.02 (−0.03, −0.01)**						**−0.03 (−0.05, −0.01)**		
**Random**						**−0.01 (−0.04, 0.01)**						**−0.03 (−0.05, −0.01)**		

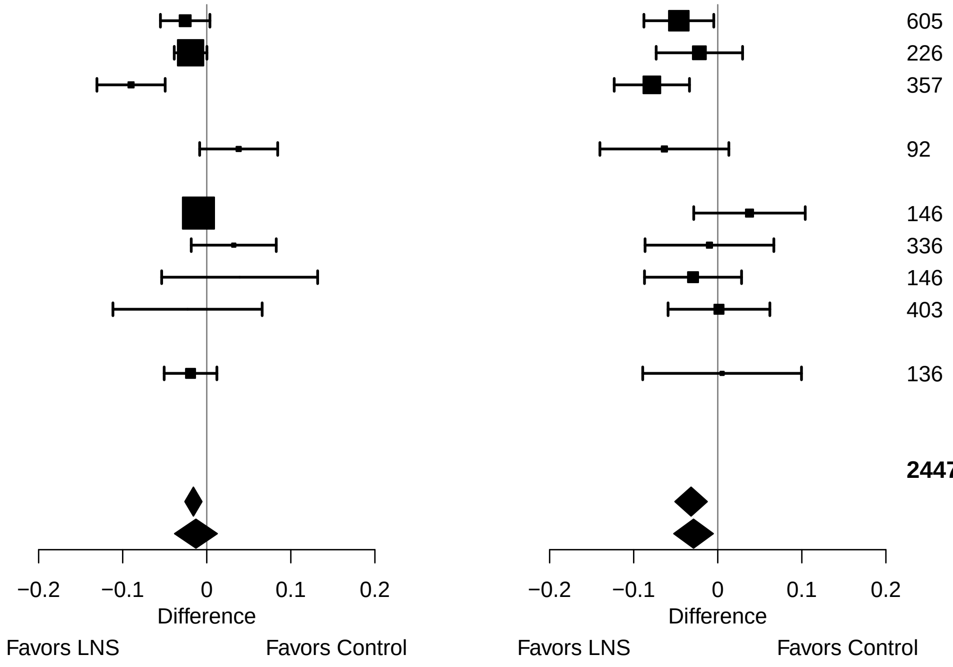

Supplemental figure 8C: Language lowest decile prevalence difference

8C3: Stratified by Household source water quality

P−for−interaction = 0.321
Difference in PDs = −0.01 (−0.04, 0.01)

Country	Trial	Tool	LNS N	Control N	Control Prevalence	Improved PD (95% CI)	Fixed W	Random W	LNS N	Control N	Control Prevalence	Unimproved PD (95% CI)	Fixed W	Random W
Bangladesh	JiVitA−4 (21)	BSID−III												
Bangladesh	RDNS (22)	CDI												
Bangladesh	WASH−B (23)	CDI	480	981	10.9	−0.04 (−0.07, −0.01)	0.36	0.28	67	117	16.2	−0.07 (−0.19, 0.05)	0.03	0.07
Burkina Faso	iLiNS−Zinc (24)	DMC	220	82	12.2	−0.07 (−0.12, −0.02)	0.11	0.14	526	293	16.7	−0.09 (−0.13, −0.05)	0.25	0.21
Ghana	GHANA (25)													
Ghana	iLiNS−DYAD−G (26)	CDI												
Haiti	HAITI (27)	Total sounds												
Kenya	WASH−B (28)	EASQ	413	1419	10.4	0.00 (−0.04, 0.04)	0.21	0.21	236	649	10.5	−0.01 (−0.05, 0.02)	0.30	0.22
Madagascar	MAHAY (29)	ASQI	405	430	12.3	0.02 (−0.07, 0.11)	0.03	0.05	1134	1133	8.1	0.01 (−0.03, 0.06)	0.23	0.21
Malawi	iLiNS−DYAD−M (30)	CDI												
Malawi	iLiNS−DOSE (31)	CDI												
Mali	PROMIS CS (32)	DMC	528	552	9.8	−0.01 (−0.05, 0.02)	0.19	0.20	357	378	14.0	−0.04 (−0.10, 0.01)	0.14	0.18
Zimbabwe	SHINE (HIV−) (33)	CDI	220	212	10.4	−0.01 (−0.06, 0.05)	0.10	0.12	125	122	12.3	0.02 (−0.06, 0.11)	0.06	0.11
Zimbabwe	SHINE (HIV+) (34)	CDI												
			**2266**	**3676**		**I^2 = 0.29, Tau^2 = 0.00**			**2445**	**2692**		**I^2 = 0.67, Tau^2 = 0.00**		
**Fixed**						**−0.02 (−0.04, −0.01)**						**−0.03 (−0.05, −0.01)**		
**Random**						**−0.02 (−0.04, 0.00)**						**−0.03 (−0.06, 0.01)**		

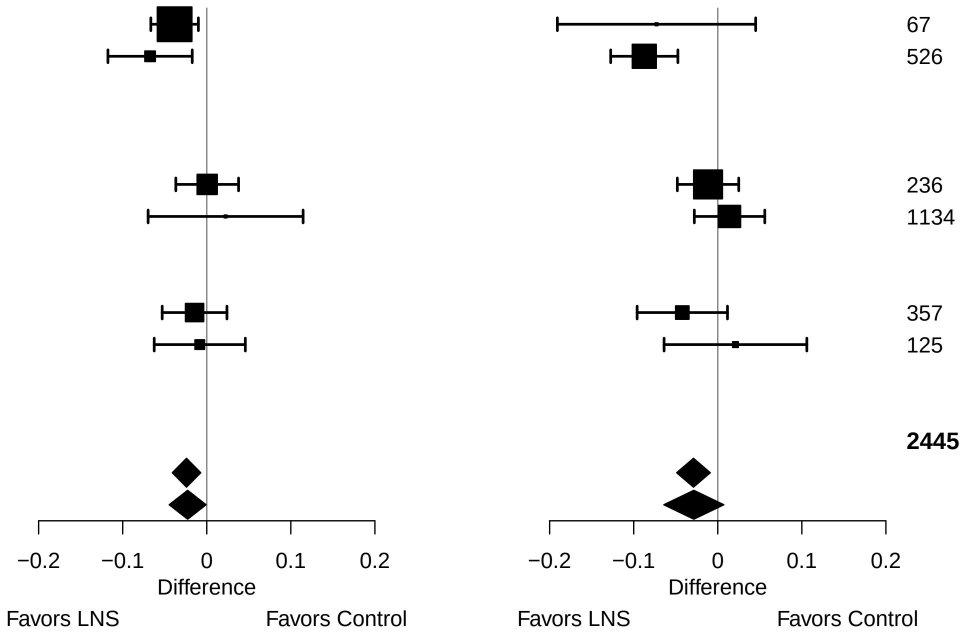

Supplemental figure 8C: Language lowest decile prevalence difference

8C4: Stratified by Household sanitation

P−for−interaction = 0.848
Difference in PDs = 0.00 (−0.04, 0.04)

Country	Trial	Tool	LNS N	Control N	Control Prevalence	Improved PD (95% CI)	Fixed W	Random W	LNS N	Control N	Control Prevalence	Unimproved PD (95% CI)	Fixed W	Random W
Bangladesh	JiVitA−4 (21)	BSID−III												
Bangladesh	RDNS (22)	CDI	1179	572	10.8	−0.03 (−0.06, 0.00)	0.57	0.57	482	241	16.2	−0.05 (−0.10, 0.00)	0.39	0.37
Bangladesh	WASH−B (23)	CDI												
Burkina Faso	iLiNS−Zinc (24)	DMC												
Ghana	GHANA (25)													
Ghana	iLiNS−DYAD−G (26)	CDI												
Haiti	HAITI (27)	Total sounds												
Kenya	WASH−B (28)	EASQ												
Madagascar	MAHAY (29)	ASQI												
Malawi	iLiNS−DYAD−M (30)	CDI												
Malawi	iLiNS−DOSE (31)	CDI												
Mali	PROMIS CS (32)	DMC	687	678	10.5	−0.02 (−0.05, 0.02)	0.33	0.33	211	242	12.4	−0.04 (−0.10, 0.02)	0.28	0.31
Zimbabwe	SHINE (HIV−) (33)	CDI	127	106	9.4	−0.04 (−0.10, 0.02)	0.10	0.10	217	229	11.8	0.02 (−0.03, 0.08)	0.32	0.33
Zimbabwe	SHINE (HIV+) (34)	CDI												
			**1993**	**1356**		**I² = 0.00, Tau² = 0.00**			**910**	**712**		**I² = 0.46, Tau² = 0.00**		
**Fixed**						**−0.03 (−0.05, −0.01)**						**−0.02 (−0.05, 0.01)**		
**Random**						**−0.03 (−0.05, −0.01)**						**−0.02 (−0.07, 0.02)**		

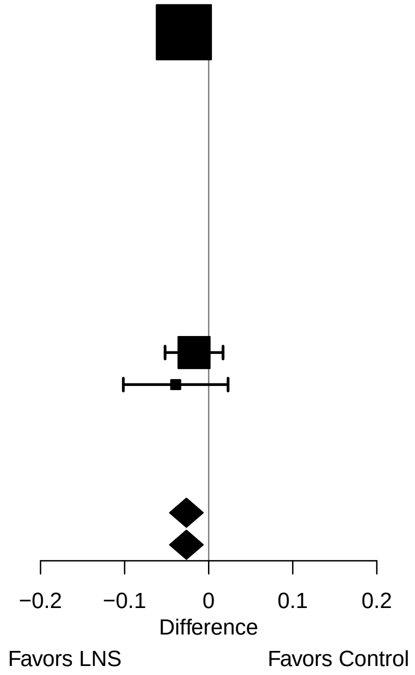

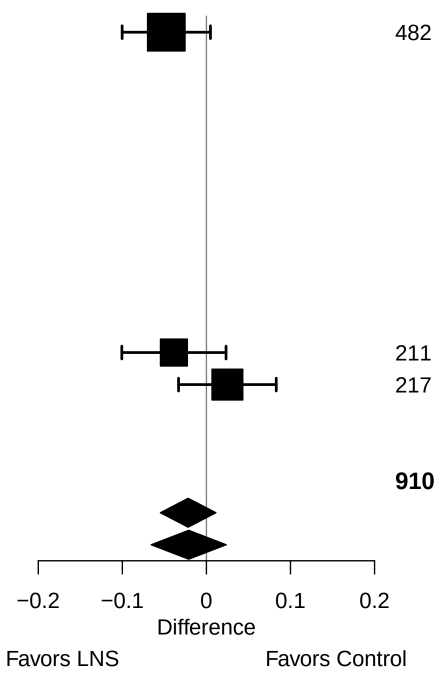

Supplemental figure 8C: Language lowest decile prevalence difference

8C5: Stratified by Home environment

P−for−interaction = 0.364
Difference in PDs = −0.01 (−0.03, 0.01)

Country	Trial	Tool	At least median: LNS N	Control N	Control Prevalence	PD (95% CI)	Fixed W	Random W	Less than median: LNS N	Control N	Control Prevalence	PD (95% CI)	Fixed W	Random W
Bangladesh	JiVitA−4 (21)	BSID−III												
Bangladesh	RDNS (22)	CDI	1192	581	9.0	−0.04 (−0.06, −0.01)	0.14	0.15	468	230	20.4	−0.02 (−0.10, 0.05)	0.07	0.11
Bangladesh	WASH−B (23)	CDI	630	1652	5.9	−0.02 (−0.03, 0.00)	0.30	0.22	479	1700	15.1	−0.01 (−0.05, 0.03)	0.29	0.18
Burkina Faso	iLiNS−Zinc (24)	DMC	473	199	5.5	0.00 (−0.03, 0.02)	0.16	0.16	272	175	27.4	−0.16 (−0.25, −0.08)	0.06	0.10
Ghana	GHANA (25)													
Ghana	iLiNS−DYAD−G (26)	CDI	213	410	6.3	0.00 (−0.04, 0.04)	0.05	0.07	118	248	15.3	0.02 (−0.06, 0.11)	0.06	0.11
Haiti	HAITI (27)	Total sounds												
Kenya	WASH−B (28)	EASQ	809	2682	6.9	0.01 (−0.01, 0.03)	0.22	0.19	552	2060	14.3	−0.02 (−0.05, 0.02)	0.34	0.18
Madagascar	MAHAY (29)	ASQI	843	839	7.7	0.01 (−0.03, 0.04)	0.06	0.08	770	764	10.9	0.03 (−0.04, 0.09)	0.10	0.13
Malawi	iLiNS−DYAD−M (30)	CDI	137	290	6.6	0.00 (−0.05, 0.05)	0.03	0.05	78	149	18.8	−0.05 (−0.15, 0.06)	0.04	0.08
Malawi	iLiNS−DOSE (31)	CDI	375	135	5.9	−0.01 (−0.05, 0.03)	0.04	0.07	269	86	17.4	0.00 (−0.10, 0.09)	0.05	0.10
Mali	PROMIS CS (32)	DMC												
Zimbabwe	SHINE (HIV−) (33)	CDI												
Zimbabwe	SHINE (HIV+) (34)	CDI												
			**4672**	**6788**		**I² = 0.24, Tau² = 0.00**			**3006**	**5412**		**I² = 0.51, Tau² = 0.00**		
**Fixed**						**−0.01 (−0.02, 0.00)**						**−0.02 (−0.04, 0.00)**		
**Random**						**−0.01 (−0.02, 0.00)**						**−0.02 (−0.06, 0.02)**		

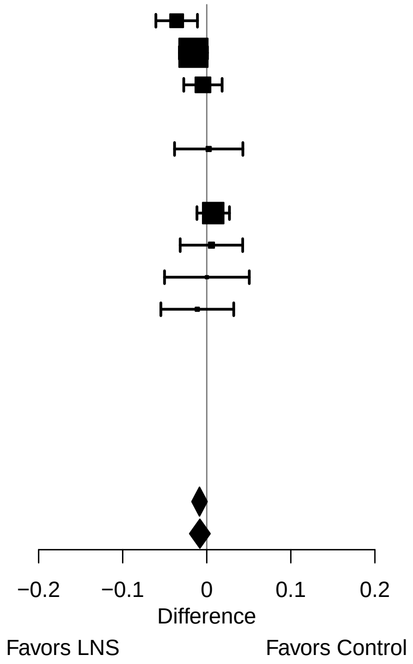

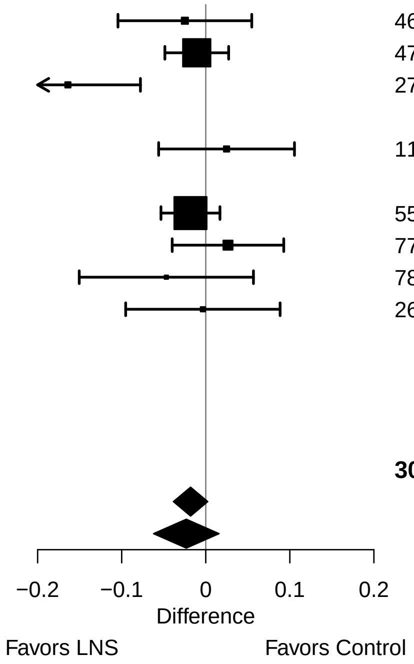

Supplemental figure 8C: Language lowest decile prevalence difference

8C6: Stratified by Season at the time of assessment

P−for−interaction = 0.556
Difference in PDs = −0.01 (−0.03, 0.01)

Country	Trial	Tool	Dry LNS N	Dry Control N	Dry Control Prevalence	Dry PD (95% CI)	Dry Fixed W	Dry Random W	Rainy LNS N	Rainy Control N	Rainy Control Prevalence	Rainy PD (95% CI)	Rainy Fixed W	Rainy Random W
Bangladesh	JiVitA−4 (21)	BSID−III	251	70	11.4	−0.05 (−0.13, 0.04)	0.02	0.04	194	73	11.0	0.02 (−0.06, 0.10)	0.03	0.07
Bangladesh	RDNS (22)	CDI	739	359	12.0	−0.02 (−0.05, 0.02)	0.16	0.15	924	455	12.7	−0.05 (−0.08, −0.01)	0.15	0.17
Bangladesh	WASH−B (23)	CDI	629	1944	9.4	−0.01 (−0.03, 0.02)	0.33	0.22	480	1409	12.1	−0.04 (−0.07, −0.01)	0.21	0.19
Burkina Faso	iLiNS−Zinc (24)	DMC	514	274	16.1	−0.09 (−0.13, −0.04)	0.10	0.12	232	101	14.9	−0.08 (−0.19, 0.04)	0.01	0.04
Ghana	GHANA (25)													
Ghana	iLiNS−DYAD−G (26)	CDI	194	388	10.1	−0.02 (−0.07, 0.03)	0.08	0.10	137	270	9.3	0.05 (−0.01, 0.12)	0.04	0.09
Haiti	HAITI (27)	Total sounds												
Kenya	WASH−B (28)	EASQ	315	1059	13.5	0.00 (−0.05, 0.05)	0.07	0.09	1046	3682	9.2	−0.01 (−0.03, 0.01)	0.45	0.22
Madagascar	MAHAY (29)	ASQI												
Malawi	iLiNS−DYAD−M (30)	CDI	153	311	10.3	0.00 (−0.06, 0.05)	0.06	0.08	62	127	11.8	−0.04 (−0.13, 0.06)	0.02	0.05
Malawi	iLiNS−DOSE (31)	CDI	394	118	12.7	−0.04 (−0.10, 0.03)	0.05	0.07	244	100	8.0	0.03 (−0.04, 0.10)	0.04	0.08
Mali	PROMIS CS (32)	DMC												
Zimbabwe	SHINE (HIV−) (33)	CDI	582	555	11.4	−0.01 (−0.05, 0.03)	0.14	0.14	235	226	9.3	−0.02 (−0.08, 0.04)	0.05	0.10
Zimbabwe	SHINE (HIV+) (34)	CDI												
			**3771**	**5078**		**I² = 0.32, Tau² = 0.00**			**3554**	**6443**		**I² = 0.44, Tau² = 0.00**		
**Fixed**						**−0.02 (−0.03, −0.01)**						**−0.02 (−0.03, 0.00)**		
**Random**						**−0.02 (−0.04, 0.00)**						**−0.01 (−0.04, 0.01)**		

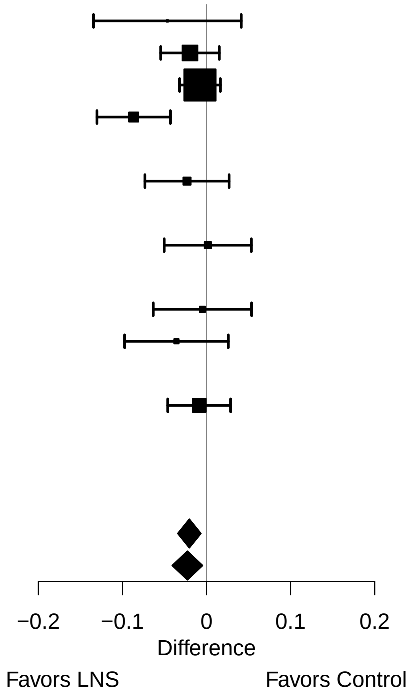

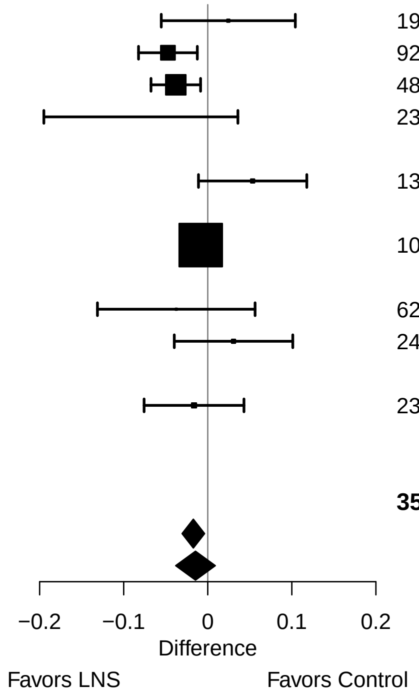

Supplemental figure 8D: Mean difference in social-emotional z-score

8D1: Stratified by Household socio-economic status

P−for−interaction = 0.982
Difference in MDs = 0.00 (−0.06, 0.06)

Country	Trial	Tool	LNS N	Control N	Control Mean	At least median MD (95% CI)	Fixed W	Random W	LNS N	Control N	Control Mean	Less than median MD (95% CI)	Fixed W	Random W
Bangladesh	JiVitA−4 (21)													
Bangladesh	RDNS (22)	DMC	830	407	0.00	0.09 (−0.06, 0.23)	0.09	0.11	827	408	−0.07	0.04 (−0.09, 0.16)	0.14	0.14
Bangladesh	WASH−B (23)	EASQ	545	1659	0.11	0.09 (−0.01, 0.19)	0.19	0.12	521	1607	−0.17	0.16 (0.05, 0.26)	0.20	0.17
Burkina Faso	iLiNS−Zinc (24)	DMC	459	163	−0.25	0.41 (0.14, 0.69)	0.03	0.06	287	212	−0.22	0.28 (0.05, 0.51)	0.04	0.05
Ghana	GHANA (25)													
Ghana	iLiNS−DYAD−G (26)	PSED	153	341	0.00	0.00 (−0.19, 0.19)	0.05	0.09	179	314	0.00	0.01 (−0.17, 0.20)	0.06	0.08
Haiti	HAITI (27)													
Kenya	WASH−B (28)	EASQ	778	2706	0.06	0.01 (−0.07, 0.08)	0.32	0.13	582	2036	−0.08	0.02 (−0.07, 0.11)	0.25	0.20
Madagascar	MAHAY (29)	ASQI	741	845	0.20	−0.06 (−0.24, 0.12)	0.06	0.09	823	724	−0.16	−0.04 (−0.26, 0.18)	0.04	0.05
Malawi	iLiNS−DYAD−M (30)	PSED	118	230	0.00	0.08 (−0.15, 0.31)	0.04	0.07	97	207	−0.02	−0.06 (−0.29, 0.18)	0.04	0.05
Malawi	iLiNS−DOSE (31)	PSED	278	94	−0.02	0.00 (−0.23, 0.22)	0.04	0.07	270	96	0.01	0.06 (−0.18, 0.30)	0.04	0.05
Mali	PROMIS CS (32)	DMC	494	450	0.03	0.05 (−0.14, 0.25)	0.05	0.08	433	494	−0.17	0.23 (0.03, 0.42)	0.05	0.07
Zimbabwe	SHINE (HIV−) (33)	MDAT	406	359	−0.07	0.22 (0.08, 0.35)	0.10	0.11	389	391	−0.08	0.07 (−0.07, 0.21)	0.11	0.11
Zimbabwe	SHINE (HIV+) (34)	MDAT	91	78	−0.23	0.45 (0.18, 0.72)	0.03	0.06	74	67	−0.07	0.19 (−0.12, 0.49)	0.02	0.03
			**4893**	**7332**		**I² = 0.57, Tau² = 0.01**			**4482**	**6556**		**I² = 0.24, Tau² = 0.00**		
**Fixed**						**0.07 (0.03, 0.12)**						**0.08 (0.03, 0.12)**		
**Random**						**0.10 (0.01, 0.19)**						**0.08 (0.02, 0.13)**		

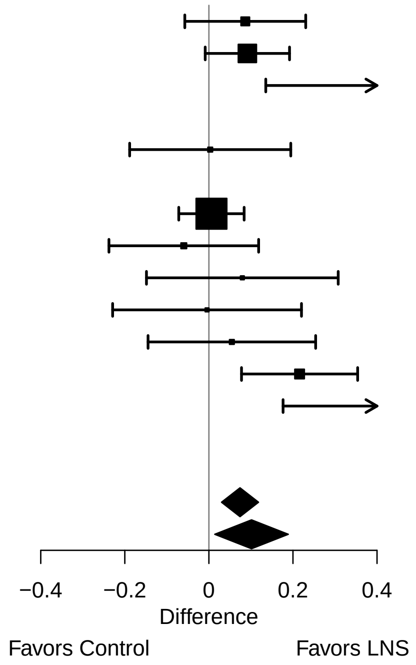

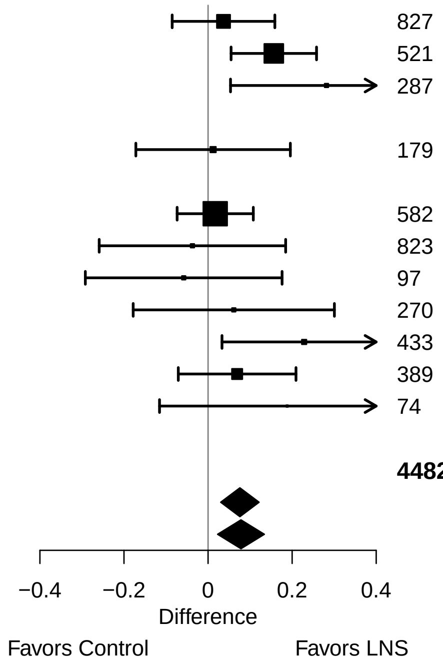

Supplemental figure 8D: Mean difference in social-emotional z-score

8D2: Stratified by Household food insecurity

P−for−interaction = 0.347
Difference in MDs = −0.03 (−0.10, 0.04)

Country	Trial	Tool	LNS N	Control N	Control Mean	Mild to secure MD (95% CI)	Fixed W	Random W	LNS N	Control N	Control Mean	Moderate to severe MD (95% CI)	Fixed W	Random W
Bangladesh	JiVitA−4 (21)													
Bangladesh	RDNS (22)	DMC	1054	493	0.02	0.07 (−0.04, 0.18)	0.12	0.12	603	322	−0.11	0.04 (−0.08, 0.16)	0.23	0.17
Bangladesh	WASH−B (23)	EASQ	853	2539	0.01	0.13 (0.05, 0.21)	0.25	0.13	213	727	−0.17	0.08 (−0.07, 0.24)	0.14	0.13
Burkina Faso	iLiNS−Zinc (24)	DMC	389	162	−0.22	0.36 (0.09, 0.64)	0.02	0.08	357	213	−0.25	0.34 (0.17, 0.51)	0.12	0.12
Ghana	GHANA (25)													
Ghana	iLiNS−DYAD−G (26)	PSED	239	452	0.03	0.00 (−0.15, 0.15)	0.06	0.11	92	202	−0.08	0.02 (−0.23, 0.28)	0.05	0.07
Haiti	HAITI (27)													
Kenya	WASH−B (28)	EASQ	1214	4244	0.00	0.02 (−0.05, 0.08)	0.36	0.13	146	487	−0.07	−0.03 (−0.22, 0.15)	0.10	0.11
Madagascar	MAHAY (29)	ASQI	940	892	0.06	−0.08 (−0.29, 0.13)	0.03	0.09	336	389	−0.11	−0.05 (−0.28, 0.18)	0.07	0.08
Malawi	iLiNS−DYAD−M (30)	PSED	68	118	0.06	−0.11 (−0.43, 0.20)	0.02	0.07	146	314	−0.04	0.08 (−0.12, 0.27)	0.10	0.10
Malawi	iLiNS−DOSE (31)	PSED	134	51	0.03	−0.11 (−0.43, 0.20)	0.01	0.06	402	133	−0.04	0.10 (−0.09, 0.30)	0.09	0.10
Mali	PROMIS CS (32)	DMC												
Zimbabwe	SHINE (HIV−) (33)	MDAT	631	586	−0.09	0.18 (0.06, 0.30)	0.10	0.12	140	154	−0.02	−0.06 (−0.27, 0.15)	0.08	0.09
Zimbabwe	SHINE (HIV+) (34)	MDAT	119	105	−0.20	0.46 (0.23, 0.69)	0.03	0.09	41	37	−0.10	0.01 (−0.41, 0.44)	0.02	0.03
			**5641**	**9642**		**I² = 0.69, Tau² = 0.02**			**2476**	**2978**		**I² = 0.36, Tau² = 0.00**		
**Fixed**						**0.08 (0.04, 0.12)**						**0.07 (0.01, 0.13)**		
**Random**						**0.09 (−0.02, 0.21)**						**0.07 (−0.01, 0.14)**		

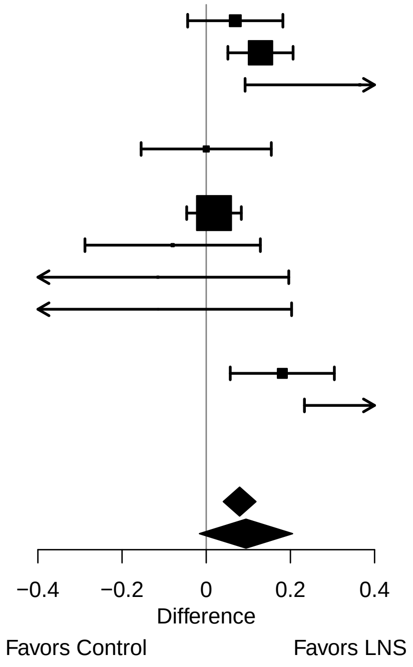

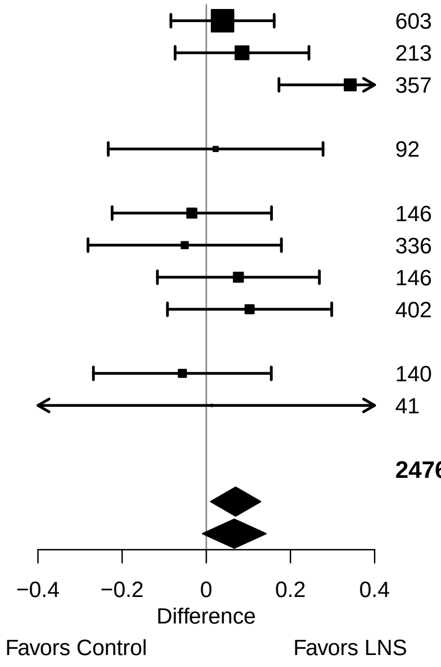

Supplemental figure 8D: Mean difference in social-emotional z-score

8D3: Stratified by Household source water quality

P−for−interaction = 0.939
Difference in MDs = 0.00 (−0.09, 0.10)

Country	Trial	Tool	LNS N	Control N	Control Mean	Improved MD (95% CI)	Fixed W	Random W	LNS N	Control N	Control Mean	Unimproved MD (95% CI)	Fixed W	Random W
Bangladesh	JiVitA−4 (21)													
Bangladesh	RDNS (22)	DMC												
Bangladesh	WASH−B (23)	EASQ	461	981	−0.17	0.22 (0.11, 0.34)	0.25	0.17	66	117	−0.33	0.26 (−0.02, 0.55)	0.07	0.10
Burkina Faso	iLiNS−Zinc (24)	DMC	220	82	−0.11	0.32 (0.10, 0.54)	0.07	0.09	526	293	−0.27	0.35 (0.17, 0.54)	0.16	0.16
Ghana	GHANA (25)													
Ghana	iLiNS−DYAD−G (26)	PSED												
Haiti	HAITI (27)													
Kenya	WASH−B (28)	EASQ	413	1419	0.02	−0.04 (−0.16, 0.09)	0.21	0.16	236	649	0.04	−0.01 (−0.15, 0.13)	0.29	0.20
Madagascar	MAHAY (29)	ASQI	405	430	−0.04	−0.05 (−0.41, 0.31)	0.03	0.05	1134	1133	0.06	−0.09 (−0.28, 0.10)	0.16	0.16
Malawi	iLiNS−DYAD−M (30)	PSED	195	400	0.01	0.01 (−0.15, 0.18)	0.12	0.13	20	36	−0.26	0.08 (−0.56, 0.72)	0.01	0.03
Malawi	iLiNS−DOSE (31)	PSED	514	177	0.00	0.03 (−0.14, 0.21)	0.11	0.12	44	15	−0.06	−0.06 (−0.58, 0.46)	0.02	0.04
Mali	PROMIS CS (32)	DMC	528	552	−0.04	0.12 (−0.09, 0.33)	0.08	0.10	357	378	−0.15	0.17 (−0.05, 0.39)	0.12	0.13
Zimbabwe	SHINE (HIV−) (33)	MDAT	228	220	−0.04	0.11 (−0.08, 0.30)	0.09	0.11	131	122	−0.13	0.09 (−0.12, 0.30)	0.12	0.14
Zimbabwe	SHINE (HIV+) (34)	MDAT	37	41	−0.25	0.30 (0.00, 0.60)	0.04	0.06	25	25	0.05	−0.07 (−0.48, 0.34)	0.03	0.06
			**3001**	**4302**		**I² = 0.53, Tau² = 0.01**			**2539**	**2768**		**I² = 0.51, Tau² = 0.01**		
**Fixed**						**0.11 (0.05, 0.16)**						**0.09 (0.01, 0.16)**		
**Random**						**0.11 (0.02, 0.20)**						**0.10 (−0.01, 0.20)**		

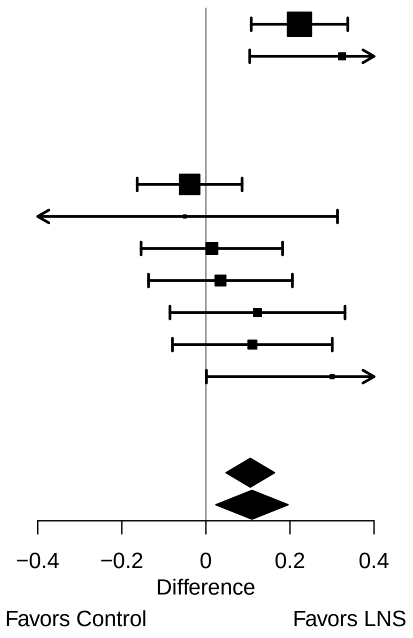

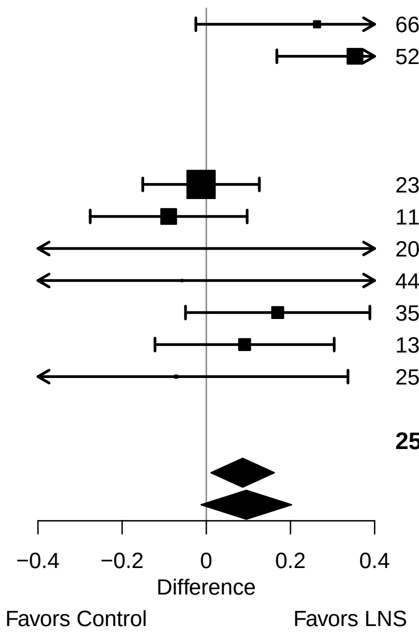

Supplemental figure 8D: Mean difference in social-emotional z-score

8D4: Stratified by Household sanitation

P−for−interaction = 0.550
Difference in MDs = −0.03 (−0.14, 0.07)

Country	Trial	Tool	LNS N	Control N	Control Mean	Improved MD (95% CI)	Fixed W	Random W	LNS N	Control N	Control Mean	Unimproved MD (95% CI)	Fixed W	Random W
Bangladesh	JiVitA−4 (21)													
Bangladesh	RDNS (22)	DMC	1174	573	−0.01	0.08 (−0.02, 0.19)	0.30	0.14	481	241	−0.09	0.01 (−0.16, 0.19)	0.11	0.12
Bangladesh	WASH−B (23)	EASQ	474	998	−0.17	0.23 (0.12, 0.34)	0.27	0.14	27	53	−0.35	0.17 (−0.28, 0.61)	0.02	0.03
Burkina Faso	iLiNS−Zinc (24)	DMC	16	11	−0.47	0.81 (0.30, 1.33)	0.01	0.06	730	364	−0.23	0.34 (0.17, 0.51)	0.12	0.13
Ghana	GHANA (25)													
Ghana	iLiNS−DYAD−G (26)	PSED	321	639	0.00	0.00 (−0.14, 0.13)	0.18	0.14	10	16	−0.10	0.25 (−0.70, 1.20)	0.00	0.01
Haiti	HAITI (27)													
Kenya	WASH−B (28)	EASQ	94	337	0.11	−0.03 (−0.29, 0.22)	0.05	0.11	555	1732	0.01	−0.02 (−0.13, 0.08)	0.30	0.21
Madagascar	MAHAY (29)	ASQI												
Malawi	iLiNS−DYAD−M (30)	PSED	19	42	0.35	−0.53 (−1.04, −0.03)	0.01	0.07	196	394	−0.05	0.07 (−0.10, 0.25)	0.12	0.13
Malawi	iLiNS−DOSE (31)	PSED	12	9	−0.09	0.37 (−0.35, 1.10)	0.01	0.04	546	183	0.00	0.02 (−0.15, 0.18)	0.12	0.13
Mali	PROMIS CS (32)	DMC	687	678	−0.05	0.15 (−0.04, 0.34)	0.09	0.12	211	242	−0.10	0.11 (−0.12, 0.33)	0.07	0.09
Zimbabwe	SHINE (HIV−) (33)	MDAT	133	108	−0.06	0.07 (−0.16, 0.30)	0.06	0.12	225	235	−0.08	0.12 (−0.06, 0.30)	0.11	0.12
Zimbabwe	SHINE (HIV+) (34)	MDAT	25	17	−0.22	0.28 (−0.26, 0.82)	0.01	0.06	37	49	−0.11	0.10 (−0.23, 0.43)	0.03	0.04
			**2955**	**3412**		**I² = 0.61, Tau² = 0.05**			**3018**	**3509**		**I² = 0.38, Tau² = 0.00**		
**Fixed**						**0.11 (0.05, 0.17)**						**0.07 (0.01, 0.13)**		
**Random**						**0.12 (−0.06, 0.29)**						**0.09 (0.01, 0.16)**		

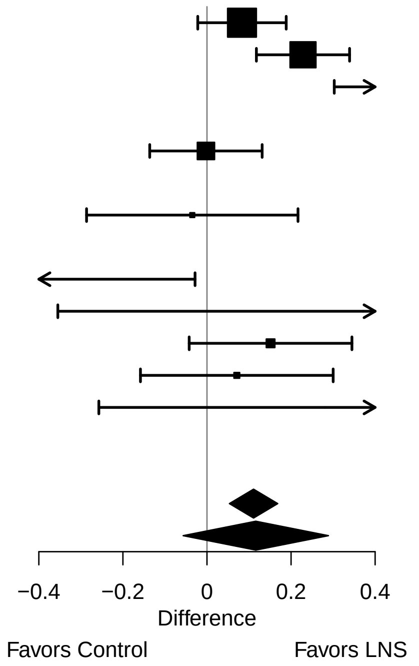

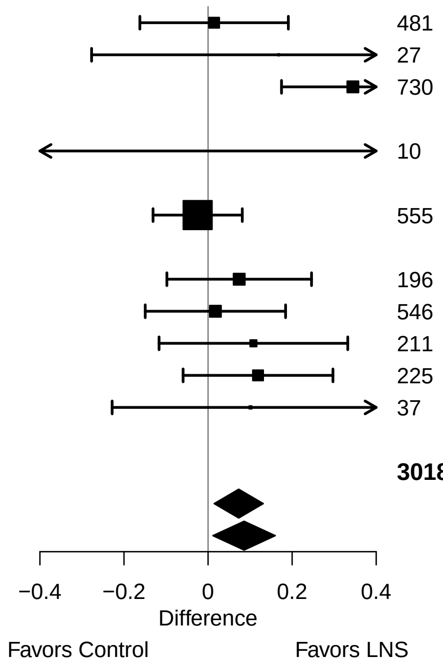

Supplemental figure 8D: Mean difference in social-emotional z-score

8D5: Stratified by Home environment

P−for−interaction = 0.363
Difference in MDs = 0.03 (−0.04, 0.10)

Country	Trial	Tool	LNS N	Control N	Control Mean	At least median MD (95% CI)	Fixed W	Random W	LNS N	Control N	Control Mean	Less than median MD (95% CI)	Fixed W	Random W
Bangladesh	JiVitA−4 (21)													
Bangladesh	RDNS (22)	DMC	1187	582	0.12	0.00 (−0.08, 0.09)	0.23	0.17	467	230	−0.39	0.20 (0.00, 0.40)	0.08	0.10
Bangladesh	WASH−B (23)	EASQ	603	1599	0.15	0.13 (0.03, 0.23)	0.18	0.16	463	1666	−0.20	0.05 (−0.05, 0.16)	0.30	0.25
Burkina Faso	iLiNS−Zinc (24)	DMC	473	199	0.00	0.28 (0.13, 0.43)	0.08	0.12	272	175	−0.50	0.35 (0.07, 0.63)	0.04	0.06
Ghana	GHANA (25)													
Ghana	iLiNS−DYAD−G (26)	PSED	213	410	−0.05	−0.02 (−0.18, 0.14)	0.07	0.11	119	247	0.08	0.07 (−0.15, 0.29)	0.06	0.09
Haiti	HAITI (27)													
Kenya	WASH−B (28)	EASQ	809	2682	0.17	−0.02 (−0.10, 0.06)	0.29	0.18	552	2060	−0.22	0.04 (−0.06, 0.13)	0.35	0.27
Madagascar	MAHAY (29)	ASQI	843	839	0.15	−0.08 (−0.28, 0.11)	0.05	0.09	770	764	−0.09	−0.07 (−0.28, 0.15)	0.07	0.09
Malawi	iLiNS−DYAD−M (30)	PSED	137	290	−0.13	0.04 (−0.17, 0.24)	0.04	0.09	78	148	0.24	−0.05 (−0.32, 0.22)	0.04	0.06
Malawi	iLiNS−DOSE (31)	PSED	376	135	−0.21	0.03 (−0.16, 0.22)	0.05	0.09	268	86	0.26	0.01 (−0.23, 0.25)	0.05	0.08
Mali	PROMIS CS (32)	DMC												
Zimbabwe	SHINE (HIV−) (33)	MDAT												
Zimbabwe	SHINE (HIV+) (34)	MDAT												
			**4641**	**6736**		**I² = 0.61, Tau² = 0.01**			**2989**	**5376**		**I² = 0.16, Tau² = 0.00**		
**Fixed**						**0.04 (0.00, 0.08)**						**0.06 (0.00, 0.11)**		
**Random**						**0.05 (−0.03, 0.12)**						**0.06 (−0.01, 0.13)**		

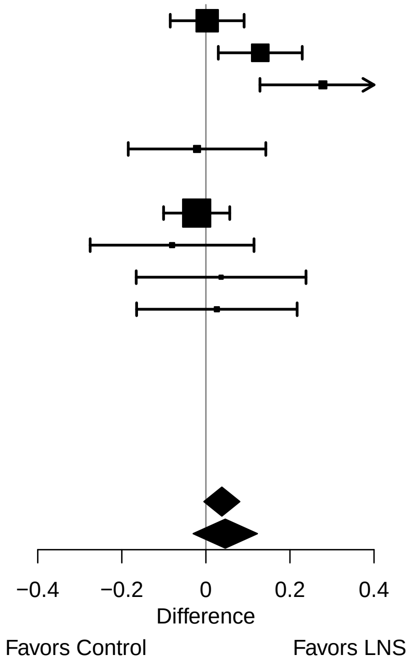

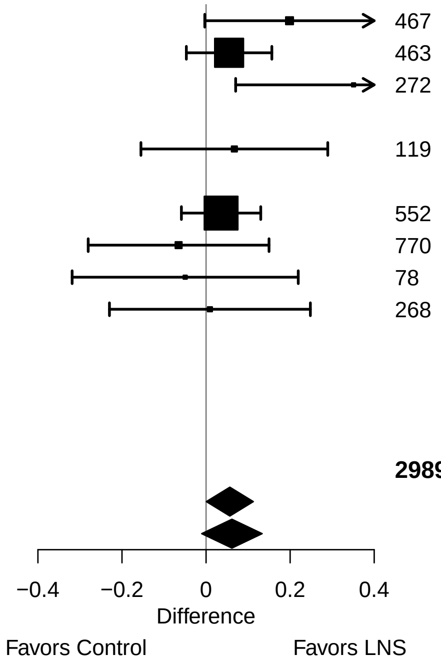

Supplemental figure 8D: Mean difference in social-emotional z-score

8D6: Stratified by Season at the time of assessment

P−for−interaction = 0.734
Difference in MDs = −0.01 (−0.08, 0.06)

Country	Trial	Tool	LNS N	Control N	Control Mean	Dry MD (95% CI)	Fixed W	Random W	LNS N	Control N	Control Mean	Rainy MD (95% CI)	Fixed W	Random W
Bangladesh	JiVitA−4 (21)													
Bangladesh	RDNS (22)	DMC	737	359	−0.03	0.06 (−0.08, 0.21)	0.10	0.12	920	456	−0.04	0.06 (−0.05, 0.17)	0.19	0.14
Bangladesh	WASH−B (23)	EASQ	604	1891	−0.02	0.09 (0.00, 0.17)	0.30	0.22	462	1375	−0.04	0.17 (0.06, 0.29)	0.16	0.14
Burkina Faso	iLiNS−Zinc (24)	DMC	514	274	−0.25	0.36 (0.14, 0.57)	0.05	0.06	232	101	−0.21	0.35 (0.04, 0.67)	0.02	0.08
Ghana	GHANA (25)													
Ghana	iLiNS−DYAD−G (26)	PSED	195	387	0.03	0.01 (−0.17, 0.19)	0.07	0.09	137	270	−0.05	0.01 (−0.19, 0.20)	0.06	0.12
Haiti	HAITI (27)													
Kenya	WASH−B (28)	EASQ	315	1059	−0.24	0.09 (−0.02, 0.20)	0.18	0.17	1046	3682	0.07	0.00 (−0.08, 0.07)	0.42	0.15
Madagascar	MAHAY (29)	ASQI												
Malawi	iLiNS−DYAD−M (30)	PSED	153	310	−0.10	0.07 (−0.13, 0.27)	0.05	0.07	62	127	0.25	−0.14 (−0.41, 0.14)	0.03	0.09
Malawi	iLiNS−DOSE (31)	PSED	394	118	−0.16	0.17 (−0.03, 0.37)	0.05	0.07	243	100	0.14	−0.14 (−0.37, 0.09)	0.04	0.10
Mali	PROMIS CS (32)	DMC												
Zimbabwe	SHINE (HIV−) (33)	MDAT	603	568	−0.04	0.09 (−0.02, 0.20)	0.17	0.16	238	231	−0.11	0.21 (0.02, 0.41)	0.06	0.11
Zimbabwe	SHINE (HIV+) (34)	MDAT	121	110	−0.13	0.30 (0.05, 0.56)	0.03	0.05	49	41	−0.36	0.51 (0.13, 0.89)	0.02	0.06
			**3636**	**5076**		**I² = 0.18, Tau² = 0.00**			**3389**	**6383**		**I² = 0.64, Tau² = 0.02**		
**Fixed**						**0.10 (0.06, 0.15)**						**0.06 (0.01, 0.10)**		
**Random**						**0.11 (0.05, 0.17)**						**0.09 (−0.03, 0.21)**		

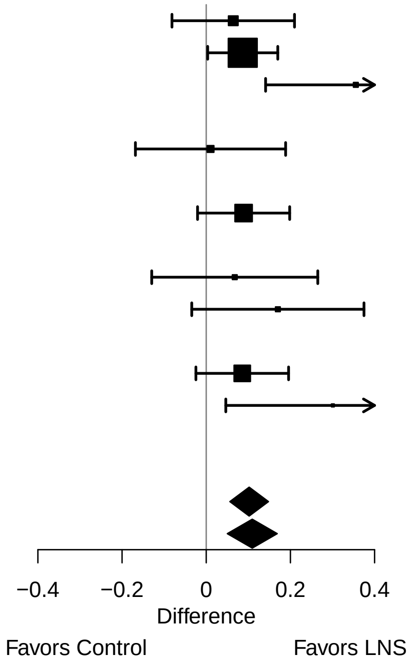

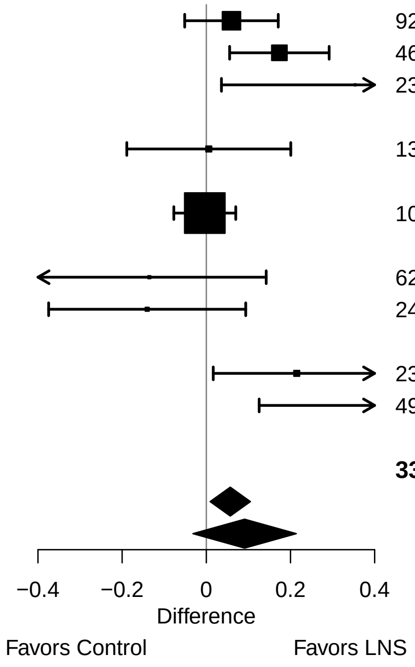

Supplemental figure 8E: Social-emotional lowest decile prevalence ratio

8E1: Stratified by Household socio-economic status

P−for−interaction = 0.655
Ratio of PRs = 0.96 (0.79, 1.16)

Country	Trial	Tool	LNS N	Control N	Control Prevalence	At least median PR (95% CI)	Fixed W	Random W	LNS N	Control N	Control Prevalence	Less than median PR (95% CI)	Fixed W	Random W
Bangladesh	JiVitA−4 (21)													
Bangladesh	RDNS (22)	DMC	830	407	10.1	0.77 (0.52, 1.13)	0.14	0.11	827	408	12.7	0.90 (0.67, 1.22)	0.17	0.17
Bangladesh	WASH−B (23)	EASQ	545	1659	7.4	1.02 (0.67, 1.57)	0.12	0.10	521	1607	13.8	0.70 (0.53, 0.93)	0.19	0.19
Burkina Faso	iLiNS−Zinc (24)	DMC	459	163	14.7	0.31 (0.17, 0.56)	0.06	0.09	287	212	17.9	0.58 (0.35, 0.98)	0.06	0.06
Ghana	GHANA (25)													
Ghana	iLiNS−DYAD−G (26)	PSED	153	341	8.5	1.61 (0.95, 2.74)	0.08	0.09	179	314	11.8	0.57 (0.30, 1.06)	0.04	0.04
Haiti	HAITI (27)													
Kenya	WASH−B (28)	EASQ	778	2706	9.2	0.80 (0.59, 1.09)	0.22	0.11	582	2036	11.8	0.96 (0.74, 1.23)	0.24	0.24
Madagascar	MAHAY (29)	ASQI	741	845	5.7	1.38 (0.78, 2.44)	0.07	0.09	823	724	13.1	0.98 (0.62, 1.54)	0.07	0.07
Malawi	iLiNS−DYAD−M (30)	PSED	118	230	10.4	0.97 (0.51, 1.88)	0.05	0.08	97	207	10.1	1.02 (0.50, 2.07)	0.03	0.03
Malawi	iLiNS−DOSE (31)	PSED	278	94	6.4	1.75 (0.75, 4.06)	0.03	0.06	270	96	11.5	0.81 (0.41, 1.58)	0.03	0.03
Mali	PROMIS CS (32)	DMC	494	450	9.6	0.85 (0.52, 1.39)	0.09	0.10	433	494	13.0	0.73 (0.48, 1.12)	0.08	0.08
Zimbabwe	SHINE (HIV−) (33)	MDAT	406	359	13.1	0.58 (0.38, 0.88)	0.12	0.10	389	391	11.8	0.76 (0.48, 1.23)	0.07	0.07
Zimbabwe	SHINE (HIV+) (34)	MDAT	91	78	16.7	0.40 (0.17, 0.93)	0.03	0.06	74	67	10.4	0.65 (0.21, 2.01)	0.01	0.01
			**4893**	**7332**		**I^2 = 0.67, Tau^2 = 0.18**			**4482**	**6556**		**I^2 = 0.00, Tau^2 = 0.00**		
**Fixed**						**0.83 (0.71, 0.95)**						**0.81 (0.72, 0.92)**		
**Random**						**0.84 (0.62, 1.13)**						**0.81 (0.72, 0.92)**		

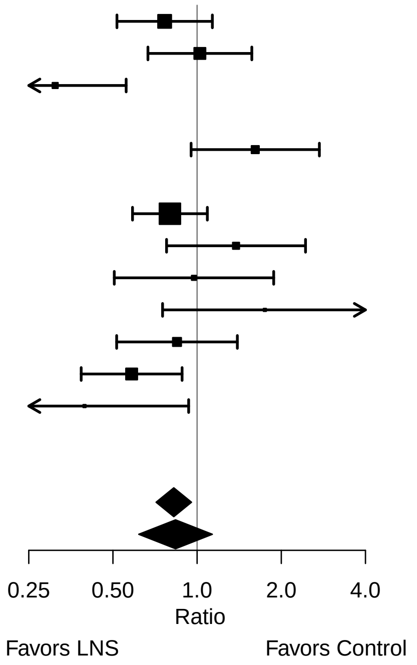

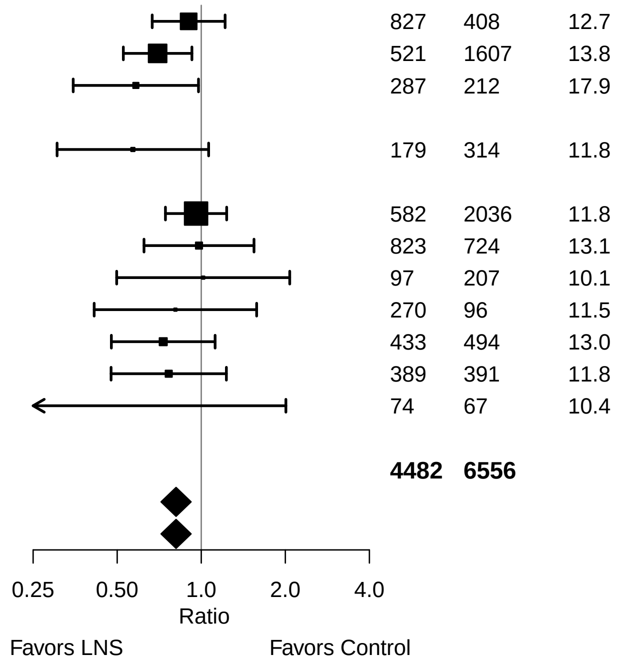

Supplemental figure 8E: Social-emotional lowest decile prevalence ratio

8E2: Stratified by Household food insecurity

P−for−interaction = 0.325
Ratio of PRs = 1.11 (0.90, 1.37)

Country	Trial	Tool	LNS N	Control N	Control Prevalence	Mild to secure PR (95% CI)	Fixed W	Random W	LNS N	Control N	Control Prevalence	Moderate to severe PR (95% CI)	Fixed W	Random W
Bangladesh	JiVitA−4 (21)													
Bangladesh	RDNS (22)	DMC	1054	493	10.1	0.77 (0.57, 1.04)	0.18	0.16	603	322	13.4	0.96 (0.69, 1.32)	0.25	0.17
Bangladesh	WASH−B (23)	EASQ	853	2539	9.6	0.76 (0.56, 1.02)	0.19	0.16	213	727	13.8	0.99 (0.66, 1.48)	0.16	0.15
Burkina Faso	iLiNS−Zinc (24)	DMC	389	162	14.8	0.43 (0.22, 0.85)	0.04	0.08	357	213	17.8	0.41 (0.28, 0.59)	0.20	0.16
Ghana	GHANA (25)													
Ghana	iLiNS−DYAD−G (26)	PSED	239	452	8.6	1.07 (0.65, 1.76)	0.07	0.11	92	202	12.9	0.93 (0.48, 1.80)	0.06	0.09
Haiti	HAITI (27)													
Kenya	WASH−B (28)	EASQ	1214	4244	10.2	0.83 (0.65, 1.05)	0.30	0.18	146	487	11.1	1.24 (0.73, 2.08)	0.10	0.12
Madagascar	MAHAY (29)	ASQI	940	892	8.4	1.13 (0.65, 1.95)	0.06	0.10	336	389	10.8	1.16 (0.70, 1.92)	0.10	0.12
Malawi	iLiNS−DYAD−M (30)	PSED	68	118	8.5	1.91 (0.86, 4.26)	0.03	0.06	146	314	11.1	0.68 (0.35, 1.29)	0.06	0.10
Malawi	iLiNS−DOSE (31)	PSED												
Mali	PROMIS CS (32)	DMC												
Zimbabwe	SHINE (HIV−) (33)	MDAT	631	586	12.1	0.68 (0.48, 0.97)	0.14	0.15	140	154	13.6	0.68 (0.36, 1.29)	0.06	0.10
Zimbabwe	SHINE (HIV+) (34)	MDAT												
			**5388**	**9486**		**I² = 0.39, Tau² = 0.06**			**2033**	**2808**		**I² = 0.66, Tau² = 0.08**		
**Fixed**						**0.81 (0.71, 0.92)**						**0.81 (0.69, 0.95)**		
**Random**						**0.83 (0.66, 1.05)**						**0.83 (0.64, 1.08)**		

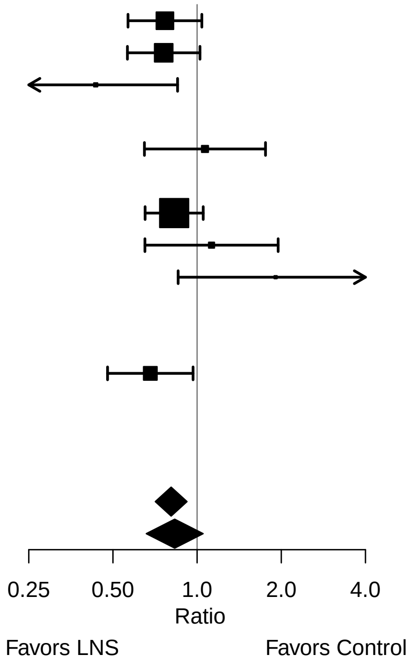

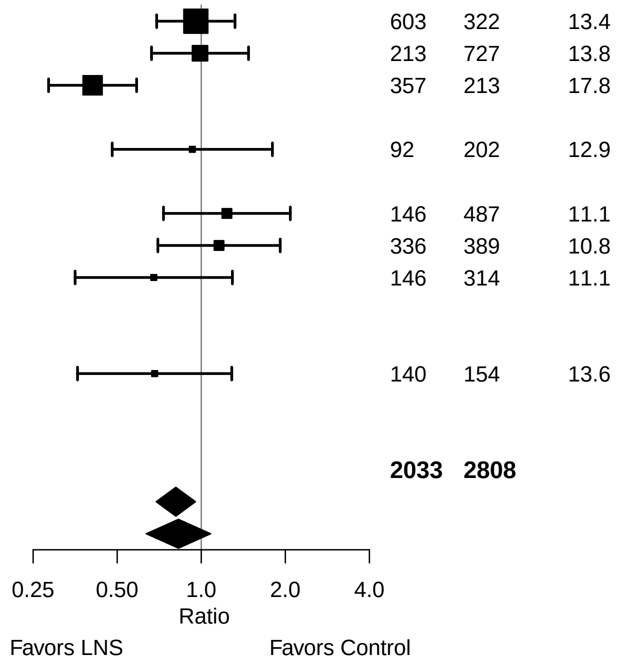

Supplemental figure 8E: Social-emotional lowest decile prevalence ratio

8E3: Stratified by Household source water quality

P−for−interaction = 0.534
Ratio of PRs = 1.10 (0.81, 1.49)

Country	Trial	Tool	Improved LNS N	Improved Control N	Improved Control Prevalence	Improved PR (95% CI)	Improved Fixed W	Improved Random W	Unimproved LNS N	Unimproved Control N	Unimproved Control Prevalence	Unimproved PR (95% CI)	Unimproved Fixed W	Unimproved Random W
Bangladesh	JiVitA−4 (21)													
Bangladesh	RDNS (22)	DMC												
Bangladesh	WASH−B (23)	EASQ	461	981	12.9	0.70 (0.48, 1.03)	0.29	0.27	66	117	18.8	0.64 (0.31, 1.34)	0.09	0.12
Burkina Faso	iLiNS−Zinc (24)	DMC	220	82	13.4	0.34 (0.15, 0.75)	0.07	0.08	526	293	17.4	0.45 (0.29, 0.69)	0.25	0.20
Ghana	GHANA (25)													
Ghana	iLiNS−DYAD−G (26)	PSED												
Haiti	HAITI (27)													
Kenya	WASH−B (28)	EASQ	413	1419	10.2	0.73 (0.47, 1.14)	0.22	0.21	236	649	8.2	1.14 (0.70, 1.87)	0.19	0.18
Madagascar	MAHAY (29)	ASQI	405	430	11.9	1.04 (0.56, 1.93)	0.11	0.12	1134	1133	8.1	1.24 (0.75, 2.06)	0.18	0.18
Malawi	iLiNS−DYAD−M (30)	PSED												
Malawi	iLiNS−DOSE (31)	PSED												
Mali	PROMIS CS (32)	DMC	528	552	9.8	0.85 (0.55, 1.31)	0.22	0.22	357	378	14.0	0.70 (0.44, 1.11)	0.22	0.19
Zimbabwe	SHINE (HIV−) (33)	MDAT	228	220	9.1	0.87 (0.45, 1.68)	0.09	0.11	131	122	11.5	0.67 (0.31, 1.43)	0.08	0.12
Zimbabwe	SHINE (HIV+) (34)	MDAT												
			**2255**	**3684**		**I² = 0.10, Tau² = 0.01**			**2450**	**2692**		**I² = 0.59, Tau² = 0.09**		
**Fixed**						**0.75 (0.61, 0.92)**						**0.76 (0.61, 0.94)**		
**Random**						**0.75 (0.60, 0.94)**						**0.76 (0.55, 1.06)**		

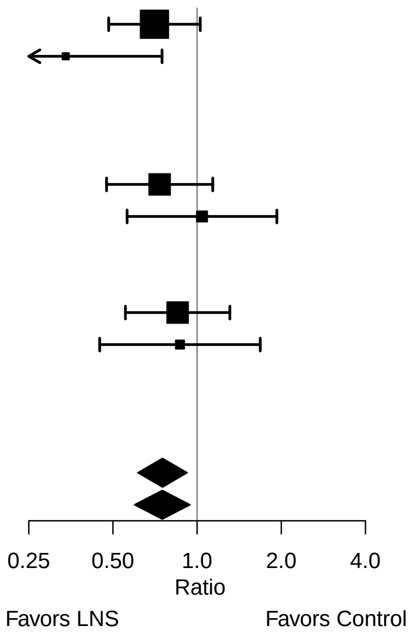

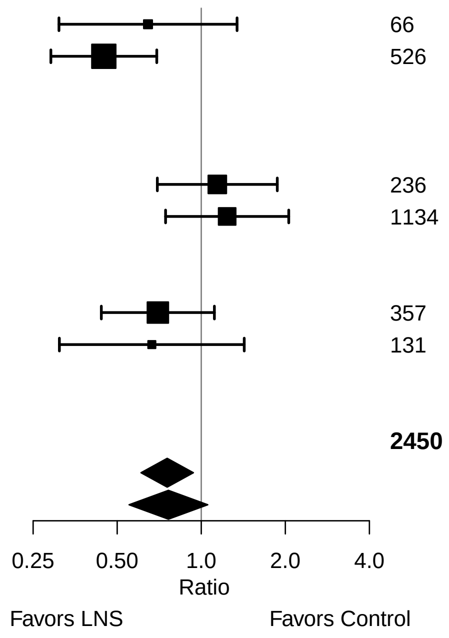

Supplemental figure 8E: Social-emotional lowest decile prevalence ratio

8E4: Stratified by Household sanitation

P−for−interaction = 0.304
Ratio of PRs = 0.84 (0.60, 1.17)

Country	Trial	Tool	LNS N	Control N	Control Prevalence	Improved PR (95% CI)	Fixed W	Random W	LNS N	Control N	Control Prevalence	Unimproved PR (95% CI)	Fixed W	Random W
Bangladesh	JiVitA−4 (21)													
Bangladesh	RDNS (22)	DMC	1174	573	10.1	0.85 (0.64, 1.13)	0.56	0.56	481	241	14.5	0.82 (0.55, 1.21)	0.35	0.35
Bangladesh	WASH−B (23)	EASQ												
Burkina Faso	iLiNS−Zinc (24)	DMC												
Ghana	GHANA (25)													
Ghana	iLiNS−DYAD−G (26)	PSED												
Haiti	HAITI (27)													
Kenya	WASH−B (28)	EASQ	94	337	6.8	0.78 (0.31, 1.97)	0.05	0.05	555	1732	10.1	0.86 (0.58, 1.26)	0.36	0.36
Madagascar	MAHAY (29)	ASQI												
Malawi	iLiNS−DYAD−M (30)	PSED												
Malawi	iLiNS−DOSE (31)	PSED												
Mali	PROMIS CS (32)	DMC	687	678	10.5	0.83 (0.57, 1.21)	0.32	0.32	211	242	12.4	0.69 (0.37, 1.27)	0.14	0.14
Zimbabwe	SHINE (HIV−) (33)	MDAT	133	108	6.5	1.62 (0.73, 3.61)	0.07	0.07	225	235	11.5	0.54 (0.29, 1.01)	0.14	0.14
Zimbabwe	SHINE (HIV+) (34)	MDAT												
			**2088**	**1696**		**I² = 0.00, Tau² = 0.00**			**1472**	**2450**		**I² = 0.00, Tau² = 0.00**		
**Fixed**						**0.88 (0.71, 1.09)**						**0.76 (0.61, 0.97)**		
**Random**						**0.88 (0.71, 1.09)**						**0.76 (0.61, 0.97)**		

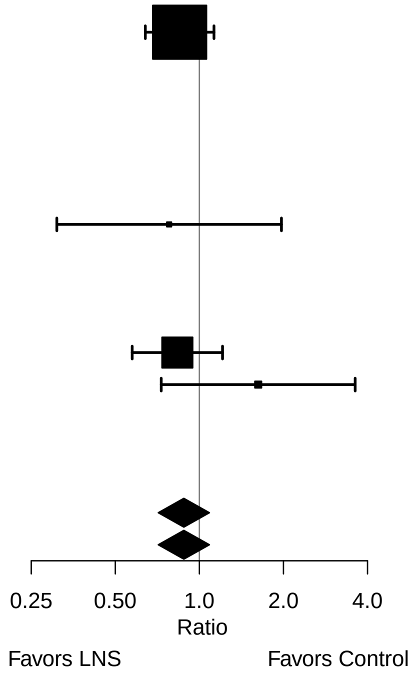

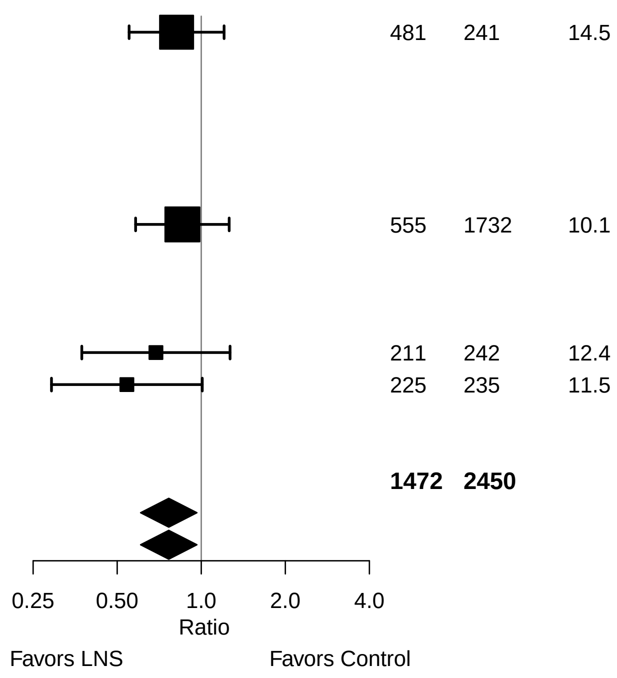

Supplemental figure 8E: Social-emotional lowest decile prevalence ratio

8E5: Stratified by Home environment

P−for−interaction = 0.863
Ratio of PRs = 1.02 (0.84, 1.23)

			At least median							Less than median					
**Country**	**Trial**	**Tool**	**LNS N**	**Control N**	**Control Prevalence**	**PR (95% CI)**	**Fixed W**	**Random W**	**LNS N**	**Control N**	**Control Prevalence**	**PR (95% CI)**	**Fixed W**	**Random W**
Bangladesh	JiVitA−4 (21)													
Bangladesh	RDNS (22)	DMC	1187	582	8.4	0.87 (0.65, 1.16)	0.26	0.23	467	230	18.3	0.84 (0.57, 1.24)	0.13	0.16
Bangladesh	WASH−B (23)	EASQ	603	1599	6.9	0.88 (0.63, 1.25)	0.17	0.17	463	1666	13.9	0.84 (0.64, 1.09)	0.29	0.21
Burkina Faso	iLiNS−Zinc (24)	DMC	473	199	8.0	0.45 (0.25, 0.81)	0.06	0.07	272	175	26.3	0.48 (0.31, 0.73)	0.11	0.14
Ghana	GHANA (25)													
Ghana	iLiNS−DYAD−G (26)	PSED	213	410	10.5	1.07 (0.67, 1.72)	0.09	0.10	119	247	9.3	0.81 (0.39, 1.70)	0.04	0.07
Haiti	HAITI (27)													
Kenya	WASH−B (28)	EASQ	809	2682	7.3	0.88 (0.64, 1.21)	0.20	0.19	552	2060	14.2	0.90 (0.70, 1.16)	0.31	0.21
Madagascar	MAHAY (29)	ASQI	843	839	7.6	1.01 (0.61, 1.67)	0.08	0.09	770	764	11.1	1.26 (0.77, 2.05)	0.08	0.12
Malawi	iLiNS−DYAD−M (30)	PSED	137	290	12.4	0.82 (0.46, 1.47)	0.06	0.07	78	148	6.1	1.69 (0.68, 4.20)	0.02	0.05
Malawi	iLiNS−DOSE (31)	PSED	376	135	11.1	1.24 (0.73, 2.14)	0.07	0.08	268	86	7.0	0.75 (0.30, 1.89)	0.02	0.05
Mali	PROMIS CS (32)	DMC												
Zimbabwe	SHINE (HIV−) (33)	MDAT												
Zimbabwe	SHINE (HIV+) (34)	MDAT												
			**4641**	**6736**		**I² = 0.08, Tau² = 0.01**			**2989**	**5376**		**I² = 0.41, Tau² = 0.04**		
**Fixed**						**0.89 (0.77, 1.02)**						**0.85 (0.74, 0.98)**		
**Random**						**0.89 (0.76, 1.04)**						**0.85 (0.68, 1.06)**		

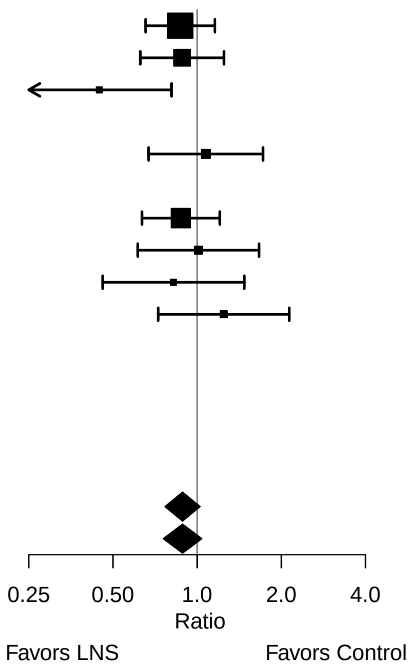

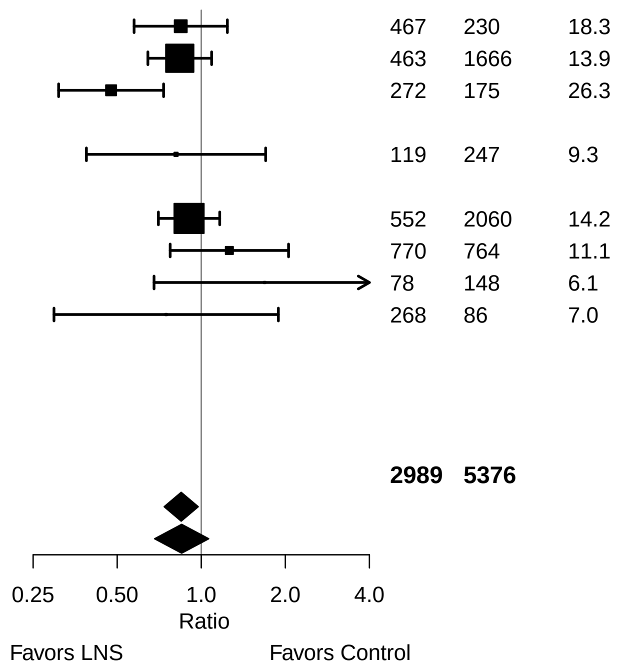

Supplemental figure 8E: Social-emotional lowest decile prevalence ratio

8E6: Stratified by Season at the time of assessment

P−for−interaction = 0.584
Ratio of PRs = 1.06 (0.85, 1.33)

Country	Trial	Tool	LNS N	Control N	Control Prevalence	Dry PR (95% CI)	Fixed W	Random W	LNS N	Control N	Control Prevalence	Rainy PR (95% CI)	Fixed W	Random W
Bangladesh	JiVitA−4 (21)													
Bangladesh	RDNS (22)	DMC	737	359	12.8	0.77 (0.55, 1.08)	0.21	0.21	920	456	10.3	0.91 (0.67, 1.23)	0.25	0.22
Bangladesh	WASH−B (23)	EASQ	604	1891	11.3	0.83 (0.61, 1.13)	0.25	0.24	462	1375	9.4	0.78 (0.54, 1.14)	0.17	0.19
Burkina Faso	iLiNS−Zinc (24)	DMC	514	274	15.7	0.43 (0.26, 0.72)	0.09	0.10	232	101	18.8	0.37 (0.16, 0.83)	0.04	0.08
Ghana	GHANA (25)													
Ghana	iLiNS−DYAD−G (26)	PSED	195	387	10.6	0.92 (0.55, 1.54)	0.09	0.10	137	270	9.3	1.10 (0.59, 2.05)	0.06	0.11
Haiti	HAITI (27)													
Kenya	WASH−B (28)	EASQ	315	1059	15.2	0.71 (0.49, 1.04)	0.17	0.17	1046	3682	8.9	0.94 (0.74, 1.21)	0.39	0.25
Madagascar	MAHAY (29)	ASQI												
Malawi	iLiNS−DYAD−M (30)	PSED												
Malawi	iLiNS−DOSE (31)	PSED												
Mali	PROMIS CS (32)	DMC												
Zimbabwe	SHINE (HIV−) (33)	MDAT	603	568	11.1	0.79 (0.56, 1.13)	0.19	0.19	238	231	13.9	0.55 (0.33, 0.92)	0.09	0.14
Zimbabwe	SHINE (HIV+) (34)	MDAT												
			**2968**	**4538**		**I^2 = 0.11, Tau^2 = 0.01**			**3035**	**6115**		**I^2 = 0.42, Tau^2 = 0.06**		
**Fixed**						**0.75 (0.64, 0.88)**						**0.84 (0.72, 0.98)**		
**Random**						**0.75 (0.63, 0.89)**						**0.79 (0.61, 1.03)**		

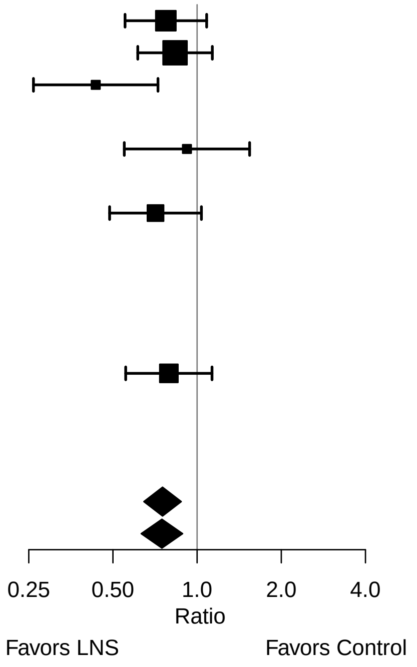

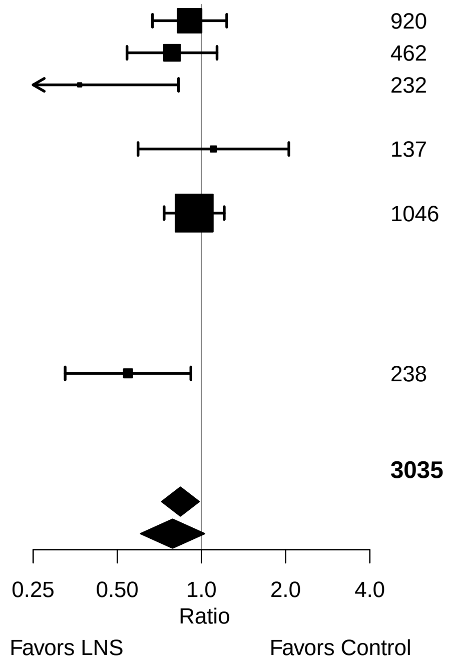

Supplemental figure 8F: Social-emotional lowest decile prevalence difference

8F1: Stratified by Household socio-economic status

P−for−interaction = 0.160
Difference in PDs = −0.01 (−0.03, 0.00)

Country	Trial	Tool	LNS N	Control N	Control Prevalence	At least median PD (95% CI)	Fixed W	Random W	LNS N	Control N	Control Prevalence	Less than median PD (95% CI)	Fixed W	Random W
Bangladesh	JiVitA−4 (21)													
Bangladesh	RDNS (22)	DMC	830	407	10.1	−0.02 (−0.06, 0.01)	0.11	0.11	827	408	12.7	−0.01 (−0.05, 0.02)	0.14	0.14
Bangladesh	WASH−B (23)	EASQ	545	1659	7.4	0.00 (−0.03, 0.03)	0.15	0.11	521	1607	13.8	−0.04 (−0.07, −0.01)	0.21	0.21
Burkina Faso	iLiNS−Zinc (24)	DMC	459	163	14.7	−0.10 (−0.16, −0.04)	0.04	0.08	287	212	17.9	−0.07 (−0.15, 0.00)	0.04	0.04
Ghana	GHANA (25)													
Ghana	iLiNS−DYAD−G (26)	PSED	153	341	8.5	0.05 (−0.01, 0.11)	0.05	0.08	179	314	11.8	−0.05 (−0.11, 0.00)	0.06	0.06
Haiti	HAITI (27)													
Kenya	WASH−B (28)	EASQ	778	2706	9.2	−0.02 (−0.04, 0.01)	0.28	0.12	582	2036	11.8	0.00 (−0.03, 0.02)	0.23	0.23
Madagascar	MAHAY (29)	ASQI	741	845	5.7	0.02 (−0.02, 0.06)	0.10	0.10	823	724	13.1	0.00 (−0.06, 0.06)	0.05	0.05
Malawi	iLiNS−DYAD−M (30)	PSED	118	230	10.4	0.00 (−0.07, 0.07)	0.03	0.07	97	207	10.1	0.00 (−0.07, 0.07)	0.04	0.04
Malawi	iLiNS−DOSE (31)	PSED	278	94	6.4	0.05 (−0.02, 0.12)	0.03	0.07	270	96	11.5	−0.02 (−0.09, 0.05)	0.04	0.04
Mali	PROMIS CS (32)	DMC	494	450	9.6	−0.01 (−0.06, 0.03)	0.08	0.10	433	494	13.0	−0.03 (−0.08, 0.01)	0.10	0.10
Zimbabwe	SHINE (HIV−) (33)	MDAT	406	359	13.1	−0.05 (−0.10, −0.01)	0.09	0.10	389	391	11.8	−0.03 (−0.08, 0.02)	0.08	0.08
Zimbabwe	SHINE (HIV+) (34)	MDAT	91	78	16.7	−0.10 (−0.19, −0.01)	0.02	0.06	74	67	10.4	−0.04 (−0.13, 0.06)	0.02	0.02
			**4893**	**7332**		**I^2 = 0.64, Tau^2 = 0.00**			**4482**	**6556**		**I^2 = 0.00, Tau^2 = 0.00**		
**Fixed**						**−0.01 (−0.03, 0.00)**						**−0.02 (−0.04, −0.01)**		
**Random**						**−0.02 (−0.04, 0.01)**						**−0.02 (−0.04, −0.01)**		

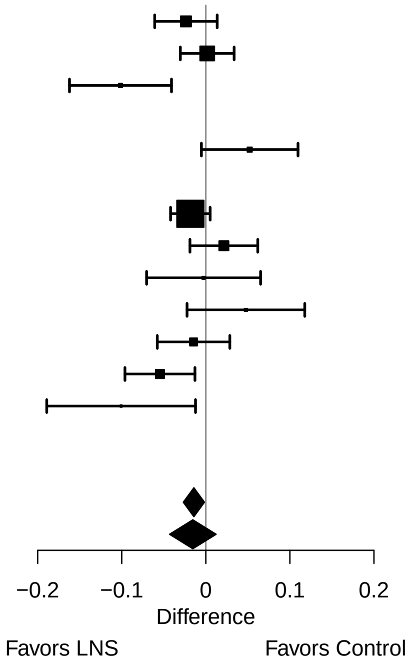

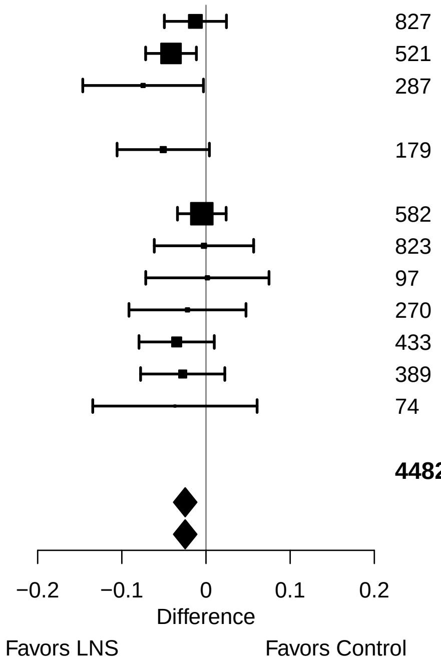

Supplemental figure 8F: Social-emotional lowest decile prevalence difference

8F2: Stratified by Household food insecurity

P−for−interaction = 0.599
Difference in PDs = 0.01 (−0.02, 0.03)

Country	Trial	Tool	LNS N	Control N	Control Prevalence	Mild to secure PD (95% CI)	Fixed W	Random W	LNS N	Control N	Control Prevalence	Moderate to severe PD (95% CI)	Fixed W	Random W
Bangladesh	JiVitA−4 (21)													
Bangladesh	RDNS (22)	DMC	1054	493	10.1	−0.02 (−0.05, 0.00)	0.17	0.16	603	322	13.4	−0.01 (−0.05, 0.04)	0.22	0.16
Bangladesh	WASH−B (23)	EASQ	853	2539	9.6	−0.02 (−0.05, 0.00)	0.25	0.18	213	727	13.8	0.00 (−0.06, 0.05)	0.13	0.13
Burkina Faso	iLiNS−Zinc (24)	DMC	389	162	14.8	−0.08 (−0.16, −0.01)	0.02	0.06	357	213	17.8	−0.11 (−0.15, −0.06)	0.20	0.16
Ghana	GHANA (25)													
Ghana	iLiNS−DYAD−G (26)	PSED	239	452	8.6	0.01 (−0.04, 0.05)	0.07	0.12	92	202	12.9	−0.01 (−0.09, 0.07)	0.06	0.09
Haiti	HAITI (27)													
Kenya	WASH−B (28)	EASQ	1214	4244	10.2	−0.02 (−0.04, 0.00)	0.31	0.19	146	487	11.1	0.03 (−0.04, 0.09)	0.09	0.11
Madagascar	MAHAY (29)	ASQI	940	892	8.4	0.01 (−0.04, 0.06)	0.06	0.11	336	389	10.8	0.02 (−0.04, 0.08)	0.11	0.12
Malawi	iLiNS−DYAD−M (30)	PSED	68	118	8.5	0.08 (−0.02, 0.17)	0.02	0.04	146	314	11.1	−0.04 (−0.10, 0.02)	0.11	0.13
Malawi	iLiNS−DOSE (31)	PSED												
Mali	PROMIS CS (32)	DMC												
Zimbabwe	SHINE (HIV−) (33)	MDAT	631	586	12.1	−0.04 (−0.08, 0.00)	0.10	0.14	140	154	13.6	−0.04 (−0.11, 0.03)	0.08	0.10
Zimbabwe	SHINE (HIV+) (34)	MDAT												
			**5388**	**9486**		**I^2 = 0.35, Tau^2 = 0.00**			**2033**	**2808**		**I^2 = 0.62, Tau^2 = 0.00**		
**Fixed**						**−0.02 (−0.03, −0.01)**						**−0.03 (−0.05, −0.01)**		
**Random**						**−0.02 (−0.04, 0.01)**						**−0.02 (−0.05, 0.01)**		

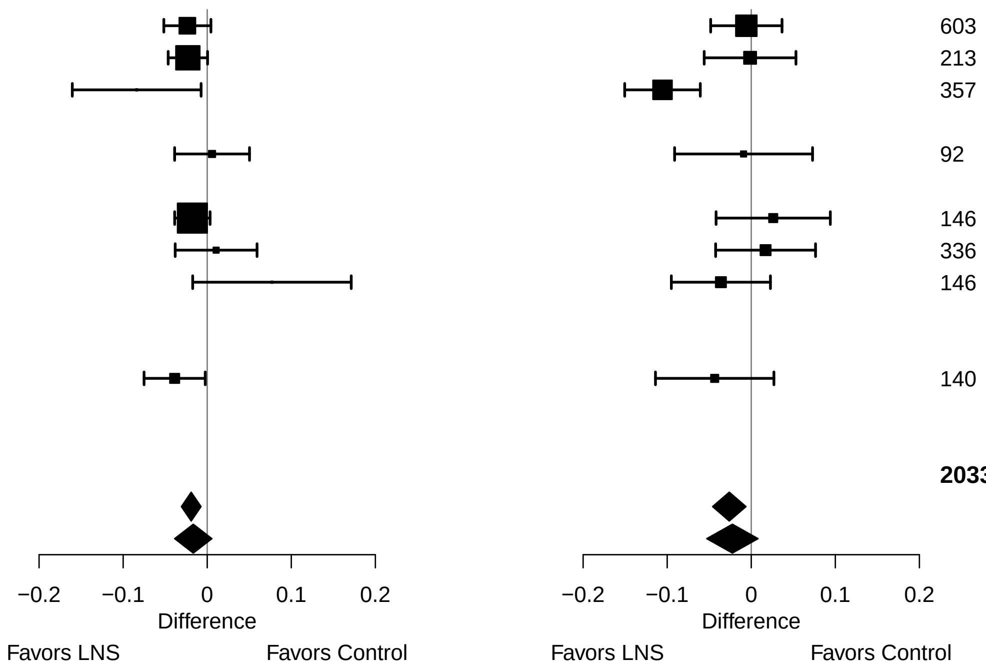

Supplemental figure 8F: Social-emotional lowest decile prevalence difference

8F3: Stratified by Household source water quality

P−for−interaction = 0.782
Difference in PDs = 0.00 (−0.02, 0.03)

Country	Trial	Tool	Improved: LNS N	Improved: Control N	Improved: Control Prevalence	Improved: PD (95% CI)	Improved: Fixed W	Improved: Random W	Unimproved: LNS N	Unimproved: Control N	Unimproved: Control Prevalence	Unimproved: PD (95% CI)	Unimproved: Fixed W	Unimproved: Random W
Bangladesh	JiVitA−4 (21)													
Bangladesh	RDNS (22)	DMC												
Bangladesh	WASH−B (23)	EASQ	461	981	12.9	−0.04 (−0.08, 0.00)	0.24	0.24	66	117	18.8	−0.07 (−0.17, 0.04)	0.05	0.09
Burkina Faso	iLiNS−Zinc (24)	DMC	220	82	13.4	−0.09 (−0.17, −0.01)	0.06	0.06	526	293	17.4	−0.10 (−0.15, −0.05)	0.20	0.19
Ghana	GHANA (25)													
Ghana	iLiNS−DYAD−G (26)	PSED												
Haiti	HAITI (27)													
Kenya	WASH−B (28)	EASQ	413	1419	10.2	−0.03 (−0.06, 0.01)	0.29	0.29	236	649	8.2	0.01 (−0.03, 0.06)	0.25	0.20
Madagascar	MAHAY (29)	ASQI	405	430	11.9	0.00 (−0.07, 0.08)	0.06	0.06	1134	1133	8.1	0.02 (−0.03, 0.07)	0.23	0.20
Malawi	iLiNS−DYAD−M (30)	PSED												
Malawi	iLiNS−DOSE (31)	PSED												
Mali	PROMIS CS (32)	DMC	528	552	9.8	−0.01 (−0.05, 0.02)	0.24	0.24	357	378	14.0	−0.04 (−0.10, 0.01)	0.17	0.18
Zimbabwe	SHINE (HIV−) (33)	MDAT	228	220	9.1	−0.01 (−0.07, 0.04)	0.11	0.11	131	122	11.5	−0.04 (−0.11, 0.03)	0.10	0.14
Zimbabwe	SHINE (HIV+) (34)	MDAT												
			**2255**	**3684**		**I² = 0.00, Tau² = 0.00**			**2450**	**2692**		**I² = 0.67, Tau² = 0.00**		
**Fixed**						**−0.03 (−0.05, −0.01)**						**−0.03 (−0.05, 0.00)**		
**Random**						**−0.03 (−0.05, −0.01)**						**−0.03 (−0.07, 0.01)**		

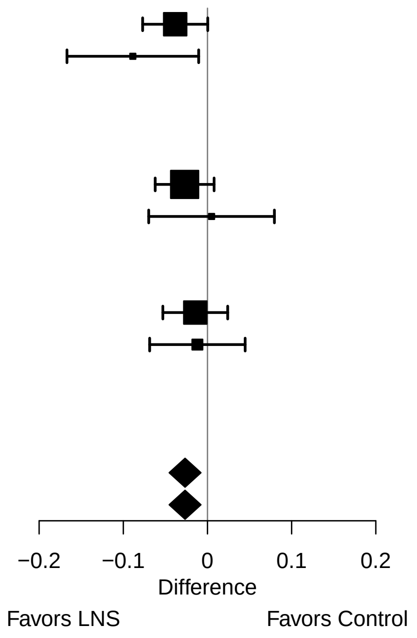

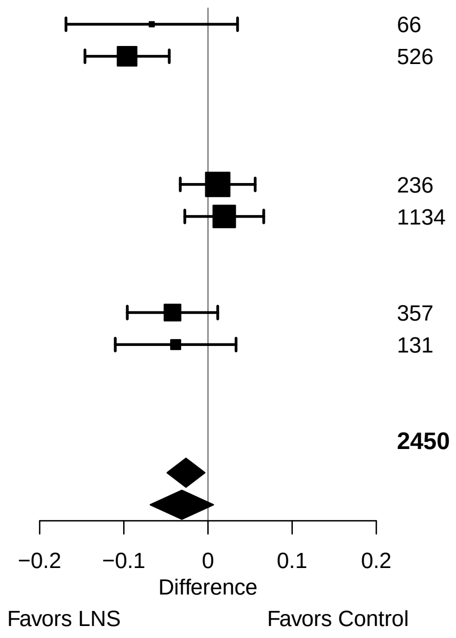

Supplemental figure 8F: Social-emotional lowest decile prevalence difference

8F4: Stratified by Household sanitation

P−for−interaction = 0.189
Difference in PDs = −0.02 (−0.05, 0.01)

Country	Trial	Tool	Improved LNS N	Improved Control N	Improved Control Prevalence	Improved PD (95% CI)	Improved Fixed W	Improved Random W	Unimproved LNS N	Unimproved Control N	Unimproved Control Prevalence	Unimproved PD (95% CI)	Unimproved Fixed W	Unimproved Random W
Bangladesh	JiVitA−4 (21)													
Bangladesh	RDNS (22)	DMC	1174	573	10.1	−0.02 (−0.04, 0.01)	0.48	0.48	481	241	14.5	−0.03 (−0.08, 0.03)	0.20	0.20
Bangladesh	WASH−B (23)	EASQ												
Burkina Faso	iLiNS−Zinc (24)	DMC												
Ghana	GHANA (25)													
Ghana	iLiNS−DYAD−G (26)	PSED												
Haiti	HAITI (27)													
Kenya	WASH−B (28)	EASQ	94	337	6.8	−0.02 (−0.07, 0.04)	0.13	0.13	555	1732	10.1	−0.01 (−0.05, 0.02)	0.46	0.46
Madagascar	MAHAY (29)	ASQI												
Malawi	iLiNS−DYAD−M (30)	PSED												
Malawi	iLiNS−DOSE (31)	PSED												
Mali	PROMIS CS (32)	DMC	687	678	10.5	−0.02 (−0.05, 0.02)	0.30	0.30	211	242	12.4	−0.04 (−0.10, 0.02)	0.14	0.14
Zimbabwe	SHINE (HIV−) (33)	MDAT	133	108	6.5	0.04 (−0.02, 0.10)	0.09	0.09	225	235	11.5	−0.05 (−0.11, 0.00)	0.19	0.19
Zimbabwe	SHINE (HIV+) (34)	MDAT												
			**2088**	**1696**		**I² = 0.00, Tau² = 0.00**			**1472**	**2450**		**I² = 0.00, Tau² = 0.00**		
**Fixed**						**−0.01 (−0.03, 0.01)**						**−0.03 (−0.05, 0.00)**		
**Random**						**−0.01 (−0.03, 0.01)**						**−0.03 (−0.05, 0.00)**		

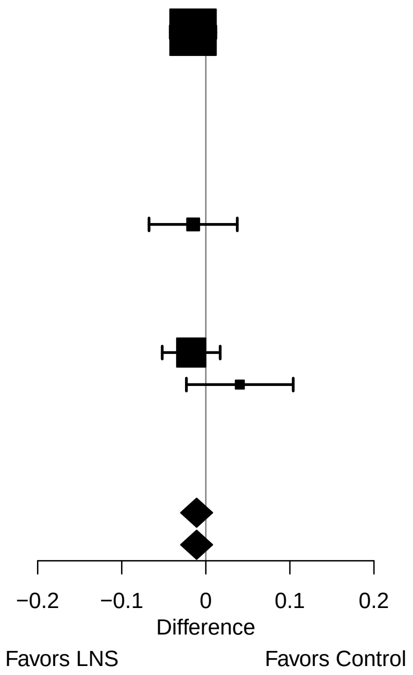

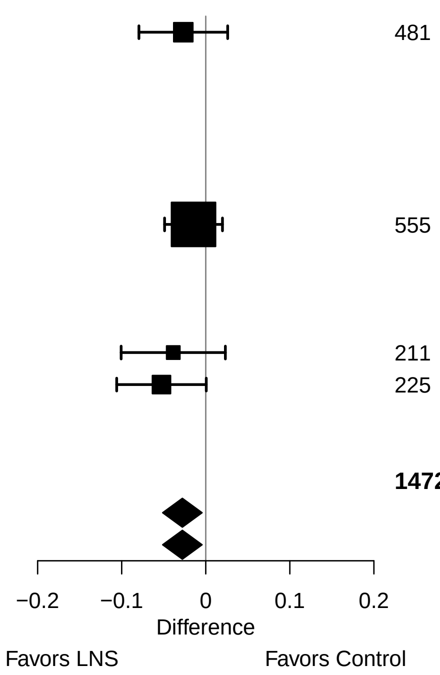

Supplemental figure 8F: Social-emotional lowest decile prevalence difference

8F5: Stratified by Home environment

P−for−interaction = 0.545
Difference in PDs = −0.01 (−0.03, 0.01)

Country	Trial	Tool	LNS N	Control N	Control Prevalence	At least median PD (95% CI)	Fixed W	Random W	LNS N	Control N	Control Prevalence	Less than median PD (95% CI)	Fixed W	Random W
Bangladesh	JiVitA−4 (21)													
Bangladesh	RDNS (22)	DMC	1187	582	8.4	−0.01 (−0.03, 0.01)	0.23	0.23	467	230	18.3	−0.03 (−0.10, 0.04)	0.07	0.11
Bangladesh	WASH−B (23)	EASQ	603	1599	6.9	−0.01 (−0.03, 0.01)	0.24	0.24	463	1666	13.9	−0.02 (−0.05, 0.01)	0.29	0.17
Burkina Faso	iLiNS−Zinc (24)	DMC	473	199	8.0	−0.04 (−0.08, −0.01)	0.08	0.08	272	175	26.3	−0.14 (−0.22, −0.06)	0.05	0.10
Ghana	GHANA (25)													
Ghana	iLiNS−DYAD−G (26)	PSED	213	410	10.5	0.01 (−0.04, 0.06)	0.04	0.04	119	247	9.3	−0.02 (−0.08, 0.04)	0.08	0.12
Haiti	HAITI (27)													
Kenya	WASH−B (28)	EASQ	809	2682	7.3	−0.01 (−0.03, 0.01)	0.26	0.26	552	2060	14.2	−0.01 (−0.05, 0.02)	0.28	0.16
Madagascar	MAHAY (29)	ASQI	843	839	7.6	0.00 (−0.04, 0.04)	0.08	0.08	770	764	11.1	0.03 (−0.03, 0.09)	0.08	0.12
Malawi	iLiNS−DYAD−M (30)	PSED	137	290	12.4	−0.02 (−0.09, 0.04)	0.03	0.03	78	148	6.1	0.04 (−0.03, 0.11)	0.06	0.10
Malawi	iLiNS−DOSE (31)	PSED	376	135	11.1	0.03 (−0.04, 0.09)	0.03	0.03	268	86	7.0	−0.02 (−0.07, 0.04)	0.10	0.13
Mali	PROMIS CS (32)	DMC												
Zimbabwe	SHINE (HIV−) (33)	MDAT												
Zimbabwe	SHINE (HIV+) (34)	MDAT												
			**4641**	**6736**		**I² = 0.00, Tau² = 0.00**			**2989**	**5376**		**I² = 0.51, Tau² = 0.00**		
**Fixed**						**−0.01 (−0.02, 0.00)**						**−0.02 (−0.03, 0.00)**		
**Random**						**−0.01 (−0.02, 0.00)**						**−0.02 (−0.05, 0.01)**		

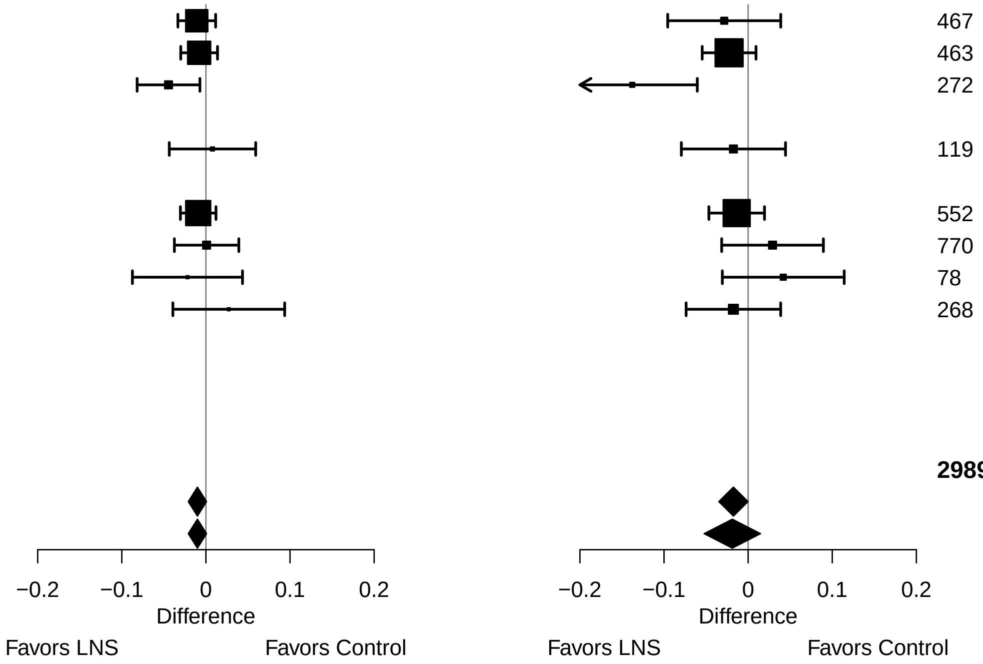

Supplemental figure 8F: Social-emotional lowest decile prevalence difference

8F6: Stratified by Season at the time of assessment

P−for−interaction = 0.493
Difference in PDs = 0.01 (−0.01, 0.03)

Country	Trial	Tool	Dry LNS N	Dry Control N	Dry Control Prevalence	Dry PD (95% CI)	Dry Fixed W	Dry Random W	Rainy LNS N	Rainy Control N	Rainy Control Prevalence	Rainy PD (95% CI)	Rainy Fixed W	Rainy Random W
Bangladesh	JiVitA−4 (21)													
Bangladesh	RDNS (22)	DMC	737	359	12.8	−0.03 (−0.07, 0.01)	0.18	0.18	920	456	10.3	−0.01 (−0.04, 0.02)	0.21	0.22
Bangladesh	WASH−B (23)	EASQ	604	1891	11.3	−0.02 (−0.05, 0.01)	0.29	0.27	462	1375	9.4	−0.02 (−0.05, 0.01)	0.23	0.23
Burkina Faso	iLiNS−Zinc (24)	DMC	514	274	15.7	−0.09 (−0.15, −0.03)	0.07	0.08	232	101	18.8	−0.12 (−0.24, 0.00)	0.01	0.04
Ghana	GHANA (25)													
Ghana	iLiNS−DYAD−G (26)	PSED	195	387	10.6	−0.01 (−0.06, 0.04)	0.10	0.11	137	270	9.3	0.01 (−0.05, 0.07)	0.05	0.11
Haiti	HAITI (27)													
Kenya	WASH−B (28)	EASQ	315	1059	15.2	−0.04 (−0.09, 0.00)	0.14	0.15	1046	3682	8.9	−0.01 (−0.03, 0.02)	0.43	0.27
Madagascar	MAHAY (29)	ASQI												
Malawi	iLiNS−DYAD−M (30)	PSED												
Malawi	iLiNS−DOSE (31)	PSED												
Mali	PROMIS CS (32)	DMC												
Zimbabwe	SHINE (HIV−) (33)	MDAT	603	568	11.1	−0.02 (−0.06, 0.01)	0.22	0.21	238	231	13.9	−0.06 (−0.12, −0.01)	0.06	0.12
Zimbabwe	SHINE (HIV+) (34)	MDAT												
			**2968**	**4538**		**I² = 0.06, Tau² = 0.00**			**3035**	**6115**		**I² = 0.33, Tau² = 0.00**		
**Fixed**						**−0.03 (−0.05, −0.01)**						**−0.01 (−0.03, 0.00)**		
**Random**						**−0.03 (−0.05, −0.01)**						**−0.02 (−0.04, 0.00)**		

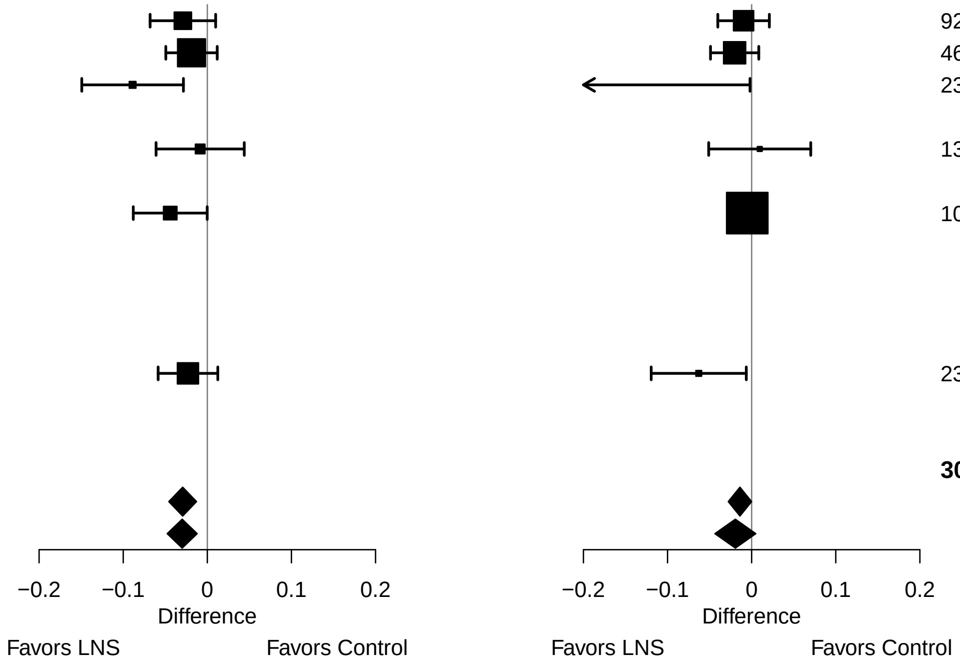

Supplemental figure 8G: Mean difference in motor z-score

8G1: Stratified by Household socio-economic status

P−for−interaction = 0.077
Difference in MDs = 0.05 (−0.01, 0.11)

Country	Trial	Tool	At least median: LNS N	Control N	Control Mean	MD (95% CI)	Fixed W	Random W	Less than median: LNS N	Control N	Control Mean	MD (95% CI)	Fixed W	Random W
Bangladesh	JiVitA−4 (21)	BSID−III	236	77	0.21	−0.09 (−0.29, 0.11)	0.04	0.07	208	66	−0.21	0.07 (−0.19, 0.32)	0.03	0.03
Bangladesh	RDNS (22)	DMC	783	381	0.04	0.09 (−0.01, 0.19)	0.16	0.13	773	372	−0.13	0.11 (0.01, 0.21)	0.20	0.20
Bangladesh	WASH−B (23)	EASQ	546	1663	0.12	0.05 (−0.04, 0.14)	0.20	0.14	528	1616	−0.17	0.16 (0.07, 0.26)	0.23	0.23
Burkina Faso	iLiNS−Zinc (24)	DMC	459	163	−0.16	0.36 (0.15, 0.58)	0.03	0.06	287	212	−0.34	0.38 (0.15, 0.60)	0.04	0.04
Ghana	GHANA (25)													
Ghana	iLiNS−DYAD−G (26)	KDI	143	311	0.01	0.09 (−0.11, 0.29)	0.04	0.07	159	288	−0.02	−0.03 (−0.22, 0.16)	0.06	0.06
Haiti	HAITI (27)													
Kenya	WASH−B (28)	EASQ	778	2706	0.07	−0.03 (−0.10, 0.04)	0.30	0.15	582	2036	−0.10	0.07 (−0.04, 0.17)	0.19	0.19
Madagascar	MAHAY (29)	ASQI	741	845	0.14	0.07 (−0.10, 0.24)	0.05	0.08	823	724	−0.25	0.13 (−0.12, 0.38)	0.03	0.03
Malawi	iLiNS−DYAD−M (30)	KDI	118	227	−0.02	0.02 (−0.17, 0.22)	0.04	0.07	96	208	0.01	0.03 (−0.22, 0.29)	0.03	0.03
Malawi	iLiNS−DOSE (31)	KDI	278	94	0.11	0.05 (−0.18, 0.27)	0.03	0.06	271	96	−0.15	0.07 (−0.16, 0.31)	0.04	0.04
Mali	PROMIS CS (32)	DMC	479	445	−0.03	0.15 (−0.06, 0.37)	0.03	0.06	423	476	−0.09	0.10 (−0.09, 0.28)	0.06	0.06
Zimbabwe	SHINE (HIV−) (33)	MDAT	406	359	−0.04	0.13 (−0.02, 0.28)	0.07	0.09	389	391	−0.08	0.11 (−0.03, 0.26)	0.09	0.09
Zimbabwe	SHINE (HIV+) (34)	MDAT	91	78	−0.11	0.30 (−0.01, 0.61)	0.02	0.03	74	67	−0.18	0.26 (−0.07, 0.60)	0.02	0.02
			**5058**	**7349**		**I² = 0.48, Tau² = 0.01**			**4613**	**6552**		**I² = 0.00, Tau² = 0.00**		
**Fixed**						**0.05 (0.01, 0.09)**						**0.11 (0.07, 0.16)**		
**Random**						**0.08 (0.01, 0.14)**						**0.11 (0.07, 0.16)**		

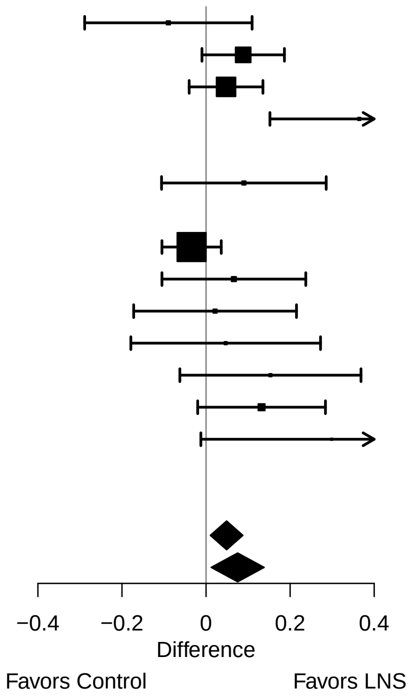

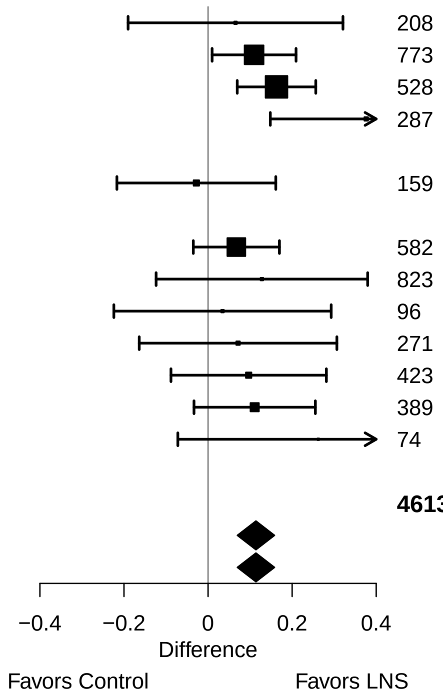

Supplemental figure 8G: Mean difference in motor z-score

8G2: Stratified by Household food insecurity

P−for−interaction = 0.898
Difference in MDs = 0.00 (−0.07, 0.08)

Country	Trial	Tool	Mild to secure: LNS N	Mild to secure: Control N	Mild to secure: Control Mean	Mild to secure: MD (95% CI)	Mild to secure: Fixed W	Mild to secure: Random W	Moderate to severe: LNS N	Moderate to severe: Control N	Moderate to severe: Control Mean	Moderate to severe: MD (95% CI)	Moderate to severe: Fixed W	Moderate to severe: Random W
Bangladesh	JiVitA−4 (21)	BSID−III	329	111	0.07	−0.04 (−0.22, 0.14)	0.04	0.09	115	32	−0.15	0.06 (−0.29, 0.42)	0.03	0.03
Bangladesh	RDNS (22)	DMC	987	456	0.05	0.04 (−0.06, 0.15)	0.12	0.12	569	297	−0.18	0.18 (0.07, 0.28)	0.32	0.32
Bangladesh	WASH−B (23)	EASQ	853	2550	0.01	0.11 (0.05, 0.18)	0.29	0.13	221	729	−0.14	0.05 (−0.11, 0.21)	0.14	0.14
Burkina Faso	iLiNS−Zinc (24)	DMC	389	162	−0.30	0.47 (0.22, 0.73)	0.02	0.06	357	213	−0.23	0.34 (0.11, 0.56)	0.07	0.07
Ghana	GHANA (25)													
Ghana	iLiNS−DYAD−G (26)	KDI	217	412	0.02	0.07 (−0.08, 0.22)	0.06	0.10	84	186	−0.05	−0.09 (−0.38, 0.20)	0.04	0.04
Haiti	HAITI (27)													
Kenya	WASH−B (28)	EASQ	1214	4244	0.01	0.01 (−0.06, 0.08)	0.30	0.13	146	487	−0.11	0.01 (−0.19, 0.21)	0.09	0.09
Madagascar	MAHAY (29)	ASQI	940	892	0.05	0.04 (−0.16, 0.24)	0.03	0.08	336	389	−0.27	0.12 (−0.23, 0.48)	0.03	0.03
Malawi	iLiNS−DYAD−M (30)	KDI	67	116	−0.01	−0.02 (−0.31, 0.26)	0.02	0.05	146	314	0.00	0.04 (−0.15, 0.23)	0.10	0.10
Malawi	iLiNS−DOSE (31)	KDI	134	51	0.11	−0.01 (−0.31, 0.28)	0.02	0.05	403	133	−0.06	0.09 (−0.12, 0.29)	0.09	0.09
Mali	PROMIS CS (32)	DMC												
Zimbabwe	SHINE (HIV−) (33)	MDAT	631	586	−0.04	0.12 (0.00, 0.25)	0.08	0.11	140	154	−0.14	0.11 (−0.09, 0.31)	0.09	0.09
Zimbabwe	SHINE (HIV+) (34)	MDAT	119	105	−0.15	0.36 (0.12, 0.59)	0.02	0.07	41	37	−0.14	0.07 (−0.44, 0.58)	0.01	0.01
			**5880**	**9685**		**I² = 0.57, Tau² = 0.01**			**2558**	**2971**		**I² = 0.00, Tau² = 0.00**		
**Fixed**						**0.07 (0.04, 0.11)**						**0.11 (0.05, 0.17)**		
**Random**						**0.09 (0.01, 0.18)**						**0.11 (0.05, 0.17)**		

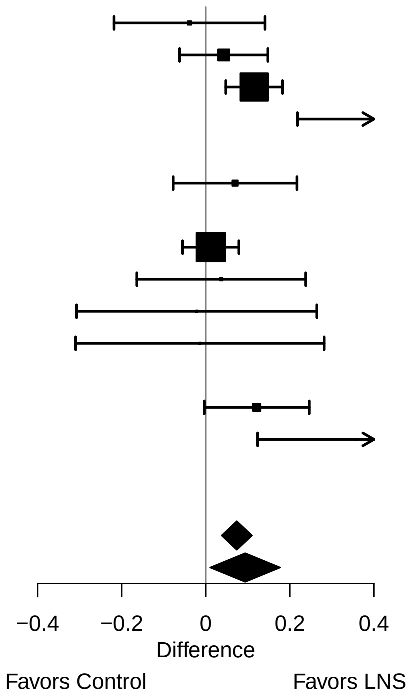

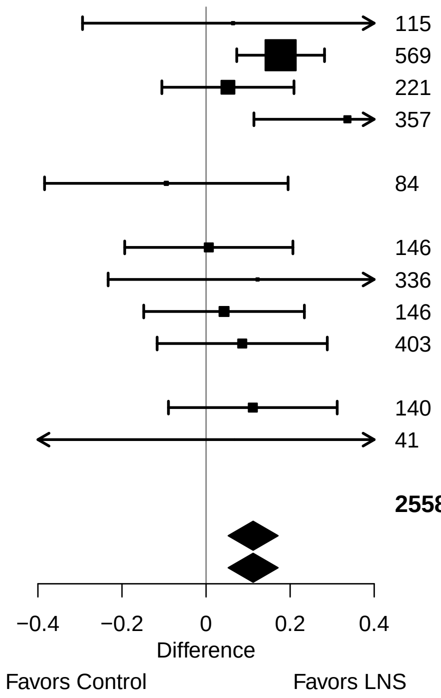

Supplemental figure 8G: Mean difference in motor z-score

8G3: Stratified by Household source water quality

P−for−interaction = 0.514
Difference in MDs = 0.04 (−0.07, 0.14)

Country	Trial	Tool	Improved LNS N	Improved Control N	Improved Control Mean	Improved MD (95% CI)	Improved Fixed W	Improved Random W	Unimproved LNS N	Unimproved Control N	Unimproved Control Mean	Unimproved MD (95% CI)	Unimproved Fixed W	Unimproved Random W
Bangladesh	JiVitA−4 (21)	BSID−III												
Bangladesh	RDNS (22)	DMC												
Bangladesh	WASH−B (23)	EASQ	460	981	−0.09	0.20 (0.08, 0.31)	0.25	0.21	67	117	−0.12	0.20 (−0.08, 0.48)	0.08	0.10
Burkina Faso	iLiNS−Zinc (24)	DMC	220	82	−0.07	0.32 (0.07, 0.57)	0.05	0.07	526	293	−0.32	0.41 (0.25, 0.57)	0.26	0.21
Ghana	GHANA (25)													
Ghana	iLiNS−DYAD−G (26)	KDI												
Haiti	HAITI (27)													
Kenya	WASH−B (28)	EASQ	413	1419	−0.01	0.02 (−0.10, 0.14)	0.25	0.21	236	649	0.05	0.04 (−0.12, 0.21)	0.23	0.20
Madagascar	MAHAY (29)	ASQI	405	430	−0.10	0.08 (−0.26, 0.41)	0.03	0.04	1134	1133	−0.01	0.08 (−0.13, 0.29)	0.14	0.15
Malawi	iLiNS−DYAD−M (30)	KDI	194	398	0.02	−0.01 (−0.17, 0.16)	0.13	0.13	20	36	−0.16	0.32 (−0.24, 0.87)	0.02	0.03
Malawi	iLiNS−DOSE (31)	KDI	515	177	0.00	0.06 (−0.11, 0.23)	0.12	0.13	44	15	−0.26	0.21 (−0.41, 0.84)	0.02	0.02
Mali	PROMIS CS (32)	DMC	514	539	−0.04	0.16 (−0.07, 0.38)	0.07	0.08	347	368	−0.12	0.07 (−0.15, 0.28)	0.14	0.15
Zimbabwe	SHINE (HIV−) (33)	MDAT	228	220	0.11	−0.04 (−0.24, 0.16)	0.08	0.10	131	122	−0.11	0.10 (−0.18, 0.38)	0.08	0.10
Zimbabwe	SHINE (HIV+) (34)	MDAT	37	41	−0.01	0.04 (−0.34, 0.42)	0.02	0.03	25	25	−0.25	−0.10 (−0.70, 0.50)	0.02	0.03
			**2986**	**4287**		**I² = 0.28, Tau² = 0.00**			**2530**	**2758**		**I² = 0.42, Tau² = 0.01**		
**Fixed**						**0.09 (0.03, 0.15)**						**0.17 (0.09, 0.25)**		
**Random**						**0.09 (0.02, 0.16)**						**0.16 (0.06, 0.26)**		

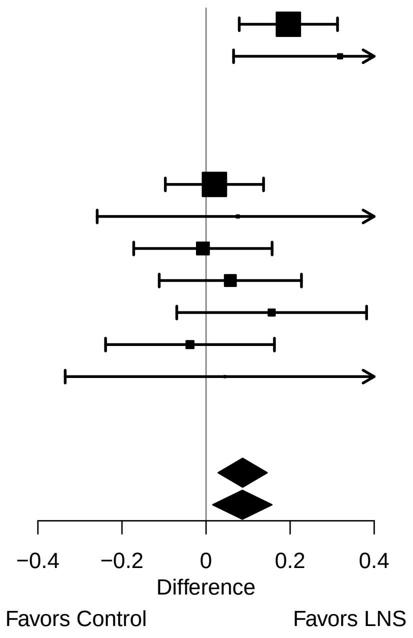

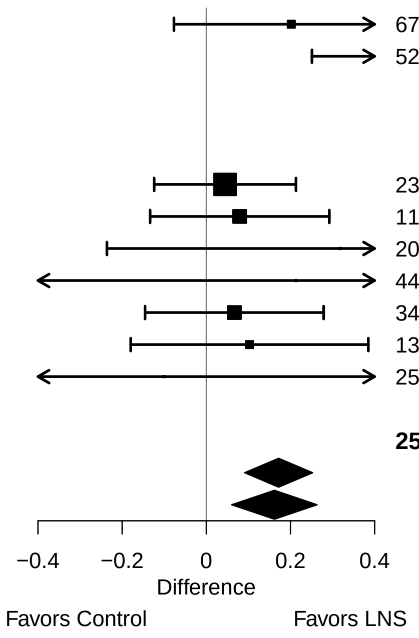

Supplemental figure 8G: Mean difference in motor z-score

8G4: Stratified by Household sanitation

P−for−interaction = 0.193
Difference in MDs = −0.07 (−0.17, 0.03)

Country	Trial	Tool	Improved LNS N	Improved Control N	Improved Control Mean	Improved MD (95% CI)	Improved Fixed W	Improved Random W	Unimproved LNS N	Unimproved Control N	Unimproved Control Mean	Unimproved MD (95% CI)	Unimproved Fixed W	Unimproved Random W
Bangladesh	JiVitA−4 (21)	BSID−III	353	112	0.08	−0.02 (−0.18, 0.14)	0.09	0.11	91	31	−0.20	−0.02 (−0.56, 0.52)	0.01	0.02
Bangladesh	RDNS (22)	DMC	1103	531	−0.01	0.11 (0.03, 0.18)	0.41	0.30	451	222	−0.11	0.08 (−0.05, 0.21)	0.18	0.16
Bangladesh	WASH−B (23)	EASQ	472	998	−0.09	0.21 (0.10, 0.32)	0.20	0.20	29	53	−0.20	0.18 (−0.39, 0.74)	0.01	0.02
Burkina Faso	iLiNS−Zinc (24)	DMC	16	11	−0.37	0.74 (−0.01, 1.49)	0.00	0.01	730	364	−0.26	0.39 (0.22, 0.56)	0.11	0.12
Ghana	GHANA (25)													
Ghana	iLiNS−DYAD−G (26)	KDI	292	586	−0.01	0.03 (−0.11, 0.16)	0.12	0.14	9	13	0.07	0.02 (−0.74, 0.79)	0.01	0.01
Haiti	HAITI (27)													
Kenya	WASH−B (28)	EASQ	94	337	0.12	0.09 (−0.13, 0.30)	0.05	0.07	555	1732	−0.01	0.02 (−0.08, 0.13)	0.31	0.20
Madagascar	MAHAY (29)	ASQI												
Malawi	iLiNS−DYAD−M (30)	KDI	19	41	−0.13	0.27 (−0.16, 0.69)	0.01	0.02	195	393	0.02	0.00 (−0.17, 0.16)	0.11	0.13
Malawi	iLiNS−DOSE (31)	KDI	12	9	0.31	0.19 (−0.44, 0.83)	0.01	0.01	547	183	−0.04	0.08 (−0.09, 0.24)	0.11	0.13
Mali	PROMIS CS (32)	DMC	665	658	−0.08	0.17 (−0.03, 0.37)	0.06	0.08	210	241	0.00	−0.01 (−0.26, 0.25)	0.05	0.07
Zimbabwe	SHINE (HIV−) (33)	MDAT	133	108	0.05	0.08 (−0.17, 0.34)	0.04	0.05	225	235	0.02	−0.03 (−0.22, 0.16)	0.09	0.11
Zimbabwe	SHINE (HIV+) (34)	MDAT	25	17	−0.02	−0.22 (−0.78, 0.34)	0.01	0.01	37	49	−0.13	0.09 (−0.28, 0.46)	0.02	0.04
			**3184**	**3408**		**I² = 0.17, Tau² = 0.00**			**3079**	**3516**		**I² = 0.42, Tau² = 0.01**		
**Fixed**						**0.11 (0.06, 0.16)**						**0.07 (0.02, 0.13)**		
**Random**						**0.11 (0.05, 0.17)**						**0.08 (0.00, 0.16)**		

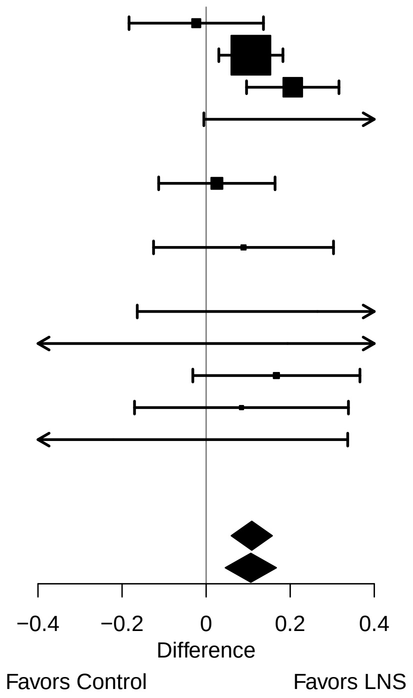

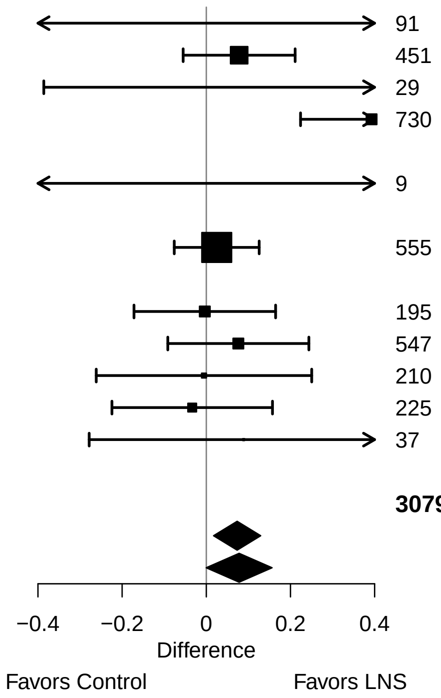

Supplemental figure 8G: Mean difference in motor z-score

8G5: Stratified by Home environment

P−for−interaction = 0.565
Difference in MDs = −0.02 (−0.09, 0.05)

						At least median						Less than median		
Country	Trial	Tool	LNS N	Control N	Control Mean	MD (95% CI)	Fixed W	Random W	LNS N	Control N	Control Mean	MD (95% CI)	Fixed W	Random W
Bangladesh	JiVitA−4 (21)	BSID−III												
Bangladesh	RDNS (22)	DMC	1115	548	0.07	0.08 (0.02, 0.14)	0.38	0.34	439	203	−0.35	0.17 (−0.01, 0.34)	0.10	0.13
Bangladesh	WASH−B (23)	EASQ	605	1614	0.13	0.12 (0.02, 0.21)	0.16	0.17	469	1664	−0.17	0.04 (−0.06, 0.14)	0.33	0.21
Burkina Faso	iLiNS−Zinc (24)	DMC	473	199	0.11	0.24 (0.07, 0.41)	0.05	0.06	272	175	−0.67	0.45 (0.18, 0.73)	0.04	0.08
Ghana	GHANA (25)													
Ghana	iLiNS−DYAD−G (26)	KDI	191	377	0.10	0.05 (−0.10, 0.21)	0.06	0.07	111	224	−0.18	−0.02 (−0.27, 0.24)	0.05	0.09
Haiti	HAITI (27)													
Kenya	WASH−B (28)	EASQ	809	2682	0.15	0.01 (−0.07, 0.09)	0.23	0.23	552	2060	−0.20	−0.01 (−0.11, 0.09)	0.32	0.21
Madagascar	MAHAY (29)	ASQI	843	839	0.05	0.11 (−0.12, 0.34)	0.03	0.03	770	764	−0.13	0.03 (−0.17, 0.24)	0.08	0.12
Malawi	iLiNS−DYAD−M (30)	KDI	136	287	0.11	0.02 (−0.15, 0.20)	0.05	0.05	78	149	−0.22	0.05 (−0.25, 0.36)	0.03	0.07
Malawi	iLiNS−DOSE (31)	KDI	376	135	0.03	0.06 (−0.13, 0.25)	0.04	0.05	269	86	−0.14	0.04 (−0.21, 0.29)	0.05	0.09
Mali	PROMIS CS (32)	DMC												
Zimbabwe	SHINE (HIV−) (33)	MDAT												
Zimbabwe	SHINE (HIV+) (34)	MDAT												
			**4548**	**6681**		**I² = 0.10, Tau² = 0.00**			**2960**	**5325**		**I² = 0.39, Tau² = 0.01**		
**Fixed**						**0.07 (0.04, 0.11)**						**0.05 (−0.01, 0.11)**		
**Random**						**0.07 (0.03, 0.12)**						**0.07 (−0.02, 0.16)**		

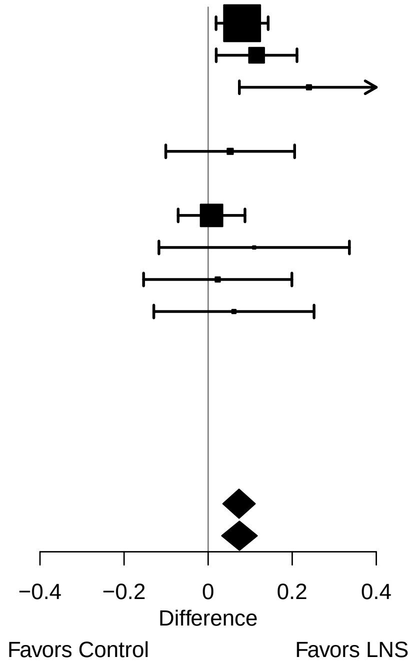

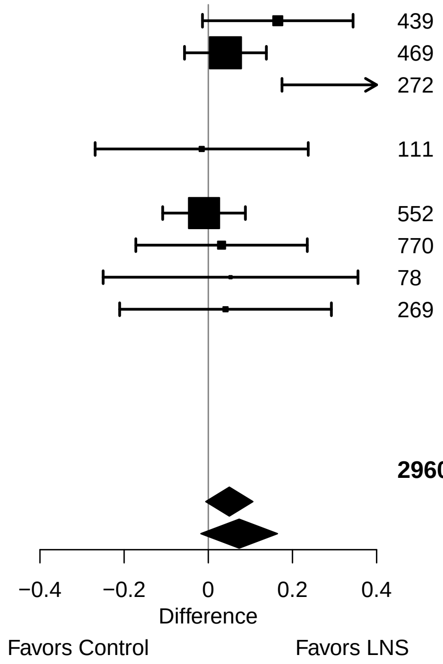

Supplemental figure 8G: Mean difference in motor z-score

8G6: Stratified by Season at the time of assessment

P−for−interaction = 0.827
Difference in MDs = −0.01 (−0.07, 0.05)

			Dry						Rainy					
**Country**	**Trial**	**Tool**	**LNS N**	**Control N**	**Control Mean**	**MD (95% CI)**	**Fixed W**	**Random W**	**LNS N**	**Control N**	**Control Mean**	**MD (95% CI)**	**Fixed W**	**Random W**
Bangladesh	JiVitA−4 (21)	BSID−III	251	70	0.26	−0.03 (−0.22, 0.17)	0.05	0.08	194	73	−0.22	−0.09 (−0.38, 0.20)	0.02	0.05
Bangladesh	RDNS (22)	DMC	698	326	−0.02	0.09 (−0.01, 0.18)	0.20	0.13	858	427	−0.06	0.11 (0.02, 0.20)	0.25	0.18
Bangladesh	WASH−B (23)	EASQ	608	1896	0.04	0.08 (−0.01, 0.17)	0.24	0.14	466	1383	−0.11	0.14 (0.04, 0.24)	0.19	0.17
Burkina Faso	iLiNS−Zinc (24)	DMC	514	274	−0.31	0.42 (0.21, 0.63)	0.04	0.08	232	101	−0.13	0.34 (0.15, 0.54)	0.05	0.09
Ghana	GHANA (25)													
Ghana	iLiNS−DYAD−G (26)	KDI	177	356	0.02	−0.02 (−0.20, 0.15)	0.06	0.09	125	245	−0.04	0.11 (−0.12, 0.33)	0.04	0.08
Haiti	HAITI (27)													
Kenya	WASH−B (28)	EASQ	315	1059	−0.30	0.09 (−0.01, 0.20)	0.17	0.13	1046	3682	0.08	−0.01 (−0.09, 0.07)	0.33	0.20
Madagascar	MAHAY (29)	ASQI												
Malawi	iLiNS−DYAD−M (30)	KDI	153	308	0.02	0.04 (−0.15, 0.23)	0.05	0.08	61	127	−0.06	−0.01 (−0.30, 0.28)	0.02	0.05
Malawi	iLiNS−DOSE (31)	KDI	395	118	−0.09	0.01 (−0.19, 0.21)	0.04	0.08	244	100	0.02	0.14 (−0.11, 0.38)	0.03	0.07
Mali	PROMIS CS (32)	DMC												
Zimbabwe	SHINE (HIV−) (33)	MDAT	603	568	−0.08	0.14 (0.02, 0.25)	0.13	0.12	238	231	0.03	0.02 (−0.18, 0.23)	0.05	0.08
Zimbabwe	SHINE (HIV+) (34)	MDAT	121	110	−0.19	0.30 (0.05, 0.55)	0.03	0.06	49	41	−0.09	0.32 (−0.07, 0.71)	0.01	0.03
			**3835**	**5085**		**I² = 0.46, Tau² = 0.01**			**3513**	**6410**		**I² = 0.49, Tau² = 0.01**		
**Fixed**						**0.09 (0.05, 0.14)**						**0.08 (0.04, 0.12)**		
**Random**						**0.10 (0.02, 0.18)**						**0.10 (0.02, 0.17)**		

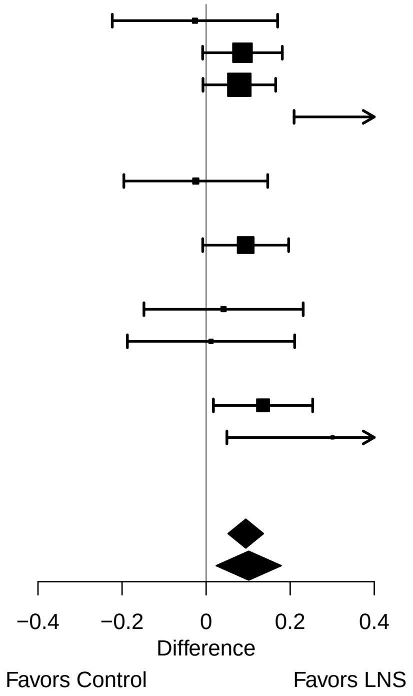

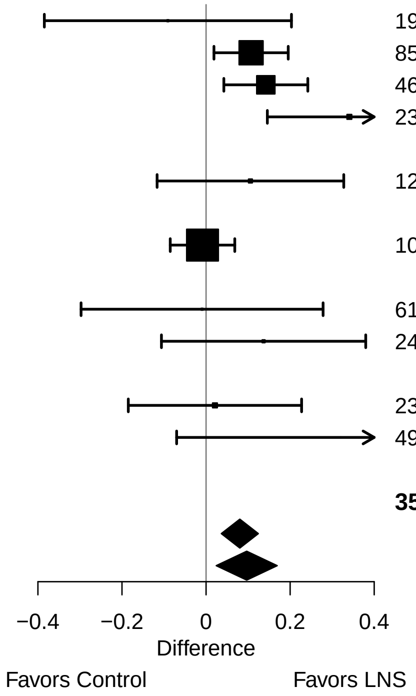

Supplemental figure 8H: Motor lowest decile prevalence ratio

8H1: Stratified by Household socio-economic status

P−for−interaction = 0.878
Ratio of PRs = 0.99 (0.83, 1.17)

Country	Trial	Tool	At least median: LNS N	Control N	Control Prevalence	PR (95% CI)	Fixed W	Random W	Less than median: LNS N	Control N	Control Prevalence	PR (95% CI)	Fixed W	Random W
Bangladesh	JiVitA−4 (21)	BSID−III												
Bangladesh	RDNS (22)	DMC	783	381	7.1	1.08 (0.71, 1.65)	0.10	0.11	773	372	15.3	0.73 (0.56, 0.97)	0.19	0.19
Bangladesh	WASH−B (23)	EASQ	546	1663	7.6	0.79 (0.57, 1.09)	0.17	0.15	528	1616	13.6	0.83 (0.63, 1.11)	0.18	0.18
Burkina Faso	iLiNS−Zinc (24)	DMC	459	163	16.0	0.38 (0.24, 0.62)	0.08	0.09	287	212	14.6	0.67 (0.39, 1.13)	0.05	0.05
Ghana	GHANA (25)													
Ghana	iLiNS−DYAD−G (26)	KDI	143	311	10.0	0.98 (0.54, 1.79)	0.05	0.07	159	288	9.0	1.32 (0.76, 2.31)	0.05	0.05
Haiti	HAITI (27)													
Kenya	WASH−B (28)	EASQ	778	2706	9.3	0.84 (0.65, 1.08)	0.28	0.19	582	2036	11.6	0.89 (0.69, 1.14)	0.23	0.23
Madagascar	MAHAY (29)	ASQI	741	845	6.2	0.90 (0.50, 1.62)	0.05	0.07	823	724	15.7	0.84 (0.52, 1.36)	0.06	0.06
Malawi	iLiNS−DYAD−M (30)	KDI	118	227	8.8	0.96 (0.47, 1.99)	0.03	0.05	96	208	12.5	0.75 (0.37, 1.54)	0.03	0.03
Malawi	iLiNS−DOSE (31)	KDI	278	94	6.4	1.35 (0.57, 3.21)	0.02	0.04	271	96	12.5	0.83 (0.44, 1.56)	0.04	0.04
Mali	PROMIS CS (32)	DMC	479	445	11.0	0.83 (0.52, 1.33)	0.08	0.10	423	476	11.1	0.79 (0.51, 1.20)	0.08	0.08
Zimbabwe	SHINE (HIV−) (33)	MDAT	406	359	10.6	0.93 (0.59, 1.46)	0.09	0.10	389	391	11.3	0.87 (0.58, 1.30)	0.09	0.09
Zimbabwe	SHINE (HIV+) (34)	MDAT	91	78	11.5	0.67 (0.27, 1.62)	0.02	0.03	74	67	13.4	0.70 (0.27, 1.82)	0.02	0.02
			**4822**	**7272**		**I² = 0.29, Tau² = 0.03**			**4405**	**6486**		**I² = 0.00, Tau² = 0.00**		
**Fixed**						**0.83 (0.72, 0.95)**						**0.83 (0.74, 0.93)**		
**Random**						**0.83 (0.69, 0.99)**						**0.83 (0.74, 0.93)**		

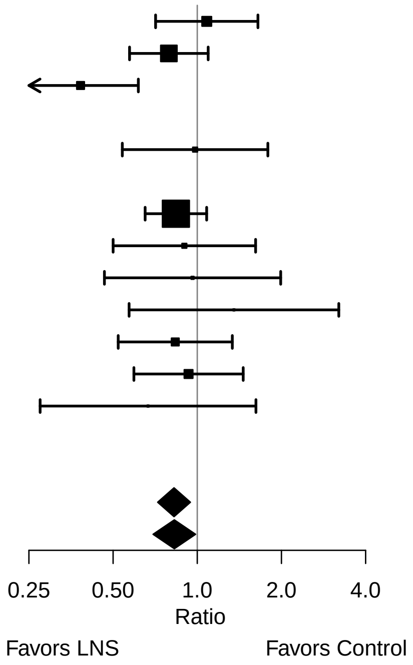

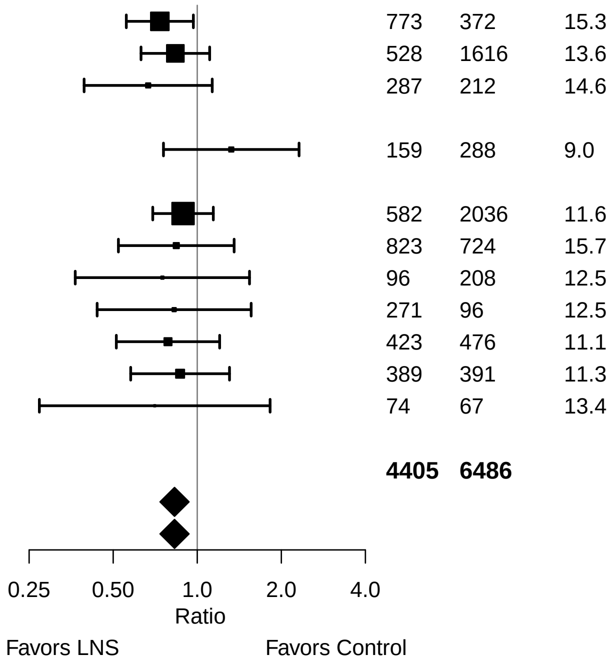

Supplemental figure 8H: Motor lowest decile prevalence ratio

8H2: Stratified by Household food insecurity

P−for−interaction = 0.594
Ratio of PRs = 0.94 (0.75, 1.18)

Country	Trial	Tool	Mild to secure: LNS N	Mild to secure: Control N	Mild to secure: Control Prevalence	Mild to secure: PR (95% CI)	Mild to secure: Fixed W	Mild to secure: Random W	Moderate to severe: LNS N	Moderate to severe: Control N	Moderate to severe: Control Prevalence	Moderate to severe: PR (95% CI)	Moderate to severe: Fixed W	Moderate to severe: Random W
Bangladesh	JiVitA−4 (21)	BSID−III												
Bangladesh	RDNS (22)	DMC	987	456	7.2	1.15 (0.79, 1.66)	0.11	0.14	569	297	17.2	0.67 (0.47, 0.94)	0.27	0.26
Bangladesh	WASH−B (23)	EASQ	853	2550	10.0	0.79 (0.63, 0.99)	0.27	0.19	221	729	12.8	0.92 (0.62, 1.37)	0.20	0.19
Burkina Faso	iLiNS−Zinc (24)	DMC	389	162	15.4	0.43 (0.28, 0.66)	0.08	0.12	357	213	15.0	0.56 (0.34, 0.93)	0.12	0.13
Ghana	GHANA (25)													
Ghana	iLiNS−DYAD−G (26)	KDI	217	412	9.2	1.00 (0.60, 1.67)	0.05	0.09	84	186	10.2	1.52 (0.79, 2.92)	0.07	0.08
Haiti	HAITI (27)													
Kenya	WASH−B (28)	EASQ	1214	4244	10.0	0.88 (0.71, 1.08)	0.31	0.19	146	487	13.6	0.76 (0.45, 1.29)	0.11	0.12
Madagascar	MAHAY (29)	ASQI	940	892	9.0	0.90 (0.53, 1.53)	0.05	0.09	336	389	15.4	0.93 (0.50, 1.73)	0.08	0.08
Malawi	iLiNS−DYAD−M (30)	KDI	67	116	11.2	1.07 (0.47, 2.44)	0.02	0.05	146	314	10.2	0.74 (0.38, 1.43)	0.07	0.08
Malawi	iLiNS−DOSE (31)	KDI												
Mali	PROMIS CS (32)	DMC												
Zimbabwe	SHINE (HIV−) (33)	MDAT	631	586	10.4	0.96 (0.66, 1.39)	0.10	0.13	140	154	13.0	0.72 (0.35, 1.44)	0.06	0.07
Zimbabwe	SHINE (HIV+) (34)	MDAT												
			**5298**	**9418**		**I² = 0.50, Tau² = 0.04**			**1999**	**2769**		**I² = 0.07, Tau² = 0.00**		
**Fixed**						**0.85 (0.75, 0.95)**						**0.78 (0.65, 0.93)**		
**Random**						**0.85 (0.69, 1.04)**						**0.78 (0.65, 0.94)**		

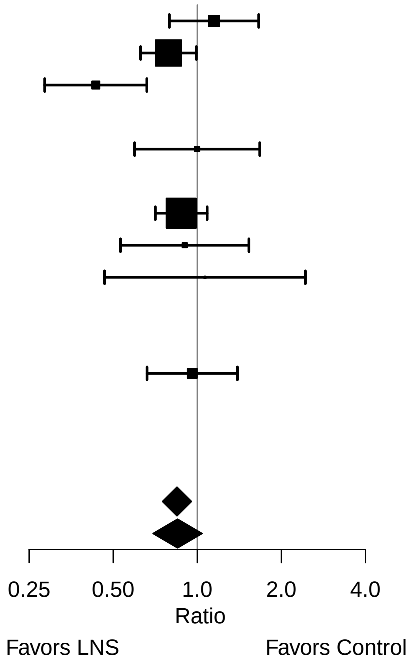

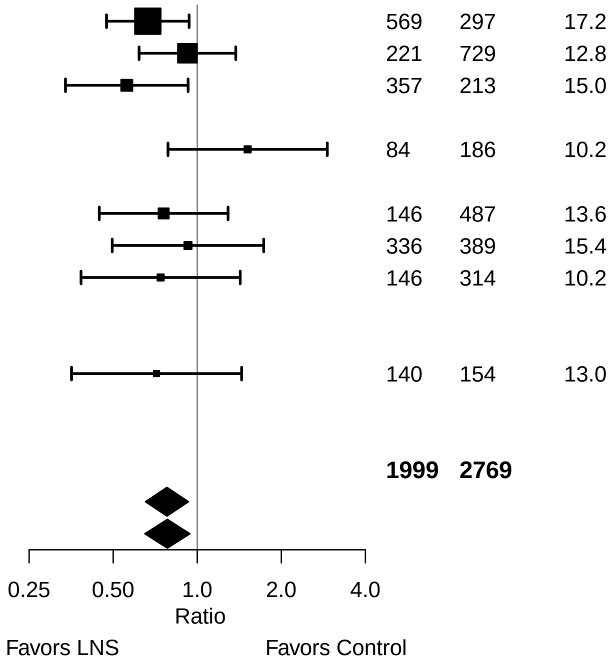

Supplemental figure 8H: Motor lowest decile prevalence ratio

8H3: Stratified by Household source water quality

P−for−interaction = 0.723
Ratio of PRs = 1.06 (0.78, 1.42)

Country	Trial	Tool	Improved LNS N	Improved Control N	Improved Control Prevalence	Improved PR (95% CI)	Improved Fixed W	Improved Random W	Unimproved LNS N	Unimproved Control N	Unimproved Control Prevalence	Unimproved PR (95% CI)	Unimproved Fixed W	Unimproved Random W
Bangladesh	JiVitA−4 (21)	BSID−III												
Bangladesh	RDNS (22)	DMC												
Bangladesh	WASH−B (23)	EASQ	460	981	11.6	0.84 (0.61, 1.17)	0.33	0.31	67	117	12.0	0.62 (0.23, 1.71)	0.04	0.05
Burkina Faso	iLiNS−Zinc (24)	DMC	220	82	12.2	0.41 (0.20, 0.85)	0.07	0.07	526	293	16.0	0.53 (0.39, 0.72)	0.43	0.35
Ghana	GHANA (25)													
Ghana	iLiNS−DYAD−G (26)	KDI												
Haiti	HAITI (27)													
Kenya	WASH−B (28)	EASQ	413	1419	10.2	1.02 (0.71, 1.47)	0.27	0.26	236	649	9.2	0.87 (0.55, 1.37)	0.19	0.21
Madagascar	MAHAY (29)	ASQI	405	430	14.0	0.87 (0.44, 1.70)	0.08	0.09	1134	1133	9.4	0.92 (0.54, 1.58)	0.14	0.16
Malawi	iLiNS−DYAD−M (30)	KDI												
Malawi	iLiNS−DOSE (31)	KDI												
Mali	PROMIS CS (32)	DMC	514	539	10.2	0.69 (0.42, 1.11)	0.15	0.16	347	368	12.8	0.90 (0.52, 1.56)	0.13	0.15
Zimbabwe	SHINE (HIV−) (33)	MDAT	228	220	9.5	0.87 (0.49, 1.55)	0.11	0.12	131	122	9.8	1.16 (0.52, 2.59)	0.06	0.08
Zimbabwe	SHINE (HIV+) (34)	MDAT												
			**2240**	**3671**		**I² = 0.08, Tau² = 0.01**			**2441**	**2682**		**I² = 0.32, Tau² = 0.02**		
**Fixed**						**0.82 (0.68, 0.99)**						**0.72 (0.59, 0.88)**		
**Random**						**0.82 (0.67, 1.00)**						**0.75 (0.59, 0.95)**		

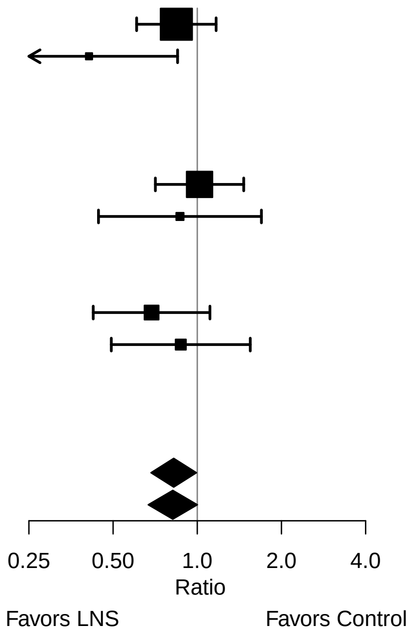

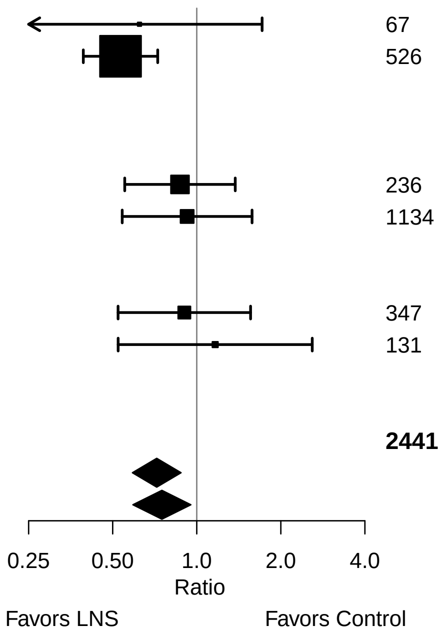

Supplemental figure 8H: Motor lowest decile prevalence ratio

8H4: Stratified by Household sanitation

P−for−interaction = 0.228
Ratio of PRs = 1.24 (0.87, 1.76)

Country	Trial	Tool	Improved LNS N	Improved Control N	Improved Control Prevalence	Improved PR (95% CI)	Improved Fixed W	Improved Random W	Unimproved LNS N	Unimproved Control N	Unimproved Control Prevalence	Unimproved PR (95% CI)	Unimproved Fixed W	Unimproved Random W
Bangladesh	JiVitA−4 (21)	BSID−III												
Bangladesh	RDNS (22)	DMC	1103	531	10.4	0.81 (0.59, 1.12)	0.52	0.52	451	222	13.1	0.92 (0.58, 1.45)	0.22	0.22
Bangladesh	WASH−B (23)	EASQ												
Burkina Faso	iLiNS−Zinc (24)	DMC												
Ghana	GHANA (25)													
Ghana	iLiNS−DYAD−G (26)	KDI												
Haiti	HAITI (27)													
Kenya	WASH−B (28)	EASQ	94	337	9.2	0.81 (0.35, 1.89)	0.07	0.07	555	1732	10.0	0.99 (0.72, 1.34)	0.49	0.49
Madagascar	MAHAY (29)	ASQI												
Malawi	iLiNS−DYAD−M (30)	KDI												
Malawi	iLiNS−DOSE (31)	KDI												
Mali	PROMIS CS (32)	DMC	665	658	10.8	0.81 (0.53, 1.23)	0.31	0.31	210	241	10.8	0.93 (0.54, 1.60)	0.16	0.16
Zimbabwe	SHINE (HIV−) (33)	MDAT	133	108	10.2	0.66 (0.32, 1.39)	0.10	0.10	225	235	9.4	1.19 (0.66, 2.15)	0.13	0.13
Zimbabwe	SHINE (HIV+) (34)	MDAT												
			**1995**	**1634**		**I² = 0.00, Tau² = 0.00**			**1441**	**2430**		**I² = 0.00, Tau² = 0.00**		
**Fixed**						**0.80 (0.63, 1.00)**						**0.98 (0.79, 1.22)**		
**Random**						**0.80 (0.63, 1.00)**						**0.98 (0.79, 1.22)**		

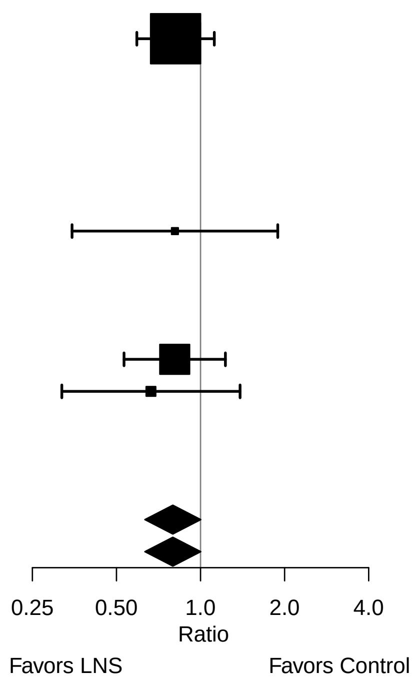

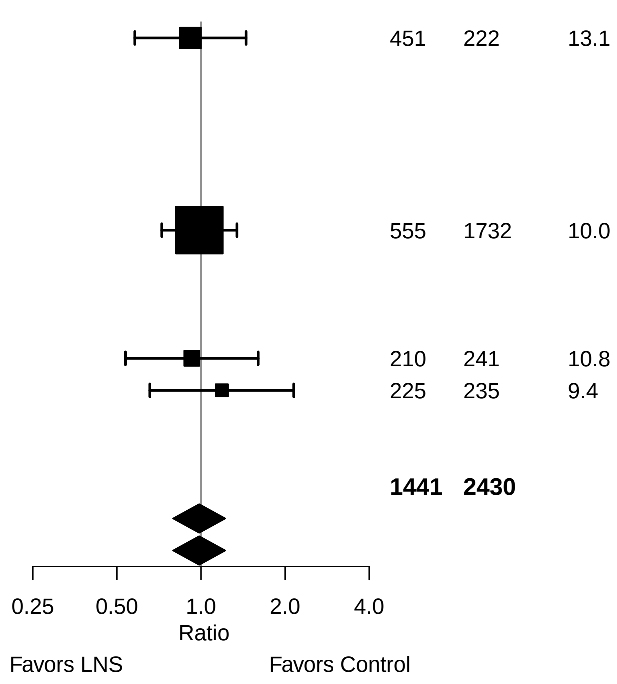

Supplemental figure 8H: Motor lowest decile prevalence ratio

8H5: Stratified by Home environment

P−for−interaction = 0.766
Ratio of PRs = 1.03 (0.84, 1.27)

			At least median						Less than median					
**Country**	**Trial**	**Tool**	**LNS N**	**Control N**	**Control Prevalence**	**PR (95% CI)**	**Fixed W**	**Random W**	**LNS N**	**Control N**	**Control Prevalence**	**PR (95% CI)**	**Fixed W**	**Random W**
Bangladesh	JiVitA−4 (21)	BSID−III												
Bangladesh	RDNS (22)	DMC	1115	548	8.0	0.75 (0.54, 1.04)	0.24	0.23	439	203	19.2	0.94 (0.67, 1.32)	0.16	0.16
Bangladesh	WASH−B (23)	EASQ	605	1614	7.4	0.67 (0.44, 1.00)	0.15	0.15	469	1664	13.6	0.98 (0.75, 1.29)	0.25	0.23
Burkina Faso	iLiNS−Zinc (24)	DMC	473	199	6.5	0.58 (0.34, 1.00)	0.09	0.09	272	175	25.1	0.56 (0.37, 0.83)	0.11	0.12
Ghana	GHANA (25)													
Ghana	iLiNS−DYAD−G (26)	KDI	191	377	6.6	1.18 (0.64, 2.19)	0.07	0.07	111	224	14.7	1.10 (0.65, 1.87)	0.07	0.08
Haiti	HAITI (27)													
Kenya	WASH−B (28)	EASQ	809	2682	7.2	1.04 (0.78, 1.40)	0.29	0.28	552	2060	14.4	0.76 (0.58, 0.99)	0.25	0.23
Madagascar	MAHAY (29)	ASQI	843	839	9.3	0.78 (0.41, 1.46)	0.06	0.07	770	764	11.8	1.03 (0.64, 1.65)	0.08	0.09
Malawi	iLiNS−DYAD−M (30)	KDI	136	287	7.7	0.96 (0.47, 1.97)	0.05	0.05	78	149	16.1	0.72 (0.35, 1.46)	0.04	0.04
Malawi	iLiNS−DOSE (31)	KDI	376	135	8.1	1.08 (0.56, 2.07)	0.06	0.06	269	86	11.6	1.06 (0.54, 2.05)	0.04	0.05
Mali	PROMIS CS (32)	DMC												
Zimbabwe	SHINE (HIV−) (33)	MDAT												
Zimbabwe	SHINE (HIV+) (34)	MDAT												
			**4548**	**6681**		**I² = 0.07, Tau² = 0.00**			**2960**	**5325**		**I² = 0.17, Tau² = 0.01**		
**Fixed**						**0.85 (0.72, 0.99)**						**0.86 (0.75, 0.99)**		
**Random**						**0.84 (0.72, 1.00)**						**0.86 (0.74, 1.00)**		

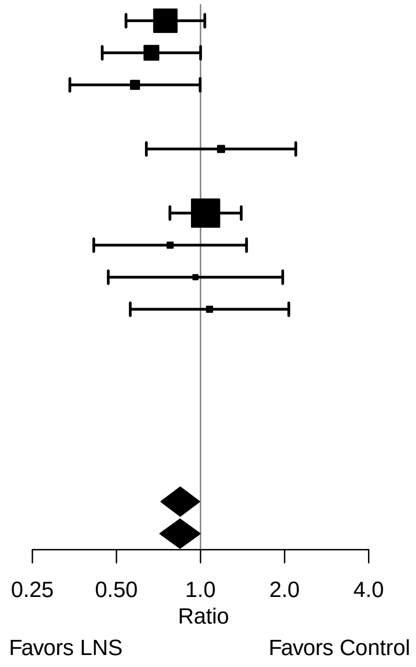

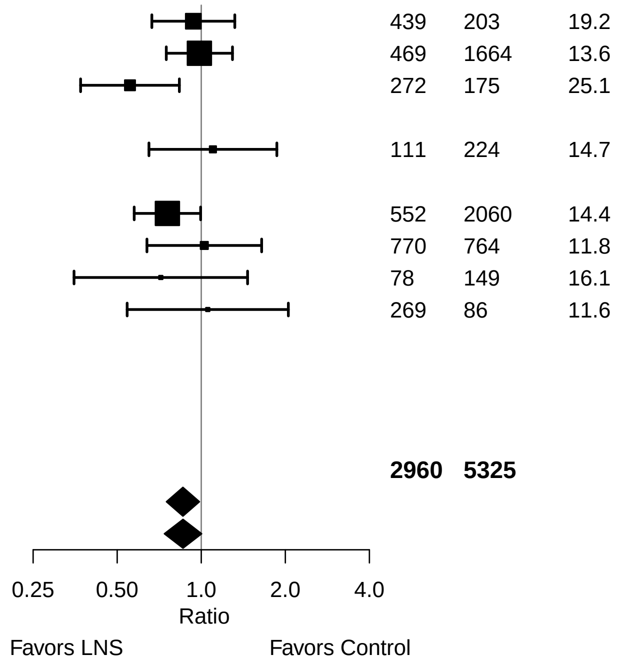

Supplemental figure 8H: Motor lowest decile prevalence ratio

8H6: Stratified by Season at the time of assessment

P−for−interaction = 0.839
Ratio of PRs = 1.02 (0.83, 1.26)

Country	Trial	Tool	LNS N	Control N	Control Prevalence	Dry PR (95% CI)	Fixed W	Random W	LNS N	Control N	Control Prevalence	Rainy PR (95% CI)	Fixed W	Random W
Bangladesh	JiVitA−4 (21)	BSID−III												
Bangladesh	RDNS (22)	DMC	698	326	11.7	0.87 (0.62, 1.22)	0.14	0.15	858	427	10.8	0.82 (0.58, 1.17)	0.20	0.20
Bangladesh	WASH−B (23)	EASQ	608	1896	10.0	0.91 (0.70, 1.18)	0.23	0.19	466	1383	11.4	0.71 (0.50, 1.01)	0.20	0.20
Burkina Faso	iLiNS−Zinc (24)	DMC	514	274	16.8	0.52 (0.37, 0.74)	0.13	0.14	232	101	10.9	0.44 (0.21, 0.89)	0.05	0.05
Ghana	GHANA (25)													
Ghana	iLiNS−DYAD−G (26)	KDI	177	356	9.0	1.19 (0.70, 2.05)	0.06	0.08	125	245	10.6	1.06 (0.57, 1.95)	0.07	0.07
Haiti	HAITI (27)													
Kenya	WASH−B (28)	EASQ	315	1059	17.4	0.71 (0.54, 0.93)	0.22	0.19	1046	3682	8.3	0.93 (0.70, 1.23)	0.32	0.32
Madagascar	MAHAY (29)	ASQI												
Malawi	iLiNS−DYAD−M (30)	KDI	153	308	10.1	0.84 (0.46, 1.57)	0.04	0.06	61	127	11.8	0.83 (0.34, 2.04)	0.03	0.03
Malawi	iLiNS−DOSE (31)	KDI	395	118	10.2	1.07 (0.58, 1.96)	0.04	0.06	244	100	9.0	1.00 (0.48, 2.10)	0.05	0.05
Mali	PROMIS CS (32)	DMC												
Zimbabwe	SHINE (HIV−) (33)	MDAT	603	568	10.9	0.84 (0.58, 1.19)	0.13	0.14	238	231	9.1	1.20 (0.70, 2.07)	0.08	0.08
Zimbabwe	SHINE (HIV+) (34)	MDAT												
			**3463**	**4905**		**I² = 0.35, Tau² = 0.02**			**3270**	**6296**		**I² = 0.00, Tau² = 0.00**		
**Fixed**						**0.80 (0.71, 0.91)**						**0.85 (0.73, 1.00)**		
**Random**						**0.81 (0.69, 0.96)**						**0.85 (0.73, 1.00)**		

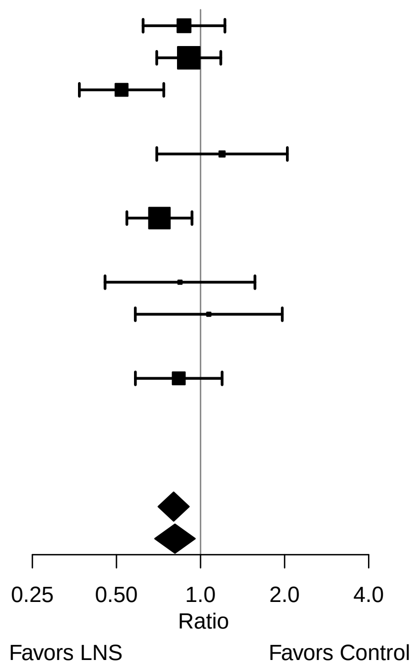

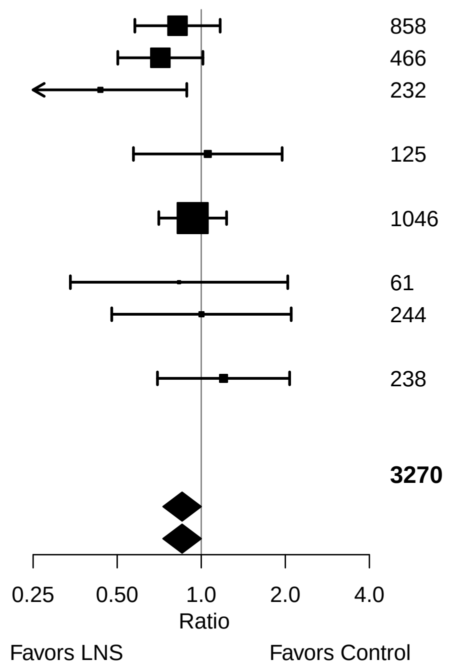

Supplemental figure 8I: Motor lowest decile prevalence difference

8I1: Stratified by Household socio-economic status

P−for−interaction = 0.325
Difference in PDs = −0.01 (−0.03, 0.01)

Country	Trial	Tool	LNS N	Control N	Control Prevalence	At least median PD (95% CI)	Fixed W	Random W	LNS N	Control N	Control Prevalence	Less than median PD (95% CI)	Fixed W	Random W
Bangladesh	JiVitA−4 (21)	BSID−III												
Bangladesh	RDNS (22)	DMC	783	381	7.1	0.01 (−0.03, 0.04)	0.12	0.13	773	372	15.3	−0.04 (−0.08, 0.00)	0.14	0.14
Bangladesh	WASH−B (23)	EASQ	546	1663	7.6	−0.02 (−0.04, 0.00)	0.27	0.19	528	1616	13.6	−0.02 (−0.06, 0.01)	0.17	0.17
Burkina Faso	iLiNS−Zinc (24)	DMC	459	163	16.0	−0.10 (−0.15, −0.04)	0.04	0.05	287	212	14.6	−0.05 (−0.11, 0.01)	0.05	0.05
Ghana	GHANA (25)													
Ghana	iLiNS−DYAD−G (26)	KDI	143	311	10.0	0.00 (−0.06, 0.06)	0.03	0.05	159	288	9.0	0.03 (−0.03, 0.09)	0.05	0.05
Haiti	HAITI (27)													
Kenya	WASH−B (28)	EASQ	778	2706	9.3	−0.02 (−0.04, 0.01)	0.26	0.19	582	2036	11.6	−0.01 (−0.04, 0.01)	0.26	0.26
Madagascar	MAHAY (29)	ASQI	741	845	6.2	−0.01 (−0.04, 0.03)	0.10	0.12	823	724	15.7	−0.03 (−0.09, 0.04)	0.04	0.04
Malawi	iLiNS−DYAD−M (30)	KDI	118	227	8.8	0.00 (−0.07, 0.06)	0.03	0.05	96	208	12.5	−0.03 (−0.11, 0.05)	0.03	0.03
Malawi	iLiNS−DOSE (31)	KDI	278	94	6.4	0.02 (−0.04, 0.09)	0.03	0.04	271	96	12.5	−0.02 (−0.09, 0.05)	0.04	0.04
Mali	PROMIS CS (32)	DMC	479	445	11.0	−0.02 (−0.07, 0.03)	0.05	0.07	423	476	11.1	−0.02 (−0.06, 0.02)	0.11	0.11
Zimbabwe	SHINE (HIV−) (33)	MDAT	406	359	10.6	−0.01 (−0.05, 0.04)	0.05	0.08	389	391	11.3	−0.01 (−0.06, 0.03)	0.10	0.10
Zimbabwe	SHINE (HIV+) (34)	MDAT	91	78	11.5	−0.04 (−0.12, 0.04)	0.02	0.03	74	67	13.4	−0.04 (−0.14, 0.06)	0.02	0.02
			**4822**	**7272**		**I² = 0.20, Tau² = 0.00**			**4405**	**6486**		**I² = 0.00, Tau² = 0.00**		
**Fixed**						**−0.01 (−0.02, 0.00)**						**−0.02 (−0.03, −0.01)**		
**Random**						**−0.01 (−0.03, 0.00)**						**−0.02 (−0.03, −0.01)**		

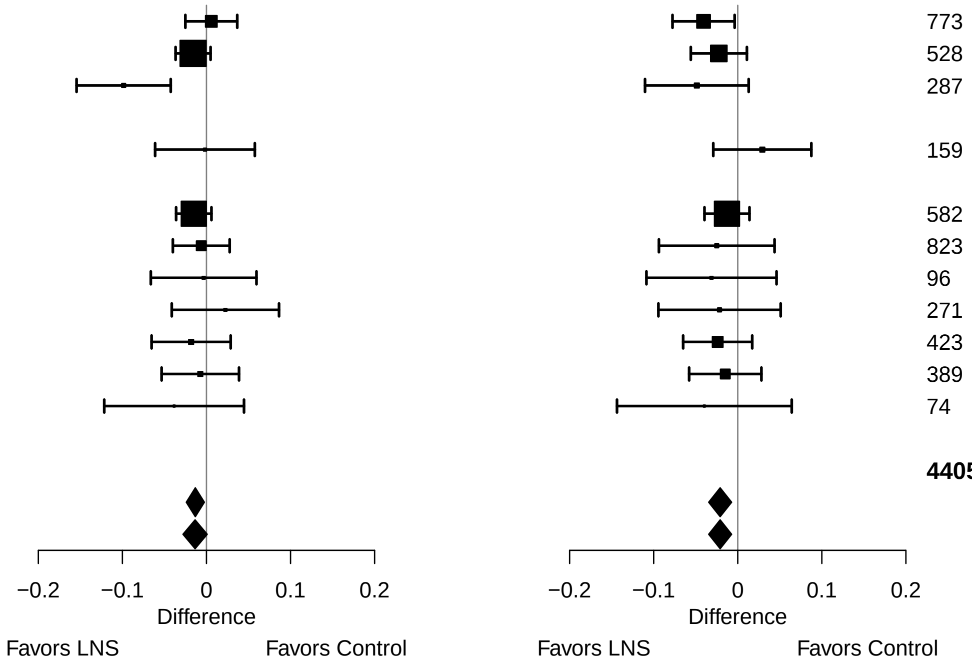

Supplemental figure 8I: Motor lowest decile prevalence difference

8I2: Stratified by Household food insecurity

P−for−interaction = 0.291
Difference in PDs = −0.01 (−0.04, 0.01)

Country	Trial	Tool	LNS N	Control N	Control Prevalence	Mild to secure PD (95% CI)	Fixed W	Random W	LNS N	Control N	Control Prevalence	Moderate to severe PD (95% CI)	Fixed W	Random W
Bangladesh	JiVitA−4 (21)	BSID−III												
Bangladesh	RDNS (22)	DMC	987	456	7.2	0.01 (−0.02, 0.04)	0.14	0.15	569	297	17.2	−0.06 (−0.11, −0.01)	0.19	0.19
Bangladesh	WASH−B (23)	EASQ	853	2550	10.0	−0.02 (−0.04, 0.00)	0.31	0.18	221	729	12.8	−0.01 (−0.06, 0.04)	0.20	0.20
Burkina Faso	iLiNS−Zinc (24)	DMC	389	162	15.4	−0.09 (−0.13, −0.05)	0.07	0.12	357	213	15.0	−0.07 (−0.13, 0.00)	0.12	0.12
Ghana	GHANA (25)													
Ghana	iLiNS−DYAD−G (26)	KDI	217	412	9.2	0.00 (−0.05, 0.05)	0.05	0.10	84	186	10.2	0.05 (−0.03, 0.14)	0.07	0.07
Haiti	HAITI (27)													
Kenya	WASH−B (28)	EASQ	1214	4244	10.0	−0.01 (−0.03, 0.01)	0.29	0.18	146	487	13.6	−0.03 (−0.09, 0.02)	0.14	0.14
Madagascar	MAHAY (29)	ASQI	940	892	9.0	−0.01 (−0.05, 0.04)	0.05	0.11	336	389	15.4	−0.01 (−0.10, 0.08)	0.06	0.06
Malawi	iLiNS−DYAD−M (30)	KDI	67	116	11.2	0.01 (−0.09, 0.10)	0.01	0.04	146	314	10.2	−0.03 (−0.08, 0.03)	0.14	0.14
Malawi	iLiNS−DOSE (31)	KDI												
Mali	PROMIS CS (32)	DMC												
Zimbabwe	SHINE (HIV−) (33)	MDAT	631	586	10.4	0.00 (−0.04, 0.03)	0.08	0.12	140	154	13.0	−0.04 (−0.11, 0.04)	0.08	0.08
Zimbabwe	SHINE (HIV+) (34)	MDAT												
			**5298**	**9418**		**I² = 0.58, Tau² = 0.00**			**1999**	**2769**		**I² = 0.02, Tau² = 0.00**		
**Fixed**						**−0.02 (−0.03, 0.00)**						**−0.03 (−0.05, −0.01)**		
**Random**						**−0.02 (−0.04, 0.01)**						**−0.03 (−0.05, −0.01)**		

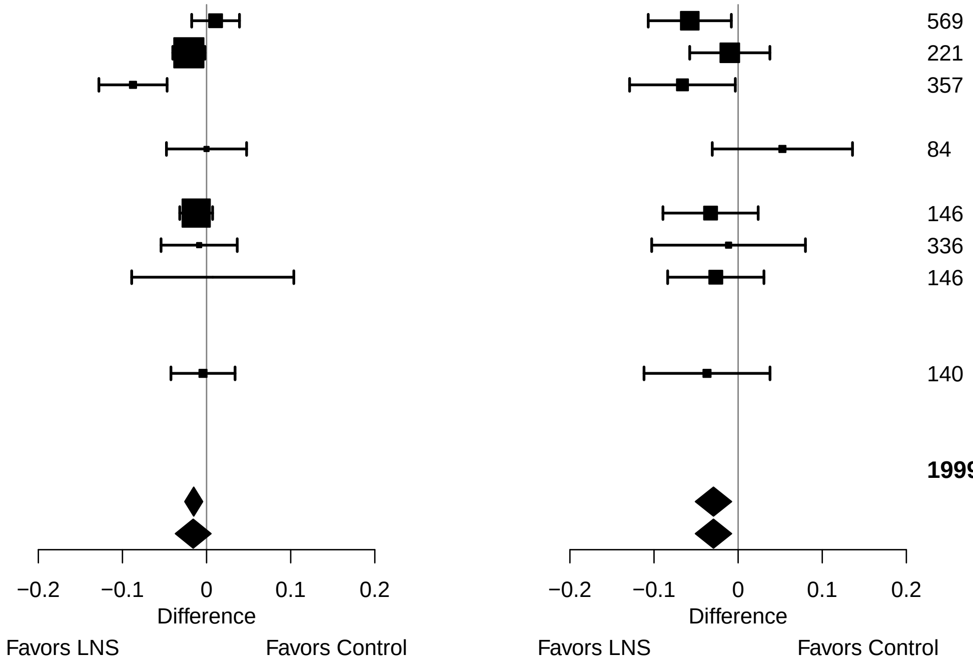

Supplemental figure 8I: Motor lowest decile prevalence difference

8I3: Stratified by Household source water quality

P−for−interaction = 0.795
Difference in PDs = 0.00 (−0.02, 0.03)

Country	Trial	Tool	LNS N	Control N	Control Prevalence	Improved PD (95% CI)	Fixed W	Random W
Bangladesh	JiVitA−4 (21)	BSID−III						
Bangladesh	RDNS (22)	DMC						
Bangladesh	WASH−B (23)	EASQ	460	981	11.6	−0.02 (−0.05, 0.02)	0.29	0.29
Burkina Faso	iLiNS−Zinc (24)	DMC	220	82	12.2	−0.07 (−0.14, 0.00)	0.07	0.07
Ghana	GHANA (25)							
Ghana	iLiNS−DYAD−G (26)	KDI						
Haiti	HAITI (27)							
Kenya	WASH−B (28)	EASQ	413	1419	10.2	0.00 (−0.04, 0.04)	0.24	0.24
Madagascar	MAHAY (29)	ASQI	405	430	14.0	−0.02 (−0.10, 0.07)	0.05	0.05
Malawi	iLiNS−DYAD−M (30)	KDI						
Malawi	iLiNS−DOSE (31)	KDI						
Mali	PROMIS CS (32)	DMC	514	539	10.2	−0.03 (−0.07, 0.01)	0.22	0.22
Zimbabwe	SHINE (HIV−) (33)	MDAT	228	220	9.5	−0.01 (−0.06, 0.04)	0.13	0.13
Zimbabwe	SHINE (HIV+) (34)	MDAT						
			**2240**	**3671**		**I² = 0.00, Tau² = 0.00**		
**Fixed**						**−0.02 (−0.04, 0.00)**		
**Random**						**−0.02 (−0.04, 0.00)**		

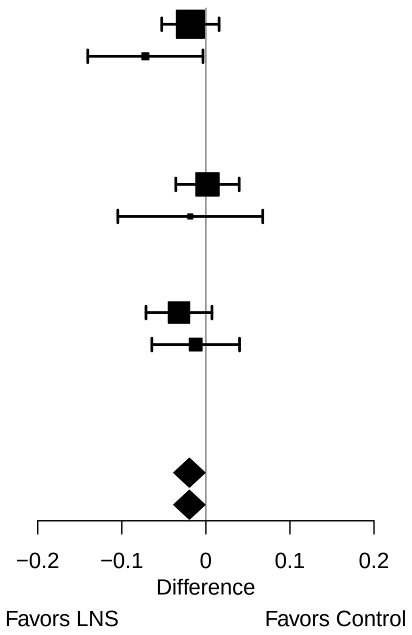

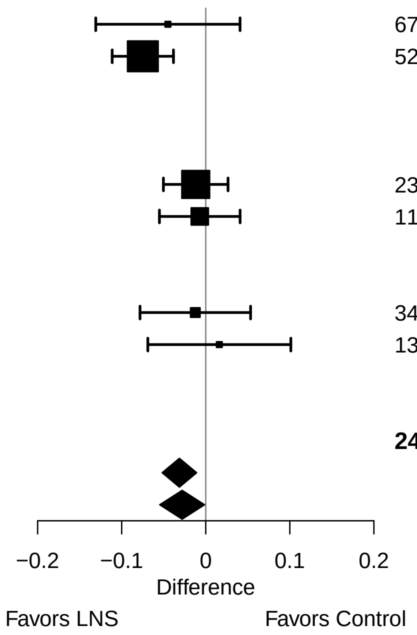

Country	Trial	LNS N	Control N	Control Prevalence	Unimproved PD (95% CI)	Fixed W	Random W
Bangladesh	JiVitA−4 (21)						
Bangladesh	RDNS (22)						
Bangladesh	WASH−B (23)	67	117	12.0	−0.05 (−0.13, 0.04)	0.06	0.08
Burkina Faso	iLiNS−Zinc (24)	526	293	16.0	−0.07 (−0.11, −0.04)	0.32	0.26
Ghana	GHANA (25)						
Ghana	iLiNS−DYAD−G (26)						
Haiti	HAITI (27)						
Kenya	WASH−B (28)	236	649	9.2	−0.01 (−0.05, 0.03)	0.29	0.25
Madagascar	MAHAY (29)	1134	1133	9.4	−0.01 (−0.06, 0.04)	0.18	0.19
Malawi	iLiNS−DYAD−M (30)						
Malawi	iLiNS−DOSE (31)						
Mali	PROMIS CS (32)	347	368	12.8	−0.01 (−0.08, 0.05)	0.10	0.13
Zimbabwe	SHINE (HIV−) (33)	131	122	9.8	0.02 (−0.07, 0.10)	0.06	0.08
Zimbabwe	SHINE (HIV+) (34)						
		**2441**	**2682**		**I² = 0.45, Tau² = 0.00**		
**Fixed**					**−0.03 (−0.05, −0.01)**		
**Random**					**−0.03 (−0.05, 0.00)**		

Supplemental figure 8I: Motor lowest decile prevalence difference

8I4: Stratified by Household sanitation

P−for−interaction = 0.308
Difference in PDs = 0.02 (−0.02, 0.05)

Country	Trial	Tool	Improved LNS N	Improved Control N	Improved Control Prevalence	Improved PD (95% CI)	Improved Fixed W	Improved Random W	Unimproved LNS N	Unimproved Control N	Unimproved Control Prevalence	Unimproved PD (95% CI)	Unimproved Fixed W	Unimproved Random W
Bangladesh	JiVitA−4 (21)	BSID−III												
Bangladesh	RDNS (22)	DMC	1103	531	10.4	−0.02 (−0.05, 0.01)	0.50	0.50	451	222	13.1	−0.01 (−0.07, 0.05)	0.15	0.15
Bangladesh	WASH−B (23)	EASQ												
Burkina Faso	iLiNS−Zinc (24)	DMC												
Ghana	GHANA (25)													
Ghana	iLiNS−DYAD−G (26)	KDI												
Haiti	HAITI (27)													
Kenya	WASH−B (28)	EASQ	94	337	9.2	−0.02 (−0.08, 0.05)	0.11	0.11	555	1732	10.0	0.00 (−0.03, 0.03)	0.55	0.55
Madagascar	MAHAY (29)	ASQI												
Malawi	iLiNS−DYAD−M (30)	KDI												
Malawi	iLiNS−DOSE (31)	KDI												
Mali	PROMIS CS (32)	DMC	665	658	10.8	−0.02 (−0.06, 0.02)	0.29	0.29	210	241	10.8	−0.01 (−0.06, 0.05)	0.16	0.16
Zimbabwe	SHINE (HIV−) (33)	MDAT	133	108	10.2	−0.03 (−0.10, 0.03)	0.10	0.10	225	235	9.4	0.02 (−0.04, 0.08)	0.14	0.14
Zimbabwe	SHINE (HIV+) (34)	MDAT												
			**1995**	**1634**		**I² = 0.00, Tau² = 0.00**			**1441**	**2430**		**I² = 0.00, Tau² = 0.00**		
**Fixed**						**−0.02 (−0.04, 0.00)**						**0.00 (−0.02, 0.02)**		
**Random**						**−0.02 (−0.04, 0.00)**						**0.00 (−0.02, 0.02)**		

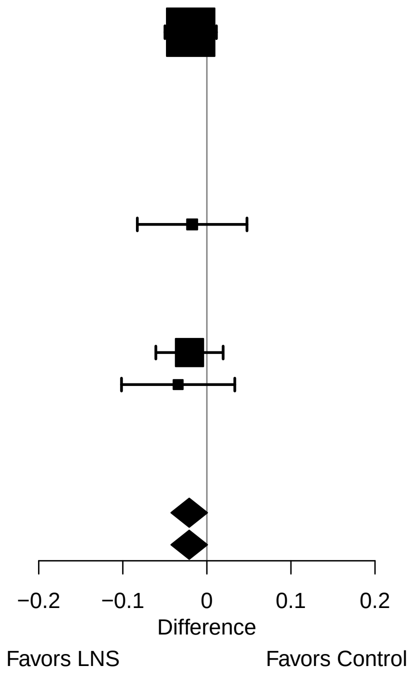

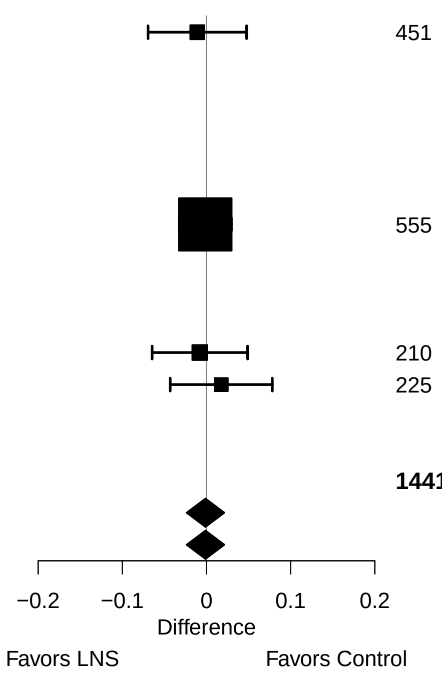

Supplemental figure 8I: Motor lowest decile prevalence difference

8I5: Stratified by Home environment

P−for−interaction = 0.562
Difference in PDs = −0.01 (−0.03, 0.01)

Country	Trial	Tool	At least median: LNS N	Control N	Control Prevalence	PD (95% CI)	Fixed W	Random W	Less than median: LNS N	Control N	Control Prevalence	PD (95% CI)	Fixed W	Random W
Bangladesh	JiVitA−4 (21)	BSID−III												
Bangladesh	RDNS (22)	DMC	1115	548	8.0	−0.02 (−0.04, 0.00)	0.21	0.21	439	203	19.2	−0.01 (−0.08, 0.05)	0.09	0.11
Bangladesh	WASH−B (23)	EASQ	605	1614	7.4	−0.02 (−0.05, 0.00)	0.23	0.23	469	1664	13.6	0.00 (−0.04, 0.03)	0.27	0.24
Burkina Faso	iLiNS−Zinc (24)	DMC	473	199	6.5	−0.03 (−0.06, 0.00)	0.14	0.14	272	175	25.1	−0.11 (−0.20, −0.03)	0.05	0.07
Ghana	GHANA (25)													
Ghana	iLiNS−DYAD−G (26)	KDI	191	377	6.6	0.01 (−0.03, 0.06)	0.06	0.06	111	224	14.7	0.01 (−0.07, 0.10)	0.05	0.07
Haiti	HAITI (27)													
Kenya	WASH−B (28)	EASQ	809	2682	7.2	0.00 (−0.02, 0.03)	0.24	0.24	552	2060	14.4	−0.03 (−0.07, 0.00)	0.33	0.27
Madagascar	MAHAY (29)	ASQI	843	839	9.3	−0.02 (−0.07, 0.03)	0.05	0.05	770	764	11.8	0.00 (−0.05, 0.06)	0.11	0.13
Malawi	iLiNS−DYAD−M (30)	KDI	136	287	7.7	0.00 (−0.06, 0.05)	0.04	0.04	78	149	16.1	−0.05 (−0.14, 0.05)	0.04	0.05
Malawi	iLiNS−DOSE (31)	KDI	376	135	8.1	0.01 (−0.05, 0.06)	0.04	0.04	269	86	11.6	0.01 (−0.07, 0.09)	0.06	0.07
Mali	PROMIS CS (32)	DMC												
Zimbabwe	SHINE (HIV−) (33)	MDAT												
Zimbabwe	SHINE (HIV+) (34)	MDAT												
			**4548**	**6681**		**I² = 0.00, Tau² = 0.00**			**2960**	**5325**		**I² = 0.17, Tau² = 0.00**		
**Fixed**						**−0.01 (−0.02, 0.00)**						**−0.02 (−0.04, 0.00)**		
**Random**						**−0.01 (−0.02, 0.00)**						**−0.02 (−0.04, 0.00)**		

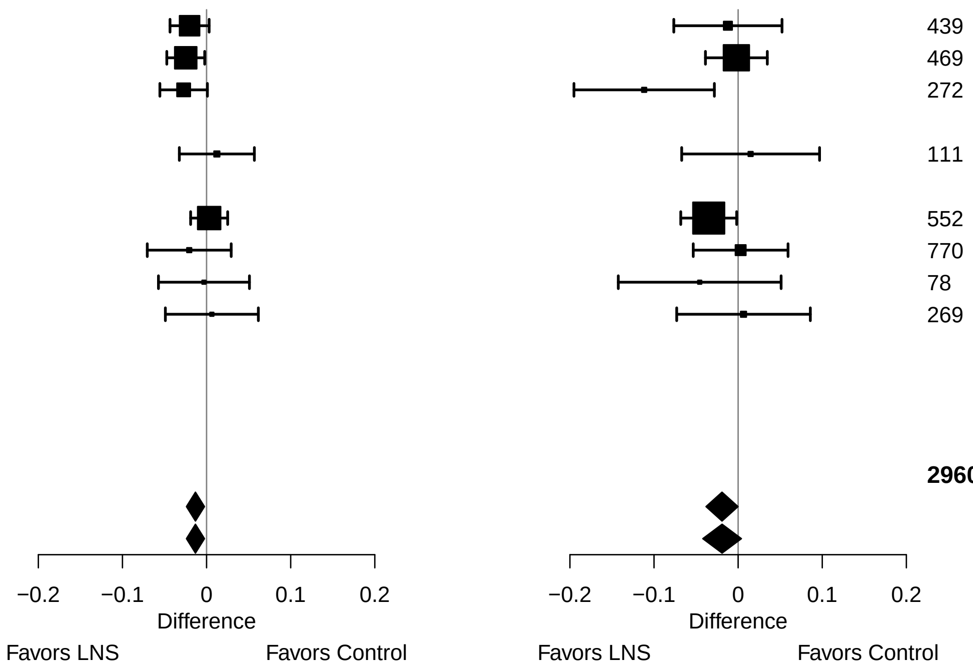

Supplemental figure 8I: Motor lowest decile prevalence difference

8I6: Stratified by Season at the time of assessment

P−for−interaction = 0.586
Difference in PDs = 0.01 (−0.02, 0.03)

Country	Trial	Tool	Dry LNS N	Dry Control N	Dry Control Prevalence	Dry PD (95% CI)	Dry Fixed W	Dry Random W	Rainy LNS N	Rainy Control N	Rainy Control Prevalence	Rainy PD (95% CI)	Rainy Fixed W	Rainy Random W
Bangladesh	JiVitA−4 (21)	BSID−III												
Bangladesh	RDNS (22)	DMC	698	326	11.7	−0.01 (−0.05, 0.02)	0.14	0.15	858	427	10.8	−0.02 (−0.05, 0.02)	0.16	0.16
Bangladesh	WASH−B (23)	EASQ	608	1896	10.0	−0.01 (−0.03, 0.02)	0.33	0.20	466	1383	11.4	−0.03 (−0.06, 0.00)	0.21	0.21
Burkina Faso	iLiNS−Zinc (24)	DMC	514	274	16.8	−0.08 (−0.13, −0.03)	0.09	0.11	232	101	10.9	−0.06 (−0.12, −0.01)	0.06	0.06
Ghana	GHANA (25)													
Ghana	iLiNS−DYAD−G (26)	KDI	177	356	9.0	0.02 (−0.04, 0.07)	0.07	0.10	125	245	10.6	0.01 (−0.06, 0.07)	0.04	0.04
Haiti	HAITI (27)													
Kenya	WASH−B (28)	EASQ	315	1059	17.4	−0.05 (−0.09, −0.01)	0.11	0.13	1046	3682	8.3	−0.01 (−0.03, 0.02)	0.40	0.40
Madagascar	MAHAY (29)	ASQI												
Malawi	iLiNS−DYAD−M (30)	KDI	153	308	10.1	−0.02 (−0.07, 0.04)	0.06	0.09	61	127	11.8	−0.02 (−0.12, 0.08)	0.02	0.02
Malawi	iLiNS−DOSE (31)	KDI	395	118	10.2	0.01 (−0.06, 0.07)	0.05	0.08	244	100	9.0	0.00 (−0.07, 0.07)	0.04	0.04
Mali	PROMIS CS (32)	DMC												
Zimbabwe	SHINE (HIV−) (33)	MDAT	603	568	10.9	−0.02 (−0.05, 0.02)	0.15	0.15	238	231	9.1	0.02 (−0.04, 0.07)	0.07	0.07
Zimbabwe	SHINE (HIV+) (34)	MDAT												
			**3463**	**4905**		**I² = 0.40, Tau² = 0.00**			**3270**	**6296**		**I² = 0.00, Tau² = 0.00**		
**Fixed**						**−0.02 (−0.03, −0.01)**						**−0.01 (−0.03, 0.00)**		
**Random**						**−0.02 (−0.04, 0.00)**						**−0.01 (−0.03, 0.00)**		

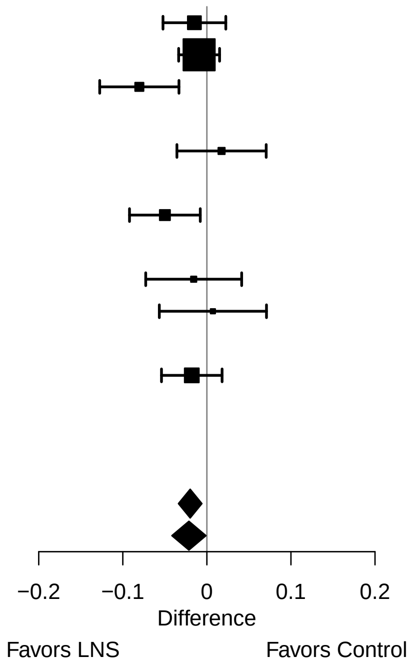

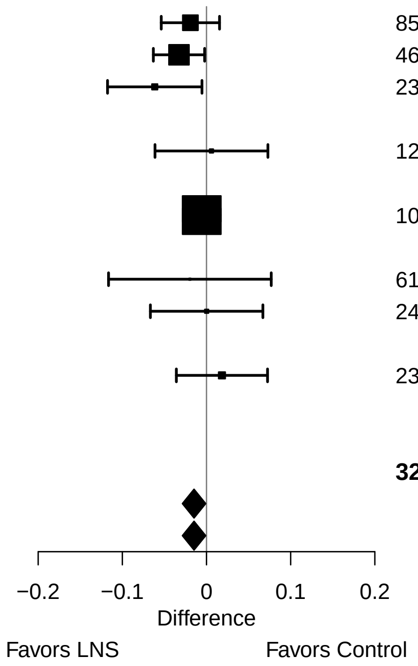

Supplemental figure 8J: Mean difference in gross motor z-score

8J1: Stratified by Household socio-economic status

P−for−interaction = 0.091
Difference in MDs = 0.05 (−0.01, 0.10)

Country	Trial	Tool	At least median: LNS N	Control N	Control Mean	MD (95% CI)	Fixed W	Random W	Less than median: LNS N	Control N	Control Mean	MD (95% CI)	Fixed W	Random W
Bangladesh	JiVitA−4 (21)	BSID−III	236	77	0.26	−0.15 (−0.35, 0.05)	0.04	0.06	208	66	−0.12	−0.04 (−0.27, 0.19)	0.03	0.03
Bangladesh	RDNS (22)	DMC	788	382	0.02	0.09 (0.00, 0.18)	0.18	0.15	782	376	−0.09	0.08 (−0.01, 0.18)	0.19	0.19
Bangladesh	WASH−B (23)	EASQ	546	1663	0.12	0.05 (−0.04, 0.14)	0.18	0.15	528	1616	−0.17	0.16 (0.07, 0.26)	0.20	0.20
Burkina Faso	iLiNS−Zinc (24)	DMC												
Ghana	GHANA (25)													
Ghana	iLiNS−DYAD−G (26)	KDI	143	311	0.02	0.09 (−0.12, 0.29)	0.03	0.06	159	288	−0.04	−0.05 (−0.24, 0.14)	0.05	0.05
Haiti	HAITI (27)													
Kenya	WASH−B (28)	EASQ	778	2706	0.07	−0.03 (−0.10, 0.04)	0.29	0.16	582	2036	−0.10	0.07 (−0.04, 0.17)	0.16	0.16
Madagascar	MAHAY (29)	ASQI	741	845	0.08	0.07 (−0.09, 0.23)	0.06	0.08	823	724	−0.18	0.11 (−0.08, 0.29)	0.05	0.05
Malawi	iLiNS−DYAD−M (30)	KDI	118	227	0.02	−0.05 (−0.25, 0.14)	0.04	0.07	96	208	0.01	−0.01 (−0.27, 0.26)	0.02	0.02
Malawi	iLiNS−DOSE (31)	KDI	278	94	0.16	−0.05 (−0.27, 0.17)	0.03	0.05	271	96	−0.15	0.10 (−0.13, 0.32)	0.03	0.03
Mali	PROMIS CS (32)	DMC	499	457	−0.03	0.10 (−0.04, 0.23)	0.08	0.10	441	500	−0.06	0.07 (−0.04, 0.17)	0.16	0.16
Zimbabwe	SHINE (HIV−) (33)	MDAT	406	359	−0.01	0.12 (−0.03, 0.27)	0.06	0.09	389	391	−0.11	0.11 (−0.04, 0.26)	0.08	0.08
Zimbabwe	SHINE (HIV+) (34)	MDAT	91	78	−0.17	0.38 (0.07, 0.70)	0.01	0.03	74	67	−0.16	0.25 (−0.05, 0.55)	0.02	0.02
			**4624**	**7199**		**I² = 0.41, Tau² = 0.00**			**4353**	**6368**		**I² = 0.00, Tau² = 0.00**		
**Fixed**						**0.03 (0.00, 0.07)**						**0.09 (0.05, 0.13)**		
**Random**						**0.04 (−0.02, 0.10)**						**0.09 (0.05, 0.13)**		

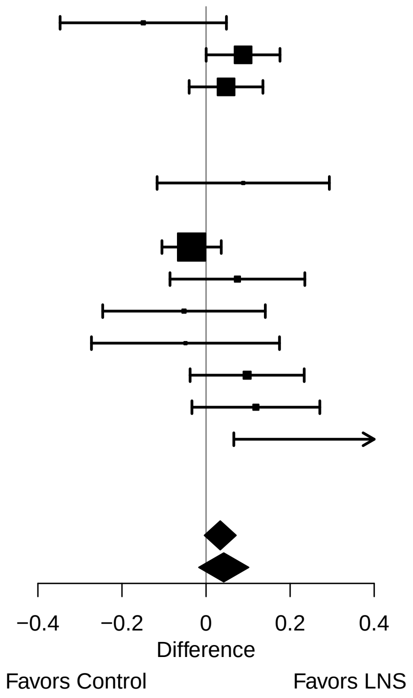

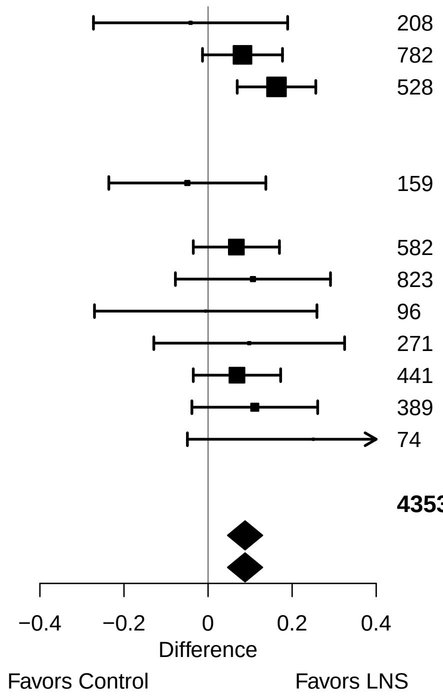

Supplemental figure 8J: Mean difference in gross motor z-score

8J2: Stratified by Household food insecurity

P−for−interaction = 0.951
Difference in MDs = 0.00 (−0.07, 0.08)

			Mild to secure						Moderate to severe					
**Country**	**Trial**	**Tool**	**LNS N**	**Control N**	**Control Mean**	**MD (95% CI)**	**Fixed W**	**Random W**	**LNS N**	**Control N**	**Control Mean**	**MD (95% CI)**	**Fixed W**	**Random W**
Bangladesh	JiVitA−4 (21)	BSID−III	329	111	0.12	−0.12 (−0.29, 0.05)	0.04	0.09	115	32	−0.06	−0.02 (−0.37, 0.33)	0.03	0.03
Bangladesh	RDNS (22)	DMC	994	459	0.04	0.05 (−0.04, 0.14)	0.16	0.14	576	299	−0.14	0.14 (0.03, 0.24)	0.33	0.33
Bangladesh	WASH−B (23)	EASQ	853	2550	0.01	0.11 (0.05, 0.18)	0.28	0.15	221	729	−0.14	0.05 (−0.11, 0.21)	0.14	0.14
Burkina Faso	iLiNS−Zinc (24)	DMC												
Ghana	GHANA (25)													
Ghana	iLiNS−DYAD−G (26)	KDI	217	412	−0.01	0.04 (−0.12, 0.21)	0.05	0.09	84	186	0.00	−0.06 (−0.31, 0.19)	0.05	0.05
Haiti	HAITI (27)													
Kenya	WASH−B (28)	EASQ	1214	4244	0.01	0.01 (−0.06, 0.08)	0.29	0.15	146	487	−0.11	0.01 (−0.19, 0.21)	0.09	0.09
Madagascar	MAHAY (29)	ASQI	940	892	0.04	0.04 (−0.12, 0.21)	0.05	0.09	336	389	−0.19	0.08 (−0.15, 0.32)	0.06	0.06
Malawi	iLiNS−DYAD−M (30)	KDI	67	116	0.05	−0.13 (−0.39, 0.14)	0.02	0.06	146	314	0.01	0.00 (−0.20, 0.20)	0.09	0.09
Malawi	iLiNS−DOSE (31)	KDI	134	51	0.18	−0.08 (−0.36, 0.20)	0.02	0.05	403	133	−0.08	0.08 (−0.12, 0.28)	0.09	0.09
Mali	PROMIS CS (32)	DMC												
Zimbabwe	SHINE (HIV−) (33)	MDAT	631	586	−0.05	0.12 (−0.01, 0.25)	0.08	0.11	140	154	−0.09	0.05 (−0.14, 0.24)	0.10	0.10
Zimbabwe	SHINE (HIV+) (34)	MDAT	119	105	−0.18	0.39 (0.15, 0.63)	0.02	0.07	41	37	−0.12	0.13 (−0.21, 0.47)	0.03	0.03
			**5498**	**9526**		**I^2 = 0.55, Tau^2 = 0.01**			**2208**	**2760**		**I^2 = 0.00, Tau^2 = 0.00**		
**Fixed**						**0.06 (0.02, 0.09)**						**0.07 (0.01, 0.13)**		
**Random**						**0.05 (−0.03, 0.13)**						**0.07 (0.01, 0.13)**		

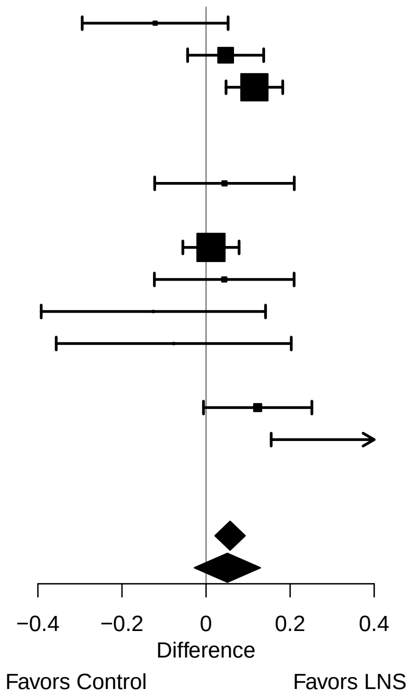

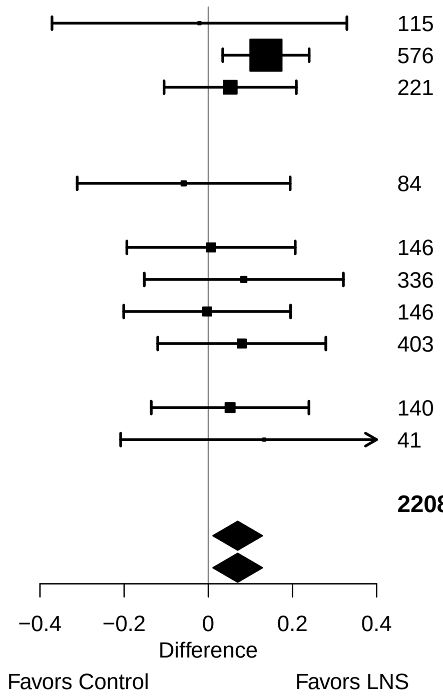

Supplemental figure 8J: Mean difference in gross motor z-score

8J3: Stratified by Household source water quality

P−for−interaction = 0.883
Difference in MDs = −0.01 (−0.12, 0.10)

Country	Trial	Tool	Improved LNS N	Improved Control N	Improved Control Mean	Improved MD (95% CI)	Improved Fixed W	Improved Random W	Unimproved LNS N	Unimproved Control N	Unimproved Control Mean	Unimproved MD (95% CI)	Unimproved Fixed W	Unimproved Random W
Bangladesh	JiVitA−4 (21)	BSID−III												
Bangladesh	RDNS (22)	DMC												
Bangladesh	WASH−B (23)	EASQ	460	981	−0.09	0.20 (0.08, 0.31)	0.21	0.19	67	117	−0.12	0.20 (−0.08, 0.48)	0.09	0.09
Burkina Faso	iLiNS−Zinc (24)	DMC												
Ghana	GHANA (25)													
Ghana	iLiNS−DYAD−G (26)	KDI												
Haiti	HAITI (27)													
Kenya	WASH−B (28)	EASQ	413	1419	−0.01	0.02 (−0.10, 0.14)	0.21	0.19	236	649	0.05	0.04 (−0.12, 0.21)	0.24	0.24
Madagascar	MAHAY (29)	ASQI	405	430	−0.04	0.10 (−0.14, 0.35)	0.05	0.06	1134	1133	−0.04	0.07 (−0.10, 0.24)	0.23	0.23
Malawi	iLiNS−DYAD−M (30)	KDI	194	398	0.03	−0.07 (−0.23, 0.10)	0.10	0.12	20	36	−0.08	0.23 (−0.31, 0.77)	0.02	0.02
Malawi	iLiNS−DOSE (31)	KDI	515	177	0.01	0.03 (−0.14, 0.20)	0.11	0.12	44	15	−0.08	0.08 (−0.53, 0.69)	0.02	0.02
Mali	PROMIS CS (32)	DMC	539	561	−0.05	0.15 (0.04, 0.27)	0.22	0.20	359	382	−0.04	0.02 (−0.14, 0.18)	0.27	0.27
Zimbabwe	SHINE (HIV−) (33)	MDAT	228	220	0.09	0.01 (−0.19, 0.20)	0.08	0.09	131	122	−0.10	0.09 (−0.18, 0.36)	0.10	0.10
Zimbabwe	SHINE (HIV+) (34)	MDAT	37	41	−0.02	0.08 (−0.28, 0.44)	0.02	0.03	25	25	−0.25	0.12 (−0.31, 0.55)	0.04	0.04
			**2791**	**4227**		**I² = 0.31, Tau² = 0.00**			**2016**	**2479**		**I² = 0.00, Tau² = 0.00**		
**Fixed**						**0.08 (0.03, 0.14)**						**0.07 (−0.01, 0.15)**		
**Random**						**0.08 (0.01, 0.14)**						**0.07 (−0.01, 0.15)**		

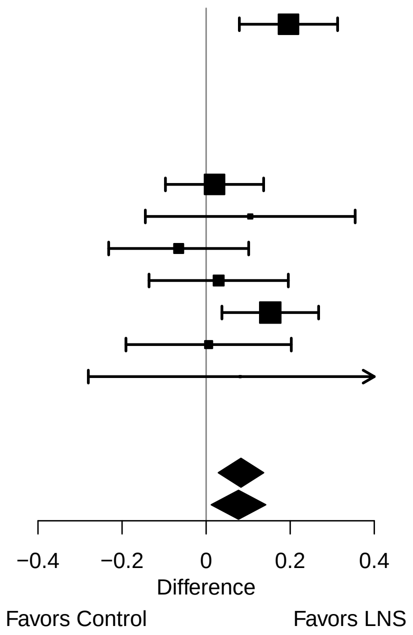

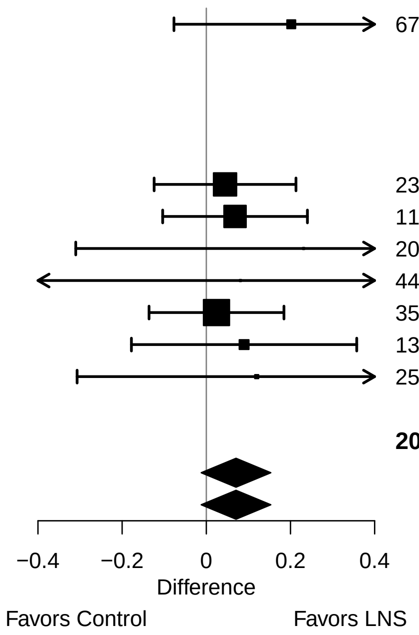

Supplemental figure 8J: Mean difference in gross motor z-score

8J4: Stratified by Household sanitation

P−for−interaction = 0.943
Difference in MDs = 0.00 (−0.10, 0.09)

Country	Trial	Tool	Improved LNS N	Improved Control N	Improved Control Mean	Improved MD (95% CI)	Improved Fixed W	Improved Random W	Unimproved LNS N	Unimproved Control N	Unimproved Control Mean	Unimproved MD (95% CI)	Unimproved Fixed W	Unimproved Random W
Bangladesh	JiVitA−4 (21)	BSID−III	353	112	0.14	−0.11 (−0.27, 0.06)	0.08	0.10	91	31	−0.12	−0.10 (−0.61, 0.40)	0.01	0.01
Bangladesh	RDNS (22)	DMC	1116	532	−0.02	0.10 (0.02, 0.18)	0.34	0.26	452	226	−0.07	0.04 (−0.07, 0.15)	0.24	0.24
Bangladesh	WASH−B (23)	EASQ	472	998	−0.09	0.21 (0.10, 0.32)	0.17	0.17	29	53	−0.20	0.18 (−0.39, 0.74)	0.01	0.01
Burkina Faso	iLiNS−Zinc (24)	DMC												
Ghana	GHANA (25)													
Ghana	iLiNS−DYAD−G (26)	KDI	292	586	0.00	0.00 (−0.14, 0.14)	0.10	0.12	9	13	−0.13	0.54 (−0.21, 1.28)	0.01	0.01
Haiti	HAITI (27)													
Kenya	WASH−B (28)	EASQ	94	337	0.12	0.09 (−0.13, 0.30)	0.04	0.06	555	1732	−0.01	0.02 (−0.08, 0.13)	0.29	0.29
Madagascar	MAHAY (29)	ASQI												
Malawi	iLiNS−DYAD−M (30)	KDI	19	41	−0.09	0.12 (−0.29, 0.53)	0.01	0.02	195	393	0.03	−0.06 (−0.23, 0.11)	0.10	0.10
Malawi	iLiNS−DOSE (31)	KDI	12	9	0.43	−0.01 (−0.57, 0.56)	0.01	0.01	547	183	−0.02	0.05 (−0.12, 0.21)	0.11	0.11
Mali	PROMIS CS (32)	DMC	695	688	−0.04	0.05 (−0.05, 0.16)	0.20	0.19	216	244	−0.04	0.18 (0.02, 0.34)	0.12	0.12
Zimbabwe	SHINE (HIV−) (33)	MDAT	133	108	0.09	0.05 (−0.21, 0.30)	0.03	0.05	225	235	−0.01	0.02 (−0.17, 0.21)	0.08	0.08
Zimbabwe	SHINE (HIV+) (34)	MDAT	25	17	0.02	−0.04 (−0.49, 0.41)	0.01	0.02	37	49	−0.15	0.14 (−0.20, 0.48)	0.03	0.03
			**3211**	**3428**		**I^2 = 0.28, Tau^2 = 0.00**			**2356**	**3159**		**I^2 = 0.00, Tau^2 = 0.00**		
**Fixed**						**0.08 (0.03, 0.12)**						**0.05 (−0.01, 0.10)**		
**Random**						**0.07 (0.01, 0.13)**						**0.05 (−0.01, 0.10)**		

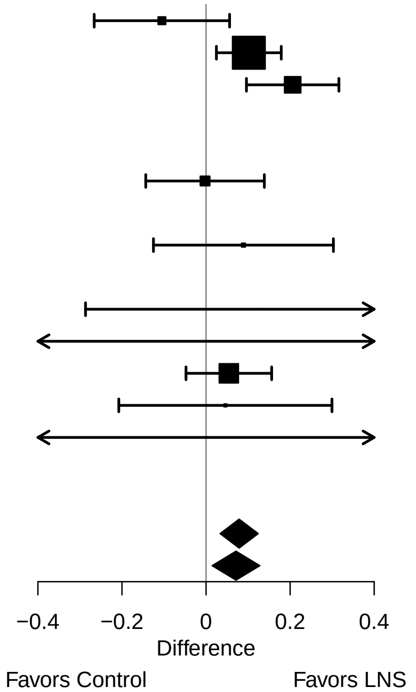

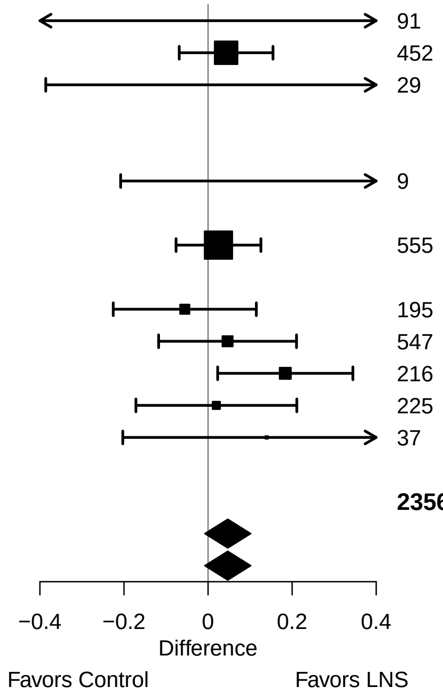

Supplemental figure 8J: Mean difference in gross motor z-score

8J5: Stratified by Home environment

P−for−interaction = 0.300
Difference in MDs = −0.04 (−0.10, 0.03)

			At least median						Less than median					
**Country**	**Trial**	**Tool**	**LNS N**	**Control N**	**Control Mean**	**MD (95% CI)**	**Fixed W**	**Random W**	**LNS N**	**Control N**	**Control Mean**	**MD (95% CI)**	**Fixed W**	**Random W**
Bangladesh	JiVitA−4 (21)	BSID−III												
Bangladesh	RDNS (22)	DMC	1126	551	0.06	0.07 (0.01, 0.12)	0.47	0.47	442	205	−0.30	0.14 (−0.02, 0.31)	0.11	0.11
Bangladesh	WASH−B (23)	EASQ	605	1614	0.13	0.12 (0.02, 0.21)	0.15	0.15	469	1664	−0.17	0.04 (−0.06, 0.14)	0.32	0.32
Burkina Faso	iLiNS−Zinc (24)	DMC												
Ghana	GHANA (25)													
Ghana	iLiNS−DYAD−G (26)	KDI	191	377	0.07	0.06 (−0.11, 0.22)	0.05	0.05	111	224	−0.12	−0.07 (−0.31, 0.17)	0.05	0.05
Haiti	HAITI (27)													
Kenya	WASH−B (28)	EASQ	809	2682	0.15	0.01 (−0.07, 0.09)	0.22	0.22	552	2060	−0.20	−0.01 (−0.11, 0.09)	0.31	0.31
Madagascar	MAHAY (29)	ASQI	843	839	0.03	0.11 (−0.07, 0.30)	0.04	0.04	770	764	−0.11	0.03 (−0.12, 0.19)	0.12	0.12
Malawi	iLiNS−DYAD−M (30)	KDI	136	287	0.14	−0.03 (−0.21, 0.15)	0.04	0.04	78	149	−0.23	−0.01 (−0.32, 0.30)	0.03	0.03
Malawi	iLiNS−DOSE (31)	KDI	376	135	0.04	0.03 (−0.16, 0.22)	0.04	0.04	269	86	−0.07	−0.03 (−0.27, 0.22)	0.05	0.05
Mali	PROMIS CS (32)	DMC												
Zimbabwe	SHINE (HIV−) (33)	MDAT												
Zimbabwe	SHINE (HIV+) (34)	MDAT												
			**4086**	**6485**		**I² = 0.00, Tau² = 0.00**			**2691**	**5152**		**I² = 0.00, Tau² = 0.00**		
**Fixed**						**0.06 (0.02, 0.09)**						**0.02 (−0.03, 0.08)**		
**Random**						**0.06 (0.02, 0.09)**						**0.02 (−0.03, 0.08)**		

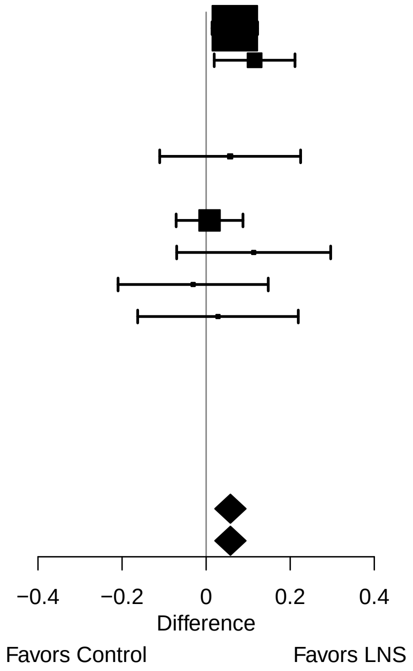

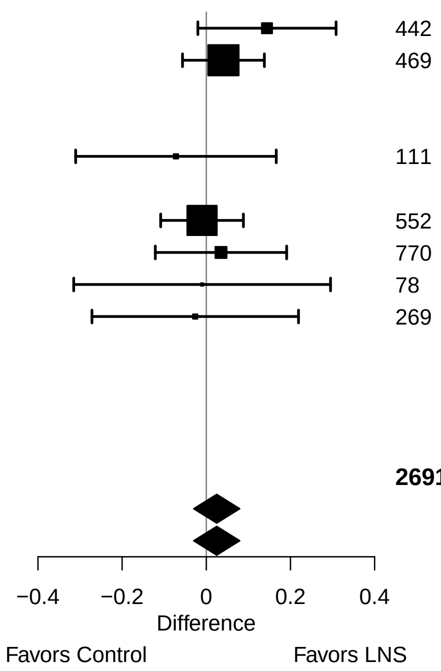

Supplemental figure 8J: Mean difference in gross motor z-score

8J6: Stratified by Season at the time of assessment

P−for−interaction = 0.958
Difference in MDs = 0.00 (−0.07, 0.06)

Country	Trial	Tool	Dry LNS N	Dry Control N	Dry Control Mean	Dry MD (95% CI)	Dry Fixed W	Dry Random W	Rainy LNS N	Rainy Control N	Rainy Control Mean	Rainy MD (95% CI)	Rainy Fixed W	Rainy Random W
Bangladesh	JiVitA−4 (21)	BSID−III	251	70	0.32	−0.10 (−0.30, 0.11)	0.04	0.07	194	73	−0.14	−0.18 (−0.43, 0.06)	0.03	0.07
Bangladesh	RDNS (22)	DMC	703	330	−0.03	0.08 (−0.01, 0.17)	0.21	0.16	867	428	−0.04	0.09 (0.00, 0.17)	0.28	0.21
Bangladesh	WASH−B (23)	EASQ	608	1896	0.04	0.08 (−0.01, 0.17)	0.24	0.17	466	1383	−0.11	0.14 (0.04, 0.24)	0.20	0.19
Burkina Faso	iLiNS−Zinc (24)	DMC												
Ghana	GHANA (25)													
Ghana	iLiNS−DYAD−G (26)	KDI	177	356	−0.05	−0.03 (−0.20, 0.15)	0.06	0.09	125	245	0.07	0.06 (−0.16, 0.29)	0.04	0.08
Haiti	HAITI (27)													
Kenya	WASH−B (28)	EASQ	315	1059	−0.30	0.09 (−0.01, 0.20)	0.17	0.15	1046	3682	0.08	−0.01 (−0.09, 0.07)	0.33	0.22
Madagascar	MAHAY (29)	ASQI												
Malawi	iLiNS−DYAD−M (30)	KDI	153	308	0.04	−0.02 (−0.21, 0.18)	0.05	0.08	61	127	−0.05	−0.06 (−0.34, 0.21)	0.03	0.06
Malawi	iLiNS−DOSE (31)	KDI	395	118	−0.02	−0.07 (−0.27, 0.13)	0.05	0.07	244	100	0.02	0.12 (−0.11, 0.36)	0.04	0.07
Mali	PROMIS CS (32)	DMC												
Zimbabwe	SHINE (HIV−) (33)	MDAT	603	568	−0.07	0.12 (0.00, 0.23)	0.13	0.14	238	231	0.01	0.05 (−0.17, 0.28)	0.04	0.08
Zimbabwe	SHINE (HIV+) (34)	MDAT	121	110	−0.17	0.31 (0.10, 0.52)	0.04	0.07	49	41	−0.21	0.43 (0.01, 0.85)	0.01	0.03
			**3326**	**4815**		**I^2 = 0.34, Tau^2 = 0.00**			**3290**	**6310**		**I^2 = 0.42, Tau^2 = 0.01**		
**Fixed**						**0.07 (0.03, 0.11)**						**0.06 (0.01, 0.10)**		
**Random**						**0.06 (0.00, 0.13)**						**0.06 (−0.02, 0.13)**		

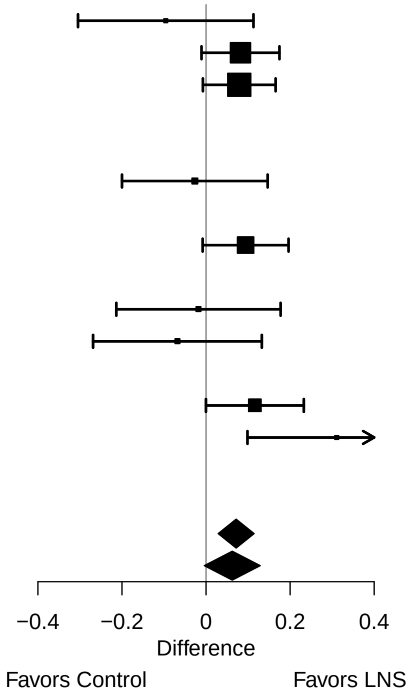

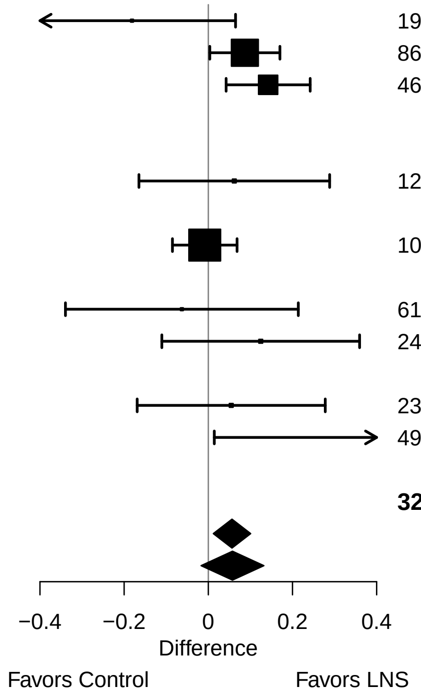

Supplemental figure 8K: Mean difference in fine motor z-score

8K1: Stratified by Household socio-economic status

P−for−interaction = 0.967
Difference in MDs = 0.00 (−0.08, 0.08)

			At least median						Less than median					
**Country**	**Trial**	**Tool**	**LNS N**	**Control N**	**Control Mean**	**MD (95% CI)**	**Fixed W**	**Random W**	**LNS N**	**Control N**	**Control Mean**	**MD (95% CI)**	**Fixed W**	**Random W**
Bangladesh	JiVitA−4 (21)	BSID−III	236	77	0.03	0.07 (−0.18, 0.32)	0.06	0.06	208	66	−0.27	0.23 (−0.03, 0.50)	0.06	0.06
Bangladesh	RDNS (22)	DMC	827	403	0.05	0.08 (−0.06, 0.22)	0.20	0.20	814	401	−0.16	0.12 (0.01, 0.23)	0.32	0.32
Bangladesh	WASH−B (23)	EASQ												
Burkina Faso	iLiNS−Zinc (24)	DMC												
Ghana	GHANA (25)													
Ghana	iLiNS−DYAD−G (26)	KDI	143	311	0.00	0.06 (−0.11, 0.24)	0.13	0.13	159	288	0.01	0.00 (−0.18, 0.19)	0.12	0.12
Haiti	HAITI (27)													
Kenya	WASH−B (28)	EASQ												
Madagascar	MAHAY (29)	ASQI	741	845	0.16	0.04 (−0.14, 0.21)	0.13	0.13	823	724	−0.25	0.11 (−0.17, 0.39)	0.05	0.05
Malawi	iLiNS−DYAD−M (30)	KDI	118	227	−0.06	0.11 (−0.10, 0.31)	0.09	0.09	96	208	0.02	0.05 (−0.19, 0.29)	0.07	0.07
Malawi	iLiNS−DOSE (31)	KDI	278	94	0.03	0.14 (−0.07, 0.36)	0.09	0.09	271	96	−0.11	0.03 (−0.22, 0.28)	0.06	0.06
Mali	PROMIS CS (32)	DMC	482	448	−0.02	0.13 (−0.12, 0.38)	0.06	0.06	423	476	−0.08	0.08 (−0.15, 0.31)	0.07	0.07
Zimbabwe	SHINE (HIV−) (33)	MDAT	406	359	−0.07	0.10 (−0.05, 0.25)	0.18	0.18	389	391	−0.02	0.08 (−0.06, 0.21)	0.22	0.22
Zimbabwe	SHINE (HIV+) (34)	MDAT	91	78	−0.01	0.12 (−0.17, 0.41)	0.05	0.05	74	67	−0.14	0.18 (−0.18, 0.54)	0.03	0.03
			**3322**	**2842**		**I² = 0.00, Tau² = 0.00**			**3257**	**2717**		**I² = 0.00, Tau² = 0.00**		
**Fixed**						**0.09 (0.02, 0.15)**						**0.09 (0.03, 0.15)**		
**Random**						**0.09 (0.02, 0.15)**						**0.09 (0.03, 0.15)**		

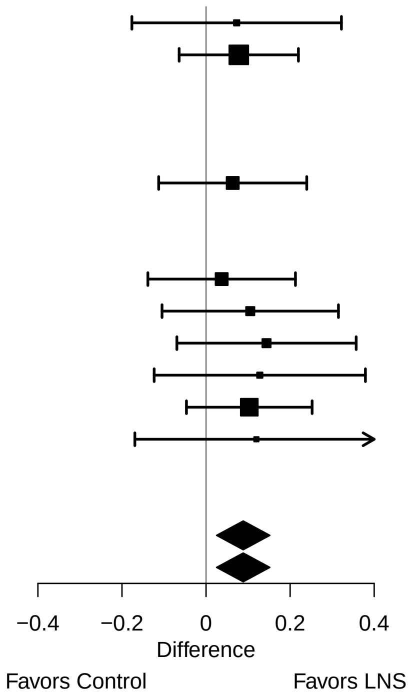

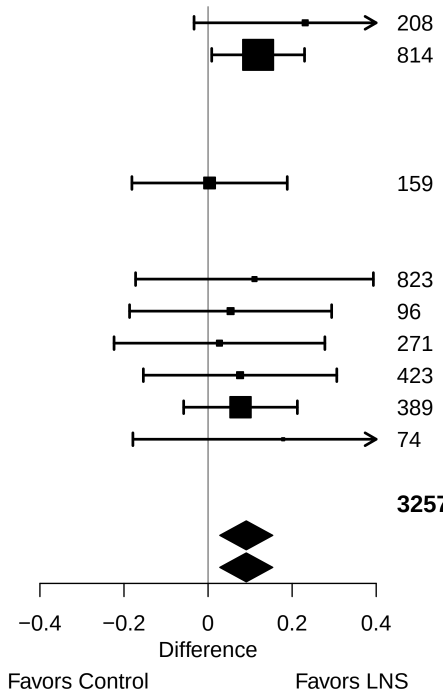

Supplemental figure 8K: Mean difference in fine motor z-score

8K2: Stratified by Household food insecurity

P−for−interaction = 0.352
Difference in MDs = 0.05 (−0.05, 0.15)

			Mild to secure						Moderate to severe					
**Country**	**Trial**	**Tool**	**LNS N**	**Control N**	**Control Mean**	**MD (95% CI)**	**Fixed W**	**Random W**	**LNS N**	**Control N**	**Control Mean**	**MD (95% CI)**	**Fixed W**	**Random W**
Bangladesh	JiVitA−4 (21)	BSID−III	329	111	−0.06	0.14 (−0.09, 0.36)	0.07	0.07	115	32	−0.26	0.19 (−0.10, 0.48)	0.07	0.07
Bangladesh	RDNS (22)	DMC	1045	486	0.04	0.04 (−0.10, 0.17)	0.20	0.20	596	318	−0.19	0.19 (0.06, 0.31)	0.37	0.37
Bangladesh	WASH−B (23)	EASQ												
Burkina Faso	iLiNS−Zinc (24)	DMC												
Ghana	GHANA (25)													
Ghana	iLiNS−DYAD−G (26)	KDI	217	412	0.04	0.07 (−0.06, 0.20)	0.22	0.22	84	186	−0.07	−0.09 (−0.38, 0.21)	0.07	0.07
Haiti	HAITI (27)													
Kenya	WASH−B (28)	EASQ												
Madagascar	MAHAY (29)	ASQI	940	892	0.05	0.02 (−0.20, 0.24)	0.08	0.08	336	389	−0.26	0.12 (−0.28, 0.53)	0.04	0.04
Malawi	iLiNS−DYAD−M (30)	KDI	67	116	−0.08	0.11 (−0.21, 0.43)	0.04	0.04	146	314	0.00	0.07 (−0.11, 0.26)	0.18	0.18
Malawi	iLiNS−DOSE (31)	KDI	134	51	−0.01	0.06 (−0.26, 0.38)	0.04	0.04	403	133	−0.02	0.08 (−0.12, 0.27)	0.15	0.15
Mali	PROMIS CS (32)	DMC												
Zimbabwe	SHINE (HIV−) (33)	MDAT	631	586	−0.02	0.08 (−0.03, 0.20)	0.29	0.29	140	154	−0.14	0.14 (−0.09, 0.37)	0.11	0.11
Zimbabwe	SHINE (HIV+) (34)	MDAT	119	105	−0.07	0.19 (−0.05, 0.42)	0.07	0.07	41	37	−0.11	0.03 (−0.54, 0.60)	0.02	0.02
			**3482**	**2759**		**I² = 0.00, Tau² = 0.00**			**1861**	**1563**		**I² = 0.00, Tau² = 0.00**		
**Fixed**						**0.08 (0.02, 0.14)**						**0.12 (0.04, 0.20)**		
**Random**						**0.08 (0.02, 0.14)**						**0.12 (0.04, 0.20)**		

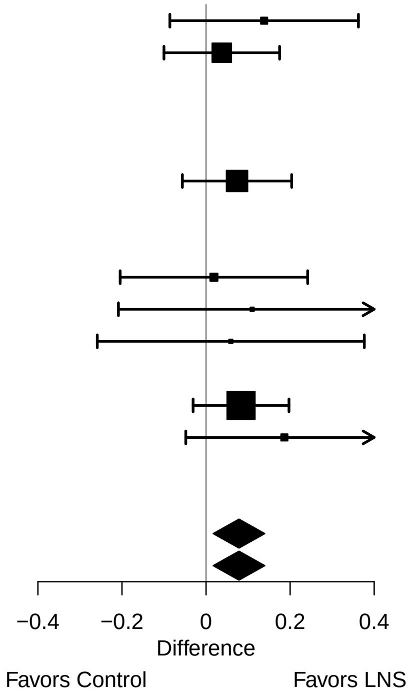

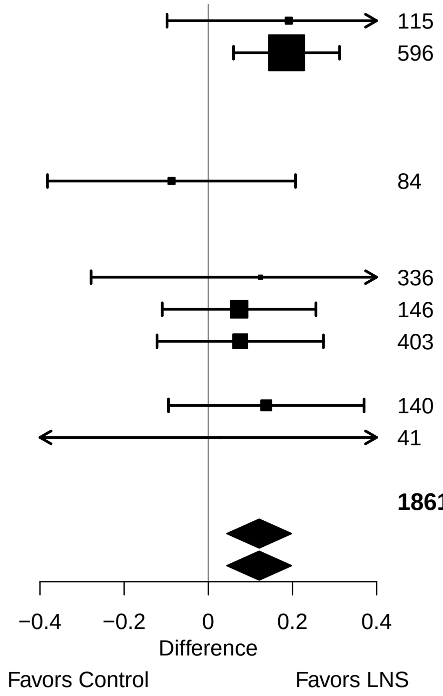

Supplemental figure 8K: Mean difference in fine motor z-score

8K3: Stratified by Household source water quality

P−for−interaction = 0.410
Difference in MDs = 0.07 (−0.10, 0.25)

Country	Trial	Tool	LNS N	Control N	Control Mean	Improved MD (95% CI)	Fixed W	Random W	LNS N	Control N	Control Mean	Unimproved MD (95% CI)	Fixed W	Random W
Bangladesh	JiVitA−4 (21)	BSID−III												
Bangladesh	RDNS (22)	DMC												
Bangladesh	WASH−B (23)	EASQ												
Burkina Faso	iLiNS−Zinc (24)	DMC												
Ghana	GHANA (25)													
Ghana	iLiNS−DYAD−G (26)	KDI												
Haiti	HAITI (27)													
Kenya	WASH−B (28)	EASQ												
Madagascar	MAHAY (29)	ASQI	405	430	−0.14	0.02 (−0.37, 0.41)	0.05	0.05	1134	1133	0.02	0.07 (−0.16, 0.29)	0.34	0.34
Malawi	iLiNS−DYAD−M (30)	KDI	194	398	0.00	0.05 (−0.11, 0.22)	0.30	0.30	20	36	−0.22	0.34 (−0.23, 0.91)	0.05	0.05
Malawi	iLiNS−DOSE (31)	KDI	515	177	−0.01	0.08 (−0.09, 0.25)	0.28	0.28	44	15	−0.38	0.29 (−0.30, 0.89)	0.05	0.05
Mali	PROMIS CS (32)	DMC	516	540	−0.02	0.09 (−0.19, 0.37)	0.10	0.10	348	370	−0.13	0.06 (−0.17, 0.29)	0.30	0.30
Zimbabwe	SHINE (HIV−) (33)	MDAT	228	220	0.10	−0.09 (−0.28, 0.11)	0.22	0.22	131	122	−0.09	0.09 (−0.19, 0.37)	0.22	0.22
Zimbabwe	SHINE (HIV+) (34)	MDAT	37	41	0.01	−0.01 (−0.40, 0.38)	0.05	0.05	25	25	−0.16	−0.21 (−0.85, 0.42)	0.04	0.04
			**1895**	**1806**		**I^2 = 0.00, Tau^2 = 0.00**			**1702**	**1701**		**I^2 = 0.00, Tau^2 = 0.00**		
**Fixed**						**0.03 (−0.06, 0.12)**						**0.08 (−0.05, 0.21)**		
**Random**						**0.03 (−0.06, 0.12)**						**0.08 (−0.05, 0.21)**		

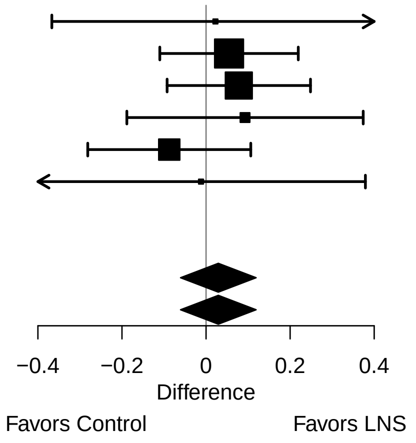

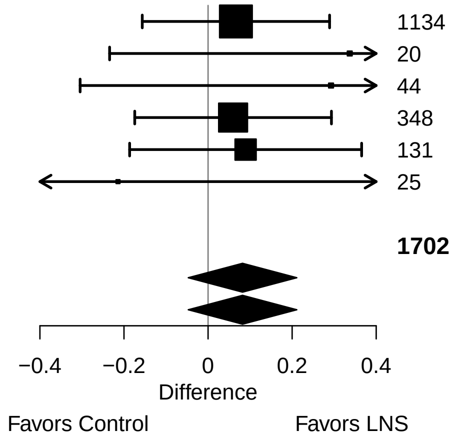

Supplemental figure 8K: Mean difference in fine motor z-score

8K4: Stratified by Household sanitation

P−for−interaction = 0.172
Difference in MDs = −0.09 (−0.23, 0.04)

			Improved						Unimproved					
**Country**	**Trial**	**Tool**	**LNS N**	**Control N**	**Control Mean**	**MD (95% CI)**	**Fixed W**	**Random W**	**LNS N**	**Control N**	**Control Mean**	**MD (95% CI)**	**Fixed W**	**Random W**
Bangladesh	JiVitA−4 (21)	BSID−III	353	112	−0.07	0.14 (−0.06, 0.35)	0.09	0.09	91	31	−0.26	0.13 (−0.32, 0.59)	0.03	0.03
Bangladesh	RDNS (22)	DMC	1161	568	−0.01	0.09 (0.00, 0.17)	0.50	0.50	478	235	−0.16	0.13 (−0.08, 0.34)	0.16	0.16
Bangladesh	WASH−B (23)	EASQ												
Burkina Faso	iLiNS−Zinc (24)	DMC												
Ghana	GHANA (25)													
Ghana	iLiNS−DYAD−G (26)	KDI	292	586	0.00	0.05 (−0.08, 0.17)	0.23	0.23	9	13	0.22	−0.47 (−1.23, 0.30)	0.01	0.01
Haiti	HAITI (27)													
Kenya	WASH−B (28)	EASQ												
Madagascar	MAHAY (29)	ASQI												
Malawi	iLiNS−DYAD−M (30)	KDI	19	41	−0.15	0.36 (−0.14, 0.87)	0.02	0.02	195	393	−0.01	0.05 (−0.12, 0.22)	0.25	0.25
Malawi	iLiNS−DOSE (31)	KDI	12	9	0.08	0.35 (−0.29, 0.99)	0.01	0.01	547	183	−0.05	0.09 (−0.08, 0.26)	0.25	0.25
Mali	PROMIS CS (32)	DMC	668	660	−0.07	0.18 (−0.06, 0.43)	0.07	0.07	210	242	0.02	−0.15 (−0.48, 0.18)	0.06	0.06
Zimbabwe	SHINE (HIV−) (33)	MDAT	133	108	0.00	0.09 (−0.14, 0.32)	0.07	0.07	225	235	0.04	−0.09 (−0.28, 0.11)	0.19	0.19
Zimbabwe	SHINE (HIV+) (34)	MDAT	25	17	−0.05	−0.24 (−0.83, 0.35)	0.01	0.01	37	49	−0.06	0.00 (−0.39, 0.39)	0.05	0.05
			**2663**	**2101**		**I² = 0.00, Tau² = 0.00**			**1792**	**1381**		**I² = 0.00, Tau² = 0.00**		
**Fixed**						**0.09 (0.03, 0.15)**						**0.03 (−0.06, 0.11)**		
**Random**						**0.09 (0.03, 0.15)**						**0.03 (−0.06, 0.11)**		

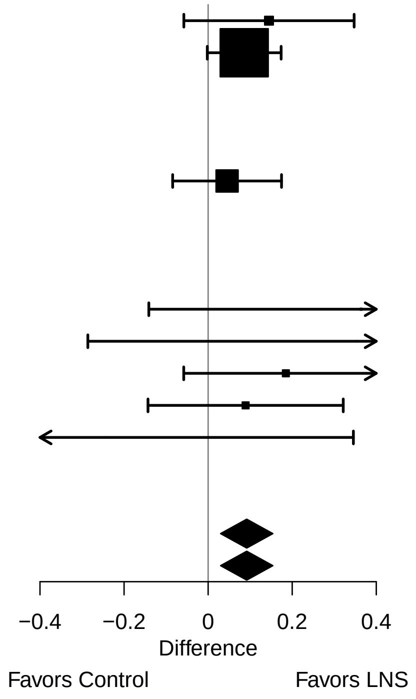

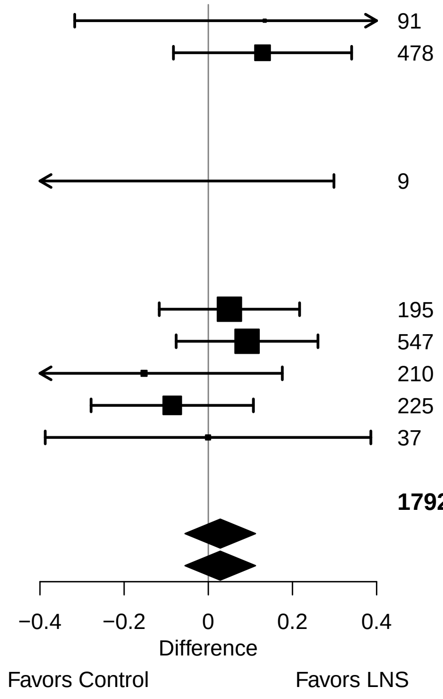

Supplemental figure 8K: Mean difference in fine motor z-score

8K5: Stratified by Home environment

P−for−interaction = 0.860
Difference in MDs = 0.01 (−0.11, 0.13)

Country	Trial	Tool	At least median: LNS N	Control N	Control Mean	MD (95% CI)	Fixed W	Random W	Less than median: LNS N	Control N	Control Mean	MD (95% CI)	Fixed W	Random W
Bangladesh	JiVitA−4 (21)	BSID−III												
Bangladesh	RDNS (22)	DMC	1177	576	0.09	0.06 (−0.04, 0.15)	0.48	0.48	461	225	−0.40	0.20 (−0.05, 0.44)	0.21	0.21
Bangladesh	WASH−B (23)	EASQ												
Burkina Faso	iLiNS−Zinc (24)	DMC												
Ghana	GHANA (25)													
Ghana	iLiNS−DYAD−G (26)	KDI	191	377	0.09	0.04 (−0.10, 0.19)	0.21	0.21	111	224	−0.15	0.03 (−0.22, 0.27)	0.21	0.21
Haiti	HAITI (27)													
Kenya	WASH−B (28)	EASQ												
Madagascar	MAHAY (29)	ASQI	843	839	0.05	0.07 (−0.16, 0.30)	0.08	0.08	770	764	−0.10	0.02 (−0.22, 0.26)	0.22	0.22
Malawi	iLiNS−DYAD−M (30)	KDI	136	287	0.05	0.08 (−0.10, 0.26)	0.12	0.12	78	149	−0.16	0.09 (−0.20, 0.39)	0.15	0.15
Malawi	iLiNS−DOSE (31)	KDI	376	135	0.01	0.09 (−0.10, 0.27)	0.12	0.12	269	86	−0.18	0.10 (−0.15, 0.35)	0.20	0.20
Mali	PROMIS CS (32)	DMC												
Zimbabwe	SHINE (HIV−) (33)	MDAT												
Zimbabwe	SHINE (HIV+) (34)	MDAT												
			**2723**	**2214**		**I^2 = 0.00, Tau^2 = 0.00**			**1689**	**1448**		**I^2 = 0.00, Tau^2 = 0.00**		
**Fixed**						**0.06 (0.00, 0.13)**						**0.09 (−0.03, 0.20)**		
**Random**						**0.06 (0.00, 0.13)**						**0.09 (−0.03, 0.20)**		

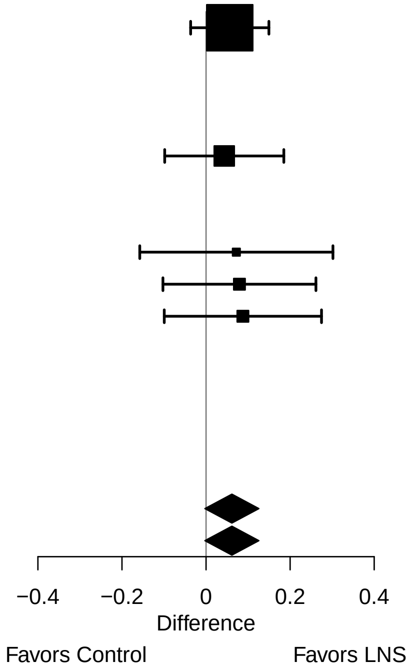

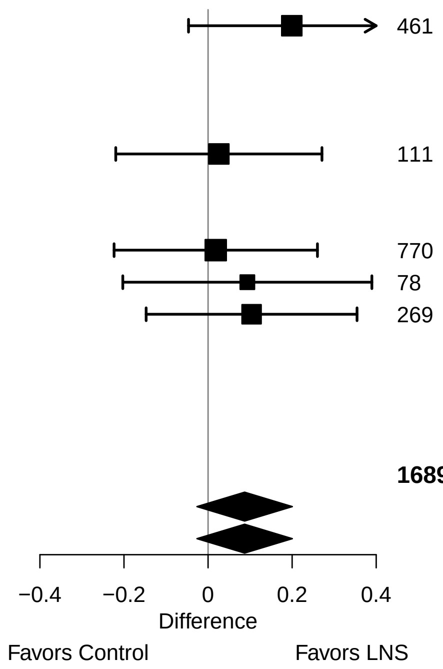

Supplemental figure 8K: Mean difference in fine motor z-score

8K6: Stratified by Season at the time of assessment

P−for−interaction = 0.993
Difference in MDs = 0.00 (−0.09, 0.09)

Country	Trial	Tool	Dry LNS N	Dry Control N	Dry Control Mean	Dry MD (95% CI)	Dry Fixed W	Dry Random W	Rainy LNS N	Rainy Control N	Rainy Control Mean	Rainy MD (95% CI)	Rainy Fixed W	Rainy Random W
Bangladesh	JiVitA−4 (21)	BSID−III	251	70	0.06	0.11 (−0.09, 0.31)	0.09	0.09	194	73	−0.27	0.13 (−0.22, 0.48)	0.05	0.05
Bangladesh	RDNS (22)	DMC	731	352	−0.01	0.07 (−0.03, 0.18)	0.31	0.31	910	452	−0.08	0.12 (−0.02, 0.26)	0.33	0.33
Bangladesh	WASH−B (23)	EASQ												
Burkina Faso	iLiNS−Zinc (24)	DMC												
Ghana	GHANA (25)													
Ghana	iLiNS−DYAD−G (26)	KDI	177	356	0.08	0.00 (−0.17, 0.16)	0.12	0.12	125	245	−0.12	0.10 (−0.10, 0.30)	0.16	0.16
Haiti	HAITI (27)													
Kenya	WASH−B (28)	EASQ												
Madagascar	MAHAY (29)	ASQI												
Malawi	iLiNS−DYAD−M (30)	KDI	153	308	−0.01	0.09 (−0.10, 0.28)	0.10	0.10	61	127	−0.06	0.06 (−0.24, 0.35)	0.08	0.08
Malawi	iLiNS−DOSE (31)	KDI	395	118	−0.14	0.10 (−0.09, 0.30)	0.09	0.09	244	100	0.02	0.11 (−0.14, 0.36)	0.11	0.11
Mali	PROMIS CS (32)	DMC												
Zimbabwe	SHINE (HIV−) (33)	MDAT	603	568	−0.06	0.11 (0.00, 0.23)	0.25	0.25	238	231	0.04	−0.02 (−0.21, 0.16)	0.20	0.20
Zimbabwe	SHINE (HIV+) (34)	MDAT	121	110	−0.14	0.20 (−0.08, 0.48)	0.04	0.04	49	41	0.08	0.08 (−0.22, 0.37)	0.07	0.07
			**2431**	**1882**		**I^2 = 0.00, Tau^2 = 0.00**			**1821**	**1269**		**I^2 = 0.00, Tau^2 = 0.00**		
**Fixed**						**0.09 (0.03, 0.14)**						**0.08 (0.00, 0.16)**		
**Random**						**0.09 (0.03, 0.14)**						**0.08 (0.00, 0.16)**		

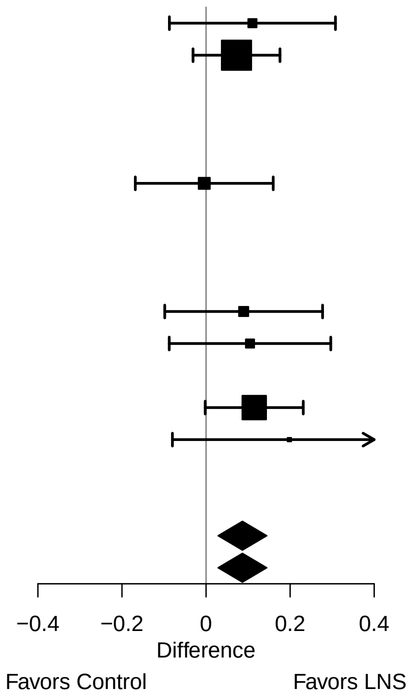

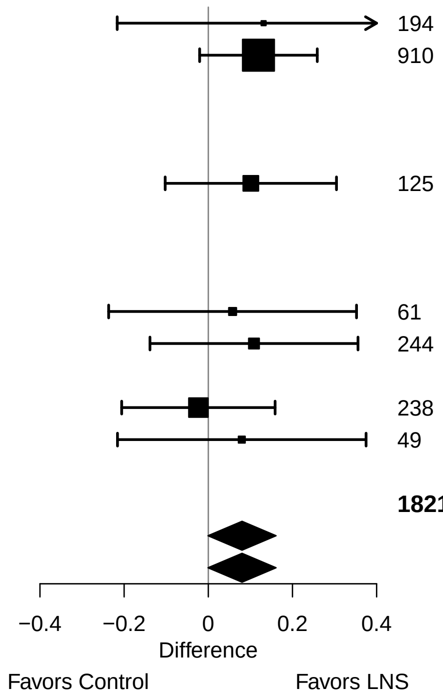

Supplemental figure 8L: Mean difference in executive function z-score

8L1: Stratified by Household socio-economic status

P−for−interaction = 0.033
Difference in MDs = 0.09 (0.01, 0.18)

At least median

Country	Trial	Tool	LNS N	Control N	Control Mean	MD (95% CI)	Fixed W	Random W
Bangladesh	JiVitA−4 (21)							
Bangladesh	RDNS (22)	A not B task	269	133	−0.01	0.07 (−0.11, 0.25)	0.12	0.12
Bangladesh	WASH−B (23)	A not B task	566	1691	0.04	−0.05 (−0.15, 0.05)	0.43	0.43
Burkina Faso	iLiNS−Zinc (24)							
Ghana	GHANA (25)							
Ghana	iLiNS−DYAD−G (26)	A not B task	140	297	−0.04	0.08 (−0.12, 0.28)	0.10	0.10
Haiti	HAITI (27)							
Kenya	WASH−B (28)							
Madagascar	MAHAY (29)							
Malawi	iLiNS−DYAD−M (30)	A not B task	102	192	0.00	−0.13 (−0.37, 0.11)	0.07	0.07
Malawi	iLiNS−DOSE (31)	A not B task	203	72	0.06	−0.02 (−0.28, 0.24)	0.06	0.06
Mali	PROMIS CS (32)							
Zimbabwe	SHINE (HIV−) (33)	A not B task	377	340	−0.02	−0.05 (−0.20, 0.10)	0.17	0.17
Zimbabwe	SHINE (HIV+) (34)	A not B task	88	72	0.13	−0.21 (−0.49, 0.07)	0.05	0.05
			**1745**	**2797**		**I² = 0.00, Tau² = 0.00**		
**Fixed**						**−0.04 (−0.10, 0.03)**		
**Random**						**−0.04 (−0.10, 0.03)**		

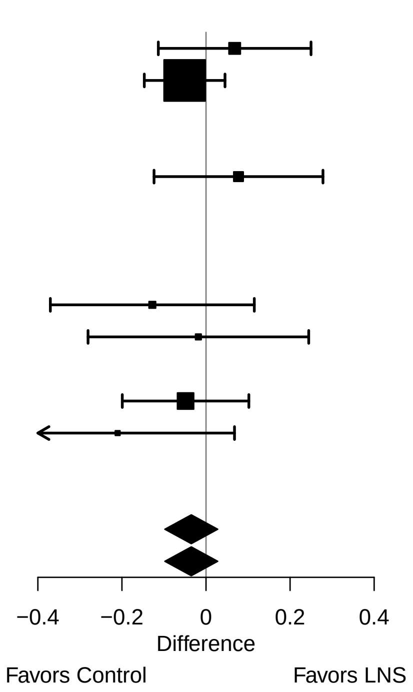

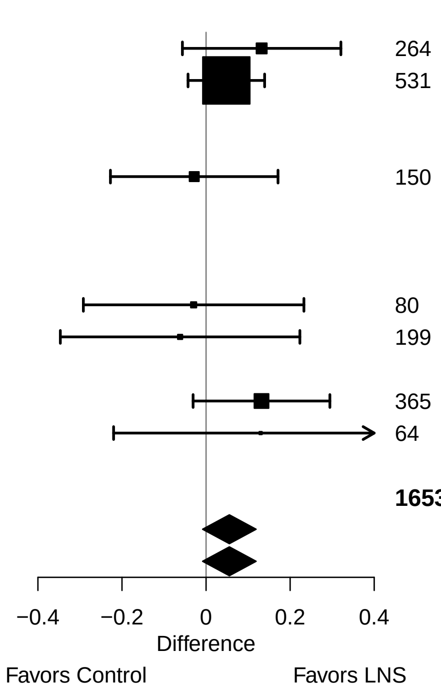

Less than median

Country	Trial	LNS N	Control N	Control Mean	MD (95% CI)	Fixed W	Random W
Bangladesh	JiVitA−4 (21)						
Bangladesh	RDNS (22)	264	133	−0.13	0.13 (−0.06, 0.32)	0.11	0.11
Bangladesh	WASH−B (23)	531	1636	−0.04	0.05 (−0.04, 0.14)	0.49	0.49
Burkina Faso	iLiNS−Zinc (24)						
Ghana	GHANA (25)						
Ghana	iLiNS−DYAD−G (26)	150	277	0.02	−0.03 (−0.23, 0.17)	0.10	0.10
Haiti	HAITI (27)						
Kenya	WASH−B (28)						
Madagascar	MAHAY (29)						
Malawi	iLiNS−DYAD−M (30)	80	176	0.05	−0.03 (−0.29, 0.23)	0.06	0.06
Malawi	iLiNS−DOSE (31)	199	69	0.03	−0.06 (−0.35, 0.22)	0.05	0.05
Mali	PROMIS CS (32)						
Zimbabwe	SHINE (HIV−) (33)	365	352	−0.03	0.13 (−0.03, 0.29)	0.15	0.15
Zimbabwe	SHINE (HIV+) (34)	64	56	−0.04	0.13 (−0.22, 0.48)	0.03	0.03
		**1653**	**2699**		**I² = 0.00, Tau² = 0.00**		
**Fixed**					**0.06 (−0.01, 0.12)**		
**Random**					**0.06 (−0.01, 0.12)**		

Supplemental figure 8L: Mean difference in executive function z-score

8L2: Stratified by Household food insecurity

P−for−interaction = 0.984
Difference in MDs = 0.00 (−0.10, 0.10)

Country	Trial	Tool	LNS N	Control N	Control Mean	Mild to secure MD (95% CI)	Fixed W	Random W	LNS N	Control N	Control Mean	Moderate to severe MD (95% CI)	Fixed W	Random W
Bangladesh	JiVitA−4 (21)													
Bangladesh	RDNS (22)	A not B task	334	164	0.10	−0.05 (−0.20, 0.11)	0.13	0.13	199	102	−0.34	0.34 (0.13, 0.54)	0.16	0.16
Bangladesh	WASH−B (23)	A not B task	876	2590	0.00	0.02 (−0.06, 0.10)	0.50	0.50	221	737	0.01	−0.08 (−0.22, 0.06)	0.33	0.21
Burkina Faso	iLiNS−Zinc (24)													
Ghana	GHANA (25)													
Ghana	iLiNS−DYAD−G (26)	A not B task	209	401	0.01	−0.03 (−0.19, 0.14)	0.11	0.11	80	173	−0.06	0.14 (−0.13, 0.40)	0.10	0.13
Haiti	HAITI (27)													
Kenya	WASH−B (28)													
Madagascar	MAHAY (29)													
Malawi	iLiNS−DYAD−M (30)	A not B task	55	99	−0.09	−0.12 (−0.46, 0.22)	0.03	0.03	126	264	0.07	−0.07 (−0.28, 0.15)	0.15	0.16
Malawi	iLiNS−DOSE (31)	A not B task	103	37	0.11	−0.07 (−0.44, 0.29)	0.02	0.02	294	99	0.02	−0.02 (−0.24, 0.21)	0.12	0.15
Mali	PROMIS CS (32)													
Zimbabwe	SHINE (HIV−) (33)	A not B task	591	547	−0.04	0.08 (−0.05, 0.21)	0.17	0.17	131	137	−0.01	−0.13 (−0.38, 0.12)	0.11	0.13
Zimbabwe	SHINE (HIV+) (34)	A not B task	112	94	0.03	−0.04 (−0.32, 0.25)	0.04	0.04	36	31	0.08	−0.07 (−0.50, 0.36)	0.04	0.07
			**2280**	**3932**		**I² = 0.00, Tau² = 0.00**			**1087**	**1543**		**I² = 0.58, Tau² = 0.01**		
**Fixed**						**0.01 (−0.05, 0.06)**						**0.01 (−0.07, 0.09)**		
**Random**						**0.01 (−0.05, 0.06)**						**0.02 (−0.11, 0.15)**		

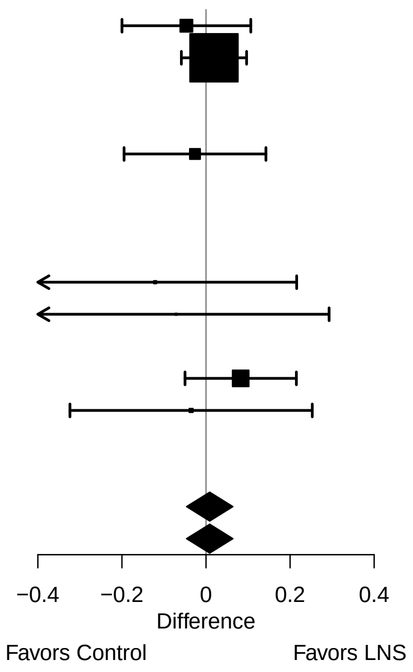

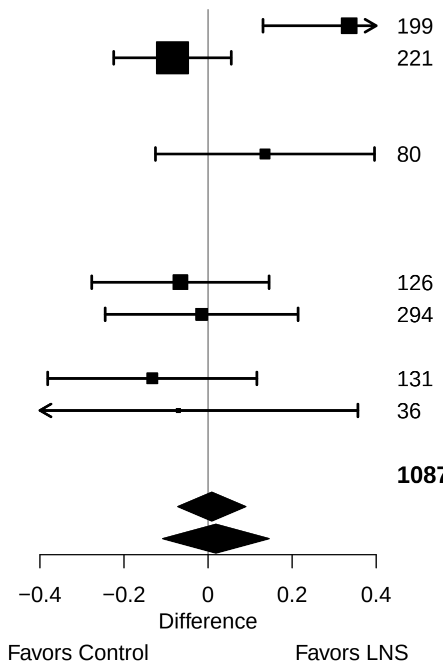

Supplemental figure 8L: Mean difference in executive function z-score

8L3: Stratified by Household source water quality

P−for−interaction = 0.700
Difference in MDs = 0.04 (−0.16, 0.24)

Country	Trial	Tool	LNS N	Control N	Control Mean	Improved MD (95% CI)	Fixed W	Random W	LNS N	Control N	Control Mean	Unimproved MD (95% CI)	Fixed W	Random W
Bangladesh	JiVitA−4 (21)													
Bangladesh	RDNS (22)	A not B task												
Bangladesh	WASH−B (23)	A not B task	474	981	−0.06	0.05 (−0.06, 0.15)	0.51	0.51	66	117	−0.13	0.10 (−0.21, 0.41)	0.33	0.33
Burkina Faso	iLiNS−Zinc (24)													
Ghana	GHANA (25)													
Ghana	iLiNS−DYAD−G (26)	A not B task												
Haiti	HAITI (27)													
Kenya	WASH−B (28)													
Madagascar	MAHAY (29)													
Malawi	iLiNS−DYAD−M (30)	A not B task	168	335	0.02	−0.09 (−0.28, 0.09)	0.17	0.17	14	32	0.03	0.04 (−0.65, 0.73)	0.07	0.07
Malawi	iLiNS−DOSE (31)	A not B task	380	131	0.07	−0.06 (−0.26, 0.14)	0.15	0.15	30	11	−0.19	0.11 (−0.60, 0.81)	0.06	0.06
Mali	PROMIS CS (32)													
Zimbabwe	SHINE (HIV−) (33)	A not B task	215	212	0.02	−0.06 (−0.27, 0.14)	0.14	0.14	121	108	−0.01	−0.02 (−0.29, 0.25)	0.43	0.43
Zimbabwe	SHINE (HIV+) (34)	A not B task	35	38	0.03	−0.08 (−0.53, 0.38)	0.03	0.03	23	17	0.22	−0.36 (−0.90, 0.18)	0.11	0.11
			**1272**	**1697**		**I² = 0.00, Tau² = 0.00**			**254**	**285**		**I² = 0.00, Tau² = 0.00**		
**Fixed**						**−0.01 (−0.09, 0.06)**						**−0.01 (−0.18, 0.17)**		
**Random**						**−0.01 (−0.09, 0.06)**						**−0.01 (−0.18, 0.17)**		

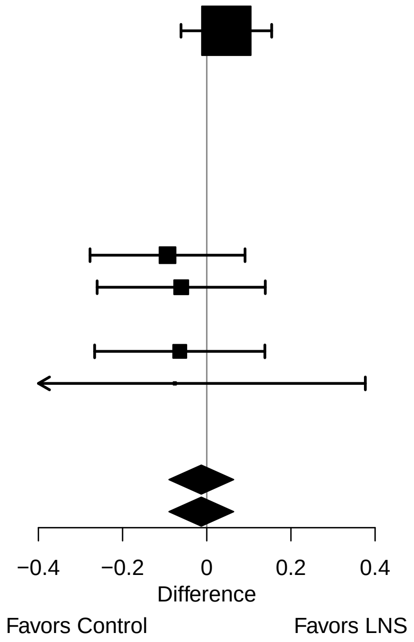

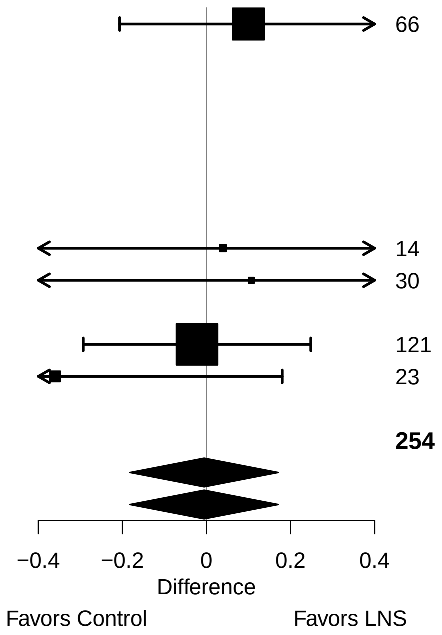

Supplemental figure 8L: Mean difference in executive function z-score

8L4: Stratified by Household sanitation

P−for−interaction = 0.947
Difference in MDs = 0.01 (−0.18, 0.19)

Country	Trial	Tool	LNS N	Control N	Control Mean	Improved MD (95% CI)	Fixed W	Random W	LNS N	Control N	Control Mean	Unimproved MD (95% CI)	Fixed W	Random W
Bangladesh	JiVitA−4 (21)													
Bangladesh	RDNS (22)	A not B task	370	192	−0.06	0.12 (−0.04, 0.27)	0.21	0.22	163	74	−0.09	0.06 (−0.18, 0.31)	0.15	0.17
Bangladesh	WASH−B (23)	A not B task	487	998	−0.04	0.04 (−0.07, 0.14)	0.45	0.44	27	53	−0.46	0.35 (−0.25, 0.94)	0.03	0.05
Burkina Faso	iLiNS−Zinc (24)													
Ghana	GHANA (25)													
Ghana	iLiNS−DYAD−G (26)	A not B task	281	561	−0.02	0.04 (−0.10, 0.19)	0.25	0.25	8	13	0.47	−0.73 (−1.49, 0.02)	0.02	0.03
Haiti	HAITI (27)													
Kenya	WASH−B (28)													
Madagascar	MAHAY (29)													
Malawi	iLiNS−DYAD−M (30)	A not B task	18	31	−0.13	0.26 (−0.34, 0.87)	0.01	0.01	164	336	0.04	−0.12 (−0.30, 0.07)	0.26	0.22
Malawi	iLiNS−DOSE (31)	A not B task	9	7	0.62	−0.83 (−1.94, 0.28)	0.00	0.00	401	135	0.02	−0.01 (−0.21, 0.18)	0.24	0.21
Mali	PROMIS CS (32)													
Zimbabwe	SHINE (HIV−) (33)	A not B task	121	102	0.13	−0.18 (−0.47, 0.11)	0.06	0.06	214	219	−0.05	0.00 (−0.18, 0.19)	0.25	0.22
Zimbabwe	SHINE (HIV+) (34)	A not B task	23	15	−0.17	0.06 (−0.62, 0.75)	0.01	0.01	35	40	0.18	−0.25 (−0.63, 0.13)	0.06	0.10
			**1309**	**1906**		**I² = 0.01, Tau² = 0.00**			**1012**	**870**		**I² = 0.23, Tau² = 0.01**		
**Fixed**						**0.04 (−0.03, 0.11)**						**−0.04 (−0.14, 0.05)**		
**Random**						**0.04 (−0.03, 0.12)**						**−0.05 (−0.19, 0.09)**		

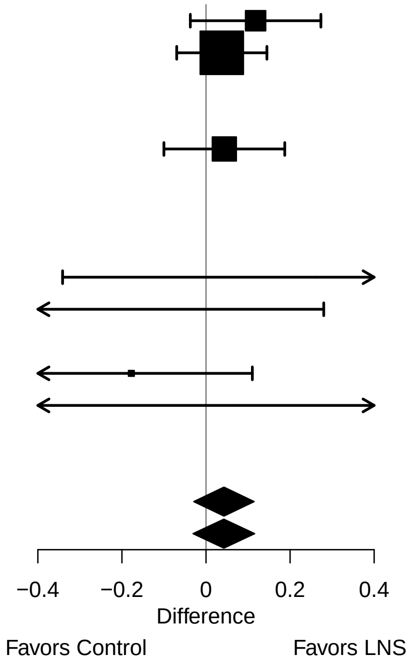

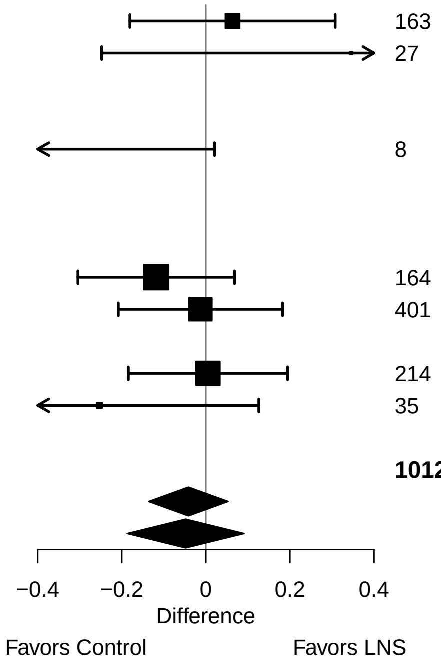

Supplemental figure 8L: Mean difference in executive function z-score

8L5: Stratified by Home environment

P−for−interaction = 0.142
Difference in MDs = −0.08 (−0.18, 0.03)

			At least median						Less than median					
**Country**	**Trial**	**Tool**	**LNS N**	**Control N**	**Control Mean**	**MD (95% CI)**	**Fixed W**	**Random W**	**LNS N**	**Control N**	**Control Mean**	**MD (95% CI)**	**Fixed W**	**Random W**
Bangladesh	JiVitA−4 (21)													
Bangladesh	RDNS (22)	A not B task	385	195	−0.06	0.15 (0.02, 0.29)	0.21	0.23	146	70	−0.08	−0.05 (−0.32, 0.21)	0.09	0.09
Bangladesh	WASH−B (23)	A not B task	623	1638	0.08	−0.01 (−0.09, 0.08)	0.50	0.37	474	1688	−0.08	−0.03 (−0.12, 0.07)	0.66	0.66
Burkina Faso	iLiNS−Zinc (24)													
Ghana	GHANA (25)													
Ghana	iLiNS−DYAD−G (26)	A not B task	185	363	−0.04	0.11 (−0.07, 0.29)	0.12	0.16	105	213	0.05	−0.12 (−0.35, 0.11)	0.12	0.12
Haiti	HAITI (27)													
Kenya	WASH−B (28)													
Madagascar	MAHAY (29)													
Malawi	iLiNS−DYAD−M (30)	A not B task	120	254	0.02	−0.06 (−0.27, 0.15)	0.09	0.13	62	115	0.06	−0.14 (−0.48, 0.19)	0.06	0.06
Malawi	iLiNS−DOSE (31)	A not B task	291	99	0.05	−0.07 (−0.30, 0.16)	0.08	0.11	183	65	0.14	−0.18 (−0.46, 0.11)	0.08	0.08
Mali	PROMIS CS (32)													
Zimbabwe	SHINE (HIV−) (33)	A not B task												
Zimbabwe	SHINE (HIV+) (34)	A not B task												
			**1604**	**2549**		**I² = 0.32, Tau² = 0.00**			**970**	**2151**		**I² = 0.00, Tau² = 0.00**		
**Fixed**						**0.03 (−0.03, 0.09)**						**−0.06 (−0.14, 0.02)**		
**Random**						**0.04 (−0.05, 0.12)**						**−0.06 (−0.14, 0.02)**		

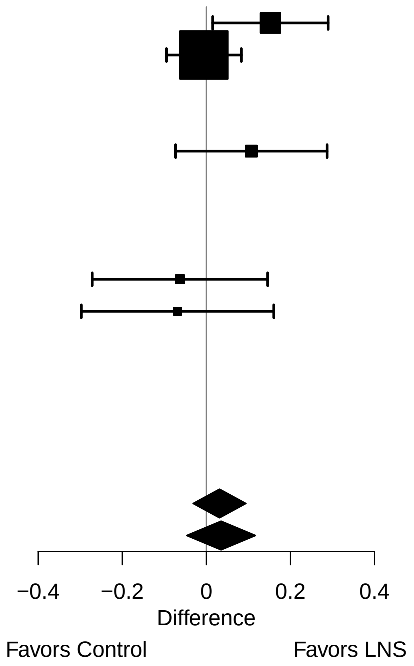

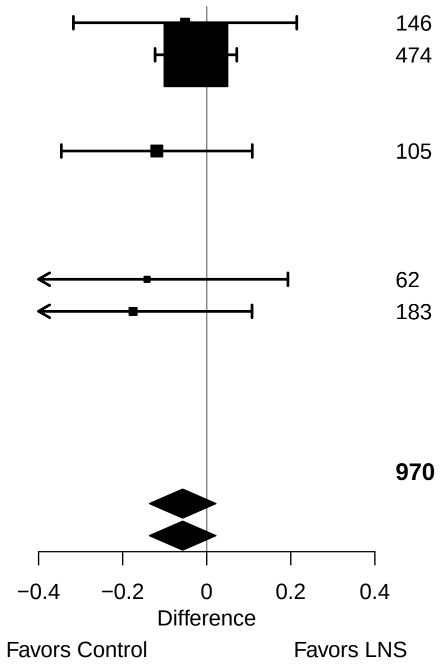

Supplemental figure 8L: Mean difference in executive function z-score

8L6: Stratified by Season at the time of assessment

P−for−interaction = 0.766
Difference in MDs = 0.01 (−0.08, 0.11)

Country	Trial	Tool	LNS N	Control N	Control Mean	Dry MD (95% CI)	Fixed W	Random W	LNS N	Control N	Control Mean	Rainy MD (95% CI)	Fixed W	Random W
Bangladesh	JiVitA−4 (21)													
Bangladesh	RDNS (22)	A not B task	242	120	−0.06	0.05 (−0.15, 0.26)	0.09	0.09	291	146	−0.07	0.14 (−0.04, 0.32)	0.16	0.18
Bangladesh	WASH−B (23)	A not B task	620	1921	−0.01	0.02 (−0.08, 0.12)	0.38	0.38	477	1406	0.01	−0.03 (−0.14, 0.07)	0.46	0.26
Burkina Faso	iLiNS−Zinc (24)													
Ghana	GHANA (25)													
Ghana	iLiNS−DYAD−G (26)	A not B task	172	346	0.02	−0.07 (−0.26, 0.12)	0.10	0.10	118	230	−0.04	0.16 (−0.05, 0.37)	0.11	0.15
Haiti	HAITI (27)													
Kenya	WASH−B (28)													
Madagascar	MAHAY (29)													
Malawi	iLiNS−DYAD−M (30)	A not B task	130	261	−0.07	−0.01 (−0.22, 0.20)	0.08	0.08	52	107	0.29	−0.28 (−0.60, 0.03)	0.05	0.09
Malawi	iLiNS−DOSE (31)	A not B task	295	89	0.21	−0.25 (−0.48, −0.02)	0.07	0.07	176	73	−0.06	0.05 (−0.24, 0.33)	0.06	0.10
Mali	PROMIS CS (32)													
Zimbabwe	SHINE (HIV−) (33)	A not B task	563	519	−0.05	0.03 (−0.10, 0.16)	0.21	0.21	224	220	0.04	0.06 (−0.12, 0.25)	0.15	0.17
Zimbabwe	SHINE (HIV+) (34)	A not B task	112	95	0.05	−0.02 (−0.26, 0.21)	0.07	0.07	45	38	0.06	−0.28 (−0.79, 0.24)	0.02	0.04
			**2134**	**3351**		**I² = 0.00, Tau² = 0.00**			**1383**	**2220**		**I² = 0.38, Tau² = 0.01**		
**Fixed**						**−0.01 (−0.07, 0.05)**						**0.02 (−0.05, 0.09)**		
**Random**						**−0.01 (−0.07, 0.05)**						**0.02 (−0.09, 0.13)**		

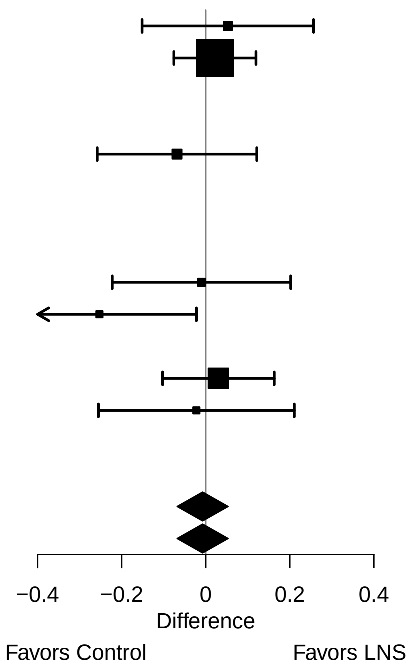

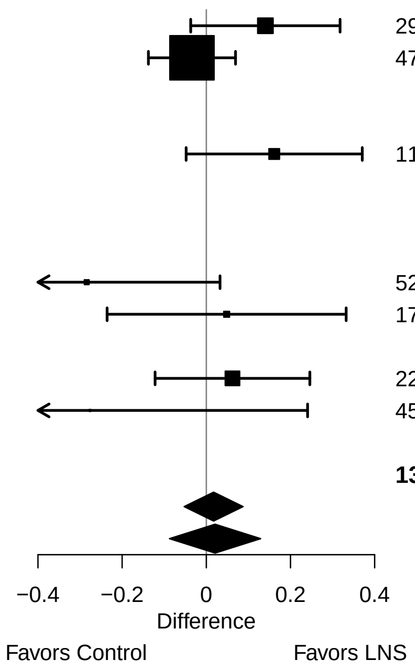

Supplemental figure 8M: Executive function lowest decile prevalence ratio

8M1: Stratified by Household socio-economic status

P−for−interaction = 0.389
Ratio of PRs = 0.88 (0.66, 1.17)

Country	Trial	Tool	At least median: LNS N	Control N	Control Prevalence	PR (95% CI)	Fixed W	Random W	Less than median: LNS N	Control N	Control Prevalence	PR (95% CI)	Fixed W	Random W
Bangladesh	JiVitA−4 (21)													
Bangladesh	RDNS (22)	A not B task	269	133	9.8	0.80 (0.42, 1.52)	0.10	0.12	264	133	12.0	0.94 (0.58, 1.53)	0.18	0.18
Bangladesh	WASH−B (23)	A not B task	566	1691	8.8	1.17 (0.87, 1.57)	0.50	0.46	531	1636	11.6	0.78 (0.58, 1.05)	0.48	0.48
Burkina Faso	iLiNS−Zinc (24)													
Ghana	GHANA (25)													
Ghana	iLiNS−DYAD−G (26)	A not B task	140	297	13.1	0.60 (0.32, 1.13)	0.11	0.12	150	277	9.0	1.03 (0.55, 1.93)	0.11	0.11
Haiti	HAITI (27)													
Kenya	WASH−B (28)													
Madagascar	MAHAY (29)													
Malawi	iLiNS−DYAD−M (30)	A not B task												
Malawi	iLiNS−DOSE (31)	A not B task	203	72	8.3	1.00 (0.41, 2.45)	0.05	0.06	199	69	7.2	1.73 (0.69, 4.35)	0.05	0.05
Mali	PROMIS CS (32)													
Zimbabwe	SHINE (HIV−) (33)	A not B task	377	340	12.1	0.84 (0.55, 1.28)	0.24	0.25	365	352	11.1	0.77 (0.48, 1.22)	0.19	0.19
Zimbabwe	SHINE (HIV+) (34)	A not B task												
			**1555**	**2533**		**I² = 0.13, Tau² = 0.01**			**1509**	**2467**		**I² = 0.00, Tau² = 0.00**		
**Fixed**						**0.96 (0.78, 1.18)**						**0.86 (0.70, 1.06)**		
**Random**						**0.94 (0.75, 1.18)**						**0.86 (0.70, 1.06)**		

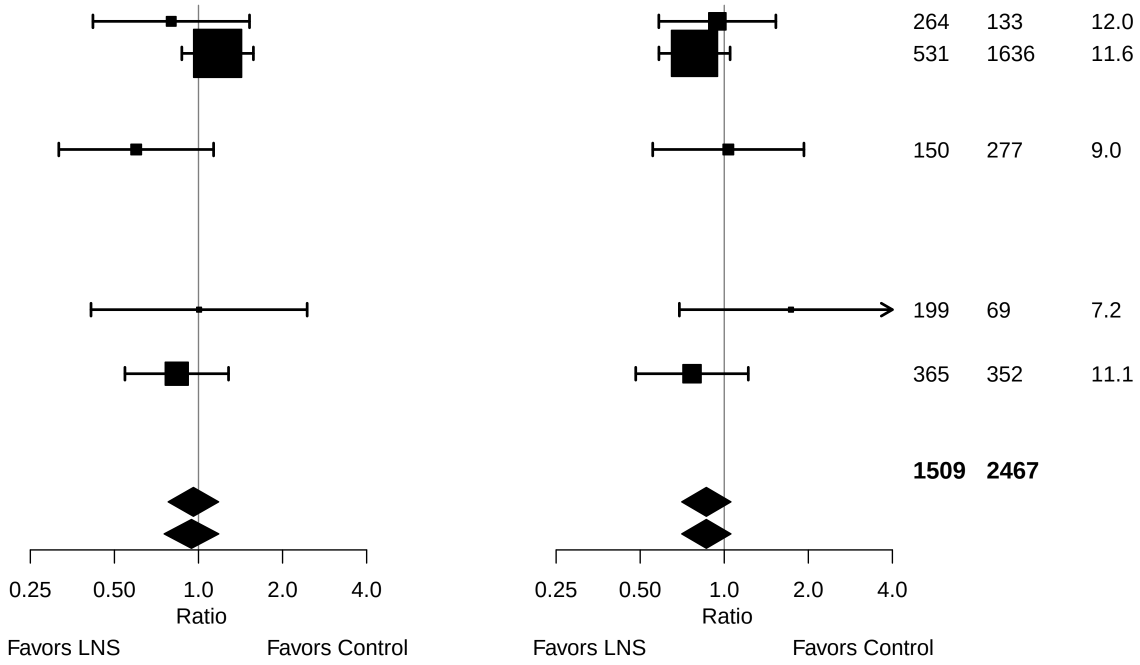

Supplemental figure 8M: Executive function lowest decile prevalence ratio

8M2: Stratified by Household food insecurity

P−for−interaction = 0.640
Ratio of PRs = 1.08 (0.79, 1.47)

Country	Trial	Tool	LNS N	Control N	Control Prevalence	Mild to secure PR (95% CI)	Fixed W	Random W	LNS N	Control N	Control Prevalence	Moderate to severe PR (95% CI)	Fixed W	Random W
Bangladesh	JiVitA−4 (21)													
Bangladesh	RDNS (22)	A not B task	334	164	7.3	1.10 (0.60, 2.02)	0.08	0.08	199	102	16.7	0.72 (0.45, 1.18)	0.26	0.26
Bangladesh	WASH−B (23)	A not B task	876	2590	10.3	0.92 (0.72, 1.17)	0.52	0.52	221	737	9.5	1.10 (0.72, 1.66)	0.36	0.36
Burkina Faso	iLiNS−Zinc (24)													
Ghana	GHANA (25)													
Ghana	iLiNS−DYAD−G (26)	A not B task	209	401	12.0	0.72 (0.43, 1.20)	0.12	0.12	80	173	9.2	0.95 (0.41, 2.21)	0.09	0.09
Haiti	HAITI (27)													
Kenya	WASH−B (28)													
Madagascar	MAHAY (29)													
Malawi	iLiNS−DYAD−M (30)	A not B task	55	99	13.1	0.83 (0.33, 2.06)	0.04	0.04	126	264	10.2	0.78 (0.39, 1.55)	0.13	0.13
Malawi	iLiNS−DOSE (31)	A not B task												
Mali	PROMIS CS (32)													
Zimbabwe	SHINE (HIV−) (33)	A not B task	591	547	11.5	0.78 (0.54, 1.11)	0.24	0.24	131	137	11.7	0.98 (0.53, 1.83)	0.16	0.16
Zimbabwe	SHINE (HIV+) (34)	A not B task												
			**2065**	**3801**		**I² = 0.00, Tau² = 0.00**			**757**	**1413**		**I² = 0.00, Tau² = 0.00**		
**Fixed**						**0.87 (0.73, 1.04)**						**0.91 (0.71, 1.17)**		
**Random**						**0.87 (0.73, 1.04)**						**0.91 (0.71, 1.17)**		

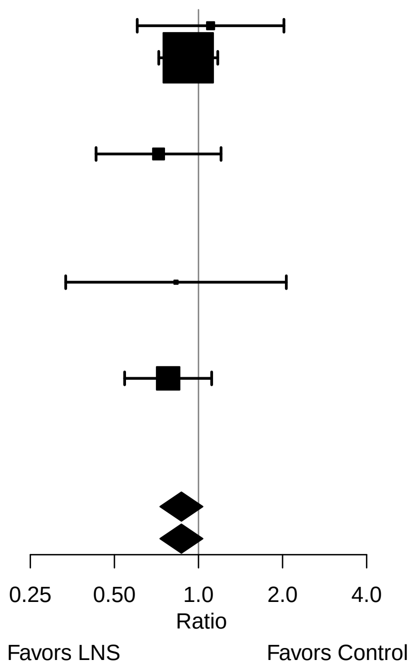

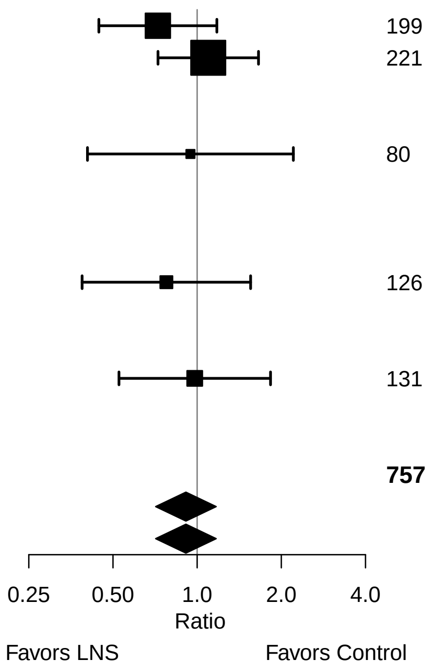

Supplemental figure 8M: Executive function lowest decile prevalence ratio

8M3: Stratified by Household source water quality (insufficient comparisons)

Supplemental figure 8M: Executive function lowest decile prevalence ratio

8M4: Stratified by Household sanitation (insufficient comparisons)

Supplemental figure 8M: Executive function lowest decile prevalence ratio

8M5: Stratified by Home environment

P−for−interaction = 0.287
Ratio of PRs = 1.18 (0.87, 1.59)

Country	Trial	Tool	LNS N	Control N	Control Prevalence	At least median PR (95% CI)	Fixed W	Random W	LNS N	Control N	Control Prevalence	Less than median PR (95% CI)	Fixed W	Random W
Bangladesh	JiVitA−4 (21)													
Bangladesh	RDNS (22)	A not B task	385	195	11.3	0.67 (0.41, 1.08)	0.22	0.22	146	70	10.0	1.51 (0.78, 2.91)	0.11	0.11
Bangladesh	WASH−B (23)	A not B task	623	1638	7.6	0.98 (0.72, 1.33)	0.53	0.53	474	1688	12.6	1.00 (0.78, 1.29)	0.73	0.73
Burkina Faso	iLiNS−Zinc (24)													
Ghana	GHANA (25)													
Ghana	iLiNS−DYAD−G (26)	A not B task	185	363	11.8	0.68 (0.39, 1.20)	0.16	0.16	105	213	9.9	0.97 (0.47, 1.98)	0.09	0.09
Haiti	HAITI (27)													
Kenya	WASH−B (28)													
Madagascar	MAHAY (29)													
Malawi	iLiNS−DYAD−M (30)	A not B task	120	254	8.7	0.87 (0.41, 1.82)	0.09	0.09	62	115	15.7	0.72 (0.32, 1.63)	0.07	0.07
Malawi	iLiNS−DOSE (31)	A not B task												
Mali	PROMIS CS (32)													
Zimbabwe	SHINE (HIV−) (33)	A not B task												
Zimbabwe	SHINE (HIV+) (34)	A not B task												
			**1313**	**2450**		**I² = 0.00, Tau² = 0.00**			**787**	**2086**		**I² = 0.00, Tau² = 0.00**		
**Fixed**						**0.84 (0.67, 1.05)**						**1.02 (0.82, 1.27)**		
**Random**						**0.84 (0.67, 1.05)**						**1.02 (0.82, 1.27)**		

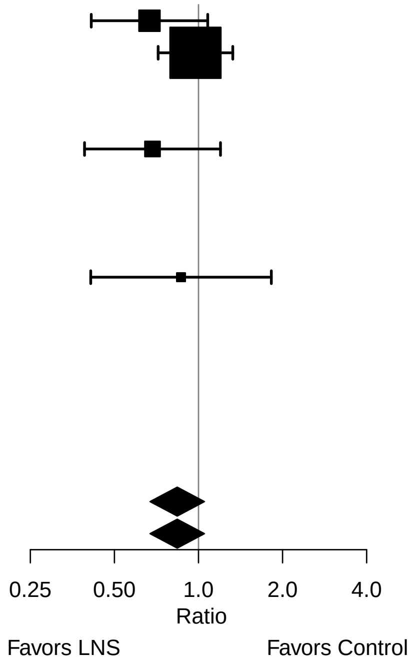

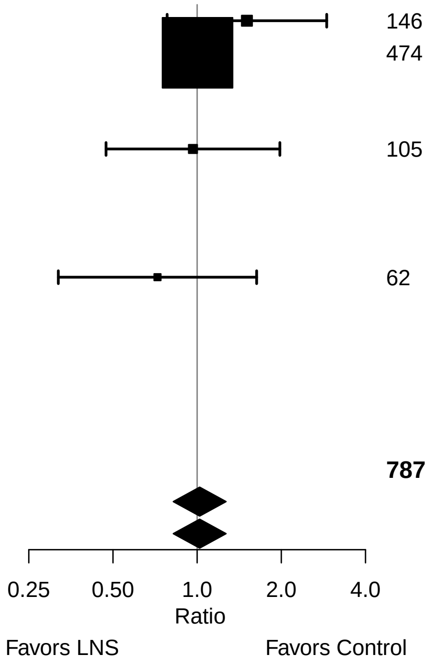

Supplemental figure 8M: Executive function lowest decile prevalence ratio

8M6: Stratified by Season at the time of assessment

P−for−interaction = 0.819
Ratio of PRs = 1.04 (0.75, 1.44)

Country	Trial	Tool	Dry LNS N	Dry Control N	Dry Control Prevalence	Dry PR (95% CI)	Dry Fixed W	Dry Random W	Rainy LNS N	Rainy Control N	Rainy Control Prevalence	Rainy PR (95% CI)	Rainy Fixed W	Rainy Random W
Bangladesh	JiVitA−4 (21)													
Bangladesh	RDNS (22)	A not B task	242	120	11.7	0.81 (0.42, 1.57)	0.10	0.10	291	146	10.3	0.94 (0.56, 1.57)	0.23	0.23
Bangladesh	WASH−B (23)	A not B task	620	1921	10.2	0.95 (0.72, 1.26)	0.52	0.52	477	1406	10.1	0.95 (0.69, 1.32)	0.59	0.59
Burkina Faso	iLiNS−Zinc (24)													
Ghana	GHANA (25)													
Ghana	iLiNS−DYAD−G (26)	A not B task												
Haiti	HAITI (27)													
Kenya	WASH−B (28)													
Madagascar	MAHAY (29)													
Malawi	iLiNS−DYAD−M (30)	A not B task												
Malawi	iLiNS−DOSE (31)	A not B task												
Mali	PROMIS CS (32)													
Zimbabwe	SHINE (HIV−) (33)	A not B task	563	519	12.5	0.78 (0.56, 1.09)	0.38	0.38	224	220	9.1	0.83 (0.46, 1.52)	0.17	0.17
Zimbabwe	SHINE (HIV+) (34)	A not B task												
			**1425**	**2560**		**I^2 = 0.00, Tau^2 = 0.00**			**992**	**1772**		**I^2 = 0.00, Tau^2 = 0.00**		
**Fixed**						**0.87 (0.71, 1.07)**						**0.93 (0.72, 1.19)**		
**Random**						**0.87 (0.71, 1.07)**						**0.93 (0.72, 1.19)**		

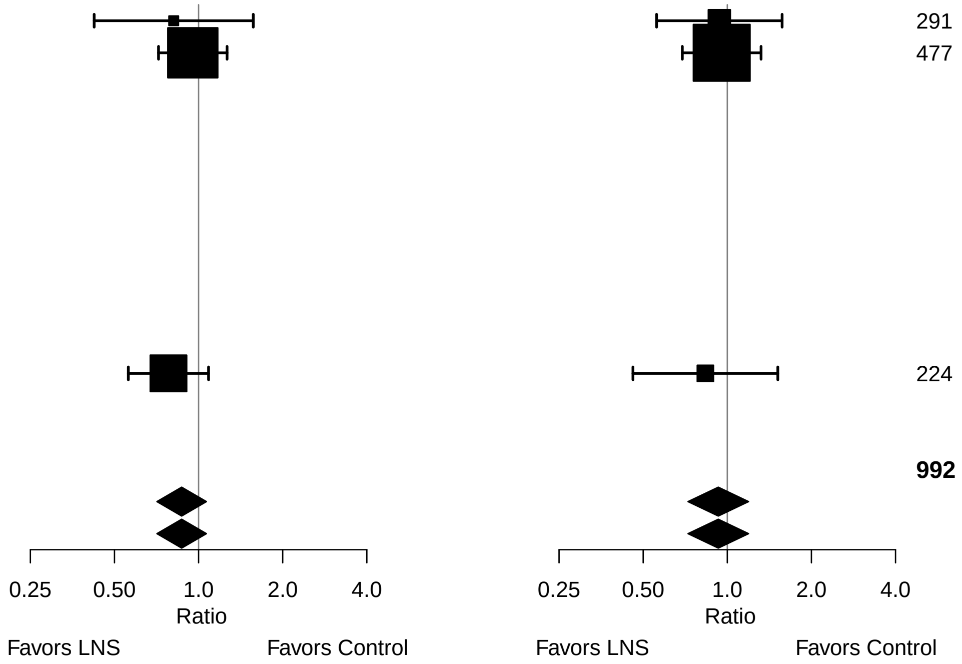

Supplemental figure 8N: Executive function lowest decile prevalence difference

8N1: Stratified by Household socio-economic status

P−for−interaction = 0.517
Difference in PDs = −0.01 (−0.04, 0.02)

Country	Trial	Tool	LNS N	Control N	Control Prevalence	At least median PD (95% CI)	Fixed W	Random W	LNS N	Control N	Control Prevalence	Less than median PD (95% CI)	Fixed W	Random W
Bangladesh	JiVitA−4 (21)													
Bangladesh	RDNS (22)	A not B task	269	133	9.8	−0.02 (−0.08, 0.04)	0.13	0.15	264	133	12.0	−0.01 (−0.06, 0.05)	0.12	0.12
Bangladesh	WASH−B (23)	A not B task	566	1691	8.8	0.01 (−0.01, 0.04)	0.50	0.45	531	1636	11.6	−0.03 (−0.05, 0.00)	0.51	0.51
Burkina Faso	iLiNS−Zinc (24)													
Ghana	GHANA (25)													
Ghana	iLiNS−DYAD−G (26)	A not B task	140	297	13.1	−0.05 (−0.12, 0.01)	0.10	0.12	150	277	9.0	0.00 (−0.05, 0.06)	0.12	0.12
Haiti	HAITI (27)													
Kenya	WASH−B (28)													
Madagascar	MAHAY (29)													
Malawi	iLiNS−DYAD−M (30)	A not B task												
Malawi	iLiNS−DOSE (31)	A not B task	203	72	8.3	0.00 (−0.07, 0.08)	0.08	0.09	199	69	7.2	0.05 (−0.03, 0.14)	0.05	0.05
Mali	PROMIS CS (32)													
Zimbabwe	SHINE (HIV−) (33)	A not B task	377	340	12.1	−0.02 (−0.07, 0.03)	0.19	0.20	365	352	11.1	−0.03 (−0.07, 0.02)	0.20	0.20
Zimbabwe	SHINE (HIV+) (34)	A not B task												
			**1555**	**2533**		**I² = 0.13, Tau² = 0.00**			**1509**	**2467**		**I² = 0.00, Tau² = 0.00**		
**Fixed**						**0.00 (−0.02, 0.02)**						**−0.02 (−0.04, 0.00)**		
**Random**						**−0.01 (−0.03, 0.02)**						**−0.02 (−0.04, 0.00)**		

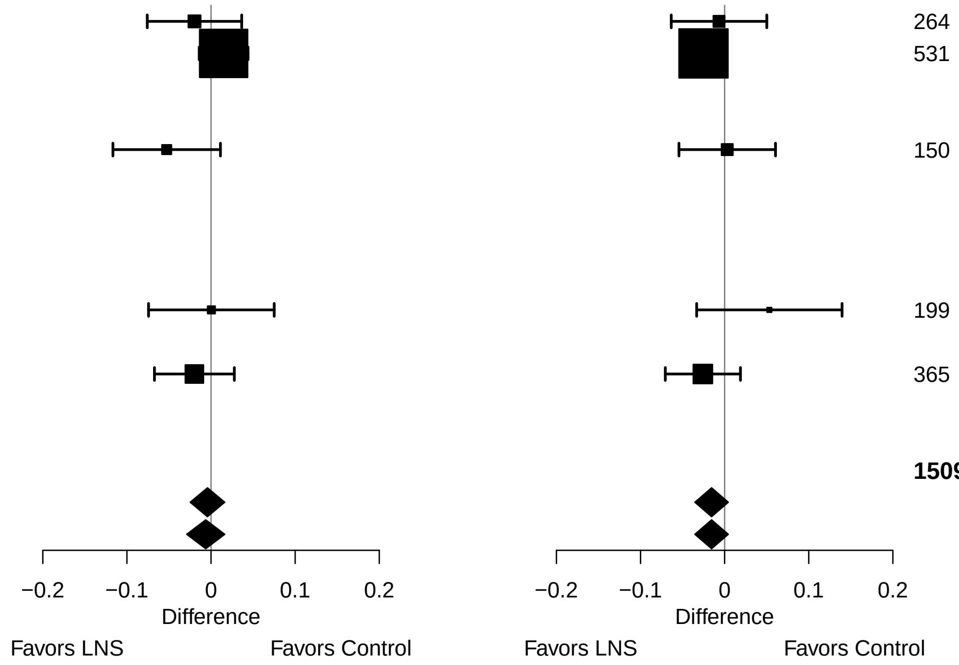

Supplemental figure 8N: Executive function lowest decile prevalence difference

8N2: Stratified by Household food insecurity

P−for−interaction = 0.586
Difference in PDs = 0.01 (−0.03, 0.04)

Country	Trial	Tool	LNS N	Control N	Control Prevalence	Mild to secure PD (95% CI)	Fixed W	Random W	LNS N	Control N	Control Prevalence	Moderate to severe PD (95% CI)	Fixed W	Random W
Bangladesh	JiVitA−4 (21)													
Bangladesh	RDNS (22)	A not B task	334	164	7.3	0.01 (−0.04, 0.05)	0.14	0.14	199	102	16.7	−0.05 (−0.11, 0.02)	0.16	0.16
Bangladesh	WASH−B (23)	A not B task	876	2590	10.3	−0.01 (−0.03, 0.02)	0.53	0.53	221	737	9.5	0.01 (−0.03, 0.05)	0.40	0.40
Burkina Faso	iLiNS−Zinc (24)													
Ghana	GHANA (25)													
Ghana	iLiNS−DYAD−G (26)	A not B task	209	401	12.0	−0.03 (−0.09, 0.02)	0.11	0.11	80	173	9.2	0.00 (−0.08, 0.07)	0.12	0.12
Haiti	HAITI (27)													
Kenya	WASH−B (28)													
Madagascar	MAHAY (29)													
Malawi	iLiNS−DYAD−M (30)	A not B task	55	99	13.1	−0.02 (−0.13, 0.09)	0.02	0.02	126	264	10.2	−0.02 (−0.09, 0.04)	0.18	0.18
Malawi	iLiNS−DOSE (31)	A not B task												
Mali	PROMIS CS (32)													
Zimbabwe	SHINE (HIV−) (33)	A not B task	591	547	11.5	−0.03 (−0.06, 0.01)	0.20	0.20	131	137	11.7	0.00 (−0.07, 0.07)	0.14	0.14
Zimbabwe	SHINE (HIV+) (34)	A not B task												
			**2065**	**3801**		**I^2 = 0.00, Tau^2 = 0.00**			**757**	**1413**		**I^2 = 0.00, Tau^2 = 0.00**		
**Fixed**						**−0.01 (−0.03, 0.00)**						**−0.01 (−0.04, 0.02)**		
**Random**						**−0.01 (−0.03, 0.00)**						**−0.01 (−0.04, 0.02)**		

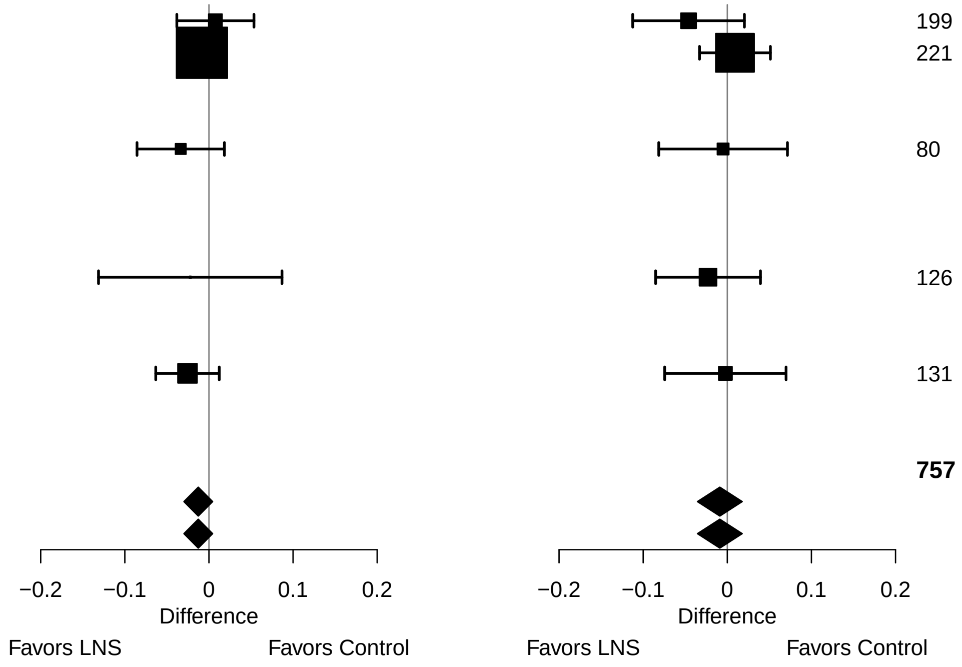

Supplemental figure 8N: Executive function lowest decile prevalence difference

8N3: Stratified by Household source water quality (insufficient comparisons)

Supplemental figure 8N: Executive function lowest decile prevalence difference

8N4: Stratified by Household sanitation (insufficient comparisons)

Supplemental figure 8N: Executive function lowest decile prevalence difference

8N5: Stratified by Home environment

P−for−interaction = 0.441
Difference in PDs = 0.01 (−0.02, 0.05)

Country	Trial	Tool	LNS N	Control N	Control Prevalence	At least median PD (95% CI)	Fixed W	Random W	LNS N	Control N	Control Prevalence	Less than median PD (95% CI)	Fixed W	Random W
Bangladesh	JiVitA−4 (21)													
Bangladesh	RDNS (22)	A not B task	385	195	11.3	−0.04 (−0.08, 0.01)	0.17	0.17	146	70	10.0	0.05 (−0.02, 0.13)	0.12	0.12
Bangladesh	WASH−B (23)	A not B task	623	1638	7.6	0.00 (−0.02, 0.02)	0.63	0.63	474	1688	12.6	0.00 (−0.03, 0.03)	0.67	0.67
Burkina Faso	iLiNS−Zinc (24)													
Ghana	GHANA (25)													
Ghana	iLiNS−DYAD−G (26)	A not B task	185	363	11.8	−0.04 (−0.09, 0.02)	0.11	0.11	105	213	9.9	0.00 (−0.07, 0.07)	0.14	0.14
Haiti	HAITI (27)													
Kenya	WASH−B (28)													
Madagascar	MAHAY (29)													
Malawi	iLiNS−DYAD−M (30)	A not B task	120	254	8.7	−0.01 (−0.07, 0.05)	0.09	0.09	62	115	15.7	−0.04 (−0.15, 0.06)	0.06	0.06
Malawi	iLiNS−DOSE (31)	A not B task												
Mali	PROMIS CS (32)													
Zimbabwe	SHINE (HIV−) (33)	A not B task												
Zimbabwe	SHINE (HIV+) (34)	A not B task												
			**1313**	**2450**		**I² = 0.00, Tau² = 0.00**			**787**	**2086**		**I² = 0.00, Tau² = 0.00**		
**Fixed**						**−0.01 (−0.03, 0.01)**						**0.00 (−0.02, 0.03)**		
**Random**						**−0.01 (−0.03, 0.01)**						**0.00 (−0.02, 0.03)**		

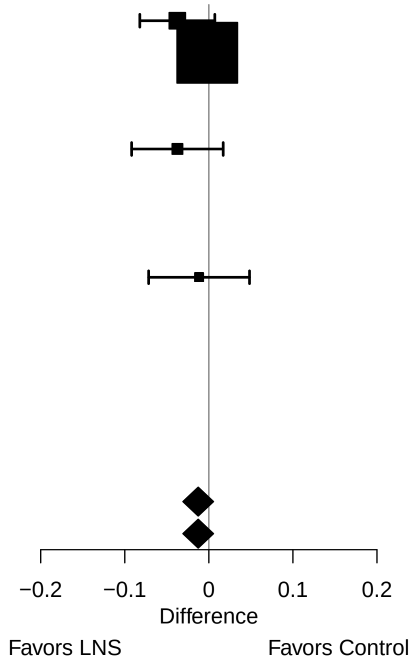

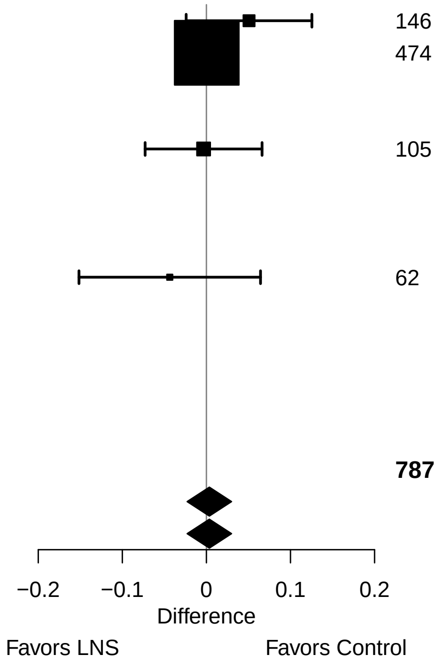

Supplemental figure 8N: Executive function lowest decile prevalence difference

8N6: Stratified by Season at the time of assessment

P−for−interaction = 0.752
Difference in PDs = 0.01 (−0.03, 0.04)

Country	Trial	Tool	Dry LNS N	Dry Control N	Dry Control Prevalence	Dry PD (95% CI)	Dry Fixed W	Dry Random W	Rainy LNS N	Rainy Control N	Rainy Control Prevalence	Rainy PD (95% CI)	Rainy Fixed W	Rainy Random W
Bangladesh	JiVitA−4 (21)													
Bangladesh	RDNS (22)	A not B task	242	120	11.7	−0.02 (−0.09, 0.05)	0.09	0.09	291	146	10.3	−0.01 (−0.06, 0.05)	0.21	0.21
Bangladesh	WASH−B (23)	A not B task	620	1921	10.2	0.00 (−0.03, 0.02)	0.60	0.60	477	1406	10.1	0.00 (−0.04, 0.03)	0.56	0.56
Burkina Faso	iLiNS−Zinc (24)													
Ghana	GHANA (25)													
Ghana	iLiNS−DYAD−G (26)	A not B task												
Haiti	HAITI (27)													
Kenya	WASH−B (28)													
Madagascar	MAHAY (29)													
Malawi	iLiNS−DYAD−M (30)	A not B task												
Malawi	iLiNS−DOSE (31)	A not B task												
Mali	PROMIS CS (32)													
Zimbabwe	SHINE (HIV−) (33)	A not B task	563	519	12.5	−0.03 (−0.07, 0.01)	0.32	0.32	224	220	9.1	−0.02 (−0.06, 0.03)	0.23	0.23
Zimbabwe	SHINE (HIV+) (34)	A not B task												
			**1425**	**2560**		**I² = 0.00, Tau² = 0.00**			**992**	**1772**		**I² = 0.00, Tau² = 0.00**		
**Fixed**						**−0.01 (−0.03, 0.01)**						**−0.01 (−0.03, 0.02)**		
**Random**						**−0.01 (−0.03, 0.01)**						**−0.01 (−0.03, 0.02)**		

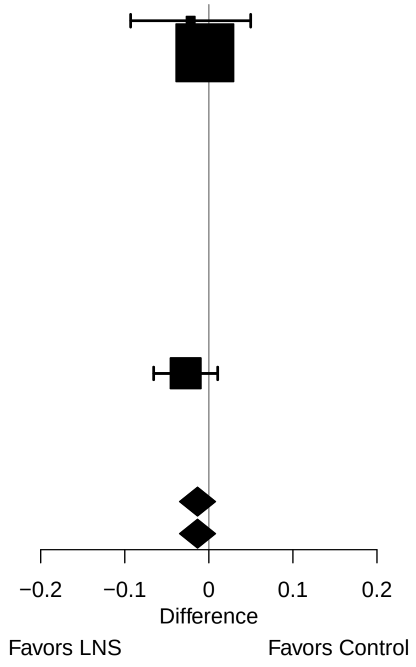

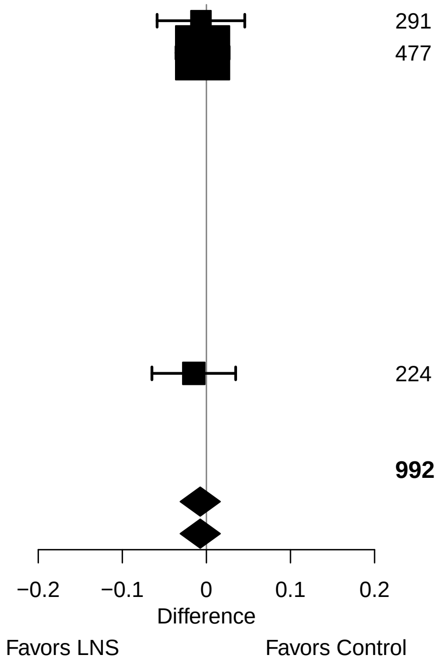

Supplemental figure 8O: 12-mo walking without support prevalence ratio

8O1: Stratified by Household socio-economic status

P−for−interaction = 0.721
Ratio of PRs = 1.01 (0.94, 1.10)

At least median

Country	Trial	LNS N	Control N	Control Prevalence	PR (95% CI)	Fixed W	Random W
Bangladesh	JiVitA−4 (21)	1516	692	42.6	1.03 (0.95, 1.11)	0.50	0.22
Bangladesh	RDNS (22)	811	396	72.0	1.22 (1.01, 1.48)	0.08	0.13
Bangladesh	WASH−B (23)	247	735	70.3	1.28 (1.06, 1.56)	0.08	0.13
Burkina Faso	iLiNS−Zinc (24)						
Ghana	GHANA (25)						
Ghana	iLiNS−DYAD−G (26)	151	349	49.9	1.11 (0.93, 1.32)	0.10	0.14
Haiti	HAITI (27)	44	31	54.8	0.91 (0.54, 1.53)	0.01	0.03
Kenya	WASH−B (28)	240	913	59.3	0.95 (0.81, 1.12)	0.12	0.15
Madagascar	MAHAY (29)						
Malawi	iLiNS−DYAD−M (30)	117	227	49.8	1.24 (1.03, 1.50)	0.08	0.13
Malawi	iLiNS−DOSE (31)	247	81	74.1	1.67 (1.13, 2.48)	0.02	0.05
Mali	PROMIS CS (32)	67	76	73.7	0.91 (0.47, 1.76)	0.01	0.02
Zimbabwe	SHINE (HIV−) (33)						
Zimbabwe	SHINE (HIV+) (34)						
		**3440**	**3500**		**I² = 0.50, Tau² = 0.01**		
**Fixed**					**1.08 (1.03, 1.14)**		
**Random**					**1.13 (1.02, 1.25)**		

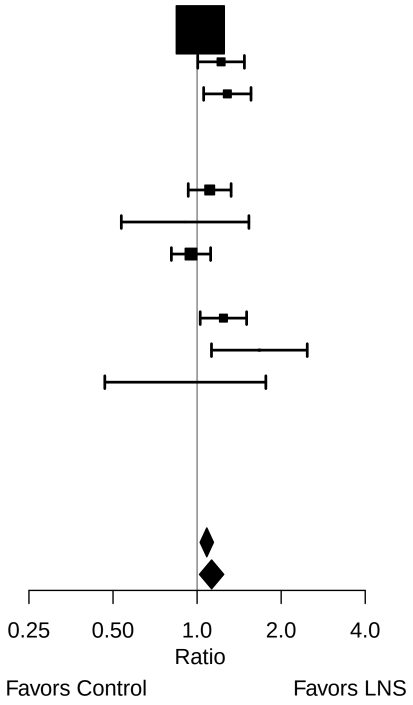

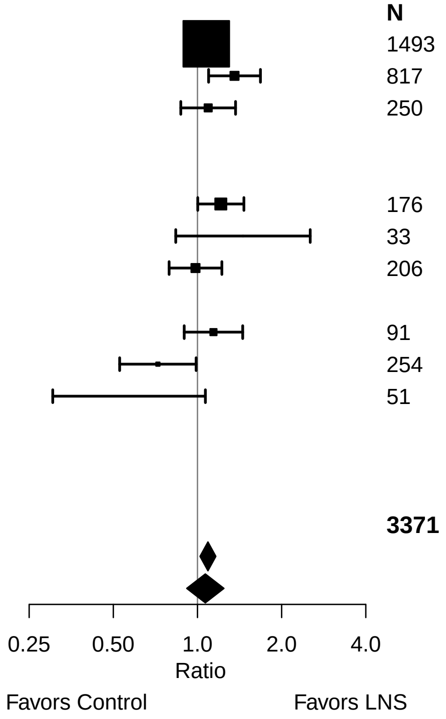

Less than median

Country	Trial	LNS N	Control N	Control Prevalence	PR (95% CI)	Fixed W	Random W
Bangladesh	JiVitA−4 (21)	1493	671	49.5	1.08 (0.98, 1.18)	0.48	0.16
Bangladesh	RDNS (22)	817	394	76.9	1.36 (1.10, 1.68)	0.09	0.13
Bangladesh	WASH−B (23)	250	739	78.8	1.09 (0.87, 1.37)	0.08	0.13
Burkina Faso	iLiNS−Zinc (24)						
Ghana	GHANA (25)						
Ghana	iLiNS−DYAD−G (26)	176	313	56.9	1.21 (1.00, 1.47)	0.12	0.14
Haiti	HAITI (27)	33	42	66.7	1.45 (0.84, 2.53)	0.01	0.05
Kenya	WASH−B (28)	206	689	60.5	0.98 (0.79, 1.22)	0.09	0.13
Madagascar	MAHAY (29)						
Malawi	iLiNS−DYAD−M (30)	91	197	52.8	1.14 (0.90, 1.45)	0.07	0.12
Malawi	iLiNS−DOSE (31)	254	88	59.1	0.72 (0.53, 0.99)	0.04	0.10
Mali	PROMIS CS (32)	51	75	58.7	0.57 (0.30, 1.07)	0.01	0.04
Zimbabwe	SHINE (HIV−) (33)						
Zimbabwe	SHINE (HIV+) (34)						
		**3371**	**3208**		**I² = 0.56, Tau² = 0.04**		
**Fixed**					**1.09 (1.02, 1.16)**		
**Random**					**1.07 (0.92, 1.24)**		

Supplemental figure 8O: 12-mo walking without support prevalence ratio

8O2: Stratified by Household food insecurity

P−for−interaction = 0.812
Ratio of PRs = 0.99 (0.90, 1.08)

		Mild to secure						Moderate to severe					
Country	Trial	LNS N	Control N	Control Prevalence	PR (95% CI)	Fixed W	Random W	LNS N	Control N	Control Prevalence	PR (95% CI)	Fixed W	Random W
Bangladesh	JiVitA−4 (21)	2132	947	44.9	1.05 (0.97, 1.12)	0.53	0.30	877	416	48.6	1.05 (0.94, 1.17)	0.47	0.25
Bangladesh	RDNS (22)	1034	483	72.9	1.19 (0.97, 1.48)	0.06	0.11	594	307	76.9	1.44 (1.16, 1.80)	0.11	0.16
Bangladesh	WASH−B (23)	398	1144	73.3	1.27 (1.08, 1.48)	0.11	0.16	99	330	79.1	0.82 (0.56, 1.20)	0.04	0.08
Burkina Faso	iLiNS−Zinc (24)												
Ghana	GHANA (25)												
Ghana	iLiNS−DYAD−G (26)	230	454	52.4	1.16 (1.00, 1.35)	0.12	0.17	96	206	54.9	1.11 (0.86, 1.42)	0.09	0.14
Haiti	HAITI (27)												
Kenya	WASH−B (28)	400	1448	59.0	0.96 (0.84, 1.11)	0.14	0.19	46	153	66.7	0.98 (0.60, 1.59)	0.02	0.05
Madagascar	MAHAY (29)												
Malawi	iLiNS−DYAD−M (30)	64	112	56.2	1.21 (0.89, 1.66)	0.03	0.06	142	307	48.5	1.19 (1.00, 1.41)	0.19	0.20
Malawi	iLiNS−DOSE (31)	120	48	70.8	1.43 (0.88, 2.33)	0.01	0.03	371	118	61.9	0.94 (0.72, 1.23)	0.08	0.13
Mali	PROMIS CS (32)												
Zimbabwe	SHINE (HIV−) (33)												
Zimbabwe	SHINE (HIV+) (34)												
		**4378**	**4636**		**I² = 0.45, Tau² = 0.00**			**2225**	**1837**		**I² = 0.46, Tau² = 0.01**		
**Fixed**					**1.09 (1.03, 1.14)**						**1.10 (1.02, 1.18)**		
**Random**					**1.12 (1.03, 1.21)**						**1.10 (0.97, 1.24)**		

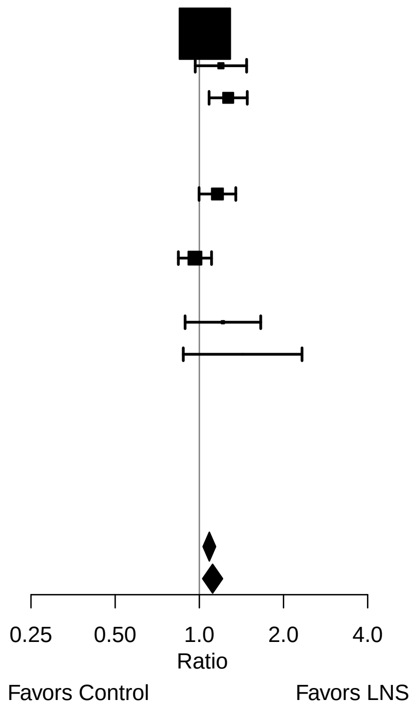

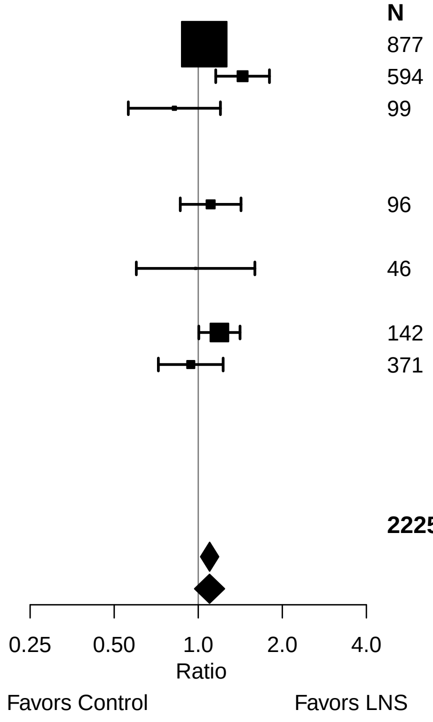

Supplemental figure 8O: 12-mo walking without support prevalence ratio

8O3: Stratified by Household source water quality

P−for−interaction = 0.190
Ratio of PRs = 1.17 (0.92, 1.48)

Country	Trial	Improved LNS N	Improved Control N	Improved Control Prevalence	Improved PR (95% CI)	Improved Fixed W	Improved Random W	Unimproved LNS N	Unimproved Control N	Unimproved Control Prevalence	Unimproved PR (95% CI)	Unimproved Fixed W	Unimproved Random W
Bangladesh	JiVitA−4 (21)												
Bangladesh	RDNS (22)												
Bangladesh	WASH−B (23)	201	428	74.5	1.17 (0.91, 1.51)	0.17	0.17	38	56	76.8	1.36 (0.75, 2.48)	0.12	0.18
Burkina Faso	iLiNS−Zinc (24)												
Ghana	GHANA (25)												
Ghana	iLiNS−DYAD−G (26)												
Haiti	HAITI (27)												
Kenya	WASH−B (28)	118	469	58.2	0.89 (0.68, 1.16)	0.16	0.16	84	234	60.3	1.17 (0.89, 1.53)	0.57	0.34
Madagascar	MAHAY (29)												
Malawi	iLiNS−DYAD−M (30)	190	395	50.9	1.17 (1.00, 1.37)	0.44	0.44	18	28	53.6	1.56 (0.95, 2.54)	0.17	0.22
Malawi	iLiNS−DOSE (31)	470	157	66.2	1.09 (0.85, 1.40)	0.18	0.18	42	15	60.0	0.95 (0.46, 1.98)	0.08	0.14
Mali	PROMIS CS (32)	65	86	68.6	0.88 (0.56, 1.40)	0.05	0.05	48	63	61.9	0.49 (0.22, 1.12)	0.06	0.12
Zimbabwe	SHINE (HIV−) (33)												
Zimbabwe	SHINE (HIV+) (34)												
		**1044**	**1535**		**I² = 0.01, Tau² = 0.00**			**230**	**396**		**I² = 0.34, Tau² = 0.07**		
**Fixed**					**1.09 (0.98, 1.21)**						**1.17 (0.95, 1.43)**		
**Random**					**1.09 (0.98, 1.21)**						**1.12 (0.80, 1.57)**		

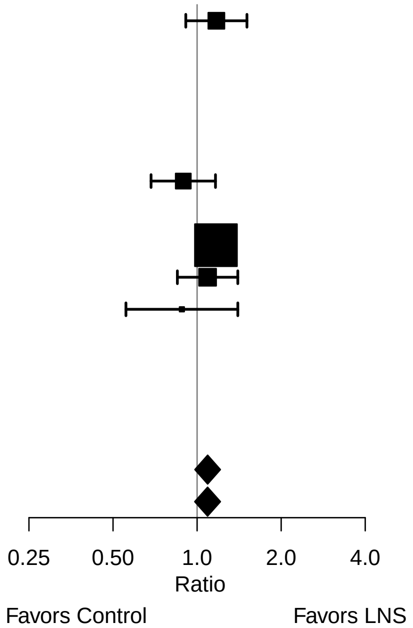

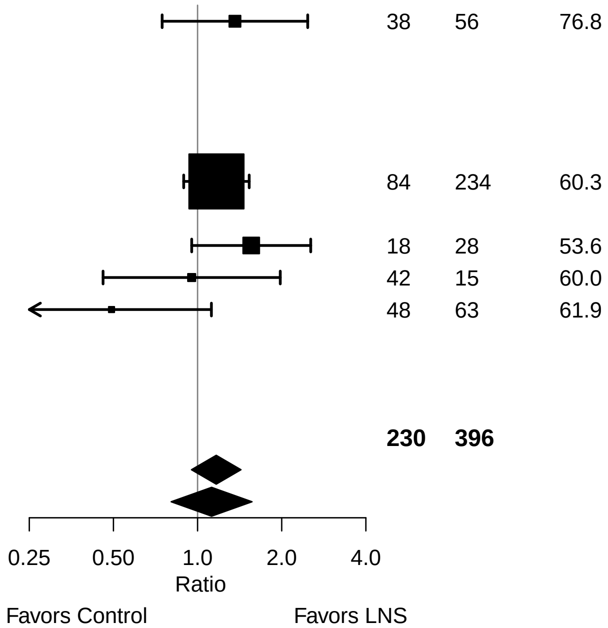

Supplemental figure 8O: 12-mo walking without support prevalence ratio

8O4: Stratified by Household sanitation

P−for−interaction = 0.336
Ratio of PRs = 0.94 (0.84, 1.06)

Country	Trial	Improved LNS N	Improved Control N	Improved Control Prevalence	Improved PR (95% CI)	Improved Fixed W	Improved Random W	Unimproved LNS N	Unimproved Control N	Unimproved Control Prevalence	Unimproved PR (95% CI)	Unimproved Fixed W	Unimproved Random W
Bangladesh	JiVitA−4 (21)	2335	1047	46.0	1.07 (1.00, 1.15)	0.85	0.35	674	316	45.9	0.98 (0.87, 1.11)	0.46	0.34
Bangladesh	RDNS (22)	1149	563	73.4	1.27 (1.02, 1.59)	0.09	0.26	477	226	77.0	1.29 (1.00, 1.67)	0.11	0.17
Bangladesh	WASH−B (23)												
Burkina Faso	iLiNS−Zinc (24)												
Ghana	GHANA (25)												
Ghana	iLiNS−DYAD−G (26)												
Haiti	HAITI (27)												
Kenya	WASH−B (28)	35	106	58.5	1.17 (0.72, 1.89)	0.02	0.13	167	598	59.0	0.96 (0.77, 1.20)	0.14	0.20
Madagascar	MAHAY (29)												
Malawi	iLiNS−DYAD−M (30)	19	41	43.9	0.94 (0.57, 1.56)	0.02	0.12	189	382	51.8	1.23 (1.05, 1.44)	0.28	0.28
Malawi	iLiNS−DOSE (31)												
Mali	PROMIS CS (32)	87	108	63.0	0.65 (0.42, 1.00)	0.02	0.14	27	38	73.7	0.84 (0.28, 2.57)	0.01	0.01
Zimbabwe	SHINE (HIV−) (33)												
Zimbabwe	SHINE (HIV+) (34)												
		**3625**	**1865**		**I² = 0.49, Tau² = 0.03**			**1534**	**1560**		**I² = 0.51, Tau² = 0.01**		
**Fixed**					**1.07 (1.00, 1.15)**						**1.07 (0.99, 1.16)**		
**Random**					**1.04 (0.84, 1.28)**						**1.09 (0.96, 1.24)**		

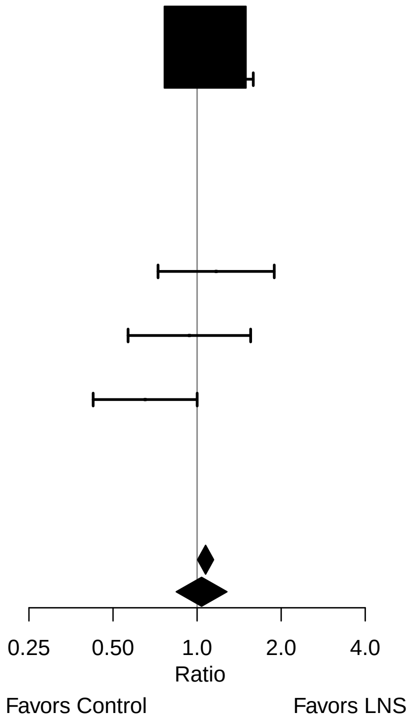

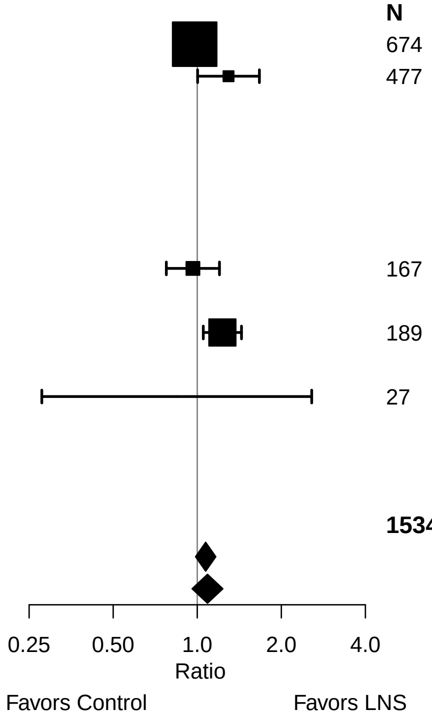

Supplemental figure 8O: 12-mo walking without support prevalence ratio

8O5: Stratified by Home environment

P−for−interaction = 0.458
Ratio of PRs = 0.95 (0.84, 1.08)

Country	Trial	LNS N	Control N	Control Prevalence	At least median PR (95% CI)	Fixed W	Random W	LNS N	Control N	Control Prevalence	Less than median PR (95% CI)	Fixed W	Random W
Bangladesh	JiVitA−4 (21)												
Bangladesh	RDNS (22)	1158	560	74.5	1.34 (1.09, 1.64)	0.13	0.15	448	217	74.7	1.18 (0.90, 1.55)	0.15	0.15
Bangladesh	WASH−B (23)	243	689	73.9	1.32 (1.10, 1.58)	0.17	0.18	231	717	75.2	1.12 (0.90, 1.38)	0.24	0.24
Burkina Faso	iLiNS−Zinc (24)												
Ghana	GHANA (25)												
Ghana	iLiNS−DYAD−G (26)	198	373	50.4	1.16 (0.99, 1.36)	0.23	0.20	110	233	57.5	1.07 (0.83, 1.38)	0.17	0.17
Haiti	HAITI (27)												
Kenya	WASH−B (28)	248	834	55.6	0.93 (0.79, 1.09)	0.23	0.20	164	608	65.5	1.09 (0.87, 1.38)	0.20	0.20
Madagascar	MAHAY (29)												
Malawi	iLiNS−DYAD−M (30)	130	278	49.3	1.17 (0.97, 1.40)	0.17	0.17	72	136	55.1	1.30 (0.99, 1.70)	0.15	0.15
Malawi	iLiNS−DOSE (31)	306	109	65.1	1.15 (0.86, 1.54)	0.07	0.10	224	67	59.7	0.84 (0.60, 1.19)	0.09	0.09
Mali	PROMIS CS (32)												
Zimbabwe	SHINE (HIV−) (33)												
Zimbabwe	SHINE (HIV+) (34)												
		**2283**	**2843**		**I² = 0.56, Tau² = 0.01**			**1249**	**1978**		**I² = 0.00, Tau² = 0.00**		
**Fixed**					**1.15 (1.07, 1.24)**						**1.11 (1.00, 1.23)**		
**Random**					**1.16 (1.04, 1.30)**						**1.11 (1.00, 1.23)**		

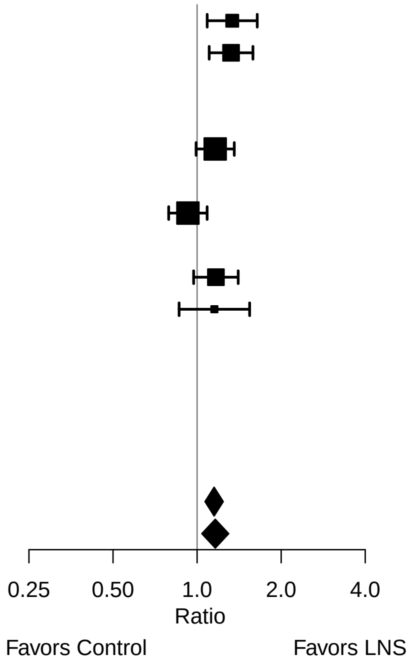

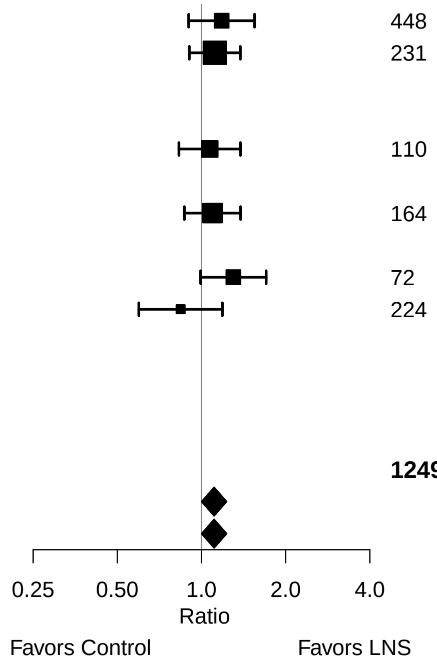

Supplemental figure 8O: 12-mo walking without support prevalence ratio

8O6: Stratified by Season at the time of assessment

P−for−interaction = 0.845
Ratio of PRs = 0.99 (0.88, 1.11)

Country	Trial	Dry: LNS N	Dry: Control N	Dry: Control Prevalence	Dry: PR (95% CI)	Dry: Fixed W	Dry: Random W	Rainy: LNS N	Rainy: Control N	Rainy: Control Prevalence	Rainy: PR (95% CI)	Rainy: Fixed W	Rainy: Random W
Bangladesh	JiVitA−4 (21)												
Bangladesh	RDNS (22)	899	430	77.4	1.28 (1.04, 1.59)	0.15	0.16	729	360	70.8	1.28 (1.03, 1.60)	0.14	0.14
Bangladesh	WASH−B (23)	267	774	72.6	1.19 (0.99, 1.43)	0.19	0.18	230	700	76.7	1.21 (0.99, 1.49)	0.17	0.17
Burkina Faso	iLiNS−Zinc (24)												
Ghana	GHANA (25)	53	51	78.4	2.01 (1.10, 3.69)	0.02	0.03	37	36	69.4	1.33 (0.71, 2.49)	0.02	0.02
Ghana	iLiNS−DYAD−G (26)	163	320	48.4	1.15 (0.98, 1.36)	0.25	0.21	164	343	57.4	1.13 (0.93, 1.38)	0.17	0.17
Haiti	HAITI (27)												
Kenya	WASH−B (28)	131	461	62.5	0.92 (0.69, 1.22)	0.08	0.11	316	1143	58.6	0.99 (0.86, 1.13)	0.36	0.36
Madagascar	MAHAY (29)												
Malawi	iLiNS−DYAD−M (30)	150	311	53.7	1.24 (1.03, 1.49)	0.20	0.19	58	115	44.3	1.12 (0.86, 1.45)	0.10	0.10
Malawi	iLiNS−DOSE (31)	437	152	65.8	1.04 (0.81, 1.34)	0.10	0.13	165	48	64.6	1.04 (0.68, 1.61)	0.04	0.04
Mali	PROMIS CS (32)												
Zimbabwe	SHINE (HIV−) (33)												
Zimbabwe	SHINE (HIV+) (34)												
		**2100**	**2499**		**I² = 0.23, Tau² = 0.01**			**1699**	**2745**		**I² = 0.00, Tau² = 0.00**		
**Fixed**					**1.17 (1.08, 1.27)**						**1.11 (1.02, 1.20)**		
**Random**					**1.17 (1.05, 1.31)**						**1.11 (1.02, 1.20)**		

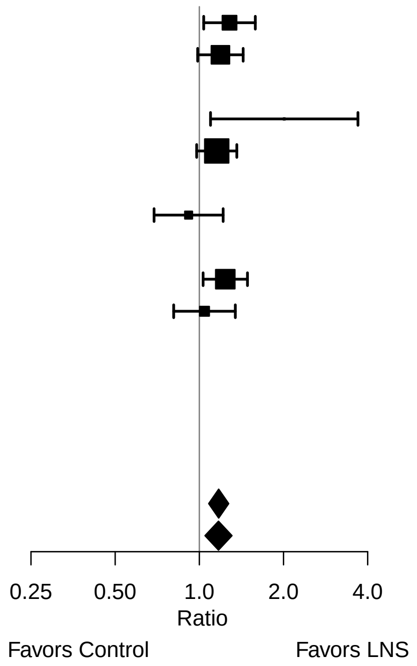

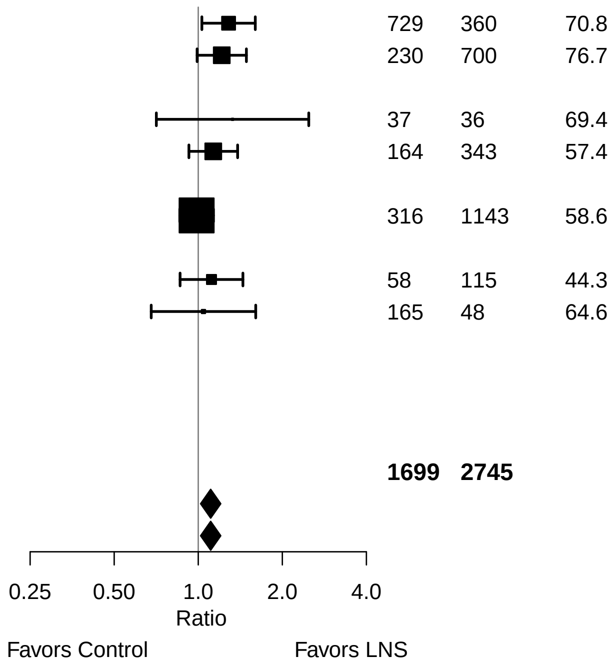

Supplemental figure 8P: 12-mo walking without support prevalence difference

8P1: Stratified by Household socio-economic status

P−for−interaction = 0.521
Difference in PDs = −0.01 (−0.05, 0.02)

		At least median							Less than median						
**Country**	**Trial**	**LNS N**	**Control N**	**Control Prevalence**	**PD (95% CI)**	**Fixed W**	**Random W**	**LNS N**	**Control N**	**Control Prevalence**	**PD (95% CI)**	**Fixed W**	**Random W**
Bangladesh	JiVitA−4 (21)	1516	692	42.6	0.02 (−0.03, 0.06)	0.32	0.19	1493	671	49.5	0.04 (−0.01, 0.09)	0.26	0.15
Bangladesh	RDNS (22)	811	396	72.0	0.06 (0.00, 0.12)	0.19	0.17	817	394	76.9	0.08 (0.03, 0.13)	0.23	0.15
Bangladesh	WASH−B (23)	247	735	70.3	0.08 (0.01, 0.15)	0.13	0.14	250	739	78.8	0.02 (−0.03, 0.07)	0.24	0.15
Burkina Faso	iLiNS−Zinc (24)												
Ghana	GHANA (25)												
Ghana	iLiNS−DYAD−G (26)	151	349	49.9	0.05 (−0.04, 0.15)	0.07	0.10	176	313	56.9	0.09 (0.00, 0.18)	0.07	0.12
Haiti	HAITI (27)	44	31	54.8	−0.04 (−0.27, 0.19)	0.01	0.03	33	42	66.7	0.15 (−0.07, 0.38)	0.01	0.05
Kenya	WASH−B (28)	240	913	59.3	−0.02 (−0.08, 0.04)	0.16	0.16	206	689	60.5	−0.01 (−0.09, 0.08)	0.09	0.12
Madagascar	MAHAY (29)												
Malawi	iLiNS−DYAD−M (30)	117	227	49.8	0.12 (0.01, 0.23)	0.05	0.09	91	197	52.8	0.07 (−0.06, 0.19)	0.04	0.09
Malawi	iLiNS−DOSE (31)	247	81	74.1	0.17 (0.05, 0.30)	0.04	0.08	254	88	59.1	−0.11 (−0.23, 0.00)	0.05	0.10
Mali	PROMIS CS (32)	67	76	73.7	−0.02 (−0.19, 0.14)	0.02	0.05	51	75	58.7	−0.18 (−0.36, 0.00)	0.02	0.06
Zimbabwe	SHINE (HIV−) (33)												
Zimbabwe	SHINE (HIV+) (34)												
		**3440**	**3500**		**I² = 0.45, Tau² = 0.00**			**3371**	**3208**		**I² = 0.59, Tau² = 0.00**		
**Fixed**					**0.04 (0.02, 0.07)**						**0.03 (0.01, 0.06)**		
**Random**					**0.05 (0.01, 0.09)**						**0.02 (−0.03, 0.08)**		

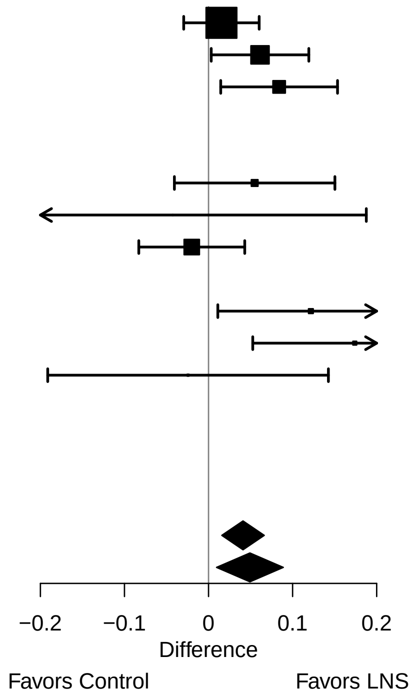

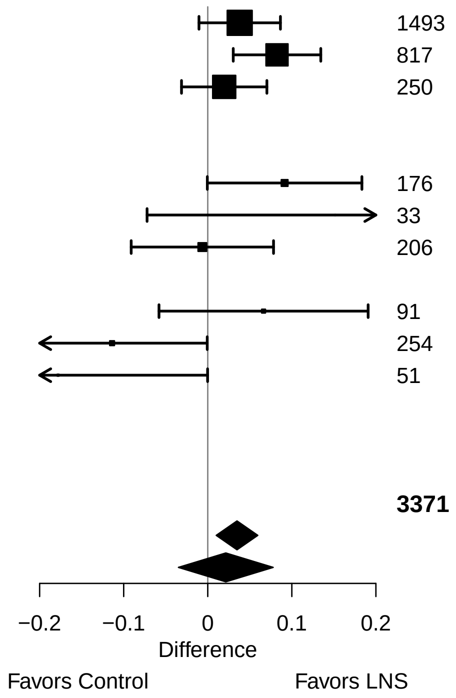

Supplemental figure 8P: 12-mo walking without support prevalence difference

8P2: Stratified by Household food insecurity

P−for−interaction = 0.547
Difference in PDs = −0.01 (−0.05, 0.03)

Country	Trial	LNS N	Control N	Control Prevalence	Mild to secure PD (95% CI)	Fixed W	Random W	LNS N	Control N	Control Prevalence	Moderate to severe PD (95% CI)	Fixed W	Random W
Bangladesh	JiVitA−4 (21)	2132	947	44.9	0.03 (−0.01, 0.07)	0.33	0.27	877	416	48.6	0.03 (−0.03, 0.08)	0.27	0.21
Bangladesh	RDNS (22)	1034	483	72.9	0.05 (−0.01, 0.11)	0.15	0.16	594	307	76.9	0.10 (0.04, 0.16)	0.27	0.21
Bangladesh	WASH−B (23)	398	1144	73.3	0.07 (0.02, 0.12)	0.22	0.21	99	330	79.1	−0.04 (−0.11, 0.03)	0.19	0.18
Burkina Faso	iLiNS−Zinc (24)												
Ghana	GHANA (25)												
Ghana	iLiNS−DYAD−G (26)	230	454	52.4	0.08 (0.00, 0.16)	0.08	0.11	96	206	54.9	0.05 (−0.07, 0.17)	0.06	0.09
Haiti	HAITI (27)												
Kenya	WASH−B (28)	400	1448	59.0	−0.01 (−0.07, 0.04)	0.18	0.19	46	153	66.7	−0.01 (−0.17, 0.15)	0.03	0.06
Madagascar	MAHAY (29)												
Malawi	iLiNS−DYAD−M (30)	64	112	56.2	0.09 (−0.06, 0.25)	0.02	0.03	142	307	48.5	0.10 (0.00, 0.20)	0.09	0.12
Malawi	iLiNS−DOSE (31)	120	48	70.8	0.12 (−0.04, 0.29)	0.02	0.03	371	118	61.9	−0.02 (−0.12, 0.08)	0.09	0.12
Mali	PROMIS CS (32)												
Zimbabwe	SHINE (HIV−) (33)												
Zimbabwe	SHINE (HIV+) (34)												
		**4378**	**4636**		**I² = 0.29, Tau² = 0.00**			**2225**	**1837**		**I² = 0.53, Tau² = 0.00**		
**Fixed**					**0.04 (0.02, 0.06)**						**0.04 (0.01, 0.07)**		
**Random**					**0.04 (0.01, 0.07)**						**0.03 (−0.01, 0.08)**		

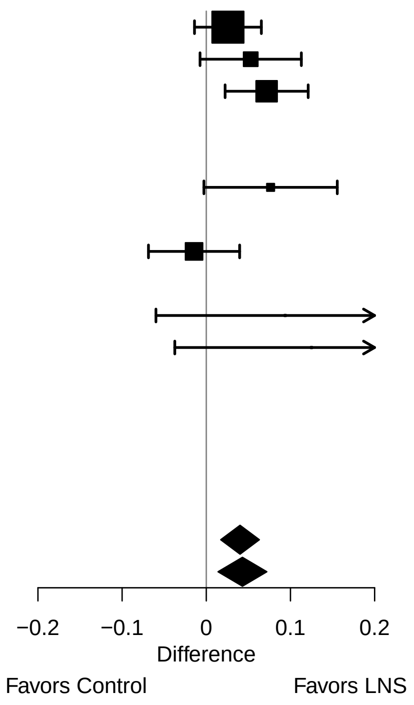

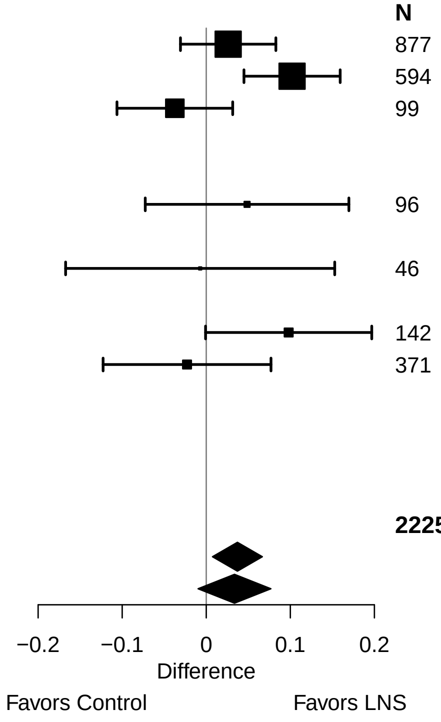

Supplemental figure 8P: 12-mo walking without support prevalence difference

8P3: Stratified by Household source water quality

P−for−interaction = 0.476
Difference in PDs = 0.03 (−0.06, 0.13)

Country	Trial	Improved LNS N	Improved Control N	Improved Control Prevalence	Improved PD (95% CI)	Improved Fixed W	Improved Random W	Unimproved LNS N	Unimproved Control N	Unimproved Control Prevalence	Unimproved PD (95% CI)	Unimproved Fixed W	Unimproved Random W
Bangladesh	JiVitA−4 (21)												
Bangladesh	RDNS (22)												
Bangladesh	WASH−B (23)	201	428	74.5	0.04 (−0.03, 0.11)	0.32	0.30	38	56	76.8	0.08 (−0.07, 0.23)	0.27	0.26
Burkina Faso	iLiNS−Zinc (24)												
Ghana	GHANA (25)												
Ghana	iLiNS−DYAD−G (26)												
Haiti	HAITI (27)												
Kenya	WASH−B (28)	118	469	58.2	−0.05 (−0.15, 0.06)	0.16	0.17	84	234	60.3	0.07 (−0.05, 0.19)	0.45	0.30
Madagascar	MAHAY (29)												
Malawi	iLiNS−DYAD−M (30)	190	395	50.9	0.08 (0.00, 0.17)	0.22	0.22	18	28	53.6	0.26 (−0.03, 0.55)	0.07	0.13
Malawi	iLiNS−DOSE (31)	470	157	66.2	0.03 (−0.06, 0.12)	0.21	0.21	42	15	60.0	−0.02 (−0.31, 0.27)	0.07	0.13
Mali	PROMIS CS (32)	65	86	68.6	−0.04 (−0.17, 0.10)	0.09	0.10	48	63	61.9	−0.19 (−0.41, 0.03)	0.13	0.19
Zimbabwe	SHINE (HIV−) (33)												
Zimbabwe	SHINE (HIV+) (34)												
		**1044**	**1535**		**I^2 = 0.14, Tau^2 = 0.00**			**230**	**396**		**I^2 = 0.45, Tau^2 = 0.01**		
**Fixed**					**0.03 (−0.01, 0.07)**						**0.05 (−0.03, 0.12)**		
**Random**					**0.03 (−0.02, 0.07)**						**0.04 (−0.09, 0.17)**		

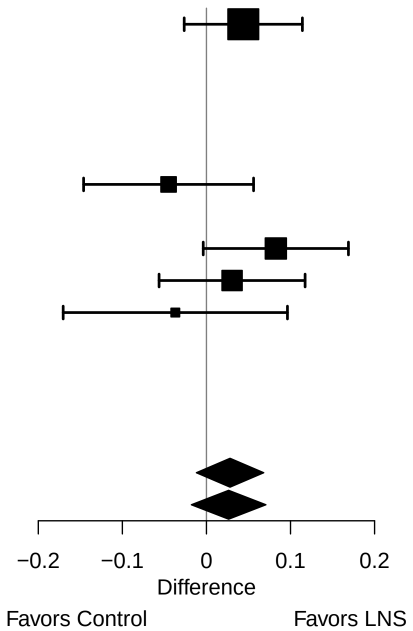

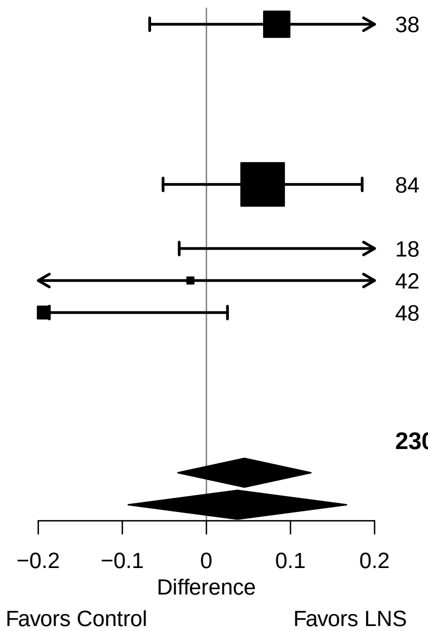

Supplemental figure 8P: 12-mo walking without support prevalence difference

8P4: Stratified by Household sanitation

P−for−interaction = 0.417
Difference in PDs = −0.02 (−0.07, 0.03)

Country	Trial	LNS N	Control N	Control Prevalence	Improved PD (95% CI)	Fixed W	Random W	LNS N	Control N	Control Prevalence	Unimproved PD (95% CI)	Fixed W	Random W
Bangladesh	JiVitA−4 (21)	2335	1047	46.0	0.04 (0.00, 0.08)	0.64	0.36	674	316	45.9	−0.01 (−0.08, 0.05)	0.30	0.27
Bangladesh	RDNS (22)	1149	563	73.4	0.07 (0.01, 0.13)	0.26	0.31	477	226	77.0	0.07 (0.00, 0.13)	0.33	0.28
Bangladesh	WASH−B (23)												
Burkina Faso	iLiNS−Zinc (24)												
Ghana	GHANA (25)												
Ghana	iLiNS−DYAD−G (26)												
Haiti	HAITI (27)												
Kenya	WASH−B (28)	35	106	58.5	0.07 (−0.15, 0.29)	0.02	0.09	167	598	59.0	−0.01 (−0.10, 0.07)	0.17	0.20
Madagascar	MAHAY (29)												
Malawi	iLiNS−DYAD−M (30)	19	41	43.9	−0.03 (−0.31, 0.24)	0.01	0.06	189	382	51.8	0.11 (0.02, 0.20)	0.17	0.21
Malawi	iLiNS−DOSE (31)												
Mali	PROMIS CS (32)	87	108	63.0	−0.13 (−0.25, −0.01)	0.07	0.19	27	38	73.7	−0.04 (−0.30, 0.22)	0.02	0.04
Zimbabwe	SHINE (HIV−) (33)												
Zimbabwe	SHINE (HIV+) (34)												
		**3625**	**1865**		**I² = 0.55, Tau² = 0.00**			**1534**	**1560**		**I² = 0.46, Tau² = 0.00**		
**Fixed**					**0.04 (0.00, 0.07)**						**0.04 (0.00, 0.07)**		
**Random**					**0.02 (−0.06, 0.09)**						**0.03 (−0.02, 0.09)**		

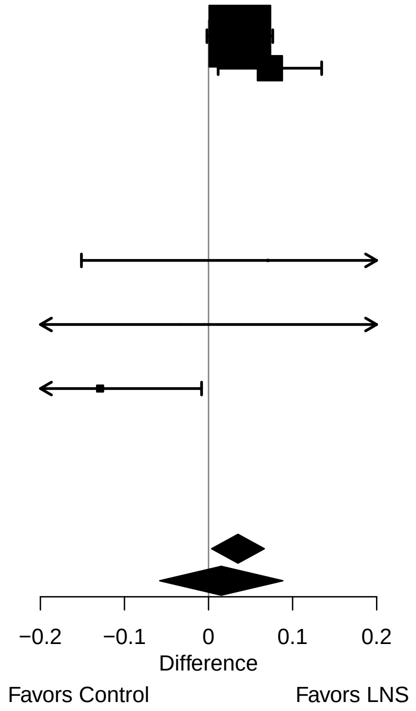

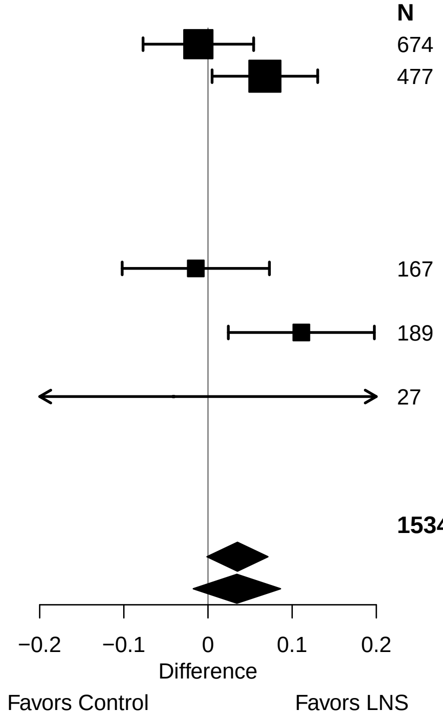

Supplemental figure 8P: 12-mo walking without support prevalence difference

8P5: Stratified by Home environment

P−for−interaction = 0.302
Difference in PDs = −0.02 (−0.07, 0.02)

Country	Trial	At least median: LNS N	Control N	Control Prevalence	PD (95% CI)	Fixed W	Random W	Less than median: LNS N	Control N	Control Prevalence	PD (95% CI)	Fixed W	Random W
Bangladesh	JiVitA−4 (21)												
Bangladesh	RDNS (22)	1158	560	74.5	0.09 (0.03, 0.14)	0.26	0.22	448	217	74.7	0.05 (−0.03, 0.12)	0.23	0.23
Bangladesh	WASH−B (23)	243	689	73.9	0.08 (0.03, 0.14)	0.26	0.22	231	717	75.2	0.03 (−0.03, 0.09)	0.38	0.38
Burkina Faso	iLiNS−Zinc (24)												
Ghana	GHANA (25)												
Ghana	iLiNS−DYAD−G (26)	198	373	50.4	0.08 (−0.01, 0.17)	0.12	0.14	110	233	57.5	0.03 (−0.08, 0.14)	0.10	0.10
Haiti	HAITI (27)												
Kenya	WASH−B (28)	248	834	55.6	−0.03 (−0.10, 0.03)	0.20	0.20	164	608	65.5	0.03 (−0.05, 0.12)	0.17	0.17
Madagascar	MAHAY (29)												
Malawi	iLiNS−DYAD−M (30)	130	278	49.3	0.09 (−0.02, 0.19)	0.08	0.11	72	136	55.1	0.13 (−0.01, 0.28)	0.06	0.06
Malawi	iLiNS−DOSE (31)	306	109	65.1	0.05 (−0.05, 0.16)	0.08	0.10	224	67	59.7	−0.06 (−0.19, 0.07)	0.07	0.07
Mali	PROMIS CS (32)												
Zimbabwe	SHINE (HIV−) (33)												
Zimbabwe	SHINE (HIV+) (34)												
		**2283**	**2843**		**I^2 = 0.47, Tau^2 = 0.00**			**1249**	**1978**		**I^2 = 0.00, Tau^2 = 0.00**		
**Fixed**					**0.06 (0.03, 0.09)**						**0.03 (0.00, 0.07)**		
**Random**					**0.06 (0.02, 0.10)**						**0.03 (0.00, 0.07)**		

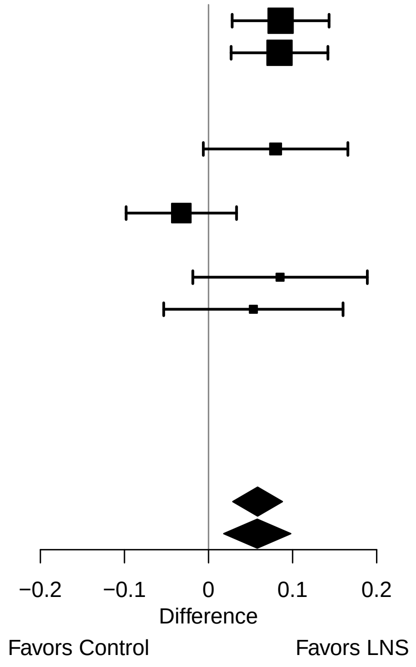

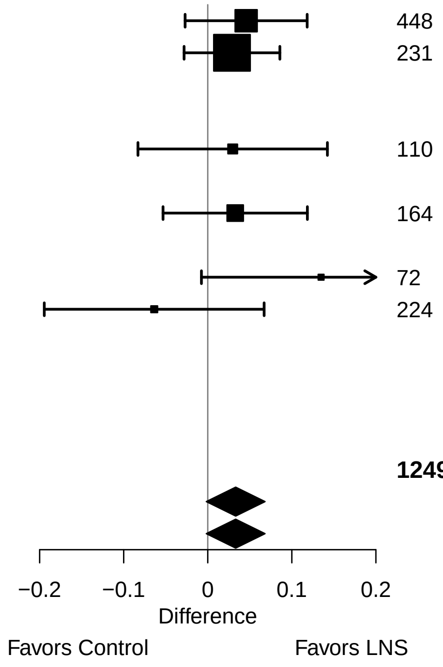

Supplemental figure 8P: 12-mo walking without support prevalence difference

8P6: Stratified by Season at the time of assessment

P−for−interaction = 0.935
Difference in PDs = 0.00 (−0.04, 0.05)

Country	Trial	Dry LNS N	Dry Control N	Dry Control Prevalence	Dry PD (95% CI)	Dry Fixed W	Dry Random W	Rainy LNS N	Rainy Control N	Rainy Control Prevalence	Rainy PD (95% CI)	Rainy Fixed W	Rainy Random W
Bangladesh	JiVitA−4 (21)												
Bangladesh	RDNS (22)	899	430	77.4	0.06 (0.01, 0.11)	0.34	0.23	729	360	70.8	0.08 (0.01, 0.15)	0.19	0.19
Bangladesh	WASH−B (23)	267	774	72.6	0.05 (−0.01, 0.11)	0.25	0.21	230	700	76.7	0.05 (0.00, 0.10)	0.31	0.31
Burkina Faso	iLiNS−Zinc (24)												
Ghana	GHANA (25)	53	51	78.4	0.22 (0.04, 0.40)	0.03	0.05	37	36	69.4	0.10 (−0.12, 0.32)	0.02	0.02
Ghana	iLiNS−DYAD−G (26)	163	320	48.4	0.08 (−0.01, 0.17)	0.10	0.13	164	343	57.4	0.06 (−0.04, 0.15)	0.11	0.11
Haiti	HAITI (27)												
Kenya	WASH−B (28)	131	461	62.5	−0.03 (−0.13, 0.07)	0.09	0.12	316	1143	58.6	−0.01 (−0.06, 0.05)	0.29	0.29
Madagascar	MAHAY (29)												
Malawi	iLiNS−DYAD−M (30)	150	311	53.7	0.11 (0.01, 0.21)	0.09	0.12	58	115	44.3	0.06 (−0.09, 0.22)	0.04	0.04
Malawi	iLiNS−DOSE (31)	437	152	65.8	0.01 (−0.07, 0.10)	0.11	0.14	165	48	64.6	0.02 (−0.14, 0.17)	0.04	0.04
Mali	PROMIS CS (32)												
Zimbabwe	SHINE (HIV−) (33)												
Zimbabwe	SHINE (HIV+) (34)												
		**2100**	**2499**		**I² = 0.31, Tau² = 0.00**			**1699**	**2745**		**I² = 0.00, Tau² = 0.00**		
**Fixed**					**0.06 (0.03, 0.09)**						**0.04 (0.01, 0.07)**		
**Random**					**0.06 (0.01, 0.10)**						**0.04 (0.01, 0.07)**		

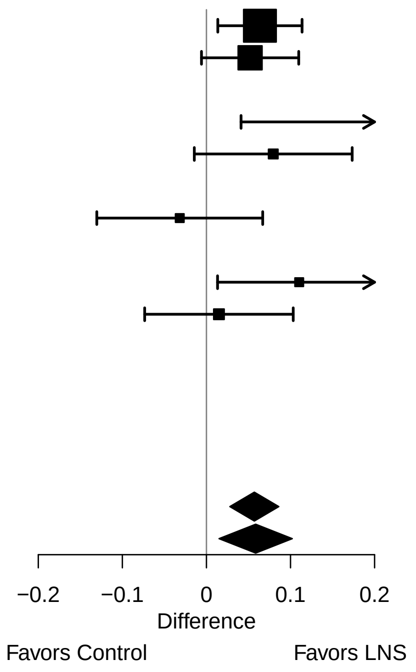

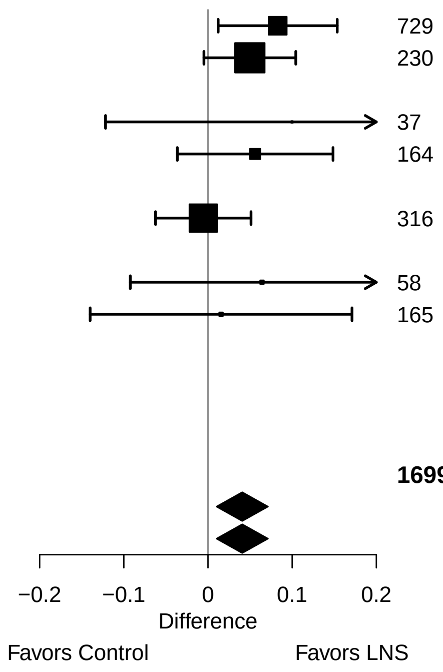

Supplement: nqab277_Supplemental_Files [file nqab277_supplemental_files.zip › ipdd_suppfig8_20210707.pdf]
